# Unique nigral and cortical pathways implicated by epigenomic and transcriptional analyses in rotenone Parkinson’s model

**DOI:** 10.1038/s41531-025-01049-1

**Published:** 2025-07-24

**Authors:** Maria Tsalenchuk, Kyle Farmer, Sandra Castro, Abigail Scheirer, Yuqian Ye, J. Timothy Greenamyre, Emily M. Rocha, Sarah J. Marzi

**Affiliations:** 1 UK Dementia Research Institute, Imperial College London, London, UK; 2 Department of Brain Sciences, Imperial College London, London, UK; 3 Pittsburgh Institute for Neurodegenerative Diseases, University of Pittsburgh School of Medicine, Pittsburgh, PA USA; 4 Department of Neurology, University of Pittsburgh School of Medicine, Pittsburgh, PA USA; 5 Department of Clinical Neurosciences, University of Cambridge, Cambridge, UK; 6 UK Dementia Research Institute, King’s College London, London, UK; 7 Department of Basic and Clinical Neuroscience, Institute of Psychiatry, Psychology and Neuroscience, King’s College London, London, UK

**Keywords:** Neuroscience, Neurodegeneration, Parkinson's disease

## Abstract

Pesticide exposure is increasingly recognized as a potential environmental factor in idiopathic Parkinson’s disease, though the molecular mechanisms remain unclear. This study explores how pesticide exposure alters gene regulation in key brain regions using the rotenone rat model. We performed H3K27ac ChIP-sequencing to profile active regulatory elements in the substantia nigra and motor cortex. Despite uniform complex I inhibition across regions, we observed region-specific epigenomic changes associated with rotenone exposure. RNA-sequencing confirmed transcriptomic alterations. We identified a strong, rotenone-induced immune response in the substantia nigra, including increased activity in the C1q complement pathway, suggesting immune involvement driven by regulatory mechanisms. In contrast, the cortex showed dysregulation of synaptic function at the gene regulatory level. Our results highlight a role for gene regulatory mechanisms potentially mediating the effects of pesticide exposure, driving region-specific functional responses in the brain that may contribute to the pathology and selective vulnerability that characterise Parkinson’s disease.

## Introduction

Parkinson’s disease (PD) is the fastest growing neurological disorder globally^1^. It is marked by progressive neurodegeneration of dopaminergic neurons in the substantia nigra pars compacta (SNpc) leading to the loss of dopamine in the striatum, neuroinflammatory activation of glial cells and aggregation of phosphorylated α-synuclein^2^. While the precise causes of PD remain unclear, a combination of genetic and environmental factors is believed to drive its onset and progression. Environmental exposures, including pesticides, have been increasingly recognised as important contributors to idiopathic PD^3^. However, the molecular mechanisms through which these environmental agents contribute to neurodegeneration are not yet fully understood.

Pesticide exposure, particularly mitochondrial complex I inhibitors like rotenone, induce a PD-like pathology in animal models of the disease, recapitulating many of the motor and non-motor symptoms observed in PD patients^4^. In adult rats, rotenone administration reproduces several pathogenic pathways, including oxidative stress, α-synuclein (α-syn) phosphorylation and aggregation in surviving SNpc DA neurons and in the gastrointestinal tract, lysosomal and proteasomal dysfunction and nigral iron accumulation^5,6^. Additionally, rotenone induces systemic changes affecting both the brain and peripheral tissues, which is significant as PD is increasingly recognised as a multi-system disorder. Studies have shown that rotenone exposure can lead to gastrointestinal issues and cardiac dysfunction^7,8^, mirroring non-motor symptoms observed in PD patients^9,10^.

While PD is traditionally characterised by nigrostriatal degeneration, there is increasing recognition of cortical involvement, particularly as the disease progresses^11^. Cortical pathology has been linked to non-motor symptoms, such as cognitive deficits and psychiatric disturbances, which significantly impact patient quality of life^12,13^. Moreover, growing evidence suggests that primary motor cortex dysfunction may contribute to motor impairments in PD, as abnormalities in motor cortex function have been observed in both PD patients and animal models. The motor cortex, which is intricately linked to the basal ganglia, undergoes functional alterations in PD, including disrupted excitability and impaired plasticity^14–16^. Direct stimulation of the motor cortex has been shown to alleviate PD symptoms, underscoring its relevance as a potential therapeutic target^15^. Additionally, rotenone-exposed rats exhibit α-syn accumulation in the frontal cortex, as well as progressive behavioural and neuropathological changes reminiscent of PD^17^. Despite these findings, the molecular mechanisms underlying cortical dysfunction in PD remain poorly understood, particularly in the context of epigenetic and transcriptional regulation.

Gene regulation and chromatin dynamics are increasingly recognised as central mechanisms in the development of neurodegenerative diseases^18^. Epigenetic modifications, such as histone acetylation, play a role in regulating gene expression and maintaining cellular identity. Epigenetic investigations in PD remain scarce, with only one study delving into the role of H3K27ac in the prefrontal cortex of PD patients^19^. Understanding these regulatory alterations in response to environmental insults is crucial for identifying molecular drivers of PD^20^.

In this study, we explored how rotenone exposure alters the epigenetic and transcriptomic signatures of two key brain regions involved in PD pathology: the SN and the motor cortex. This approach not only seeks to uncover potential vulnerabilities in the SN compared to the motor cortex but also aims to provide a comprehensive understanding of regional PD-linked gene regulatory signatures. Here, we used H3K27ac chromatin immunoprecipitation (ChIP)-seq and RNA sequencing (RNA-seq) to profile gene regulatory variation in the SN and motor cortex of rats exposed to rotenone compared to vehicle controls. Our results reveal widespread epigenetic and transcriptomic changes, highlighting distinct functional responses across different brain regions. This study represents the first comprehensive assessment of gene regulatory alterations in the rotenone rat model.

## Results

**Fig. 1:**
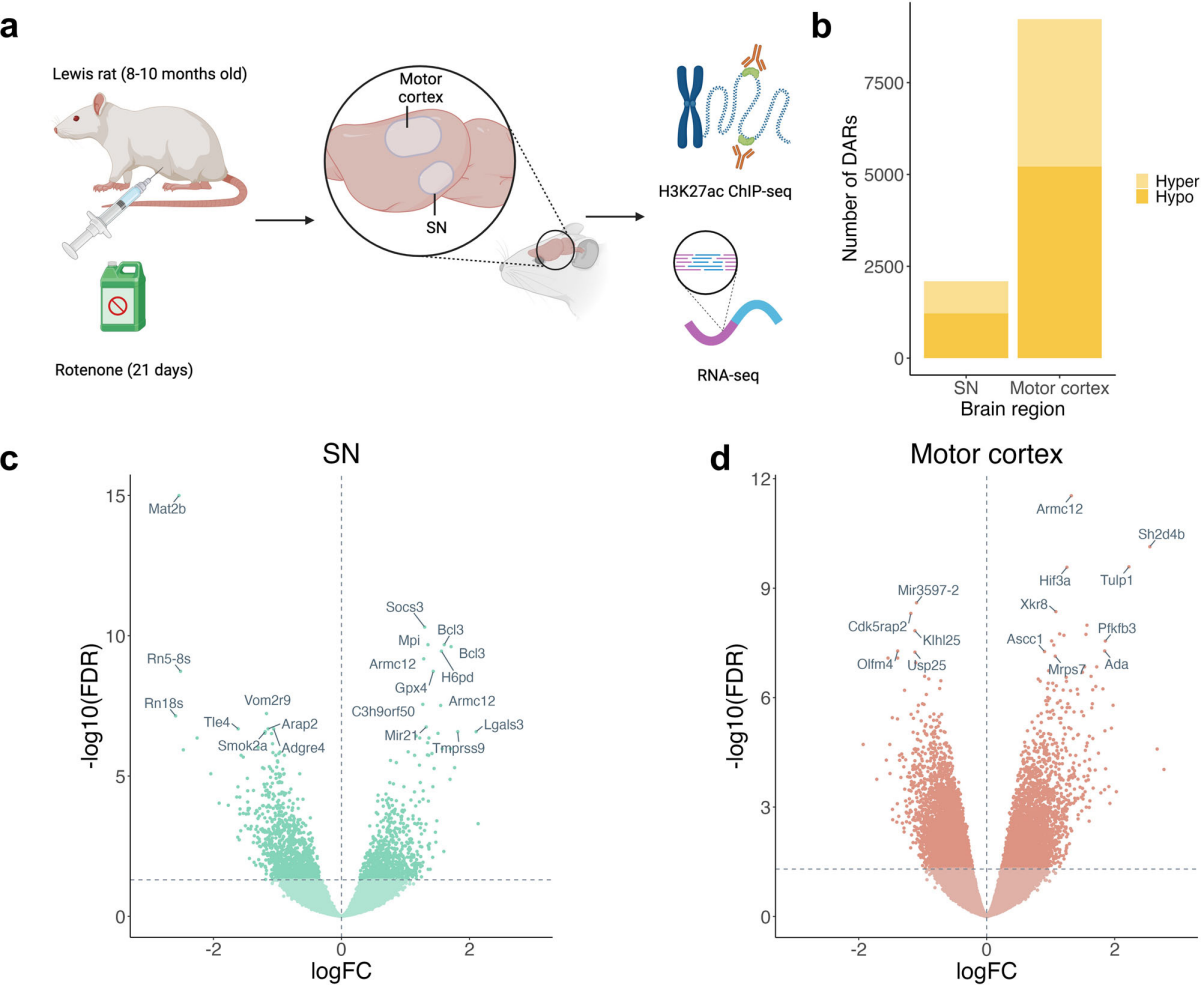
Epigenomic profiling of rotenone exposed rat motor cortex and SN reveals widespread changes in H3K27 acetylation. **a** Experimental workflow: Male Lewis rats were exposed to 2.8 mg/kg rotenone or vehicle intraperitoneally daily for 21 days. 24-h following the final injection, the motor cortex and SN regions were dissected from each brain (*n* = 5 for rotenone-treated, *n* = 4 for vehicle-treated, per brain region). ChIP-seq targeting H3K27ac was performed on these samples, complemented by RNA-seq. **b** Stacked bar plot showing the number of differentially acetylated regions (DARs) identified in the motor cortex and SN. **c** Volcano plot showing DARs in the SN. **d** Volcano plot showing DARs in the motor cortex.

Aged (8–10 months) male Lewis rats received daily systemic exposure to rotenone for 21 days (ChIP-seq; *N* = 9, RNA-seq; *N* = 20). At the end of the dosing regimen, rats have extensive PD pathology in the brain. To confirm rotenone caused a nigrostriatal lesion, tyrosine hydroxylase-positive terminals were assessed in the striatum (Supplementary Fig. 1). Subsequently, we dissected the motor cortex and SN to characterise the transcriptomic and epigenetic patterns caused by rotenone. To profile the histone acetylation landscape, we conducted ChIP-seq for H3K27ac, which marks active promoters and enhancers across the genome. To link our epigenetic data to variation in gene expression levels, we performed RNA-seq to profile mRNA transcripts in a separate set of animals (Fig. 1a).

After stringent quality control and pre-processing, we obtained high-quality H3K27ac ChIP-seq data from the SN and motor cortex of nine rats (rotenone *n* = 5, vehicle *n* = 4) and RNA-seq data from 10 SN and 8 motor cortex samples from equal numbers of rats treated with either rotenone or vehicle (Fig. 1a). On average, we obtained 32,790,067 sequencing reads per ChIP-seq sample (SD = 21,518,441) with no significant difference in read depth between the rotenone-exposed groups and controls (*p*-value = 0.54). In total we called 38,281 peaks in the SN and 80,763 in the motor cortex. We observed widespread differences in active promoters and enhancers across both brain regions in response to rotenone, accompanied by corresponding changes in gene expression. In the SN, 874 peaks showed increased acetylation and 1216 showed reduced acetylation upon rotenone exposure (Supplementary Tables 1 and 2), with significant hypoacetylated peaks being more prevalent (3.18% vs 2.28%, 3.33 × 10^−14^, exact binomial test). In the motor cortex, 4016 peaks were hyperacetylated and 5213 were hypoacetylated upon rotenone exposure (Supplementary Tables 3 and 4), with 11.4% of the peaks showing differential acetylation and a higher frequency of hypoacetylation (*p*-value = 2.2 × 10^−16^, exact binomial test). Of note, two of the top hyperacetylated peaks in the SN were annotated to *BCL3:* a promoter peak (logFC = 1.61, *p* = 2.18 × 10^−^¹⁴) and an intronic peak (logFC = 1.71, *p* = 3.20 × 10⁻¹⁴) (Supplementary Fig. 2). *BCL3*, a gene involved in immune signalling, has been implicated in late-onset Alzheimer’s disease and is linked to hyperinflammation in microglia^21^. Notably, *BCL3* was also one of the most overexpressed genes in the SN (logFC = 5.33, *p* < 4.42 × 1013) (Supplementary Table 5). Conversely, the top hypoacetylated peak in the SN was annotated to *MAT2B*, a gene involved in coenzyme metabolic processes, which has been found to be downregulated in patients with Lewy Body dementia^22^. One of the strongest hyperacetylated signatures in the motor cortex was observed for *HIF3A*, a gene typically upregulated in response to hypoxia in the brain^23^, while *OLFM4*, a gene involved in regulating synaptic function and inflammation^24^, was found to be hypoacetylated.

**Fig. 2:**
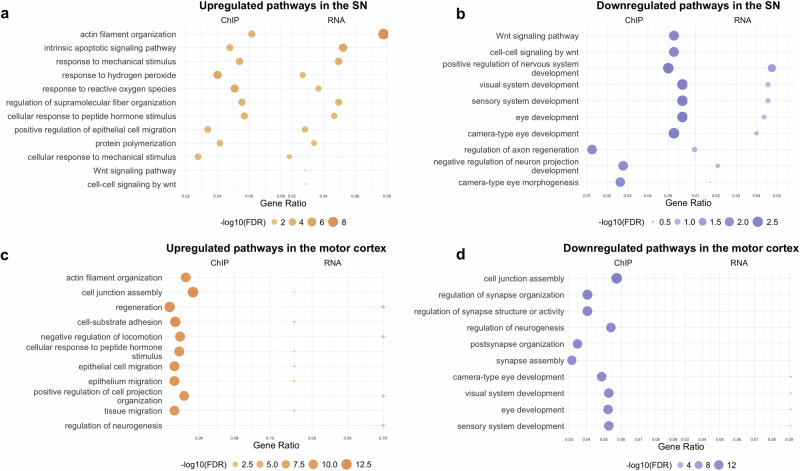
Pathway enrichment analysis reveals dysregulation of pathways linked to stress response and Wnt signalling in the SN, and hypoxia and synapse organisation in the motor cortex. Dot plots illustrating (**a**) upregulated pathways in the SN, (**b**) downregulated pathways in the SN, (**c**) upregulated pathways in the motor cortex, and (**d**) downregulated pathways in the motor cortex. Notably, there appears to be a stronger correlation between ChIP and RNA data in the SN.

To reveal dysregulation in biological pathways upon exposure to rotenone, we investigated functional enrichments amongst differentially acetylated regions and differentially expressed genes using pathway enrichment analysis (Fig. 2). We identified several consistently altered pathways across both brain regions, including upregulation of GO terms “cellular response to peptide hormone stimulus” (SN; *p* = 3.11 × 10^−7^, motor cortex; *p* = 4.25 × 10^−15^) and “actin filament organization” (SN; *p* = 3.08 × 10^−7^, motor cortex; *p* = 1.62 × 10^−^^14^), which may indicate a response to mitochondrial dysfunction and associated damage to the axon and cytoskeleton^25^. However, not all pathways were consistent between brain regions. In the motor cortex, other enriched terms in the hyperacetylated peaks included those associated with epithelial cell migration (epithelial cell migration; *p* = 5.69 × 10^−15^, epithelium migration; *p* = 8.14 × 10^−15^) potentially representing activation of survival pathways in response to rotenone (Fig. 2c). In contrast, in the SN, pathways associated with oxidative stress (response to hydrogen peroxide; *p* = 1.55 × 10^−9^, response to reactive oxygen species; *p* = 4.57 × 10^−9^) were among the most enriched in the hyperacetylated peaks (Fig. 2a). The pathways also diverged in the hypoacetylated peaks. The SN showed downregulation of pathways associated with Wnt signalling (Wnt signaling pathway; *p* = 6.24 × 10^−6^, cell-cell signaling by wnt; *p* = 6.91 × 10^−6^) (Fig. 2b), while the motor cortex showed downregulation of pathways associated with regulation of neurogenesis (regulation of neurogenesis; *p* = 4.07 × 10^−16^) (Fig. 2d). The pathway enrichment analysis appears to show a stronger correlation between ChIP and RNA data in the SN compared to the motor cortex. For an overall assessment of the concordance between ChIP-seq and RNA-seq, we correlated logFC values between promoter-associated DARs and corresponding gene expression (Supplementary Fig. 3). We observed a significant correlation between ChIP-seq and RNA-seq data, with a stronger association in the SN samples (*R* = 0.57) compared to the motor cortex samples (*R* = 0.42). This indicates a general concordance between changes in H3K27 acetylation and gene expression.

### Transcriptomic analysis with RNA-seq supports gene regulatory responses identified from histone acetylation signatures

**Fig. 3:**
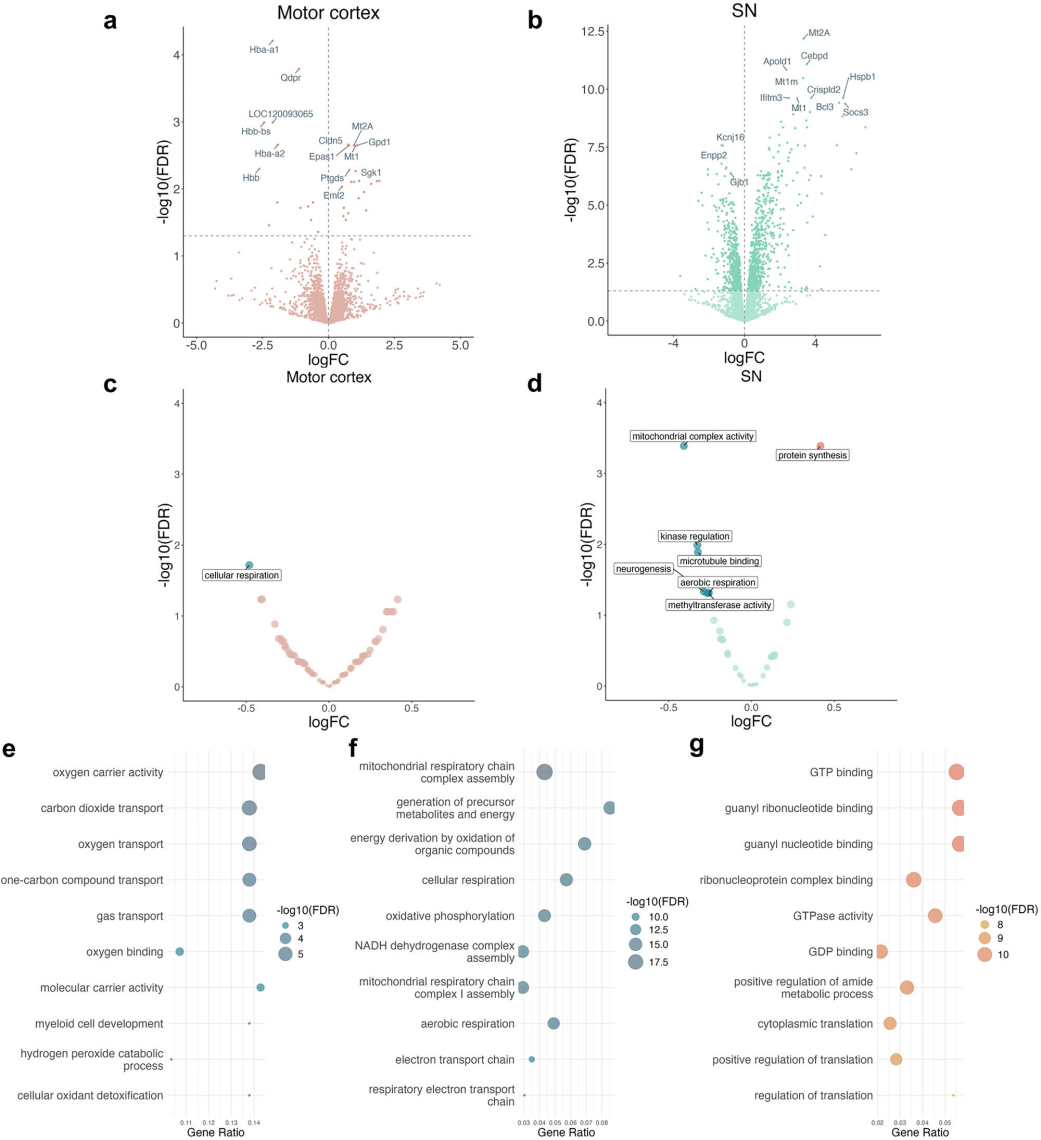
Gene co-expression network analysis reveals the upregulation of protein synthesis pathways and the downregulation of mitochondrial function-related gene modules in the SN. Volcano plot of differentially expressed genes in (**a**) the motor cortex and (**b**) the SN. Volcano plot of differentially expressed gene modules in (**c**) the motor cortex and (**d**) the SN. **e** Dotplot depicting enriched pathways in the downregulated module in the motor cortex. **f** Dotplot depicting enriched pathways in the most significantly downregulated module in the SN. **g** Dotplot depicting enriched pathways in the upregulated module in the SN.

To complement the histone acetylation signatures seen in both regions exposed to rotenone with transcriptomic activity, we performed low coverage RNA-seq (average number of reads = 953,647, SD = 516,771) on ten samples from the SN per condition and eight samples from the motor cortex per condition. We identified widespread changes in the SN transcriptome, where 791 genes were upregulated and 652 downregulated (FDR range = 6.68 × 10^−13^–4.99 × 10^−2^, log2FC range = −3.62 to 7.65), following exposure to rotenone (Fig. 3a). In the motor cortex, 21 genes were found to be upregulated, and 13 genes were downregulated (FDR range = 6.15 × 10^−5^–4.38 × 10^−2^, log2FC range = −2.63 to 1.92) (Fig. 3b). To investigate whether genes were showing concerted expression changes across gene regulatory networks, we used weighted gene co-expression network analysis (WGCNA) to classify and analyse gene modules. These modules were then tested for differences in expression between the rotenone and control groups. In the motor cortex, only one gene module showed significant differential expression, whereas in the SN, seven modules were downregulated, and one was upregulated (Fig. 3c, d). Pathway enrichment analysis revealed that the downregulated module in the motor cortex was associated with a disturbance in cellular respiration as evidenced by enriched terms such as oxygen transport (*p* = 1.42 × 10^−8^), carbon dioxide transport (*p* = 4.65 × 10^−9^) and gas transport (*p* = 6.86 × 10^−8^). The decrease in peroxidase activity (*p* = 9.08 × 10^−5^) and oxidoreductase activity (*p* = 1.01 × 10^−4^), also points towards alterations in oxidative stress response mechanisms (Fig. 3e). In contrast, the most significantly downregulated module in the SN was associated with cellular respiration, with all terms referring to mitochondrial processes, for example, mitochondrial respiratory chain complex assembly (*p* = 7.49 × 10^−20^) and NADH dehydrogenase complex assembly (*p* = 7.12 × 10^−18^) (Fig. 3f). Meanwhile, a module upregulated in the SN pertained to protein synthesis, including terms like ribonucleoprotein complex binding (*p* = 1.84 × 10^−13^), ribosome biogenesis (FDR = 1.01 × 10^−9^) and regulation of translation (FDR = 3.56 × 10^−11^) (Fig. 3g). This is an intriguing observation as previous research has indicated a decrease in RNA translation in response to rotenone exposure and in fibroblasts of *LRRK2* G2019S PD patients^26^. Therefore, our data may suggest a compensatory RNA translation mechanism in response to rotenone.

### Differential acetylation patterns reveal unique rotenone-associated immune-related changes in the SN

**Fig. 4:**
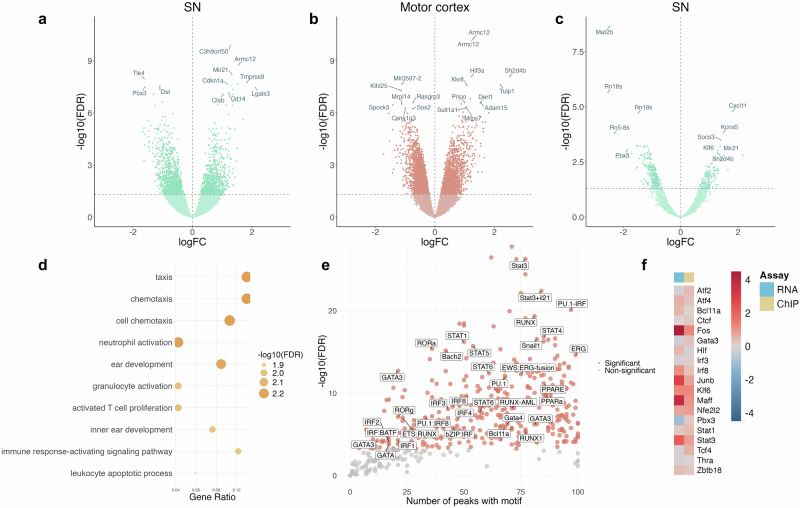
Immune genes show upregulation at both the transcriptional and epigenetic levels in the SN following exposure to rotenone. **a** Volcano plot of peaks shared between motor cortex and SN, with values specific to the SN shown. **b** Volcano plot of peaks shared between motor cortex, with values specific to the motor cortex shown. **c** Volcano plot of H3K27ac peaks unique to the SN. **d** Pathway enrichment of hyperacetylated peaks unique to the SN. **e** Motif analysis shows enrichment of immune motifs (labelled) in hyperacetylated peaks in the SN. **f** Heatmap showing log fold change for corresponding H3K27 acetylation and gene expression of immune-related transcription factors. Stars indicate significant log fold change (FDR < 0.05).

To identify pathways unique to each brain region, we performed an overlap of all the peaks from the motor cortex and SN. A total of 24,878 peaks were shared between the two brain regions (Fig. 4a, b). Among these shared peaks, the motor cortex exhibited 1,788 significantly hyperacetylated regions and 2224 significantly hypoacetylated regions (FDR range = 5.96 × 10^−9^–4.99 × 10^−2^, log_2_FC range = −2.53 to 1.41). In contrast, the SN showed 643 hyperacetylated peaks and 890 hypoacetylated peaks (FDR range = 2.72 × 10^−11^–4.99 × 10^−2^, log_2_FC range = −1.63 to 2.72). Of the significant peaks (FDR < 0.05) in both SN and motor cortex, the direction of effect was the same for 80% of peaks. It should be noted there are more overall reads in the motor cortex than the SN, so there is more power to detect differences, which may account for the disparity in number of DARs between brain regions. Pathway analysis across consistently altered peaks in both brain regions re-confirmed upregulation of the pathway “cellular response to peptide hormone stimulus” (motor cortex; *p* = 8.69 × 10^−^^13^, SN; *p* = 4.33 × 10^−8^).

When looking at the peaks unique to each brain region, 5205 peaks were found to be unique to the SN. Of these, 136 were hyperacetylated and 250 were hypoacetylated (Fig. 4a). Intriguingly, there were several hyperacetylated pathways that were uniquely enriched in the SN in response to rotenone exposure (Fig. 4b). These pathways were associated with the immune response, including chemotaxis (*p* = 5.77 × 10^−^^6^), activated T cell proliferation (*p* = 2.74 × 10^−5^), leukocyte apoptotic process (*p* = 6.00 × 10^−5^) and myeloid leukocyte activation (*p* = 7.29 × 10^−5^). These findings suggest a heightened immune and cellular activation response associated with rotenone exposure, which seems to be unique to the SN. These findings are consistent with published reports demonstrating rotenone induced SN inflammation^27^. T-cell activation is particularly interesting, given that in PD, autoimmune responses by T cells to α-syn are strongest in the earlier stages of disease and are gone 10 years after diagnosis^28^. Chemotaxis also implies an immune response wherein microglia migrate towards the site of inflammation in response to cytokines^29^. Furthermore, we identified a peak annotated to *CXCL11* among the top hyperacetylated peaks unique to the SN. *CXCL11* is a cytokine, which is known for its chemotactic properties for activated T cells^30^.

To further our understanding of the upstream regulators of the rotenone response, we performed transcription factor motif enrichment analysis using HOMER on significantly hyper- and hypoacetylated peaks SN (Fig. 4c and Supplementary Tables 6 and 7). Strikingly, and in agreement with the observed pathway enrichments, regions within the SN exhibiting increased acetylation were found to be enriched with immune-related transcription factor motifs. This included PU.1, the IRF family, RUNX1, the STAT family, GATA3, and IL-21. This result suggests heightened activity of transcription factors that regulate immune function in the SN of rotenone-exposed rats. Using a gene list of transcription factors involved in immune function and differentiation^31^, we identified which motifs were immune related and examined whether their corresponding genes showed differential expression or acetylation in the SN (Fig. 4d). Concordant changes in both gene expression and binding sites could indicate biological relevance, while the expression signature may help distinguish the binding patterns of these transcription factors, despite their overlapping motifs. We found that the majority of transcription factors (including *Fos*, *Irf8, Stat3* and *Maff*) showed both increased expression and elevated H3K27ac levels (Fig. 4e). *Irf8* is particularly interesting in the context of PD: Knock-down of *IRF8* has been shown to alleviate neuroinflammation and behavioural deficits in a MPTP mouse model of PD. This suggests that IRF8 may be a critical mediator of inflammatory processes driving neurodegeneration in PD^32^.

**Fig. 5:**
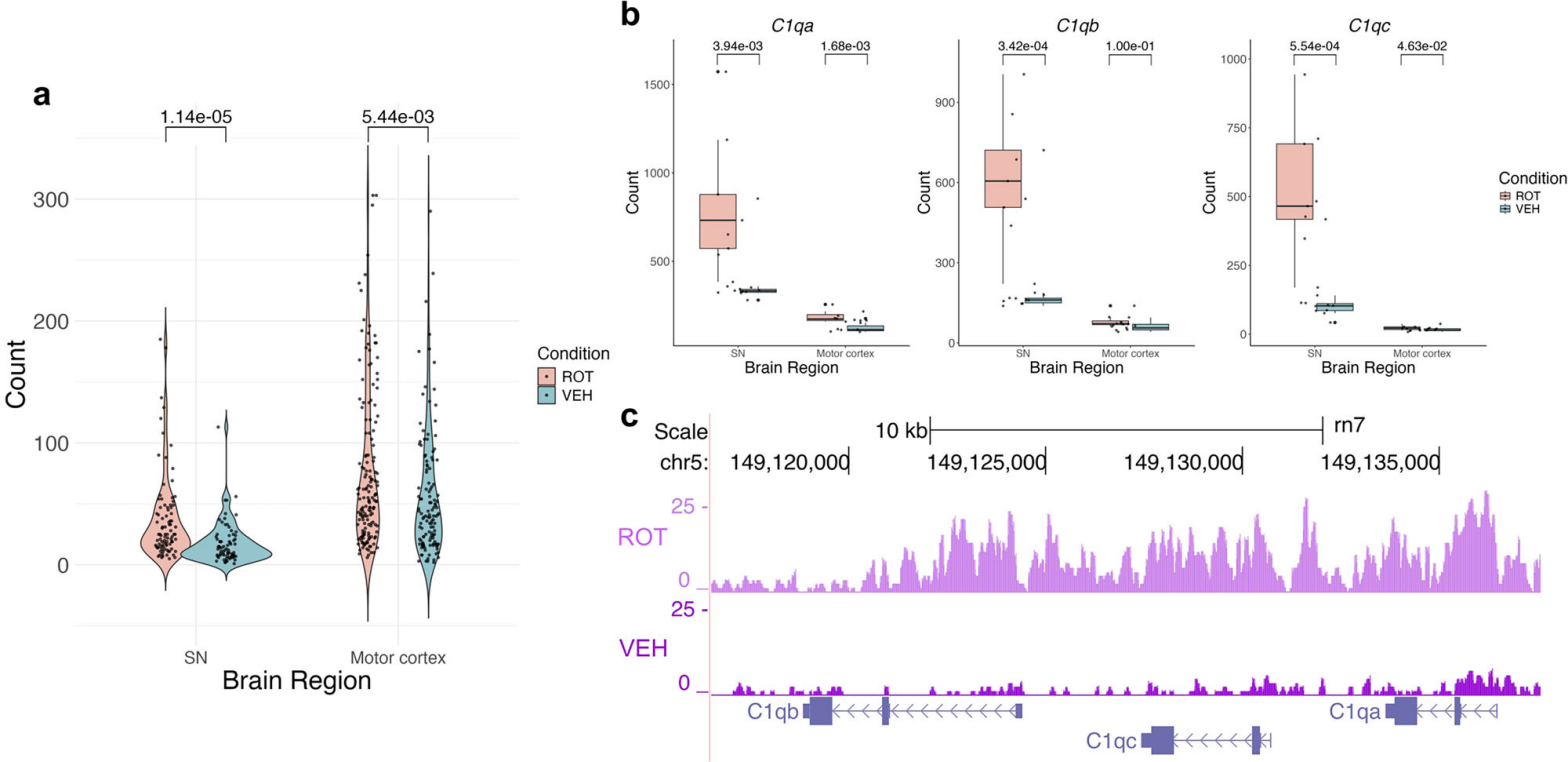
Upregulation of the complement system genes in the SN following rotenone exposure. **a** Violin plot showing increased acetylation of complement system genes in the SN and motor cortex of rotenone-treated samples. **b** Boxplot displaying elevated gene expression of the A-, B- and C-chains of C1q in the SN of the rotenone-treated group. **c** Genome browser track showing higher H3K27ac signals in C1q chain genes in rotenone samples compared to controls.

Expanding on the immune response observed in the SN following rotenone exposure, we identified the complement system as upregulated in both epigenomic and transcriptomic analyses (Fig. 5). The complement system, a crucial part of the innate immune response, is responsible for recognizing and clearing dying cells^33^. It has also been implicated in abnormal synaptic pruning in brain region-specific models of AD, where inhibition of C1q rescues synaptic loss. In this study, the SN of exposed rats showed cumulative hyperacetylation of 56 genes previously reported to be involved in the complement system (Fig. 5a)^34^. Of note the A-, B- and C-chains of complement component q1 (C1q) of the classical complement pathway are upregulated both at the level of gene expression (Fig. 5b) and acetylation in the SN but not motor cortex of rotenone-exposed rats (Fig. 5c and Supplementary Table 8).

**Fig. 6:**
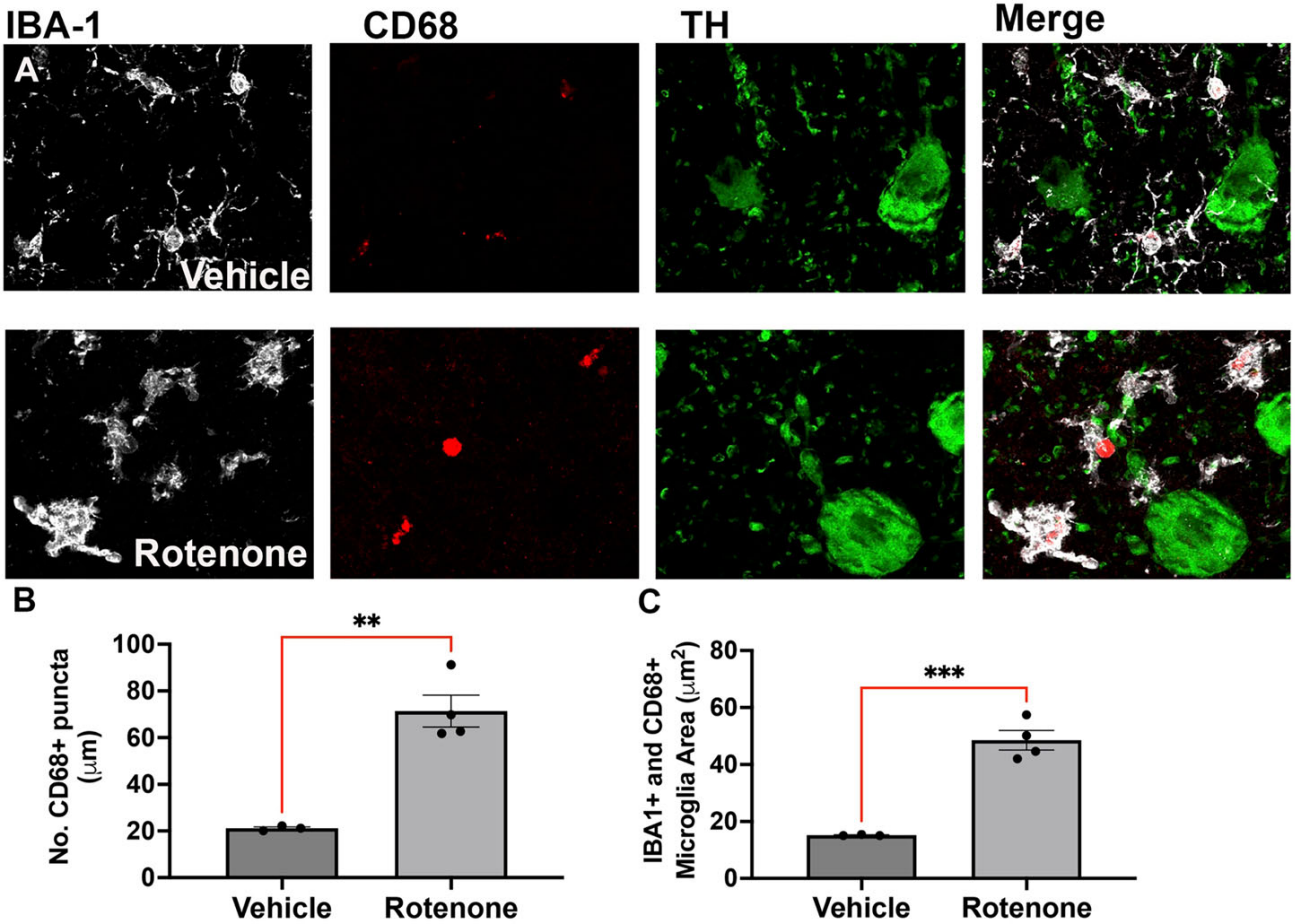
Chronic rotenone exposure causes neuroinflammation in the substantia nigra pars compacta. **A** Nigrostriatal microglial reactivity was assessed by immunohistochemistry using specific markers; Ionized calcium binding adaptor molecule 1 (Iba1; white) for microglia, cluster of differentiation 68 (CD68; red) for reactive microglia, and tyrosine hydroxylase (TH; green) for dopamine neurons of aged rats treated with 21 doses of rotenone. We found that rotenone increased both the number (**B**) and the size (**C**) of the CD68+ reactive Iba1+ microglia within the SNpc. Each data point represents a separate animal. Data was analyzed using an unpaired *t*-test, ** *p* < 0.001, *** *p* < 0.0001.

To confirm that the rotenone-induced immune response observed at the gene level stemmed primarily from activated microglia within the SNpc, we performed immunohistochemistry using a separate cohort of rats dosed with rotenone or vehicle. Tissue was stained for Iba1 to identify microglia, CD68, a lysosomal protein that is upregulated in reactive microglia while they are actively phagocytic and TH for dopaminergic neurons (Fig. 6A). SNpc samples from rotenone-exposed rats showed a higher density of reactive microglia, quantified by the number of CD68+ microglia (Fig. 6B). The surface area of these reactive microglia (Iba+/CD68+) within the SN was significantly larger in rats treated with rotenone in comparison to rotenone treated rats (Fig. 6C).

### Epigenetic analyses highlight synaptic dysregulation in motor cortex following rotenone exposure

**Fig. 7:**
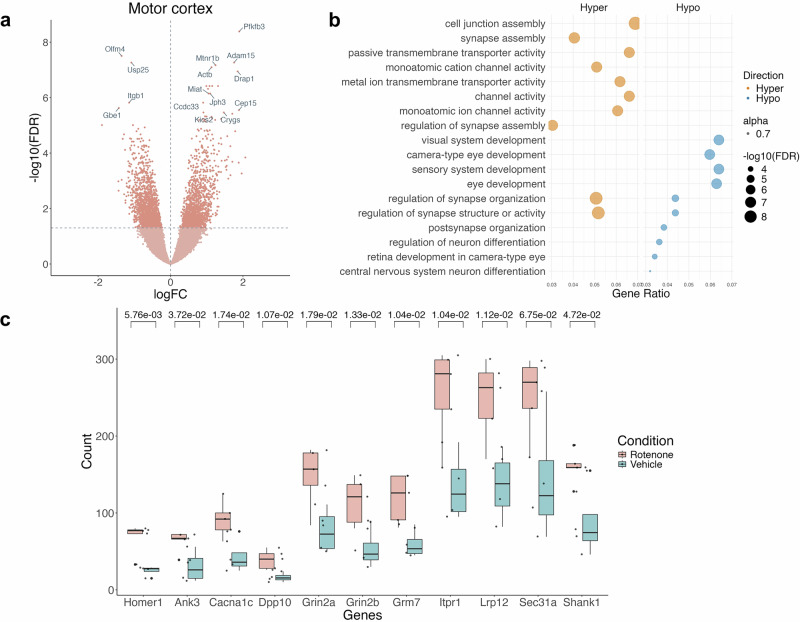
Dysregulation of synaptic pathways in the motor cortex upon exposure to rotenone. **a** Volcano plot of H3K27ac peaks unique to the motor cortex. **b** Pathway enrichment of hyper- and hypo- acetylated peaks unique to the motor cortex. **c** Box plots showing hyperacetylation of Homer1 and its known interactors in the motor cortex, showing the most significant peak for each gene.

We identified 36,296 acetylation peaks unique to the motor cortex, including 1394 hyperacetylated and 1655 hypoacetylated peaks (Fig. 7a). Unlike in the SN, pathways enriched in motor cortex-specific acetylation peaks suggest dysregulation of synaptic genes following rotenone exposure. Both hyper- and hypoacetylated regions are associated with processes such as synapse assembly (hyper; *p* = 2.61 × 10^−^^10^), regulation of synapse organisation (hypo; *p* = 1.90 × 10^−^^11^), and postsynapse organisation (hypo; *p* = 5.98 × 10^−^^11^) (Fig. 7b).

Among these hyperacetylated genes are *HOMER1, GRIN2B* and *SHANK1*, which are known to interact^35,36^. HOMER1 directly binds to GRIN2B, and its hyperacetylation may enhance this interaction, potentially leading to dysregulation of NMDA receptor activity, a pathway implicated in excitotoxicity. Moreover, HOMER1 scaffolds with SHANK1 at excitatory synapses, where it plays a critical role in maintaining dendritic spine integrity. The hyperacetylation of *HOMER1* may stabilise these synaptic complexes in response to rotenone-induced stress, serving as a compensatory mechanism.

However, given HOMER1’s broader role in synaptic protein networks, particularly through its interactions with proteins containing the Homer1-binding PPXXF motif, such as ankyrin-G^36^, hyperacetylation could excessively stabilise these complexes. This may drive overactive excitatory signalling, further contributing to excitotoxicity, a well-known mechanism linked to PD progression^37^. Supporting this, we also observe hyperacetylation in genes encoding postsynaptic proteins that contain the PPXXF motif and are known to bind HOMER1 (Fig. 7c).

Importantly, several of these genes have been previously implicated in PD. *HOMER1* has been linked to psychotic symptoms in PD through genetic mutations^38^, while *GRIN2B* polymorphisms have been associated with impulse control behaviours in PD^39^. Additionally, other genes that interact with Homer1 have been associated with PD, including *CACNA1C*^40^, *GRIN2A*^41^, *SEC31A*^42^, *LRP12* and *SHANK1*^43^.

## Discussion

Rotenone is a pesticide, also readily used to control nuisance fish populations, and serves as a well-established agent for inducing PD-like symptoms and pathology in rat models. To induce a Parkinsonian phenotype in rats, the toxin is administered systemically (intra-peritoneal cavity) and inhibits mitochondrial complex I uniformly throughout the brain. Strikingly, rotenone causes selective DA neurodegeneration in the SN despite reaching other DA populations^27^. The underlying mechanism responsible for this selective vulnerability is not clear, therefore we sought to better understand rotenone-induced epigenetic and transcriptomic changes in the SN. Identifying the source of this selective vulnerability will provide insight into the underlying causes of PD and may identify new therapeutic avenues. In this study, we quantified levels of histone acetylation (H3K27ac) and gene expression in the SN and motor cortex of rats exposed to rotenone, revealing widespread variation in both assays.

Consistent with known mechanisms implicated in rotenone toxicity, our findings underscore oxidative stress and mitochondrial impairment as core elements of its neurotoxic effects^27,44^. Oxidative stress and mitochondrial dysfunction can exacerbate each other, contributing to a cascade that impairs neuronal survival and function^45^. In line with this, differential H3K27 acetylation analysis identified genes implicated in oxidative stress, as upregulated in both the SN and motor cortex. Mitochondrial dysfunction is strongly reflected in our transcriptomic data, which shows downregulation of gene modules linked to mitochondrial function and cellular respiration, as identified through WGCNA. The gene expression changes we observed in both the motor cortex and SN are consistent with previous findings, further supporting the downregulation of mitochondrial pathways. For example, rotenone exposure in induced pluripotent stem cell-derived DA neurons led to a reduction in oxidative phosphorylation-related genes, as shown by single-cell RNA-seq^46^. Mitochondrial dysfunction and oxidative stress have also been noted in extra-nigral areas such as the striatum and cortex^47^. Additionally, another study found upregulation of oxidative stress genes across various tissues, including the brain^48^. In our data, oxidative stress pathways were especially enriched in the SN, suggesting a potential role in DA neuron loss in this region. This is notable as it underscores a differential gene-level response to oxidative stress between the motor cortex and SN. While oxidative stress and mitochondrial dysfunction are well-established mechanisms of rotenone neurotoxicity, our study demonstrates that these processes are regulated at the epigenetic level, particularly through alterations in histone acetylation. The brain region-specific differences in H3K27ac signatures suggest that epigenetic modifications may contribute to the selective vulnerability of the SN.

We also observed an upregulation of gene expression of genes involved with protein synthesis in the SN, particularly those related to translation initiation and ribosome assembly. This may represent a cellular attempt to counteract protein synthesis inhibition caused by rotenone exposure, suggesting a compensatory mechanism aimed at maintaining protein synthesis under stress conditions^49^. Indeed, such an effect has been observed in iPSC-derived dopaminergic neurons exposed to rotenone, leading to an upregulation of genes involved in translation and ribosome structure^46^.

Of note, epigenetically upregulated genes in the SN showed enrichment in immune response pathways, while this response was not observed in the motor cortex. We confirmed microglial activation and reactivity by immunohistochemistry in a separate cohort of rats. This is consistent with previous evidence demonstrating heightened immune response in the SN, as indicated by highly reactive microglia^50^. Rotenone has been shown to trigger a midbrain-restricted inflammatory response, with heightened expression of glial markers observed only in the nigra, and not in extra-nigral regions in rodent models^17,27,47^. The immune response in PD has been recognized since the 1920s, initially observed through an increase in the number of reactive microglia^51^. Post-mortem brain samples of PD patients have revealed increased levels of the pro-inflammatory cytokine TNF-α and the T cell-associated chemokines CXCL12 in the SN, preceding the loss of DANs^52^, a pattern that aligns with our observations of H3K27ac in rotenone-exposed rats. In a rotenone mouse model, specific microglial activation in the SN was observed in response to rotenone, although the cortex, striatum, and globus pallidus did not exhibit a corresponding increase in immune activity^27^. Our data suggests that the immune response may be driven by the variety of immune related transcription factors identified by the motif analysis, including the STAT family, IRF8 and PU.1. Although additional validation is warranted to confirm their specific roles in rotenone-induced neurotoxicity, their enrichment in our dataset suggests that they may serve as key mediators of the selective inflammatory response observed in the SN. Importantly, these findings raise the possibility that targeting epigenetic modifications or transcription factors regulating immune activation could provide a novel therapeutic strategy for PD. An example of such an approach has already been demonstrated, where knockdown of IRF8 ameliorated neuroinflammation and neurotoxicity-associated deficits in an MPTP model of PD^32^. This supports the idea that modulating specific transcriptional regulators may mitigate dopaminergic neuron loss and suggests that further exploration of transcription factor-targeted interventions in rotenone models is warranted.

We also observed a striking, nigra-specific upregulation of both acetylation and expression of *C1QA*, *C1QB* and *C1QC*, key components of the classical complement pathway, suggesting an enhanced inflammatory response in this region. Complement activation has been previously linked to PD and PD models. In vivo, α-syn preformed fibril and A53T α-syn transgenic mouse models show upregulation of the complement cascade, particularly C3, compared to controls^53^. In vitro studies have demonstrated that α-syn directly activates the classical complement pathway, which mediates α-syn-dependent cytotoxicity^54^. In humans, complement activation has been shown to occur on Lewy bodies and melanised neurons in the SN. Specifically, early complement activation (shown by iC3b) is increased on melanised neurons in PD compared to age-matched controls^55^. Additionally, C1q is upregulated in PD SNpc microglia, where it is hypothesised to mediate phagocytosis and clearance of debris from degenerating neurons^56^.

In contrast, analysis of cortical H3K27ac data indicated alterations in synapse function. While limited research exists on the response to rotenone in the motor cortex, a study demonstrated that primary cortical cells exposed to rotenone showed impaired axonogenesis^57^. Another study using neocortical neurons treated with rotenone revealed mitochondrial dysfunction and an increase in apoptotic genes^58^. Our data underscore the necessity for further investigations in the motor cortex, given the significant alterations we observed, particularly of synaptic pathways at the epigenetic level. Furthermore, cortical pathology is evident in PD patients, including cortical atrophy and reduced synaptic density, which progresses from the SN to cortical regions as the disease advances^59,60^, mirroring observations in the cortex ChIP-seq data. Pathway analyses of altered histone acetylation following rotenone exposure suggest that rotenone-associated synaptic dysfunction may be regulated at least partially at the epigenetic level.

Our study is subject to several limitations that warrant consideration. Firstly, there is an imbalance in sequencing depth between the ChIP-seq and RNA-seq samples. While the ChIP-seq samples were sequenced at a depth of 30 million reads, the RNA-seq samples were sequenced at a depth of only 5 million reads per sample. This discrepancy in sequencing depth may have led to the underrepresentation of certain transcripts in the RNA-seq data, potentially reducing our ability to fully characterise the underlying transcriptional mechanisms associated with rotenone toxicity. In light of this, we performed primary analyses on the H3K27ac ChIP-seq data and used the low-coverage gene expression to contextualise and interpret results observed at the epigenetic level. Of note, while we detected more DARs in motor cortex, the ChIP-seq data had higher read coverage in the motor cortex compared to the SN, increasing our power to detect differentially acetylated regions and could partially explain the different number of DARs between the two brain regions. Secondly, the use of bulk tissue genomic techniques provides an averaged view of histone acetylation and gene expression across the constituent cell types under investigation. This approach may obscure potential cellular heterogeneity within the sampled tissue, limiting our ability to detect subtle changes in gene regulation that may be specific to certain cell types or subregions. Of particular relevance in this context are dopaminergic neurons and microglia, both of which occur at low proportions in the surveyed brain regions. In this context, it is of note that we are able to detect a strong SN-specific immune response in the bulk tissue samples. Additionally, the relatively small number of samples used in our study may have impacted the statistical power of our findings. While the rotenone rat model is well-established, we acknowledge that no single model can recapitulate human disease. Advances in machine learning for cross-species translation of genomic results, are promising in this regard^61^. Finally, our study focused on H3K27ac as a marker of active enhancers, given its well-documented enrichment in the gene regulatory regions of genes implicated in neurodegenerative diseases, such as Alzheimer’s disease^18,62^. However, it is important to acknowledge that other epigenetic mechanisms, such as DNA methylation or histone methylation, could also play a role in mediating the effects of rotenone on gene expression and neuronal function. Future studies incorporating a broader range of epigenetic markers may provide a more comprehensive understanding of the molecular mechanisms underlying rotenone-induced neurotoxicity.

Our study provides compelling evidence that cellular response to rotenone is not only characterized by oxidative stress and mitochondrial dysfunction but is epigenetically regulated in a brain-region-specific manner. By integrating histone acetylation and transcriptomic signatures, we identify a distinct transcriptional program in the SN, characterised by enhanced immune activation and complement cascade upregulation. These findings suggest that targeting transcription factors such as STATs, IRF8 and PU.1, or modulating epigenetic marks like H3K27ac, could represent novel therapeutic strategies for PD. Future studies incorporating pharmacological or genetic manipulation of these regulatory pathways could provide deeper insights into their role in PD pathogenesis and therapeutic potential.

## Methods

### Rotenone rat generation

Retired breeder male Lewis rats were purchased from Envigo and single housed for experimental procedures. Animals were allowed to acclimate for 2-weeks and handled for an additional 1-week prior to experimentation. Animals were housed in standard conditions in a dark/light cycle of 12 h, with *ad libitum* access to food and water. Rats were randomly assigned to each experimental group and all experimental analysis were carried out blinded. All animal procedures were performed in accordance with National Institute of Health guidelines and were approved by the Institutional Animal Care and Use Committee (IACUC) at University of Pittsburgh.

Male Lewis rats aged 8–10 months received a single daily interperitoneal (i.p.) injection of 2.8 mg/kg of rotenone resuspended in 2% DMSO, 98% Miglyol 812 N as previously described^6^. Rats received daily rotenone dosing until they reached their behavioral endpoint. Endpoint for each animal is defined as a rat’s inability to successfully perform the postural instability test or 25% body mass was lost as previously described^6^. Animals were terminally anaesthetized using 0.3 mg/kg pentobarbital, followed by transcardial perfusion with 25 ml phosphate-buffered 0.9% saline (PBS). For ChIP-seq experiments, five rats were exposed to rotenone and four rats were in the control group for both brain regions. In each treatment group for RNA-seq experiments of the SN, there were 10 rats, while for the motor cortex, there were eight rats per treatment group. 24 h after the final rotenone injection, rats were perfused using a saline-buffered solution. The motor cortex and SN were microdissected and immediately placed on dry ice.

### Striatal TH terminal intensity

Serial striatal brain sections (every 6th section) were stained for TH and analyzed using near-infrared imaging for density of dopamine neuron terminals (LiCor). Regions of interest (ROI) were drawn around the dorsal lateral region of the striatum and the average intensity for each striatal section was recorded using Odyssey software (V3.0).

### Microglia reactivity

Animals were terminally anaesthetized using sodium pentobarbital (PB) and perfused transcardially with 25 ml phosphate-buffered 0.9% saline (PBS). Brains were removed, sagittal dissection down the midline, ½ brain post-fixed in ice-cold 4% paraformaldehyde in phosphate buffer for 48 h at 4 °C and placed in 30% sucrose. 35 μm thick free-floating sections were collected using a microtome and stored at −20 °C in cryoprotectant until use. Tissue sections were stained free-floating and mounted onto Superfrost Plus glass slides (Fisher, 12–550-15). Fluorescent images were taken using an Olympus BX61 confocal microscope and Fluoview 1000 software (Melville, NY). A negative control (secondary antibody only) slide was prepared to subtract the background fluorescence. Imaging parameters were monitored to ensure that images were above background level and below pixel saturation. For quantitative comparisons between groups, all imaging parameters (e.g., laser power, exposure, and pinhole) were held constant across specimens. Confocal images were captured using a UPlansApo 60×/1.35 oil objective lens. A minimum of 3 images per tissue section and 3 tissue sections per animal were used for each analysis. See Table 1 for a list of antibodies used.

**Table. 1:** Antibody list for confocal imaging

Antibody	Catalogue No.	Vendor	Concentration
Iba-1	Ab5076	Abcam	1:1000
CD68	MCA341GA	BioRad	1:500
TH	AB152	Millpore	1:2000

Confocal images were analyzed using Nikon NIS-Elements Advanced Research software (Version 4.5, Nikon, Melville, NY). Standard threshold parameters were set up for each analysis and used to identify each puncta or ‘objects’. The number of CD68 objects (# of objects) were collected within each Iba1-positive cell body within the substantia nigra and averaged across each image and animal.

### Nuclei isolation for ChIP-seq

Nuclei isolation from rat midbrain and cortical tissue was performed following a previously published protocol with slight modifications^63^. Briefly, fresh frozen brain tissue weighing approximately 2–10 mg was homogenised using a Kimble Dounce tissue grinder in 1% formaldehyde in phosphate buffered saline (PBS). To quench the fixation, glycine was added at a final concentration of 0.125 M. The homogenate was then pelleted at 1100 × *g* for 5 min at 4 °C. Subsequent steps were carried out on ice or at 4 °C. The homogenates were washed twice with NF1 buffer (10 mM Tris-HCl pH 7.4, 1 mM EDTA pH 8.0, 5 mM MgCl_2_, 0.1 M sucrose, 0.5% (vol/vol) Triton X-100) and then incubated in 5 mL of NF1 buffer for 30 min. The homogenates were passed through a 70-μm cell strainer. Homogenates were underlaid with a 1.2 M sucrose cushion (1.17 M sucrose, 10 mM Tris-HCl pH 7.4, 3 mM MgCl_2_, 1 mM DTT), before centrifuging at 3900 × *g* for 30 min at 4 °C with the brake set to “low.” The pelleted nuclei were washed with NF1 buffer, followed by a wash with FANS buffer (PBS, 1% (wt/vol) bovine serum albumin (BSA) and 1 mM EDTA pH 8.0).

### ChIP-seq library preparation

ChIP-seq was conducted based on a previously published protocol with minor modifications^64^. Fixed nuclei were resuspended in LB3 buffer (10 mM Tris-HCl pH 7.4, 100 mM NaCl, 1 mM EDTA PH 8.0, 0.5 mM EGTA, 0.1% sodium deoxycholate, 0.5% N-Lauroylsarcosine, 1× protease inhibitor), and then transferred to Covaris microtubes with AFA fiber. All subsequent steps were carried out on ice or at 4 °C. The samples were sonicated using a Covaris E220 focused-ultrasonicator (Covaris, MA) for 10 min (Duty: 5, PIP: 140, Cycles: 200, AMP/Vel/Dwell: 0.0). To serve as DNA input controls, 1% of the sample was stored at −20 °C. For the ChIP procedure, 25 µL Protein G Dynabeads (Invitrogen, 10004D) and 3 µL H3K27ac antibody (Active Motif, 39133) were added to the samples, and rotated at 4 °C overnight. The Dynabeads were subsequently washed three times with WB1 (20 mM Tris-HCl pH 7.4, 150 mM NaCl, 2 mM EDTA pH 8.0, 0.1% SDS, 1% Triton X-100), three times with WB3 (10 mM Tris-HCl pH 7.4, 250 mM LiCl, 1% Triton X-100, 1 mM EDTA PH 8.0, 0.7% sodium deoxycholate), three times with TET (10 mM Tris-HCl pH 7.4, 1 mM EDTA PH 8.0, 0.2% Tween20), and finally with Te-NaCl (10 mM Tris-HCl pH 7.4, 1 mM EDTA PH 8.0, 50 mM NaCl). The beads were resuspended in 25 μL of TT (10 mM Tris-HCl pH 7.4, 0.05% Tween 20). Input samples were adjusted to 25 μL with TT, and input libraries were generated in parallel with the ChIP libraries. For library preparation, the NEBNext Ultra II DNA Library Prep kit (New England BioLabs, E7645) was used for End Prep and Adapter Ligation, following the manufacturer’s instructions. Unique barcoded adapters (NextFlex, Bioo Scientific) were added. To decrosslink, libraries were incubated with RNase A (Thermo Scientific, EN0531) and Proteinase K (New England BioLabs, P8107) at 55 °C for 1 h and 65 °C overnight. Libraries were then PCR amplified for 14 cycles with NEBNext Ultra II Q5® Master Mix (New England BioLabs, M0544). Gel extraction using a 10% TBE gel was performed to size-select libraries for 200–500 bp fragments. Samples were sequenced by the Imperial BRC Genomics Facility on an Illumina NextSeq2000 platform using paired-end sequencing and a read length of 75 bp, targeting a depth of 30 million reads.

### RNA-seq library preparation

Samples were homogenised in 1 mL of Trizol reagent (Thermofisher Scientific, Waltham, MA) and total RNA was extracted using the Life Technologies min-RNA purification kit (Qiagen, Valencia, CA) according to the manufacturer’s protocol. Libraries were constructed using the 3′QuantSeq kit (Lexogen) with the following modifications. To enable the removal of PCR duplications, a custom oligo-dT primer was designed using a 12-bp unique molecular identifier. Samples were sequenced to a depth of 5 million, single-end reads, each on a Illumina Novaseq platform (Illumina, San Diego, CA).

### Data analysis

Initial screening for sample anomalies was performed with FastQC (version 0.11.9). Adapter sequences were trimmed using Trim Galore! (version 0.6.7). For ChIP-seq, the trimmed FASTQ files were then mapped to the rat reference genome (mRatBN7.2) using Bowtie2 (version 2.4.4), followed by the removal of duplicate reads with Picard (version 2.6.0). Peak calling was conducted using MACS2 (version 2.2.7.1) on merged samples. For normalisation, we used merged control inputs pooled from multiple animals. Specifically, reads from individual replicates were combined into a single dataset prior to peak calling, allowing us to detect regions consistently enriched across samples. The pooled input control ensured accurate background normalisation across the merged data. The parameters for calling peaks were set for paired-end BAM files, using a genome size of 2.7 billion, a shift size of 50, and all duplicate reads were retained. Finally, FeatureCounts (version 2.0.5) quantified the alignment of reads to these identified peak regions. For RNA-seq, FASTQ files were aligned, and transcripts were quantified using Salmon (version V1.10.0). Differential analysis to pinpoint epigenomic and transcriptomic differences between rotenone and control groups was performed using a quasi-likelihood *F* test in EdgeR (4.0.16). Normalisation factors were calculated based on sample-specific library compositions, and a quasi-likelihood model was fitted to the data using glmQLFit() and glmQLFTest() functions. Effect sizes are reported as log(fold change), which refers to the log_2_-transformed ratio of normalised counts between cases and controls. Transcripts and H3K27ac peaks with FDR-corrected *p*-value < 0.05 were considered differentially expressed (upregulated or downregulated) or differentially acetylated (hyperacetylated or hypoacetylated) between the two experimental groups. Weighted gene co-expression network analysis (WGCNA) was performed on transcript counts in the motor cortex and SN, respectively using the WGCNA R package (1.72.5). The H3K27ac peaks were assigned to genes using the annotatePeak() function in ChIPseeker (1.38.0) using “org.Rn.eg.db” as the annotation package and taking into account regulatory regions within 3 kb of the transcription start site. Gene ontology enrichment was performed using the enrichGO() function of the package clusterProfiler (4.10.0) for gene ontology enrichment analysis. Motif analysis was performed using HOMER, using all detectable H3K27ac peaks as background. Detailed annotated code used for analysis can be found at https://github.com/Marzi-lab/rotenone_rat_ChIP/.

## Supplementary information

Supplementary information

## Data Availability

FASTQ files have been deposited in the NCBI’s Gene Expression Omnibus^65^ and are accessible through GEO Series accession number GSE280519 for RNA-seq dataset and GSE280520 for the ChIP-seq dataset. All the data and code required to reproduce the figures in this manuscript are available in our GitHub repository: https://github.com/Marzi-lab/rotenone_rat_ChIP/.
