# Supplementary material for: Unique nigral and cortical pathways implicated by epigenomic and transcriptional analyses in rotenone Parkinson’s model
Source: NPJ Parkinsons Dis. 2025 Jul 24;11:217. doi: 10.1038/s41531-025-01049-1 (PMC12289881; doi:10.1038/s41531-025-01049-1)
Supplement: Supplementary file 1 — Supplementary information [file 41531_2025_1049_MOESM1_ESM.pdf]

Supplementary information

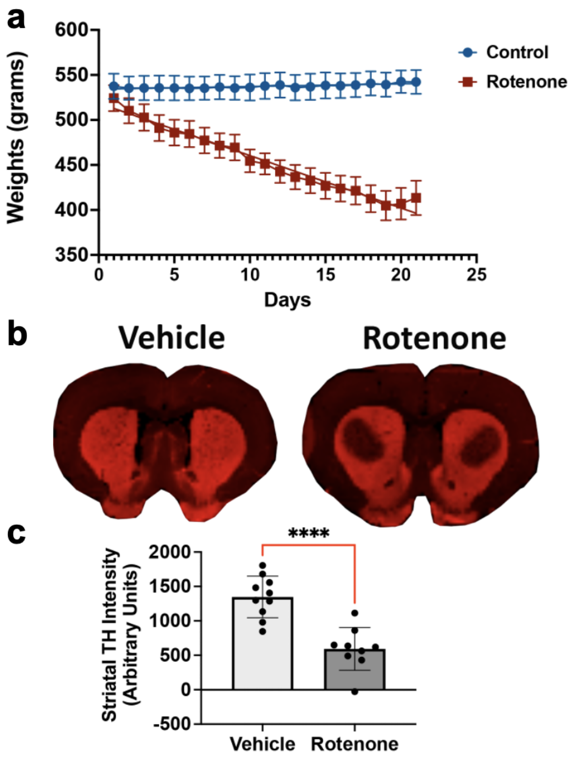

Supplementary Figure 1. Rotenone caused a loss of striatal dopaminergic terminals. Male Lewis rats (8-10 months of age) received a single dose of vehicle (Miglyol) or rotenone (2.8 mg/kg) for 21 days. **a)** Lewis rats dosed with rotenone consistently lost weight beginning on day 3. **b-c)** Striatal dopamine terminal loss was assessed by immunohistochemistry using specific markers; tyrosine hydrolase (TH) for dopaminergic neurons. **b)** Representative photomicrographs of striatal dopaminergic terminals of rats treated with vehicle or rotenone. **c)** Quantification of striatal dopaminergic terminals loss in rats; n=10/grp. Data are analyzed using unpaired T-test, *** $p < 0.0001$ graphs are expressed as mean ± SEM. Symbols represent individual brains.

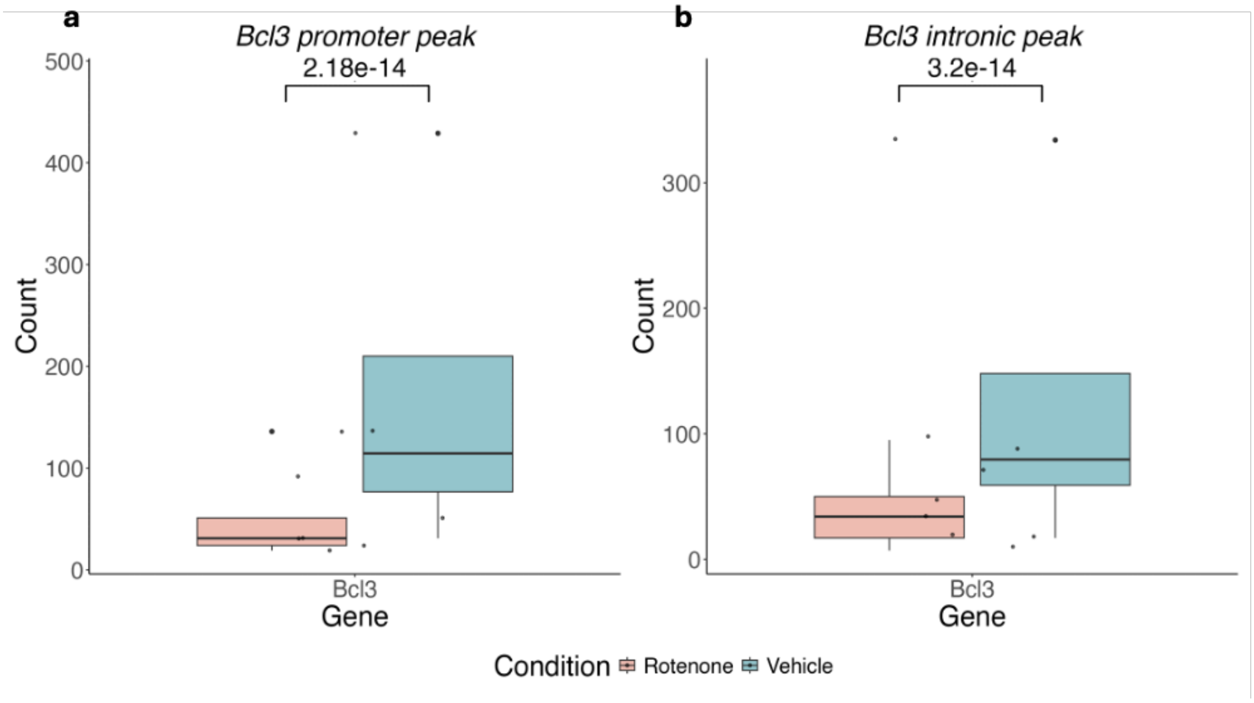

Supplementary Figure 2: Box plots showing the hyperacetylation levels of two *Bcl3* peaks in the SN following rotenone exposure. a) Promoter peak**. b)** Intronic peak.

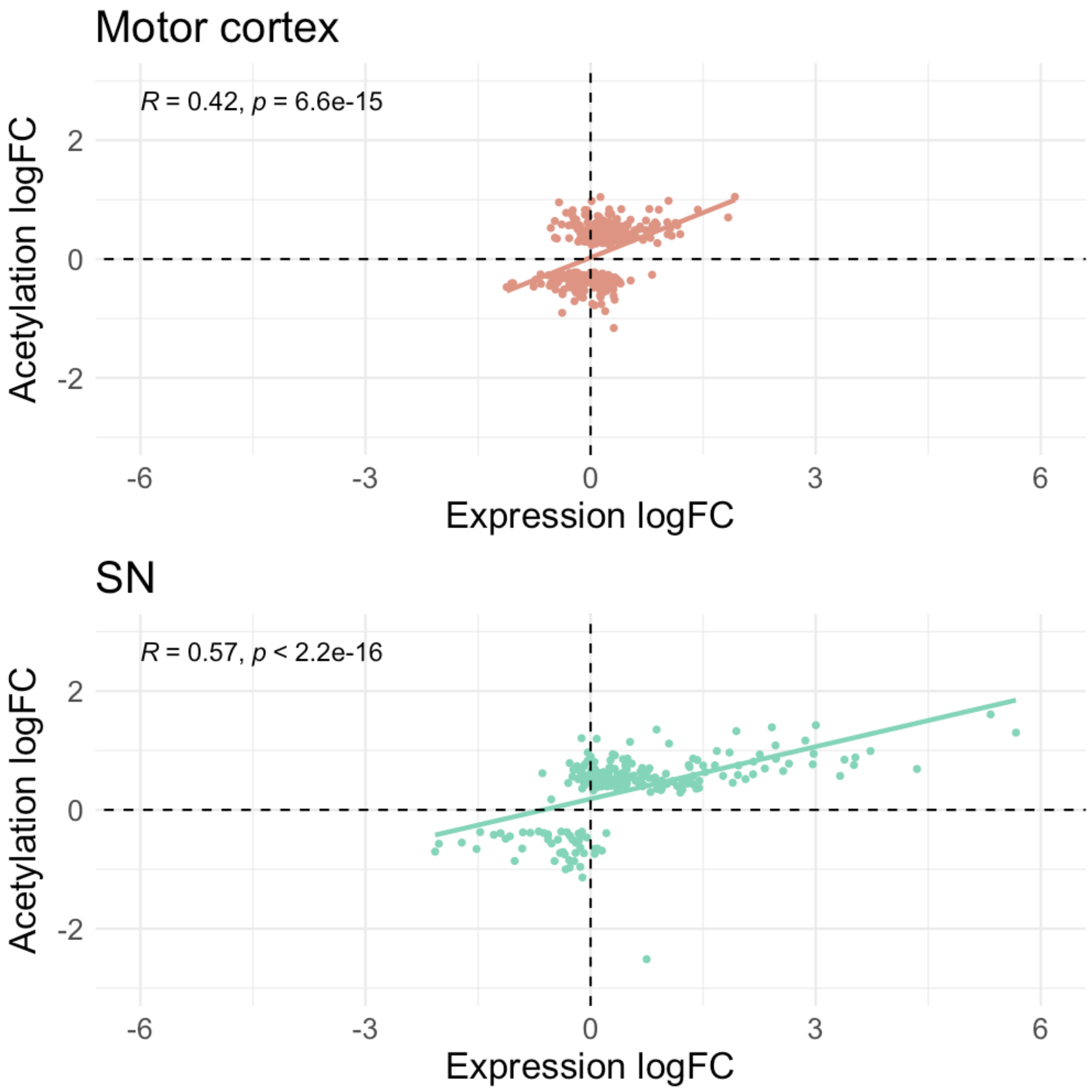

Supplementary Figure 3: RNA-seq and ChIP-seq signals correlate across differentially acetylated promoters and their genes. Correlation of effect sizes (logFC) in acetylation and gene expression, comparing rotenone-exposed rats to vehicle controls in **a**) motor cortex and **b**) SN. Correlations were performed using the Pearson's product moment correlation coefficient.

Supplementary Table 1: Significantly hyperacetylated peaks in the substantia nigra

chr	*start*	*end*	*annotation*	*logFC*	*PValue*	*FDR*	*GENE*	*distanceToTSS*	*DAR*
chr10	103189547	103198327	Promoter (<=1kb)	1301	257E-15	492E-11	Socs3	0	Hyper
chr8	57947171	57956547	Promoter (<=1kb)	1352	167E-14	209E-10	Mpi	0	Hyper
chr1	79484390	79487679	Promoter (<=1kb)	1607	218E-14	209E-10	Bcl3	0	Hyper
chr1	79478543	79481302	Intron (NM_001109422/680611, intron 2 of 8)	1713	320E-14	245E-10	Bcl3	4305	Hyper
chr5	160437747	160440568	3' UTR	1563	557E-14	356E-10	H6pd	29603	Hyper
chr20	6572963	6577068	Distal Intergenic	1286	122E-13	667E-10	Armc12	-10791	Hyper
chr7	9629701	9634926	Intron (NM_001108068/314619, intron 5 of 32)	1435	435E-13	185E-09	Gpx4	17586	Hyper
chr3	14055036	14058883	Distal Intergenic	1272	729E-12	279E-08	C3h9orf50	28095	Hyper
chr20	6569837	6572393	Distal Intergenic	1550	878E-12	306E-08	Armc12	-15466	Hyper
chr10	71406338	71409628	Promoter (<=1kb)	1325	651E-11	178E-07	Mir21	-990	Hyper
chr7	8832962	8834452	Promoter (1-2kb)	1817	137E-10	263E-07	Tmprss9	-1797	Hyper
chr15	20597938	20599843	Distal Intergenic	2104	138E-10	263E-07	Lgals3	-20240	Hyper
chr8	57943925	57946908	Promoter (2-3kb)	1509	173E-10	301E-07	Fam219b	2623	Hyper
chr20	7151953	7154588	Promoter (2-3kb)	1167	234E-10	374E-07	Cdkn1a	2667	Hyper
chr16	16780936	16783311	Distal Intergenic	1367	303E-10	442E-07	Sh2d4b	-11849	Hyper
chr18	28335371	28338854	Promoter (<=1kb)	1224	312E-10	442E-07	Cd14	0	Hyper
chr12	33512636	33515385	Distal Intergenic	1347	479E-10	655E-07	Tmem120b	-25379	Hyper
chr16	16774657	16776490	Distal Intergenic	1573	880E-10	106E-06	Sh2d4b	-5570	Hyper
chr17	13370877	13372512	Distal Intergenic	1705	888E-10	106E-06	Gadd45g	6075	Hyper
chr15	37394256	37400632	Intron (NM_022597/64529, intron 1 of 9)	1043	122E-09	138E-06	Ctsb	4620	Hyper
chr5	141426543	141428627	Exon (NM_001108003/313806, exon 6 of 8)	1414	152E-09	157E-06	Rnf19b	8377	Hyper
chr18	61317914	61319768	Distal Intergenic	1343	181E-09	177E-06	Ptpn2	-23359	Hyper
chr2	174775354	174777887	Distal Intergenic	1363	201E-09	183E-06	Adam15	-10256	Hyper
chr20	6580564	6582743	Distal Intergenic	1144	209E-09	183E-06	Armc12	-5116	Hyper
chr19	23171935	23179293	Promoter (<=1kb)	0766	334E-09	278E-06	Junb	0	Hyper
chr10	36179144	36183636	Distal Intergenic	0858	425E-09	332E-06	Cdkn2aipnl	-47498	Hyper
chr11	84873550	84875883	Intron (NM_001100988/498119, intron 7 of 18)	1219	537E-09	410E-06	Cebpd	108880	Hyper
chr14	15659932	15662326	Intron (NM_001012034/305235, intron 2 of 9)	1769	737E-09	504E-06	Cxcl11	-27106	Hyper
chr4	159380739	159383079	Distal Intergenic	1447	802E-09	539E-06	Kcna5	-23443	Hyper
chr10	103107421	103109060	Distal Intergenic	1697	254E-08	132E-05	Birc5	33944	Hyper
chr13	70727870	70730176	Intron (NM_001170603/304900, intron 8 of 22)	1232	354E-08	171E-05	Mir488	44806	Hyper

chr11	84763575	84768990	Promoter (<=1kb)	0755	373E-08	172E-05	Cebpd	0	Hyper
chr10	85861507	85864350	Promoter (<=1kb)	0813	421E-08	190E-05	Stat3	0	Hyper
chr5	149122084	149124344	Promoter (<=1kb)	1428	552E-08	240E-05	C1qb	48	Hyper
chr13	83659934	83663858	Exon (NM_001011907/289218, exon 3 of 13)	0976	558E-08	240E-05	Ndufs2	3087	Hyper
chr10	36138760	36140484	Distal Intergenic	1278	864E-08	338E-05	Jade2	-18675	Hyper
chr9	1942012	1945025	Promoter (<=1kb)	1141	952E-08	368E-05	Tnfsf9	0	Hyper
chr10	16648996	16658159	Distal Intergenic	0696	125E-07	438E-05	Dusp1	-22330	Hyper
chr7	9543488	9561426	Promoter (<=1kb)	0572	144E-07	482E-05	Midn	0	Hyper
chr6	24318043	24323470	Promoter (<=1kb)	0738	196E-07	620E-05	Fosl2	0	Hyper
chr3	156472451	156474404	Distal Intergenic	1275	216E-07	666E-05	Cebpb	74416	Hyper
chr19	23468106	23477452	Promoter (<=1kb)	0642	231E-07	700E-05	Nacc1	0	Hyper
chr8	57989459	57994912	Promoter (<=1kb)	0783	232E-07	700E-05	Ulk3	0	Hyper
chr14	74181738	74183800	Intron (NM_001113365/360956, intron 2 of 14)	1087	240E-07	719E-05	Tbc1d14	4833	Hyper
chr1	77743022	77750464	Promoter (<=1kb)	0704	249E-07	738E-05	Hif3a	0	Hyper
chr14	1018135	1022988	Promoter (<=1kb)	0858	262E-07	759E-05	Fgfrl1	0	Hyper
chr10	103201975	103204185	Distal Intergenic	1358	273E-07	785E-05	Socs3	-5877	Hyper
chr2	183224426	183237083	3' UTR	0567	275E-07	785E-05	Mcl1	5206	Hyper
chr5	145439476	145441769	Promoter (<=1kb)	1218	295E-07	830E-05	Map3k6	0	Hyper
chr2	135691576	135696857	Promoter (<=1kb)	0721	338E-07	932E-05	Mgst2	0	Hyper
chr4	59684529	59688193	Distal Intergenic	0962	401E-07	107E-04	Mir29b1	-33462	Hyper
chr1	76690383	76695966	Distal Intergenic	0737	424E-07	111E-04	Bicra	-3996	Hyper
chr17	12280064	12285193	Promoter (<=1kb)	0796	428E-07	112E-04	Nfil3	0	Hyper
chr8	58003964	58006490	3' UTR	1160	461E-07	117E-04	Cplx3	3807	Hyper
chr17	13375042	13379893	Promoter (<=1kb)	0654	476E-07	119E-04	Gadd45g	0	Hyper
chr4	59710674	59717486	Distal Intergenic	0749	554E-07	133E-04	Mir29b1	-59607	Hyper
chr1	84467688	84471765	Promoter (<=1kb)	0839	677E-07	152E-04	Spred3	0	Hyper
chr2	140313167	140315843	Distal Intergenic	0984	737E-07	165E-04	Mab21l1	369671	Hyper
chr19	53043395	53045075	Intron (NM_175596/307940, intron 1 of 12)	0950	748E-07	166E-04	Disc1	28620	Hyper
chr3	156018218	156021706	3' UTR	0814	783E-07	170E-04	B4galt5	12277	Hyper
chr12	31561604	31564392	Exon (NM_001108334/360801, exon 13 of 47)	0830	815E-07	176E-04	Rflna	77925	Hyper
chr7	114718687	114721836	Promoter (<=1kb)	0944	833E-07	179E-04	Tspo	0	Hyper
chr9	42222896	42228068	Intron (NM_001106904/301363, intron 1 of 28)	0786	897E-07	188E-04	Map4k4	21276	Hyper
chr3	8682480	8685531	Distal Intergenic	0963	922E-07	191E-04	Kcnt1	-12474	Hyper

chr3	13711289	13721086	3' UTR	0544	976E-07	201E-04	Ptpa	21468	Hyper
chr8	119736359	119739888	Distal Intergenic	0827	109E-06	217E-04	Csrnp1	-25542	Hyper
chr2	175702652	175705225	Promoter (<=1kb)	0700	113E-06	223E-04	Slc39a1	0	Hyper
chr13	45667859	45680285	Intron (NM_017155/29290, intron 1 of 1)	0567	117E-06	226E-04	Adora1	14517	Hyper
chr7	93592491	93596917	Promoter (<=1kb)	0756	121E-06	233E-04	Myc	0	Hyper
chr3	115674466	115679330	Distal Intergenic	0880	123E-06	236E-04	LOC102550367	115804	Hyper
chr4	158150172	158153578	Promoter (<=1kb)	0741	138E-06	257E-04	Tnfrsf1a	0	Hyper
chr16	74679850	74681755	Distal Intergenic	1118	142E-06	262E-04	Kbtbd11	-56962	Hyper
chr1	197623768	197627076	Intron (NM_001025420/361680, intron 1 of 10)	0942	150E-06	270E-04	Lsp1	9081	Hyper
chr17	51836107	51839851	Distal Intergenic	0928	157E-06	281E-04	Arhgap12	247265	Hyper
chr1	181615628	181617922	Intron (NM_022715/64681, intron 1 of 14)	0937	171E-06	304E-04	Mvp	4414	Hyper
chr6	105118713	105122480	Promoter (<=1kb)	0689	172E-06	304E-04	Fos	0	Hyper
chr5	115913533	115916486	Distal Intergenic	0778	183E-06	318E-04	Jak1	-32133	Hyper
chr10	72869130	72874268	3' UTR	0744	192E-06	330E-04	Vezf1	9226	Hyper
chr14	6678361	6680218	Intron (NM_001270556/25272, intron 4 of 13)	1118	200E-06	339E-04	Arhgap24	169277	Hyper
chr4	170472559	170474937	Intron (NM_001376935/312812, intron 1 of 20)	1043	201E-06	339E-04	Eps8	11936	Hyper
chr2	183210053	183211593	Distal Intergenic	1216	206E-06	346E-04	Mcl1	-7627	Hyper
chrX	152215916	152219595	Promoter (1-2kb)	0937	225E-06	364E-04	G6pd	1268	Hyper
chr11	75710638	75714999	Distal Intergenic	0655	234E-06	375E-04	Mir28	260609	Hyper
chr14	15657306	15659321	Exon (NM_001012034/305235, exon 3 of 10)	1490	251E-06	395E-04	Cxcl11	-30111	Hyper
chr8	57939948	57943755	Promoter (<=1kb)	0724	256E-06	399E-04	Fam219b	0	Hyper
chr17	44675354	44685227	Intron (NM_001108415/361251, intron 4 of 21)	0617	278E-06	429E-04	Elmo1	137561	Hyper
chr4	11938029	11941867	Distal Intergenic	0779	283E-06	435E-04	Kmt2e	-210656	Hyper
chr10	103213986	103216345	Distal Intergenic	0833	286E-06	438E-04	Socs3	-17888	Hyper
chr4	158153745	158158153	Promoter (2-3kb)	0758	314E-06	470E-04	Tnfrsf1a	2851	Hyper
chr3	106162040	106165883	3' UTR	0813	314E-06	470E-04	Gchfr	3994	Hyper
chr15	7526184	7529511	Promoter (1-2kb)	0922	321E-06	477E-04	Nr1d2	1927	Hyper
chr1	197541717	197543729	Promoter (2-3kb)	1197	326E-06	482E-04	Ctsd	-2374	Hyper
chr18	28266059	28269049	Promoter (<=1kb)	1145	334E-06	491E-04	Eif4ebp3	0	Hyper
chr16	18869784	18872786	Intron (NM_001107304/306347, intron 1 of 11)	0968	338E-06	493E-04	Ell	18305	Hyper
chr5	149127558	149128451	Promoter (2-3kb)	2136	351E-06	500E-04	C1qc	2281	Hyper
chr16	16783476	16786053	3' UTR	1245	353E-06	500E-04	Sh2d4b	-14389	Hyper
chr10	71444743	71446283	Intron (NM_138839/192129, intron 7 of 11)	1323	366E-06	516E-04	Mir21	-39395	Hyper

chr20	40904666	40906839	Distal Intergenic	1177	375E-06	526E-04	Marcks	-213654	Hyper
chr4	157944215	157951984	Promoter (<=1kb)	0560	423E-06	579E-04	Iffo1	0	Hyper
chr7	14430112	14433398	Distal Intergenic	1022	445E-06	592E-04	Zfp414	9854	Hyper
chr15	45239595	45241649	Exon (NM_001007622/290354, exon 7 of 10)	1035	449E-06	595E-04	C15h8orf58	-6341	Hyper
chr20	3770914	3774838	Promoter (<=1kb)	0747	455E-06	600E-04	Clic1	0	Hyper
chr3	11839339	11862192	Promoter (<=1kb)	0442	457E-06	601E-04	Ralgds	0	Hyper
chr19	48610826	48614593	Distal Intergenic	0653	513E-06	659E-04	Gins2	24746	Hyper
chr20	6478808	6480780	Intron (NM_001012174/361810, intron 5 of 10)	0972	528E-06	672E-04	Tulp1	-54735	Hyper
chr15	98776466	98780990	Intron (NM_001105759/259237, intron 1 of 52)	0728	551E-06	695E-04	Dock9	102163	Hyper
chr17	64611301	64613158	Distal Intergenic	1233	558E-06	700E-04	Klf6	-62850	Hyper
chr15	39049596	39052464	Intron (NM_213626/305967, intron 39 of 41)	0999	572E-06	712E-04	Kif13b	141278	Hyper
chr14	78367383	78370820	Promoter (<=1kb)	0845	577E-06	712E-04	Pla2g3	0	Hyper
chr8	52437434	52440932	Promoter (<=1kb)	0876	617E-06	749E-04	Mir6329	0	Hyper
chr16	16790682	16793812	Exon (NM_001169127/306324, exon 4 of 9)	0908	652E-06	785E-04	Sh2d4b	-21595	Hyper
chr6	72834724	72839214	Distal Intergenic	0713	672E-06	794E-04	Nfkbia	22727	Hyper
chr1	155221294	155225269	Distal Intergenic	0761	750E-06	857E-04	Relt	-7098	Hyper
chr19	53045663	53046598	Intron (NM_175596/307940, intron 1 of 12)	1299	768E-06	869E-04	Disc1	30888	Hyper
chr18	53954836	53957316	Promoter (<=1kb)	0824	781E-06	877E-04	Smim3	0	Hyper
chr15	39039311	39048264	Intron (NM_213626/305967, intron 39 of 41)	0592	813E-06	902E-04	Kif13b	130993	Hyper
chr6	93159579	93162093	Distal Intergenic	0773	818E-06	903E-04	Snapc1	474794	Hyper
chr6	105146469	105148262	Distal Intergenic	1058	826E-06	908E-04	Fos	25299	Hyper
chr5	101180857	101183362	Promoter (<=1kb)	0966	837E-06	918E-04	Plin2	0	Hyper
chr7	109092173	109094981	Distal Intergenic	0738	863E-06	941E-04	Rbfox2	-37770	Hyper
chr3	14078530	14083040	3' UTR	0711	931E-06	990E-04	C3h9orf50	3938	Hyper
chr12	6122756	6124684	Intron (NM_001006956/288449, intron 3 of 10)	0970	937E-06	990E-04	Katnal1	19870	Hyper
chr2	135687809	135690332	Distal Intergenic	0911	947E-06	996E-04	Mgst2	-3224	Hyper
chr5	131803491	131811146	Promoter (<=1kb)	0592	951E-06	996E-04	Ptprf	0	Hyper
chr4	104579582	104581039	Distal Intergenic	1469	980E-06	101E-03	Capg	-19023	Hyper
chr10	54334695	54337086	Promoter (<=1kb)	0835	982E-06	101E-03	Shbg	0	Hyper
chr3	167808628	167812612	Distal Intergenic	0755	108E-05	109E-03	Gid8	-13524	Hyper
chr1	193882179	193884293	Promoter (<=1kb)	0854	117E-05	116E-03	Dpysl4	0	Hyper
chr19	39427999	39430125	Distal Intergenic	1249	117E-05	116E-03	Fa2h	-63846	Hyper
chr9	91615883	91617927	Distal Intergenic	1018	129E-05	125E-03	Lrrfip1	-25364	Hyper

chr5	117247636	117250882	Exon (NM_017031/24626, exon 2 of 15)	0792	133E-05	129E-03	Pde4b	254200	Hyper
chr1	220716839	220724051	3' UTR	0580	133E-05	129E-03	Klf9	16731	Hyper
chr5	131631562	131634922	Intron (NM_031697/64445, intron 2 of 11)	0775	142E-05	136E-03	St3gal3	35373	Hyper
chr3	156631756	156634043	Distal Intergenic	0864	151E-05	144E-03	Ptpn1	-4768	Hyper
chr7	62754290	62761056	Distal Intergenic	0495	154E-05	145E-03	Atp23	-24467	Hyper
chr1	78922421	78926057	Promoter (<=1kb)	0621	157E-05	147E-03	Vasp	0	Hyper
chr5	141100889	141103134	Promoter (<=1kb)	0807	157E-05	147E-03	A3galt2	0	Hyper
chr10	62924922	62927649	Promoter (<=1kb)	0706	159E-05	148E-03	Flot2	0	Hyper
chr3	14029380	14038956	Distal Intergenic	0498	185E-05	168E-03	C3h9orf50	48022	Hyper
chr1	117519694	117528432	Intron (NM_001109147/499171, intron 1 of 1)	0519	190E-05	172E-03	Klf13	8194	Hyper
chr7	109838842	109840968	Intron (NM_001127304/500904, intron 7 of 9)	1082	191E-05	172E-03	Ncf4	12822	Hyper
chr4	59690808	59695914	Distal Intergenic	0676	201E-05	179E-03	Mir29b1	-39741	Hyper
chr17	13342798	13344575	Distal Intergenic	1064	201E-05	179E-03	Gadd45g	34012	Hyper
chr2	61497040	61498367	Intron (NM_022940/65034, intron 1 of 22)	1096	202E-05	179E-03	Pdzd2	122779	Hyper
chr12	27390259	27392622	Distal Intergenic	0900	211E-05	184E-03	Sfswap	-210375	Hyper
chr10	36230577	36232339	Promoter (<=1kb)	0780	214E-05	186E-03	Cdkn2aipnl	0	Hyper
chr10	105906875	105912618	Promoter (<=1kb)	0536	218E-05	188E-03	Mafg	0	Hyper
chr19	48180822	48184902	Distal Intergenic	0794	219E-05	188E-03	6430548M08Rikl	-13307	Hyper
chr19	23491059	23498352	Promoter (<=1kb)	0519	227E-05	194E-03	Ier2	0	Hyper
chr16	7780891	7783827	Intron (NM_001107296/306274, intron 5 of 18)	0691	234E-05	198E-03	Ercc6	11944	Hyper
chr5	151215810	151217818	Promoter (<=1kb)	0811	238E-05	201E-03	Tmco4	0	Hyper
chr15	41060127	41065094	Intron (NM_001105717/25416, intron 3 of 13)	0629	242E-05	203E-03	Dpysl2	7763	Hyper
chr19	53026170	53028491	Intron (NM_175596/307940, intron 1 of 12)	0700	246E-05	205E-03	Disc1	11395	Hyper
chr12	22035776	22040305	Intron (NM_031727/65172, intron 2 of 15)	0606	246E-05	205E-03	Limk1	9079	Hyper
chr3	10989822	10993419	Promoter (<=1kb)	0635	248E-05	206E-03	Rxra	0	Hyper
chr11	69549422	69552007	Intron (NM_001034006/619382, intron 1 of 22)	0804	265E-05	218E-03	Acap2	10876	Hyper
chr10	71399605	71403803	Promoter (1-2kb)	0656	266E-05	218E-03	Mir21	1545	Hyper
chr4	167022680	167024977	Intron (NM_001037353/312777, intron 2 of 7)	1082	268E-05	219E-03	Etv6	173649	Hyper
chr1	106823242	106825069	Promoter (<=1kb)	0746	277E-05	225E-03	Nipa2	0	Hyper
chr1	40592494	40593679	Intron (NM_001108462/361472, intron 26 of 27)	1476	283E-05	228E-03	Akap12	-136444	Hyper
chr12	22172416	22177880	Intron (NM_021997/29264, intron 1 of 16)	0574	284E-05	228E-03	Clip2	9198	Hyper
chr6	31223661	31224961	Distal Intergenic	1019	285E-05	228E-03	Gdf7	-41214	Hyper
chr8	69811577	69812841	Distal Intergenic	1214	285E-05	228E-03	Ice2	-231729	Hyper

chr2	137215952	137217395	Intron (NM_001109183/499615, intron 2 of 3)	1069	288E-05	230E-03	Lhfpl6	89467	Hyper
chr4	59719315	59722198	Distal Intergenic	0802	289E-05	230E-03	Mir29b1	-68248	Hyper
chr10	55365576	55370289	Promoter (<=1kb)	0494	302E-05	237E-03	Pfn1	0	Hyper
chr3	154141058	154143134	3' UTR	1110	304E-05	238E-03	Slc13a3	61470	Hyper
chr2	92012493	92014508	Distal Intergenic	0852	310E-05	243E-03	Pag1	-42708	Hyper
chr4	58892228	58895156	Intron (NM_001172127/296956, intron 1 of 6)	0864	313E-05	243E-03	N5	7441	Hyper
chr6	130788715	130790799	Intron (NM_001037769/299341, intron 3 of 4)	0942	319E-05	247E-03	Coa8	16472	Hyper
chr12	31439668	31441763	Distal Intergenic	1041	333E-05	256E-03	Ncor2	-24825	Hyper
chr4	141066712	141067761	Distal Intergenic	1376	336E-05	257E-03	Setmar	20625	Hyper
chr1	22978851	22989224	Promoter (<=1kb)	0457	346E-05	262E-03	Sgk1	0	Hyper
chr16	74634231	74638171	Distal Intergenic	0588	349E-05	263E-03	Kbtbd11	-11343	Hyper
chr12	8324683	8327855	Intron (NM_001166576/360763, intron 1 of 8)	0673	361E-05	271E-03	Usp12	14606	Hyper
chr19	52170442	52172541	Distal Intergenic	0848	364E-05	272E-03	Cox6c-ps1	20929	Hyper
chr7	115769766	115772361	Distal Intergenic	0735	381E-05	281E-03	Prr5	-41593	Hyper
chr15	39075577	39078070	Distal Intergenic	0691	381E-05	281E-03	Kif13b	167259	Hyper
chr14	95031794	95036578	Distal Intergenic	0643	384E-05	282E-03	Lgalsl	-27295	Hyper
chr7	107910765	107917727	Promoter (<=1kb)	0596	389E-05	285E-03	Plec	0	Hyper
chr4	59681466	59684348	Distal Intergenic	0747	397E-05	288E-03	Mir29b1	-30399	Hyper
chr7	91205206	91209898	Promoter (<=1kb)	0607	410E-05	295E-03	LOC690120	0	Hyper
chr3	106260424	106261335	3' UTR	1354	416E-05	299E-03	Rhov	8144	Hyper
chr7	110911353	110913427	Promoter (<=1kb)	0991	430E-05	307E-03	Maff	0	Hyper
chr8	90781961	90784286	Intron (NM_001108175/315880, intron 4 of 13)	1000	436E-05	310E-03	Tbc1d2b	30546	Hyper
chr8	108227772	108230982	Promoter (<=1kb)	0608	452E-05	319E-03	Rassf1	0	Hyper
chr10	4640916	4642601	Distal Intergenic	0957	452E-05	319E-03	Litaf	-40412	Hyper
chr3	156012226	156013582	Distal Intergenic	1233	463E-05	323E-03	B4galt5	20401	Hyper
chr1	202781239	202783889	Promoter (<=1kb)	0651	469E-05	327E-03	Efemp2	0	Hyper
chr1	155774058	155786354	Distal Intergenic	0496	476E-05	330E-03	Pde2a	-37236	Hyper
chr10	87202235	87203330	Promoter (2-3kb)	1256	486E-05	336E-03	Hrob	-2719	Hyper
chr1	86323883	86328098	Distal Intergenic	0557	493E-05	341E-03	Fxyd3	-11564	Hyper
chr1	187847377	187849064	Distal Intergenic	1062	504E-05	347E-03	Ctbp2	-35529	Hyper
chr6	121875169	121876144	Distal Intergenic	1366	515E-05	352E-03	Moap1	8356	Hyper
chr19	49643834	49647900	Intron (NM_001044259/498959, intron 1 of 8)	0613	528E-05	359E-03	Fbxo31	8110	Hyper
chr5	159886522	159888935	Distal Intergenic	0737	530E-05	359E-03	Rbp7	5403	Hyper

chr16	7578017	7580280	Promoter (1-2kb)	0788	544E-05	365E-03	Ogdhl	-1623	Hyper
chr5	129914774	129916633	Promoter (<=1kb)	0826	564E-05	375E-03	Mast2	0	Hyper
chr20	7382371	7385957	3' UTR	0590	576E-05	383E-03	Pi16	5924	Hyper
chr3	152370255	152372191	Intron (NM_153469/266709, intron 1 of 2)	0868	596E-05	393E-03	Pkig	39716	Hyper
chr3	77281346	77283562	Intron (NM_001013918/295930, intron 3 of 8)	0812	597E-05	393E-03	Arfgap2	44937	Hyper
chr3	15004405	15008106	Intron (NM_001100850/311860, intron 1 of 10)	0710	603E-05	397E-03	Abl1	24552	Hyper
chr6	119760438	119762092	Distal Intergenic	0947	607E-05	398E-03	Ttc7b	-55251	Hyper
chr13	67258832	67260637	Distal Intergenic	0862	609E-05	399E-03	Ier5	11589	Hyper
chr1	83706191	83709920	Intron (NM_001107498/308473, intron 1 of 11)	0650	613E-05	400E-03	Samd4b	3933	Hyper
chr5	58181262	58182405	Distal Intergenic	1368	614E-05	401E-03	Ccin	-24271	Hyper
chr6	11373222	11374478	Distal Intergenic	1077	615E-05	401E-03	Pkdcc	90524	Hyper
chr14	80887730	80889899	Intron (NM_001042354/24245, intron 2 of 20)	0831	620E-05	402E-03	Camk2b	44095	Hyper
chr11	84771985	84773875	Exon (NM_001100988/498119, exon 16 of 19)	1046	626E-05	405E-03	Cebpd	7315	Hyper
chr11	84817577	84820541	Intron (NM_001100988/498119, intron 9 of 18)	0703	637E-05	410E-03	Cebpd	52907	Hyper
chr3	60602412	60605059	Intron (NM_031789/83619, intron 1 of 4)	0849	639E-05	411E-03	Nfe2l2	16612	Hyper
chr10	98159524	98171984	Distal Intergenic	0447	703E-05	446E-03	Mir297	44560	Hyper
chr1	196155574	196158052	Distal Intergenic	0733	707E-05	448E-03	B4galnt4	-13342	Hyper
chr4	59672168	59679228	Distal Intergenic	0558	712E-05	450E-03	Mir29b1	-21101	Hyper
chr17	34600434	34604739	Promoter (<=1kb)	0543	725E-05	455E-03	E2f3	0	Hyper
chr12	41468647	41472564	3' UTR	0615	726E-05	456E-03	Unc119b	-6244	Hyper
chr6	138346160	138347883	Distal Intergenic	1062	730E-05	457E-03	Mir153	182565	Hyper
chr10	99759358	99762435	Promoter (<=1kb)	0699	742E-05	462E-03	Dnai2	0	Hyper
chr10	102857818	102859545	Distal Intergenic	0922	767E-05	477E-03	Tmc8	-140799	Hyper
chr1	256251843	256252817	Intron (NM_001372054/307989, intron 1 of 24)	1181	788E-05	488E-03	Ablim1	23678	Hyper
chr20	18869377	18870431	Intron (NM_031805/361833, intron 1 of 44)	1600	789E-05	488E-03	Ank3	59698	Hyper
chr14	46744436	46747061	Intron (NM_001107216/305367, intron 2 of 31)	0732	831E-05	511E-03	Arap2	25676	Hyper
chr5	131800897	131803345	Exon (NM_019249/360406, exon 2 of 31)	0690	850E-05	516E-03	Ptprf	6678	Hyper
chr11	68848371	68856661	Distal Intergenic	0447	869E-05	523E-03	LOC102549772	10347	Hyper
chr10	34681512	34683338	Distal Intergenic	0838	887E-05	532E-03	Hnrnph1	-12217	Hyper
chr1	198234962	198249110	Promoter (<=1kb)	0408	902E-05	539E-03	Cd81	0	Hyper
chr6	41646589	41649596	Exon (NM_053795/116478, exon 11 of 30)	0842	907E-05	540E-03	Kidins220	28295	Hyper
chr13	64670256	64675859	Exon (NM_001105957/289080, exon 6 of 20)	0602	912E-05	540E-03	Tsen15	-164639	Hyper
chr5	149133535	149136488	Promoter (<=1kb)	0755	912E-05	540E-03	C1qa	0	Hyper

chr1	7271192	7273829	Distal Intergenic	0619	921E-05	542E-03	Stx11	46231	Hyper
chr12	20802415	20803816	Distal Intergenic	0693	940E-05	549E-03	Hspb1	-6740	Hyper
chr8	41529810	41532734	Promoter (<=1kb)	0686	957E-05	556E-03	Ubash3b	0	Hyper
chr19	53213440	53215593	Intron (NM_175596/307940, intron 12 of 12)	0659	962E-05	558E-03	Disc1	198665	Hyper
chr20	42851639	42853245	5' UTR	1044	980E-05	562E-03	Fyn	83580	Hyper
chr1	185496996	185500843	Promoter (<=1kb)	0661	981E-05	562E-03	Htra1	0	Hyper
chr3	167815698	167818284	Distal Intergenic	0568	984E-05	562E-03	Gid8	-7852	Hyper
chr16	9883801	9885426	Intron (NM_001277165/498587, intron 8 of 13)	0748	998E-05	568E-03	Ldb3	33023	Hyper
chr3	11024910	11031175	Intron (NM_012805/25271, intron 1 of 9)	0518	101E-04	571E-03	Rxra	34994	Hyper
chr10	101338235	101343068	Promoter (<=1kb)	0587	102E-04	574E-03	Trim47	0	Hyper
chr12	41478794	41480756	Promoter (<=1kb)	0820	102E-04	574E-03	Unc119b	0	Hyper
chr1	117564181	117569383	Distal Intergenic	0524	102E-04	574E-03	Klf13	-27555	Hyper
chr8	19829651	19835389	Promoter (<=1kb)	0538	102E-04	576E-03	Cdkn2d	0	Hyper
chr3	154887635	154904864	Promoter (<=1kb)	0397	103E-04	576E-03	Sulf2	0	Hyper
chr8	57999441	58001905	Distal Intergenic	0832	106E-04	592E-03	Ulk3	6410	Hyper
chr10	61459790	61462105	Distal Intergenic	0935	109E-04	601E-03	Abr	-54335	Hyper
chr9	11455408	11459287	Distal Intergenic	0670	110E-04	604E-03	Mocs1	108503	Hyper
chr11	82577892	82587385	3' UTR	0472	112E-04	615E-03	Txnrd2	-9736	Hyper
chr4	179600583	179601908	Distal Intergenic	1098	113E-04	619E-03	Stk38l	-32367	Hyper
chr12	31582450	31589265	Exon (NM_001108334/360801, exon 18 of 47)	0496	113E-04	619E-03	Rflna	53052	Hyper
chr20	28143168	28152667	Distal Intergenic	0431	114E-04	620E-03	Chst3	-22201	Hyper
chr3	16164374	16166224	Distal Intergenic	0837	115E-04	625E-03	Niban2	-8450	Hyper
chr19	11123428	11130177	Intron (NM_017327/50664, intron 2 of 8)	0499	116E-04	626E-03	Gnao1	62316	Hyper
chr8	119713204	119718032	Promoter (2-3kb)	0553	118E-04	630E-03	Csrnp1	-2387	Hyper
chr7	1335859	1341048	Promoter (<=1kb)	0476	118E-04	630E-03	Cd63	0	Hyper
chr13	92137026	92150715	Intron (NM_019312/54260, intron 2 of 7)	0417	118E-04	631E-03	Itpkb	67810	Hyper
chr12	20213995	20215920	Intron (NM_053860/116639, intron 2 of 22)	1193	118E-04	631E-03	Cux1	106684	Hyper
chr1	97370853	97373268	Promoter (<=1kb)	0736	120E-04	635E-03	Ldha	0	Hyper
chr13	84740975	84744459	Exon (NM_012505/24212, exon 8 of 23)	0746	120E-04	638E-03	Atp1a2	10085	Hyper
chr6	87670568	87672383	Promoter (<=1kb)	0903	121E-04	639E-03	Dnaaf2	-301	Hyper
chr20	7460988	7463250	Distal Intergenic	0763	121E-04	639E-03	Fgd2	36187	Hyper
chr11	44798748	44801505	5' UTR	0632	121E-04	640E-03	Nfkbiz	16072	Hyper
chr13	84830285	84835030	3' UTR	0661	122E-04	640E-03	Pigm	-3299	Hyper

chr4	146777930	146780099	3' UTR	1130	124E-04	652E-03	Vhl	5447	Hyper
chr10	102469606	102471889	Intron (NM_176856/83788, intron 2 of 11)	0910	125E-04	657E-03	Septin9	-5651	Hyper
chr2	30499668	30502266	Distal Intergenic	0752	126E-04	659E-03	Tnpo1	-230283	Hyper
chr10	56058749	56061299	Distal Intergenic	0691	129E-04	673E-03	Nlrp1a	-233569	Hyper
chr6	106524616	106532531	Promoter (<=1kb)	0429	130E-04	675E-03	Irf2bpl	0	Hyper
chr2	215743190	215744986	Distal Intergenic	0814	134E-04	689E-03	Larp7	267823	Hyper
chr1	204460831	204462741	3' UTR	0876	134E-04	689E-03	Rcor2	6088	Hyper
chr3	14471084	14473566	Exon (NM_001107828/311857, exon 14 of 18)	0837	134E-04	689E-03	Gpr107	36361	Hyper
chr19	33422563	33426073	Intron (NM_001011927/291969, intron 1 of 7)	0727	136E-04	699E-03	Atp6v0d1	21262	Hyper
chr14	40347147	40350584	Distal Intergenic	0720	136E-04	699E-03	Shisa3	217660	Hyper
chr8	109261706	109266768	Promoter (<=1kb)	0465	136E-04	699E-03	Ndufaf3	0	Hyper
chr7	95739363	95741400	Intron (NM_001126267/299909, intron 1 of 12)	0961	138E-04	703E-03	Cyrib	19188	Hyper
chr7	125727052	125731182	Promoter (<=1kb)	0578	139E-04	707E-03	Twf1	0	Hyper
chr12	38224161	38225865	Distal Intergenic	0911	139E-04	707E-03	Spring1	103273	Hyper
chr9	36877646	36884560	Distal Intergenic	0559	140E-04	713E-03	Prss40	-106667	Hyper
chr5	9226648	9227818	Promoter (<=1kb)	0910	141E-04	713E-03	Ppp1r42	0	Hyper
chr6	31373641	31376268	Distal Intergenic	0777	142E-04	720E-03	Rhob	-7718	Hyper
chr13	43998719	44003750	3' UTR	0628	145E-04	730E-03	Cntn2	-22834	Hyper
chr9	1559081	1561042	Distal Intergenic	0954	146E-04	734E-03	Ndufa11	8594	Hyper
chr18	71300521	71302693	Downstream (<=300bp)	0662	149E-04	744E-03	Atp5f1a	8115	Hyper
chr9	93474946	93480266	Promoter (<=1kb)	0560	149E-04	744E-03	Rnpepl1	0	Hyper
chr6	105234258	105236277	Distal Intergenic	0963	150E-04	744E-03	Jdp2	-34863	Hyper
chr10	83272153	83274427	Intron (NM_147142/257650, intron 2 of 3)	0819	150E-04	744E-03		4821	Hyper
chr1	4959674	4961000	Promoter (<=1kb)	1239	150E-04	744E-03	Rab32	3	Hyper
chr18	60942744	60944988	Promoter (<=1kb)	0882	150E-04	745E-03	Tubb6	0	Hyper
chr10	62334054	62353143	Promoter (<=1kb)	0383	152E-04	752E-03	Git1	0	Hyper
chr16	17198915	17199985	Distal Intergenic	1081	157E-04	771E-03	Sin3b	-38937	Hyper
chr2	169553460	169554947	Exon (NM_001108552/361970, exon 2 of 12)	1131	157E-04	771E-03	Trim2	9475	Hyper
chr16	1192075	1198438	Intron (NM_001108393/361103, intron 6 of 23)	0511	157E-04	771E-03	Ppif	-58759	Hyper
chr3	156459982	156461146	Distal Intergenic	1028	158E-04	777E-03	Cebpb	61947	Hyper
chr7	109380223	109388446	Intron (NM_013194/25745, intron 2 of 39)	0484	159E-04	777E-03	Myh9	7914	Hyper
chr2	33990861	33992562	Distal Intergenic	0969	161E-04	784E-03	Cd180	134870	Hyper
chr13	44144787	44152422	Promoter (<=1kb)	0534	161E-04	784E-03	Nfasc	0	Hyper

chr7	93661941	93663558	Distal Intergenic	1061	163E-04	788E-03	Myc	68236	Hyper
chr13	83247730	83248560	Promoter (1-2kb)	1426	164E-04	788E-03	Fcgr3a	-1345	Hyper
chr1	199693859	199696370	Promoter (<=1kb)	0763	164E-04	788E-03		0	Hyper
chr9	75876993	75890095	Promoter (<=1kb)	0370	164E-04	788E-03	Tmbim1	0	Hyper
chr7	21029518	21031092	Distal Intergenic	1114	165E-04	788E-03	Hcfc2	-9736	Hyper
chr9	1514226	1515936	Distal Intergenic	0864	166E-04	790E-03	Vmac	33639	Hyper
chr14	77454401	77461639	Promoter (<=1kb)	0456	167E-04	796E-03	Ctbp1	0	Hyper
chr10	92689071	92695413	Exon (NM_080692/140725, exon 2 of 4)	0506	168E-04	798E-03	Cacng1	-23459	Hyper
chr1	50931716	50935203	Distal Intergenic	0564	171E-04	809E-03	Pabpc6	-110061	Hyper
chr3	153998896	154000654	Distal Intergenic	0879	174E-04	824E-03	Slc35c2	22824	Hyper
chr5	151460338	151464403	Intron (NM_001005903/298584, intron 1 of 8)	0591	175E-04	826E-03	Capzb	24634	Hyper
chr1	117500592	117519365	3' UTR	0379	177E-04	834E-03	Klf13	17261	Hyper
chr8	115852540	115854354	Intron (NM_031132/81810, intron 1 of 7)	0743	179E-04	840E-03	Tgfbr2	28884	Hyper
chr19	45339038	45342385	Intron (NM_001163273/292051, intron 1 of 20)	0741	184E-04	860E-03	Cmip	34441	Hyper
chr10	62653623	62658605	Promoter (<=1kb)	0513	185E-04	861E-03	Myo18a	0	Hyper
chr5	145896254	145897797	Intron (NM_001010966/366478, intron 2 of 3)	0982	185E-04	861E-03	Pigv	3736	Hyper
chr10	43987942	43989414	Promoter (<=1kb)	0807	187E-04	870E-03	Mrpl55	0	Hyper
chr1	68908759	68914788	Promoter (<=1kb)	0471	189E-04	876E-03	Ssc5d	0	Hyper
chr18	28271110	28272994	Promoter (<=1kb)	0690	190E-04	877E-03	Sra1	0	Hyper
chr16	7551784	7554074	Distal Intergenic	0707	193E-04	888E-03	Ogdhl	-27829	Hyper
chr15	29093998	29098159	Promoter (<=1kb)	0539	193E-04	888E-03	Irf9	0	Hyper
chr2	102435260	102437899	Promoter (1-2kb)	0719	204E-04	926E-03	Cp	-1725	Hyper
chr1	154892347	154895821	Distal Intergenic	0653	204E-04	926E-03	Coa4	10131	Hyper
chr9	1095779	1101766	Distal Intergenic	0511	205E-04	927E-03	Fem1a	4084	Hyper
chr3	69872724	69874297	Promoter (<=1kb)	0910	208E-04	934E-03	Ube2l6	0	Hyper
chr1	98963499	98965095	Intron (NM_138529/171563, intron 1 of 37)	1174	213E-04	950E-03	Nav2	5101	Hyper
chr7	131820944	131828597	Distal Intergenic	0448	215E-04	954E-03	Galnt6	-5439	Hyper
chr7	109397009	109402511	Promoter (<=1kb)	0490	216E-04	958E-03	Myh9	-649	Hyper
chr5	138953233	138956522	Distal Intergenic	0666	222E-04	976E-03	Psmb2	-14042	Hyper
chr1	80863926	80871842	Promoter (<=1kb)	0425	223E-04	979E-03	Cic	0	Hyper
chr16	18672347	18676772	Promoter (<=1kb)	0573	225E-04	984E-03	Ifi30	0	Hyper
chr3	154881141	154887429	5' UTR	0520	227E-04	988E-03	Sulf2	16986	Hyper
chr9	102991644	102997240	Intron (NM_001108235/316663, intron 6 of 8)	0496	228E-04	988E-03	Fbxl17	208456	Hyper

chr15	18261282	18262569	Distal Intergenic	1089	234E-04	101E-02	Ptger2	43997	Hyper
chr16	69464658	69467081	Exon (NM_017223/29502, exon 10 of 11)	0632	236E-04	102E-02	Vdac3	29629	Hyper
chr2	45052624	45055504	Distal Intergenic	0746	239E-04	102E-02	Cspg4b	112544	Hyper
chr3	165403087	165405845	Intron (NM_214459/362284, intron 7 of 11)	0733	241E-04	103E-02	Phactr3	59813	Hyper
chr12	124413	127654	Promoter (<=1kb)	0684	244E-04	104E-02	N4bp2l1	0	Hyper
chr1	182658508	182660142	Promoter (<=1kb)	1085	246E-04	104E-02	Itgam	0	Hyper
chr7	131292997	131297029	Distal Intergenic	0570	247E-04	105E-02	Atf1	-64933	Hyper
chr10	45407015	45411329	Promoter (<=1kb)	0496	251E-04	105E-02	Flii	0	Hyper
chr14	83304474	83306614	Intron (NM_001170459/360980, intron 2 of 28)	0808	253E-04	106E-02	Tns3	37524	Hyper
chr17	43556478	43557907	Distal Intergenic	0961	255E-04	107E-02	Trim27	-5845	Hyper
chr16	1269392	1275396	3' UTR	0501	257E-04	108E-02	Ppif	12195	Hyper
chr5	165792093	165794206	Distal Intergenic	0584	258E-04	108E-02	Faap20	-14451	Hyper
chr3	8103291	8105150	Promoter (<=1kb)	0977	260E-04	108E-02	Lrrc26	930	Hyper
chr4	30663725	30665760	Intron (NM_001191861/114483, intron 4 of 6)	0853	268E-04	111E-02	Fam133b	-47702	Hyper
chr10	63970455	63972711	Intron (NM_001382488/108348076, intron 13 of 17)	0798	269E-04	112E-02	Lgals9	-40410	Hyper
chr18	27469834	27474799	Distal Intergenic	0460	272E-04	113E-02	Cxxc5	14516	Hyper
chr17	4918689	4920435	Distal Intergenic	0738	276E-04	114E-02	Isca1	13398	Hyper
chr10	53822412	53824711	Promoter (<=1kb)	0695	278E-04	114E-02	Hes7	-863	Hyper
chr6	106621558	106625517	Distal Intergenic	0495	279E-04	115E-02	Zdhhc22	13352	Hyper
chr1	96057929	96062624	3' UTR	0583	280E-04	115E-02	Bcat2	15258	Hyper
chr6	106480399	106484776	Distal Intergenic	0537	280E-04	115E-02	Irf2bpl	45625	Hyper
chr12	20105635	20109320	Promoter (<=1kb)	0518	282E-04	115E-02	Cux1	0	Hyper
chr5	130597289	130600131	Distal Intergenic	0509	282E-04	115E-02	Plk3	12186	Hyper
chr3	152454668	152456485	Distal Intergenic	0898	284E-04	116E-02	Ada	-31814	Hyper
chr5	144929288	144931582	3' UTR	0747	286E-04	116E-02	Xkr8	13613	Hyper
chr3	68841811	68847027	Distal Intergenic	0518	286E-04	116E-02	Zc3h15	97103	Hyper
chr3	22283585	22289393	Promoter (<=1kb)	0472	290E-04	117E-02	Nek6	0	Hyper
chr12	31537932	31543038	Exon (NM_001108334/360801, exon 6 of 47)	0585	291E-04	118E-02	Ncor2	71344	Hyper
chr2	120239601	120240484	Intron (NM_019305/54250, intron 1 of 2)	1275	293E-04	118E-02	Fgf2	3273	Hyper
chr7	133103208	133104854	Distal Intergenic	0784	297E-04	120E-02	Krt8	26802	Hyper
chr5	153964711	153973616	Intron (NM_001191767/313667, intron 1 of 19)	0413	298E-04	120E-02	Plekhm2	4790	Hyper
chr20	11388242	11390157	Distal Intergenic	0620	300E-04	120E-02	Pofut2	-10454	Hyper
chr10	62360361	62364496	Promoter (<=1kb)	0584	302E-04	121E-02	Tp53i13	0	Hyper

chr8	76378295	76381378	Promoter (<=1kb)	0569	303E-04	121E-02	Lysmd2	0	Hyper
chr10	86393023	86397466	Promoter (<=1kb)	0509	303E-04	121E-02	Vat1	0	Hyper
chr11	65168060	65169820	Intron (NM_001107091/303901, intron 1 of 21)	0919	307E-04	122E-02	Sema5b	55491	Hyper
chr5	138443460	138445301	Promoter (<=1kb)	0780	307E-04	122E-02	Sh3d21	0	Hyper
chr6	39363092	39370374	Intron (NM_001012111/313977, intron 1 of 20)	0438	307E-04	122E-02	Lpin1	9802	Hyper
chr10	105310061	105324789	Promoter (2-3kb)	0407	308E-04	122E-02	Aatk	2323	Hyper
chr1	155730261	155731621	Intron (NM_001013069/293150, intron 2 of 6)	1157	310E-04	123E-02	Stard10	7563	Hyper
chr1	234092288	234095106	Intron (NM_001129882/681178, intron 1 of 8)	0607	311E-04	123E-02	Pcgf5	28434	Hyper
chr5	116119483	116121416	Distal Intergenic	0892	317E-04	125E-02	Dnajc6	-9260	Hyper
chr1	85677835	85679420	Promoter (<=1kb)	0933	317E-04	125E-02	Hcst	0	Hyper
chr13	79436700	79438666	Promoter (<=1kb)	0689	324E-04	128E-02	Uck2	0	Hyper
chr10	62671274	62683958	Intron (NM_001172137/360570, intron 2 of 41)	0368	329E-04	129E-02	Myo18a	16993	Hyper
chr17	51919962	51921199	Distal Intergenic	1227	332E-04	130E-02	Zeb1	194015	Hyper
chr8	20024638	20027391	Exon (NM_013199/25751, exon 6 of 21)	0784	332E-04	130E-02	Tmed1	35121	Hyper
chr15	34907800	34909566	Distal Intergenic	0752	333E-04	130E-02	C1qtnf9	-5763	Hyper
chr13	45439215	45441596	Distal Intergenic	0711	333E-04	130E-02	Fmod	-51921	Hyper
chr5	155633116	155634315	Promoter (1-2kb)	0990	335E-04	130E-02	Pdpn	1341	Hyper
chr2	135699623	135703649	Intron (NM_001106430/295037, intron 2 of 4)	0613	335E-04	130E-02	Mgst2	6067	Hyper
chr14	104524606	104529145	Intron (NM_001025140/498433, intron 3 of 46)	0563	336E-04	130E-02	Psme4	18890	Hyper
chr16	1126962	1127990	Intron (NM_001108393/361103, intron 3 of 23)	1195	337E-04	130E-02	Mir3075	25899	Hyper
chr2	195935539	195942767	Intron (NM_031767/83576, intron 1 of 19)	0432	339E-04	131E-02	Sort1	11177	Hyper
chr9	74424071	74425369	Intron (NM_013122/25662, intron 1 of 3)	1133	341E-04	132E-02	Igfbp2	8497	Hyper
chr1	204463106	204465395	Exon (NM_021699/60328, exon 16 of 17)	0747	344E-04	132E-02	Rcor2	8363	Hyper
chr13	44628182	44629307	Distal Intergenic	1001	347E-04	133E-02	Ppp1r15b	50250	Hyper
chr14	83203000	83206535	Intron (NM_001170459/360980, intron 18 of 28)	0597	352E-04	134E-02	Tns3	137603	Hyper
chr14	78996659	78997801	Promoter (2-3kb)	1207	356E-04	135E-02	Sec14l2	-2919	Hyper
chr1	83663181	83679285	Promoter (<=1kb)	0342	356E-04	136E-02	Plekhg2	0	Hyper
chr2	225569910	225570974	Distal Intergenic	1124	359E-04	136E-02	Emcn	-320126	Hyper
chr3	156397384	156401463	Promoter (<=1kb)	0600	366E-04	138E-02	Cebpb	0	Hyper
chr6	98622810	98624039	Distal Intergenic	1007	368E-04	139E-02	Zfp36l1	311709	Hyper
chr5	72601171	72603369	Promoter (1-2kb)	0864	368E-04	139E-02	Akap2	-1460	Hyper
chr2	27852880	27854714	Intron (NM_138516/171525, intron 3 of 14)	0777	368E-04	139E-02	Polk	27599	Hyper
chr1	136159840	136161724	Intron (NM_031238/81921, intron 1 of 8)	0849	370E-04	139E-02	Sh3gl3	35338	Hyper

chr20	9535641	9537879	Intron (NM_138543/191569, intron 7 of 18)	0807	372E-04	140E-02	Wdr4		65523	Hyper
chr10	102465572	102469240	Intron (NM_176856/83788, intron 2 of 11)	0576	383E-04	143E-02	Septin9		-8300	Hyper
chr7	93758683	93759747	Distal Intergenic	1162	383E-04	143E-02	Myc		164978	Hyper
chr9	25838179	25840682	Promoter (1-2kb)	0636	384E-04	143E-02	Ogfrl1		1637	Hyper
chr9	93716250	93717796	Distal Intergenic	0882	385E-04	143E-02	Mab21l4		-15744	Hyper
chr18	71309186	71312121	Promoter (<=1kb)	0626	387E-04	144E-02	Pstpip2		0	Hyper
chr9	57192509	57193457	Intron (NM_053456/84587, intron 3 of 5)	1198	388E-04	144E-02	Plcl1		290936	Hyper
chr10	94412946	94414107	Distal Intergenic	0915	389E-04	144E-02	Amz2		11367	Hyper
chr7	113216061	113217819	Distal Intergenic	0643	389E-04	144E-02	L3mbtl2		29644	Hyper
chr2	183218289	183221777	Promoter (<=1kb)	0489	392E-04	145E-02	Mcl1		0	Hyper
chr9	90162745	90166550	Distal Intergenic	0599	393E-04	145E-02	Agap1		-21475	Hyper
chr5	136960817	136963114	3' UTR	0698	396E-04	146E-02	Sf3a3		-4599	Hyper
chr6	25462511	25464122	Promoter (<=1kb)	0890	396E-04	146E-02	Emilin1		0	Hyper
chr5	136192254	136193435	Distal Intergenic	1101	400E-04	147E-02	Rragc		43954	Hyper
chr7	93702853	93705007	Distal Intergenic	0652	401E-04	147E-02	Myc		109148	Hyper
chr5	145161183	145163571	Distal Intergenic	0577	404E-04	148E-02	Fam76a		-25629	Hyper
chr17	27000433	27003674	Promoter (<=1kb)	0585	407E-04	148E-02	Rreb1		0	Hyper
chr2	61539463	61541032	Intron (NM_022940/65034, intron 1 of 22)	0939	408E-04	148E-02	Pdzd2		80114	Hyper
chr1	90449665	90453384	Distal Intergenic	0634	409E-04	149E-02	Uri1		251272	Hyper
chr8	20896562	20897996	Distal Intergenic	0981	409E-04	149E-02	Rp9		107179	Hyper
chr19	13455720	13457014	Distal Intergenic	0800	410E-04	149E-02	Hmox1		-10244	Hyper
chr5	21866762	21868235	Intron (NM_001107906/312974, intron 1 of 37)	0965	411E-04	149E-02	Chd7		54752	Hyper
chr10	54130034	54136081	Promoter (<=1kb)	0422	412E-04	149E-02	Kdm6b		-234	Hyper
chr16	4911755	4914001	Promoter (<=1kb)	0627	417E-04	150E-02	Cacna2d3		0	Hyper
chr5	22197200	22198446	Distal Intergenic	0991	418E-04	151E-02	Lnc056		-34083	Hyper
chr5	47663366	47664274	Distal Intergenic	1175	428E-04	153E-02	Pnrc1		-13251	Hyper
chr18	67337652	67339521	Promoter (1-2kb)	0881	432E-04	153E-02	Elac1		1415	Hyper
chr2	135684004	135687630	Distal Intergenic	0510	437E-04	155E-02	Mgst2		-5926	Hyper
chr5	76141439	76147233	Intron (NM_019340/54293, intron 10 of 15)	0531	440E-04	155E-02	Rgs3		62947	Hyper
chr3	130045513	130048195	Intron (NM_001107783/311478, intron 22 of 25)	0664	440E-04	155E-02	Kif16b		205824	Hyper
chr2	183544401	183547386	3' UTR	0687	441E-04	155E-02	Plekho1		4986	Hyper
chr4	78009743	78011584	Promoter (<=1kb)	0779	444E-04	156E-02	Gpnmb		0	Hyper
chr19	48583146	48589294	Distal Intergenic	0419	449E-04	157E-02	Gins2		50045	Hyper

chr9	66292714	66294044	Distal Intergenic	0954	450E-04	157E-02	Cryge	138477	Hyper
chr12	37989222	37990888	Distal Intergenic	0930	452E-04	157E-02	Spring1	338250	Hyper
chr2	233608331	233610495	Intron (NM_001100518/292155, intron 1 of 6)	0690	453E-04	157E-02	Hs2st1	31274	Hyper
chr4	71236164	71241727	Promoter (<=1kb)	0431	453E-04	157E-02	Zyx	0	Hyper
chr5	145840219	145841692	Exon (NM_001039339/362613, exon 3 of 10)	0897	453E-04	157E-02	Zdhhc18	18204	Hyper
chr17	17446343	17451089	Exon (NM_001108881/364681, exon 4 of 8)	0654	458E-04	158E-02	Rnf144b	111671	Hyper
chr3	156467942	156469021	Distal Intergenic	1259	459E-04	158E-02	Cebpb	69907	Hyper
chr11	31564067	31567399	Distal Intergenic	0649	466E-04	161E-02	C11h21orf140	-11810	Hyper
chr7	20866548	20869446	Promoter (<=1kb)	0522	471E-04	162E-02	Txnrd1	0	Hyper
chr14	5924586	5926907	Intron (NM_001107206/305152, intron 3 of 19)	0786	474E-04	162E-02	Aff1	37044	Hyper
chr5	161267406	161269413	Distal Intergenic	0716	482E-04	165E-02	Errfi1	-54592	Hyper
chr6	127401883	127406821	Intron (NM_001025741/362783, intron 3 of 21)	0558	489E-04	166E-02	Eml1	32624	Hyper
chr11	76851116	76863551	Promoter (<=1kb)	0351	495E-04	168E-02	Bcl6	0	Hyper
chr18	69081330	69082139	Intron (NM_001108891/364900, intron 3 of 4)	1024	501E-04	169E-02	Ctif	66752	Hyper
chr10	36119438	36125692	Promoter (<=1kb)	0385	502E-04	169E-02	Jade2	0	Hyper
chr16	19523892	19527397	Promoter (<=1kb)	0478	509E-04	171E-02	Tssk6	0	Hyper
chr3	11862496	11880333	3' UTR	0360	511E-04	171E-02	Ralgds	3068	Hyper
chr1	82524531	82527648	Promoter (<=1kb)	0519	512E-04	171E-02	Coq8b	0	Hyper
chr9	50676604	50677947	Intron (NM_001108795/363228, intron 4 of 8)	1237	513E-04	172E-02	Tmeff2	55528	Hyper
chr5	146572981	146575679	3' UTR	0640	516E-04	172E-02	Slc30a2	12518	Hyper
chr11	68152456	68156019	Promoter (<=1kb)	0538	518E-04	173E-02	Tnk2	0	Hyper
chr10	16610117	16611522	Distal Intergenic	1144	522E-04	173E-02	Dusp1	-68967	Hyper
chr12	34260276	34262012	Intron (NM_001107141/304488, intron 2 of 5)	0884	526E-04	174E-02	Pptc7	26327	Hyper
chr20	9145385	9149383	Intron (NM_053502/85264, intron 2 of 14)	0523	527E-04	174E-02	Abcg1	18698	Hyper
chr13	94210607	94211669	Distal Intergenic	0815	540E-04	178E-02	Capn2	-9638	Hyper
chr4	59793596	59795916	Distal Intergenic	0739	541E-04	178E-02	Mir29b1	-142529	Hyper
chr3	155440842	155448437	Intron (NM_001135718/311647, intron 1 of 39)	0454	546E-04	179E-02	Prex1	8251	Hyper
chr10	98130617	98132566	Distal Intergenic	0825	547E-04	179E-02	Mir297	83978	Hyper
chr10	39514999	39518439	3' UTR	0621	548E-04	179E-02	Sparc	19710	Hyper
chr10	64557651	64559972	Intron (NM_001107023/303337, intron 1 of 14)	0739	557E-04	182E-02	Rab11fip4	7474	Hyper
chr5	160350659	160353045	Distal Intergenic	0640	563E-04	183E-02	Spsb1	18217	Hyper
chr1	35136526	35137521	Distal Intergenic	0911	573E-04	186E-02	Zfp58	-103310	Hyper
chr3	117476046	117478302	Promoter (<=1kb)	0611	574E-04	186E-02	Nop56	0	Hyper

chr12	20242386	20245267	Intron (NM_053860/116639, intron 2 of 22)	0690	576E-04	186E-02	Cux1	135075	Hyper
chr1	232543754	232544824	Distal Intergenic	1175	578E-04	186E-02	Kif20b	115392	Hyper
chr7	119939002	119959362	Promoter (<=1kb)	0333	579E-04	187E-02	Pim3	0	Hyper
chr1	48616635	48620417	Intron (NM_133406/170919, intron 1 of 8)	0562	581E-04	187E-02	Agpat4	12806	Hyper
chr7	131297916	131300836	Distal Intergenic	0688	585E-04	188E-02	Atf1	-61126	Hyper
chr18	27927781	27929016	Distal Intergenic	1020	588E-04	188E-02	Nrg2	-129276	Hyper
chr3	119497148	119498173	Promoter (<=1kb)	0711	591E-04	189E-02	Tmem230	0	Hyper
chr3	116784744	116786331	Distal Intergenic	1035	597E-04	190E-02	Sirpb3	-21498	Hyper
chr20	6748943	6752422	Promoter (<=1kb)	0484	601E-04	191E-02	Slc26a8	0	Hyper
chr10	30275643	30277124	Distal Intergenic	0955	612E-04	193E-02	Clint1	-21580	Hyper
chr6	130822404	130825557	Promoter (<=1kb)	0514	613E-04	194E-02	Klc1	0	Hyper
chr4	168003033	168004188	Promoter (<=1kb)	0865	615E-04	194E-02	Hebp1	0	Hyper
chr1	79010170	79013082	Promoter (<=1kb)	0547	615E-04	194E-02	Polr1g	0	Hyper
chr7	107920948	107925696	Promoter (<=1kb)	0526	617E-04	194E-02	Plec	0	Hyper
chrX	10675432	10677686	Distal Intergenic	0657	627E-04	196E-02	Bcor	-10046	Hyper
chr1	134358541	134363453	Promoter (<=1kb)	0498	635E-04	199E-02	Furin	0	Hyper
chr1	84097565	84104211	Promoter (<=1kb)	0386	646E-04	201E-02	Hnrnpl	0	Hyper
chr6	3890245	3893505	Intron (NM_021767/60391, intron 16 of 21)	0637	655E-04	203E-02	Nrxn1	708955	Hyper
chr4	11942013	11943479	Distal Intergenic	0938	655E-04	203E-02	Kmt2e	-214640	Hyper
chr5	141607805	141608954	Distal Intergenic	1150	655E-04	203E-02	Sync	-23423	Hyper
chr14	83312371	83315198	Intron (NM_001170459/360980, intron 2 of 28)	0711	660E-04	204E-02	Tns3	28940	Hyper
chr10	91032661	91034465	Intron (NM_001107058/303604, intron 2 of 15)	0831	664E-04	204E-02	Map3k3	12487	Hyper
chr6	105195081	105196138	Distal Intergenic	1171	667E-04	205E-02	Fos	73911	Hyper
chr5	160468338	160470653	Promoter (<=1kb)	0547	676E-04	207E-02	H6pd	0	Hyper
chr5	124415204	124418061	Promoter (<=1kb)	0570	685E-04	209E-02	Cdkn2c	0	Hyper
chr3	19291570	19292283	Distal Intergenic	1387	692E-04	211E-02	Ndufa8	107176	Hyper
chr9	64125390	64127882	Promoter (2-3kb)	0703	693E-04	211E-02	Nrp2	2013	Hyper
chr9	14862573	14863297	Exon (NM_198756/301249, exon 2 of 6)	1355	693E-04	211E-02	Mrps18a	6522	Hyper
chr11	57326008	57329794	Intron (NM_001105880/288105, intron 6 of 12)	0487	696E-04	212E-02	Mir568	-271935	Hyper
chr11	61801086	61805862	Intron (NM_001013120/303926, intron 1 of 6)	0465	704E-04	213E-02	Igsf11	62486	Hyper
chr15	97482120	97483834	Intron (NM_001111064/680445, intron 2 of 8)	0843	708E-04	213E-02	Mbnl2	96876	Hyper
chr8	65514418	65515385	Exon (NM_001106831/300783, exon 6 of 11)	1058	718E-04	216E-02	Hacd3	23122	Hyper
chr10	10675260	10680764	Intron (NM_001013964/302938, intron 1 of 16)	0472	718E-04	216E-02	Mgrn1	7532	Hyper

chr5	150544838	150547373	Promoter (2-3kb)	0737	719E-04	216E-02	Pink1	-2203	Hyper
chr13	47086066	47090656	Distal Intergenic	0484	722E-04	216E-02	Csrp1	-67515	Hyper
chr20	6387532	6390352	Promoter (<=1kb)	0542	725E-04	217E-02	Rpl10a	0	Hyper
chr7	57083647	57085409	Exon (NM_001106786/299827, exon 14 of 21)	0793	725E-04	217E-02	Tbk1	25459	Hyper
chr20	42866893	42869334	Intron (NM_012755/25150, intron 3 of 13)	0795	726E-04	217E-02	Fyn	98834	Hyper
chr1	196571224	196575984	Promoter (<=1kb)	0452	728E-04	217E-02	Polr2l	0	Hyper
chr7	55615036	55616349	Distal Intergenic	0684	729E-04	217E-02	Irak3	96697	Hyper
chr7	55710638	55714262	Promoter (<=1kb)	0527	736E-04	218E-02	Irak3	0	Hyper
chr3	153623494	153633772	3' UTR	0396	736E-04	218E-02	Zfp335	3366	Hyper
chr1	186148761	186150201	Promoter (<=1kb)	0712	739E-04	218E-02	C1h10orf88	0	Hyper
chr10	85344085	85346440	Promoter (<=1kb)	0626	744E-04	220E-02	P3h4	0	Hyper
chr1	196275350	196277513	Promoter (<=1kb)	0636	750E-04	220E-02	Rnh1	0	Hyper
chr2	174762519	174766608	Promoter (<=1kb)	0486	759E-04	222E-02	Adam15	0	Hyper
chr3	90481222	90485374	Promoter (<=1kb)	0488	760E-04	222E-02	C3h11orf91	0	Hyper
chr16	8449802	8450878	Distal Intergenic	1132	765E-04	223E-02	Arhgap22	-130906	Hyper
chr10	105193341	105195669	Promoter (1-2kb)	0898	767E-04	223E-02	Chmp6	1369	Hyper
chr8	41166810	41168781	3' UTR	0792	772E-04	224E-02	Hspa8	-14616	Hyper
chr4	126256621	126258441	Intron (NM_001030045/500261, intron 12 of 22)	0871	773E-04	224E-02	Magi1	552318	Hyper
chr6	95093290	95098592	Distal Intergenic	0396	779E-04	225E-02	Zbtb25	-9228	Hyper
chr4	159422843	159425124	Distal Intergenic	0664	788E-04	227E-02	Kcna1	46000	Hyper
chr7	69562316	69563678	Distal Intergenic	0791	790E-04	227E-02	Klf10	-88590	Hyper
chr2	135384263	135388171	Distal Intergenic	0407	793E-04	227E-02	Elf2	-12220	Hyper
chr6	88001371	88002605	Distal Intergenic	1050	796E-04	228E-02	Arf6	147629	Hyper
chr10	16447582	16448428	Intron (NM_001277157/303016, intron 1 of 8)	1087	807E-04	230E-02	Crebrf	13571	Hyper
chr14	34986178	34988748	Distal Intergenic	0606	813E-04	231E-02	Ociad1	-70939	Hyper
chr10	64018118	64020916	Intron (NM_001382488/108348076, intron 2 of 17)	0593	814E-04	231E-02	Ksr1	77160	Hyper
chr2	224171847	224173668	Distal Intergenic	0794	818E-04	232E-02	Nfkb1	-40016	Hyper
chr10	98192703	98196439	Distal Intergenic	0568	819E-04	232E-02	Mir297	20105	Hyper
chr3	108569606	108571014	Distal Intergenic	1007	823E-04	233E-02	Mfap1a	-112862	Hyper
chr3	13982254	14004220	Distal Intergenic	0300	833E-04	235E-02	C3h9orf50	82758	Hyper
chr6	92490871	92492777	Downstream (<=300bp)	0951	834E-04	235E-02	Hif1a	-131613	Hyper
chr1	204122682	204127034	Promoter (<=1kb)	0568	835E-04	235E-02	Kcnk4	0	Hyper
chr16	46917362	46919728	Promoter (<=1kb)	0615	836E-04	235E-02	Cyp4v3	0	Hyper

chr10	73812416	73814150	Promoter (<=1kb)	0694	838E-04	235E-02	Trim25	0	Hyper
chr14	12665938	12668515	Distal Intergenic	0695	849E-04	238E-02	Anxa3	113119	Hyper
chr10	16449357	16451269	Intron (NM_001277157/303016, intron 1 of 8)	0823	851E-04	238E-02	Crebrf	10730	Hyper
chr10	101599974	101602646	Distal Intergenic	0606	852E-04	238E-02	Foxj1	-29744	Hyper
chr6	103827073	103830372	Promoter (<=1kb)	0501	852E-04	238E-02	Mideas	0	Hyper
chr4	153666602	153669509	Promoter (<=1kb)	0692	861E-04	240E-02	Il17ra	0	Hyper
chr6	98858874	98861453	Distal Intergenic	0749	865E-04	241E-02	Zfp36l1	74295	Hyper
chr6	106436965	106439191	Distal Intergenic	0827	887E-04	245E-02	Lrrc74a	50618	Hyper
chr4	104433860	104437888	Distal Intergenic	0545	892E-04	246E-02	Vamp8	7239	Hyper
chr6	121914606	121919481	3' UTR	0444	896E-04	246E-02	Ubr7	15976	Hyper
chr2	192522221	192530064	Intron (NM_001107712/310760, intron 3 of 5)	0418	904E-04	248E-02	Cttnbp2nl	24484	Hyper
chr4	84090443	84091776	Intron (NM_001109236/500133, intron 1 of 11)	0991	905E-04	248E-02	Nod1	19628	Hyper
chr4	103259149	103263521	Promoter (<=1kb)	0440	908E-04	248E-02	Krcc1	0	Hyper
chr17	54983830	54985469	Distal Intergenic	0792	912E-04	249E-02	Rab18	39613	Hyper
chr5	134132528	134134398	Exon (NM_001134873/313560, exon 10 of 19)	0814	915E-04	250E-02	Slfnl1	14894	Hyper
chr19	23167801	23171207	Distal Intergenic	0565	915E-04	250E-02	Hook2	-3620	Hyper
chr5	149124589	149125529	Promoter (<=1kb)	1223	921E-04	251E-02	C1qb	-197	Hyper
chr1	144054981	144065386	Promoter (<=1kb)	0353	928E-04	252E-02	Picalm	0	Hyper
chr16	16794106	16797113	Exon (NM_001169127/306324, exon 3 of 9)	0602	929E-04	252E-02	Sh2d4b	-25019	Hyper
chr13	67223940	67226047	Distal Intergenic	0788	932E-04	253E-02	Ier5	46179	Hyper
chr1	239174273	239177595	Distal Intergenic	0581	943E-04	255E-02	Pdlim1	-83305	Hyper
chr13	50137864	50138930	Distal Intergenic	1117	944E-04	255E-02	Nek7	-44399	Hyper
chr3	16560610	16562068	Distal Intergenic	0868	951E-04	257E-02	Angptl2	43425	Hyper
chr13	49442799	49443688	Distal Intergenic	1041	953E-04	257E-02	Mir181a-1	-41839	Hyper
chr3	156637685	156641168	Promoter (<=1kb)	0517	954E-04	257E-02	Ptpn1	0	Hyper
chr9	90211911	90215598	5' UTR	0595	954E-04	257E-02	Agap1	23886	Hyper
chr14	79176032	79177576	Distal Intergenic	0774	956E-04	257E-02	Lif	41371	Hyper
chr9	87222911	87224721	Intron (NM_001108806/363272, intron 1 of 4)	0686	957E-04	257E-02	Pde6d	13205	Hyper
chr19	23969407	23973583	Distal Intergenic	0511	961E-04	257E-02	Mir181c	-9940	Hyper
chr2	25369530	25372057	Intron (NM_001106402/294643, intron 1 of 2)	0647	963E-04	258E-02	Lhfpl2	23359	Hyper
chr8	68484463	68485346	Distal Intergenic	1160	964E-04	258E-02	LOC100911360	-147506	Hyper
chr11	33040344	33043298	Intron (NM_001191660/304077, intron 1 of 35)	0522	971E-04	259E-02	Dop1b	12295	Hyper
chr14	79957455	79962430	Promoter (<=1kb)	0523	974E-04	259E-02	Gas2l1	0	Hyper

chr20	27903960	27909582	Distal Intergenic	0444	975E-04	260E-02	Ddit4	-9872	Hyper
chr13	44713136	44716865	Distal Intergenic	0494	981E-04	261E-02	Golt1a	-43515	Hyper
chr9	65400116	65401523	Distal Intergenic	0900	982E-04	261E-02	Klf7	83197	Hyper
chr2	33791955	33795347	Distal Intergenic	0484	986E-04	261E-02	Cd180	-60644	Hyper
chr20	32737954	32740143	Distal Intergenic	0592	987E-04	261E-02	Cep85l	-57790	Hyper
chr1	135875631	135877478	Promoter (<=1kb)	0607	994E-04	262E-02	Hdgfl3	0	Hyper
chr12	11605268	11610641	Promoter (<=1kb)	0441	100E-03	263E-02	Fscn1	0	Hyper
chr18	5338459	5340372	Distal Intergenic	0727	100E-03	264E-02	Zfp521	-271233	Hyper
chr10	81917732	81926776	3' UTR	0361	101E-03	264E-02	Pnpo	4068	Hyper
chr7	69857393	69859109	Exon (NM_001011992/299971, exon 8 of 13)	0979	101E-03	266E-02	Atp6v1c1	22810	Hyper
chr19	51474608	51476240	Distal Intergenic	0615	102E-03	267E-02	Def8	-3598	Hyper
chr20	21462417	21464355	Promoter (<=1kb)	0664	103E-03	269E-02	Jmjd1c	0	Hyper
chr1	204485766	204488143	Intron (NM_021699/60328, intron 1 of 16)	0623	103E-03	269E-02	Rcor2	31023	Hyper
chr5	117286081	117289379	Intron (NM_017031/24626, intron 5 of 14)	0637	103E-03	270E-02	Sgip1	-248367	Hyper
chr10	101360261	101361786	Promoter (2-3kb)	0966	104E-03	271E-02	Trim65	2250	Hyper
chr13	83525729	83527440	Promoter (<=1kb)	0612	104E-03	271E-02	Cfap126	0	Hyper
chr2	233689099	233691266	Distal Intergenic	0721	105E-03	272E-02	Selenof	47024	Hyper
chr7	131809482	131811280	Intron (NM_001172063/100361647, intron 2 of 9)	0744	105E-03	272E-02	Galnt6	4225	Hyper
chr2	210737490	210739289	Promoter (<=1kb)	0674	105E-03	272E-02	Fnbp1l	0	Hyper
chr3	154070124	154071324	Distal Intergenic	0949	105E-03	273E-02	Elmo2	-8967	Hyper
chr7	8868602	8869715	Promoter (<=1kb)	0990	106E-03	273E-02	Lingo3	0	Hyper
chr7	69695658	69698313	Distal Intergenic	0612	106E-03	274E-02	Azin1	-14080	Hyper
chr8	20014314	20018231	Exon (NM_013199/25751, exon 3 of 21)	0494	106E-03	274E-02	Dnm2	35864	Hyper
chr20	5819952	5821963	Exon (NM_001047939/499407, exon 13 of 23)	0716	107E-03	276E-02		11092	Hyper
chr17	44655541	44663465	Exon (NM_001108415/361251, exon 6 of 22)	0431	107E-03	276E-02	Elmo1	159323	Hyper
chr10	100938147	100940697	5' UTR	0550	108E-03	277E-02	Grb2	8496	Hyper
chr15	52073043	52075683	Distal Intergenic	0678	108E-03	277E-02	Serp2	-169078	Hyper
chr16	58859740	58860801	Distal Intergenic	1026	108E-03	277E-02	Purg	-96708	Hyper
chr1	184766170	184768360	Intron (NM_001109892/25022, intron 10 of 17)	0650	109E-03	279E-02	Fgfr2	82266	Hyper
chr4	136146307	136147448	Distal Intergenic	1064	110E-03	281E-02	Cntn3	-1020694	Hyper
chr12	19023771	19025625	Intron (NM_001107131/304375, intron 1 of 11)	0634	110E-03	282E-02	Agfg2	11513	Hyper
chr7	9635980	9637786	Intron (NM_001108068/314619, intron 6 of 32)	0702	111E-03	282E-02	Gpx4	14726	Hyper
chr6	104757703	104759785	Promoter (<=1kb)	0536	112E-03	284E-02	Dlst	0	Hyper

chr14	83309852	83311994	Intron (NM_001170459/360980, intron 2 of 28)	0767	112E-03	284E-02	Tns3	32144	Hyper
chr3	9465033	9467985	Distal Intergenic	0541	112E-03	284E-02	Dipk1b	8624	Hyper
chr4	157565970	157571830	Promoter (<=1kb)	0373	113E-03	285E-02	Atn1	0	Hyper
chr11	30268061	30270824	Promoter (<=1kb)	0482	113E-03	286E-02	Synj1	0	Hyper
chr16	66825737	66827441	Distal Intergenic	0653	113E-03	286E-02	Tacc1	-17600	Hyper
chr3	156627092	156628814	Distal Intergenic	0838	113E-03	286E-02	Ptpn1	-9997	Hyper
chr13	64903882	64905349	Promoter (<=1kb)	0683	114E-03	288E-02	Ralgdsl1	0	Hyper
chr18	36972990	36976774	Intron (NM_001170534/307449, intron 3 of 18)	0492	115E-03	289E-02	Dcp2	222258	Hyper
chr2	174836911	174842762	Promoter (<=1kb)	0404	115E-03	289E-02	Cks1b	0	Hyper
chr6	40775596	40778326	Distal Intergenic	0602	115E-03	289E-02	Itgb1bp1	57421	Hyper
chr7	98887851	98889119	Distal Intergenic	0930	115E-03	290E-02	St3gal1	-32198	Hyper
chr2	102442052	102443910	Promoter (2-3kb)	0774	117E-03	293E-02	Cp	2428	Hyper
chr10	37909653	37912235	Distal Intergenic	0491	117E-03	293E-02	Irf1	-4929	Hyper
chr11	69169747	69171485	Distal Intergenic	0847	118E-03	294E-02	Dlg1	-66944	Hyper
chr15	4327726	4328559	Intron (NM_031754/80850, intron 1 of 3)	1164	118E-03	294E-02	Gng2	11090	Hyper
chr7	113905077	113906586	Promoter (<=1kb)	0866	118E-03	294E-02	Cyp2d5	-666	Hyper
chr7	8950722	8954117	Promoter (2-3kb)	0597	118E-03	294E-02	Dot1l	2306	Hyper
chr6	118628671	118631808	Intron (NM_001108047/314374, intron 3 of 6)	0601	119E-03	295E-02	Foxn3	210593	Hyper
chr12	11637481	11641424	Distal Intergenic	0486	119E-03	296E-02	Actb	-21688	Hyper
chr3	15948866	15950384	Intron (NM_001010968/497010, intron 2 of 14)	0907	121E-03	299E-02	Eng	14300	Hyper
chr14	76238653	76240108	Exon (NM_001024771/305451, exon 3 of 5)	0924	121E-03	300E-02	Tnip2	10341	Hyper
chr8	59710249	59713953	Exon (NM_001100723/315722, exon 3 of 7)	0551	121E-03	300E-02	Adpgk	10805	Hyper
chr4	119276266	119277686	Distal Intergenic	0874	122E-03	300E-02	Aak1	-23542	Hyper
chr3	77978909	77981304	Intron (NM_001005562/362165, intron 1 of 11)	0719	123E-03	302E-02	Creb3l1	12152	Hyper
chr1	135770722	135772941	Promoter (<=1kb)	0451	124E-03	304E-02	Btbd1	0	Hyper
chr10	62696750	62706785	Intron (NM_001172137/360570, intron 2 of 41)	0362	125E-03	305E-02	Myo18a	42469	Hyper
chr3	167909691	167910973	Distal Intergenic	0973	126E-03	307E-02	Bhlhe23	-18468	Hyper
chr13	92154321	92156775	Intron (NM_019312/54260, intron 4 of 7)	0719	126E-03	308E-02	Itpkb	85105	Hyper
chr2	25399666	25404252	5' UTR	0493	127E-03	308E-02	Lhfpl2	53495	Hyper
chr14	104008300	104009816	Promoter (<=1kb)	0785	127E-03	310E-02	Sptbn1	0	Hyper
chr1	117379201	117385573	Distal Intergenic	0411	129E-03	312E-02	Klf13	151053	Hyper
chr1	95297683	95303646	Promoter (<=1kb)	0406	129E-03	313E-02	Atf5	0	Hyper
chr14	80732693	80734346	Distal Intergenic	0782	129E-03	313E-02	Aebp1	-4546	Hyper

chr1	84578640	84580798	Promoter (<=1kb)	0705	129E-03	313E-02	Spint2	0	Hyper
chr3	8219864	8226612	Promoter (<=1kb)	0395	129E-03	313E-02	Npdc1	0	Hyper
chr3	149350188	149351559	Intron (NM_022615/64550, intron 9 of 20)	0867	129E-03	313E-02	Plcg1	-34028	Hyper
chr1	175166522	175168977	Promoter (<=1kb)	0539	130E-03	313E-02	Uqcrc2	0	Hyper
chr1	52671089	52674458	Distal Intergenic	0673	131E-03	316E-02	Rnaset2	85109	Hyper
chr7	112771674	112772682	Distal Intergenic	1087	132E-03	317E-02	Mchr1	10120	Hyper
chr1	83410635	83411764	Intron (NM_001164657/100303643, intron 18 of 19)	0862	133E-03	318E-02	Fcgbpl1	-13024	Hyper
chr2	82593347	82594372	Exon (NM_001004078/294864, exon 8 of 11)	0963	133E-03	318E-02	Cct5	8531	Hyper
chr14	79904472	79906689	Exon (NM_001308298/29663, exon 5 of 23)	0646	136E-03	324E-02	Ap1b1	24957	Hyper
chr17	15431666	15437023	Promoter (2-3kb)	0441	136E-03	324E-02	Card19	-2093	Hyper
chr2	117534471	117559428	Promoter (<=1kb)	0301	137E-03	325E-02	Sox2	0	Hyper
chr1	164833033	164835370	Distal Intergenic	0564	138E-03	326E-02	Ampd3	-49950	Hyper
chr20	3582884	3586779	Promoter (<=1kb)	0393	138E-03	327E-02	Ddx39b	0	Hyper
chr5	150351955	150353784	Intron (NM_001106693/298573, intron 9 of 30)	0676	139E-03	328E-02	Hp1bp3	-82003	Hyper
chr7	25666475	25668967	Promoter (<=1kb)	0469	139E-03	328E-02	Tmpo	0	Hyper
chr1	13079008	13081434	Distal Intergenic	0563	139E-03	328E-02	Hebp2	120343	Hyper
chr13	83140128	83141283	Distal Intergenic	0956	140E-03	330E-02	Dusp12	-8843	Hyper
chr10	35888039	35889440	Promoter (2-3kb)	0917	140E-03	330E-02	Rmnd5b	2753	Hyper
chr5	143035924	143038011	Distal Intergenic	0665	140E-03	330E-02	Laptm5	-49748	Hyper
chr6	7867292	7872776	Promoter (<=1kb)	0437	140E-03	330E-02	Epas1	0	Hyper
chr9	17037690	17039956	Intron (NM_175578/140666, intron 2 of 4)	0683	141E-03	331E-02	Enpp5	-120210	Hyper
chr19	47491557	47492621	5' UTR	1137	141E-03	331E-02	Osgin1	6307	Hyper
chr14	95090222	95091781	Distal Intergenic	0855	142E-03	332E-02	Lgalsl	-85723	Hyper
chr3	88076724	88077843	Intron (NM_001080150/362171, intron 1 of 8)	0943	142E-03	332E-02	Prr5l	4920	Hyper
chr4	159398971	159415542	Distal Intergenic	0331	142E-03	333E-02	Kcna5	-41675	Hyper
chr16	5669849	5670729	Intron (NM_001191831/361109, intron 3 of 9)	1076	143E-03	334E-02	Dcp1a	12924	Hyper
chr8	44562883	44563993	Distal Intergenic	1042	143E-03	334E-02	Ccdc153	-13843	Hyper
chr4	57869851	57871725	Distal Intergenic	0590	143E-03	334E-02	Garin1a	-8294	Hyper
chr2	209336366	209341625	Distal Intergenic	0423	144E-03	334E-02	Tlcd4	-16793	Hyper
chr20	46645109	46646239	Intron (NM_001104640/309860, intron 2 of 5)	1026	144E-03	335E-02	Sobp	14645	Hyper
chr4	10292898	10294155	Intron (NM_013216/26954, intron 1 of 7)	1077	144E-03	335E-02	Rheb	13292	Hyper
chr5	145227831	145235163	Promoter (<=1kb)	0357	145E-03	336E-02	Ahdc1	0	Hyper
chr9	73164255	73165698	Promoter (<=1kb)	0610	145E-03	336E-02	Atic	0	Hyper

chr8	109702696	109706706	Promoter (<=1kb)	0485	145E-03	336E-02	Shisa5	0	Hyper
chr6	72273461	72274758	Promoter (<=1kb)	0581	145E-03	336E-02	Snx6	0	Hyper
chr9	14152764	14154791	Promoter (<=1kb)	0749	145E-03	336E-02	Bicral	0	Hyper
chr1	79586484	79587712	Distal Intergenic	0903	145E-03	336E-02	Pvr	-9784	Hyper
chr10	4552176	4556511	Exon (NM_001127532/302899, exon 6 of 12)	0506	146E-03	337E-02	Snn	25095	Hyper
chr12	27676736	27679279	Promoter (<=1kb)	0504	146E-03	337E-02	Ran	0	Hyper
chr8	97300426	97308441	Distal Intergenic	0398	146E-03	337E-02	Zbtb38	-11908	Hyper
chr1	161594100	161602744	Intron (NM_001100582/308918, intron 5 of 23)	0401	146E-03	337E-02	Cyb5r2	61353	Hyper
chr8	68449930	68450971	Distal Intergenic	0872	147E-03	337E-02	LOC100911360	-112973	Hyper
chr1	151684009	151684974	Promoter (<=1kb)	0806	147E-03	338E-02	Alg8	0	Hyper
chr10	102842495	102845208	Distal Intergenic	0584	148E-03	339E-02	Tmc8	-155136	Hyper
chr6	104181665	104183502	Distal Intergenic	0646	148E-03	339E-02	Vsx2	-33728	Hyper
chr5	130608624	130612849	Promoter (<=1kb)	0510	149E-03	341E-02	Plk3	0	Hyper
chr10	71414642	71415743	Intron (NM_138839/192129, intron 10 of 11)	1117	149E-03	341E-02	Mir21	-9294	Hyper
chr1	127134754	127138031	Intron (NM_001107524/308739, intron 1 of 3)	0581	150E-03	342E-02	Rgma	5820	Hyper
chr3	76485367	76488437	Intron (NM_017269/29645, intron 1 of 23)	0590	150E-03	342E-02	Ptprj	73232	Hyper
chr6	16473251	16474683	Distal Intergenic	0893	150E-03	342E-02	Strn	137881	Hyper
chr14	78383111	78385835	Promoter (<=1kb)	0580	151E-03	344E-02	Inpp5j	0	Hyper
chr10	78902218	78904453	Promoter (<=1kb)	0488	151E-03	344E-02	Nme2	0	Hyper
chr14	75201369	75203907	Distal Intergenic	0551	151E-03	345E-02	Trmt44	19108	Hyper
chr6	100516649	100519485	Distal Intergenic	0631	152E-03	347E-02	Srsf5	-85996	Hyper
chr2	169572998	169574733	Distal Intergenic	0712	154E-03	348E-02	Trim2	-8576	Hyper
chr5	61228798	61231046	Intron (NM_031802/83633, intron 1 of 19)	0706	154E-03	348E-02	Gabbr2	57058	Hyper
chr4	96025665	96027082	Distal Intergenic	0856	155E-03	350E-02	Gng12	-9164	Hyper
chr8	65730311	65732681	Promoter (<=1kb)	0513	155E-03	350E-02	Parp16	0	Hyper
chr7	18171866	18173207	Distal Intergenic	0831	156E-03	351E-02	Pwp1	-173138	Hyper
chr15	64427644	64432180	Promoter (<=1kb)	0518	156E-03	352E-02	Pcdh20	0	Hyper
chr16	19449350	19451141	Intron (NM_001013881/290669, intron 1 of 12)	0570	157E-03	353E-02	Gatad2a	19772	Hyper
chr10	106541770	106543746	Promoter (<=1kb)	0588	158E-03	354E-02	Foxk2	0	Hyper
chr11	84860191	84865751	Intron (NM_001100988/498119, intron 7 of 18)	0395	158E-03	354E-02	Cebpd	95521	Hyper
chr13	102805818	102807648	Distal Intergenic	0535	159E-03	354E-02	Atf3	-41193	Hyper
chr1	84986595	84987866	Promoter (<=1kb)	0570	159E-03	355E-02	Zfp84	0	Hyper
chr1	153885498	153887186	Exon (NM_012910/25387, exon 3 of 16)	0793	159E-03	355E-02	Mir326	31469	Hyper

chr12	19600272	19601593	Promoter (<=1kb)	0793	160E-03	356E-02	Serpine1	0	Hyper
chr13	66153061	66154137	Distal Intergenic	1041	160E-03	357E-02	Glul	127420	Hyper
chr5	165709300	165711104	Distal Intergenic	0698	160E-03	357E-02	Morn1	62304	Hyper
chr12	36373941	36375930	Intron (NM_001371959/304512, intron 23 of 23)	0727	161E-03	357E-02	Rbm19	75103	Hyper
chr17	83369691	83370581	Intron (NM_001191693/307178, intron 10 of 24)	1018	161E-03	358E-02	Arhgap21	101621	Hyper
chr17	67205234	67207912	Distal Intergenic	0464	161E-03	358E-02	Pfkfb3	170139	Hyper
chr1	245247636	245250160	Exon (NM_001106364/294010, exon 2 of 11)	0695	161E-03	358E-02	Actr1a	6333	Hyper
chr10	94446474	94448782	Promoter (<=1kb)	0511	162E-03	359E-02	Slc16a6	0	Hyper
chr5	145808560	145810185	Promoter (<=1kb)	0619	163E-03	360E-02	Gpn2	0	Hyper
chr8	66101204	66106345	Distal Intergenic	0404	163E-03	360E-02	Pif1	-5379	Hyper
chr13	66107634	66108889	Distal Intergenic	1012	163E-03	360E-02	Glul	81993	Hyper
chr1	96256278	96257257	Intron (NM_001039665/292915, intron 1 of 6)	1172	164E-03	360E-02	Sult2b1	4038	Hyper
chr13	38710801	38713275	Intron (NM_023095/65271, intron 1 of 17)	0596	164E-03	362E-02	Mgat5	34682	Hyper
chr10	101661584	101666273	Distal Intergenic	0420	166E-03	365E-02	Prpsap1	56519	Hyper
chr3	116404417	116405599	Distal Intergenic	0864	166E-03	365E-02	Slc20a1	-22360	Hyper
chr2	183548821	183553125	Promoter (<=1kb)	0471	166E-03	365E-02	Plekho1	0	Hyper
chr19	23185037	23190390	Promoter (<=1kb)	0418	167E-03	366E-02	Prdx2	0	Hyper
chr6	21126359	21127714	Promoter (<=1kb)	0569	167E-03	366E-02	Dpy30	0	Hyper
chr6	106533039	106545539	Promoter (2-3kb)	0311	168E-03	366E-02	Irf2bpl	-2638	Hyper
chr11	62082695	62084094	Intron (NM_001105879/288093, intron 1 of 11)	0599	168E-03	367E-02	Arhgap31	44060	Hyper
chr17	63728994	63731823	Promoter (<=1kb)	0518	168E-03	367E-02	Pfkp	0	Hyper
chr4	159396478	159398753	Distal Intergenic	0714	169E-03	368E-02	Kcna5	-39182	Hyper
chr14	74244844	74249141	Exon (NM_001113365/360956, exon 7 of 15)	0490	169E-03	368E-02	Tbc1d14	9593	Hyper
chr4	59165529	59167356	Exon (NM_001013083/296959, exon 5 of 11)	0547	169E-03	368E-02	Cpa2	5172	Hyper
chr12	11804481	11806964	Intron (NM_001107123/304302, intron 10 of 29)	0716	170E-03	369E-02	Tnrc18	49089	Hyper
chr12	6148920	6150620	Intron (NM_001006956/288449, intron 9 of 10)	0747	170E-03	370E-02	Katnal1	46034	Hyper
chr7	8412887	8416365	Intron (NM_001009967/314641, intron 1 of 18)	0498	170E-03	370E-02	Pip5k1c	9623	Hyper
chr6	16398523	16399961	Exon (NM_019148/29149, exon 8 of 18)	0794	171E-03	370E-02	Strn	63153	Hyper
chr10	45610886	45612745	Promoter (2-3kb)	0760	171E-03	371E-02	Map2k3	2741	Hyper
chr8	20540663	20543041	Exon (NM_001106806/300445, exon 8 of 18)	0725	172E-03	372E-02	Prkcsh	5722	Hyper
chr1	98956763	98961142	Promoter (<=1kb)	0473	174E-03	375E-02	Nav2	0	Hyper
chr20	7502586	7503685	Distal Intergenic	1038	174E-03	376E-02	Pim1	-51236	Hyper
chr12	26593636	26595521	Intron (NM_001011903/288617, intron 1 of 5)	0600	175E-03	376E-02	Tpst1	10154	Hyper

chr10	60529877	60532232	Promoter (<=1kb)	0483	175E-03	377E-02	Crk	0	Hyper
chr13	92505953	92507669	Distal Intergenic	0660	176E-03	378E-02	Acbd3	50140	Hyper
chr6	129706941	129713387	Promoter (<=1kb)	0366	176E-03	378E-02	Hsp90aa1	0	Hyper
chr7	120254155	120260678	Promoter (<=1kb)	0400	176E-03	379E-02	Plxnb2	0	Hyper
chr8	59695393	59710103	Promoter (<=1kb)	0346	177E-03	379E-02	Adpgk	0	Hyper
chr12	20141692	20143683	Intron (NM_053860/116639, intron 1 of 22)	0715	178E-03	380E-02	Cux1	34381	Hyper
chr2	104531209	104532162	Distal Intergenic	0949	179E-03	382E-02	Tbl1xr1	-269559	Hyper
chr6	72817335	72820287	Distal Intergenic	0547	180E-03	384E-02	Psma6	38126	Hyper
chr10	100624099	100628806	Distal Intergenic	0412	180E-03	384E-02	Hid1	-14058	Hyper
chr1	194687433	194709319	Distal Intergenic	0310	180E-03	384E-02	Utf1	-31020	Hyper
chr12	19388552	19394009	Promoter (2-3kb)	0446	180E-03	384E-02	Trip6	2340	Hyper
chr19	51036162	51038392	Intron (NM_001374009/365023, intron 1 of 12)	0588	181E-03	385E-02	Ankrd11	60570	Hyper
chr1	213613591	213614820	Intron (NM_031036/81666, intron 3 of 6)	0931	182E-03	387E-02	Gna14	-101200	Hyper
chr10	98151891	98153967	Distal Intergenic	0672	182E-03	387E-02	Mir297	62577	Hyper
chr15	34867221	34868313	Intron (NM_001191686/305938, intron 4 of 12)	0913	183E-03	388E-02	C1qtnf9	-47016	Hyper
chr17	80783747	80787322	Distal Intergenic	0452	184E-03	389E-02	Mllt10	-35264	Hyper
chr8	119710111	119712228	Promoter (<=1kb)	0740	185E-03	390E-02	Csrnp1	0	Hyper
chrX	134554464	134556546	Promoter (<=1kb)	0664	185E-03	390E-02	Fhl1	0	Hyper
chr4	80621259	80622086	Intron (NM_001013085/297096, intron 1 of 6)	1250	185E-03	392E-02	Snx10	8563	Hyper
chr14	43789238	43790874	Distal Intergenic	0755	186E-03	393E-02	Mir328b	126170	Hyper
chr8	57973738	57974638	Intron (NM_023955/65168, intron 1 of 8)	1110	186E-03	393E-02	Scamp2	8093	Hyper
chr12	35406733	35412895	Exon (NM_001177593/25622, exon 11 of 16)	0460	187E-03	394E-02	Ptpn11	41297	Hyper
chr4	158113665	158116167	Promoter (<=1kb)	0488	187E-03	394E-02	Ltbr	0	Hyper
chr1	80910228	80911480	Exon (NM_053628/114029, exon 2 of 41)	0916	188E-03	395E-02	Megf8	7654	Hyper
chr8	60056865	60063510	Promoter (<=1kb)	0358	194E-03	404E-02	Pkm	0	Hyper
chr13	96392644	96397826	Promoter (<=1kb)	0395	194E-03	405E-02	Mtarc2	0	Hyper
chr17	31974555	31976523	Distal Intergenic	0723	194E-03	405E-02	Gmds	-118994	Hyper
chr10	62756788	62757786	Distal Intergenic	0945	195E-03	405E-02	Pipox	24584	Hyper
chr5	132713312	132740892	Promoter (<=1kb)	0290	196E-03	406E-02	Slc2a1	0	Hyper
chr10	4673259	4675203	Distal Intergenic	0813	196E-03	406E-02	Litaf	-7810	Hyper
chr6	85606053	85610156	Promoter (<=1kb)	0436	196E-03	406E-02	Mdga2	0	Hyper
chr7	53321360	53323900	Distal Intergenic	0516	200E-03	414E-02	Mdm2	-6445	Hyper
chr8	33546496	33548666	Distal Intergenic	0547	200E-03	414E-02	Srpra	-11709	Hyper

chr7	134501304	134502858	Promoter (<=1kb)	0806	201E-03	415E-02	Itga5	0	Hyper
chr1	183102336	183104233	Promoter (<=1kb)	0495	201E-03	415E-02	Bag3	0	Hyper
chr6	123766551	123769494	Intron (NM_001106755/299285, intron 1 of 12)	0587	202E-03	416E-02	Clmn	37778	Hyper
chr4	63541125	63543953	Intron (NM_001009709/362334, intron 1 of 1)	0574	202E-03	416E-02	Tmem140	4648	Hyper
chr9	14133164	14135413	Distal Intergenic	0730	203E-03	417E-02	Bicral	-18796	Hyper
chr1	182899153	182901827	Distal Intergenic	0658	203E-03	417E-02	Ahsp	14619	Hyper
chr7	107364749	107366629	Promoter (<=1kb)	0685	204E-03	418E-02	Top1mt	0	Hyper
chr8	65719212	65722416	Distal Intergenic	0497	205E-03	419E-02	Parp16	-9197	Hyper
chr7	120503003	120505032	Promoter (<=1kb)	0524	205E-03	419E-02	Chkb	0	Hyper
chr13	44671232	44673032	Distal Intergenic	0619	206E-03	421E-02	Golt1a	-87348	Hyper
chr15	35817566	35821462	Distal Intergenic	0460	208E-03	423E-02	Kcnrg	43074	Hyper
chr1	200954552	200957297	Distal Intergenic	0552	208E-03	423E-02	RGD1311946	9311	Hyper
chr15	13972870	13973810	Intron (NM_021774/60398, intron 1 of 7)	0938	210E-03	426E-02	Fhit	4988	Hyper
chr6	106774846	106776179	Exon (NM_001047114/688673, exon 9 of 21)	0787	210E-03	426E-02	Gstz1	-18477	Hyper
chr6	129554117	129555644	Intron (NM_001191112/691318, intron 2 of 13)	0809	210E-03	426E-02	Ppp2r5c	51248	Hyper
chr1	245489698	245498331	Promoter (<=1kb)	0374	210E-03	426E-02	Wbp1l	0	Hyper
chr18	77416297	77417716	Distal Intergenic	0736	211E-03	427E-02	Ptgr3	-36719	Hyper
chr1	155697406	155698492	Exon (NM_001191560/308865, exon 4 of 18)	0805	211E-03	427E-02	Atg16l2	4697	Hyper
chr1	196492793	196497785	Promoter (<=1kb)	0420	212E-03	428E-02	Taldo1	0	Hyper
chr17	64542833	64549733	Promoter (<=1kb)	0342	212E-03	428E-02	Klf6	0	Hyper
chr3	142446822	142449341	3' UTR	0668	212E-03	428E-02	Bpifb4	23251	Hyper
chr19	27142957	27144268	Intron (NM_001108444/361388, intron 1 of 9)	1002	213E-03	428E-02	Gab1	11695	Hyper
chr1	82521024	82523307	Promoter (<=1kb)	0473	213E-03	428E-02	Itpkc	0	Hyper
chr20	3694538	3697089	Promoter (<=1kb)	0506	213E-03	428E-02	G4	0	Hyper
chr11	69434169	69436410	Promoter (2-3kb)	0617	213E-03	429E-02	Apod	2908	Hyper
chr16	66527701	66531355	Promoter (2-3kb)	0485	214E-03	429E-02	Fgfr1	2245	Hyper
chr3	154863002	154865862	Exon (NM_001034927/311642, exon 3 of 21)	0620	215E-03	430E-02	Sulf2	38553	Hyper
chr1	197520912	197528100	3' UTR	0445	216E-03	432E-02	Ctsd	11243	Hyper
chr9	93485475	93488288	3' UTR	0517	216E-03	432E-02	Rnpepl1	8837	Hyper
chr5	149128659	149131064	Promoter (<=1kb)	0644	217E-03	433E-02	C1qc	0	Hyper
chr8	57980034	57981208	Exon (NM_023955/65168, exon 2 of 9)	0978	218E-03	436E-02	Ulk3	-11823	Hyper
chr10	60500987	60505516	Promoter (<=1kb)	0430	218E-03	436E-02	Myo1c	0	Hyper
chr6	88626513	88628238	Promoter (<=1kb)	0720	219E-03	436E-02	Nin	0	Hyper

chr6	100511845	100514763	Distal Intergenic	0531	220E-03	438E-02	Srsf5	-90718	Hyper
chr13	84658723	84668712	Promoter (<=1kb)	0324	221E-03	439E-02	Pea15	0	Hyper
chr2	153876070	153877593	Promoter (<=1kb)	0593	222E-03	440E-02	B3galnt1	0	Hyper
chr18	24065618	24067835	3' UTR	0630	222E-03	441E-02	Bin1	55814	Hyper
chr10	12581484	12583400	Distal Intergenic	0555	223E-03	442E-02	Zfp213	15881	Hyper
chr7	108345032	108346694	Promoter (<=1kb)	0638	223E-03	442E-02	Vps28	0	Hyper
chr7	105102448	105107443	Promoter (<=1kb)	0421	224E-03	443E-02	Ago2	0	Hyper
chr3	156093362	156095185	Distal Intergenic	0652	225E-03	444E-02	Slc9a8	-52920	Hyper
chr18	59114522	59117769	Intron (NM_001107382/307362, intron 2 of 9)	0483	225E-03	445E-02	Zfp532	17829	Hyper
chr3	11003019	11006302	Intron (NM_012805/25271, intron 1 of 9)	0571	225E-03	445E-02	Rxra	13103	Hyper
chr3	13740278	13745946	Promoter (<=1kb)	0400	226E-03	446E-02	Ier5l	0	Hyper
chr7	113570172	113571494	Promoter (<=1kb)	0597	227E-03	447E-02	Snu13	0	Hyper
chr19	27078382	27080457	Distal Intergenic	0698	227E-03	448E-02	Gab1	-50805	Hyper
chr8	64838400	64840960	Promoter (<=1kb)	0423	228E-03	448E-02	Dis3l	0	Hyper
chr3	16173668	16182486	Promoter (<=1kb)	0348	228E-03	448E-02	Niban2	0	Hyper
chr8	106885598	106887270	Promoter (2-3kb)	0767	229E-03	449E-02	Alas1	2582	Hyper
chr2	92014812	92015869	Distal Intergenic	1170	230E-03	450E-02	Pag1	-41347	Hyper
chr10	6952100	6953620	Intron (NM_152790/260416, intron 1 of 3)	0769	230E-03	450E-02	Carhsp1	5141	Hyper
chr13	90791076	90792248	Intron (NM_001025762/498295, intron 9 of 11)	1033	232E-03	452E-02	Smyd3	473961	Hyper
chr7	89405131	89408122	3' UTR	0526	232E-03	452E-02	Derl1	18973	Hyper
chr19	10832161	10834611	Promoter (<=1kb)	0571	233E-03	453E-02	Mt2A	0	Hyper
chr7	69699952	69702066	Distal Intergenic	0676	234E-03	453E-02	Azin1	-18374	Hyper
chr1	173276732	173278265	Distal Intergenic	0899	235E-03	455E-02	Gprc5b	62668	Hyper
chr17	12276706	12277562	Promoter (2-3kb)	0965	235E-03	455E-02	Nfil3	-2937	Hyper
chr1	239701963	239702780	Distal Intergenic	0865	235E-03	455E-02	Zfp518a	-17483	Hyper
chr7	105628813	105644715	Distal Intergenic	0299	236E-03	456E-02	Ptp4a3	-10391	Hyper
chr18	40132117	40141276	Intron (NM_001108430/361324, intron 1 of 18)	0355	237E-03	457E-02	Sema6a	30678	Hyper
chr16	24984915	24987814	5' UTR	0543	237E-03	457E-02	Msmo1	4235	Hyper
chr5	76814944	76818104	Promoter (<=1kb)	0457	237E-03	457E-02	Akna	0	Hyper
chr2	233552770	233553770	Intron (NM_001100518/292155, intron 1 of 6)	1046	237E-03	457E-02	Hs2st1	87999	Hyper
chr15	39130342	39131360	Distal Intergenic	0935	237E-03	457E-02	Extl3	188112	Hyper
chr1	117464125	117465252	Distal Intergenic	0874	237E-03	457E-02	Klf13	71374	Hyper
chr4	5511468	5514025	Intron (NM_001013209/362293, intron 8 of 9)	0651	238E-03	457E-02	Dnajb6	42606	Hyper

chr1	161612851	161618116	Exon (NM_001100582/308918, exon 7 of 24)	0442	238E-03	458E-02	Cyb5r2	45981	Hyper
chr20	11036045	11039992	Exon (NM_001013238/365548, exon 3 of 6)	0559	238E-03	458E-02	Pttg1ip	7290	Hyper
chr5	101225838	101228423	Distal Intergenic	0503	240E-03	459E-02	Plin2	-42802	Hyper
chr5	21855308	21857437	Intron (NM_001107906/312974, intron 1 of 37)	0562	240E-03	459E-02	Chd7	43298	Hyper
chr13	83658391	83659697	Exon (NM_001011907/289218, exon 4 of 13)	0889	241E-03	461E-02	Fcer1g	-4511	Hyper
chr11	24112282	24115697	Intron (NM_019288/54226, intron 6 of 17)	0516	242E-03	463E-02	App	120732	Hyper
chr4	77524348	77526302	Promoter (<=1kb)	0696	243E-03	463E-02	Rarres2	0	Hyper
chr1	8586422	8588053	Distal Intergenic	0558	243E-03	463E-02	Hivep2	227133	Hyper
chr3	156146960	156149306	Promoter (<=1kb)	0418	243E-03	463E-02	Slc9a8	0	Hyper
chr7	95379387	95382310	Distal Intergenic	0577	244E-03	464E-02	Gsdmc	223796	Hyper
chr1	76716777	76720731	Distal Intergenic	0512	244E-03	464E-02	Zfp541	-29505	Hyper
chr4	104567575	104569318	Distal Intergenic	0915	244E-03	465E-02	Capg	-30744	Hyper
chr1	134325466	134328181	Promoter (<=1kb)	0449	245E-03	466E-02	Man2a2	0	Hyper
chr12	34252766	34258678	3' UTR	0418	245E-03	466E-02	Pptc7	29661	Hyper
chr20	3846640	3847623	Promoter (<=1kb)	0686	246E-03	466E-02	Lsm2	0	Hyper
chr1	23307621	23309298	Distal Intergenic	0692	246E-03	468E-02	Sgk1	-319524	Hyper
chr1	236243895	236245240	Promoter (<=1kb)	0763	247E-03	468E-02	Plce1	0	Hyper
chr15	34895343	34898264	Exon (NM_001191686/305938, exon 9 of 13)	0461	247E-03	468E-02	C1qtnf9	-17065	Hyper
chr10	13949019	13951322	Intron (NM_001100673/302983, intron 1 of 30)	0661	248E-03	469E-02	Mapk8ip3	6871	Hyper
chr3	140110138	140116913	Intron (NM_001106525/296267, intron 2 of 5)	0367	249E-03	471E-02	Snph	22429	Hyper
chr3	146075865	146077138	Distal Intergenic	0959	250E-03	472E-02	Manbal	40783	Hyper
chr5	117257315	117259818	Intron (NM_017031/24626, intron 2 of 14)	0648	251E-03	473E-02	Pde4b	263879	Hyper
chr2	169602585	169605690	Distal Intergenic	0474	251E-03	473E-02	Trim2	-38163	Hyper
chr12	37984606	37985827	Distal Intergenic	0798	251E-03	473E-02	Spring1	343311	Hyper
chr18	54665176	54667129	Promoter (<=1kb)	0657	252E-03	473E-02	Slc26a2	0	Hyper
chr2	152748394	152753255	Exon (NM_001100666/295105, exon 5 of 11)	0436	253E-03	475E-02	Il12a	-212514	Hyper
chr8	67586726	67588089	Promoter (<=1kb)	0571	254E-03	475E-02	Lactb	0	Hyper
chr1	182005261	182006371	Distal Intergenic	0654	254E-03	476E-02	Zfp689	66877	Hyper
chr12	46408633	46410118	Promoter (<=1kb)	0679	255E-03	477E-02	Pgam5	0	Hyper
chr10	57079068	57088127	Exon (NM_001144991/100270678, exon 3 of 13)	0350	255E-03	477E-02	Spns2	17830	Hyper
chr2	215805134	215806920	Distal Intergenic	0721	256E-03	478E-02	Larp7	205889	Hyper
chr5	112747465	112751656	Intron (NM_012988/25492, intron 10 of 10)	0463	257E-03	479E-02	Nfia	305711	Hyper
chr1	46517990	46527348	Promoter (<=1kb)	0353	257E-03	479E-02	Synj2	0	Hyper

chr1	185501069	185502998	Intron (NM_031721/65164, intron 1 of 8)	0784	257E-03	479E-02	Htra1	3254	Hyper
chr2	135450111	135451265	Distal Intergenic	0575	257E-03	479E-02	Naa15	-6753	Hyper
chr6	120255085	120261032	Distal Intergenic	0441	257E-03	479E-02	Ppp4r3a	83561	Hyper
chr6	100465941	100470096	Distal Intergenic	0413	257E-03	479E-02	Plekhd1	125286	Hyper
chr5	130052954	130054995	Promoter (<=1kb)	0578	258E-03	479E-02	Ccdc17	0	Hyper
chr3	156483626	156486738	Distal Intergenic	0514	259E-03	481E-02	Cebpb	85591	Hyper
chr5	141121551	141123952	Exon (NM_001191612/297879, exon 6 of 8)	0603	259E-03	481E-02	Zfp362	17789	Hyper
chr19	47890761	47899160	Intron (NM_001108452/361422, intron 2 of 3)	0386	261E-03	485E-02	Cotl1	6850	Hyper
chr12	8143610	8145180	Promoter (<=1kb)	0631	265E-03	490E-02	Gtf3a	0	Hyper
chr8	67449300	67451180	Promoter (<=1kb)	0545	265E-03	490E-02	Aph1b	0	Hyper
chr5	128586185	128590187	Promoter (<=1kb)	0440	265E-03	491E-02	Tal1	0	Hyper
chr17	23505372	23507036	Intron (NM_001135576/690806, intron 1 of 1)	0731	268E-03	494E-02	Smim13	8669	Hyper
chr2	174802066	174814419	Promoter (2-3kb)	0294	268E-03	494E-02	Zbtb7b	-2622	Hyper
chr7	118802778	118805414	Intron (NM_001191991/500915, intron 3 of 3)	0562	269E-03	494E-02	Tafa5	188889	Hyper
chr13	82868396	82877768	Promoter (<=1kb)	0356	270E-03	496E-02	Olfml2b	0	Hyper
chr20	10440553	10443277	Promoter (1-2kb)	0556	271E-03	496E-02	Trappc10	1816	Hyper
chr3	117947191	117949801	Exon (NM_001109206/499891, exon 27 of 37)	0611	272E-03	498E-02	Slc4a11	-34517	Hyper
chr2	149883037	149884858	Distal Intergenic	0466	272E-03	498E-02	Lekr1	-6248	Hyper

Supplementary Table 2: Significantly hypoacetylated peaks in the substantia nigra

chr	*start*	*end*	*annotation*	*logFC*	*PValue*	*FDR*	*GENE*	*distanceToTSS*	*DAR*
chr10	24322063	24322570	Distal Intergenic	-2538	265E-20	101E-15	Mat2b	797925	Hypo
chr3	2234868	2235409	Promoter (<=1kb)	-2514	389E-13	185E-09	Rn5-8s	119	Hypo
chr1	55017846	55020490	Distal Intergenic	-1172	187E-11	595E-08	Vom2r9	159560	Hypo
chr3	2225900	2227414	Distal Intergenic	-2591	245E-11	721E-08	Rn18s	-4393	Hypo
chr1	211816885	211818368	Distal Intergenic	-1614	900E-11	209E-07	Tle4	-19023	Hypo
chr14	46237187	46237870	Distal Intergenic	-1146	907E-11	209E-07	Arap2	-480890	Hypo
chr9	10034566	10041042	Distal Intergenic	-1059	926E-11	209E-07	Adgre4	274211	Hypo
chr1	53338230	53341810	Intron (NM_001191619/292316, intron 1 of 1)	-1191	136E-10	263E-07	Smok2a	57863	Hypo
chr6	133340973	133348232	Distal Intergenic	-1198	165E-10	301E-07	Adam6	673999	Hypo
chr3	1259402	1260967	Distal Intergenic	-1092	185E-10	307E-07	Rn18s	-970840	Hypo
chr3	2264615	2265306	Distal Intergenic	-2255	305E-10	442E-07	LOC257642	-25594	Hypo
chr9	36409598	36412528	Intron (NM_001108208/316313, intron 35 of 92)	-1076	535E-10	707E-07	Dst	173513	Hypo
chr15	1053771	1055444	Distal Intergenic	-1301	752E-10	959E-07	Kcnma1	751694	Hypo
chr3	2262619	2263247	Distal Intergenic	-2471	100E-09	117E-06	LOC257642	-23598	Hypo
chrX	21273	24361	Distal Intergenic	-0960	128E-09	140E-06	Spaca5	897639	Hypo
chr3	1034229	1039159	Distal Intergenic	-1071	138E-09	147E-06	Rn18s	-1192648	Hypo
chr16	20214048	20221476	Distal Intergenic	-0977	158E-09	159E-06	Lzts1	-321333	Hypo
chr1	55025699	55036692	Distal Intergenic	-0892	192E-09	183E-06	Dact2	153247	Hypo
chr3	17588006	17590356	Intron (NM_001107834/311876, intron 2 of 8)	-1567	197E-09	183E-06	Pbx3	91994	Hypo
chr8	38212009	38215532	Distal Intergenic	-1026	211E-09	183E-06	Or8b101d	-87325	Hypo
chr3	195834	196970	Distal Intergenic	-1534	248E-09	211E-06	Rn18s	-2034837	Hypo
chr7	90697927	90700712	Distal Intergenic	-0999	355E-09	289E-06	Mtss1	-70085	Hypo
chr9	10068960	10074688	Distal Intergenic	-0995	400E-09	319E-06	Adgre4	308605	Hypo
chr16	20011905	20016368	Distal Intergenic	-1127	643E-09	464E-06	Zfp868	249176	Hypo
chr16	20020944	20024870	Distal Intergenic	-1012	682E-09	483E-06	Zfp868	258215	Hypo
chr17	82888771	82890510	Distal Intergenic	-1232	735E-09	504E-06	Arhgap21	581692	Hypo
chr16	20206385	20207899	Distal Intergenic	-1210	820E-09	541E-06	Lzts1	-334910	Hypo
chr4	147322531	147325420	Intron (NM_024372/79213, intron 4 of 13)	-1107	846E-09	549E-06	Slc6a11	24559	Hypo
chr16	20292297	20302936	Distal Intergenic	-0917	894E-09	570E-06	Lzts1	-239873	Hypo
chr1	53231478	53233627	Distal Intergenic	-1078	106E-08	668E-06	Smok2a	-46740	Hypo
chr3	28726945	28729508	Distal Intergenic	-0981	134E-08	830E-06	Gtdc1	432111	Hypo

chr6	19950637	19961446	Promoter (<=1kb)	-0651	139E-08	832E-06	Rasgrp3	0	Hypo
chr3	2265552	2266142	Distal Intergenic	-2041	141E-08	832E-06	LOC257642	-26531	Hypo
chr9	10025933	10031979	Distal Intergenic	-0896	141E-08	832E-06	Adgre4	265578	Hypo
chr10	12908471	12911361	Distal Intergenic	-1026	147E-08	853E-06	Csap1	-3004	Hypo
chr9	79940016	79943620	Exon (NM_001004252/301544, exon 2 of 17)	-0902	151E-08	864E-06	Farsb	3417	Hypo
chr1	55048753	55051549	Distal Intergenic	-0958	154E-08	867E-06	Dact2	138390	Hypo
chr1	53241244	53245020	Distal Intergenic	-0954	181E-08	100E-05	Smok2a	-35347	Hypo
chr3	1155863	1161827	Distal Intergenic	-0970	199E-08	109E-05	Rn18s	-1069980	Hypo
chr1	53333990	53338066	Intron (NM_001191619/292316, intron 1 of 1)	-1108	206E-08	111E-05	Smok2a	53623	Hypo
chr6	133383651	133391559	Distal Intergenic	-1022	227E-08	121E-05	Adam6	716677	Hypo
chr3	7172187	7173895	3' UTR	-1470	231E-08	121E-05	Psd4	35032	Hypo
chr19	21921891	21924048	Distal Intergenic	-0965	311E-08	159E-05	Or7c19	-88321	Hypo
chr4	175343778	175346129	3' UTR	-0933	335E-08	169E-05	Spx	-9915	Hypo
chr6	133392551	133401371	Distal Intergenic	-0982	343E-08	171E-05	Adam6	725577	Hypo
chr1	217998333	218001703	Distal Intergenic	-0933	349E-08	171E-05	Aldh1a1	-109382	Hypo
chr4	699561	706194	Distal Intergenic	-0970	359E-08	171E-05	Il6	4512984	Hypo
chr19	13779367	13780898	Distal Intergenic	-0984	365E-08	171E-05	Ces1f	-15725	Hypo
chr9	10032120	10034042	Distal Intergenic	-0992	366E-08	171E-05	Adgre4	271765	Hypo
chr18	12557057	12559303	Distal Intergenic	-1079	396E-08	180E-05	Mep1b	190931	Hypo
chr19	22349882	22351756	Distal Intergenic	-1163	429E-08	191E-05	LOC291863	-209014	Hypo
chr16	20244739	20246092	Distal Intergenic	-1210	446E-08	196E-05	Lzts1	-296717	Hypo
chr1	105495602	105496232	Distal Intergenic	-1617	573E-08	242E-05	Tubgcp5	-1140294	Hypo
chr1	53234230	53235559	Distal Intergenic	-1105	575E-08	242E-05	Smok2a	-44808	Hypo
chr6	133401551	133404425	Distal Intergenic	-1196	589E-08	245E-05	Adam6	734577	Hypo
chr8	66641070	66645326	Exon (NM_053411/84471, exon 5 of 15)	-0790	707E-08	291E-05	Snx1	23422	Hypo
chr3	1560357	1562807	Distal Intergenic	-1162	780E-08	318E-05	Rn18s	-669000	Hypo
chr2	39842254	39843652	Exon (NM_001191844/361895, exon 3 of 7)	-1374	813E-08	328E-05	Elovl7	12840	Hypo
chr10	87515714	87517235	Distal Intergenic	-1182	841E-08	335E-05	Fzd2	-44631	Hypo
chr12	16433738	16434759	3' UTR	-1465	852E-08	336E-05	Cyp3a62	35740	Hypo
chr6	133414129	133423055	Distal Intergenic	-0929	976E-08	374E-05	Adam6	747155	Hypo
chr18	48029926	48032731	Distal Intergenic	-0815	102E-07	386E-05	Zfp608	377734	Hypo
chr17	2202350	2204037	Distal Intergenic	-1122	103E-07	386E-05	Fbp1	-3994	Hypo
chr8	113447413	113450343	Distal Intergenic	-0904	104E-07	387E-05	Pdcd6ip	196430	Hypo

chr13	69704272	69706317	Exon (NM_053571/89868, exon 15 of 29)	-1128	109E-07	400E-05	Sec16b	19981	Hypo
chr3	153244598	153246579	Distal Intergenic	-1139	110E-07	402E-05	Pigt	16835	Hypo
chr7	32079614	32080576	Distal Intergenic	-0870	114E-07	413E-05	Dcn	-200676	Hypo
chr7	90729030	90731241	Distal Intergenic	-0943	120E-07	429E-05	Mtss1	-101188	Hypo
chr1	55047145	55048408	Distal Intergenic	-1067	124E-07	438E-05	Dact2	141531	Hypo
chr16	20249868	20252554	Distal Intergenic	-0975	126E-07	438E-05	Lzts1	-290255	Hypo
chr3	1048092	1050528	Distal Intergenic	-1154	135E-07	467E-05	Rn18s	-1181279	Hypo
chr1	53342020	53346839	Intron (NM_001191619/292316, intron 1 of 1)	-0892	144E-07	482E-05	Smok2a	61653	Hypo
chr8	68300419	68301845	Distal Intergenic	-1235	145E-07	482E-05	LOC100911360	35112	Hypo
chr5	147602040	147603669	Distal Intergenic	-1180	145E-07	482E-05	Srrm1	-10250	Hypo
chr17	8010318	8012234	Distal Intergenic	-1135	149E-07	491E-05	Tgfbi	-25480	Hypo
chr5	124552607	124555016	Intron (NM_130406/140657, intron 7 of 18)	-1046	159E-07	520E-05	Faf1	126543	Hypo
chr3	17586526	17587394	Intron (NM_001107834/311876, intron 2 of 8)	-1636	173E-07	562E-05	Pbx3	94956	Hypo
chr19	17645020	17646236	Distal Intergenic	-1588	177E-07	565E-05	Sall1	-361267	Hypo
chr1	53257393	53258415	Distal Intergenic	-1133	177E-07	565E-05	Smok2a	-21952	Hypo
chr1	176265579	176271182	Distal Intergenic	-0684	212E-07	666E-05	Usp31	-13206	Hypo
chr2	938051	938928	Distal Intergenic	-1071	215E-07	666E-05	Erap1	-2993014	Hypo
chr8	23675588	23690032	Distal Intergenic	-0601	220E-07	673E-05	LOC500959	-26487	Hypo
chr3	1129033	1133471	Distal Intergenic	-0979	251E-07	738E-05	Rn18s	-1098336	Hypo
chr18	14953638	14957835	Distal Intergenic	-0811	256E-07	749E-05	Mapre2	-113536	Hypo
chr3	118075991	118077965	Distal Intergenic	-0998	293E-07	830E-05	Dnaaf9	-23361	Hypo
chr3	28451756	28452967	Intron (NM_001013917/295635, intron 11 of 13)	-1464	302E-07	843E-05	Arhgap15	462123	Hypo
chr3	17585267	17586382	Intron (NM_001107834/311876, intron 2 of 8)	-1911	335E-07	929E-05	Pbx3	95968	Hypo
chr2	142958082	142961271	Distal Intergenic	-0909	350E-07	951E-05	Siah2	-26330	Hypo
chr14	4430661	4434742	Distal Intergenic	-0754	350E-07	951E-05	Lrrc8b	-69581	Hypo
chr3	1622824	1626000	Distal Intergenic	-0986	399E-07	107E-04	Rn18s	-605807	Hypo
chr4	524173	524762	Distal Intergenic	-1768	415E-07	110E-04	Il6	4694416	Hypo
chr16	20238715	20239849	Distal Intergenic	-1137	419E-07	111E-04	Lzts1	-302960	Hypo
chr3	1039322	1043388	Distal Intergenic	-0944	442E-07	114E-04	Rn18s	-1188419	Hypo
chr1	55068055	55069755	Distal Intergenic	-1220	444E-07	114E-04	Dact2	120184	Hypo
chr4	65384720	65386426	Distal Intergenic	-1231	448E-07	114E-04	Ptn	-9414	Hypo
chr3	1026888	1029831	Distal Intergenic	-1025	473E-07	119E-04	Rn18s	-1201976	Hypo
chr2	148873284	148876056	Distal Intergenic	-1122	506E-07	126E-04	Vom2r47	108943	Hypo

chr16	20240681	20241658	Distal Intergenic	-1168	518E-07	128E-04	Lzts1	-301151	Hypo
chr14	71613875	71616958	Distal Intergenic	-0829	524E-07	128E-04	Hs3st1	398856	Hypo
chr8	61633299	61635248	Distal Intergenic	-1113	526E-07	128E-04	LOC691000	126634	Hypo
chr3	1552016	1554952	Distal Intergenic	-1006	545E-07	132E-04	Rn18s	-676855	Hypo
chr7	129123195	129126632	Promoter (<=1kb)	-0763	568E-07	135E-04	Col2a1	914	Hypo
chr19	22355078	22356714	Distal Intergenic	-0907	569E-07	135E-04	LOC291863	-204056	Hypo
chr11	83919926	83922010	3' UTR	-0961	595E-07	141E-04	Ppil2	22157	Hypo
chr17	85134610	85140272	Intron (NM_024397/79249, intron 2 of 10)	-0674	602E-07	141E-04	Abi1	39481	Hypo
chr2	246278550	246280092	Intron (NM_021682/59318, intron 6 of 6)	-1066	607E-07	142E-04	Zranb2	-293287	Hypo
chr5	133597081	133598019	Distal Intergenic	-1456	618E-07	143E-04	Hivep3	-60033	Hypo
chr3	60961286	60962343	Intron (NM_001127481/140928, intron 15 of 19)	-1438	618E-07	143E-04	Ift70a1	-55936	Hypo
chr1	254831834	254833398	Intron (NM_001191052/679869, intron 4 of 14)	-1000	623E-07	143E-04	Tcf7l2	45743	Hypo
chr19	21915552	21915989	Distal Intergenic	-1278	627E-07	143E-04	Or7c19	-81982	Hypo
chr4	3690226	3691424	Distal Intergenic	-1585	661E-07	150E-04	Il6	1527754	Hypo
chr17	17852361	17856453	Intron (NM_001107462/308173, intron 2 of 39)	-0770	750E-07	166E-04	Kif13a	85764	Hypo
chr3	17579377	17580992	Intron (NM_001107834/311876, intron 2 of 8)	-1460	758E-07	167E-04	Pbx3	101358	Hypo
chr9	6724021	6725747	3' UTR	-1326	773E-07	169E-04	LOC301165	15968	Hypo
chr18	62369011	62370377	Distal Intergenic	-1316	849E-07	182E-04	Mc2r	-353523	Hypo
chr14	32251431	32252916	Exon (NM_013062/25589, exon 27 of 30)	-1147	860E-07	183E-04	Kdr	33545	Hypo
chr1	55054326	55057225	Distal Intergenic	-0962	878E-07	186E-04	Dact2	132714	Hypo
chr6	41677785	41679693	Intron (NM_053795/116478, intron 22 of 29)	-0974	900E-07	188E-04	Kidins220	59491	Hypo
chr9	2908962	2911156	Distal Intergenic	-0938	901E-07	188E-04	Plcl2	-382212	Hypo
chr13	33789031	33792241	Distal Intergenic	-1036	103E-06	210E-04	Ddx18	-1045870	Hypo
chr7	27337914	27340193	Distal Intergenic	-0865	106E-06	214E-04	Nedd1	-63428	Hypo
chr19	13765587	13767573	3' UTR	-0873	106E-06	214E-04		27707	Hypo
chr9	10974504	10975640	Intron (NM_001135011/501095, intron 7 of 9)	-1533	106E-06	214E-04	Rftn1	169893	Hypo
chr7	17278146	17279402	Distal Intergenic	-1345	107E-06	214E-04	Syn3	-110734	Hypo
chr15	47721833	47722912	Intron (NM_001107278/306022, intron 8 of 22)	-1454	112E-06	221E-04	Fndc3a	109668	Hypo
chr1	55037929	55038854	Distal Intergenic	-1489	116E-06	226E-04	Dact2	151085	Hypo
chr13	33625031	33625992	Distal Intergenic	-1588	116E-06	226E-04	Ddx18	-881870	Hypo
chr4	123843611	123845582	Distal Intergenic	-1233	116E-06	226E-04	Wnt7a	63399	Hypo
chr19	4866149	4868462	Distal Intergenic	-0901	124E-06	237E-04	Cdh8	-625712	Hypo
chr1	151525669	151527310	Intron (NM_053417/84477, intron 1 of 9)	-0983	126E-06	239E-04	Gab2	95715	Hypo

chr10	84445179	84447754	Promoter (2-3kb)	-0945	132E-06	249E-04	Krt23	2968	Hypo
chr4	124184936	124186617	Distal Intergenic	-0983	135E-06	253E-04	Slc6a6	-8730	Hypo
chr16	58820058	58821985	Distal Intergenic	-0958	135E-06	253E-04	Purg	-57026	Hypo
chr18	74706976	74709761	Distal Intergenic	-0780	139E-06	257E-04	Sall3	-293148	Hypo
chr18	47982038	47984279	Distal Intergenic	-0917	146E-06	265E-04	Zfp608	426186	Hypo
chrX	132956136	132958265	Distal Intergenic	-1028	146E-06	265E-04	Pabir2	56807	Hypo
chr3	1547458	1548618	Distal Intergenic	-1282	146E-06	265E-04	Rn18s	-683189	Hypo
chr9	50778072	50783893	Distal Intergenic	-0750	151E-06	271E-04	Tmeff2	-44597	Hypo
chr18	52790637	52791841	Distal Intergenic	-1197	176E-06	309E-04	Minar2	250720	Hypo
chr1	241122814	241125183	Intron (NM_134401/171438, intron 5 of 14)	-0810	176E-06	309E-04	Mir3085	-33821	Hypo
chr19	27183349	27216019	Exon (NM_001108444/361388, exon 2 of 10)	-0486	177E-06	310E-04	Gab1	52087	Hypo
chr6	105699336	105700938	Distal Intergenic	-1115	179E-06	312E-04	Tgfb3	25037	Hypo
chr3	63977999	63979133	Distal Intergenic	-1304	189E-06	325E-04	Itga4	-183952	Hypo
chr9	50754130	50758088	Distal Intergenic	-0737	193E-06	330E-04	Tmeff2	-20655	Hypo
chr2	163776231	163778565	Distal Intergenic	-0804	194E-06	330E-04	Rapgef2	543592	Hypo
chr2	203027889	203030137	Distal Intergenic	-0971	208E-06	347E-04	Olfm3	117296	Hypo
chr17	45551830	45553417	Distal Intergenic	-1043	208E-06	347E-04	Stard3nl	-29861	Hypo
chr18	74755542	74757910	Distal Intergenic	-0803	213E-06	353E-04	Sall3	-341714	Hypo
chr5	101602592	101608629	Intron (NM_031743/84550, intron 1 of 9)	-0645	218E-06	358E-04	Slc24a2	130708	Hypo
chr7	68238491	68240842	Distal Intergenic	-0813	218E-06	358E-04	Zfp706	-64449	Hypo
chr7	30884845	30887321	Distal Intergenic	-0876	221E-06	361E-04	Eea1	279852	Hypo
chr11	54877893	54879844	Distal Intergenic	-1084	222E-06	361E-04	Plcxd2	76893	Hypo
chr1	136218495	136222530	Exon (NM_031238/81921, exon 4 of 9)	-0676	225E-06	364E-04	Adamtsl3	-66263	Hypo
chr10	79188445	79190751	Distal Intergenic	-0790	228E-06	367E-04	Tob1	25352	Hypo
chr16	20246641	20248509	Distal Intergenic	-0934	241E-06	385E-04	Lzts1	-294300	Hypo
chr18	48989108	48992663	Distal Intergenic	-0745	247E-06	393E-04	Zfp608	-578643	Hypo
chr18	55246692	55248372	Promoter (2-3kb)	-0963	250E-06	395E-04	Afap1l1	-2546	Hypo
chr2	86654086	86656481	Distal Intergenic	-0859	252E-06	395E-04		53558	Hypo
chr18	25654563	25656042	Distal Intergenic	-1084	271E-06	422E-04	Epb41l4a	-167464	Hypo
chr15	46100438	46101895	Distal Intergenic	-1162	276E-06	428E-04	Gfra2	157987	Hypo
chr5	59627369	59631831	Exon (NM_001107935/313242, exon 3 of 7)	-0682	288E-06	440E-04	Dcaf10	19509	Hypo
chr1	53349950	53353549	Intron (NM_001191619/292316, intron 1 of 1)	-0914	298E-06	452E-04	Smok2a	69583	Hypo
chr3	1199461	1201613	Distal Intergenic	-0983	299E-06	452E-04	Rn18s	-1030194	Hypo

chr8	72779775	72786989	Intron (NM_013176/25720, intron 3 of 20)	-0607	309E-06	466E-04	Tcf12	12212	Hypo
chr4	123033292	123034934	Distal Intergenic	-0973	318E-06	473E-04	Aldh1l1	-25083	Hypo
chr18	51981345	51987562	Distal Intergenic	-0592	336E-06	493E-04	Slc27a6	-53512	Hypo
chr10	80570465	80572525	Distal Intergenic	-0851	343E-06	499E-04	Phb1	-32743	Hypo
chr5	74460177	74462621	Exon (NM_001025697/313200, exon 7 of 11)	-0849	345E-06	500E-04	Hsdl2	16244	Hypo
chr1	53262663	53265240	Distal Intergenic	-0744	346E-06	500E-04	Smok2a	-15127	Hypo
chr1	55057515	55058959	Distal Intergenic	-0993	349E-06	500E-04	Dact2	130980	Hypo
chr5	46717704	46719502	Promoter (<=1kb)	-0962	349E-06	500E-04	Bach2	683	Hypo
chr3	146004348	146008157	Intron (NM_031577/29446, intron 1 of 4)	-0741	351E-06	500E-04	Ghrh	3732	Hypo
chr19	48367674	48370755	Distal Intergenic	-0672	374E-06	526E-04	6430548M08Rikl	169465	Hypo
chr19	21916179	21917391	Distal Intergenic	-0903	379E-06	529E-04	Or7c19	-82609	Hypo
chr3	60951834	60954431	Exon (NM_001127481/140928, exon 16 of 20)	-0810	395E-06	550E-04	Ift70a1	-46484	Hypo
chr4	167479833	167482062	Intron (NM_001108650/362452, intron 2 of 3)	-0891	412E-06	570E-04	Borcs5	6460	Hypo
chr1	13960302	13962606	Distal Intergenic	-1043	413E-06	570E-04	Olig3	-118653	Hypo
chr3	17532611	17543903	Exon (NM_001107834/311876, exon 3 of 9)	-0558	419E-06	575E-04	Pbx3	138447	Hypo
chr17	56542973	56544515	Distal Intergenic	-1042	419E-06	575E-04	Wac	619850	Hypo
chr9	9994710	9997315	Distal Intergenic	-0729	430E-06	586E-04	Adgre4	234355	Hypo
chr1	193671377	193677599	Intron (NM_144746/246255, intron 2 of 8)	-0703	438E-06	592E-04	Ppp2r2d	5414	Hypo
chr2	233835925	233839856	Exon (NM_001077356/499721, exon 6 of 14)	-0711	439E-06	592E-04	Clca4l	6281	Hypo
chr17	56570656	56573189	Distal Intergenic	-0779	440E-06	592E-04	Wac	647533	Hypo
chr3	1077069	1077814	Distal Intergenic	-1500	441E-06	592E-04	Rn18s	-1153993	Hypo
chr14	102691178	102693106	Distal Intergenic	-0989	444E-06	592E-04	Efemp1	80270	Hypo
chr6	133334386	133338345	Distal Intergenic	-0912	444E-06	592E-04	Adam6	667412	Hypo
chr1	55062834	55063810	Distal Intergenic	-1410	474E-06	621E-04	Dact2	126129	Hypo
chr4	108506203	108507381	Distal Intergenic	-1320	477E-06	623E-04	LRRTM1	-1194434	Hypo
chr1	55052521	55053558	Distal Intergenic	-0995	480E-06	625E-04	Dact2	136381	Hypo
chr13	21387836	21389673	Intron (NM_001106305/293561, intron 2 of 2)	-1014	485E-06	630E-04	Rnf152	13427	Hypo
chr19	13775660	13777122	Distal Intergenic	-0854	498E-06	644E-04	Ces1f	-19501	Hypo
chr3	123684672	123692802	Intron (NM_001107781/311450, intron 1 of 10)	-0626	499E-06	644E-04	Pak5	11128	Hypo
chr6	133570900	133573040	Distal Intergenic	-1071	516E-06	661E-04	Adam6	903926	Hypo
chr3	1043772	1047946	Distal Intergenic	-0822	528E-06	672E-04	Rn18s	-1183861	Hypo
chr4	116089528	116091728	Distal Intergenic	-0736	530E-06	672E-04	Stambp	-8980	Hypo
chr16	38405813	38408095	Exon (NM_001170346/290729, exon 8 of 11)	-0812	552E-06	695E-04	Neil3	46508	Hypo

chr19	13769777	13772389	Distal Intergenic	-0710	559E-06	700E-04	Ces1f	-24234	Hypo
chr1	204341116	204342829	Intron (NM_139337/246233, intron 3 of 10)	-0883	568E-06	708E-04	Otub1	52666	Hypo
chr6	72555301	72557034	Exon (NM_001108022/314128, exon 5 of 8)	-0892	575E-06	712E-04	RGD1304624	30136	Hypo
chr1	53235718	53241037	Distal Intergenic	-0818	593E-06	730E-04	Smok2a	-39330	Hypo
chr3	935955	937583	Distal Intergenic	-0982	600E-06	736E-04	Rn18s	-1294224	Hypo
chr12	21206	21853	Distal Intergenic	-1066	602E-06	736E-04	Brca2	-37966	Hypo
chr3	1133722	1134508	Distal Intergenic	-1459	614E-06	749E-04	Rn18s	-1097299	Hypo
chr3	110515308	110516625	Distal Intergenic	-1236	624E-06	756E-04	Sqor	668658	Hypo
chr3	29519767	29520450	Distal Intergenic	-1421	642E-06	775E-04	Zeb2-as1	176689	Hypo
chr9	23307267	23308536	Distal Intergenic	-1061	656E-06	788E-04	Paqr8	-33070	Hypo
chr2	171415021	171416491	Distal Intergenic	-1137	659E-06	788E-04	Rps3a	112563	Hypo
chr14	46240510	46241192	Distal Intergenic	-1337	670E-06	794E-04	Arap2	-477568	Hypo
chr6	35598051	35601554	Distal Intergenic	-0676	671E-06	794E-04	Mycn	122036	Hypo
chr3	17568398	17574356	Intron (NM_001107834/311876, intron 2 of 8)	-0692	671E-06	794E-04	Pbx3	107994	Hypo
chr16	67898458	67899296	Distal Intergenic	-1259	678E-06	799E-04	Tcim	302696	Hypo
chr20	17837537	17841268	Distal Intergenic	-0664	699E-06	820E-04	Phyhipl	-190537	Hypo
chr4	179245510	179248790	Intron (NM_031046/81678, intron 26 of 56)	-0674	700E-06	820E-04	Itpr2	155374	Hypo
chr1	53347342	53349798	Intron (NM_001191619/292316, intron 1 of 1)	-1007	716E-06	836E-04	Smok2a	66975	Hypo
chr10	101131966	101135818	Promoter (<=1kb)	-0703	721E-06	838E-04	Smim5	0	Hypo
chr3	1485107	1487428	Distal Intergenic	-0899	724E-06	840E-04	Rn18s	-744379	Hypo
chr19	21918420	21921254	Distal Intergenic	-0738	728E-06	841E-04	Or7c19	-84850	Hypo
chr3	27566040	27567794	Distal Intergenic	-0890	742E-06	855E-04	Kynu	-210961	Hypo
chr12	15773743	15776594	Exon (NM_001047924/498155, exon 2 of 11)	-0820	745E-06	856E-04		10249	Hypo
chr18	47970503	47974327	Distal Intergenic	-0661	748E-06	857E-04	Zfp608	436138	Hypo
chr15	4736243	4738648	Promoter (1-2kb)	-0925	757E-06	863E-04	Spetex2a	-1090	Hypo
chr6	133546225	133548149	Distal Intergenic	-1198	767E-06	869E-04	Adam6	879251	Hypo
chr10	93799888	93802168	Intron (NM_001105849/287776, intron 24 of 26)	-0802	772E-06	869E-04	Axin2	-97077	Hypo
chr3	1481411	1482685	Distal Intergenic	-1236	772E-06	869E-04	Rn18s	-749122	Hypo
chr8	38172216	38175013	Distal Intergenic	-0822	789E-06	883E-04	Or8b101c	104399	Hypo
chr3	17582526	17583320	Intron (NM_001107834/311876, intron 2 of 8)	-1574	796E-06	888E-04	Pbx3	99030	Hypo
chr14	94001459	94003112	Distal Intergenic	-0952	808E-06	899E-04	Spred2	-146582	Hypo
chr13	64936343	64938285	Distal Intergenic	-0754	819E-06	903E-04	Ncf2	-17383	Hypo
chr1	55070024	55072057	Distal Intergenic	-0985	840E-06	919E-04	Dact2	117882	Hypo

chr4	9729784	9731635	Distal Intergenic	-0811	872E-06	948E-04	Cct8l2	-122813	Hypo
chr3	984988	986403	Distal Intergenic	-1226	879E-06	951E-04	Rn18s	-1245404	Hypo
chr12	4882881	4884411	Promoter (<=1kb)	-1179	880E-06	951E-04	Lnc001	455	Hypo
chr6	132738350	132740561	Distal Intergenic	-0834	888E-06	955E-04	Adam6	71376	Hypo
chr6	133411704	133413959	Distal Intergenic	-0932	888E-06	955E-04	Adam6	744730	Hypo
chr14	93042263	93045318	Distal Intergenic	-0726	903E-06	968E-04	Meis1	248277	Hypo
chrX	25073	25831	Distal Intergenic	-1119	919E-06	983E-04	Spaca5	896169	Hypo
chr3	150379988	150381138	Intron (NM_001108603/362263, intron 11 of 27)	-1047	926E-06	988E-04	Ptprt	607525	Hypo
chr5	114861785	114862714	Intron (NM_001108671/362550, intron 1 of 8)	-1304	935E-06	990E-04	Ror1	117474	Hypo
chr4	116057296	116059112	Intron (NM_138531/171565, intron 10 of 10)	-0932	939E-06	990E-04	Actg2	-20938	Hypo
chr6	98815986	98817679	Distal Intergenic	-1115	953E-06	996E-04	Zfp36l1	118069	Hypo
chr9	87355993	87359644	Promoter (<=1kb)	-0660	955E-06	996E-04	Dis3l2	0	Hypo
chr1	204828754	204830168	Distal Intergenic	-0954	961E-06	998E-04	Lgals12	12591	Hypo
chr19	13778701	13779209	Distal Intergenic	-1164	962E-06	998E-04	Ces1f	-17414	Hypo
chr6	88899721	88901294	Distal Intergenic	-1002	974E-06	101E-03	Trim9	-43179	Hypo
chr12	28012995	28014304	Distal Intergenic	-1197	977E-06	101E-03	Piwil1	-42415	Hypo
chr9	9987063	9991327	Distal Intergenic	-0588	997E-06	102E-03	Adgre4	226708	Hypo
chr9	89428753	89430874	Distal Intergenic	-0755	101E-05	103E-03	Arl4c	-124983	Hypo
chr2	173661107	173674800	Promoter (2-3kb)	-0486	104E-05	106E-03	Mir9-1	-2339	Hypo
chr15	44792167	44797530	3' UTR	-0632	105E-05	106E-03	Chmp7	8686	Hypo
chr1	126944859	126946463	Distal Intergenic	-0828	108E-05	109E-03	Rgma	-182471	Hypo
chr1	40353219	40359087	Intron (NM_001190999/679812, intron 2 of 15)	-0666	109E-05	110E-03	Plekhg1	4535	Hypo
chr3	21274652	21275444	Promoter (<=1kb)	-1116	111E-05	111E-03	Gpr21	0	Hypo
chr6	133595698	133598742	Distal Intergenic	-0950	113E-05	113E-03	Adam6	928724	Hypo
chr3	104603220	104604596	Distal Intergenic	-1098	115E-05	115E-03	Rasgrp1	-373164	Hypo
chr3	1469503	1471356	Distal Intergenic	-1081	117E-05	116E-03	Rn18s	-760451	Hypo
chr14	4030037	4031301	3' UTR	-0992	117E-05	116E-03	Lrrc8d	14083	Hypo
chr13	73115958	73118146	Distal Intergenic	-0742	118E-05	117E-03	Rabgap1l	-124592	Hypo
chr8	23697638	23711759	Distal Intergenic	-0515	119E-05	117E-03	Septin7	-8012	Hypo
chr2	148888587	148890297	Distal Intergenic	-1029	121E-05	119E-03	Vom2r47	94702	Hypo
chr6	133601869	133604658	Distal Intergenic	-0959	126E-05	123E-03	Adam6	934895	Hypo
chr1	2097735	2099272	Intron (NM_001106217/292462, intron 6 of 7)	-0963	126E-05	123E-03	Lrp11	18131	Hypo
chr16	51370549	51372836	Intron (NM_001011921/290771, intron 2 of 5)	-0744	127E-05	124E-03	Pdgfrl	35014	Hypo

chr10	8052662	8054939	Distal Intergenic	-0783	129E-05	125E-03	Rbfox1	437834	Hypo
chr16	20287420	20287939	Distal Intergenic	-1361	140E-05	135E-03	Lzts1	-254870	Hypo
chr2	142350513	142351528	Distal Intergenic	-1123	142E-05	136E-03	Pfn2	-277705	Hypo
chr1	230165283	230167783	Intron (NM_181386/353229, intron 2 of 9)	-0906	148E-05	141E-03	Sgms1	91809	Hypo
chr2	185639537	185641217	Intron (NM_024358/29492, intron 1 of 33)	-0810	151E-05	143E-03	Notch2	28943	Hypo
chr6	133259049	133263367	Distal Intergenic	-0903	153E-05	145E-03	Adam6	592075	Hypo
chr16	20253032	20254367	Distal Intergenic	-0966	153E-05	145E-03	Lzts1	-288442	Hypo
chr2	160150532	160153276	Distal Intergenic	-0905	154E-05	145E-03	Serpini2	-106261	Hypo
chr3	79727848	79728703	Intron (NM_001107751/311215, intron 8 of 12)	-1620	163E-05	152E-03	Ext2	69356	Hypo
chr12	28192793	28193743	Distal Intergenic	-1308	165E-05	153E-03	Tmem132d	-76969	Hypo
chr13	20819286	20821367	Distal Intergenic	-0759	167E-05	155E-03	Cdh20	-179292	Hypo
chr1	50338320	50339612	Distal Intergenic	-1151	172E-05	159E-03	Cahm	47347	Hypo
chr3	1626173	1627570	Distal Intergenic	-1142	174E-05	160E-03	Rn18s	-604237	Hypo
chr9	10041801	10042981	Distal Intergenic	-1079	175E-05	161E-03	Adgre4	281446	Hypo
chr8	105675903	105677479	Intron (NM_001106852/300974, intron 4 of 9)	-1025	176E-05	162E-03	Mrpl3	5719	Hypo
chr4	178934046	178936189	Distal Intergenic	-0797	177E-05	162E-03	Sspn	36899	Hypo
chr1	163957611	163971921	Exon (NM_001107546/308942, exon 2 of 23)	-0455	179E-05	163E-03	Dennd5a	22237	Hypo
chr20	20620807	20627031	Distal Intergenic	-0613	179E-05	163E-03	Rtkn2	-10856	Hypo
chr18	62547670	62550322	Distal Intergenic	-0806	189E-05	171E-03	Tcf4	-509134	Hypo
chr16	20290604	20291982	Distal Intergenic	-0918	191E-05	172E-03	Lzts1	-250827	Hypo
chr6	133439461	133440501	Distal Intergenic	-1286	195E-05	175E-03	Adam6	772487	Hypo
chr11	20152734	20155564	Intron (NM_203409/288280, intron 1 of 17)	-0755	196E-05	175E-03	Ncam2	47688	Hypo
chr10	9137912	9139709	Distal Intergenic	-0786	196E-05	175E-03	Rbfox1	-645139	Hypo
chr18	29924280	29925851	Distal Intergenic	-1186	198E-05	177E-03	Fchsd1	-119814	Hypo
chr2	76391641	76400931	Exon (NM_001034912/619558, exon 3 of 9)	-0507	202E-05	179E-03	Retreg1	56032	Hypo
chr5	123333386	123335310	Exon (NM_177931/313479, exon 4 of 16)	-0836	204E-05	180E-03	Orc1	5050	Hypo
chr9	98655099	98657399	Distal Intergenic	-0787	206E-05	181E-03	Nudt12	97640	Hypo
chr6	83443206	83444139	Distal Intergenic	-1157	207E-05	182E-03	Mis18bp1	-211823	Hypo
chrX	100104202	100110797	3' UTR	-0580	210E-05	184E-03	Morf4l2	-10544	Hypo
chr1	230176323	230179599	Intron (NM_181386/353229, intron 2 of 9)	-0730	210E-05	184E-03	Sgms1	79993	Hypo
chr10	30602507	30606559	Promoter (1-2kb)	-0660	216E-05	187E-03	Nipal4	-1867	Hypo
chr7	35726372	35727243	Distal Intergenic	-1597	217E-05	188E-03	LOC500827	314817	Hypo
chr9	10098745	10099730	Distal Intergenic	-1112	219E-05	189E-03	Adgre4	338390	Hypo

chr6	9897970	9903493	Exon (NM_001008519/313867, exon 21 of 38)	-0601	223E-05	191E-03	Lrpprc	38103	Hypo
chr7	90738383	90740475	Distal Intergenic	-0858	227E-05	194E-03	Mtss1	-110541	Hypo
chr10	71494557	71499815	Intron (NM_138839/192129, intron 1 of 11)	-0649	227E-05	194E-03	Vmp1	5181	Hypo
chr10	92971038	92978707	Intron (NM_001105713/24680, intron 5 of 15)	-0488	228E-05	194E-03	Cacng5	-134037	Hypo
chr6	133405855	133408511	Distal Intergenic	-0943	229E-05	195E-03	Adam6	738881	Hypo
chr18	51963887	51966814	Distal Intergenic	-0694	238E-05	201E-03	Slc27a6	-74260	Hypo
chr15	87552942	87554767	Distal Intergenic	-1089	241E-05	203E-03	Slitrk6	15358	Hypo
chr2	148879883	148881942	Distal Intergenic	-0968	243E-05	203E-03	Vom2r47	103057	Hypo
chr4	147337450	147340417	Intron (NM_024372/79213, intron 5 of 13)	-0743	251E-05	208E-03	Slc6a11	39478	Hypo
chr8	49975979	49988467	Intron (NM_031521/24586, intron 1 of 18)	-0461	255E-05	212E-03	Ttc12	-130294	Hypo
chr3	427545	428636	Distal Intergenic	-1279	261E-05	216E-03	Rn18s	-1803171	Hypo
chr3	1619793	1621575	Distal Intergenic	-1086	265E-05	218E-03	Rn18s	-610232	Hypo
chr9	2905805	2907362	Distal Intergenic	-1124	265E-05	218E-03	Plcl2	-386006	Hypo
chr10	30636837	30638879	Intron (NM_001106996/303073, intron 25 of 29)	-0737	267E-05	219E-03	Nipal4	-36197	Hypo
chr16	64152810	64154680	Distal Intergenic	-0792	269E-05	219E-03	Smok	-303696	Hypo
chr4	130644327	130648175	Distal Intergenic	-0664	278E-05	225E-03	Mitf	235110	Hypo
chr17	82882917	82884956	Distal Intergenic	-0889	281E-05	227E-03	Arhgap21	587246	Hypo
chr19	22352342	22353507	Distal Intergenic	-0829	282E-05	227E-03	LOC291863	-207263	Hypo
chr15	80510940	80514148	Intron (NM_001014139/361087, intron 1 of 5)	-0628	284E-05	228E-03	Slain1	12293	Hypo
chr15	7522343	7525921	Promoter (<=1kb)	-0566	290E-05	230E-03	Nr1d2	0	Hypo
chr1	130960196	130962344	Distal Intergenic	-0712	290E-05	230E-03	Klhl25	1235246	Hypo
chr6	133587318	133588228	Distal Intergenic	-1376	296E-05	234E-03	Adam6	920344	Hypo
chr10	72956789	72959340	Intron (NM_001013971/303419, intron 1 of 10)	-0581	297E-05	234E-03	Mrps23	-39693	Hypo
chr1	78959344	78960275	Promoter (<=1kb)	-1187	301E-05	237E-03	Fosb	897	Hypo
chr18	51027557	51028275	Distal Intergenic	-1315	312E-05	243E-03	LOC100910189	-78898	Hypo
chr1	189408390	189409606	Distal Intergenic	-0974	312E-05	243E-03	Dock1	-57537	Hypo
chr11	71931366	71934325	Distal Intergenic	-0769	317E-05	246E-03	Mb21d2	105618	Hypo
chr20	17813092	17815190	Distal Intergenic	-0734	321E-05	248E-03	Phyhipl	-216615	Hypo
chr3	80568	81658	Distal Intergenic	-1130	331E-05	256E-03	Rn18s	-2150149	Hypo
chr8	67913706	67915318	Distal Intergenic	-0897	333E-05	256E-03	Mir190a-2	-62635	Hypo
chr12	42797133	42799094	Distal Intergenic	-0962	334E-05	256E-03	Selplg	-8784	Hypo
chr5	116201376	116207409	Intron (NM_001107949/313409, intron 1 of 17)	-0556	337E-05	258E-03	Dnajc6	70700	Hypo
chr1	50276520	50279225	Intron (NM_001077677/499021, intron 4 of 4)	-0775	339E-05	258E-03	Cahm	107734	Hypo

chr5	120479845	120482442	Distal Intergenic	-0729	340E-05	259E-03	Plpp3	552760	Hypo
chr3	150712917	150716601	Intron (NM_001108603/362263, intron 6 of 27)	-0655	341E-05	259E-03	Ptprt	272062	Hypo
chr4	30429968	30434969	Exon (NM_001134781/368062, exon 11 of 20)	-0593	344E-05	261E-03	Tmbim7	64287	Hypo
chr3	1084657	1086788	Distal Intergenic	-1031	358E-05	270E-03	Rn18s	-1145019	Hypo
chr12	26906417	26909232	Intron (NM_001017486/498174, intron 10 of 10)	-0648	359E-05	270E-03	Psph	15431	Hypo
chr3	1094755	1095852	Distal Intergenic	-1184	362E-05	271E-03	Rn18s	-1135955	Hypo
chr4	42253004	42253861	Distal Intergenic	-1224	365E-05	272E-03	LOC100363521	-109664	Hypo
chr5	100401955	100403456	Distal Intergenic	-1088	368E-05	274E-03	Rraga	-709885	Hypo
chr19	15457642	15458642	Intron (NM_001039713/291905, intron 8 of 8)	-1116	370E-05	275E-03	Fto	233414	Hypo
chr9	107003261	107005536	Intron (NM_001168632/29616, intron 7 of 32)	-0764	376E-05	279E-03	Ptprm	338162	Hypo
chr8	121467023	121472706	Distal Intergenic	-0479	379E-05	280E-03	Hhatl	-13022	Hypo
chr10	64605314	64607850	Intron (NM_001107023/303337, intron 3 of 14)	-0749	383E-05	282E-03	Rab11fip4	55137	Hypo
chr3	29587846	29590488	Distal Intergenic	-0643	389E-05	285E-03	Zeb2-as1	244768	Hypo
chr5	75779900	75784644	Distal Intergenic	-0581	389E-05	285E-03	Slc31a1	-30614	Hypo
chr3	463527	464608	Distal Intergenic	-0812	392E-05	286E-03	Rn18s	-1767199	Hypo
chr4	162631892	162634558	Distal Intergenic	-0790	393E-05	286E-03	Clec2g	13980	Hypo
chr10	70524242	70525296	Intron (NM_001173430/363662, intron 24 of 25)	-1221	397E-05	288E-03	Tbx2	-154931	Hypo
chr1	140515267	140517533	Distal Intergenic	-0791	400E-05	289E-03	Folh1	-13888	Hypo
chr3	1025262	1026485	Distal Intergenic	-1136	408E-05	295E-03	Rn18s	-1205322	Hypo
chr13	89135327	89138270	Intron (NM_031575/29414, intron 1 of 11)	-0819	410E-05	295E-03	Akt3	77566	Hypo
chr4	24392003	24399153	Intron (NM_001105712/24416, intron 1 of 5)	-0487	422E-05	303E-03	Grm3	26888	Hypo
chr3	1072245	1073967	Distal Intergenic	-1022	425E-05	304E-03	Rn18s	-1157840	Hypo
chr3	1058490	1059746	Distal Intergenic	-1063	435E-05	309E-03	Rn18s	-1172061	Hypo
chr9	39046689	39048084	Distal Intergenic	-0920	435E-05	309E-03	Zap70	56939	Hypo
chr2	173696844	173704405	Distal Intergenic	-0510	441E-05	313E-03	Rhbg	12916	Hypo
chr1	170519447	170521486	Distal Intergenic	-0836	451E-05	319E-03	Rps13	53869	Hypo
chrX	139808652	139809802	Distal Intergenic	-0901	455E-05	319E-03	Magec2	-800808	Hypo
chr9	9992727	9993658	Distal Intergenic	-0937	456E-05	319E-03	Adgre4	232372	Hypo
chr3	153565884	153567258	Promoter (1-2kb)	-0959	456E-05	319E-03	Spata25	-1404	Hypo
chr10	6814568	6815200	Distal Intergenic	-1279	456E-05	319E-03	Hapstr1	-39576	Hypo
chr1	54944945	54945597	Distal Intergenic	-1184	471E-05	328E-03	Vom2r9	86659	Hypo
chr15	4752574	4753581	Intron (NM_001009976/364261, intron 1 of 3)	-1263	476E-05	330E-03	Spetex2b	12803	Hypo
chr6	133381562	133383318	Distal Intergenic	-0977	496E-05	342E-03	Adam6	714588	Hypo

chr1	47800218	47805199	Distal Intergenic	-0515	508E-05	349E-03	Tcp1	31545	Hypo
chr2	81286787	81289453	Distal Intergenic	-0837	510E-05	350E-03	Ctnnd2	-40201	Hypo
chr9	57414636	57416189	Distal Intergenic	-0837	517E-05	353E-03	Plcl1	513063	Hypo
chr16	300160	301491	Distal Intergenic	-1109	523E-05	357E-03	Rps24	210523	Hypo
chr1	91535733	91537124	Distal Intergenic	-1013	526E-05	358E-03	LOC365238	-452343	Hypo
chr19	55445223	55449143	Exon (NM_031235/81918, exon 20 of 25)	-0583	528E-05	359E-03	Pard3	364941	Hypo
chr19	52588026	52589772	Distal Intergenic	-0786	532E-05	359E-03	Capn9	38578	Hypo
chr3	1182962	1184348	Distal Intergenic	-0944	536E-05	362E-03	Rn18s	-1047459	Hypo
chr13	49280776	49286868	Distal Intergenic	-0543	539E-05	363E-03	Mir181a-1	-198659	Hypo
chr2	157993349	157995612	Distal Intergenic	-0687	542E-05	364E-03	Slitrk3	-296794	Hypo
chr2	117312698	117319147	Distal Intergenic	-0429	547E-05	367E-03	Sox2	-217782	Hypo
chr3	193986	195205	Distal Intergenic	-1027	554E-05	371E-03	Rn18s	-2036602	Hypo
chr18	51103621	51106698	Distal Intergenic	-0717	555E-05	371E-03	LOC100910189	-154962	Hypo
chr3	1089873	1091317	Distal Intergenic	-1089	562E-05	375E-03	Rn18s	-1140490	Hypo
chr12	20317963	20320714	Intron (NM_053860/116639, intron 8 of 22)	-0804	587E-05	389E-03	Sh2b2	-108329	Hypo
chr1	221637281	221640667	Distal Intergenic	-0720	587E-05	389E-03	Tjp2	197628	Hypo
chr12	42789590	42791266	Distal Intergenic	-1005	595E-05	393E-03	Selplg	-16612	Hypo
chr5	73557314	73560733	Distal Intergenic	-0556	609E-05	399E-03	Or2k2	22894	Hypo
chr3	17583594	17584863	Intron (NM_001107834/311876, intron 2 of 8)	-1168	620E-05	402E-03	Pbx3	97487	Hypo
chr18	14950725	14953222	Distal Intergenic	-0927	621E-05	402E-03	Mapre2	-118149	Hypo
chr13	69371702	69373318	Intron (NM_001107188/304893, intron 1 of 13)	-0831	634E-05	409E-03	Rasal2	58317	Hypo
chrX	11121677	11122968	Distal Intergenic	-1005	640E-05	411E-03	Bcor	433945	Hypo
chr13	21382249	21383587	Intron (NM_001106305/293561, intron 2 of 2)	-0864	648E-05	416E-03	Rnf152	19513	Hypo
chr5	158597319	158599369	3' UTR	-0773	653E-05	418E-03	Fbxo2	4393	Hypo
chr2	76386006	76391464	Intron (NM_001034912/619558, intron 2 of 8)	-0501	666E-05	426E-03	Retreg1	50397	Hypo
chr9	107039948	107041066	Intron (NM_001168632/29616, intron 4 of 32)	-0909	679E-05	433E-03	Ptprm	302632	Hypo
chr19	13755904	13758170	Intron (NM_001014221/363337, intron 1 of 4)	-0731	680E-05	433E-03		18024	Hypo
chr5	129617099	129620596	3' UTR	-0558	681E-05	433E-03	Lurap1	8055	Hypo
chr3	150719284	150724548	Intron (NM_001108603/362263, intron 6 of 27)	-0554	710E-05	449E-03	Ptprt	264115	Hypo
chr17	53731024	53733306	Distal Intergenic	-0793	716E-05	452E-03	Lyzl1	-187158	Hypo
chr16	73008876	73012975	Intron (NM_001037327/364634, intron 3 of 69)	-0573	723E-05	455E-03	Csmd1	790111	Hypo
chr15	38921416	38926398	Exon (NM_213626/305967, exon 2 of 47)	-0529	730E-05	457E-03	Kif13b	13098	Hypo
chr7	133538497	133543424	Promoter (<=1kb)	-0454	742E-05	462E-03	Sp1	0	Hypo

chr9	10077925	10078486	Distal Intergenic	-1213	754E-05	469E-03	Adgre4	317570	Hypo
chr1	55011963	55013463	Distal Intergenic	-0942	786E-05	487E-03	Vom2r9	153677	Hypo
chr12	28098856	28103378	Promoter (<=1kb)	-0551	791E-05	488E-03	Fzd10	0	Hypo
chr15	3502944	3509962	Promoter (<=1kb)	-0499	791E-05	488E-03	Camk2g	0	Hypo
chr16	26640088	26642229	Intron (NM_001107310/306404, intron 4 of 11)	-0832	828E-05	510E-03	Spock3	173376	Hypo
chr6	69832782	69835180	Distal Intergenic	-0691	832E-05	511E-03	Arhgap5	-153030	Hypo
chr8	45868874	45870263	Intron (NM_001108141/315615, intron 3 of 32)	-0985	834E-05	511E-03	Dscaml1	128576	Hypo
chr7	85206266	85209965	Distal Intergenic	-0712	839E-05	513E-03	Samd12	-142209	Hypo
chr6	9993200	9995855	Intron (NM_001013940/298767, intron 11 of 11)	-0844	842E-05	514E-03	Abcg5	28082	Hypo
chr10	7234714	7236130	Distal Intergenic	-0850	845E-05	515E-03	Tmem114	64968	Hypo
chr1	56997175	56999082	Distal Intergenic	-0820	845E-05	515E-03	Chd1	333121	Hypo
chr16	26648598	26652865	Intron (NM_001107310/306404, intron 2 of 11)	-0597	856E-05	520E-03	Spock3	162740	Hypo
chr19	22960603	22961554	Distal Intergenic	-1061	860E-05	521E-03	Man2b1	112818	Hypo
chr5	5087279	5088325	Distal Intergenic	-1094	865E-05	523E-03	Xkr9	473843	Hypo
chr4	119758871	119760302	Intron (NM_001044249/362393, intron 1 of 17)	-0787	868E-05	523E-03	Antxr1	17930	Hypo
chr2	148882204	148884289	Distal Intergenic	-0953	869E-05	523E-03	Vom2r47	100710	Hypo
chr3	144154716	144157408	Exon (NM_001100748/362247, exon 6 of 19)	-0748	880E-05	529E-03	Trpc4ap	35578	Hypo
chr9	107459796	107460934	Distal Intergenic	-0995	889E-05	533E-03	Ptprm	-116098	Hypo
chr3	28718615	28720405	Distal Intergenic	-0745	894E-05	535E-03	Gtdc1	441214	Hypo
chr17	4326722	4332498	Distal Intergenic	-0506	907E-05	540E-03	Dapk1	-235731	Hypo
chr7	68510808	68512353	Intron (NM_001134527/299979, intron 13 of 15)	-0874	909E-05	540E-03	Grhl2	110331	Hypo
chr12	773904	775550	Distal Intergenic	-0868	911E-05	540E-03	Stard13	-39300	Hypo
chr1	53259507	53260338	Distal Intergenic	-0986	914E-05	540E-03	Smok2a	-20029	Hypo
chr19	22962990	22964492	Distal Intergenic	-0851	918E-05	542E-03	Man2b1	109880	Hypo
chr5	100791371	100792725	Distal Intergenic	-1055	920E-05	542E-03	Rraga	-320616	Hypo
chr7	86865513	86867266	Intron (NM_001130548/314981, intron 34 of 47)	-0777	923E-05	542E-03	Mtbp	-105803	Hypo
chr8	23667408	23675352	Distal Intergenic	-0492	924E-05	542E-03	LOC500959	-18307	Hypo
chr2	153279066	153289122	Promoter (<=1kb)	-0445	939E-05	549E-03	Trim59	0	Hypo
chr1	236660855	236665926	Exon (NM_001377114/685933, exon 5 of 13)	-0536	941E-05	549E-03	Hells	-35822	Hypo
chr19	38632831	38636658	Intron (NM_177930/307842, intron 12 of 18)	-0574	942E-05	549E-03	Vac14	42262	Hypo
chrX	33974839	33977029	Exon (NM_001104643/100125595, exon 3 of 6)	-0862	950E-05	552E-03	Rs1	15086	Hypo
chr3	42729605	42733418	Distal Intergenic	-0564	967E-05	558E-03	Cytip	-3612	Hypo
chr16	72883884	72886154	Intron (NM_001037327/364634, intron 3 of 69)	-0756	969E-05	558E-03	Csmd1	665119	Hypo

chr10	91982384	91983478	Distal Intergenic	-1095	969E-05	558E-03	C10h17orf58	6935	Hypo
chr1	13965095	13966340	Distal Intergenic	-1151	970E-05	558E-03	Olig3	-114919	Hypo
chr3	133719500	133725361	Distal Intergenic	-0507	970E-05	558E-03	Crnkl1	-365171	Hypo
chr14	7849229	7851595	Intron (NM_031242/81925, intron 3 of 12)	-0773	976E-05	561E-03	Cds1	31142	Hypo
chr17	44876722	44880333	Distal Intergenic	-0569	982E-05	562E-03	Elmo1	-53934	Hypo
chr1	184889715	184894032	Distal Intergenic	-0479	984E-05	562E-03	Fgfr2	-39089	Hypo
chr8	30455148	30462023	Distal Intergenic	-0491	989E-05	563E-03		-45278	Hypo
chr9	3844713	3846096	Intron (NM_001134762/501088, intron 3 of 21)	-0848	101E-04	571E-03	Tbc1d5	170817	Hypo
chr2	141865603	141868128	Distal Intergenic	-0665	101E-04	571E-03	Commd2	-12696	Hypo
chr5	17046818	17048852	Distal Intergenic	-0791	101E-04	571E-03	Sdr16c5	-5848	Hypo
chr1	135658951	135660099	Promoter (<=1kb)	-1143	104E-04	581E-03	Homer2	0	Hypo
chr3	161946268	161951155	Distal Intergenic	-0552	104E-04	581E-03	Pck1	16012	Hypo
chr3	76847058	76848809	Exon (NM_001106488/295922, exon 12 of 13)	-0881	104E-04	583E-03	Mtch2	16509	Hypo
chr4	65639314	65640255	Intron (NM_198782/688705, intron 6 of 32)	-1058	104E-04	583E-03	Dgki	232478	Hypo
chr1	209484330	209485241	Distal Intergenic	-1175	106E-04	589E-03	Dtx4	-3257	Hypo
chr5	164532222	164533442	Promoter (1-2kb)	-1203	107E-04	593E-03	Dffb	1111	Hypo
chr3	110995436	110997503	Distal Intergenic	-0959	107E-04	595E-03	Sema6d	-886369	Hypo
chr3	122252656	122254697	Intron (NM_001077641/24654, intron 3 of 32)	-0929	109E-04	601E-03	Plcb1	192688	Hypo
chr17	7434691	7435872	Distal Intergenic	-1225	109E-04	604E-03	Trpc7	-273711	Hypo
chr3	146458172	146459161	Intron (NM_001024870/296320, intron 5 of 15)	-1137	111E-04	614E-03	Ctnnbl1	70232	Hypo
chr18	25780658	25783425	Distal Intergenic	-0565	113E-04	619E-03	Apc	-80797	Hypo
chr5	96237512	96239844	Distal Intergenic	-0764	113E-04	619E-03	Mpdz	-317013	Hypo
chr3	59488392	59489196	Promoter (1-2kb)	-1137	114E-04	620E-03	Lnpk	-1434	Hypo
chr3	56322465	56324637	Intron (NM_001107812/311748, intron 1 of 9)	-0780	114E-04	620E-03	Dlx1	-31553	Hypo
chr9	908660	910476	Promoter (<=1kb)	-0652	114E-04	620E-03	Hdgfl2	0	Hypo
chr3	118692217	118694746	Distal Intergenic	-0680	115E-04	625E-03	Smox	-56877	Hypo
chr10	64459143	64465316	Promoter (<=1kb)	-0486	115E-04	625E-03	Omg	0	Hypo
chr1	187540087	187544993	Intron (NM_001107556/309060, intron 4 of 4)	-0532	116E-04	628E-03	Fam53b	46193	Hypo
chr1	87663003	87667032	Intron (NM_001009641/292808, intron 11 of 14)	-0596	117E-04	630E-03	Cebpg	25740	Hypo
chr7	126134280	126135615	Intron (NM_001271239/500921, intron 6 of 7)	-0913	118E-04	630E-03	Tmem117	376689	Hypo
chr10	87703731	87705948	Distal Intergenic	-0876	118E-04	630E-03	Adam11	-18859	Hypo
chr1	117088483	117093078	Distal Intergenic	-0600	118E-04	630E-03	Chrna7	-251260	Hypo
chr7	11053156	11054376	Distal Intergenic	-1021	118E-04	630E-03	Ilvbl	-3232	Hypo

chr13	89114481	89117799	Intron (NM_031575/29414, intron 1 of 11)	-0665	120E-04	635E-03	Akt3	98037	Hypo
chr8	46375958	46377352	Intron (NM_001271216/684112, intron 1 of 24)	-0974	120E-04	635E-03	Sik3	63658	Hypo
chr19	13743693	13745193	Intron (NM_001014221/363337, intron 1 of 4)	-0777	121E-04	638E-03		5813	Hypo
chr3	28514866	28516564	Intron (NM_001013917/295635, intron 12 of 13)	-0936	124E-04	651E-03	Arhgap15	525233	Hypo
chr15	4699160	4700622	Intron (NM_001009976/364261, intron 2 of 3)	-0977	126E-04	659E-03	Spetex2h	17243	Hypo
chr1	211469927	211471093	Distal Intergenic	-1146	126E-04	659E-03	Tle4	326769	Hypo
chr18	25800961	25802446	Distal Intergenic	-0713	127E-04	662E-03	Apc	-61776	Hypo
chr15	923741	924659	Exon (NM_031828/83731, exon 25 of 31)	-1101	128E-04	668E-03	Kcnma1	621664	Hypo
chr7	19183392	19185869	Distal Intergenic	-0558	130E-04	677E-03	Tcp11l2	-6424	Hypo
chr3	17566585	17568196	Intron (NM_001107834/311876, intron 2 of 8)	-0941	131E-04	677E-03	Pbx3	114154	Hypo
chr6	63149641	63150702	Distal Intergenic	-1153	132E-04	684E-03	Nova1	754841	Hypo
chr17	86124923	86126886	Distal Intergenic	-0892	134E-04	690E-03	Gpr137b	-103395	Hypo
chr6	100081781	100083372	Distal Intergenic	-1027	137E-04	703E-03	Exd2	-33495	Hypo
chr20	41688750	41695465	Distal Intergenic	-0501	141E-04	713E-03	Lama4	-696803	Hypo
chr1	223076967	223080548	Distal Intergenic	-0592	141E-04	713E-03	Dmrt1	-62311	Hypo
chr11	67733759	67736548	Exon (NM_001014230/363796, exon 3 of 11)	-0679	142E-04	718E-03	Iqcg	3512	Hypo
chr8	73607012	73611417	Intron (NM_001037651/315806, intron 11 of 19)	-0563	143E-04	721E-03	Pygo1	-55050	Hypo
chr9	9983289	9986193	Distal Intergenic	-0557	143E-04	721E-03	Adgre4	222934	Hypo
chr17	6768939	6773586	Intron (NM_001271297/306759, intron 2 of 10)	-0521	143E-04	722E-03	Spock1	27426	Hypo
chr19	5681168	5683971	Intron (NM_053393/84408, intron 3 of 11)	-0628	146E-04	734E-03	Cdh8	186994	Hypo
chr2	173674943	173693606	Promoter (<=1kb)	-0380	147E-04	738E-03	Mir9-1	0	Hypo
chr9	58958493	58962655	3' UTR	-0554	149E-04	744E-03	RGD1306941	8648	Hypo
chr18	60734174	60735419	Intron (NM_001191836/24611, intron 5 of 11)	-1064	152E-04	755E-03	Chmp1b	-4930	Hypo
chr2	176070141	176071209	Distal Intergenic	-1100	153E-04	755E-03	S100a3	-15726	Hypo
chr8	36575300	36578789	Exon (NM_031066/81730, exon 6 of 10)	-0599	156E-04	770E-03	Fez1	30765	Hypo
chr11	63366216	63367016	Intron (NM_001271250/288080, intron 1 of 28)	-1074	156E-04	771E-03	Stxbp5l	31549	Hypo
chr14	103156737	103162231	Distal Intergenic	-0472	159E-04	777E-03	Prorsd1	98017	Hypo
chr1	93988346	93989728	Distal Intergenic	-1082	160E-04	782E-03	Zfp819	-48520	Hypo
chr3	118047186	118049094	Intron (NM_001109206/499891, intron 1 of 36)	-0797	161E-04	785E-03	Dnaaf9	3536	Hypo
chr15	14781079	14783532	Intron (NM_021774/60398, intron 3 of 7)	-0715	162E-04	788E-03	Fhit	813197	Hypo
chr18	21367286	21368287	Distal Intergenic	-1044	163E-04	788E-03	Pik3c3	-477075	Hypo
chr1	197078885	197080376	Distal Intergenic	-0962	163E-04	788E-03	Mob2	69526	Hypo
chr6	122450018	122451324	Distal Intergenic	-1085	164E-04	788E-03	Fam181a	-18016	Hypo

chr6	98794348	98797625	Distal Intergenic	-0611	164E-04	788E-03	Zfp36l1	138123	Hypo
chr13	94231405	94233337	Distal Intergenic	-0857	164E-04	788E-03	Capn8	-19717	Hypo
chr4	39067249	39069111	Distal Intergenic	-0699	164E-04	788E-03	Ndufa4	940273	Hypo
chr3	1061069	1062280	Distal Intergenic	-1001	165E-04	788E-03	Rn18s	-1169527	Hypo
chr8	48108639	48110438	Intron (NM_001012201/363058, intron 1 of 11)	-0827	165E-04	788E-03	Cadm1	260803	Hypo
chr4	16730827	16731878	Distal Intergenic	-0975	166E-04	790E-03	Gnai1	-82137	Hypo
chr5	126846956	126848115	Distal Intergenic	-1067	166E-04	790E-03	Skint1	60487	Hypo
chr5	62976530	62982654	Intron (NM_023020/63845, intron 7 of 9)	-0483	167E-04	795E-03	Cavin4	-14321	Hypo
chr4	65515536	65522880	Intron (NM_198782/688705, intron 21 of 32)	-0496	168E-04	798E-03	Ptn	-140230	Hypo
chr5	23814905	23816211	Distal Intergenic	-1002	169E-04	802E-03	Cfap418	-180507	Hypo
chr11	31106056	31108446	Exon (NM_001136096/29491, exon 26 of 39)	-0736	176E-04	829E-03	Atp5po	63084	Hypo
chr6	98327721	98331740	Distal Intergenic	-0517	177E-04	835E-03	Zfyve26	-232241	Hypo
chr8	22852854	22858429	Distal Intergenic	-0494	179E-04	840E-03	Dpy19l1	163032	Hypo
chr3	97073823	97075325	Distal Intergenic	-0982	179E-04	840E-03	Slc5a12	-104001	Hypo
chr18	61762263	61764031	Intron (NM_001271365/679578, intron 2 of 4)	-0934	180E-04	844E-03	Ldlrad4	115552	Hypo
chr15	80514381	80535195	5' UTR	-0379	184E-04	860E-03	Slain1	15734	Hypo
chrX	99867113	99867926	Distal Intergenic	-1437	186E-04	864E-03	Tceal1	-190597	Hypo
chr14	67914080	67916984	Distal Intergenic	-0618	188E-04	872E-03	C1qtnf7	-432507	Hypo
chr17	85826321	85827566	Distal Intergenic	-0858	188E-04	872E-03	Ero1b	-130121	Hypo
chr9	23358694	23360548	Downstream (<=300bp)	-0827	189E-04	875E-03	Efhc1	-15292	Hypo
chr4	11322192	11324639	Distal Intergenic	-0698	190E-04	877E-03	Tomm7	17070	Hypo
chr1	175538452	175540487	Distal Intergenic	-0657	193E-04	888E-03	Cdr2	-10706	Hypo
chr1	195852177	195853444	3' UTR	-0695	194E-04	890E-03	Syce1	9730	Hypo
chr14	26495881	26497036	Intron (NM_130822/170641, intron 10 of 24)	-0833	195E-04	894E-03	Adgrl3	327408	Hypo
chr1	13383530	13384573	Distal Intergenic	-0922	202E-04	925E-03	Perp	-157494	Hypo
chr6	48554015	48556998	Promoter (<=1kb)	-0470	203E-04	925E-03	Hbp1	0	Hypo
chr1	236036975	236040551	Promoter (2-3kb)	-0601	203E-04	925E-03	Lgi1	-2825	Hypo
chr14	78021986	78023202	Exon (NM_001191682/305467, exon 8 of 33)	-0956	203E-04	926E-03	Sfi1	25053	Hypo
chr16	77132374	77134179	Distal Intergenic	-0793	204E-04	926E-03	Tubgcp3	280515	Hypo
chr12	32075672	32076883	Intron (NM_001191665/304469, intron 1 of 6)	-1098	204E-04	926E-03	Rilpl1	3802	Hypo
chr1	55059336	55060317	Distal Intergenic	-0878	204E-04	926E-03	Dact2	129622	Hypo
chr10	45094130	45098056	Intron (NM_001108277/360537, intron 1 of 11)	-0602	204E-04	926E-03	Tom1l2	59976	Hypo
chr3	127861847	127862833	Distal Intergenic	-0980	205E-04	927E-03	Flrt3	144940	Hypo

chr16	18932850	18935072	Promoter (<=1kb)	-0848	206E-04	931E-03	Crlf1	925	Hypo
chr9	10053576	10054930	Distal Intergenic	-0779	206E-04	931E-03	Adgre4	293221	Hypo
chr3	65213098	65214271	Distal Intergenic	-0878	207E-04	934E-03	Dnajc10	-18426	Hypo
chr6	69935537	69938289	Distal Intergenic	-0574	208E-04	934E-03	Arhgap5	-49921	Hypo
chr2	120140852	120142560	Distal Intergenic	-0678	208E-04	934E-03	Il21	-13911	Hypo
chr4	11687724	11689710	Exon (NM_001100851/311968, exon 6 of 27)	-0720	208E-04	934E-03	Kmt2e	37663	Hypo
chr2	62269910	62271883	Distal Intergenic	-0672	210E-04	941E-03	Cdh6	-44682	Hypo
chr3	17551851	17553323	Intron (NM_001107834/311876, intron 2 of 8)	-0889	211E-04	943E-03	Pbx3	129027	Hypo
chr5	62982797	62986378	Exon (NM_023020/63845, exon 8 of 10)	-0632	212E-04	945E-03	Cavin4	-10597	Hypo
chr8	9339705	9339898	Distal Intergenic	-1139	213E-04	950E-03	Jrkl	887170	Hypo
chr9	48558612	48561381	Distal Intergenic	-0519	214E-04	952E-03	Mstn	-99803	Hypo
chr9	10121312	10121793	Distal Intergenic	-1294	215E-04	954E-03	Adgre4	360957	Hypo
chr5	160905757	160911714	Intron (NM_053885/116665, intron 2 of 22)	-0443	215E-04	954E-03	Mir6332	68453	Hypo
chr15	34726728	34728221	Distal Intergenic	-0735	217E-04	959E-03	Spata13	-50315	Hypo
chr18	39746934	39747899	Intron (NM_001004276/361323, intron 3 of 6)	-1043	217E-04	959E-03	Commd10	5633	Hypo
chr5	77627989	77629835	Distal Intergenic	-0824	217E-04	959E-03	Tnc	-191951	Hypo
chr20	12384223	12391757	Promoter (2-3kb)	-0445	219E-04	968E-03	S100b	-2604	Hypo
chr4	525864	526929	Distal Intergenic	-1126	220E-04	969E-03	Il6	4692249	Hypo
chr13	21203653	21204529	Distal Intergenic	-1109	222E-04	975E-03	Rnf152	198571	Hypo
chr7	23674749	23676077	Distal Intergenic	-0754	223E-04	979E-03	Ano4	-23017	Hypo
chr18	50896838	50900992	Distal Intergenic	-0533	224E-04	980E-03	Ctxn3	-30461	Hypo
chr3	90032741	90035439	Intron (NM_134403/171440, intron 1 of 16)	-0656	224E-04	980E-03	Abtb2	78015	Hypo
chr15	44807946	44809025	Promoter (1-2kb)	-1001	227E-04	988E-03	Chmp7	-1730	Hypo
chr3	17592764	17596045	Intron (NM_001107834/311876, intron 2 of 8)	-0636	227E-04	988E-03	Pbx3	86305	Hypo
chr15	4454203	4458037	Exon (NM_001047874/302247, exon 8 of 10)	-0555	227E-04	988E-03	Rtraf	6847	Hypo
chr1	55073218	55074021	Distal Intergenic	-1181	228E-04	988E-03	Dact2	115918	Hypo
chr10	70213181	70214718	Promoter (<=1kb)	-0751	231E-04	100E-02	Bcas3	0	Hypo
chr14	87039259	87041200	Distal Intergenic	-0707	231E-04	100E-02	Cobl	-59720	Hypo
chr11	57821844	57825395	Distal Intergenic	-0454	234E-04	101E-02	Zbtb20	-30630	Hypo
chr19	24195581	24196421	Promoter (<=1kb)	-1060	235E-04	101E-02	Asf1b	0	Hypo
chr7	111247427	111249002	Promoter (<=1kb)	-0735	235E-04	101E-02	Gtpbp1	0	Hypo
chr6	119567167	119568700	Distal Intergenic	-0656	237E-04	102E-02	Calm1	79476	Hypo
chr6	26266009	26270811	3' UTR	-0514	237E-04	102E-02	Garem2	-24247	Hypo

chr16	68774017	68778574	Exon (NM_001007731/361171, exon 3 of 5)	-0539	237E-04	102E-02	Golga7	6642	Hypo
chr3	691109	691638	Distal Intergenic	-1079	237E-04	102E-02	Rn18s	-1540169	Hypo
chr9	75337110	75338709	Distal Intergenic	-0915	239E-04	102E-02	Pincl	-337350	Hypo
chr1	53260519	53261383	Distal Intergenic	-0927	239E-04	102E-02	Smok2a	-18984	Hypo
chr19	11473277	11474263	Exon (NM_001012056/307660, exon 3 of 13)	-1072	240E-04	103E-02	Ces5a	3808	Hypo
chr12	684951	687030	Intron (NM_001109060/498130, intron 1 of 13)	-0642	243E-04	103E-02	Stard13	47574	Hypo
chr6	133338526	133340807	Distal Intergenic	-0853	246E-04	104E-02	Adam6	671552	Hypo
chr3	143830236	143832841	Exon (NM_181637/353304, exon 5 of 12)	-0636	246E-04	104E-02	Pigu	24389	Hypo
chr20	9099488	9102208	Distal Intergenic	-0604	248E-04	105E-02	Abcg1	-24479	Hypo
chr10	78958119	78959153	Intron (NM_001108290/360600, intron 1 of 29)	-1034	249E-04	105E-02	Spag9	14640	Hypo
chrX	42649984	42651837	Distal Intergenic	-0856	249E-04	105E-02	Prrg1	-43438	Hypo
chr4	54128945	54133390	Distal Intergenic	-0494	250E-04	105E-02	Gpr37	27537	Hypo
chr4	110743224	110744365	Distal Intergenic	-1062	250E-04	105E-02	Reg3b	-117410	Hypo
chr10	104922122	104923414	Intron (NM_001134499/287871, intron 1 of 33)	-0980	253E-04	106E-02	Rptor	43635	Hypo
chr8	22678588	22680357	Intron (NM_001106808/300458, intron 3 of 9)	-0613	258E-04	108E-02	Npsr1	71642	Hypo
chr5	144525433	144527252	Promoter (1-2kb)	-0741	261E-04	109E-02	Trnau1ap	-1447	Hypo
chr17	4997702	4999730	Distal Intergenic	-0784	261E-04	109E-02	Spata31d1	54948	Hypo
chr3	82044	82922	Distal Intergenic	-0950	268E-04	111E-02	Rn18s	-2148885	Hypo
chr12	16913775	16917924	Distal Intergenic	-0593	271E-04	112E-02	Azgp1	-13119	Hypo
chr6	118963027	118964428	Distal Intergenic	-0933	271E-04	112E-02	Foxn3	-120626	Hypo
chr2	56374245	56376123	Exon (NM_001108938/365691, exon 6 of 22)	-0867	273E-04	113E-02	Egflam	100175	Hypo
chr6	67181300	67182246	Distal Intergenic	-0986	274E-04	113E-02	Foxg1	506503	Hypo
chr3	114877417	114879662	Intron (NM_012798/25263, intron 1 of 3)	-0633	275E-04	113E-02	Mal	8474	Hypo
chr9	11360279	11365391	Distal Intergenic	-0520	276E-04	114E-02	Mocs1	202399	Hypo
chr13	64245068	64245862	Distal Intergenic	-1131	277E-04	114E-02	RGD1309104	-85975	Hypo
chr3	88518164	88519136	Distal Intergenic	-0928	285E-04	116E-02	Trim44	209501	Hypo
chr8	106808590	106809890	Distal Intergenic	-1038	285E-04	116E-02	Wdr82	-4679	Hypo
chr10	98838901	98841340	Exon (NM_001108303/360652, exon 40 of 42)	-0608	287E-04	117E-02	Cdc42ep4	-41693	Hypo
chr1	52376396	52379218	Promoter (2-3kb)	-0507	290E-04	118E-02	T2	-2871	Hypo
chr2	86637455	86638973	Distal Intergenic	-0897	293E-04	118E-02		36927	Hypo
chr1	50199793	50203859	Intron (NM_001077677/499021, intron 4 of 4)	-0461	294E-04	118E-02	Cahm	183100	Hypo
chr1	53541481	53541926	Intron (NM_001191619/292316, intron 1 of 1)	-1365	296E-04	119E-02	Smok2a	261114	Hypo
chr9	2904217	2905592	Distal Intergenic	-1093	297E-04	120E-02	Plcl2	-387776	Hypo

chr1	184957671	184960231	Distal Intergenic	-0662	300E-04	120E-02	Fgfr2	-107045	Hypo
chr9	15256877	15258558	Distal Intergenic	-0757	307E-04	122E-02	Mrpl14	49861	Hypo
chr1	90949371	90950283	Distal Intergenic	-1015	308E-04	122E-02	Pop4	18077	Hypo
chr10	36927482	36928425	Intron (NM_001107000/303130, intron 2 of 14)	-1012	317E-04	125E-02	Fstl4	16062	Hypo
chr7	68259870	68261351	Distal Intergenic	-0908	318E-04	125E-02	Zfp706	-85828	Hypo
chr16	20288544	20289446	Distal Intergenic	-0896	325E-04	128E-02	Lzts1	-253363	Hypo
chr4	117856939	117858133	Intron (NM_153298/261737, intron 1 of 13)	-0908	328E-04	129E-02	Sfxn5	11649	Hypo
chr20	21623242	21625165	Distal Intergenic	-0800	330E-04	129E-02	Reep3	129374	Hypo
chr3	149843557	149844582	Distal Intergenic	-0866	331E-04	130E-02	Chd6	-85802	Hypo
chr3	112181365	112182272	Distal Intergenic	-0923	332E-04	130E-02	Slc24a5	-137687	Hypo
chr1	55014924	55015679	Distal Intergenic	-1123	333E-04	130E-02	Vom2r9	156638	Hypo
chr1	52372310	52375840	Promoter (<=1kb)	-0516	334E-04	130E-02	T2	0	Hypo
chr4	65362370	65366747	Intron (NM_017066/24924, intron 1 of 4)	-0489	334E-04	130E-02	Ptn	8559	Hypo
chr3	17523660	17526598	Exon (NM_001107834/311876, exon 4 of 9)	-0702	335E-04	130E-02	Pbx3	155752	Hypo
chr15	8103200	8104700	Distal Intergenic	-0684	335E-04	130E-02	Thrb	-72512	Hypo
chr6	19934406	19944162	5' UTR	-0410	340E-04	131E-02	Rasgrp3	14326	Hypo
chr3	1163684	1165063	Distal Intergenic	-0893	342E-04	132E-02	Rn18s	-1066744	Hypo
chr8	99968873	99971139	Promoter (<=1kb)	-0726	343E-04	132E-02	Mras	724	Hypo
chr3	112182424	112183313	Distal Intergenic	-0840	348E-04	133E-02	Slc24a5	-136646	Hypo
chr18	16026537	16028575	Intron (NM_001271332/100360334, intron 1 of 26)	-0726	349E-04	134E-02	Fhod3	33213	Hypo
chr6	85049141	85050058	Intron (NM_199269/314180, intron 5 of 15)	-1042	350E-04	134E-02	Mdga2	558173	Hypo
chr13	63148437	63149511	Distal Intergenic	-1025	350E-04	134E-02	Hmcn1	-63913	Hypo
chr1	234525843	234527987	Distal Intergenic	-0633	353E-04	135E-02	Tnks2	-40203	Hypo
chr2	230357239	230362203	Intron (NM_199407/362049, intron 1 of 15)	-0543	354E-04	135E-02	Unc5c	176175	Hypo
chr3	833105	833990	Distal Intergenic	-1270	355E-04	135E-02	Rn18s	-1397817	Hypo
chr13	106285560	106286733	Intron (NM_001105988/289392, intron 4 of 31)	-0866	357E-04	136E-02	Plxna2	122457	Hypo
chr2	5526188	5528413	Distal Intergenic	-0703	358E-04	136E-02	Rfesd	-10422	Hypo
chr15	80500024	80509529	Promoter (1-2kb)	-0423	364E-04	138E-02	Slain1	1377	Hypo
chr1	164004750	164006414	Distal Intergenic	-0785	365E-04	138E-02	Dennd5a	-10592	Hypo
chr10	78782530	78785352	Distal Intergenic	-0628	371E-04	139E-02	Utp18	53502	Hypo
chr4	130692503	130693543	Distal Intergenic	-0879	372E-04	140E-02	Mitf	283286	Hypo
chr15	4702454	4706430	Intron (NM_001009976/364261, intron 2 of 3)	-0654	374E-04	140E-02	Spetex2h	11435	Hypo
chr3	1577340	1577885	Distal Intergenic	-1232	379E-04	142E-02	Rn18s	-653922	Hypo

chr16	73165778	73168055	Intron (NM_001037327/364634, intron 5 of 69)	-0706	379E-04	142E-02	Csmd1	947013	Hypo
chr8	23632414	23636267	Distal Intergenic	-0558	381E-04	142E-02	LOC500959	12834	Hypo
chr5	21999503	22004492	Distal Intergenic	-0422	385E-04	143E-02	Lnc056	158625	Hypo
chr17	85376881	85378392	Distal Intergenic	-0978	390E-04	144E-02	Yme1l1	88865	Hypo
chr11	31462603	31466040	Distal Intergenic	-0568	393E-04	145E-02	Kcne2	-56962	Hypo
chr20	13288809	13290557	Distal Intergenic	-0874	394E-04	145E-02	Adora2a	42829	Hypo
chr4	581497	583261	Distal Intergenic	-0879	395E-04	146E-02	Il6	4635917	Hypo
chr1	173239064	173243245	Distal Intergenic	-0524	400E-04	147E-02	Knop1	-39719	Hypo
chr19	52591032	52592638	Distal Intergenic	-0909	401E-04	147E-02	C19h1orf198	36798	Hypo
chr4	77702464	77704548	Distal Intergenic	-0690	402E-04	147E-02	Gimap5	8131	Hypo
chr19	17805446	17809520	Distal Intergenic	-0535	405E-04	148E-02	Sall1	-197983	Hypo
chr15	41196332	41198499	Promoter (<=1kb)	-0565	405E-04	148E-02	Bnip3l	0	Hypo
chr6	133437324	133439102	Distal Intergenic	-1023	412E-04	149E-02	Adam6	770350	Hypo
chr14	83374832	83382102	Distal Intergenic	-0427	413E-04	149E-02	Tns3	-30694	Hypo
chr9	27121302	27130321	Exon (NM_139189/246046, exon 4 of 16)	-0424	413E-04	149E-02	Lmbrd1	24915	Hypo
chr1	176271866	176278756	Distal Intergenic	-0451	416E-04	150E-02	Usp31	-19493	Hypo
chr5	142057805	142059025	Distal Intergenic	-0928	416E-04	150E-02	Tmem39b	-11256	Hypo
chrX	17254363	17254576	Distal Intergenic	-1338	418E-04	150E-02	Mycs	216100	Hypo
chr2	150164768	150166586	Distal Intergenic	-0661	421E-04	151E-02	Ccnl1	30822	Hypo
chr3	113058708	113066403	Distal Intergenic	-0442	422E-04	152E-02	Secisbp2l	-22824	Hypo
chr13	42931265	42933547	Intron (NM_001134958/360840, intron 2 of 21)	-0760	423E-04	152E-02	Srgap2	33511	Hypo
chr7	25496214	25497581	3' UTR	-0792	423E-04	152E-02	Apaf1	81959	Hypo
chr14	103391924	103396144	Distal Intergenic	-0498	424E-04	152E-02	Rtn4	-54046	Hypo
chr3	150754763	150757949	Intron (NM_001108603/362263, intron 6 of 27)	-0588	425E-04	152E-02	Ptprt	230714	Hypo
chr3	49020828	49021975	Distal Intergenic	-0798	427E-04	153E-02	Fign	-260123	Hypo
chr8	120171137	120173649	Intron (NM_182844/360034, intron 11 of 15)	-0588	428E-04	153E-02	Eif1b	-29912	Hypo
chr3	118029079	118043950	Exon (NM_001109206/499891, exon 2 of 37)	-0393	428E-04	153E-02	Dnaaf9	8680	Hypo
chr4	80818311	80819898	Distal Intergenic	-0840	429E-04	153E-02	Snx10	205615	Hypo
chr5	160945261	160948380	Intron (NM_053885/116665, intron 4 of 22)	-0598	431E-04	153E-02	Mir6332	107957	Hypo
chr12	33868742	33869743	Distal Intergenic	-1013	431E-04	153E-02	P2rx4	-6477	Hypo
chr20	52859242	52860456	Distal Intergenic	-0822	432E-04	153E-02	Grik2	-26181	Hypo
chr4	53488226	53489565	Distal Intergenic	-0812	433E-04	154E-02	Tmem229a	-42899	Hypo
chr12	46282471	46284435	Distal Intergenic	-0693	435E-04	154E-02	Lrcol1	53518	Hypo

chr5	151266784	151268215	Exon (NM_001034949/500573, exon 11 of 15)	-0826	438E-04	155E-02	Htr6	43697	Hypo
chr4	121387861	121392135	Distal Intergenic	-0555	439E-04	155E-02	Tpra1	23623	Hypo
chr15	42235610	42236963	Distal Intergenic	-0832	440E-04	155E-02	Nefl	-64957	Hypo
chr4	148791444	148792760	Exon (NM_001191573/680858, exon 10 of 10)	-0758	442E-04	155E-02	Tmem40	12108	Hypo
chr4	129419130	129421469	Distal Intergenic	-0637	442E-04	155E-02	Tafa4	249780	Hypo
chr5	116193387	116201218	Intron (NM_001107949/313409, intron 1 of 17)	-0421	443E-04	155E-02	Dnajc6	62711	Hypo
chr3	123701886	123704950	Promoter (<=1kb)	-0522	444E-04	156E-02	Pak5	0	Hypo
chr2	32363651	32364930	Distal Intergenic	-0751	445E-04	156E-02	Slc30a5	-387807	Hypo
chr2	86374735	86379427	Distal Intergenic	-0507	450E-04	157E-02		-221101	Hypo
chr19	52621431	52631235	Promoter (<=1kb)	-0415	451E-04	157E-02	C19h1orf198	0	Hypo
chr16	20085680	20086391	Distal Intergenic	-0903	454E-04	157E-02	Zfp868	322951	Hypo
chr20	18023151	18031169	Promoter (<=1kb)	-0390	457E-04	158E-02	Phyhipl	-636	Hypo
chr5	113872113	113878528	Intron (NM_001107948/313391, intron 1 of 10)	-0421	458E-04	158E-02	Atg4c	4895	Hypo
chr20	12760114	12763221	Exon (NM_001025728/361825, exon 8 of 9)	-0572	458E-04	158E-02	Derl3	3806	Hypo
chr12	708709	711310	Intron (NM_001109060/498130, intron 1 of 13)	-0542	459E-04	159E-02	Stard13	23294	Hypo
chr3	143797575	143799912	Intron (NM_181637/353304, intron 10 of 11)	-0776	461E-04	159E-02	Map1lc3a	14551	Hypo
chr3	17528449	17530297	Intron (NM_001107834/311876, intron 3 of 8)	-0865	466E-04	160E-02	Pbx3	152053	Hypo
chr19	17904884	17908041	Distal Intergenic	-0533	468E-04	161E-02	Sall1	-99462	Hypo
chr4	175895674	175897935	Intron (NM_012813/25280, intron 1 of 4)	-0720	469E-04	161E-02	St8sia1	22642	Hypo
chr13	98872720	98874594	Distal Intergenic	-0692	470E-04	162E-02	Gpatch2	87703	Hypo
chr3	15240037	15243090	Exon (NM_001108578/362107, exon 3 of 6)	-0546	473E-04	162E-02	Aif1l	10561	Hypo
chr5	102847404	102849111	Distal Intergenic	-0910	476E-04	163E-02	Hacd4	66199	Hypo
chrX	59065934	59066669	Distal Intergenic	-1254	479E-04	164E-02	Klhl15	70268	Hypo
chr13	30680991	30683436	Distal Intergenic	-0634	483E-04	165E-02	Tmem185b	33458	Hypo
chr3	14727470	14728258	Distal Intergenic	-1040	484E-04	165E-02	Ass1	-25810	Hypo
chr11	30440359	30445138	Distal Intergenic	-0460	486E-04	165E-02	Olig2	-30372	Hypo
chr6	133391835	133392364	Distal Intergenic	-1190	486E-04	165E-02	Adam6	724861	Hypo
chr3	1511775	1513172	Distal Intergenic	-0936	491E-04	167E-02	Rn18s	-718635	Hypo
chr8	67875800	67877291	Distal Intergenic	-0677	492E-04	167E-02	Mir190a-2	-24729	Hypo
chr6	100029061	100030193	Distal Intergenic	-1037	492E-04	167E-02	Exd2	-86674	Hypo
chr1	53256441	53257225	Distal Intergenic	-0733	496E-04	168E-02	Smok2a	-23142	Hypo
chr9	103209455	103211022	Distal Intergenic	-0633	497E-04	168E-02	Fbxl17	-3759	Hypo
chr8	30123355	30124490	Distal Intergenic	-0724	499E-04	168E-02	Tmem45b	-212902	Hypo

chr9	6619506	6620888	Distal Intergenic	-0815	501E-04	169E-02	Pp2d1	-85867	Hypo
chr13	82898201	82904524	Intron (NM_001107195/304960, intron 6 of 7)	-0470	505E-04	170E-02	Olfml2b	28768	Hypo
chr2	78170688	78175325	Intron (NM_053714/114506, intron 1 of 11)	-0524	506E-04	170E-02	Ankh	17662	Hypo
chr10	57452206	57453201	Distal Intergenic	-1192	508E-04	171E-02	Cyb5d2	-39209	Hypo
chr8	110466573	110467655	Promoter (<=1kb)	-0840	509E-04	171E-02	Kif9	618	Hypo
chr3	981187	982133	Distal Intergenic	-0888	516E-04	172E-02	Rn18s	-1249674	Hypo
chr10	38866496	38869228	Intron (NM_001126089/691031, intron 2 of 5)	-0658	516E-04	172E-02	Cdc42se2	35828	Hypo
chr1	155003417	155013546	Promoter (<=1kb)	-0363	517E-04	172E-02	Plekhb1	0	Hypo
chr1	230153705	230156014	Intron (NM_181386/353229, intron 3 of 9)	-0821	519E-04	173E-02	Sgms1	103578	Hypo
chr7	107786377	107795248	Promoter (<=1kb)	-0376	519E-04	173E-02	Puf60	0	Hypo
chr10	37679498	37680559	Distal Intergenic	-1002	520E-04	173E-02	Septin8	-4080	Hypo
chr3	68908478	68910949	Distal Intergenic	-0547	521E-04	173E-02	Zswim2	104462	Hypo
chr12	43177554	43179715	Distal Intergenic	-0606	522E-04	173E-02	Wscd2	-88963	Hypo
chr6	20961596	20962861	Promoter (<=1kb)	-0638	529E-04	175E-02	Yipf4	0	Hypo
chr15	27787129	27788067	Distal Intergenic	-0899	531E-04	175E-02	Oxa1l	-25621	Hypo
chr4	119555516	119559046	Distal Intergenic	-0445	535E-04	176E-02	Gfpt1	58759	Hypo
chr20	9178573	9182543	3' UTR	-0553	535E-04	177E-02	Tff3	15426	Hypo
chr5	61545022	61547417	Intron (NM_001100535/298069, intron 5 of 39)	-0686	537E-04	177E-02	Col15a1	43059	Hypo
chr19	47168724	47172178	Intron (NM_138889/192248, intron 7 of 13)	-0508	544E-04	179E-02	Hsbp1	-228861	Hypo
chr19	48638833	48639931	Promoter (<=1kb)	-0864	548E-04	179E-02	Gins2	0	Hypo
chr9	10066441	10067377	Distal Intergenic	-0726	552E-04	181E-02	Adgre4	306086	Hypo
chr3	118049309	118054960	Promoter (<=1kb)	-0399	554E-04	181E-02	Dnaaf9	0	Hypo
chr1	154153302	154154219	Distal Intergenic	-1184	556E-04	181E-02	Neu3	-4489	Hypo
chr16	24613125	24614224	Distal Intergenic	-1054	556E-04	181E-02	Tmem192	146770	Hypo
chr15	52766640	52767885	Distal Intergenic	-1012	556E-04	181E-02	Dnajc15	481790	Hypo
chr4	83667443	83668490	Intron (NM_001025063/502776, intron 2 of 7)	-0720	559E-04	182E-02	Scrn1	25382	Hypo
chr2	235794528	235805428	Distal Intergenic	-0367	565E-04	184E-02	Prkacb	-68476	Hypo
chr11	16131301	16133672	Distal Intergenic	-0625	570E-04	185E-02	Mir99a	-66771	Hypo
chr9	62580253	62581179	Distal Intergenic	-1031	573E-04	186E-02	Icos	196421	Hypo
chr4	169733347	169737248	Distal Intergenic	-0518	573E-04	186E-02	Art4	13417	Hypo
chr9	3513812	3515202	3' UTR	-0889	575E-04	186E-02	Plcl2	220444	Hypo
chr6	62639162	62640849	Distal Intergenic	-0804	580E-04	187E-02	Stxbp6	-481775	Hypo
chr17	1190181	1195595	Distal Intergenic	-0400	581E-04	187E-02	Hsd17b3	162952	Hypo

chr8	70945990	70951262	Intron (NM_173101/25484, intron 1 of 27)	-0468	581E-04	187E-02	Myo1e	58056	Hypo
chr1	88366227	88367688	Exon (NM_001135835/308519, exon 16 of 19)	-0973	586E-04	188E-02	Dpy19l3	49651	Hypo
chr4	45495405	45496569	Distal Intergenic	-1052	589E-04	189E-02	Cav2	-120197	Hypo
chr20	27433885	27438900	Exon (NM_001106398/294560, exon 3 of 8)	-0471	593E-04	190E-02	Oit3	-23193	Hypo
chr1	171698991	171700162	Intron (NM_022295/64133, intron 1 of 11)	-0801	594E-04	190E-02	Xylt1	55066	Hypo
chr19	8640628	8643028	Distal Intergenic	-0537	595E-04	190E-02	Got2	-531276	Hypo
chr2	61583606	61586268	Intron (NM_022940/65034, intron 1 of 22)	-0545	595E-04	190E-02	Pdzd2	34878	Hypo
chr3	9237825	9239617	Intron (NM_001276417/100360302, intron 22 of 29)	-0640	599E-04	191E-02	Inpp5e	-8375	Hypo
chr8	23010427	23013537	Intron (NM_001191791/315496, intron 1 of 21)	-0616	600E-04	191E-02	Dpy19l1	7924	Hypo
chr16	51334327	51335500	Exon (NM_178093/306487, exon 4 of 10)	-0798	604E-04	192E-02	Mtus1	39090	Hypo
chr3	1201902	1202932	Distal Intergenic	-0962	604E-04	192E-02	Rn18s	-1028875	Hypo
chr1	207551272	207557599	3' UTR	-0485	607E-04	192E-02	Prpf19	9653	Hypo
chr4	10101114	10103609	Distal Intergenic	-0636	607E-04	192E-02	Prkag2	-102079	Hypo
chr14	18901802	18904213	Exon (NM_053424/84484, exon 15 of 26)	-0681	608E-04	192E-02		-62044	Hypo
chr9	6704882	6706972	Promoter (1-2kb)	-0886	609E-04	193E-02	LOC301165	-1081	Hypo
chr1	53357184	53358189	Intron (NM_001191619/292316, intron 1 of 1)	-1009	612E-04	193E-02	Smok2a	76817	Hypo
chr10	106518536	106520500	Promoter (<=1kb)	-0517	617E-04	194E-02	Narf	0	Hypo
chr3	60980808	60981841	Exon (NM_001127481/140928, exon 13 of 20)	-0904	619E-04	195E-02	Ift70a1	-75458	Hypo
chr8	67907729	67913186	Distal Intergenic	-0513	621E-04	195E-02	Mir190a-2	-56658	Hypo
chr17	81687066	81690170	Distal Intergenic	-0545	621E-04	195E-02	Pip4k2a	-19037	Hypo
chr16	20232354	20232854	Distal Intergenic	-0936	624E-04	196E-02	Lzts1	-309955	Hypo
chr2	163904407	163907052	Distal Intergenic	-0647	625E-04	196E-02	Rapgef2	415105	Hypo
chr1	90314006	90318668	Distal Intergenic	-0537	636E-04	199E-02	Uri1	385988	Hypo
chr6	124594653	124602955	Promoter (2-3kb)	-0400	638E-04	199E-02	Gskip	2115	Hypo
chr2	248986529	248988302	Intron (NM_199408/362065, intron 2 of 12)	-0609	638E-04	199E-02	Wls	54613	Hypo
chr17	54301186	54303767	3' UTR	-0610	642E-04	200E-02	Crem	10868	Hypo
chr3	41833113	41836903	5' UTR	-0556	643E-04	200E-02	Gpd2	30339	Hypo
chr13	44936833	44938855	Distal Intergenic	-0622	648E-04	201E-02	Sox13	-61311	Hypo
chr6	10940237	10942012	Distal Intergenic	-0821	650E-04	202E-02	Haao	94452	Hypo
chr2	93043199	93044696	Distal Intergenic	-0746	651E-04	202E-02	Hey1	-52010	Hypo
chr5	111039868	111041643	Distal Intergenic	-0717	653E-04	203E-02	Cyp2j10	-44753	Hypo
chr3	17678295	17679844	Promoter (2-3kb)	-0860	653E-04	203E-02	Pbx3	2506	Hypo
chr1	3144552	3153155	Distal Intergenic	-0443	656E-04	203E-02	Ust	-182508	Hypo

chr1	127080247	127081793	Distal Intergenic	-0789	661E-04	204E-02	Rgma	-47141	Hypo
chr18	48009344	48014610	Distal Intergenic	-0445	662E-04	204E-02	Zfp608	395855	Hypo
chr2	21048456	21049673	Intron (NM_001006999/309995, intron 6 of 7)	-0747	663E-04	204E-02	Xrcc4	147912	Hypo
chr5	35668480	35670558	Distal Intergenic	-0709	668E-04	205E-02	Faxc	188078	Hypo
chr3	49621489	49626788	Intron (NM_031623/58844, intron 2 of 13)	-0472	669E-04	205E-02	Grb14	57017	Hypo
chr19	27223096	27226711	Intron (NM_001108444/361388, intron 6 of 9)	-0584	672E-04	206E-02	Smarca5	-45207	Hypo
chr1	242395466	242397676	Distal Intergenic	-0581	676E-04	207E-02	Got1	-14843	Hypo
chr1	168993873	169002663	Promoter (<=1kb)	-0374	677E-04	207E-02	Insc	841	Hypo
chr3	53140426	53142160	Intron (NM_019362/54348, intron 1 of 17)	-0695	677E-04	207E-02	Stk39	36900	Hypo
chr15	19642932	19644309	Distal Intergenic	-0830	683E-04	209E-02	Bmp4	-20651	Hypo
chr11	71230622	71232120	Intron (NM_001191658/288026, intron 1 of 29)	-0835	684E-04	209E-02	Atp13a4	4161	Hypo
chr7	8542807	8547939	Promoter (<=1kb)	-0465	697E-04	212E-02	Pias4	0	Hypo
chr4	36489956	36492841	Intron (NM_001177442/296884, intron 4 of 10)	-0532	698E-04	212E-02	Glcci1	182000	Hypo
chr5	159675912	159680096	Exon (NM_057200/117548, exon 18 of 49)	-0527	698E-04	212E-02	Kif1b	62682	Hypo
chr18	13148733	13151619	Distal Intergenic	-0578	703E-04	213E-02	Klhl14	-230905	Hypo
chr3	96469580	96473640	Intron (NM_173328/286994, intron 1 of 17)	-0530	704E-04	213E-02	Lgr4	21720	Hypo
chr18	27267499	27272260	3' UTR	-0430	705E-04	213E-02	Spata24	-10440	Hypo
chr2	202991681	202993537	Distal Intergenic	-0807	705E-04	213E-02	Olfm3	81088	Hypo
chr3	134371066	134373102	Distal Intergenic	-0597	706E-04	213E-02	Xrn2	-64043	Hypo
chr9	10117634	10118422	Distal Intergenic	-1006	707E-04	213E-02	Adgre4	357279	Hypo
chr10	859744	861790	Intron (NM_053347/83836, intron 3 of 7)	-0691	707E-04	213E-02	Nde1	14423	Hypo
chr1	54120277	54120633	Distal Intergenic	-1056	710E-04	214E-02	Vom2r7	-171851	Hypo
chr11	30546616	30549749	Distal Intergenic	-0525	722E-04	216E-02	Olig1	32237	Hypo
chr15	80488540	80496607	Promoter (2-3kb)	-0408	725E-04	217E-02	Slain1	-2040	Hypo
chr7	99991464	99994243	Intron (NM_001134957/362925, intron 3 of 15)	-0623	726E-04	217E-02	Zfat	60031	Hypo
chr14	70817785	70821565	Distal Intergenic	-0497	727E-04	217E-02	Hs3st1	-393454	Hypo
chr15	35766542	35772213	Promoter (2-3kb)	-0442	730E-04	217E-02	Kcnrg	-2279	Hypo
chr7	58498596	58500860	Intron (NM_001271079/314897, intron 4 of 9)	-0491	730E-04	217E-02	Mirlet7i	141709	Hypo
chr8	38175415	38176710	Distal Intergenic	-0949	732E-04	217E-02	Or8b101c	107598	Hypo
chr18	48075332	48079033	Distal Intergenic	-0525	734E-04	218E-02	Zfp608	331432	Hypo
chr16	20001836	20002424	Distal Intergenic	-1088	735E-04	218E-02	Zfp868	239107	Hypo
chr6	44235880	44237123	Distal Intergenic	-0897	736E-04	218E-02	Sox11	-225526	Hypo
chr9	9220929	9222119	Intron (NM_001100888/301155, intron 3 of 6)	-0965	738E-04	218E-02	St6gal2	38530	Hypo

chr1	151557349	151561455	Intron (NM_053417/84477, intron 2 of 9)	-0441	742E-04	219E-02	Kctd21	-103187	Hypo
chr18	6544949	6545816	Distal Intergenic	-1032	744E-04	220E-02	Aqp4	-20591	Hypo
chr10	88105942	88106421	Distal Intergenic	-0997	745E-04	220E-02	Fmnl1	-9588	Hypo
chr9	10047843	10049232	Distal Intergenic	-0840	747E-04	220E-02	Adgre4	287488	Hypo
chr13	91399308	91404132	Promoter (<=1kb)	-0437	748E-04	220E-02	Sccpdh	0	Hypo
chr1	78779154	78781028	Intron (NM_001025642/292686, intron 1 of 1)	-0607	748E-04	220E-02	Fbxo46	8998	Hypo
chr10	98755271	98756905	Distal Intergenic	-0858	749E-04	220E-02	Fam104a	-26945	Hypo
chr3	79729351	79730404	Intron (NM_001107751/311215, intron 8 of 12)	-1061	751E-04	220E-02	Ext2	67655	Hypo
chr16	20308967	20310776	Distal Intergenic	-0724	753E-04	220E-02	Lzts1	-232033	Hypo
chr8	87606690	87607805	Intron (NM_012600/24552, intron 6 of 13)	-0801	754E-04	220E-02	Me1	52446	Hypo
chr3	17244701	17249120	Distal Intergenic	-0455	754E-04	220E-02	Mvb12b	-52043	Hypo
chr1	98625644	98626732	Distal Intergenic	-0818	754E-04	220E-02	E2f8	-41546	Hypo
chr1	144353458	144355487	Exon (NM_001108492/361604, exon 10 of 20)	-0591	755E-04	220E-02	Ccdc89	-30765	Hypo
chr16	46267859	46269494	Promoter (<=1kb)	-0570	759E-04	222E-02	Ankrd37	0	Hypo
chr10	46479607	46481409	Intron (NM_001191645/303206, intron 15 of 27)	-0707	761E-04	222E-02	Prpsap2	-33765	Hypo
chr13	87244208	87245212	Intron (NM_019343/54296, intron 2 of 17)	-1016	766E-04	223E-02	Rgs7	163411	Hypo
chr12	4847762	4851104	Distal Intergenic	-0596	767E-04	223E-02	Lnc001	-31322	Hypo
chr11	57567481	57569639	Intron (NM_001105880/288105, intron 3 of 12)	-0543	767E-04	223E-02	Zbtb20	221575	Hypo
chr4	122072126	122073517	Promoter (<=1kb)	-0740	771E-04	224E-02	Txnrd3	0	Hypo
chr18	35105023	35107111	Distal Intergenic	-0601	773E-04	224E-02	Ppp2r2b	-24357	Hypo
chr7	86306070	86312717	Distal Intergenic	-0370	774E-04	224E-02	Enpp2	-23596	Hypo
chr9	61261890	61263574	Intron (NM_080407/140590, intron 3 of 12)	-0735	775E-04	224E-02	Bmpr2	69172	Hypo
chr6	69925662	69927377	Distal Intergenic	-0717	776E-04	224E-02	Arhgap5	-60833	Hypo
chr19	9611806	9613848	Promoter (2-3kb)	-0655	779E-04	225E-02	Cfap20	2827	Hypo
chrX	42223817	42225614	Promoter (<=1kb)	-0686	780E-04	225E-02	Sts	0	Hypo
chr10	87486043	87487346	Distal Intergenic	-0869	785E-04	226E-02	Fzd2	-74520	Hypo
chr1	48541739	48544786	Intron (NM_133406/170919, intron 3 of 8)	-0604	788E-04	227E-02	Agpat4	88437	Hypo
chr9	60402690	60406896	Intron (NM_133560/171086, intron 1 of 15)	-0442	790E-04	227E-02	Trak2	7122	Hypo
chr3	174340	175006	Distal Intergenic	-0862	794E-04	228E-02	Rn18s	-2056801	Hypo
chr13	45519065	45521161	Distal Intergenic	-0638	796E-04	228E-02	Btg2	14481	Hypo
chr1	47805407	47806818	Distal Intergenic	-0836	798E-04	228E-02	Tcp1	29926	Hypo
chr6	138570286	138576763	Distal Intergenic	-0436	804E-04	230E-02	Rapgef5	-25011	Hypo
chr3	167209520	167211878	Promoter (<=1kb)	-0554	807E-04	230E-02	Osbpl2	0	Hypo

chr6	70594312	70597563	Intron (NM_022618/64553, intron 12 of 13)	-0507	812E-04	231E-02	Akap6	410137	Hypo
chr1	230139742	230140572	Intron (NM_181386/353229, intron 3 of 9)	-0968	813E-04	231E-02	Sgms1	119020	Hypo
chr6	133434606	133435993	Distal Intergenic	-0903	814E-04	231E-02	Adam6	767632	Hypo
chr15	85618808	85620250	Distal Intergenic	-0735	817E-04	232E-02	Slitrk1	107040	Hypo
chr16	25082303	25085718	Intron (NM_013128/25669, intron 1 of 8)	-0610	818E-04	232E-02	Cpe	52027	Hypo
chr8	23717017	23724057	Promoter (<=1kb)	-0365	819E-04	232E-02	Septin7	0	Hypo
chr6	105469892	105470592	Intron (NM_199109/314323, intron 9 of 9)	-1171	820E-04	232E-02	Erg28	12486	Hypo
chr7	131477636	131478656	Distal Intergenic	-0839	829E-04	234E-02	Mettl7a	25426	Hypo
chr6	133408699	133410608	Distal Intergenic	-0806	829E-04	234E-02	Adam6	741725	Hypo
chr18	40053997	40056576	Exon (NM_001108430/361324, exon 19 of 19)	-0632	835E-04	235E-02	Sema6a	115378	Hypo
chr20	12372961	12382613	Promoter (<=1kb)	-0373	836E-04	235E-02	S100b	0	Hypo
chr2	45660458	45662069	Distal Intergenic	-0827	839E-04	235E-02	Hspb3	-364459	Hypo
chr3	115566876	115568904	Distal Intergenic	-0589	841E-04	236E-02	Bcl2l11	200093	Hypo
chr9	90326125	90327254	Exon (NM_001108230/316611, exon 10 of 16)	-0943	841E-04	236E-02	Agap1	138100	Hypo
chr4	159515273	159520312	Distal Intergenic	-0386	845E-04	237E-02	Kcna1	-44149	Hypo
chr10	104296681	104297663	Promoter (<=1kb)	-0774	851E-04	238E-02	Cbx8	0	Hypo
chr1	243062035	243064061	Intron (NM_001106352/293937, intron 2 of 12)	-0590	853E-04	238E-02	Pkd2l1	5970	Hypo
chr1	222784723	222786117	Intron (NM_001037793/499337, intron 24 of 47)	-0791	866E-04	241E-02	Dock8	135414	Hypo
chr2	101534986	101537743	Distal Intergenic	-0631	871E-04	242E-02	Armc1	114437	Hypo
chr19	14878206	14881318	Distal Intergenic	-0542	871E-04	242E-02	Crnde	234319	Hypo
chr3	1151808	1153272	Distal Intergenic	-0922	873E-04	242E-02	Rn18s	-1078535	Hypo
chr1	90394118	90395356	Distal Intergenic	-0919	876E-04	243E-02	Uri1	309300	Hypo
chr10	8218960	8220214	Intron (NM_001106974/302920, intron 8 of 12)	-0911	877E-04	243E-02	Rbfox1	272559	Hypo
chr3	42625742	42635533	Promoter (<=1kb)	-0392	878E-04	243E-02	Ermn	0	Hypo
chr16	20087478	20088101	Distal Intergenic	-0967	878E-04	243E-02	Zfp868	324749	Hypo
chr15	92242106	92243000	Promoter (2-3kb)	-0972	883E-04	244E-02	Gpc5	2560	Hypo
chr1	7263007	7264067	Distal Intergenic	-0798	886E-04	245E-02	Utrn	-38694	Hypo
chr1	213335187	213341519	Distal Intergenic	-0405	888E-04	245E-02	Cep78	-60063	Hypo
chr8	38170306	38171176	Distal Intergenic	-1015	889E-04	245E-02	Or8b101c	102489	Hypo
chr4	96089335	96090552	Intron (NM_053661/114120, intron 2 of 3)	-0884	891E-04	246E-02	Gng12	53089	Hypo
chr5	56570388	56578171	Distal Intergenic	-0359	893E-04	246E-02	Ubap1	49645	Hypo
chr9	81191085	81192210	Promoter (2-3kb)	-0861	895E-04	246E-02	Serpine2	-2269	Hypo
chr18	52171491	52173242	Exon (NM_001014242/364879, exon 4 of 6)	-0685	897E-04	246E-02	Isoc1	12701	Hypo

chr18	49246861	49249967	Distal Intergenic	-0478	904E-04	248E-02	Gramd2b	-634047	Hypo
chr2	91929043	91929696	Distal Intergenic	-1017	906E-04	248E-02	Pag1	-127520	Hypo
chr13	66239821	66242451	Distal Intergenic	-0599	906E-04	248E-02	Glul	214180	Hypo
chr1	189838652	189839811	Intron (NM_001143858/309081, intron 29 of 54)	-0911	917E-04	250E-02	Insyn2a	-85730	Hypo
chr5	96222415	96224300	Distal Intergenic	-0685	917E-04	250E-02	Mpdz	-301916	Hypo
chr3	89776662	89778154	Distal Intergenic	-0819	919E-04	250E-02	Elf5	-31433	Hypo
chr18	78087472	78091134	Distal Intergenic	-0602	924E-04	251E-02	Cndp2	-30442	Hypo
chr10	98852205	98857156	Exon (NM_001108303/360652, exon 35 of 42)	-0488	925E-04	251E-02	Cdc42ep4	-54997	Hypo
chr13	82189385	82190658	Promoter (<=1kb)	-0819	930E-04	252E-02	Hsd17b7	0	Hypo
chr8	41697775	41702494	Distal Intergenic	-0405	939E-04	254E-02	Lnc215	-143233	Hypo
chr9	23320136	23326330	Distal Intergenic	-0450	951E-04	257E-02	Paqr8	-15276	Hypo
chr1	24668638	24672075	Distal Intergenic	-0527	952E-04	257E-02	Trdn	-258225	Hypo
chr1	218143013	218144312	Exon (NM_022407/24188, exon 10 of 13)	-0803	954E-04	257E-02	Aldh1a1	31928	Hypo
chr3	125723486	125725271	Distal Intergenic	-0636	955E-04	257E-02	Btbd3	110793	Hypo
chr8	110914854	110917960	Distal Intergenic	-0571	958E-04	257E-02	Lrrc2	-20303	Hypo
chr1	154423245	154424463	Exon (NM_001024750/293144, exon 11 of 12)	-0823	960E-04	257E-02	Pold3	32217	Hypo
chr20	43926933	43930297	Distal Intergenic	-0412	960E-04	257E-02	Cdk19	156524	Hypo
chr3	36865264	36866365	Distal Intergenic	-0869	967E-04	258E-02	Arl5a	36997	Hypo
chr12	4884599	4888160	Promoter (2-3kb)	-0509	971E-04	259E-02	Lnc001	2173	Hypo
chr16	55250038	55251860	Distal Intergenic	-0715	972E-04	259E-02	LOC688765	-3396	Hypo
chr3	58326912	58337179	5' UTR	-0349	980E-04	261E-02	Wipf1	35563	Hypo
chr15	4700849	4701659	Intron (NM_001009976/364261, intron 2 of 3)	-1043	984E-04	261E-02	Spetex2h	16206	Hypo
chr11	55568970	55573000	Exon (NM_213630/407756, exon 3 of 6)	-0516	985E-04	261E-02	Btla	13982	Hypo
chr2	189370278	189371834	Distal Intergenic	-0709	986E-04	261E-02	Slc22a15	-11488	Hypo
chr14	31400349	31402420	Distal Intergenic	-0758	986E-04	261E-02	Paics	-184583	Hypo
chr20	12774808	12780846	Distal Intergenic	-0408	986E-04	261E-02	Derl3	-7781	Hypo
chr10	12568237	12570057	Distal Intergenic	-0725	994E-04	262E-02	Zfp213	29224	Hypo
chr5	49584860	49588031	Intron (NM_001108970/366352, intron 2 of 4)	-0615	995E-04	262E-02	Cga	97792	Hypo
chr18	26409971	26413739	Distal Intergenic	-0438	995E-04	262E-02	Reep2	-24607	Hypo
chr16	56057436	56059150	Distal Intergenic	-0586	998E-04	263E-02	Prag1	82003	Hypo
chr4	118281215	118290836	Promoter (<=1kb)	-0420	998E-04	263E-02	Nat8f5	0	Hypo
chr16	18976216	18980057	3' UTR	-0571	100E-03	264E-02	Crtc1	-8214	Hypo
chr7	119506056	119508034	Distal Intergenic	-0701	101E-03	264E-02	Zdhhc25	-184645	Hypo

chr6	44548559	44549526	Distal Intergenic	-1007	101E-03	265E-02	Sox11	-538205	Hypo
chr4	10160040	10161672	Distal Intergenic	-0600	102E-03	268E-02	Prkag2	-44016	Hypo
chr6	22465856	22466855	Distal Intergenic	-0924	103E-03	268E-02	Lbh	126201	Hypo
chr17	19932061	19944365	Distal Intergenic	-0349	103E-03	270E-02	Dtnbp1	246843	Hypo
chr2	41263549	41265410	Intron (NM_001113329/24627, intron 2 of 15)	-0662	104E-03	271E-02	Pde4d	40863	Hypo
chr8	106141852	106142688	Distal Intergenic	-0845	105E-03	272E-02	Atp2c1	-10538	Hypo
chr12	35972099	35975740	Promoter (<=1kb)	-0451	105E-03	272E-02	Tpcn1	0	Hypo
chr19	53424730	53426135	Exon (NM_001009704/361442, exon 11 of 24)	-0884	106E-03	274E-02	Sipa1l2	114293	Hypo
chr4	116611137	116615892	Intron (NM_001107869/312492, intron 30 of 52)	-0419	106E-03	274E-02	Dysf	108553	Hypo
chr5	123992088	123993802	Distal Intergenic	-0754	107E-03	276E-02	Osbpl9	-29909	Hypo
chr17	34818932	34821398	Distal Intergenic	-0618	107E-03	276E-02	Cdkal1	100252	Hypo
chr7	111471916	111474430	Intron (NM_199117/362962, intron 2 of 5)	-0524	107E-03	277E-02	Cbx7	3543	Hypo
chr19	27862422	27868189	Promoter (<=1kb)	-0417	108E-03	277E-02	Hhip	0	Hypo
chr5	46702117	46704652	Distal Intergenic	-0540	108E-03	277E-02	Bach2	-12369	Hypo
chr1	153656752	153663102	Distal Intergenic	-0370	109E-03	279E-02	Serpinh1	-5947	Hypo
chr1	137630997	137633533	Intron (NM_001105749/116996, intron 12 of 18)	-0502	109E-03	280E-02	Stard5	24846	Hypo
chr13	66118598	66123741	Distal Intergenic	-0379	109E-03	280E-02	Glul	92957	Hypo
chr5	101572990	101575371	Intron (NM_031743/84550, intron 3 of 9)	-0654	110E-03	280E-02	Slc24a2	163966	Hypo
chr19	27216261	27218724	Exon (NM_001108444/361388, exon 4 of 10)	-0654	110E-03	280E-02	Smarca5	-53194	Hypo
chr6	39430559	39434326	3' UTR	-0478	111E-03	282E-02	Ntsr2	6235	Hypo
chr1	55015924	55016802	Distal Intergenic	-0963	111E-03	283E-02	Vom2r9	157638	Hypo
chr8	105664561	105666823	Distal Intergenic	-0506	111E-03	283E-02	Mrpl3	-3361	Hypo
chr10	97864950	97866401	Distal Intergenic	-0714	111E-03	283E-02	Sox9	58465	Hypo
chr5	63757636	63758708	Promoter (<=1kb)	-0937	112E-03	283E-02	Acnat2	-115	Hypo
chr19	15782839	15784468	3' UTR	-0700	112E-03	283E-02	Aktip	-82473	Hypo
chr14	40260272	40262285	Distal Intergenic	-0590	112E-03	284E-02	Grxcr1	-133701	Hypo
chr19	47151871	47153376	Exon (NM_138889/192248, exon 7 of 14)	-0837	113E-03	286E-02	Hsbp1	-247663	Hypo
chr11	31336918	31344254	Distal Intergenic	-0393	114E-03	286E-02	Slc5a3	23071	Hypo
chr14	36517571	36519689	Intron (NM_012956/25450, intron 3 of 8)	-0714	114E-03	288E-02	Gabrb1	29251	Hypo
chr10	38846305	38849920	Intron (NM_001126089/691031, intron 3 of 5)	-0449	114E-03	288E-02	Cdc42se2	55136	Hypo
chr2	195421313	195425853	Distal Intergenic	-0416	115E-03	288E-02	Csf1	-25022	Hypo
chr1	24688486	24692871	Distal Intergenic	-0519	115E-03	289E-02	Trdn	-278073	Hypo
chr7	96467343	96468584	Exon (NM_017142/29241, exon 11 of 18)	-0819	116E-03	291E-02	Adcy8	197327	Hypo

chr20	36499952	36501097	Distal Intergenic	-0772	116E-03	291E-02	Hsf2	-318767	Hypo
chr10	37631743	37635220	Distal Intergenic	-0455	117E-03	293E-02	Gdf9	36019	Hypo
chr17	5016038	5018237	Distal Intergenic	-0655	118E-03	294E-02	Naa35	68123	Hypo
chr1	126949417	126951908	Distal Intergenic	-0578	118E-03	294E-02	Rgma	-177026	Hypo
chr14	46896850	46901088	Exon (NM_001107216/305367, exon 27 of 32)	-0497	118E-03	294E-02	Arap2	178090	Hypo
chr5	152337807	152339626	Intron (NM_001271454/298591, intron 3 of 9)	-0654	119E-03	295E-02	Igsf21	172548	Hypo
chr17	6773864	6775043	Intron (NM_001271297/306759, intron 2 of 10)	-0896	119E-03	295E-02	Spock1	32351	Hypo
chr10	10494246	10499291	3' UTR	-0485	119E-03	295E-02	Ubn1	32719	Hypo
chr1	164955940	164957509	Promoter (<=1kb)	-0652	121E-03	299E-02	rnf141	0	Hypo
chr14	20390138	20390612	Distal Intergenic	-1076	121E-03	299E-02	Csn1s1	-30524	Hypo
chr14	77048161	77049625	Distal Intergenic	-0739	121E-03	299E-02	Tacc3	15527	Hypo
chr3	136343345	136345987	Promoter (2-3kb)	-0631	121E-03	300E-02	Cst3	-2549	Hypo
chr19	20473477	20474348	Intron (NM_001033689/291922, intron 9 of 13)	-0976	122E-03	300E-02	Lonp2	24516	Hypo
chr9	3871787	3874345	Intron (NM_001134762/501088, intron 2 of 21)	-0695	122E-03	301E-02	Tbc1d5	142568	Hypo
chr15	5294462	5295545	Exon (NM_001013992/305691, exon 2 of 8)	-1073	122E-03	301E-02	Zfp385d	127822	Hypo
chr3	88506020	88507442	Distal Intergenic	-0760	122E-03	301E-02	Trim44	221195	Hypo
chr8	26754859	26756534	Distal Intergenic	-0557	123E-03	303E-02	Opcml	-33134	Hypo
chr3	56609119	56610887	Distal Intergenic	-0638	124E-03	304E-02	Itga6	-6657	Hypo
chr15	50158823	50160885	Intron (NM_001134727/502020, intron 2 of 18)	-0669	124E-03	304E-02	Lrch1	88772	Hypo
chr10	38854426	38857542	Intron (NM_001126089/691031, intron 3 of 5)	-0585	124E-03	304E-02	Cdc42se2	47514	Hypo
chr1	40347875	40352957	Promoter (<=1kb)	-0439	124E-03	305E-02	Plekhg1	0	Hypo
chr3	149308465	149311024	Intron (NM_022615/64550, intron 2 of 20)	-0568	125E-03	305E-02	Top1	14807	Hypo
chr6	10858938	10862006	Downstream (<=300bp)	-0639	125E-03	307E-02	Haao	13153	Hypo
chr10	66297388	66303746	Intron (NM_001034014/25364, intron 1 of 9)	-0450	126E-03	307E-02	Asic2	-159009	Hypo
chr9	6720014	6720950	3' UTR	-1176	126E-03	307E-02	LOC301165	11961	Hypo
chr1	172110699	172111721	Distal Intergenic	-0890	126E-03	308E-02	Rps15a	314316	Hypo
chr9	73950408	73951983	3' UTR	-0627	126E-03	308E-02	Xrcc5	-3275	Hypo
chr1	185422264	185423349	Distal Intergenic	-0785	127E-03	308E-02	Plekha1	-4699	Hypo
chr6	98799254	98800892	Distal Intergenic	-0704	128E-03	310E-02	Zfp36l1	134856	Hypo
chr13	62616656	62620824	Exon (NM_001271292/289094, exon 108 of 115)	-0431	128E-03	310E-02	Prg4	-112537	Hypo
chr2	104429724	104432579	Distal Intergenic	-0573	128E-03	311E-02	Tbl1xr1	-369142	Hypo
chr20	9782303	9783435	Promoter (<=1kb)	-1005	129E-03	313E-02	Cryaa	-170	Hypo
chr9	74884419	74885208	Distal Intergenic	-1068	131E-03	315E-02	Pincl	103852	Hypo

chr4	125320860	125321649	Intron (NM_001107877/312566, intron 30 of 46)	-1096	131E-03	316E-02	Prickle2	-106102	Hypo
chr4	104762809	104764112	Intron (NM_001107865/312451, intron 3 of 11)	-0775	131E-03	316E-02	Tcf7l1	81175	Hypo
chr14	74547051	74548008	Promoter (<=1kb)	-1052	131E-03	316E-02	Psapl1	0	Hypo
chr6	133253784	133255343	Distal Intergenic	-0796	132E-03	317E-02	Adam6	586810	Hypo
chr6	69894624	69896583	Distal Intergenic	-0650	132E-03	317E-02	Arhgap5	-91627	Hypo
chr3	136332459	136341787	Promoter (<=1kb)	-0373	132E-03	317E-02	Cst3	0	Hypo
chr19	38657153	38658636	Intron (NM_177930/307842, intron 14 of 18)	-0670	132E-03	317E-02	Mtss2	-34578	Hypo
chr9	50731511	50736601	Promoter (<=1kb)	-0378	132E-03	317E-02	Tmeff2	0	Hypo
chr3	133737778	133739098	Distal Intergenic	-0845	132E-03	317E-02	Crnkl1	-383449	Hypo
chr11	67805691	67806801	Intron (NM_001371837/303883, intron 9 of 20)	-0950	132E-03	317E-02	Lrch3	65463	Hypo
chr20	109265	110287	Intron (NM_133387/170898, intron 4 of 8)	-0875	133E-03	317E-02	Tmlhe	17954	Hypo
chr1	171757742	171759713	Intron (NM_022295/64133, intron 2 of 11)	-0625	134E-03	319E-02	Xylt1	113817	Hypo
chr19	18287399	18289004	Distal Intergenic	-0807	134E-03	320E-02	Cyld	84646	Hypo
chr13	44884563	44885645	Distal Intergenic	-0749	134E-03	320E-02	Sox13	-9041	Hypo
chr10	38262871	38264109	Intron (NM_001108275/360526, intron 9 of 15)	-0791	135E-03	321E-02	P4ha2	19776	Hypo
chr2	135245033	135246459	Distal Intergenic	-0841	135E-03	323E-02	Noct	-24730	Hypo
chr7	34325415	34326340	Distal Intergenic	-1000	136E-03	323E-02	Dusp6	232459	Hypo
chr17	84998013	85000140	Intron (NM_001100577/307171, intron 4 of 12)	-0637	136E-03	324E-02	Apbb1ip	15072	Hypo
chr17	30729387	30732791	Distal Intergenic	-0445	136E-03	324E-02	Psmg4	-11363	Hypo
chr10	72911943	72916691	Intron (NM_001013971/303419, intron 1 of 10)	-0410	136E-03	324E-02	Cuedc1	19306	Hypo
chr6	133450847	133451716	Distal Intergenic	-1006	137E-03	325E-02	Adam6	783873	Hypo
chr7	100103978	100104936	Distal Intergenic	-0908	139E-03	328E-02	Mir30b	27739	Hypo
chr1	15367595	15369067	Intron (NM_080894/140929, intron 2 of 12)	-0808	140E-03	329E-02	Pde7b	123833	Hypo
chr11	66816020	66818793	3' UTR	-0721	140E-03	330E-02	Umps	9911	Hypo
chr3	105518024	105519340	Promoter (<=1kb)	-0840	141E-03	331E-02	Bmf	764	Hypo
chr14	83650030	83651399	Distal Intergenic	-0691	141E-03	331E-02	Hus1	52043	Hypo
chr3	147037187	147038430	3' UTR	-0711	142E-03	333E-02	Snhg11	5776	Hypo
chr3	103958368	103960100	Distal Intergenic	-0558	142E-03	333E-02	Spred1	-23929	Hypo
chr10	38870589	38875066	5' UTR	-0455	142E-03	333E-02	Cdc42se2	29990	Hypo
chr13	89138657	89139886	Intron (NM_031575/29414, intron 1 of 11)	-0950	142E-03	333E-02	Akt3	75950	Hypo
chr19	24785651	24787365	Promoter (<=1kb)	-0554	143E-03	334E-02	Elmod2	0	Hypo
chr4	36361465	36362668	Intron (NM_001177442/296884, intron 2 of 10)	-0875	143E-03	334E-02	Glcci1	53509	Hypo
chr5	124550829	124551747	Intron (NM_130406/140657, intron 7 of 18)	-0769	143E-03	334E-02	Faf1	124765	Hypo

chr1	175261847	175263389	Distal Intergenic	-0660	143E-03	334E-02	Vwa3a	-30484	Hypo
chr7	86398393	86399985	Distal Intergenic	-0672	145E-03	336E-02	Taf2	79631	Hypo
chr1	3385411	3386342	Distal Intergenic	-0886	145E-03	336E-02	Ust	-423367	Hypo
chr13	74867143	74869329	Distal Intergenic	-0510	146E-03	337E-02	Dnm3	-29035	Hypo
chr2	214277710	214284936	Intron (NM_019276/50555, intron 2 of 5)	-0378	147E-03	337E-02	Ugt8	47724	Hypo
chr3	69164938	69166540	Distal Intergenic	-0642	147E-03	337E-02	Zswim2	-149527	Hypo
chr2	86381342	86385955	Distal Intergenic	-0419	147E-03	339E-02		-214573	Hypo
chr1	206053770	206057275	Distal Intergenic	-0464	148E-03	340E-02	Asrgl1	-26655	Hypo
chr14	78860557	78864216	Exon (NM_001044228/289740, exon 5 of 14)	-0491	148E-03	340E-02	Gal3st1	-6577	Hypo
chr10	98250342	98252494	Distal Intergenic	-0518	149E-03	340E-02	Mir297	-33798	Hypo
chr2	95093575	95096168	Distal Intergenic	-0508	149E-03	341E-02	Pkia	-619976	Hypo
chr15	52650970	52653600	Distal Intergenic	-0495	150E-03	343E-02	Dnajc15	596075	Hypo
chr18	1586279	1587355	Distal Intergenic	-0794	151E-03	344E-02	Esco1	82938	Hypo
chr10	86044459	86049816	Promoter (2-3kb)	-0443	151E-03	345E-02	Retreg3	2759	Hypo
chr1	53228010	53228277	Distal Intergenic	-1016	153E-03	347E-02	Smok2a	-52090	Hypo
chr18	12584144	12586131	Distal Intergenic	-0664	153E-03	347E-02	Mep1b	218018	Hypo
chr10	16957813	16960408	Distal Intergenic	-0569	153E-03	347E-02	Efcab9	151837	Hypo
chr5	90429034	90439346	Distal Intergenic	-0362	153E-03	347E-02	Dmac1	-1185277	Hypo
chr2	204564364	204569373	Exon (NM_001107719/310807, exon 8 of 14)	-0419	153E-03	347E-02	Trmt13	-17575	Hypo
chr4	122014425	122015858	Intron (NM_001106608/297436, intron 1 of 7)	-0784	153E-03	347E-02	Chchd6	8343	Hypo
chr6	100086190	100086911	Distal Intergenic	-1102	153E-03	348E-02	Exd2	-29956	Hypo
chr1	40366275	40370817	Intron (NM_001190999/679812, intron 2 of 15)	-0475	154E-03	348E-02	Plekhg1	17591	Hypo
chrX	152115136	152116596	Promoter (<=1kb)	-0695	155E-03	350E-02	Plxna3	0	Hypo
chr3	9360547	9361439	Distal Intergenic	-0949	155E-03	350E-02	Notch1	-37016	Hypo
chr18	49923024	49927114	Intron (NM_001014011/307288, intron 1 of 13)	-0500	155E-03	350E-02	Gramd2b	39010	Hypo
chr10	61648110	61650922	Exon (NM_012836/25306, exon 7 of 21)	-0553	156E-03	351E-02	Cpd	36569	Hypo
chr18	78230687	78232262	Exon (NM_022245/64001, exon 2 of 5)	-0842	156E-03	351E-02	Cyb5a	17535	Hypo
chr3	54296999	54297962	Exon (NM_030827/29216, exon 7 of 79)	-0785	156E-03	352E-02	Lrp2	48746	Hypo
chr11	48356446	48359319	Intron (NM_031753/79559, intron 1 of 15)	-0545	157E-03	352E-02	Alcam	19931	Hypo
chr13	103210807	103211884	Distal Intergenic	-0622	157E-03	353E-02	Ints7	55684	Hypo
chr1	152629026	152630641	Distal Intergenic	-0578	157E-03	353E-02	Tsku	40927	Hypo
chr11	40781202	40782477	Distal Intergenic	-0754	158E-03	353E-02	Arl6	69063	Hypo
chr3	29493183	29494544	Distal Intergenic	-0639	158E-03	354E-02	Zeb2-as1	150105	Hypo

chr4	158167046	158168095	Distal Intergenic	-0964	158E-03	354E-02	Tnfrsf1a	16152	Hypo
chr17	19268113	19269481	Distal Intergenic	-0720	158E-03	354E-02	Mylip	38814	Hypo
chr4	80142325	80144710	Distal Intergenic	-0625	159E-03	355E-02	Mir148a	189148	Hypo
chr3	56045588	56047653	Intron (NM_001270624/116659, intron 3 of 16)	-0728	161E-03	357E-02	Dync1i2	11671	Hypo
chr3	65589326	65590323	Distal Intergenic	-0736	161E-03	358E-02	Nup35	30316	Hypo
chr19	48667089	48673413	Distal Intergenic	-0354	162E-03	358E-02	Gins2	-27750	Hypo
chr20	12783085	12783752	Distal Intergenic	-0965	162E-03	360E-02	Mif	-7167	Hypo
chr5	102617704	102619628	Distal Intergenic	-0657	163E-03	360E-02	Mllt3	-259878	Hypo
chr1	34416855	34418432	Intron (NM_031007/81636, intron 2 of 24)	-0759	163E-03	360E-02	Adcy2	40944	Hypo
chr5	117016625	117019961	Intron (NM_017031/24626, intron 1 of 14)	-0498	163E-03	360E-02	Pde4b	23189	Hypo
chr2	45565598	45567618	Distal Intergenic	-0585	163E-03	360E-02	Hspb3	-269599	Hypo
chr11	55025010	55027230	Distal Intergenic	-0548	165E-03	362E-02	Abhd10	-54110	Hypo
chr8	87586536	87588248	Intron (NM_012600/24552, intron 6 of 13)	-0621	165E-03	362E-02	Rwdd2a	50360	Hypo
chrX	100094418	100102442	Promoter (<=1kb)	-0392	165E-03	364E-02	Morf4l2	-760	Hypo
chr5	165115380	165117500	Distal Intergenic	-0588	166E-03	364E-02	Actrt2	119992	Hypo
chr4	67481008	67486788	Exon (NM_001108622/362342, exon 7 of 20)	-0406	166E-03	365E-02	Clec2l	82742	Hypo
chr8	73576927	73578912	Intron (NM_001037651/315806, intron 2 of 19)	-0606	167E-03	366E-02	Prtg	28461	Hypo
chr9	82107892	82109916	Distal Intergenic	-0578	168E-03	366E-02	Cul3	-456379	Hypo
chr10	92962817	92966275	Intron (NM_001105713/24680, intron 8 of 15)	-0466	168E-03	366E-02	Cacng5	-125816	Hypo
chr12	1255711	1256541	Intron (NM_017071/24954, intron 3 of 21)	-1020	168E-03	367E-02	Insr	74342	Hypo
chr6	90827884	90829713	Intron (NM_053865/116644, intron 3 of 8)	-0721	169E-03	367E-02	Ccdc175	-82921	Hypo
chr8	62193613	62194363	Distal Intergenic	-1089	169E-03	368E-02	Rplp1	200978	Hypo
chr5	134560185	134568085	Intron (NM_001100669/298500, intron 1 of 9)	-0392	171E-03	370E-02	Smap2	8853	Hypo
chr6	62554154	62556869	Distal Intergenic	-0563	171E-03	371E-02	Stxbp6	-396767	Hypo
chr3	105519500	105520684	Promoter (<=1kb)	-0760	171E-03	371E-02	Bmf	0	Hypo
chr15	19956190	19958789	Distal Intergenic	-0494	172E-03	372E-02	Cdkn3	-63820	Hypo
chr20	44324796	44327214	Promoter (<=1kb)	-0423	173E-03	374E-02	Cdc40	0	Hypo
chr15	80496757	80499775	Promoter (<=1kb)	-0519	174E-03	375E-02	Slain1	0	Hypo
chr1	87511940	87514319	Distal Intergenic	-0603	174E-03	376E-02	Pepd	-22331	Hypo
chr8	37331621	37333630	Intron (NM_001108759/363045, intron 3 of 9)	-0521	174E-03	376E-02	Siae	12874	Hypo
chr10	37669201	37673083	Distal Intergenic	-0453	176E-03	379E-02	Septin8	-11556	Hypo
chr5	147754438	147756548	Exon (NM_001025772/500566, exon 6 of 9)	-0522	177E-03	379E-02	Stpg1	27792	Hypo
chr1	163846641	163848810	Distal Intergenic	-0513	177E-03	380E-02	Nrip3	-31605	Hypo

chr1	166846412	166847256	Intron (NM_001198589/361630, intron 1 of 12)	-1095	177E-03	380E-02	Tead1	53792	Hypo
chr4	67474799	67480859	Exon (NM_001108622/362342, exon 8 of 20)	-0366	178E-03	380E-02	Clec2l	76533	Hypo
chr14	4429290	4430239	Distal Intergenic	-0725	178E-03	381E-02	Lrrc8b	-68210	Hypo
chr6	39594646	39596464	Promoter (2-3kb)	-0725	178E-03	381E-02	E2f6	2144	Hypo
chr4	118323910	118325360	Promoter (2-3kb)	-0706	180E-03	385E-02	Nat8f2	-2135	Hypo
chr11	23272956	23274049	Distal Intergenic	-0944	181E-03	385E-02	Mir155hg	-499419	Hypo
chr9	3846671	3847756	5' UTR	-0944	181E-03	386E-02	Tbc1d5	169157	Hypo
chr20	12311972	12318263	Exon (NM_001191564/690211, exon 4 of 38)	-0389	182E-03	387E-02	Dip2a	27318	Hypo
chr20	30531699	30532990	Promoter (<=1kb)	-0711	182E-03	387E-02	Kifbp	0	Hypo
chr17	1006111	1009420	Exon (NM_001106098/290959, exon 7 of 12)	-0513	183E-03	388E-02	Hsd17b3	-17809	Hypo
chr5	90419788	90424283	Distal Intergenic	-0417	183E-03	388E-02	Dmac1	-1176031	Hypo
chr7	108603563	108606459	Promoter (1-2kb)	-0489	184E-03	389E-02	Zfp7	-1230	Hypo
chr3	17554752	17557164	Intron (NM_001107834/311876, intron 2 of 8)	-0667	184E-03	390E-02	Pbx3	125186	Hypo
chr6	53234514	53237304	Intron (NM_001008386/493574, intron 6 of 9)	-0496	187E-03	393E-02	Crppa	113076	Hypo
chr3	18790661	18792781	Intron (NM_145674/246766, intron 1 of 6)	-0701	187E-03	393E-02	Ggta1	16339	Hypo
chr12	33152019	33155786	Distal Intergenic	-0476	187E-03	394E-02	Lrrc43	-59762	Hypo
chr6	133423477	133425039	Distal Intergenic	-0852	189E-03	396E-02	Adam6	756503	Hypo
chr20	3738535	3741469	Promoter (1-2kb)	-0456	189E-03	396E-02	Ly6g6f	1076	Hypo
chr20	41893741	41894733	Distal Intergenic	-0993	189E-03	397E-02	Lama4	-497535	Hypo
chr12	20496065	20497215	Distal Intergenic	-0823	190E-03	398E-02	Orai2	-3093	Hypo
chr2	118012473	118013147	Distal Intergenic	-0904	190E-03	398E-02	Sox2	475544	Hypo
chr5	96546303	96547356	Distal Intergenic	-0877	191E-03	400E-02	Nfib	425626	Hypo
chr5	147459151	147464023	Exon (NM_031818/83718, exon 4 of 6)	-0438	191E-03	400E-02	Clic4	49255	Hypo
chr17	2044770	2045871	Intron (NM_001012346/290963, intron 2 of 14)	-0813	191E-03	400E-02	Aopep	31305	Hypo
chr11	78524042	78524908	Intron (NM_013126/25666, intron 21 of 24)	-0831	191E-03	400E-02	Etv5	-83802	Hypo
chr14	78023622	78024516	Intron (NM_001191682/305467, intron 7 of 32)	-0800	191E-03	400E-02	Sfi1	23739	Hypo
chr11	79311427	79312413	Distal Intergenic	-0743	191E-03	400E-02	Ehhadh	69500	Hypo
chr2	14933234	14937300	Distal Intergenic	-0413	193E-03	404E-02	Mir3597-2	-735406	Hypo
chr19	13754259	13755713	Intron (NM_001014221/363337, intron 1 of 4)	-0698	193E-03	404E-02		16379	Hypo
chr18	72322176	72323144	Distal Intergenic	-0845	193E-03	404E-02	Slc14a2	-282714	Hypo
chr19	19366173	19367651	Intron (NM_053583/94188, intron 5 of 7)	-0733	194E-03	404E-02	Cbln1	-241112	Hypo
chr17	38430335	38433074	Distal Intergenic	-0579	194E-03	404E-02	Hdgfl1	222429	Hypo
chr3	139463819	139465906	Intron (NM_001106524/296259, intron 5 of 13)	-0691	195E-03	405E-02	Acss1	34419	Hypo

chr7	114028596	114030435	Intron (NM_001130574/366964, intron 1 of 4)	-0741	195E-03	406E-02	Tcf20	21404	Hypo
chr3	111371913	111373500	Distal Intergenic	-0760	196E-03	406E-02	Sema6d	-510372	Hypo
chr11	36756592	36758110	Intron (NM_001002802/288227, intron 2 of 8)	-0735	196E-03	406E-02	Bace2	49119	Hypo
chr11	61686053	61688233	Distal Intergenic	-0633	197E-03	407E-02	Igsf11	180115	Hypo
chr3	58548409	58554166	Promoter (<=1kb)	-0367	198E-03	411E-02	Chn1	0	Hypo
chr6	16388107	16390045	Exon (NM_019148/29149, exon 6 of 18)	-0596	201E-03	415E-02	Strn	52737	Hypo
chr6	40898025	40899544	Intron (NM_020306/57027, intron 4 of 18)	-0771	202E-03	416E-02	Adam17	21156	Hypo
chr19	35995362	35996463	Distal Intergenic	-0931	202E-03	416E-02	Psmd7	-493757	Hypo
chr3	150699005	150701380	Intron (NM_001108603/362263, intron 6 of 27)	-0486	202E-03	416E-02	Ptprt	287283	Hypo
chr11	1699470	1700440	Distal Intergenic	-0946	202E-03	416E-02	Epha3	-225570	Hypo
chr2	242384126	242385202	Intron (NM_019123/29758, intron 2 of 4)	-0851	203E-03	417E-02	St6galnac3	259864	Hypo
chr5	69727379	69731333	Distal Intergenic	-0432	203E-03	417E-02	Rad23b	-358899	Hypo
chr10	87560078	87562484	Promoter (<=1kb)	-0553	203E-03	417E-02	Fzd2	0	Hypo
chr6	10801029	10806686	Distal Intergenic	-0406	204E-03	418E-02	Haao	-39099	Hypo
chr7	8309973	8311256	Distal Intergenic	-0824	204E-03	418E-02	Dohh	14949	Hypo
chr9	10056776	10057424	Distal Intergenic	-0919	204E-03	419E-02	Adgre4	296421	Hypo
chr16	79551708	79552895	Distal Intergenic	-0833	205E-03	419E-02	Lig4	33240	Hypo
chr18	40047426	40053060	3' UTR	-0360	205E-03	419E-02	Sema6a	118894	Hypo
chr17	83580912	83581946	Intron (NM_001106127/291355, intron 3 of 8)	-0895	205E-03	419E-02	Prtfdc1	72571	Hypo
chr10	52834593	52837246	Intron (NM_031656/59074, intron 7 of 7)	-0567	206E-03	420E-02	Stx8	178843	Hypo
chr18	74695972	74696513	Distal Intergenic	-1037	206E-03	420E-02	Sall3	-282144	Hypo
chr2	19923520	19925781	Distal Intergenic	-0570	206E-03	420E-02	Hapln1	-705859	Hypo
chr16	10390842	10393543	Intron (NM_024378/79219, intron 4 of 16)	-0490	207E-03	421E-02	Mir346	172724	Hypo
chr1	181849105	181850087	Distal Intergenic	-0893	208E-03	423E-02	Septin1	-12539	Hypo
chr20	42042773	42045547	Distal Intergenic	-0521	208E-03	423E-02	Lama4	-346721	Hypo
chr12	35041221	35043086	3' UTR	-0630	209E-03	425E-02	Adam1a	23788	Hypo
chr14	2318100	2318840	Distal Intergenic	-0867	209E-03	425E-02	Lpcat2b	23132	Hypo
chr8	49971711	49975666	Intron (NM_031521/24586, intron 1 of 18)	-0440	210E-03	426E-02	Ttc12	-126026	Hypo
chr3	1154626	1155578	Distal Intergenic	-0880	210E-03	426E-02	Rn18s	-1076229	Hypo
chr1	3298913	3301703	Distal Intergenic	-0461	211E-03	427E-02	Ust	-336869	Hypo
chr10	98845545	98847908	Intron (NM_001108303/360652, intron 37 of 41)	-0653	211E-03	427E-02	Cdc42ep4	-48337	Hypo
chr4	126685208	126686418	Intron (NM_001030045/500261, intron 1 of 22)	-0777	213E-03	428E-02	Magi1	124341	Hypo
chr15	80509878	80510792	Intron (NM_001014139/361087, intron 1 of 5)	-0895	213E-03	428E-02	Slain1	11231	Hypo

chr4	130619258	130621119	3' UTR	-0605	213E-03	428E-02	Mitf	210041	Hypo
chr18	47771952	47774130	Distal Intergenic	-0653	214E-03	429E-02	Csnk1g3	472373	Hypo
chr3	139472859	139478150	Exon (NM_001106524/296259, exon 3 of 14)	-0410	216E-03	433E-02	Acss1	22175	Hypo
chr15	1162338	1163293	Distal Intergenic	-0795	217E-03	433E-02	Kcnma1	860261	Hypo
chr15	70228842	70229767	Intron (NM_001191688/306091, intron 1 of 3)	-0967	217E-03	433E-02	Pcdh9	5898	Hypo
chr13	45760554	45762653	Exon (NM_080409/140592, exon 25 of 29)	-0588	218E-03	435E-02	Myog	15099	Hypo
chr8	72617107	72620996	Intron (NM_013176/25720, intron 5 of 20)	-0483	218E-03	435E-02	Tcf12	178205	Hypo
chr1	197074466	197076689	Distal Intergenic	-0567	219E-03	436E-02	Mob2	73213	Hypo
chr10	4279006	4284453	Exon (NM_001109526/689142, exon 2 of 21)	-0453	220E-03	439E-02	Snx29	14202	Hypo
chr3	139673230	139676564	Exon (NM_001024314/499913, exon 3 of 13)	-0515	220E-03	439E-02	Abhd12	42958	Hypo
chr9	75221496	75223217	Distal Intergenic	-0544	221E-03	439E-02	Pincl	-221736	Hypo
chr1	53254636	53255393	Distal Intergenic	-0854	221E-03	439E-02	Smok2a	-24974	Hypo
chr4	169742399	169744349	Intron (NM_001173509/312806, intron 2 of 2)	-0578	221E-03	440E-02	Art4	6316	Hypo
chr1	40360120	40362370	Intron (NM_001190999/679812, intron 2 of 15)	-0604	223E-03	442E-02	Plekhg1	11436	Hypo
chr14	103343209	103345686	Distal Intergenic	-0536	223E-03	442E-02	Clhc1	55075	Hypo
chr7	127707045	127712350	Distal Intergenic	-0397	224E-03	444E-02	Scaf11	-83130	Hypo
chr7	82995687	82998521	Distal Intergenic	-0535	226E-03	445E-02	Eif3h	175915	Hypo
chr14	71741318	71745256	Distal Intergenic	-0433	226E-03	446E-02	Wdr1	-512776	Hypo
chr2	58057547	58058771	Distal Intergenic	-0861	226E-03	446E-02	Nadk2	-58903	Hypo
chr12	34984150	34986245	Distal Intergenic	-0512	228E-03	448E-02	Mapkapk5	-7847	Hypo
chr2	59608856	59610704	Intron (NM_001011947/294804, intron 2 of 16)	-0584	228E-03	448E-02	Rai14	71897	Hypo
chr3	17575400	17576935	Intron (NM_001107834/311876, intron 2 of 8)	-0889	228E-03	448E-02	Pbx3	105415	Hypo
chr5	83867430	83874269	Exon (NM_173134/286919, exon 15 of 37)	-0362	228E-03	448E-02	Cdk5rap2	86513	Hypo
chr15	41997067	41998701	Exon (NM_001372012/305987, exon 44 of 52)	-0685	229E-03	449E-02	Gnrh1	24585	Hypo
chr5	147073866	147075962	Promoter (<=1kb)	-0502	229E-03	449E-02	Maco1	0	Hypo
chr4	117449999	117451641	Intron (NM_001109246/500233, intron 6 of 21)	-0766	229E-03	449E-02	Exoc6b	98533	Hypo
chr7	63743003	63745926	Distal Intergenic	-0468	230E-03	450E-02	Rdh7	-53628	Hypo
chr10	98322243	98324053	Intron (NM_001013042/287796, intron 7 of 9)	-0600	230E-03	450E-02	Mir297	-105699	Hypo
chr3	17619796	17622289	Intron (NM_001107834/311876, intron 2 of 8)	-0632	231E-03	451E-02	Pbx3	60061	Hypo
chr1	225525260	225527247	Intron (NM_001012172/361746, intron 6 of 15)	-0618	231E-03	451E-02	Rfx3	181595	Hypo
chr8	65614582	65615617	Distal Intergenic	-0780	231E-03	452E-02	Igdcc4	-6280	Hypo
chr1	133506362	133507592	Distal Intergenic	-0733	232E-03	452E-02	Rhcg	48242	Hypo
chr2	115901323	115908244	Promoter (<=1kb)	-0386	232E-03	452E-02	Pex5l	0	Hypo

chr17	40085744	40090830	Exon (NM_001100512/291132, exon 22 of 25)	-0393	233E-03	453E-02	Mrs2	21782	Hypo
chr3	153673326	153674120	Distal Intergenic	-0990	233E-03	453E-02	Mmp9	-10038	Hypo
chr8	84943108	84944755	Intron (NM_019267/29711, intron 8 of 10)	-0606	233E-03	453E-02	Bckdhb	97844	Hypo
chr1	198521296	198522210	Intron (NM_032073/84020, intron 11 of 15)	-0817	233E-03	453E-02	Cdkn1c	135837	Hypo
chr8	102926858	102930774	Intron (NM_001104528/24338, intron 1 of 15)	-0426	233E-03	453E-02	Ephb1	14065	Hypo
chr4	127523541	127524304	Distal Intergenic	-1039	235E-03	455E-02	Kbtbd8	-151837	Hypo
chr1	183157843	183162881	Distal Intergenic	-0392	235E-03	455E-02	Inpp5f	-27599	Hypo
chr11	63369862	63370771	Intron (NM_001271250/288080, intron 1 of 28)	-0900	235E-03	455E-02	Stxbp5l	35195	Hypo
chr15	80481601	80488288	Distal Intergenic	-0363	235E-03	455E-02	Slain1	-10359	Hypo
chr8	113235640	113236730	Distal Intergenic	-0875	236E-03	456E-02	Pdcd6ip	410043	Hypo
chr9	70506103	70506923	Intron (NM_021687/59323, intron 1 of 28)	-0855	237E-03	457E-02	Erbb4	89052	Hypo
chr8	49654238	49655084	Distal Intergenic	-1080	237E-03	457E-02	Drd2	-53843	Hypo
chr15	40683473	40686297	Distal Intergenic	-0527	237E-03	457E-02	Stmn4	142047	Hypo
chr2	135827971	135829499	Intron (NM_001107675/310405, intron 2 of 4)	-0826	239E-03	459E-02	Mgst2	134415	Hypo
chr9	2859518	2860398	Distal Intergenic	-0997	239E-03	459E-02	Vom2r79	353621	Hypo
chr10	79228039	79229113	Distal Intergenic	-0912	239E-03	459E-02	Luc7l3	47425	Hypo
chr4	147326353	147329983	Exon (NM_024372/79213, exon 5 of 14)	-0500	240E-03	459E-02	Slc6a11	28381	Hypo
chr14	35082681	35085841	Distal Intergenic	-0522	241E-03	462E-02	Zar1	111541	Hypo
chr4	65361247	65362223	Intron (NM_017066/24924, intron 1 of 4)	-0761	242E-03	463E-02	Ptn	13083	Hypo
chr20	28080621	28082973	Distal Intergenic	-0517	243E-03	463E-02	Chst3	37994	Hypo
chr13	48962051	48963538	Distal Intergenic	-0620	245E-03	466E-02	Mir181a-1	-521989	Hypo
chr1	249686085	249688134	Distal Intergenic	-0519	245E-03	466E-02	Sorcs1	-91806	Hypo
chr8	67928264	67929483	Distal Intergenic	-0801	247E-03	468E-02	Mir190a-2	-77193	Hypo
chr18	14937508	14940743	Distal Intergenic	-0543	248E-03	470E-02	Mapre2	-130628	Hypo
chr3	49603635	49605182	Intron (NM_031623/58844, intron 3 of 13)	-0672	249E-03	471E-02	Grb14	78623	Hypo
chr15	4349332	4350902	Intron (NM_031754/80850, intron 2 of 3)	-0585	249E-03	471E-02	Gng2	32696	Hypo
chr1	87828695	87830305	Promoter (<=1kb)	-0730	249E-03	471E-02	Slc7a10	0	Hypo
chr5	117034961	117036982	Intron (NM_017031/24626, intron 1 of 14)	-0579	250E-03	471E-02	Pde4b	41525	Hypo
chr7	11255356	11257660	Intron (NM_001100903/362844, intron 1 of 18)	-0554	250E-03	472E-02	Brd4	38369	Hypo
chr1	3153351	3160873	Distal Intergenic	-0394	251E-03	473E-02	Ust	-191307	Hypo
chr6	39288088	39289100	Distal Intergenic	-0748	251E-03	473E-02	Lpin1	91076	Hypo
chr2	43953031	43954867	Intron (NM_001270039/361898, intron 5 of 9)	-0687	251E-03	473E-02	Ankrd55	38521	Hypo
chr6	19961809	19963293	Distal Intergenic	-0774	251E-03	473E-02	Rasgrp3	-3321	Hypo

chr19	52398522	52402995	Promoter (<=1kb)	-0485	251E-03	473E-02	Pgbd5	0	Hypo
chr18	74271087	74272293	Intron (NM_001106130/291411, intron 11 of 29)	-0779	252E-03	474E-02	Atp9b	96660	Hypo
chr4	179029984	179036893	3' UTR	-0360	253E-03	475E-02	Sspn	132837	Hypo
chr18	62535543	62537031	Distal Intergenic	-0662	254E-03	475E-02	Mc2r	-520055	Hypo
chr7	90714010	90715497	Distal Intergenic	-0602	254E-03	475E-02	Mtss1	-86168	Hypo
chr4	63622214	63623534	Distal Intergenic	-0766	254E-03	475E-02	Wdr91	-30481	Hypo
chr9	10075018	10075630	Distal Intergenic	-0848	255E-03	477E-02	Adgre4	314663	Hypo
chr8	41815944	41819155	Distal Intergenic	-0390	258E-03	481E-02	Lnc215	-26572	Hypo
chr12	16942321	16943059	Distal Intergenic	-0740	259E-03	482E-02	Azgp1	11278	Hypo
chr1	53301677	53302169	Intron (NM_001191619/292316, intron 1 of 1)	-1185	261E-03	484E-02	Smok2a	21310	Hypo
chr11	57600175	57602331	Intron (NM_001105880/288105, intron 3 of 12)	-0555	262E-03	485E-02	Zbtb20	188883	Hypo
chr3	16775477	16777253	Distal Intergenic	-0546	263E-03	487E-02	Zbtb43	-11662	Hypo
chr1	56742514	56743561	Distal Intergenic	-1002	263E-03	487E-02	Chd1	78460	Hypo
chr11	43322709	43324394	Intron (NM_001371895/304019, intron 2 of 18)	-0664	264E-03	489E-02	Tbc1d23	19354	Hypo
chr2	146841014	146842084	Distal Intergenic	-0821	265E-03	490E-02	Dhx36	52488	Hypo
chr9	6894788	6896416	Intron (NM_133547/171072, intron 6 of 9)	-0835	265E-03	490E-02	Sult1c2a	4695	Hypo
chr5	137157259	137159738	Exon (NM_001135709/298528, exon 6 of 19)	-0480	266E-03	492E-02	Epha10	16602	Hypo
chr1	121730203	121736768	Intron (NM_052807/25718, intron 2 of 20)	-0348	266E-03	492E-02	Igf1r	179462	Hypo
chr3	144518685	144520234	Distal Intergenic	-0618	267E-03	492E-02	Ergic3	-4853	Hypo
chr8	78364828	78369082	Intron (NM_001014268/367113, intron 1 of 13)	-0402	268E-03	494E-02	Lrrc1	37017	Hypo
chr10	96485163	96489252	Distal Intergenic	-0386	268E-03	494E-02	Kcnj2	418888	Hypo
chr1	174417679	174419663	Distal Intergenic	-0495	269E-03	494E-02	Ldaf1	-64917	Hypo
chr6	102976053	102980551	Intron (NM_001191818/299186, intron 3 of 8)	-0469	269E-03	495E-02	Dpf3	144942	Hypo
chr3	63462695	63466908	Distal Intergenic	-0403	270E-03	496E-02	RGD1564319	-177707	Hypo
chr1	49484517	49485594	Intron (NM_020093/56816, intron 3 of 11)	-0780	270E-03	496E-02	Prkn	396853	Hypo
chr15	52042563	52043651	Distal Intergenic	-0823	270E-03	496E-02	Serp2	-138598	Hypo
chr1	204806299	204814336	Exon (NM_017060/24913, exon 4 of 5)	-0365	270E-03	496E-02	Plaat3	23575	Hypo
chr9	80113934	80118315	Distal Intergenic	-0390	271E-03	496E-02	Acsl3	-22681	Hypo
chr18	59234491	59238306	Exon (NM_001106139/291549, exon 5 of 15)	-0384	272E-03	498E-02	Oacyl	22137	Hypo
chr2	214290966	214301808	Intron (NM_019276/50555, intron 2 of 5)	-0347	273E-03	499E-02	Ugt8	30852	Hypo

Supplementary Table 3: Significantly hyperacetylated peaks in the cortex

chr	*start*	*end*	*annotation*	*logFC*	*PValue*	*FDR*	*GENE*	*distanceToTSS*	*DAR*
chr20	6567705	6577688	Distal Intergenic	1322	363E-17	293E-12	Armc12	-10171	Hyper
chr16	16774604	16776552	Distal Intergenic	2549	181E-15	730E-11	Sh2d4b	-5517	Hyper
chr20	6478703	6480826	Intron (NM_001012174/361810, intron 5 of 10)	2222	966E-15	260E-10	Tulp1	-54630	Hyper
chr1	77743074	77750549	Promoter (<=1kb)	1253	133E-14	268E-10	Hif3a	0	Hyper
chr5	144929203	144933280	3' UTR	1080	327E-13	440E-09	Xkr8	11915	Hyper
chr2	174775303	174778131	Distal Intergenic	1566	102E-12	103E-08	Adam15	-10205	Hyper
chr19	53843800	53847474	Distal Intergenic	1140	222E-12	179E-08	Kcnk1	-112183	Hyper
chr7	89405329	89407748	3' UTR	1551	251E-12	185E-08	Derl1	19347	Hyper
chr14	78366818	78370820	Promoter (<=1kb)	1206	291E-12	196E-08	Pla2g3	0	Hyper
chr10	81941462	81946671	3' UTR	1014	451E-12	280E-08	Pnpo	-10618	Hyper
chr17	67006179	67007509	Distal Intergenic	1853	487E-12	281E-08	Pfkfb3	-27586	Hyper
chr1	181274085	181278834	Promoter (<=1kb)	1048	675E-12	363E-08	Sult1a1	0	Hyper
chr3	152454773	152456551	Distal Intergenic	1844	111E-11	528E-08	Ada	-31919	Hyper
chr20	27918138	27924970	Distal Intergenic	0904	122E-11	548E-08	Ascc1	-16430	Hyper
chr10	100830392	100834620	Exon (NM_001108304/360658, exon 4 of 16)	1071	181E-11	732E-08	Mrps7	-9071	Hyper
chr10	36138841	36140519	Distal Intergenic	1719	433E-11	143E-07	Jade2	-18756	Hyper
chr8	109175661	109177803	Promoter (<=1kb)	1529	443E-11	143E-07	Lamb2	-606	Hyper
chr2	135691885	135696870	Promoter (<=1kb)	0968	591E-11	184E-07	Mgst2	0	Hyper
chr17	44660163	44663522	Exon (NM_001108415/361251, exon 6 of 22)	1496	675E-11	202E-07	Elmo1	159266	Hyper
chr16	16781160	16783260	Distal Intergenic	1631	938E-11	261E-07	Sh2d4b	-12073	Hyper
chr4	30663907	30665910	Intron (NM_001191861/114483, intron 4 of 6)	1242	103E-10	277E-07	Fam133b	-47884	Hyper
chr20	27904233	27917997	Distal Intergenic	0805	123E-10	310E-07	Ddit4	-10145	Hyper
chr20	6578629	6583407	Distal Intergenic	0836	149E-10	355E-07	Armc12	-4452	Hyper
chr5	160350807	160353304	Distal Intergenic	1245	155E-10	358E-07	Spsb1	17958	Hyper
chr6	31222527	31225042	Distal Intergenic	1032	180E-10	404E-07	Gdf7	-40080	Hyper
chr4	122970884	122977245	Intron (NM_053536/85497, intron 3 of 3)	0962	185E-10	404E-07	Klf15	5077	Hyper
chr12	11669049	11671439	Distal Intergenic	1089	224E-10	476E-07	Actb	5937	Hyper
chr2	174772664	174775005	Distal Intergenic	1705	239E-10	495E-07	Adam15	-7566	Hyper
chr14	79083195	79087529	Promoter (<=1kb)	0947	288E-10	567E-07	Castor1	0	Hyper
chr1	202724790	202725556	Distal Intergenic	1815	300E-10	577E-07	Drap1	8869	Hyper
chr20	6583705	6585793	Promoter (2-3kb)	1414	313E-10	588E-07	Armc12	-2066	Hyper

chr12	42343738	42345809	Intron (NM_001105935/288736, intron 2 of 11)	1656	333E-10	611E-07	Foxn4	3415	Hyper
chr8	12800299	12802377	Intron (NM_138544/191571, intron 6 of 25)	1195	346E-10	620E-07	Mtnr1b	-147562	Hyper
chr1	121317269	121319544	Intron (NM_001025681/308708, intron 9 of 10)	1052	363E-10	629E-07	Synm	44108	Hyper
chr15	45239264	45241340	Exon (NM_001007622/290354, exon 7 of 10)	1310	383E-10	644E-07	C15h8orf58	-6010	Hyper
chr2	135703527	135705566	Intron (NM_001106430/295037, intron 2 of 4)	1424	487E-10	802E-07	Mgst2	9971	Hyper
chr12	41468476	41472598	3' UTR	0954	622E-10	985E-07	Unc119b	-6210	Hyper
chr17	44675455	44684397	Intron (NM_001108415/361251, intron 4 of 21)	0982	679E-10	105E-06	Elmo1	138391	Hyper
chr6	130977361	130979541	Distal Intergenic	1150	822E-10	122E-06	LOC102548399	-6146	Hyper
chr8	42807771	42815785	Intron (NM_001014089/315591, intron 7 of 7)	0956	827E-10	122E-06	Tecta	-28064	Hyper
chr8	109946950	109950279	Intron (NM_001024278/367171, intron 1 of 16)	0922	831E-10	122E-06	Map4	21330	Hyper
chr6	103174964	103177290	Exon (NM_001108716/362762, exon 8 of 14)	1109	961E-10	139E-06	Dcaf4	20097	Hyper
chr20	27864831	27868535	Distal Intergenic	1104	995E-10	141E-06	Ddit4	25553	Hyper
chr4	141066331	141067804	Distal Intergenic	1651	106E-09	148E-06	Setmar	20244	Hyper
chr1	29592495	29593508	Exon (NM_001039722/664630, exon 2 of 12)	1268	109E-09	148E-06	Slc6a19	6290	Hyper
chr20	7152274	7154884	Promoter (2-3kb)	0938	119E-09	155E-06	Cdkn1a	2988	Hyper
chrX	10645562	10646289	Distal Intergenic	1975	127E-09	162E-06	Bcor	-41443	Hyper
chr11	76018304	76020992	Intron (NM_001013864/288010, intron 6 of 11)	1296	150E-09	187E-06	Mir28	-42696	Hyper
chr2	135699588	135703270	Intron (NM_001106430/295037, intron 2 of 4)	1176	155E-09	188E-06	Mgst2	6032	Hyper
chr5	120959060	120960882	Distal Intergenic	1257	156E-09	188E-06	Usp24	-119880	Hyper
chr6	105195116	105196161	Distal Intergenic	1641	161E-09	191E-06	Fos	73946	Hyper
chr5	140187540	140190153	Distal Intergenic	1030	192E-09	224E-06	LOC682102	135662	Hyper
chr10	50907410	50909108	Intron (NM_001191922/497926, intron 3 of 4)	1484	214E-09	243E-06	Shisa6	282265	Hyper
chr11	65729666	65732362	Distal Intergenic	0944	217E-09	243E-06	Adcy5	-111565	Hyper
chr2	182135469	182137847	3' UTR	0991	225E-09	247E-06	Celf3	19405	Hyper
chr9	57198431	57201256	Intron (NM_053456/84587, intron 5 of 5)	1238	229E-09	247E-06	Plcl1	296858	Hyper
chr11	69909004	69910706	Distal Intergenic	1364	229E-09	247E-06	Fam43a	220013	Hyper
chr12	11688015	11690744	Distal Intergenic	0852	257E-09	273E-06	Actb	24903	Hyper
chr19	49776587	49779012	Distal Intergenic	1049	282E-09	287E-06	Jph3	-15311	Hyper
chr4	158046348	158047406	Distal Intergenic	1514	314E-09	313E-06	Cd27	-10756	Hyper
chr20	45737593	45740802	Intron (NM_001106395/294515, intron 2 of 3)	0801	322E-09	317E-06	Foxo3	23804	Hyper
chr12	44759971	44761998	Distal Intergenic	1007	387E-09	364E-06	Miat	345351	Hyper
chr7	120335112	120338413	Exon (NM_001106789/300146, exon 9 of 25)	1160	404E-09	369E-06	Sbf1	46489	Hyper
chr2	241338171	241340781	Distal Intergenic	1136	407E-09	369E-06	Zzz3	-18168	Hyper

chr9	92026060	92029541	Exon (NM_031678/63840, exon 9 of 23)	0969	411E-09	369E-06	Per2	19918	Hyper
chr10	64028479	64037093	Intron (NM_001382488/108348076, intron 1 of 17)	0709	552E-09	470E-06	Ksr1	60983	Hyper
chr8	58475200	58478268	Exon (NM_001014091/315712, exon 10 of 20)	0853	570E-09	480E-06	Ccdc33	39317	Hyper
chr10	81939122	81940660	Distal Intergenic	1154	598E-09	493E-06	Pnpo	-8278	Hyper
chr1	196514111	196517489	Promoter (1-2kb)	1284	863E-09	664E-06	Gatd1	-1611	Hyper
chr5	142522047	142524371	Promoter (<=1kb)	1210	891E-09	679E-06	Tinagl1	0	Hyper
chr1	85500830	85501475	Intron (NM_001191623/308492, intron 20 of 32)	1949	913E-09	689E-06	Ovol3	-12238	Hyper
chr12	30952004	30955313	Intron (NM_001134536/304458, intron 2 of 7)	0744	929E-09	694E-06	Tmem132b	42996	Hyper
chr3	17071926	17074105	Intron (NM_001271232/362118, intron 7 of 9)	1315	967E-09	717E-06	Mvb12b	118553	Hyper
chr1	117379503	117387944	Distal Intergenic	0637	112E-08	813E-06	Klf13	148682	Hyper
chr15	13065342	13066054	Intron (NM_134356/171357, intron 5 of 32)	1848	113E-08	815E-06	Cep15	-230294	Hyper
chr7	112872733	112876687	Intron (NM_001126269/300083, intron 1 of 8)	0835	119E-08	835E-06	Slc25a17	10327	Hyper
chr8	43238855	43241752	Distal Intergenic	0968	147E-08	971E-06	Grik4	-33778	Hyper
chr13	83659918	83663623	Exon (NM_001011907/289218, exon 3 of 13)	0891	149E-08	971E-06	Ndufs2	3322	Hyper
chr5	161337290	161341694	Downstream (<=300bp)	0794	153E-08	980E-06	Errfi1	13285	Hyper
chr12	21826377	21827765	Distal Intergenic	1226	159E-08	100E-05	Mettl27	-60058	Hyper
chr11	78199090	78200129	Exon (NM_001012016/303830, exon 8 of 9)	1432	165E-08	102E-05	Crygs	-7041	Hyper
chr13	45553850	45555732	Distal Intergenic	0999	166E-08	102E-05	Chit1	-10109	Hyper
chr17	44719131	44720884	Intron (NM_001108415/361251, intron 2 of 21)	1422	174E-08	106E-05	Elmo1	101904	Hyper
chr6	118303353	118304314	Distal Intergenic	1604	189E-08	114E-05	Cpg1	-7906	Hyper
chr7	57303688	57307730	Distal Intergenic	0870	191E-08	114E-05	Kics2	19466	Hyper
chr2	135683971	135689758	Distal Intergenic	0842	200E-08	119E-05	Mgst2	-3798	Hyper
chr5	63916200	63917373	Distal Intergenic	1353	230E-08	133E-05	Aldob	-14114	Hyper
chr17	44691107	44693636	Intron (NM_001108415/361251, intron 4 of 21)	0988	232E-08	133E-05	Elmo1	129152	Hyper
chr7	25798842	25799508	Distal Intergenic	1659	233E-08	133E-05	Tmpo	-131131	Hyper
chr2	82591144	82594334	3' UTR	1197	247E-08	138E-05	Cct5	8569	Hyper
chr6	24101223	24103637	Exon (NM_138898/192259, exon 50 of 58)	1043	258E-08	143E-05	Plb1	106480	Hyper
chr18	3374422	3378325	Exon (NM_001009638/291784, exon 13 of 20)	1044	261E-08	144E-05	Rmc1	14574	Hyper
chr14	95031735	95034424	Distal Intergenic	0726	278E-08	152E-05	Lgalsl	-27236	Hyper
chr10	57187430	57189888	Distal Intergenic	0906	281E-08	152E-05	Spns3	-18669	Hyper
chr1	117501022	117519037	3' UTR	0593	314E-08	167E-05	Klf13	17589	Hyper
chr13	30092006	30093413	Distal Intergenic	1571	339E-08	175E-05	Gli2	-89189	Hyper
chr1	197623883	197626555	Intron (NM_001025420/361680, intron 1 of 10)	0891	339E-08	175E-05	Lsp1	9196	Hyper

chr4	83549514	83553026	Promoter (<=1kb)	0746	341E-08	175E-05	Wipf3	0	Hyper
chr2	187607615	187608545	Distal Intergenic	1191	348E-08	177E-05	Gdap2	79064	Hyper
chr12	31752010	31754120	Distal Intergenic	1067	350E-08	177E-05	Ccdc92	-42564	Hyper
chr10	66523054	66524602	Intron (NM_001034014/25364, intron 1 of 9)	1089	352E-08	177E-05	Mir6327	73881	Hyper
chr9	102312142	102314486	Distal Intergenic	0846	377E-08	186E-05	Efna5	280648	Hyper
chr2	140313657	140315950	Distal Intergenic	1024	402E-08	192E-05	Mab21l1	370161	Hyper
chr17	67001287	67003000	Distal Intergenic	0769	402E-08	192E-05	Pfkfb3	-32095	Hyper
chr2	11330134	11331979	Distal Intergenic	0951	415E-08	195E-05	Arrdc3	192434	Hyper
chr11	65587817	65590894	Intron (NM_022600/64532, intron 1 of 20)	0882	441E-08	206E-05	Adcy5	27207	Hyper
chr11	30088877	30092492	Distal Intergenic	0600	465E-08	215E-05	Urb1	-23650	Hyper
chr17	44672452	44673412	Intron (NM_001108415/361251, intron 4 of 21)	1560	471E-08	215E-05	Elmo1	149376	Hyper
chr13	46147360	46149117	Distal Intergenic	1104	492E-08	222E-05	Syt2	-36165	Hyper
chr7	107449098	107451164	Intron (NM_001134865/300032, intron 9 of 11)	0928	508E-08	228E-05	Rhpn1	57114	Hyper
chr12	11691365	11696177	Distal Intergenic	0652	530E-08	234E-05	Actb	28253	Hyper
chr16	50220734	50224106	Distal Intergenic	0887	533E-08	234E-05	Frg1	-701697	Hyper
chr14	79069660	79072599	Intron (NM_001015022/360968, intron 1 of 8)	0829	539E-08	235E-05	Castor1	-13537	Hyper
chr1	155760977	155762521	Distal Intergenic	1233	553E-08	240E-05	Stard10	38279	Hyper
chr19	54717798	54721507	Distal Intergenic	0756	555E-08	240E-05	Tomm20	213681	Hyper
chr1	85501828	85502137	Exon (NM_001191623/308492, exon 20 of 33)	2663	621E-08	257E-05	Ovol3	-13236	Hyper
chr2	241367547	241369690	Intron (NM_001134549/310958, intron 1 of 10)	0940	677E-08	273E-05	Zzz3	8598	Hyper
chr3	18977137	18981698	Intron (NM_138710/192126, intron 1 of 15)	0907	679E-08	273E-05	Dab2ip	61847	Hyper
chr11	82288376	82290569	Distal Intergenic	1169	681E-08	273E-05	Cldn5	-74128	Hyper
chr16	32807421	32808970	Distal Intergenic	0933	688E-08	275E-05	Scrg1	-19122	Hyper
chr19	50285577	50286187	Distal Intergenic	1541	702E-08	279E-05	Zfpm1	-48495	Hyper
chr7	110912354	110913629	Promoter (<=1kb)	1097	715E-08	283E-05	Maff	0	Hyper
chr17	23126645	23128953	Distal Intergenic	0800	728E-08	287E-05	Tmem170b	40610	Hyper
chr6	119769626	119772507	Distal Intergenic	0740	747E-08	289E-05	Ttc7b	-64439	Hyper
chr5	145440155	145441303	Promoter (<=1kb)	1245	749E-08	289E-05	Map3k6	0	Hyper
chr1	84067503	84075461	Promoter (1-2kb)	0611	752E-08	289E-05	Rinl	-1804	Hyper
chr7	65478679	65481552	Distal Intergenic	0902	789E-08	302E-05	Laptm4b	44154	Hyper
chr4	120187968	120191425	Promoter (<=1kb)	0774	802E-08	304E-05	Efcc1	0	Hyper
chr17	20587676	20590445	Distal Intergenic	0798	821E-08	310E-05	LOC690414	-24048	Hyper
chr20	18755417	18757391	Exon (NM_031805/361833, exon 11 of 45)	0931	837E-08	312E-05	Ank3	172738	Hyper

chr7	105634529	105655840	Promoter (<=1kb)	0570	837E-08	312E-05	Ptp4a3	0	Hyper
chr17	33267886	33268401	Distal Intergenic	1747	917E-08	333E-05	Exoc2	-237921	Hyper
chr9	90176842	90185617	Promoter (2-3kb)	0648	923E-08	334E-05	Agap1	-2408	Hyper
chr10	64009689	64014823	Intron (NM_001382488/108348076, intron 2 of 17)	0656	959E-08	342E-05	Lgals9	-79644	Hyper
chr2	92012807	92014294	Distal Intergenic	1120	965E-08	342E-05	Pag1	-42922	Hyper
chr20	45372240	45374702	Intron (NM_001106396/294518, intron 1 of 7)	0898	987E-08	345E-05	LOC499469	36443	Hyper
chr5	163983140	163985068	Intron (NM_001101014/687031, intron 1 of 5)	1040	101E-07	351E-05	Ajap1	35249	Hyper
chr12	4522663	4524321	Exon (NM_001170398/304244, exon 52 of 61)	0973	102E-07	351E-05	Tgap1-ps1	-173243	Hyper
chr10	81917755	81925233	3' UTR	0663	102E-07	351E-05	Pnpo	5611	Hyper
chr5	122142590	122143843	Intron (NM_199383/298312, intron 9 of 9)	1305	111E-07	376E-05	Ndc1	-12152	Hyper
chr9	82537913	82540253	Distal Intergenic	0935	111E-07	376E-05	Cul3	-886400	Hyper
chr8	49059724	49087765	Exon (NM_001013181/353227, exon 3 of 7)	0541	111E-07	376E-05	Zbtb16	89246	Hyper
chr9	105279817	105280629	Intron (NM_145094/246324, intron 5 of 6)	1391	115E-07	386E-05	Txndc2	-44735	Hyper
chr19	47724221	47726008	Intron (NM_133581/171112, intron 1 of 6)	1076	119E-07	394E-05	Wfdc1	3793	Hyper
chr6	39399895	39401369	Distal Intergenic	1111	125E-07	406E-05	Lpin1	-19719	Hyper
chr1	120851334	120853563	3' UTR	0819	135E-07	433E-05	Mef2a	128397	Hyper
chr9	57191880	57193918	Intron (NM_053456/84587, intron 3 of 5)	1186	142E-07	448E-05	Plcl1	290307	Hyper
chr10	102354782	102358127	Exon (NM_001108309/360668, exon 9 of 17)	0665	146E-07	456E-05	Sec14l1	34862	Hyper
chr6	119776156	119782760	Distal Intergenic	0638	147E-07	456E-05	Ttc7b	-70969	Hyper
chr12	35302405	35304789	Distal Intergenic	0983	148E-07	457E-05	Rpl6	46252	Hyper
chr7	9049255	9052590	3' UTR	0662	149E-07	457E-05	Mknk2	9484	Hyper
chr1	163878229	163879582	Distal Intergenic	1108	150E-07	460E-05	Nrip3	-63193	Hyper
chr17	14368383	14373373	Exon (NM_001082409/361219, exon 3 of 10)	0676	153E-07	464E-05	Fbxw17	8829	Hyper
chr1	117519791	117526185	Intron (NM_001109147/499171, intron 1 of 1)	0660	153E-07	464E-05	Klf13	10441	Hyper
chr7	33771384	33775180	Intron (NM_053311/29598, intron 1 of 21)	0704	155E-07	465E-05	Atp2b1	35409	Hyper
chr3	165403129	165405688	Intron (NM_214459/362284, intron 7 of 11)	0973	157E-07	469E-05	Phactr3	59855	Hyper
chr10	36178389	36183091	Distal Intergenic	0574	170E-07	497E-05	Cdkn2aipnl	-48043	Hyper
chr1	56376019	56376654	Exon (NM_001107466/308218, exon 8 of 10)	1608	170E-07	497E-05	Fam120b	36890	Hyper
chr17	85119234	85121464	Exon (NM_024397/79249, exon 4 of 11)	0895	170E-07	497E-05	Abi1	58289	Hyper
chr16	6018720	6022859	Intron (NM_198774/361110, intron 1 of 7)	0872	172E-07	498E-05	Stimate	5328	Hyper
chr13	100776643	100778504	Intron (NM_172042/170899, intron 6 of 6)	0983	181E-07	518E-05	Kcnk2	128877	Hyper
chr2	33791950	33795291	Distal Intergenic	0736	187E-07	533E-05	Cd180	-60700	Hyper
chr19	51203602	51205864	Promoter (<=1kb)	0906	191E-07	539E-05	Sult5a1	0	Hyper

chr8	43514511	43516736	Intron (NM_001105745/116544, intron 5 of 12)	0957	193E-07	542E-05	Tlcd5	-28223	Hyper
chr10	61156180	61156794	Intron (NM_001108285/360577, intron 1 of 7)	1532	200E-07	556E-05	Mrm3	60228	Hyper
chr1	196551895	196556454	Promoter (<=1kb)	0565	204E-07	563E-05	Pnpla2	0	Hyper
chr2	30499554	30502754	Distal Intergenic	0632	207E-07	569E-05	Tnpo1	-230169	Hyper
chr1	175427707	175429994	Exon (NM_012947/25435, exon 2 of 17)	0831	208E-07	571E-05	Eef2k	16089	Hyper
chr3	154642047	154646343	Distal Intergenic	0577	215E-07	586E-05	Zmynd8	-14881	Hyper
chr5	75890181	75892054	Distal Intergenic	1045	215E-07	586E-05	Wdr31	25339	Hyper
chr19	47717653	47723096	Promoter (<=1kb)	0897	217E-07	588E-05	Wfdc1	0	Hyper
chr7	98715120	98719808	5' UTR	0800	222E-07	599E-05	Ndrg1	6061	Hyper
chr14	30602776	30604190	Distal Intergenic	1360	230E-07	616E-05	Igfbp7	-133224	Hyper
chr8	108290400	108295299	Exon (NM_031035/81664, exon 2 of 9)	0590	233E-07	621E-05	Sema3b	-11386	Hyper
chr4	115807151	115808135	Intron (NM_212512/297386, intron 26 of 26)	1079	238E-07	632E-05	Mthfd2	14524	Hyper
chr12	11791822	11793181	Intron (NM_001107123/304302, intron 4 of 29)	1083	243E-07	637E-05	Tnrc18	36430	Hyper
chr5	57982507	57983726	Distal Intergenic	1181	251E-07	649E-05	Or13j1	-20178	Hyper
chr6	100462643	100470670	Distal Intergenic	0575	253E-07	654E-05	Plekhd1	121988	Hyper
chr8	57951075	57952287	Exon (NM_001004081/300741, exon 5 of 8)	1136	255E-07	657E-05	Mpi	3826	Hyper
chr10	66234476	66235865	Intron (NM_001034014/25364, intron 1 of 9)	0924	259E-07	662E-05	Asic2	-96097	Hyper
chr6	72835366	72839125	Distal Intergenic	0740	270E-07	680E-05	Nfkbia	22816	Hyper
chr3	165413502	165419023	Exon (NM_214459/362284, exon 8 of 12)	0732	273E-07	683E-05	Phactr3	70228	Hyper
chr12	21828018	21829109	Distal Intergenic	1447	286E-07	707E-05	Mettl27	-61699	Hyper
chr2	144763160	144765999	Intron (NM_001191566/282635, intron 1 of 7)	0716	290E-07	713E-05	Mbnl1	65327	Hyper
chr17	61158264	61160574	Intron (NM_001107361/307070, intron 12 of 18)	1009	312E-07	757E-05	Larp4b	56782	Hyper
chr5	101633756	101635773	Intron (NM_031743/84550, intron 1 of 9)	0853	324E-07	773E-05	Slc24a2	103564	Hyper
chr16	7227198	7229743	Distal Intergenic	1002	332E-07	784E-05	Oxnad1	-63727	Hyper
chr10	60461438	60464759	Exon (NM_017231/29525, exon 8 of 12)	0709	332E-07	784E-05	Inpp5k	-11152	Hyper
chr1	46731424	46735935	Intron (NM_001109137/499016, intron 1 of 13)	0620	337E-07	790E-05	Tulp4	20689	Hyper
chr6	105194001	105194932	Distal Intergenic	1080	338E-07	790E-05	Fos	72831	Hyper
chr10	29100618	29103913	Promoter (<=1kb)	0874	338E-07	790E-05	Ebf1	63	Hyper
chr3	15004972	15008677	Intron (NM_001100850/311860, intron 1 of 10)	0644	356E-07	814E-05	Abl1	25119	Hyper
chr7	113782843	113784967	Distal Intergenic	0769	366E-07	828E-05	Septin3	-4886	Hyper
chr7	111493661	111498705	Distal Intergenic	0624	366E-07	828E-05	Cbx7	-15688	Hyper
chr20	10020597	10021518	Distal Intergenic	1345	368E-07	828E-05	Sik1	-61658	Hyper
chr5	151338318	151342973	3' UTR	0683	375E-07	834E-05	Nbl1	-8370	Hyper

chr1	197521604	197525388	Distal Intergenic	0666	382E-07	846E-05	Ctsd	13955	Hyper
chr12	33512614	33515187	Distal Intergenic	0751	397E-07	869E-05	Tmem120b	-25357	Hyper
chr1	56375150	56375894	Exon (NM_001107466/308218, exon 7 of 10)	1348	404E-07	879E-05	Fam120b	36021	Hyper
chr12	35773697	35774246	Promoter (1-2kb)	1858	414E-07	895E-05	Oas1k	-1987	Hyper
chr3	119193582	119197752	Intron (NM_012631/24686, intron 2 of 2)	0620	416E-07	896E-05	Prnp	7414	Hyper
chr19	48377431	48382582	Distal Intergenic	0668	420E-07	899E-05	6430548M08Rikl	179222	Hyper
chr6	95577015	95577581	Intron (NM_172034/64511, intron 4 of 11)	1601	420E-07	899E-05	Fntb	40428	Hyper
chr4	63542540	63544001	Intron (NM_001009709/362334, intron 1 of 1)	0943	422E-07	899E-05	Tmem140	6063	Hyper
chr14	76562152	76568218	Promoter (<=1kb)	0581	428E-07	909E-05	Mxd4	0	Hyper
chr8	46504786	46507165	Exon (NM_001271216/684112, exon 16 of 25)	0796	434E-07	913E-05	Apoa1	-20086	Hyper
chr12	19023520	19025608	Intron (NM_001107131/304375, intron 1 of 11)	0801	436E-07	913E-05	Agfg2	11262	Hyper
chr3	119935948	119936829	Distal Intergenic	1128	442E-07	919E-05	Gpcpd1	-103495	Hyper
chr3	13945627	13951030	Distal Intergenic	0535	444E-07	919E-05	C3h9orf50	135948	Hyper
chr5	160437876	160440565	3' UTR	0612	453E-07	931E-05	H6pd	29606	Hyper
chr5	146268128	146268416	Distal Intergenic	2770	455E-07	933E-05	Lin28a	-24006	Hyper
chr2	82594643	82595545	Exon (NM_001004078/294864, exon 6 of 11)	1696	478E-07	970E-05	Cct5	7358	Hyper
chr19	51308485	51314156	Exon (NM_001108455/361435, exon 30 of 42)	0767	488E-07	983E-05	Vps9d1	-17871	Hyper
chr20	28012242	28016012	Intron (NM_001007632/294512, intron 9 of 10)	0612	488E-07	983E-05	Spock2	-21580	Hyper
chr1	69358667	69359273	Promoter (<=1kb)	1613	491E-07	986E-05	Eps8l1	0	Hyper
chr6	28281360	28285742	Intron (NM_001106713/298867, intron 2 of 13)	0598	499E-07	999E-05	Klhl29	104905	Hyper
chr19	54741643	54743329	Distal Intergenic	0771	500E-07	999E-05	Tomm20	191859	Hyper
chr6	95576241	95576813	Intron (NM_172034/64511, intron 4 of 11)	1373	506E-07	101E-04	Fntb	39654	Hyper
chr10	103524411	103525243	5' UTR	1287	509E-07	101E-04	Usp36	3634	Hyper
chr10	13665333	13673576	Promoter (<=1kb)	0642	510E-07	101E-04	Nherf2	0	Hyper
chr7	69741366	69744311	Distal Intergenic	0833	514E-07	102E-04	Azin1	-59788	Hyper
chr5	135302965	135306426	Intron (NM_001108676/362586, intron 1 of 10)	0676	520E-07	103E-04	Trit1	7631	Hyper
chr10	71406204	71409489	Promoter (<=1kb)	0724	527E-07	104E-04	Mir21	-856	Hyper
chr5	15014389	15016589	Promoter (<=1kb)	1019	534E-07	104E-04	Sox17	-142	Hyper
chr19	50518847	50520992	Promoter (2-3kb)	0819	542E-07	105E-04	Snai3	2494	Hyper
chr16	6425480	6428679	Downstream (<=300bp)	0793	560E-07	108E-04	Sema3g	11891	Hyper
chr1	187461846	187466236	Intron (NM_001009706/361663, intron 6 of 6)	0601	563E-07	108E-04	Lhpp	58395	Hyper
chr14	83240731	83242763	Intron (NM_001170459/360980, intron 14 of 28)	0923	564E-07	108E-04	Tns3	101375	Hyper
chr6	127612502	127614440	Promoter (<=1kb)	1070	579E-07	111E-04	Degs2	0	Hyper

chr2	30871222	30880921	Intron (NM_019217/29456, intron 2 of 6)	0521	585E-07	111E-04	Map1b	29396	Hyper
chr17	44724389	44726811	Intron (NM_001108415/361251, intron 2 of 21)	1012	585E-07	111E-04	Elmo1	95977	Hyper
chr1	46433754	46437753	Intron (NM_001127637/683687, intron 1 of 17)	0695	603E-07	114E-04	Snx9	8786	Hyper
chr19	39758989	39763812	Intron (NM_199378/292027, intron 5 of 6)	0650	609E-07	115E-04	Bcar1	-56706	Hyper
chr1	205881978	205886632	Promoter (<=1kb)	0673	621E-07	116E-04	Ahnak	0	Hyper
chr16	76529541	76531831	Exon (NM_053951/117020, exon 9 of 31)	0850	625E-07	116E-04	F7	-28953	Hyper
chr8	65671924	65674654	Intron (NM_001372159/315759, intron 2 of 13)	0762	660E-07	121E-04	Igdcc3	10759	Hyper
chr7	25798049	25798655	Distal Intergenic	1361	668E-07	123E-04	Tmpo	-130338	Hyper
chr19	24445225	24448223	Intron (NM_017175/29355, intron 1 of 21)	0724	676E-07	123E-04	Pkn1	3095	Hyper
chr10	60798662	60800020	Promoter (1-2kb)	0994	682E-07	124E-04	Rph3al	1863	Hyper
chr10	36546235	36548029	Exon (NM_031353/83529, exon 4 of 9)	0845	700E-07	126E-04	Vdac1	13929	Hyper
chr10	44577848	44581142	Distal Intergenic	0858	703E-07	126E-04	Flcn	26607	Hyper
chr7	63861224	63862941	Intron (NM_001012113/314553, intron 2 of 12)	0839	710E-07	127E-04	Ptdss1	16207	Hyper
chr19	20374913	20375563	Distal Intergenic	1079	721E-07	128E-04	Siah1	-3345	Hyper
chr12	35176386	35184798	3' UTR	0504	737E-07	130E-04	Trafd1	10780	Hyper
chr12	37984581	37985703	Distal Intergenic	1134	745E-07	131E-04	Spring1	343435	Hyper
chr14	41615892	41619116	Distal Intergenic	0680	761E-07	133E-04	Uchl1	-120302	Hyper
chr8	65514221	65515471	Exon (NM_001106831/300783, exon 6 of 11)	1324	776E-07	135E-04	Hacd3	23036	Hyper
chr2	30770168	30771622	Intron (NM_001108543/361883, intron 4 of 4)	0815	791E-07	136E-04	Mrps27	30135	Hyper
chr19	429103	430321	3' UTR	0990	792E-07	136E-04	Car7	8146	Hyper
chr4	11740335	11746816	Distal Intergenic	0490	793E-07	136E-04	Kmt2e	-12962	Hyper
chr9	13488166	13490453	Promoter (1-2kb)	0579	795E-07	136E-04	Taf8	-1484	Hyper
chr18	32310663	32312552	Intron (NM_001172155/291618, intron 1 of 1)	0834	822E-07	139E-04	Kctd16	142329	Hyper
chr6	95577843	95578576	Intron (NM_172034/64511, intron 4 of 11)	1580	829E-07	139E-04	Fntb	41256	Hyper
chr5	161720636	161722617	Intron (NM_001195559/362665, intron 6 of 23)	0957	830E-07	139E-04	Vamp3	-211932	Hyper
chr19	48567397	48571830	Distal Intergenic	0655	840E-07	141E-04	Gins2	67509	Hyper
chr3	119937044	119945941	Distal Intergenic	0533	865E-07	145E-04	Chgb	-97883	Hyper
chr11	82286449	82287533	Distal Intergenic	1289	870E-07	145E-04	Cldn5	-72201	Hyper
chr10	16724019	16724638	Distal Intergenic	1378	878E-07	146E-04	Dusp1	43530	Hyper
chr12	35776155	35776981	Promoter (2-3kb)	1519	883E-07	146E-04	Oas3	-2137	Hyper
chr13	44695125	44697537	Distal Intergenic	0782	887E-07	146E-04	Golt1a	-62843	Hyper
chr3	119946916	119949601	Distal Intergenic	0792	893E-07	147E-04	Chgb	-94223	Hyper
chr7	67594141	67595582	Distal Intergenic	0961	894E-07	147E-04	Ankrd46	72587	Hyper

chr10	102333327	102340063	Intron (NM_001108309/360668, intron 2 of 16)	0558	903E-07	147E-04	Sec14l1	13407	Hyper
chr4	33556527	33561132	Distal Intergenic	0712	904E-07	147E-04	Pdk4	40666	Hyper
chr15	44868980	44872228	3' UTR	0747	932E-07	151E-04	Rhobtb2	16208	Hyper
chr4	10813402	10814173	Promoter (<=1kb)	1304	958E-07	154E-04	Nos3	0	Hyper
chr14	1071840	1075060	3' UTR	0670	967E-07	154E-04	Dgkq	12670	Hyper
chr3	10747998	10749456	Intron (NM_001106563/296603, intron 1 of 27)	1151	989E-07	157E-04	Vav2	4596	Hyper
chr7	98831879	98832882	Distal Intergenic	1343	999E-07	158E-04	St3gal1	22771	Hyper
chr10	41518802	41519778	Intron (NM_031608/50592, intron 15 of 15)	1138	100E-06	158E-04	Mfap3	-177010	Hyper
chr1	69322287	69324472	Promoter (2-3kb)	0764	100E-06	158E-04	Ppp1r12c	2032	Hyper
chr7	46659090	46662514	Intron (NM_001309455/314824, intron 2 of 23)	0644	101E-06	159E-04	Osbpl8	62107	Hyper
chr10	52346660	52358481	Exon (NM_053484/85246, exon 5 of 14)	0540	103E-06	162E-04	Rcvrn	-30225	Hyper
chr4	158041387	158042724	Distal Intergenic	1289	105E-06	164E-04	Cd27	-5795	Hyper
chr5	14694027	14694822	Intron (NM_013006/25514, intron 2 of 8)	1168	106E-06	165E-04	Lypla1	13876	Hyper
chr12	34572690	34576562	Intron (NM_001271380/288665, intron 2 of 22)	0675	107E-06	167E-04	Cux2	51731	Hyper
chr9	105271978	105273238	Intron (NM_145094/246324, intron 5 of 6)	0982	108E-06	168E-04	Txndc2	-36896	Hyper
chr1	76623228	76627130	Promoter (<=1kb)	0616	112E-06	172E-04	Rpl9	0	Hyper
chr6	131559848	131560589	Distal Intergenic	1426	112E-06	172E-04	Tmem179	19716	Hyper
chr12	34251903	34263970	3' UTR	0492	114E-06	173E-04	Pptc7	24369	Hyper
chr10	41517348	41518649	Intron (NM_031608/50592, intron 15 of 15)	1157	119E-06	179E-04	Mfap3	-178139	Hyper
chr19	20389784	20391430	Intron (NM_080905/140941, intron 1 of 1)	0962	120E-06	179E-04	Siah1	10876	Hyper
chr17	3803945	3804600	Promoter (<=1kb)	1589	120E-06	179E-04	Ctla2a	101	Hyper
chr1	46505815	46506759	Exon (NM_001127637/683687, exon 17 of 18)	1275	121E-06	180E-04	Synj2	-11950	Hyper
chr1	117563433	117569869	Distal Intergenic	0602	123E-06	182E-04	Klf13	-26807	Hyper
chr20	27937818	27939568	Promoter (1-2kb)	0840	123E-06	182E-04	Ascc1	-1832	Hyper
chr20	4183143	4184784	Promoter (<=1kb)	0893	123E-06	182E-04	Notch4	0	Hyper
chr13	44863548	44864586	Intron (NM_001105952/289026, intron 1 of 13)	1194	124E-06	182E-04	Sox13	10936	Hyper
chr5	140778388	140781781	Distal Intergenic	0614	126E-06	184E-04	Hmgb4	-161666	Hyper
chr10	87464616	87466115	Distal Intergenic	0970	127E-06	185E-04	Grn	76944	Hyper
chr11	66420461	66422552	Intron (NM_032062/84009, intron 5 of 59)	0794	127E-06	186E-04	Kalrn	165492	Hyper
chr10	91197455	91202861	Promoter (<=1kb)	0484	131E-06	190E-04	Smarcd2	0	Hyper
chr3	156571191	156571624	Distal Intergenic	1552	133E-06	191E-04	Ptpn1	-67187	Hyper
chr7	105631515	105634350	Distal Intergenic	0939	137E-06	197E-04	Ptp4a3	-20756	Hyper
chr14	43111839	43114409	Distal Intergenic	0813	144E-06	205E-04	Wdr19	-5551	Hyper

chr7	111545080	111548548	Exon (NM_031524/24628, exon 3 of 7)	0694	146E-06	207E-04	Pdgfb	9436	Hyper
chr4	160890667	160892347	Promoter (2-3kb)	0914	151E-06	212E-04	Tspan9	-2601	Hyper
chr2	174763381	174766711	Promoter (<=1kb)	0618	152E-06	213E-04	Adam15	0	Hyper
chr12	6148767	6150650	Intron (NM_001006956/288449, intron 9 of 10)	0847	154E-06	215E-04	Katnal1	45881	Hyper
chr10	86314574	86316739	Exon (NM_013098/25634, exon 4 of 5)	0725	154E-06	215E-04	G6pc1	7174	Hyper
chr11	30312332	30314307	5' UTR	0812	155E-06	215E-04	RGD1562726	10997	Hyper
chr7	120324271	120327709	Exon (NM_001106789/300146, exon 5 of 25)	0844	156E-06	216E-04	Ppp6r2	38865	Hyper
chr10	14615543	14617292	Distal Intergenic	1107	156E-06	216E-04	Sox8	-25725	Hyper
chr5	131808001	131815571	Promoter (<=1kb)	0581	158E-06	217E-04	Ptprf	0	Hyper
chr5	102649779	102651872	Distal Intergenic	0678	160E-06	219E-04	Hacd4	263438	Hyper
chr20	27861146	27862681	Distal Intergenic	0878	161E-06	220E-04	Ddit4	31407	Hyper
chr6	26398968	26402406	Intron (NM_053486/85248, intron 5 of 7)	0697	163E-06	222E-04	Kif3c	31876	Hyper
chr12	31439746	31441640	Distal Intergenic	0796	165E-06	224E-04	Ncor2	-24948	Hyper
chr1	160090558	160093028	Promoter (1-2kb)	0761	166E-06	225E-04	Ilk	1670	Hyper
chr5	46199235	46201387	Distal Intergenic	0743	167E-06	226E-04	Map3k7	-156544	Hyper
chr1	202801446	202805304	Downstream (<=300bp)	0588	171E-06	230E-04	Cfl1	3647	Hyper
chr10	64137901	64141148	Distal Intergenic	0662	171E-06	230E-04	Ksr1	-39825	Hyper
chr17	23097396	23100203	Distal Intergenic	0797	175E-06	233E-04	Tmem170b	69360	Hyper
chr2	187608653	187609362	Distal Intergenic	1247	178E-06	236E-04	Gdap2	80102	Hyper
chr15	54646179	54646966	Distal Intergenic	1294	179E-06	238E-04	Rgcc	28354	Hyper
chr19	20373801	20374819	Distal Intergenic	0820	180E-06	238E-04	Siah1	-4089	Hyper
chr18	5338387	5340094	Distal Intergenic	0730	180E-06	238E-04	Zfp521	-271161	Hyper
chr12	35374037	35379631	Intron (NM_001177593/25622, intron 1 of 15)	0577	189E-06	246E-04	Ptpn11	8601	Hyper
chr10	88725788	88727001	Distal Intergenic	0949	190E-06	247E-04	Wnt3	45540	Hyper
chr5	163932225	163934774	Intron (NM_001101014/687031, intron 2 of 5)	0701	194E-06	252E-04	Ajap1	85543	Hyper
chr10	103512481	103524334	Exon (NM_001107069/303700, exon 4 of 20)	0533	196E-06	254E-04	Usp36	4543	Hyper
chr6	95575079	95575996	Intron (NM_172034/64511, intron 4 of 11)	1128	198E-06	255E-04	Fntb	38492	Hyper
chr5	101528471	101530178	Intron (NM_031743/84550, intron 6 of 9)	0838	198E-06	255E-04	Acer2	136586	Hyper
chr17	44700613	44701711	Intron (NM_001108415/361251, intron 4 of 21)	1297	203E-06	260E-04	Elmo1	121077	Hyper
chr6	72857372	72863194	Promoter (<=1kb)	0550	204E-06	260E-04	Nfkbia	0	Hyper
chr6	121808167	121809324	Intron (NM_001191985/500709, intron 2 of 9)	0950	205E-06	260E-04	Itpk1	34714	Hyper
chr2	183669910	183672458	Intron (NM_001107697/310677, intron 1 of 11)	0777	215E-06	269E-04	Otud7b	7955	Hyper
chr10	60279749	60281177	Promoter (<=1kb)	0893	219E-06	274E-04	Serpinf2	0	Hyper

chr19	42692845	42693982	Intron (NM_001106188/292041, intron 6 of 8)	1090	221E-06	275E-04	Wwox	260605	Hyper
chr8	115364156	115364487	Distal Intergenic	1908	221E-06	275E-04	Stt3b	-370129	Hyper
chr7	8758118	8760979	3' UTR	0735	224E-06	278E-04	Gadd45b	19054	Hyper
chr6	124446148	124447038	Distal Intergenic	1078	228E-06	281E-04	Bdkrb2	-25528	Hyper
chr9	15822850	15823391	Distal Intergenic	1459	232E-06	285E-04	Cdc5l	257901	Hyper
chr7	130451337	130458046	3' UTR	0572	237E-06	289E-04	Prpf40b	11529	Hyper
chr18	63241099	63245280	Exon (NM_053369/84382, exon 8 of 17)	0645	237E-06	289E-04	Tcf4	181643	Hyper
chr8	37237038	37242279	Promoter (<=1kb)	0656	239E-06	291E-04	Esam	0	Hyper
chr4	83599374	83600354	Intron (NM_147211/259242, intron 2 of 7)	0879	241E-06	292E-04	Wipf3	48906	Hyper
chr1	121552133	121553967	Promoter (1-2kb)	0830	244E-06	293E-04	Igf1r	1392	Hyper
chr14	6678167	6680316	Intron (NM_001270556/25272, intron 4 of 13)	0923	244E-06	293E-04	Arhgap24	169179	Hyper
chr10	3965422	3970386	Intron (NM_001109526/689142, intron 18 of 20)	0546	245E-06	293E-04	Cpped1	263900	Hyper
chr3	139556666	139557597	Distal Intergenic	1157	252E-06	300E-04	Entpd6	-18103	Hyper
chr18	76963399	76965514	Distal Intergenic	0822	255E-06	302E-04	Ptgr3	-488921	Hyper
chr19	9898687	9902410	Promoter (<=1kb)	0729	255E-06	302E-04	Kifc3	0	Hyper
chr8	73087017	73087397	Distal Intergenic	1664	258E-06	303E-04	Mns1	-61480	Hyper
chr1	86139132	86141366	Distal Intergenic	0888	263E-06	307E-04	Cd22	-6810	Hyper
chr4	120610728	120611201	Distal Intergenic	1497	264E-06	308E-04	Gata2	-47785	Hyper
chr3	167309977	167312672	Intron (NM_001191609/140433, intron 2 of 78)	0825	266E-06	310E-04	Lama5	5698	Hyper
chr4	63557564	63559204	Exon (NM_001127298/312225, exon 14 of 15)	1041	275E-06	319E-04	Cyren	-5962	Hyper
chr3	9124678	9127599	Distal Intergenic	0785	277E-06	319E-04	Gpsm1	-13265	Hyper
chr11	68112351	68115747	3' UTR	0731	278E-06	320E-04	Tnk2	38262	Hyper
chr16	47271423	47273239	Intron (NM_031819/83720, intron 2 of 26)	0943	279E-06	320E-04	Fat1	22868	Hyper
chr19	24478666	24480140	Promoter (<=1kb)	0977	281E-06	321E-04	Gipc1	0	Hyper
chr12	41516646	41520762	Distal Intergenic	0739	283E-06	322E-04	Acads	22996	Hyper
chr20	8648033	8650469	Distal Intergenic	0705	284E-06	322E-04	Btbd9	-8189	Hyper
chr7	67595785	67596318	Distal Intergenic	1371	284E-06	322E-04	Ankrd46	71851	Hyper
chr19	49740061	49741729	Distal Intergenic	0682	292E-06	329E-04	Jph3	-52594	Hyper
chr8	103486593	103489377	Intron (NM_080402/140585, intron 13 of 14)	0635	293E-06	329E-04	Ryk	67093	Hyper
chr18	53954921	53957364	Promoter (<=1kb)	0576	297E-06	330E-04	Smim3	0	Hyper
chr11	82577845	82589723	3' UTR	0462	300E-06	333E-04	Txnrd2	-9689	Hyper
chr10	105234840	105242482	Exon (NM_057196/117542, exon 2 of 14)	0502	300E-06	333E-04	Baiap2	11708	Hyper
chr2	167338368	167339310	Distal Intergenic	0994	302E-06	334E-04	Asic5	30384	Hyper

chr6	127610512	127612301	Promoter (<=1kb)	0891	303E-06	335E-04	Degs2	992	Hyper
chr13	102119288	102119664	Distal Intergenic	1842	304E-06	335E-04	Rps6kc1	370634	Hyper
chr1	181156119	181157940	3' UTR	1011	305E-06	336E-04	Eif3c	-3630	Hyper
chr16	2524441	2529941	Intron (NM_001106061/290541, intron 1 of 9)	0526	306E-06	337E-04	Arhgef3	11930	Hyper
chr17	51335581	51339087	3' UTR	0669	310E-06	340E-04	B3galnt2	38382	Hyper
chr4	124236991	124249002	Exon (NM_017206/29464, exon 6 of 15)	0470	310E-06	340E-04	Slc6a6	41644	Hyper
chr12	42727586	42732587	Distal Intergenic	0608	314E-06	343E-04	Coro1c	-8279	Hyper
chr19	53045589	53046643	Intron (NM_175596/307940, intron 1 of 12)	0965	315E-06	343E-04	Disc1	30814	Hyper
chr1	260180010	260181537	Intron (NM_030829/59075, intron 4 of 15)	0940	316E-06	344E-04	Grk5	151717	Hyper
chr10	49725090	49727075	Distal Intergenic	0762	320E-06	345E-04	Elac2	92676	Hyper
chr19	51306652	51308019	Exon (NM_001108455/361435, exon 36 of 42)	1244	322E-06	347E-04	Vps9d1	-16038	Hyper
chr4	15528152	15533088	Intron (NM_053621/113970, intron 1 of 22)	0594	323E-06	347E-04	Magi2	336949	Hyper
chr2	175329236	175333626	Intron (NM_017020/24499, intron 1 of 9)	0603	334E-06	356E-04	Il6r	13898	Hyper
chr6	130910024	130910906	Intron (NM_001108062/314465, intron 3 of 15)	1006	335E-06	357E-04	Ppp1r13b	18743	Hyper
chr14	5957054	5958671	Intron (NM_001107206/305152, intron 1 of 19)	0620	336E-06	357E-04	Aff1	5280	Hyper
chr3	156652647	156658395	Intron (NM_012637/24697, intron 1 of 8)	0483	339E-06	360E-04	Ptpn1	13836	Hyper
chr10	13476508	13480202	Exon (NM_001185046/681359, exon 3 of 14)	0715	339E-06	360E-04	E4f1	5772	Hyper
chr7	132064244	132072481	Exon (NM_019266/29710, exon 6 of 27)	0494	340E-06	360E-04	Scn8a	81764	Hyper
chr8	57999488	58001833	Distal Intergenic	0803	341E-06	360E-04	Ulk3	6457	Hyper
chr1	86933887	86936504	Distal Intergenic	0704	346E-06	364E-04	Garre1	-19451	Hyper
chr5	151450886	151453382	Intron (NM_001005903/298584, intron 1 of 8)	0702	348E-06	365E-04	Capzb	15182	Hyper
chr2	182422223	182423444	Exon (NM_001107693/310658, exon 4 of 17)	0871	350E-06	367E-04	Pogz	9349	Hyper
chr6	45482716	45484308	Intron (NM_001012192/362721, intron 5 of 8)	0974	352E-06	367E-04	Eipr1	94265	Hyper
chr12	26489919	26491411	Promoter (1-2kb)	0930	360E-06	375E-04	Rabgef1	-1903	Hyper
chr16	18631011	18632018	Promoter (<=1kb)	1051	362E-06	376E-04	Il12rb1	751	Hyper
chr17	15190460	15193321	Exon (NM_001008556/306808, exon 13 of 13)	0919	363E-06	376E-04	Ippk	36109	Hyper
chr3	64553947	64555296	Intron (NM_001107738/311146, intron 9 of 17)	0711	365E-06	377E-04	Itprid2	17240	Hyper
chr10	57632014	57634836	Promoter (2-3kb)	0675	367E-06	379E-04	Camkk1	-2574	Hyper
chr11	76021394	76022011	Intron (NM_001013864/288010, intron 6 of 11)	2027	368E-06	379E-04	Mir28	-45786	Hyper
chr1	181931882	181932232	Intron (NM_001033998/308995, intron 11 of 30)	1537	369E-06	380E-04	Itgal	13699	Hyper
chr13	91904910	91906571	3' UTR	0914	372E-06	381E-04	Coq8a	24860	Hyper
chr5	146572894	146575901	3' UTR	0686	373E-06	382E-04	Slc30a2	12431	Hyper
chr15	3716010	3718872	Exon (NM_001008863/408223, exon 6 of 20)	0765	380E-06	387E-04	Usp54	23356	Hyper

chr20	28142420	28152567	Distal Intergenic	0477	385E-06	389E-04	Chst3	-21453	Hyper
chr2	32734926	32736936	Distal Intergenic	0725	387E-06	390E-04	Pik3r1	216271	Hyper
chr13	30157192	30159246	Distal Intergenic	0734	387E-06	390E-04	Gli2	-154375	Hyper
chr20	44978686	44981401	Exon (NM_001106397/294520, exon 8 of 24)	0620	390E-06	392E-04	Mical1	4548	Hyper
chr17	44655663	44660046	Intron (NM_001108415/361251, intron 6 of 21)	0753	392E-06	392E-04	Elmo1	162742	Hyper
chr17	6924822	6928572	Intron (NM_001271297/306759, intron 2 of 10)	0695	395E-06	394E-04	Spock1	183309	Hyper
chr2	183223626	183229549	Distal Intergenic	0507	397E-06	396E-04	Mcl1	4406	Hyper
chr10	82459946	82463310	Distal Intergenic	0750	405E-06	399E-04	Srcin1	105389	Hyper
chr8	75711686	75713519	Intron (NM_001106838/300836, intron 2 of 12)	1029	405E-06	399E-04	Atosa	16664	Hyper
chr11	66836251	66839073	Exon (NM_147139/257645, exon 11 of 15)	0608	408E-06	401E-04	Umps	30142	Hyper
chr4	158155061	158157448	Intron (NM_013091/25625, intron 1 of 9)	0873	412E-06	405E-04	Tnfrsf1a	4167	Hyper
chr13	102118617	102119128	Distal Intergenic	1783	418E-06	409E-04	Rps6kc1	371170	Hyper
chr1	14252139	14253284	Distal Intergenic	0832	420E-06	410E-04	Ifngr1	-79981	Hyper
chr11	66833279	66835137	Exon (NM_147139/257645, exon 12 of 15)	0828	423E-06	412E-04	Umps	27170	Hyper
chr6	102092135	102102015	Exon (NM_139330/246212, exon 15 of 21)	0465	427E-06	415E-04	Sipa1l1	85955	Hyper
chr16	17573830	17574706	Distal Intergenic	0981	429E-06	416E-04	Ap1m1	27644	Hyper
chr4	120664247	120665347	Exon (NM_033442/25159, exon 6 of 7)	1103	434E-06	418E-04	Gata2	5261	Hyper
chr3	18599327	18602328	Distal Intergenic	0502	434E-06	418E-04	Gsn	-9159	Hyper
chr12	14842473	14846041	Promoter (<=1kb)	0584	434E-06	418E-04	Mafk	0	Hyper
chr4	120318392	120319566	Distal Intergenic	0904	436E-06	419E-04	Cnbp	-6747	Hyper
chr15	98450753	98452221	Intron (NM_001127494/361092, intron 1 of 10)	0908	436E-06	419E-04	Stk24	6719	Hyper
chr11	33037925	33039559	Intron (NM_001191660/304077, intron 1 of 35)	1043	437E-06	419E-04	Dop1b	9876	Hyper
chr10	47439076	47440302	Distal Intergenic	0808	438E-06	419E-04	Zfp286a	20860	Hyper
chr7	8682855	8685154	Promoter (2-3kb)	0638	438E-06	419E-04	Diras1	-2810	Hyper
chr5	70243080	70246856	Distal Intergenic	0573	440E-06	419E-04	Klf4	36473	Hyper
chr9	57214911	57216937	Intron (NM_053456/84587, intron 5 of 5)	0805	441E-06	419E-04	Plcl1	313338	Hyper
chr17	32837599	32839413	Distal Intergenic	1094	445E-06	422E-04	Foxq1	75964	Hyper
chr18	55895589	55897823	Intron (NM_012853/25324, intron 5 of 6)	0862	446E-06	422E-04	Fbxo38	106138	Hyper
chr3	106162456	106165878	Intron (NM_001191740/311329, intron 10 of 10)	0614	447E-06	422E-04	Gchfr	4410	Hyper
chr5	136545838	136547112	Distal Intergenic	0948	450E-06	424E-04	Lnc081	154956	Hyper
chr3	8298563	8299765	Distal Intergenic	1166	452E-06	426E-04	Ptgds	-13730	Hyper
chr20	6485101	6486231	Intron (NM_001012174/361810, intron 5 of 10)	1309	470E-06	437E-04	Fkbp5	55604	Hyper
chr10	41509716	41516252	Intron (NM_031608/50592, intron 14 of 15)	0558	470E-06	437E-04	Mfap3	-180536	Hyper

chr18	4117300	4119557	Promoter (<=1kb)	0785	473E-06	439E-04	Impact	-573	Hyper
chr13	92139582	92146818	Intron (NM_019312/54260, intron 2 of 7)	0533	476E-06	440E-04	Itpkb	70366	Hyper
chr14	5924644	5926440	Intron (NM_001107206/305152, intron 3 of 19)	0734	479E-06	442E-04	Aff1	37511	Hyper
chr6	10755109	10757944	Distal Intergenic	0659	483E-06	444E-04	Haao	-87841	Hyper
chr6	39326273	39328168	Intron (NM_001012111/313977, intron 14 of 20)	0810	485E-06	446E-04	Lpin1	52008	Hyper
chr7	8685409	8686633	Promoter (1-2kb)	0924	489E-06	449E-04	Diras1	-1331	Hyper
chr6	130685991	130686459	Intron (NM_130749/170577, intron 12 of 17)	1505	489E-06	449E-04	Ckb	45842	Hyper
chr14	6022351	6024992	Distal Intergenic	0672	491E-06	450E-04	C14h4orf36	-33589	Hyper
chr4	71042319	71043302	Distal Intergenic	0883	501E-06	457E-04	Tas2r144	-42880	Hyper
chr3	143598534	143600200	Distal Intergenic	0938	507E-06	460E-04	Ahcy	-14175	Hyper
chr3	90838182	90839120	Intron (NM_031787/83617, intron 2 of 15)	0995	508E-06	461E-04	Hipk3	27192	Hyper
chr11	35351404	35353595	Exon (NM_001371922/304061, exon 11 of 41)	0755	516E-06	466E-04	Mir6324	-19464	Hyper
chr4	145970182	145974084	Intron (NM_001191975/500287, intron 1 of 21)	0652	517E-06	466E-04	Srgap3	95640	Hyper
chr12	36891329	36893755	Promoter (<=1kb)	0906	519E-06	467E-04	Tbx3	0	Hyper
chr1	151376593	151377970	Intron (NM_001034921/293128, intron 9 of 11)	1010	526E-06	472E-04	Gab2	-51984	Hyper
chr1	11318214	11318785	Distal Intergenic	1508	530E-06	475E-04	Cited2	-993641	Hyper
chr2	183231811	183236328	3' UTR	0509	531E-06	475E-04	Adamtsl4	10520	Hyper
chr1	23132149	23133957	Distal Intergenic	0738	535E-06	478E-04	Sgk1	-144052	Hyper
chr13	92166735	92167234	Distal Intergenic	1276	549E-06	487E-04	Itpkb	97519	Hyper
chr7	110957384	110961543	Intron (NM_001173370/362959, intron 1 of 8)	0548	551E-06	487E-04	Tmem184b	6395	Hyper
chr16	16768632	16770256	Promoter (<=1kb)	1266	552E-06	487E-04	Sh2d4b	0	Hyper
chr18	28335782	28337359	Promoter (<=1kb)	0822	552E-06	487E-04	Cd14	0	Hyper
chr2	78161743	78164226	Intron (NM_053714/114506, intron 1 of 11)	0708	571E-06	500E-04	Ankh	8717	Hyper
chr1	256180473	256181492	Intron (NM_001372054/307989, intron 6 of 24)	1059	582E-06	508E-04	Ablim1	-8131	Hyper
chr9	102451075	102452430	Intron (NM_053903/116683, intron 1 of 4)	0826	585E-06	509E-04	Efna5	142704	Hyper
chr10	20130394	20131226	Exon (NM_031321/83467, exon 29 of 36)	1169	588E-06	510E-04	Mir218-2	61943	Hyper
chr5	161201412	161203529	Distal Intergenic	0704	594E-06	513E-04	Slc45a1	-72109	Hyper
chr6	72770311	72771309	Distal Intergenic	0970	594E-06	513E-04	Psma6	-7900	Hyper
chr3	91067758	91069605	Exon (NM_001109202/499846, exon 7 of 10)	0667	595E-06	513E-04	Tcp11l1	21982	Hyper
chr4	124217534	124227848	Intron (NM_017206/29464, intron 2 of 14)	0422	599E-06	515E-04	Slc6a6	22187	Hyper
chr20	27889831	27902453	Promoter (<=1kb)	0427	604E-06	519E-04	Ddit4	0	Hyper
chr15	12479705	12483543	Intron (NM_013219/26989, intron 3 of 28)	0590	606E-06	519E-04	Cadps	189443	Hyper
chr10	42270172	42271875	Distal Intergenic	0751	606E-06	519E-04	Cnot8	-9904	Hyper

chr17	4918617	4920337	Distal Intergenic	0777	611E-06	522E-04	Isca1	13326	Hyper
chr10	105056625	105062009	Intron (NM_001134499/287871, intron 7 of 33)	0502	612E-06	522E-04	Chmp6	-129963	Hyper
chr17	13343431	13344385	Distal Intergenic	1007	621E-06	529E-04	Gadd45g	34202	Hyper
chr1	151142324	151144676	Intron (NM_001191628/308831, intron 13 of 31)	0722	631E-06	533E-04	Nars2	-155826	Hyper
chr10	73629985	73630476	Exon (NM_053665/114124, exon 3 of 10)	1478	637E-06	538E-04	Akap1	5770	Hyper
chr6	100177651	100180781	Intron (NM_001100863/362760, intron 1 of 14)	0639	647E-06	543E-04	Galnt16	7283	Hyper
chr3	13280936	13282733	Exon (NM_171983/64159, exon 27 of 56)	0876	651E-06	545E-04	Dync2i2	39388	Hyper
chr1	206717209	206717953	Intron (NM_031344/83512, intron 5 of 11)	1208	657E-06	550E-04	Fads2	29189	Hyper
chr1	76690323	76695762	Distal Intergenic	0470	660E-06	551E-04	Bicra	-3936	Hyper
chr5	155468807	155470154	Intron (NM_001077648/313678, intron 6 of 9)	0951	666E-06	554E-04	Prdm2	61671	Hyper
chr1	22978961	22989670	Promoter (<=1kb)	0417	666E-06	554E-04	Sgk1	0	Hyper
chr3	16017500	16020676	Intron (NM_001108579/362111, intron 2 of 11)	0719	679E-06	563E-04	Sh2d3c	6875	Hyper
chr9	65490853	65497504	Distal Intergenic	0462	687E-06	567E-04	Klf7	-6133	Hyper
chr12	43072702	43074586	Intron (NM_001271178/360824, intron 3 of 7)	0828	689E-06	567E-04	Wscd2	14005	Hyper
chr10	60359192	60359723	Promoter (<=1kb)	1376	697E-06	571E-04	Scarf1	0	Hyper
chr4	179645273	179646942	Intron (NM_001083336/691337, intron 1 of 14)	0698	700E-06	573E-04	Stk38l	10998	Hyper
chr9	26324890	26325329	Distal Intergenic	1419	702E-06	573E-04	B3gat2	157716	Hyper
chr3	118346370	118347985	Promoter (<=1kb)	0960	714E-06	581E-04	Hspa12b	16	Hyper
chr20	45412771	45414797	Distal Intergenic	0620	717E-06	582E-04	LOC499469	76974	Hyper
chr10	73814428	73816017	Promoter (1-2kb)	0966	724E-06	586E-04	Trim25	1610	Hyper
chr1	79486419	79487619	Promoter (<=1kb)	0910	726E-06	586E-04	Bcl3	-812	Hyper
chr14	78371067	78371541	Promoter (2-3kb)	1425	727E-06	586E-04	Pla2g3	2834	Hyper
chr1	28395745	28396502	Distal Intergenic	1048	735E-06	591E-04	Rnf146	-68663	Hyper
chr4	30826275	30830152	Promoter (<=1kb)	0591	737E-06	591E-04	Cdk6	0	Hyper
chr11	82598978	82600866	Intron (NM_001131013/303798, intron 3 of 17)	0908	750E-06	600E-04	Txnrd2	-30822	Hyper
chr7	108472403	108475145	Exon (NM_173122/500901, exon 3 of 12)	0670	751E-06	600E-04	MGC94207	-26287	Hyper
chr20	9687246	9687711	Intron (NM_001013074/294322, intron 3 of 9)	1505	755E-06	602E-04	Pknox1	6865	Hyper
chr11	69698051	69699402	Distal Intergenic	1128	760E-06	604E-04	Acap2	159505	Hyper
chr6	117923479	117924271	Intron (NM_138862/192225, intron 10 of 11)	0894	764E-06	606E-04	Spata7	43584	Hyper
chr9	92739559	92745418	Intron (NM_053449/363287, intron 1 of 26)	0499	765E-06	606E-04	Hdac4	4746	Hyper
chr1	13079015	13081446	Distal Intergenic	0644	767E-06	607E-04	Hebp2	120331	Hyper
chr3	145049204	145054059	3' UTR	0521	780E-06	615E-04	Aar2	-3367	Hyper
chr7	8412829	8416359	Intron (NM_001009967/314641, intron 1 of 18)	0561	788E-06	619E-04	Pip5k1c	9629	Hyper

chr10	61155280	61156084	Intron (NM_001108285/360577, intron 1 of 7)	1105	788E-06	619E-04	Mrm3	59328	Hyper
chr20	12797165	12802284	Distal Intergenic	0508	798E-06	627E-04	Mif	6246	Hyper
chr2	102632969	102635379	Exon (NM_031043/81675, exon 4 of 6)	0680	801E-06	627E-04	Gyg1	16223	Hyper
chr2	144760725	144762756	Intron (NM_001191566/282635, intron 1 of 7)	0647	803E-06	628E-04	Mbnl1	62892	Hyper
chr8	66624829	66625738	Distal Intergenic	1238	809E-06	630E-04	Snx22	-11979	Hyper
chr1	203214486	203218007	Distal Intergenic	0551	823E-06	636E-04	Cdc42ep2	-3655	Hyper
chr16	1091967	1102540	Promoter (<=1kb)	0469	824E-06	636E-04	Mir3075	0	Hyper
chr17	22459262	22461629	Promoter (<=1kb)	0655	827E-06	637E-04	Edn1	0	Hyper
chr4	63555492	63557271	Intron (NM_001127298/312225, intron 14 of 14)	0853	832E-06	640E-04	Cyren	-3890	Hyper
chr8	110670221	110671538	Intron (NM_001108783/363151, intron 2 of 6)	1016	836E-06	642E-04	Ccdc12	34511	Hyper
chr9	13482642	13487955	Distal Intergenic	0476	839E-06	643E-04	Taf8	-3982	Hyper
chr2	11374571	11379515	Distal Intergenic	0606	842E-06	644E-04	Arrdc3	236871	Hyper
chr3	16376378	16381524	Distal Intergenic	0598	847E-06	646E-04	Slc2a8	-91914	Hyper
chr16	3036688	3039081	Intron (NM_170787/259269, intron 3 of 16)	0717	848E-06	646E-04	Erc2	188010	Hyper
chr19	24398245	24399529	Promoter (<=1kb)	0793	851E-06	648E-04	Adgre5	0	Hyper
chr16	18522562	18523876	Exon (NM_001106070/290640, exon 3 of 7)	0960	854E-06	649E-04	Map1s	3886	Hyper
chr1	90898076	90902031	Distal Intergenic	0646	857E-06	651E-04	Plekhf1	8076	Hyper
chr18	3827155	3827840	Intron (NM_001077231/686179, intron 1 of 13)	1047	857E-06	651E-04	Ttc39c	58353	Hyper
chr12	35237379	35239949	Distal Intergenic	0624	859E-06	652E-04	Trafd1	71773	Hyper
chr7	8163468	8169473	Promoter (<=1kb)	0474	864E-06	654E-04	Gna11	0	Hyper
chr6	106575935	106576575	Distal Intergenic	1127	870E-06	658E-04	Cipc	-18444	Hyper
chr19	48224724	48225440	Distal Intergenic	1279	873E-06	659E-04	6430548M08Rikl	26515	Hyper
chr7	72874911	72879022	Intron (NM_001197907/117520, intron 3 of 16)	0518	880E-06	664E-04	Oxr1	-12368	Hyper
chr3	11038782	11042497	Intron (NM_012805/25271, intron 1 of 9)	0650	886E-06	668E-04	Rxra	48866	Hyper
chr14	79349703	79351072	Exon (NM_001012038/305482, exon 14 of 19)	0861	888E-06	668E-04	Hormad2	-49793	Hyper
chr1	220480810	220483151	Exon (NM_001191562/309407, exon 18 of 25)	0666	893E-06	672E-04	Mir204	163879	Hyper
chr5	138953117	138956365	Distal Intergenic	0778	896E-06	672E-04	Psmb2	-14199	Hyper
chr15	39791302	39801125	Distal Intergenic	0452	901E-06	675E-04	Scara5	-78929	Hyper
chr17	84476960	84477194	Distal Intergenic	1612	904E-06	676E-04	Gad2	-286436	Hyper
chr5	63871751	63874812	Promoter (<=1kb)	0527	905E-06	676E-04	Mrpl50	0	Hyper
chr5	147541116	147541822	Distal Intergenic	1385	910E-06	678E-04	Clic4	-27838	Hyper
chr12	20802377	20803815	Distal Intergenic	0625	914E-06	680E-04	Hspb1	-6702	Hyper
chr9	39913899	39922361	Distal Intergenic	0460	943E-06	699E-04	Mgat4a	-147009	Hyper

chr20	13028575	13029895	Distal Intergenic	0974	944E-06	699E-04	Susd2	11098	Hyper
chr19	54653987	54655302	Distal Intergenic	0824	946E-06	699E-04	Tomm20	279886	Hyper
chr16	6422441	6425287	3' UTR	0549	951E-06	702E-04	Sema3g	8852	Hyper
chr6	72765484	72770202	Distal Intergenic	0594	960E-06	704E-04	Psma6	-9007	Hyper
chr16	46592264	46594236	Intron (NM_053770/114901, intron 2 of 22)	0819	963E-06	704E-04	Sorbs2	32212	Hyper
chr4	120659548	120660244	Promoter (<=1kb)	1199	967E-06	706E-04	Gata2	562	Hyper
chr5	144928401	144929114	Intron (NM_001107910/313027, intron 17 of 17)	1113	979E-06	714E-04	Xkr8	16081	Hyper
chr16	17519592	17524781	Promoter (<=1kb)	0676	998E-06	725E-04	Klf2	0	Hyper
chr2	173221389	173224872	Promoter (2-3kb)	0582	100E-05	725E-04	Pear1	2568	Hyper
chr1	117559663	117563285	Distal Intergenic	0669	100E-05	725E-04	Klf13	-23037	Hyper
chr6	72818359	72820369	Distal Intergenic	0597	101E-05	726E-04	Psma6	39150	Hyper
chr7	124527414	124529174	Exon (NM_001034940/362990, exon 6 of 8)	0767	102E-05	733E-04	Zcrb1	9390	Hyper
chr3	90822836	90823353	Intron (NM_031787/83617, intron 2 of 15)	1149	104E-05	742E-04	Hipk3	42959	Hyper
chr15	13972848	13973980	Intron (NM_021774/60398, intron 1 of 7)	0961	104E-05	743E-04	Fhit	4966	Hyper
chr1	45803346	45804553	Distal Intergenic	0833	104E-05	744E-04	Ldhal6b	194715	Hyper
chr17	27000660	27003716	Promoter (<=1kb)	0527	106E-05	751E-04	Rreb1	0	Hyper
chr8	67632947	67633501	Distal Intergenic	1288	108E-05	764E-04	Tpm1	23403	Hyper
chr8	57430083	57432926	Intron (NM_001013040/266611, intron 1 of 12)	0657	110E-05	773E-04	Ptpn9	38124	Hyper
chr6	122884133	122885225	Intron (NM_022519/24648, intron 2 of 5)	1169	110E-05	774E-04	Serpina1	3114	Hyper
chr5	148510749	148512980	Distal Intergenic	0702	110E-05	774E-04	RGD1564482	4539	Hyper
chr8	20190485	20192080	Exon (NM_134368/171379, exon 7 of 34)	0774	111E-05	778E-04	Smarca4	22768	Hyper
chr19	49686607	49695707	Distal Intergenic	0413	111E-05	778E-04	Map1lc3b	20628	Hyper
chr5	155457137	155460297	Intron (NM_001077648/313678, intron 6 of 9)	0589	111E-05	778E-04	Prdm2	71528	Hyper
chr11	69729026	69734275	Distal Intergenic	0463	112E-05	780E-04	Acap2	190480	Hyper
chr14	82990056	82990495	Distal Intergenic	1357	113E-05	784E-04	Tns3	353643	Hyper
chr17	6922770	6923617	Intron (NM_001271297/306759, intron 2 of 10)	1318	114E-05	794E-04	Spock1	181257	Hyper
chr8	66681428	66681733	Intron (NM_001008327/300797, intron 4 of 4)	1925	114E-05	794E-04	Ciao2a	10895	Hyper
chr5	158522614	158523410	Distal Intergenic	1131	116E-05	802E-04	Agtrap	-3578	Hyper
chr7	69731685	69732974	Distal Intergenic	0992	116E-05	803E-04	Azin1	-50107	Hyper
chr5	146791838	146796266	Exon (NM_001108687/362625, exon 6 of 12)	0463	116E-05	804E-04	Mtfr1l	-45109	Hyper
chr13	94183579	94186866	Intron (NM_017116/29154, intron 2 of 20)	0650	117E-05	808E-04	Capn2	14103	Hyper
chr8	114537703	114538342	Promoter (<=1kb)	1305	117E-05	809E-04	Cmtm8	60	Hyper
chr17	1807159	1814521	Promoter (<=1kb)	0458	119E-05	814E-04	Mir3074	0	Hyper

chr19	10814170	10815630	Distal Intergenic	0736	123E-05	833E-04	Mt1	11418	Hyper
chr18	69072423	69073986	Intron (NM_001108891/364900, intron 3 of 4)	0712	124E-05	839E-04	Ctif	74905	Hyper
chr10	102475462	102479743	Promoter (<=1kb)	0424	124E-05	841E-04	Septin9	0	Hyper
chr17	15195094	15197554	Intron (NM_001008556/306808, intron 11 of 12)	0689	125E-05	843E-04	Ippk	31876	Hyper
chr14	74233813	74241131	Promoter (<=1kb)	0462	125E-05	847E-04	Tbc1d14	0	Hyper
chr5	132684696	132685119	Distal Intergenic	1302	125E-05	847E-04	Slc2a1	-32080	Hyper
chr12	7090347	7091722	Distal Intergenic	0840	126E-05	849E-04	Mtus2	-12016	Hyper
chr10	104801713	104805151	Distal Intergenic	0549	126E-05	849E-04	Nptx1	15207	Hyper
chr4	7356016	7356630	Distal Intergenic	1250	128E-05	858E-04	Insig1	-32091	Hyper
chr9	90162629	90176462	Distal Intergenic	0480	128E-05	858E-04	Agap1	-11563	Hyper
chr12	4530462	4532039	Exon (NM_001170398/304244, exon 51 of 61)	0888	128E-05	859E-04	Tgap1-ps1	-181042	Hyper
chr6	103821910	103824754	Intron (NM_001169116/314306, intron 1 of 12)	0656	130E-05	868E-04	Mideas	4424	Hyper
chr2	174804076	174814426	Distal Intergenic	0428	131E-05	870E-04	Zbtb7b	-4632	Hyper
chr15	98811448	98814707	Intron (NM_001105759/259237, intron 1 of 52)	0538	131E-05	870E-04	Dock9	68446	Hyper
chr7	69696306	69698170	Distal Intergenic	0742	138E-05	909E-04	Azin1	-14728	Hyper
chr3	165394242	165402889	Intron (NM_214459/362284, intron 7 of 11)	0494	138E-05	912E-04	Phactr3	50968	Hyper
chr10	89142761	89146112	Intron (NM_017212/29477, intron 1 of 9)	0554	139E-05	916E-04	Mapt	4113	Hyper
chr3	13541478	13542634	Promoter (1-2kb)	0905	140E-05	922E-04	Phyhd1	1069	Hyper
chr16	4664022	4667297	Intron (NM_175595/306243, intron 3 of 36)	0580	140E-05	922E-04	Cacna2d3	245054	Hyper
chr5	161022114	161024756	Intron (NM_053885/116665, intron 11 of 22)	0648	142E-05	930E-04	Slc45a1	104547	Hyper
chr3	141203506	141204899	Distal Intergenic	0864	142E-05	932E-04	Id1	-6394	Hyper
chr2	174771196	174772590	Distal Intergenic	1121	144E-05	939E-04	Adam15	-6098	Hyper
chr3	168785998	168788393	Promoter (<=1kb)	0904	144E-05	941E-04	Sox18	0	Hyper
chr1	206718099	206718832	Intron (NM_031344/83512, intron 5 of 11)	0922	145E-05	942E-04	Fads2	28310	Hyper
chr9	101916886	101918591	Distal Intergenic	0794	145E-05	942E-04	Efna5	676543	Hyper
chr10	60800234	60802061	Promoter (<=1kb)	0793	147E-05	953E-04	Rph3al	0	Hyper
chr4	59655133	59659738	Distal Intergenic	0515	147E-05	956E-04	Mir29b1	-4066	Hyper
chr5	141469106	141469915	Distal Intergenic	1057	148E-05	957E-04	Hpca	-5113	Hyper
chr10	72908438	72911813	Intron (NM_001013971/303419, intron 1 of 10)	0572	148E-05	957E-04	Cuedc1	15801	Hyper
chr3	147414428	147419305	Promoter (2-3kb)	0532	149E-05	957E-04	Fam83d	-2636	Hyper
chr12	31514447	31517411	Intron (NM_001108334/360801, intron 1 of 46)	0635	149E-05	961E-04	Ncor2	47859	Hyper
chr6	132025608	132032507	Distal Intergenic	0468	150E-05	966E-04	Nudt14	-8310	Hyper
chr19	18306423	18309197	Distal Intergenic	0745	151E-05	971E-04	Cyld	64453	Hyper

chr7	108178963	108186053	Promoter (2-3kb)	0427	152E-05	979E-04	Scx	2355	Hyper
chr6	72817359	72818196	Distal Intergenic	1107	153E-05	980E-04	Psma6	38150	Hyper
chr18	62393736	62396188	Distal Intergenic	0645	155E-05	989E-04	Mc2r	-378248	Hyper
chr20	44973735	44975983	Promoter (<=1kb)	0620	156E-05	993E-04	Mical1	0	Hyper
chr2	138434182	138435285	Intron (NM_001083115/84494, intron 3 of 10)	0971	156E-05	993E-04	Trpc4	83096	Hyper
chr20	6996194	6997216	Distal Intergenic	0927	156E-05	994E-04	Stk38	43927	Hyper
chr1	244351182	244351739	Intron (NM_001007148/361765, intron 6 of 15)	1227	157E-05	994E-04	Poll	56923	Hyper
chr14	74245055	74248529	Exon (NM_001113365/360956, exon 7 of 15)	0523	158E-05	100E-03	Tbc1d14	9804	Hyper
chr1	220625543	220626584	Distal Intergenic	0892	159E-05	100E-03	Klf9	-73524	Hyper
chr2	203672433	203672779	Distal Intergenic	1555	159E-05	100E-03	S1pr1	-43323	Hyper
chr5	151520026	151521376	Intron (NM_001005903/298584, intron 4 of 8)	0873	159E-05	100E-03	Slc66a1	28449	Hyper
chr8	110058647	110060559	Exon (NM_001024278/367171, exon 15 of 17)	0911	161E-05	101E-03	Dhx30	36270	Hyper
chrX	1343399	1345194	Distal Intergenic	0670	161E-05	102E-03	Araf	-104326	Hyper
chr4	120611313	120611562	Distal Intergenic	1601	163E-05	102E-03	Gata2	-47424	Hyper
chr2	173225276	173226400	Promoter (1-2kb)	0981	163E-05	103E-03	Pear1	1040	Hyper
chr1	90902168	90903157	Distal Intergenic	1005	164E-05	103E-03	Plekhf1	6950	Hyper
chr6	121793021	121796539	Intron (NM_001191985/500709, intron 2 of 9)	0454	164E-05	103E-03	Itpk1	47499	Hyper
chr12	34809580	34811106	Distal Intergenic	0791	167E-05	104E-03	Sh2b3	59731	Hyper
chr15	41057322	41059777	Intron (NM_001105717/25416, intron 3 of 13)	0638	169E-05	104E-03	Dpysl2	13080	Hyper
chr8	121431687	121435297	Distal Intergenic	0589	169E-05	105E-03	Klhl40	-5988	Hyper
chr10	63418557	63420991	3' UTR	0556	171E-05	105E-03	Poldip2	9791	Hyper
chr3	18983682	18985494	Intron (NM_138710/192126, intron 1 of 15)	0741	172E-05	106E-03	Dab2ip	68392	Hyper
chr14	34423788	34426536	Distal Intergenic	0657	173E-05	106E-03	Spata18	132119	Hyper
chr17	17461954	17463070	Intron (NM_001108881/364681, intron 2 of 7)	1127	173E-05	107E-03	Rnf144b	99690	Hyper
chr8	60411448	60412671	Distal Intergenic	0880	175E-05	107E-03	Myo9a	262214	Hyper
chr6	127603733	127604558	Intron (NM_001017457/314438, intron 1 of 2)	1124	176E-05	108E-03	Degs2	8735	Hyper
chr5	116251777	116254832	Exon (NM_001107949/313409, exon 11 of 18)	0603	177E-05	108E-03	Leprot	-35011	Hyper
chr7	113358012	113359456	Distal Intergenic	0831	178E-05	108E-03	Tob2	11967	Hyper
chr11	80767099	80767833	Exon (NM_001109057/498112, exon 17 of 30)	1215	178E-05	108E-03	Map6d1	32951	Hyper
chr1	70077701	70081370	Distal Intergenic	0577	179E-05	109E-03	Ttyh1	-5856	Hyper
chr4	150295327	150296503	Distal Intergenic	0935	180E-05	110E-03	Cxcl12	-91823	Hyper
chr2	195935004	195942768	Intron (NM_031767/83576, intron 1 of 19)	0396	182E-05	110E-03	Sort1	10642	Hyper
chr1	86175193	86180107	Promoter (2-3kb)	0564	182E-05	110E-03	Hamp	-2328	Hyper

chr9	1167942	1172744	Intron (NM_001044236/301128, intron 1 of 22)	0522	182E-05	110E-03	Kdm4b	9163	Hyper
chr5	131109949	131112402	Distal Intergenic	0708	182E-05	110E-03	Dmap1	39340	Hyper
chr16	19393641	19395789	Exon (NM_001106077/290668, exon 2 of 19)	0637	185E-05	112E-03	Mau2	9756	Hyper
chr16	76517383	76520745	Exon (NM_053951/117020, exon 19 of 31)	0596	186E-05	112E-03	F7	-16795	Hyper
chr17	23828605	23829205	Intron (NM_001001511/306860, intron 1 of 2)	1258	187E-05	113E-03	Gcnt2	9965	Hyper
chr12	38495067	38497570	Intron (NM_001107145/304522, intron 6 of 10)	0603	188E-05	113E-03	Fbxw8	68568	Hyper
chr4	170473119	170474736	Intron (NM_001376935/312812, intron 1 of 20)	0809	189E-05	114E-03	Eps8	12137	Hyper
chr4	115705508	115708432	Distal Intergenic	0526	190E-05	114E-03	Slc4a5	-17160	Hyper
chr2	174059338	174061342	Distal Intergenic	0738	190E-05	114E-03	Ssr2	11047	Hyper
chr17	32639568	32640313	Distal Intergenic	1185	191E-05	114E-03	Foxc1	-4207	Hyper
chr2	61306796	61309147	Distal Intergenic	0645	192E-05	115E-03	Golph3	-38883	Hyper
chr19	50577593	50579186	Intron (NM_001077200/361430, intron 1 of 50)	0721	196E-05	117E-03	Piezo1	27315	Hyper
chr17	73705535	73707184	Intron (NM_001191821/307128, intron 14 of 22)	0743	196E-05	117E-03	Prpf18	74918	Hyper
chr8	70904983	70906133	Intron (NM_173101/25484, intron 1 of 27)	0944	197E-05	117E-03	Myo1e	17049	Hyper
chr9	42290852	42293592	Exon (NM_001106904/301363, exon 10 of 29)	0514	197E-05	117E-03	Map4k4	89232	Hyper
chr7	131292954	131297058	Distal Intergenic	0485	198E-05	118E-03	Atf1	-64904	Hyper
chr16	67595455	67596681	Promoter (<=1kb)	0828	198E-05	118E-03	Tcim	0	Hyper
chr2	45484914	45487926	Distal Intergenic	0547	201E-05	119E-03	Hspb3	-188915	Hyper
chr6	72777663	72780188	Promoter (<=1kb)	0489	205E-05	120E-03	Psma6	0	Hyper
chr16	6386183	6388357	Exon (NM_001376918/306255, exon 4 of 14)	0640	205E-05	120E-03	Nisch	12311	Hyper
chr2	101085719	101087197	Distal Intergenic	0751	206E-05	120E-03	Cyp7b1	-416021	Hyper
chr9	17622352	17624513	Promoter (<=1kb)	0928	208E-05	122E-03	Adgrf5	0	Hyper
chr10	52659591	52662217	Exon (NM_031656/59074, exon 3 of 8)	0546	208E-05	122E-03	Stx8	3841	Hyper
chr12	21144382	21145246	Intron (NM_001100475/192154, intron 1 of 30)	0941	209E-05	122E-03	Hip1	10974	Hyper
chr17	32629791	32631429	3' UTR	0905	210E-05	122E-03	Foxc1	3932	Hyper
chr4	1860086	1860664	Distal Intergenic	0868	210E-05	122E-03	Il6	3358514	Hyper
chr14	76134829	76135801	Exon (NM_016990/24170, exon 4 of 15)	0987	211E-05	123E-03	Mfsd10	30619	Hyper
chr3	105887455	105889526	Distal Intergenic	0665	214E-05	124E-03	Chst14	-26955	Hyper
chr1	47174057	47174557	Exon (NM_001309449/308097, exon 7 of 9)	1182	214E-05	124E-03	Tagap	4198	Hyper
chr14	35046628	35050651	Distal Intergenic	0507	214E-05	124E-03	Ociad1	-131389	Hyper
chr5	109927214	109928372	Distal Intergenic	0752	218E-05	125E-03	Jun	-29946	Hyper
chr5	142443817	142445707	Distal Intergenic	0768	218E-05	125E-03	Pef1	-6084	Hyper
chr20	18758283	18764117	Exon (NM_031805/361833, exon 9 of 45)	0527	220E-05	126E-03	Ank3	166012	Hyper

chr7	97883883	97886096	Intron (NM_031597/29682, intron 2 of 15)	0655	221E-05	126E-03	Kcnq3	139556	Hyper
chr2	210026288	210026956	Distal Intergenic	1076	222E-05	126E-03	Arhgap29	-44243	Hyper
chr1	185509720	185517157	Intron (NM_031721/65164, intron 1 of 8)	0471	225E-05	127E-03	Htra1	11905	Hyper
chr6	95601760	95602386	Intron (NM_172034/64511, intron 9 of 11)	1242	225E-05	127E-03	Max	59580	Hyper
chr5	149357433	149357966	Distal Intergenic	1484	225E-05	127E-03	Zbtb40	-73708	Hyper
chr1	198990986	198991948	Exon (NM_181480/353255, exon 14 of 21)	0945	227E-05	128E-03	Nadsyn1	17806	Hyper
chr14	83675607	83676882	Distal Intergenic	0903	227E-05	128E-03	Hus1	26560	Hyper
chr6	103851654	103856625	Distal Intergenic	0439	228E-05	128E-03	Mideas	-22476	Hyper
chr14	5915956	5917154	Intron (NM_001107206/305152, intron 3 of 19)	0763	228E-05	129E-03	Aff1	46797	Hyper
chr8	104146908	104148618	Distal Intergenic	0692	230E-05	129E-03	Bfsp2	-83509	Hyper
chr3	8516622	8517602	Distal Intergenic	1242	230E-05	129E-03	Bmyc	4053	Hyper
chr7	112771555	112773164	Distal Intergenic	0740	231E-05	129E-03	Mchr1	10001	Hyper
chr10	61006583	61012322	Intron (NM_001105813/287535, intron 4 of 21)	0482	232E-05	129E-03	Rpl37l1	-21477	Hyper
chr3	139396956	139399209	Promoter (<=1kb)	0650	232E-05	129E-03	Cst7	106	Hyper
chr7	107446681	107447631	Intron (NM_001134865/300032, intron 9 of 11)	1001	233E-05	130E-03	Rhpn1	54697	Hyper
chr4	158360232	158361083	Promoter (<=1kb)	1224	233E-05	130E-03	Vwf	80	Hyper
chr10	79642910	79646119	Distal Intergenic	0580	234E-05	130E-03	Xylt2	-23518	Hyper
chr17	7130379	7135662	Intron (NM_001271297/306759, intron 5 of 10)	0472	235E-05	130E-03	Spock1	388866	Hyper
chr8	51219959	51227357	Distal Intergenic	0455	237E-05	131E-03	Ppp2r1b	23770	Hyper
chr2	169654634	169655651	Distal Intergenic	0885	238E-05	131E-03	Trim2	-90212	Hyper
chr4	139393111	139394144	Intron (NM_053879/116658, intron 7 of 24)	0792	239E-05	131E-03	Il5ra	269825	Hyper
chr15	29281350	29282724	Promoter (<=1kb)	0869	241E-05	133E-03	Adcy4	0	Hyper
chr17	44705874	44709311	Exon (NM_001108415/361251, exon 3 of 22)	0690	241E-05	133E-03	Elmo1	113477	Hyper
chr12	22098315	22099570	3' UTR	0782	242E-05	133E-03	Lat2	-9882	Hyper
chr15	39806049	39808727	Distal Intergenic	0622	247E-05	135E-03	Scara5	-71327	Hyper
chr7	97882941	97883791	Intron (NM_031597/29682, intron 2 of 15)	0912	248E-05	135E-03	Kcnq3	141861	Hyper
chr9	60196473	60199029	Intron (NM_057138/117279, intron 2 of 5)	0549	248E-05	135E-03	Cflar	11097	Hyper
chr9	102450320	102450866	Intron (NM_053903/116683, intron 1 of 4)	1182	251E-05	137E-03	Efna5	144268	Hyper
chr7	111502762	111503653	Distal Intergenic	0979	251E-05	137E-03	Cbx7	-24789	Hyper
chr8	43147644	43149296	Intron (NM_012572/24406, intron 2 of 20)	0881	252E-05	137E-03	Grik4	55781	Hyper
chr10	100827667	100828807	Exon (NM_001108304/360658, exon 12 of 16)	0897	253E-05	137E-03	Mrps7	-14884	Hyper
chr1	86150333	86153400	Exon (NM_017190/29409, exon 9 of 12)	0750	253E-05	137E-03	Mag	10240	Hyper
chr10	85893068	85896741	Promoter (<=1kb)	0651	255E-05	138E-03	Cavin1	0	Hyper

chr4	63547141	63553196	Promoter (<=1kb)	0479	256E-05	138E-03	Cyren	0	Hyper
chr14	72267774	72270206	Exon (NM_001014135/360950, exon 3 of 15)	0617	257E-05	139E-03	Wdr1	9742	Hyper
chr11	69769853	69778675	Distal Intergenic	0488	257E-05	139E-03	Acap2	231307	Hyper
chr2	137137911	137138422	Intron (NM_001109183/499615, intron 2 of 3)	1043	257E-05	139E-03	Lhfpl6	11426	Hyper
chr12	19600407	19601587	Promoter (<=1kb)	0735	260E-05	140E-03	Serpine1	0	Hyper
chr19	51525118	51525760	Intron (NM_001108456/361436, intron 8 of 16)	1116	260E-05	140E-03	Afg3l1	16506	Hyper
chr1	152081483	152082680	Distal Intergenic	0838	262E-05	141E-03	Aqp11	-24808	Hyper
chr5	146472593	146474076	Promoter (<=1kb)	0810	269E-05	143E-03	Fam110d	0	Hyper
chr10	89147532	89186087	Intron (NM_017212/29477, intron 1 of 9)	0356	272E-05	144E-03	Mapt	8884	Hyper
chr6	16684909	16686593	Exon (NM_053600/94269, exon 6 of 9)	0722	273E-05	145E-03	Fez2	30218	Hyper
chr12	40188228	40189729	Intron (NM_053612/113906, intron 2 of 2)	0841	276E-05	146E-03	Hspb8	11669	Hyper
chr19	33694924	33697394	Exon (NM_001135875/361396, exon 3 of 14)	0661	276E-05	146E-03	Ranbp10	19292	Hyper
chrX	9289422	9291736	Intron (NM_001100967/302516, intron 2 of 2)	0774	277E-05	147E-03	Nyx	10164	Hyper
chr15	18520625	18522953	Intron (NM_138528/171562, intron 1 of 15)	0659	278E-05	147E-03	Ero1a	7487	Hyper
chr9	44850955	44851972	Distal Intergenic	0936	279E-05	147E-03	Pantr1	91659	Hyper
chr17	30670942	30672569	Intron (NM_022624/64559, intron 4 of 10)	0837	282E-05	148E-03	Psmg4	45455	Hyper
chr5	161367202	161368124	Exon (NM_001277249/117287, exon 5 of 7)	0965	282E-05	148E-03	Park7	3998	Hyper
chr7	107913499	107917504	Promoter (<=1kb)	0500	282E-05	148E-03	Plec	0	Hyper
chr8	60065037	60070784	5' UTR	0424	284E-05	148E-03	Pkm	7197	Hyper
chr4	15124732	15126922	Intron (NM_053621/113970, intron 2 of 22)	0641	284E-05	148E-03	Magi2	743115	Hyper
chr19	33722109	33723610	Exon (NM_001109130/498944, exon 2 of 16)	0781	285E-05	149E-03	Tsnaxip1	5062	Hyper
chr3	131694655	131695469	Intron (NM_001106519/296201, intron 2 of 3)	0885	287E-05	150E-03	Ovol2	10935	Hyper
chr19	49152259	49153226	Promoter (<=1kb)	1097	289E-05	150E-03	Fendrr	0	Hyper
chr15	98959251	98965574	Promoter (<=1kb)	0457	291E-05	151E-03	Ubac2	0	Hyper
chr20	5826881	5832007	Exon (NM_001047939/499407, exon 19 of 23)	0450	291E-05	151E-03		18021	Hyper
chr6	100492607	100493851	Distal Intergenic	0685	292E-05	151E-03	Srsf5	-111630	Hyper
chr8	92986994	92988107	Promoter (<=1kb)	0709	296E-05	153E-03	Plscr4	0	Hyper
chr7	112886260	112888066	Promoter (<=1kb)	0537	297E-05	153E-03	Slc25a17	0	Hyper
chr18	54231414	54232670	Exon (NM_022672/29284, exon 4 of 1)	0873	300E-05	154E-03	Rps14	3560	Hyper
chr4	123200030	123214805	Distal Intergenic	0370	304E-05	155E-03	Slc41a3	72267	Hyper
chr6	131012487	131014120	Distal Intergenic	0719	306E-05	156E-03	LOC102548399	26800	Hyper
chr6	45381340	45382997	Distal Intergenic	0865	308E-05	157E-03	Eipr1	-5454	Hyper
chr8	42800841	42802627	Intron (NM_001014089/315591, intron 7 of 7)	0816	309E-05	157E-03	Tecta	-21134	Hyper

chr14	57046553	57047562	Distal Intergenic	0918	311E-05	158E-03	Stim2	-42374	Hyper
chr1	213613989	213614711	Intron (NM_031036/81666, intron 3 of 6)	1074	314E-05	159E-03	Gna14	-101309	Hyper
chr7	96070394	96071828	Distal Intergenic	0759	314E-05	159E-03	Asap1	-12025	Hyper
chr20	9591192	9591983	Exon (NM_001135667/690032, exon 7 of 11)	1016	316E-05	160E-03	Wdr4	11419	Hyper
chr11	82230701	82231442	Distal Intergenic	1049	317E-05	160E-03	Cldn5	-16453	Hyper
chr7	603190	603854	Distal Intergenic	1207	319E-05	161E-03	Rbms2	-3056	Hyper
chr7	108951490	108953673	Intron (NM_001079895/362950, intron 1 of 12)	0637	323E-05	162E-03	Rbfox2	100730	Hyper
chr8	49056178	49059534	Exon (NM_001013181/353227, exon 4 of 7)	0577	325E-05	163E-03	Nnmt	-109523	Hyper
chr6	34444329	34444645	Distal Intergenic	1430	325E-05	163E-03	Vsnl1	-284850	Hyper
chr9	90203227	90205326	Intron (NM_001108230/316611, intron 2 of 15)	0780	327E-05	164E-03	Agap1	15202	Hyper
chr16	77427285	77430208	Distal Intergenic	0761	328E-05	164E-03	Tex29	233908	Hyper
chr8	12740883	12742056	Exon (NM_138544/191571, exon 18 of 26)	0848	331E-05	165E-03	Mtnr1b	-88146	Hyper
chr3	12907312	12908979	Distal Intergenic	0712	336E-05	167E-03	Trub2	150852	Hyper
chr3	123261310	123262135	Exon (NM_024353/25031, exon 16 of 39)	1043	336E-05	167E-03	Lamp5	-110327	Hyper
chr10	63134999	63138014	Promoter (2-3kb)	0535	337E-05	167E-03	Sdf2	-2549	Hyper
chr1	82446712	82448940	Promoter (<=1kb)	0588	341E-05	168E-03	Cyp2t1	0	Hyper
chr16	7156735	7158034	Distal Intergenic	1053	341E-05	168E-03	Oxnad1	-135436	Hyper
chr5	135486802	135490121	Exon (NM_001107976/313574, exon 6 of 6)	0532	342E-05	168E-03	Nt5c1a	13571	Hyper
chr10	83134291	83135039	Intron (NM_022272/64039, intron 1 of 9)	1161	345E-05	170E-03	Fbxl20	7535	Hyper
chr13	91584614	91588286	Distal Intergenic	0531	346E-05	170E-03	Ahctf1	-48223	Hyper
chr13	45681221	45683398	Intron (NM_017155/29290, intron 1 of 1)	0664	347E-05	170E-03	Adora1	11404	Hyper
chrX	9301726	9302916	Promoter (<=1kb)	0805	347E-05	171E-03	Nyx	0	Hyper
chr1	79485223	79485964	Promoter (<=1kb)	1064	348E-05	171E-03	Bcl3	0	Hyper
chr5	145317691	145319859	Distal Intergenic	0590	350E-05	171E-03	Wasf2	-58716	Hyper
chr13	63816752	63817581	Intron (NM_022242/63912, intron 12 of 13)	1022	350E-05	172E-03	Edem3	-41135	Hyper
chr7	114762775	114763982	3' UTR	0937	351E-05	172E-03	Ttll12	-11538	Hyper
chr19	48334086	48335590	Distal Intergenic	0798	352E-05	172E-03	6430548M08Rikl	135877	Hyper
chr12	11793756	11794684	Intron (NM_001107123/304302, intron 4 of 29)	0953	355E-05	173E-03	Tnrc18	38364	Hyper
chr9	40422911	40427555	Intron (NM_001191887/363220, intron 15 of 21)	0414	358E-05	175E-03	Rev1	-97082	Hyper
chr15	78375722	78377005	Distal Intergenic	0901	361E-05	176E-03	Commd6	97377	Hyper
chr6	42518588	42519298	Distal Intergenic	1037	361E-05	176E-03	Rnf144a	500412	Hyper
chr19	52855129	52858549	Promoter (<=1kb)	0422	361E-05	176E-03	Exoc8	0	Hyper
chr20	9586376	9591110	3' UTR	0484	361E-05	176E-03	Wdr4	12292	Hyper

chr1	185283471	185285095	Intron (NM_001004415/309025, intron 7 of 21)	0803	364E-05	177E-03	Btbd16	-50634	Hyper
chr5	71439929	71442060	Distal Intergenic	0646	370E-05	179E-03	Actl7b	4860	Hyper
chr1	160078781	160084829	3' UTR	0461	371E-05	179E-03	Rrp8	3811	Hyper
chr1	86200211	86202715	Promoter (<=1kb)	0756	372E-05	179E-03	Lsr	0	Hyper
chr1	11969251	11970624	Distal Intergenic	0663	375E-05	181E-03	Cited2	-341802	Hyper
chr10	16564681	16566073	Distal Intergenic	0858	376E-05	181E-03	Atp6v0e1	-61949	Hyper
chr12	31785067	31787276	Distal Intergenic	0758	377E-05	181E-03	Ccdc92	-9408	Hyper
chr5	63310536	63311146	Distal Intergenic	1093	377E-05	181E-03	Plppr1	-280887	Hyper
chr2	193244844	193245263	Intron (NM_001005765/295347, intron 1 of 7)	1212	382E-05	183E-03	Rap1a	41210	Hyper
chr7	8680600	8682033	Exon (NM_001008356/314637, exon 4 of 4)	0881	384E-05	183E-03	Slc39a3	3755	Hyper
chr8	33631211	33632953	Distal Intergenic	0756	386E-05	184E-03	Rpusd4	13827	Hyper
chr6	100475413	100476693	Distal Intergenic	0740	386E-05	184E-03	Srsf5	-128788	Hyper
chr20	7327315	7329482	Intron (NM_199379/294311, intron 1 of 8)	0784	387E-05	184E-03	RGD735065	4724	Hyper
chr6	95654237	95656765	Intron (NM_022210/60661, intron 2 of 4)	0585	387E-05	184E-03	Max	5201	Hyper
chr10	98188763	98190394	Distal Intergenic	0639	389E-05	185E-03	Mir297	26150	Hyper
chr6	72844085	72845734	Distal Intergenic	0674	391E-05	185E-03	Nfkbia	16207	Hyper
chr9	14514531	14518053	Intron (NM_001271230/316229, intron 12 of 13)	0666	392E-05	186E-03	Ttbk1	17779	Hyper
chr7	113047195	113048695	Distal Intergenic	0707	394E-05	186E-03	Rbx1	56360	Hyper
chr14	103908459	103911886	Intron (NM_001013130/305614, intron 2 of 36)	0505	395E-05	186E-03	Sptbn1	96621	Hyper
chr12	27391623	27392487	Distal Intergenic	1021	395E-05	186E-03	Sfswap	-211739	Hyper
chr6	127222360	127223170	Distal Intergenic	1253	396E-05	187E-03	Cyp46a1	-20145	Hyper
chr14	35038932	35041551	Distal Intergenic	0603	398E-05	187E-03	Ociad1	-123693	Hyper
chr10	62328287	62332942	Distal Intergenic	0527	403E-05	189E-03	Git1	-9323	Hyper
chr7	20398257	20403174	Intron (NM_001108742/362861, intron 1 of 10)	0491	404E-05	189E-03	Slc41a2	14543	Hyper
chr12	20794929	20795705	Promoter (<=1kb)	0954	404E-05	189E-03	Hspb1	0	Hyper
chr7	1359629	1362477	Promoter (<=1kb)	0670	405E-05	189E-03	Itga7	0	Hyper
chr1	82789536	82790099	Promoter (2-3kb)	1120	410E-05	191E-03	Prx	2719	Hyper
chr4	129754562	129756477	Promoter (<=1kb)	0508	412E-05	192E-03	Eogt	81	Hyper
chr11	65573008	65576150	Intron (NM_022600/64532, intron 1 of 20)	0555	414E-05	192E-03	Adcy5	41951	Hyper
chrX	104184861	104185509	Distal Intergenic	1057	417E-05	194E-03	Tsc22d3	36014	Hyper
chr6	10771193	10772031	Distal Intergenic	1118	421E-05	195E-03	Haao	-73754	Hyper
chr13	94196496	94198113	Promoter (2-3kb)	0785	421E-05	195E-03	Capn2	2856	Hyper
chr16	17574894	17575888	Distal Intergenic	0812	421E-05	195E-03	Ap1m1	26462	Hyper

chr8	45812858	45814112	Intron (NM_001108141/315615, intron 3 of 32)	0803	425E-05	196E-03	Dscaml1	72560	Hyper
chr3	90481287	90485350	Promoter (<=1kb)	0422	425E-05	196E-03	C3h11orf91	0	Hyper
chr7	27821392	27823326	Distal Intergenic	0857	426E-05	196E-03	Elk3	-17308	Hyper
chr6	132226707	132228834	Promoter (<=1kb)	0485	427E-05	197E-03	Crip1	0	Hyper
chr10	49752773	49754857	Distal Intergenic	0703	428E-05	197E-03	Elac2	120359	Hyper
chr7	9631380	9633277	Intron (NM_001108068/314619, intron 5 of 32)	0701	429E-05	198E-03	Gpx4	19235	Hyper
chr17	23109177	23113561	Distal Intergenic	0536	431E-05	198E-03	Tmem170b	56002	Hyper
chr19	52396874	52402354	Promoter (<=1kb)	0390	432E-05	199E-03	Pgbd5	0	Hyper
chr18	53945825	53950029	Intron (NM_173126/286910, intron 1 of 1)	0472	433E-05	199E-03	Smim3	6911	Hyper
chr6	56989749	56990799	Distal Intergenic	0873	433E-05	199E-03	Arl4a	93357	Hyper
chr9	45411566	45413629	Intron (NM_031677/63839, intron 1 of 4)	0601	439E-05	200E-03	Fhl2	4930	Hyper
chr14	43105117	43106335	Promoter (<=1kb)	0576	439E-05	201E-03	Wdr19	0	Hyper
chr20	7382506	7385782	3' UTR	0463	440E-05	201E-03	Pi16	6059	Hyper
chr18	54232947	54234041	Distal Intergenic	0661	441E-05	201E-03	Rps14	5093	Hyper
chr2	97724846	97725690	Distal Intergenic	0862	442E-05	201E-03	Hnf4g	310902	Hyper
chr15	3702870	3712311	Intron (NM_001008863/408223, intron 2 of 19)	0473	442E-05	201E-03	Usp54	10216	Hyper
chr10	81950080	81951644	5' UTR	0752	444E-05	202E-03	Sp2	18904	Hyper
chr11	75710990	75714535	Distal Intergenic	0439	444E-05	202E-03	Mir28	261073	Hyper
chr8	57949452	57950989	Exon (NM_001004081/300741, exon 6 of 8)	0902	449E-05	203E-03	Mpi	5124	Hyper
chr17	53226199	53226871	Distal Intergenic	1096	451E-05	204E-03	Mtpap	114610	Hyper
chr7	107440331	107442524	3' UTR	0514	453E-05	205E-03	Rhpn1	48347	Hyper
chr7	9123488	9130123	Promoter (<=1kb)	0402	455E-05	205E-03	Abhd17a	0	Hyper
chr10	10784310	10785943	Intron (NM_001008360/360480, intron 2 of 6)	0685	456E-05	205E-03	Cdip1	9605	Hyper
chr9	65501194	65504150	Distal Intergenic	0437	456E-05	205E-03	Klf7	-16474	Hyper
chr14	34027671	34028974	Distal Intergenic	0783	458E-05	206E-03	Rasl11b	-8525	Hyper
chr4	65441410	65442979	Intron (NM_198782/688705, intron 28 of 32)	0657	458E-05	206E-03	Ptn	-66104	Hyper
chr13	78427125	78428056	Distal Intergenic	0809	458E-05	206E-03	Gpa33	45984	Hyper
chr5	18193869	18196507	Distal Intergenic	0569	459E-05	206E-03	Bpnt2	-391299	Hyper
chr11	82280058	82280786	Distal Intergenic	0778	460E-05	206E-03	Cldn5	-65810	Hyper
chr4	59972646	59974347	Distal Intergenic	0637	460E-05	206E-03	Mkln1	-28151	Hyper
chr17	85117998	85118860	Exon (NM_024397/79249, exon 6 of 11)	0963	462E-05	207E-03	Abi1	60893	Hyper
chr3	7700714	7704028	Intron (NM_001108572/362078, intron 1 of 25)	0539	462E-05	207E-03	Ehmt1	24909	Hyper
chr6	21658043	21662572	Intron (NM_138890/192249, intron 1 of 5)	0538	462E-05	207E-03	Ehd3	3029	Hyper

chr6	42063446	42064239	Distal Intergenic	0976	464E-05	207E-03	Id2	-321053	Hyper
chr19	49633484	49643757	Exon (NM_001044259/498959, exon 2 of 9)	0413	469E-05	209E-03	Fbxo31	12253	Hyper
chr9	39850615	39851914	Distal Intergenic	0819	470E-05	209E-03	Mgat4a	-83725	Hyper
chr14	83219501	83220260	Intron (NM_001170459/360980, intron 18 of 28)	1216	472E-05	210E-03	Tns3	123878	Hyper
chr19	50522955	50525339	Promoter (<=1kb)	0694	472E-05	210E-03	Snai3	0	Hyper
chr7	113345238	113347359	Distal Intergenic	0581	473E-05	210E-03	Tef	18527	Hyper
chrX	71278604	71279289	Exon (NM_053291/24644, exon 4 of 11)	1173	473E-05	210E-03	Pgk1	7150	Hyper
chr5	58005930	58006627	Distal Intergenic	1179	475E-05	210E-03	Or13c7d	13938	Hyper
chr3	13533903	13541259	Promoter (<=1kb)	0384	475E-05	210E-03	Phyhd1	0	Hyper
chr5	15037739	15038146	Distal Intergenic	1428	477E-05	211E-03	Sox17	21008	Hyper
chr7	129754450	129756705	Intron (NM_012821/25289, intron 2 of 21)	0641	478E-05	211E-03	Adcy6	6246	Hyper
chr14	43491734	43494412	Promoter (2-3kb)	0550	479E-05	211E-03	Klf3	2797	Hyper
chr17	44685965	44687974	Intron (NM_001108415/361251, intron 4 of 21)	0776	480E-05	212E-03	Elmo1	134814	Hyper
chr20	45368196	45370995	Intron (NM_001106396/294518, intron 1 of 7)	0682	483E-05	213E-03	LOC499469	32399	Hyper
chr17	76667160	76669904	Promoter (<=1kb)	0696	483E-05	213E-03	Vim	0	Hyper
chr16	76706866	76709779	Exon (NM_001107324/306600, exon 5 of 29)	0680	484E-05	213E-03	Atp11a	57861	Hyper
chr16	44994213	44996182	Intron (NM_001134863/306459, intron 1 of 3)	0527	485E-05	213E-03	Stox2	19682	Hyper
chr17	18660673	18663774	Distal Intergenic	0464	488E-05	214E-03	Atxn1	-73759	Hyper
chr9	93593479	93597182	Distal Intergenic	0569	490E-05	214E-03	Aqp12a	38952	Hyper
chr11	34219296	34221620	Intron (NM_013192/25743, intron 2 of 5)	0495	492E-05	214E-03	Kcnj6	87138	Hyper
chr8	110341338	110343465	Intron (NM_001100966/301024, intron 4 of 23)	0543	494E-05	215E-03	Scap	35307	Hyper
chr12	31278857	31281006	Distal Intergenic	0611	495E-05	215E-03	Scarb1	-15150	Hyper
chr12	32294250	32294833	Intron (NM_001170554/288654, intron 6 of 22)	1136	499E-05	217E-03	Mphosph9	18326	Hyper
chr3	130787883	130788647	Distal Intergenic	0881	505E-05	218E-03	Pcsk2	-91775	Hyper
chr20	27935079	27937679	Distal Intergenic	0588	508E-05	219E-03	Ascc1	-3721	Hyper
chr3	7741221	7742802	Promoter (<=1kb)	0578	508E-05	219E-03	Arrdc1	0	Hyper
chr17	53268342	53269602	Distal Intergenic	0969	508E-05	219E-03	Mtpap	71879	Hyper
chr12	25593637	25595955	Intron (NM_001025112/288611, intron 5 of 10)	0603	514E-05	221E-03	Galnt17	269769	Hyper
chr14	103619533	103620305	Distal Intergenic	1072	515E-05	221E-03	Rtn4	169343	Hyper
chr6	7865666	7871751	Promoter (<=1kb)	0480	515E-05	221E-03	Epas1	0	Hyper
chr1	188518597	188521336	Exon (NM_001108505/361666, exon 3 of 7)	0524	518E-05	221E-03	Bccip	6053	Hyper
chr1	78495841	78496792	Promoter (<=1kb)	0871	521E-05	223E-03	Micb	62	Hyper
chr19	35928173	35930082	Distal Intergenic	0702	522E-05	223E-03	Psmd7	-426568	Hyper

chr17	32840428	32844238	Distal Intergenic	0770	523E-05	223E-03	Foxq1	71139	Hyper
chr12	15657066	15661890	Exon (NM_012801/25266, exon 6 of 8)	0567	526E-05	223E-03	Pdgfa	11517	Hyper
chr8	110650789	110652562	Intron (NM_001108783/363151, intron 1 of 6)	0584	529E-05	224E-03	Ccdc12	15079	Hyper
chr10	64159537	64160783	Distal Intergenic	0791	530E-05	224E-03	Wsb1	45674	Hyper
chr8	76351131	76357754	Promoter (<=1kb)	0434	532E-05	225E-03	Tmod2	0	Hyper
chr6	120046047	120050006	Distal Intergenic	0579	537E-05	226E-03	Dglucy	-5489	Hyper
chr6	7850188	7861275	Intron (NM_023090/29452, intron 1 of 16)	0442	537E-05	226E-03	Epas1	9971	Hyper
chr17	45617645	45618667	Distal Intergenic	0799	537E-05	226E-03	Stard3nl	34367	Hyper
chr12	35775476	35775941	Distal Intergenic	1248	537E-05	226E-03	Oas3	-3177	Hyper
chr6	127683485	127685849	Distal Intergenic	0570	538E-05	226E-03	Yy1	-21789	Hyper
chr9	90185717	90192316	Promoter (<=1kb)	0484	538E-05	226E-03	Agap1	0	Hyper
chr1	46512410	46514680	Distal Intergenic	0678	544E-05	228E-03	Synj2	-4029	Hyper
chr6	72815758	72817133	Distal Intergenic	0833	544E-05	228E-03	Psma6	36549	Hyper
chr3	10554968	10555867	Exon (NM_053664/114123, exon 10 of 21)	1061	545E-05	228E-03	Sardh	17706	Hyper
chr3	141906009	141916039	Distal Intergenic	0435	546E-05	228E-03	Kif3b	147543	Hyper
chr6	39384191	39384982	Distal Intergenic	0980	552E-05	230E-03	Lpin1	-4015	Hyper
chr8	103507855	103509942	Distal Intergenic	0611	556E-05	231E-03	Slco2a1	-78974	Hyper
chr1	33796967	33797960	3' UTR	0800	557E-05	232E-03	Tent4a	30329	Hyper
chr6	95498877	95500821	Promoter (1-2kb)	0706	566E-05	234E-03	Gpx2	-2000	Hyper
chr12	35382377	35384367	Exon (NM_001177593/25622, exon 2 of 16)	0694	567E-05	234E-03	Ptpn11	16941	Hyper
chr14	94201008	94203684	Intron (NM_001047094/305539, intron 1 of 5)	0526	567E-05	234E-03	Spred2	51314	Hyper
chr1	117493100	117500813	Distal Intergenic	0414	570E-05	235E-03	Klf13	35813	Hyper
chr20	6540369	6542659	Promoter (<=1kb)	0576	574E-05	236E-03	Fkbp5	0	Hyper
chr2	185856574	185857270	Distal Intergenic	0928	575E-05	237E-03	Hmgcs2	-18364	Hyper
chr2	101084693	101085521	Distal Intergenic	0757	579E-05	238E-03	Cyp7b1	-414995	Hyper
chr1	245755116	245756878	Exon (NM_001011942/294014, exon 6 of 8)	0645	581E-05	238E-03	Cnnm2	111348	Hyper
chr10	68918370	68920606	Intron (NM_053419/84479, intron 20 of 22)	0649	589E-05	241E-03		11076	Hyper
chr8	89243390	89244696	Distal Intergenic	0791	593E-05	242E-03	Nt5e	-26350	Hyper
chr3	156303838	156304080	Distal Intergenic	1601	597E-05	244E-03	Ube2v1	36118	Hyper
chr17	58294554	58297921	Exon (NM_030864/81522, exon 27 of 33)	0489	599E-05	244E-03	Mtr	74483	Hyper
chr17	51784150	51785507	Distal Intergenic	0679	599E-05	244E-03	Arhgap12	195308	Hyper
chr12	38598726	38600409	Intron (NM_001108338/360818, intron 3 of 11)	0691	603E-05	246E-03	Fbxo21	4724	Hyper
chr6	45329999	45336975	Distal Intergenic	0391	603E-05	246E-03	Adi1	23111	Hyper

chr5	132732184	132738172	Intron (NM_138827/24778, intron 2 of 9)	0435	604E-05	246E-03	Slc2a1	14985	Hyper
chr12	42741295	42746178	Promoter (<=1kb)	0472	608E-05	247E-03	Coro1c	429	Hyper
chr13	46203216	46204627	3' UTR	0905	609E-05	247E-03	Syt2	17934	Hyper
chr17	66459045	66460156	Distal Intergenic	0996	610E-05	247E-03	Calml3	37412	Hyper
chr20	6006484	6008596	Intron (NM_001107613/309639, intron 1 of 23)	0592	610E-05	247E-03	Anks1a	42806	Hyper
chr2	112910748	112912310	Intron (NM_001106423/294924, intron 1 of 16)	0785	611E-05	247E-03	Mecom	36264	Hyper
chr1	90445754	90449113	Distal Intergenic	0591	612E-05	248E-03	Uri1	255543	Hyper
chr6	26395658	26397335	Intron (NM_053486/85248, intron 5 of 7)	0711	612E-05	248E-03	Kif3c	28566	Hyper
chr17	57454487	57455466	Intron (NM_001191833/361261, intron 1 of 9)	0949	613E-05	248E-03	Ccny	57394	Hyper
chr12	38134520	38137019	Distal Intergenic	0650	614E-05	248E-03	Spring1	192119	Hyper
chr14	5940385	5942480	Intron (NM_001107206/305152, intron 1 of 19)	0590	615E-05	248E-03	Aff1	21471	Hyper
chr6	94034816	94036260	Promoter (<=1kb)	0608	621E-05	250E-03	Rhoj	0	Hyper
chr1	187545248	187548347	Exon (NM_001107556/309060, exon 4 of 5)	0483	624E-05	251E-03	Fam53b	42839	Hyper
chr5	151515194	151517076	Intron (NM_001005903/298584, intron 4 of 8)	0675	626E-05	251E-03	Slc66a1	32749	Hyper
chr1	222316065	222317479	Promoter (<=1kb)	0867	628E-05	252E-03	Tmem252	0	Hyper
chr13	66220356	66221074	Distal Intergenic	1079	628E-05	252E-03	Glul	194715	Hyper
chr8	108238794	108245547	Promoter (<=1kb)	0423	629E-05	252E-03	Hyal2	0	Hyper
chr1	152958820	152963636	Distal Intergenic	0451	632E-05	252E-03	Thap12	-39969	Hyper
chr5	142515177	142516620	Exon (NM_053582/94174, exon 5 of 12)	0863	638E-05	254E-03	Tinagl1	6470	Hyper
chr17	27176773	27180302	Promoter (<=1kb)	0470	640E-05	254E-03	LOC100909970	0	Hyper
chr2	225319978	225321703	Intron (NM_017041/24674, intron 2 of 13)	0600	642E-05	255E-03	Ppp3ca	153867	Hyper
chr6	24369061	24370974	3' UTR	0812	644E-05	256E-03	Fosl2	-49904	Hyper
chr3	130014110	130014630	Intron (NM_001107783/311478, intron 24 of 25)	1078	647E-05	256E-03	Kif16b	239389	Hyper
chr3	167306802	167309510	Intron (NM_001191609/140433, intron 2 of 78)	0724	648E-05	257E-03	Lama5	8860	Hyper
chr19	10826206	10827815	Promoter (<=1kb)	0620	652E-05	258E-03	Mt1	0	Hyper
chr2	113402409	113404064	Intron (NM_001106423/294924, intron 2 of 16)	0792	653E-05	258E-03	Mecom	527925	Hyper
chr19	48276263	48281035	Distal Intergenic	0419	655E-05	258E-03	6430548M08Rikl	78054	Hyper
chr15	39072917	39078060	Distal Intergenic	0455	665E-05	261E-03	Kif13b	164599	Hyper
chr10	85981751	85985152	Exon (NM_031604/29757, exon 20 of 22)	0457	665E-05	261E-03	Hsd17b1	-24576	Hyper
chr5	15016705	15017904	Promoter (<=1kb)	0905	666E-05	261E-03	Sox17	0	Hyper
chr17	6887763	6891052	Intron (NM_001271297/306759, intron 2 of 10)	0532	669E-05	262E-03	Spock1	146250	Hyper
chr3	107096261	107098468	Intron (NM_139324/192204, intron 2 of 5)	0698	672E-05	262E-03	Ehd4	23517	Hyper
chr2	104675148	104676261	Distal Intergenic	0822	674E-05	263E-03	Tbl1xr1	-125460	Hyper

chr11	33040335	33046112	Intron (NM_001191660/304077, intron 1 of 35)	0419	674E-05	263E-03	Dop1b	12286	Hyper
chr20	10221962	10222720	Exon (NM_031769/83578, exon 2 of 11)	0850	679E-05	264E-03	Pdxk	11569	Hyper
chr17	4915176	4917367	3' UTR	0662	686E-05	266E-03	Isca1	9885	Hyper
chr2	61070417	61070808	Distal Intergenic	1174	688E-05	267E-03	Sub1	-50045	Hyper
chr3	153623489	153633054	3' UTR	0397	692E-05	268E-03	Zfp335	4084	Hyper
chr10	10908898	10917606	Exon (NM_001191639/192276, exon 8 of 28)	0368	694E-05	269E-03	Vasn	10653	Hyper
chr2	78165551	78167129	Intron (NM_053714/114506, intron 1 of 11)	0655	699E-05	270E-03	Ankh	12525	Hyper
chr14	41537382	41537922	Distal Intergenic	1182	702E-05	271E-03	Uchl1	-41792	Hyper
chr18	15625437	15625977	Distal Intergenic	1158	702E-05	271E-03	Galnt1	97231	Hyper
chr14	81460601	81468053	3' UTR	0454	702E-05	271E-03	Nacad	5728	Hyper
chr19	428094	428940	Distal Intergenic	0942	704E-05	271E-03	Car7	9527	Hyper
chr5	154517947	154519302	Intron (NM_001014070/313672, intron 1 of 7)	0731	708E-05	272E-03	Tmem51	-142065	Hyper
chr11	68109345	68111572	Distal Intergenic	0585	711E-05	273E-03	Tnk2	42437	Hyper
chr2	183433920	183435186	Distal Intergenic	0836	712E-05	273E-03	Aph1a	-3257	Hyper
chr5	110436593	110438198	Intron (NM_001013932/298250, intron 2 of 15)	0728	714E-05	274E-03	Fggy	37722	Hyper
chr8	90590678	90591987	Distal Intergenic	0783	722E-05	276E-03	Ctsh	-16954	Hyper
chr16	3107849	3110486	Intron (NM_170787/259269, intron 4 of 16)	0551	730E-05	279E-03	Erc2	259171	Hyper
chr16	55993834	55995140	Distal Intergenic	0791	732E-05	279E-03	Prag1	146013	Hyper
chr10	60281766	60283530	Promoter (1-2kb)	0638	733E-05	279E-03	Serpinf2	-1282	Hyper
chr5	155066882	155070321	Distal Intergenic	0471	741E-05	282E-03	Kazn	-287973	Hyper
chr18	23562979	23564512	Intron (NM_001012163/361303, intron 1 of 8)	0698	742E-05	282E-03	Lims2	9042	Hyper
chr2	11370187	11371902	Distal Intergenic	0944	742E-05	282E-03	Arrdc3	232487	Hyper
chr17	30553103	30554936	Intron (NM_022624/64559, intron 1 of 10)	0682	745E-05	283E-03	Slc22a23	8997	Hyper
chr1	150976788	150977846	Intron (NM_001191628/308831, intron 4 of 31)	0786	745E-05	283E-03	Tenm4	196407	Hyper
chr19	48634180	48635155	Exon (NM_001106190/292058, exon 3 of 5)	1188	752E-05	285E-03	Gins2	4184	Hyper
chr3	9407539	9411260	Promoter (<=1kb)	0581	753E-05	285E-03	Egfl7	0	Hyper
chr2	195465904	195466979	Distal Intergenic	0919	761E-05	287E-03	Eps8l3	-47713	Hyper
chr10	82544568	82548052	Exon (NM_019378/56029, exon 2 of 18)	0509	766E-05	289E-03	Srcin1	20647	Hyper
chr1	96047556	96048463	Exon (NM_022400/64203, exon 2 of 11)	1044	767E-05	289E-03	Bcat2	4885	Hyper
chr10	10676794	10680721	Intron (NM_001013964/302938, intron 1 of 16)	0477	772E-05	291E-03	Mgrn1	7575	Hyper
chr4	117884624	117886070	Distal Intergenic	0809	775E-05	292E-03	Sfxn5	-14842	Hyper
chr5	132633732	132634860	Distal Intergenic	0912	775E-05	292E-03	Lao1	60034	Hyper
chr16	74721222	74733728	Distal Intergenic	0426	777E-05	292E-03	Cln8	25825	Hyper

chr1	252361909	252368437	Intron (NM_013160/25701, intron 3 of 5)	0387	792E-05	296E-03	Mxi1	18624	Hyper
chr8	76309696	76311041	Promoter (<=1kb)	0580	792E-05	296E-03	Tmod3	0	Hyper
chr1	40410201	40412990	Exon (NM_001190999/679812, exon 10 of 18)	0611	793E-05	296E-03	Mthfd1l	-31018	Hyper
chr4	63559772	63561572	Exon (NM_001127298/312225, exon 12 of 15)	0753	793E-05	296E-03	Cyren	-8170	Hyper
chr11	82297691	82302112	Distal Intergenic	0611	801E-05	298E-03	Septin5	-71489	Hyper
chr6	98683869	98685130	Distal Intergenic	0770	813E-05	301E-03	Zfp36l1	250618	Hyper
chr2	184089760	184098205	Promoter (<=1kb)	0391	813E-05	301E-03	Txnip	0	Hyper
chr5	116257990	116259737	Exon (NM_001107949/313409, exon 13 of 18)	0645	813E-05	301E-03	Leprot	-30106	Hyper
chr7	90547500	90549704	Intron (NM_001130563/362918, intron 4 of 15)	0630	814E-05	301E-03	Ndufb9	66487	Hyper
chr11	82653052	82654538	Intron (NM_001108323/360738, intron 1 of 8)	0664	815E-05	301E-03	Tango2	3477	Hyper
chr12	44354168	44355219	Intron (NM_001008508/288719, intron 1 of 6)	0865	818E-05	302E-03	Tpst2	7110	Hyper
chr10	52514665	52516031	Distal Intergenic	0756	820E-05	302E-03	Dhrs7c	19403	Hyper
chr1	56371718	56372584	Intron (NM_001107466/308218, intron 6 of 9)	0931	821E-05	302E-03	Fam120b	32589	Hyper
chr3	22537806	22540394	Distal Intergenic	0549	824E-05	303E-03	Nr5a1	-51478	Hyper
chr8	106834559	106839469	Downstream (<=300bp)	0396	824E-05	303E-03	Twf2	-5784	Hyper
chr7	111289110	111293386	Distal Intergenic	0418	824E-05	303E-03	Dnal4	31872	Hyper
chr5	150130145	150134137	Exon (NM_053596/94204, exon 3 of 19)	0547	829E-05	304E-03	Ece1	52466	Hyper
chr3	11070997	11073302	Exon (NM_012805/25271, exon 9 of 10)	0531	836E-05	307E-03	Rxra	81081	Hyper
chr5	139132116	139134349	Exon (NM_001159655/100294508, exon 17 of 21)	0537	838E-05	307E-03	LOC100294508	84078	Hyper
chr14	3175848	3176550	Intron (NM_001139484/305127, intron 8 of 9)	1012	847E-05	309E-03	Zfp644	70832	Hyper
chr7	69903046	69904602	Distal Intergenic	0666	847E-05	309E-03	Baalc	-14396	Hyper
chr3	152314184	152315460	Exon (NM_001008312/296350, exon 3 of 10)	0712	847E-05	309E-03	Serinc3	5867	Hyper
chr3	141533535	141535048	Distal Intergenic	0759	858E-05	312E-03	Xkr7	32251	Hyper
chr10	104561525	104566865	Promoter (2-3kb)	0488	861E-05	313E-03	Eif4a3	-2493	Hyper
chr12	41478754	41480780	Promoter (<=1kb)	0562	868E-05	315E-03	Unc119b	0	Hyper
chr12	8766280	8767993	Intron (NM_001109061/498140, intron 4 of 12)	0581	875E-05	317E-03	Cdk8	36184	Hyper
chr4	148330470	148331144	Intron (NM_019159/29179, intron 10 of 10)	0916	878E-05	317E-03	Timp4	-17912	Hyper
chr9	92033064	92038294	Exon (NM_031678/63840, exon 5 of 23)	0391	878E-05	317E-03	Per2	11165	Hyper
chr10	64026994	64028294	Intron (NM_001382488/108348076, intron 1 of 17)	0774	883E-05	318E-03	Ksr1	69782	Hyper
chr17	6236311	6237758	Distal Intergenic	0736	884E-05	319E-03	Hnrnpk	-28054	Hyper
chr17	31474599	31475467	Promoter (<=1kb)	1018	888E-05	319E-03	Serpinb6b	0	Hyper
chr14	74283454	74285050	Intron (NM_001170455/289717, intron 1 of 1)	0715	889E-05	319E-03	Tada2b	3980	Hyper
chr3	77117740	77118757	Intron (NM_053585/94193, intron 31 of 33)	0954	892E-05	320E-03	Mybpc3	22488	Hyper

chr1	95301603	95304165	Promoter (2-3kb)	0498	899E-05	322E-03	Nup62	2067	Hyper
chr4	59879004	59883538	Distal Intergenic	0402	905E-05	324E-03	Mkln1	-118960	Hyper
chr17	72151933	72152531	Distal Intergenic	0896	906E-05	324E-03	Echdc3	81236	Hyper
chr1	82788665	82789409	Promoter (1-2kb)	0901	910E-05	325E-03	Prx	1848	Hyper
chr3	16519277	16525325	Promoter (2-3kb)	0396	910E-05	325E-03	Angptl2	2092	Hyper
chr1	11319112	11319533	Distal Intergenic	1174	912E-05	325E-03	Cited2	-992893	Hyper
chr1	136228556	136232011	Exon (NM_031238/81921, exon 7 of 9)	0515	918E-05	327E-03	Adamtsl3	-56782	Hyper
chr8	49202197	49203049	Distal Intergenic	0905	920E-05	328E-03	Zbtb16	-25186	Hyper
chr16	18618391	18621366	Exon (NM_001170604/171333, exon 16 of 16)	0661	925E-05	329E-03	Il12rb1	11403	Hyper
chr3	149391875	149396378	Intron (NM_013187/25738, intron 1 of 31)	0571	929E-05	330E-03	Plcg1	6288	Hyper
chr3	7531856	7537931	Intron (NM_147141/257648, intron 3 of 45)	0422	932E-05	330E-03	Cacna1b	8026	Hyper
chr12	42506264	42507845	Exon (NM_001107153/304579, exon 8 of 13)	0675	933E-05	330E-03	Alkbh2	9970	Hyper
chr5	121715067	121718680	Intron (NM_053358/84354, intron 4 of 16)	0586	937E-05	332E-03	Ssbp3	23674	Hyper
chr5	162513239	162517676	Promoter (<=1kb)	0391	938E-05	332E-03	Klhl21	0	Hyper
chr2	192520687	192529905	Intron (NM_001107712/310760, intron 3 of 5)	0403	940E-05	332E-03	Cttnbp2nl	24643	Hyper
chr2	30523179	30524795	Distal Intergenic	0732	942E-05	333E-03	Ptcd2	215147	Hyper
chr5	161586855	161588139	Intron (NM_001195559/362665, intron 10 of 23)	0807	948E-05	334E-03	Vamp3	-78151	Hyper
chr1	203588444	203595564	Intron (NM_001011939/293692, intron 2 of 4)	0387	948E-05	334E-03	Ehd1	8594	Hyper
chr1	205598892	205601024	Distal Intergenic	0598	950E-05	334E-03	Slc3a2	9513	Hyper
chr11	62316756	62318727	Promoter (1-2kb)	0555	955E-05	336E-03	Pla1a	-1768	Hyper
chr19	853391	854376	Promoter (<=1kb)	0898	959E-05	336E-03	Cdh5	0	Hyper
chr10	91511506	91514564	Distal Intergenic	0589	962E-05	337E-03	Ern1	-119627	Hyper
chr9	61353337	61355782	Intron (NM_001108797/363236, intron 1 of 7)	0478	962E-05	337E-03	Fam117b	12384	Hyper
chr4	154384280	154385268	Distal Intergenic	0795	964E-05	338E-03	Pex26	-29581	Hyper
chr3	16051121	16052766	Promoter (2-3kb)	0689	964E-05	338E-03	Tor2a	2555	Hyper
chr1	185519176	185520122	Intron (NM_031721/65164, intron 1 of 8)	1076	969E-05	339E-03	Htra1	21361	Hyper
chr3	154847644	154851450	Intron (NM_001034927/311642, intron 3 of 20)	0497	972E-05	340E-03	Sulf2	52965	Hyper
chr12	38585471	38591317	Exon (NM_001108338/360818, exon 7 of 12)	0411	975E-05	340E-03	Fbxo21	13816	Hyper
chr10	72212640	72213436	Intron (NM_001129777/497976, intron 7 of 8)	0863	977E-05	341E-03	Rad51c	17765	Hyper
chr10	4676309	4683347	Promoter (<=1kb)	0440	982E-05	342E-03	Litaf	0	Hyper
chr9	79682490	79685019	Distal Intergenic	0651	988E-05	343E-03	Pax3	-18448	Hyper
chr12	32710081	32711581	Promoter (<=1kb)	0614	992E-05	344E-03	Hcar1	0	Hyper
chr3	165534492	165535029	Exon (NM_001191745/311693, exon 3 of 15)	1117	993E-05	344E-03	Cdh26	13448	Hyper

chr10	28002795	28004708	Intron (NM_001037773/303059, intron 1 of 4)	0710	100E-04	347E-03	Ccnjl	6494	Hyper
chr5	163937141	163941805	Intron (NM_001101014/687031, intron 2 of 5)	0440	100E-04	347E-03	Ajap1	78512	Hyper
chr10	16649134	16659749	Distal Intergenic	0428	101E-04	350E-03	Dusp1	-20740	Hyper
chr12	25888814	25889838	Intron (NM_001077201/363909, intron 4 of 5)	0837	102E-04	351E-03	Caln1	364232	Hyper
chr1	86924268	86927313	Distal Intergenic	0493	102E-04	351E-03	Garre1	-9832	Hyper
chr3	131308330	131308898	Intron (NM_001033666/502674, intron 3 of 3)	1217	102E-04	351E-03	Dstn	23683	Hyper
chr12	42485155	42486333	3' UTR	0740	102E-04	351E-03	Ung	7151	Hyper
chr6	124444600	124445945	Distal Intergenic	0884	102E-04	352E-03	Bdkrb2	-26621	Hyper
chr8	43151280	43155327	Intron (NM_012572/24406, intron 2 of 20)	0542	102E-04	352E-03	Grik4	49750	Hyper
chr5	144262686	144268257	Distal Intergenic	0363	103E-04	352E-03	Oprd1	72703	Hyper
chr1	19958463	19961992	Distal Intergenic	0476	103E-04	352E-03	Smlr1	121073	Hyper
chr6	10712190	10712719	Distal Intergenic	1240	103E-04	354E-03	Haao	-133066	Hyper
chr2	179220894	179222740	Promoter (<=1kb)	0605	103E-04	354E-03	S100a10	0	Hyper
chr10	86188204	86191145	Promoter (<=1kb)	0507	104E-04	355E-03	Ramp2	0	Hyper
chr19	51688244	51690061	Distal Intergenic	0707	104E-04	355E-03	Rab4a	-105552	Hyper
chr13	100586772	100589051	Distal Intergenic	0569	104E-04	355E-03	Kctd3	-38054	Hyper
chr8	65730810	65732519	Promoter (<=1kb)	0480	104E-04	356E-03	Parp16	0	Hyper
chr3	12920170	12925421	Distal Intergenic	0497	104E-04	356E-03	Trub2	134410	Hyper
chr3	119822512	119833264	Promoter (<=1kb)	0406	104E-04	356E-03	Gpcpd1	0	Hyper
chr7	130883155	130885578	Intron (NM_001108993/366984, intron 1 of 10)	0558	105E-04	357E-03	Cers5	12369	Hyper
chr3	118473195	118474627	Distal Intergenic	0675	105E-04	357E-03	Pank2	-8847	Hyper
chr8	107980550	107982042	Distal Intergenic	0696	106E-04	359E-03	Cish	8244	Hyper
chr4	158290338	158292218	Promoter (1-2kb)	0683	106E-04	360E-03	Cd9	-1202	Hyper
chr20	10165728	10166846	Distal Intergenic	0778	107E-04	361E-03	Pdxk	-43547	Hyper
chr4	148298983	148303535	Exon (NM_019159/29179, exon 4 of 11)	0479	107E-04	361E-03	Timp4	9023	Hyper
chr13	30607105	30608407	Intron (NM_053821/116546, intron 1 of 4)	0797	107E-04	361E-03	Ralb	15792	Hyper
chr6	49345816	49346280	Distal Intergenic	1148	107E-04	361E-03	Nampt	-79036	Hyper
chr10	81951848	81958216	Intron (NM_001107045/303499, intron 2 of 7)	0384	107E-04	361E-03	Sp2	12332	Hyper
chr1	201278066	201278834	Promoter (1-2kb)	0894	107E-04	361E-03	Acy3	-1017	Hyper
chr8	110319023	110320424	Intron (NM_001100966/301024, intron 1 of 23)	0733	109E-04	365E-03	Scap	12992	Hyper
chr15	29303202	29304760	Promoter (<=1kb)	0680	109E-04	365E-03	Nfatc4	-775	Hyper
chr1	199027255	199027808	Intron (NM_022389/64191, intron 7 of 8)	1162	109E-04	365E-03	Dhcr7	12174	Hyper
chr3	105743028	105746462	Distal Intergenic	0408	109E-04	366E-03	Phgr1	-11119	Hyper

chr16	69464605	69467267	Exon (NM_017223/29502, exon 9 of 11)	0506	109E-04	366E-03	Vdac3	29576	Hyper
chr8	45442082	45445167	Promoter (1-2kb)	0709	110E-04	366E-03	Scn4b	-1952	Hyper
chr8	46513503	46514962	Intron (NM_001271216/684112, intron 21 of 24)	0721	111E-04	370E-03	Apoa1	-12289	Hyper
chr3	79418794	79420170	Intron (NM_031797/83628, intron 1 of 9)	0830	111E-04	371E-03	Cd82	11188	Hyper
chr17	84475643	84476542	Distal Intergenic	0928	112E-04	372E-03	Gad2	-287088	Hyper
chr3	126143843	126145240	Distal Intergenic	0693	112E-04	372E-03	Btbd3	531150	Hyper
chr12	11642050	11643720	Distal Intergenic	0612	112E-04	373E-03	Actb	-19392	Hyper
chr12	9718832	9722187	Exon (NM_001109598/690516, exon 14 of 18)	0480	112E-04	374E-03	Smurf1	83523	Hyper
chr18	50991702	50993905	Distal Intergenic	0665	113E-04	375E-03	LOC100910189	-43043	Hyper
chr1	196420402	196423599	Exon (NM_031801/83632, exon 7 of 12)	0540	113E-04	375E-03	Tmem80	-11776	Hyper
chr16	19028443	19029353	Exon (NM_001047115/684527, exon 7 of 14)	0881	113E-04	375E-03	Comp	26231	Hyper
chr1	197084069	197088311	Distal Intergenic	0502	114E-04	377E-03	Mob2	61591	Hyper
chr10	44462362	44463611	Intron (NM_001371051/116504, intron 1 of 24)	0805	115E-04	380E-03	Mprip	8172	Hyper
chr7	108518049	108520936	Intron (NM_173122/500901, intron 1 of 11)	0454	115E-04	380E-03	Arhgap39	17895	Hyper
chr8	20003769	20010041	Exon (NM_013199/25751, exon 2 of 21)	0450	116E-04	381E-03	Dnm2	25319	Hyper
chr9	1545172	1546315	Distal Intergenic	0848	116E-04	382E-03	Vmac	3260	Hyper
chr1	90903241	90904030	Distal Intergenic	1099	116E-04	382E-03	Plekhf1	6077	Hyper
chr4	34384249	34385649	Distal Intergenic	0923	117E-04	385E-03	Sem1	306356	Hyper
chr1	203120421	203121906	Distal Intergenic	0821	118E-04	386E-03	Frmd8	41925	Hyper
chr5	131668557	131669101	Promoter (1-2kb)	1238	118E-04	386E-03	St3gal3	1194	Hyper
chr1	79088469	79088980	Intron (NM_001191071/680407, intron 8 of 16)	1310	119E-04	389E-03	Mark4	19576	Hyper
chr16	1274435	1275207	3' UTR	0813	119E-04	389E-03	Ppif	17238	Hyper
chr4	157871389	157873503	Distal Intergenic	0506	119E-04	389E-03	Acrbp	20233	Hyper
chr6	45319504	45320186	Distal Intergenic	0954	119E-04	389E-03	Adi1	12616	Hyper
chr1	118508000	118509424	Distal Intergenic	0605	119E-04	390E-03	Nsmce3	-105371	Hyper
chr14	86527946	86529925	Intron (NM_001109093/498416, intron 4 of 17)	0638	120E-04	390E-03	Ddc	-58771	Hyper
chr20	11401022	11401735	Distal Intergenic	1113	122E-04	396E-03	Pofut2	-23234	Hyper
chr7	19228610	19229255	Promoter (1-2kb)	1043	122E-04	397E-03	Ckap4	-1559	Hyper
chr15	27887725	27888256	Promoter (<=1kb)	1031	122E-04	397E-03	Mmp14	0	Hyper
chr7	88961096	88963960	Distal Intergenic	0557	123E-04	398E-03	Slc22a22	-210985	Hyper
chr15	34907941	34909536	Distal Intergenic	0716	123E-04	399E-03	C1qtnf9	-5793	Hyper
chr6	10737755	10741101	Distal Intergenic	0412	123E-04	399E-03	Haao	-104684	Hyper
chr16	79621370	79621748	Distal Intergenic	1120	124E-04	399E-03	Lig4	102902	Hyper

chr2	24556209	24557161	Intron (NM_031707/29546, intron 1 of 9)	0907	124E-04	401E-03	Homer1	12209	Hyper
chr12	34097463	34102509	Intron (NM_001110823/29693, intron 5 of 19)	0398	124E-04	401E-03	Atp2a2	24753	Hyper
chr11	82209592	82214245	Promoter (<=1kb)	0673	125E-04	401E-03	Cldn5	3	Hyper
chr5	160398098	160399152	Distal Intergenic	0885	125E-04	402E-03	Spsb1	-26836	Hyper
chr4	178317528	178319064	Distal Intergenic	0677	125E-04	402E-03	Lmntd1	77193	Hyper
chr9	14520368	14522197	Intron (NM_001271230/316229, intron 12 of 13)	0783	125E-04	402E-03	Ttbk1	23616	Hyper
chr4	52786513	52787788	Distal Intergenic	0642	126E-04	404E-03	Slc13a1	-139410	Hyper
chr15	17088398	17088944	Intron (NM_001107288/306204, intron 1 of 44)	1015	126E-04	404E-03	Flnb	6062	Hyper
chr1	154838418	154840621	Promoter (<=1kb)	0546	126E-04	404E-03	Ucp2	0	Hyper
chr8	108297393	108309602	Promoter (<=1kb)	0324	126E-04	404E-03	Gnai2	0	Hyper
chr4	120656801	120659314	Promoter (<=1kb)	0846	127E-04	406E-03	Gata2	0	Hyper
chr15	39773255	39775350	Distal Intergenic	0631	127E-04	407E-03	Scara5	-104704	Hyper
chr11	67854165	67856270	Intron (NM_001371837/303883, intron 1 of 20)	0559	127E-04	407E-03	Lrch3	15994	Hyper
chr1	83775932	83777160	Distal Intergenic	0697	128E-04	407E-03	Lrfn1	28640	Hyper
chr15	27888517	27889235	Promoter (<=1kb)	0990	128E-04	407E-03	Mmp14	722	Hyper
chr5	156746350	156751386	Promoter (<=1kb)	0384	128E-04	407E-03	Dhrs3	0	Hyper
chr13	43769563	43773347	3' UTR	0484	129E-04	409E-03	Nuak2	15709	Hyper
chr15	39310777	39314417	Intron (NM_020097/56819, intron 1 of 4)	0510	130E-04	412E-03	Extl3	5055	Hyper
chr16	18066074	18069796	Promoter (<=1kb)	0463	130E-04	413E-03	Ushbp1	0	Hyper
chr5	101638013	101642605	Intron (NM_031743/84550, intron 1 of 9)	0416	130E-04	413E-03	Slc24a2	96732	Hyper
chr8	93034867	93036102	Distal Intergenic	0877	131E-04	413E-03	Plscr4	47577	Hyper
chr3	117980453	117981657	Exon (NM_001109206/499891, exon 18 of 37)	0826	132E-04	416E-03	Slc4a11	-67779	Hyper
chr18	61317919	61319648	Distal Intergenic	0643	132E-04	416E-03	Ptpn2	-23364	Hyper
chr6	41646505	41648591	Exon (NM_053795/116478, exon 10 of 30)	0666	132E-04	416E-03	Kidins220	28211	Hyper
chr2	30577300	30585733	Distal Intergenic	0447	133E-04	418E-03	Ptcd2	154209	Hyper
chr1	151191779	151195990	Exon (NM_001191628/308831, exon 19 of 32)	0431	133E-04	419E-03	Nars2	-104512	Hyper
chr10	37112308	37113013	Intron (NM_001107000/303130, intron 3 of 14)	0949	133E-04	419E-03	Fstl4	200888	Hyper
chr3	88548266	88549890	Distal Intergenic	0745	134E-04	420E-03	Trim44	178747	Hyper
chr7	51865095	51866261	Intron (NM_053594/94202, intron 6 of 13)	0768	135E-04	425E-03	Ptprr	64896	Hyper
chr1	209476157	209482210	Promoter (<=1kb)	0379	136E-04	427E-03	Dtx4	0	Hyper
chr4	59893577	59894973	Distal Intergenic	0583	137E-04	428E-03	Mkln1	-107525	Hyper
chr18	4130633	4131779	Exon (NM_001012235/497198, exon 5 of 12)	0761	137E-04	430E-03	Impact	10503	Hyper
chr10	83271356	83275955	Intron (NM_147142/257650, intron 2 of 3)	0469	137E-04	430E-03		4024	Hyper

chr8	62862281	62865556	3' UTR	0469	138E-04	430E-03	Anp32a	33156	Hyper
chr6	131928244	131929794	Intron (NM_001170595/314480, intron 2 of 2)	0668	138E-04	430E-03	Gpr132	6151	Hyper
chr6	120050117	120051742	Distal Intergenic	0625	138E-04	431E-03	Dglucy	-3753	Hyper
chr10	83807189	83811226	Distal Intergenic	0474	139E-04	432E-03	Wipf2	-29266	Hyper
chr3	106988265	106991556	Exon (NM_001108589/362197, exon 15 of 31)	0537	139E-04	432E-03	Jmjd7	-6921	Hyper
chr9	90199266	90200810	Intron (NM_001108230/316611, intron 2 of 15)	0794	139E-04	433E-03	Agap1	11241	Hyper
chr3	141771981	141774202	Intron (NM_001106529/296284, intron 1 of 8)	0515	140E-04	435E-03	Kif3b	13515	Hyper
chr5	116216614	116221987	Exon (NM_001107949/313409, exon 2 of 18)	0387	141E-04	438E-03	Leprot	-67856	Hyper
chr17	33493246	33495425	Distal Intergenic	0651	142E-04	439E-03	Exoc2	-10897	Hyper
chr2	137129283	137131607	Promoter (2-3kb)	0538	142E-04	439E-03	Lhfpl6	2798	Hyper
chr4	104600073	104600917	Promoter (<=1kb)	0967	143E-04	441E-03	Capg	11	Hyper
chr4	119983002	119984069	Distal Intergenic	0824	143E-04	442E-03	Arhgap25	-8020	Hyper
chr5	163847763	163849622	Distal Intergenic	0563	143E-04	442E-03	Ajap1	170695	Hyper
chr1	109691911	109694430	Exon (NM_001141935/365266, exon 8 of 22)	0441	144E-04	443E-03	Atp10a	135129	Hyper
chr2	184200491	184201345	3' UTR	0717	144E-04	444E-03	Itga10	17622	Hyper
chr5	161074207	161076470	Intron (NM_053885/116665, intron 12 of 22)	0672	145E-04	445E-03	Slc45a1	52833	Hyper
chr11	82285063	82286002	Distal Intergenic	0989	145E-04	445E-03	Cldn5	-70815	Hyper
chr14	33997520	33998304	Intron (NM_001017499/498353, intron 7 of 8)	0905	145E-04	446E-03	Rasl11b	20842	Hyper
chr7	21141240	21143761	Intron (NM_001170605/362863, intron 2 of 14)	0571	146E-04	446E-03	Ttc41	9009	Hyper
chr3	10751878	10754179	Promoter (<=1kb)	0605	146E-04	447E-03	Vav2	0	Hyper
chr4	152592994	152595434	Distal Intergenic	0609	146E-04	448E-03	Wnt5b	29881	Hyper
chr6	72864539	72865580	Promoter (2-3kb)	0775	146E-04	448E-03	Nfkbia	-2598	Hyper
chr3	155985928	155986270	Distal Intergenic	1327	146E-04	448E-03	Rpl38	5244	Hyper
chr6	137507078	137509289	Intron (NM_031600/29714, intron 1 of 22)	0554	147E-04	449E-03	Ptprn2	67506	Hyper
chr6	124531272	124533709	Distal Intergenic	0516	147E-04	449E-03	Bdkrb1	20293	Hyper
chr6	131647789	131660199	Distal Intergenic	0344	147E-04	449E-03	Siva1	-45240	Hyper
chr8	57937501	57938160	Distal Intergenic	0905	147E-04	449E-03	Fam219b	-3142	Hyper
chr19	50286358	50286725	Distal Intergenic	1209	148E-04	449E-03	Zfpm1	-47957	Hyper
chr14	42796381	42797378	Intron (NM_001037792/364154, intron 1 of 4)	0842	148E-04	450E-03	Smim14	13020	Hyper
chr15	45408306	45409989	Intron (NM_001107275/306009, intron 1 of 9)	0668	148E-04	450E-03	Slc39a14	13535	Hyper
chr6	26734695	26735272	Intron (NM_001012191/362715, intron 15 of 18)	1005	148E-04	450E-03	Dnmt3a	-87337	Hyper
chr18	15290596	15292096	Distal Intergenic	0537	149E-04	450E-03	Zfp24	15115	Hyper
chr15	98823962	98825780	Intron (NM_001105759/259237, intron 1 of 52)	0519	149E-04	451E-03	Dock9	57373	Hyper

chr10	61342807	61347567	Intron (NM_001105814/287537, intron 2 of 22)	0435	149E-04	451E-03	Abr	57888	Hyper
chr3	114700347	114702201	Intron (NM_032462/65199, intron 2 of 8)	0711	149E-04	452E-03	Fahd2a	-35012	Hyper
chr9	90207761	90211740	5' UTR	0507	150E-04	452E-03	Agap1	19736	Hyper
chr9	42645535	42647019	Distal Intergenic	0678	150E-04	452E-03	Il1rl1	-29656	Hyper
chr20	20302951	20303959	Distal Intergenic	0732	150E-04	453E-03	Arid5b	-3772	Hyper
chr7	133261867	133263722	Promoter (1-2kb)	0726	150E-04	453E-03	Spryd3	1439	Hyper
chr4	11642422	11644305	Intron (NM_001106575/296753, intron 12 of 14)	0570	151E-04	455E-03	Kmt2e	83068	Hyper
chr1	86930621	86931903	Distal Intergenic	0737	151E-04	455E-03	Garre1	-16185	Hyper
chr10	102341217	102347671	Intron (NM_001108309/360668, intron 2 of 16)	0343	152E-04	456E-03	Sec14l1	21297	Hyper
chr5	139037333	139039213	3' UTR	0689	152E-04	456E-03	Tfap2e	-7197	Hyper
chr1	12357835	12358280	Distal Intergenic	1147	152E-04	456E-03	Cited2	45409	Hyper
chr10	3769343	3770743	Intron (NM_001013963/302890, intron 2 of 3)	0711	153E-04	460E-03	Cpped1	67821	Hyper
chr5	165702334	165704390	Intron (NM_001005544/298676, intron 13 of 13)	0548	154E-04	460E-03	Morn1	55338	Hyper
chr9	93485511	93488166	3' UTR	0556	154E-04	460E-03	Rnpepl1	8873	Hyper
chr4	65606347	65609514	Intron (NM_198782/688705, intron 12 of 32)	0492	155E-04	463E-03	Ptn	-231041	Hyper
chr2	56475407	56477139	Promoter (<=1kb)	0699	155E-04	464E-03	Egflam	0	Hyper
chr11	68458635	68460524	Promoter (<=1kb)	0399	156E-04	464E-03	Ubxn7	0	Hyper
chr10	98706094	98711763	3' UTR	0425	156E-04	464E-03	Cog1	10397	Hyper
chr10	1107654	1108115	Distal Intergenic	1113	156E-04	464E-03	Mir193b	-209627	Hyper
chr9	98577996	98578783	Distal Intergenic	0980	156E-04	464E-03	Macir	157236	Hyper
chr8	67633910	67634658	Distal Intergenic	0853	156E-04	465E-03	Tpm1	22246	Hyper
chr6	26735346	26735923	Intron (NM_001012191/362715, intron 15 of 18)	1043	156E-04	465E-03	Dnmt3a	-86686	Hyper
chr14	42574735	42575886	Intron (NM_001083624/305343, intron 1 of 31)	0749	156E-04	465E-03	Pds5a	22088	Hyper
chr1	117468783	117469251	Distal Intergenic	1105	158E-04	469E-03	Klf13	67375	Hyper
chr9	29580628	29581522	Distal Intergenic	0816	158E-04	469E-03	Adgrb3	-1432078	Hyper
chr9	1162145	1164590	Intron (NM_001044236/301128, intron 1 of 22)	0584	158E-04	469E-03	Kdm4b	3366	Hyper
chr4	30712044	30712962	Intron (NM_001191861/114483, intron 3 of 6)	0929	158E-04	469E-03	Fam133b	-96021	Hyper
chr4	76396122	76397729	Distal Intergenic	0697	159E-04	470E-03	Cul1	-154264	Hyper
chr5	55973437	55974308	Intron (NM_053287/24390, intron 1 of 5)	0846	159E-04	470E-03	B4galt1	8153	Hyper
chr4	124199669	124206572	Intron (NM_017206/29464, intron 1 of 14)	0374	160E-04	471E-03	Slc6a6	4322	Hyper
chr8	89247190	89248450	Distal Intergenic	0707	160E-04	472E-03	Nt5e	-22596	Hyper
chr3	22872685	22874173	Distal Intergenic	0646	161E-04	475E-03	Golga1	-60100	Hyper
chr11	70128750	70132197	Promoter (<=1kb)	0393	161E-04	475E-03	Fam43a	0	Hyper

chr8	109178332	109179013	Promoter (<=1kb)	0883	162E-04	477E-03	Lamb2	0	Hyper
chr6	127742217	127745084	3' UTR	0547	162E-04	477E-03	Slc25a29	7737	Hyper
chr19	47726243	47726948	Intron (NM_133581/171112, intron 1 of 6)	1080	162E-04	477E-03	Wfdc1	5815	Hyper
chr19	48180765	48183240	Distal Intergenic	0541	163E-04	478E-03	6430548M08Rikl	-14969	Hyper
chr6	26224991	26229085	3' UTR	0546	163E-04	478E-03	Garem2	12677	Hyper
chr4	125386908	125387421	Intron (NM_001107877/312566, intron 21 of 46)	1015	163E-04	478E-03	Adamts9	75508	Hyper
chr7	107910483	107913307	Promoter (<=1kb)	0564	163E-04	479E-03	Plec	0	Hyper
chr4	147966992	147968967	Intron (NM_001015004/297523, intron 1 of 4)	0565	164E-04	479E-03	Vgll4	18890	Hyper
chr12	27390520	27391255	Distal Intergenic	0940	164E-04	480E-03	Sfswap	-210636	Hyper
chr2	215626630	215628275	Distal Intergenic	0551	165E-04	482E-03	Larp7	384534	Hyper
chr19	18528969	18530409	Intron (NM_001271381/364952, intron 3 of 9)	0660	165E-04	482E-03	Nkd1	18971	Hyper
chr18	30880919	30881963	Intron (NM_001107389/307459, intron 1 of 23)	0780	165E-04	482E-03	Arhgap26	42248	Hyper
chr4	119346227	119348301	Promoter (2-3kb)	0525	165E-04	482E-03	Rpl22	2582	Hyper
chr6	127401620	127406913	Intron (NM_001025741/362783, intron 3 of 21)	0448	165E-04	483E-03	Eml1	32361	Hyper
chr20	13033283	13033649	Distal Intergenic	1232	166E-04	483E-03	Susd2	15806	Hyper
chr2	192474633	192475213	Distal Intergenic	0862	166E-04	483E-03	Wnt2b	-6034	Hyper
chr4	171030037	171031071	Promoter (<=1kb)	0702	166E-04	483E-03	Mgst1	371	Hyper
chr8	57155907	57159019	Distal Intergenic	0465	166E-04	484E-03	Cimap1c	92770	Hyper
chr9	36649879	36650665	3' UTR	0878	166E-04	484E-03	Imp4	-3462	Hyper
chr6	29600903	29602628	Distal Intergenic	0655	166E-04	484E-03	Klhl29	-1210256	Hyper
chr6	15725884	15728214	Intron (NM_001048044/313838, intron 1 of 1)	0473	167E-04	485E-03	Cdc42ep3	14990	Hyper
chr8	69374581	69375868	Distal Intergenic	0761	167E-04	485E-03	Ice2	-668702	Hyper
chrX	151534929	151535597	Exon (NM_001135836/293856, exon 4 of 8)	0960	168E-04	487E-03	Ssr4	10598	Hyper
chr2	153753791	153754820	Intron (NM_001107681/310506, intron 1 of 3)	0726	168E-04	488E-03	B3galnt1	121820	Hyper
chr1	213577542	213580568	Intron (NM_031036/81666, intron 2 of 6)	0549	169E-04	491E-03	Gna14	-135452	Hyper
chr12	39302226	39307570	Promoter (<=1kb)	0371	170E-04	491E-03	Pebp1	0	Hyper
chr17	6820790	6822960	Intron (NM_001271297/306759, intron 2 of 10)	0509	170E-04	492E-03	Spock1	79277	Hyper
chr8	25473411	25474362	Exon (NM_001034000/315508, exon 18 of 36)	0688	171E-04	494E-03	Ncapd3	36288	Hyper
chr1	79479045	79480109	Intron (NM_001109422/680611, intron 2 of 8)	0899	172E-04	495E-03	Bcl3	5498	Hyper
chr10	100839075	100841168	Promoter (2-3kb)	0661	173E-04	499E-03	Mrps7	-2523	Hyper
chr8	61333291	61333990	Exon (NM_001195564/315732, exon 14 of 19)	0920	174E-04	500E-03	Uaca	73814	Hyper
chr5	147545036	147546437	Distal Intergenic	0657	174E-04	501E-03	Clic4	-31758	Hyper
chr17	32839656	32840298	Distal Intergenic	1092	175E-04	501E-03	Foxq1	75079	Hyper

chr9	32978844	32979815	Distal Intergenic	0745	175E-04	501E-03	Phf3	156179	Hyper
chr13	71846367	71848831	Distal Intergenic	0482	175E-04	501E-03	Tnr	-242754	Hyper
chr13	46201292	46202336	3' UTR	0767	176E-04	502E-03	Syt2	16010	Hyper
chr8	98810822	98811404	Distal Intergenic	0997	176E-04	502E-03	Nmnat3	-81067	Hyper
chr12	4520894	4521475	Intron (NM_001170398/304244, intron 53 of 60)	0980	176E-04	502E-03	Tgap1-ps1	-171474	Hyper
chr11	24661148	24663852	Promoter (<=1kb)	0652	177E-04	503E-03	Cyyr1	0	Hyper
chr2	30882220	30884682	Intron (NM_019217/29456, intron 2 of 6)	0528	177E-04	504E-03	Map1b	25635	Hyper
chr20	45415639	45421318	Distal Intergenic	0355	177E-04	504E-03	LOC499469	79842	Hyper
chr16	57513965	57517346	Distal Intergenic	0488	177E-04	504E-03	Dusp4	-123922	Hyper
chr17	57480180	57482338	Intron (NM_001191833/361261, intron 1 of 9)	0564	178E-04	505E-03	Ccny	30522	Hyper
chr3	7395913	7396389	Exon (NM_147141/257648, exon 39 of 46)	1107	178E-04	505E-03	Cacna1b	149568	Hyper
chr3	83290716	83291842	Intron (NM_001107753/311236, intron 4 of 6)	0715	179E-04	507E-03	Lrrc4c	985472	Hyper
chr4	173196022	173197538	Distal Intergenic	0511	179E-04	507E-03	Plekha5	-136517	Hyper
chr2	23107614	23109319	Promoter (1-2kb)	0627	180E-04	508E-03	Ckmt2	1068	Hyper
chr8	109489840	109491752	Intron (NM_021660/59268, intron 2 of 6)	0593	180E-04	509E-03	Ip6k2	3661	Hyper
chr5	153872629	153873251	Distal Intergenic	0865	180E-04	509E-03	Spen	-24289	Hyper
chr7	115769013	115772402	Distal Intergenic	0395	181E-04	510E-03	Prr5	-41552	Hyper
chr5	57269077	57270614	Promoter (<=1kb)	0581	181E-04	512E-03	Atosb	-511	Hyper
chr8	62852965	62860011	Exon (NM_012903/25379, exon 2 of 7)	0393	181E-04	512E-03	Anp32a	23840	Hyper
chr11	63077272	63080192	Distal Intergenic	0443	182E-04	513E-03	Ndufb4	13470	Hyper
chr16	9912161	9913987	Intron (NM_001277165/498587, intron 2 of 13)	0635	182E-04	513E-03	Ldb3	4462	Hyper
chr2	61286980	61289560	3' UTR	0532	183E-04	516E-03	Golph3	-58470	Hyper
chr2	167339470	167339971	Distal Intergenic	0867	184E-04	517E-03	Asic5	31486	Hyper
chr8	66215473	66217426	3' UTR	0581	184E-04	517E-03	Oaz2	15779	Hyper
chr7	17611956	17614483	Intron (NM_017109/29130, intron 5 of 12)	0578	185E-04	519E-03	Timp3	-40117	Hyper
chr6	100176629	100177454	Intron (NM_001100863/362760, intron 1 of 14)	0892	185E-04	519E-03	Galnt16	6261	Hyper
chr9	105350763	105351944	Intron (NM_145094/246324, intron 1 of 6)	0783	185E-04	519E-03	Rab31	29282	Hyper
chr1	128252910	128253890	Intron (NM_177481/140915, intron 2 of 9)	0794	186E-04	521E-03	Slco3a1	133997	Hyper
chr1	180965151	180966537	Intron (NM_001007692/308983, intron 5 of 7)	0655	188E-04	526E-03	Nfatc2ip	5310	Hyper
chr7	51925607	51926054	Intron (NM_053594/94202, intron 13 of 13)	1094	188E-04	526E-03	Ptprb	-27321	Hyper
chr10	36423806	36425223	Distal Intergenic	0608	189E-04	527E-03	Skp1	21819	Hyper
chr17	81557497	81562292	Intron (NM_053926/116723, intron 3 of 9)	0475	189E-04	528E-03	Pip4k2a	105737	Hyper
chr8	109988279	109990193	Intron (NM_001024278/367171, intron 2 of 16)	0620	190E-04	530E-03	Map4	62659	Hyper

chr8	106847675	106849564	Promoter (2-3kb)	0537	190E-04	530E-03	Twf2	2422	Hyper
chr4	117505151	117507684	Intron (NM_001109246/500233, intron 1 of 21)	0534	191E-04	532E-03	Exoc6b	42490	Hyper
chr2	174854121	174855198	3' UTR	0724	191E-04	532E-03	Pbxip1	-3811	Hyper
chr4	161571492	161572408	Distal Intergenic	0853	192E-04	534E-03	Rhno1	66963	Hyper
chr1	220684565	220686250	Distal Intergenic	0620	192E-04	534E-03	Klf9	-13858	Hyper
chr6	131001442	131003060	Distal Intergenic	0638	193E-04	536E-03	LOC102548399	15755	Hyper
chr19	24789539	24790971	Intron (NM_001109506/688581, intron 3 of 8)	0736	195E-04	540E-03	Elmod2	3347	Hyper
chr1	161606433	161609950	Intron (NM_001100582/308918, intron 6 of 23)	0491	196E-04	541E-03	Cyb5r2	54147	Hyper
chr7	63804039	63805261	Distal Intergenic	0845	196E-04	541E-03	Uqcrb	14889	Hyper
chr8	65517583	65521221	Exon (NM_001106831/300783, exon 3 of 11)	0408	197E-04	542E-03	Hacd3	17286	Hyper
chr5	151455357	151464492	Intron (NM_001005903/298584, intron 1 of 8)	0355	197E-04	542E-03	Capzb	19653	Hyper
chr3	10989725	10993381	Promoter (<=1kb)	0405	197E-04	542E-03	Rxra	0	Hyper
chr6	14842771	14845430	Intron (NM_001007704/313843, intron 4 of 6)	0487	197E-04	543E-03	Srsf7	30956	Hyper
chr4	181301385	181303984	Distal Intergenic	0678	198E-04	544E-03	Rps4y2	31878	Hyper
chr7	69876747	69878039	Distal Intergenic	0691	199E-04	545E-03	Baalc	-40959	Hyper
chr5	153320585	153321804	3' UTR	0759	200E-04	549E-03	Mfap2	5874	Hyper
chr7	14519883	14522571	3' UTR	0548	200E-04	549E-03	Rab11b	12018	Hyper
chr10	14898372	14903129	Promoter (<=1kb)	0501	201E-04	550E-03	Wfikkn1	0	Hyper
chr7	53165436	53165812	Distal Intergenic	1266	202E-04	552E-03	Cpm	-59884	Hyper
chr1	83410362	83412495	Intron (NM_001164657/100303643, intron 18 of 19)	0533	202E-04	553E-03	Fcgbpl1	-12293	Hyper
chr2	175262100	175263627	Distal Intergenic	0862	203E-04	554E-03	Ube2q1	63227	Hyper
chr12	8470147	8471329	Distal Intergenic	0642	204E-04	555E-03	Gpr12	-52156	Hyper
chr9	98577334	98577785	Distal Intergenic	1000	204E-04	556E-03	Macir	156574	Hyper
chr1	197525658	197527854	3' UTR	0586	204E-04	557E-03	Ctsd	11489	Hyper
chr10	101292693	101298920	3' UTR	0382	206E-04	559E-03	Unc13d	12593	Hyper
chr3	154881438	154887240	5' UTR	0398	206E-04	559E-03	Sulf2	17175	Hyper
chr1	209487971	209493144	Distal Intergenic	0352	206E-04	559E-03	Dtx4	-6898	Hyper
chr5	63888647	63890284	3' UTR	0593	206E-04	560E-03	Aldob	11802	Hyper
chr5	33124667	33125517	Exon (NM_001024757/297930, exon 15 of 25)	0879	207E-04	561E-03	Rmdn1	54280	Hyper
chr15	34110340	34112109	Distal Intergenic	0613	207E-04	561E-03	Nup58	294894	Hyper
chr12	32928456	32930327	Intron (NM_031745/65201, intron 1 of 24)	0510	207E-04	562E-03	Clip1	17465	Hyper
chr4	145996562	145998445	Intron (NM_001191975/500287, intron 1 of 21)	0581	208E-04	563E-03	Srgap3	71279	Hyper
chr4	179846674	179847479	Intron (NM_001107896/312855, intron 1 of 25)	0857	208E-04	563E-03	Ppfibp1	8909	Hyper

chr4	146753118	146754862	Promoter (2-3kb)	0596	208E-04	564E-03	Brk1	2297	Hyper
chr7	113673931	113676758	Intron (NM_001033694/300095, intron 1 of 19)	0660	212E-04	570E-03	Srebf2	10729	Hyper
chr16	46432980	46433559	Distal Intergenic	0944	213E-04	573E-03	Pdlim3	-49339	Hyper
chr8	70901618	70904634	Intron (NM_173101/25484, intron 1 of 27)	0481	214E-04	575E-03	Myo1e	13684	Hyper
chr4	146967906	146970382	Exon (NM_012508/24215, exon 5 of 22)	0660	215E-04	576E-03	Sec13	-76776	Hyper
chr15	98881563	98884540	Promoter (<=1kb)	0370	215E-04	576E-03	Dock9	0	Hyper
chr13	63422229	63424170	Distal Intergenic	0658	215E-04	576E-03	Ivns1abp	-14220	Hyper
chr1	170685026	170685555	Promoter (1-2kb)	1040	215E-04	576E-03	Pik3c2a	-1554	Hyper
chr2	44391588	44392411	Distal Intergenic	0982	215E-04	576E-03	Plpp1	-46960	Hyper
chr10	104868997	104869428	Distal Intergenic	1188	216E-04	579E-03	Rptor	-9059	Hyper
chr10	102941573	102944638	Distal Intergenic	0413	218E-04	582E-03	Tmc8	-55706	Hyper
chr7	61836357	61837590	Distal Intergenic	0771	219E-04	582E-03		-125646	Hyper
chr15	15463081	15463758	Distal Intergenic	1054	219E-04	582E-03	Cfap20dc	-767541	Hyper
chr6	24092116	24093264	Intron (NM_138898/192259, intron 56 of 57)	0883	219E-04	582E-03	Ppp1cb	-99381	Hyper
chr14	41648589	41651531	Distal Intergenic	0428	219E-04	584E-03	Uchl1	-152999	Hyper
chr6	11455753	11456695	3' UTR	0959	220E-04	584E-03	Pkdcc	8307	Hyper
chr12	6643454	6645266	Intron (NM_013111/25648, intron 1 of 11)	0661	220E-04	585E-03	Slc7a1	15240	Hyper
chr10	86167211	86170094	Distal Intergenic	0426	221E-04	586E-03	Ezh1	-14873	Hyper
chr10	37079359	37080565	Intron (NM_001107000/303130, intron 3 of 14)	0792	221E-04	586E-03	Fstl4	167939	Hyper
chr17	23064109	23065145	Distal Intergenic	0692	221E-04	586E-03		-80325	Hyper
chr8	11214840	11216252	Distal Intergenic	0692	221E-04	587E-03	Sesn3	80923	Hyper
chr9	42504919	42505523	Distal Intergenic	0870	222E-04	588E-03	Il1r1	-34836	Hyper
chr2	30628706	30631649	Distal Intergenic	0537	224E-04	590E-03	Ptcd2	108293	Hyper
chr4	172224122	172224947	Distal Intergenic	0735	224E-04	590E-03	Rergl	167831	Hyper
chr12	8003504	8006550	Promoter (<=1kb)	0399	225E-04	593E-03	Polr1d	0	Hyper
chr1	20027665	20028500	Distal Intergenic	0843	226E-04	593E-03	Akap7	-58990	Hyper
chr13	103341068	103345231	Exon (NM_001109376/679692, exon 4 of 7)	0409	226E-04	594E-03	Lpgat1	45247	Hyper
chr1	201568141	201568745	Exon (NM_001108514/361699, exon 8 of 15)	1132	226E-04	594E-03	Ssh3	-3483	Hyper
chr10	41467265	41468899	Intron (NM_031608/50592, intron 11 of 15)	0662	227E-04	595E-03	Mfap3	-227889	Hyper
chr9	16975008	16976633	Intron (NM_175578/140666, intron 4 of 4)	0617	228E-04	596E-03	Enpp5	-57528	Hyper
chr1	44417027	44418586	Distal Intergenic	0630	228E-04	597E-03	Nox3	-114176	Hyper
chr20	27902556	27903806	Distal Intergenic	0780	228E-04	598E-03	Ddit4	-8468	Hyper
chr5	151467655	151469609	Intron (NM_001005903/298584, intron 1 of 8)	0662	230E-04	600E-03	Capzb	31951	Hyper

chr7	97888187	97890821	Intron (NM_031597/29682, intron 2 of 15)	0495	230E-04	600E-03	Kcnq3	134831	Hyper
chr7	110342051	110343082	Exon (NM_001130554/315120, exon 12 of 21)	0876	231E-04	601E-03	Mfng	-13398	Hyper
chr5	57981464	57982235	Distal Intergenic	0884	231E-04	601E-03	Or13j1	-19135	Hyper
chr18	59114525	59121177	Intron (NM_001107382/307362, intron 2 of 9)	0344	231E-04	602E-03	Zfp532	17832	Hyper
chr13	46077613	46078221	Distal Intergenic	0819	232E-04	603E-03	Kdm5b	75071	Hyper
chr3	168774008	168775977	Promoter (1-2kb)	0592	232E-04	604E-03	Linc00176	1395	Hyper
chr14	41592657	41594636	Distal Intergenic	0564	234E-04	607E-03	Uchl1	-97067	Hyper
chr8	110717632	110718983	Promoter (<=1kb)	0820	235E-04	610E-03	Pth1r	-440	Hyper
chr17	45787280	45789264	Intron (NM_022217/60668, intron 11 of 20)	0466	237E-04	614E-03	Amph	193543	Hyper
chr10	12998527	13000215	Distal Intergenic	0648	238E-04	615E-03	Prss30	10815	Hyper
chr12	41462509	41463555	Intron (NM_001013983/304543, intron 1 of 4)	0723	239E-04	617E-03	Mlec	4275	Hyper
chr7	9150180	9151225	Promoter (2-3kb)	0683	239E-04	617E-03	Rexo1	-2836	Hyper
chr15	98739226	98740243	Intron (NM_001105759/259237, intron 9 of 52)	0768	240E-04	618E-03	Dock9	142910	Hyper
chr9	39140002	39143013	Distal Intergenic	0437	240E-04	619E-03	Zap70	150252	Hyper
chr10	83608672	83610382	Distal Intergenic	0550	242E-04	622E-03	Ormdl3	-10052	Hyper
chr5	144729404	144731810	Distal Intergenic	0476	243E-04	624E-03	Sesn2	-8163	Hyper
chr16	18286647	18289490	Exon (NM_053580/94172, exon 3 of 13)	0487	243E-04	625E-03	Slc27a1	7663	Hyper
chr10	36668847	36669832	Distal Intergenic	0751	244E-04	625E-03	C10h5orf15	104889	Hyper
chr1	7159335	7160534	Intron (NM_013070/25600, intron 2 of 74)	0641	244E-04	625E-03	Utrn	63779	Hyper
chr1	77511136	77517632	Promoter (<=1kb)	0401	244E-04	626E-03	Prkd2	0	Hyper
chr1	203115276	203116379	Distal Intergenic	0741	244E-04	626E-03	Frmd8	47452	Hyper
chr2	115811598	115813264	Intron (NM_173152/286937, intron 1 of 13)	0595	245E-04	627E-03	Pex5l	94718	Hyper
chr10	14097926	14105065	Promoter (<=1kb)	0410	245E-04	627E-03	Tmem204	0	Hyper
chr4	146675308	146676528	Promoter (2-3kb)	0786	245E-04	628E-03	Emc3	2448	Hyper
chr1	90896499	90897262	Distal Intergenic	0915	245E-04	628E-03	Plekhf1	12845	Hyper
chr19	48130882	48132543	Distal Intergenic	0652	245E-04	628E-03	Zdhhc7	24130	Hyper
chr5	146372133	146373964	Exon (NM_001080151/362622, exon 7 of 13)	0634	246E-04	629E-03	Sh3bgrl3	-16608	Hyper
chr19	24441022	24444767	Promoter (<=1kb)	0360	246E-04	630E-03	Pkn1	0	Hyper
chr19	20385164	20387027	Intron (NM_080905/140941, intron 1 of 1)	0493	248E-04	633E-03	Siah1	6256	Hyper
chr8	98620638	98623859	Intron (NM_134377/171394, intron 1 of 17)	0445	248E-04	634E-03	Clstn2	12944	Hyper
chr6	99075455	99077287	Intron (NM_031005/81634, intron 1 of 20)	0656	249E-04	634E-03	Actn1	15900	Hyper
chr9	105705902	105707705	Promoter (2-3kb)	0570	250E-04	636E-03	Ndufv2	2964	Hyper
chr9	54856454	54857156	Distal Intergenic	0991	250E-04	636E-03	Slc39a10	-53846	Hyper

chr12	19036527	19037483	Exon (NM_001107131/304375, exon 6 of 12)	0737	250E-04	636E-03	Irs3	-15665	Hyper
chr2	55960588	55961362	Distal Intergenic	0931	252E-04	641E-03	Osmr	-10891	Hyper
chr1	117136443	117139607	Distal Intergenic	0522	254E-04	643E-03	Chrna7	-299220	Hyper
chr8	20454188	20455197	Promoter (2-3kb)	0752	254E-04	644E-03	Tmem205	2631	Hyper
chr18	36973457	36976832	Intron (NM_001170534/307449, intron 3 of 18)	0457	255E-04	646E-03	Dcp2	222725	Hyper
chr13	47198061	47199008	Distal Intergenic	0987	257E-04	650E-03	Phlda3	4811	Hyper
chr15	3713560	3715186	Exon (NM_001008863/408223, exon 3 of 20)	0794	259E-04	654E-03	Usp54	20906	Hyper
chr12	38513738	38516946	3' UTR	0458	259E-04	654E-03	Fbxw8	87239	Hyper
chr7	132234543	132235285	Distal Intergenic	0932	260E-04	655E-03	Acvrl1	-10226	Hyper
chr8	44563078	44563436	Distal Intergenic	1248	260E-04	655E-03	Ccdc153	-14400	Hyper
chr12	33785694	33787643	Distal Intergenic	0618	263E-04	662E-03	Camkk2	-3447	Hyper
chr1	150804372	150806638	Intron (NM_001191628/308831, intron 2 of 31)	0561	264E-04	662E-03	Tenm4	23991	Hyper
chr15	98752551	98753302	Intron (NM_001105759/259237, intron 5 of 52)	0751	264E-04	662E-03	Dock9	129851	Hyper
chr9	45957454	45958030	Distal Intergenic	0950	264E-04	662E-03	Ecrg4	-34924	Hyper
chr12	35196037	35197083	Distal Intergenic	0898	264E-04	664E-03	Trafd1	30431	Hyper
chr4	131898305	131899544	Distal Intergenic	0606	265E-04	664E-03	Foxp1	-101550	Hyper
chr2	238178695	238179928	Intron (NM_001190475/171447, intron 2 of 25)	0651	266E-04	666E-03	Adgrl2	147213	Hyper
chr19	34230860	34231858	Intron (NM_053605/94338, intron 1 of 8)	0736	266E-04	666E-03	Smpd3	13891	Hyper
chr7	9636389	9637605	Intron (NM_001108068/314619, intron 6 of 32)	0695	268E-04	669E-03	Gpx4	14907	Hyper
chr18	68996442	69002186	Exon (NM_030858/81516, exon 3 of 4)	0405	268E-04	670E-03	Smad7	8013	Hyper
chr1	187530662	187533762	Intron (NM_001107556/309060, intron 4 of 4)	0513	269E-04	672E-03	Fam53b	57424	Hyper
chr17	61175768	61176905	Intron (NM_001107361/307070, intron 4 of 18)	0912	270E-04	673E-03	Larp4b	40451	Hyper
chr1	27337457	27338877	Distal Intergenic	0591	270E-04	673E-03	Cenpw	-186447	Hyper
chr4	63535812	63537656	Promoter (<=1kb)	0519	270E-04	674E-03	Tmem140	0	Hyper
chr13	82868675	82877355	Promoter (<=1kb)	0350	272E-04	677E-03	Olfml2b	0	Hyper
chr4	125518450	125519413	Distal Intergenic	0930	272E-04	677E-03	Adamts9	-55521	Hyper
chr17	53217357	53218097	Distal Intergenic	0907	273E-04	678E-03	Mtpap	123384	Hyper
chr4	158403730	158404272	Intron (NM_053889/116669, intron 10 of 51)	1162	273E-04	678E-03	Vwf	43578	Hyper
chr7	9866289	9866873	Distal Intergenic	0974	273E-04	679E-03	Ptbp1	-13957	Hyper
chr12	41459534	41460573	Promoter (1-2kb)	0811	274E-04	680E-03	Mlec	1300	Hyper
chr7	20392085	20395798	Intron (NM_001108742/362861, intron 1 of 10)	0487	274E-04	680E-03	Slc41a2	8371	Hyper
chr8	68907871	68908988	Distal Intergenic	0734	274E-04	680E-03	LOC100911360	-570914	Hyper
chr2	24550097	24552678	Intron (NM_031707/29546, intron 1 of 9)	0584	274E-04	680E-03	Homer1	6097	Hyper

chr10	10652720	10653647	Exon (NM_001013964/302938, exon 10 of 17)	0727	274E-04	680E-03	Mir3568	7797	Hyper
chr12	41368130	41370046	Exon (NM_001011904/288710, exon 9 of 17)	0580	275E-04	681E-03	Pop5	11290	Hyper
chr10	37366852	37367940	Distal Intergenic	0744	275E-04	682E-03	Hspa4	81061	Hyper
chr17	13263682	13264170	Distal Intergenic	1136	275E-04	682E-03	Gadd45g	114417	Hyper
chr17	52608152	52609180	Distal Intergenic	0857	276E-04	682E-03	Svil	158080	Hyper
chr5	129487213	129493666	Exon (NM_001369126/100911581, exon 2 of 15)	0386	276E-04	683E-03	Faah	5011	Hyper
chr3	145675468	145677444	Distal Intergenic	0716	276E-04	683E-03	Dsn1	-9615	Hyper
chr13	45684013	45687818	Intron (NM_017155/29290, intron 1 of 1)	0424	277E-04	683E-03	Adora1	6984	Hyper
chr8	12736343	12738293	Intron (NM_138544/191571, intron 19 of 25)	0610	277E-04	683E-03	Mtnr1b	-83606	Hyper
chr19	24094464	24096511	Promoter (2-3kb)	0511	278E-04	685E-03	Rfx1	-2420	Hyper
chr19	48159674	48160712	Distal Intergenic	0786	278E-04	686E-03	Zdhhc7	-3001	Hyper
chr3	18594353	18599116	Distal Intergenic	0400	278E-04	686E-03	Gsn	-12371	Hyper
chr10	85975730	85978317	Exon (NM_031604/29757, exon 19 of 22)	0536	279E-04	688E-03	Hsd17b1	-31411	Hyper
chr14	42103209	42104845	Distal Intergenic	0603	280E-04	688E-03	Rbm47	-57042	Hyper
chr12	41092655	41095631	Intron (NM_001012147/360820, intron 1 of 9)	0633	281E-04	690E-03	Pxn	12289	Hyper
chr9	98576503	98577080	Distal Intergenic	1022	281E-04	690E-03	Macir	155743	Hyper
chr19	49969651	49970500	Distal Intergenic	0733	281E-04	690E-03	Slc7a5	-5828	Hyper
chr7	9107412	9109110	Promoter (1-2kb)	0653	281E-04	690E-03	Adat3	1332	Hyper
chr6	75883017	75884970	Promoter (<=1kb)	0618	282E-04	690E-03	Clec14a	0	Hyper
chr17	22458029	22459117	Promoter (1-2kb)	0745	283E-04	693E-03	Edn1	1695	Hyper
chr7	130836828	130846783	Promoter (<=1kb)	0392	284E-04	694E-03	Gpd1	0	Hyper
chr3	147413022	147414093	Intron (NM_001191072/680616, intron 10 of 10)	0762	286E-04	698E-03	Fam83d	-7848	Hyper
chr3	140593925	140606045	Promoter (<=1kb)	0312	287E-04	700E-03	Srxn1	0	Hyper
chr3	140131915	140133436	Intron (NM_001106525/296267, intron 2 of 5)	0679	288E-04	703E-03	Snph	5906	Hyper
chr20	10005408	10005883	Distal Intergenic	1122	289E-04	703E-03	Sik1	-46469	Hyper
chr7	113399065	113400450	Intron (NM_024398/79250, intron 1 of 17)	0729	289E-04	704E-03	Aco2	13388	Hyper
chr7	100087850	100088768	Distal Intergenic	0638	290E-04	705E-03	Zfat	-33576	Hyper
chr16	6413553	6414233	Promoter (<=1kb)	0970	290E-04	705E-03	Sema3g	0	Hyper
chr1	181930691	181931807	Intron (NM_001033998/308995, intron 11 of 30)	0873	291E-04	706E-03	Itgal	12508	Hyper
chr3	140606482	140607882	Promoter (1-2kb)	0648	291E-04	706E-03	Srxn1	1992	Hyper
chr10	3976955	3985841	Exon (NM_001109526/689142, exon 17 of 21)	0398	292E-04	707E-03	Cpped1	275433	Hyper
chr6	103123183	103126680	Promoter (<=1kb)	0476	292E-04	707E-03	Dpf3	0	Hyper
chr5	165850260	165852613	Exon (NM_022507/25522, exon 8 of 18)	0493	292E-04	707E-03	Faap20	41603	Hyper

chr8	19983894	19994974	Intron (NM_013199/25751, intron 1 of 20)	0351	293E-04	709E-03	Dnm2	5444	Hyper
chr11	83322210	83323726	Exon (NM_001108325/360743, exon 5 of 18)	0775	293E-04	710E-03	Med15	41356	Hyper
chr12	31777000	31778957	Distal Intergenic	0550	294E-04	711E-03	Ccdc92	-17727	Hyper
chr6	10776177	10776720	Distal Intergenic	0878	294E-04	711E-03	Haao	-69065	Hyper
chr12	46172155	46173590	Distal Intergenic	0625	294E-04	711E-03	Galnt9	-43577	Hyper
chr7	107443290	107444437	Exon (NM_001134865/300032, exon 10 of 12)	0768	294E-04	711E-03	Rhpn1	51306	Hyper
chr19	48335784	48339599	Distal Intergenic	0418	295E-04	711E-03	6430548M08Rikl	137575	Hyper
chr1	53191745	53192395	Distal Intergenic	0756	295E-04	712E-03	Smok2a	-87972	Hyper
chr12	15289514	15290839	Distal Intergenic	0664	296E-04	714E-03	Mir339	11968	Hyper
chr9	92834425	92835658	Distal Intergenic	0739	297E-04	716E-03	Hdac4	-84261	Hyper
chr6	93158953	93162481	Distal Intergenic	0360	299E-04	718E-03	Snapc1	474168	Hyper
chr13	46963430	46966304	Distal Intergenic	0486	299E-04	718E-03	Ipo9	-100736	Hyper
chr17	9819775	9827687	Promoter (<=1kb)	0341	299E-04	718E-03	Tspan17	0	Hyper
chr10	37125162	37127720	Intron (NM_001107000/303130, intron 3 of 14)	0485	299E-04	718E-03	Fstl4	213742	Hyper
chr7	9039661	9042522	Promoter (<=1kb)	0362	299E-04	718E-03	Mknk2	0	Hyper
chr3	8291192	8292113	Distal Intergenic	0818	299E-04	718E-03	Ptgds	-6359	Hyper
chr16	1269322	1272400	Distal Intergenic	0503	301E-04	722E-03	Ppif	12125	Hyper
chr19	48160923	48163676	Distal Intergenic	0624	301E-04	722E-03	Zdhhc7	-4250	Hyper
chr1	175766884	175768201	Distal Intergenic	0683	302E-04	722E-03	Igsf6	-146242	Hyper
chr1	84231423	84236202	Intron (NM_031675/63836, intron 1 of 20)	0374	303E-04	725E-03	Actn4	15616	Hyper
chr1	13966566	13967238	Distal Intergenic	0844	304E-04	726E-03	Olig3	-114021	Hyper
chr7	109289638	109290807	Promoter (<=1kb)	0780	304E-04	726E-03	Apol3l1	913	Hyper
chr5	101405182	101405768	Exon (NM_001107943/313339, exon 3 of 6)	0952	306E-04	731E-03	Acer2	13297	Hyper
chr5	141473257	141474782	Distal Intergenic	0538	307E-04	731E-03	Fndc5	-8921	Hyper
chr6	7825246	7828209	Intron (NM_023090/29452, intron 2 of 16)	0605	308E-04	733E-03	Epas1	43037	Hyper
chr1	172481170	172482900	Distal Intergenic	0582	309E-04	735E-03	Arl6ip1	-41180	Hyper
chr2	182653428	182656466	Intron (NM_001042621/365865, intron 1 of 14)	0560	309E-04	736E-03	Pip5k1a	14995	Hyper
chr7	115428876	115429766	Intron (NM_001134780/362973, intron 8 of 12)	0896	309E-04	736E-03	Parvg	-26443	Hyper
chr20	10006159	10007899	Distal Intergenic	0615	310E-04	737E-03	Sik1	-47220	Hyper
chr8	58120090	58121819	Distal Intergenic	0562	310E-04	737E-03	Cyp1a1	24001	Hyper
chr7	40705685	40707065	Intron (NM_001171177/299762, intron 1 of 11)	0572	310E-04	738E-03	Tmtc2	99586	Hyper
chr10	86955300	86957528	Exon (NM_053668/114202, exon 12 of 19)	0528	311E-04	738E-03	Mpp3	17083	Hyper
chr6	31323517	31326399	Distal Intergenic	0515	312E-04	740E-03	Rhob	39524	Hyper

chr17	12157638	12158272	Distal Intergenic	0849	312E-04	740E-03	Nfil3	-122227	Hyper
chrX	41476044	41477896	Distal Intergenic	0516	313E-04	742E-03	Tbl1x	-172476	Hyper
chr3	7704150	7705736	Intron (NM_001108572/362078, intron 1 of 25)	0580	315E-04	745E-03	Ehmt1	23201	Hyper
chr1	23382825	23386824	Distal Intergenic	0508	316E-04	747E-03	Clvs2	-348448	Hyper
chr17	81593482	81595152	Intron (NM_053926/116723, intron 1 of 9)	0665	317E-04	750E-03	Pip4k2a	72877	Hyper
chr10	82660790	82665030	Distal Intergenic	0419	319E-04	752E-03	Cisd3	-14315	Hyper
chr10	57444640	57449849	Distal Intergenic	0355	319E-04	752E-03	Cyb5d2	-31643	Hyper
chr19	48344612	48346033	Distal Intergenic	0710	319E-04	752E-03	6430548M08Rikl	146403	Hyper
chr4	123750474	123752378	Distal Intergenic	0566	319E-04	753E-03	Hdac11	100317	Hyper
chr1	29595576	29596632	Exon (NM_001039722/664630, exon 4 of 12)	0899	320E-04	753E-03	Slc6a19	9371	Hyper
chr11	77619106	77620021	Distal Intergenic	0810	321E-04	755E-03	St6gal1	-49903	Hyper
chr2	78579967	78583152	Intron (NM_001107658/310192, intron 33 of 56)	0427	321E-04	755E-03	Otulinl	-146142	Hyper
chr4	60179060	60182651	Promoter (<=1kb)	0493	322E-04	756E-03	Podxl	0	Hyper
chr10	101619742	101621708	Distal Intergenic	0563	324E-04	760E-03	Foxj1	-49512	Hyper
chr2	219666015	219668810	Promoter (<=1kb)	0618	325E-04	760E-03	Lef1	0	Hyper
chr2	192293540	192294229	3' UTR	0941	326E-04	762E-03	Rhoc	6299	Hyper
chr3	43061058	43061736	Distal Intergenic	0890	326E-04	763E-03	Acvr1	-13858	Hyper
chr5	22786929	22788286	Promoter (2-3kb)	0747	326E-04	763E-03	Asph	-2411	Hyper
chr4	124496541	124498176	Promoter (<=1kb)	0786	327E-04	764E-03	Fgd5	0	Hyper
chr11	67087585	67096203	Distal Intergenic	0334	329E-04	766E-03	Itgb5	-143354	Hyper
chr2	173491779	173493952	Promoter (2-3kb)	0524	331E-04	769E-03	Hapln2	2634	Hyper
chr17	6966478	6969081	Intron (NM_001271297/306759, intron 3 of 10)	0439	331E-04	770E-03	Spock1	224965	Hyper
chr12	718485	721182	Intron (NM_001109060/498130, intron 1 of 13)	0588	331E-04	770E-03	Stard13	13422	Hyper
chr1	181285141	181288578	3' UTR	0403	331E-04	770E-03	Slx1b	3161	Hyper
chr6	42517993	42518468	Distal Intergenic	1006	332E-04	770E-03	Rnf144a	501242	Hyper
chr13	92131072	92132787	Intron (NM_019312/54260, intron 2 of 7)	0570	332E-04	771E-03	Itpkb	61856	Hyper
chr10	52358631	52369499	Exon (NM_053484/85246, exon 8 of 14)	0371	333E-04	772E-03	Rcvrn	-19207	Hyper
chr12	6133849	6136612	Intron (NM_001006956/288449, intron 4 of 10)	0487	333E-04	773E-03	Katnal1	30963	Hyper
chr8	58035555	58037402	Promoter (1-2kb)	0565	335E-04	776E-03	Csk	-1183	Hyper
chr11	76327606	76328819	Distal Intergenic	0679	336E-04	776E-03	Lpp	-27041	Hyper
chr20	10122358	10123655	Promoter (1-2kb)	0483	338E-04	781E-03	Hsf2bp	-1116	Hyper
chr6	130686697	130687407	Intron (NM_130749/170577, intron 12 of 17)	0849	338E-04	781E-03	Ckb	44894	Hyper
chr19	45463458	45466025	Exon (NM_001163273/292051, exon 5 of 21)	0460	340E-04	783E-03	Plcg2	-81391	Hyper

chr1	256362482	256363742	Distal Intergenic	0642	340E-04	784E-03	Ablim1	-85987	Hyper
chr6	57441432	57442740	Distal Intergenic	0666	340E-04	785E-03	Ifrd1	-152526	Hyper
chr2	174643246	174644602	Intron (NM_001107691/310641, intron 8 of 8)	0679	341E-04	785E-03	Mir92b	-9274	Hyper
chr11	77677425	77677961	Distal Intergenic	0968	342E-04	786E-03	Adipoq	57603	Hyper
chr11	67223657	67227773	Intron (NM_153625/266733, intron 4 of 12)	0381	342E-04	786E-03	Slc12a8	38031	Hyper
chr5	101229691	101230714	Distal Intergenic	1006	342E-04	786E-03	Plin2	-46655	Hyper
chr1	209473389	209476064	Exon (NM_001047855/293774, exon 4 of 8)	0536	342E-04	786E-03	Dtx4	5009	Hyper
chr3	167317149	167318878	Promoter (<=1kb)	0762	344E-04	789E-03	Lama5	0	Hyper
chr8	48784498	48786246	Distal Intergenic	0621	344E-04	789E-03	Nxpe1	21926	Hyper
chr11	67602965	67604107	Distal Intergenic	0614	346E-04	793E-03	Osbpl11	-6764	Hyper
chr12	28487020	28488402	Intron (NM_198727/288750, intron 2 of 9)	0697	347E-04	795E-03	Tmem132d	216308	Hyper
chr17	8927165	8929744	Distal Intergenic	0574	348E-04	796E-03	Txndc15	-16734	Hyper
chr11	70960967	70966233	Distal Intergenic	0359	348E-04	796E-03	Opa1	218838	Hyper
chr17	83583650	83584712	Intron (NM_001106127/291355, intron 3 of 8)	0763	349E-04	798E-03	Prtfdc1	69805	Hyper
chr16	45781915	45783317	Intron (NM_012820/25288, intron 3 of 19)	0732	351E-04	801E-03	Acsl1	16453	Hyper
chr6	129846083	129847612	Intron (NM_001010965/362787, intron 1 of 11)	0649	352E-04	803E-03	Mok	7012	Hyper
chr10	101300837	101302246	Exon (NM_138844/192177, exon 26 of 34)	0641	353E-04	804E-03	Unc13d	9267	Hyper
chr18	56070269	56070583	5' UTR	1077	354E-04	807E-03	Spink7	-20570	Hyper
chr15	7701611	7704342	Intron (NM_001270854/24831, intron 8 of 10)	0595	356E-04	809E-03	Nr1d2	177354	Hyper
chr9	71187416	71188121	Promoter (1-2kb)	0846	357E-04	811E-03	Ikzf2	1240	Hyper
chr20	6804556	6806519	Intron (NM_031020/81649, intron 10 of 11)	0608	357E-04	812E-03	Mapk13	-28801	Hyper
chr15	31746553	31748081	Intron (NM_001106042/290280, intron 9 of 22)	0512	359E-04	814E-03	Eef1akmt1	-39169	Hyper
chr16	16790790	16793850	Exon (NM_001169127/306324, exon 4 of 9)	0493	359E-04	815E-03	Sh2d4b	-21703	Hyper
chr7	79570831	79571184	Distal Intergenic	0956	360E-04	816E-03	Trps1	2570170	Hyper
chr1	98921154	98922531	Distal Intergenic	0634	361E-04	816E-03	Nav2	-35867	Hyper
chr11	35088128	35089424	Distal Intergenic	0591	361E-04	817E-03	Ets2	66592	Hyper
chr12	35184878	35192195	Distal Intergenic	0357	362E-04	817E-03	Trafd1	19272	Hyper
chr9	17620018	17620754	Intron (NM_139110/245977, intron 1 of 20)	1070	362E-04	817E-03	Adgrf5	3214	Hyper
chr5	156898571	156900756	Intron (NM_001108006/313825, intron 64 of 69)	0552	364E-04	821E-03	Dhrs3	149952	Hyper
chr7	51953065	51954415	Promoter (<=1kb)	0787	365E-04	822E-03	Ptprb	0	Hyper
chr5	148445116	148446149	Distal Intergenic	0756	366E-04	824E-03	Tcea3	-30792	Hyper
chr6	121800725	121803878	Intron (NM_001191985/500709, intron 2 of 9)	0432	366E-04	824E-03	Itpk1	40160	Hyper
chr3	141989671	141991241	Distal Intergenic	0597	366E-04	824E-03	Commd7	123089	Hyper

chr20	11234224	11235184	Intron (NM_001111055/25367, intron 1 of 10)	0738	367E-04	825E-03	Adarb1	11629	Hyper
chr1	181919835	181925940	Promoter (1-2kb)	0375	367E-04	825E-03	Itgal	1652	Hyper
chr1	226736810	226746691	3' UTR	0358	368E-04	826E-03	Ak3	17877	Hyper
chr11	30801575	30802881	Distal Intergenic	0678	368E-04	826E-03	Ifngr2	21842	Hyper
chr1	155222639	155224965	Distal Intergenic	0474	368E-04	826E-03	Relt	-8443	Hyper
chr6	103850236	103851497	Distal Intergenic	0743	370E-04	830E-03	Mideas	-21058	Hyper
chr15	32949463	32950089	Distal Intergenic	1056	370E-04	830E-03	RGD1563302	352805	Hyper
chr10	102519560	102520434	Intron (NM_176856/83788, intron 3 of 11)	0845	371E-04	831E-03	Septin9	-24125	Hyper
chr7	33993620	33996132	Distal Intergenic	0428	373E-04	833E-03	Galnt4	68391	Hyper
chr4	46833692	46834454	Intron (NM_001114401/282587, intron 14 of 22)	0788	373E-04	834E-03	Cttnbp2	130177	Hyper
chr4	168924796	168926572	Intron (NM_012574/24410, intron 1 of 12)	0604	378E-04	842E-03	Grin2b	115707	Hyper
chr10	38253431	38256629	Exon (NM_001108275/360526, exon 6 of 16)	0399	379E-04	844E-03	P4ha2	10336	Hyper
chr11	80538100	80538838	Intron (NM_053924/116721, intron 17 of 29)	0807	380E-04	846E-03	Parl	-55239	Hyper
chr6	28198734	28199554	Intron (NM_001106713/298867, intron 3 of 13)	0882	381E-04	848E-03	Klhl29	191093	Hyper
chr11	33032875	33034709	Intron (NM_001191660/304077, intron 1 of 35)	0682	382E-04	848E-03	Dop1b	4826	Hyper
chr11	84081307	84083324	Intron (NM_175755/287931, intron 2 of 7)	0513	383E-04	851E-03	Ppm1f	11036	Hyper
chr8	75710545	75711341	Intron (NM_001106838/300836, intron 2 of 12)	0856	384E-04	851E-03	Atosa	15523	Hyper
chr10	105265723	105268819	Intron (NM_057196/117542, intron 6 of 13)	0498	385E-04	854E-03	Mir3065	-29087	Hyper
chr3	12912112	12912850	Distal Intergenic	0842	387E-04	856E-03	Trub2	146981	Hyper
chr1	196111300	196112211	Promoter (<=1kb)	0831	387E-04	856E-03	Ifitm3	0	Hyper
chr1	8378554	8380400	Intron (NM_024137/29721, intron 1 of 8)	0614	387E-04	856E-03	Hivep2	19265	Hyper
chr9	75605577	75606953	Intron (NM_001191810/301509, intron 3 of 34)	0722	387E-04	856E-03	Tns1	95805	Hyper
chr10	35869623	35871500	Distal Intergenic	0404	388E-04	857E-03	Nhp2	-5535	Hyper
chr9	56666111	56666590	Distal Intergenic	0909	388E-04	857E-03	Mob4	48737	Hyper
chr15	64297150	64299393	Distal Intergenic	0671	388E-04	857E-03	Pcdh20	132397	Hyper
chr3	152369140	152373203	Intron (NM_153469/266709, intron 1 of 2)	0375	389E-04	859E-03	Pkig	38601	Hyper
chr20	11172090	11174154	Distal Intergenic	0581	389E-04	859E-03	Adarb1	-48441	Hyper
chr1	121456312	121456776	Distal Intergenic	1090	391E-04	863E-03	Synm	-92660	Hyper
chr2	218736737	218737454	Distal Intergenic	0990	392E-04	863E-03	Sec24b	-104038	Hyper
chr20	18146320	18148468	Distal Intergenic	0545	392E-04	863E-03	Phyhipl	114515	Hyper
chr10	17273417	17274023	Intron (NM_001106993/303024, intron 2 of 11)	0872	392E-04	863E-03	Fbxw11	40638	Hyper
chr10	104655843	104657546	Distal Intergenic	0503	393E-04	866E-03	Eif4a3	-96811	Hyper
chr11	62082620	62084094	Intron (NM_001105879/288093, intron 1 of 11)	0554	394E-04	867E-03	Arhgap31	43985	Hyper

chr18	23564656	23565343	Intron (NM_001012163/361303, intron 1 of 8)	0835	394E-04	867E-03	Lims2	10719	Hyper
chr3	9151724	9152579	Exon (NM_144745/246254, exon 8 of 14)	0838	396E-04	870E-03	Gpsm1	-9472	Hyper
chr11	70230124	70230761	Distal Intergenic	0911	397E-04	871E-03	Lsg1	86225	Hyper
chr10	101310896	101314865	Promoter (<=1kb)	0467	397E-04	872E-03	Unc13d	0	Hyper
chr5	116249222	116251608	Exon (NM_001107949/313409, exon 9 of 18)	0505	397E-04	872E-03	Leprot	-38235	Hyper
chr5	130167100	130168768	3' UTR	0753	398E-04	873E-03	Mmachc	3821	Hyper
chr10	16602590	16604297	Distal Intergenic	0627	398E-04	873E-03	Dusp1	-76192	Hyper
chr7	129743721	129745809	3' UTR	0541	400E-04	877E-03	Adcy6	17142	Hyper
chr19	23502089	23504537	Distal Intergenic	0437	401E-04	879E-03	Ier2	7538	Hyper
chr10	91514694	91516328	Distal Intergenic	0490	401E-04	879E-03	Ern1	-122815	Hyper
chr7	116451707	116452171	Intron (NM_133313/170821, intron 1 of 11)	1032	401E-04	879E-03	Atxn10	9939	Hyper
chr18	27994433	27996033	Distal Intergenic	0719	403E-04	882E-03	Pfdn1	71031	Hyper
chr19	33951586	33953963	Exon (NM_001106181/291978, exon 14 of 16)	0403	403E-04	883E-03	Nfatc3	-6889	Hyper
chr2	192286578	192289772	Promoter (<=1kb)	0435	404E-04	884E-03	Rhoc	0	Hyper
chr17	23123807	23124422	Distal Intergenic	0865	404E-04	884E-03	Tmem170b	45141	Hyper
chr4	179309217	179311890	Intron (NM_031046/81678, intron 9 of 56)	0555	405E-04	885E-03	Itpr2	92274	Hyper
chr5	60787281	60789047	Intron (NM_001106655/298071, intron 2 of 5)	0736	405E-04	885E-03	Nans	6840	Hyper
chr18	39379482	39380026	Distal Intergenic	0939	406E-04	887E-03	Eif1a	53787	Hyper
chr12	32441897	32451685	5' UTR	0331	407E-04	889E-03	Arl6ip4	30083	Hyper
chr14	58936110	58939060	Distal Intergenic	0462	409E-04	893E-03	Dhx15	148959	Hyper
chr12	14122361	14125739	Distal Intergenic	0415	409E-04	893E-03	Chst12	-10077	Hyper
chr10	36452291	36453402	Distal Intergenic	0695	409E-04	893E-03	Skp1	50304	Hyper
chr10	102360757	102366965	3' UTR	0382	410E-04	893E-03	Sec14l1	40837	Hyper
chr20	10993445	10994265	Intron (NM_001106380/294331, intron 3 of 6)	0835	410E-04	893E-03	Ube2g2	11203	Hyper
chr7	107842915	107845055	Distal Intergenic	0647	410E-04	894E-03	Nrbp2	-37685	Hyper
chr5	150330927	150332173	Exon (NM_001106693/298573, exon 8 of 31)	0668	411E-04	894E-03	Eif4g3	79785	Hyper
chr5	132738461	132740892	Exon (NM_138827/24778, exon 3 of 10)	0589	411E-04	895E-03	Slc2a1	21262	Hyper
chr19	11785709	11786693	Intron (NM_001108439/361368, intron 5 of 12)	0736	412E-04	895E-03	Large1	262237	Hyper
chr2	223696804	223697162	Distal Intergenic	1082	413E-04	898E-03	Bdh2	-5250	Hyper
chr4	146459003	146461318	Promoter (2-3kb)	0578	415E-04	902E-03	Brpf1	2659	Hyper
chr7	112638443	112643445	Distal Intergenic	0359	416E-04	902E-03	Mchr1	-118109	Hyper
chr10	72863840	72865026	Exon (NM_001304355/287615, exon 2 of 6)	0831	417E-04	904E-03	Vezf1	3936	Hyper
chr12	38583025	38584906	Intron (NM_001108338/360818, intron 9 of 11)	0648	418E-04	906E-03	Fbxo21	20227	Hyper

chr20	6320513	6323422	Intron (NM_013141/25682, intron 2 of 7)	0515	419E-04	906E-03	Ppard	21728	Hyper
chr18	62427847	62428212	Distal Intergenic	1134	420E-04	907E-03	Mc2r	-412359	Hyper
chr14	41707305	41709566	Distal Intergenic	0563	421E-04	909E-03	Uchl1	-211715	Hyper
chr18	44159252	44160759	Distal Intergenic	0578	422E-04	910E-03	Prr16	-263897	Hyper
chr18	28140358	28141968	3' UTR	0537	422E-04	911E-03	Slc4a9	11609	Hyper
chr17	32913482	32915302	Promoter (<=1kb)	0724	422E-04	912E-03	Foxq1	75	Hyper
chr12	36045598	36048253	Intron (NM_001017488/498185, intron 2 of 15)	0411	425E-04	914E-03	Slc8b1	4778	Hyper
chr18	47314238	47315912	Intron (NM_022855/64823, intron 1 of 12)	0668	425E-04	915E-03	Csnk1g3	14659	Hyper
chr1	46530541	46537024	Intron (NM_032071/84018, intron 1 of 27)	0398	426E-04	917E-03	Synj2	11783	Hyper
chr19	48294550	48297659	Distal Intergenic	0452	426E-04	917E-03	6430548M08Rikl	96341	Hyper
chr13	46787943	46788983	Intron (NM_001107179/304816, intron 1 of 3)	0777	427E-04	918E-03	Shisa4	14720	Hyper
chr4	159399170	159427920	Distal Intergenic	0304	427E-04	918E-03	Kcna5	-41874	Hyper
chr1	45093289	45095085	Distal Intergenic	0493	428E-04	920E-03	Nox3	-790438	Hyper
chr17	30681656	30682876	Intron (NM_022624/64559, intron 4 of 10)	0801	429E-04	921E-03	Psmg4	35148	Hyper
chr1	240951378	240955483	Promoter (<=1kb)	0402	433E-04	928E-03		0	Hyper
chr10	13481657	13482732	Exon (NM_001185046/681359, exon 2 of 14)	0886	434E-04	929E-03	E4f1	3242	Hyper
chr4	104373538	104374871	3' UTR	0775	434E-04	929E-03	Sftpb	14142	Hyper
chr17	66460650	66461085	Distal Intergenic	1056	438E-04	935E-03	Calml3	39017	Hyper
chr16	17357666	17359757	Intron (NM_001109107/498599, intron 4 of 5)	0527	438E-04	935E-03	Slc35e1	6678	Hyper
chr9	41614441	41618422	Exon (NM_001108214/316351, exon 16 of 21)	0409	439E-04	936E-03	Rpl31	-28979	Hyper
chr3	167179597	167182507	3' UTR	0494	440E-04	936E-03	Mtg2	13612	Hyper
chr7	115430129	115433758	Intron (NM_001134780/362973, intron 8 of 12)	0440	440E-04	936E-03	Parvg	-22451	Hyper
chr16	6428919	6429522	Distal Intergenic	0982	441E-04	937E-03	Sema3g	15330	Hyper
chr7	128926601	128929060	Distal Intergenic	0534	441E-04	937E-03	Slc48a1	11378	Hyper
chr2	174117025	174119858	3' UTR	0479	441E-04	937E-03	Arhgef2	26249	Hyper
chr3	36979633	36980198	Intron (NM_001105733/58942, intron 1 of 12)	0793	441E-04	937E-03	Cacnb4	67017	Hyper
chr5	33090876	33093922	3' UTR	0573	442E-04	937E-03	Rmdn1	20489	Hyper
chr13	44869368	44870745	Intron (NM_001105952/289026, intron 1 of 13)	0699	442E-04	938E-03	Sox13	4777	Hyper
chr1	199544882	199548768	Intron (NM_201350/171093, intron 16 of 22)	0513	443E-04	938E-03	Cttn	86400	Hyper
chr15	74907779	74909786	Distal Intergenic	0697	443E-04	938E-03	Setl1	-394012	Hyper
chr19	48164021	48166188	Distal Intergenic	0596	444E-04	939E-03	Zdhhc7	-7348	Hyper
chr4	159963278	159963820	Distal Intergenic	0817	444E-04	939E-03	Tigar	-17220	Hyper
chr20	28033260	28034033	Distal Intergenic	0922	444E-04	939E-03	Spock2	-3559	Hyper

chr5	121700738	121703515	Intron (NM_053358/84354, intron 4 of 16)	0620	446E-04	941E-03	Ssbp3	9345	Hyper
chr10	106114327	106115062	Distal Intergenic	0890	446E-04	941E-03	Fasn	-24068	Hyper
chr12	20757178	20758885	Intron (NM_019376/56010, intron 1 of 2)	0614	447E-04	941E-03	Ywhag	12598	Hyper
chr20	9955989	9959055	Promoter (<=1kb)	0474	447E-04	941E-03	Sik1	0	Hyper
chr7	108926753	108928946	Intron (NM_001079895/362950, intron 1 of 12)	0407	447E-04	942E-03	Rbfox2	125457	Hyper
chr2	241594693	241595256	Distal Intergenic	0922	447E-04	942E-03	Ak5	-13235	Hyper
chr8	109495874	109497467	Intron (NM_021660/59268, intron 2 of 6)	0563	448E-04	942E-03	Ip6k2	9695	Hyper
chr10	106535015	106537251	3' UTR	0516	448E-04	942E-03	Foxk2	-5798	Hyper
chr20	9646793	9647277	Distal Intergenic	1052	448E-04	943E-03	Pknox1	-33104	Hyper
chr16	45243734	45245688	Intron (NM_001107311/306460, intron 1 of 7)	0591	449E-04	945E-03	Enpp6	48389	Hyper
chr18	21845276	21845811	Promoter (<=1kb)	0744	449E-04	945E-03	Pik3c3	0	Hyper
chr2	142921794	142923453	Intron (NM_134457/140593, intron 1 of 1)	0609	451E-04	947E-03	Siah2	8299	Hyper
chr4	88380403	88382694	Distal Intergenic	0436	451E-04	947E-03	Tigd2	-31672	Hyper
chr12	38502308	38504240	Exon (NM_001107145/304522, exon 7 of 11)	0683	452E-04	948E-03	Fbxw8	75809	Hyper
chr15	13291410	13292834	Intron (NM_134356/171357, intron 2 of 32)	0663	452E-04	948E-03	Ptprg	259352	Hyper
chr16	18673882	18676768	Promoter (<=1kb)	0418	454E-04	952E-03	Ifi30	0	Hyper
chr2	219440226	219440656	Distal Intergenic	1079	456E-04	955E-03	Rpl34	-152118	Hyper
chr4	115421621	115422733	Intron (NM_019272/29745, intron 5 of 13)	0670	456E-04	955E-03	Sema4f	14988	Hyper
chr1	117529204	117533823	Promoter (2-3kb)	0431	458E-04	957E-03	Klf13	2803	Hyper
chr3	117982268	117983505	Intron (NM_001109206/499891, intron 17 of 36)	0708	458E-04	957E-03	Dnaaf9	69125	Hyper
chr5	76105609	76106481	Intron (NM_019340/54293, intron 9 of 15)	0837	459E-04	959E-03	Rgs3	27117	Hyper
chr12	22619069	22621750	Promoter (<=1kb)	0364	461E-04	961E-03	Castor2	0	Hyper
chr7	113559731	113561718	Exon (NM_139080/25019, exon 10 of 12)	0515	463E-04	964E-03	Snu13	9153	Hyper
chr11	82281190	82284788	Distal Intergenic	0473	463E-04	965E-03	Cldn5	-66942	Hyper
chr1	138346320	138350235	Exon (NM_012781/25243, exon 2 of 19)	0448	464E-04	966E-03	Arnt2	42531	Hyper
chr1	121214508	121215566	Intron (NM_001108486/361588, intron 5 of 9)	0729	464E-04	967E-03	Lrrc28	30197	Hyper
chr5	165858359	165864307	Exon (NM_022507/25522, exon 5 of 18)	0345	466E-04	969E-03	Faap20	49702	Hyper
chr4	123422291	123423644	Distal Intergenic	0620	468E-04	972E-03	Nup210	186230	Hyper
chr3	9397809	9400250	Distal Intergenic	0524	469E-04	974E-03	Egfl7	-7294	Hyper
chr7	63100420	63100896	Exon (NM_001004239/299850, exon 3 of 14)	0980	470E-04	975E-03	Dctn2	8269	Hyper
chr19	20381413	20382776	Promoter (2-3kb)	0712	471E-04	976E-03	Siah1	2505	Hyper
chr1	180120053	180120617	Intron (NM_133380/25084, intron 1 of 10)	1046	471E-04	977E-03	Il4r	4936	Hyper
chr1	164903519	164906114	Intron (NM_031544/25095, intron 2 of 14)	0493	471E-04	977E-03	Ampd3	18199	Hyper

chr6	106677336	106678458	Intron (NM_001108045/314332, intron 1 of 24)	0726	472E-04	978E-03	Tmem63c	4402	Hyper
chr19	39747074	39747638	Intron (NM_199378/292027, intron 5 of 6)	1039	474E-04	981E-03	Bcar1	-44791	Hyper
chr10	36539325	36542065	Intron (NM_031353/83529, intron 1 of 8)	0423	475E-04	982E-03	Vdac1	7019	Hyper
chr1	151182339	151191687	Exon (NM_001191628/308831, exon 18 of 32)	0342	475E-04	982E-03	Nars2	-108815	Hyper
chr4	152600065	152601600	Distal Intergenic	0642	476E-04	983E-03	Wnt5b	23715	Hyper
chr7	43806188	43807732	Distal Intergenic	0661	476E-04	983E-03	Pawr	160413	Hyper
chr1	82795009	82795709	Exon (NM_023976/78960, exon 5 of 7)	0714	477E-04	985E-03	Prx	8192	Hyper
chr1	33651363	33652000	Distal Intergenic	0796	478E-04	986E-03	Nsun2	33862	Hyper
chr4	149054204	149055336	Distal Intergenic	0772	478E-04	986E-03	Plxnd1	-11735	Hyper
chr8	107075451	107080300	Promoter (<=1kb)	0354	479E-04	986E-03	Acy1	0	Hyper
chr19	50159043	50159706	Distal Intergenic	0787	479E-04	986E-03	Banp	151179	Hyper
chr8	103361758	103362842	Distal Intergenic	0788	480E-04	988E-03	Ryk	-56658	Hyper
chr4	109638084	109640729	Intron (NM_001106598/297357, intron 7 of 18)	0538	482E-04	992E-03	LRRTM1	-61086	Hyper
chr13	102924549	102929046	Exon (NM_001007679/305070, exon 5 of 8)	0377	483E-04	992E-03	Pacc1	19825	Hyper
chr10	85344226	85346510	Promoter (<=1kb)	0526	484E-04	993E-03	P3h4	0	Hyper
chr1	155280391	155290878	Distal Intergenic	0326	484E-04	993E-03	P2ry6	29164	Hyper
chr11	82294995	82296901	Distal Intergenic	0527	486E-04	997E-03	Septin5	-76700	Hyper
chr6	121790956	121792504	Intron (NM_001191985/500709, intron 2 of 9)	0661	486E-04	997E-03	Itpk1	51534	Hyper
chr5	136439996	136442254	Distal Intergenic	0564	486E-04	997E-03	Lnc081	49114	Hyper
chr1	85999431	86002105	Promoter (<=1kb)	0517	489E-04	100E-02	Sbsn	0	Hyper
chr11	39887127	39887749	Distal Intergenic	0787	489E-04	100E-02	Arl6	-824390	Hyper
chr1	171918833	171920670	Intron (NM_022295/64133, intron 11 of 11)	0602	493E-04	101E-02	Xylt1	274908	Hyper
chr9	84644841	84645801	Intron (NM_001127492/316561, intron 1 of 13)	0742	493E-04	101E-02	Sphkap	11645	Hyper
chr2	141515656	141516489	Distal Intergenic	0832	493E-04	101E-02	Tm4sf1	-49510	Hyper
chr18	54227210	54229937	Promoter (<=1kb)	0373	494E-04	101E-02	Rps14	0	Hyper
chr2	61262560	61267545	Exon (NM_001012077/310155, exon 7 of 15)	0327	494E-04	101E-02	Mtmr12	38694	Hyper
chr6	121460402	121460810	Intron (NM_001108052/314397, intron 1 of 8)	1076	495E-04	101E-02	Rin3	28596	Hyper
chr2	235759126	235760537	Distal Intergenic	0567	496E-04	101E-02	Prkacb	-33074	Hyper
chr7	111876829	111880735	Intron (NM_020084/56827, intron 3 of 37)	0517	497E-04	101E-02	Cacna1i	30091	Hyper
chr1	56367578	56368080	Intron (NM_001107466/308218, intron 6 of 9)	0901	497E-04	101E-02	Fam120b	28449	Hyper
chr2	219460925	219461205	Distal Intergenic	1292	497E-04	101E-02	Rpl34	-172817	Hyper
chr5	95679023	95679438	Distal Intergenic	1011	499E-04	101E-02	Mpdz	241061	Hyper
chr8	20010466	20012788	Intron (NM_013199/25751, intron 2 of 20)	0566	500E-04	102E-02	Dnm2	32016	Hyper

chr19	48025862	48026493	Distal Intergenic	1000	503E-04	102E-02	Crispld2	-43846	Hyper
chr6	123771479	123777181	Intron (NM_001106755/299285, intron 1 of 12)	0404	504E-04	102E-02	Clmn	30091	Hyper
chr3	141200091	141200754	Distal Intergenic	0804	504E-04	102E-02	Id1	-10539	Hyper
chr16	1273149	1274361	Distal Intergenic	0695	505E-04	102E-02	Ppif	15952	Hyper
chr3	154656756	154659831	Distal Intergenic	0421	505E-04	102E-02	Zmynd8	-29590	Hyper
chr17	44737540	44739768	Intron (NM_001108415/361251, intron 1 of 21)	0582	505E-04	102E-02	Elmo1	83020	Hyper
chr7	65704525	65705910	Promoter (<=1kb)	0557	507E-04	102E-02	Rida	0	Hyper
chr5	42949903	42950354	Distal Intergenic	1017	509E-04	103E-02	Epha7	-25124	Hyper
chr9	84645889	84646755	Intron (NM_001127492/316561, intron 1 of 13)	0704	509E-04	103E-02	Sphkap	10691	Hyper
chr5	161354305	161354717	3' UTR	1095	509E-04	103E-02	Park7	17405	Hyper
chr11	66828811	66832142	3' UTR	0471	511E-04	103E-02	Umps	22702	Hyper
chr8	110312345	110313073	Intron (NM_001100966/301024, intron 1 of 23)	0830	512E-04	103E-02	Scap	6314	Hyper
chr7	65934519	65935670	Distal Intergenic	0797	512E-04	103E-02	Nipal2	-49712	Hyper
chr6	9616738	9617519	Intron (NM_017216/29484, intron 7 of 9)	0924	514E-04	103E-02	Slc3a1	24362	Hyper
chr5	144226188	144228392	Distal Intergenic	0623	515E-04	103E-02	Oprd1	112568	Hyper
chr10	101340727	101342625	Promoter (<=1kb)	0590	515E-04	104E-02	Trim47	0	Hyper
chr8	101852820	101854169	Distal Intergenic	0669	516E-04	104E-02	Ppp2r3a	-54299	Hyper
chr14	12770282	12770753	Intron (NM_012823/25291, intron 2 of 12)	1026	517E-04	104E-02	Anxa3	10881	Hyper
chr16	80478289	80479851	Distal Intergenic	0712	519E-04	104E-02	Arglu1	-273464	Hyper
chr1	80971634	80978368	Exon (NM_012859/25330, exon 2 of 10)	0362	521E-04	104E-02	Lipe	5942	Hyper
chr9	65482707	65484917	Promoter (<=1kb)	0511	523E-04	105E-02	Klf7	0	Hyper
chr3	154679144	154679825	Distal Intergenic	0726	523E-04	105E-02	Zmynd8	-51978	Hyper
chr8	11222198	11225472	Distal Intergenic	0433	524E-04	105E-02	Kdm4d	79448	Hyper
chr7	51954991	51955555	Promoter (1-2kb)	1015	526E-04	105E-02	Ptprb	1616	Hyper
chr17	32637873	32639387	Promoter (2-3kb)	0690	528E-04	105E-02	Foxc1	-2512	Hyper
chr11	69667909	69669762	Distal Intergenic	0522	528E-04	105E-02	Acap2	129363	Hyper
chr4	77600142	77600720	Promoter (<=1kb)	0923	529E-04	105E-02	Gimap8	0	Hyper
chr8	43300454	43301992	Distal Intergenic	0612	529E-04	105E-02	Grik4	-95377	Hyper
chr10	20089467	20091198	Intron (NM_031321/83467, intron 20 of 35)	0625	530E-04	106E-02	Mir218-2	21016	Hyper
chr10	36119452	36125015	Promoter (<=1kb)	0304	531E-04	106E-02	Jade2	0	Hyper
chr3	135866072	135867177	Promoter (<=1kb)	0685	533E-04	106E-02	Thbd	0	Hyper
chr4	83545479	83546746	Distal Intergenic	0727	534E-04	106E-02	Wipf3	-3722	Hyper
chr7	115378201	115379689	Intron (NM_001134780/362973, intron 1 of 12)	0735	534E-04	106E-02	Parvb	17947	Hyper

chr4	123220597	123222566	Distal Intergenic	0485	535E-04	106E-02	Slc41a3	92834	Hyper
chr4	14792612	14798048	Intron (NM_053621/113970, intron 6 of 22)	0350	536E-04	106E-02	Phtf2	577349	Hyper
chr2	152835761	152841507	Intron (NM_001100666/295105, intron 5 of 10)	0346	536E-04	106E-02	Il12a	-124262	Hyper
chr14	33967529	33968809	Intron (NM_001017499/498353, intron 6 of 8)	0661	536E-04	106E-02	Rasl11b	50337	Hyper
chr4	63544193	63544790	Intron (NM_001009709/362334, intron 1 of 1)	0915	537E-04	106E-02	Cyren	6812	Hyper
chr10	34476529	34478170	Exon (NM_001108274/360520, exon 7 of 23)	0692	539E-04	107E-02	Tbc1d9b	16576	Hyper
chr1	86232107	86233357	Distal Intergenic	0772	540E-04	107E-02	Lsr	-30155	Hyper
chr7	94111895	94112313	Distal Intergenic	1071	540E-04	107E-02	Myc	518190	Hyper
chr1	146860125	146862805	Distal Intergenic	0480	540E-04	107E-02	Rab30	-13766	Hyper
chr3	17133132	17134778	Exon (NM_001271232/362118, exon 5 of 10)	0611	541E-04	107E-02	Mvb12b	57880	Hyper
chr1	187832350	187834266	Distal Intergenic	0549	543E-04	107E-02	Ctbp2	-20502	Hyper
chr8	46043684	46046358	Exon (NM_001108141/315615, exon 21 of 33)	0515	546E-04	108E-02	Bace1	-95766	Hyper
chr3	55400622	55401714	Exon (NM_017007/24379, exon 13 of 17)	0791	546E-04	108E-02	Gad1	30918	Hyper
chr9	105358658	105359022	Intron (NM_145094/246324, intron 1 of 6)	1066	547E-04	108E-02	Rab31	22204	Hyper
chr13	43998628	44000981	3' UTR	0445	549E-04	108E-02	Cntn2	-22743	Hyper
chr17	72011591	72012782	Distal Intergenic	0717	549E-04	108E-02	Usp6nl	-55661	Hyper
chr2	182631204	182638566	Exon (NM_001042621/365865, exon 8 of 15)	0331	552E-04	109E-02	Psmd4	-23010	Hyper
chr5	134171020	134172856	Distal Intergenic	0523	552E-04	109E-02	Ctps1	-16865	Hyper
chr3	168805134	168806890	Promoter (2-3kb)	0582	553E-04	109E-02	Rgs19	2888	Hyper
chr19	21199267	21200590	Intron (NM_001014152/361377, intron 1 of 29)	0554	553E-04	109E-02	Phkb	10015	Hyper
chr18	73763315	73765293	Intron (NM_001107372/307234, intron 2 of 2)	0551	554E-04	109E-02	Kcng2	43430	Hyper
chr11	55559391	55560136	Distal Intergenic	0771	555E-04	109E-02	Btla	26846	Hyper
chr10	91509710	91511102	Distal Intergenic	0626	556E-04	109E-02	Ern1	-117831	Hyper
chr2	140307816	140308451	Distal Intergenic	0821	556E-04	109E-02	Mab21l1	364320	Hyper
chr15	39847512	39849631	Distal Intergenic	0569	559E-04	110E-02	Scara5	-30423	Hyper
chr10	105232362	105234274	Intron (NM_057196/117542, intron 1 of 13)	0548	560E-04	110E-02	Baiap2	9230	Hyper
chr3	125398423	125399320	Distal Intergenic	0657	561E-04	110E-02	Btbd3	-213373	Hyper
chr16	19449456	19451174	Intron (NM_001013881/290669, intron 1 of 12)	0423	564E-04	110E-02	Gatad2a	19878	Hyper
chr14	103995588	103998333	Intron (NM_001013130/305614, intron 1 of 36)	0494	564E-04	110E-02	Sptbn1	10174	Hyper
chr20	9998644	10000249	Distal Intergenic	0660	564E-04	110E-02	Sik1	-39705	Hyper
chr10	37066373	37067121	Intron (NM_001107000/303130, intron 3 of 14)	0893	565E-04	111E-02	Fstl4	154953	Hyper
chr15	2881167	2882175	Intron (NM_012895/25368, intron 10 of 10)	0663	566E-04	111E-02	Dusp29	287589	Hyper
chr1	196137303	196138101	Distal Intergenic	0838	566E-04	111E-02	Ifitm3	-25168	Hyper

chr1	230079139	230082219	Intron (NM_181386/353229, intron 5 of 9)	0482	566E-04	111E-02	Asah2	-139977	Hyper
chr2	143053217	143053982	Distal Intergenic	0885	567E-04	111E-02	Clrn1	76966	Hyper
chr11	65593164	65595645	Intron (NM_022600/64532, intron 1 of 20)	0547	567E-04	111E-02	Adcy5	22456	Hyper
chr1	155266441	155268068	Distal Intergenic	0680	568E-04	111E-02	Mir3102	-22549	Hyper
chr3	79757500	79762186	Intron (NM_001107751/311215, intron 7 of 12)	0385	570E-04	111E-02	Ext2	35873	Hyper
chr4	58579306	58580440	Distal Intergenic	0689	570E-04	111E-02	Nrf1	-84550	Hyper
chr11	78366198	78367166	Distal Intergenic	0714	574E-04	112E-02	Dgkg	-17296	Hyper
chr9	94315805	94316431	3' UTR	0970	576E-04	112E-02	Dtymk	7955	Hyper
chr1	121826984	121828941	Intron (NM_052807/25718, intron 20 of 20)	0577	577E-04	112E-02	Igf1r	276243	Hyper
chr12	6483118	6484404	Intron (NM_001015030/363869, intron 1 of 4)	0659	578E-04	112E-02	Ubl3	9434	Hyper
chr8	19292645	19295768	Distal Intergenic	0393	579E-04	112E-02	Olfm2	-29194	Hyper
chr1	176862615	176863382	Intron (NM_012713/25023, intron 2 of 17)	0886	580E-04	112E-02	Prkcb	29930	Hyper
chr1	52513581	52514196	Distal Intergenic	0868	580E-04	112E-02	Ccr6	-15011	Hyper
chr11	67199637	67200294	Intron (NM_153625/266733, intron 4 of 12)	0841	583E-04	113E-02	Slc12a8	65510	Hyper
chr10	13655135	13656789	Promoter (<=1kb)	0566	583E-04	113E-02	Tsc2	0	Hyper
chr3	168762524	168763592	Exon (NM_001079766/366276, exon 15 of 21)	0666	586E-04	113E-02	Linc00176	-9021	Hyper
chr16	71387712	71388242	Distal Intergenic	0860	586E-04	113E-02	Angpt2	299348	Hyper
chr17	74527683	74529007	Intron (NM_001025034/498796, intron 1 of 3)	0645	587E-04	113E-02	Fam107b	20595	Hyper
chr3	156673845	156680557	Exon (NM_012637/24697, exon 4 of 9)	0384	588E-04	113E-02	Ptpn1	35034	Hyper
chr3	76445645	76447476	Exon (NM_017269/29645, exon 6 of 24)	0492	588E-04	113E-02	Or4x13	81613	Hyper
chr20	10384135	10385144	Promoter (<=1kb)	0715	589E-04	113E-02	Agpat3	0	Hyper
chr3	9216911	9220123	3' UTR	0551	590E-04	114E-02	Pmpca	9180	Hyper
chr1	239509148	239510879	Intron (NM_022587/64519, intron 1 of 9)	0539	590E-04	114E-02	Entpd1	35706	Hyper
chr2	152856583	152861267	Intron (NM_001100666/295105, intron 6 of 10)	0365	592E-04	114E-02	Il12a	-104502	Hyper
chr2	184206769	184207818	Distal Intergenic	0640	593E-04	114E-02	Itga10	23900	Hyper
chr5	122704509	122704869	Distal Intergenic	1250	594E-04	114E-02	Cpt2	-22463	Hyper
chr2	142223824	142225396	Distal Intergenic	0517	596E-04	114E-02	Pfn2	-151016	Hyper
chr5	116181948	116188238	Intron (NM_001107949/313409, intron 1 of 17)	0411	596E-04	114E-02	Dnajc6	51272	Hyper
chr1	205872948	205874148	Distal Intergenic	0603	596E-04	114E-02	Ahnak	-8125	Hyper
chr17	1801226	1805871	3' UTR	0456	597E-04	114E-02	Mir3074	-7057	Hyper
chr4	67711511	67714863	Intron (NM_012687/24886, intron 4 of 13)	0417	597E-04	115E-02	Tbxas1	46504	Hyper
chr14	75665812	75670171	3' UTR	0388	600E-04	115E-02	Lrpap1	14441	Hyper
chr1	254101713	254103512	Distal Intergenic	0572	600E-04	115E-02	Gpam	67049	Hyper

chr4	87744930	87745753	Promoter (<=1kb)	0728	600E-04	115E-02	Abcg2	0	Hyper
chr18	69014700	69016396	3' UTR	0562	601E-04	115E-02	Smad7	26271	Hyper
chr7	10098698	10100790	Distal Intergenic	0609	601E-04	115E-02	C2cd4c	6777	Hyper
chr4	179402815	179404874	Promoter (<=1kb)	0389	603E-04	115E-02	Itpr2	0	Hyper
chr4	1859058	1859922	Distal Intergenic	0540	604E-04	115E-02	Il6	3359256	Hyper
chr10	101662248	101665747	Distal Intergenic	0344	606E-04	116E-02	Prpsap1	57045	Hyper
chr16	77335510	77336014	Distal Intergenic	0999	606E-04	116E-02	Tex29	328102	Hyper
chr12	7221629	7222251	Distal Intergenic	0780	607E-04	116E-02	Pomp	-46957	Hyper
chr12	14839273	14841177	Intron (NM_145673/246760, intron 1 of 2)	0482	607E-04	116E-02	Mafk	4168	Hyper
chrX	120435426	120436699	Intron (NM_001112742/29628, intron 10 of 15)	0701	608E-04	116E-02	Gria3	196438	Hyper
chr3	16020857	16021913	Intron (NM_001108579/362111, intron 2 of 11)	0864	610E-04	116E-02	Sh2d3c	10232	Hyper
chr5	144926067	144926898	Intron (NM_001107910/313027, intron 16 of 17)	0852	610E-04	116E-02	Xkr8	18297	Hyper
chr1	181264088	181265025	Exon (NM_001114502/293488, exon 4 of 9)	0750	612E-04	116E-02	Sult1a1	10518	Hyper
chr1	45744760	45746407	Distal Intergenic	0524	612E-04	116E-02	Ldhal6b	252861	Hyper
chr4	157505218	157506218	Exon (NM_001012189/362434, exon 4 of 13)	0783	612E-04	116E-02	Emg1	11322	Hyper
chr1	46528647	46530403	Intron (NM_032071/84018, intron 1 of 27)	0529	612E-04	116E-02	Synj2	9889	Hyper
chr12	36027429	36031093	3' UTR	0386	613E-04	117E-02	Slc8b1	21938	Hyper
chr1	220628825	220629787	Distal Intergenic	0716	615E-04	117E-02	Klf9	-70321	Hyper
chr5	165878355	165882162	Intron (NM_022507/25522, intron 4 of 17)	0394	616E-04	117E-02	Prkcz	48205	Hyper
chr10	86394726	86397527	Promoter (<=1kb)	0366	616E-04	117E-02	Vat1	0	Hyper
chr2	152841912	152844671	Intron (NM_001100666/295105, intron 5 of 10)	0481	617E-04	117E-02	Il12a	-121098	Hyper
chr1	108196464	108197296	Intron (NM_024370/79211, intron 2 of 9)	0720	617E-04	117E-02	Gabrg3	49441	Hyper
chr1	161581769	161583502	Intron (NM_001100582/308918, intron 3 of 23)	0532	618E-04	117E-02	Ppfibp2	76879	Hyper
chr8	58137253	58138746	Distal Intergenic	0526	618E-04	117E-02	Clk3	27029	Hyper
chr14	12766443	12767092	Intron (NM_012823/25291, intron 2 of 12)	0894	619E-04	117E-02	Anxa3	14542	Hyper
chr17	44728587	44735638	5' UTR	0410	620E-04	118E-02	Elmo1	87150	Hyper
chr12	42115104	42117023	Distal Intergenic	0638	621E-04	118E-02	Mvk	41835	Hyper
chr4	174824000	174824536	Distal Intergenic	1015	624E-04	118E-02	Slco1a4	-59245	Hyper
chr3	168790771	168791356	Distal Intergenic	0741	627E-04	118E-02	Sox18	-3481	Hyper
chr18	74965093	74967260	Distal Intergenic	0468	629E-04	119E-02	Sall3	-551265	Hyper
chr12	41095781	41096955	Intron (NM_001012147/360820, intron 1 of 9)	0757	630E-04	119E-02	Pxn	10965	Hyper
chr3	119856326	119860504	Distal Intergenic	0370	630E-04	119E-02	Gpcpd1	-23873	Hyper
chr19	10030757	10035359	Intron (NM_152242/260326, intron 1 of 13)	0395	631E-04	119E-02	Adgrg1	5763	Hyper

chr10	105974502	105977525	Distal Intergenic	0442	631E-04	119E-02	Lrrc45	-16994	Hyper
chr12	21133219	21135077	Promoter (<=1kb)	0465	632E-04	119E-02	Hip1	0	Hyper
chr16	18878531	18884014	Intron (NM_001107304/306347, intron 1 of 11)	0355	633E-04	119E-02	Ell	7077	Hyper
chr8	72423959	72425086	Promoter (1-2kb)	0853	633E-04	119E-02	Cgnl1	-1042	Hyper
chr13	96430835	96432112	Promoter (<=1kb)	0672	634E-04	119E-02	RGD1310587	0	Hyper
chr2	164613483	164614182	Intron (NM_001271167/310538, intron 14 of 16)	0809	636E-04	119E-02	Fnip2	93719	Hyper
chr15	39118314	39119222	Distal Intergenic	0698	637E-04	119E-02	Extl3	200250	Hyper
chr10	90962184	90963211	Exon (NM_053937/116745, exon 7 of 13)	0535	637E-04	119E-02	Dcaf7	-10884	Hyper
chr19	35309298	35311884	Promoter (<=1kb)	0412	638E-04	120E-02	Nqo1	0	Hyper
chr1	97368454	97368914	Promoter (2-3kb)	0955	639E-04	120E-02	Ldha	-2909	Hyper
chr14	73891960	73901131	Intron (NM_057116/117256, intron 1 of 8)	0328	640E-04	120E-02	Ppp2r2c	30047	Hyper
chr8	20231793	20233852	Exon (NM_134368/171379, exon 25 of 34)	0519	640E-04	120E-02	Ldlr	-36244	Hyper
chr10	16724863	16725338	Distal Intergenic	1046	643E-04	120E-02	Dusp1	44374	Hyper
chr3	90823527	90824243	Intron (NM_031787/83617, intron 2 of 15)	0857	643E-04	120E-02	Hipk3	42069	Hyper
chr8	54255348	54256557	Distal Intergenic	0600	645E-04	120E-02	Sln	11806	Hyper
chr10	82055863	82057224	Distal Intergenic	0566	645E-04	120E-02	Osbpl7	19821	Hyper
chr5	103873925	103875790	Promoter (<=1kb)	0431	646E-04	121E-02	Mtap	905	Hyper
chr17	32633004	32635114	Promoter (<=1kb)	0647	646E-04	121E-02	Foxc1	247	Hyper
chr18	25453195	25454685	Intron (NM_001107397/307514, intron 7 of 17)	0687	647E-04	121E-02	Epb41l4a	32414	Hyper
chr6	27699664	27702637	Distal Intergenic	0537	649E-04	121E-02	Fam228a	47424	Hyper
chr17	5174402	5180116	Exon (NM_001106100/290986, exon 13 of 27)	0426	650E-04	121E-02	Agtpbp1	40704	Hyper
chr14	77891497	77895532	Distal Intergenic	0393	651E-04	121E-02	Depdc5	-28727	Hyper
chr2	11354279	11356797	Distal Intergenic	0515	651E-04	121E-02	Arrdc3	216579	Hyper
chr5	135296769	135298788	Promoter (1-2kb)	0515	652E-04	121E-02	Trit1	1435	Hyper
chr12	34391305	34392172	Intron (NM_001270983/24669, intron 1 of 7)	0676	653E-04	121E-02	Ppp1cc	8381	Hyper
chr11	67101695	67103301	Distal Intergenic	0692	654E-04	121E-02	Itgb5	-157464	Hyper
chr3	14068946	14070831	Distal Intergenic	0610	654E-04	121E-02	C3h9orf50	16147	Hyper
chr10	67880433	67883241	Distal Intergenic	0542	654E-04	121E-02	Unc45b	34971	Hyper
chr14	80974406	80975239	Distal Intergenic	0835	654E-04	121E-02	Camk2b	-40412	Hyper
chrX	151233645	151234477	Intron (NM_133288/29599, intron 2 of 22)	0788	656E-04	122E-02	Atp2b3	17138	Hyper
chr18	26056114	26062021	Exon (NM_001106158/291694, exon 12 of 21)	0347	657E-04	122E-02	Fam13b	44566	Hyper
chr4	181911463	181913342	Promoter (<=1kb)	0592	657E-04	122E-02	Sinhcaf	0	Hyper
chr19	48352751	48355447	Distal Intergenic	0415	659E-04	122E-02	6430548M08Rikl	154542	Hyper

chr3	88168604	88169242	Distal Intergenic	0946	660E-04	122E-02	Commd9	-6751	Hyper
chr8	6535447	6538901	Distal Intergenic	0467	660E-04	122E-02	Pgr	462231	Hyper
chrX	21917366	21917990	Intron (NM_001047893/317435, intron 1 of 9)	0921	660E-04	122E-02	Shroom2	65509	Hyper
chr10	15936853	15940738	Distal Intergenic	0363	660E-04	122E-02	Bod1	-52716	Hyper
chr1	108195946	108196245	Intron (NM_024370/79211, intron 2 of 9)	1004	660E-04	122E-02	Gabrg3	50492	Hyper
chr13	102918399	102921391	Exon (NM_001007679/305070, exon 3 of 8)	0482	667E-04	123E-02	Pacc1	13675	Hyper
chr10	44759508	44761221	Intron (NM_001127302/497914, intron 1 of 1)	0509	668E-04	123E-02	Rasd1	6937	Hyper
chr5	75961383	75962960	3' UTR	0596	668E-04	123E-02	Hdhd3	-6608	Hyper
chr7	65732445	65732950	3' UTR	0834	669E-04	123E-02	Pop1	27058	Hyper
chr5	60983424	60985137	Intron (NM_031802/83633, intron 13 of 19)	0599	670E-04	123E-02	Tbc1d2	-52072	Hyper
chr4	83600437	83601180	Intron (NM_147211/259242, intron 2 of 7)	0824	670E-04	123E-02	Wipf3	49969	Hyper
chr8	42816732	42817908	Intron (NM_001014089/315591, intron 7 of 7)	0718	670E-04	123E-02	Tbcel	36644	Hyper
chr10	100847355	100848845	Promoter (2-3kb)	0579	671E-04	123E-02	Mif4gd	2788	Hyper
chr12	41098837	41100348	Intron (NM_001012147/360820, intron 1 of 9)	0560	672E-04	124E-02	Pxn	7572	Hyper
chr20	18675041	18676074	Exon (NM_031805/361833, exon 27 of 45)	0651	674E-04	124E-02	Mrln	-253231	Hyper
chr5	96924650	96926571	Intron (NM_031566/29227, intron 2 of 10)	0539	676E-04	124E-02	Nfib	46411	Hyper
chr1	175396910	175399437	Distal Intergenic	0537	680E-04	125E-02	Eef2k	-12181	Hyper
chr8	109729953	109731708	Promoter (<=1kb)	0425	682E-04	125E-02	Tma7	0	Hyper
chr5	76080268	76084642	Promoter (1-2kb)	0420	684E-04	125E-02	Rgs3	1776	Hyper
chr3	10344552	10350849	3' UTR	0400	684E-04	125E-02	Slc2a6	4359	Hyper
chr4	78058803	78059670	Intron (NM_001106593/297082, intron 3 of 3)	0729	685E-04	125E-02	Malsu1	7713	Hyper
chr2	173946481	173956313	Exon (NM_001002016/60374, exon 2 of 12)	0302	687E-04	126E-02	Lmna	4110	Hyper
chr6	102030634	102035980	Intron (NM_139330/246212, intron 4 of 20)	0337	689E-04	126E-02	Sipa1l1	24454	Hyper
chr2	227364287	227367742	Intron (NM_001004090/362048, intron 1 of 7)	0439	689E-04	126E-02	Tspan5	70070	Hyper
chr17	12602258	12603243	Distal Intergenic	0878	689E-04	126E-02	Syk	58162	Hyper
chr3	141198208	141199997	Distal Intergenic	0637	693E-04	126E-02	Id1	-11296	Hyper
chr1	203688170	203689378	Promoter (1-2kb)	0723	699E-04	127E-02	Pygm	-1172	Hyper
chr1	77566482	77567565	Promoter (<=1kb)	0780	700E-04	127E-02	Gng8	73	Hyper
chr13	45731183	45733714	Distal Intergenic	0449	700E-04	127E-02	Myog	-11741	Hyper
chr1	232101642	232103637	Promoter (<=1kb)	0478	701E-04	127E-02	Ifit2	0	Hyper
chr12	13077620	13078402	Distal Intergenic	0809	703E-04	128E-02	Gna12	-727296	Hyper
chr10	42257260	42257685	Distal Intergenic	1017	708E-04	128E-02	Cnot8	-24094	Hyper
chr14	41560389	41565121	Distal Intergenic	0377	709E-04	129E-02	Uchl1	-64799	Hyper

chr19	18309905	18311501	Distal Intergenic	0627	709E-04	129E-02	Cyld	62149	Hyper
chr13	67317599	67319117	Promoter (<=1kb)	0451	710E-04	129E-02	Mr1	0	Hyper
chr12	21216339	21217456	Exon (NM_001100475/192154, exon 3 of 31)	0742	712E-04	129E-02	Pom121	-59489	Hyper
chr1	45141305	45141719	Distal Intergenic	0857	712E-04	129E-02	Nox3	-838454	Hyper
chr20	31817504	31818392	Intron (NM_001164157/294400, intron 1 of 4)	0694	713E-04	129E-02	Nus1	5687	Hyper
chr8	71602762	71606217	Intron (NM_012597/24538, intron 1 of 8)	0512	715E-04	129E-02	Lipc	29247	Hyper
chr4	158402778	158403123	Intron (NM_053889/116669, intron 10 of 51)	1249	715E-04	129E-02	Vwf	42626	Hyper
chr1	23353700	23358620	Distal Intergenic	0443	717E-04	130E-02	Sgk1	-365603	Hyper
chr8	45024061	45026363	Exon (NM_001191578/171434, exon 9 of 22)	0440	719E-04	130E-02	Phldb1	18161	Hyper
chr6	121810512	121812471	Intron (NM_001191985/500709, intron 2 of 9)	0536	720E-04	130E-02	Itpk1	31567	Hyper
chr20	13740106	13740828	Distal Intergenic	0737	720E-04	130E-02	Gnaz	-70199	Hyper
chr6	25289994	25291921	Distal Intergenic	0536	721E-04	130E-02	Slc30a3	12549	Hyper
chr3	9222459	9223552	Intron (NM_053632/114089, intron 1 of 9)	0679	725E-04	131E-02	Inpp5e	5898	Hyper
chr7	132233876	132234444	Distal Intergenic	0889	729E-04	131E-02	Acvrl1	-11067	Hyper
chr1	197185885	197186351	Promoter (2-3kb)	0825	732E-04	131E-02	Dusp8	-2964	Hyper
chr12	1329602	1331688	Promoter (<=1kb)	0366	733E-04	132E-02	Insr	0	Hyper
chr1	81288752	81290043	Intron (NM_001013147/308444, intron 4 of 18)	0727	734E-04	132E-02	Axl	6000	Hyper
chr6	10707013	10709492	Distal Intergenic	0467	734E-04	132E-02	Haao	-136293	Hyper
chr3	152393066	152397507	3' UTR	0423	735E-04	132E-02	Ada	25347	Hyper
chr10	47299054	47301163	Distal Intergenic	0565	735E-04	132E-02	Trpv2	26112	Hyper
chr1	230162824	230163756	5' UTR	0763	735E-04	132E-02	Sgms1	95836	Hyper
chr1	68750132	68755337	Exon (NM_057136/117277, exon 3 of 11)	0393	736E-04	132E-02	Epn1	3531	Hyper
chr10	85460215	85464206	Promoter (<=1kb)	0352	745E-04	133E-02	Acly	0	Hyper
chr4	146104114	146105368	Distal Intergenic	0603	745E-04	133E-02	Srgap3	-34390	Hyper
chr3	77992238	77993748	Promoter (<=1kb)	0617	749E-04	133E-02	Creb3l1	0	Hyper
chr20	19043945	19051477	Intron (NM_031805/361833, intron 1 of 44)	0322	752E-04	134E-02	Ank3	34527	Hyper
chr10	62333916	62352910	Promoter (<=1kb)	0291	752E-04	134E-02	Git1	0	Hyper
chr20	54405135	54408113	Distal Intergenic	0348	754E-04	134E-02	Cd99	14720	Hyper
chr10	62325298	62326350	Distal Intergenic	0734	754E-04	134E-02	Coro6	12223	Hyper
chr2	97725880	97726510	Distal Intergenic	0778	754E-04	134E-02	Hnf4g	310082	Hyper
chr9	1397483	1400256	Exon (NM_001303144/301126, exon 14 of 21)	0431	756E-04	134E-02	Tincr	-8146	Hyper
chr10	94041430	94041925	Distal Intergenic	0937	756E-04	134E-02	Axin2	142185	Hyper
chr3	155219763	155220961	Distal Intergenic	0618	758E-04	134E-02	Prex1	235727	Hyper

chr5	165890391	165893816	Intron (NM_022507/25522, intron 4 of 17)	0440	759E-04	135E-02	Prkcz	36551	Hyper
chr17	6853486	6854475	Intron (NM_001271297/306759, intron 2 of 10)	0721	760E-04	135E-02	Spock1	111973	Hyper
chr14	78097815	78098427	3' UTR	0827	762E-04	135E-02	Drg1	21481	Hyper
chr17	54508475	54509697	Exon (NM_001309462/100362678, exon 11 of 14)	0636	763E-04	135E-02	Epc1	84728	Hyper
chr10	59411273	59412845	Distal Intergenic	0461	764E-04	135E-02	Cluh	-96881	Hyper
chr10	64001086	64002927	Intron (NM_001382488/108348076, intron 2 of 17)	0508	765E-04	135E-02	Lgals9	-71041	Hyper
chr4	154198176	154207770	Exon (NM_001191085/362427, exon 21 of 33)	0321	765E-04	135E-02	Bid	-62023	Hyper
chr19	48413891	48418783	Distal Intergenic	0391	765E-04	135E-02	6430548M08Rikl	215682	Hyper
chr3	76526091	76528266	Intron (NM_017269/29645, intron 1 of 23)	0502	766E-04	135E-02	Ptprj	33403	Hyper
chr3	15934472	15936252	Promoter (<=1kb)	0610	767E-04	135E-02	Eng	0	Hyper
chr7	109493410	109494690	Distal Intergenic	0773	767E-04	135E-02	Txn2	15688	Hyper
chr7	33142820	33143329	Distal Intergenic	0831	767E-04	135E-02	Atp2b1	-592646	Hyper
chr1	70035398	70038045	Promoter (<=1kb)	0376	768E-04	136E-02	Cdc42ep5	0	Hyper
chr8	43851078	43852640	Distal Intergenic	0538	769E-04	136E-02	Trim29	168857	Hyper
chr5	147472580	147474651	Intron (NM_031818/83718, intron 2 of 5)	0447	770E-04	136E-02	Clic4	38627	Hyper
chr19	36835164	36837329	Distal Intergenic	0531	770E-04	136E-02	Pmfbp1	-622129	Hyper
chr1	23350748	23353586	Distal Intergenic	0412	772E-04	136E-02	Sgk1	-362651	Hyper
chr11	58405690	58406671	Intron (NM_017195/29423, intron 1 of 2)	0733	774E-04	136E-02	Gap43	29319	Hyper
chr14	72195828	72196110	Distal Intergenic	1244	775E-04	136E-02	Wdr1	-61922	Hyper
chr10	46295718	46296802	Distal Intergenic	0715	775E-04	136E-02	Epn2	-36076	Hyper
chr13	47075670	47076655	Distal Intergenic	0680	776E-04	136E-02	Csrp1	-81516	Hyper
chr1	153885645	153890906	Exon (NM_012910/25387, exon 3 of 16)	0337	777E-04	136E-02	Mir326	31616	Hyper
chr6	132465678	132466172	Distal Intergenic	0929	777E-04	136E-02	Adam6	-200802	Hyper
chr11	62719546	62720615	Distal Intergenic	0683	778E-04	137E-02	Gpr156	68073	Hyper
chr3	118566820	118568850	Distal Intergenic	0424	780E-04	137E-02	Rnf24	-40508	Hyper
chr5	56863184	56866176	Distal Intergenic	0386	780E-04	137E-02	Cntfr	-10703	Hyper
chr4	115683393	115685920	Promoter (<=1kb)	0352	782E-04	137E-02	Dctn1	0	Hyper
chr4	63149266	63151570	Intron (NM_199382/296973, intron 1 of 2)	0441	782E-04	137E-02	Bpgm	9157	Hyper
chr10	83337427	83339580	Distal Intergenic	0358	783E-04	137E-02	Ppp1r1b	-8151	Hyper
chr5	160414507	160418639	Distal Intergenic	0340	784E-04	137E-02	Spsb1	-43245	Hyper
chr8	65522130	65523923	Exon (NM_001106831/300783, exon 2 of 11)	0471	785E-04	137E-02	Hacd3	14584	Hyper
chr3	153723367	153726327	Exon (NM_134363/171373, exon 15 of 27)	0486	786E-04	137E-02	Slc12a5	19465	Hyper
chr5	158526816	158527366	Distal Intergenic	0909	786E-04	137E-02	Agtrap	-7780	Hyper

chr2	195987872	195991391	Exon (NM_031767/83576, exon 15 of 20)	0420	786E-04	137E-02	Mybphl	-13934	Hyper
chr2	30869236	30870985	Intron (NM_019217/29456, intron 2 of 6)	0558	786E-04	137E-02	Map1b	39332	Hyper
chr5	101403906	101404521	Intron (NM_001107943/313339, intron 1 of 5)	0953	788E-04	137E-02	Acer2	12021	Hyper
chr20	7666394	7668295	Exon (NM_001025059/502414, exon 11 of 13)	0562	788E-04	137E-02	Rnf8	-14094	Hyper
chr9	105303352	105305058	Intron (NM_145094/246324, intron 3 of 6)	0571	789E-04	138E-02	Txndc2	-68270	Hyper
chr10	86120378	86128529	3' UTR	0330	789E-04	138E-02	Cntnap1	8735	Hyper
chr19	52018447	52019170	Exon (NM_001135708/292087, exon 3 of 9)	0912	789E-04	138E-02	Urb2	5147	Hyper
chr18	32268098	32269442	Intron (NM_001172155/291618, intron 1 of 1)	0563	791E-04	138E-02	Kctd16	99764	Hyper
chr7	9087919	9089107	Intron (NM_001033870/65278, intron 1 of 11)	0678	791E-04	138E-02	Csnk1g2	5945	Hyper
chr2	112317358	112318084	Distal Intergenic	0701	791E-04	138E-02	Prkci	64221	Hyper
chr8	48986562	48987850	Distal Intergenic	0678	791E-04	138E-02	Nnmt	-39907	Hyper
chr5	142516779	142517436	Exon (NM_053582/94174, exon 4 of 12)	0975	792E-04	138E-02	Tinagl1	5654	Hyper
chr5	116048795	116050241	Intron (NM_017135/29223, intron 1 of 4)	0637	792E-04	138E-02	Ak4	9008	Hyper
chr13	45675199	45680323	Intron (NM_017155/29290, intron 1 of 1)	0344	793E-04	138E-02	Adora1	14479	Hyper
chr1	203581065	203583280	Promoter (1-2kb)	0525	793E-04	138E-02	Ehd1	1215	Hyper
chr6	119766769	119769377	Distal Intergenic	0474	795E-04	138E-02	Ttc7b	-61582	Hyper
chr4	104448518	104449087	Distal Intergenic	0879	796E-04	138E-02	Vamp8	-3391	Hyper
chr18	15688979	15690105	Distal Intergenic	0684	796E-04	138E-02	Mir187	41192	Hyper
chr5	141456044	141457193	3' UTR	0538	797E-04	138E-02	Hpca	6800	Hyper
chr3	142794608	142794915	Distal Intergenic	1058	799E-04	139E-02	Cdk5rap1	77754	Hyper
chr5	142919410	142920610	Exon (NM_001108684/362609, exon 11 of 22)	0624	801E-04	139E-02	Sdc3	-45049	Hyper
chr5	8332957	8333949	Distal Intergenic	0685	802E-04	139E-02	Prex2	-146157	Hyper
chr10	93886160	93886595	Distal Intergenic	0997	802E-04	139E-02	Axin2	-12650	Hyper
chr5	131628628	131629172	Intron (NM_031697/64445, intron 2 of 11)	0833	803E-04	139E-02	St3gal3	41123	Hyper
chr10	85858407	85860300	Promoter (2-3kb)	0488	805E-04	139E-02	Stat3	2757	Hyper
chr10	104797266	104800782	Distal Intergenic	0395	810E-04	140E-02	Nptx1	19576	Hyper
chr1	194290976	194294550	Intron (NM_001108923/365382, intron 4 of 15)	0472	810E-04	140E-02	Nkx6-2	88965	Hyper
chr17	10006289	10010267	Intron (NM_053835/116561, intron 2 of 5)	0434	811E-04	140E-02	Cltb	4686	Hyper
chr20	11145433	11149379	3' UTR	0376	811E-04	140E-02	Slx9	30801	Hyper
chr18	27635417	27636450	Intron (NM_001136151/432361, intron 4 of 9)	0672	813E-04	140E-02	Psd2	69830	Hyper
chr17	30656686	30661086	Intron (NM_022624/64559, intron 3 of 10)	0397	816E-04	141E-02	Psmg4	56938	Hyper
chr9	13234774	13238012	Distal Intergenic	0401	818E-04	141E-02	Pgc	27670	Hyper
chr16	18752967	18754769	Distal Intergenic	0531	819E-04	141E-02	Lsm4	6132	Hyper

chr19	10823525	10823944	Distal Intergenic	1109	821E-04	141E-02	Mt1	3104	Hyper
chr15	40395008	40398099	Exon (NM_017318/50646, exon 3 of 31)	0397	821E-04	141E-02	Chrna2	52691	Hyper
chr8	33861594	33863158	Distal Intergenic	0508	822E-04	142E-02	Ddx25	47109	Hyper
chr4	118017291	118019597	Exon (NM_001107871/312504, exon 4 of 15)	0449	825E-04	142E-02	Fbxo41	19809	Hyper
chr10	36205010	36206773	Distal Intergenic	0606	825E-04	142E-02	Cdkn2aipnl	-24361	Hyper
chr7	34897123	34901154	Promoter (1-2kb)	0398	829E-04	143E-02	Kitlg	1048	Hyper
chr3	16073120	16075385	Promoter (<=1kb)	0501	829E-04	143E-02	Cfap157	0	Hyper
chr1	196507577	196509789	Promoter (2-3kb)	0472	831E-04	143E-02	Gatd1	2711	Hyper
chr1	224225334	224226519	Exon (NM_001004446/361745, exon 5 of 35)	0667	832E-04	143E-02	Smarca2	34209	Hyper
chr8	100984935	100985941	Exon (NM_001173438/501043, exon 6 of 7)	0616	836E-04	143E-02	Il20rb	24001	Hyper
chr4	86038838	86040239	5' UTR	0549	838E-04	144E-02	Kbtbd2	9972	Hyper
chr10	36043159	36043898	Intron (NM_001009622/287276, intron 3 of 7)	0821	839E-04	144E-02	Sar1b	18716	Hyper
chr16	73163000	73163683	Intron (NM_001037327/364634, intron 5 of 69)	0774	839E-04	144E-02	Csmd1	944235	Hyper
chr8	89267056	89268262	Promoter (2-3kb)	0661	839E-04	144E-02	Nt5e	-2784	Hyper
chr1	46440091	46440640	Intron (NM_001127637/683687, intron 1 of 17)	0934	840E-04	144E-02	Snx9	15123	Hyper
chr19	48604240	48608946	Distal Intergenic	0372	841E-04	144E-02	Gins2	30393	Hyper
chr14	35053075	35060596	Distal Intergenic	0352	844E-04	144E-02	Zar1	136786	Hyper
chr10	45533495	45538353	3' UTR	0397	844E-04	144E-02	Tmem11	19217	Hyper
chr1	84108550	84111336	3' UTR	0476	845E-04	144E-02	Ech1	-3254	Hyper
chr15	20597550	20598119	Distal Intergenic	0952	846E-04	144E-02	Lgals3	-21964	Hyper
chr1	231058849	231060008	Intron (NM_001014167/361751, intron 6 of 7)	0718	848E-04	144E-02	Rps4x-ps1	-150269	Hyper
chr3	8515794	8516232	Distal Intergenic	0969	848E-04	144E-02	Bmyc	3225	Hyper
chr1	181932451	181933578	Exon (NM_001033998/308995, exon 12 of 31)	0693	855E-04	145E-02	Itgal	14268	Hyper
chr2	173461656	173463050	Exon (NM_001033665/25393, exon 6 of 14)	0593	855E-04	145E-02	Bcan	4410	Hyper
chr7	131379402	131388842	Exon (NM_001100895/315305, exon 3 of 7)	0344	858E-04	145E-02	Atf1	17440	Hyper
chr15	54649294	54650396	Distal Intergenic	0722	860E-04	146E-02	Rgcc	24924	Hyper
chr6	26387675	26389646	Intron (NM_053486/85248, intron 1 of 7)	0485	861E-04	146E-02	Kif3c	20583	Hyper
chr7	34824915	34825899	Distal Intergenic	0672	865E-04	146E-02	Kitlg	-70176	Hyper
chr19	622062	623707	3' UTR	0565	867E-04	147E-02	Cmtm3	5726	Hyper
chr3	133265523	133266114	Intron (NM_001107786/311494, intron 6 of 12)	0795	867E-04	147E-02	Naa20	-55950	Hyper
chr5	166319516	166323660	Intron (NM_001025657/298681, intron 1 of 4)	0426	870E-04	147E-02	Ssu72	5845	Hyper
chr10	57064481	57066948	3' UTR	0430	871E-04	147E-02	Mybbp1a	7263	Hyper
chr10	105972177	105974217	Distal Intergenic	0460	874E-04	148E-02	Lrrc45	-20302	Hyper

chr8	114536689	114537125	Promoter (1-2kb)	0983	877E-04	148E-02	Cmtm8	1277	Hyper
chr12	38234059	38235507	Distal Intergenic	0506	878E-04	148E-02	Spring1	93631	Hyper
chr19	34303393	34305234	Distal Intergenic	0530	878E-04	148E-02	Zfp90	-16813	Hyper
chr10	65701007	65704871	Intron (NM_012983/25485, intron 1 of 21)	0397	879E-04	148E-02	Myo1d	60941	Hyper
chr1	78854317	78855513	Exon (NM_138921/192360, exon 17 of 22)	0725	879E-04	148E-02	Gpr4	-19546	Hyper
chr5	165882373	165890192	Intron (NM_022507/25522, intron 4 of 17)	0333	879E-04	148E-02	Prkcz	40175	Hyper
chr4	152187196	152188786	Intron (NM_012517/24239, intron 6 of 49)	0588	880E-04	148E-02	Cacna1c	190668	Hyper
chr3	156104148	156104526	Distal Intergenic	1143	881E-04	148E-02	Slc9a8	-43579	Hyper
chr18	25967374	25973746	Promoter (2-3kb)	0320	882E-04	148E-02	Reep5	2763	Hyper
chr10	6630505	6631598	Distal Intergenic	0628	882E-04	148E-02	Hapstr1	143394	Hyper
chr13	84830913	84836021	Promoter (2-3kb)	0367	884E-04	149E-02	Pigm	-2308	Hyper
chr17	83464632	83466216	Intron (NM_001191693/307178, intron 1 of 24)	0525	886E-04	149E-02	Arhgap21	5986	Hyper
chr19	48047864	48048384	Distal Intergenic	0909	887E-04	149E-02	Crispld2	-21955	Hyper
chr6	3823219	3824881	Intron (NM_021767/60391, intron 16 of 21)	0504	890E-04	149E-02	Nrxn1	641929	Hyper
chr2	61838084	61840244	Distal Intergenic	0474	893E-04	150E-02	RGD1306502	24507	Hyper
chr10	102975912	102977224	Distal Intergenic	0612	894E-04	150E-02	Tmc8	-23120	Hyper
chr4	146669210	146671500	Exon (NM_001008355/312640, exon 2 of 8)	0453	895E-04	150E-02	Emc3	7476	Hyper
chr4	70511500	70512615	Exon (NM_053686/114246, exon 7 of 15)	0743	895E-04	150E-02	Trpv6	10395	Hyper
chr10	61487850	61489838	Distal Intergenic	0484	896E-04	150E-02	Trarg1	-31269	Hyper
chr10	14091316	14094118	Intron (NM_001009620/287129, intron 1 of 2)	0449	897E-04	150E-02	Tmem204	7802	Hyper
chr1	200954901	200956565	Distal Intergenic	0516	897E-04	150E-02	RGD1311946	9660	Hyper
chr14	6748012	6750519	Intron (NM_001270556/25272, intron 11 of 13)	0522	899E-04	150E-02	Arhgap24	98976	Hyper
chr3	76453923	76459906	Exon (NM_017269/29645, exon 2 of 24)	0375	900E-04	151E-02	Or4x13	89891	Hyper
chr16	556658	559803	Distal Intergenic	0456	901E-04	151E-02	Rps24	467021	Hyper
chr5	12326543	12327872	Promoter (2-3kb)	0560	903E-04	151E-02	Pcmtd1	-2503	Hyper
chr10	98755186	98756865	Distal Intergenic	0614	905E-04	151E-02	Fam104a	-26860	Hyper
chr17	30565468	30565938	Intron (NM_022624/64559, intron 1 of 10)	0824	906E-04	151E-02	Slc22a23	21362	Hyper
chr13	44818718	44819907	Intron (NM_001108343/360843, intron 2 of 2)	0713	906E-04	151E-02	Etnk2	7494	Hyper
chr2	142178447	142180009	Distal Intergenic	0570	908E-04	151E-02	Pfn2	-105639	Hyper
chr6	119774079	119775405	Distal Intergenic	0570	911E-04	152E-02	Ttc7b	-68892	Hyper
chr19	49970914	49972647	Distal Intergenic	0578	913E-04	152E-02	Slc7a5	-7091	Hyper
chr9	75872740	75874193	3' UTR	0711	913E-04	152E-02	Aamp	-3552	Hyper
chr1	121972230	121973217	Distal Intergenic	0866	916E-04	152E-02	LOC682259	260382	Hyper

chr3	13284993	13287613	Exon (NM_171983/64159, exon 32 of 56)	0543	918E-04	153E-02	Dync2i2	34508	Hyper
chr7	113904935	113906835	Promoter (<=1kb)	0627	921E-04	153E-02	Cyp2d5	-524	Hyper
chr1	196512662	196513547	Promoter (<=1kb)	0476	922E-04	153E-02	Gatd1	-162	Hyper
chr17	6857948	6859536	Intron (NM_001271297/306759, intron 2 of 10)	0570	926E-04	154E-02	Spock1	116435	Hyper
chr1	220581242	220582297	Distal Intergenic	0760	929E-04	154E-02	Klf9	-117811	Hyper
chr3	16363490	16365943	Distal Intergenic	0429	931E-04	154E-02	Slc2a8	-79026	Hyper
chr19	48008342	48012283	Exon (NM_001034146/307905, exon 11 of 14)	0535	932E-04	154E-02	Usp10	35168	Hyper
chr6	130912208	130915105	Intron (NM_001108062/314465, intron 3 of 15)	0461	933E-04	154E-02	Ppp1r13b	14544	Hyper
chr20	45732415	45736801	Intron (NM_001106395/294515, intron 2 of 3)	0383	933E-04	155E-02	Foxo3	27805	Hyper
chr5	164921386	164921828	Distal Intergenic	1005	935E-04	155E-02	Arhgef16	-55174	Hyper
chr18	61706146	61709496	Intron (NM_001271365/679578, intron 2 of 4)	0367	936E-04	155E-02	Ldlrad4	59435	Hyper
chr3	18982328	18983581	Intron (NM_138710/192126, intron 1 of 15)	0654	936E-04	155E-02	Dab2ip	67038	Hyper
chr5	60483887	60484904	Distal Intergenic	0624	936E-04	155E-02	Xpa	-8161	Hyper
chr1	220631309	220631815	Distal Intergenic	0847	936E-04	155E-02	Klf9	-68293	Hyper
chr5	150545787	150546661	Distal Intergenic	0752	937E-04	155E-02	Pink1	-3152	Hyper
chr1	197105055	197108849	Intron (NM_001109159/499288, intron 1 of 5)	0475	938E-04	155E-02	Mob2	41053	Hyper
chr6	105738430	105739708	Intron (NM_001134525/299209, intron 2 of 8)	0615	940E-04	155E-02	Ift43	8643	Hyper
chr1	259366380	259368068	Promoter (<=1kb)	0382	942E-04	155E-02	Fam204a	0	Hyper
chr9	57147273	57150368	Intron (NM_053456/84587, intron 1 of 5)	0347	942E-04	156E-02	Plcl1	245700	Hyper
chr17	53128245	53131643	Distal Intergenic	0343	943E-04	156E-02	Mtpap	209838	Hyper
chr7	133857460	133862336	Distal Intergenic	0400	944E-04	156E-02	Calcoco1	-8038	Hyper
chr10	45891753	45893652	Promoter (<=1kb)	0472	945E-04	156E-02	Aldh3a1	0	Hyper
chr6	119754076	119762357	Distal Intergenic	0293	945E-04	156E-02	Ttc7b	-48889	Hyper
chr17	71676860	71680674	Intron (NM_001083586/29428, intron 8 of 12)	0354	948E-04	156E-02	Celf2	267250	Hyper
chr8	63030952	63032453	Distal Intergenic	0516	948E-04	156E-02	Itga11	-113511	Hyper
chr1	77679159	77680738	Promoter (<=1kb)	0587	950E-04	156E-02	Ccdc8	0	Hyper
chr1	128250540	128252755	Intron (NM_177481/140915, intron 2 of 9)	0599	953E-04	157E-02	Slco3a1	135132	Hyper
chr10	60277765	60278240	Promoter (2-3kb)	1071	953E-04	157E-02	Serpinf2	2244	Hyper
chr1	197055384	197057041	Distal Intergenic	0560	955E-04	157E-02	Tollip	-72933	Hyper
chr3	53904944	53905628	Promoter (<=1kb)	0799	956E-04	157E-02	Nostrin	117	Hyper
chr1	84104281	84108215	Exon (NM_001134760/80846, exon 5 of 12)	0411	956E-04	157E-02	Hnrnpl	3396	Hyper
chr15	99952289	99954603	Intron (NM_019330/687008, intron 21 of 23)	0449	957E-04	157E-02	Ggact	38236	Hyper
chr1	134301963	134305244	Promoter (2-3kb)	0394	957E-04	157E-02	Hddc3	2491	Hyper

chr11	69671019	69672570	Distal Intergenic	0604	957E-04	157E-02	Acap2	132473	Hyper
chr5	124056220	124058529	Intron (NR_158458/313474, intron 1 of 24)	0383	959E-04	157E-02	Eps15	10294	Hyper
chr2	175294631	175303830	3' UTR	0352	960E-04	157E-02	Il6r	43694	Hyper
chr1	239142877	239146189	Distal Intergenic	0436	961E-04	158E-02	Pdlim1	-51909	Hyper
chr6	31192061	31194522	Distal Intergenic	0391	965E-04	158E-02	Gdf7	-9614	Hyper
chr10	30851418	30854312	Promoter (<=1kb)	0367	967E-04	158E-02	Med7	0	Hyper
chr9	76740276	76743469	Exon (NM_053881/116660, exon 20 of 23)	0409	967E-04	158E-02	Dnajb2	9211	Hyper
chr14	34987629	34988710	Distal Intergenic	0679	968E-04	158E-02	Ociad1	-72390	Hyper
chr1	172802618	172804076	Promoter (<=1kb)	0523	971E-04	158E-02	Itpripl2	0	Hyper
chr5	96927339	96928784	Intron (NM_031566/29227, intron 2 of 10)	0610	974E-04	159E-02	Nfib	44198	Hyper
chr8	87827059	87828498	Intron (NM_031728/65178, intron 2 of 30)	0643	976E-04	159E-02	Snap91	23869	Hyper
chr10	61205969	61206900	Intron (NM_001108285/360577, intron 1 of 7)	0636	977E-04	159E-02	Nxn	40637	Hyper
chr6	45247550	45248796	Distal Intergenic	0743	977E-04	159E-02	Rps7	22268	Hyper
chr5	135834004	135834889	Intron (NM_001135758/362587, intron 1 of 97)	0656	977E-04	159E-02	Macf1	111016	Hyper
chr10	82813335	82815321	Intron (NM_032613/29278, intron 3 of 7)	0450	977E-04	159E-02	Lasp1	18158	Hyper
chr9	84297288	84299548	Promoter (<=1kb)	0468	978E-04	159E-02	Slc19a3	0	Hyper
chr10	13473161	13476428	3' UTR	0509	978E-04	159E-02	E4f1	9546	Hyper
chr14	74863970	74868005	3' UTR	0401	979E-04	159E-02	Afap1	-52851	Hyper
chr2	175293314	175294306	Distal Intergenic	0746	980E-04	159E-02	Il6r	53218	Hyper
chr5	57659360	57660147	Distal Intergenic	0744	981E-04	159E-02	Tesk1	-31822	Hyper
chr12	41371267	41373291	Exon (NM_001011904/288710, exon 14 of 17)	0507	981E-04	159E-02	Pop5	8045	Hyper
chr12	42688824	42690236	Distal Intergenic	0617	985E-04	160E-02	Coro1c	-50630	Hyper
chr12	35384577	35385961	Intron (NM_001177593/25622, intron 2 of 15)	0591	985E-04	160E-02	Ptpn11	19141	Hyper
chr4	146677857	146679411	Promoter (<=1kb)	0388	985E-04	160E-02	Emc3	0	Hyper
chr15	3637098	3640310	Promoter (1-2kb)	0303	986E-04	160E-02	Synpo2l	1338	Hyper
chr5	161076844	161078734	Intron (NM_053885/116665, intron 12 of 22)	0574	989E-04	160E-02	Slc45a1	50569	Hyper
chr9	1524269	1527698	Distal Intergenic	0411	990E-04	160E-02	Vmac	21877	Hyper
chr10	68940802	68942603	Intron (NM_001270836/360580, intron 1 of 1)	0495	991E-04	160E-02		-9120	Hyper
chr14	43696154	43696509	Distal Intergenic	1236	992E-04	160E-02	Mir328b	33086	Hyper
chr10	39518707	39520446	Intron (NM_012656/24791, intron 8 of 9)	0564	994E-04	161E-02	Sparc	17703	Hyper
chr5	145294261	145296733	Downstream (<=300bp)	0466	996E-04	161E-02	Ahdc1	65336	Hyper
chr10	103954311	103958836	Distal Intergenic	0411	100E-03	162E-02	Rbfox3	-209780	Hyper
chr14	73879024	73886032	Intron (NM_057116/117256, intron 1 of 8)	0363	100E-03	162E-02	Ppp2r2c	45146	Hyper

chr10	45384977	45388913	Exon (NM_001304613/54265, exon 5 of 22)	0377	100E-03	162E-02	Llgl1	5398	Hyper
chr8	110677140	110679974	Intron (NM_001108783/363151, intron 2 of 6)	0472	101E-03	162E-02	Pth1r	37218	Hyper
chr16	63483463	63483755	Distal Intergenic	1015	101E-03	162E-02	Unc5d	623034	Hyper
chr1	154299112	154300887	Distal Intergenic	0489	101E-03	163E-02	Chrdl2	-42314	Hyper
chr13	21931152	21933621	Promoter (2-3kb)	0518	101E-03	163E-02	Tnfrsf11a	2744	Hyper
chr13	84737719	84740791	Intron (NM_012505/24212, intron 13 of 22)	0399	101E-03	163E-02	Atp1a2	13753	Hyper
chr1	86369033	86370046	Exon (NM_001014160/361550, exon 17 of 18)	0635	101E-03	163E-02	Scn1b	-5294	Hyper
chr9	61194478	61195606	Promoter (1-2kb)	0600	102E-03	163E-02	Bmpr2	1760	Hyper
chr1	197169478	197173451	3' UTR	0427	102E-03	163E-02	Dusp8	9470	Hyper
chr8	43047100	43048406	Intron (NM_012572/24406, intron 5 of 20)	0720	102E-03	163E-02	Grik4	156671	Hyper
chr9	75612544	75615410	Intron (NM_001191810/301509, intron 3 of 34)	0553	102E-03	163E-02	Tns1	87348	Hyper
chr9	44395625	44396487	Distal Intergenic	0472	102E-03	163E-02	Pantr1	547144	Hyper
chr16	76304234	76305463	Distal Intergenic	0646	102E-03	163E-02	Adprhl1	21035	Hyper
chr10	10920066	10921758	5' UTR	0585	102E-03	163E-02	Vasn	6501	Hyper
chr18	53951062	53952740	Intron (NM_173126/286910, intron 1 of 1)	0515	102E-03	164E-02	Smim3	4200	Hyper
chr1	48604645	48610887	Intron (NM_133406/170919, intron 1 of 8)	0323	102E-03	164E-02	Agpat4	22336	Hyper
chr18	27625838	27628843	Exon (NM_001136151/432361, exon 6 of 10)	0434	102E-03	164E-02	Psd2	60251	Hyper
chr1	185216318	185217323	Intron (NM_001004415/309025, intron 4 of 21)	0726	103E-03	164E-02	Tacc2	76933	Hyper
chr1	133942471	133943689	Distal Intergenic	0587	103E-03	164E-02	Arpin	-69347	Hyper
chr11	84838507	84840886	Intron (NM_001100988/498119, intron 9 of 18)	0440	103E-03	164E-02	Cebpd	73837	Hyper
chr5	130164139	130166013	Distal Intergenic	0539	103E-03	164E-02	Mmachc	6576	Hyper
chr13	93683289	93684056	Intron (NM_001012150/360891, intron 1 of 12)	0773	103E-03	165E-02	Enah	48279	Hyper
chr5	131700465	131700896	Exon (NM_001107966/313539, exon 9 of 21)	1010	103E-03	165E-02	Kdm4a	18605	Hyper
chr8	31893096	31894127	Distal Intergenic	0661	103E-03	165E-02	Ets1	787683	Hyper
chr2	183210116	183211561	Distal Intergenic	0544	103E-03	165E-02	Mcl1	-7659	Hyper
chr16	73514786	73516546	Intron (NM_001037327/364634, intron 15 of 69)	0582	104E-03	165E-02	Myom2	1073594	Hyper
chr12	41089947	41090988	Intron (NM_001012147/360820, intron 1 of 9)	0816	104E-03	165E-02	Pxn	16932	Hyper
chr4	119371321	119375994	Exon (NM_001173450/500244, exon 2 of 21)	0371	104E-03	166E-02	Rpl22	-20438	Hyper
chr8	20282464	20284110	Exon (NM_175762/300438, exon 6 of 18)	0496	104E-03	166E-02	Ldlr	12368	Hyper
chr13	68797732	68799335	Promoter (<=1kb)	0454	104E-03	166E-02	Tor3a	0	Hyper
chr9	90201995	90202832	Intron (NM_001108230/316611, intron 2 of 15)	0779	104E-03	166E-02	Agap1	13970	Hyper
chr3	141482314	141484364	Promoter (<=1kb)	0398	104E-03	166E-02	Pdrg1	0	Hyper
chr3	118345530	118346248	Promoter (<=1kb)	0848	105E-03	167E-02	Hspa12b	-106	Hyper

chr9	14522480	14524904	Intron (NM_001271230/316229, intron 12 of 13)	0384	105E-03	167E-02	Slc22a7	-23005	Hyper
chr8	37104111	37106070	Distal Intergenic	0439	105E-03	167E-02	Robo4	-13918	Hyper
chr2	174837857	174842771	Promoter (<=1kb)	0316	105E-03	167E-02	Shc1	0	Hyper
chr5	74731590	74734273	Intron (NM_001106651/298033, intron 1 of 8)	0462	105E-03	167E-02	Snx30	47570	Hyper
chr5	122703451	122704301	Distal Intergenic	0758	105E-03	167E-02	Cpt2	-21405	Hyper
chr14	79580301	79583483	Distal Intergenic	0405	106E-03	167E-02	Uqcr10	-11893	Hyper
chr1	49045937	49046823	Intron (NM_020093/56816, intron 7 of 11)	0708	106E-03	167E-02	Agpat4	-412714	Hyper
chr9	76264285	76265474	Promoter (<=1kb)	0616	106E-03	168E-02	Cyp27a1	0	Hyper
chr5	116254923	116256528	Intron (NM_001107949/313409, intron 11 of 17)	0465	106E-03	168E-02	Leprot	-33315	Hyper
chr11	79240888	79242997	Promoter (<=1kb)	0500	106E-03	168E-02	Ehhadh	0	Hyper
chr12	20761097	20764044	Intron (NM_019376/56010, intron 1 of 2)	0491	106E-03	168E-02	Ywhag	16517	Hyper
chr11	65580742	65582079	Intron (NM_022600/64532, intron 1 of 20)	0566	106E-03	168E-02	Adcy5	36022	Hyper
chr10	105548659	105551861	Distal Intergenic	0467	106E-03	168E-02	Actg1	70726	Hyper
chr2	136449841	136451007	Distal Intergenic	0568	107E-03	168E-02	Foxo1	137673	Hyper
chr2	215526317	215526796	Distal Intergenic	0874	107E-03	168E-02	Larp7	486013	Hyper
chr1	165387501	165387875	Distal Intergenic	0925	107E-03	168E-02	Eif4g2	-193918	Hyper
chr3	141945469	141948644	Distal Intergenic	0470	107E-03	168E-02	Commd7	165686	Hyper
chr3	106966710	106968926	Intron (NM_001108589/362197, intron 1 of 30)	0457	107E-03	168E-02	Mapkbp1	18883	Hyper
chr1	99522991	99523670	Intron (NM_053557/89820, intron 10 of 15)	0750	107E-03	169E-02	Prmt3	30223	Hyper
chr3	88304319	88309955	Distal Intergenic	0340	108E-03	169E-02	Commd9	128326	Hyper
chr10	106523535	106524664	Intron (NM_001039207/360681, intron 2 of 10)	0688	108E-03	169E-02	Narf	3981	Hyper
chr1	133685580	133686203	Promoter (<=1kb)	0831	108E-03	170E-02	Pex11a	969	Hyper
chr3	133264777	133265352	Exon (NM_001107786/311494, exon 6 of 13)	0700	108E-03	170E-02	Naa20	-56712	Hyper
chr1	82854558	82856081	Intron (NM_001109664/292739, intron 6 of 9)	0556	108E-03	170E-02	RGD1307554	10228	Hyper
chr12	38517120	38519020	3' UTR	0534	108E-03	170E-02	Fbxo21	86113	Hyper
chr1	43567037	43570294	Intron (NM_001012061/308113, intron 1 of 12)	0495	108E-03	170E-02	Cnksr3	21336	Hyper
chr15	88852939	88854312	Promoter (1-2kb)	0485	108E-03	170E-02	Slitrk5	1535	Hyper
chr15	97803231	97806152	Distal Intergenic	0461	108E-03	170E-02	Rap2a	206383	Hyper
chr12	26535956	26537565	Distal Intergenic	0558	109E-03	170E-02	Kctd7	-3818	Hyper
chr5	55516251	55517359	Distal Intergenic	0768	109E-03	171E-02	Tmem215	-95209	Hyper
chr7	115364210	115366197	Intron (NM_001134780/362973, intron 1 of 12)	0568	109E-03	171E-02	Parvb	3956	Hyper
chr4	49851637	49853318	Intron (NM_031730/65180, intron 1 of 5)	0535	109E-03	171E-02	Kcnd2	74935	Hyper
chr2	137215693	137217300	Intron (NM_001109183/499615, intron 2 of 3)	0577	110E-03	171E-02	Lhfpl6	89208	Hyper

chr1	175479427	175485614	Exon (NM_001108503/361640, exon 7 of 21)	0323	110E-03	171E-02	Polr3e	13304	Hyper
chr1	197120027	197122787	Intron (NM_001109159/499288, intron 1 of 5)	0396	110E-03	171E-02	Mob2	27115	Hyper
chr3	9153092	9157348	Exon (NM_144745/246254, exon 9 of 14)	0365	110E-03	171E-02	Gpsm1	-4703	Hyper
chr10	14677866	14680473	Distal Intergenic	0504	110E-03	171E-02	Gng13	-60706	Hyper
chr2	96049403	96052621	Promoter (<=1kb)	0323	110E-03	171E-02	Pex2	0	Hyper
chr1	96153024	96154199	Promoter (<=1kb)	0638	110E-03	171E-02	Sec1	-689	Hyper
chr9	75919865	75921272	Promoter (2-3kb)	0588	110E-03	172E-02	Pnkd	2030	Hyper
chr18	56385384	56387559	Distal Intergenic	0534	110E-03	172E-02	Napg	-52848	Hyper
chr7	52858566	52859781	3' UTR	0640	110E-03	172E-02	Yeats4	5673	Hyper
chr19	33292109	33292952	Promoter (<=1kb)	0678	111E-03	172E-02	Lrrc36	35	Hyper
chr5	113362500	113363981	3' UTR	0463	111E-03	173E-02	Kank4	101253	Hyper
chr1	184774415	184776291	Intron (NM_001109892/25022, intron 9 of 17)	0608	111E-03	173E-02	Fgfr2	74335	Hyper
chr12	35171848	35174601	Exon (NM_053760/114635, exon 3 of 12)	0446	111E-03	173E-02	Trafd1	6242	Hyper
chr20	1519954	1523316	3' UTR	0504	111E-03	173E-02	Zfp57	6361	Hyper
chr14	73597654	73600041	Distal Intergenic	0518	112E-03	173E-02	Crmp1	-41477	Hyper
chr4	147132141	147133025	Intron (NM_012508/24215, intron 2 of 21)	0706	112E-03	173E-02	Atp2b2	74953	Hyper
chr7	131310446	131312447	Distal Intergenic	0575	112E-03	174E-02	Atf1	-49515	Hyper
chr3	76606610	76607762	Distal Intergenic	0568	112E-03	174E-02	Ptprj	-44941	Hyper
chr17	57389388	57390084	Intron (NM_001191833/361261, intron 8 of 9)	0789	112E-03	174E-02	Gjd4	-36942	Hyper
chr19	39532151	39533589	Distal Intergenic	0513	113E-03	174E-02	Ldhd	61986	Hyper
chr8	20388199	20388531	Exon (NM_001108997/367039, exon 3 of 48)	1137	113E-03	174E-02	Dock6	6021	Hyper
chr6	119908378	119912763	Intron (NM_001108048/314384, intron 3 of 15)	0348	113E-03	174E-02	Rps6ka5	93381	Hyper
chr19	52451764	52455178	Distal Intergenic	0403	113E-03	174E-02	Cog2	-38754	Hyper
chr5	131672480	131675244	Promoter (2-3kb)	0437	113E-03	174E-02	St3gal3	-2185	Hyper
chr11	68141032	68143312	Intron (NM_001008336/303882, intron 1 of 14)	0468	113E-03	174E-02	Tnk2	10697	Hyper
chr1	246696422	246697003	Distal Intergenic	0904	113E-03	174E-02	Gsto1	-24240	Hyper
chr5	101411736	101412860	Intron (NM_001107943/313339, intron 3 of 5)	0719	114E-03	175E-02	Acer2	19851	Hyper
chr5	160399511	160406302	Distal Intergenic	0379	114E-03	175E-02	Spsb1	-28249	Hyper
chr19	30519638	30520824	Intron (NM_001109501/688429, intron 3 of 22)	0601	114E-03	175E-02	Arhgap10	71004	Hyper
chr10	85861694	85863158	Promoter (<=1kb)	0367	114E-03	176E-02	Stat3	0	Hyper
chr1	202363080	202365461	Distal Intergenic	0454	115E-03	176E-02	Rin1	7351	Hyper
chr16	3048009	3048914	Intron (NM_170787/259269, intron 3 of 16)	0653	115E-03	176E-02	Erc2	199331	Hyper
chr1	29582706	29583505	Promoter (2-3kb)	0865	115E-03	176E-02	Slc6a19	-2700	Hyper

chr12	35305980	35306708	Distal Intergenic	0672	115E-03	176E-02	Rpl6	44333	Hyper
chr2	210083416	210084162	Intron (NM_001009405/310833, intron 1 of 23)	0688	115E-03	177E-02	Arhgap29	12217	Hyper
chr3	77641097	77647263	3' UTR	0337	115E-03	177E-02	Arhgap1	19408	Hyper
chr10	82354799	82356568	Distal Intergenic	0551	115E-03	177E-02	Mrpl45	46149	Hyper
chr9	87105416	87106545	Distal Intergenic	0648	116E-03	177E-02	Tex44	29732	Hyper
chr6	7821606	7825095	Intron (NM_023090/29452, intron 2 of 16)	0417	116E-03	177E-02	Epas1	46151	Hyper
chr3	117948347	117949008	Intron (NM_001109206/499891, intron 27 of 36)	0790	116E-03	177E-02	Slc4a11	-35673	Hyper
chr4	119364312	119371225	Intron (NM_001173450/500244, intron 1 of 20)	0313	116E-03	178E-02	Rpl22	-13429	Hyper
chr6	57063019	57065863	Distal Intergenic	0377	116E-03	178E-02	Arl4a	18293	Hyper
chr4	30666395	30666867	Intron (NM_001191861/114483, intron 4 of 6)	0947	116E-03	178E-02	Fam133b	-50372	Hyper
chr10	46166265	46168632	Promoter (<=1kb)	0503	116E-03	178E-02	Mfap4	0	Hyper
chr15	34096586	34098098	Distal Intergenic	0631	117E-03	179E-02	Shisa2	289871	Hyper
chr8	45456990	45457594	Exon (NM_001008880/315611, exon 4 of 5)	0775	117E-03	179E-02	Scn4b	9871	Hyper
chr11	59934270	59935577	Distal Intergenic	0531	118E-03	179E-02		-372249	Hyper
chr1	109493081	109493917	Distal Intergenic	0715	118E-03	180E-02	Atp10a	-62865	Hyper
chr5	139110733	139112948	Exon (NM_001159655/100294508, exon 6 of 21)	0409	118E-03	180E-02	LOC100294508	62695	Hyper
chr20	3743238	3743635	Distal Intergenic	0909	118E-03	180E-02	Ly6g6e	3101	Hyper
chr6	99079228	99079973	Intron (NM_031005/81634, intron 1 of 20)	0792	118E-03	180E-02	Actn1	13214	Hyper
chr13	74829873	74830479	Intron (NM_138538/171574, intron 1 of 20)	0805	119E-03	181E-02	Dnm3	7629	Hyper
chr20	7527136	7528530	Distal Intergenic	0507	119E-03	181E-02	Pim1	-26391	Hyper
chr20	7407347	7410446	Promoter (2-3kb)	0408	119E-03	181E-02	Mtch1	2980	Hyper
chr6	112468561	112469362	Distal Intergenic	0644	119E-03	181E-02		-952085	Hyper
chr20	28235556	28239054	Exon (NM_001190236/25524, exon 9 of 15)	0425	119E-03	181E-02	Psap	21285	Hyper
chr19	39754665	39755489	Intron (NM_199378/292027, intron 5 of 6)	0735	119E-03	181E-02	Bcar1	-52382	Hyper
chr11	40827285	40828089	Distal Intergenic	0684	119E-03	181E-02	Riox2	56456	Hyper
chr8	82531832	82534848	Promoter (<=1kb)	0460	119E-03	181E-02	Htr1b	0	Hyper
chr10	14689422	14692707	Distal Intergenic	0388	119E-03	181E-02	Gng13	-48472	Hyper
chr13	92128333	92130784	Intron (NM_019312/54260, intron 2 of 7)	0436	120E-03	181E-02	Itpkb	59117	Hyper
chr9	86310315	86311844	Promoter (<=1kb)	0500	120E-03	181E-02	Sp100	0	Hyper
chr16	63327123	63328348	Intron (NM_001107319/306534, intron 8 of 17)	0773	120E-03	182E-02	Unc5d	466694	Hyper
chr12	42122402	42123950	Distal Intergenic	0656	120E-03	182E-02	Mvk	34908	Hyper
chrX	150705284	150705649	Exon (NM_031733/65187, exon 5 of 9)	1021	120E-03	182E-02	Gabrq	8842	Hyper
chr8	81510048	81510468	Distal Intergenic	0974	120E-03	182E-02	Impg1	-120353	Hyper

chr10	98712860	98715013	Intron (NM_001034958/619573, intron 2 of 2)	0556	120E-03	182E-02	MGC95210	-12750	Hyper
chr19	54289104	54290784	Distal Intergenic	0549	120E-03	182E-02	Kcnk1	329447	Hyper
chr9	16761231	16762630	Intron (NM_053603/94272, intron 2 of 6)	0526	121E-03	183E-02	Clic5	50797	Hyper
chr6	40776513	40777828	Distal Intergenic	0553	121E-03	183E-02	Itgb1bp1	57919	Hyper
chr8	73719031	73721137	Promoter (<=1kb)	0362	121E-03	183E-02	Pierce2	0	Hyper
chr3	119957699	119961110	Distal Intergenic	0390	121E-03	183E-02	Chgb	-82714	Hyper
chr1	150990483	150991077	Intron (NM_001191628/308831, intron 4 of 31)	0824	121E-03	183E-02	Tenm4	210102	Hyper
chr5	19539373	19539873	Exon (NM_181389/353233, exon 17 of 32)	0873	122E-03	184E-02	Nsmaf	37754	Hyper
chr14	52780243	52781565	Intron (NM_001004087/360942, intron 1 of 2)	0553	122E-03	184E-02	Pcdh7	117908	Hyper
chr3	16548097	16556715	Distal Intergenic	0312	122E-03	184E-02	Angptl2	30912	Hyper
chr10	63416653	63418467	3' UTR	0559	122E-03	184E-02	Poldip2	7887	Hyper
chr7	114719772	114721020	Promoter (<=1kb)	0578	122E-03	184E-02	Tspo	0	Hyper
chr10	105311024	105315707	Intron (NM_001168703/690853, intron 1 of 14)	0334	122E-03	184E-02	Aatk	11405	Hyper
chr13	77299897	77300945	Distal Intergenic	0659	122E-03	184E-02	Xcl1	-47589	Hyper
chr2	241399374	241401655	Intron (NM_001108951/365985, intron 13 of 13)	0553	122E-03	184E-02	Zzz3	40425	Hyper
chr10	27049577	27052063	Exon (NM_012957/25451, exon 5 of 9)	0489	122E-03	184E-02	Gabrb2	111337	Hyper
chr10	102247150	102250744	Distal Intergenic	0347	122E-03	184E-02	Sec14l1	-69176	Hyper
chr14	41730379	41731413	Distal Intergenic	0592	123E-03	185E-02	Nsun7	203536	Hyper
chr7	86434826	86436177	Exon (NM_133319/170844, exon 23 of 26)	0575	123E-03	185E-02	Taf2	43439	Hyper
chr2	184234872	184236168	Promoter (1-2kb)	0584	123E-03	185E-02	Pias3	1583	Hyper
chr6	77276246	77278407	Distal Intergenic	0422	123E-03	185E-02	Fbxo33	-343909	Hyper
chr10	101315607	101320814	Promoter (<=1kb)	0291	123E-03	185E-02	Wbp2	0	Hyper
chr2	219400175	219401642	Distal Intergenic	0795	124E-03	186E-02	Rpl34	-112067	Hyper
chr10	59692163	59692680	Distal Intergenic	1012	124E-03	186E-02	Mnt	-6905	Hyper
chr5	101670053	101677213	Intron (NM_031743/84550, intron 1 of 9)	0293	124E-03	186E-02	Slc24a2	62124	Hyper
chr4	146425004	146428255	Promoter (2-3kb)	0508	124E-03	186E-02	Cpne9	-2585	Hyper
chr12	41357792	41359175	Intron (NM_001011904/288710, intron 2 of 16)	0589	124E-03	186E-02	Rnf10	16075	Hyper
chr11	66416485	66418245	Intron (NM_032062/84009, intron 5 of 59)	0633	125E-03	187E-02	Kalrn	161516	Hyper
chr11	80555876	80556443	Intron (NM_053924/116721, intron 25 of 29)	0905	125E-03	187E-02	Parl	-37634	Hyper
chr8	110631308	110631872	Distal Intergenic	0912	125E-03	187E-02	Ccdc12	-3838	Hyper
chr5	162759945	162761561	Exon (NM_013214/26759, exon 7 of 9)	0686	125E-03	187E-02	Gpr153	-20026	Hyper
chr8	75714945	75719408	Intron (NM_001106838/300836, intron 2 of 12)	0318	125E-03	187E-02	Atosa	19923	Hyper
chr10	64003013	64005618	Intron (NM_001382488/108348076, intron 2 of 17)	0526	125E-03	187E-02	Lgals9	-72968	Hyper

chr13	44376305	44378305	Intron (NM_001177368/289020, intron 1 of 1)	0478	125E-03	187E-02	Lrrn2	54361	Hyper
chr20	4132552	4134294	Promoter (<=1kb)	0503	125E-03	187E-02	Egfl8	0	Hyper
chr14	73749508	73750448	Distal Intergenic	0672	125E-03	187E-02	Jakmip1	-35515	Hyper
chr2	137149363	137150181	Intron (NM_001109183/499615, intron 2 of 3)	0808	126E-03	187E-02	Lhfpl6	22878	Hyper
chr2	135596637	135600326	Intron (NM_001109558/689954, intron 1 of 7)	0312	126E-03	187E-02	Setd7	5142	Hyper
chr6	49993664	49995036	Distal Intergenic	0485	126E-03	187E-02	Efcab10	-9443	Hyper
chr9	1178404	1181120	Intron (NM_001044236/301128, intron 1 of 22)	0438	126E-03	187E-02	Kdm4b	19625	Hyper
chr2	210253948	210254220	Intron (NM_001107721/310836, intron 28 of 53)	1242	126E-03	187E-02	Abca4	89135	Hyper
chr1	202230254	202231631	Promoter (2-3kb)	0596	126E-03	187E-02	Dpp3	-2888	Hyper
chr17	21603736	21604545	Intron (NM_214457/306844, intron 9 of 13)	0666	126E-03	188E-02	Tbc1d7	72742	Hyper
chr15	13062764	13063721	Intron (NM_134356/171357, intron 5 of 32)	0687	127E-03	188E-02	Cep15	-227716	Hyper
chr7	63108214	63110171	3' UTR	0629	127E-03	188E-02	Mbd6	3103	Hyper
chr4	52942343	52942687	Intron (NM_001034130/296936, intron 9 of 12)	0945	127E-03	188E-02	Iqub	51108	Hyper
chr1	65559912	65561974	Promoter (<=1kb)	0482	127E-03	188E-02	Mir3572	0	Hyper
chr19	12235950	12237352	Distal Intergenic	0594	127E-03	189E-02	Large1	-187020	Hyper
chr4	30734487	30737046	Intron (NM_001191861/114483, intron 3 of 6)	0470	127E-03	189E-02	Cdk6	89734	Hyper
chr7	66322563	66323260	Promoter (<=1kb)	0597	127E-03	189E-02	Stk3	0	Hyper
chr5	161078913	161081067	Intron (NM_053885/116665, intron 12 of 22)	0511	127E-03	189E-02	Slc45a1	48236	Hyper
chr20	4141210	4141953	Promoter (1-2kb)	0826	128E-03	190E-02	Agpat1	1307	Hyper
chr1	196149372	196150856	Distal Intergenic	0593	128E-03	190E-02	B4galnt4	-20538	Hyper
chr1	171665205	171667304	Intron (NM_022295/64133, intron 1 of 11)	0373	128E-03	190E-02	Xylt1	21280	Hyper
chr10	101015723	101018581	Distal Intergenic	0394	128E-03	190E-02	Mir3577	-5288	Hyper
chr1	160087656	160090097	Promoter (<=1kb)	0386	128E-03	190E-02	Rrp8	0	Hyper
chr13	66146076	66147471	Distal Intergenic	0619	128E-03	190E-02	Glul	120435	Hyper
chr1	96485212	96485870	Exon (NM_031013/81642, exon 9 of 31)	0798	129E-03	190E-02	Abcc6	15594	Hyper
chr18	32590978	32591527	Distal Intergenic	1006	129E-03	190E-02	Kctd16	422644	Hyper
chr1	95583238	95584851	Promoter (<=1kb)	0443	129E-03	190E-02	Fcgrt	0	Hyper
chr7	72889741	72892409	Promoter (<=1kb)	0362	129E-03	191E-02	Oxr1	0	Hyper
chr5	150287351	150288970	Intron (NM_001106693/298573, intron 1 of 30)	0573	129E-03	191E-02	Eif4g3	36209	Hyper
chr11	66884188	66887546	Intron (NM_147139/257645, intron 5 of 14)	0396	129E-03	191E-02	Itgb5	56685	Hyper
chr3	22758673	22765684	Promoter (<=1kb)	0290	130E-03	191E-02	Arpc5l	0	Hyper
chr19	23522807	23527894	Exon (NM_012918/25398, exon 44 of 47)	0333	130E-03	191E-02	Ier2	28256	Hyper
chr2	11347477	11349068	Distal Intergenic	0616	130E-03	192E-02	Arrdc3	209777	Hyper

chr7	108265849	108266701	3' UTR	0769	130E-03	192E-02	Slc52a2	3264	Hyper
chr13	44156580	44162188	Intron (NM_001160314/116690, intron 1 of 29)	0371	130E-03	192E-02	Nfasc	-7544	Hyper
chr15	76504156	76505426	Distal Intergenic	0656	131E-03	192E-02	Klf5	439898	Hyper
chr9	105289139	105289840	Intron (NM_145094/246324, intron 5 of 6)	0811	131E-03	193E-02	Txndc2	-54057	Hyper
chr8	97350348	97351905	Distal Intergenic	0416	132E-03	193E-02	Zbtb38	-61830	Hyper
chr2	12745874	12746392	Distal Intergenic	0783	132E-03	194E-02	Cetn3	656457	Hyper
chr5	101225676	101228437	Distal Intergenic	0385	132E-03	194E-02	Plin2	-42640	Hyper
chr3	133826048	133828465	Distal Intergenic	0460	132E-03	194E-02	Crnkl1	-471719	Hyper
chr4	141230136	141233916	Intron (NM_001007235/25262, intron 4 of 61)	0397	133E-03	194E-02	Itpr1	42718	Hyper
chr5	165821399	165825329	Intron (NM_022507/25522, intron 16 of 17)	0422	133E-03	195E-02	Faap20	12742	Hyper
chr1	7134707	7136169	Intron (NM_013070/25600, intron 2 of 74)	0579	133E-03	195E-02	Utrn	88144	Hyper
chr10	57101118	57108192	Promoter (<=1kb)	0330	133E-03	195E-02	Spns2	0	Hyper
chr6	131940971	131943598	Distal Intergenic	0445	133E-03	195E-02	Gpr132	-5026	Hyper
chr8	109992183	109993279	Intron (NM_001024278/367171, intron 3 of 16)	0649	133E-03	195E-02	Map4	66563	Hyper
chr20	12304265	12308286	Exon (NM_001191564/690211, exon 3 of 38)	0387	134E-03	195E-02	Dip2a	19611	Hyper
chr15	40195411	40196269	Exon (NM_053021/24854, exon 7 of 10)	0684	134E-03	195E-02	Scara3	-22545	Hyper
chr4	60107706	60109295	Exon (NM_031359/83536, exon 16 of 18)	0502	134E-03	196E-02	Podxl	72534	Hyper
chr13	92505642	92507443	Distal Intergenic	0441	134E-03	196E-02	Acbd3	49829	Hyper
chr11	45759045	45759649	Distal Intergenic	0746	134E-03	196E-02	Nfkbiz	976369	Hyper
chr5	14954570	14956286	Distal Intergenic	0529	135E-03	196E-02	Sox17	-60445	Hyper
chr14	83004669	83005065	Distal Intergenic	1085	135E-03	197E-02	Tns3	339073	Hyper
chr1	201279427	201280352	Promoter (<=1kb)	0709	135E-03	197E-02	Acy3	0	Hyper
chr7	63005193	63007134	Promoter (1-2kb)	0560	135E-03	197E-02	Slc26a10	-1106	Hyper
chr15	41040075	41045605	Intron (NM_001105717/25416, intron 3 of 13)	0327	136E-03	198E-02	Dpysl2	27252	Hyper
chr3	147294023	147302263	3' UTR	0327	136E-03	198E-02	Actr5	11399	Hyper
chr7	113309780	113310997	3' UTR	0595	136E-03	198E-02	Tef	-15714	Hyper
chr5	141880384	141881147	Promoter (<=1kb)	0539	136E-03	198E-02	Hdac1	0	Hyper
chr1	170682283	170684778	Promoter (<=1kb)	0328	137E-03	198E-02	Pik3c2a	0	Hyper
chr9	42230454	42233160	Intron (NM_001106904/301363, intron 1 of 28)	0453	137E-03	198E-02	Map4k4	28834	Hyper
chr1	136728222	136728650	Intron (NM_001107534/308789, intron 15 of 19)	0885	137E-03	198E-02	Efl1	89695	Hyper
chr10	69206541	69211743	Intron (NM_022193/60581, intron 39 of 53)	0406	137E-03	199E-02	Acaca	123719	Hyper
chr20	7369968	7371048	Distal Intergenic	0593	137E-03	199E-02	Pi16	-5399	Hyper
chr1	44798514	44799170	Distal Intergenic	0710	137E-03	199E-02	Nox3	-495663	Hyper

chr4	2813563	2814394	Distal Intergenic	0674	137E-03	199E-02	Il6	2404784	Hyper
chr10	103195985	103197905	Promoter (<=1kb)	0480	137E-03	199E-02	Socs3	0	Hyper
chr1	95498882	95501983	Promoter (<=1kb)	0356	138E-03	199E-02	Rras	0	Hyper
chr5	162221104	162223293	Intron (NM_001195559/362665, intron 3 of 23)	0481	138E-03	199E-02	Camta1	133406	Hyper
chr3	8099112	8106922	Promoter (<=1kb)	0338	138E-03	199E-02	Lrrc26	0	Hyper
chr20	8651766	8653098	Distal Intergenic	0602	138E-03	200E-02	Btbd9	-11922	Hyper
chr12	44366928	44369237	Distal Intergenic	0369	138E-03	200E-02	Tpst2	-4599	Hyper
chr7	8741220	8742771	Intron (NM_024138/58979, intron 1 of 2)	0561	139E-03	201E-02	Gng7	26527	Hyper
chr19	45260714	45261775	Distal Intergenic	0636	139E-03	201E-02	Cmip	-42822	Hyper
chr5	153605659	153606643	Promoter (<=1kb)	0704	139E-03	201E-02	Epha2	15	Hyper
chr12	44340823	44341906	Intron (NM_001008508/288719, intron 1 of 6)	0757	139E-03	201E-02	Tpst2	20423	Hyper
chr6	117924372	117924872	Exon (NM_138862/192225, exon 11 of 12)	0898	139E-03	201E-02	Spata7	44477	Hyper
chr3	90636552	90639100	Distal Intergenic	0371	140E-03	201E-02	C3h11orf91	151945	Hyper
chr4	124227965	124235387	5' UTR	0302	140E-03	201E-02	Slc6a6	32618	Hyper
chr4	149541176	149541780	Intron (NM_012822/25290, intron 8 of 15)	0804	140E-03	201E-02	Alox5	36916	Hyper
chr7	22804267	22814191	Distal Intergenic	0373	140E-03	201E-02	Dram1	-27462	Hyper
chr5	156855610	156856009	Intron (NM_001108006/313825, intron 66 of 69)	1001	140E-03	202E-02	Dhrs3	106991	Hyper
chr17	15227881	15230341	Promoter (<=1kb)	0341	141E-03	202E-02	Ippk	0	Hyper
chr13	91596967	91598373	Distal Intergenic	0618	141E-03	202E-02	Ahctf1	-60576	Hyper
chr13	48139937	48140864	Distal Intergenic	0604	141E-03	203E-02	Zfp281	43762	Hyper
chr8	61208910	61210289	Distal Intergenic	0583	142E-03	203E-02	Larp6	24794	Hyper
chr17	6490108	6491212	Promoter (<=1kb)	0479	142E-03	203E-02	Idnk	0	Hyper
chr1	240518599	240519215	Intron (NM_022953/65047, intron 4 of 36)	0747	142E-03	203E-02	Slit1	38912	Hyper
chr17	52619157	52625809	Distal Intergenic	0337	142E-03	203E-02	Svil	141451	Hyper
chr1	213431912	213432569	Intron (NM_031036/81666, intron 1 of 6)	0793	142E-03	203E-02	Gnaq	6186	Hyper
chr15	99000887	99001733	Promoter (<=1kb)	0692	142E-03	204E-02	Gpr18	0	Hyper
chr12	45886017	45888753	Exon (NM_001025563/304567, exon 5 of 6)	0444	142E-03	204E-02	Ep400	-3163	Hyper
chr3	53676132	53678083	Distal Intergenic	0494	143E-03	204E-02	Nostrin	-226744	Hyper
chr17	4279037	4280190	Distal Intergenic	0497	143E-03	204E-02	Dapk1	-188046	Hyper
chr3	16583758	16584431	Distal Intergenic	0812	143E-03	204E-02	Angptl2	66573	Hyper
chr12	44333395	44340683	5' UTR	0316	143E-03	205E-02	Tfip11	-21544	Hyper
chr4	45639947	45642730	Promoter (<=1kb)	0395	143E-03	205E-02	Cav1	0	Hyper
chr5	71550148	71550465	Intron (NM_001106649/298019, intron 2 of 18)	0999	144E-03	205E-02	Ctnnal1	18966	Hyper

chr9	79804884	79805462	Intron (NM_001191811/301543, intron 1 of 4)	0746	144E-03	205E-02	Sgpp2	41490	Hyper
chr1	236262358	236263739	Intron (NM_053758/114633, intron 1 of 32)	0602	144E-03	205E-02	Plce1	17675	Hyper
chr4	57815900	57817986	Promoter (<=1kb)	0525	144E-03	205E-02	Impdh1	0	Hyper
chr6	21506740	21507207	Distal Intergenic	0830	144E-03	206E-02	Xdh	-23256	Hyper
chr1	46525064	46527374	Intron (NM_032071/84018, intron 1 of 27)	0501	144E-03	206E-02	Synj2	6306	Hyper
chr8	44648287	44649737	3' UTR	0589	144E-03	206E-02	C2cd2l	8603	Hyper
chr1	196155745	196157987	Distal Intergenic	0348	144E-03	206E-02	B4galnt4	-13407	Hyper
chr7	33775373	33779423	Intron (NM_053311/29598, intron 1 of 21)	0393	144E-03	206E-02	Atp2b1	39398	Hyper
chr10	89193136	89194493	Intron (NM_017212/29477, intron 2 of 9)	0515	145E-03	206E-02	Mapt	54488	Hyper
chr19	50330661	50331891	Promoter (2-3kb)	0592	145E-03	206E-02	Zfpm1	-2791	Hyper
chr16	66883973	66886496	Exon (NM_001004107/306562, exon 9 of 13)	0443	145E-03	206E-02	Tacc1	38932	Hyper
chr10	90676314	90677319	Intron (NM_001191653/303599, intron 4 of 25)	0693	145E-03	206E-02	Tanc2	123082	Hyper
chr14	72676345	72676897	Intron (NM_001012151/360953, intron 7 of 10)	0897	145E-03	206E-02	Nsg1	27574	Hyper
chr1	154997450	154999412	3' UTR	0545	145E-03	206E-02	Plekhb1	13657	Hyper
chr10	98209492	98220311	Promoter (<=1kb)	0323	145E-03	206E-02	Mir297	0	Hyper
chr16	18731836	18732717	Distal Intergenic	0714	145E-03	206E-02	Jund	3082	Hyper
chr17	61695938	61697707	Intron (NM_001107362/307075, intron 10 of 12)	0552	145E-03	206E-02	Idi1	-58581	Hyper
chr2	61758992	61760064	Distal Intergenic	0715	145E-03	206E-02	RGD1306502	104687	Hyper
chr7	836678	840908	Promoter (<=1kb)	0274	145E-03	206E-02	Nabp2	-127	Hyper
chr18	21851761	21853181	Intron (NM_022958/65052, intron 3 of 24)	0627	146E-03	207E-02	Pik3c3	6399	Hyper
chr11	30804986	30807734	3' UTR	0369	146E-03	207E-02	Ifngr2	25253	Hyper
chr6	119790865	119794612	Distal Intergenic	0346	146E-03	207E-02	Ttc7b	-85678	Hyper
chr1	13180156	13183384	Distal Intergenic	0343	146E-03	207E-02	Hebp2	18393	Hyper
chr4	157408065	157408640	Exon (NM_001002804/408246, exon 5 of 5)	0773	146E-03	207E-02	C1r	-4078	Hyper
chr20	6021325	6022827	Intron (NM_001107613/309639, intron 1 of 23)	0590	146E-03	207E-02	Anks1a	57647	Hyper
chr2	24553032	24555128	Intron (NM_031707/29546, intron 1 of 9)	0563	147E-03	207E-02	Homer1	9032	Hyper
chr1	197109751	197111827	Intron (NM_001109159/499288, intron 1 of 5)	0428	147E-03	208E-02	Mob2	38075	Hyper
chr15	13293037	13293900	Intron (NM_134356/171357, intron 2 of 32)	0671	147E-03	208E-02	Ptprg	258286	Hyper
chr19	48313300	48314974	Distal Intergenic	0521	147E-03	208E-02	6430548M08Rikl	115091	Hyper
chr7	116786014	116791187	Distal Intergenic	0433	147E-03	208E-02	Mirlet7c2	-12584	Hyper
chr2	138955792	138957818	Distal Intergenic	0436	147E-03	208E-02	Alg5	18291	Hyper
chr15	80197853	80198196	Distal Intergenic	0915	147E-03	208E-02	Mycbp2	-22421	Hyper
chr5	154446190	154447468	Intron (NM_001014070/313672, intron 2 of 7)	0568	147E-03	208E-02	Tmem51	-70308	Hyper

chr7	20366193	20367342	Distal Intergenic	0456	147E-03	208E-02	Nopchap1	-6664	Hyper
chr9	91796895	91798117	Intron (NM_031645/58965, intron 2 of 2)	0645	147E-03	208E-02	Ramp1	31334	Hyper
chr1	96984584	96986150	Distal Intergenic	0559	147E-03	208E-02	Kcnc1	81631	Hyper
chr6	88472602	88473211	Intron (NM_001009831/362750, intron 14 of 14)	0846	147E-03	208E-02	Sav1	26910	Hyper
chr3	15941959	15943925	Intron (NM_001010968/497010, intron 1 of 14)	0568	148E-03	208E-02	Eng	7393	Hyper
chr20	18910236	18912325	Intron (NM_031805/361833, intron 1 of 44)	0434	148E-03	208E-02	Ank3	17804	Hyper
chr1	17192332	17193823	Intron (NM_001029902/360302, intron 1 of 30)	0604	148E-03	208E-02	Ptprk	42864	Hyper
chr9	98419807	98437195	Promoter (<=1kb)	0260	148E-03	208E-02	Macir	0	Hyper
chr18	29677099	29681831	Exon (NM_001107393/307483, exon 24 of 29)	0335	148E-03	208E-02	Pcdhgc5	29898	Hyper
chr14	41536612	41537206	Distal Intergenic	0943	148E-03	208E-02	Uchl1	-41022	Hyper
chr8	104719239	104719845	Intron (NM_001108181/315973, intron 13 of 19)	0799	148E-03	209E-02	Acad11	37823	Hyper
chr7	17568631	17572418	Promoter (<=1kb)	0346	148E-03	209E-02	Timp3	0	Hyper
chr13	44454396	44454864	Intron (NM_001012026/304798, intron 5 of 10)	0881	148E-03	209E-02	Mdm4	19345	Hyper
chr2	27812523	27813253	Distal Intergenic	0785	148E-03	209E-02	Polk	69060	Hyper
chr13	47121472	47122352	Distal Intergenic	0790	149E-03	209E-02	Csrp1	-35819	Hyper
chr17	15333011	15333965	Promoter (2-3kb)	0699	149E-03	209E-02	Fgd3	-2857	Hyper
chr8	41900969	41902850	Promoter (<=1kb)	0531	149E-03	210E-02	Mir100	0	Hyper
chr1	235802192	235802975	Intron (NM_001354115/309499, intron 1 of 52)	0791	149E-03	210E-02	Myof	19359	Hyper
chr8	66629465	66630135	3' UTR	0735	150E-03	210E-02	Snx22	-16615	Hyper
chr7	90575678	90577172	Intron (NM_001130563/362918, intron 4 of 15)	0574	150E-03	210E-02	Mtss1	50670	Hyper
chr18	6180711	6182661	Intron (NM_001100516/291772, intron 1 of 4)	0492	150E-03	210E-02	Kctd1	34900	Hyper
chr5	29283890	29284556	Distal Intergenic	0758	150E-03	210E-02	Calb1	-91103	Hyper
chr15	45263002	45264814	Exon (NM_001005762/282843, exon 12 of 22)	0512	150E-03	210E-02	Pdlim2	-13760	Hyper
chr14	76819348	76822227	Exon (NM_001008339/305455, exon 2 of 11)	0453	150E-03	210E-02	Nelfa	10428	Hyper
chr10	75198122	75200264	Intron (NM_001100764/690286, intron 2 of 3)	0423	150E-03	211E-02	Hlf	11090	Hyper
chr13	65791903	65793017	Distal Intergenic	0657	150E-03	211E-02	Rgs8	-23417	Hyper
chr17	15289603	15291099	Intron (NM_001033674/306809, intron 1 of 6)	0513	150E-03	211E-02	Bicd2	13790	Hyper
chr4	167821224	167823719	Promoter (<=1kb)	0476	150E-03	211E-02	Apold1	0	Hyper
chr19	48552118	48554782	Distal Intergenic	0394	150E-03	211E-02	Gins2	84557	Hyper
chr15	17092427	17095106	Promoter (<=1kb)	0416	150E-03	211E-02	Flnb	0	Hyper
chr10	10858664	10860785	Exon (NM_001038596/360481, exon 10 of 11)	0488	151E-03	211E-02	Nmral1	16669	Hyper
chr17	35008847	35009560	Distal Intergenic	0721	151E-03	211E-02	Cdkal1	290167	Hyper
chr5	142358639	142365376	3' UTR	0327	151E-03	211E-02	Col16a1	-23537	Hyper

chr1	84226935	84230701	Intron (NM_031675/63836, intron 1 of 20)	0393	151E-03	211E-02	Actn4	21117	Hyper
chr4	104290176	104293257	Promoter (<=1kb)	0468	151E-03	211E-02	Atoh8	0	Hyper
chr1	15327727	15330652	Intron (NM_080894/140929, intron 2 of 12)	0462	151E-03	211E-02	Pde7b	162248	Hyper
chr19	51690252	51692032	Distal Intergenic	0468	151E-03	211E-02	Rab4a	-103581	Hyper
chr8	6729855	6731004	Distal Intergenic	0578	151E-03	211E-02	Pgr	656639	Hyper
chr2	169602073	169605740	Distal Intergenic	0309	152E-03	212E-02	Trim2	-37651	Hyper
chr2	223939082	223940176	Intron (NM_001031655/310864, intron 5 of 16)	0598	152E-03	212E-02	Manba	28650	Hyper
chr19	52281784	52283068	Intron (NM_001106196/292090, intron 1 of 13)	0629	152E-03	212E-02	Galnt2	6835	Hyper
chr17	6892286	6893207	Intron (NM_001271297/306759, intron 2 of 10)	0712	152E-03	212E-02	Spock1	150773	Hyper
chr10	90887585	90890926	Promoter (<=1kb)	0375	152E-03	212E-02	Cyb561	0	Hyper
chr6	92298138	92298428	Intron (NM_031085/81749, intron 1 of 13)	1157	153E-03	213E-02	Prkch	6138	Hyper
chr8	45452965	45456112	Exon (NM_001008880/315611, exon 2 of 5)	0401	154E-03	214E-02	Scn4b	5846	Hyper
chr1	117526384	117529008	Intron (NM_001109147/499171, intron 1 of 1)	0493	154E-03	214E-02	Klf13	7618	Hyper
chr1	46517916	46523082	Promoter (<=1kb)	0337	154E-03	214E-02	Synj2	0	Hyper
chr20	4935185	4937857	Promoter (<=1kb)	0325	154E-03	214E-02	B3galt4	0	Hyper
chr19	50605841	50607268	Promoter (<=1kb)	0526	154E-03	214E-02	Piezo1	0	Hyper
chr13	44451283	44454164	Exon (NM_001012026/304798, exon 6 of 11)	0389	154E-03	214E-02	Mdm4	20045	Hyper
chr13	64086080	64087264	Intron (NM_001105959/289084, intron 2 of 5)	0564	154E-03	214E-02	RGD1309104	71829	Hyper
chr5	57636539	57638037	Distal Intergenic	0500	154E-03	214E-02	Tesk1	-53932	Hyper
chr4	148859854	148861773	Exon (NM_181362/192226, exon 14 of 15)	0470	154E-03	214E-02	Rpl32	5839	Hyper
chr14	2488679	2490383	Promoter (<=1kb)	0363	154E-03	214E-02	Tgfbr3	0	Hyper
chr4	178917407	178918119	Intron (NM_001109255/500364, intron 1 of 2)	0716	154E-03	214E-02	Sspn	20260	Hyper
chr17	27777898	27778380	Distal Intergenic	0969	155E-03	215E-02	F13a1	-37343	Hyper
chr6	88798075	88800136	Intron (NM_130420/155812, intron 3 of 9)	0489	155E-03	215E-02	Trim9	56406	Hyper
chr7	14526109	14527603	Intron (NM_032617/79434, intron 1 of 4)	0633	155E-03	215E-02	Rab11b	6986	Hyper
chr4	178290543	178290994	Distal Intergenic	1199	155E-03	215E-02	Kras	-75209	Hyper
chr18	26727769	26729599	Promoter (<=1kb)	0383	156E-03	215E-02	Ctnna1	0	Hyper
chr2	152800225	152801175	Intron (NM_001100666/295105, intron 5 of 10)	0728	156E-03	216E-02	Il12a	-164594	Hyper
chr14	96826408	96827576	Intron (NM_001107240/305571, intron 2 of 2)	0676	156E-03	216E-02	B3gnt2	5722	Hyper
chr12	42238472	42240075	Intron (NM_001009973/494521, intron 2 of 6)	0642	156E-03	216E-02	Kctd10	8192	Hyper
chr12	5794346	5794987	Distal Intergenic	0821	157E-03	217E-02	Alox5ap	-21360	Hyper
chr6	25462749	25464141	Promoter (<=1kb)	0558	157E-03	217E-02	Emilin1	0	Hyper
chr9	75914553	75919156	Promoter (<=1kb)	0310	157E-03	217E-02	Pnkd	0	Hyper

chr5	160381813	160386456	Distal Intergenic	0359	157E-03	217E-02	Spsb1	-10551	Hyper
chr13	75449642	75450670	Distal Intergenic	0627	157E-03	217E-02	Fmo3	-121458	Hyper
chr3	79427341	79428978	Promoter (2-3kb)	0498	158E-03	217E-02	Cd82	2380	Hyper
chr10	86614957	86615879	Distal Intergenic	0683	158E-03	217E-02	Arl4d	14270	Hyper
chr20	30455765	30459299	Promoter (<=1kb)	0429	158E-03	217E-02	Srgn	0	Hyper
chr10	71399347	71403334	Promoter (2-3kb)	0402	159E-03	219E-02	Mir21	2014	Hyper
chr10	38950408	38951445	Distal Intergenic	0653	159E-03	219E-02	Lyrm7	31818	Hyper
chr6	130639831	130640503	Intron (NM_130749/170577, intron 1 of 17)	0801	159E-03	219E-02	Mark3	12349	Hyper
chr15	41046303	41056800	Intron (NM_001105717/25416, intron 3 of 13)	0325	159E-03	219E-02	Dpysl2	16057	Hyper
chr6	31301903	31305700	Distal Intergenic	0421	159E-03	219E-02	Rhob	60223	Hyper
chr11	84763897	84768887	Promoter (<=1kb)	0327	159E-03	219E-02	Cebpd	0	Hyper
chr2	175283125	175283979	Distal Intergenic	0758	160E-03	219E-02	Il6r	63545	Hyper
chr18	55132849	55135303	Distal Intergenic	0396	160E-03	219E-02	Il17b	-5891	Hyper
chr7	10096247	10097406	Distal Intergenic	0668	160E-03	219E-02	C2cd4c	4326	Hyper
chr10	8356963	8358384	Intron (NM_001106974/302920, intron 1 of 12)	0612	160E-03	219E-02	Rbfox1	134389	Hyper
chr8	84473539	84473849	Intron (NM_001137647/501026, intron 1 of 3)	1031	160E-03	220E-02	Sh3bgrl2	12418	Hyper
chr19	49669240	49670427	Exon (NM_022867/64862, exon 2 of 4)	0672	160E-03	220E-02	Map1lc3b	3261	Hyper
chr10	82034774	82037288	Promoter (<=1kb)	0383	160E-03	220E-02	Osbpl7	0	Hyper
chr1	151014553	151016816	Intron (NM_001191628/308831, intron 4 of 31)	0445	161E-03	220E-02	Tenm4	234172	Hyper
chrX	9297709	9300695	Promoter (1-2kb)	0386	161E-03	220E-02	Nyx	1205	Hyper
chr3	156253191	156254838	Downstream (<=300bp)	0523	161E-03	220E-02	Snai1	4712	Hyper
chr7	9845358	9852460	Promoter (<=1kb)	0287	161E-03	220E-02	Ptbp1	0	Hyper
chr11	70075667	70076403	Distal Intergenic	0799	161E-03	220E-02	Fam43a	54316	Hyper
chr11	34296950	34297932	Intron (NM_013192/25743, intron 1 of 5)	0621	161E-03	220E-02	Kcnj6	10826	Hyper
chr7	30864889	30865445	Distal Intergenic	0794	161E-03	220E-02	Eea1	259896	Hyper
chr7	110484014	110486912	Promoter (<=1kb)	0439	161E-03	220E-02	Lgals1	0	Hyper
chr4	141803298	141804515	Intron (NM_001305279/297504, intron 1 of 11)	0573	161E-03	220E-02	Edem1	5597	Hyper
chr17	15261337	15263150	3' UTR	0506	161E-03	220E-02	Ippk	-31907	Hyper
chr20	5292095	5293274	Promoter (<=1kb)	0630	161E-03	221E-02	Lemd2	472	Hyper
chr1	117558131	117559085	Distal Intergenic	0807	162E-03	221E-02	Klf13	-21505	Hyper
chr19	53457712	53458788	Intron (NM_001009704/361442, intron 3 of 23)	0649	162E-03	221E-02	Sipa1l2	81640	Hyper
chr12	26548194	26549879	Intron (NM_001011903/288617, intron 4 of 5)	0551	162E-03	221E-02	Kctd7	-16056	Hyper
chr2	30885314	30891663	Intron (NM_019217/29456, intron 2 of 6)	0362	162E-03	221E-02	Map1b	18654	Hyper

chr3	8289144	8289697	Distal Intergenic	0987	162E-03	221E-02	Ptgds	-4311	Hyper
chr1	108136774	108138686	Intron (NM_024370/79211, intron 3 of 9)	0489	163E-03	222E-02	Gabrg3	108051	Hyper
chr1	45643578	45645494	Distal Intergenic	0468	163E-03	222E-02	Ldhal6b	353774	Hyper
chr14	1367967	1370908	Promoter (1-2kb)	0320	163E-03	222E-02	Pde6b	-1517	Hyper
chr8	96360067	96362553	Distal Intergenic	0380	163E-03	222E-02	Pls1	-10334	Hyper
chr5	165709452	165710918	Distal Intergenic	0461	163E-03	222E-02	Morn1	62456	Hyper
chr7	111727611	111728662	Distal Intergenic	0656	163E-03	222E-02	Mgat3	-5057	Hyper
chr9	1212192	1217309	Intron (NM_001044236/301128, intron 8 of 22)	0365	164E-03	222E-02	Kdm4b	53413	Hyper
chr12	45134564	45135253	Distal Intergenic	0833	164E-03	223E-02	Mn1	84849	Hyper
chr17	45789369	45793264	Exon (NM_022217/60668, exon 9 of 21)	0298	164E-03	223E-02	Amph	189543	Hyper
chr12	15386587	15387621	Intron (NM_001163320/288518, intron 1 of 8)	0660	164E-03	223E-02	Get4	6432	Hyper
chr12	39211626	39212910	Exon (NM_001107146/304528, exon 5 of 11)	0577	164E-03	223E-02	Rfc5	4142	Hyper
chr12	13397663	13398210	Distal Intergenic	0803	164E-03	223E-02	Gna12	-407488	Hyper
chr3	154579136	154582047	Exon (NM_001100838/296374, exon 6 of 22)	0415	164E-03	223E-02	Zmynd8	45119	Hyper
chr2	76435231	76436016	Intron (NM_001034912/619558, intron 3 of 8)	0763	164E-03	223E-02	Zfp622	-44535	Hyper
chr4	129896745	129897838	Distal Intergenic	0635	164E-03	223E-02	Arl6ip5	81270	Hyper
chr13	91548545	91551101	Distal Intergenic	0365	164E-03	223E-02	Ahctf1	-12154	Hyper
chr20	27926261	27926812	Distal Intergenic	0854	165E-03	223E-02	Ascc1	-14588	Hyper
chrX	67121277	67123792	Distal Intergenic	0443	165E-03	223E-02	Ercc6l	137430	Hyper
chr3	56596067	56596545	Distal Intergenic	0822	165E-03	223E-02	Itga6	-20999	Hyper
chr4	148628956	148630628	Exon (NM_001014057/312649, exon 8 of 12)	0484	166E-03	224E-02	Tsen2	26085	Hyper
chr16	76592004	76594895	Intron (NM_053951/117020, intron 1 of 30)	0394	166E-03	224E-02	Mcf2l	17687	Hyper
chr15	98807851	98809018	Intron (NM_001105759/259237, intron 1 of 52)	0659	166E-03	224E-02	Dock9	74135	Hyper
chr4	139262288	139265025	Intron (NM_053879/116658, intron 4 of 24)	0444	166E-03	225E-02	Il5ra	398944	Hyper
chr3	15464103	15467609	Promoter (<=1kb)	0365	166E-03	225E-02	Prrc2b	0	Hyper
chr10	104619536	104620821	Distal Intergenic	0542	166E-03	225E-02	Eif4a3	-60504	Hyper
chr3	18945408	18946153	Intron (NM_138710/192126, intron 1 of 15)	0723	166E-03	225E-02	Dab2ip	30118	Hyper
chr7	108939036	108939936	Intron (NM_001079895/362950, intron 1 of 12)	0692	166E-03	225E-02	Rbfox2	114467	Hyper
chr12	6784347	6784725	Exon (NM_001374100/498136, exon 7 of 15)	0958	167E-03	225E-02	Mtus2	-33210	Hyper
chr3	13543019	13544111	Promoter (2-3kb)	0554	167E-03	225E-02	Phyhd1	2610	Hyper
chr7	57781598	57782890	Promoter (<=1kb)	0353	167E-03	225E-02	Rxylt1	0	Hyper
chr5	144145865	144147331	Distal Intergenic	0538	167E-03	225E-02	Srsf4	84402	Hyper
chr1	78911374	78912423	Exon (NM_001108475/361517, exon 10 of 13)	0651	167E-03	226E-02	Vasp	12517	Hyper

chr3	106955517	106957042	Intron (NM_001108589/362197, intron 1 of 30)	0449	168E-03	226E-02	Mapkbp1	7690	Hyper
chr12	42254703	42256109	Distal Intergenic	0548	168E-03	226E-02	Kctd10	24423	Hyper
chr4	11757751	11759550	Distal Intergenic	0475	168E-03	226E-02	Kmt2e	-30378	Hyper
chr7	58637588	58639023	Distal Intergenic	0572	168E-03	226E-02	Mirlet7i	3546	Hyper
chr17	14383779	14384748	Intron (NR_144439/689316, intron 2 of 2)	0610	168E-03	226E-02	Fbxw17	24225	Hyper
chr5	58152452	58153138	Intron (NM_001107954/313488, intron 12 of 19)	0777	168E-03	226E-02	Reck	49465	Hyper
chr7	114466313	114467744	Intron (NM_001044273/503165, intron 2 of 15)	0589	168E-03	226E-02	Arfgap3	11429	Hyper
chr7	51907895	51908989	Exon (NM_053594/94202, exon 12 of 14)	0713	168E-03	226E-02	Ptprb	-44386	Hyper
chr11	66228041	66232942	Distal Intergenic	0323	168E-03	226E-02	Kalrn	-22027	Hyper
chr2	245516914	245517290	Distal Intergenic	0984	169E-03	227E-02	Negr1	-107303	Hyper
chr15	6669894	6670559	Distal Intergenic	0658	169E-03	227E-02	Ube2e2	-315705	Hyper
chr5	101635963	101637368	Intron (NM_031743/84550, intron 1 of 9)	0543	169E-03	227E-02	Slc24a2	101969	Hyper
chr20	10024947	10026532	Distal Intergenic	0612	169E-03	227E-02	Sik1	-66008	Hyper
chr3	7499719	7504227	Exon (NM_147141/257648, exon 8 of 46)	0395	169E-03	227E-02	Cacna1b	41730	Hyper
chr1	87295119	87295647	Exon (NM_001107504/308511, exon 4 of 9)	0787	169E-03	227E-02	Kctd15	-23140	Hyper
chr10	54603674	54605799	Promoter (<=1kb)	0345	169E-03	227E-02	Kctd11	0	Hyper
chr14	75734304	75736360	Exon (NM_019339/54292, exon 4 of 16)	0430	169E-03	227E-02	Hgfac	-20162	Hyper
chr4	157744644	157745518	Distal Intergenic	0791	169E-03	227E-02	Mlf2	4505	Hyper
chr6	130949674	130951739	Distal Intergenic	0414	170E-03	227E-02	Ppp1r13b	-20025	Hyper
chr14	77362377	77367083	Exon (NM_001008319/298982, exon 4 of 9)	0356	170E-03	227E-02	Uvssa	-10474	Hyper
chr11	83717797	83721622	Exon (NM_022301/64161, exon 46 of 56)	0420	170E-03	227E-02	Serpind1	-42278	Hyper
chr1	199633720	199635843	Promoter (<=1kb)	0345	170E-03	227E-02	Cttn	0	Hyper
chr1	220007711	220010438	Promoter (<=1kb)	0387	170E-03	228E-02	Trpm3	0	Hyper
chr19	54724155	54724653	Distal Intergenic	0954	170E-03	228E-02	Tomm20	210535	Hyper
chr17	53227268	53227623	Distal Intergenic	0988	170E-03	228E-02	Mtpap	113858	Hyper
chr2	244336009	244336654	Distal Intergenic	0777	171E-03	228E-02	Lrriq3	215973	Hyper
chr10	42246711	42247410	Distal Intergenic	0716	171E-03	228E-02	Cnot8	-34369	Hyper
chr3	79410524	79411908	5' UTR	0642	171E-03	228E-02	Cd82	19450	Hyper
chr8	58607805	58618107	Distal Intergenic	0309	171E-03	228E-02	Islr	-34405	Hyper
chr5	123873697	123876262	Intron (NM_001044234/298369, intron 4 of 22)	0361	171E-03	229E-02	Osbpl9	85917	Hyper
chr10	104812297	104814271	3' UTR	0482	172E-03	229E-02	Nptx1	6087	Hyper
chr12	6882111	6887511	Intron (NM_001374100/498136, intron 4 of 14)	0299	172E-03	229E-02	Mtus2	-130974	Hyper
chr4	162989394	162991365	3' UTR	0420	172E-03	229E-02	Gabarapl1	9085	Hyper

chr17	15013121	15013854	Promoter (<=1kb)	0495	172E-03	229E-02	Nol8	0	Hyper
chr3	88715592	88716447	Intron (NM_001013203/362172, intron 1 of 4)	0678	172E-03	229E-02	Trim44	12190	Hyper
chr10	66090062	66091247	Intron (NM_001034014/25364, intron 1 of 9)	0633	173E-03	230E-02	Asic2	47132	Hyper
chr9	98481824	98484960	Distal Intergenic	0356	173E-03	230E-02	Macir	61064	Hyper
chr6	123741966	123742977	Intron (NM_001106755/299285, intron 3 of 12)	0667	173E-03	230E-02	Clmn	64295	Hyper
chr13	92298892	92300457	Distal Intergenic	0533	173E-03	230E-02	Parp1	-7136	Hyper
chr1	41868285	41869497	Distal Intergenic	0532	173E-03	230E-02	Myct1	-148640	Hyper
chr5	161066664	161070046	Intron (NM_053885/116665, intron 12 of 22)	0454	173E-03	230E-02	Slc45a1	59257	Hyper
chr17	26266306	26266621	Distal Intergenic	1060	173E-03	230E-02	Txndc5	-23259	Hyper
chr5	116209332	116211691	Intron (NM_001107949/313409, intron 1 of 17)	0512	173E-03	230E-02	Leprot	-78152	Hyper
chr18	58772432	58774121	Distal Intergenic	0536	173E-03	230E-02	Mir3591	-13634	Hyper
chr9	60225613	60226283	Intron (NM_001033864/117279, intron 9 of 10)	0783	174E-03	231E-02	Casp8	-37580	Hyper
chr9	11566458	11568175	Promoter (<=1kb)	0380	174E-03	231E-02	Mocs1	0	Hyper
chr19	18496512	18501046	Intron (NM_001271381/364952, intron 3 of 9)	0436	174E-03	231E-02	Nkd1	48334	Hyper
chr10	59497231	59500356	Distal Intergenic	0313	174E-03	231E-02	Cluh	-9370	Hyper
chr10	103491542	103493541	Distal Intergenic	0437	174E-03	231E-02	Cyth1	-5782	Hyper
chr19	39710539	39716575	Distal Intergenic	0317	175E-03	232E-02	Bcar1	-8256	Hyper
chr17	9811468	9812584	Distal Intergenic	0626	175E-03	232E-02	Tspan17	14215	Hyper
chr14	76144853	76152367	Intron (NM_016990/24170, intron 1 of 14)	0286	175E-03	232E-02	Add1	14774	Hyper
chr4	84369477	84371155	Distal Intergenic	0537	175E-03	232E-02	Inmt	-46984	Hyper
chr1	84764490	84765793	Distal Intergenic	0559	175E-03	232E-02	Sipa1l3	-60688	Hyper
chr17	18441866	18442145	Distal Intergenic	1091	175E-03	232E-02	Rbm24	-154447	Hyper
chr15	51961207	51963501	Distal Intergenic	0433	175E-03	232E-02	Serp2	-57242	Hyper
chr3	22581109	22582140	Distal Intergenic	0607	176E-03	232E-02	Mir181a2	-33705	Hyper
chr1	117929216	117929981	Intron (NM_001191633/309256, intron 7 of 12)	0685	176E-03	232E-02	Fan1	14445	Hyper
chr14	83194329	83195988	Exon (NM_001170459/360980, exon 19 of 29)	0541	176E-03	232E-02	Tns3	148150	Hyper
chr4	69859275	69859744	Distal Intergenic	0827	176E-03	232E-02	Prss3b	15774	Hyper
chr12	44174375	44176632	Exon (NM_001134538/304554, exon 6 of 17)	0489	176E-03	232E-02	Asphd2	-71513	Hyper
chr6	57080403	57085103	Promoter (<=1kb)	0319	176E-03	232E-02	Arl4a	0	Hyper
chr17	6819929	6820611	Intron (NM_001271297/306759, intron 2 of 10)	0767	176E-03	233E-02	Spock1	78416	Hyper
chr3	147309895	147311238	Distal Intergenic	0528	176E-03	233E-02	Ppp1r16b	-12888	Hyper
chr3	87706334	87709262	Distal Intergenic	0410	177E-03	233E-02	Rag2	-193111	Hyper
chr7	46634721	46637407	Intron (NM_001309455/314824, intron 1 of 23)	0418	177E-03	233E-02	Osbpl8	37738	Hyper

chr15	48656369	48659531	Distal Intergenic	0402	177E-03	233E-02	Med4	-37011	Hyper
chr5	68720300	68721322	Distal Intergenic	0717	177E-03	233E-02	Tmem38b	259996	Hyper
chr2	175188330	175191941	Promoter (<=1kb)	0311	177E-03	233E-02	Chrnb2	0	Hyper
chr1	90453649	90455258	Distal Intergenic	0524	178E-03	234E-02	Uri1	249398	Hyper
chr4	154209575	154213918	Intron (NM_001191085/362427, intron 20 of 32)	0389	178E-03	234E-02	Bid	-73422	Hyper
chr5	148776948	148778149	3' UTR	0572	178E-03	234E-02	Kdm1a	60170	Hyper
chr5	35515456	35518477	Intron (NM_001271065/366333, intron 4 of 5)	0377	178E-03	234E-02	Faxc	35054	Hyper
chr12	667706	669364	Intron (NM_001109060/498130, intron 1 of 13)	0524	178E-03	235E-02	Stard13	65240	Hyper
chr5	122005742	122006985	Distal Intergenic	0622	179E-03	235E-02	Lrrc42	28807	Hyper
chr2	211114897	211115421	Intron (NM_001106468/295425, intron 1 of 14)	0809	179E-03	235E-02	Usp53	5528	Hyper
chr12	44344433	44349438	Intron (NM_001008508/288719, intron 1 of 6)	0324	179E-03	235E-02	Tpst2	12891	Hyper
chr4	117137173	117137942	Intron (NM_001109246/500233, intron 20 of 21)	0764	179E-03	235E-02	Cyp26b1	-78545	Hyper
chr13	21955495	21956860	Exon (NM_001271235/498206, exon 3 of 10)	0629	179E-03	235E-02	Tnfrsf11a	27087	Hyper
chr13	34343816	34344448	Distal Intergenic	0742	179E-03	235E-02	Dpp10	909864	Hyper
chr3	19349599	19350796	Distal Intergenic	0550	179E-03	235E-02	Ndufa8	48663	Hyper
chr11	69989163	69990197	Distal Intergenic	0647	179E-03	235E-02	Fam43a	140522	Hyper
chr6	122365081	122365995	Intron (NM_001108721/690195, intron 3 of 4)	0749	180E-03	236E-02	Prima1	23840	Hyper
chr3	88714946	88715369	Intron (NM_001013203/362172, intron 1 of 4)	0974	180E-03	236E-02	Trim44	13268	Hyper
chr2	28900552	28900930	Distal Intergenic	0861	180E-03	236E-02	Hexb	-396387	Hyper
chr10	91447122	91449624	Distal Intergenic	0424	180E-03	236E-02	Ern1	-55243	Hyper
chr13	45661881	45662979	3' UTR	0569	180E-03	236E-02	Mybph	8594	Hyper
chr1	187814832	187817597	Promoter (2-3kb)	0355	180E-03	236E-02	Ctbp2	-2984	Hyper
chr19	39428672	39429900	Distal Intergenic	0646	181E-03	236E-02	Fa2h	-64519	Hyper
chr3	69872880	69874465	Promoter (<=1kb)	0475	181E-03	237E-02	Ube2l6	0	Hyper
chr1	246785653	246786907	Promoter (<=1kb)	0460	182E-03	238E-02	Mir3584	0	Hyper
chr17	9098383	9099881	3' UTR	0645	182E-03	238E-02	Ddx41	-3142	Hyper
chr2	61853689	61854352	Exon (NM_001107654/310158, exon 7 of 9)	0824	182E-03	238E-02	RGD1306502	10399	Hyper
chr2	94589821	94590198	Distal Intergenic	1124	182E-03	238E-02	Pkia	-116222	Hyper
chr5	131803741	131805222	Intron (NM_019249/360406, intron 1 of 30)	0628	182E-03	238E-02	Ptprf	4801	Hyper
chr6	69309545	69311286	Distal Intergenic	0494	182E-03	238E-02	Dtd2	145144	Hyper
chr6	127750964	127754161	Promoter (<=1kb)	0350	183E-03	238E-02	Slc25a29	0	Hyper
chr1	117469688	117473119	Distal Intergenic	0364	183E-03	238E-02	Klf13	63507	Hyper
chr3	77947686	77951493	Distal Intergenic	0336	183E-03	238E-02	Dgkz	-13646	Hyper

chr1	120843691	120845101	Distal Intergenic	0631	183E-03	238E-02	Mef2a	136859	Hyper
chr10	73816528	73816944	Intron (NM_001009536/494338, intron 1 of 8)	0912	183E-03	238E-02	Trim25	3710	Hyper
chr19	48048487	48049173	Distal Intergenic	0804	183E-03	239E-02	Crispld2	-21166	Hyper
chr19	48610615	48614651	Distal Intergenic	0330	183E-03	239E-02	Gins2	24688	Hyper
chr20	4738072	4738820	Distal Intergenic	0701	184E-03	239E-02	Brd2	9897	Hyper
chr7	107922100	107926861	Promoter (<=1kb)	0317	184E-03	239E-02	Plec	0	Hyper
chr12	38597124	38598501	Intron (NM_001108338/360818, intron 3 of 11)	0639	184E-03	239E-02	Fbxo21	6632	Hyper
chr1	96617733	96619817	Exon (NM_013039/25559, exon 18 of 39)	0413	184E-03	239E-02	Kcnj11	-23651	Hyper
chr1	23380800	23382701	Distal Intergenic	0479	184E-03	239E-02	Clvs2	-352571	Hyper
chr2	27852148	27854790	Intron (NM_138516/171525, intron 3 of 14)	0445	184E-03	239E-02	Polk	27523	Hyper
chr6	132063148	132065512	Exon (NM_001106761/299347, exon 4 of 18)	0495	185E-03	240E-02	Btbd6	5018	Hyper
chr1	220170754	220173262	Intron (NM_001191562/309407, intron 1 of 24)	0398	185E-03	240E-02	Mir204	-143669	Hyper
chr17	17464661	17465380	Intron (NM_001108881/364681, intron 2 of 7)	0689	185E-03	240E-02	Rnf144b	97380	Hyper
chr5	68222356	68222951	Intron (NM_053492/85254, intron 15 of 15)	0809	185E-03	240E-02	Fktn	-116852	Hyper
chr1	23307504	23309248	Distal Intergenic	0436	185E-03	240E-02	Sgk1	-319407	Hyper
chr2	204379535	204381957	Promoter (<=1kb)	0388	186E-03	240E-02	Cdc14a	0	Hyper
chr8	19996073	19999158	Intron (NM_013199/25751, intron 1 of 20)	0414	186E-03	240E-02	Dnm2	17623	Hyper
chr4	120193393	120194759	Intron (NM_001163921/685202, intron 2 of 7)	0667	186E-03	240E-02	Efcc1	4923	Hyper
chr2	55514069	55515970	Distal Intergenic	0466	186E-03	240E-02	Dab2	-28916	Hyper
chr2	152098504	152099208	Distal Intergenic	0780	186E-03	240E-02	Schip1	-27963	Hyper
chr5	109606620	109608349	Promoter (<=1kb)	0469	186E-03	240E-02	Tek	0	Hyper
chr2	203683737	203684617	Distal Intergenic	0722	186E-03	241E-02	S1pr1	-54627	Hyper
chr3	8006348	8008615	Promoter (<=1kb)	0419	186E-03	241E-02	Tor4a	0	Hyper
chr2	223941573	223942505	Intron (NM_001031655/310864, intron 5 of 16)	0673	186E-03	241E-02	Manba	31141	Hyper
chr6	102802626	102804531	Distal Intergenic	0415	186E-03	241E-02	Dpf3	320962	Hyper
chr16	17356439	17357283	Intron (NM_001109107/498599, intron 4 of 5)	0763	187E-03	241E-02	Slc35e1	5451	Hyper
chr3	119201433	119201983	3' UTR	0850	187E-03	241E-02	Prnd	-13573	Hyper
chr14	96748143	96748471	Distal Intergenic	1082	187E-03	241E-02	B3gnt2	84827	Hyper
chr18	62836785	62837539	Distal Intergenic	0690	187E-03	241E-02	Tcf4	-221917	Hyper
chr5	141507851	141509823	Intron (NM_001134628/500551, intron 5 of 6)	0468	187E-03	241E-02	Fndc5	24148	Hyper
chr16	1281117	1282496	Exon (NM_001108394/361104, exon 3 of 4)	0576	187E-03	242E-02	Ppif	23920	Hyper
chr9	93774050	93775156	Promoter (<=1kb)	0614	188E-03	242E-02	Sned1	0	Hyper
chr3	106950973	106955433	Intron (NM_001108589/362197, intron 1 of 30)	0347	188E-03	242E-02	Mapkbp1	3146	Hyper

chr4	118263648	118264314	Intron (NM_080884/114020, intron 1 of 1)	0734	188E-03	242E-02	Nat8	15985	Hyper
chr5	84021116	84021543	Intron (NM_001107940/313270, intron 3 of 5)	0741	188E-03	242E-02	Cdk5rap2	-60334	Hyper
chr5	74735035	74736815	Intron (NM_001106651/298033, intron 1 of 8)	0536	188E-03	242E-02	Snx30	51015	Hyper
chr14	78365541	78366432	Promoter (1-2kb)	0827	188E-03	242E-02	Pla2g3	-1801	Hyper
chr10	104659034	104660155	Distal Intergenic	0519	189E-03	243E-02	Eif4a3	-100002	Hyper
chr3	106962247	106965438	Intron (NM_001108589/362197, intron 1 of 30)	0365	189E-03	243E-02	Mapkbp1	14420	Hyper
chr6	21489253	21489804	Distal Intergenic	0820	189E-03	243E-02	Xdh	-40659	Hyper
chr20	11388206	11390104	Distal Intergenic	0419	189E-03	243E-02	Pofut2	-10418	Hyper
chr16	18368108	18368982	Intron (NM_022861/64829, intron 9 of 42)	0718	189E-03	243E-02	Unc13a	12823	Hyper
chr2	224256193	224257308	Promoter (<=1kb)	0552	189E-03	243E-02	Slc39a8	0	Hyper
chr4	30704027	30705144	Intron (NM_001191861/114483, intron 3 of 6)	0600	189E-03	243E-02	Fam133b	-88004	Hyper
chr12	33051490	33053093	Distal Intergenic	0467	189E-03	243E-02	Diablo	-4018	Hyper
chr2	174650358	174650883	Exon (NM_001107691/310641, exon 4 of 9)	0845	190E-03	243E-02	Trim46	3245	Hyper
chr10	86533915	86534800	Distal Intergenic	0513	190E-03	243E-02	Tmem106a	26820	Hyper
chr1	80978631	80982563	Promoter (1-2kb)	0323	190E-03	244E-02	Lipe	1747	Hyper
chr5	146871623	146876199	Intron (NM_001108687/362625, intron 1 of 11)	0344	190E-03	244E-02	Man1c1	37058	Hyper
chr3	9455574	9459443	Promoter (<=1kb)	0311	190E-03	244E-02	Dipk1b	0	Hyper
chr6	103180955	103181527	3' UTR	0869	190E-03	244E-02	Dcaf4	26088	Hyper
chr12	41025321	41025806	Exon (NM_001168664/690632, exon 24 of 58)	0944	191E-03	244E-02	Gcn1	26725	Hyper
chr8	110652727	110653848	Intron (NM_001108783/363151, intron 1 of 6)	0570	191E-03	245E-02	Ccdc12	17017	Hyper
chr3	155694379	155694678	Exon (NM_001109907/84496, exon 3 of 12)	0969	191E-03	245E-02	Stau1	31231	Hyper
chr1	234736495	234737552	Intron (NM_001191917/368042, intron 35 of 37)	0614	191E-03	245E-02	Btaf1	68043	Hyper
chr10	82402624	82404848	Distal Intergenic	0470	191E-03	245E-02	Mrpl45	93974	Hyper
chr1	199491591	199501810	Intron (NM_201350/171093, intron 16 of 22)	0293	191E-03	245E-02	Shank2	35211	Hyper
chr2	210752716	210753702	Distal Intergenic	0669	191E-03	245E-02	Fnbp1l	-14454	Hyper
chr4	68022863	68024250	Distal Intergenic	0536	192E-03	245E-02	Chmp4bl1	-25492	Hyper
chr2	219401948	219402627	Distal Intergenic	0740	192E-03	245E-02	Rpl34	-113840	Hyper
chr6	122885345	122886061	Promoter (2-3kb)	0729	192E-03	245E-02	Serpina1	2278	Hyper
chr6	5890121	5893109	Distal Intergenic	0409	192E-03	245E-02	Ston1	-28024	Hyper
chr19	52851141	52853210	Distal Intergenic	0487	192E-03	245E-02	Exoc8	4289	Hyper
chr7	23490768	23492067	Intron (NM_001106778/299714, intron 5 of 25)	0554	192E-03	245E-02	Ano4	159665	Hyper
chr7	14613805	14614899	Exon (NM_001106772/299643, exon 3 of 4)	0618	192E-03	245E-02	Ndufa7	4522	Hyper
chr7	115325487	115330105	Exon (NM_001004241/300111, exon 5 of 15)	0366	192E-03	245E-02	Samm50	8015	Hyper

chr19	49732852	49735106	Distal Intergenic	0376	192E-03	246E-02	Jph3	-59217	Hyper
chr12	6497839	6503278	Intron (NM_001015030/363869, intron 1 of 4)	0334	193E-03	246E-02	Ubl3	24155	Hyper
chr3	78461780	78463396	Distal Intergenic	0442	193E-03	246E-02	Slc35c1	-33305	Hyper
chr3	19051984	19052789	Intron (NM_138710/192126, intron 3 of 15)	0694	193E-03	246E-02	Dab2ip	136694	Hyper
chr3	141893815	141896267	Distal Intergenic	0542	193E-03	246E-02	Kif3b	135349	Hyper
chr1	78807046	78808986	Exon (NM_012714/25024, exon 8 of 15)	0457	193E-03	246E-02	Gipr	5476	Hyper
chr2	233570924	233573082	Intron (NM_001100518/292155, intron 1 of 6)	0447	194E-03	247E-02	Hs2st1	68687	Hyper
chr1	79586574	79587726	Distal Intergenic	0501	194E-03	247E-02	Pvr	-9874	Hyper
chr1	213548328	213549646	Intron (NM_031036/81666, intron 2 of 6)	0485	194E-03	247E-02	Gnaq	122602	Hyper
chr12	14126578	14129929	Distal Intergenic	0306	194E-03	247E-02	Chst12	-14294	Hyper
chr18	53870820	53871144	Promoter (<=1kb)	0970	194E-03	247E-02	MGC105567	0	Hyper
chr17	6883833	6884513	Intron (NM_001271297/306759, intron 2 of 10)	0831	195E-03	248E-02	Spock1	142320	Hyper
chr1	205919975	205921265	Distal Intergenic	0526	195E-03	248E-02	Ahnak	37702	Hyper
chr6	100195784	100196366	Intron (NM_001100863/362760, intron 1 of 14)	0824	195E-03	248E-02	Galnt16	25416	Hyper
chr4	119343162	119344135	Intron (NM_001173450/500244, intron 1 of 20)	0603	195E-03	248E-02	Rpl22	6748	Hyper
chr16	18616795	18617536	Distal Intergenic	0798	196E-03	248E-02	Arrdc2	14898	Hyper
chr3	13351139	13351608	Intron (NM_001047861/296619, intron 9 of 25)	0945	197E-03	249E-02	Zdhhc12	14603	Hyper
chr1	117388649	117389600	Distal Intergenic	0607	197E-03	249E-02	Klf13	147026	Hyper
chr16	63551448	63551970	Distal Intergenic	0840	197E-03	249E-02	Unc5d	691019	Hyper
chr3	49849026	49849417	Distal Intergenic	0915	197E-03	249E-02	Grb14	-165221	Hyper
chr4	67882896	67884438	Promoter (<=1kb)	0489	197E-03	249E-02	Parp12	0	Hyper
chr10	52340273	52343156	Exon (NM_053484/85246, exon 4 of 14)	0450	197E-03	249E-02	Rcvrn	-45550	Hyper
chr5	74702456	74704767	Intron (NM_001106651/298033, intron 1 of 8)	0385	197E-03	249E-02	Snx30	18436	Hyper
chr10	105967138	105968768	Distal Intergenic	0557	197E-03	249E-02	Lrrc45	-25751	Hyper
chr10	82518703	82521676	Exon (NM_019378/56029, exon 12 of 18)	0414	197E-03	250E-02	Srcin1	47023	Hyper
chr8	120839098	120839959	Distal Intergenic	0589	198E-03	250E-02	Ctnnb1	182513	Hyper
chr1	155144411	155147281	Intron (NM_001108494/361614, intron 2 of 8)	0440	198E-03	250E-02	Relt	66915	Hyper
chr8	62039382	62040876	Distal Intergenic	0627	198E-03	250E-02	Tle3	181182	Hyper
chr15	28549241	28552802	Intron (NM_001098803/305888, intron 1 of 9)	0378	198E-03	250E-02	Zfhx2	12326	Hyper
chr18	26084740	26086046	Intron (NM_001106158/291694, intron 7 of 20)	0572	198E-03	251E-02	Fam13b	20541	Hyper
chr19	18501746	18502485	Intron (NM_001271381/364952, intron 3 of 9)	0787	199E-03	251E-02	Nkd1	46895	Hyper
chr16	51327659	51328955	Intron (NM_178093/306487, intron 3 of 9)	0654	199E-03	251E-02	Mtus1	32422	Hyper
chr19	48562736	48563863	Distal Intergenic	0559	199E-03	251E-02	Gins2	75476	Hyper

chr20	28123038	28124657	Promoter (2-3kb)	0627	199E-03	251E-02	Chst3	-2071	Hyper
chr8	109075998	109077032	Promoter (2-3kb)	0619	199E-03	251E-02	LOC498675	-3000	Hyper
chr1	46431876	46433476	Intron (NM_001127637/683687, intron 1 of 17)	0518	200E-03	251E-02	Snx9	6908	Hyper
chr5	19485708	19486136	Distal Intergenic	0839	200E-03	251E-02	Sdcbp	-3865	Hyper
chr13	22707354	22707905	Intron (NM_016993/24224, intron 1 of 1)	0787	200E-03	251E-02	Bcl2	144333	Hyper
chr11	30310317	30311057	3' UTR	0701	200E-03	252E-02	RGD1562726	14247	Hyper
chr10	64095097	64098487	Promoter (<=1kb)	0292	200E-03	252E-02	Ksr1	0	Hyper
chr19	30233653	30234272	Promoter (<=1kb)	0851	200E-03	252E-02	Ednra	0	Hyper
chr4	80472838	80473712	Distal Intergenic	0803	200E-03	252E-02	Nfe2l3	-33044	Hyper
chr6	105665725	105670385	Distal Intergenic	0327	201E-03	252E-02	Tgfb3	55590	Hyper
chr3	149415304	149420728	3' UTR	0308	201E-03	252E-02	Zhx3	15861	Hyper
chr2	185855798	185856475	Distal Intergenic	0704	201E-03	252E-02	Hmgcs2	-19159	Hyper
chr4	124886172	124893149	Intron (NM_001107876/312563, intron 7 of 7)	0310	201E-03	252E-02	Trh	-141535	Hyper
chr12	41392538	41393172	Distal Intergenic	0761	201E-03	252E-02	Pop5	-11202	Hyper
chr8	99512557	99512882	Distal Intergenic	1084	201E-03	252E-02	Faim	-31087	Hyper
chr10	85654957	85657440	Intron (NM_001105840/287709, intron 1 of 5)	0424	201E-03	253E-02	Rab5c	8695	Hyper
chr10	103337482	103337898	Distal Intergenic	0965	201E-03	253E-02	Socs3	-141384	Hyper
chr10	59393306	59397022	Distal Intergenic	0347	202E-03	253E-02	Rap1gap2	-83631	Hyper
chr3	156051359	156053377	Distal Intergenic	0493	202E-03	253E-02	B4galt5	-17376	Hyper
chr14	7641553	7644047	Intron (NM_001170551/305164, intron 2 of 65)	0437	202E-03	253E-02	Wdfy3	34925	Hyper
chr10	79943995	79946950	Exon (NM_053474/84686, exon 3 of 10)	0454	202E-03	253E-02	Ppp1r9b	5929	Hyper
chr7	43807867	43808886	Distal Intergenic	0685	202E-03	254E-02	Pawr	162092	Hyper
chr2	219662386	219663744	Distal Intergenic	0644	203E-03	254E-02	Lef1	-4046	Hyper
chr5	147527085	147528705	Distal Intergenic	0462	203E-03	254E-02	Clic4	-13807	Hyper
chr20	7339116	7340150	Intron (NM_199379/294311, intron 4 of 8)	0702	204E-03	254E-02	RGD735065	16525	Hyper
chr2	31719146	31719603	Distal Intergenic	0769	204E-03	255E-02	Ocln	-12026	Hyper
chr10	87865090	87866247	Distal Intergenic	0584	204E-03	255E-02	Gfap	-3525	Hyper
chr20	18650465	18653679	Exon (NM_031805/361833, exon 35 of 45)	0412	204E-03	255E-02	Mrln	-228655	Hyper
chr20	42818482	42821163	Intron (NM_012755/25150, intron 2 of 13)	0391	204E-03	255E-02	Fyn	50423	Hyper
chr1	244768561	244769673	Distal Intergenic	0590	205E-03	255E-02	Hps6	-83583	Hyper
chr3	128561597	128563263	Distal Intergenic	0439	205E-03	255E-02	Flrt3	-553824	Hyper
chr14	103860042	103862367	Exon (NM_001013130/305614, exon 27 of 39)	0432	205E-03	255E-02	Sptbn1	146140	Hyper
chr9	1095737	1102678	Distal Intergenic	0303	205E-03	255E-02	Fem1a	4042	Hyper

chr8	23615436	23616235	Promoter (1-2kb)	0631	205E-03	255E-02	Herpud2	1639	Hyper
chr1	119226604	119228010	Exon (NM_001014020/308701, exon 6 of 13)	0527	206E-03	256E-02	Tars3	13375	Hyper
chr4	159386671	159396330	Distal Intergenic	0270	206E-03	256E-02	Kcna5	-29375	Hyper
chr6	129230362	129230909	Distal Intergenic	0836	206E-03	256E-02	Mir1247	53732	Hyper
chr12	8410253	8411160	Distal Intergenic	0684	206E-03	256E-02	Usp12	100176	Hyper
chr9	55644948	55645245	Distal Intergenic	1066	206E-03	256E-02	Hecw2	-64621	Hyper
chr7	113311164	113311599	Distal Intergenic	0962	206E-03	256E-02	Tef	-15112	Hyper
chr12	38180341	38181002	Distal Intergenic	0707	207E-03	257E-02	Spring1	148136	Hyper
chr3	16703621	16704385	Distal Intergenic	0695	207E-03	258E-02	Zbtb43	59430	Hyper
chr12	42738775	42741203	Promoter (<=1kb)	0360	208E-03	258E-02	Coro1c	0	Hyper
chr10	106252960	106259817	Promoter (<=1kb)	0268	208E-03	258E-02	Csnk1d	0	Hyper
chr12	21812490	21813358	Distal Intergenic	0648	209E-03	258E-02	Mettl27	-46171	Hyper
chr3	43063583	43066076	Distal Intergenic	0432	209E-03	259E-02	Acvr1	-16383	Hyper
chr5	162739649	162741149	Intron (NM_013214/26759, intron 5 of 8)	0473	209E-03	259E-02	Gpr153	-40438	Hyper
chr1	98966811	98971408	Intron (NM_138529/171563, intron 1 of 37)	0315	209E-03	259E-02	Nav2	8413	Hyper
chr1	90912313	90915889	Promoter (2-3kb)	0363	210E-03	259E-02	Plekhf1	-2206	Hyper
chr18	73716447	73716880	Intron (NM_001013189/361352, intron 2 of 4)	0888	210E-03	259E-02	Slc66a2	12451	Hyper
chr10	89196755	89198943	Exon (NM_017212/29477, exon 3 of 10)	0543	210E-03	260E-02	Mapt	58107	Hyper
chr9	77070444	77071527	Distal Intergenic	0561	210E-03	260E-02	Slc4a3	33428	Hyper
chr3	118282556	118283611	Promoter (<=1kb)	0642	210E-03	260E-02	Adam33	0	Hyper
chr19	51545315	51545586	Promoter (2-3kb)	1045	210E-03	260E-02	Dbndd1	2855	Hyper
chr4	174826403	174827944	Distal Intergenic	0542	210E-03	260E-02	Slco1a4	-61648	Hyper
chr1	256168028	256173923	Promoter (<=1kb)	0353	210E-03	260E-02	Ablim1	0	Hyper
chr15	49573205	49574285	Distal Intergenic	0595	210E-03	260E-02	Htr2a	-376911	Hyper
chr20	11387724	11388077	Distal Intergenic	0910	211E-03	260E-02	Pofut2	-9936	Hyper
chr6	130887513	130894038	3' UTR	0285	211E-03	260E-02	Zfyve21	13502	Hyper
chr3	157382579	157383345	Distal Intergenic	0637	211E-03	260E-02	Nfatc2	-68462	Hyper
chr3	124438998	124443583	Promoter (<=1kb)	0379	211E-03	261E-02	Jag1	0	Hyper
chr17	21793835	21794309	Intron (NM_214457/306844, intron 3 of 13)	0820	212E-03	261E-02	Phactr1	244931	Hyper
chr13	83526236	83527303	Promoter (<=1kb)	0392	212E-03	261E-02	Cfap126	0	Hyper
chr9	16771233	16772193	Intron (NM_053603/94272, intron 1 of 6)	0702	212E-03	261E-02	Clic5	41234	Hyper
chr2	52455700	52460520	Distal Intergenic	0321	212E-03	261E-02	Selenop	-37603	Hyper
chr14	78996726	78998980	Promoter (2-3kb)	0482	212E-03	261E-02	Sec14l2	-2986	Hyper

chr8	52320490	52321236	Distal Intergenic	0726	212E-03	261E-02	Fdx1	-33146	Hyper
chr6	105544413	105549661	Distal Intergenic	0294	212E-03	261E-02	Erg28	-61335	Hyper
chr14	86511401	86512321	Intron (NM_001109093/498416, intron 7 of 17)	0658	213E-03	261E-02	Ddc	-42226	Hyper
chr10	94556564	94557374	Intron (NM_001127297/303630, intron 4 of 12)	0734	213E-03	261E-02	Wipi1	22414	Hyper
chr18	4108134	4114099	Distal Intergenic	0307	213E-03	262E-02	Impact	-6031	Hyper
chr10	69233137	69235271	Intron (NM_022193/60581, intron 43 of 53)	0532	213E-03	262E-02	Acaca	150315	Hyper
chr6	28165354	28165846	Intron (NM_001106713/298867, intron 3 of 13)	0897	213E-03	262E-02	Klhl29	224801	Hyper
chr4	141797523	141799963	Promoter (<=1kb)	0359	214E-03	262E-02	Edem1	0	Hyper
chr8	20247651	20249579	Intron (NM_134368/171379, intron 29 of 33)	0497	214E-03	262E-02	Ldlr	-20517	Hyper
chr3	13464416	13465932	Exon (NM_001013164/311844, exon 3 of 13)	0631	214E-03	262E-02	Kyat1	7803	Hyper
chr5	109613525	109614189	Intron (NM_001105737/89804, intron 1 of 22)	0745	214E-03	262E-02	Tek	6448	Hyper
chr1	220690729	220691626	Distal Intergenic	0701	214E-03	262E-02	Klf9	-8482	Hyper
chr19	48239612	48240941	Distal Intergenic	0609	215E-03	263E-02	6430548M08Rikl	41403	Hyper
chr3	55654959	55655935	Distal Intergenic	0582	215E-03	263E-02	Tlk1	-42717	Hyper
chr18	58431283	58433672	Intron (NM_001008300/291553, intron 1 of 30)	0381	215E-03	263E-02	Nedd4l	36306	Hyper
chr6	130000271	130001802	Distal Intergenic	0584	215E-03	264E-02	Ankrd9	6633	Hyper
chr7	9415579	9417730	Promoter (<=1kb)	0334	215E-03	264E-02	Rps15	0	Hyper
chr10	101617358	101619379	Distal Intergenic	0465	215E-03	264E-02	Foxj1	-47128	Hyper
chr7	90831997	90833209	Distal Intergenic	0516	215E-03	264E-02	Sqle	-35533	Hyper
chr2	182867317	182869316	Promoter (1-2kb)	0564	215E-03	264E-02	Mindy1	1896	Hyper
chr8	37106781	37108737	Distal Intergenic	0449	216E-03	264E-02	Robo4	-11251	Hyper
chr14	76824949	76825631	Intron (NM_001008339/305455, intron 3 of 10)	0699	216E-03	265E-02	Nelfa	16029	Hyper
chr7	133375858	133377088	Promoter (2-3kb)	0537	216E-03	265E-02	Rarg	2128	Hyper
chr8	42803282	42803596	Intron (NM_001014089/315591, intron 7 of 7)	1208	216E-03	265E-02	Tecta	-23575	Hyper
chr9	75611014	75612017	Intron (NM_001191810/301509, intron 3 of 34)	0684	216E-03	265E-02	Tns1	90741	Hyper
chr15	124565	125712	Intron (NM_001372003/305645, intron 2 of 31)	0550	216E-03	265E-02	Dlg5	48779	Hyper
chr5	166776328	166777279	Distal Intergenic	0729	217E-03	265E-02	Agrn	-5725	Hyper
chr7	108464239	108467732	Exon (NM_173122/500901, exon 4 of 12)	0343	217E-03	265E-02	MGC94207	-18123	Hyper
chr17	32847897	32849087	Distal Intergenic	0623	217E-03	265E-02	Foxq1	66290	Hyper
chr3	160806122	160807306	Promoter (<=1kb)	0701	217E-03	265E-02	Cbln4	0	Hyper
chr16	44257449	44259661	Promoter (<=1kb)	0400	218E-03	266E-02	Wwc2	0	Hyper
chr20	11905758	11907619	Distal Intergenic	0531	218E-03	266E-02	Col6a2	-114148	Hyper
chr10	65298773	65301351	Promoter (<=1kb)	0367	218E-03	266E-02	Rhbdl3	0	Hyper

chr8	98924790	98926100	Intron (NM_001013224/363118, intron 1 of 4)	0651	218E-03	266E-02	Nmnat3	32319	Hyper
chr20	6749589	6752372	Promoter (<=1kb)	0285	219E-03	267E-02	Mapk14	0	Hyper
chr4	122964644	122970550	Promoter (<=1kb)	0295	219E-03	267E-02	Klf15	0	Hyper
chr3	7542388	7543775	Promoter (2-3kb)	0570	220E-03	268E-02	Cacna1b	2182	Hyper
chr5	139547594	139550244	Intron (NM_001301876/286923, intron 8 of 11)	0373	220E-03	268E-02	Smim12	-19220	Hyper
chr13	102129528	102130081	Distal Intergenic	0791	220E-03	268E-02	Rps6kc1	360217	Hyper
chr5	158648078	158649528	Distal Intergenic	0479	220E-03	268E-02	Fbxo2	55152	Hyper
chr20	27836924	27850240	3' UTR	0271	220E-03	268E-02	Dnajb12	8708	Hyper
chr5	116189734	116190706	Intron (NM_001107949/313409, intron 1 of 17)	0719	221E-03	269E-02	Dnajc6	59058	Hyper
chr7	11032315	11033922	Promoter (<=1kb)	0463	221E-03	269E-02	Syde1	0	Hyper
chr1	117464069	117465268	Distal Intergenic	0551	221E-03	269E-02	Klf13	71358	Hyper
chr1	254148745	254150067	Intron (NM_017274/29653, intron 1 of 20)	0566	221E-03	269E-02	Gpam	20494	Hyper
chr7	8833015	8834093	Promoter (1-2kb)	0602	222E-03	269E-02	Tmprss9	-1850	Hyper
chr4	141804978	141807271	Exon (NM_001305279/297504, exon 2 of 12)	0350	222E-03	270E-02	Edem1	7277	Hyper
chr16	4670985	4671883	Intron (NM_175595/306243, intron 3 of 36)	0668	222E-03	270E-02	Cacna2d3	240468	Hyper
chr1	155268793	155271960	Distal Intergenic	0383	222E-03	270E-02	Mir3102	-24901	Hyper
chr18	26618818	26619744	Distal Intergenic	0611	223E-03	270E-02	Hspa9	-64524	Hyper
chr5	150006616	150007054	Promoter (<=1kb)	0875	224E-03	271E-02	Alpl	-192	Hyper
chr13	22125930	22126823	Intron (NM_001271042/304695, intron 1 of 7)	0669	224E-03	271E-02	Zcchc2	6362	Hyper
chr2	73202723	73203544	Distal Intergenic	0611	224E-03	271E-02	Cdh18	-141461	Hyper
chr10	44490608	44492111	Exon (NM_001371051/116504, exon 3 of 25)	0482	224E-03	271E-02	Mprip	36418	Hyper
chr1	117085198	117086882	Distal Intergenic	0501	224E-03	271E-02	Chrna7	-247975	Hyper
chr6	92298670	92299084	Intron (NM_031085/81749, intron 1 of 13)	1047	224E-03	271E-02	Prkch	6670	Hyper
chr10	52608202	52611880	Promoter (<=1kb)	0360	224E-03	271E-02	Usp43	0	Hyper
chr10	36748118	36748592	Distal Intergenic	0929	224E-03	271E-02	Fstl4	-162828	Hyper
chr13	77973208	77974495	Distal Intergenic	0659	225E-03	272E-02	Rcsd1	-8899	Hyper
chr1	145606040	145607214	Intron (NM_022282/64053, intron 2 of 22)	0557	225E-03	272E-02	Dlg2	478865	Hyper
chr5	72548759	72550268	Distal Intergenic	0517	225E-03	272E-02	Akap2	-54561	Hyper
chr19	49975691	49978549	Intron (NM_019293/54233, intron 5 of 6)	0432	225E-03	272E-02	Slc7a5	-11868	Hyper
chr9	11504007	11504512	Distal Intergenic	0863	225E-03	272E-02	Mocs1	63278	Hyper
chr17	66499256	66500019	Distal Intergenic	0694	225E-03	272E-02	Calml3	77623	Hyper
chr16	6299548	6300841	Distal Intergenic	0548	225E-03	272E-02	Nt5dc2	-21644	Hyper
chr5	60304376	60305994	Intron (NM_138871/85425, intron 16 of 17)	0548	225E-03	272E-02	Tmod1	-10451	Hyper

chr17	27377752	27378201	Distal Intergenic	0920	225E-03	272E-02	Ly86	109059	Hyper
chr10	85985232	85991080	3' UTR	0277	225E-03	272E-02	Hsd17b1	-18648	Hyper
chr10	79076301	79077392	3' UTR	0573	226E-03	272E-02	Tob1	-85701	Hyper
chr18	16089855	16091536	Intron (NM_001271332/100360334, intron 3 of 26)	0387	226E-03	272E-02	Fhod3	96531	Hyper
chr6	105697725	105698913	Distal Intergenic	0630	226E-03	272E-02	Tgfb3	27062	Hyper
chr1	118226571	118228564	Intron (NM_031780/83610, intron 2 of 13)	0461	226E-03	272E-02	Apba2	123346	Hyper
chr12	9499965	9501560	Distal Intergenic	0548	226E-03	272E-02	Arpc1b	-4226	Hyper
chr7	110428836	110429604	Distal Intergenic	0720	226E-03	272E-02	Gga1	-9349	Hyper
chr19	47714255	47717429	Promoter (2-3kb)	0505	226E-03	272E-02	Wfdc1	-2999	Hyper
chr7	112126237	112127107	Intron (NM_001034944/366962, intron 1 of 7)	0612	226E-03	272E-02	Grap2	9095	Hyper
chr10	66537985	66539956	Intron (NM_001034014/25364, intron 1 of 9)	0371	227E-03	272E-02	Mir6327	58527	Hyper
chr17	1820738	1822505	Intron (NM_001012346/290963, intron 13 of 14)	0525	227E-03	273E-02	Mir23b	-6975	Hyper
chr6	119611580	119614321	Exon (NM_001108719/362768, exon 14 of 15)	0437	227E-03	273E-02	Ttc7b	90866	Hyper
chr1	181158067	181158833	Exon (NM_001006971/293485, exon 13 of 16)	0777	227E-03	273E-02	Eif3c	-5578	Hyper
chr10	82456229	82459853	Distal Intergenic	0472	227E-03	273E-02	Srcin1	108846	Hyper
chr9	98440790	98443917	3' UTR	0408	228E-03	273E-02	Macir	20030	Hyper
chr16	60957622	60959190	Distal Intergenic	0428	228E-03	273E-02	Fut10	-6237	Hyper
chr20	9584347	9585317	Distal Intergenic	0688	228E-03	273E-02	Wdr4	18085	Hyper
chr10	100950384	100952318	Promoter (1-2kb)	0388	228E-03	273E-02	Grb2	-1191	Hyper
chr8	60755402	60759877	Distal Intergenic	0298	228E-03	273E-02	Lrrc49	400818	Hyper
chr6	45323829	45324806	Distal Intergenic	0739	228E-03	273E-02	Adi1	16941	Hyper
chr17	45874288	45875084	Exon (NM_022217/60668, exon 2 of 21)	0559	228E-03	273E-02	Amph	107723	Hyper
chr6	29529206	29530196	Distal Intergenic	0599	228E-03	274E-02	Klhl29	-1138559	Hyper
chr19	39350369	39352965	Intron (NM_001135583/307855, intron 1 of 6)	0446	229E-03	274E-02	Fa2h	11188	Hyper
chr6	130917619	130921237	Intron (NM_001108062/314465, intron 3 of 15)	0388	229E-03	274E-02	Ppp1r13b	8412	Hyper
chr20	18189876	18191120	Distal Intergenic	0596	229E-03	274E-02	Phyhipl	158071	Hyper
chr2	138156349	138157579	Distal Intergenic	0572	229E-03	274E-02	Ufm1	-178729	Hyper
chr1	220583058	220584017	Distal Intergenic	0644	230E-03	275E-02	Klf9	-116091	Hyper
chr19	23512634	23513146	Distal Intergenic	0745	230E-03	275E-02	Ier2	18083	Hyper
chr11	80235579	80237416	Distal Intergenic	0369	231E-03	276E-02	Psmd2	21575	Hyper
chr10	75200828	75207209	Intron (NM_001100764/690286, intron 2 of 3)	0305	231E-03	276E-02	Hlf	4145	Hyper
chr1	50766730	50767387	Distal Intergenic	0852	231E-03	276E-02	Pabpc6	-277877	Hyper
chr12	31047170	31048210	Distal Intergenic	0639	232E-03	277E-02	Tmem132b	-48861	Hyper

chr2	26972988	26973434	Promoter (<=1kb)	0877	232E-03	277E-02	F2rl2	934	Hyper
chr8	106119855	106121479	Intron (NM_131907/170699, intron 2 of 27)	0518	233E-03	277E-02	Atp2c1	9835	Hyper
chr1	53295171	53295721	Intron (NM_001191619/292316, intron 1 of 1)	0800	233E-03	278E-02	Smok2a	14804	Hyper
chr10	94040592	94041326	Distal Intergenic	0610	233E-03	278E-02	Axin2	141347	Hyper
chr8	43318881	43320200	Distal Intergenic	0549	234E-03	279E-02	Grik4	-113804	Hyper
chr3	22769735	22772379	3' UTR	0343	234E-03	279E-02	Arpc5l	9837	Hyper
chr12	42130266	42132942	Distal Intergenic	0444	235E-03	280E-02	Mvk	25916	Hyper
chr10	35415513	35416799	Distal Intergenic	0430	236E-03	280E-02	Zfp354a	18744	Hyper
chr4	109530567	109530944	Intron (NM_001106598/297357, intron 9 of 18)	0827	236E-03	280E-02	LRRTM1	-170871	Hyper
chr10	47513293	47513757	Distal Intergenic	0752	236E-03	280E-02	Trim16	26972	Hyper
chr3	9427179	9428497	Promoter (<=1kb)	0630	236E-03	280E-02	Agpat2	0	Hyper
chr1	133419762	133420378	Distal Intergenic	0689	236E-03	280E-02	Mir9-3	-19671	Hyper
chr6	58073014	58073821	Distal Intergenic	0679	237E-03	281E-02	Lrrn3	446365	Hyper
chr17	32635517	32636737	Promoter (<=1kb)	0649	237E-03	281E-02	Foxc1	-156	Hyper
chr17	17438524	17440469	Exon (NM_001108881/364681, exon 6 of 8)	0557	237E-03	281E-02	Rnf144b	122291	Hyper
chr1	144385512	144388232	Promoter (<=1kb)	0350	237E-03	281E-02	Ccdc89	0	Hyper
chr14	43106469	43107175	Promoter (<=1kb)	0405	237E-03	281E-02	Wdr19	-181	Hyper
chr7	14594931	14596287	Promoter (<=1kb)	0601	237E-03	281E-02	Kank3	509	Hyper
chr18	77293387	77295605	Distal Intergenic	0430	237E-03	281E-02	Ptgr3	-158830	Hyper
chr10	78899859	78900978	Promoter (2-3kb)	0551	238E-03	282E-02	Nme2	2622	Hyper
chr16	73163781	73164939	Intron (NM_001037327/364634, intron 5 of 69)	0508	238E-03	282E-02	Csmd1	945016	Hyper
chr1	252947559	252949092	Distal Intergenic	0539	238E-03	282E-02	Shoc2	-10658	Hyper
chr1	8506120	8509162	Intron (NM_024137/29721, intron 2 of 8)	0375	238E-03	282E-02	Hivep2	146831	Hyper
chr12	43433911	43434962	Distal Intergenic	0756	239E-03	282E-02	Sgsm1	112655	Hyper
chr1	95081139	95085878	Promoter (<=1kb)	0284	239E-03	282E-02	Kcnc3	179	Hyper
chr15	38991716	38993543	Exon (NM_213626/305967, exon 11 of 47)	0613	239E-03	282E-02	Kif13b	83398	Hyper
chr4	79431587	79434661	Distal Intergenic	0428	239E-03	282E-02	Gsdme	-110783	Hyper
chr14	7792095	7793399	Intron (NM_001170551/305164, intron 56 of 65)	0546	239E-03	283E-02	Cds1	89338	Hyper
chr1	201341408	201343823	Promoter (1-2kb)	0449	239E-03	283E-02	Gstp1	-1178	Hyper
chr11	82267354	82268716	Distal Intergenic	0622	239E-03	283E-02	Cldn5	-53106	Hyper
chr6	95498164	95498660	Promoter (1-2kb)	0798	240E-03	283E-02	Gpx2	-1287	Hyper
chr20	6379183	6379832	Exon (NM_001191718/309643, exon 6 of 10)	0700	240E-03	283E-02	Fance	3609	Hyper
chr2	11384700	11385687	Distal Intergenic	0622	241E-03	284E-02	Arrdc3	247000	Hyper

chr10	82167114	82168301	Exon (NM_017063/24917, exon 5 of 23)	0629	241E-03	284E-02	Kpnb1	6136	Hyper
chr4	174824839	174826131	Distal Intergenic	0604	241E-03	284E-02	Slco1a4	-60084	Hyper
chr13	65885456	65889794	Promoter (<=1kb)	0343	242E-03	284E-02	Rgs16	0	Hyper
chr4	104580255	104580720	Distal Intergenic	0903	242E-03	285E-02	Capg	-19342	Hyper
chr1	117535185	117551491	Promoter (<=1kb)	0273	243E-03	285E-02	Klf13	0	Hyper
chr11	34181332	34182966	Intron (NM_013192/25743, intron 2 of 5)	0487	243E-03	286E-02	Kcnj6	125792	Hyper
chr4	52744950	52745508	Distal Intergenic	0697	243E-03	286E-02	Slc13a1	-97847	Hyper
chr15	28067137	28069211	Promoter (2-3kb)	0436	244E-03	286E-02	RGD1565222	-2863	Hyper
chr8	50214677	50216210	Distal Intergenic	0560	244E-03	286E-02	Ncam1	-49140	Hyper
chr19	48495368	48495888	Distal Intergenic	0886	244E-03	286E-02	Gins2	143451	Hyper
chr16	9705970	9706488	Promoter (<=1kb)	0906	244E-03	286E-02	Mmrn2	17	Hyper
chr7	131287099	131292798	Distal Intergenic	0323	245E-03	287E-02	Atf1	-69164	Hyper
chr18	16240775	16242092	Intron (NM_001271332/100360334, intron 5 of 26)	0428	245E-03	287E-02	Fhod3	247451	Hyper
chr7	111498937	111499380	Distal Intergenic	0954	245E-03	287E-02	Cbx7	-20964	Hyper
chr17	57456510	57458615	Intron (NM_001191833/361261, intron 1 of 9)	0476	245E-03	287E-02	Ccny	54245	Hyper
chr6	68009435	68010125	Intron (NM_001276715/85421, intron 1 of 18)	0744	245E-03	287E-02	Prkd1	28572	Hyper
chr14	76356803	76357154	Distal Intergenic	0871	246E-03	287E-02	Rnf4	65410	Hyper
chr10	83680098	83683665	Intron (NM_001034079/619436, intron 3 of 25)	0316	246E-03	287E-02	Med24	6458	Hyper
chrX	115781355	115782344	Distal Intergenic	0682	246E-03	288E-02	Pgrmc1	-50540	Hyper
chr4	155940555	155942129	Promoter (1-2kb)	0492	246E-03	288E-02	Nanog	-1608	Hyper
chr8	103900789	103901538	Distal Intergenic	0716	246E-03	288E-02	Inhca	-42828	Hyper
chr6	137263555	137267730	Intron (NM_001271169/299488, intron 6 of 21)	0338	246E-03	288E-02	Esyt2	43848	Hyper
chr19	49459549	49460787	Distal Intergenic	0596	247E-03	288E-02	Fbxo31	195223	Hyper
chr1	119085748	119087531	Distal Intergenic	0479	247E-03	289E-02	Tars3	-125698	Hyper
chr20	28026922	28027930	Intron (NM_001007632/294512, intron 9 of 10)	0633	247E-03	289E-02	Spock2	-9662	Hyper
chr3	47000180	47001309	Intron (NM_012789/25253, intron 10 of 27)	0610	248E-03	289E-02	Dpp4	42561	Hyper
chr10	36453506	36454247	Distal Intergenic	0744	248E-03	289E-02	Skp1	51519	Hyper
chr1	69056874	69058460	Promoter (2-3kb)	0354	248E-03	289E-02	Rpl28	-2165	Hyper
chr5	75968318	75969514	Promoter (2-3kb)	0584	248E-03	290E-02	Alad	2820	Hyper
chr4	174759837	174765444	Promoter (<=1kb)	0443	249E-03	290E-02	Slco1a4	0	Hyper
chr7	112190894	112193254	Downstream (<=300bp)	0340	249E-03	290E-02	Fam83f	-17910	Hyper
chr4	144446045	144447918	Intron (NM_031040/81672, intron 8 of 9)	0469	250E-03	291E-02	Grm7	715183	Hyper
chr6	27686995	27687880	Distal Intergenic	0623	250E-03	291E-02	Fam228a	62181	Hyper

chr3	119024832	119027042	Distal Intergenic	0425	250E-03	291E-02	Prnp	-159126	Hyper
chr15	17086128	17087797	Intron (NM_001107288/306204, intron 1 of 44)	0502	250E-03	291E-02	Flnb	7209	Hyper
chr2	182625892	182630993	3' UTR	0330	251E-03	292E-02	Psmd4	-17698	Hyper
chr13	44499696	44505806	Intron (NM_001105951/289021, intron 1 of 31)	0324	251E-03	292E-02	Pik3c2b	4646	Hyper
chr7	130535914	130537477	Intron (NM_019381/24822, intron 3 of 9)	0552	251E-03	292E-02	Tmbim6	8601	Hyper
chr2	137527160	137529447	Distal Intergenic	0426	252E-03	292E-02	Proser1	-12931	Hyper
chr9	102883427	102889328	Intron (NM_001108235/316663, intron 7 of 8)	0298	252E-03	292E-02	Efna5	-288293	Hyper
chr16	3105873	3107647	Intron (NM_170787/259269, intron 4 of 16)	0455	252E-03	292E-02	Erc2	257195	Hyper
chr11	30055214	30056298	Exon (NM_001191661/304104, exon 5 of 39)	0740	252E-03	293E-02	Urb1	8929	Hyper
chr10	35068127	35068964	Intron (NM_001137622/287899, intron 4 of 20)	0610	252E-03	293E-02	Zfp354c	75438	Hyper
chr12	11148203	11149698	Intron (NM_001013122/304290, intron 1 of 4)	0592	253E-03	293E-02	Kdelr2	7419	Hyper
chr2	61046823	61048520	Distal Intergenic	0415	253E-03	293E-02	Sub1	-26451	Hyper
chr5	142511669	142512456	Distal Intergenic	0686	253E-03	293E-02	Tinagl1	10634	Hyper
chr3	106400281	106401336	Intron (NM_001261404/296084, intron 25 of 34)	0617	253E-03	293E-02	Ino80	47028	Hyper
chr4	9602149	9602798	Distal Intergenic	0806	253E-03	293E-02	Cct8l2	4173	Hyper
chr5	161336480	161337153	3' UTR	0732	254E-03	293E-02	Errfi1	12475	Hyper
chr6	121752165	121753319	Intron (NM_001191985/500709, intron 3 of 9)	0503	254E-03	294E-02	Chga	56114	Hyper
chr7	111641764	111647260	Intron (NM_019166/29205, intron 1 of 3)	0299	254E-03	294E-02	Syngr1	5846	Hyper
chr7	128874128	128874893	Distal Intergenic	0694	255E-03	294E-02	Endou	-7586	Hyper
chr13	84740883	84744390	Exon (NM_012505/24212, exon 8 of 23)	0423	255E-03	295E-02	Atp1a2	10154	Hyper
chr6	31349401	31349983	Distal Intergenic	0903	256E-03	295E-02	Rhob	15940	Hyper
chr5	62941812	62942922	Exon (NM_023020/63845, exon 5 of 10)	0595	256E-03	295E-02	Tmeff1	31072	Hyper
chr9	32977263	32978762	Distal Intergenic	0565	256E-03	296E-02	Phf3	157232	Hyper
chr5	163155468	163156293	Distal Intergenic	0702	257E-03	296E-02	Nphp4	167098	Hyper
chr1	53192682	53193589	Distal Intergenic	0621	257E-03	296E-02	Smok2a	-86778	Hyper
chr4	157057241	157058033	Distal Intergenic	0642	257E-03	296E-02	Cd163	-27060	Hyper
chr1	246204575	246205132	Intron (NM_001107606/309460, intron 7 of 13)	0759	257E-03	296E-02	Sh3pxd2a	112666	Hyper
chr10	44566476	44570610	3' UTR	0345	257E-03	296E-02	Flcn	37139	Hyper
chr10	44501660	44508626	Intron (NM_001371051/116504, intron 3 of 24)	0307	258E-03	297E-02	Mprip	47470	Hyper
chr3	56897614	56899632	Intron (NM_001100642/252857, intron 4 of 30)	0472	258E-03	297E-02	Rapgef4	88203	Hyper
chr5	8249261	8250535	Distal Intergenic	0501	259E-03	297E-02	Prex2	-62461	Hyper
chr16	21999638	22001997	Distal Intergenic	0380	259E-03	297E-02	Nat3	149306	Hyper
chr3	167555812	167556691	Promoter (2-3kb)	0665	259E-03	297E-02	Slco4a1	-2625	Hyper

chr4	118262136	118263414	Intron (NM_080884/114020, intron 1 of 1)	0592	259E-03	298E-02	Nat8f3	-16126	Hyper
chr1	173276693	173278268	Distal Intergenic	0461	259E-03	298E-02	Gprc5b	62665	Hyper
chr19	24401820	24402808	Promoter (2-3kb)	0608	259E-03	298E-02	Adgre5	2905	Hyper
chr3	156397333	156401571	Promoter (<=1kb)	0318	260E-03	298E-02	Cebpb	0	Hyper
chr17	1832166	1836793	Intron (NM_001012346/290963, intron 12 of 14)	0371	260E-03	298E-02	Mir23b	-18403	Hyper
chr5	103013765	103014823	Distal Intergenic	0502	260E-03	298E-02	Ifnb1	6700	Hyper
chr15	13406424	13407626	Intron (NM_134356/171357, intron 1 of 32)	0569	260E-03	298E-02	Ptprg	144560	Hyper
chr5	28532076	28532484	Intron (NM_022302/64169, intron 3 of 14)	0726	260E-03	298E-02	Necab1	46158	Hyper
chr5	165742535	165744495	Distal Intergenic	0476	260E-03	299E-02	Faap20	-64162	Hyper
chr6	102127465	102128940	Distal Intergenic	0447	261E-03	299E-02	Sipa1l1	121285	Hyper
chr14	81128377	81130244	Promoter (<=1kb)	0359	261E-03	299E-02	Tmed4	0	Hyper
chr5	46024989	46026286	Distal Intergenic	0502	261E-03	299E-02	Map3k7	-331645	Hyper
chr1	134731466	134733189	Intron (NM_001108489/361598, intron 5 of 37)	0505	262E-03	300E-02	Iqgap1	36566	Hyper
chr19	13552244	13553771	Distal Intergenic	0438	262E-03	300E-02	Rasd2	-40520	Hyper
chr11	66236224	66238939	Distal Intergenic	0405	262E-03	300E-02	Kalrn	-16030	Hyper
chr17	64564651	64565394	Distal Intergenic	0663	263E-03	300E-02	Klf6	-16200	Hyper
chr17	7077269	7079050	Intron (NM_001271297/306759, intron 4 of 10)	0582	263E-03	301E-02	Spock1	335756	Hyper
chr5	151453474	151454228	Intron (NM_001005903/298584, intron 1 of 8)	0817	263E-03	301E-02	Capzb	17770	Hyper
chr20	10220721	10221886	Intron (NM_031769/83578, intron 1 of 10)	0554	264E-03	302E-02	Pdxk	10328	Hyper
chr6	121773690	121781685	Intron (NM_001191985/500709, intron 2 of 9)	0288	264E-03	302E-02	Itpk1	62353	Hyper
chr13	94198322	94201984	Promoter (<=1kb)	0293	264E-03	302E-02	Capn2	0	Hyper
chr1	175620193	175622161	Promoter (<=1kb)	0378	265E-03	303E-02	Igsf6	0	Hyper
chr1	90904669	90910328	Promoter (<=1kb)	0322	265E-03	303E-02	Plekhf1	0	Hyper
chr4	77694412	77695890	Promoter (<=1kb)	0647	266E-03	303E-02	Gimap5	79	Hyper
chr8	84937184	84938713	Intron (NM_019267/29711, intron 8 of 10)	0592	266E-03	303E-02	Bckdhb	91920	Hyper
chr1	239476885	239479385	Intron (NM_022587/64519, intron 1 of 9)	0433	266E-03	303E-02	Entpd1	3443	Hyper
chr6	88802398	88804369	Exon (NM_130420/155812, exon 2 of 10)	0401	266E-03	303E-02	Trim9	52173	Hyper
chr19	51525863	51526294	Exon (NM_001108456/361436, exon 9 of 17)	0843	266E-03	303E-02	Afg3l1	17251	Hyper
chr20	18681427	18682018	Intron (NM_031805/361833, intron 25 of 44)	0782	266E-03	303E-02	Ank3	248111	Hyper
chr17	42479118	42479615	Promoter (<=1kb)	0748	266E-03	303E-02	Hist1h4b	-852	Hyper
chr7	579221	579793	Intron (NM_001025403/288771, intron 2 of 11)	0865	266E-03	304E-02	Rbms2	20341	Hyper
chr10	90974058	90975743	Promoter (<=1kb)	0346	267E-03	304E-02	Dcaf7	0	Hyper
chr5	162773158	162775896	Intron (NM_013214/26759, intron 8 of 8)	0327	267E-03	304E-02	Gpr153	-5691	Hyper

chr17	53286327	53286833	Distal Intergenic	0889	267E-03	304E-02	Mtpap	54648	Hyper
chr2	175343763	175348749	Promoter (<=1kb)	0296	267E-03	304E-02	Il6r	0	Hyper
chr1	121715741	121717247	Intron (NM_052807/25718, intron 2 of 20)	0572	267E-03	304E-02	Igf1r	165000	Hyper
chr10	54489299	54490007	Intron (NM_001304352/287441, intron 1 of 3)	0703	268E-03	304E-02	Zbtb4	3736	Hyper
chr20	13422526	13423830	Intron (NM_001039455/361828, intron 2 of 15)	0587	269E-03	306E-02	Specc1l	19835	Hyper
chr19	23971876	23973645	Distal Intergenic	0477	269E-03	306E-02	Mir181c	-9878	Hyper
chr11	67200879	67201602	Intron (NM_153625/266733, intron 4 of 12)	0765	269E-03	306E-02	Slc12a8	64202	Hyper
chr5	154641298	154643762	Intron (NM_001014070/313672, intron 1 of 7)	0398	269E-03	306E-02	Kazn	135147	Hyper
chr7	70807076	70810573	Distal Intergenic	0336	270E-03	307E-02	Rims2	84713	Hyper
chr4	181294084	181296348	Distal Intergenic	0453	270E-03	307E-02	Rps4y2	24577	Hyper
chr7	89409934	89410987	Exon (NM_001014202/362912, exon 7 of 8)	0530	270E-03	307E-02	Derl1	16108	Hyper
chr1	121868158	121869091	Distal Intergenic	0635	270E-03	307E-02	Igf1r	317417	Hyper
chr18	54439824	54440884	Distal Intergenic	0675	271E-03	308E-02	Slc6a7	26435	Hyper
chr18	39190593	39191551	Promoter (<=1kb)	0494	272E-03	308E-02	Mospd4	0	Hyper
chr5	151513068	151514620	Intron (NM_001005903/298584, intron 4 of 8)	0556	272E-03	308E-02	Slc66a1	35205	Hyper
chr1	226753680	226754102	Intron (NM_013218/26956, intron 2 of 4)	0871	272E-03	308E-02	Ak3	10466	Hyper
chr16	45237277	45238455	Intron (NM_001107311/306460, intron 2 of 7)	0565	272E-03	308E-02	Enpp6	55622	Hyper
chr20	10183915	10187575	Distal Intergenic	0303	272E-03	308E-02	Pdxk	-22818	Hyper
chr6	106576964	106577631	Distal Intergenic	0727	272E-03	308E-02	Cipc	-17388	Hyper
chr13	46081888	46082773	Distal Intergenic	0656	273E-03	308E-02	Kdm5b	79346	Hyper
chr6	121788760	121790599	Intron (NM_001191985/500709, intron 2 of 9)	0505	273E-03	309E-02	Itpk1	53439	Hyper
chr5	148342417	148343325	Distal Intergenic	0591	273E-03	309E-02	Id3	-29459	Hyper
chr6	10711600	10711931	Distal Intergenic	0996	273E-03	309E-02	Haao	-133854	Hyper
chr7	130648087	130652070	Intron (NM_144756/246274, intron 5 of 11)	0400	274E-03	310E-02	Faim2	7098	Hyper
chr12	35849110	35851726	Distal Intergenic	0307	275E-03	310E-02	Oas2	42351	Hyper
chr3	53684726	53685040	Distal Intergenic	0942	275E-03	310E-02	Nostrin	-219787	Hyper
chr9	39288162	39288756	Distal Intergenic	0758	276E-03	311E-02	Cnga3	-182251	Hyper
chr1	175225650	175227417	Distal Intergenic	0571	276E-03	311E-02	Pdzd9	-16461	Hyper
chr5	71704749	71706595	Distal Intergenic	0461	276E-03	311E-02	Mir32	-108597	Hyper
chr1	175423336	175426427	Intron (NM_012947/25435, intron 1 of 16)	0370	276E-03	311E-02	Eef2k	11718	Hyper
chr17	7123617	7126082	Intron (NM_001271297/306759, intron 5 of 10)	0392	276E-03	311E-02	Spock1	382104	Hyper
chr5	130678961	130681714	Distal Intergenic	0381	276E-03	311E-02	Kif2c	-16324	Hyper
chr6	104422774	104423496	Distal Intergenic	0673	276E-03	311E-02	Isca2	4265	Hyper

chr10	17249167	17250731	Intron (NM_001106993/303024, intron 1 of 11)	0470	277E-03	312E-02	Fbxw11	16388	Hyper
chr6	119915780	119916721	Intron (NM_001108048/314384, intron 3 of 15)	0575	277E-03	312E-02	Rps6ka5	89423	Hyper
chr5	146972822	146979915	Promoter (<=1kb)	0319	277E-03	312E-02	Ldlrap1	0	Hyper
chr10	63979237	63980009	Intron (NM_001382488/108348076, intron 9 of 17)	0694	277E-03	312E-02	Lgals9	-49192	Hyper
chr5	57780396	57781542	Promoter (<=1kb)	0548	278E-03	312E-02	Tpm2	-458	Hyper
chr7	65565041	65566105	Distal Intergenic	0574	278E-03	313E-02	Rpl30	85258	Hyper
chr1	166415420	166419367	Intron (NM_001139508/365352, intron 1 of 17)	0304	278E-03	313E-02	Mical2	24401	Hyper
chr2	35180146	35181990	Exon (NM_053970/117041, exon 4 of 13)	0426	279E-03	314E-02	Nln	50786	Hyper
chr20	6086869	6087661	Intron (NM_001107613/309639, intron 11 of 23)	0729	280E-03	315E-02	Tcp11	48250	Hyper
chr20	8063000	8063421	Distal Intergenic	0876	280E-03	315E-02	Zfand3	-19986	Hyper
chr6	45326618	45329838	Distal Intergenic	0427	280E-03	315E-02	Adi1	19730	Hyper
chr18	63402464	63403078	Distal Intergenic	0705	281E-03	315E-02	Ccdc68	-138818	Hyper
chr15	39749287	39750769	Distal Intergenic	0663	281E-03	315E-02	Pnoc	-111787	Hyper
chr5	160549938	160551469	Promoter (<=1kb)	0395	281E-03	315E-02	Gpr157	0	Hyper
chr17	60629001	60630067	3' UTR	0650	281E-03	315E-02	Zmynd11	50639	Hyper
chr6	121804691	121806015	Intron (NM_001191985/500709, intron 2 of 9)	0574	281E-03	315E-02	Itpk1	38023	Hyper
chr7	69951397	69967729	Intron (NM_144762/140720, intron 1 of 2)	0257	281E-03	316E-02	Baalc	32399	Hyper
chr7	27812390	27812816	Distal Intergenic	0971	282E-03	316E-02	Elk3	-8306	Hyper
chr10	60495271	60497665	3' UTR	0452	282E-03	316E-02	Myo1c	-5994	Hyper
chr3	15859835	15861759	Distal Intergenic	0392	282E-03	316E-02	Dpm2	3558	Hyper
chr10	35416972	35418396	Distal Intergenic	0523	282E-03	316E-02	Zfp354a	20203	Hyper
chr7	63350728	63352464	Downstream (<=300bp)	0400	282E-03	316E-02	Ndufa4l2	-4419	Hyper
chr7	10170254	10172146	Distal Intergenic	0511	283E-03	317E-02	Plpp2	-3804	Hyper
chr4	1857880	1858686	Distal Intergenic	0589	283E-03	317E-02	Il6	3360492	Hyper
chr7	132244049	132244809	Promoter (<=1kb)	0729	283E-03	317E-02	Acvrl1	-702	Hyper
chr14	96829631	96833890	Promoter (<=1kb)	0363	283E-03	317E-02	B3gnt2	0	Hyper
chr12	17032846	17033810	Distal Intergenic	0391	283E-03	317E-02	Cops6	-5169	Hyper
chr3	18809459	18811111	Promoter (<=1kb)	0465	284E-03	317E-02	Ggta1	-339	Hyper
chr15	96065229	96067665	Downstream (<=300bp)	0389	284E-03	317E-02	Dnajc3	39500	Hyper
chr4	158329899	158331351	Distal Intergenic	0534	284E-03	318E-02	Vwf	-28801	Hyper
chr11	76334383	76336782	Distal Intergenic	0361	285E-03	318E-02	Lpp	-33818	Hyper
chr16	50349302	50353069	Distal Intergenic	0373	285E-03	318E-02	Frg1	-572734	Hyper
chr2	76449263	76450924	Intron (NM_001034912/619558, intron 3 of 8)	0492	285E-03	319E-02	Zfp622	-29627	Hyper

chr6	121098540	121100920	Exon (NM_021702/60331, exon 2 of 11)	0384	285E-03	319E-02	Atxn3	6982	Hyper
chr1	196419699	196420246	Intron (NM_031801/83632, intron 9 of 11)	0810	286E-03	319E-02	Tmem80	-15129	Hyper
chr14	86894728	86898680	Intron (NM_001107236/305497, intron 7 of 10)	0336	286E-03	319E-02	Cobl	80859	Hyper
chr3	112599915	112602207	Exon (NM_031825/83727, exon 35 of 65)	0514	286E-03	319E-02	Dut	100457	Hyper
chr10	72875073	72876052	3' UTR	0642	286E-03	319E-02	Vezf1	15169	Hyper
chr3	102638170	102638690	Distal Intergenic	0793	286E-03	319E-02	Meis2	304807	Hyper
chr16	44397305	44398114	Intron (NM_001109111/498630, intron 16 of 22)	0583	286E-03	319E-02	Cldn22	25240	Hyper
chr3	131081631	131083068	Intron (NM_012746/25121, intron 5 of 11)	0516	287E-03	320E-02	Bfsp1	146269	Hyper
chr1	151030101	151031089	Intron (NM_001191628/308831, intron 4 of 31)	0727	287E-03	320E-02	Tenm4	249720	Hyper
chr4	60259717	60260009	Distal Intergenic	1113	287E-03	320E-02	Podxl	-77888	Hyper
chr8	43899032	43899881	Distal Intergenic	0657	288E-03	320E-02	Nectin1	-201895	Hyper
chr8	108383219	108387008	Promoter (<=1kb)	0377	288E-03	320E-02	Sema3f	75	Hyper
chr7	17527177	17531192	Intron (NM_017109/29130, intron 5 of 12)	0300	288E-03	320E-02	Timp3	40647	Hyper
chr16	10481544	10483048	Intron (NM_024378/79219, intron 4 of 16)	0406	288E-03	321E-02	Mir346	263426	Hyper
chr19	48559838	48561948	Distal Intergenic	0556	288E-03	321E-02	Gins2	77391	Hyper
chr10	14928088	14932046	Intron (NM_182675/359728, intron 1 of 5)	0330	289E-03	321E-02	Rab40c	3903	Hyper
chr7	28894100	28895853	Distal Intergenic	0395	289E-03	321E-02	Ndufa12	122770	Hyper
chr11	29945229	29945553	Distal Intergenic	0895	289E-03	321E-02	Mis18a	35505	Hyper
chr17	81595372	81596952	Intron (NM_053926/116723, intron 1 of 9)	0514	289E-03	321E-02	Pip4k2a	71077	Hyper
chr12	12144874	12146197	Intron (NM_001037219/304298, intron 1 of 8)	0654	289E-03	321E-02	Foxk1	28892	Hyper
chr20	6470012	6470373	Exon (NM_001012174/361810, exon 7 of 11)	0899	289E-03	321E-02	Tulp1	-45939	Hyper
chr1	85206856	85208188	Intron (NM_144749/246264, intron 1 of 5)	0373	289E-03	321E-02	Zfp382	18878	Hyper
chr1	82514388	82515865	Intron (NM_178094/308451, intron 1 of 6)	0591	289E-03	321E-02	Itpkc	6668	Hyper
chr5	146886123	146887652	Intron (NM_001108687/362625, intron 1 of 11)	0551	290E-03	322E-02	Man1c1	25605	Hyper
chr5	142922067	142923487	Exon (NM_001108684/362609, exon 13 of 22)	0541	290E-03	322E-02	Sdc3	-42172	Hyper
chr6	98689067	98690506	Distal Intergenic	0498	292E-03	323E-02	Zfp36l1	245242	Hyper
chr12	43435468	43436339	Distal Intergenic	0700	292E-03	323E-02	Sgsm1	114212	Hyper
chr3	13334174	13336280	Promoter (<=1kb)	0401	292E-03	323E-02	Pkn3	0	Hyper
chr12	40910581	40912106	Intron (NM_001191667/304537, intron 2 of 8)	0492	292E-03	323E-02	Bicdl1	32283	Hyper
chr3	109682351	109682926	Distal Intergenic	0699	292E-03	323E-02	Gatm	-6843	Hyper
chr4	159617452	159617937	Distal Intergenic	0822	292E-03	324E-02	Kcna6	-41263	Hyper
chr14	103597366	103597754	Distal Intergenic	0912	292E-03	324E-02	Rtn4	147176	Hyper
chr4	118657297	118657731	Intron (NM_012671/24827, intron 2 of 5)	0815	293E-03	324E-02	Tgfa	39028	Hyper

chr10	61260611	61268851	3' UTR	0290	293E-03	324E-02	Timm22	7152	Hyper
chr5	151447882	151450370	Intron (NM_001005903/298584, intron 1 of 8)	0411	293E-03	324E-02	Capzb	12178	Hyper
chr1	245513624	245518590	5' UTR	0354	293E-03	324E-02	Wbp1l	20125	Hyper
chr9	87956582	87958271	Intron (NM_001109310/501181, intron 1 of 3)	0551	293E-03	324E-02	Efhd1	18298	Hyper
chr13	79450734	79451453	Distal Intergenic	0524	293E-03	324E-02	Tmco1	-8776	Hyper
chr1	213434220	213436419	Intron (NM_031036/81666, intron 1 of 6)	0414	294E-03	324E-02	Gnaq	8494	Hyper
chr1	119138475	119139251	Distal Intergenic	0596	295E-03	325E-02	Tars3	-73978	Hyper
chr5	166377670	166388062	Promoter (<=1kb)	0269	295E-03	325E-02	Vwa1	0	Hyper
chr17	15690820	15691865	Intron (NM_001191816/291019, intron 1 of 17)	0545	295E-03	325E-02	Fam120a	6587	Hyper
chr20	18764248	18766012	Intron (NM_031805/361833, intron 8 of 44)	0503	295E-03	325E-02	Ank3	164117	Hyper
chr9	13241084	13242580	Distal Intergenic	0611	295E-03	325E-02	Pgc	23102	Hyper
chr5	160141175	160141539	Distal Intergenic	0945	295E-03	325E-02	Pik3cd	-20245	Hyper
chr15	39315398	39317671	Promoter (1-2kb)	0489	295E-03	326E-02	Extl3	1801	Hyper
chr10	102298752	102300382	Distal Intergenic	0397	296E-03	326E-02	Sec14l1	-19538	Hyper
chr6	29602749	29604939	Distal Intergenic	0376	297E-03	327E-02	Klhl29	-1212102	Hyper
chr18	26580324	26581295	Distal Intergenic	0595	297E-03	327E-02	Hspa9	-26030	Hyper
chr12	4528502	4529109	Intron (NM_001170398/304244, intron 51 of 60)	0856	298E-03	328E-02	Tgap1-ps1	-179082	Hyper
chr10	88727083	88727370	Distal Intergenic	1040	298E-03	328E-02	Wnt3	46835	Hyper
chr2	100510361	100511232	Intron (NM_019138/25429, intron 4 of 5)	0779	298E-03	328E-02	Bhlhe22	23961	Hyper
chr15	28553272	28554056	Intron (NM_001098803/305888, intron 1 of 9)	0739	298E-03	328E-02	Zfhx2	11072	Hyper
chr1	245595846	245597231	Promoter (<=1kb)	0434	299E-03	329E-02	As3mt	0	Hyper
chr12	20193233	20195083	Intron (NM_053860/116639, intron 2 of 22)	0480	299E-03	329E-02	Cux1	85922	Hyper
chr8	61121500	61125452	Exon (NM_001134469/300763, exon 8 of 17)	0295	299E-03	329E-02	Lrrc49	35243	Hyper
chr7	35074760	35075210	Distal Intergenic	0715	299E-03	329E-02	Kitlg	178685	Hyper
chr3	141201772	141203307	Distal Intergenic	0644	300E-03	329E-02	Id1	-7986	Hyper
chr2	184171014	184173158	Promoter (<=1kb)	0352	300E-03	329E-02	Pex11b	0	Hyper
chr5	96088565	96089936	Distal Intergenic	0517	300E-03	329E-02	Mpdz	-168066	Hyper
chr8	71626668	71627606	Intron (NM_012597/24538, intron 1 of 8)	0612	300E-03	329E-02	Lipc	7858	Hyper
chr1	202703626	202704512	Intron (NM_001004278/361707, intron 5 of 7)	0565	300E-03	329E-02	Sart1	-4490	Hyper
chr2	133844645	133845921	Promoter (<=1kb)	0520	301E-03	330E-02	Pcdh18	0	Hyper
chr3	16610492	16613123	Distal Intergenic	0411	301E-03	330E-02	Angptl2	93307	Hyper
chr15	7004797	7006144	Intron (NM_001108371/361013, intron 1 of 3)	0526	301E-03	330E-02	Ube2e2	18533	Hyper
chr8	61034129	61034965	Distal Intergenic	0647	301E-03	330E-02	Lrrc49	125730	Hyper

chr15	7148055	7148736	Intron (NM_001108371/361013, intron 1 of 3)	0806	301E-03	330E-02	Ube2e2	161791	Hyper
chr10	17251645	17253702	Intron (NM_001106993/303024, intron 1 of 11)	0439	302E-03	330E-02	Fbxw11	18866	Hyper
chr11	37288701	37290111	Promoter (<=1kb)	0486	302E-03	330E-02	C2cd2	0	Hyper
chr4	76103097	76103441	Distal Intergenic	0864	302E-03	330E-02	Cul1	-448552	Hyper
chr12	31544403	31546889	Intron (NM_001108334/360801, intron 7 of 46)	0440	302E-03	330E-02	Ncor2	77815	Hyper
chr5	144306050	144307161	3' UTR	0460	302E-03	331E-02	Oprd1	33799	Hyper
chr12	31366745	31367770	Distal Intergenic	0538	302E-03	331E-02	Scarb1	70589	Hyper
chr15	39286108	39286960	Distal Intergenic	0658	303E-03	331E-02	Extl3	32512	Hyper
chr3	10738012	10739374	Intron (NM_001106563/296603, intron 1 of 27)	0571	303E-03	331E-02	Vav2	14678	Hyper
chr9	79762729	79765210	Promoter (<=1kb)	0373	303E-03	331E-02	Sgpp2	0	Hyper
chr12	34271384	34272352	Intron (NM_001107141/304488, intron 1 of 5)	0636	304E-03	332E-02	Pptc7	15987	Hyper
chr10	101001897	101003631	Distal Intergenic	0510	304E-03	332E-02	Mir3577	-20238	Hyper
chr9	32879052	32880982	Distal Intergenic	0393	304E-03	332E-02	Phf3	255012	Hyper
chr13	103177688	103178687	Intron (NM_001191675/289382, intron 9 of 30)	0595	304E-03	332E-02	Ints7	22565	Hyper
chr10	72952771	72954237	Intron (NM_001013971/303419, intron 1 of 10)	0560	304E-03	332E-02	Mrps23	-44796	Hyper
chr20	3217574	3218345	Promoter (<=1kb)	0590	304E-03	332E-02	Cchcr1	0	Hyper
chr12	41797298	41798015	Distal Intergenic	0755	305E-03	332E-02	Ankrd13a	29929	Hyper
chr16	1086643	1088520	Intron (NM_001108393/361103, intron 2 of 23)	0434	305E-03	333E-02	Mir3075	-12543	Hyper
chr7	119939040	119950717	Promoter (2-3kb)	0302	305E-03	333E-02	Pim3	-2660	Hyper
chr4	11077206	11078231	Distal Intergenic	0585	306E-03	333E-02	Klhl7	-21807	Hyper
chr13	65169313	65173318	Intron (NM_001048042/289095, intron 1 of 10)	0280	306E-03	334E-02	Nmnat2	63363	Hyper
chr6	123768340	123769601	Intron (NM_001106755/299285, intron 1 of 12)	0599	307E-03	334E-02	Clmn	37671	Hyper
chr5	74517493	74519380	Distal Intergenic	0496	307E-03	334E-02	Hsdl2	73560	Hyper
chr5	75939959	75942235	Exon (NM_022261/64027, exon 4 of 6)	0462	307E-03	334E-02	Hdhd3	12540	Hyper
chr4	117675009	117676286	Promoter (<=1kb)	0399	307E-03	335E-02	Spr	0	Hyper
chr11	76147478	76148539	Intron (NM_001013864/288010, intron 4 of 11)	0648	308E-03	335E-02	Lpp	152026	Hyper
chr11	80745722	80749900	Exon (NM_001109057/498112, exon 26 of 30)	0364	308E-03	335E-02	Map6d1	11574	Hyper
chr2	164598401	164600527	3' UTR	0469	308E-03	335E-02	Fnip2	107374	Hyper
chr5	9090406	9091722	Exon (NM_001191864/362472, exon 22 of 30)	0552	308E-03	335E-02	Cspp1	89044	Hyper
chr1	205474459	205475384	Distal Intergenic	0701	308E-03	335E-02	Slc22a8	-22761	Hyper
chr10	72869189	72872786	Exon (NM_001304355/287615, exon 5 of 6)	0364	309E-03	335E-02	Vezf1	9285	Hyper
chr1	117127624	117128262	Distal Intergenic	0781	309E-03	336E-02	Chrna7	-290401	Hyper
chr19	50514470	50518674	3' UTR	0314	309E-03	336E-02	Snai3	4812	Hyper

chr9	1183362	1187607	Exon (NM_001044236/301128, exon 3 of 23)	0376	309E-03	336E-02	Kdm4b	24583	Hyper
chr2	185961473	185962040	Distal Intergenic	0737	309E-03	336E-02	Phgdh	-25313	Hyper
chr5	58006723	58007167	Distal Intergenic	0847	310E-03	336E-02	Or13c7d	13398	Hyper
chr15	39097410	39099809	Distal Intergenic	0379	310E-03	336E-02	Kif13b	189092	Hyper
chr9	103642189	103644957	Intron (NM_001106928/301737, intron 4 of 13)	0357	310E-03	336E-02	Fer	33801	Hyper
chr4	146767725	146768838	Distal Intergenic	0573	310E-03	336E-02	Vhl	-3645	Hyper
chr10	18380271	18380958	Intron (NM_001261389/65023, intron 1 of 8)	0825	310E-03	337E-02	Kcnip1	55137	Hyper
chr9	25651520	25658236	Distal Intergenic	0318	310E-03	337E-02	Mir30a	-79364	Hyper
chr16	50224252	50224570	Distal Intergenic	0979	310E-03	337E-02	Frg1	-701233	Hyper
chr4	131728594	131732098	Intron (NM_001034131/297480, intron 1 of 15)	0320	311E-03	337E-02	Foxp1	64657	Hyper
chr9	90194194	90195503	Intron (NM_001108230/316611, intron 2 of 15)	0592	311E-03	337E-02	Agap1	6169	Hyper
chr12	20167524	20168954	Intron (NM_053860/116639, intron 1 of 22)	0516	311E-03	337E-02	Cux1	60213	Hyper
chr10	93215090	93216510	Intron (NM_001105713/24680, intron 2 of 15)	0482	311E-03	337E-02	Prkca	71297	Hyper
chr8	56715050	56715606	Intron (NM_001108149/315686, intron 3 of 6)	0792	311E-03	338E-02	Peak1	103490	Hyper
chr7	109092138	109094342	Distal Intergenic	0383	312E-03	338E-02	Rbfox2	-37735	Hyper
chr14	74153155	74155166	Exon (NM_001109388/680039, exon 8 of 9)	0480	312E-03	338E-02	Tbc1d14	-17498	Hyper
chr19	45396295	45399418	Intron (NM_001163273/292051, intron 1 of 20)	0382	312E-03	338E-02	Cmip	91698	Hyper
chr5	147009682	147010322	Distal Intergenic	0778	313E-03	338E-02	Ldlrap1	-31081	Hyper
chr5	132727627	132731062	Exon (NM_138827/24778, exon 2 of 10)	0337	313E-03	339E-02	Slc2a1	10428	Hyper
chr8	110654602	110662068	Intron (NM_001108783/363151, intron 1 of 6)	0290	314E-03	340E-02	Ccdc12	18892	Hyper
chr7	22803451	22804162	Distal Intergenic	0745	315E-03	340E-02	Dram1	-26646	Hyper
chrX	64663381	64664126	Distal Intergenic	0788	315E-03	340E-02	Pja1	-77607	Hyper
chr13	42944898	42946778	Intron (NM_001134958/360840, intron 2 of 21)	0441	315E-03	340E-02	Srgap2	20280	Hyper
chr13	46967276	46972514	Distal Intergenic	0327	315E-03	340E-02	Ipo9	-104582	Hyper
chr1	185496902	185502996	Promoter (<=1kb)	0316	316E-03	341E-02	Htra1	0	Hyper
chr5	141073560	141078486	Intron (NM_001013169/313038, intron 8 of 13)	0282	317E-03	342E-02	Phc2	22718	Hyper
chr14	31002917	31004050	Distal Intergenic	0470	317E-03	342E-02	Spink2	35099	Hyper
chr12	41376000	41379410	Promoter (1-2kb)	0381	317E-03	342E-02	Pop5	1926	Hyper
chr10	19445391	19445912	Distal Intergenic	0884	317E-03	342E-02	Slit3	-125886	Hyper
chr6	119747904	119750694	Distal Intergenic	0352	317E-03	342E-02	Ttc7b	-42717	Hyper
chr13	22028294	22030735	Distal Intergenic	0416	318E-03	343E-02	Zcchc2	-88833	Hyper
chr1	220516430	220518712	Intron (NM_001191562/309407, intron 21 of 24)	0376	318E-03	343E-02	Klf9	-181396	Hyper
chr6	88003141	88005458	Distal Intergenic	0392	318E-03	343E-02	Arf6	149399	Hyper

chr20	10173406	10174047	Distal Intergenic	0883	318E-03	343E-02	Pdxk	-36346	Hyper
chr19	48593378	48600024	Distal Intergenic	0298	319E-03	343E-02	Gins2	39315	Hyper
chr7	133343749	133345881	Distal Intergenic	0511	319E-03	343E-02	Itgb7	14596	Hyper
chr12	34876900	34878165	Exon (NM_001271194/687346, exon 11 of 12)	0609	319E-03	343E-02	Brap	22865	Hyper
chr19	8894168	8895143	Distal Intergenic	0563	320E-03	344E-02	Got2	-279161	Hyper
chr11	30604218	30606005	Distal Intergenic	0402	320E-03	344E-02	Il10rb	-40648	Hyper
chr3	103903433	103904989	Distal Intergenic	0441	320E-03	344E-02	Spred1	-79040	Hyper
chr2	61069653	61070232	Distal Intergenic	0688	320E-03	344E-02	Sub1	-49281	Hyper
chr3	14965188	14966997	Promoter (2-3kb)	0571	320E-03	344E-02	Exosc2	2258	Hyper
chr17	12276704	12277457	Distal Intergenic	0733	320E-03	344E-02	Nfil3	-3042	Hyper
chr10	14228814	14229980	Distal Intergenic	0692	320E-03	344E-02	Uqcc4	26597	Hyper
chr18	26854783	26856182	Intron (NM_001007145/307505, intron 13 of 17)	0513	320E-03	344E-02	Lrrtm2	-31919	Hyper
chr14	6614255	6614984	Intron (NM_001270556/25272, intron 2 of 13)	0730	321E-03	344E-02	Mapk10	116548	Hyper
chr2	61005988	61006928	Distal Intergenic	0662	321E-03	345E-02	Sub1	13444	Hyper
chr9	16811833	16813952	Promoter (<=1kb)	0440	321E-03	345E-02	Clic5	0	Hyper
chr2	111401516	111402257	Intron (NM_001106422/294917, intron 2 of 30)	0683	322E-03	345E-02	Slc2a2	-209591	Hyper
chr1	29591872	29592336	Intron (NM_001039722/664630, intron 1 of 11)	0751	322E-03	345E-02	Slc6a19	5667	Hyper
chr7	134392866	134393981	Promoter (2-3kb)	0578	322E-03	345E-02	Nfe2	-2758	Hyper
chr1	33572718	33574768	Intron (NM_001145163/679949, intron 1 of 1)	0393	322E-03	345E-02	Ube2ql1	23904	Hyper
chr11	15277709	15277929	Distal Intergenic	1056	322E-03	345E-02	Nrip1	-298288	Hyper
chr16	18829318	18832322	Promoter (1-2kb)	0383	322E-03	345E-02	Ssbp4	1264	Hyper
chrX	66867440	66871364	Distal Intergenic	0324	322E-03	345E-02	Cxcr3	-20471	Hyper
chr7	107905885	107908107	Exon (NM_001164308/64204, exon 7 of 32)	0413	323E-03	345E-02	Plec	4104	Hyper
chr6	31305891	31308077	Distal Intergenic	0388	323E-03	345E-02	Rhob	57846	Hyper
chr16	76520855	76521769	Exon (NM_053951/117020, exon 18 of 31)	0715	323E-03	345E-02	F7	-20267	Hyper
chr1	84236286	84245202	Intron (NM_031675/63836, intron 1 of 20)	0281	323E-03	345E-02	Actn4	6616	Hyper
chr2	203684949	203685457	Distal Intergenic	0820	323E-03	346E-02	S1pr1	-55839	Hyper
chr5	158421784	158422578	Distal Intergenic	0713	323E-03	346E-02	Nppb	4918	Hyper
chr2	227151815	227152597	Distal Intergenic	0698	324E-03	346E-02	Eif4e	84431	Hyper
chr11	35954588	35956378	Exon (NM_133587/171119, exon 30 of 33)	0460	324E-03	346E-02	Pcp4	153875	Hyper
chr17	60342299	60344050	Intron (NM_012527/24260, intron 4 of 4)	0458	324E-03	346E-02	Zmynd11	-199499	Hyper
chr6	27695408	27696567	Distal Intergenic	0550	325E-03	347E-02	Fam228a	53494	Hyper
chr7	52120322	52121910	Promoter (1-2kb)	0527	325E-03	347E-02	Kcnmb4	-1224	Hyper

chr7	82045640	82046627	Intron (NM_001134837/299897, intron 5 of 6)	0568	325E-03	347E-02	Trps1	94727	Hyper
chr15	37129868	37131995	Intron (NM_001107269/305956, intron 1 of 10)	0478	325E-03	347E-02	Wdfy2	46026	Hyper
chr3	165376008	165376439	Intron (NM_214459/362284, intron 6 of 11)	0894	325E-03	347E-02	Phactr3	32734	Hyper
chr12	14841388	14842128	Intron (NM_145673/246760, intron 1 of 2)	0802	325E-03	347E-02	Mafk	3217	Hyper
chr17	14851796	14852636	Distal Intergenic	0607	325E-03	347E-02	Iars1	134581	Hyper
chr16	18824314	18829230	Promoter (<=1kb)	0263	325E-03	347E-02	Ssbp4	0	Hyper
chr16	77669993	77672757	3' UTR	0324	326E-03	347E-02	Tex29	-5877	Hyper
chr20	45371174	45371879	Intron (NM_001106396/294518, intron 1 of 7)	0698	326E-03	347E-02	LOC499469	35377	Hyper
chr15	44359670	44359927	Distal Intergenic	1154	326E-03	347E-02	Stc1	60049	Hyper
chr3	119527819	119530168	Intron (NM_053643/114101, intron 1 of 12)	0401	326E-03	347E-02	Cds2	12819	Hyper
chr1	240515505	240518494	Intron (NM_022953/65047, intron 4 of 36)	0369	326E-03	347E-02	Slit1	39633	Hyper
chr5	149677430	149677996	Distal Intergenic	0866	326E-03	347E-02	LOC690206	18294	Hyper
chr10	85750630	85751908	Intron (NM_022380/25126, intron 1 of 18)	0505	327E-03	348E-02	Stat5b	23716	Hyper
chr5	145688928	145689196	Distal Intergenic	1068	327E-03	348E-02	Tent5b	-6162	Hyper
chr8	72861353	72861874	Distal Intergenic	0814	327E-03	348E-02	Tcf12	-62152	Hyper
chr2	135574540	135577638	Exon (NM_001109558/689954, exon 5 of 8)	0378	328E-03	348E-02	Setd7	27830	Hyper
chr15	39755691	39756558	Distal Intergenic	0720	328E-03	348E-02	Pnoc	-118191	Hyper
chr3	28814118	28815651	Intron (NM_001024274/362129, intron 5 of 9)	0481	328E-03	348E-02	Gtdc1	345968	Hyper
chr7	114761621	114762425	3' UTR	0769	329E-03	349E-02	Ttll12	-10384	Hyper
chr4	180993186	180995482	Distal Intergenic	0415	329E-03	350E-02	Ergic2	127578	Hyper
chr3	19364109	19366805	Distal Intergenic	0382	330E-03	350E-02	Ndufa8	32654	Hyper
chr2	234852843	234854257	Promoter (<=1kb)	0403	330E-03	350E-02	C2h1orf52	0	Hyper
chr16	17979721	17980929	Intron (NM_001271066/25486, intron 2 of 41)	0514	330E-03	350E-02	Myo9b	34273	Hyper
chr13	79085669	79086216	Distal Intergenic	0798	330E-03	350E-02	Fmo9	-244594	Hyper
chr1	196571119	196575831	Promoter (<=1kb)	0307	330E-03	350E-02	Polr2l	0	Hyper
chr7	110292305	110292852	Distal Intergenic	0801	330E-03	350E-02	Mfng	35801	Hyper
chr12	6415231	6417237	Distal Intergenic	0389	330E-03	350E-02	Ubl3	-56447	Hyper
chr1	185206955	185209385	Intron (NM_001004415/309025, intron 4 of 21)	0382	330E-03	350E-02	Tacc2	67570	Hyper
chr19	20377811	20381236	Promoter (<=1kb)	0279	331E-03	350E-02	Siah1	0	Hyper
chr1	134743362	134743661	Intron (NM_001108489/361598, intron 2 of 37)	0960	331E-03	351E-02	Iqgap1	26094	Hyper
chr3	13491044	13493707	Distal Intergenic	0330	331E-03	351E-02	Lrrc8a	-16816	Hyper
chr1	136729136	136729780	Intron (NM_001107534/308789, intron 15 of 19)	0891	331E-03	351E-02	Efl1	90609	Hyper
chr7	122444884	122445954	Intron (NM_133611/171147, intron 7 of 9)	0537	331E-03	351E-02	Abcd2	-133242	Hyper

chr5	132019088	132019871	Promoter (<=1kb)	0752	332E-03	351E-02	Tie1	0	Hyper
chr1	79400815	79403345	Intron (NM_001012064/308417, intron 1 of 8)	0398	332E-03	351E-02	Nectin2	4005	Hyper
chr9	1160449	1162004	Promoter (1-2kb)	0581	333E-03	352E-02	Kdm4b	1670	Hyper
chr1	143278315	143281182	Promoter (<=1kb)	0318	333E-03	352E-02	Fzd4	0	Hyper
chr17	63790370	63790952	Intron (NM_206847/60416, intron 19 of 21)	0657	333E-03	352E-02	Pitrm1	36361	Hyper
chr4	45616379	45617753	Promoter (<=1kb)	0530	333E-03	352E-02	Cav2	0	Hyper
chr5	136417784	136418652	Distal Intergenic	0514	334E-03	353E-02	Lnc081	26902	Hyper
chr6	121796993	121797486	Intron (NM_001191985/500709, intron 2 of 9)	0861	334E-03	353E-02	Itpk1	46552	Hyper
chr2	5861870	5862172	Distal Intergenic	0839	335E-03	353E-02	Mctp1	-209464	Hyper
chr16	69833590	69834892	Promoter (<=1kb)	0358	335E-03	353E-02	Vps36	0	Hyper
chr18	16173938	16175152	Intron (NM_001271332/100360334, intron 3 of 26)	0538	335E-03	353E-02	Fhod3	180614	Hyper
chr1	196360819	196365128	Exon (NM_139093/245925, exon 10 of 17)	0317	335E-03	353E-02	Irf7	5319	Hyper
chr1	245377988	245380110	Intron (NM_001128083/688785, intron 1 of 5)	0429	335E-03	353E-02	Trim8	8356	Hyper
chr2	224024251	224026378	Exon (NM_001276711/81736, exon 18 of 25)	0435	335E-03	353E-02	Nfkb1	105453	Hyper
chr6	124365715	124369362	Distal Intergenic	0321	335E-03	353E-02	Bdkrb2	-103204	Hyper
chr14	77509010	77513176	Promoter (<=1kb)	0413	335E-03	353E-02	Spon2	0	Hyper
chr18	4121873	4123535	Promoter (1-2kb)	0489	335E-03	353E-02	Impact	1743	Hyper
chr9	961671	962357	Promoter (1-2kb)	0649	336E-03	354E-02	Sema6b	-1092	Hyper
chr8	42805845	42807174	Intron (NM_001014089/315591, intron 7 of 7)	0588	336E-03	354E-02	Tecta	-26138	Hyper
chr19	24074069	24076104	Promoter (1-2kb)	0435	336E-03	354E-02	Mir709	-1171	Hyper
chr4	124949591	124952566	Intron (NM_001107876/312563, intron 2 of 7)	0392	336E-03	354E-02	Trh	-204954	Hyper
chr13	103489841	103492498	Promoter (<=1kb)	0322	337E-03	355E-02	Slc30a1	0	Hyper
chr5	134536666	134540471	Exon (NM_001100669/298500, exon 5 of 10)	0367	337E-03	355E-02	Smap2	36467	Hyper
chr3	168257200	168259060	Distal Intergenic	0471	338E-03	355E-02	Kcnq2	-3460	Hyper
chr9	11440106	11442413	Distal Intergenic	0452	338E-03	355E-02	Mocs1	125377	Hyper
chr8	20189002	20190239	Exon (NM_134368/171379, exon 6 of 34)	0564	338E-03	355E-02	Smarca4	21285	Hyper
chr5	155195138	155196132	Distal Intergenic	0605	338E-03	355E-02	Prdm2	335693	Hyper
chr3	147018565	147020145	Distal Intergenic	0413	338E-03	355E-02	Snhg11	-11266	Hyper
chr16	76044965	76048678	Promoter (<=1kb)	0310	339E-03	356E-02	Gas6	0	Hyper
chr13	94210509	94211810	Distal Intergenic	0448	339E-03	356E-02	Capn2	-9540	Hyper
chr4	104003037	104005095	Intron (NM_031772/83581, intron 28 of 34)	0418	339E-03	356E-02	Polr1a	52986	Hyper
chr9	850040	852295	Exon (NM_031239/81922, exon 2 of 10)	0439	339E-03	356E-02	Mpnd	9842	Hyper
chr1	167272622	167273584	Distal Intergenic	0571	339E-03	356E-02	Bmal1	-58236	Hyper

chr3	13838739	13839521	Distal Intergenic	0636	339E-03	356E-02	Ier5l	-93092	Hyper
chr14	76330963	76332007	Distal Intergenic	0602	340E-03	356E-02	Rnf4	90557	Hyper
chr13	92152142	92157067	Exon (NM_019312/54260, exon 4 of 8)	0290	340E-03	356E-02	Itpkb	82926	Hyper
chr1	176861504	176862459	Intron (NM_012713/25023, intron 2 of 17)	0567	340E-03	357E-02	Prkcb	28819	Hyper
chr5	132655939	132657826	Distal Intergenic	0446	341E-03	357E-02	Slc2a1	-59373	Hyper
chr3	60602343	60603761	Intron (NM_031789/83619, intron 1 of 4)	0486	341E-03	357E-02	Nfe2l2	17910	Hyper
chr3	168966758	168968921	Exon (NM_001107810/311726, exon 4 of 7)	0399	341E-03	357E-02	Pcmtd2	6446	Hyper
chr3	140579904	140588342	Promoter (<=1kb)	0255	341E-03	357E-02	Scrt2	0	Hyper
chr2	246272415	246272754	Intron (NM_021682/59318, intron 6 of 6)	0863	341E-03	357E-02	Zranb2	-300625	Hyper
chr9	1516885	1517931	Distal Intergenic	0711	342E-03	357E-02	Vmac	31644	Hyper
chr10	55395848	55398720	Promoter (2-3kb)	0419	342E-03	357E-02	Camta2	2838	Hyper
chr2	33991225	33991812	Distal Intergenic	0848	342E-03	358E-02	Cd180	135234	Hyper
chr4	120662699	120663784	Exon (NM_033442/25159, exon 5 of 7)	0634	343E-03	358E-02	Gata2	3713	Hyper
chr1	55232514	55233732	Distal Intergenic	0607	344E-03	359E-02	Smoc2	-28798	Hyper
chr1	78728080	78731341	Promoter (<=1kb)	0379	345E-03	360E-02	Dmpk	0	Hyper
chr4	141070755	141071076	Distal Intergenic	0947	345E-03	360E-02	Setmar	24668	Hyper
chr2	34021530	34024229	Distal Intergenic	0455	345E-03	360E-02	Cd180	165539	Hyper
chr5	144228544	144229126	Distal Intergenic	0746	345E-03	360E-02	Oprd1	111834	Hyper
chr17	4126255	4130427	Distal Intergenic	0376	345E-03	360E-02	Dapk1	-35264	Hyper
chr7	72896930	72897518	Intron (NM_001197907/117520, intron 3 of 16)	0638	345E-03	360E-02	Oxr1	5540	Hyper
chr14	78361539	78365286	Promoter (2-3kb)	0337	346E-03	360E-02	Pla2g3	-2947	Hyper
chr18	54981917	54984119	Exon (NM_001100974/307398, exon 3 of 16)	0383	346E-03	360E-02	Arhgef37	16550	Hyper
chr7	33887573	33889051	Distal Intergenic	0418	346E-03	360E-02	Galnt4	-36178	Hyper
chr1	98778880	98781013	Distal Intergenic	0527	346E-03	361E-02	Nav2	-177385	Hyper
chr10	85884511	85885141	Exon (NM_001105841/287710, exon 2 of 2)	0667	346E-03	361E-02	Cavin1	10943	Hyper
chr20	28028092	28028564	3' UTR	0773	347E-03	361E-02	Spock2	-9028	Hyper
chr3	62116666	62117528	Distal Intergenic	0644	347E-03	362E-02	Sestd1	81482	Hyper
chr7	14612948	14613418	Intron (NM_001106772/299643, intron 2 of 3)	0798	348E-03	362E-02	Ndufa7	3665	Hyper
chr4	167766447	167769068	Distal Intergenic	0328	348E-03	362E-02	Cdkn1b	6266	Hyper
chr19	47527770	47529863	Downstream (<=300bp)	0470	348E-03	362E-02	Slc38a8	24863	Hyper
chr10	60405484	60407642	Exon (NM_001105812/287532, exon 7 of 14)	0398	350E-03	364E-02	Pitpna	-23134	Hyper
chr1	76974250	76975227	Distal Intergenic	0602	351E-03	364E-02	Inafm1	3065	Hyper
chr2	197465443	197467267	3' UTR	0443	351E-03	364E-02	Ntng1	359730	Hyper

chr3	15455912	15459056	Distal Intergenic	0338	351E-03	364E-02	Prrc2b	-6238	Hyper
chr7	8755339	8757819	5' UTR	0410	351E-03	364E-02	Gadd45b	22214	Hyper
chr2	179232865	179233520	Distal Intergenic	0703	351E-03	364E-02	S100a10	11853	Hyper
chr5	137570840	137571311	Distal Intergenic	0917	351E-03	364E-02	Zc3h12a	-185489	Hyper
chr4	52783986	52785689	Distal Intergenic	0460	351E-03	365E-02	Slc13a1	-136883	Hyper
chr3	141986141	141989520	Distal Intergenic	0295	353E-03	365E-02	Commd7	124810	Hyper
chr1	187915636	187919231	Distal Intergenic	0323	353E-03	365E-02	Ctbp2	-103788	Hyper
chr11	32152316	32152924	Distal Intergenic	0712	353E-03	366E-02	Runx1	-216885	Hyper
chr4	95157793	95158176	Distal Intergenic	0835	354E-03	366E-02	RSA-14-44	-93481	Hyper
chr4	63068536	63069866	Distal Intergenic	0482	355E-03	367E-02	Akr1b7	-7077	Hyper
chr2	219461363	219462017	Distal Intergenic	0780	355E-03	367E-02	Rpl34	-173255	Hyper
chr20	12007451	12008909	Distal Intergenic	0502	356E-03	368E-02	Col6a2	-12858	Hyper
chr5	62996251	62997186	Promoter (<=1kb)	0583	356E-03	368E-02	Cavin4	0	Hyper
chr3	130788786	130789169	Distal Intergenic	0671	356E-03	368E-02	Pcsk2	-91253	Hyper
chr7	128875085	128877513	3' UTR	0345	357E-03	369E-02	Endou	-8543	Hyper
chr18	30736456	30737958	Distal Intergenic	0462	357E-03	369E-02	Fgf1	-26175	Hyper
chr4	68005609	68008342	Distal Intergenic	0393	357E-03	369E-02	Chmp4bl1	-41400	Hyper
chr7	46349123	46349795	Promoter (<=1kb)	0621	358E-03	369E-02	Csrp2	14	Hyper
chr4	130526708	130528352	Intron (NM_001191089/25094, intron 1 of 9)	0418	358E-03	369E-02	Mitf	117491	Hyper
chr10	70207137	70209762	3' UTR	0434	358E-03	369E-02	Bcas3	-4340	Hyper
chr19	20394992	20398319	Intron (NM_080905/140941, intron 1 of 1)	0378	358E-03	369E-02	Siah1	16084	Hyper
chr17	6977351	6979515	Intron (NM_001271297/306759, intron 3 of 10)	0421	359E-03	370E-02	Spock1	235838	Hyper
chr9	75846058	75846806	Exon (NM_001106919/301511, exon 8 of 11)	0696	359E-03	370E-02	Gpbar1	-15345	Hyper
chr19	49702883	49703784	Distal Intergenic	0619	359E-03	370E-02	Map1lc3b	36904	Hyper
chr1	117921867	117924930	Exon (NM_001191633/309256, exon 10 of 13)	0338	360E-03	370E-02	Fan1	19496	Hyper
chr18	59985414	59986647	Promoter (<=1kb)	0588	360E-03	371E-02	Pmaip1	0	Hyper
chr20	45964226	45965889	Promoter (2-3kb)	0509	360E-03	371E-02	Afg1l	2017	Hyper
chr4	116002191	116005239	Exon (NM_001106602/297389, exon 2 of 7)	0342	360E-03	371E-02	Dguok	9494	Hyper
chr3	114786919	114787493	Distal Intergenic	0630	360E-03	371E-02	Prom2	-25132	Hyper
chr5	145157870	145159234	Distal Intergenic	0482	360E-03	371E-02	Fam76a	-22316	Hyper
chr8	57369103	57369779	Intron (NM_001004270/316108, intron 5 of 8)	0748	361E-03	371E-02	Snupn	20945	Hyper
chr5	9643107	9643743	Distal Intergenic	0832	361E-03	371E-02	Vxn	40941	Hyper
chr8	44821391	44824945	Intron (NM_001106817/300673, intron 2 of 10)	0346	361E-03	371E-02	Bcl9l	9414	Hyper

chr2	242637326	242638827	Intron (NM_019123/29758, intron 1 of 4)	0540	363E-03	373E-02	St6galnac3	6239	Hyper
chr16	46594388	46597330	5' UTR	0391	363E-03	373E-02	Sorbs2	29118	Hyper
chr7	111647812	111650689	Intron (NM_019166/29205, intron 1 of 3)	0437	364E-03	373E-02	Syngr1	11894	Hyper
chr6	132143584	132145984	Distal Intergenic	0432	364E-03	373E-02	Mta1	-32869	Hyper
chr7	119957631	119959148	Distal Intergenic	0485	364E-03	373E-02	Pim3	4254	Hyper
chr7	111208812	111209650	Intron (NM_001130581/300074, intron 1 of 17)	0547	364E-03	373E-02	Fam227a	6833	Hyper
chr4	19189059	19189995	Intron (NM_012919/25399, intron 3 of 41)	0672	364E-03	374E-02	Cacna2d1	184689	Hyper
chr10	97260057	97261012	Distal Intergenic	0573	364E-03	374E-02	Sox9	-545473	Hyper
chr13	64902072	64904237	Promoter (<=1kb)	0349	365E-03	374E-02	Ralgdsl1	0	Hyper
chr2	11726356	11726981	Distal Intergenic	0682	365E-03	374E-02	Lysmd3	-206806	Hyper
chr10	86535100	86536170	Distal Intergenic	0648	366E-03	374E-02	Rdm1	-26701	Hyper
chr13	90203589	90204598	Intron (NM_001105977/289280, intron 2 of 5)	0558	366E-03	374E-02	Efcab2	51404	Hyper
chr6	106581356	106581706	Distal Intergenic	0918	366E-03	374E-02	Cipc	-13313	Hyper
chr19	2218371	2219304	Intron (NM_053392/84407, intron 2 of 12)	0601	366E-03	374E-02	Cdh11	69924	Hyper
chr1	253372981	253373749	Distal Intergenic	0562	366E-03	375E-02	Adra2a	311501	Hyper
chr13	94189446	94193956	Intron (NM_017116/29154, intron 2 of 20)	0305	366E-03	375E-02	Capn2	7013	Hyper
chr11	50380740	50384481	Distal Intergenic	0287	366E-03	375E-02	Bbx	-96132	Hyper
chr18	58293779	58296427	Distal Intergenic	0344	367E-03	375E-02	Nedd4l	-98550	Hyper
chr5	119977718	119981220	Exon (NM_138905/192270, exon 4 of 6)	0364	368E-03	376E-02	Plpp3	50633	Hyper
chr6	70277972	70278621	Intron (NM_022618/64553, intron 1 of 13)	0651	368E-03	376E-02	Akap6	93797	Hyper
chr4	132143086	132144315	Distal Intergenic	0515	369E-03	377E-02	Eif4e3	157493	Hyper
chr6	32033108	32033744	Distal Intergenic	0597	369E-03	377E-02	Wdr35	261748	Hyper
chr15	37368233	37370911	Distal Intergenic	0363	369E-03	377E-02	Ctsb	-18725	Hyper
chr12	12146278	12147932	Intron (NM_001037219/304298, intron 1 of 8)	0460	370E-03	378E-02	Foxk1	27157	Hyper
chr13	102873690	102876253	Distal Intergenic	0375	371E-03	378E-02	Nenf	18551	Hyper
chr11	31543875	31544399	Distal Intergenic	0694	371E-03	378E-02	C11h21orf140	7858	Hyper
chr10	91650351	91652814	Promoter (<=1kb)	0451	371E-03	378E-02	Pecam1	0	Hyper
chr1	163858590	163859913	Distal Intergenic	0483	371E-03	378E-02	Nrip3	-43554	Hyper
chr8	69522587	69524695	Distal Intergenic	0361	371E-03	378E-02	Ice2	-519875	Hyper
chr15	25035848	25036660	Promoter (2-3kb)	0593	372E-03	378E-02	Sall2	2258	Hyper
chr11	30819692	30820352	Intron (NM_001025014/360698, intron 1 of 6)	0740	372E-03	378E-02	Tmem50b	17292	Hyper
chr5	146206178	146208543	Exon (NM_001011978/298541, exon 8 of 9)	0472	372E-03	378E-02	Hmgn2	-10598	Hyper
chr1	239201334	239202153	Distal Intergenic	0659	372E-03	378E-02	Pdlim1	-110366	Hyper

chr14	75807060	75811541	Distal Intergenic	0300	372E-03	378E-02	Rgs12	-12464	Hyper
chr9	14120209	14121184	Distal Intergenic	0750	372E-03	378E-02	Tbcc	-20183	Hyper
chr17	29490777	29492666	Distal Intergenic	0476	372E-03	378E-02	Cdyl	-286464	Hyper
chr19	13455830	13457041	Distal Intergenic	0422	372E-03	378E-02	Hmox1	-10217	Hyper
chr1	76731995	76739120	Distal Intergenic	0245	372E-03	378E-02	Zfp541	-11116	Hyper
chr10	102330747	102331722	Intron (NM_001108309/360668, intron 2 of 16)	0559	372E-03	378E-02	Sec14l1	10827	Hyper
chr5	71995848	71997714	Distal Intergenic	0426	372E-03	378E-02	LOC100910620	103356	Hyper
chr5	134547785	134551507	Intron (NM_001100669/298500, intron 1 of 9)	0340	373E-03	379E-02	Smap2	25431	Hyper
chr16	69152071	69152869	Intron (NM_001100570/306571, intron 1 of 15)	0637	373E-03	379E-02	Kat6a	10737	Hyper
chr18	54798964	54801322	Intron (NM_176075/291567, intron 1 of 11)	0421	373E-03	379E-02	Mir3557	-57215	Hyper
chr10	13604755	13605772	Exon (NM_001257352/24650, exon 20 of 46)	0588	374E-03	379E-02	Pkd1	30413	Hyper
chr19	50452819	50454271	Intron (NM_201416/292067, intron 3 of 17)	0496	374E-03	379E-02	Zc3h18	8828	Hyper
chr7	109288711	109289413	Promoter (<=1kb)	0749	374E-03	379E-02	Apol3l1	0	Hyper
chr10	102973824	102975414	Distal Intergenic	0490	374E-03	380E-02	Tmc8	-24930	Hyper
chr6	119788107	119789182	Distal Intergenic	0611	374E-03	380E-02	Ttc7b	-82920	Hyper
chr14	41580605	41582200	Distal Intergenic	0577	374E-03	380E-02	Uchl1	-85015	Hyper
chr8	37119350	37119750	Promoter (<=1kb)	0844	375E-03	380E-02	Robo4	-238	Hyper
chr1	220572037	220573095	Distal Intergenic	0496	375E-03	380E-02	Klf9	-127013	Hyper
chr7	110538153	110539027	Intron (NM_001013220/362956, intron 10 of 22)	0553	375E-03	380E-02	Triobp	29567	Hyper
chr5	72631102	72631899	Intron (NM_001011974/298024, intron 2 of 3)	0696	375E-03	380E-02	Akap2	26273	Hyper
chr14	35061543	35062611	Distal Intergenic	0531	375E-03	380E-02	Zar1	134771	Hyper
chr14	66990208	66991595	Promoter (<=1kb)	0713	376E-03	381E-02	Prom1	48	Hyper
chr8	119703545	119706556	3' UTR	0358	376E-03	381E-02	Csrnp1	4261	Hyper
chr20	11136361	11139296	Exon (NM_001008307/294333, exon 4 of 6)	0404	376E-03	381E-02	Slx9	21729	Hyper
chr1	199715197	199716054	Promoter (1-2kb)	0643	377E-03	381E-02	Ppfia1	1193	Hyper
chr7	120614288	120621791	Exon (NM_021676/59312, exon 20 of 22)	0280	377E-03	381E-02	Acr	-16530	Hyper
chr5	166701648	166702342	Promoter (<=1kb)	0535	377E-03	381E-02	C5h1orf159	0	Hyper
chr4	118689490	118691016	Intron (NM_012671/24827, intron 3 of 5)	0607	377E-03	381E-02	Tgfa	71221	Hyper
chr8	33476831	33480025	Intron (NM_153302/266605, intron 3 of 5)	0391	377E-03	382E-02	St3gal4	-12341	Hyper
chr10	79947372	79948524	Exon (NM_053474/84686, exon 4 of 10)	0601	378E-03	382E-02	Samd14	-9283	Hyper
chr1	117903482	117906012	Exon (NM_001100846/309255, exon 14 of 16)	0431	378E-03	382E-02	Fan1	38414	Hyper
chr15	34867573	34868315	Intron (NM_001191686/305938, intron 4 of 12)	0663	378E-03	382E-02	C1qtnf9	-47014	Hyper
chr10	3993805	3995522	Intron (NM_001109526/689142, intron 16 of 20)	0419	378E-03	382E-02	Cpped1	292283	Hyper

chr2	70193725	70194458	Distal Intergenic	0526	378E-03	383E-02	Cdh10	1564945	Hyper
chr17	17435140	17435875	Intron (NM_001108881/364681, intron 7 of 7)	0652	378E-03	383E-02	Rnf144b	126885	Hyper
chr17	7126303	7130278	Intron (NM_001271297/306759, intron 5 of 10)	0308	378E-03	383E-02	Spock1	384790	Hyper
chr9	4051222	4052580	Distal Intergenic	0473	379E-03	383E-02	Tbc1d5	-34309	Hyper
chr1	69338265	69339651	Exon (NM_001191946/499076, exon 7 of 22)	0519	379E-03	383E-02	Ppp1r12c	18010	Hyper
chr10	61013290	61014053	Intron (NM_001105813/287535, intron 4 of 21)	0617	380E-03	384E-02	Rpl37l1	-19746	Hyper
chr20	26473148	26476597	Exon (NM_001191604/294429, exon 20 of 29)	0357	380E-03	384E-02	Ranbp2	30931	Hyper
chr15	99590242	99592569	Distal Intergenic	0468	381E-03	384E-02	Zic2	13545	Hyper
chr7	53566948	53567728	Distal Intergenic	0622	381E-03	385E-02	Rap1b	-133325	Hyper
chr10	83822894	83824886	Distal Intergenic	0381	381E-03	385E-02	Wipf2	-15606	Hyper
chr9	75609116	75610581	Intron (NM_001191810/301509, intron 3 of 34)	0557	381E-03	385E-02	Tns1	92177	Hyper
chr8	121074823	121079123	Intron (NM_001134565/316085, intron 2 of 15)	0335	382E-03	385E-02	Trak1	27468	Hyper
chr1	127083008	127086874	Distal Intergenic	0288	382E-03	385E-02	Rgma	-42060	Hyper
chr2	78301434	78302696	Exon (NM_001302889/100362554, exon 4 of 7)	0552	382E-03	385E-02	Otulin	13726	Hyper
chr4	59904120	59906649	Distal Intergenic	0338	382E-03	385E-02	Mkln1	-95849	Hyper
chrX	152031063	152031540	Promoter (<=1kb)	0776	383E-03	386E-02	Flna	-11	Hyper
chr12	35203567	35204116	Distal Intergenic	0841	383E-03	386E-02	Trafd1	37961	Hyper
chr1	209463615	209471616	Exon (NM_001047855/293774, exon 6 of 8)	0259	383E-03	386E-02	Dtx4	9457	Hyper
chr14	72270991	72271752	Intron (NM_001014135/360950, intron 3 of 14)	0669	383E-03	386E-02	Wdr1	12959	Hyper
chr14	73960627	73961158	Distal Intergenic	0708	383E-03	386E-02	Man2b2	-7921	Hyper
chr14	79175985	79177670	Distal Intergenic	0377	384E-03	387E-02	Lif	41324	Hyper
chr17	30693914	30696402	Exon (NM_022624/64559, exon 8 of 11)	0392	384E-03	387E-02	Psmg4	21622	Hyper
chr12	10444142	10445415	Intron (NM_001137641/304286, intron 9 of 14)	0431	384E-03	387E-02	Bhlha15	-19501	Hyper
chr3	19031896	19034755	Intron (NM_138710/192126, intron 3 of 15)	0417	387E-03	389E-02	Dab2ip	116606	Hyper
chr8	118732739	118733896	Promoter (<=1kb)	0544	387E-03	389E-02	Ctdspl	-343	Hyper
chr18	15592328	15593295	Distal Intergenic	0505	387E-03	389E-02	Galnt1	64122	Hyper
chr12	26634541	26636260	Exon (NM_053670/114205, exon 5 of 6)	0451	388E-03	389E-02	Crcp	23390	Hyper
chr12	32712230	32712669	Promoter (1-2kb)	0856	388E-03	389E-02	Hcar1	1203	Hyper
chr2	175286628	175288377	Distal Intergenic	0474	388E-03	389E-02	Il6r	59147	Hyper
chr20	18526012	18529089	Distal Intergenic	0311	388E-03	389E-02	Mrln	-104202	Hyper
chr9	73264943	73265731	Promoter (<=1kb)	0605	388E-03	390E-02	Fn1	-266	Hyper
chr13	66959229	66961153	Distal Intergenic	0334	389E-03	390E-02	Cacna1e	-64779	Hyper
chr3	18965540	18967904	Intron (NM_138710/192126, intron 1 of 15)	0411	389E-03	390E-02	Dab2ip	50250	Hyper

chr9	55727135	55727483	Distal Intergenic	0917	389E-03	390E-02	Ccdc150	-82427	Hyper
chr3	14278714	14281114	5' UTR	0408	390E-03	391E-02	Usp20	5776	Hyper
chr2	225217345	225218098	Intron (NM_017041/24674, intron 1 of 13)	0626	390E-03	391E-02	Ppp3ca	51234	Hyper
chr15	44137918	44138394	Distal Intergenic	0880	391E-03	391E-02	Stc1	-161227	Hyper
chr9	1206142	1208272	Intron (NM_001044236/301128, intron 7 of 22)	0450	391E-03	391E-02	Kdm4b	47363	Hyper
chr16	24870287	24872289	Distal Intergenic	0489	391E-03	392E-02	Msmo1	-108391	Hyper
chr20	4948292	4949484	Promoter (2-3kb)	0534	392E-03	392E-02	Pfdn6	2333	Hyper
chr7	27744599	27745376	Exon (NM_001108082/314743, exon 7 of 17)	0663	392E-03	392E-02	Elk3	58708	Hyper
chr9	90915788	90917540	Distal Intergenic	0456	393E-03	392E-02	Ackr3	116071	Hyper
chr2	137150256	137150651	Intron (NM_001109183/499615, intron 2 of 3)	0772	393E-03	393E-02	Lhfpl6	23771	Hyper
chr9	91861768	91862479	Intron (NM_001008381/363284, intron 3 of 8)	0676	394E-03	393E-02	Ube2f	15730	Hyper
chr1	133708017	133709098	Intron (NM_001191949/499189, intron 6 of 16)	0586	394E-03	393E-02	Wdr93	13842	Hyper
chr3	79772280	79773414	Intron (NM_001107751/311215, intron 4 of 12)	0623	394E-03	393E-02	Ext2	24645	Hyper
chr12	42724225	42727278	Distal Intergenic	0426	394E-03	394E-02	Coro1c	-13588	Hyper
chr1	243885026	243886794	Promoter (2-3kb)	0530	395E-03	394E-02	Lzts2	2085	Hyper
chr3	154710061	154710709	Distal Intergenic	0660	395E-03	394E-02	Ncoa3	-27872	Hyper
chr12	20795802	20796367	Promoter (<=1kb)	0709	395E-03	394E-02	Hspb1	-127	Hyper
chr14	102990492	102992181	Promoter (<=1kb)	0332	395E-03	394E-02	Cfap36	0	Hyper
chr20	18086664	18088071	Distal Intergenic	0489	395E-03	394E-02	Phyhipl	54859	Hyper
chr5	12329897	12331354	Distal Intergenic	0556	396E-03	394E-02	Pcmtd1	-5857	Hyper
chr17	19076176	19086571	Intron (NM_012726/25049, intron 7 of 8)	0256	396E-03	395E-02	Gmpr	102903	Hyper
chr13	84828441	84829570	Intron (NM_031602/29718, intron 1 of 1)	0590	397E-03	396E-02	Pigm	-8759	Hyper
chr3	79820009	79820925	Promoter (<=1kb)	0446	397E-03	396E-02	Accs	0	Hyper
chr10	83343869	83355262	Promoter (<=1kb)	0240	398E-03	396E-02	Ppp1r1b	0	Hyper
chr4	148329301	148330363	Intron (NM_019159/29179, intron 10 of 10)	0479	398E-03	396E-02	Timp4	-16743	Hyper
chr10	83341244	83342616	Distal Intergenic	0505	398E-03	396E-02	Ppp1r1b	-5115	Hyper
chr1	50728057	50728545	Distal Intergenic	0743	400E-03	397E-02	Pabpc6	-316719	Hyper
chr14	95274767	95275827	Intron (NM_001100565/305549, intron 1 of 6)	0586	400E-03	398E-02	Peli1	20107	Hyper
chr14	2549275	2549629	Intron (NM_017256/29610, intron 2 of 16)	1009	400E-03	398E-02	Tgfbr3	59878	Hyper
chr7	20429135	20429823	Intron (NM_001108742/362861, intron 6 of 10)	0726	400E-03	398E-02	Slc41a2	45421	Hyper
chr4	179664393	179665622	Intron (NM_001083336/691337, intron 2 of 14)	0561	401E-03	398E-02	Stk38l	30118	Hyper
chr6	127591767	127593430	Exon (NM_024147/79115, exon 11 of 13)	0532	401E-03	398E-02	Degs2	19863	Hyper
chr11	64902154	64903335	Promoter (<=1kb)	0474	402E-03	399E-02	Parp14	0	Hyper

chr3	47787359	47788040	Intron (NM_131912/170739, intron 2 of 15)	0586	402E-03	399E-02	Kcnh7	34082	Hyper
chr6	25152197	25154428	Promoter (<=1kb)	0307	403E-03	399E-02	Ppm1g	0	Hyper
chr6	10560830	10564611	Distal Intergenic	0321	403E-03	400E-02	Zfp36l2	70798	Hyper
chr10	101530492	101532718	Exon (NM_022691/64632, exon 6 of 18)	0396	404E-03	400E-02	Exoc7	7697	Hyper
chr8	97287283	97289296	Promoter (<=1kb)	0459	405E-03	400E-02	Zbtb38	0	Hyper
chr11	34216311	34218802	Intron (NM_013192/25743, intron 2 of 5)	0321	405E-03	401E-02	Kcnj6	89956	Hyper
chr1	85718377	85719218	Promoter (<=1kb)	0646	405E-03	401E-02	Kirrel2	0	Hyper
chr16	58238966	58240536	Promoter (<=1kb)	0472	405E-03	401E-02	Rbpms	0	Hyper
chr8	96759738	96762489	Distal Intergenic	0306	406E-03	401E-02	Tfdp2	-64480	Hyper
chr1	11801464	11803588	Distal Intergenic	0347	406E-03	401E-02	Cited2	-508838	Hyper
chr7	114697631	114698477	Intron (NM_001191788/315173, intron 2 of 3)	0539	406E-03	402E-02	Mcat	6065	Hyper
chr5	68122609	68126472	Intron (NM_001033852/85254, intron 3 of 15)	0345	406E-03	402E-02	Slc44a1	58991	Hyper
chr8	99512959	99513568	Distal Intergenic	0765	407E-03	402E-02	Faim	-30401	Hyper
chr18	63246105	63249580	Intron (NM_053369/84382, intron 8 of 16)	0341	407E-03	402E-02	Tcf4	186649	Hyper
chr14	52661225	52661579	Intron (NM_001004087/360942, intron 2 of 2)	0760	408E-03	402E-02	Pcdh7	237894	Hyper
chr9	56302125	56303802	Intron (NM_001191807/301415, intron 2 of 27)	0515	408E-03	403E-02	Ankrd44	123706	Hyper
chr17	26651066	26651488	Distal Intergenic	0870	408E-03	403E-02	Snrnp48	-35026	Hyper
chr9	1288717	1289604	Intron (NM_019140/25529, intron 1 of 33)	0721	408E-03	403E-02	Ptprs	17341	Hyper
chr9	87020048	87020595	Distal Intergenic	0589	409E-03	403E-02	Ncl	-11936	Hyper
chr8	45249839	45250492	Intron (NM_207610/315608, intron 13 of 19)	0654	409E-03	404E-02	Atp5mg	-16257	Hyper
chr7	117915972	117916540	Distal Intergenic	0748	410E-03	404E-02	Cerk	-647213	Hyper
chr9	16992041	16993926	Intron (NM_175578/140666, intron 2 of 4)	0501	411E-03	404E-02	Enpp5	-74561	Hyper
chr11	82214365	82215026	Promoter (<=1kb)	0626	411E-03	404E-02	Cldn5	-117	Hyper
chr1	151754420	151756528	Distal Intergenic	0336	411E-03	404E-02	Thrsp	-26680	Hyper
chr1	44121767	44123384	Intron (NM_181474/308140, intron 5 of 6)	0437	411E-03	405E-02	Cldn20	-10385	Hyper
chr14	73700159	73703448	Exon (NM_001033894/305434, exon 3 of 13)	0326	411E-03	405E-02	Jakmip1	10545	Hyper
chr3	19370706	19371744	Distal Intergenic	0614	412E-03	405E-02	Ndufa8	27715	Hyper
chr14	74197861	74200312	Intron (NM_001113365/360956, intron 3 of 14)	0408	412E-03	405E-02	Tbc1d14	20956	Hyper
chr8	57848387	57850201	Exon (NM_031726/65171, exon 5 of 8)	0448	412E-03	405E-02	Ppcdc	-9619	Hyper
chr12	19012175	19014132	Promoter (<=1kb)	0342	413E-03	405E-02	Agfg2	0	Hyper
chr8	109601848	109602660	Promoter (2-3kb)	0732	413E-03	405E-02	Col7a1	-2353	Hyper
chr13	51924817	51925039	Intron (NM_198762/304827, intron 14 of 27)	0939	413E-03	406E-02	Kcnt2	260131	Hyper
chr5	130115639	130116087	Distal Intergenic	0877	414E-03	406E-02	Akr1a1	-6064	Hyper

chr8	29532166	29533108	Distal Intergenic	0606	414E-03	407E-02	St14	48409	Hyper
chr14	1724576	1725397	Distal Intergenic	0695	414E-03	407E-02	Mtf2	-41119	Hyper
chr15	79662651	79663069	Distal Intergenic	0860	414E-03	407E-02	Acod1	-208758	Hyper
chr17	30802671	30803297	Intron (NM_001037206/361239, intron 6 of 6)	0726	415E-03	407E-02	Tubb2a	5829	Hyper
chr2	195943871	195944609	Intron (NM_031767/83576, intron 1 of 19)	0648	415E-03	407E-02	Sort1	19509	Hyper
chr10	59354453	59358883	Distal Intergenic	0294	415E-03	407E-02	Rap1gap2	-44778	Hyper
chr12	35393179	35397311	Intron (NM_001177593/25622, intron 6 of 15)	0317	415E-03	407E-02	Ptpn11	27743	Hyper
chr1	90773160	90773792	Distal Intergenic	0666	415E-03	407E-02	Ccne1	17396	Hyper
chr6	132105432	132107913	Distal Intergenic	0405	416E-03	408E-02	Brf1	-24154	Hyper
chr2	245993912	245995998	Intron (NM_021682/59318, intron 2 of 6)	0394	416E-03	408E-02	Negr1	369319	Hyper
chr3	13615451	13618253	3' UTR	0382	416E-03	408E-02	Sh3glb2	13485	Hyper
chr10	50387493	50388324	Intron (NM_001030023/287398, intron 3 of 10)	0620	417E-03	408E-02	Map2k4	59555	Hyper
chr4	67584136	67584836	Intron (NM_001108622/362342, intron 1 of 17)	0657	417E-03	408E-02	Hipk2	34387	Hyper
chr2	112909463	112910660	Intron (NM_001106423/294924, intron 1 of 16)	0649	417E-03	408E-02	Mecom	34979	Hyper
chr7	129760384	129764253	Promoter (<=1kb)	0275	417E-03	408E-02	Adcy6	0	Hyper
chr8	92808952	92810065	Promoter (<=1kb)	0531	417E-03	408E-02	Plscr2	0	Hyper
chr13	20783852	20785733	Distal Intergenic	0467	418E-03	409E-02	Cdh20	-214926	Hyper
chr2	191890068	191891121	Distal Intergenic	0455	418E-03	409E-02	Lrig2	121458	Hyper
chr10	69823782	69831037	Promoter (<=1kb)	0294	418E-03	409E-02	Car4	0	Hyper
chr4	146758984	146759713	Intron (NM_001195476/679934, intron 1 of 2)	0662	419E-03	409E-02	Brk1	8163	Hyper
chr20	30160122	30161377	Intron (NM_001115032/679462, intron 1 of 7)	0533	419E-03	409E-02	Tspan15	13535	Hyper
chr12	22035716	22040289	Intron (NM_031727/65172, intron 2 of 15)	0339	419E-03	409E-02	Limk1	9019	Hyper
chr4	67596322	67597329	Intron (NM_001108622/362342, intron 1 of 17)	0595	419E-03	409E-02	Hipk2	21894	Hyper
chr6	130700032	130700796	Intron (NM_130749/170577, intron 14 of 17)	0700	419E-03	410E-02	Ckb	31505	Hyper
chr13	71812593	71815578	Distal Intergenic	0443	419E-03	410E-02	Mir1843b	261045	Hyper
chr2	76101001	76102030	Promoter (<=1kb)	0627	419E-03	410E-02	Myo10	14	Hyper
chr10	82655669	82656494	Distal Intergenic	0598	420E-03	410E-02	Cisd3	-22851	Hyper
chr10	16483919	16495155	3' UTR	0261	420E-03	410E-02	Atp6v0e1	7577	Hyper
chr12	32353508	32356201	Distal Intergenic	0394	420E-03	410E-02	Pitpnm2	-24604	Hyper
chr3	78830207	78831225	Distal Intergenic	0557	420E-03	410E-02	Syt13	-45975	Hyper
chr5	162826742	162827822	Distal Intergenic	0443	420E-03	410E-02	Rpl22	-7429	Hyper
chr8	37119942	37121124	Promoter (<=1kb)	0565	420E-03	410E-02	Robo4	0	Hyper
chr14	12771500	12771804	Intron (NM_012823/25291, intron 2 of 12)	0974	421E-03	410E-02	Anxa3	9830	Hyper

chr4	124956344	124957444	Intron (NM_001107876/312563, intron 2 of 7)	0535	421E-03	410E-02	Trh	-211707	Hyper
chr16	3035730	3036534	Intron (NM_170787/259269, intron 3 of 16)	0597	421E-03	411E-02	Erc2	187052	Hyper
chr1	118230885	118233150	Intron (NM_031780/83610, intron 3 of 13)	0394	422E-03	411E-02	Apba2	127660	Hyper
chr4	124207519	124209469	Intron (NM_017206/29464, intron 1 of 14)	0471	422E-03	411E-02	Slc6a6	12172	Hyper
chr1	196492739	196496200	Promoter (<=1kb)	0288	422E-03	411E-02	Taldo1	0	Hyper
chr4	130014904	130016518	Distal Intergenic	0500	422E-03	411E-02	Arl6ip5	199429	Hyper
chr1	48553461	48554994	Intron (NM_133406/170919, intron 2 of 8)	0485	422E-03	411E-02	Agpat4	78229	Hyper
chr1	240950354	240951146	Distal Intergenic	0589	423E-03	412E-02		-3306	Hyper
chr12	35324722	35326515	Distal Intergenic	0474	423E-03	412E-02	Rpl6	24526	Hyper
chr3	60600158	60601209	Intron (NM_031789/83619, intron 1 of 4)	0479	423E-03	412E-02	Nfe2l2	20462	Hyper
chr9	42042103	42042868	Distal Intergenic	0548	424E-03	412E-02	Map4k4	-158752	Hyper
chr15	13271460	13273697	Intron (NM_134356/171357, intron 2 of 32)	0444	424E-03	413E-02	Ptprg	278489	Hyper
chr4	81858519	81859623	Intron (NM_001004199/246244, intron 12 of 17)	0621	425E-03	413E-02	Tax1bp1	36453	Hyper
chr4	119323543	119324839	Intron (NM_001173450/500244, intron 1 of 20)	0542	425E-03	413E-02	Aak1	22315	Hyper
chr3	13350205	13350948	Intron (NM_001047861/296619, intron 9 of 25)	0730	425E-03	413E-02	Pkn3	15068	Hyper
chr17	15274303	15288037	Exon (NM_001033674/306809, exon 2 of 7)	0239	425E-03	413E-02	Bicd2	16852	Hyper
chr10	43974770	43976074	Intron (NM_001013115/303179, intron 1 of 7)	0582	425E-03	413E-02	Guk1	3720	Hyper
chr18	27736803	27737508	Intron (NM_001136151/432361, intron 1 of 9)	0632	425E-03	413E-02	Nrg2	60997	Hyper
chr2	144782190	144782885	Intron (NM_001191566/282635, intron 2 of 7)	0693	426E-03	414E-02	Mbnl1	84357	Hyper
chr4	144430370	144432014	Exon (NM_031040/81672, exon 8 of 10)	0419	426E-03	414E-02	Grm7	699508	Hyper
chr18	67409033	67410353	Distal Intergenic	0550	426E-03	414E-02	Me2	-8046	Hyper
chr10	64556918	64560257	Intron (NM_001107023/303337, intron 1 of 14)	0346	427E-03	414E-02	Rab11fip4	6741	Hyper
chr1	86197968	86198744	Intron (NM_032616/64355, intron 2 of 9)	0553	427E-03	414E-02	Lsr	3208	Hyper
chr19	52877182	52879747	Distal Intergenic	0395	427E-03	414E-02	Sprtn	19314	Hyper
chr5	122829268	122830182	Intron (NM_138508/25541, intron 12 of 15)	0606	427E-03	414E-02	Scp2	51067	Hyper
chr7	112144981	112147553	Intron (NM_001034944/366962, intron 1 of 7)	0425	428E-03	415E-02	Grap2	27839	Hyper
chr16	67069232	67070631	Intron (NM_001014772/290834, intron 14 of 21)	0483	429E-03	415E-02	Adam32	-31804	Hyper
chr4	52992548	52994998	Promoter (<=1kb)	0376	429E-03	415E-02	Iqub	0	Hyper
chr1	84714624	84715699	Distal Intergenic	0611	430E-03	416E-02	Sipa1l3	-10822	Hyper
chr2	242623665	242624091	Intron (NM_019123/29758, intron 1 of 4)	0819	430E-03	416E-02	St6galnac3	20975	Hyper
chr19	34042381	34043281	Promoter (<=1kb)	0571	430E-03	416E-02	Esrp2	-655	Hyper
chr7	14549383	14550079	Distal Intergenic	0693	430E-03	416E-02	Angptl4	6440	Hyper
chr5	146476121	146476565	Promoter (2-3kb)	0876	430E-03	416E-02	Fam110d	-2102	Hyper

chr4	125187427	125187958	Intron (NM_001107876/312563, intron 1 of 7)	0648	432E-03	418E-02	Prickle2	26800	Hyper
chr7	130575895	130577333	Distal Intergenic	0498	432E-03	418E-02	Nckap5l	-8558	Hyper
chr10	62362264	62364462	Promoter (<=1kb)	0413	433E-03	418E-02	Abhd15	0	Hyper
chr14	46744788	46746631	Intron (NM_001107216/305367, intron 2 of 31)	0429	433E-03	418E-02	Arap2	26028	Hyper
chr3	13531382	13533751	Intron (NM_001024782/311846, intron 3 of 3)	0385	433E-03	418E-02	Phyhd1	-6658	Hyper
chr4	167819996	167820937	Promoter (1-2kb)	0660	433E-03	418E-02	Apold1	-1887	Hyper
chr6	100477285	100479444	Distal Intergenic	0430	434E-03	419E-02	Srsf5	-126037	Hyper
chr6	131674675	131675618	Distal Intergenic	0681	434E-03	419E-02	Siva1	-29821	Hyper
chr10	86792902	86794481	Distal Intergenic	0480	435E-03	419E-02	Meox1	43082	Hyper
chr20	18720066	18721210	Intron (NM_031805/361833, intron 22 of 44)	0591	435E-03	419E-02	Ank3	208919	Hyper
chr6	31371495	31373293	Distal Intergenic	0542	435E-03	419E-02	Rhob	-5572	Hyper
chr10	90800816	90802711	Exon (NM_001191653/303599, exon 13 of 26)	0355	435E-03	419E-02	Cyb561	85911	Hyper
chr7	129588810	129590701	Promoter (1-2kb)	0647	435E-03	419E-02	Or8s8	-1069	Hyper
chr7	8390661	8391543	Promoter (2-3kb)	0717	436E-03	419E-02	Tbxa2r	-2485	Hyper
chr1	46999031	46999779	Intron (NM_019357/54319, intron 1 of 12)	0718	436E-03	419E-02	Ezr	11726	Hyper
chr16	17571749	17572890	Distal Intergenic	0600	436E-03	419E-02	Ap1m1	29460	Hyper
chr18	12428567	12429090	Distal Intergenic	0783	436E-03	420E-02	Mep1b	62441	Hyper
chr1	107021783	107022947	Exon (NM_001107520/308669, exon 55 of 92)	0501	436E-03	420E-02	Oca2	-93331	Hyper
chr10	91476309	91477574	Distal Intergenic	0542	436E-03	420E-02	Ern1	-84430	Hyper
chr14	41598391	41601257	Distal Intergenic	0365	437E-03	420E-02	Uchl1	-102801	Hyper
chr19	23345705	23346231	Distal Intergenic	0938	437E-03	420E-02	Nfix	-9267	Hyper
chr1	95602082	95603368	Promoter (1-2kb)	0542	437E-03	420E-02	Mir150	-1656	Hyper
chr1	48549638	48552130	Intron (NM_133406/170919, intron 2 of 8)	0338	438E-03	421E-02	Agpat4	81093	Hyper
chr20	28194177	28198030	Distal Intergenic	0345	438E-03	421E-02	Psap	-16241	Hyper
chr4	104424972	104428448	Promoter (<=1kb)	0358	438E-03	421E-02	Vamp5	0	Hyper
chr1	133686374	133687788	Promoter (<=1kb)	0405	438E-03	421E-02	Pex11a	0	Hyper
chr11	67929257	67931057	Distal Intergenic	0403	438E-03	421E-02	Fyttd1	-28548	Hyper
chr2	62133715	62134338	Intron (NM_012927/25409, intron 2 of 15)	0699	438E-03	421E-02	Cdh6	90890	Hyper
chr3	145241788	145244555	Intron (NM_173145/286930, intron 6 of 11)	0381	439E-03	421E-02	Myl9	-40162	Hyper
chr18	51863930	51865421	Distal Intergenic	0426	439E-03	421E-02	Fbn2	-159954	Hyper
chr1	101087079	101088002	Distal Intergenic	0650	440E-03	422E-02	Slc17a6	-124487	Hyper
chr8	44898867	44900073	Distal Intergenic	0551	441E-03	422E-02	Ddx6	-31901	Hyper
chr10	4673320	4674201	Distal Intergenic	0750	441E-03	422E-02	Litaf	-8812	Hyper

chr19	582087	582677	Intron (NM_001172151/498902, intron 1 of 3)	0710	441E-03	423E-02	Cmtm4	5884	Hyper
chr14	73886325	73891872	Intron (NM_057116/117256, intron 1 of 8)	0287	441E-03	423E-02	Ppp2r2c	39306	Hyper
chr13	103992445	103995836	Intron (NM_031742/65198, intron 10 of 11)	0354	442E-03	423E-02	Kcnh1	270200	Hyper
chr10	63183500	63187670	Distal Intergenic	0284	442E-03	423E-02	Spag5	-11098	Hyper
chr2	220019216	220019759	Distal Intergenic	0772	442E-03	423E-02	Papss1	-20625	Hyper
chr5	70265768	70266662	Distal Intergenic	0564	442E-03	423E-02	Klf4	16667	Hyper
chr9	98444982	98446637	Distal Intergenic	0471	442E-03	423E-02	Macir	24222	Hyper
chr1	146259783	146261273	Intron (NM_022282/64053, intron 13 of 22)	0457	442E-03	423E-02	Ccdc90b	-371483	Hyper
chr10	18658845	18659267	Intron (NM_130421/155918, intron 3 of 20)	0805	442E-03	423E-02	Lcp2	16187	Hyper
chr14	104023501	104023943	Distal Intergenic	0853	443E-03	423E-02	Sptbn1	-14994	Hyper
chr16	7158232	7158998	Distal Intergenic	0642	443E-03	423E-02	Oxnad1	-134472	Hyper
chr5	136420969	136422450	Distal Intergenic	0449	444E-03	424E-02	Lnc081	30087	Hyper
chr16	44375093	44375652	Exon (NM_001109111/498630, exon 9 of 23)	0729	444E-03	424E-02	Cldn22	47702	Hyper
chr1	117864189	117866086	Intron (NM_001100846/309255, intron 2 of 15)	0450	444E-03	424E-02	Mtmr10	4834	Hyper
chr5	145654156	145655559	Distal Intergenic	0453	445E-03	425E-02	Tent5b	-39799	Hyper
chr9	105376557	105384632	Promoter (<=1kb)	0236	445E-03	425E-02	Rab31	0	Hyper
chr3	143030423	143031884	Intron (NM_001168542/296293, intron 8 of 10)	0502	445E-03	425E-02	C3h20orf144	-22251	Hyper
chr16	5065063	5067836	Distal Intergenic	0348	446E-03	426E-02	Selenok	-84230	Hyper
chr5	130798367	130800550	Distal Intergenic	0483	446E-03	426E-02	Tmem53	76232	Hyper
chr1	121248126	121250600	Promoter (2-3kb)	0411	446E-03	426E-02	Lrrc28	-2363	Hyper
chr14	36539101	36539530	Intron (NM_012956/25450, intron 3 of 8)	0650	446E-03	426E-02	Gabrb1	9410	Hyper
chr8	101707881	101708967	Intron (NM_001012202/363122, intron 12 of 12)	0565	447E-03	426E-02	Pccb	-66668	Hyper
chr9	106505491	106507746	Promoter (<=1kb)	0344	448E-03	427E-02	Rab12	0	Hyper
chr9	90046081	90047974	Distal Intergenic	0492	448E-03	427E-02	Agap1	-140051	Hyper
chr7	9035138	9037586	Promoter (2-3kb)	0313	448E-03	427E-02	Mknk2	-2185	Hyper
chr9	105338507	105339512	Intron (NM_145094/246324, intron 1 of 6)	0567	449E-03	427E-02	Rab31	41714	Hyper
chr1	220477296	220478607	Exon (NM_001191562/309407, exon 17 of 25)	0465	449E-03	427E-02	Mir204	160365	Hyper
chr8	29855921	29858497	Exon (NM_001108133/315523, exon 23 of 26)	0393	449E-03	428E-02	Nfrkb	24109	Hyper
chr3	135896675	135899159	Promoter (<=1kb)	0498	449E-03	428E-02	Cd93	0	Hyper
chr1	83381687	83382545	Intron (NM_001164657/100303643, intron 3 of 19)	0702	449E-03	428E-02	Fcgbp	6479	Hyper
chr19	53964207	53966610	Intron (NM_021688/59324, intron 1 of 2)	0410	449E-03	428E-02	Kcnk1	4550	Hyper
chr1	78522200	78523984	Distal Intergenic	0424	449E-03	428E-02	Pglyrp1	-7831	Hyper
chr1	83868458	83869708	Intron (NM_001106238/292756, intron 1 of 9)	0608	450E-03	428E-02	Pak4	19480	Hyper

chr20	11270267	11270945	Intron (NM_001111055/25367, intron 1 of 10)	0591	450E-03	428E-02	Adarb1	47672	Hyper
chr4	13829435	13830521	Exon (NM_001107845/311984, exon 20 of 26)	0576	450E-03	428E-02	Gsap	77293	Hyper
chr10	63968153	63968459	Exon (NM_001382488/108348076, exon 17 of 18)	1141	450E-03	428E-02	Lgals9	-38108	Hyper
chr1	183163842	183165336	Distal Intergenic	0462	450E-03	428E-02	Inpp5f	-25144	Hyper
chr17	51330117	51333207	Exon (NM_001012161/361255, exon 13 of 17)	0356	450E-03	428E-02	Tbce	39870	Hyper
chr18	64232276	64233132	Intron (NM_001115025/680172, intron 6 of 7)	0624	452E-03	429E-02	Mbd2	58274	Hyper
chr3	16162843	16166156	Distal Intergenic	0381	453E-03	430E-02	Niban2	-8518	Hyper
chr18	58553023	58554479	Intron (NM_001008300/291553, intron 2 of 30)	0516	454E-03	430E-02	Nedd4l	158046	Hyper
chr1	117468145	117468583	Distal Intergenic	0858	454E-03	430E-02	Klf13	68043	Hyper
chr6	99073145	99074262	Intron (NM_031005/81634, intron 1 of 20)	0605	454E-03	430E-02	Actn1	18925	Hyper
chr12	38497792	38500524	Intron (NM_001107145/304522, intron 6 of 10)	0413	455E-03	431E-02	Fbxw8	71293	Hyper
chr10	62697179	62703818	Intron (NM_001172137/360570, intron 2 of 41)	0279	455E-03	431E-02	Myo18a	42898	Hyper
chr6	123766722	123768232	Intron (NM_001106755/299285, intron 1 of 12)	0492	456E-03	431E-02	Clmn	39040	Hyper
chr9	14153301	14154613	Promoter (<=1kb)	0499	456E-03	431E-02	Bicral	0	Hyper
chr7	110358360	110360239	Promoter (<=1kb)	0534	456E-03	431E-02	Card10	0	Hyper
chr12	5360125	5361027	Distal Intergenic	0602	456E-03	432E-02	Hsph1	-29963	Hyper
chr4	58591271	58592673	Distal Intergenic	0522	456E-03	432E-02	Nrf1	-72317	Hyper
chr16	13037941	13038617	Distal Intergenic	0735	456E-03	432E-02	Ghitm	-140054	Hyper
chr9	947401	950043	Promoter (<=1kb)	0315	457E-03	433E-02	Lrg1	0	Hyper
chr17	51889240	51891048	Distal Intergenic	0472	458E-03	433E-02	Zeb1	224166	Hyper
chr10	35424217	35425550	Distal Intergenic	0596	458E-03	433E-02	Zfp354a	27448	Hyper
chr1	230096519	230099761	Intron (NM_181386/353229, intron 4 of 9)	0347	459E-03	433E-02	Asah2	-157357	Hyper
chr17	12279841	12280290	Promoter (<=1kb)	0844	460E-03	434E-02	Nfil3	-209	Hyper
chr5	72536888	72538849	Distal Intergenic	0374	460E-03	434E-02	Akap2	-65980	Hyper
chr6	26835823	26838624	Exon (NM_001003958/444984, exon 2 of 22)	0346	460E-03	434E-02	Dnmt3a	13214	Hyper
chr7	19756780	19757335	Distal Intergenic	0769	461E-03	434E-02	LOC100910996	300578	Hyper
chr12	42240160	42240556	Intron (NM_001009973/494521, intron 2 of 6)	0800	461E-03	434E-02	Kctd10	9880	Hyper
chr9	89660023	89663499	Promoter (<=1kb)	0325	461E-03	434E-02	Sh3bp4	0	Hyper
chr13	44873541	44876142	Promoter (<=1kb)	0369	461E-03	435E-02	Sox13	0	Hyper
chr20	10241045	10243775	Distal Intergenic	0412	463E-03	436E-02	Cstb	3730	Hyper
chr9	42212408	42213564	Intron (NM_001106904/301363, intron 1 of 28)	0528	463E-03	436E-02	Map4k4	10788	Hyper
chr14	65433034	65433844	Distal Intergenic	0423	463E-03	436E-02	Fam184b	-41645	Hyper
chr19	45007085	45007478	Intron (NM_001191786/315037, intron 2 of 3)	0830	464E-03	436E-02	Atmin	10579	Hyper

chr3	9352568	9354025	Distal Intergenic	0643	464E-03	437E-02	Notch1	-29037	Hyper
chr14	43107843	43108579	Promoter (1-2kb)	0694	465E-03	437E-02	Wdr19	-1555	Hyper
chr5	60966812	60969132	Exon (NM_031802/83633, exon 14 of 20)	0338	465E-03	437E-02	Tbc1d2	-35460	Hyper
chr20	19419763	19420415	Distal Intergenic	0652	465E-03	437E-02	Rhobtb1	-16751	Hyper
chr6	10731679	10735736	Distal Intergenic	0322	466E-03	437E-02	Haao	-110049	Hyper
chr3	152846825	152850558	3' UTR	0326	466E-03	438E-02	Kcns1	-3865	Hyper
chr5	160373002	160373378	Promoter (1-2kb)	0841	466E-03	438E-02	Spsb1	-1740	Hyper
chr1	155273454	155280295	Distal Intergenic	0331	466E-03	438E-02	Mir3102	-29562	Hyper
chr11	8731511	8731867	Promoter (2-3kb)	0806	466E-03	438E-02	Gbe1	-2995	Hyper
chr19	48326332	48327574	Distal Intergenic	0507	467E-03	438E-02	6430548M08Rikl	128123	Hyper
chr8	109497689	109508061	5' UTR	0246	467E-03	438E-02	Nckipsd	-4122	Hyper
chr4	119090986	119091722	Distal Intergenic	0745	467E-03	438E-02	Mxd1	26029	Hyper
chr3	19289332	19292280	Distal Intergenic	0371	468E-03	439E-02	Ndufa8	107179	Hyper
chr17	30584236	30585540	Intron (NM_022624/64559, intron 2 of 10)	0546	468E-03	439E-02	Slc22a23	40130	Hyper
chr17	17826459	17827535	Intron (NM_001107462/308173, intron 1 of 39)	0620	468E-03	439E-02	Kif13a	59862	Hyper
chr6	107683385	107685938	Intron (NM_053817/116508, intron 1 of 21)	0396	469E-03	439E-02	Nrxn3	41401	Hyper
chr20	12252482	12253851	Distal Intergenic	0627	469E-03	439E-02	Dip2a	-30803	Hyper
chr19	54705048	54706609	Distal Intergenic	0539	469E-03	440E-02	Tomm20	228579	Hyper
chr10	13032153	13034621	Promoter (<=1kb)	0389	470E-03	440E-02	Prss22	0	Hyper
chr2	182553403	182554948	Exon (NM_031083/81747, exon 3 of 13)	0517	470E-03	440E-02	Pi4kb	13026	Hyper
chr6	124886726	124887573	Distal Intergenic	0508	471E-03	441E-02	Vrk1	-27328	Hyper
chr7	107967362	107968311	Distal Intergenic	0591	472E-03	441E-02	Grina	5168	Hyper
chr10	91196188	91197160	Exon (NM_031983/83833, exon 5 of 13)	0644	472E-03	441E-02	Smarcd2	5459	Hyper
chr6	28183858	28186097	Intron (NM_001106713/298867, intron 3 of 13)	0454	473E-03	442E-02	Klhl29	204550	Hyper
chr3	79762291	79765268	Exon (NM_001107751/311215, exon 7 of 13)	0334	473E-03	442E-02	Ext2	32791	Hyper
chr10	103654352	103655717	Distal Intergenic	0427	473E-03	442E-02	Cant1	-4251	Hyper
chr20	6313568	6314979	Intron (NM_013141/25682, intron 2 of 7)	0453	473E-03	442E-02	Ppard	14783	Hyper
chr4	110313938	110316039	Intron (NM_001106598/297357, intron 1 of 18)	0382	474E-03	442E-02	Ctnna2	127374	Hyper
chr5	138571170	138573153	3' UTR	0430	474E-03	442E-02	Trappc3	11932	Hyper
chr4	125156617	125157311	Intron (NM_001107876/312563, intron 1 of 7)	0612	474E-03	442E-02	Prickle2	57447	Hyper
chr4	82427396	82429239	Intron (NM_001134621/500131, intron 2 of 11)	0411	474E-03	442E-02	Creb5	33666	Hyper
chr6	98734046	98735956	Distal Intergenic	0424	475E-03	443E-02	Zfp36l1	199792	Hyper
chr12	7297270	7298896	Promoter (<=1kb)	0555	475E-03	443E-02	Flt1	0	Hyper

chr3	140119869	140121506	Intron (NM_001106525/296267, intron 2 of 5)	0446	475E-03	443E-02	Snph	17836	Hyper
chr8	106840586	106842543	Promoter (2-3kb)	0348	475E-03	443E-02	Twf2	-2710	Hyper
chr6	25258109	25259604	Promoter (<=1kb)	0419	475E-03	443E-02	Trim54	0	Hyper
chr9	105451931	105452873	Distal Intergenic	0636	476E-03	444E-02	Ralbp1	39749	Hyper
chr1	96600198	96601139	Exon (NM_013039/25559, exon 35 of 39)	0599	477E-03	445E-02	Kcnj11	-6116	Hyper
chr4	123004796	123005178	Distal Intergenic	0912	478E-03	445E-02	Klf15	38989	Hyper
chr10	66777182	66777815	Intron (NM_001034014/25364, intron 1 of 9)	0687	479E-03	446E-02	Asic2	162762	Hyper
chr5	119036572	119037266	Distal Intergenic	0693	479E-03	446E-02	Dab1	-219762	Hyper
chr10	37910197	37912473	Distal Intergenic	0296	479E-03	446E-02	Irf1	-4691	Hyper
chrX	35198196	35199603	Intron (NM_053360/84357, intron 1 of 17)	0537	480E-03	447E-02	Sh3kbp1	23144	Hyper
chr2	108513716	108514857	Intron (NM_053868/116647, intron 4 of 6)	0519	481E-03	447E-02	Nlgn1	487074	Hyper
chr7	19646272	19646641	Distal Intergenic	0905	481E-03	447E-02	Nuak1	316238	Hyper
chr11	44726259	44728890	Promoter (<=1kb)	0285	483E-03	449E-02	Nxpe3	0	Hyper
chr12	44258931	44261932	Distal Intergenic	0349	484E-03	449E-02	Asphd2	10786	Hyper
chr8	122223651	122231301	Distal Intergenic	0260	484E-03	449E-02	Mir138-1	-69665	Hyper
chr17	27212743	27213264	Distal Intergenic	0762	484E-03	449E-02	LOC100909970	35926	Hyper
chr17	7152047	7153162	Intron (NM_001271297/306759, intron 6 of 10)	0580	484E-03	450E-02	Spock1	410534	Hyper
chr12	46418799	46419865	Intron (NM_001047901/360829, intron 14 of 14)	0564	484E-03	450E-02	Pgam5	9451	Hyper
chr16	18371954	18372532	Exon (NM_022861/64829, exon 5 of 43)	0694	485E-03	450E-02	Unc13a	9273	Hyper
chr1	28446497	28446955	Distal Intergenic	0798	485E-03	450E-02	Rnf146	-18210	Hyper
chr12	20805288	20806452	Distal Intergenic	0391	485E-03	450E-02	Hspb1	-9613	Hyper
chr3	165337495	165343316	Promoter (<=1kb)	0290	485E-03	450E-02	Phactr3	0	Hyper
chr5	142992413	142993462	Intron (NM_053893/116673, intron 3 of 4)	0598	485E-03	450E-02	Sdc3	26754	Hyper
chr20	9944826	9946791	Distal Intergenic	0408	485E-03	450E-02	Sik1	12148	Hyper
chr1	230045015	230047045	Intron (NM_181386/353229, intron 5 of 9)	0410	485E-03	450E-02	Asah2	-105853	Hyper
chr13	44547897	44549915	Exon (NM_001105951/289021, exon 26 of 32)	0417	485E-03	450E-02	Ppp1r15b	-28017	Hyper
chr2	164393702	164396044	Distal Intergenic	0328	487E-03	451E-02	Rapgef2	-71545	Hyper
chr5	135203929	135205165	Distal Intergenic	0520	487E-03	451E-02	Mfsd2a	35525	Hyper
chr1	95508531	95509475	Distal Intergenic	0638	487E-03	451E-02	Rras	7949	Hyper
chr8	120159446	120162282	Intron (NM_182844/360034, intron 10 of 15)	0359	487E-03	451E-02	Eif1b	-41279	Hyper
chr2	153626839	153628688	Intron (NM_001107681/310506, intron 1 of 3)	0420	488E-03	452E-02	Ppm1l	60186	Hyper
chr7	51866855	51867431	Intron (NM_053594/94202, intron 6 of 13)	0692	488E-03	452E-02	Ptprr	66656	Hyper
chr7	108495911	108505653	Intron (NM_173122/500901, intron 1 of 11)	0275	488E-03	452E-02	Arhgap39	33178	Hyper

chr1	234195077	234195972	Distal Intergenic	0629	489E-03	452E-02	Hectd2	-79733	Hyper
chr8	20255257	20257379	Exon (NM_134368/171379, exon 30 of 34)	0463	490E-03	453E-02	Ldlr	-12717	Hyper
chr7	110326802	110329284	Promoter (<=1kb)	0398	490E-03	453E-02	Mfng	0	Hyper
chr12	15338274	15340828	Intron (NM_133567/171097, intron 1 of 10)	0398	490E-03	453E-02	Adap1	14037	Hyper
chr3	152885915	152889108	Promoter (1-2kb)	0470	490E-03	453E-02	Wfdc15b	1492	Hyper
chr1	151851330	151853262	Intron (NM_001191629/308837, intron 22 of 22)	0385	490E-03	453E-02	Aamdc	38782	Hyper
chr7	130086337	130088293	Distal Intergenic	0448	490E-03	453E-02	Tuba1b	5351	Hyper
chr7	58587379	58592240	Intron (NM_001271079/314897, intron 9 of 9)	0299	491E-03	453E-02	Mirlet7i	50329	Hyper
chr11	80539152	80539570	Intron (NM_053924/116721, intron 17 of 29)	0902	491E-03	453E-02	Parl	-54507	Hyper
chr12	45983050	45983489	Exon (NM_001107149/304569, exon 39 of 52)	0836	491E-03	454E-02	Ddx51	32506	Hyper
chr10	52876659	52877226	Intron (NM_031656/59074, intron 7 of 7)	0720	492E-03	454E-02	Ntn1	207093	Hyper
chr1	154288691	154290786	Distal Intergenic	0424	492E-03	454E-02	Chrdl2	-52415	Hyper
chr5	101199855	101201010	Distal Intergenic	0468	493E-03	455E-02	Plin2	-16819	Hyper
chr4	125190843	125194781	Intron (NM_001107876/312563, intron 1 of 7)	0358	494E-03	455E-02	Prickle2	19977	Hyper
chr1	220023578	220024538	Intron (NM_001191562/309407, intron 1 of 24)	0614	495E-03	456E-02	Trpm3	14608	Hyper
chr3	154080160	154082948	Distal Intergenic	0385	496E-03	457E-02	Elmo2	-19003	Hyper
chr18	37072010	37074855	Intron (NM_001170534/307449, intron 3 of 18)	0279	497E-03	457E-02	Mcc	180183	Hyper
chr16	18696186	18699286	Exon (NM_001276765/290646, exon 3 of 16)	0433	497E-03	457E-02	Rab3a	-7889	Hyper
chr8	64753554	64755261	Promoter (<=1kb)	0316	497E-03	457E-02	Map2k1	0	Hyper
chr2	152768044	152769199	Intron (NM_001100666/295105, intron 5 of 10)	0486	498E-03	457E-02	Il12a	-196570	Hyper
chr17	81564394	81565720	Exon (NM_053926/116723, exon 3 of 10)	0510	498E-03	457E-02	Pip4k2a	102309	Hyper
chr2	29554400	29555629	Distal Intergenic	0542	498E-03	457E-02	Arhgef28	-25800	Hyper
chr8	65893568	65896284	Distal Intergenic	0400	498E-03	457E-02	Rasl12	-21556	Hyper
chr5	162588297	162589602	Intron (NM_201272/310999, intron 1 of 21)	0445	498E-03	458E-02	Plekhg5	10226	Hyper
chr4	104434124	104435464	Distal Intergenic	0462	499E-03	458E-02	Vamp5	-9064	Hyper
chr20	1523627	1524960	3' UTR	0537	499E-03	458E-02	Zfp57	4717	Hyper
chr10	100853757	100855618	Promoter (2-3kb)	0485	499E-03	458E-02	Mif4gd	-2124	Hyper
chr1	163543691	163545256	3' UTR	0478	499E-03	458E-02	Rpl27a	3938	Hyper
chr10	85627953	85628996	Promoter (2-3kb)	0527	500E-03	458E-02	Dhx58	2529	Hyper
chr15	45234384	45235018	Promoter (1-2kb)	0755	501E-03	459E-02	C15h8orf58	-1130	Hyper
chr11	29944273	29944994	Distal Intergenic	0713	501E-03	459E-02	Mis18a	36064	Hyper
chr1	19957717	19958384	Distal Intergenic	0686	501E-03	459E-02	Smlr1	120327	Hyper
chr2	31103428	31103969	Distal Intergenic	0629	501E-03	459E-02	Cartpt	153151	Hyper

chr15	38110412	38110664	Intron (NM_001083337/305963, intron 5 of 6)	0935	501E-03	459E-02	Pinx1	12385	Hyper
chr1	47660397	47660954	Distal Intergenic	0461	502E-03	460E-02	Wtap	-5011	Hyper
chr10	73812409	73814094	Promoter (<=1kb)	0495	503E-03	460E-02	Trim25	0	Hyper
chr7	29156932	29158043	Intron (NM_001108084/314751, intron 1 of 3)	0621	503E-03	460E-02	Tmcc3	56757	Hyper
chr11	84727172	84728110	Promoter (<=1kb)	0529	503E-03	461E-02	Pkp2	0	Hyper
chr10	42418635	42419058	Distal Intergenic	0820	504E-03	461E-02	Igtp	44045	Hyper
chr13	74815983	74817137	Intron (NM_138538/171574, intron 1 of 20)	0534	504E-03	461E-02	Dnm3	20971	Hyper
chr1	109560305	109562659	Intron (NM_001141935/365266, intron 2 of 21)	0408	504E-03	461E-02	Atp10a	3523	Hyper
chr5	166781142	166783226	Promoter (2-3kb)	0340	505E-03	461E-02	Isg15	2209	Hyper
chr9	94308980	94311629	Exon (NM_001025711/316640, exon 3 of 7)	0362	505E-03	462E-02	Atg4b	4025	Hyper
chr7	10011769	10012765	Exon (NM_057183/29252, exon 3 of 5)	0625	506E-03	462E-02	Gzmm	3873	Hyper
chr1	12033222	12033927	Distal Intergenic	0515	506E-03	462E-02	Cited2	-278499	Hyper
chr1	199693926	199695986	Promoter (<=1kb)	0461	507E-03	462E-02		-220	Hyper
chr10	39523637	39524123	Exon (NM_012656/24791, exon 5 of 10)	0780	507E-03	462E-02	Sparc	14026	Hyper
chr6	106309900	106310724	Distal Intergenic	0510	507E-03	463E-02	Angel1	63983	Hyper
chr13	76253308	76253897	Intron (NM_001105964/289168, intron 18 of 19)	0636	507E-03	463E-02	Scyl3	-24531	Hyper
chr1	256094439	256094962	Exon (NM_001044394/307989, exon 11 of 18)	0705	508E-03	463E-02	Afap1l2	-33619	Hyper
chr2	34232499	34233444	Distal Intergenic	0621	509E-03	464E-02	Cd180	376508	Hyper
chr7	130431179	130434426	Distal Intergenic	0273	509E-03	464E-02	Prpf40b	-5382	Hyper
chr2	173454552	173455154	3' UTR	0738	509E-03	464E-02	Bcan	12306	Hyper
chr7	57138639	57139692	Exon (NM_001108102/314879, exon 7 of 18)	0729	509E-03	464E-02	Xpot	12155	Hyper
chr3	149467786	149469390	Distal Intergenic	0476	509E-03	464E-02	Zhx3	-31197	Hyper
chr3	15008838	15009242	Intron (NM_001100850/311860, intron 1 of 10)	0776	510E-03	465E-02	Abl1	28985	Hyper
chr5	136431688	136433985	Distal Intergenic	0378	510E-03	465E-02	Lnc081	40806	Hyper
chr14	75775881	75776578	Intron (NM_019339/54292, intron 2 of 15)	0741	510E-03	465E-02	Rgs12	18018	Hyper
chr10	81909789	81910468	Distal Intergenic	0768	510E-03	465E-02	Cdk5rap3	-6234	Hyper
chr3	17106476	17108537	Intron (NM_001271232/362118, intron 7 of 9)	0427	512E-03	466E-02	Mvb12b	84121	Hyper
chr5	160373817	160377952	Promoter (2-3kb)	0358	512E-03	466E-02	Spsb1	-2555	Hyper
chr19	50627023	50629031	Promoter (<=1kb)	0302	512E-03	466E-02	Aprt	0	Hyper
chr2	78591964	78593093	Exon (NM_001107658/310192, exon 32 of 58)	0566	513E-03	466E-02	Otulinl	-158139	Hyper
chr10	99866728	99867740	Distal Intergenic	0562	514E-03	467E-02	Gprc5c	-19981	Hyper
chr15	34777822	34781138	Promoter (<=1kb)	0326	514E-03	467E-02	Spata13	0	Hyper
chr5	116207614	116209164	Intron (NM_001107949/313409, intron 1 of 17)	0473	514E-03	467E-02	Dnajc6	76938	Hyper

chr1	201075852	201080326	Promoter (<=1kb)	0246	514E-03	467E-02	Chka	0	Hyper
chr11	69702927	69703822	Distal Intergenic	0578	515E-03	467E-02	Acap2	164381	Hyper
chr17	45896373	45899413	Intron (NM_022217/60668, intron 1 of 20)	0319	515E-03	467E-02	Amph	83394	Hyper
chr5	154776457	154780085	Promoter (<=1kb)	0276	515E-03	467E-02	Kazn	0	Hyper
chr3	141251904	141270721	3' UTR	0239	515E-03	467E-02	Cox4i2	23461	Hyper
chr1	83847276	83847689	Distal Intergenic	0689	516E-03	468E-02	Nccrp1	-8600	Hyper
chr6	130947206	130949026	Distal Intergenic	0442	516E-03	468E-02	Ppp1r13b	-17557	Hyper
chr10	100649742	100652818	3' UTR	0347	516E-03	468E-02	Mrpl58	3368	Hyper
chr1	187468081	187469047	Intron (NM_001009706/361663, intron 6 of 6)	0590	516E-03	468E-02	Lhpp	64630	Hyper
chr1	19969704	19970622	Distal Intergenic	0642	517E-03	468E-02	Akap7	-116868	Hyper
chr20	44988776	44990332	Promoter (<=1kb)	0385	517E-03	468E-02	Smpd2	0	Hyper
chr14	103897228	103902162	Exon (NM_001013130/305614, exon 4 of 39)	0308	518E-03	469E-02	Sptbn1	106345	Hyper
chr12	11048064	11057055	3' UTR	0273	518E-03	469E-02	Rac1	11036	Hyper
chr10	57140622	57141519	Intron (NM_001109035/497946, intron 7 of 10)	0546	518E-03	469E-02	Spns3	27242	Hyper
chr13	38023665	38024336	Distal Intergenic	0607	518E-03	469E-02	Mgat5	-651783	Hyper
chr8	110060819	110064439	3' UTR	0394	518E-03	469E-02	Dhx30	32390	Hyper
chr6	104181755	104183640	Distal Intergenic	0351	519E-03	470E-02	Vsx2	-33590	Hyper
chr3	147034937	147036960	3' UTR	0451	519E-03	470E-02	Snhg11	3526	Hyper
chr2	185528150	185533637	Distal Intergenic	0276	519E-03	470E-02	Sec22b	28121	Hyper
chr5	68148928	68150166	Intron (NM_001033852/85254, intron 4 of 15)	0483	520E-03	470E-02	Slc44a1	85310	Hyper
chr2	110100981	110103420	Intron (NM_001127524/294930, intron 1 of 4)	0368	520E-03	470E-02	Nceh1	25996	Hyper
chr12	34277341	34278670	Intron (NM_001107141/304488, intron 1 of 5)	0490	520E-03	470E-02	Pptc7	9669	Hyper
chr3	19026402	19028484	Intron (NM_138710/192126, intron 3 of 15)	0427	521E-03	471E-02	Dab2ip	111112	Hyper
chr15	37194102	37194521	Intron (NM_001107269/305956, intron 6 of 10)	0838	521E-03	471E-02	Defb42	47047	Hyper
chr19	15922197	15924275	Promoter (<=1kb)	0298	522E-03	472E-02	Rbl2	0	Hyper
chr2	32951869	32952843	Promoter (<=1kb)	0554	522E-03	472E-02	Pik3r1	364	Hyper
chr10	72526674	72528829	Intron (NM_001135921/303412, intron 2 of 8)	0432	522E-03	472E-02	Supt4h1	-10562	Hyper
chr5	131679971	131695988	Exon (NM_001107966/313539, exon 11 of 21)	0241	523E-03	472E-02	St3gal3	-9676	Hyper
chr8	48749692	48750222	Distal Intergenic	0734	523E-03	472E-02	Nxpe1	-12350	Hyper
chr4	123219165	123220506	Distal Intergenic	0540	523E-03	472E-02	Slc41a3	91402	Hyper
chr20	5840285	5850095	Exon (NM_001039607/294154, exon 3 of 5)	0262	523E-03	472E-02	Ilrun	25917	Hyper
chr12	22464466	22468195	Exon (NM_001001512/353256, exon 17 of 31)	0325	523E-03	472E-02	Ncf1	-17256	Hyper
chr1	127081718	127082880	Distal Intergenic	0572	525E-03	473E-02	Rgma	-46054	Hyper

chr15	50201874	50204524	Intron (NM_001134727/502020, intron 1 of 18)	0404	525E-03	473E-02	Lrch1	45133	Hyper
chr12	35193740	35195836	Distal Intergenic	0385	525E-03	473E-02	Trafd1	28134	Hyper
chr2	231275070	231276454	Distal Intergenic	0426	525E-03	473E-02	Gbp4	-28790	Hyper
chr15	50245137	50245506	Intron (NM_001134727/502020, intron 1 of 18)	0883	525E-03	473E-02	Lrch1	4151	Hyper
chr1	187466507	187467835	Intron (NM_001009706/361663, intron 6 of 6)	0478	526E-03	473E-02	Lhpp	63056	Hyper
chr11	79693148	79696967	Distal Intergenic	0304	526E-03	473E-02	Ephb3	162403	Hyper
chr19	52884115	52885815	Distal Intergenic	0430	527E-03	474E-02	Sprtn	26247	Hyper
chr10	5891891	5893754	Intron (NM_012573/24409, intron 6 of 15)	0413	527E-03	474E-02	Grin2a	261207	Hyper
chr6	106943648	106945147	Distal Intergenic	0462	527E-03	474E-02	Ahsa1	30109	Hyper
chr5	132683946	132684414	Distal Intergenic	0735	528E-03	475E-02	Slc2a1	-32785	Hyper
chr19	11937699	11940801	Intron (NM_001108439/361368, intron 1 of 12)	0337	528E-03	475E-02	Large1	108129	Hyper
chr2	30781139	30782696	Intron (NM_001108543/361883, intron 4 of 4)	0430	528E-03	475E-02	Mrps27	41106	Hyper
chr10	13622173	13623328	Exon (NM_012680/24855, exon 37 of 42)	0566	528E-03	475E-02	Tsc2	32368	Hyper
chr2	32954561	32957359	Promoter (1-2kb)	0328	529E-03	475E-02	Pik3r1	-1354	Hyper
chr5	145328520	145332330	Distal Intergenic	0311	529E-03	475E-02	Wasf2	-46245	Hyper
chr1	164889365	164892559	Intron (NM_031544/25095, intron 1 of 14)	0270	529E-03	475E-02	Ampd3	4045	Hyper
chr10	85651721	85654019	Intron (NM_001105840/287709, intron 1 of 5)	0435	531E-03	476E-02	Hspb9	10437	Hyper
chr1	28396762	28397373	Distal Intergenic	0648	531E-03	476E-02	Rnf146	-67792	Hyper
chr1	231798982	231799452	Promoter (<=1kb)	0758	531E-03	476E-02	Fas	19	Hyper
chr15	40455049	40467302	Intron (NM_017318/50646, intron 1 of 30)	0249	531E-03	476E-02	Ptk2b	13802	Hyper
chr14	83184867	83190308	Exon (NM_001170459/360980, exon 23 of 29)	0278	531E-03	477E-02	Tns3	153830	Hyper
chr4	151495873	151497688	Distal Intergenic	0461	532E-03	477E-02	Bms1	97439	Hyper
chr1	213559182	213559865	Intron (NM_031036/81666, intron 2 of 6)	0641	533E-03	477E-02	Gnaq	133456	Hyper
chr6	88535730	88536262	Exon (NM_001106737/299117, exon 29 of 30)	0873	534E-03	478E-02	Sav1	-35609	Hyper
chr6	43011994	43023732	Promoter (<=1kb)	0236	535E-03	479E-02	Rnf144a	0	Hyper
chr8	57307417	57308202	Distal Intergenic	0710	535E-03	479E-02	Snx33	19336	Hyper
chr18	47222220	47223293	Distal Intergenic	0518	535E-03	479E-02	Cep120	-32256	Hyper
chr18	26502690	26505023	3' UTR	0338	535E-03	479E-02	Etf1	25730	Hyper
chr1	256913075	256914547	Distal Intergenic	0521	535E-03	479E-02	Trub1	397513	Hyper
chr5	141151377	141154572	Distal Intergenic	0302	535E-03	479E-02	Zfp362	-9636	Hyper
chr5	156834226	156839721	Exon (NM_001108006/313825, exon 68 of 70)	0263	536E-03	479E-02	Dhrs3	85607	Hyper
chr10	63089681	63090612	Promoter (<=1kb)	0548	536E-03	479E-02	Proca1	0	Hyper
chr7	100978659	100979725	Intron (NM_022249/64015, intron 7 of 8)	0545	536E-03	479E-02	Khdrbs3	140725	Hyper

chr19	48203689	48205439	Exon (NM_001107436/307907, exon 6 of 13)	0475	536E-03	480E-02	6430548M08Rikl	5480	Hyper
chr8	121687940	121689282	Distal Intergenic	0580	536E-03	480E-02	Pomgnt2	-40664	Hyper
chr10	105698056	105698623	Intron (NM_080577/140639, intron 3 of 16)	0715	537E-03	480E-02	Nploc4	11290	Hyper
chr17	6941438	6943555	Intron (NM_001271297/306759, intron 2 of 10)	0415	537E-03	480E-02	Spock1	199925	Hyper
chr9	60786933	60787686	Distal Intergenic	0617	537E-03	480E-02	Als2	-101400	Hyper
chr1	86122669	86123810	Exon (NM_001107503/308501, exon 8 of 15)	0619	538E-03	481E-02	Cd22	8512	Hyper
chr6	27911953	27913250	Promoter (<=1kb)	0377	538E-03	481E-02	Ubxn2a	0	Hyper
chr3	9464992	9467763	Distal Intergenic	0278	538E-03	481E-02	Dipk1b	8583	Hyper
chr1	119092711	119094925	Distal Intergenic	0291	539E-03	481E-02	Tars3	-118304	Hyper
chr17	7056540	7058173	Intron (NM_001271297/306759, intron 3 of 10)	0501	539E-03	481E-02	Spock1	315027	Hyper
chr1	83485852	83487410	3' UTR	0521	539E-03	481E-02	Dyrk1b	6705	Hyper
chr13	105867233	105868871	Distal Intergenic	0396	540E-03	482E-02	Plxna2	-294232	Hyper
chr10	82425201	82428280	Distal Intergenic	0387	540E-03	482E-02	Mrpl45	116551	Hyper
chr2	24557653	24560508	Intron (NM_031707/29546, intron 1 of 9)	0370	540E-03	482E-02	Homer1	13653	Hyper
chr1	15271439	15272476	Intron (NM_080894/140929, intron 2 of 12)	0537	540E-03	482E-02	Mtfr2	148207	Hyper
chr15	21330673	21334308	Distal Intergenic	0307	540E-03	482E-02	Peli2	-58954	Hyper
chr3	36720730	36722189	Distal Intergenic	0433	541E-03	482E-02	Arl5a	181173	Hyper
chr5	142936237	142937401	Intron (NM_001108684/362609, intron 16 of 21)	0558	542E-03	483E-02	Sdc3	-28258	Hyper
chrX	61543329	61544484	Promoter (<=1kb)	0500	542E-03	483E-02	Gpr165	0	Hyper
chr18	30879722	30880323	Intron (NM_001107389/307459, intron 1 of 23)	0629	543E-03	484E-02	Arhgap26	41051	Hyper
chr1	80821177	80821527	Exon (NM_017344/50686, exon 4 of 14)	0870	544E-03	484E-02	Gsk3a	4164	Hyper
chr6	95350664	95351359	Exon (NM_212522/314251, exon 14 of 32)	0707	544E-03	484E-02	Sptb	85759	Hyper
chr17	14851216	14851647	Distal Intergenic	0719	544E-03	484E-02	Iars1	135570	Hyper
chr19	27137984	27141159	Intron (NM_001108444/361388, intron 1 of 9)	0363	545E-03	485E-02	Gab1	6722	Hyper
chr10	14617592	14624758	Distal Intergenic	0263	546E-03	485E-02	Sox8	-27774	Hyper
chr14	55800787	55801308	Distal Intergenic	0735	546E-03	485E-02	Stim2	1202871	Hyper
chr16	74650591	74652642	Distal Intergenic	0386	546E-03	485E-02	Kbtbd11	-27703	Hyper
chr11	77715632	77716353	Distal Intergenic	0638	546E-03	485E-02	Adipoq	19211	Hyper
chr18	24784813	24788331	Intron (NM_012727/25050, intron 9 of 12)	0374	546E-03	486E-02	Stard4	41693	Hyper
chr7	132239105	132240973	Distal Intergenic	0490	547E-03	486E-02	Acvrl1	-4538	Hyper
chr5	150156427	150158609	Intron (NM_053596/94204, intron 8 of 18)	0391	547E-03	486E-02	Ece1	78748	Hyper
chr3	117964333	117964985	Intron (NM_001109206/499891, intron 20 of 36)	0643	547E-03	486E-02	Slc4a11	-51659	Hyper
chr9	13318514	13320498	5' UTR	0352	548E-03	486E-02	Frs3	-23132	Hyper

chr1	12663664	12665609	Promoter (<=1kb)	0295	548E-03	486E-02	Abracl	0	Hyper
chr1	44483928	44485247	Distal Intergenic	0452	550E-03	487E-02	Nox3	-181077	Hyper
chr15	28021476	28022195	Exon (NM_053503/85265, exon 7 of 8)	0643	550E-03	488E-02	Ajuba	7826	Hyper
chr6	24890329	24891162	Distal Intergenic	0704	551E-03	488E-02	Slc4a1ap	42444	Hyper
chr19	33142290	33142904	Promoter (<=1kb)	0671	551E-03	488E-02	Fbxl8	0	Hyper
chr6	27374160	27375232	Intron (NM_001108012/313929, intron 3 of 24)	0622	552E-03	489E-02	Ncoa1	100432	Hyper
chr5	149122472	149124352	Promoter (<=1kb)	0438	553E-03	489E-02	C1qb	40	Hyper
chr1	106739382	106739999	Intron (NM_001107517/308666, intron 1 of 30)	0667	553E-03	489E-02	Cyfip1	28334	Hyper
chr5	144943813	144944763	Promoter (<=1kb)	0565	553E-03	489E-02	Xkr8	432	Hyper
chr16	19522811	19527413	Promoter (<=1kb)	0252	554E-03	490E-02	Tssk6	0	Hyper
chr5	158517566	158519625	Promoter (<=1kb)	0336	555E-03	490E-02	Agtrap	0	Hyper
chr6	121784161	121786554	Intron (NM_001191985/500709, intron 2 of 9)	0313	555E-03	490E-02	Itpk1	57484	Hyper
chr3	58304422	58305254	Distal Intergenic	0646	555E-03	490E-02	Gpr155	-34147	Hyper
chr12	35216138	35217729	Distal Intergenic	0461	556E-03	491E-02	Trafd1	50532	Hyper
chr7	27699400	27700085	Intron (NM_001108082/314743, intron 1 of 16)	0587	556E-03	491E-02	Cdk17	15510	Hyper
chr12	19363565	19365251	Distal Intergenic	0409	557E-03	491E-02	Slc12a9	-3819	Hyper
chr7	11235685	11236842	Exon (NM_001100903/362844, exon 8 of 19)	0546	558E-03	492E-02	Ephx3	-23563	Hyper
chr1	76716610	76718034	Distal Intergenic	0440	558E-03	492E-02	Bicra	-30223	Hyper
chr3	58250066	58250322	Intron (NM_001107811/311730, intron 7 of 15)	0893	558E-03	492E-02	Gpr155	19953	Hyper
chr10	54601364	54602441	Promoter (<=1kb)	0603	558E-03	492E-02	Tmem95	0	Hyper
chr9	14429821	14431349	Exon (NM_001109302/501099, exon 3 of 7)	0444	558E-03	492E-02	Srf	3349	Hyper
chr2	224462312	224463700	Distal Intergenic	0458	559E-03	492E-02	Slc39a8	205658	Hyper
chr3	77653441	77654424	Exon (NM_001271212/362164, exon 18 of 23)	0535	559E-03	493E-02	Atg13	26604	Hyper
chrX	2425241	2426102	Intron (NM_207600/302302, intron 1 of 1)	0621	560E-03	493E-02	Chst7	6726	Hyper
chr3	90458580	90461716	Promoter (<=1kb)	0282	560E-03	493E-02	Cd59b	0	Hyper
chr15	16809181	16812326	Intron (NM_182821/306203, intron 1 of 17)	0367	561E-03	494E-02	Pxk	16096	Hyper
chr4	123438174	123439577	Distal Intergenic	0470	561E-03	494E-02	Nup210	170297	Hyper
chr6	13467259	13468350	Intron (NM_001270772/29715, intron 1 of 6)	0515	561E-03	494E-02	Slc8a1	196038	Hyper
chr17	21761130	21762319	Intron (NM_214457/306844, intron 3 of 13)	0524	561E-03	494E-02	Tbc1d7	230136	Hyper
chr2	78589052	78591792	Exon (NM_001107658/310192, exon 33 of 58)	0423	561E-03	494E-02	Otulinl	-155227	Hyper
chr16	17475892	17477719	Intron (NM_001029921/361120, intron 21 of 23)	0470	562E-03	495E-02	Klf2	46004	Hyper
chr4	104277666	104280729	Exon (NM_001109241/500200, exon 2 of 3)	0432	562E-03	495E-02	Atoh8	11439	Hyper
chr17	4278037	4278940	Distal Intergenic	0549	562E-03	495E-02	Dapk1	-187046	Hyper

chr3	123473552	123473930	Intron (NM_001107781/311450, intron 4 of 10)	0874	563E-03	495E-02	Lamp5	101090	Hyper
chr7	113175265	113176383	Distal Intergenic	0544	564E-03	495E-02	L3mbtl2	-10034	Hyper
chr7	115391784	115394264	Intron (NM_001134780/362973, intron 1 of 12)	0424	564E-03	495E-02	Parvb	31530	Hyper
chr1	79194797	79197092	Intron (NM_001107482/308398, intron 1 of 12)	0431	564E-03	495E-02	Ppp1r37	15520	Hyper
chr20	43011040	43012677	Promoter (<=1kb)	0413	564E-03	496E-02	Traf3ip2	0	Hyper
chr1	153696511	153697357	Intron (NM_001109152/499211, intron 2 of 17)	0633	564E-03	496E-02	Gdpd5	16793	Hyper
chr10	103479163	103480533	Intron (NM_053910/116691, intron 1 of 12)	0435	565E-03	496E-02	Cyth1	5227	Hyper
chr16	75371082	75371594	Intron (NM_053901/116681, intron 2 of 15)	0825	566E-03	497E-02	Dlgap2	124786	Hyper
chr8	119837962	119839674	Promoter (2-3kb)	0424	566E-03	497E-02	Slc25a38	2416	Hyper
chr10	6781945	6782406	Distal Intergenic	0762	567E-03	497E-02	Hapstr1	-6953	Hyper
chr6	95557032	95557795	Intron (NM_172034/64511, intron 2 of 11)	0569	567E-03	498E-02	Fntb	20445	Hyper
chr20	7133343	7134711	Distal Intergenic	0488	568E-03	498E-02	Cdkn1a	-14575	Hyper
chr16	76594991	76597204	Intron (NM_053951/117020, intron 1 of 30)	0399	568E-03	498E-02	Mcf2l	15378	Hyper
chr2	184086095	184086405	Distal Intergenic	0875	569E-03	498E-02	Txnip	-6674	Hyper
chr1	163879721	163880486	Distal Intergenic	0677	569E-03	499E-02	Nrip3	-64685	Hyper
chr4	157651931	157653090	Intron (NM_001106620/297595, intron 8 of 13)	0516	569E-03	499E-02	Gnb3	-6760	Hyper
chr3	117475976	117478197	Promoter (<=1kb)	0275	569E-03	499E-02	Nop56	0	Hyper
chr1	204175387	204175947	Promoter (1-2kb)	0641	570E-03	499E-02	Vegfb	1997	Hyper
chr1	134880418	134880806	Distal Intergenic	0798	570E-03	499E-02	Nmb	-8228	Hyper
chr7	100979799	100980723	Intron (NM_022249/64015, intron 7 of 8)	0533	570E-03	499E-02	Khdrbs3	141865	Hyper
chr8	62841472	62842782	Intron (NM_012903/25379, intron 1 of 6)	0497	571E-03	500E-02	Anp32a	12347	Hyper
chr17	66996118	66997450	Distal Intergenic	0537	571E-03	500E-02	Pfkfb3	-37645	Hyper
chr3	130045554	130048492	Intron (NM_001107783/311478, intron 22 of 25)	0312	571E-03	500E-02	Kif16b	205527	Hyper
chr2	191691074	191691830	Intron (NM_139084/245903, intron 1 of 20)	0574	571E-03	500E-02	Magi3	24895	Hyper
chr14	81956251	81960996	Exon (NM_001107239/305509, exon 4 of 20)	0269	571E-03	500E-02	Adcy1	45011	Hyper

Supplementary Table 4: Significantly hypoacetylated peaks in the cortex

chr	*start*	*end*	*annotation*	*logFC*	*PValue*	*FDR*	*GENE*	*distanceToTSS*	*DAR*
chr2	14933183	14937114	Distal Interger	-1097	156E-13	252E-09	Mir3597-2	-735355	Hypo
chr5	83961006	83963492	Promoter (<=1	-1185	427E-13	492E-09	Cdk5rap2	-224	Hypo
chr1	130960540	130962583	Distal Interger	-1122	164E-12	147E-08	Klhl25	1235590	Hypo
chr15	55412947	55414781	Intron (NM_0(	-1391	108E-11	528E-08	Olfm4	5799	Hypo
chr11	15873474	15875843	Distal Interger	-1122	133E-11	567E-08	Usp25	269593	Hypo
chr16	26640201	26641932	Intron (NM_0(	-1391	216E-11	823E-08	Spock3	173673	Hypo
chr1	195850942	195853646	3' UTR	-1543	224E-11	823E-08	Syce1	9528	Hypo
chr9	15258795	15261887	Distal Interger	-1109	315E-11	111E-07	Mrpl14	46532	Hypo
chr18	47586560	47589233	Distal Interger	-0971	903E-11	261E-07	Csnk1g3	286981	Hypo
chr1	58129148	58132332	Promoter (<=1	-0905	118E-10	307E-07	Lix1	187	Hypo
chr6	19955499	19963340	Promoter (<=1	-0716	141E-10	346E-07	Rasgrp3	0	Hypo
chr2	117312032	117319158	Distal Interger	-0735	279E-10	563E-07	Sox2	-217771	Hypo
chr5	76243956	76245912	Distal Interger	-1284	366E-10	629E-07	Rgs3	165464	Hypo
chr10	24072936	24075594	Distal Interger	-0943	508E-10	821E-07	Mat2b	1044901	Hypo
chr1	137631383	137633403	Intron (NM_0(	-1002	110E-09	148E-06	Stard5	25232	Hypo
chr7	90729183	90731317	Distal Interger	-0980	116E-09	154E-06	Mtss1	-101341	Hypo
chr2	62269894	62272029	Distal Interger	-1068	129E-09	162E-06	Cdh6	-44666	Hypo
chr5	135125897	135128623	Exon (NM_02	-1078	212E-09	243E-06	Ppt1	4721	Hypo
chr1	144146533	144149466	Distal Interger	-0825	268E-09	282E-06	Picalm	89772	Hypo
chr19	56614011	56615380	Distal Interger	-1182	282E-09	287E-06	Itgb1	-89825	Hypo
chr17	1190611	1194862	Distal Interger	-0706	284E-09	287E-06	Hsd17b3	163382	Hypo
chr3	17246569	17249117	Distal Interger	-0748	343E-09	334E-06	Mvb12b	-53911	Hypo
chr2	120140710	120142716	Distal Interger	-0886	348E-09	335E-06	Il21	-13769	Hypo
chr11	9158822	9159690	Distal Interger	-1475	374E-09	355E-06	Gbe1	423960	Hypo
chr2	146842594	146844125	Distal Interger	-1144	408E-09	369E-06	Dhx36	50447	Hypo
chr15	1053755	1054574	Distal Interger	-1394	433E-09	384E-06	Kcnma1	751678	Hypo
chr15	4455149	4457967	Exon (NM_00	-0830	482E-09	419E-06	Rtraf	7793	Hypo
chr14	95499023	95501701	Promoter (2-3	-0778	482E-09	419E-06	Ugp2	-2328	Hypo
chr3	110995597	110997503	Distal Interger	-1023	505E-09	434E-06	Sema6d	-886369	Hypo
chr11	57752097	57754822	Intron (NM_0(	-0735	587E-09	489E-06	Zbtb20	36392	Hypo
chr2	230359382	230361501	Intron (NM_1!	-0950	671E-09	548E-06	Unc5c	178318	Hypo

chr11	25676651	25678170	Distal Interger	-0961	752E-09	608E-06	Adamts5	-629849	Hypo
chr18	34125209	34128653	Intron (NM_0(	-0758	761E-09	608E-06	Sh3rf2	78330	Hypo
chr2	118012623	118014804	Distal Interger	-0875	805E-09	632E-06	Sox2	475694	Hypo
chr16	21100324	21102441	Distal Interger	-0861	806E-09	632E-06	Csgalnact1	-133567	Hypo
chr1	58174998	58177333	Intron (NM_0(	-0791	844E-09	656E-06	Lix1	46037	Hypo
chr1	13031046	13035447	Distal Interger	-0720	110E-08	811E-06	Ccdc28a	-158340	Hypo
chr18	51103636	51106521	Distal Interger	-0813	114E-08	818E-06	LOC10091018	-154977	Hypo
chr6	19989026	19990602	Distal Interger	-0977	116E-08	825E-06	Rasgrp3	-30538	Hypo
chr20	28295227	28301274	Exon (NM_001	-0666	123E-08	859E-06	Vsir	13608	Hypo
chr4	53488034	53489601	Distal Interger	-1149	129E-08	890E-06	Tmem229a	-42707	Hypo
chr2	57822310	57831657	Promoter (<=1	-0650	130E-08	890E-06	Slc1a3	0	Hypo
chr14	91730308	91733123	Distal Interger	-0835	131E-08	891E-06	C1d	69357	Hypo
chr9	50778190	50780974	Distal Interger	-0826	142E-08	953E-06	Tmeff2	-44715	Hypo
chr13	71389234	71390803	Distal Interger	-1079	147E-08	971E-06	Cop1	-76361	Hypo
chr17	41056058	41058424	Intron (NM_0(	-0957	149E-08	971E-06	Scgn	-48386	Hypo
chr4	148091827	148094517	Intron (NM_0(	-0810	153E-08	980E-06	Tamm41	9194	Hypo
chr1	176266896	176270872	Distal Interger	-0861	158E-08	100E-05	Usp31	-14523	Hypo
chr4	65384917	65386756	Distal Interger	-0940	161E-08	101E-05	Ptn	-9611	Hypo
chr9	97818504	97820175	Distal Interger	-0936	176E-08	107E-05	Slco6d1	-78744	Hypo
chr1	140515280	140517401	Distal Interger	-0777	205E-08	120E-05	Folh1	-13901	Hypo
chr13	98872718	98874893	Distal Interger	-0832	206E-08	120E-05	Gpatch2	87701	Hypo
chr5	145342954	145345918	Distal Interger	-0754	218E-08	126E-05	Wasf2	-32657	Hypo
chr7	93222132	93223342	Distal Interger	-1083	245E-08	138E-05		342876	Hypo
chr3	127861898	127863261	Distal Interger	-1073	258E-08	143E-05	Flrt3	144512	Hypo
chr14	102690849	102692048	Distal Interger	-1030	289E-08	156E-05	Efemp1	79941	Hypo
chr6	8319895	8323059	Intron (NM_0	-0822	305E-08	163E-05	Prkce	128260	Hypo
chr1	8615244	8623271	Distal Interger	-0629	336E-08	175E-05	Hivep2	255955	Hypo
chr2	20668223	20672440	5' UTR	-0641	341E-08	175E-05	Hapln1	36583	Hypo
chr12	16434158	16436156	Distal Interger	-0875	353E-08	177E-05	Cyp3a62	36160	Hypo
chr7	126134177	126135738	Intron (NM_0(	-0830	358E-08	178E-05	Tmem117	376586	Hypo
chr17	49621374	49625164	Intron (NM_0{	-0662	378E-08	186E-05	Gli3	84548	Hypo
chr13	61925542	61928197	Intron (NM_1:	-0730	389E-08	190E-05	Pla2g4a	94064	Hypo
chr13	38498264	38498743	Distal Interger	-1930	398E-08	192E-05	Mgat5	-177376	Hypo

chr17	45551541	45552375	Distal Interger	-1514	398E-08	192E-05	Stard3nl	-30903	Hypo
chr3	34604126	34606718	Distal Interger	-0796	412E-08	195E-05	Mmadhc	119830	Hypo
chr10	80570329	80572407	Distal Interger	-0886	413E-08	195E-05	Phb1	-32861	Hypo
chr2	76386405	76391232	Intron (NM_0(	-0592	467E-08	215E-05	Retreg1	50796	Hypo
chr1	78958221	78962360	Promoter (<=1	-0627	467E-08	215E-05	Fosb	0	Hypo
chr8	114289694	114291746	Exon (NM_001	-0871	481E-08	218E-05	Cnot10	38231	Hypo
chr1	159866474	159867677	Distal Interger	-0988	511E-08	228E-05	Smpd1	-25269	Hypo
chr2	203775117	203776974	Distal Interger	-0864	521E-08	231E-05	Dph5	-27646	Hypo
chr15	47086158	47089000	Distal Interger	-0709	558E-08	240E-05	Fndc3a	743580	Hypo
chr18	38503775	38509138	Distal Interger	-0572	562E-08	240E-05	Kcnn2	389549	Hypo
chr12	774094	776215	Distal Interger	-0815	567E-08	241E-05	Stard13	-39490	Hypo
chr10	73711539	73714436	Exon (NM_13:	-0753	570E-08	241E-05	Scpep1	18393	Hypo
chr15	31277065	31279419	Distal Interger	-0847	583E-08	245E-05	Gjb2	-10698	Hypo
chr6	30655604	30656930	Distal Interger	-1115	605E-08	252E-05	Apob	-187456	Hypo
chr17	2202389	2204557	Distal Interger	-0827	606E-08	252E-05	Fbp1	-3474	Hypo
chr17	79106827	79107488	Intron (NM_0(	-1132	632E-08	260E-05	Plxdc2	-88881	Hypo
chr17	22949766	22951453	Intron (NM_0(	-0909	636E-08	261E-05	Adtrp	19026	Hypo
chr11	55565443	55566827	Exon (NM_21:	-1074	641E-08	261E-05	Btla	20155	Hypo
chr8	76439752	76443635	Promoter (<=1	-0586	734E-08	287E-05	Scg3	0	Hypo
chr14	70818179	70819632	Distal Interger	-0795	736E-08	287E-05	Hs3st1	-395387	Hypo
chr1	33508898	33509927	Distal Interger	-1106	798E-08	304E-05	Med10	-33174	Hypo
chr10	73161288	73162180	Distal Interger	-1008	826E-08	310E-05	Ccdc182	71060	Hypo
chr11	74000452	74003392	Distal Interger	-0710	849E-08	315E-05	Il1rap	196057	Hypo
chr2	8859874	8862074	Distal Interger	-0870	857E-08	316E-05	Nr2f1	-809537	Hypo
chr2	165860707	165863363	Distal Interger	-0838	877E-08	322E-05	Mir2985	93135	Hypo
chr19	39654573	39658323	Promoter (<=1	-0560	885E-08	324E-05	Ctrb1	0	Hypo
chr10	73300173	73301096	Distal Interger	-1155	931E-08	336E-05	Ccdc182	209945	Hypo
chr17	70095132	70097388	Distal Interger	-0772	944E-08	339E-05	Celf2	-1312222	Hypo
chr3	118075908	118078047	Distal Interger	-0872	961E-08	342E-05	Dnaaf9	-23278	Hypo
chr1	231239223	231240375	Intron (NM_0(	-0960	972E-08	343E-05	Rps4x-ps1	28946	Hypo
chr11	15882638	15886587	Distal Interger	-0666	986E-08	345E-05	Usp25	278757	Hypo
chr9	91271771	91272549	Distal Interger	-1328	102E-07	351E-05	Cops8	64344	Hypo
chr4	35217030	35217981	Distal Interger	-1065	104E-07	355E-05	Sdhaf3	73899	Hypo

chr5	111420804	111421248	Distal Interger	-1362	114E-07	383E-05	C5h1orf87	-10855	Hypo
chr6	109673030	109680336	Promoter (<=1	-0643	117E-07	391E-05	Dio2	0	Hypo
chr20	50107541	50108948	Distal Interger	-1033	119E-07	394E-05	Hace1	1071642	Hypo
chr2	20691330	20693166	3' UTR	-0826	120E-07	395E-05	Hapln1	59690	Hypo
chr11	61335353	61337231	Distal Interger	-0806	122E-07	399E-05	Igsf11	531117	Hypo
chr18	17080029	17082407	Distal Interger	-0790	122E-07	399E-05	Celf4	-15243	Hypo
chr1	154555916	154559936	Intron (NR_13	-0789	130E-07	420E-05	LOC10091207	11435	Hypo
chr10	64417173	64421703	Exon (NM_01	-0587	133E-07	428E-05	Omg	42381	Hypo
chr7	30886355	30886898	Distal Interger	-1311	135E-07	433E-05	Eea1	281362	Hypo
chr7	63258133	63262235	Intron (NM_0	-0655	136E-07	433E-05	R3hdm2	25787	Hypo
chr1	21155111	21157343	Distal Interger	-0725	138E-07	437E-05	Stx7	79870	Hypo
chr2	41575830	41579954	Distal Interger	-0669	142E-07	448E-05	Rab3c	262619	Hypo
chr9	106922818	106924794	Exon (NM_00	-0870	146E-07	456E-05	Rab12	-416019	Hypo
chr18	50896242	50900844	Distal Interger	-0543	147E-07	456E-05	Ctxn3	-30609	Hypo
chr3	17538286	17540414	Intron (NM_0	-0827	147E-07	456E-05	Pbx3	141936	Hypo
chr14	34608203	34611120	Distal Interger	-0704	152E-07	462E-05	Lrrc66	26885	Hypo
chr19	17639299	17644658	Distal Interger	-0656	155E-07	465E-05	Sall1	-362845	Hypo
chr6	98304258	98306295	Distal Interger	-0700	158E-07	470E-05	Zfyve26	-208778	Hypo
chr4	114148464	114149409	Distal Interger	-1014	162E-07	482E-05	LOC10091137	313699	Hypo
chr8	22855414	22857593	Distal Interger	-0720	165E-07	489E-05	Dpy19l1	163868	Hypo
chr2	59608777	59610727	Intron (NM_0	-0731	169E-07	497E-05	Rai14	71874	Hypo
chr4	114179703	114180359	Distal Interger	-1266	175E-07	506E-05	LOC10091137	282749	Hypo
chr3	153940847	153943080	Intron (NM_0	-0816	178E-07	513E-05	Cdh22	27508	Hypo
chr4	108507104	108507444	Distal Interger	-1568	180E-07	517E-05	LRRTM1	-1194371	Hypo
chr17	79107710	79108371	Intron (NM_0	-1361	183E-07	522E-05	Plxdc2	-87998	Hypo
chr18	21110897	21111706	Distal Interger	-1122	190E-07	539E-05	Pik3c3	-733656	Hypo
chr8	102029380	102030520	Distal Interger	-1008	193E-07	542E-05	Ppp2r3a	-230859	Hypo
chr2	214256172	214258587	Distal Interger	-0882	194E-07	543E-05	Ugt8	74073	Hypo
chr2	226594724	226595915	Distal Interger	-0931	199E-07	553E-05	Mttp	58324	Hypo
chr18	6017343	6020392	Distal Interger	-0656	215E-07	586E-05	Psma8	153697	Hypo
chr17	70380741	70382882	Distal Interger	-0690	223E-07	599E-05	Celf2	-1026728	Hypo
chr20	12385929	12391714	Distal Interger	-0568	233E-07	621E-05	Prmt2	-3143	Hypo
chr20	14820126	14822058	Distal Interger	-0824	238E-07	632E-05	Pcdh15	-120093	Hypo

chr18	74707143	74709720	Distal Interger	-0753	240E-07	635E-05	Sall3	-293315	Hypo
chr15	38604572	38608966	Intron (NM_0	-0606	242E-07	637E-05	Msra	67530	Hypo
chr14	65643999	65650357	Distal Interger	-0565	248E-07	648E-05	Clrn2	15836	Hypo
chr11	44998626	45000529	Distal Interger	-0704	250E-07	649E-05	Nfkbiz	215950	Hypo
chr1	202284602	202295125	Promoter (<=:	-0663	250E-07	649E-05	Npas4	0	Hypo
chr2	103388296	103391282	Distal Interger	-0686	258E-07	661E-05	Agtr1b	-468064	Hypo
chr1	206053567	206057770	Distal Interger	-0595	264E-07	672E-05	Asrgl1	-26452	Hypo
chrX	4098822	4100183	Distal Interger	-1042	265E-07	672E-05	Dusp21	388694	Hypo
chr2	160151348	160153848	Distal Interger	-0655	267E-07	676E-05	Serpini2	-107077	Hypo
chr8	85177113	85180020	Distal Interger	-0614	268E-07	677E-05	Bckdhb	331849	Hypo
chr15	24207870	24211261	Distal Interger	-0683	271E-07	680E-05	Rnase10	-16662	Hypo
chr6	19988052	19988836	Distal Interger	-1051	277E-07	691E-05	Rasgrp3	-29564	Hypo
chr1	231237385	231239132	Intron (NM_0	-0740	284E-07	706E-05	Rps4x-ps1	27108	Hypo
chr6	19987079	19987826	Distal Interger	-1133	286E-07	707E-05	Rasgrp3	-28591	Hypo
chr6	88899608	88901270	Distal Interger	-0808	294E-07	721E-05	Trim9	-43066	Hypo
chr4	82025168	82029337	Distal Interger	-0589	304E-07	745E-05	Tax1bp1	203102	Hypo
chr17	49386588	49388124	Distal Interger	-0834	307E-07	748E-05	Inhba	-277606	Hypo
chr2	137062341	137063056	Exon (NM_00	-1155	309E-07	751E-05	Cog6	36120	Hypo
chr20	12377119	12382666	Promoter (<=:	-0534	314E-07	758E-05	S100b	0	Hypo
chr14	36517192	36520184	Intron (NM_0	-0625	317E-07	764E-05	Gabrb1	28756	Hypo
chr14	36787194	36788432	Distal Interger	-0970	318E-07	764E-05	Gabra4	196412	Hypo
chr8	40902160	40903174	Distal Interger	-1004	320E-07	766E-05	Gramd1b	-80369	Hypo
chr9	15256604	15258578	Distal Interger	-0617	331E-07	784E-05	Mrpl14	49841	Hypo
chr5	61544694	61547665	Intron (NM_0	-0712	331E-07	784E-05	Col15a1	42731	Hypo
chr13	56406791	56407506	Distal Interger	-1295	338E-07	790E-05	Rgs18	108356	Hypo
chr1	253192510	253194846	Distal Interger	-0823	342E-07	793E-05	Adra2a	131030	Hypo
chr1	93976911	93977612	Distal Interger	-1083	342E-07	793E-05	Zfp819	-60636	Hypo
chr11	20190953	20193068	Intron (NM_2	-0644	348E-07	805E-05	Ncam2	85907	Hypo
chr9	15231830	15234312	Distal Interger	-0734	349E-07	805E-05	Mrpl14	74107	Hypo
chr20	2967998	2970114	Distal Interger	-0984	353E-07	812E-05	Ier3	-38416	Hypo
chr2	231027648	231030402	Exon (NM_05	-0630	355E-07	814E-05	Pdlim5	89821	Hypo
chr3	131418959	131419950	Distal Interger	-1116	359E-07	820E-05	Dstn	134312	Hypo
chr2	81286999	81287949	Distal Interger	-0996	366E-07	828E-05	Ctnnd2	-41705	Hypo

chr20	23353386	23354663	Distal Interger	-0915	367E-07	828E-05	Lrrtm3	-1160964	Hypo
chr15	54687168	54687884	Distal Interger	-0995	370E-07	830E-05	Rgcc	-11848	Hypo
chr8	95502469	95503241	Intron (NM_0(	-1078	371E-07	830E-05	Slc9a9	269564	Hypo
chr13	95350150	95352351	Distal Interger	-0716	375E-07	834E-05	Dusp10	-261941	Hypo
chr6	52484259	52485000	Distal Interger	-1148	377E-07	836E-05	Agr3	-158391	Hypo
chr1	221235704	221237404	Intron (NM_0(	-0767	386E-07	851E-05	Cfap95	5559	Hypo
chr5	132093198	132095362	Distal Interger	-0848	387E-07	851E-05	Tmem125	30036	Hypo
chr19	17632447	17634696	Distal Interger	-0847	395E-07	867E-05	Sall1	-372807	Hypo
chr15	951522	953452	Exon (NM_03:	-0711	398E-07	869E-05	Kcnma1	649445	Hypo
chr19	17628285	17632317	Distal Interger	-0683	409E-07	888E-05	Sall1	-375186	Hypo
chr9	35777271	35779210	Exon (NM_00:	-0875	411E-07	889E-05	Prim2	125284	Hypo
chr20	35755233	35764096	Promoter (<=:	-0540	422E-07	899E-05	Gja1	0	Hypo
chr17	45552459	45553380	Distal Interger	-1106	429E-07	909E-05	Stard3nl	-29898	Hypo
chr3	136372402	136372978	Distal Interger	-1133	432E-07	912E-05	Cst3	-31606	Hypo
chr4	51324051	51326988	Distal Interger	-0615	435E-07	913E-05	Ptprz1	-70613	Hypo
chr1	52253235	52259096	Distal Interger	-0549	437E-07	913E-05	Tbxt	46768	Hypo
chr20	45865422	45868191	Intron (NM_0(	-0615	442E-07	919E-05	Afg1l	99715	Hypo
chr2	86653988	86656585	Distal Interger	-0696	443E-07	919E-05		53460	Hypo
chr2	115827329	115833238	Intron (NM_1:	-0609	448E-07	925E-05	Pex5l	74744	Hypo
chr5	126846908	126847536	Distal Interger	-1154	452E-07	931E-05	Skint1	60439	Hypo
chr15	54326685	54329567	Distal Interger	-0631	460E-07	941E-05	Rgcc	345753	Hypo
chr1	241122816	241126008	Exon (NM_13	-0589	474E-07	966E-05	Mir3085	-33823	Hypo
chr2	161278717	161279885	Intron (NM_0(	-1045	477E-07	970E-05	Fstl5	420728	Hypo
chr4	134604401	134607191	Distal Interger	-0727	485E-07	983E-05	Cntn3	518422	Hypo
chr20	28280128	28287212	Promoter (<=:	-0551	527E-07	104E-04	Vsir	0	Hypo
chr8	47191972	47194577	Distal Interger	-0656	528E-07	104E-04	LOC500990	-581498	Hypo
chr6	22217961	22220367	Distal Interger	-0745	541E-07	105E-04	Capn13	146116	Hypo
chr10	73301323	73302423	Distal Interger	-0919	545E-07	106E-04	Ccdc182	211095	Hypo
chr15	42235104	42237899	Distal Interger	-0620	564E-07	108E-04	Nefl	-64021	Hypo
chr17	38430301	38432819	Distal Interger	-0714	565E-07	108E-04	Hdgfl1	222395	Hypo
chr16	64152782	64154792	Distal Interger	-0816	595E-07	113E-04	Smok	-303584	Hypo
chr17	70555757	70556777	Distal Interger	-1028	597E-07	113E-04	Celf2	-852833	Hypo
chr18	18078298	18078989	Distal Interger	-1097	608E-07	115E-04		174017	Hypo

chr8	28210264	28212899	Distal Interger	-0668	610E-07	115E-04	Ntm	-406036	Hypo
chr19	17645026	17647592	Distal Interger	-0729	612E-07	115E-04	Sall1	-359911	Hypo
chr2	95115574	95116188	Distal Interger	-1210	618E-07	116E-04	Pkia	-641975	Hypo
chr10	12908721	12911243	Distal Interger	-0747	631E-07	117E-04	Csap1	-3122	Hypo
chr13	33626240	33626569	Distal Interger	-1511	651E-07	121E-04	Ddx18	-883079	Hypo
chr18	78087447	78091635	Distal Interger	-0563	653E-07	121E-04	Cndp2	-30417	Hypo
chr2	103716027	103717643	Distal Interger	-0810	659E-07	121E-04	Agtr1b	-795795	Hypo
chr18	13148838	13150994	Distal Interger	-0722	670E-07	123E-04	Klhl14	-231010	Hypo
chr4	130891222	130893041	Distal Interger	-0870	679E-07	124E-04	Mitf	482005	Hypo
chr19	2465053	2467367	Distal Interger	-0659	680E-07	124E-04	Cdh11	316606	Hypo
chr13	82022298	82024381	Distal Interger	-0720	685E-07	124E-04	Rgs4	-79230	Hypo
chr19	56615571	56616324	Distal Interger	-1063	685E-07	124E-04	Itgb1	-88881	Hypo
chr13	68295622	68298092	Intron (NM_0(	-0671	697E-07	126E-04	Fam163a	42957	Hypo
chr8	102784692	102786760	Intron (NM_0(	-0741	699E-07	126E-04	Ephb1	158079	Hypo
chr4	80143618	80144457	Distal Interger	-1057	701E-07	126E-04	Mir148a	189401	Hypo
chr2	214294732	214299128	Intron (NM_0	-0588	714E-07	127E-04	Ugt8	33532	Hypo
chr13	39514540	39518765	Distal Interger	-0606	730E-07	129E-04	Mir3473	15294	Hypo
chr15	94960113	94962854	Distal Interger	-0689	738E-07	130E-04	Tgds	232723	Hypo
chr20	41688961	41691583	Distal Interger	-0700	751E-07	132E-04	Lama4	-700685	Hypo
chr19	11279682	11281391	Distal Interger	-0803	759E-07	133E-04	Gnao1	-87189	Hypo
chr10	73276396	73279340	Distal Interger	-0649	776E-07	135E-04	Ccdc182	186168	Hypo
chr1	170519336	170521387	Distal Interger	-0735	777E-07	135E-04	Rps13	53968	Hypo
chr3	58327143	58332606	Exon (NM_05	-0622	788E-07	136E-04	Wipf1	40136	Hypo
chr2	104429858	104432606	Distal Interger	-0771	788E-07	136E-04	Tbl1xr1	-369115	Hypo
chr14	46658950	46661445	Distal Interger	-0649	792E-07	136E-04	Arap2	-57315	Hypo
chr18	43662278	43663937	Distal Interger	-0858	799E-07	136E-04	Fam170a	137831	Hypo
chr18	61735227	61739265	Intron (NM_0(	-0589	807E-07	137E-04	Ldlrad4	88516	Hypo
chr15	1054738	1055614	Distal Interger	-1137	809E-07	137E-04	Kcnma1	752661	Hypo
chr10	2784289	2784962	Distal Interger	-1044	812E-07	137E-04	Rrn3	-619048	Hypo
chr7	43453938	43454850	Distal Interger	-1011	813E-07	137E-04	Ppp1r12a	-28119	Hypo
chr18	47775324	47777986	Distal Interger	-0680	813E-07	137E-04	Csnk1g3	475745	Hypo
chr1	258549961	258551233	Distal Interger	-0884	873E-07	145E-04	Pdzd8	-43133	Hypo
chr7	9975129	9978167	Exon (NM_05	-0836	876E-07	146E-04	Hcn2	10665	Hypo

chr15	54593579	54594373	Distal Intergeı	-1124	882E-07	146E-04	Rgcc	80947	Hypo
chr14	12692255	12693895	Distal Intergeı	-0738	889E-07	146E-04	Anxa3	87739	Hypo
chr13	69704478	69705114	Exon (NM_05:	-1235	901E-07	147E-04	Sec16b	20187	Hypo
chr7	43862864	43865313	Intron (NM_0(	-0712	910E-07	148E-04	Pawr	217089	Hypo
chr18	80807130	80807766	Distal Intergeı	-1193	929E-07	151E-04	Cbln2	865718	Hypo
chr18	75029404	75030173	Distal Intergeı	-1211	930E-07	151E-04	Sall3	-615576	Hypo
chr14	46899176	46901020	Intron (NM_0(	-0812	941E-07	152E-04	Arap2	180416	Hypo
chr17	73828886	73833425	Intron (NM_0(	-0601	953E-07	153E-04	Frmd4a	113219	Hypo
chr7	125963569	125965128	Intron (NM_0(	-0860	957E-07	154E-04	Tmem117	205978	Hypo
chrX	145082919	145083453	Distal Intergeı	-1341	967E-07	154E-04	Slitrk2	-166736	Hypo
chr6	55671347	55672871	Exon (NM_00:	-0780	987E-07	157E-04	Etv1	80176	Hypo
chr3	117688422	117694425	Intron (NM_0	-0517	100E-06	158E-04	Ptpra	38239	Hypo
chr13	96768220	96769925	Intron (NM_0(	-0809	102E-06	160E-04	Rab3gap2	10658	Hypo
chr8	61642070	61643904	Distal Intergeı	-0685	102E-06	160E-04	LOC691000	135405	Hypo
chr9	3444933	3447973	Intron (NM_0(	-0635	106E-06	165E-04	Plcl2	151565	Hypo
chr18	61732079	61733025	Intron (NM_0(	-0973	109E-06	169E-04	Ldlrad4	85368	Hypo
chr13	64936449	64939264	Distal Intergeı	-0594	110E-06	169E-04	Ncf2	-16404	Hypo
chr7	82995611	82998236	Distal Intergeı	-0610	111E-06	171E-04	Eif3h	176200	Hypo
chr2	110885490	110886758	Distal Intergeı	-0875	112E-06	172E-04	Pld1	-6865	Hypo
chr1	105597105	105602834	Distal Intergeı	-0502	113E-06	173E-04	Tubgcp5	-1033692	Hypo
chr8	52236454	52241242	Distal Intergeı	-0543	113E-06	173E-04	Fdx1	46102	Hypo
chr12	28192803	28193148	Distal Intergeı	-1718	114E-06	173E-04	Tmem132d	-77564	Hypo
chr7	132358967	132362858	Distal Intergeı	-0528	114E-06	173E-04	Nr4a1	-18533	Hypo
chr13	42917966	42925291	Intron (NM_0(	-0546	115E-06	174E-04	Srgap2	41767	Hypo
chr1	50334700	50341560	Distal Intergeı	-0524	115E-06	174E-04	Cahm	45399	Hypo
chr18	26678614	26680756	Distal Intergeı	-0624	116E-06	174E-04	Ctnna1	-47762	Hypo
chr6	39424275	39428048	Promoter (<=:	-0553	116E-06	175E-04	Ntsr2	0	Hypo
chr9	34021876	34023110	Distal Intergeı	-0931	118E-06	178E-04	Khdrbs2	-423946	Hypo
chr1	145439770	145441337	Intron (NM_0:	-0778	119E-06	179E-04	Dlg2	312595	Hypo
chr1	8186547	8187379	Intron (NM_0(	-0918	120E-06	179E-04	Aig1	81573	Hypo
chr14	91762307	91765754	Distal Intergeı	-0596	124E-06	183E-04	C1d	101356	Hypo
chr13	39567236	39568669	Distal Intergeı	-0707	124E-06	183E-04	R3hdm1	-27179	Hypo
chr6	35004679	35009834	Distal Intergeı	-0538	126E-06	184E-04	Cyria	-35374	Hypo

chr2	83650428	83652028	Intron (NM_0(	-0866	127E-06	185E-04	Sema5a	340585	Hypo
chr12	27518225	27520248	Distal Interger	-0855	128E-06	186E-04	Ran	158000	Hypo
chr17	1007409	1009052	Exon (NM_00:	-0885	129E-06	187E-04	Hsd17b3	-18177	Hypo
chr17	81688405	81689060	Distal Interger	-1027	129E-06	187E-04	Pip4k2a	-20376	Hypo
chr10	2785051	2786577	Distal Interger	-0819	130E-06	189E-04	Rrn3	-619810	Hypo
chr13	39447853	39452429	Distal Interger	-0544	135E-06	194E-04	Mir3473	-46817	Hypo
chr8	68300554	68301829	Distal Interger	-0862	136E-06	196E-04	LOC10091136	35128	Hypo
chr7	23674358	23676153	Distal Interger	-0787	137E-06	196E-04	Ano4	-22626	Hypo
chr5	25493132	25493674	Distal Interger	-1176	139E-06	198E-04	Pdp1	-38085	Hypo
chr9	15239140	15240983	Distal Interger	-0579	139E-06	198E-04	Mrpl14	67436	Hypo
chr4	169189977	169193013	Distal Interger	-0562	140E-06	200E-04		-61236	Hypo
chr17	23009695	23011145	Distal Interger	-0988	146E-06	207E-04		-25911	Hypo
chr3	76939102	76960404	Distal Interger	-0438	149E-06	211E-04	Celf1	-18909	Hypo
chr7	122767057	122769592	Distal Interger	-0541	150E-06	212E-04	Slc2a13	-40452	Hypo
chr7	31963761	31965167	Distal Interger	-0861	152E-06	213E-04	Dcn	-316085	Hypo
chr20	52903294	52903894	Distal Interger	-1132	152E-06	213E-04	Grik2	-70233	Hypo
chr2	146841356	146842512	Distal Interger	-0965	152E-06	213E-04	Dhx36	52060	Hypo
chr5	24075417	24079375	Distal Interger	-0603	154E-06	215E-04	Plekhf2	27067	Hypo
chr5	5087309	5088349	Distal Interger	-1028	156E-06	216E-04	Xkr9	473819	Hypo
chr1	58157132	58157571	Intron (NM_0(	-1168	158E-06	217E-04	Lix1	28171	Hypo
chr2	57924451	57926168	Distal Interger	-0625	158E-06	217E-04	Slc1a3	-93846	Hypo
chr14	87057783	87063581	Distal Interger	-0506	160E-06	220E-04	Cobl	-78244	Hypo
chr1	155439234	155441723	Distal Interger	-0708	161E-06	220E-04	Fchsd2	-19206	Hypo
chr2	146844308	146845157	Distal Interger	-0971	165E-06	224E-04	Dhx36	49415	Hypo
chr14	71614543	71615237	Distal Interger	-1056	166E-06	225E-04	Hs3st1	399524	Hypo
chr17	61894980	61898557	Intron (NM_1:	-0596	171E-06	230E-04	Idi1	-257623	Hypo
chr1	107252731	107254721	Exon (NM_00:	-0862	171E-06	230E-04	Oca2	136453	Hypo
chr2	165524160	165525690	Distal Interger	-0768	173E-06	232E-04	Mir2985	430808	Hypo
chr17	68232463	68234232	Distal Interger	-0696	174E-06	233E-04	Sfmbt2	-109892	Hypo
chr15	55377064	55378958	Distal Interger	-0732	176E-06	235E-04	Olfm4	-28190	Hypo
chr9	26168247	26171420	Promoter (1-2	-0575	177E-06	236E-04	B3gat2	1073	Hypo
chr10	71992904	71994086	Intron (NM_0(	-0964	179E-06	238E-04	Trim37	49495	Hypo
chr2	26812948	26815632	Distal Interger	-0677	179E-06	238E-04	F2rl1	-27722	Hypo

chr2	27096313	27102218	Distal Interger	-0532	181E-06	238E-04	Ndufs6-ps1	-99495	Hypo
chr4	151009207	151011768	3' UTR	-0593	182E-06	239E-04	Zfp637	3882	Hypo
chr7	121082471	121083064	Distal Interger	-1159	183E-06	240E-04	Syt10	-161741	Hypo
chr15	2484377	2487782	Distal Interger	-0548	183E-06	240E-04	Vdac2	-7829	Hypo
chr8	42760873	42762263	Intron (NM_0(	-0805	187E-06	244E-04	Tecta	17444	Hypo
chr7	45372354	45373483	Intron (NM_0(	-0734	189E-06	246E-04	Nav3	18776	Hypo
chr9	91271014	91271584	Distal Interger	-1180	189E-06	246E-04	Cops8	63587	Hypo
chr2	32991109	32995635	Distal Interger	-0509	190E-06	247E-04	Pik3r1	-37902	Hypo
chr1	154154220	154154878	Distal Interger	-1086	195E-06	253E-04	Neu3	-5407	Hypo
chr9	98085080	98086785	Distal Interger	-0711	197E-06	254E-04	Pam	-36247	Hypo
chr13	89135987	89138419	Intron (NM_0:	-0604	201E-06	258E-04	Akt3	77417	Hypo
chr6	2617395	2618615	Distal Interger	-0715	204E-06	260E-04	Nrxn1	-562675	Hypo
chr15	54574307	54579626	Distal Interger	-0504	204E-06	260E-04	Rgcc	95694	Hypo
chr11	64013861	64017063	Promoter (<=:	-0536	205E-06	260E-04	Slc15a2	0	Hypo
chr2	42707028	42710882	Distal Interger	-0575	207E-06	262E-04	Actbl2	-147138	Hypo
chr9	44795929	44799574	Distal Interger	-0502	207E-06	262E-04	Pantr1	144057	Hypo
chr8	47138210	47140966	Distal Interger	-0556	207E-06	262E-04	LOC500990	-527736	Hypo
chr4	75806748	75807246	Distal Interger	-1071	208E-06	263E-04	Cul1	-744747	Hypo
chr8	42762543	42764315	Exon (NM_00:	-0793	210E-06	265E-04	Tecta	15392	Hypo
chr10	24075914	24076901	Distal Interger	-1127	210E-06	265E-04	Mat2b	1043594	Hypo
chr7	27338022	27340097	Distal Interger	-0749	211E-06	266E-04	Nedd1	-63536	Hypo
chr17	70352162	70355006	Distal Interger	-0788	214E-06	268E-04	Celf2	-1054604	Hypo
chr20	7227541	7229735	Exon (NM_00:	-0676	218E-06	273E-04	Cpne5	59227	Hypo
chr7	45373699	45376039	Intron (NM_0(	-0673	219E-06	273E-04	Nav3	16220	Hypo
chr9	36409210	36412273	Intron (NM_0(	-0606	220E-06	274E-04	Dst	173125	Hypo
chr7	100421711	100424114	Distal Interger	-0689	224E-06	278E-04	Mir30d	-285239	Hypo
chr13	66238884	66242713	Distal Interger	-0535	225E-06	278E-04	Glul	213243	Hypo
chr4	117848666	117852588	Intron (NM_1!	-0495	227E-06	281E-04	Sfxn5	17194	Hypo
chr2	92891299	92894629	Distal Interger	-0664	230E-06	284E-04		-7250	Hypo
chr1	217974365	217975282	Distal Interger	-0982	233E-06	286E-04	Anxa1	-97171	Hypo
chr8	42479435	42483235	Intron (NM_0!	-0555	236E-06	289E-04	Sorl1	20993	Hypo
chr12	28099411	28103405	Promoter (<=:	-0605	237E-06	289E-04	Fzd10	0	Hypo
chr2	163776214	163778436	Distal Interger	-0649	237E-06	289E-04	Rapgef2	543721	Hypo

chr3	44769164	44772070	Distal Interger	-0648	240E-06	292E-04	Mir6216	-12226	Hypo
chr13	39633991	39636735	Intron (NM_0(	-0597	241E-06	292E-04	R3hdm1	38143	Hypo
chr3	163232554	163237042	Promoter (<=1	-0572	241E-06	292E-04	Ctsz	0	Hypo
chr2	98735725	98736828	Distal Interger	-0860	242E-06	292E-04	Ythdf3	385064	Hypo
chr13	61915483	61916294	Intron (NM_1:	-0874	243E-06	293E-04	Pla2g4a	105967	Hypo
chr16	43445260	43447252	Distal Interger	-0657	245E-06	293E-04	Tenm3	-80742	Hypo
chr17	8353055	8355811	Distal Interger	-0734	245E-06	294E-04	Neurog1	-7067	Hypo
chr4	108506301	108506749	Distal Interger	-1153	250E-06	298E-04	LRRTM1	-1195066	Hypo
chr14	30578573	30580084	Distal Interger	-0767	251E-06	299E-04	Igfbp7	-157330	Hypo
chr17	16497453	16500432	Distal Interger	-0600	255E-06	302E-04	Id4	-105497	Hypo
chr9	11608362	11610343	Distal Interger	-0715	255E-06	302E-04	Mocs1	-40572	Hypo
chr17	82527883	82528686	Distal Interger	-0993	256E-06	302E-04	Ptf1a	476602	Hypo
chr17	5597890	5604995	Intron (NM_0	-0510	256E-06	302E-04	Ntrk2	264126	Hypo
chr14	91716655	91718810	Distal Interger	-0626	256E-06	302E-04	C1d	55704	Hypo
chr2	14208376	14209474	Distal Interger	-0813	258E-06	303E-04	Mir3597-2	-10548	Hypo
chr18	25739598	25742629	Distal Interger	-0562	258E-06	303E-04	Apc	-121593	Hypo
chr2	71472145	71474410	Distal Interger	-0583	262E-06	307E-04	Cdh18	-1870595	Hypo
chr8	76423699	76427090	Exon (NM_05:	-0609	263E-06	307E-04	Scg3	14925	Hypo
chr5	145061781	145066320	3' UTR	-0560	269E-06	313E-04	Ppp1r8	-13028	Hypo
chr18	18079175	18080336	Distal Interger	-0844	269E-06	313E-04		172670	Hypo
chr3	56291961	56292939	Intron (NM_0(	-0954	271E-06	314E-04	Metap1d	12468	Hypo
chr1	33523638	33524696	Distal Interger	-0843	274E-06	318E-04	Ube2ql1	-24118	Hypo
chr2	170350003	170355123	Distal Interger	-0522	276E-06	319E-04	Dear	-49821	Hypo
chr17	70862786	70864318	Distal Interger	-0768	277E-06	319E-04	Celf2	-545292	Hypo
chr1	134999340	135001766	3' UTR	-0634	279E-06	320E-04	Alpk3	-12775	Hypo
chr11	81975097	81975821	Distal Interger	-1003	280E-06	320E-04	Hira	157391	Hypo
chr11	57906867	57907987	Distal Interger	-0895	280E-06	320E-04	Zbtb20	-115653	Hypo
chr2	212272976	212274853	Distal Interger	-0635	283E-06	322E-04	Tram1l1	-236277	Hypo
chr1	145389656	145394266	Intron (NM_0:	-0528	283E-06	322E-04	Dlg2	262481	Hypo
chr4	75807378	75808204	Distal Interger	-0966	284E-06	322E-04	Cul1	-743789	Hypo
chr7	30885623	30886116	Distal Interger	-1281	285E-06	323E-04	Eea1	280630	Hypo
chr18	67742448	67745173	Distal Interger	-0576	286E-06	323E-04	Ska1	59864	Hypo
chr11	66141013	66143169	Distal Interger	-0679	290E-06	327E-04	Ropn1	-13865	Hypo

chr1	50384535	50397454	Promoter (<=1	-0444	291E-06	328E-04	Cahm	0	Hypo
chr2	225500127	225501497	Distal Intergen	-0852	293E-06	329E-04	Ppp3ca	334016	Hypo
chr6	10306078	10308609	Intron (NM_0	-0576	294E-06	329E-04	Thada	120079	Hypo
chr5	58552462	58553951	Intron (NM_0	-0706	294E-06	329E-04	Melk	12013	Hypo
chr1	3144653	3148495	Distal Intergen	-0648	295E-06	329E-04	Ust	-182609	Hypo
chr4	16860677	16863711	Intron (NM_0	-0601	295E-06	329E-04	Gnai1	46662	Hypo
chr2	57777417	57781786	Intron (NM_0	-0632	298E-06	331E-04	Slc1a3	48798	Hypo
chr7	32006179	32007856	Distal Intergen	-0731	302E-06	334E-04	Dcn	-273396	Hypo
chr4	118060411	118062322	Distal Intergen	-0672	308E-06	339E-04	Egr4	-10083	Hypo
chr6	71728758	71730089	Distal Intergen	-0874	310E-06	340E-04	Egln3	-52992	Hypo
chr1	186276856	186277267	Distal Intergen	-1214	315E-06	343E-04	Hmx3	-35970	Hypo
chr19	20708871	20711647	Distal Intergen	-0620	315E-06	343E-04	Abcc12	159466	Hypo
chr8	67207295	67209230	Distal Intergen	-0691	318E-06	345E-04	Usp3	-53186	Hypo
chr4	176588558	176590790	Distal Intergen	-0644	319E-06	345E-04	Etnk1	462378	Hypo
chr5	133567621	133571135	Distal Intergen	-0638	319E-06	345E-04	Hivep3	-86917	Hypo
chr4	176593613	176595394	Distal Intergen	-0703	319E-06	345E-04	Etnk1	467433	Hypo
chr10	65651371	65653213	Intron (NM_0	-0680	321E-06	346E-04	Myo1d	112599	Hypo
chr18	48009922	48014024	Distal Intergen	-0574	322E-06	346E-04	Zfp608	396441	Hypo
chr1	233855822	233856552	Distal Intergen	-1059	324E-06	347E-04	Ankrd1	-31413	Hypo
chr7	69563850	69565763	Distal Intergen	-0734	326E-06	349E-04	Klf10	-90124	Hypo
chr18	67748794	67751033	Distal Intergen	-0591	328E-06	350E-04	Ska1	54004	Hypo
chr3	136371573	136372074	Distal Intergen	-1199	328E-06	350E-04	Cst3	-30777	Hypo
chr1	235159945	235161548	Exon (NM_001	-0741	340E-06	360E-04	Hhex	-28907	Hypo
chr18	49919285	49925859	Intron (NM_0	-0517	342E-06	361E-04	Gramd2b	35271	Hypo
chr14	36805955	36807390	Distal Intergen	-0781	344E-06	363E-04	Gabra4	215173	Hypo
chr9	11614110	11614511	Distal Intergen	-1182	347E-06	365E-04	Mocs1	-46320	Hypo
chr14	2365989	2374380	Promoter (<=1	-0477	347E-06	365E-04	Ephx4	0	Hypo
chr8	61644209	61645398	Distal Intergen	-0852	350E-06	367E-04	LOC691000	137544	Hypo
chr1	213109571	213112668	Distal Intergen	-0506	354E-06	370E-04	Psat1	105896	Hypo
chr13	74411081	74414490	Intron (NM_1	-0528	359E-06	374E-04	RGD1309106	-54759	Hypo
chr5	36621324	36623306	Distal Intergen	-0659	363E-06	376E-04	Pou3f2	-537486	Hypo
chr2	233837463	233839473	Intron (NM_0	-0704	367E-06	379E-04	Clca4l	6664	Hypo
chr4	176590983	176592440	Distal Intergen	-0757	367E-06	379E-04	Etnk1	464803	Hypo

chr4	59341017	59341981	Distal Interger	-0836	369E-06	380E-04	Mest	-12464	Hypo
chr15	85077509	85079051	Distal Interger	-0689	370E-06	380E-04	Slitrk1	648239	Hypo
chr11	67419788	67422791	Distal Interger	-0552	372E-06	381E-04	Zfp148	-34016	Hypo
chr17	69867619	69869557	Distal Interger	-0646	374E-06	382E-04	Gata3	1222706	Hypo
chr16	56337606	56338801	Distal Interger	-0795	379E-06	386E-04	Cldn23	-74876	Hypo
chr1	33508084	33508727	Distal Interger	-1095	381E-06	388E-04	Med10	-32360	Hypo
chr7	43455047	43455668	Distal Interger	-1098	382E-06	388E-04	Ppp1r12a	-27301	Hypo
chr17	8010242	8012255	Distal Interger	-0730	383E-06	388E-04	Tgfbi	-25404	Hypo
chr4	149975629	149976420	Promoter (2-3	-0903	383E-06	389E-04	Tmem72	-2079	Hypo
chr20	42475506	42477124	Exon (NM_00	-0884	386E-06	390E-04	Fam229b	75124	Hypo
chr17	82888738	82890687	Distal Interger	-0782	387E-06	390E-04	Arhgap21	581515	Hypo
chr4	114208187	114210439	Distal Interger	-0534	388E-06	390E-04	LOC10091137	252669	Hypo
chr8	49971728	49988416	Intron (NM_0	-0432	390E-06	392E-04	Ttc12	-126043	Hypo
chr6	104388798	104390341	Distal Interger	-0829	392E-06	392E-04	Npc2	27820	Hypo
chr19	17904898	17907959	Distal Interger	-0630	395E-06	394E-04	Sall1	-99544	Hypo
chr17	43858486	43860025	Intron (NM_0	-0814	397E-06	396E-04	Or2p2	119027	Hypo
chr8	121150276	121151768	Distal Interger	-0756	401E-06	399E-04	Cck	8316	Hypo
chr18	25800861	25802823	Distal Interger	-0616	402E-06	399E-04	Apc	-61399	Hypo
chr10	39254517	39256703	Exon (NM_00	-0637	402E-06	399E-04	Slc36a3	13864	Hypo
chr2	226596242	226597062	Distal Interger	-1037	403E-06	399E-04	Mttp	57177	Hypo
chr13	68551577	68553432	Distal Interger	-0713	404E-06	399E-04	Soat1	44023	Hypo
chr1	209036506	209037898	Promoter (2-3	-0804	404E-06	399E-04	Or4d6	2119	Hypo
chr2	170711002	170715960	Distal Interger	-0478	405E-06	399E-04	Fhip1a	375522	Hypo
chr2	188996525	188999346	Distal Interger	-0669	413E-06	405E-04	Atp1a1	49480	Hypo
chr6	109866553	109869352	Distal Interger	-0605	415E-06	407E-04	Dio2	-186744	Hypo
chr17	70761483	70766119	Distal Interger	-0584	419E-06	409E-04	Celf2	-643491	Hypo
chr19	17625744	17628142	Distal Interger	-0642	423E-06	412E-04	Sall1	-379361	Hypo
chr9	56644891	56649618	Distal Interger	-0487	428E-06	416E-04	Mob4	27517	Hypo
chr13	45547914	45550209	Distal Interger	-0467	429E-06	416E-04	Btg2	-12272	Hypo
chr8	102729417	102730966	Intron (NM_0	-0691	430E-06	417E-04	Ephb1	213873	Hypo
chr14	87069677	87071039	Distal Interger	-0688	432E-06	418E-04	Cobl	-90138	Hypo
chr17	5836111	5837265	Exon (NM_01	-0724	434E-06	418E-04	Ntrk2	31856	Hypo
chr3	60980645	60981865	Exon (NM_00	-0848	438E-06	419E-04	Ift70a1	-75295	Hypo

chr15	14694955	14695858	Intron (NM_0:	-0886	439E-06	419E-04	Fhit	727073	Hypo
chr1	159864928	159866275	Distal Interger	-0821	440E-06	419E-04	Smpd1	-26671	Hypo
chr9	107003260	107005312	Intron (NM_0(	-0670	441E-06	419E-04	Ptprm	338386	Hypo
chr5	57106679	57107192	Distal Interger	-1102	444E-06	422E-04	Spata31f3	-13515	Hypo
chr3	90722680	90728297	Distal Interger	-0471	446E-06	422E-04	Hipk3	138015	Hypo
chr7	26784688	26785816	Distal Interger	-0826	451E-06	425E-04	Mir135a	-172599	Hypo
chr2	143482496	143485151	Promoter (<=:	-0691	453E-06	426E-04	P2ry12	261	Hypo
chr6	35598385	35601994	Distal Interger	-0729	456E-06	429E-04	Mycn	121596	Hypo
chr1	47079752	47082822	Distal Interger	-0513	457E-06	429E-04	Ezr	-68247	Hypo
chrX	2624051	2626876	Distal Interger	-0663	460E-06	431E-04	Chst7	-191223	Hypo
chr4	117836397	117841599	Intron (NM_1!	-0534	461E-06	432E-04	Sfxn5	28183	Hypo
chr2	203766373	203768125	Distal Interger	-0654	465E-06	434E-04	Dph5	-36495	Hypo
chr16	46649427	46652349	Distal Interger	-0632	465E-06	434E-04	Sorbs2	-22979	Hypo
chr8	112420960	112421897	Distal Interger	-0905	468E-06	436E-04	Arpp21	-267929	Hypo
chr20	41693191	41695196	Distal Interger	-0721	472E-06	438E-04	Lama4	-697072	Hypo
chr1	184889702	184893878	Distal Interger	-0564	472E-06	438E-04	Fgfr2	-39076	Hypo
chr17	5862084	5870718	Promoter (<=:	-0410	477E-06	440E-04	Ntrk2	0	Hypo
chr20	20078832	20079741	Intron (NM_0(	-0983	477E-06	440E-04	Cabcoco1	20299	Hypo
chr3	116256264	116258558	Distal Interger	-0586	481E-06	444E-04	Ttl	-40312	Hypo
chr2	51647411	51649271	Promoter (<=:	-0592	492E-06	450E-04	Hmgcs1	-325	Hypo
chr13	87277652	87279414	Intron (NM_0	-0771	497E-06	454E-04	Rgs7	129209	Hypo
chr5	119936832	119941969	Intron (NM_1:	-0510	497E-06	454E-04	Plpp3	9747	Hypo
chr3	27517303	27519630	Distal Interger	-0629	502E-06	458E-04	Kynu	-259125	Hypo
chr2	41738056	41742266	Exon (NM_13:	-0548	505E-06	460E-04	Rab3c	100307	Hypo
chr12	45187339	45189182	Intron (NM_0(	-0657	506E-06	460E-04	Mn1	30920	Hypo
chr15	38956010	38960691	Intron (NM_2	-0504	511E-06	463E-04	Kif13b	47692	Hypo
chr14	72422357	72424149	Intron (NM_0(	-0682	513E-06	464E-04	Drd5	66625	Hypo
chr5	126250984	126252158	Intron (NM_0(	-0926	513E-06	464E-04	Bend5	-38490	Hypo
chr6	98884070	98888167	Distal Interger	-0464	521E-06	469E-04	Zfp36l1	47581	Hypo
chr13	80339644	80344894	3' UTR	-0546	526E-06	472E-04	Pbx1	243272	Hypo
chr1	213141978	213143757	Distal Interger	-0779	530E-06	475E-04	Psat1	74807	Hypo
chr13	95086077	95088031	Distal Interger	-0668	530E-06	475E-04	Taf1a	73917	Hypo
chr2	203765124	203766174	Distal Interger	-0808	537E-06	479E-04	Dph5	-38446	Hypo

chr7	32530053	32533886	Distal Interger	-0501	538E-06	479E-04	Ccer1	44860	Hypo
chr6	47881338	47882893	Distal Interger	-0786	540E-06	481E-04	Dld	41921	Hypo
chr1	154727906	154729580	Intron (NM_0(	-0683	541E-06	481E-04	C2cd3	12353	Hypo
chr9	36258967	36260772	Intron (NM_0(	-0765	542E-06	481E-04	Dst	22882	Hypo
chr3	124762037	124762956	Distal Interger	-0850	542E-06	481E-04	Jag1	-319817	Hypo
chr4	148222457	148229907	Intron (NM_0	-0445	550E-06	487E-04	Syn2	36187	Hypo
chr2	145541863	145542570	Distal Interger	-0953	551E-06	487E-04	Rap2b	-57144	Hypo
chr16	5240939	5245366	Intron (NM_0	-0478	555E-06	489E-04	Chdh	46670	Hypo
chr2	81455788	81458233	Intron (NM_0(	-0602	558E-06	491E-04	Ctnnd2	126134	Hypo
chr7	86866637	86867280	Intron (NM_0(	-1029	560E-06	492E-04	Mtbp	-105789	Hypo
chr14	26495845	26497065	Intron (NM_1:	-0743	561E-06	492E-04	Adgrl3	327379	Hypo
chr1	249686275	249687942	Distal Interger	-0751	567E-06	497E-04	Sorcs1	-91996	Hypo
chr11	57181785	57192499	Intron (NM_0(	-0458	575E-06	503E-04	Mir568	-127712	Hypo
chr7	127709565	127712437	Distal Interger	-0598	579E-06	506E-04	Scaf11	-85650	Hypo
chr8	114652600	114653974	Distal Interger	-0827	583E-06	508E-04	Gpd1l1	-31829	Hypo
chr2	165762485	165766071	Distal Interger	-0522	585E-06	509E-04	Mir2985	190427	Hypo
chr15	38601805	38604355	Intron (NM_0!	-0560	586E-06	509E-04	Msra	72141	Hypo
chr1	12998859	13001519	Distal Interger	-0627	586E-06	509E-04	Ccdc28a	-126153	Hypo
chr19	14819217	14821808	Distal Interger	-0611	592E-06	513E-04	Crnde	175330	Hypo
chr7	29822094	29825057	Distal Interger	-0539	592E-06	513E-04	Cradd	127850	Hypo
chr20	36200973	36204361	Distal Interger	-0544	595E-06	513E-04	Gja1	444938	Hypo
chrX	28122825	28127751	Distal Interger	-0483	599E-06	515E-04	Gpm6b	-21237	Hypo
chr1	217973001	217974233	Distal Interger	-0829	602E-06	517E-04	Anxa1	-95807	Hypo
chr10	72712191	72716537	Promoter (2-3	-0521	607E-06	519E-04	Olr1523	-2978	Hypo
chr11	54877855	54879590	Distal Interger	-0723	608E-06	519E-04	Plcxd2	76855	Hypo
chr9	32998652	33001032	Distal Interger	-0634	617E-06	526E-04	Phf3	134962	Hypo
chr15	82377106	82379501	Distal Interger	-0457	624E-06	530E-04	Spry2	315823	Hypo
chr2	120698768	120701403	Distal Interger	-0471	625E-06	530E-04	Spry1	141492	Hypo
chr17	86177637	86178939	Distal Interger	-0895	628E-06	532E-04	Lyst	-100580	Hypo
chr7	47900187	47905529	Distal Interger	-0482	629E-06	532E-04	Kcnc2	198766	Hypo
chr11	57914089	57916725	Distal Interger	-0658	629E-06	532E-04	Zbtb20	-122875	Hypo
chr1	121697501	121698502	Intron (NM_0!	-0864	630E-06	532E-04	Igf1r	146760	Hypo
chr14	87068471	87069523	Distal Interger	-0777	641E-06	541E-04	Cobl	-88932	Hypo

chr1	50397586	50400054	Intron (NM_0(	-0596	644E-06	542E-04	Qki	9888	Hypo
chr8	61635557	61636599	Distal Interger	-0843	644E-06	542E-04	LOC691000	128892	Hypo
chr9	86949700	86952175	Distal Interger	-0646	646E-06	543E-04	B3gnt7	-4045	Hypo
chr6	138467279	138475087	Distal Interger	-0448	650E-06	545E-04	Rapgef5	-126687	Hypo
chr5	94598260	94600504	Distal Interger	-0666	658E-06	550E-04	Tyrp1	-680478	Hypo
chr4	174472532	174473985	5' UTR	-0741	659E-06	551E-04	Slco1c1	5901	Hypo
chr14	4029971	4030683	3' UTR	-1092	667E-06	555E-04	Lrrc8d	14701	Hypo
chr7	34136757	34139770	Distal Interger	-0591	672E-06	558E-04	Dusp6	43801	Hypo
chr12	42796054	42797987	Distal Interger	-0703	674E-06	560E-04	Selplg	-9891	Hypo
chr15	21357407	21358797	Distal Interger	-0751	678E-06	562E-04	Peli2	-34465	Hypo
chr2	81691930	81694730	Intron (NM_0(	-0680	682E-06	564E-04	Ctnnd2	362276	Hypo
chr2	49333900	49337290	Distal Interger	-0500	685E-06	566E-04	Hcn1	-158481	Hypo
chr4	30430240	30434276	Intron (NM_0(	-0523	686E-06	566E-04	Tmbim7	64980	Hypo
chr3	83110461	83111263	Intron (NM_0(	-0952	686E-06	566E-04	Lrrc4c	805217	Hypo
chr7	63180299	63185528	Promoter (1-2	-0448	688E-06	567E-04	Inhbe	-1316	Hypo
chr7	25178711	25182876	Exon (NM_001	-0511	693E-06	569E-04	Apaf1	396664	Hypo
chr2	26441585	26443611	Distal Interger	-0660	696E-06	571E-04	Pde8b	-60278	Hypo
chr12	26906718	26909036	Intron (NM_0(	-0551	699E-06	573E-04	Psph	15732	Hypo
chr9	55299734	55300916	Distal Interger	-0812	701E-06	573E-04	Stk17b	37169	Hypo
chr6	27508879	27511067	Distal Interger	-0610	703E-06	573E-04	Ncoa1	-33215	Hypo
chr2	57892906	57895662	Distal Interger	-0501	704E-06	573E-04	Slc1a3	-62301	Hypo
chr16	75170420	75172365	Intron (NM_0!	-0605	706E-06	574E-04	Dlgap2	324015	Hypo
chr13	37478558	37480279	Distal Interger	-0721	711E-06	578E-04	Lypd1	-110065	Hypo
chr3	153244657	153246209	Distal Interger	-0715	720E-06	584E-04	Pigt	16894	Hypo
chr5	57034131	57035097	Distal Interger	-0892	724E-06	586E-04	Ccl21	-52470	Hypo
chr1	44074095	44076640	Distal Interger	-0586	726E-06	586E-04	Cldn20	-57129	Hypo
chr9	3175403	3178311	Distal Interger	-0525	727E-06	586E-04	Plcl2	-115057	Hypo
chr13	87311319	87312847	Intron (NM_0	-0769	728E-06	587E-04	Rgs7	95776	Hypo
chr2	153211249	153213465	Intron (NM_0(	-0597	733E-06	590E-04	Ift80	26559	Hypo
chr9	5247275	5249156	Distal Interger	-0692	737E-06	591E-04	Kcnh8	-256888	Hypo
chr7	119516177	119516697	Distal Interger	-1208	743E-06	595E-04	Zdhhc25	-175982	Hypo
chr1	78983867	78988285	Distal Interger	-0455	744E-06	595E-04	Ercc1	-8451	Hypo
chr8	102378130	102379023	Distal Interger	-1003	753E-06	601E-04	Ephb1	565816	Hypo

chr9	11670207	11672169	Distal Interger	-0619	754E-06	601E-04	Mocs1	-102417	Hypo
chr20	2965775	2967868	Distal Interger	-0689	758E-06	603E-04	Ier3	-36193	Hypo
chr9	33677564	33678318	Distal Interger	-0984	760E-06	604E-04	Lgsn	257080	Hypo
chr7	109916032	109917667	Distal Interger	-0654	766E-06	606E-04	Cimip4	27248	Hypo
chr10	16607971	16614130	Distal Interger	-0462	771E-06	609E-04	Dusp1	-66359	Hypo
chr16	56214349	56215033	Distal Interger	-0922	774E-06	611E-04	Prag1	-73196	Hypo
chr10	64459502	64465630	Promoter (<=1	-0417	784E-06	618E-04	Omg	0	Hypo
chr4	118309858	118312460	Distal Interger	-0539	790E-06	620E-04	Nat8f2	9315	Hypo
chr10	67792479	67796533	Distal Interger	-0590	800E-06	627E-04	Rffl	-27686	Hypo
chr5	4954778	4958623	Distal Interger	-0518	802E-06	628E-04	Msc	403442	Hypo
chr16	10391000	10393390	Intron (NM_0	-0511	805E-06	628E-04	Mir346	172882	Hypo
chr16	46665889	46668886	Distal Interger	-0548	806E-06	629E-04	Sorbs2	-39441	Hypo
chr19	20919679	20920997	Distal Interger	-0768	808E-06	630E-04	Phkb	289608	Hypo
chr17	49625357	49627988	Intron (NM_0	-0575	810E-06	631E-04	Gli3	81724	Hypo
chr20	36211051	36211970	Distal Interger	-0874	811E-06	631E-04	Gja1	455016	Hypo
chr8	28233465	28241807	Distal Interger	-0453	812E-06	631E-04	Ntm	-429237	Hypo
chr14	46896612	46899068	Exon (NM_00	-0631	817E-06	633E-04	Arap2	177852	Hypo
chr3	104276184	104284171	Distal Interger	-0439	817E-06	633E-04	Rasgrp1	-46128	Hypo
chr13	42930055	42934743	Intron (NM_0	-0485	821E-06	636E-04	Srgap2	32315	Hypo
chr13	30024087	30029649	Distal Interger	-0500	823E-06	636E-04	Gli2	-21270	Hypo
chr17	19874524	19876527	Distal Interger	-0599	824E-06	636E-04	Dtnbp1	189306	Hypo
chr1	52262147	52264577	Distal Interger	-0609	835E-06	642E-04	Tbxt	41287	Hypo
chr6	69925751	69927395	Distal Interger	-0715	835E-06	642E-04	Arhgap5	-60815	Hypo
chr5	16723685	16725640	Exon (NM_00	-0741	839E-06	643E-04	Lyn	84173	Hypo
chr5	151012678	151013851	Promoter (2-3	-0759	840E-06	643E-04	Pla2g2d	-2519	Hypo
chr4	83497152	83498240	Distal Interger	-0949	846E-06	646E-04	Wipf3	-52228	Hypo
chr15	24169849	24179523	Promoter (<=1	-0511	848E-06	646E-04	Pnp	0	Hypo
chr2	152336255	152337080	Intron (NM_0	-1029	863E-06	654E-04	Schip1	209084	Hypo
chr13	104791287	104793238	Distal Interger	-0680	865E-06	654E-04	G0s2	14025	Hypo
chr3	134230954	134233017	Distal Interger	-0669	896E-06	672E-04	Xrn2	-204128	Hypo
chr17	70620990	70625808	Distal Interger	-0467	896E-06	672E-04	Celf2	-783802	Hypo
chr2	120283502	120285453	Intron (NM_0	-0702	901E-06	675E-04	Nudt6	20716	Hypo
chr2	15098457	15098892	Distal Interger	-1266	906E-06	677E-04	Ccnh	-735941	Hypo

chr10	98250372	98252232	Distal Interger	-0624	909E-06	678E-04	Mir297	-33828	Hypo
chr3	63077444	63079413	Distal Interger	-0620	914E-06	680E-04	Zfp385b	-450001	Hypo
chr1	235772326	235774945	Intron (NM_0(	-0630	921E-06	684E-04	Myof	47389	Hypo
chr1	188963318	188966399	Distal Interger	-0510	939E-06	697E-04	LOC10030246	184665	Hypo
chr2	149890288	149891853	Promoter (<=1	-0492	944E-06	699E-04	Lekr1	0	Hypo
chr4	175896780	175897847	Intron (NM_0	-0761	948E-06	701E-04	St8sia1	22730	Hypo
chr14	12693979	12695457	Distal Interger	-0652	957E-06	704E-04	Anxa3	86177	Hypo
chr15	82366459	82369840	Distal Interger	-0431	957E-06	704E-04	Spry2	325484	Hypo
chr1	109311293	109313476	Distal Interger	-0494	958E-06	704E-04	Atp10a	-243306	Hypo
chr16	73169468	73171891	Intron (NM_0(	-0608	959E-06	704E-04	Csmd1	950703	Hypo
chr8	119769086	119774736	Distal Interger	-0473	959E-06	704E-04	Cx3cr1	24695	Hypo
chr1	211816811	211818151	Distal Interger	-0926	960E-06	704E-04	Tle4	-18949	Hypo
chr9	81095598	81096028	Promoter (2-3	-1161	961E-06	704E-04	Wdfy1	-2805	Hypo
chr5	126847670	126848444	Distal Interger	-1081	961E-06	704E-04	Skint1	61201	Hypo
chr18	61761313	61766302	Intron (NM_0(	-0502	964E-06	704E-04	Ldlrad4	114602	Hypo
chr15	24180094	24184598	Distal Interger	-0516	974E-06	711E-04	Pnp	9476	Hypo
chr7	63712339	63714827	Distal Interger	-0594	989E-06	720E-04	Rdh7	-22964	Hypo
chr20	28247775	28248932	Exon (NM_05	-0947	994E-06	723E-04	Vsir	-32687	Hypo
chr13	61916542	61918034	Intron (NM_1	-0684	996E-06	724E-04	Pla2g4a	104227	Hypo
chr2	209796714	209799450	Distal Interger	-0508	999E-06	725E-04	F3	-27611	Hypo
chr4	119027011	119030598	Distal Interger	-0489	100E-05	725E-04	Mxd1	87153	Hypo
chr2	157972401	157973587	Distal Interger	-0814	100E-05	726E-04	Slitrk3	-275846	Hypo
chr17	70427877	70431920	Distal Interger	-0503	101E-05	727E-04	Celf2	-977690	Hypo
chr1	122281444	122283337	Distal Interger	-0702	101E-05	730E-04	LOC682259	-47845	Hypo
chr7	76192131	76194188	Distal Interger	-0754	101E-05	730E-04	Kcnv1	73652	Hypo
chr15	8102845	8104634	Distal Interger	-0661	101E-05	730E-04	Thrb	-72157	Hypo
chr4	15759312	15762561	Intron (NM_0	-0564	102E-05	730E-04	Magi2	107476	Hypo
chr4	39066784	39069304	Distal Interger	-0615	102E-05	732E-04	Ndufa4	940080	Hypo
chr8	27037152	27038615	Intron (NM_0	-0589	103E-05	736E-04	Opcml	247484	Hypo
chr8	62369416	62370064	Distal Interger	-1049	103E-05	736E-04	Rplp1	25277	Hypo
chr15	21355599	21356495	Distal Interger	-0948	103E-05	741E-04	Peli2	-36767	Hypo
chr2	170754482	170757448	Distal Interger	-0599	104E-05	743E-04	Fhip1a	334034	Hypo
chr5	164877057	164877932	Distal Interger	-0767	104E-05	744E-04	Arhgef16	-10845	Hypo

chr16	36971402	36973573	Distal Intergen	-0570	105E-05	745E-04	Gpm6a	-217242	Hypo
chr6	66010137	66011174	Distal Intergen	-0747	105E-05	745E-04	Foxg1	-663623	Hypo
chr19	20752187	20754133	Distal Intergen	-0561	105E-05	745E-04	Abcc12	202782	Hypo
chrX	35927125	35928695	Distal Intergen	-0786	107E-05	758E-04	Rps6ka3	-304055	Hypo
chr20	27238456	27239225	Distal Intergen	-0994	107E-05	758E-04	Sowahc	43258	Hypo
chr2	165771789	165772631	Distal Intergen	-0838	107E-05	760E-04	Mir2985	183867	Hypo
chr4	179136801	179144040	Exon (NM_03:	-0447	107E-05	760E-04	Sspn	239654	Hypo
chr1	126944939	126946706	Distal Intergen	-0687	108E-05	761E-04	Rgma	-182228	Hypo
chr7	129264019	129266608	Promoter (2-3	-0534	108E-05	763E-04	Asb8	2328	Hypo
chr1	138500569	138502726	Distal Intergen	-0639	109E-05	768E-04	Fah	68779	Hypo
chr8	57025149	57034441	Promoter (<=:	-0447	109E-05	770E-04	Lingo1	0	Hypo
chr2	116168270	116173473	Distal Intergen	-0484	109E-05	770E-04	Pex5l	-260288	Hypo
chr2	239969825	239970539	Distal Intergen	-0888	110E-05	771E-04	Adgrl4	-384402	Hypo
chr8	44297238	44299598	Distal Intergen	-0524	110E-05	775E-04	Thy1	-89936	Hypo
chr1	151525777	151527229	Intron (NM_0:	-0646	111E-05	778E-04	Gab2	95823	Hypo
chr6	36771461	36771958	Distal Intergen	-1200	112E-05	784E-04	Lratd1	150185	Hypo
chr2	170716151	170718615	Distal Intergen	-0583	113E-05	784E-04	Fhip1a	372867	Hypo
chrX	19404694	19407203	Distal Intergen	-0493	113E-05	786E-04	Alas2	-60721	Hypo
chr1	202295221	202299036	Distal Intergen	-0678	115E-05	796E-04	Npas4	-8497	Hypo
chr7	30109283	30112316	Distal Intergen	-0514	115E-05	797E-04	Mrpl42	29323	Hypo
chr8	113446329	113450749	Distal Intergen	-0458	116E-05	804E-04	Pdcd6ip	196024	Hypo
chr3	134371522	134372907	Distal Intergen	-0612	117E-05	809E-04	Xrn2	-64238	Hypo
chr1	128508332	128509638	Distal Intergen	-0817	117E-05	809E-04	Slco3a1	-120445	Hypo
chr5	21547645	21547989	Distal Intergen	-1397	118E-05	811E-04	Rab2a	-128230	Hypo
chr1	50344751	50347933	Distal Intergen	-0547	118E-05	811E-04	Cahm	39026	Hypo
chr1	90315009	90318695	Distal Intergen	-0580	118E-05	811E-04	Uri1	385961	Hypo
chr5	110708421	110709891	Intron (NM_0(	-0843	119E-05	815E-04	Hook1	-114610	Hypo
chr14	65532656	65534452	Exon (NM_00:	-0638	119E-05	815E-04	Fam184b	57167	Hypo
chr8	42071776	42074874	Distal Intergen	-0503	119E-05	815E-04	Vof16	118714	Hypo
chr8	49134647	49149207	Intron (NM_0(	-0442	120E-05	823E-04	Zbtb16	27804	Hypo
chr2	111388918	111391187	Intron (NM_0(	-0589	120E-05	823E-04	Tnik	204531	Hypo
chr5	136271246	136273257	Distal Intergen	-0605	121E-05	823E-04	Lnc081	-117625	Hypo
chr3	11635498	11636239	Distal Intergen	-0949	121E-05	823E-04	Olfm1	102190	Hypo

chr8	28206440	28209332	Distal Intergei	-0493	121E-05	825E-04	Ntm	-402212	Hypo
chr11	71880296	71882283	Intron (NM_0	-0671	121E-05	827E-04	Mb21d2	54548	Hypo
chr7	76131964	76142799	Distal Intergei	-0400	122E-05	827E-04	Kcnv1	125041	Hypo
chr20	26908157	26909998	Distal Intergei	-0707	122E-05	831E-04	Septin10	284767	Hypo
chr16	46697167	46700574	Distal Intergei	-0502	122E-05	831E-04	Sorbs2	-70719	Hypo
chr6	76936147	76937744	Distal Intergei	-0726	122E-05	831E-04	Fbxo33	-3810	Hypo
chr3	123684583	123690494	Intron (NM_0	-0491	126E-05	849E-04	Pak5	13436	Hypo
chr6	69935284	69938110	Distal Intergei	-0491	126E-05	851E-04	Arhgap5	-50100	Hypo
chr12	42786393	42788522	Distal Intergei	-0624	127E-05	855E-04	Selplg	-19356	Hypo
chr7	82246686	82248653	Distal Intergei	-0551	128E-05	858E-04	Trps1	-105332	Hypo
chr13	89115902	89117757	Intron (NM_0	-0507	128E-05	858E-04	Akt3	98079	Hypo
chr18	14734420	14737280	Distal Intergei	-0533	129E-05	861E-04	Mapre2	-334091	Hypo
chr8	95329202	95330112	Intron (NM_0	-0807	130E-05	868E-04	Slc9a9	96297	Hypo
chr4	82008986	82011557	Distal Intergei	-0552	130E-05	868E-04	Tax1bp1	186920	Hypo
chr10	92728790	92732885	Intron (NM_0	-0495	130E-05	870E-04	Cacng4	12716	Hypo
chr17	15403553	15407440	Promoter (2-3	-0570	132E-05	876E-04	Susd3	2708	Hypo
chr10	63046285	63050590	3' UTR	-0446	132E-05	878E-04	Traf4	9694	Hypo
chr17	68239626	68242883	Distal Intergei	-0512	132E-05	879E-04	Sfmbt2	-117055	Hypo
chr1	135214024	135217962	Intron (NM_1	-0517	133E-05	880E-04	Pde8a	47787	Hypo
chr3	11767793	11769793	Distal Intergei	-0654	133E-05	884E-04	Mrps2	-31517	Hypo
chrX	33975057	33975941	Intron (NM_0	-0946	134E-05	891E-04	Rs1	16174	Hypo
chr7	90142649	90144297	Distal Intergei	-0789	135E-05	892E-04	Fam91a1	173044	Hypo
chr5	111421604	111422337	Distal Intergei	-0975	136E-05	903E-04	C5h1orf87	-11655	Hypo
chr17	85135955	85139451	Intron (NM_0	-0471	139E-05	914E-04	Abi1	40302	Hypo
chr1	24678985	24682006	Distal Intergei	-0581	139E-05	914E-04	Trdn	-268572	Hypo
chr4	80142438	80143425	Distal Intergei	-0851	141E-05	924E-04	Mir148a	190433	Hypo
chr1	52226525	52228333	Distal Intergei	-0704	141E-05	928E-04	Tbxt	77531	Hypo
chr20	40803869	40805309	Distal Intergei	-0752	142E-05	929E-04	Marcks	-112857	Hypo
chr1	234425069	234427846	Distal Intergei	-0509	143E-05	934E-04	Ppp1r3c	38115	Hypo
chr16	74153156	74155144	Distal Intergei	-0632	143E-05	935E-04	Myom2	434996	Hypo
chr18	38597606	38598756	Distal Intergei	-0775	144E-05	940E-04	Trim36	314103	Hypo
chr17	70100426	70100701	Distal Intergei	-1248	145E-05	942E-04	Celf2	-1308909	Hypo
chr5	115320321	115320884	Distal Intergei	-1016	145E-05	942E-04	Cachd1	-90949	Hypo

chr3	103325506	103326318	Distal Interger	-0879	145E-05	944E-04	Meis2	-382009	Hypo
chr1	192507682	192508683	Distal Interger	-0710	147E-05	956E-04	Glrx3	265901	Hypo
chr7	25846583	25847355	Distal Interger	-0920	148E-05	956E-04	Tmpo	-178872	Hypo
chr6	92850592	92851611	Distal Interger	-0855	148E-05	957E-04	Snapc1	165807	Hypo
chr5	120479854	120482388	Distal Interger	-0633	148E-05	957E-04	Plpp3	552769	Hypo
chr18	25780704	25783663	Distal Interger	-0557	149E-05	957E-04	Apc	-80559	Hypo
chr5	111337009	111338324	Distal Interger	-0702	150E-05	964E-04	C5h1orf87	71625	Hypo
chr17	8342035	8343356	Distal Interger	-0765	153E-05	979E-04	Neurog1	-19522	Hypo
chr14	102692144	102693215	Distal Interger	-0825	153E-05	982E-04	Efemp1	81236	Hypo
chr9	79280673	79283963	Distal Interger	-0455	154E-05	985E-04	Epha4	-322534	Hypo
chr1	209038113	209038937	Promoter (1-2	-0843	154E-05	987E-04	Or4d6	1080	Hypo
chr7	86865784	86866472	Intron (NM_0(	-0823	155E-05	989E-04	Mtbp	-106597	Hypo
chr5	57142765	57152443	Distal Interger	-0427	155E-05	989E-04	Dnajb5	-24402	Hypo
chr2	118199556	118203044	Distal Interger	-0470	155E-05	989E-04	Dcun1d1	564274	Hypo
chr2	93044256	93044729	Distal Interger	-1047	156E-05	994E-04	Hey1	-51977	Hypo
chrX	145254665	145256026	Distal Interger	-0814	157E-05	994E-04	Slitrk2	4476	Hypo
chr5	115629219	115632609	Intron (NM_0(	-0477	157E-05	995E-04	Raver2	-68717	Hypo
chr1	173041842	173052710	Promoter (<=1	-0413	157E-05	997E-04	Ccp110	0	Hypo
chr1	3613720	3615153	Distal Interger	-0758	158E-05	999E-04	Samd5	475756	Hypo
chr1	213338490	213342228	Distal Interger	-0441	159E-05	100E-03	Cep78	-63366	Hypo
chr8	112146589	112154726	Promoter (<=1	-0429	159E-05	100E-03	Arpp21	0	Hypo
chr16	45864046	45866451	Distal Interger	-0578	162E-05	102E-03	Acsl1	-64276	Hypo
chr13	42643737	42646092	Intron (NM_0	-0596	164E-05	103E-03	Eif2d	27817	Hypo
chr4	145894316	145896884	Intron (NM_0(	-0531	165E-05	103E-03	Rad18	-73251	Hypo
chr1	247271606	247274086	Intron (NM_0(	-0584	165E-05	103E-03	Sorcs3	172428	Hypo
chr5	31613046	31614788	Distal Interger	-0691	165E-05	103E-03	Mmp16	300766	Hypo
chr2	137047101	137050609	Distal Interger	-0482	165E-05	103E-03	Cog6	48567	Hypo
chr12	33001004	33002981	Intron (NM_0:	-0573	166E-05	103E-03	Vps33a	-21655	Hypo
chr20	32509287	32511856	Distal Interger	-0591	166E-05	103E-03	Pln	-117955	Hypo
chr1	213112802	213114462	Distal Interger	-0571	166E-05	103E-03	Psat1	104102	Hypo
chr15	80603464	80605498	Distal Interger	-0597	166E-05	104E-03	Ednrb	66617	Hypo
chr18	51963294	51966768	Distal Interger	-0561	167E-05	104E-03	Slc27a6	-74306	Hypo
chr1	132655442	132656162	Distal Interger	-1029	167E-05	104E-03	Mrpl46	51477	Hypo

chr7	126138132	126139891	Intron (NM_0(	-0668	167E-05	104E-03	Tmem117	380541	Hypo
chr2	101521338	101523703	Distal Interger	-0599	169E-05	105E-03	Armc1	128477	Hypo
chr8	95503425	95504326	Intron (NM_0(	-0801	169E-05	105E-03	Slc9a9	270520	Hypo
chr2	24535908	24545600	Promoter (<=:	-0420	172E-05	106E-03	Homer1	0	Hypo
chr2	203777108	203777873	Distal Interger	-0848	173E-05	106E-03	Dph5	-26747	Hypo
chr1	117868869	117870435	Exon (NM_00:	-0531	173E-05	106E-03	Mtmr10	9514	Hypo
chr8	72149124	72149954	Distal Interger	-0899	173E-05	106E-03	Polr2m	-9701	Hypo
chr18	47970545	47974346	Distal Interger	-0504	174E-05	107E-03	Zfp608	436119	Hypo
chr11	5382327	5384321	Distal Interger	-0585	176E-05	108E-03	Cadm2	-601995	Hypo
chr9	44696066	44699328	Distal Interger	-0493	176E-05	108E-03	Pantr1	244303	Hypo
chr7	26762215	26763908	Distal Interger	-0631	177E-05	108E-03	Mir135a	-150126	Hypo
chr18	52290837	52292753	Distal Interger	-0699	177E-05	108E-03	Adamts19	-54675	Hypo
chr4	65336853	65354025	Intron (NM_0	-0371	177E-05	108E-03	Ptn	21281	Hypo
chr3	116050860	116052125	Distal Interger	-0715	178E-05	108E-03	Tmem87b	-10091	Hypo
chr3	27886119	27887024	Intron (NM_0:	-0794	179E-05	109E-03	Arhgap15	-102609	Hypo
chr17	5017333	5022443	Distal Interger	-0489	181E-05	110E-03	Naa35	63917	Hypo
chr14	93042218	93044933	Distal Interger	-0604	182E-05	110E-03	Meis1	248662	Hypo
chr3	53114624	53120660	Exon (NM_01!	-0438	186E-05	112E-03	Stk39	58400	Hypo
chr6	111053179	111054437	Distal Interger	-0700	187E-05	113E-03	Sel1l	-273531	Hypo
chr15	97623443	97625242	Distal Interger	-0639	188E-05	113E-03	Rap2a	26595	Hypo
chr11	57600274	57602627	Intron (NM_0(	-0571	190E-05	114E-03	Zbtb20	188587	Hypo
chr7	21067265	21070971	Distal Interger	-0504	191E-05	115E-03	Tdg	26558	Hypo
chr18	71887606	71888219	Intron (NM_0	-0985	191E-05	115E-03	Slc14a2	151243	Hypo
chr1	2098615	2099423	Intron (NM_0(	-0957	192E-05	115E-03	Lrp11	19011	Hypo
chr5	83656395	83656751	Distal Interger	-1472	194E-05	116E-03	Cdk5rap2	304031	Hypo
chr4	117855300	117856642	Exon (NM_15:	-0659	195E-05	116E-03	Sfxn5	13140	Hypo
chr2	196680130	196681026	Distal Interger	-0805	195E-05	117E-03	Eeig2	-16759	Hypo
chr17	79177259	79180394	Distal Interger	-0521	196E-05	117E-03	Plxdc2	-15975	Hypo
chr19	52587300	52590793	Distal Interger	-0482	198E-05	118E-03	Capn9	37852	Hypo
chr13	39568793	39570867	Distal Interger	-0570	199E-05	118E-03	R3hdm1	-24981	Hypo
chr5	145107644	145110590	3' UTR	-0559	201E-05	119E-03	Stx12	-15267	Hypo
chr8	72148403	72148989	Distal Interger	-0906	201E-05	119E-03	Polr2m	-8980	Hypo
chrX	12339354	12342370	Distal Interger	-0498	201E-05	119E-03	RGD1561661	-27325	Hypo

chr1	51417569	51419457	Distal Interger	-0607	201E-05	119E-03	MGC94891	344532	Hypo
chr15	46100611	46101080	Distal Interger	-1111	202E-05	119E-03	Gfra2	158160	Hypo
chr6	90894744	90895959	Intron (NM_0!	-0818	202E-05	119E-03	Rtn1	87477	Hypo
chr4	11555202	11558867	Intron (NM_0(	-0472	202E-05	119E-03	Srpk2	17817	Hypo
chr20	20000189	20001735	Distal Interger	-0739	203E-05	120E-03	Cabcoco1	-56798	Hypo
chr16	78042796	78045994	3' UTR	-0509	204E-05	120E-03	Rab20	23459	Hypo
chr9	55301101	55302694	Distal Interger	-0570	205E-05	120E-03	Stk17b	35391	Hypo
chr13	32615621	32617092	Intron (NM_0(	-0741	205E-05	120E-03	Ccdc93	8003	Hypo
chr19	36537786	36541075	Distal Interger	-0567	205E-05	120E-03	Pmfbp1	-918383	Hypo
chr15	47121375	47122082	Distal Interger	-0830	208E-05	121E-03	Fndc3a	710498	Hypo
chr18	49047255	49051154	Distal Interger	-0505	208E-05	122E-03	Zfp608	-636790	Hypo
chr15	18813718	18816562	Distal Interger	-0464	210E-05	122E-03	Fermt2	-61940	Hypo
chr7	57396736	57398020	Intron (NM_0(	-0622	211E-05	123E-03	Kics2	112514	Hypo
chr2	232291878	232292515	Distal Interger	-0992	211E-05	123E-03	Pkn2	-392925	Hypo
chr9	97902416	97906528	Distal Interger	-0456	211E-05	123E-03	Slco6d1	-162656	Hypo
chr8	90211229	90211625	Distal Interger	-1193	212E-05	123E-03	Tmed3	37804	Hypo
chr19	17456986	17461206	Distal Interger	-0507	212E-05	123E-03	Sall1	-546297	Hypo
chr7	85429373	85430095	Distal Interger	-0945	212E-05	123E-03	Tnfrsf11b	164431	Hypo
chr2	57994418	57995490	Distal Interger	-0780	213E-05	123E-03	Nadk2	-122184	Hypo
chr1	185382325	185384573	Exon (NM_00:	-0576	214E-05	124E-03	Plekha1	-43475	Hypo
chr2	27309234	27311161	Intron (NM_0:	-0620	216E-05	124E-03	Ndufs6-ps1	107521	Hypo
chr8	43887374	43889410	Distal Interger	-0580	216E-05	124E-03	Trim29	205153	Hypo
chr17	64076527	64077742	Distal Interger	-0756	216E-05	124E-03	Pitrm1	-249214	Hypo
chrX	28272535	28278724	Promoter (<=:	-0431	216E-05	125E-03	Gemin8	0	Hypo
chr3	43836229	43839117	Distal Interger	-0527	217E-05	125E-03	Dapl1	-121876	Hypo
chr8	61528087	61532785	Distal Interger	-0454	217E-05	125E-03	LOC691000	21422	Hypo
chr15	55562531	55563178	Distal Interger	-1003	217E-05	125E-03	LOC306079	42199	Hypo
chr1	7997816	8000505	Distal Interger	-0528	218E-05	125E-03	Adat2	43259	Hypo
chr20	5701499	5704519	Promoter (2-3	-0480	219E-05	125E-03	Rps10	-2577	Hypo
chr2	183409703	183410851	Promoter (2-3	-0613	219E-05	125E-03	Mrps21	2842	Hypo
chr1	235768371	235772075	Intron (NM_0(	-0520	220E-05	126E-03	Myof	50259	Hypo
chr12	13601134	13605333	Distal Interger	-0551	220E-05	126E-03	Gna12	-200365	Hypo
chr10	75674323	75675673	Distal Interger	-0764	221E-05	126E-03	LOC10036342	165969	Hypo

chr5	39888502	39889255	Distal Interger	-0816	221E-05	126E-03	Manea	322889	Hypo
chr2	149840948	149842029	Distal Interger	-0767	222E-05	126E-03	Lekr1	-49077	Hypo
chr2	242383831	242385306	Intron (NM_0	-0631	222E-05	126E-03	St6galnac3	259760	Hypo
chr7	47993649	47993930	Distal Interger	-1191	223E-05	127E-03	Kcnc2	292228	Hypo
chr9	3844303	3846080	Intron (NM_0(	-0654	224E-05	127E-03	Tbc1d5	170833	Hypo
chr11	82895854	82899451	Distal Interger	-0582	224E-05	127E-03	Prodh	-10686	Hypo
chr18	48029632	48032706	Distal Interger	-0489	226E-05	128E-03	Zfp608	377759	Hypo
chr4	130894046	130896691	Distal Interger	-0567	227E-05	128E-03	Mitf	484829	Hypo
chr8	42176320	42178281	Distal Interger	-0547	228E-05	128E-03	Vof16	223258	Hypo
chr11	55024957	55027279	Distal Interger	-0538	228E-05	129E-03	Abhd10	-54061	Hypo
chr8	78677489	78680194	Distal Interger	-0582	228E-05	129E-03	Gclc	47362	Hypo
chr11	42085268	42089145	Intron (NM_2(	-0440	229E-05	129E-03	St3gal6	7647	Hypo
chr17	70088886	70094082	Distal Interger	-0422	229E-05	129E-03	Celf2	-1315528	Hypo
chr20	41756674	41757464	Distal Interger	-0832	229E-05	129E-03	Lama4	-634804	Hypo
chr7	24825264	24826681	Intron (NM_0(	-0717	230E-05	129E-03	Anks1b	511135	Hypo
chr1	258556621	258557518	Distal Interger	-0741	231E-05	129E-03	Pdzd8	-49793	Hypo
chr2	209770693	209772630	Distal Interger	-0585	232E-05	129E-03	F3	-54431	Hypo
chr14	26470790	26472003	Exon (NM_13(	-0723	232E-05	129E-03	Adgrl3	352441	Hypo
chr1	88424684	88425925	Distal Interger	-0857	232E-05	129E-03	Dpy19l3	-7345	Hypo
chr8	37667089	37669903	Distal Interger	-0537	233E-05	130E-03	Or8b9	15560	Hypo
chr5	119942516	119954784	Intron (NM_1:	-0374	233E-05	130E-03	Plpp3	15431	Hypo
chr3	46730678	46731179	Intron (NM_1;	-0907	234E-05	130E-03	Slc4a10	65350	Hypo
chr4	176644112	176645301	Distal Interger	-0800	234E-05	130E-03	Sox5	514224	Hypo
chr3	54209769	54210706	Intron (NM_0:	-0915	234E-05	130E-03	Dhrs9	61935	Hypo
chr17	56542489	56543252	Distal Interger	-0850	236E-05	131E-03	Wac	619366	Hypo
chr3	118155084	118157200	Exon (NM_03:	-0594	237E-05	131E-03	Atrn	44764	Hypo
chr4	61446650	61447539	Intron (NM_0(	-0802	237E-05	131E-03	Chchd3	169282	Hypo
chr17	20922667	20923184	Distal Interger	-1011	238E-05	131E-03	Cd83	-15964	Hypo
chr18	1730386	1733630	Intron (NM_0(	-0514	238E-05	131E-03	Snrpd1	33520	Hypo
chr2	142851899	142853941	3' UTR	-0544	239E-05	132E-03	Erich6	24069	Hypo
chr11	16096709	16101095	Distal Interger	-0418	242E-05	133E-03	Mir99a	-99348	Hypo
chr2	80782843	80783272	Distal Interger	-1178	243E-05	133E-03	Ctnnd2	-546382	Hypo
chr17	70006703	70007879	Distal Interger	-0650	243E-05	134E-03	Gata3	1361790	Hypo

chr10	73435561	73442726	Distal Intergenic	-0410	244E-05	134E-03	Akap1	193520	Hypo
chr8	42463215	42465545	Intron (NM_0	-0608	245E-05	134E-03	Sorl1	38683	Hypo
chr1	19411736	19413449	Distal Intergenic	-0653	246E-05	135E-03	Tmem200a	87739	Hypo
chr6	71836619	71838731	Distal Intergenic	-0580	247E-05	135E-03	Egln3	-160853	Hypo
chr2	232319926	232320650	Distal Intergenic	-0936	249E-05	136E-03	Pkn2	-420973	Hypo
chr17	20909828	20910833	Distal Intergenic	-0806	249E-05	136E-03	Cd83	-3125	Hypo
chr18	21173802	21176264	Distal Intergenic	-0586	252E-05	137E-03	Pik3c3	-669098	Hypo
chr2	8029147	8031803	Distal Intergenic	-0497	253E-05	137E-03	Nr2f1	18534	Hypo
chr6	69149143	69149833	Intron (NM_0	-0888	253E-05	137E-03	Strn3	-15041	Hypo
chr3	63462743	63467133	Distal Intergenic	-0509	254E-05	138E-03	RGD1564319	-177482	Hypo
chr3	123276010	123279061	Exon (NM_02	-0524	254E-05	138E-03	Lamp5	-93401	Hypo
chr19	38686229	38700988	Promoter (<=	-0388	256E-05	138E-03	Mtss2	0	Hypo
chr9	88491647	88491876	Intron (NM_0	-1257	256E-05	138E-03	Sag	22271	Hypo
chr8	71415921	71419997	Intron (NM_0	-0439	258E-05	139E-03	Adam10	69913	Hypo
chr17	8347679	8348344	Distal Intergenic	-1064	260E-05	140E-03	Neurog1	-14534	Hypo
chr1	137479630	137481477	Distal Intergenic	-0687	260E-05	140E-03	Tmc3	-70978	Hypo
chr2	57923212	57924197	Distal Intergenic	-0637	261E-05	140E-03	Slc1a3	-92607	Hypo
chr1	50368293	50372754	Distal Intergenic	-0410	261E-05	140E-03	Cahm	14205	Hypo
chr4	174361047	174363623	Intron (NM_0	-0514	262E-05	140E-03	Slco1c1	-103008	Hypo
chr2	233943547	233946395	Exon (NM_00	-0578	263E-05	141E-03	Clca1	17974	Hypo
chr8	110247204	110248929	Distal Intergenic	-0580	263E-05	141E-03	Cspg5	26539	Hypo
chr15	18775627	18779161	Distal Intergenic	-0500	263E-05	141E-03	Fermt2	-23849	Hypo
chr1	34322205	34323001	Distal Intergenic	-0890	263E-05	141E-03	Adcy2	-52910	Hypo
chr6	109964507	109966433	Distal Intergenic	-0622	265E-05	142E-03	Dio2	-284698	Hypo
chr15	82046636	82048124	Intron (NM_0	-0731	266E-05	142E-03	Ndfip2	14270	Hypo
chr15	51495843	51496540	Distal Intergenic	-0866	266E-05	142E-03	Nufip1	66267	Hypo
chr8	95756717	95757682	Intron (NM_0	-0818	268E-05	143E-03	Paqr9	-356567	Hypo
chr18	28880847	28882327	Distal Intergenic	-0478	268E-05	143E-03	Pcdhac2	77759	Hypo
chr1	226292532	226294259	Distal Intergenic	-0608	271E-05	144E-03	Slc1a1	-255673	Hypo
chr6	138480332	138487661	Distal Intergenic	-0432	271E-05	144E-03	Rapgef5	-114113	Hypo
chr5	100221406	100221799	Distal Intergenic	-1102	272E-05	144E-03	Sh3gl2	568217	Hypo
chr15	85618579	85620630	Distal Intergenic	-0576	275E-05	146E-03	Slitrk1	106660	Hypo
chr16	9660422	9662367	Intron (NM_0	-0582	277E-05	147E-03	Glud1	20110	Hypo

chr5	49888863	49889971	Distal Interger	-0714	278E-05	147E-03	RGD1359108	-97455	Hypo
chr3	88981971	88984935	Downstream (	-0460	278E-05	147E-03	Slc1a2	-20194	Hypo
chr12	42798110	42800537	Distal Interger	-0572	278E-05	147E-03	Selplg	-7341	Hypo
chr7	117645965	117646272	Distal Interger	-1247	278E-05	147E-03	Cerk	-377206	Hypo
chr6	105524838	105527331	Distal Interger	-0503	279E-05	147E-03	Erg28	-41760	Hypo
chr5	22392062	22392728	Distal Interger	-0754	279E-05	147E-03	Clvs1	-15656	Hypo
chr1	255476141	255479552	3' UTR	-0511	279E-05	147E-03	Plekhs1	-16082	Hypo
chr14	95112249	95114134	Distal Interger	-0622	280E-05	147E-03	Lgalsl	-107750	Hypo
chr16	25104015	25106611	Intron (NM_0	-0490	280E-05	147E-03	Cpe	73739	Hypo
chr12	22964156	22964627	Distal Interger	-1212	280E-05	147E-03	Castor2	-343974	Hypo
chr4	173207673	173208955	Distal Interger	-0739	281E-05	147E-03	Plekha5	-125100	Hypo
chr1	183263161	183265907	Exon (NM_00	-0486	282E-05	148E-03	Mcmbp	51068	Hypo
chr5	144773318	144775617	Promoter (<=1	-0576	282E-05	148E-03	Ptafr	0	Hypo
chr18	49913941	49918967	Intron (NM_0	-0463	287E-05	149E-03	Gramd2b	29927	Hypo
chr1	93938530	93940354	Distal Interger	-0641	287E-05	150E-03	Etfb	86622	Hypo
chr17	68279622	68281770	Distal Interger	-0624	288E-05	150E-03	Kin	142068	Hypo
chr5	119926695	119935648	Promoter (<=1	-0399	288E-05	150E-03	Plpp3	0	Hypo
chr1	58156234	58156850	Intron (NM_0	-0952	288E-05	150E-03	Lix1	27273	Hypo
chr18	48301481	48302975	Distal Interger	-0631	289E-05	150E-03	Zfp608	107490	Hypo
chr12	16318086	16318694	Promoter (2-3	-0945	291E-05	151E-03	Asmtl	2672	Hypo
chr3	159112881	159113535	Distal Interger	-0923	292E-05	151E-03	Bcas1	98385	Hypo
chr10	98368460	98371966	Intron (NM_0	-0447	292E-05	151E-03	Mir297	-151916	Hypo
chr10	3236409	3238184	Distal Interger	-0691	292E-05	151E-03	Cpped1	-463338	Hypo
chr17	56570594	56573064	Distal Interger	-0537	294E-05	152E-03	Wac	647471	Hypo
chr14	2923886	2924416	Exon (NM_00	-1094	294E-05	152E-03	Hfm1	63618	Hypo
chr4	13386519	13388354	Intron (NM_1	-0677	298E-05	153E-03	Napepld	10404	Hypo
chr3	13057014	13057836	Promoter (1-2	-0936	298E-05	154E-03	Trub2	1995	Hypo
chr14	94273249	94276186	Distal Interger	-0506	299E-05	154E-03	Actr2	57529	Hypo
chr5	141381552	141385948	Distal Interger	-0469	299E-05	154E-03	Rnf19b	-32218	Hypo
chr3	28718600	28720752	Distal Interger	-0604	301E-05	154E-03	Gtdc1	440867	Hypo
chr10	54791451	54793153	Distal Interger	-0615	301E-05	154E-03	Asgr1	15654	Hypo
chr1	254831785	254833108	Intron (NM_0	-0684	302E-05	155E-03	Tcf7l2	45694	Hypo
chr11	34039075	34040624	Distal Interger	-0449	302E-05	155E-03	Dyrk1a	122891	Hypo

chr8	75999303	76006211	Exon (NM_001	-0424	302E-05	155E-03	Myo5c	9775	Hypo
chr15	3882597	3884235	Exon (NM_001	-0589	303E-05	155E-03	Cfap70	30088	Hypo
chr3	118049278	118054906	Promoter (<=1	-0374	304E-05	155E-03	Dnaaf9	0	Hypo
chr2	91654686	91656621	Distal Intergen	-0547	308E-05	157E-03	Pmp2	43352	Hypo
chr5	90420098	90424847	Distal Intergen	-0453	308E-05	157E-03	Dmac1	-1176341	Hypo
chr10	56709247	56715595	Distal Intergen	-0409	309E-05	157E-03	Pimreg	39557	Hypo
chr8	33298526	33300502	Intron (NM_00	-0623	309E-05	157E-03	St3gal4	163988	Hypo
chr8	43880512	43882738	Distal Intergen	-0554	311E-05	158E-03	Trim29	198291	Hypo
chr17	18212672	18214057	Distal Intergen	-0611	311E-05	158E-03	Cap2	-9327	Hypo
chr1	132615159	132615701	Distal Intergen	-1053	311E-05	158E-03	Mrpl46	91938	Hypo
chr16	36749922	36755938	Promoter (<=1	-0378	312E-05	158E-03	Gpm6a	0	Hypo
chr20	41776010	41777304	Distal Intergen	-0699	313E-05	158E-03	Lama4	-614964	Hypo
chr13	61728194	61728538	Distal Intergen	-1049	314E-05	159E-03	Pla2g4a	293723	Hypo
chr18	12622650	12623744	Distal Intergen	-0665	315E-05	159E-03	Mep1b	256524	Hypo
chr1	192506309	192507537	Distal Intergen	-0663	316E-05	160E-03	Glrx3	264528	Hypo
chr14	41850387	41852869	Distal Intergen	-0586	318E-05	160E-03	Nsun7	82080	Hypo
chr9	89428016	89430904	Distal Intergen	-0671	319E-05	161E-03	Arl4c	-124246	Hypo
chr13	94232442	94233142	Distal Intergen	-1104	319E-05	161E-03	Capn8	-19912	Hypo
chr3	97306479	97306941	3' UTR	-1009	320E-05	161E-03	Muc15	11696	Hypo
chr9	31355403	31356587	Distal Intergen	-0723	326E-05	163E-03	Phf3	1779407	Hypo
chr1	246982148	246983747	Distal Intergen	-0683	326E-05	163E-03	Sorcs3	-115431	Hypo
chr18	31781352	31783181	Distal Intergen	-0609	326E-05	164E-03	Pabpc2	127134	Hypo
chr4	54128934	54133119	Distal Intergen	-0527	328E-05	164E-03	Gpr37	27808	Hypo
chr20	40782757	40784846	Distal Intergen	-0639	329E-05	165E-03	Marcks	-91745	Hypo
chr5	8184954	8187371	Promoter (<=1	-0570	330E-05	165E-03	Prex2	0	Hypo
chr11	57159874	57162171	Intron (NM_00	-0516	330E-05	165E-03	Mir568	-105801	Hypo
chr8	40901264	40901977	Distal Intergen	-0773	331E-05	165E-03	Gramd1b	-79473	Hypo
chr16	45514909	45520822	Intron (NM_00	-0471	331E-05	165E-03	Irf2	29137	Hypo
chr2	192264765	192265401	Distal Intergen	-0921	331E-05	165E-03	Ppm1j	-13468	Hypo
chr6	104385918	104386985	Distal Intergen	-0795	333E-05	166E-03	Npc2	31176	Hypo
chr19	38906883	38908791	Distal Intergen	-0574	333E-05	166E-03	St3gal2	-15208	Hypo
chr3	116536253	116538816	Promoter (<=1	-0587	334E-05	166E-03	Il1a	0	Hypo
chr18	48145850	48147561	Distal Intergen	-0632	334E-05	166E-03	Zfp608	262904	Hypo

chr7	76190762	76191916	Distal Interger	-0739	338E-05	167E-03	Kcnv1	75924	Hypo
chr1	8624367	8627171	Distal Interger	-0497	338E-05	167E-03	Hivep2	265078	Hypo
chr20	36341358	36344133	Distal Interger	-0545	338E-05	167E-03	Hsf2	-475731	Hypo
chr2	115460544	115462249	Distal Interger	-0755	339E-05	168E-03	Actl6a	-30125	Hypo
chr7	62664757	62667560	Distal Interger	-0607	340E-05	168E-03	Atp23	62263	Hypo
chr2	210681553	210683543	Intron (NM_0(	-0560	341E-05	168E-03	Fnbp1l	54719	Hypo
chr18	24473253	24474707	Distal Interger	-0517	341E-05	168E-03	Camk4	-110568	Hypo
chr13	22333596	22338875	Intron (NM_0:	-0468	342E-05	169E-03	Phlpp1	25060	Hypo
chr20	45834144	45837324	Intron (NM_0(	-0493	342E-05	169E-03	Foxo3	-69538	Hypo
chr3	87699108	87699694	Distal Interger	-1014	343E-05	169E-03	Rag2	-202679	Hypo
chr17	5540965	5547546	Distal Interger	-0383	350E-05	171E-03	Ntrk2	321575	Hypo
chr6	107944929	107946303	Intron (NM_0!	-0637	352E-05	172E-03	Nrxn3	302945	Hypo
chr10	63589985	63591839	Promoter (<=:	-0532	354E-05	173E-03	Nlk	-255	Hypo
chr8	65343041	65343664	Distal Interger	-1008	358E-05	175E-03	Rab11a	-96580	Hypo
chr10	54186418	54187187	Distal Interger	-0947	359E-05	175E-03	Kdm6b	-56618	Hypo
chr13	94801181	94801707	Intron (NM_0(	-1017	362E-05	176E-03	Disp1	64995	Hypo
chr7	68654087	68656297	Distal Interger	-0596	364E-05	177E-03	Ncald	-25326	Hypo
chr2	150037825	150038658	Exon (NM_00:	-0848	366E-05	178E-03	Lekr1	146719	Hypo
chr20	41755765	41756317	Distal Interger	-0953	367E-05	178E-03	Lama4	-635951	Hypo
chr14	94001008	94003095	Distal Interger	-0582	368E-05	178E-03	Spred2	-146599	Hypo
chr19	10210255	10216506	Distal Interger	-0404	370E-05	179E-03	Ccl17	-6487	Hypo
chr1	105495397	105496272	Distal Interger	-0858	370E-05	179E-03	Tubgcp5	-1140254	Hypo
chr3	77060402	77066352	Distal Interger	-0425	371E-05	179E-03	Spi1	-7344	Hypo
chr2	143519580	143521344	Distal Interger	-0667	372E-05	179E-03	P2ry12	-34168	Hypo
chr2	195172074	195178704	Distal Interger	-0416	372E-05	179E-03	Slc6a17	-16377	Hypo
chr14	34586465	34586857	Intron (NM_0(	-1374	372E-05	179E-03	Lrrc66	5147	Hypo
chr3	69164922	69166688	Distal Interger	-0670	373E-05	180E-03	Zswim2	-149511	Hypo
chr14	45152933	45153667	Distal Interger	-0852	375E-05	181E-03	LOC498368	-674892	Hypo
chr8	102314074	102315365	Distal Interger	-0765	377E-05	181E-03	Ppp2r3a	-515553	Hypo
chr7	45371042	45372229	Intron (NM_0(	-0675	378E-05	181E-03	Nav3	20030	Hypo
chr3	116049260	116050685	Distal Interger	-0719	378E-05	181E-03	Tmem87b	-11531	Hypo
chr2	174375381	174375820	Exon (NM_00:	-1065	380E-05	182E-03	Ash1l	28266	Hypo
chr15	51223964	51226697	Intron (NM_0:	-0508	382E-05	183E-03	Kctd4	-47598	Hypo

chr7	42146966	42147448	Intron (NM_0(	-0985	383E-05	183E-03	Acss3	302782	Hypo
chr3	116709033	116709258	Intron (NM_0(	-1219	384E-05	183E-03	Sirpb3	53988	Hypo
chr3	62235213	62235833	Distal Interger	-0979	385E-05	184E-03	Sestd1	-36203	Hypo
chr7	75924297	75927496	Intron (NM_0(	-0479	386E-05	184E-03	Smg5l1	6735	Hypo
chr17	40843043	40846446	Intron (NM_0(	-0437	386E-05	184E-03	Carmil1	34468	Hypo
chr9	73950424	73952552	Promoter (2-3	-0556	386E-05	184E-03	Xrcc5	-2706	Hypo
chr5	91784409	91787867	Distal Interger	-0438	387E-05	184E-03	Dmac1	-2540652	Hypo
chr10	16957916	16960130	Distal Interger	-0581	388E-05	184E-03	Efcab9	152115	Hypo
chr9	93133897	93135102	Promoter (2-3	-0737	388E-05	184E-03	Or9s23	2414	Hypo
chr15	47041808	47043635	Distal Interger	-0736	391E-05	185E-03	Fndc3a	788945	Hypo
chr3	103762201	103765991	Distal Interger	-0476	394E-05	186E-03	Spred1	-218038	Hypo
chr17	18216547	18218344	Distal Interger	-0468	394E-05	186E-03	Cap2	-13202	Hypo
chr12	12845434	12846182	Distal Interger	-0761	394E-05	186E-03	Foxk1	-670345	Hypo
chr3	52592264	52595046	Distal Interger	-0510	395E-05	186E-03	B3galt1	-215799	Hypo
chr6	34387843	34388538	Distal Interger	-0983	396E-05	186E-03	Vsnl1	-228364	Hypo
chr3	150719332	150728026	Intron (NM_0(	-0382	397E-05	187E-03	Ptprt	260637	Hypo
chr1	177179512	177180597	Distal Interger	-0768	397E-05	187E-03	Cacng3	-21393	Hypo
chr17	16947240	16947940	Distal Interger	-0912	398E-05	187E-03	Id4	-555284	Hypo
chr13	76624063	76627098	Promoter (<=1	-0412	400E-05	188E-03	Ccdc181	0	Hypo
chr16	124970	127049	Distal Interger	-0572	402E-05	189E-03	Rps24	35333	Hypo
chr6	72555619	72557122	Exon (NM_001	-0664	402E-05	189E-03	RGD1304624	30048	Hypo
chr6	19935835	19941451	Intron (NM_0(	-0477	403E-05	189E-03	Rasgrp3	17037	Hypo
chr7	26783835	26784518	Distal Interger	-0857	404E-05	189E-03	Mir135a	-171746	Hypo
chr12	31893234	31895063	Distal Interger	-0614	406E-05	189E-03	Atp6v0a2	84714	Hypo
chr16	82892982	82894799	Distal Interger	-0620	406E-05	190E-03	Slc10a2	-1491729	Hypo
chr5	61084758	61089542	Intron (NM_0:	-0469	408E-05	190E-03	Tbc1d2	-153406	Hypo
chr20	14118804	14120169	Intron (NM_0(	-0681	409E-05	191E-03	Gnaz	-448897	Hypo
chr5	120240803	120242522	Distal Interger	-0641	410E-05	191E-03	Plpp3	313718	Hypo
chr20	23346357	23346669	Distal Interger	-1135	412E-05	192E-03	Lrrtm3	-1168958	Hypo
chr9	50781871	50783412	Distal Interger	-0687	414E-05	192E-03	Tmeff2	-48396	Hypo
chr4	51361157	51362942	Distal Interger	-0644	415E-05	193E-03	Ptprz1	-34659	Hypo
chr2	45226559	45227973	Distal Interger	-0598	418E-05	194E-03	Snx18	30372	Hypo
chr6	138102760	138112291	Intron (NM_0:	-0367	421E-05	195E-03	Mir153	-51304	Hypo

chr9	62878265	62879787	Distal Intergen	-0679	424E-05	196E-03	Pard3b	-159218	Hypo
chr2	172263331	172265829	Intron (NM_0	-0501	425E-05	196E-03	Dclk2	72421	Hypo
chr1	257215420	257218381	Distal Intergen	-0511	425E-05	196E-03	Gfra1	332402	Hypo
chr4	26386921	26387952	Distal Intergen	-0748	433E-05	199E-03	Tex47	259441	Hypo
chr20	28249558	28250930	Exon (NM_05	-0685	434E-05	199E-03	Vsir	-30689	Hypo
chr8	95479725	95480128	Intron (NM_0	-1080	435E-05	199E-03	Slc9a9	246820	Hypo
chr14	69001085	69003381	Distal Intergen	-0574	435E-05	200E-03	Rab28	-242645	Hypo
chr1	231217465	231224801	Intron (NM_0	-0408	436E-05	200E-03	Rps4x-ps1	7188	Hypo
chr2	61583287	61586452	Intron (NM_0	-0496	436E-05	200E-03	Pdzd2	34694	Hypo
chr17	8393343	8394914	Distal Intergen	-0572	437E-05	200E-03	Neurog1	30465	Hypo
chr18	70841566	70843754	Intron (NM_0	-0669	437E-05	200E-03	Loxhd1	23290	Hypo
chr2	143521532	143524403	Distal Intergen	-0488	437E-05	200E-03	P2ry12	-36120	Hypo
chr18	14804673	14808926	Distal Intergen	-0438	440E-05	201E-03	Mapre2	-262445	Hypo
chr2	23470086	23471571	Intron (NM_0	-0647	440E-05	201E-03	Rasgrf2	-109131	Hypo
chr12	4873688	4876391	Distal Intergen	-0498	443E-05	201E-03	Lnc001	-6035	Hypo
chr16	46669123	46670490	Distal Intergen	-0756	443E-05	201E-03	Sorbs2	-42675	Hypo
chr1	207430128	207442290	Distal Intergen	-0373	444E-05	202E-03	Cd6	39344	Hypo
chr5	83195786	83197217	Distal Intergen	-0610	447E-05	203E-03	Brinp1	-702639	Hypo
chr14	65668806	65675665	Promoter (<=1	-0472	448E-05	203E-03	Qdpr	0	Hypo
chr9	6619747	6620241	Distal Intergen	-0925	449E-05	203E-03	Pp2d1	-86108	Hypo
chr18	48075859	48078343	Distal Intergen	-0609	453E-05	205E-03	Zfp608	332122	Hypo
chrX	120074642	120075585	Distal Intergen	-0885	455E-05	205E-03	Gria3	-163403	Hypo
chr2	175777664	175779773	Intron (NM_0	-0526	455E-05	205E-03	Gatad2b	28231	Hypo
chr8	119776237	119782991	Distal Intergen	-0395	456E-05	205E-03	Cx3cr1	16440	Hypo
chr1	209039154	209040145	Promoter (<=1	-0791	458E-05	206E-03	Or4d6	0	Hypo
chr2	170361266	170363567	Distal Intergen	-0579	458E-05	206E-03	Dear	-61084	Hypo
chr5	120214074	120215203	Distal Intergen	-0777	461E-05	206E-03	Plpp3	286989	Hypo
chr1	201921621	201923080	Distal Intergen	-0741	463E-05	207E-03	Rce1	-19163	Hypo
chr10	15711198	15718235	Distal Intergen	-0395	464E-05	207E-03	RGD1311343	-17253	Hypo
chr3	90696166	90698769	Distal Intergen	-0509	466E-05	208E-03	Hipk3	167543	Hypo
chr16	80193288	80196509	Distal Intergen	-0543	466E-05	208E-03	Arglu1	-556806	Hypo
chr18	3022980	3027514	Distal Intergen	-0450	469E-05	209E-03	Cables1	-49765	Hypo
chr20	28251080	28252135	Exon (NM_05	-0752	470E-05	209E-03	Vsir	-29484	Hypo

chr7	119716378	119718850	Distal Interger	-0566	471E-05	209E-03	Zdhhc25	23699	Hypo
chr20	37158773	37159313	Distal Interger	-0968	471E-05	209E-03	Smpdl3a	26181	Hypo
chr16	46656150	46656964	Distal Interger	-0728	472E-05	210E-03	Sorbs2	-29702	Hypo
chr18	48130455	48132118	Distal Interger	-0666	472E-05	210E-03	Zfp608	278347	Hypo
chr13	61953294	61955487	Intron (NM_1	-0556	475E-05	210E-03	Pla2g4a	66774	Hypo
chr16	2446776	2448626	Distal Interger	-0727	476E-05	210E-03	Arhgef3	-63885	Hypo
chr13	78559897	78562624	Distal Interger	-0478	478E-05	211E-03	Mael	-63240	Hypo
chr3	87699867	87701187	Distal Interger	-0693	483E-05	213E-03	Rag2	-201186	Hypo
chr15	12925763	12927058	Exon (NM_134	-0657	483E-05	213E-03	Cep15	-90715	Hypo
chr17	39026507	39027587	Distal Interger	-0733	485E-05	213E-03	Nrsn1	-778651	Hypo
chr20	24528221	24531309	Intron (NM_0	-0444	485E-05	213E-03	Lrrtm3	12594	Hypo
chr4	105934494	105935087	Distal Interger	-1039	485E-05	213E-03	Jkampl	-412952	Hypo
chr7	34151011	34153290	Distal Interger	-0536	485E-05	213E-03	Dusp6	58055	Hypo
chr3	52806780	52808455	Promoter (2-3	-0606	486E-05	213E-03	B3galt1	-2390	Hypo
chr2	143491122	143497978	Distal Interger	-0417	486E-05	213E-03	P2ry12	-5710	Hypo
chr13	76774619	76775677	Intron (NM_1	-0758	487E-05	213E-03	Atp1b1	31419	Hypo
chr12	11615437	11619005	Distal Interger	-0503	489E-05	214E-03	Fscn1	-5254	Hypo
chr15	77840271	77841895	Distal Interger	-0600	490E-05	214E-03	Commd6	632487	Hypo
chr2	60432519	60433041	Distal Interger	-0975	491E-05	214E-03	Tars1	-44862	Hypo
chr7	54534705	54535560	Distal Interger	-0661	491E-05	214E-03	Dyrk2	-172675	Hypo
chr10	97864965	97866446	Distal Interger	-0593	491E-05	214E-03	Sox9	58480	Hypo
chr4	53520217	53521728	Distal Interger	-0682	491E-05	214E-03	Tmem229a	-74890	Hypo
chr18	51983594	51987576	Distal Interger	-0483	493E-05	215E-03	Slc27a6	-53498	Hypo
chr13	30679650	30684180	Distal Interger	-0395	494E-05	215E-03	Tmem185b	32117	Hypo
chr1	20105219	20107837	Intron (NM_0	-0436	496E-05	216E-03	Ccnb2	-3983	Hypo
chr8	76006287	76010297	Exon (NM_00	-0465	500E-05	217E-03	Myo5c	16759	Hypo
chr2	37405095	37407241	Distal Interger	-0611	502E-05	218E-03	Htr1a	710921	Hypo
chr18	50612205	50614435	Distal Interger	-0488	504E-05	218E-03	Megf10	-35290	Hypo
chrX	11606626	11607459	Distal Interger	-0948	504E-05	218E-03	Mid1ip1	455716	Hypo
chr1	91534294	91537446	Distal Interger	-0588	504E-05	218E-03	LOC365238	-450904	Hypo
chr6	127335645	127343957	Distal Interger	-0393	505E-05	218E-03	Eml1	-25302	Hypo
chr17	70131488	70134957	Distal Interger	-0485	505E-05	218E-03	Celf2	-1274653	Hypo
chr2	33119184	33119611	Distal Interger	-1073	506E-05	218E-03	Pik3r1	-165977	Hypo

chr11	57824016	57825381	Distal Interger	-0430	506E-05	218E-03	Zbtb20	-32802	Hypo
chr18	74755468	74757581	Distal Interger	-0557	506E-05	218E-03	Sall3	-341640	Hypo
chr2	42637792	42638982	Distal Interger	-0734	508E-05	219E-03	Actbl2	-219038	Hypo
chr2	24444974	24445890	Distal Interger	-0900	508E-05	219E-03	Tent2	-12905	Hypo
chr17	70355182	70356575	Distal Interger	-0537	511E-05	220E-03	Celf2	-1053035	Hypo
chr5	90429191	90439047	Distal Interger	-0430	512E-05	220E-03	Dmac1	-1185434	Hypo
chr4	167479410	167483010	Intron (NM_0(	-0428	513E-05	220E-03	Borcs5	6037	Hypo
chr3	140280359	140281036	Distal Interger	-0949	513E-05	221E-03	Psmf1	-19813	Hypo
chr17	22033810	22041980	Promoter (<=1	-0368	513E-05	221E-03	Phactr1	0	Hypo
chr2	103387292	103387901	Distal Interger	-0935	516E-05	221E-03	Agtr1b	-467060	Hypo
chr13	84329852	84330590	Distal Interger	-0947	516E-05	221E-03	Cd84	-16152	Hypo
chr10	78484718	78485661	Distal Interger	-0824	517E-05	221E-03	Utp18	353193	Hypo
chr15	79723703	79726646	Distal Interger	-0531	517E-05	221E-03	Acod1	-145181	Hypo
chr17	54576932	54582180	Intron (NM_0(	-0397	519E-05	222E-03	Epc1	12245	Hypo
chr6	118782641	118783379	Intron (NM_0(	-0890	521E-05	222E-03	Foxn3	59022	Hypo
chr4	172742424	172743016	Intron (NM_0!	-0955	521E-05	222E-03	Plcz1	185342	Hypo
chr1	176923177	176928404	Intron (NM_0	-0398	522E-05	223E-03	Prkcb	90492	Hypo
chr5	60156284	60156542	Distal Interger	-1469	523E-05	223E-03	Stra6l	49199	Hypo
chr5	82681630	82684233	Distal Interger	-0517	524E-05	223E-03	Brinp1	-188483	Hypo
chr1	132654357	132655013	Distal Interger	-1154	524E-05	223E-03	Mrpl46	52626	Hypo
chr4	168669155	168671438	Intron (NM_0	-0581	524E-05	223E-03	Grin2b	370841	Hypo
chrX	57166700	57167291	Distal Interger	-1084	525E-05	223E-03	Mageb18	-278455	Hypo
chr2	118257340	118259326	Distal Interger	-0603	525E-05	223E-03	Dcun1d1	507992	Hypo
chr18	34100637	34107421	Exon (NM_001	-0432	528E-05	224E-03	Sh3rf2	53758	Hypo
chr2	25339838	25341141	Distal Interger	-0747	530E-05	224E-03	Lhfpl2	-5030	Hypo
chr1	216314558	216321730	Distal Interger	-0410	530E-05	224E-03	C1h9orf40	185871	Hypo
chr1	2097776	2098511	Intron (NM_0(	-0841	531E-05	225E-03	Lrp11	18172	Hypo
chr3	28452270	28452711	Intron (NM_0(	-1054	532E-05	225E-03	Arhgap15	462637	Hypo
chr3	68255112	68255415	Distal Interger	-1129	532E-05	225E-03	Zc3h15	-489293	Hypo
chr14	87597548	87599284	Distal Interger	-0631	533E-05	225E-03	Cobl	-618009	Hypo
chr9	46026011	46028447	Distal Interger	-0447	534E-05	225E-03	Tpp2	-18358	Hypo
chr2	175734659	175737316	Distal Interger	-0537	534E-05	225E-03	Gatad2b	-12117	Hypo
chr3	129590215	129591417	Distal Interger	-0658	535E-05	226E-03	Kif16b	662602	Hypo

chr1	225525240	225526210	Intron (NM_0(	-0733	535E-05	226E-03	Rfx3	182632	Hypo
chr16	67382303	67382842	Intron (NM_0(	-0854	537E-05	226E-03	Adam18	29887	Hypo
chr14	74445081	74446825	Intron (NM_0(	-0628	537E-05	226E-03	Sorcs2	73666	Hypo
chr3	17588050	17588772	Intron (NM_0(	-0943	538E-05	226E-03	Pbx3	93578	Hypo
chr8	113389537	113391734	Distal Interger	-0553	538E-05	226E-03	Pdcd6ip	255039	Hypo
chr20	36197056	36198356	Distal Interger	-0640	539E-05	226E-03	Gja1	441021	Hypo
chr5	39125785	39128209	Distal Interger	-0522	541E-05	227E-03	Ufl1	-130651	Hypo
chr13	50964344	50965208	Intron (NM_0(	-0837	544E-05	228E-03	Crb1	24053	Hypo
chr2	192773352	192773832	Distal Interger	-1131	546E-05	228E-03	Kcnd3	-164118	Hypo
chr14	94039422	94042627	Distal Interger	-0465	548E-05	229E-03	Spred2	-107067	Hypo
chr1	40355163	40359530	Intron (NM_0(	-0530	549E-05	229E-03	Plekhg1	6479	Hypo
chr3	37204556	37208841	Exon (NM_00:	-0447	550E-05	230E-03	Stam2	25969	Hypo
chr9	49270853	49273420	Distal Interger	-0549	552E-05	230E-03	Gls	-71361	Hypo
chr2	45565856	45567620	Distal Interger	-0635	552E-05	230E-03	Hspb3	-269857	Hypo
chr18	1586420	1586989	Distal Interger	-0974	556E-05	231E-03	Esco1	83304	Hypo
chr8	119792313	119799429	Promoter (<=:	-0406	557E-05	232E-03	Cx3cr1	2	Hypo
chr11	50250585	50250955	Distal Interger	-1114	560E-05	233E-03	Bbx	-229658	Hypo
chr4	128606505	128608683	Distal Interger	-0576	560E-05	233E-03	Nup50-ps1	180613	Hypo
chr14	71968725	71970401	Distal Interger	-0676	561E-05	233E-03	Wdr1	-287631	Hypo
chr4	179130947	179132116	Intron (NM_0:	-0636	562E-05	233E-03	Sspn	233800	Hypo
chr1	168506824	168508745	Distal Interger	-0602	562E-05	233E-03	Psma1	-53384	Hypo
chr6	50778960	50779986	Intron (NM_0(	-0726	563E-05	233E-03	Twist1	104050	Hypo
chr7	40890725	40891804	Distal Interger	-0722	565E-05	234E-03	Tmtc2	-84074	Hypo
chr1	130011454	130012281	Distal Interger	-0864	567E-05	234E-03	Klhl25	286504	Hypo
chr1	193455930	193457399	Distal Interger	-0636	567E-05	234E-03	Mapk1ip1	199344	Hypo
chr13	33789196	33790654	Distal Interger	-0682	567E-05	234E-03	Ddx18	-1046035	Hypo
chr2	24297875	24304338	Distal Interger	-0428	570E-05	235E-03	Tent2	127731	Hypo
chr3	58476344	58483836	Distal Interger	-0374	572E-05	236E-03	Chrna1	-6512	Hypo
chr7	106473408	106487187	3' UTR	-0404	573E-05	236E-03	Adgrb1	55956	Hypo
chr14	9471343	9472847	Intron (NM_0(	-0696	576E-05	237E-03	Tmem150c	15996	Hypo
chr1	8833868	8834434	Distal Interger	-0976	576E-05	237E-03	Vta1	246166	Hypo
chr10	9137769	9139694	Distal Interger	-0625	581E-05	239E-03	Rbfox1	-644996	Hypo
chr1	189407942	189409976	Distal Interger	-0626	583E-05	239E-03	Dock1	-57167	Hypo

chr3	118214457	118215230	Intron (NM_0:	-0852	586E-05	240E-03	Gfra4	42693	Hypo
chr17	61073727	61074930	Distal Interger	-0795	586E-05	240E-03	Dip2c	-41886	Hypo
chr19	20711817	20713027	Distal Interger	-0666	588E-05	241E-03	Abcc12	162412	Hypo
chr9	41296001	41296825	Distal Interger	-0839	592E-05	242E-03	Pdcl3	61679	Hypo
chr5	37630230	37631170	Distal Interger	-0807	592E-05	242E-03	Mms22l	-566609	Hypo
chr1	164046670	164048311	Distal Interger	-0687	595E-05	243E-03	Ipo7	-14391	Hypo
chr13	42926080	42928486	Intron (NM_0(	-0570	597E-05	244E-03	Srgap2	38572	Hypo
chr1	171393704	171395102	Distal Interger	-0681	603E-05	246E-03	Xylt1	-248823	Hypo
chr1	242910663	242912613	Promoter (1-2	-0531	604E-05	246E-03	Cyp2c23	1245	Hypo
chr2	53443482	53444423	Distal Interger	-0715	606E-05	246E-03	Plcxd3	-122599	Hypo
chr19	39186677	39187725	Intron (NM_0	-0751	611E-05	247E-03	Glg1	36453	Hypo
chr1	41844434	41845789	Distal Interger	-0575	611E-05	248E-03	Myct1	-172348	Hypo
chr19	20518445	20519762	Distal Interger	-0689	613E-05	248E-03	Lonp2	-19581	Hypo
chr4	179132243	179136675	Intron (NM_0:	-0450	615E-05	248E-03	Sspn	235096	Hypo
chr10	82869002	82872816	Distal Interger	-0460	615E-05	248E-03	Lasp1	73825	Hypo
chr16	72883869	72886904	Intron (NM_0(	-0537	616E-05	248E-03	Csmd1	665104	Hypo
chr3	21800205	21801751	Intron (NM_0(	-0650	617E-05	249E-03	Dennd1a	193122	Hypo
chr3	96703976	96704968	Distal Interger	-0873	619E-05	249E-03	Ccdc34	150984	Hypo
chr16	54623050	54623980	Intron (NM_0(	-0819	622E-05	250E-03	Mir383	582710	Hypo
chr1	4092725	4093086	Promoter (1-2	-0970	622E-05	250E-03	Samd5	-1816	Hypo
chr14	59298177	59300008	Distal Interger	-0584	624E-05	251E-03	Ppargc1a	-121598	Hypo
chr9	54527325	54529416	Distal Interger	-0535	628E-05	252E-03	Slc39a10	-381586	Hypo
chr17	22867841	22868408	Distal Interger	-0996	629E-05	252E-03	Adtrp	-62332	Hypo
chr2	60430973	60432198	Distal Interger	-0669	629E-05	252E-03	Tars1	-43316	Hypo
chr20	21689657	21690886	Distal Interger	-0724	631E-05	252E-03	Reep3	195789	Hypo
chr7	29825879	29829579	Distal Interger	-0478	631E-05	252E-03	Cradd	123328	Hypo
chr7	68593819	68594750	Intron (NM_0(	-0797	631E-05	252E-03	Ncald	34011	Hypo
chr6	3831760	3836137	Intron (NM_0:	-0451	631E-05	252E-03	Nrxn1	650470	Hypo
chr8	48104636	48106479	Intron (NM_0(	-0621	632E-05	252E-03	Cadm1	256800	Hypo
chr14	45242749	45242974	Distal Interger	-1253	632E-05	252E-03	LOC498368	-764708	Hypo
chr8	109429230	109431174	Intron (NM_0	-0622	633E-05	252E-03	Prkar2a	33395	Hypo
chr11	57782743	57792158	Promoter (<=:	-0389	634E-05	253E-03	Zbtb20	0	Hypo
chr10	3003893	3004954	Distal Interger	-0590	636E-05	254E-03	Cpped1	-696568	Hypo

chr10	88251266	88253348	Distal Interger	-0538	637E-05	254E-03	Map3k14	-35743	Hypo
chr11	69072988	69077083	Intron (NM_0	-0414	637E-05	254E-03	Dlg1	25720	Hypo
chr3	115947694	115954263	Intron (NM_0:	-0423	638E-05	254E-03	Mertk	8343	Hypo
chr19	20756481	20759984	Distal Interger	-0406	644E-05	256E-03	Abcc12	207076	Hypo
chr20	9735970	9736783	Distal Interger	-0682	646E-05	256E-03	Cbs	-3347	Hypo
chr19	11281725	11283479	Distal Interger	-0553	647E-05	256E-03	Gnao1	-89232	Hypo
chr1	249344798	249345345	Intron (NM_0(	-0869	648E-05	257E-03	Sorcs1	248934	Hypo
chr19	24869175	24871179	Intron (NM_0(	-0556	648E-05	257E-03	Ucp1	-52322	Hypo
chr11	78635088	78637548	Exon (NM_001	-0520	651E-05	257E-03	Etv5	26378	Hypo
chr17	70557082	70557562	Distal Interger	-0992	652E-05	258E-03	Celf2	-852048	Hypo
chr17	8388675	8390590	Distal Interger	-0509	653E-05	258E-03	Neurog1	25797	Hypo
chr3	17669776	17671027	Intron (NM_0(	-0698	655E-05	258E-03	Pbx3	11323	Hypo
chr1	77259219	77262395	Intron (NM_0(	-0487	655E-05	258E-03	Arhgap35	56903	Hypo
chr13	105763535	105765178	Distal Interger	-0713	656E-05	259E-03	Plxna2	-397925	Hypo
chr2	100195837	100197505	Distal Interger	-0695	657E-05	259E-03	Mir124-2	60971	Hypo
chr13	33625656	33625903	Distal Interger	-1404	658E-05	259E-03	Ddx18	-882495	Hypo
chr8	121117845	121119847	Exon (NM_001	-0594	661E-05	260E-03	Cck	40237	Hypo
chr7	44899254	44900453	Distal Interger	-0721	663E-05	261E-03	Nav3	491806	Hypo
chr2	98737205	98737806	Distal Interger	-0912	665E-05	261E-03	Ythdf3	386544	Hypo
chr8	119787465	119789967	Intron (NM_1:	-0656	666E-05	261E-03	Cx3cr1	9464	Hypo
chr10	47648282	47648708	Distal Interger	-1025	666E-05	261E-03	Tekt3	-80927	Hypo
chr15	80510898	80518844	Intron (NM_0(	-0465	667E-05	261E-03	Slain1	12251	Hypo
chr15	42146546	42148041	Intron (NM_0(	-0669	670E-05	262E-03	Dock5	10608	Hypo
chr4	140508996	140510491	Promoter (2-3	-0587	670E-05	262E-03	Lrrn1	-2726	Hypo
chr7	125920389	125920832	Intron (NM_0(	-1012	671E-05	262E-03	Tmem117	162798	Hypo
chr4	121180075	121181797	Distal Interger	-0615	671E-05	262E-03	Mgll	-10423	Hypo
chr2	81288061	81288567	Distal Interger	-0867	671E-05	262E-03	Ctnnd2	-41087	Hypo
chr1	50541037	50543377	Distal Interger	-0574	675E-05	263E-03	Qki	153339	Hypo
chr8	61495578	61497136	Distal Interger	-0597	677E-05	264E-03	LOC691000	-9529	Hypo
chr14	102759299	102760311	Distal Interger	-0718	678E-05	264E-03	Pnpt1	-117242	Hypo
chr1	138352246	138357569	Intron (NM_0	-0380	679E-05	264E-03	Arnt2	35197	Hypo
chr14	43279867	43280990	Distal Interger	-0722	682E-05	265E-03	Tmem156	56448	Hypo
chr8	8289294	8292532	Distal Interger	-0492	682E-05	265E-03	Cntn5	-839045	Hypo

chrX	98939918	98942090	Distal Intergen	-0584	682E-05	265E-03	Arxes1	-8440	Hypo
chr7	96155913	96156318	Distal Intergen	-0929	682E-05	265E-03	Asap1	-97544	Hypo
chr5	117171677	117179854	Intron (NM_0	-0445	687E-05	266E-03	Pde4b	178241	Hypo
chr17	70195096	70198198	Distal Intergen	-0496	690E-05	268E-03	Celf2	-1211412	Hypo
chr4	176077132	176078104	Distal Intergen	-0742	693E-05	269E-03	C2cd5	-21574	Hypo
chr8	61633258	61635309	Distal Intergen	-0535	694E-05	269E-03	LOC691000	126593	Hypo
chr2	134926075	134928768	Distal Intergen	-0514	694E-05	269E-03	Noct	-342421	Hypo
chr7	34325310	34326333	Distal Intergen	-0760	695E-05	269E-03	Dusp6	232354	Hypo
chr3	136359706	136362619	Distal Intergen	-0527	698E-05	270E-03	Cst3	-18910	Hypo
chr4	145430300	145430710	Intron (NM_0	-1011	698E-05	270E-03	Lmcd1	37147	Hypo
chr6	22583854	22586565	Intron (NM_0	-0498	700E-05	270E-03	Lbh	6491	Hypo
chr6	29226763	29227450	Distal Intergen	-0850	701E-05	270E-03	Klhl29	-836116	Hypo
chr1	50276033	50276400	Intron (NM_0	-1201	703E-05	271E-03	Cahm	110559	Hypo
chr9	41088630	41092308	Distal Intergen	-0485	705E-05	271E-03	Chst10	30369	Hypo
chr10	73159885	73161129	Distal Intergen	-0612	705E-05	271E-03	Ccdc182	69657	Hypo
chrX	57167701	57168353	Distal Intergen	-0992	708E-05	272E-03	Mageb18	-279456	Hypo
chr5	79572078	79572973	Intron (NM_0	-0728	709E-05	272E-03	Astn2	170900	Hypo
chr1	23286723	23289129	Distal Intergen	-0619	716E-05	274E-03	Sgk1	-298626	Hypo
chr3	116644945	116648774	Distal Intergen	-0462	717E-05	274E-03	Il1b	-61559	Hypo
chr10	69170622	69172622	Exon (NM_02	-0522	719E-05	275E-03	Acaca	87800	Hypo
chr1	25012326	25013224	Distal Intergen	-0824	720E-05	275E-03	Trdn	-601913	Hypo
chr18	70920121	70920403	Intron (NM_0	-1317	722E-05	276E-03	Loxhd1	101845	Hypo
chr2	170868871	170876136	Distal Intergen	-0390	724E-05	276E-03	Fhip1a	215346	Hypo
chr13	48961824	48964007	Distal Intergen	-0537	725E-05	277E-03	Mir181a-1	-521520	Hypo
chr13	77807070	77808015	Exon (NM_02	-0878	731E-05	279E-03	Adcy10	55758	Hypo
chr8	26754718	26756543	Distal Intergen	-0563	731E-05	279E-03	Opcml	-33125	Hypo
chr18	78488845	78490377	Distal Intergen	-0645	734E-05	279E-03	Timm21	-169483	Hypo
chr17	20985885	20987589	Distal Intergen	-0534	743E-05	282E-03	Cd83	-79182	Hypo
chr5	96256516	96259037	Distal Intergen	-0537	744E-05	282E-03	Mpdz	-336017	Hypo
chr2	115900022	115909044	Promoter (<=1	-0370	746E-05	283E-03	Pex5l	0	Hypo
chr19	4866134	4867048	Distal Intergen	-0642	748E-05	284E-03	Cdh8	-627126	Hypo
chr11	43239460	43244690	Promoter (<=1	-0429	748E-05	284E-03	Tmem30c	0	Hypo
chr13	49279898	49286392	Distal Intergen	-0475	750E-05	284E-03	Mir181a-1	-199135	Hypo

chr9	3544369	3550655	Intron (NM_0(	-0399	751E-05	284E-03	Plcl2	251001	Hypo
chr13	103215959	103218206	Distal Interger	-0560	755E-05	285E-03	Ints7	60836	Hypo
chr1	220860157	220862293	Distal Interger	-0503	756E-05	286E-03	Smc5	-21061	Hypo
chr19	16175184	16177344	Distal Interger	-0485	759E-05	286E-03	Rbl2	-251612	Hypo
chr3	52714347	52717021	Distal Interger	-0505	759E-05	286E-03	B3galt1	-93824	Hypo
chr2	220159916	220160496	Distal Interger	-0916	763E-05	288E-03	Papss1	119532	Hypo
chr17	49705068	49707065	Promoter (2-3	-0632	764E-05	288E-03	Gli3	2647	Hypo
chr19	8640953	8643026	Distal Interger	-0507	768E-05	289E-03	Got2	-531278	Hypo
chr3	116052914	116053599	Distal Interger	-0973	776E-05	292E-03	Tmem87b	-8617	Hypo
chr5	54195439	54198030	Distal Interger	-0540	777E-05	292E-03	RGD1306195	-257975	Hypo
chr20	8461876	8464128	Intron (NM_0(	-0538	779E-05	292E-03	Btbd9	175716	Hypo
chr12	28014488	28015350	Distal Interger	-0672	779E-05	292E-03	Piwil1	-43908	Hypo
chr6	130578032	130580019	Distal Interger	-0515	780E-05	292E-03	Eif5	-9181	Hypo
chr11	53043719	53044524	Distal Interger	-0878	784E-05	294E-03	Morc1	-659464	Hypo
chr2	230634044	230635786	Distal Interger	-0541	789E-05	296E-03	Bmpr1b	-59445	Hypo
chr14	5631023	5644019	Promoter (<=1	-0332	790E-05	296E-03	Sparcl1	0	Hypo
chr6	57722332	57724136	Distal Interger	-0593	792E-05	296E-03	Ifrd1	-433426	Hypo
chr16	2450986	2453941	Distal Interger	-0544	792E-05	296E-03	Arhgef3	-58570	Hypo
chr1	56997731	56998961	Distal Interger	-0732	793E-05	296E-03	Chd1	333677	Hypo
chr4	178032486	178035574	Distal Interger	-0517	793E-05	296E-03	Bcat1	-19103	Hypo
chr2	196201377	196203202	Promoter (2-3	-0525	795E-05	296E-03	Taf13	-2017	Hypo
chr10	30908782	30909766	3' UTR	-0787	796E-05	297E-03	Havcr2	26176	Hypo
chr2	74944168	74948677	Distal Interger	-0451	796E-05	297E-03	Basp1	915124	Hypo
chr1	216334440	216336260	Distal Interger	-0580	797E-05	297E-03	C1h9orf40	205753	Hypo
chr8	122100786	122101436	Distal Interger	-0868	799E-05	297E-03	Abhd5	100417	Hypo
chr5	97629083	97632656	Exon (NM_001	-0435	799E-05	297E-03	Ttc39b	75414	Hypo
chr7	119506120	119507668	Distal Interger	-0613	800E-05	297E-03	Zdhhc25	-185011	Hypo
chr8	57812496	57814280	Distal Interger	-0714	800E-05	297E-03	Ppcdc	24488	Hypo
chr1	87953969	87956079	Exon (NM_001	-0580	801E-05	298E-03	Gpatch1	18465	Hypo
chr8	47212600	47215974	Distal Interger	-0452	802E-05	298E-03	LOC500990	-602126	Hypo
chr4	65570327	65571317	Exon (NM_198	-0912	805E-05	298E-03	Ptn	-195021	Hypo
chr2	244118991	244120649	Promoter (<=1	-0427	807E-05	299E-03	Fpgt	0	Hypo
chr1	193655895	193658689	Promoter (<=1	-0559	808E-05	300E-03	Mapk1ip1	0	Hypo

chr9	25054623	25056969	Intron (NM_0	-0556	809E-05	300E-03	Rims1	139399	Hypo
chr2	12920385	12922995	Distal Interger	-0533	813E-05	301E-03	Cetn3	830968	Hypo
chr16	75006309	75012394	Intron (NM_0	-0421	816E-05	301E-03	Cln8	-246756	Hypo
chr2	176042137	176043139	Distal Interger	-0740	818E-05	302E-03	S100a16	25732	Hypo
chr14	7441421	7442858	Distal Interger	-0628	819E-05	302E-03	Wdfy3	-163770	Hypo
chr1	126936043	126939679	Distal Interger	-0441	821E-05	302E-03	Rgma	-189255	Hypo
chr13	105270916	105271983	Distal Interger	-0761	821E-05	302E-03	Mir205	-234931	Hypo
chr1	172109560	172111084	Distal Interger	-0676	823E-05	303E-03	Rps15a	314953	Hypo
chr1	252138238	252140930	Distal Interger	-0517	823E-05	303E-03	Add3	-6456	Hypo
chr18	73784218	73785612	Intron (NM_0	-0668	834E-05	306E-03	Kcng2	23111	Hypo
chr1	52214867	52220274	Distal Interger	-0405	838E-05	307E-03	Tbxt	85590	Hypo
chr1	255483328	255486429	Distal Interger	-0461	840E-05	308E-03	Plekhs1	-9205	Hypo
chr5	48359364	48360412	Distal Interger	-0670	841E-05	308E-03	Cnr1	-51608	Hypo
chr5	135481565	135483166	Intron (NM_0	-0563	844E-05	309E-03	Nt5c1a	8334	Hypo
chr7	24541770	24542373	Intron (NM_0	-0763	844E-05	309E-03	Anks1b	227641	Hypo
chr18	38153405	38155726	Intron (NM_0	-0582	846E-05	309E-03	Kcnn2	39179	Hypo
chr2	234786072	234787123	Intron (NM_0	-0743	849E-05	310E-03	Bcl10	-53757	Hypo
chr14	12776698	12778412	5' UTR	-0628	849E-05	310E-03	Anxa3	3222	Hypo
chr10	88572326	88575272	Distal Interger	-0477	849E-05	310E-03	Rprml	12078	Hypo
chr3	117869088	117877217	3' UTR	-0381	852E-05	310E-03	Ddrgk1	5463	Hypo
chr6	5485773	5488450	Distal Interger	-0539	852E-05	310E-03	Lhcgr	-173421	Hypo
chr3	96469789	96472187	Intron (NM_1	-0539	852E-05	310E-03	Lgr4	21929	Hypo
chr3	47152450	47157574	Exon (NM_13	-0451	853E-05	310E-03	Gcg	-29521	Hypo
chr4	139274985	139277463	Intron (NM_0	-0537	858E-05	312E-03	Il5ra	386506	Hypo
chr3	76588811	76590554	Distal Interger	-0621	860E-05	313E-03	Ptprj	-27142	Hypo
chr13	66159366	66161608	Distal Interger	-0511	866E-05	315E-03	Glul	133725	Hypo
chr11	64027254	64028554	Intron (NM_0	-0639	868E-05	315E-03	Slc15a2	13072	Hypo
chr5	165110648	165114904	Distal Interger	-0465	869E-05	315E-03	Actrt2	122588	Hypo
chr2	60484969	60486147	Distal Interger	-0711	870E-05	315E-03	Tars1	-97312	Hypo
chr20	42470898	42473026	Intron (NM_0	-0548	870E-05	315E-03	Lama4	78630	Hypo
chr7	124977975	124981792	Distal Interger	-0379	873E-05	316E-03	Prickle1	-319862	Hypo
chr16	37425511	37428222	Distal Interger	-0531	875E-05	317E-03	Spcs3	120153	Hypo
chr16	55259831	55261101	Distal Interger	-0701	877E-05	317E-03	LOC688765	-13189	Hypo

chr5	76752635	76753372	Intron (NM_1!	-0721	878E-05	317E-03	Orm1	-19624	Hypo
chr1	129776363	129777132	Distal Interger	-0876	879E-05	317E-03	Klhl25	51413	Hypo
chr3	29519846	29521550	Distal Interger	-0696	882E-05	318E-03	Zeb2-as1	176768	Hypo
chr17	16523921	16524840	Distal Interger	-0780	884E-05	319E-03	Id4	-131965	Hypo
chr18	42424769	42426721	Distal Interger	-0529	885E-05	319E-03	Dtwd2	475143	Hypo
chr10	92718552	92720178	Intron (NM_0	-0598	887E-05	319E-03	Cacng4	25423	Hypo
chr4	126178950	126180848	Distal Interger	-0633	888E-05	319E-03	Magi1	629911	Hypo
chr3	88949259	88953064	Intron (NM_0	-0398	888E-05	319E-03	Slc1a2	-52065	Hypo
chr4	129419272	129421649	Distal Interger	-0540	889E-05	319E-03	Tafa4	249600	Hypo
chr6	6182926	6186542	Distal Interger	-0414	889E-05	319E-03	Ppp1r21	-212242	Hypo
chr8	75791488	75797624	Exon (NM_03	-0392	889E-05	319E-03	Arpp19	11532	Hypo
chr2	232264778	232266375	Distal Interger	-0536	893E-05	320E-03	Pkn2	-365825	Hypo
chr5	53573760	53574425	Distal Interger	-0970	898E-05	322E-03	RGD1306195	-881580	Hypo
chr4	54148861	54149774	Intron (NM_0	-0823	902E-05	323E-03	Gpr37	11153	Hypo
chr19	47167796	47172280	Intron (NM_1	-0465	907E-05	324E-03	Hsbp1	-228759	Hypo
chr1	176091985	176094243	Distal Interger	-0514	907E-05	324E-03	Hs3st2	135824	Hypo
chr7	32078932	32080925	Distal Interger	-0400	912E-05	325E-03	Dcn	-200327	Hypo
chr5	160709364	160712429	Distal Interger	-0482	917E-05	327E-03	Eno1	-7522	Hypo
chr1	217998418	218000950	Distal Interger	-0531	918E-05	327E-03	Aldh1a1	-110135	Hypo
chr2	193337266	193339756	Promoter (<=	-0578	920E-05	328E-03	Tmigd3	0	Hypo
chr14	44422439	44423984	Intron (NM_0	-0618	924E-05	329E-03	LOC498368	54057	Hypo
chr3	151436982	151437844	Distal Interger	-0871	925E-05	329E-03	Srsf6	-151702	Hypo
chr9	42308061	42309726	Exon (NM_00	-0675	925E-05	329E-03	Il1r2	-74703	Hypo
chr1	53929590	53930312	Intron (NM_0	-0890	927E-05	329E-03	Afdn	24214	Hypo
chr8	65467652	65470461	Promoter (1-2	-0513	927E-05	329E-03	Slc24a1	-1654	Hypo
chr2	232792988	232794435	Distal Interger	-0708	930E-05	330E-03	Lmo4	486446	Hypo
chr10	30911797	30914126	Distal Interger	-0528	931E-05	330E-03	Havcr2	29191	Hypo
chr8	11106787	11111039	Distal Interger	-0382	933E-05	330E-03	Sesn3	-22878	Hypo
chr1	243062159	243064030	Intron (NM_0	-0523	933E-05	330E-03	Pkd2l1	6001	Hypo
chr1	13825663	13828027	Distal Interger	-0533	937E-05	332E-03	Olig3	-253232	Hypo
chr17	69865186	69867453	Distal Interger	-0488	939E-05	332E-03	Gata3	1220273	Hypo
chr1	47054591	47056196	Distal Interger	-0410	941E-05	332E-03	Ezr	-43086	Hypo
chr19	1377270	1378918	Distal Interger	-0746	944E-05	333E-03	Cdh5	-522902	Hypo

chr2	239970652	239971669	Distal Interger	-0777	946E-05	334E-03	Adgrl4	-383272	Hypo
chr1	169789338	169790660	Intron (NM_0(	-0724	947E-05	334E-03	Sox6	440362	Hypo
chr3	62903493	62904748	Distal Interger	-0596	947E-05	334E-03	Zfp385b	-276050	Hypo
chr4	108743770	108744662	Distal Interger	-0860	949E-05	334E-03	LRRTM1	-957153	Hypo
chr18	6521182	6526155	Promoter (<=1	-0422	951E-05	334E-03	Aqp4	0	Hypo
chr17	67866399	67868256	Distal Interger	-0648	957E-05	336E-03	Mir466d	135103	Hypo
chr4	156082721	156085411	Promoter (<=1	-0533	958E-05	336E-03	C3ar1	0	Hypo
chr3	28726398	28729593	Distal Interger	-0478	959E-05	337E-03	Gtdc1	432026	Hypo
chr9	49288547	49290300	Distal Interger	-0594	963E-05	337E-03	Gls	-54481	Hypo
chr2	230681056	230683050	Distal Interger	-0652	966E-05	338E-03	Bmpr1b	-106457	Hypo
chr6	138372642	138374110	Distal Interger	-0629	966E-05	338E-03	Mir153	209047	Hypo
chr8	23717872	23724182	Promoter (<=1	-0354	967E-05	338E-03	Septin7	0	Hypo
chr3	80224020	80229517	Distal Interger	-0364	974E-05	340E-03	Mir129-2	-21880	Hypo
chr11	15897592	15900353	Distal Interger	-0559	975E-05	340E-03	Usp25	293711	Hypo
chr16	56057487	56059090	Distal Interger	-0590	978E-05	341E-03	Prag1	82063	Hypo
chr1	98705748	98706223	Distal Interger	-0996	978E-05	341E-03	E2f8	-121650	Hypo
chr2	24269446	24272232	Distal Interger	-0486	978E-05	341E-03	Tent2	159837	Hypo
chr2	14170026	14202812	Promoter (<=1	-0305	979E-05	341E-03	Mir9-2	0	Hypo
chr14	66289914	66290267	Intron (NM_0(	-1126	981E-05	341E-03	Ldb2	12829	Hypo
chr5	164088530	164089571	Distal Interger	-0799	981E-05	341E-03	Ajap1	-68213	Hypo
chr19	53526836	53530736	Intron (NM_0(	-0426	985E-05	342E-03	Sipa1l2	9692	Hypo
chr19	12220968	12222727	Distal Interger	-0673	986E-05	343E-03	Large1	-172038	Hypo
chr3	79137228	79139314	Distal Interger	-0520	987E-05	343E-03	Tp53i11	-5572	Hypo
chr16	42631678	42632806	Distal Interger	-0695	990E-05	343E-03	Tenm3	-895188	Hypo
chr8	110249339	110252735	Distal Interger	-0461	991E-05	344E-03	Elp6	-27324	Hypo
chr2	116568706	116569290	Distal Interger	-0962	991E-05	344E-03	Ttc14	-84305	Hypo
chr2	14834109	14843645	Distal Interger	-0343	994E-05	344E-03	Mir3597-2	-636281	Hypo
chr9	65335739	65338246	Distal Interger	-0475	101E-04	349E-03	Klf7	146474	Hypo
chr17	28373344	28375078	Intron (NM_0(	-0566	101E-04	349E-03	Nrn1	243375	Hypo
chr8	59721170	59727721	3' UTR	-0444	102E-04	351E-03	Adpgk	21726	Hypo
chr4	121653561	121659071	Distal Interger	-0420	102E-04	351E-03	Tpra1	289323	Hypo
chr6	98815914	98817813	Distal Interger	-0602	102E-04	352E-03	Zfp36l1	117935	Hypo
chr3	123658456	123661566	Intron (NM_0(	-0471	103E-04	352E-03	Pak5	42364	Hypo

chr1	95163878	95166784	Distal Interger	-0576	103E-04	353E-03	Myh14	-5042	Hypo
chr3	34582489	34584564	Distal Interger	-0577	103E-04	354E-03	Ns5atp4l1	-116019	Hypo
chr4	134243016	134243660	Distal Interger	-0842	103E-04	355E-03	Pdzrn3	-246057	Hypo
chr9	50926098	50927375	Distal Interger	-0826	104E-04	355E-03	Tmeff2	-192623	Hypo
chr3	139464011	139465216	Intron (NM_0(	-0587	104E-04	355E-03	Acss1	35109	Hypo
chr3	29432762	29434287	Distal Interger	-0609	104E-04	355E-03	Zeb2-as1	89684	Hypo
chr18	54700453	54701761	Intron (NM_0(	-0663	104E-04	356E-03	Pde6a	23590	Hypo
chr12	13675148	13676476	Distal Interger	-0664	104E-04	356E-03	Gna12	-129222	Hypo
chr1	105628027	105629611	Distal Interger	-0596	104E-04	356E-03	Tubgcp5	-1006915	Hypo
chr1	118327792	118328441	Distal Interger	-0782	104E-04	356E-03	Nsmce3	74188	Hypo
chr17	73821095	73825412	Intron (NM_0(	-0503	105E-04	356E-03	Frmd4a	121232	Hypo
chr16	29440130	29448448	Distal Interger	-0351	105E-04	357E-03	Mfap3l	-9267	Hypo
chr12	12846299	12846863	Distal Interger	-0806	105E-04	357E-03	Foxk1	-671210	Hypo
chr17	73827674	73828351	Intron (NM_0(	-0813	105E-04	357E-03	Frmd4a	118293	Hypo
chr7	94615679	94617335	Distal Interger	-0556	105E-04	357E-03	Gsdmc	988771	Hypo
chr10	4822082	4823056	Distal Interger	-0797	105E-04	357E-03	Prm1	-48853	Hypo
chr1	169135786	169136575	Distal Interger	-0887	105E-04	357E-03	Insc	142754	Hypo
chr7	33728047	33729730	Distal Interger	-0588	105E-04	357E-03	Atp2b1	-6245	Hypo
chr11	55571924	55573077	Exon (NM_21:	-0684	106E-04	359E-03	Btla	13905	Hypo
chr1	99476209	99479561	Promoter (2-3	-0447	107E-04	361E-03	Htatip2	2196	Hypo
chr20	40803184	40803793	Distal Interger	-0837	107E-04	361E-03	Marcks	-112172	Hypo
chr2	232318281	232319354	Distal Interger	-0670	107E-04	361E-03	Pkn2	-419328	Hypo
chr20	40786217	40787649	Distal Interger	-0585	107E-04	361E-03	Marcks	-95205	Hypo
chr5	165167492	165168457	Distal Interger	-0754	107E-04	361E-03	Actrt2	69035	Hypo
chr19	20695894	20700797	Distal Interger	-0386	107E-04	361E-03	Abcc12	146489	Hypo
chr2	24829565	24830017	Distal Interger	-0974	107E-04	361E-03	Bhmt	49432	Hypo
chr15	82576417	82578104	Distal Interger	-0470	107E-04	362E-03	Spry2	117220	Hypo
chr14	20441286	20443304	Distal Interger	-0543	107E-04	362E-03	Sult1d1	-14426	Hypo
chr5	160232972	160239167	Distal Interger	-0379	108E-04	363E-03	Slc25a33	-13011	Hypo
chr17	38502738	38503526	Distal Interger	-0737	108E-04	363E-03	Hdgfl1	294832	Hypo
chr15	51296899	51299969	Intron (NM_0:	-0439	108E-04	363E-03	Kctd4	22604	Hypo
chr1	132590327	132592038	Distal Interger	-0596	108E-04	363E-03	Ntrk3	-87041	Hypo
chr2	174973371	174973996	Intron (NM_0	-1029	108E-04	363E-03	Kcnn3	36736	Hypo

chr9	88292810	88293684	Intron (NM_0	-0740	108E-04	364E-03	Inpp5d	5131	Hypo
chr19	20945055	20946863	Distal Interger	-0511	109E-04	365E-03	Phkb	263742	Hypo
chr3	143881027	143884204	Promoter (<=1	-0370	109E-04	365E-03	Tp53inp2	0	Hypo
chr2	81688489	81691380	Intron (NM_0(	-0494	109E-04	365E-03	Ctnnd2	358835	Hypo
chr3	94604269	94605202	Distal Interger	-0827	109E-04	365E-03	Kcna4	843625	Hypo
chr1	52233979	52238635	Distal Interger	-0352	109E-04	365E-03	Tbxt	67229	Hypo
chr11	57642743	57644159	Intron (NM_0(	-0593	109E-04	366E-03	Zbtb20	147055	Hypo
chr8	52498982	52499418	Intron (NM_0(	-0995	109E-04	366E-03	Zc3h12c	4897	Hypo
chr7	85535454	85535745	Distal Interger	-1097	110E-04	368E-03	Tnfrsf11b	58781	Hypo
chr2	244102264	244103065	Distal Interger	-0723	110E-04	369E-03	Fpgt	16832	Hypo
chr19	43657949	43658545	Distal Interger	-0912	111E-04	370E-03	Maf	53823	Hypo
chr7	19013212	19016724	Distal Interger	-0441	111E-04	370E-03	Polr3b	125726	Hypo
chr14	7173621	7174446	Distal Interger	-0787	111E-04	371E-03	Arhgap24	-324126	Hypo
chr7	34298887	34301785	Distal Interger	-0448	111E-04	371E-03	Dusp6	205931	Hypo
chr9	110491517	110497127	Intron (NM_0	-0390	112E-04	372E-03	Dlgap1	73694	Hypo
chr10	88959371	88961391	Distal Interger	-0553	112E-04	374E-03	Crhr1	-78812	Hypo
chr17	83781438	83783876	Distal Interger	-0480	112E-04	374E-03	Gpr158	-50693	Hypo
chr1	186962780	186967053	Distal Interger	-0467	113E-04	374E-03	Cpxm2	-15560	Hypo
chr11	78608572	78610430	Promoter (<=1	-0591	113E-04	374E-03	Etv5	0	Hypo
chr1	67437916	67439995	Distal Interger	-0506	113E-04	375E-03	Smim17	22944	Hypo
chr7	90714077	90715589	Distal Interger	-0560	113E-04	375E-03	Mtss1	-86235	Hypo
chr1	24688575	24692028	Distal Interger	-0485	113E-04	375E-03	Trdn	-278162	Hypo
chr4	65651028	65651514	Intron (NM_1	-1093	113E-04	375E-03	Dgki	221219	Hypo
chr11	57567669	57569669	Intron (NM_0(	-0452	114E-04	377E-03	Zbtb20	221545	Hypo
chr1	46117231	46119605	Intron (NM_0(	-0569	114E-04	377E-03	Zdhhc14	48104	Hypo
chr6	83163021	83165738	Distal Interger	-0467	114E-04	377E-03	Fkbp3	-37521	Hypo
chr9	68896513	68897552	Distal Interger	-0562	114E-04	377E-03	Cps1	282360	Hypo
chr7	69541473	69541745	Distal Interger	-1292	114E-04	377E-03	Klf10	-67747	Hypo
chr17	71175568	71183867	Distal Interger	-0380	115E-04	378E-03	Celf2	-225743	Hypo
chr12	23299745	23300342	Distal Interger	-0953	115E-04	379E-03	Mir6322	479777	Hypo
chr6	61409975	61412428	Intron (NM_0	-0522	115E-04	380E-03	Nrcam	7136	Hypo
chr8	71039827	71040396	Intron (NM_1	-0974	115E-04	380E-03	Ccnb2	60398	Hypo
chr2	210598425	210604505	Intron (NM_0(	-0381	116E-04	382E-03	Bcar3	73165	Hypo

chr15	95933305	95939294	Exon (NM_00:	-0413	117E-04	384E-03	Cldn10	70520	Hypo
chr18	76784399	76785832	Distal Interger	-0576	117E-04	384E-03	Zfp516	441999	Hypo
chr8	47269247	47271222	Distal Interger	-0574	117E-04	385E-03	Cadm1	-576614	Hypo
chr2	98715034	98715762	Distal Interger	-0833	118E-04	387E-03	Ythdf3	364373	Hypo
chr7	29783315	29785930	Distal Interger	-0460	118E-04	387E-03	Cradd	166977	Hypo
chr2	195167123	195170368	Distal Interger	-0379	118E-04	388E-03	Slc6a17	-11426	Hypo
chr8	84510026	84513543	Intron (NM_0(	-0438	119E-04	389E-03	Sh3bgrl2	48905	Hypo
chr3	115942503	115946521	Intron (NM_0:	-0510	119E-04	389E-03	Mertk	3152	Hypo
chr8	40691077	40711126	Exon (NM_00:	-0315	119E-04	389E-03	Scn3b	60622	Hypo
chr10	87515656	87516978	Distal Interger	-0653	120E-04	391E-03	Fzd2	-44888	Hypo
chr17	56543428	56544389	Distal Interger	-0697	120E-04	392E-03	Wac	620305	Hypo
chr1	13962988	13964820	Distal Interger	-0586	120E-04	393E-03	Olig3	-116439	Hypo
chr8	47200777	47203315	Distal Interger	-0567	120E-04	393E-03	LOC500990	-590303	Hypo
chr4	96051589	96054536	Intron (NM_0!	-0422	121E-04	394E-03	Gng12	15343	Hypo
chr8	102803657	102806448	Intron (NM_0(	-0430	121E-04	394E-03	Ephb1	138391	Hypo
chr5	133699002	133701150	Intron (NM_0(	-0571	121E-04	395E-03	Hivep3	40950	Hypo
chr20	40812564	40813111	Distal Interger	-0908	121E-04	395E-03	Marcks	-121552	Hypo
chr14	71017370	71017815	Distal Interger	-1073	121E-04	395E-03	Hs3st1	-197204	Hypo
chr11	69169646	69171802	Distal Interger	-0471	122E-04	396E-03	Dlg1	-66843	Hypo
chr8	110181824	110189048	Exon (NM_00:	-0358	122E-04	396E-03	Cspg5	-31605	Hypo
chr2	29523408	29526683	Promoter (1-2	-0444	122E-04	396E-03	Arhgef28	1917	Hypo
chr10	75577627	75578326	Distal Interger	-0728	122E-04	397E-03	LOC10036342	69273	Hypo
chr2	224036501	224039917	Exon (NM_00:	-0458	123E-04	398E-03	Nfkb1	91914	Hypo
chr8	58176375	58177051	Distal Interger	-0871	123E-04	398E-03	Clk3	-10600	Hypo
chr3	88977544	88981707	3' UTR	-0375	123E-04	399E-03	Slc1a2	-23422	Hypo
chr8	27038711	27039891	Intron (NM_0!	-0593	123E-04	399E-03	Opcml	249043	Hypo
chr11	52156788	52158640	Distal Interger	-0604	123E-04	399E-03	Trat1	121038	Hypo
chr1	255531546	255533796	Distal Interger	-0569	123E-04	399E-03	Plekhs1	35912	Hypo
chr5	102736691	102737637	Distal Interger	-0768	123E-04	399E-03	Hacd4	177673	Hypo
chr16	29362269	29362723	Distal Interger	-0918	124E-04	399E-03	Mfap3l	68140	Hypo
chr13	94122176	94124190	Distal Interger	-0475	124E-04	401E-03	Capn2	76779	Hypo
chr10	78782724	78785260	Distal Interger	-0455	124E-04	401E-03	Utp18	53594	Hypo
chr8	105925829	105928993	Intron (NM_0(	-0419	124E-04	401E-03	Aste1	-97577	Hypo

chr17	23854700	23857500	Distal Interger	-0494	125E-04	401E-03	Gcnt2	-15530	Hypo
chr2	91649390	91652197	Distal Interger	-0456	125E-04	401E-03	Pmp2	38056	Hypo
chr10	97956725	97957459	Distal Interger	-0744	125E-04	401E-03	Sox9	150240	Hypo
chr3	88993842	88997556	Distal Interger	-0407	125E-04	401E-03	Slc1a2	-7573	Hypo
chr12	8435399	8436667	Distal Interger	-0654	125E-04	402E-03	Gpr12	-86818	Hypo
chr1	154562767	154564237	Intron (NR_13	-0641	126E-04	403E-03	LOC10091207	7134	Hypo
chr12	42801980	42805379	Promoter (2-3	-0520	126E-04	403E-03	Selplg	-2499	Hypo
chr1	91206315	91207461	Distal Interger	-0749	126E-04	404E-03	LOC365238	-122925	Hypo
chr2	86375939	86379444	Distal Interger	-0460	126E-04	404E-03		-221084	Hypo
chr1	50869146	50871976	Distal Interger	-0575	126E-04	404E-03	Pabpc6	-173288	Hypo
chr2	165856415	165857538	Distal Interger	-0595	126E-04	404E-03	Mir2985	98960	Hypo
chr1	117704894	117705409	Distal Interger	-0976	127E-04	406E-03	Trpm1	-13487	Hypo
chr1	118915429	118919094	Promoter (<=1	-0430	127E-04	406E-03	Tjp1	-560	Hypo
chr16	46733899	46734610	Distal Interger	-0843	127E-04	407E-03	Tlr3	-87779	Hypo
chr6	40058774	40060372	Promoter (2-3	-0613	128E-04	407E-03	Pdia6	-2608	Hypo
chr16	67381606	67382161	Exon (NM_001	-0980	128E-04	407E-03	Adam18	29190	Hypo
chr2	233086033	233087753	Distal Interger	-0548	128E-04	407E-03	Lmo4	193128	Hypo
chr16	31390910	31391431	Intron (NM_00	-1024	128E-04	407E-03	Galntl6	196632	Hypo
chr5	22392916	22393661	Distal Interger	-0840	128E-04	407E-03	Clvs1	-14723	Hypo
chr3	136384992	136386539	Distal Interger	-0592	128E-04	407E-03	Cst3	-44196	Hypo
chr2	157993731	157995515	Distal Interger	-0563	128E-04	407E-03	Slitrk3	-297176	Hypo
chrX	127125017	127126415	Intron (NM_00	-0713	128E-04	408E-03	Ocrl	35481	Hypo
chr16	78535550	78537121	Distal Interger	-0619	129E-04	408E-03	Irs2	47301	Hypo
chr7	85530078	85531069	Distal Interger	-0653	129E-04	408E-03	Tnfrsf11b	63457	Hypo
chr15	42255391	42255970	Distal Interger	-0880	129E-04	409E-03	Nefl	-45950	Hypo
chr17	70658834	70660593	Distal Interger	-0578	130E-04	411E-03	Celf2	-749017	Hypo
chr2	139631991	139640305	Promoter (<=1	-0329	130E-04	412E-03	Dclk1	0	Hypo
chr8	71834143	71836783	Promoter (<=1	-0435	130E-04	412E-03	Aqp9	612	Hypo
chr9	111052060	111053550	Distal Interger	-0586	131E-04	415E-03	Lpin2	-30195	Hypo
chr6	83442123	83444281	Distal Interger	-0581	131E-04	415E-03	Mis18bp1	-210740	Hypo
chr13	87029985	87031281	Intron (NM_0	-0642	132E-04	416E-03	Grem2	-158476	Hypo
chr3	157158113	157159349	Distal Interger	-0743	132E-04	416E-03	Nfatc2	154768	Hypo
chr8	51162827	51165803	3' UTR	-0448	132E-04	416E-03	Ppp2r1b	-30386	Hypo

chrX	27961280	27962125	Distal Interger	-0906	132E-04	417E-03	Tceanc	3621	Hypo
chr17	61868998	61871188	Intron (NM_1:	-0543	132E-04	417E-03	Idi1	-231641	Hypo
chr2	230609818	230610412	Distal Interger	-0914	133E-04	418E-03	Bmpr1b	-35219	Hypo
chr10	3410738	3412167	Distal Interger	-0615	133E-04	420E-03	Cpped1	-289355	Hypo
chr2	43940602	43941263	Intron (NM_0(	-0734	135E-04	425E-03	Ankrd55	26092	Hypo
chr9	86869814	86871999	Intron (NM_0(	-0511	135E-04	425E-03	Armc9	66931	Hypo
chr10	56250633	56252594	Distal Interger	-0527	136E-04	426E-03	Wscd1	-143280	Hypo
chr16	57690967	57691926	Distal Interger	-0742	136E-04	427E-03	Dusp4	-300924	Hypo
chrX	4100326	4101030	Distal Interger	-0925	136E-04	427E-03	Dusp21	387847	Hypo
chr16	57816766	57818814	Distal Interger	-0491	136E-04	427E-03	Saraf	216667	Hypo
chr3	157941500	157943992	Distal Interger	-0584	137E-04	429E-03	Zfp64	-245726	Hypo
chr15	42256332	42256806	Distal Interger	-0948	137E-04	430E-03	Nefl	-45114	Hypo
chr12	13616080	13616649	Distal Interger	-0785	137E-04	430E-03	Gna12	-189049	Hypo
chr20	20690409	20698504	Distal Interger	-0374	137E-04	430E-03	Zfp365	24169	Hypo
chr3	111373101	111373618	Distal Interger	-0814	138E-04	430E-03	Sema6d	-510254	Hypo
chr1	15577758	15580008	Distal Interger	-0427	138E-04	430E-03	Pde7b	-84858	Hypo
chr4	134162113	134162787	Distal Interger	-0763	138E-04	430E-03	Pdzrn3	-165154	Hypo
chr9	29127234	29127539	Distal Interger	-1110	138E-04	431E-03	Adgrb3	-978684	Hypo
chr7	76055976	76059264	Distal Interger	-0419	139E-04	432E-03	Smg5l1	138414	Hypo
chr1	169136742	169137283	Distal Interger	-1001	139E-04	433E-03	Insc	143710	Hypo
chr7	68661228	68663011	Distal Interger	-0538	140E-04	434E-03	Ncald	-32467	Hypo
chr12	795895	796320	Distal Interger	-0941	140E-04	434E-03	Stard13	-61291	Hypo
chr7	90698350	90700197	Distal Interger	-0603	140E-04	434E-03	Mtss1	-70508	Hypo
chr19	15627676	15632866	Intron (NM_0(	-0387	140E-04	434E-03	Fto	59190	Hypo
chr2	61450966	61452289	Intron (NM_0:	-0623	140E-04	434E-03	Golph3	102936	Hypo
chr10	60018177	60025447	Promoter (<=:	-0421	140E-04	436E-03	Hic1	0	Hypo
chr15	14694125	14694389	Intron (NM_0:	-1133	141E-04	436E-03	Fhit	726243	Hypo
chr7	85206696	85209535	Distal Interger	-0495	141E-04	438E-03	Samd12	-142639	Hypo
chr1	256468031	256468486	Distal Interger	-0945	141E-04	438E-03	Mir6316	-32730	Hypo
chr9	12678723	12679553	Distal Interger	-0723	142E-04	439E-03	Trem2	-24553	Hypo
chr3	28357814	28359618	Intron (NM_0(	-0658	142E-04	439E-03	Arhgap15	368181	Hypo
chr5	60647709	60648837	Distal Interger	-0830	142E-04	439E-03	Foxe1	17682	Hypo
chr11	78531901	78533490	Intron (NM_0	-0488	142E-04	440E-03	Etv5	-75220	Hypo

chr6	41717626	41719652	Distal Interger	-0577	143E-04	441E-03	Id2	22741	Hypo
chr19	11854037	11856240	Intron (NM_0(	-0478	143E-04	441E-03	Large1	192690	Hypo
chr9	41714396	41715118	Intron (NM_0(	-0745	143E-04	442E-03	Tbc1d8	46291	Hypo
chr18	67739799	67742365	Distal Interger	-0460	144E-04	442E-03	Ska1	62672	Hypo
chr16	4800727	4803398	Intron (NM_1	-0518	144E-04	442E-03	Cacna2d3	108953	Hypo
chr9	81190839	81193184	Promoter (2-3	-0547	144E-04	444E-03	Serpine2	-2023	Hypo
chr4	148232369	148234367	Intron (NM_0	-0472	145E-04	445E-03	Syn2	46099	Hypo
chr1	22891603	22893039	Distal Interger	-0633	145E-04	445E-03	Slc2a12	-33528	Hypo
chr5	61061726	61065661	Intron (NM_0:	-0378	145E-04	445E-03	Tbc1d2	-130374	Hypo
chr2	241509116	241515187	Intron (NM_0(	-0390	145E-04	446E-03	Ak5	66271	Hypo
chr15	18761566	18764355	Distal Interger	-0434	146E-04	446E-03	Fermt2	-9788	Hypo
chr19	48751728	48753761	Distal Interger	-0574	146E-04	447E-03	Cox4i1	30048	Hypo
chr7	85535988	85536450	Distal Interger	-0845	146E-04	447E-03	Tnfrsf11b	58076	Hypo
chr17	51908748	51914861	Distal Interger	-0367	146E-04	448E-03	Zeb1	200353	Hypo
chr5	25513937	25514545	Distal Interger	-0941	146E-04	448E-03	Pdp1	-58890	Hypo
chr10	15900401	15903543	Distal Interger	-0460	147E-04	449E-03	Bod1	-89911	Hypo
chr1	226092440	226093934	Distal Interger	-0666	147E-04	449E-03	Rfx3	-383598	Hypo
chr12	32665749	32668123	Exon (NM_00	-0500	147E-04	449E-03	Ccdc62	-5904	Hypo
chr10	101132128	101134557	Promoter (<=	-0525	147E-04	449E-03	Smim5	0	Hypo
chr15	27630774	27634435	Distal Interger	-0469	147E-04	449E-03	Dad1	62649	Hypo
chr1	207491085	207495988	Promoter (<=	-0385	148E-04	450E-03	Slc15a3	0	Hypo
chr2	215832773	215834273	Distal Interger	-0588	148E-04	450E-03	Larp7	178536	Hypo
chr3	130947698	130949799	Intron (NM_0	-0509	148E-04	450E-03	Pcsk2	67276	Hypo
chr11	81974415	81974951	Distal Interger	-0932	148E-04	450E-03	Hira	158261	Hypo
chr10	4102471	4105409	Intron (NM_0(	-0433	148E-04	450E-03	Snx29	193246	Hypo
chr1	254997186	254998411	Distal Interger	-0713	148E-04	450E-03	Tcf7l2	211095	Hypo
chr15	79726797	79728021	Distal Interger	-0680	148E-04	450E-03	Acod1	-143806	Hypo
chr1	176806448	176807400	Distal Interger	-0719	148E-04	450E-03	Prkcb	-25285	Hypo
chr10	96485153	96490212	Distal Interger	-0441	148E-04	450E-03	Kcnj2	418878	Hypo
chr3	88505885	88508037	Distal Interger	-0669	148E-04	450E-03	Trim44	220600	Hypo
chr7	95813019	95819965	Exon (NM_00	-0392	149E-04	450E-03	Cyrib	-52431	Hypo
chr7	30353046	30354458	Distal Interger	-0519	149E-04	451E-03	Nudt4	-148719	Hypo
chr18	71889696	71891914	Intron (NM_0	-0548	150E-04	453E-03	Slc14a2	147548	Hypo

chr18	48579748	48581972	Distal Interger	-0553	150E-04	454E-03	Zfp608	-169283	Hypo
chr5	140440951	140442307	Distal Interger	-0666	150E-04	454E-03	LOC682102	-115136	Hypo
chr11	68918580	68920861	Intron (NM_0	-0537	151E-04	454E-03	Meltf	-12280	Hypo
chr7	92942278	92942996	Distal Interger	-0755	151E-04	455E-03	A1bg	-444181	Hypo
chr1	167544970	167546017	Distal Interger	-0770	151E-04	455E-03	Pth	-33440	Hypo
chr8	41384278	41387802	Distal Interger	-0442	151E-04	455E-03	Crtam	-6935	Hypo
chr14	84724142	84724458	Distal Interger	-1012	152E-04	456E-03	Abca13	850083	Hypo
chr14	61359417	61361498	Distal Interger	-0556	152E-04	456E-03	Kcnip4	-19201	Hypo
chr2	30085436	30087321	Exon (NM_001	-0550	152E-04	456E-03	Tmem171	-40387	Hypo
chr4	26201023	26201655	Distal Interger	-0876	152E-04	456E-03	Steap4	-101164	Hypo
chr10	30785739	30787576	Exon (NM_001	-0607	152E-04	456E-03	Itk	27109	Hypo
chr16	46645014	46645712	Distal Interger	-0794	152E-04	456E-03	Sorbs2	-18566	Hypo
chr13	66274646	66277178	Distal Interger	-0438	153E-04	460E-03	Glul	249005	Hypo
chr7	8183526	8184606	Promoter (<=1	-0711	154E-04	460E-03	Gna15	-380	Hypo
chr18	38596350	38597365	Distal Interger	-0639	154E-04	460E-03	Trim36	315494	Hypo
chr2	8485585	8487178	Distal Interger	-0628	154E-04	461E-03	Nr2f1	-435248	Hypo
chr1	170048300	170051544	Intron (NM_0(	-0443	154E-04	462E-03	Sox6	179478	Hypo
chr3	89571167	89573717	Distal Interger	-0613	155E-04	462E-03	Ehf	106280	Hypo
chr1	34320913	34321998	Distal Interger	-0648	155E-04	464E-03	Adcy2	-53913	Hypo
chr20	8278093	8284270	3' UTR	-0380	155E-04	464E-03	Zfand3	194686	Hypo
chr8	61631705	61633174	Distal Interger	-0590	156E-04	464E-03	LOC691000	125040	Hypo
chr1	73681400	73683509	Promoter (<=1	-0399	157E-04	467E-03	Mzf1	0	Hypo
chr9	65198679	65202315	Distal Interger	-0414	157E-04	467E-03	Fastkd2	29212	Hypo
chr20	9780435	9781857	Promoter (1-2	-0624	157E-04	467E-03	Cryaa	-1748	Hypo
chr11	78721411	78723049	Distal Interger	-0635	157E-04	467E-03	Tra2b	-65871	Hypo
chr3	123279194	123280428	Exon (NM_024	-0572	157E-04	467E-03	Lamp5	-92034	Hypo
chr5	121065423	121066434	Distal Interger	-0757	157E-04	467E-03	Usp24	-14328	Hypo
chr12	40064423	40070315	Intron (NM_0(	-0353	157E-04	467E-03	Srrm4	65448	Hypo
chr4	50477870	50479175	Promoter (<=1	-0422	158E-04	468E-03	Ing3	0	Hypo
chr18	14993100	14993926	Distal Interger	-0748	158E-04	468E-03	Mapre2	-77445	Hypo
chr5	133664273	133669439	Intron (NM_0(	-0412	158E-04	468E-03	Hivep3	6221	Hypo
chr1	148852695	148853465	Distal Interger	-0741	158E-04	468E-03	Fam181b	1686267	Hypo
chr10	92370750	92373807	Exon (NM_001	-0442	158E-04	468E-03	RGD1359290	4914	Hypo

chr16	74340221	74343133	Distal Interger	-0442	158E-04	468E-03	Myom2	247007	Hypo
chr2	33824563	33826363	Distal Interger	-0561	159E-04	470E-03	Cd180	-29628	Hypo
chr11	61569291	61570606	Distal Interger	-0572	159E-04	471E-03	Igsf11	297742	Hypo
chr1	212475737	212476800	Distal Interger	-0841	160E-04	472E-03	Tle4	-677875	Hypo
chr2	57817710	57818358	Intron (NM_0	-0970	160E-04	473E-03	Slc1a3	12226	Hypo
chr6	19006570	19009532	Distal Interger	-0458	161E-04	473E-03	Fam98a	-864550	Hypo
chr9	98135236	98136798	Intron (NM_0	-0555	161E-04	474E-03	Pam	12204	Hypo
chr18	48989767	48992645	Distal Interger	-0540	161E-04	474E-03	Zfp608	-579302	Hypo
chr16	75911667	75916342	Intron (NM_0:	-0371	162E-04	477E-03	Rasa3	56307	Hypo
chr18	70761142	70771804	Intron (NM_2	-0340	163E-04	478E-03	St8sia5	24477	Hypo
chr1	2535571	2536724	Distal Interger	-0742	163E-04	479E-03	Tab2	-110815	Hypo
chr8	40792109	40792376	Intron (NM_0(	-1290	164E-04	480E-03	Gramd1b	29415	Hypo
chr2	86749840	86758600	Promoter (<=:	-0346	164E-04	480E-03	Car2	0	Hypo
chr10	37588270	37590089	Promoter (<=:	-0384	164E-04	480E-03	Uqcrq	0	Hypo
chr14	71697367	71698892	Distal Interger	-0647	164E-04	480E-03	Hs3st1	482348	Hypo
chr20	17928010	17929150	Distal Interger	-0617	165E-04	482E-03	Phyhipl	-102655	Hypo
chr17	70591455	70595014	Distal Interger	-0421	165E-04	482E-03	Celf2	-814596	Hypo
chr17	1949704	1953500	Intron (NM_0(	-0443	165E-04	482E-03	Aopep	123676	Hypo
chr17	17430342	17432786	3' UTR	-0477	166E-04	484E-03	Rnf144b	129974	Hypo
chr2	14033525	14046086	Distal Interger	-0323	166E-04	484E-03	Mir9-2	-151638	Hypo
chr19	48796332	48797051	Promoter (2-3	-0778	167E-04	485E-03	Irf8	2145	Hypo
chr13	21382422	21383589	Intron (NM_0(	-0727	167E-04	485E-03	Rnf152	19511	Hypo
chr15	92829354	92829851	Intron (NM_0(	-0889	168E-04	487E-03	Gpc5	589808	Hypo
chr17	67198889	67201975	Distal Interger	-0435	168E-04	487E-03	Pfkfb3	163794	Hypo
chr7	81744677	81745140	Distal Interger	-0978	168E-04	487E-03	Trps1	396214	Hypo
chr8	23698693	23704371	Distal Interger	-0414	168E-04	488E-03	Septin7	-15400	Hypo
chr6	37644659	37645158	Distal Interger	-0846	169E-04	490E-03	Lratd1	-722516	Hypo
chr9	81085052	81086946	Intron (NM_0(	-0547	170E-04	492E-03	Wdfy1	5847	Hypo
chr7	129277708	129279112	Distal Interger	-0495	170E-04	492E-03	Asb8	-8772	Hypo
chr2	86381286	86385486	Distal Interger	-0437	170E-04	493E-03		-215042	Hypo
chr19	38656942	38659648	Intron (NM_1	-0463	171E-04	493E-03	Mtss2	-33566	Hypo
chr16	25029334	25033549	Promoter (<=:	-0333	171E-04	493E-03	Cpe	0	Hypo
chr16	63874459	63875403	Distal Interger	-0738	171E-04	494E-03	Smok	-582973	Hypo

chr19	2393063	2393743	Distal Interger	-0739	171E-04	495E-03	Cdh11	244616	Hypo
chr15	82767320	82771773	Distal Interger	-0392	172E-04	495E-03	Spry2	-71996	Hypo
chr6	110147336	110147995	Distal Interger	-0754	172E-04	495E-03	Tshr	-193590	Hypo
chr15	68121231	68122735	Distal Interger	-0594	172E-04	495E-03	Pcdh9	2112930	Hypo
chr7	124841889	124843993	Distal Interger	-0471	173E-04	498E-03	Prickle1	-183776	Hypo
chr17	1571641	1574749	Exon (NM_05:	-0481	174E-04	500E-03	Ptch1	21069	Hypo
chr5	38671950	38673781	Distal Interger	-0439	174E-04	500E-03	Gpr63	-7631	Hypo
chr17	19932062	19943282	Distal Interger	-0366	174E-04	500E-03	Dtnbp1	246844	Hypo
chr8	42498625	42504888	Promoter (<=:	-0373	174E-04	500E-03	Sorl1	0	Hypo
chr5	116808511	116809050	Distal Interger	-0839	174E-04	501E-03	Pde4b	-184386	Hypo
chr3	13056323	13056882	Promoter (2-3	-0818	174E-04	501E-03	Trub2	2949	Hypo
chr5	144818393	144826167	Distal Interger	-0358	174E-04	501E-03	Eya3	-27121	Hypo
chr8	107923606	107931652	3' UTR	-0346	175E-04	501E-03	Dock3	-20092	Hypo
chr10	53333007	53334020	Distal Interger	-0660	175E-04	501E-03	Ccdc42	43051	Hypo
chr3	15296164	15297713	Exon (NM_00:	-0582	175E-04	501E-03	Nup214	41045	Hypo
chr7	65749822	65751679	Distal Interger	-0532	175E-04	501E-03	Pop1	44435	Hypo
chr7	21308369	21309983	Intron (NM_0(	-0611	175E-04	502E-03	Nt5dc3	117815	Hypo
chr16	78530666	78532058	Distal Interger	-0583	175E-04	502E-03	Irs2	42417	Hypo
chr1	154272174	154274573	Distal Interger	-0478	176E-04	502E-03	Chrdl2	-68628	Hypo
chr8	95770410	95772438	Intron (NM_0(	-0554	176E-04	502E-03	Paqr9	-341811	Hypo
chr2	170844752	170848633	Distal Interger	-0403	176E-04	502E-03	Fhip1a	242849	Hypo
chr10	100469503	100474283	Intron (NM_0(	-0424	176E-04	502E-03	Grin2c	31873	Hypo
chr19	11317773	11319712	Distal Interger	-0592	176E-04	502E-03	Gnao1	-125280	Hypo
chr20	41879509	41881144	Distal Interger	-0653	176E-04	502E-03	Lama4	-511124	Hypo
chr17	1424168	1424662	Distal Interger	-0832	176E-04	503E-03	Ptch1	-125910	Hypo
chr1	231252931	231253467	Intron (NM_0(	-0953	176E-04	503E-03	Rps4x-ps1	42654	Hypo
chr7	127707599	127709473	Distal Interger	-0513	176E-04	503E-03	Scaf11	-83684	Hypo
chr13	81166784	81167077	Distal Interger	-1077	176E-04	503E-03	Nuf2	555688	Hypo
chr1	126964054	126968343	Distal Interger	-0394	177E-04	503E-03	Rgma	-160591	Hypo
chr9	44915522	44916678	Exon (NR_132	-0579	177E-04	503E-03	Pantr1	26953	Hypo
chr11	79182232	79183259	Intron (NM_0(	-0541	177E-04	504E-03	Map3k13	51922	Hypo
chr2	195438978	195440281	Distal Interger	-0633	178E-04	505E-03	Csf1	-42687	Hypo
chr1	168512395	168514365	Distal Interger	-0469	178E-04	505E-03	Psma1	-58955	Hypo

chr1	249310692	249314151	Intron (NM_0(	-0422	178E-04	506E-03	Sorcs1	280128	Hypo
chr7	30815722	30817500	Distal Interger	-0568	178E-04	506E-03	Eea1	210729	Hypo
chr9	62580174	62580592	Distal Interger	-1065	179E-04	507E-03	Icos	196342	Hypo
chr19	48790629	48793137	Promoter (1-2	-0591	179E-04	507E-03	Irf8	-1050	Hypo
chr1	174417489	174419522	Distal Interger	-0503	179E-04	507E-03	Ldaf1	-65058	Hypo
chr18	17590913	17591893	Distal Interger	-0667	179E-04	507E-03	Celf4	-526127	Hypo
chr1	212937599	212939597	Distal Interger	-0551	179E-04	508E-03	Psat1	278967	Hypo
chr13	39445090	39447268	Distal Interger	-0463	180E-04	508E-03	Mir3473	-51978	Hypo
chr18	3962972	3967748	Exon (NM_17	-0390	180E-04	509E-03	Cabyr	70840	Hypo
chr16	67098016	67099902	Promoter (2-3	-0592	181E-04	510E-03	Adam32	-2533	Hypo
chr19	5681625	5683111	Intron (NM_0	-0558	181E-04	512E-03	Cdh8	187451	Hypo
chr8	51820375	51824354	Distal Interger	-0397	181E-04	512E-03	Arhgap20	-250118	Hypo
chr6	94217419	94218138	Intron (NM_0(	-0775	182E-04	512E-03	Gphb5	-83849	Hypo
chr18	15134203	15136616	Intron (NM_0(	-0447	182E-04	512E-03	Mapre2	62832	Hypo
chr10	100463779	100467329	Intron (NM_0(	-0452	182E-04	513E-03	Tmem104	36472	Hypo
chr8	110258572	110261775	Distal Interger	-0414	184E-04	516E-03	Elp6	-18284	Hypo
chr9	37541000	37542324	Distal Interger	-0705	184E-04	517E-03	Plekhb2	491420	Hypo
chr6	39092622	39093297	Distal Interger	-0854	184E-04	518E-03	Lpin1	286879	Hypo
chr7	57882204	57883481	Distal Interger	-0669	185E-04	519E-03	Rxylt1	-99587	Hypo
chr1	209149601	209152457	Distal Interger	-0408	185E-04	519E-03	Or5bb12	-28059	Hypo
chr2	230629622	230632991	Distal Interger	-0527	186E-04	521E-03	Bmpr1b	-55023	Hypo
chr19	41530953	41534376	Distal Interger	-0452	187E-04	522E-03	Mon1b	-83237	Hypo
chr2	219191260	219192613	Distal Interger	-0616	187E-04	523E-03	Ostc	73463	Hypo
chr7	96095013	96097105	Distal Interger	-0533	187E-04	523E-03	Asap1	-36644	Hypo
chr2	32305419	32307668	Distal Interger	-0475	187E-04	524E-03	Slc30a5	-329575	Hypo
chr13	77796714	77798095	Exon (NM_02	-0613	187E-04	524E-03	Adcy10	45402	Hypo
chr13	103210684	103211914	Distal Interger	-0664	188E-04	526E-03	Ints7	55561	Hypo
chr17	19825810	19827421	Distal Interger	-0626	189E-04	528E-03	Dtnbp1	140592	Hypo
chr9	3448268	3451835	Intron (NM_0(	-0422	189E-04	528E-03	Plcl2	154900	Hypo
chr12	42789079	42791352	Distal Interger	-0554	189E-04	528E-03	Selplg	-16526	Hypo
chr19	2392131	2392800	Distal Interger	-0793	190E-04	530E-03	Cdh11	243684	Hypo
chrX	28198422	28207055	Distal Interger	-0335	191E-04	530E-03	Gemin8	71586	Hypo
chr15	94906901	94907722	Distal Interger	-0739	191E-04	530E-03	Tgds	287855	Hypo

chr9	110461530	110466757	Intron (NM_0	-0378	191E-04	530E-03	Dlgap1	43707	Hypo
chr19	40084969	40086061	Distal Interger	-0605	191E-04	532E-03	Terf2ip	108004	Hypo
chr2	24717712	24718997	Distal Interger	-0635	191E-04	532E-03	Bhmt	160452	Hypo
chr2	12154953	12160940	Distal Interger	-0378	192E-04	532E-03	Cetn3	65536	Hypo
chr2	134979298	134980033	Distal Interger	-0734	192E-04	533E-03	Noct	-291156	Hypo
chrX	116998545	116999398	Intron (NM_1	-0795	192E-04	534E-03	Zbtb33	35106	Hypo
chr13	39416174	39420041	Distal Interger	-0413	193E-04	534E-03	Mir3473	-79205	Hypo
chr6	15112484	15116848	Distal Interger	-0385	193E-04	535E-03	Atl2	-22223	Hypo
chr1	105496377	105497446	Distal Interger	-0696	193E-04	536E-03	Tubgcp5	-1139080	Hypo
chr4	45377193	45379203	Intron (NM_0	-0594	194E-04	537E-03	Tes	11889	Hypo
chr19	20851281	20852571	Distal Interger	-0645	194E-04	537E-03	Abcc12	301876	Hypo
chr8	107931743	107939085	Exon (NM_00	-0356	194E-04	538E-03	Mapkapk3	24483	Hypo
chr2	232262216	232263312	Distal Interger	-0730	195E-04	540E-03	Pkn2	-363263	Hypo
chr1	119896464	119896918	Intron (NM_0	-0901	195E-04	540E-03	Lrrk1	75967	Hypo
chr4	153998472	154002785	Promoter (<=	-0406	196E-04	541E-03	Slc25a18	0	Hypo
chr5	125767220	125769792	Intron (NM_0	-0477	196E-04	541E-03	Agbl4	512257	Hypo
chr2	235497720	235499477	Intron (NM_0	-0522	196E-04	541E-03	Uox	10853	Hypo
chr20	44627098	44627563	Intron (NM_0	-0977	196E-04	541E-03	Fig4	96281	Hypo
chr7	61314559	61315160	Distal Interger	-0817	196E-04	541E-03	Slc16a7	-159553	Hypo
chr6	138492565	138495981	Distal Interger	-0454	196E-04	541E-03	Rapgef5	-105793	Hypo
chr16	1479455	1481152	Distal Interger	-0600	196E-04	541E-03	Plac9	-5233	Hypo
chr1	166318975	166320547	Distal Interger	-0591	196E-04	541E-03	Dkk3	-38385	Hypo
chr16	57795630	57798131	Distal Interger	-0481	197E-04	542E-03	Saraf	237350	Hypo
chr7	132117461	132118465	Exon (NM_01	-0642	197E-04	542E-03	Acvrl1	-127046	Hypo
chr5	154994845	154997787	Distal Interger	-0505	198E-04	545E-03	Kazn	-215936	Hypo
chr4	119253264	119253847	Distal Interger	-0807	199E-04	545E-03	Anxa4	-12111	Hypo
chr2	41709459	41711125	Intron (NM_1	-0549	199E-04	545E-03	Rab3c	131448	Hypo
chr3	146011701	146014873	Promoter (<=	-0402	199E-04	545E-03	Ghrh	0	Hypo
chr17	12280442	12285670	Promoter (<=	-0369	199E-04	545E-03	Nfil3	0	Hypo
chr3	42794875	42796366	Distal Interger	-0610	199E-04	546E-03	Cytip	-68882	Hypo
chr7	46452908	46456128	Distal Interger	-0390	200E-04	547E-03	Zdhhc17	-19273	Hypo
chr18	14747710	14748209	Distal Interger	-0927	200E-04	547E-03	Mapre2	-323162	Hypo
chr12	27559922	27563197	Distal Interger	-0479	201E-04	550E-03	Ran	115051	Hypo

chr6	102452498	102453688	Intron (NM_0	-0649	201E-04	550E-03	Rgs6	187203	Hypo
chr1	121685355	121686853	Intron (NM_0	-0635	202E-04	551E-03	Igf1r	134614	Hypo
chr4	179179599	179181957	Intron (NM_0	-0431	202E-04	552E-03	Itpr2	222207	Hypo
chr11	7717802	7719210	Distal Interger	-0634	203E-04	554E-03	Gbe1	-1015652	Hypo
chr1	93952349	93954929	Distal Interger	-0531	203E-04	554E-03	Zfp819	-83319	Hypo
chr1	8839358	8839871	Distal Interger	-0855	203E-04	554E-03	Vta1	240729	Hypo
chr9	6900467	6901055	Promoter (<=1	-0662	203E-04	555E-03	Sult1c2a	56	Hypo
chr12	43041008	43046354	Distal Interger	-0368	204E-04	555E-03	Cmklr1	21008	Hypo
chr15	82276346	82277783	Distal Interger	-0562	205E-04	557E-03	Ndfip2	243980	Hypo
chr1	143944378	143945291	Distal Interger	-0689	205E-04	558E-03	Eed	-49420	Hypo
chr8	101648396	101649219	Distal Interger	-0714	205E-04	558E-03	Pccb	-7183	Hypo
chr11	78607108	78608425	Promoter (<=1	-0517	205E-04	558E-03	Etv5	-285	Hypo
chr3	100181329	100182412	Distal Interger	-0652	206E-04	559E-03	Grem1	341589	Hypo
chr1	211544912	211545575	Distal Interger	-0787	206E-04	560E-03	Tle4	252287	Hypo
chr18	43661428	43662043	Distal Interger	-0807	207E-04	561E-03	Fam170a	136981	Hypo
chr7	18952624	18953310	Distal Interger	-0811	207E-04	562E-03	Ric8b	-110256	Hypo
chr18	22771757	22775468	Intron (NM_0	-0404	208E-04	563E-03	Rit2	146180	Hypo
chr18	14746639	14747583	Distal Interger	-0721	208E-04	563E-03	Mapre2	-323788	Hypo
chr1	79371242	79372860	Promoter (<=1	-0465	208E-04	563E-03	Tomm40	-530	Hypo
chr8	78717234	78720435	Distal Interger	-0481	209E-04	564E-03	Gclc	87107	Hypo
chr8	59696012	59705684	Promoter (<=1	-0395	209E-04	564E-03	Adpgk	0	Hypo
chr3	65533237	65536489	Promoter (1-2	-0377	209E-04	565E-03	Dusp19	-1935	Hypo
chr17	70369945	70374179	Distal Interger	-0371	209E-04	565E-03	Celf2	-1035431	Hypo
chr5	19446662	19447218	Distal Interger	-0899	210E-04	566E-03	Sdcbp	-42783	Hypo
chr4	179050512	179057769	Intron (NM_0	-0369	210E-04	566E-03	Sspn	153365	Hypo
chr3	90033426	90035210	Intron (NM_1	-0557	210E-04	567E-03	Abtb2	78700	Hypo
chr13	45538061	45540861	Promoter (2-3	-0455	210E-04	567E-03	Btg2	-2419	Hypo
chr9	102999788	103001178	Intron (NM_0	-0608	210E-04	567E-03	Fbxl17	204518	Hypo
chr13	32609112	32609957	Promoter (1-2	-0747	211E-04	568E-03	Ccdc93	1494	Hypo
chr1	45873329	45877152	Distal Interger	-0394	211E-04	569E-03	Ldhal6b	122116	Hypo
chr18	4174094	4175015	Promoter (1-2	-0760	211E-04	569E-03	Rpl7a	-1184	Hypo
chr5	4829956	4830886	Distal Interger	-0842	211E-04	569E-03	Msc	278620	Hypo
chr7	82979009	82980700	Distal Interger	-0550	212E-04	570E-03	Eif3h	193736	Hypo

chr13	33518661	33519064	Distal Interger	-0886	213E-04	572E-03	Ddx18	-775500	Hypo
chr10	15957582	15958755	Distal Interger	-0660	213E-04	573E-03	Bod1	-34699	Hypo
chr9	57032604	57036089	Intron (NM_0	-0441	213E-04	573E-03	Plcl1	131031	Hypo
chr2	24419145	24419686	Intron (NM_0	-0847	214E-04	574E-03	Tent2	12383	Hypo
chr3	103326650	103327297	Distal Interger	-0789	214E-04	575E-03	Meis2	-383153	Hypo
chr9	83924899	83925947	Intron (NM_0	-0713	215E-04	576E-03	Col4a3	48895	Hypo
chr9	56552505	56555006	Promoter (<=	-0375	215E-04	576E-03	Coq10b	0	Hypo
chr2	222627313	222628760	Distal Interger	-0626	215E-04	576E-03	Cxxc4	-35847	Hypo
chr10	63595407	63597801	Distal Interger	-0391	215E-04	577E-03	Nlk	-5677	Hypo
chr19	20921100	20922942	Distal Interger	-0560	216E-04	577E-03	Phkb	287663	Hypo
chr1	241267871	241269060	Distal Interger	-0682	216E-04	578E-03	Crtac1	-34450	Hypo
chr3	161169694	161172009	Intron (NM_0	-0529	216E-04	578E-03	Cass4	6075	Hypo
chr12	42791764	42794182	Distal Interger	-0561	216E-04	578E-03	Selplg	-13696	Hypo
chr14	61262771	61264091	Distal Interger	-0619	217E-04	579E-03	Kcnip4	-116608	Hypo
chr7	28995015	28996429	Distal Interger	-0632	217E-04	579E-03	Tmcc3	-103746	Hypo
chr13	73564911	73565529	Distal Interger	-0879	217E-04	579E-03	Prdx6	-25616	Hypo
chr7	132115789	132117373	Intron (NM_0	-0484	217E-04	579E-03	Acvrl1	-128138	Hypo
chr3	150380043	150381224	Intron (NM_0	-0682	217E-04	580E-03	Ptprt	607439	Hypo
chr8	61505237	61506762	Promoter (<=	-0615	218E-04	581E-03	LOC691000	0	Hypo
chr3	78658415	78662021	Distal Interger	-0410	218E-04	581E-03	Chst1	98483	Hypo
chr18	75042642	75042918	Distal Interger	-1174	218E-04	582E-03	Sall3	-628814	Hypo
chr2	232792151	232792777	Distal Interger	-0817	219E-04	582E-03	Lmo4	488104	Hypo
chr10	84446065	84447591	Intron (NM_0	-0609	219E-04	582E-03	Krt23	3131	Hypo
chr1	255757320	255761869	Distal Interger	-0378	220E-04	584E-03	Adrb1	-10348	Hypo
chr14	14999987	15001395	Distal Interger	-0638	220E-04	585E-03	Septin11	-9288	Hypo
chr15	89953050	89953430	Distal Interger	-0990	220E-04	585E-03	Slitrk5	1101646	Hypo
chrX	8289659	8290388	Distal Interger	-0768	221E-04	585E-03	Cask	-609445	Hypo
chr2	16453103	16454259	Distal Interger	-0683	222E-04	588E-03	LOC252890	387771	Hypo
chr10	38842708	38844855	Intron (NM_0	-0505	222E-04	588E-03	Cdc42se2	60201	Hypo
chr13	87564957	87568522	Exon (NM_02	-0407	222E-04	588E-03	Kmo	7614	Hypo
chr1	234536556	234537294	Distal Interger	-0779	222E-04	588E-03	Tnks2	-30896	Hypo
chr2	170355306	170359516	Distal Interger	-0403	222E-04	588E-03	Dear	-55124	Hypo
chr2	56075009	56075803	Distal Interger	-0683	222E-04	588E-03	Osmr	-125312	Hypo

chr2	23480289	23480574	Intron (NM_0(	-1069	223E-04	588E-03	Msh3	105203	Hypo
chr13	102783695	102784398	Distal Interger	-0828	223E-04	589E-03	Atf3	-19070	Hypo
chr16	43908290	43910432	Exon (NM_00:	-0502	223E-04	589E-03	Dctd	187983	Hypo
chr2	76393982	76403107	Intron (NM_0(	-0367	223E-04	589E-03	Retreg1	58373	Hypo
chr19	9862543	9864302	Distal Interger	-0505	223E-04	590E-03	Kifc3	-37485	Hypo
chr11	25625823	25627807	Distal Interger	-0536	223E-04	590E-03	Adamts5	-579021	Hypo
chr19	17805237	17809266	Distal Interger	-0469	225E-04	592E-03	Sall1	-198237	Hypo
chr1	166320686	166327624	Distal Interger	-0337	225E-04	592E-03	Dkk3	-40096	Hypo
chr1	167280359	167281688	Distal Interger	-0605	225E-04	593E-03	Bmal1	-50132	Hypo
chr16	22272358	22275838	Distal Interger	-0485	225E-04	593E-03	Nat1	-33842	Hypo
chr1	175538482	175540469	Distal Interger	-0549	225E-04	593E-03	Cdr2	-10736	Hypo
chr3	78757294	78759706	Distal Interger	-0501	225E-04	593E-03	Syt13	-117494	Hypo
chr13	78197928	78198914	Intron (NM_0(	-0619	225E-04	593E-03	Pou2f1	64347	Hypo
chr19	29903037	29903683	Distal Interger	-0806	226E-04	593E-03	Ttc29	-152422	Hypo
chr6	39946158	39950298	Distal Interger	-0415	226E-04	593E-03	Kcnf1	14681	Hypo
chr8	115678480	115680484	Distal Interger	-0623	226E-04	594E-03	Tgfbr2	202754	Hypo
chr3	103813982	103815732	Distal Interger	-0585	226E-04	594E-03	Spred1	-168297	Hypo
chr4	118240959	118246942	Promoter (<=:	-0370	226E-04	594E-03	Nat8f3	0	Hypo
chr20	21635528	21637608	Distal Interger	-0544	227E-04	594E-03	Reep3	141660	Hypo
chr5	153224822	153225859	Intron (NM_0	-0679	227E-04	596E-03	Padi2	15769	Hypo
chr20	7744450	7746807	Exon (NM_00:	-0472	228E-04	597E-03	Cmtr1	21656	Hypo
chr20	32707571	32709312	Distal Interger	-0508	228E-04	598E-03	Cep85l	-27407	Hypo
chr2	16323837	16325577	Distal Interger	-0575	228E-04	598E-03	Rasa1	-383556	Hypo
chr1	241733286	241734408	Intron (NM_0(	-0677	229E-04	598E-03	Hps1	-157302	Hypo
chr15	79676168	79677387	Distal Interger	-0597	229E-04	598E-03	Acod1	-194440	Hypo
chr18	53367035	53367713	Distal Interger	-0793	230E-04	600E-03	Ifgga2l1	-393405	Hypo
chr17	45340696	45342039	Intron (NM_0(	-0645	230E-04	600E-03	Epdr1	6730	Hypo
chr8	43914412	43916914	Distal Interger	-0531	230E-04	600E-03	Nectin1	-184862	Hypo
chr3	105534018	105534454	Distal Interger	-1034	230E-04	601E-03	Bmf	-13914	Hypo
chr2	167707963	167709681	Promoter (<=:	-0422	230E-04	601E-03	Map9	0	Hypo
chr16	36960941	36961492	Distal Interger	-0861	231E-04	601E-03	Gpm6a	-206781	Hypo
chr8	81156573	81158780	Distal Interger	-0490	231E-04	602E-03	Senp6	167521	Hypo
chr1	77390781	77396907	Distal Interger	-0369	231E-04	602E-03	Ceacam9	-8712	Hypo

chr8	27308758	27311351	Distal Interger	-0477	233E-04	605E-03	Ntm	492877	Hypo
chr1	144889016	144891080	Distal Interger	-0607	233E-04	606E-03	Dlg2	-236095	Hypo
chr12	33875149	33878040	Distal Interger	-0399	233E-04	606E-03	P2rx4	-12884	Hypo
chr8	59717490	59720008	Exon (NM_00:	-0559	233E-04	607E-03	Adpgk	18046	Hypo
chr4	46259449	46263518	Intron (NM_0(	-0403	234E-04	607E-03	Asz1	192567	Hypo
chr14	40106505	40109651	Intron (NM_0(	-0432	234E-04	608E-03	Grxcr1	16920	Hypo
chr14	59650649	59651067	Distal Interger	-0991	234E-04	608E-03	Ppargc1a	229043	Hypo
chr1	232329723	232330814	3' UTR	-0629	235E-04	608E-03	Mir107	7039	Hypo
chr9	15244578	15245363	Distal Interger	-0902	235E-04	609E-03	Mrpl14	63056	Hypo
chr4	87601219	87602149	Promoter (<=:	-0632	236E-04	612E-03	Rps7-ps20	0	Hypo
chr14	68733654	68734482	Distal Interger	-0647	237E-04	614E-03	Rab28	-511544	Hypo
chr5	129595425	129597105	Intron (NM_0(	-0518	237E-04	614E-03	Rad54l	7945	Hypo
chr11	34097640	34100359	Intron (NM_0	-0413	237E-04	614E-03	Dyrk1a	181456	Hypo
chr9	103209251	103211094	Distal Interger	-0558	238E-04	614E-03	Fbxl17	-3555	Hypo
chr5	143477374	143481970	Distal Interger	-0402	238E-04	614E-03	Matn1	359870	Hypo
chr9	79997976	79999350	Distal Interger	-0608	238E-04	614E-03	Mogat1	17013	Hypo
chr2	83653958	83655815	Intron (NM_0(	-0578	238E-04	614E-03	Sema5a	344115	Hypo
chr12	27314233	27315191	Distal Interger	-0545	238E-04	615E-03	Sfswap	-134349	Hypo
chr11	54809297	54810692	Intron (NM_0(	-0698	239E-04	617E-03	Plcxd2	8297	Hypo
chr2	23889708	23892947	Intron (NM_1:	-0440	239E-04	617E-03	Serinc5	42790	Hypo
chrX	71220378	71222953	Distal Interger	-0590	240E-04	619E-03	Pgk1	-48501	Hypo
chr11	29159243	29162323	Promoter (<=:	-0511	240E-04	619E-03	Tiam1	0	Hypo
chr4	47281290	47283355	Distal Interger	-0499	241E-04	621E-03	Lsm8	-22904	Hypo
chr5	117034939	117036839	Intron (NM_0	-0549	241E-04	621E-03	Pde4b	41503	Hypo
chr2	220159015	220159765	Distal Interger	-0749	241E-04	621E-03	Papss1	118631	Hypo
chr11	15886916	15888371	Distal Interger	-0666	242E-04	622E-03	Usp25	283035	Hypo
chr5	154179902	154182085	Promoter (2-3	-0450	242E-04	622E-03	Efhd2	-2922	Hypo
chr20	46065695	46073869	Promoter (<=:	-0373	243E-04	624E-03	Nr2e1	0	Hypo
chr2	240082816	240083354	Distal Interger	-0857	243E-04	625E-03	Adgrl4	-271587	Hypo
chr8	59705784	59708348	Exon (NM_00:	-0612	244E-04	625E-03	Adpgk	6340	Hypo
chr10	86620313	86625050	Distal Interger	-0394	244E-04	625E-03	Arl4d	19626	Hypo
chr15	94901483	94902686	Distal Interger	-0685	246E-04	629E-03	Tgds	292891	Hypo
chr3	43886593	43888866	Distal Interger	-0453	246E-04	629E-03	Dapl1	-72127	Hypo

chr6	105031400	105032389	Distal Interger	-0772	246E-04	629E-03	Tmed10	-4695	Hypo
chr2	6768929	6770642	Exon (NM_00:	-0540	247E-04	631E-03	Slf1	103786	Hypo
chr7	129351335	129352923	Distal Interger	-0593	247E-04	632E-03	Ccdc184	51662	Hypo
chr20	21098364	21099265	Distal Interger	-0803	249E-04	635E-03	Egr2	-42802	Hypo
chr16	45434392	45434911	Distal Interger	-0839	249E-04	636E-03	Irf2	115048	Hypo
chr3	60961728	60962058	Intron (NM_0(	-0938	250E-04	636E-03	Ift70a1	-56378	Hypo
chr1	170631340	170633428	Exon (NM_00:	-0468	250E-04	636E-03	Pik3c2a	50044	Hypo
chr7	64514026	64515005	Intron (NM_0:	-0594	250E-04	636E-03	Cpq	249846	Hypo
chr2	235509456	235513252	Intron (NM_0!	-0475	250E-04	637E-03	Uox	22589	Hypo
chr4	150938355	150939607	Distal Interger	-0757	251E-04	638E-03	Zfp637	-65718	Hypo
chr17	21969768	21971413	Intron (NM_2	-0502	251E-04	638E-03	Phactr1	67827	Hypo
chr6	35012794	35013884	Distal Interger	-0591	251E-04	639E-03	Cyria	-31324	Hypo
chr9	11646484	11648024	Distal Interger	-0551	252E-04	640E-03	Mocs1	-78694	Hypo
chr6	42250090	42252838	Distal Interger	-0575	253E-04	642E-03	Id2	-507697	Hypo
chr4	175895927	175896677	Intron (NM_0	-0830	253E-04	643E-03	St8sia1	23900	Hypo
chr7	27946821	27950495	Distal Interger	-0406	254E-04	644E-03	Lta4h	-19323	Hypo
chr8	40867077	40869174	Distal Interger	-0453	254E-04	644E-03	Gramd1b	-45286	Hypo
chr11	57970261	57971115	Distal Interger	-0808	255E-04	645E-03	Zbtb20	-179047	Hypo
chr5	24022630	24025225	Distal Interger	-0466	255E-04	646E-03	Cfap418	25912	Hypo
chr18	26553134	26555586	Promoter (<=:	-0346	255E-04	646E-03	Hspa9	0	Hypo
chr14	103382041	103387033	Distal Interger	-0425	256E-04	648E-03	Rtn4	-63157	Hypo
chr3	32441322	32442277	Distal Interger	-0724	256E-04	648E-03	Acvr2a	-763246	Hypo
chr16	42943554	42943994	Distal Interger	-0923	256E-04	648E-03	Tenm3	-584000	Hypo
chr9	106331940	106339307	Distal Interger	-0349	256E-04	648E-03	Rab12	167492	Hypo
chr13	78195826	78197617	Intron (NM_0(	-0472	257E-04	651E-03	Pou2f1	65644	Hypo
chr5	61609996	61611403	Distal Interger	-0551	258E-04	652E-03	Tgfbr1	-42396	Hypo
chr6	68486956	68490349	Distal Interger	-0403	258E-04	652E-03	G2e3	-273836	Hypo
chr9	89215246	89216536	Distal Interger	-0568	258E-04	652E-03	Arl4c	87234	Hypo
chr3	63198848	63202514	Distal Interger	-0438	258E-04	652E-03	RGD1564319	-442101	Hypo
chr5	38701626	38704303	Intron (NM_0(	-0406	259E-04	653E-03	Gpr63	20214	Hypo
chr1	34483189	34483527	Intron (NM_0:	-1027	260E-04	655E-03	Adcy2	107278	Hypo
chr18	42572595	42576370	Distal Interger	-0403	260E-04	655E-03	Dtwd2	325494	Hypo
chr11	61571340	61572365	Distal Interger	-0661	260E-04	655E-03	Igsf11	295983	Hypo

chr10	36798770	36800296	Distal Interger	-0588	260E-04	655E-03	Fstl4	-111124	Hypo
chr6	9899897	9903700	Exon (NM_00:	-0434	261E-04	658E-03	Lrpprc	40030	Hypo
chr20	9178788	9182728	3' UTR	-0477	262E-04	659E-03	Tff3	15241	Hypo
chr1	127380037	127387908	Distal Interger	-0304	262E-04	659E-03	Chd2	-79537	Hypo
chr2	100724642	100725287	Distal Interger	-0815	263E-04	661E-03	Cyp7b1	-54944	Hypo
chr13	73448040	73449580	Distal Interger	-0612	264E-04	662E-03	Klhl20	-39721	Hypo
chr7	30262863	30263826	Distal Interger	-0598	264E-04	662E-03	Nudt4	-58536	Hypo
chr8	61637989	61641873	Distal Interger	-0449	265E-04	664E-03	LOC691000	131324	Hypo
chr5	143215538	143216060	Distal Interger	-1004	266E-04	666E-03	Matn1	98034	Hypo
chr7	105870600	105872168	Distal Interger	-0599	266E-04	666E-03	Ptp4a3	215494	Hypo
chr1	256601528	256605345	Distal Interger	-0380	267E-04	667E-03	Trub1	85966	Hypo
chr6	124744898	124746700	Distal Interger	-0596	267E-04	668E-03	Papola	62793	Hypo
chr17	71043110	71051872	Distal Interger	-0378	267E-04	668E-03	Celf2	-357738	Hypo
chr20	37159434	37160649	Distal Interger	-0579	267E-04	669E-03	Smpdl3a	26842	Hypo
chr9	5292531	5298737	Distal Interger	-0438	268E-04	670E-03	Kcnh8	-207307	Hypo
chr7	29024185	29024839	Distal Interger	-0884	268E-04	670E-03	Tmcc3	-75336	Hypo
chr1	132632904	132634120	Distal Interger	-0702	269E-04	672E-03	Mrpl46	73519	Hypo
chr1	194810677	194811230	Promoter (1-2	-0708	270E-04	674E-03	Tubgcp2	1446	Hypo
chr3	142655212	142658242	Distal Interger	-0423	271E-04	676E-03	Bpifa5	9109	Hypo
chr17	71184008	71186248	Distal Interger	-0579	271E-04	676E-03	Celf2	-223362	Hypo
chr9	110596381	110600832	Intron (NM_0:	-0383	271E-04	676E-03	Tgif1	156882	Hypo
chr9	75037290	75037782	Distal Interger	-0904	272E-04	676E-03	Pincl	-37530	Hypo
chr3	34118667	34120953	Exon (NM_00:	-0478	272E-04	677E-03	Lypd6b	-78646	Hypo
chr3	168411785	168414798	Distal Interger	-0488	273E-04	678E-03	Stmn3	10108	Hypo
chr17	86379072	86379756	Intron (NM_0!	-0827	273E-04	678E-03	Lyst	99553	Hypo
chr7	19036006	19036516	Distal Interger	-0908	274E-04	679E-03	Polr3b	105934	Hypo
chr20	6933629	6939962	Exon (NM_00:	-0354	275E-04	681E-03	Pnpla1	15183	Hypo
chr20	41256556	41258115	Distal Interger	-0624	276E-04	682E-03	Marcks	-565544	Hypo
chr10	65660390	65664394	Exon (NM_01:	-0389	276E-04	682E-03	Myo1d	101418	Hypo
chr1	78779403	78780148	Intron (NM_0(	-0510	276E-04	682E-03	Fbxo46	9247	Hypo
chrX	64582829	64586760	Promoter (<=:	-0368	276E-04	683E-03	Pja1	0	Hypo
chr10	16976576	16977450	Distal Interger	-0782	277E-04	683E-03	Efcab9	134795	Hypo
chr1	254210306	254211813	Distal Interger	-0582	277E-04	683E-03	Tectb	-9808	Hypo

chr5	76232136	76232576	Distal Intergen	-0976	277E-04	683E-03	Rgs3	153644	Hypo
chr1	211992204	211993603	Distal Intergen	-0623	277E-04	683E-03	Tle4	-194342	Hypo
chr2	208832949	208833510	Distal Intergen	-0819	277E-04	684E-03	Rwdd3	415343	Hypo
chr1	177199885	177204881	Promoter (<=1	-0373	277E-04	684E-03	Cacng3	0	Hypo
chr5	110895346	110897915	Distal Intergen	-0407	279E-04	687E-03	Hook1	70845	Hypo
chr1	256341339	256342495	Distal Intergen	-0644	279E-04	688E-03	Ablim1	-64844	Hypo
chr10	16802491	16804530	Distal Intergen	-0559	280E-04	688E-03	Neurl1b	-17548	Hypo
chr7	125559746	125562352	Distal Intergen	-0448	280E-04	688E-03	Irak4	-120150	Hypo
chr14	94147888	94151898	Promoter (<=1	-0417	281E-04	690E-03	Spred2	0	Hypo
chr9	23307352	23308517	Distal Intergen	-0700	281E-04	690E-03	Paqr8	-33089	Hypo
chr5	53573115	53573493	Distal Intergen	-0929	281E-04	690E-03	RGD1306195	-882512	Hypo
chr17	28875199	28878311	Distal Intergen	-0449	281E-04	690E-03	Rpp40	-69849	Hypo
chrX	47581286	47581816	Intron (NM_0	-0912	281E-04	690E-03	Dmd	308962	Hypo
chr9	95871601	95873706	Distal Intergen	-0495	281E-04	690E-03	Fam174a	128674	Hypo
chr4	47057914	47058524	Distal Intergen	-0817	282E-04	690E-03	Cttnbp2	-93283	Hypo
chr3	43429389	43429884	Intron (NM_0	-0942	282E-04	690E-03	Upp2	156341	Hypo
chr2	248104098	248105797	Distal Intergen	-0505	282E-04	691E-03	Lrrc7	-570766	Hypo
chr8	37701227	37703539	Distal Intergen	-0538	283E-04	692E-03	Or8b8	7649	Hypo
chr3	118213441	118214356	Intron (NM_0	-0739	284E-04	694E-03	Gfra4	43567	Hypo
chr4	11586261	11586674	Intron (NM_0	-0828	284E-04	695E-03	Srpk2	48876	Hypo
chr3	47160905	47166756	Exon (NM_13	-0350	285E-04	697E-03	Gcg	-37976	Hypo
chr17	5788937	5795616	Exon (NM_01	-0340	286E-04	698E-03	Ntrk2	73505	Hypo
chr8	110889913	110890714	Distal Intergen	-0712	286E-04	698E-03	Tmie	-25110	Hypo
chr5	25429166	25429787	Distal Intergen	-0754	286E-04	698E-03	Pdp1	24815	Hypo
chr9	23259214	23260562	Distal Intergen	-0571	286E-04	698E-03	Mcm3	-21900	Hypo
chr9	13420721	13422181	Distal Intergen	-0599	286E-04	698E-03	Ccnd3	-20480	Hypo
chr3	157882689	157883806	Distal Intergen	-0752	286E-04	699E-03	Zfp64	-186915	Hypo
chr9	65238194	65240575	Distal Intergen	-0469	288E-04	703E-03	Fastkd2	68727	Hypo
chr18	53365655	53366733	Distal Intergen	-0632	289E-04	703E-03	Ifgga2l1	-394385	Hypo
chr1	117672607	117675907	Distal Intergen	-0404	289E-04	703E-03	Trpm1	-42989	Hypo
chr2	78471675	78473487	Distal Intergen	-0545	289E-04	704E-03	Otulinl	-37850	Hypo
chr10	15802805	15805347	Distal Intergen	-0471	289E-04	704E-03	Cpeb4	-23693	Hypo
chr10	37660599	37664924	Distal Intergen	-0404	291E-04	706E-03	Septin8	-19715	Hypo

chr4	119612322	119613442	Intron (NM_0(	-0673	291E-04	706E-03	Gfpt1	115565	Hypo
chr17	28332235	28333432	Intron (NM_0(	-0662	291E-04	706E-03	Nrn1	202266	Hypo
chr15	34784995	34789051	Intron (NM_0(	-0422	292E-04	707E-03	Spata13	6459	Hypo
chr18	31814617	31817802	Distal Interger	-0391	293E-04	709E-03	Pabpc2	160399	Hypo
chr3	61477710	61481407	Exon (NM_00:	-0460	293E-04	710E-03	Osbpl6	38905	Hypo
chr4	76792291	76794154	Distal Interger	-0371	294E-04	711E-03	Pdia4	28091	Hypo
chr13	88771787	88772417	Intron (NM_1:	-0741	294E-04	711E-03	Sdccag8	17138	Hypo
chr17	70114825	70119397	Distal Interger	-0404	295E-04	711E-03	Celf2	-1290213	Hypo
chr6	89210688	89215382	Distal Interger	-0368	295E-04	712E-03	Frmd6	-130632	Hypo
chr2	170722119	170727426	Distal Interger	-0351	295E-04	712E-03	Fhip1a	364056	Hypo
chr8	76888188	76889205	Distal Interger	-0616	295E-04	712E-03	Gfral	73849	Hypo
chr3	147215914	147218021	Distal Interger	-0558	296E-04	714E-03	Slc32a1	-49352	Hypo
chr18	23039600	23048557	Promoter (<=:	-0340	298E-04	717E-03	Syt4	0	Hypo
chr1	170663580	170664365	Intron (NM_0(	-0740	298E-04	717E-03	Pik3c2a	19107	Hypo
chr2	103519031	103524468	Distal Interger	-0345	298E-04	718E-03	Agtr1b	-598799	Hypo
chr9	3428654	3431625	Intron (NM_0(	-0497	299E-04	718E-03	Plcl2	135286	Hypo
chr6	71197221	71198474	Distal Interger	-0613	299E-04	718E-03	Egln3	477292	Hypo
chr1	33870294	33871757	Distal Interger	-0502	299E-04	718E-03	Tent4a	103656	Hypo
chr1	242395135	242397746	Distal Interger	-0482	300E-04	720E-03	Got1	-14512	Hypo
chr9	98042241	98045321	Distal Interger	-0466	300E-04	720E-03	Pam	-77711	Hypo
chr16	83445279	83446623	Distal Interger	-0579	300E-04	721E-03	Slc10a2	-939905	Hypo
chr16	29362940	29364101	Distal Interger	-0690	301E-04	721E-03	Mfap3l	66762	Hypo
chr17	5832022	5834920	Intron (NM_0	-0432	301E-04	722E-03	Ntrk2	34201	Hypo
chr2	172535162	172536611	Intron (NM_2(	-0648	301E-04	722E-03	Kirrel1	44148	Hypo
chr6	110148221	110148853	Distal Interger	-0682	302E-04	723E-03	Tshr	-192732	Hypo
chr9	110490108	110491403	Intron (NM_0:	-0445	302E-04	723E-03	Dlgap1	72285	Hypo
chr3	48366005	48367281	Distal Interger	-0658	303E-04	724E-03	Fign	393424	Hypo
chr5	119692216	119693314	Distal Interger	-0673	303E-04	724E-03	C8a	-54541	Hypo
chr14	10212407	10218923	Distal Interger	-0361	304E-04	726E-03	Prkg2	-351535	Hypo
chr5	117292339	117295203	Intron (NM_0	-0371	304E-04	726E-03	Sgip1	-242543	Hypo
chr18	47981471	47983808	Distal Interger	-0521	304E-04	726E-03	Zfp608	426657	Hypo
chr1	3157605	3160516	Distal Interger	-0422	304E-04	726E-03	Ust	-195561	Hypo
chr2	33854945	33858640	Promoter (<=:	-0430	304E-04	726E-03	Cd180	0	Hypo

chr1	67511375	67512043	Distal Intergen	-0837	305E-04	727E-03	Smim17	-48436	Hypo
chr17	1423266	1424001	Distal Intergen	-0740	307E-04	731E-03	Ptch1	-126571	Hypo
chr6	70743758	70747625	Distal Intergen	-0389	308E-04	734E-03	Akap6	559583	Hypo
chr3	116755215	116757120	Intron (NM_0	-0455	309E-04	735E-03	Sirpb3	6126	Hypo
chr3	34581455	34582123	Distal Intergen	-0818	310E-04	737E-03	Ns5atp4l1	-114985	Hypo
chr2	165520263	165522540	Distal Intergen	-0425	311E-04	738E-03	Mir2985	433958	Hypo
chr8	4042433	4042853	Distal Intergen	-1006	312E-04	741E-03	Dync2h1	369330	Hypo
chr12	45170659	45171837	Distal Intergen	-0650	313E-04	742E-03	Mn1	48265	Hypo
chr1	147661717	147662353	Distal Intergen	-0774	314E-04	744E-03	Fam181b	495289	Hypo
chr8	71836910	71838177	Promoter (<=1	-0583	314E-04	744E-03	Aqp9	0	Hypo
chr14	16079780	16080315	Distal Intergen	-0969	314E-04	745E-03	Rchy1	-32948	Hypo
chr5	14530282	14538408	Promoter (<=1	-0321	314E-04	745E-03	Rgs20	0	Hypo
chr17	42682713	42684015	Distal Intergen	-0543	315E-04	745E-03	Hist1h4m	-14039	Hypo
chr9	11696983	11698548	Distal Intergen	-0540	315E-04	745E-03	Mocs1	-129193	Hypo
chr8	47101555	47103117	Distal Intergen	-0632	315E-04	745E-03	LOC500990	-491081	Hypo
chr9	107706651	107707244	Intron (NM_0	-0852	315E-04	745E-03	Lama1	13881	Hypo
chr2	43934028	43935966	Intron (NM_0	-0500	315E-04	745E-03	Ankrd55	19518	Hypo
chr7	20144812	20148528	Intron (NM_0	-0415	315E-04	745E-03	Appl2	9437	Hypo
chr5	148345732	148347540	Distal Intergen	-0434	316E-04	747E-03	Id3	-25244	Hypo
chr9	41136958	41137580	Distal Intergen	-0936	316E-04	748E-03	Chst10	-14281	Hypo
chr7	127758094	127760262	Intron (NM_1	-0503	318E-04	750E-03	Slc38a1	41956	Hypo
chr1	141380324	141381145	Intron (NM_0	-0726	318E-04	750E-03	Grm5	59195	Hypo
chr18	31783436	31784660	Distal Intergen	-0532	319E-04	752E-03	Pabpc2	129218	Hypo
chr6	111207087	111207796	Distal Intergen	-0636	319E-04	752E-03		308680	Hypo
chr8	5617509	5618724	Distal Intergen	-0639	320E-04	754E-03	Rpl13	79581	Hypo
chr17	69858400	69861369	Distal Intergen	-0392	321E-04	755E-03	Gata3	1213487	Hypo
chr4	168053180	168055731	Intron (NM_0	-0432	321E-04	755E-03	Fam234b	4784	Hypo
chr12	28128465	28128835	Distal Intergen	-1088	321E-04	755E-03	Fzd10	-26899	Hypo
chr11	60712577	60712999	Distal Intergen	-0914	321E-04	756E-03		404751	Hypo
chr15	99505043	99510413	3' UTR	-0376	322E-04	756E-03	Zic5	56622	Hypo
chr8	65299287	65301902	Distal Intergen	-0496	322E-04	756E-03	Rab11a	-52826	Hypo
chr8	113302039	113315382	Distal Intergen	-0352	322E-04	756E-03	Pdcd6ip	331391	Hypo
chr7	113508640	113510185	Distal Intergen	-0582	323E-04	757E-03	Pmm1	-31749	Hypo

chr6	109848119	109849580	Distal Intergen	-0609	324E-04	759E-03	Dio2	-168310	Hypo
chr5	82472149	82473976	Intron (NM_0	-0563	324E-04	759E-03	Brinp1	19171	Hypo
chr1	212299106	212301419	Distal Intergen	-0462	324E-04	759E-03	Tle4	-501244	Hypo
chr11	64023610	64027029	Exon (NM_03	-0421	325E-04	760E-03	Slc15a2	9428	Hypo
chr2	203880937	203883470	Intron (NM_0	-0408	326E-04	763E-03	Slc30a7	38639	Hypo
chr8	50015005	50021084	Intron (NM_0	-0360	326E-04	763E-03	Ncam1	144453	Hypo
chr4	88055116	88057850	Distal Intergen	-0381	326E-04	763E-03	Nap1l5	-30576	Hypo
chr3	11893156	11897975	Promoter (2-3	-0388	327E-04	764E-03	Cel	-2121	Hypo
chr16	55980111	55983250	Distal Intergen	-0404	327E-04	764E-03	Prag1	157903	Hypo
chr3	33982218	33983556	Distal Intergen	-0555	327E-04	764E-03	Kif5c	-48549	Hypo
chr17	16524973	16526467	Distal Intergen	-0524	327E-04	764E-03	Id4	-133017	Hypo
chr1	166433231	166439082	Exon (NM_00	-0354	327E-04	764E-03	Mical2	42212	Hypo
chr4	83248826	83250459	Distal Intergen	-0579	328E-04	766E-03	Chn2	-126452	Hypo
chr14	37394779	37398133	Promoter (<=1	-0386	328E-04	766E-03	Gabrg1	0	Hypo
chr8	57104139	57119954	Distal Intergen	-0324	328E-04	766E-03	Lingo1	-75966	Hypo
chr7	47885822	47887479	Distal Intergen	-0545	328E-04	766E-03	Kcnc2	184401	Hypo
chr10	97806441	97812596	Promoter (<=1	-0409	329E-04	766E-03	Sox9	0	Hypo
chr15	55561920	55562430	Distal Intergen	-0894	329E-04	766E-03	LOC306079	41588	Hypo
chr12	14420181	14425139	Intron (NM_0	-0417	329E-04	766E-03	Mad1l1	99289	Hypo
chr1	22786681	22788578	Intron (NM_0	-0469	329E-04	766E-03	Tbpl1	8200	Hypo
chr7	110550400	110551277	Intron (NM_0	-0773	329E-04	766E-03	H1f0	-41557	Hypo
chr17	16948106	16948755	Distal Intergen	-0870	329E-04	766E-03	Id4	-556150	Hypo
chr1	90250900	90254148	Distal Intergen	-0431	329E-04	766E-03	Uri1	450508	Hypo
chr10	15745782	15747090	Intron (NM_0	-0599	331E-04	769E-03	Cpeb4	32022	Hypo
chr3	54929123	54931209	Intron (NM_0	-0528	331E-04	770E-03	Myo3b	55419	Hypo
chr15	16612355	16626240	Promoter (<=1	-0328	333E-04	773E-03	Fam107a	0	Hypo
chr4	100089485	100089938	Distal Intergen	-0915	334E-04	773E-03	Rpia	2659417	Hypo
chr15	47382648	47384177	Distal Intergen	-0568	334E-04	774E-03	Fndc3a	448403	Hypo
chr6	118497859	118500221	Intron (NM_0	-0460	334E-04	774E-03	Cpg1	185639	Hypo
chr6	114820153	114828310	Intron (NM_0	-0338	335E-04	776E-03	Flrt2	41281	Hypo
chr3	88962809	88972476	Intron (NM_0	-0324	335E-04	776E-03	Slc1a2	-32653	Hypo
chr13	33790890	33791520	Distal Intergen	-0801	335E-04	776E-03	Ddx18	-1047729	Hypo
chr11	61568006	61569137	Distal Intergen	-0545	336E-04	776E-03	Igsf11	299211	Hypo

chr3	69470051	69470464	Exon (NM_01	-0985	336E-04	777E-03	Calcrl	55186	Hypo
chr2	116077135	116078722	Distal Interger	-0520	336E-04	778E-03	Pex5l	-169153	Hypo
chr2	38935417	38937248	Distal Interger	-0435	336E-04	778E-03	Kif2a	-504180	Hypo
chr3	115714867	115715360	Intron (NR_11	-0910	337E-04	779E-03	LOC10255036	79774	Hypo
chr7	92847929	92849950	Distal Interger	-0579	338E-04	780E-03	A1bg	-349832	Hypo
chr1	200327534	200329020	Distal Interger	-0535	339E-04	781E-03	Smim38	42956	Hypo
chr7	26879563	26880156	Distal Interger	-0822	339E-04	782E-03	Mir135a	-267474	Hypo
chr4	83250662	83251691	Distal Interger	-0586	339E-04	783E-03	Chn2	-125220	Hypo
chr11	31080922	31081359	Intron (NM_0	-0949	339E-04	783E-03	Itsn1	53692	Hypo
chr8	111176684	111178859	Intron (NM_0	-0521	341E-04	785E-03	Mlh1	54683	Hypo
chr4	42252851	42253658	Distal Interger	-0728	341E-04	785E-03	LOC10036352	-109511	Hypo
chr1	15760993	15764840	Distal Interger	-0347	342E-04	786E-03	Ahi1	-3207	Hypo
chr20	21623391	21626727	Distal Interger	-0439	342E-04	786E-03	Reep3	129523	Hypo
chr7	129322369	129322885	Distal Interger	-0912	343E-04	788E-03	Ccdc184	22696	Hypo
chr2	170538855	170544257	Distal Interger	-0338	343E-04	789E-03	Dear	-238673	Hypo
chr14	76584173	76585326	Intron (NM_0	-0617	345E-04	791E-03	Haus3	3624	Hypo
chr7	54843623	54844621	Distal Interger	-0639	345E-04	791E-03	Cand1	-124621	Hypo
chr16	18379854	18382635	Promoter (<=1	-0414	346E-04	793E-03	Unc13a	0	Hypo
chr15	77235011	77240185	Distal Interger	-0340	346E-04	793E-03	Klf12	-247271	Hypo
chr13	62008669	62010690	Intron (NM_1	-0541	346E-04	793E-03	Pla2g4a	11571	Hypo
chr8	10646377	10648976	Exon (NM_00	-0482	346E-04	793E-03	Mtmr2	28384	Hypo
chr5	97804408	97804714	Distal Interger	-1077	346E-04	794E-03	Snapc3	-13233	Hypo
chr2	153279389	153289430	Promoter (<=1	-0382	348E-04	796E-03	Trim59	0	Hypo
chr16	25093743	25098234	Intron (NM_0	-0371	349E-04	797E-03	Cpe	63467	Hypo
chr11	15912211	15916265	Distal Interger	-0358	349E-04	798E-03	Mir99a	-284178	Hypo
chr1	180010077	180015615	Promoter (<=1	-0317	349E-04	798E-03	Kdm8	0	Hypo
chr2	232753969	232755127	Distal Interger	-0574	349E-04	798E-03	Lmo4	525754	Hypo
chr2	220517611	220518187	Distal Interger	-0908	350E-04	799E-03	Dkk2	-50151	Hypo
chr16	13014337	13014878	Distal Interger	-0840	352E-04	803E-03	Ghitm	-116450	Hypo
chr13	95145265	95145834	Distal Interger	-0912	352E-04	803E-03	Taf1a	133105	Hypo
chr8	55652869	55654051	Intron (NM_0	-0581	352E-04	803E-03	Nrg4	4985	Hypo
chr7	76130007	76131242	Distal Interger	-0577	353E-04	804E-03	Kcnv1	136598	Hypo
chr14	98479229	98480087	Distal Interger	-0639	354E-04	806E-03	Bcl11a	448661	Hypo

chr16	75805190	75805706	Intron (NM_0(	-0809	354E-04	806E-03	Cdc16	-8633	Hypo
chr9	28240339	28241078	Distal Interger	-0797	355E-04	807E-03	Adgrb3	-91789	Hypo
chr12	12867964	12868694	Distal Interger	-0824	355E-04	807E-03	Foxk1	-692875	Hypo
chr3	116973473	116973940	Distal Interger	-0826	356E-04	810E-03	Stk35	-43010	Hypo
chr14	91071327	91072017	Distal Interger	-0802	356E-04	810E-03	Egfr	-105050	Hypo
chr6	36772124	36772599	Distal Interger	-0884	357E-04	811E-03	Lratd1	149544	Hypo
chr3	11568932	11574042	Distal Interger	-0390	357E-04	811E-03	Olfm1	35624	Hypo
chr4	119509488	119509876	Intron (NM_0(	-1013	357E-04	811E-03	Gfpt1	12731	Hypo
chr2	214291063	214293460	Intron (NM_0	-0543	357E-04	811E-03	Ugt8	39200	Hypo
chr16	36959918	36960297	Distal Interger	-0916	358E-04	812E-03	Gpm6a	-205758	Hypo
chr5	164875605	164876887	Distal Interger	-0515	358E-04	812E-03	Arhgef16	-9393	Hypo
chr3	29505913	29506940	Distal Interger	-0719	358E-04	812E-03	Zeb2-as1	162835	Hypo
chr14	60665784	60666637	Promoter (2-3	-0699	358E-04	813E-03	Gba3	-2832	Hypo
chr16	78846375	78846960	Distal Interger	-0892	359E-04	814E-03		74972	Hypo
chr6	70901067	70902597	Distal Interger	-0546	360E-04	815E-03	Akap6	716892	Hypo
chr1	206529285	206532167	Intron (NM_0(	-0441	360E-04	816E-03	Incenp	17558	Hypo
chr8	41946622	41959255	Promoter (<=1	-0289	360E-04	816E-03	Mir125b1	0	Hypo
chr5	75521248	75522350	Distal Interger	-0656	361E-04	816E-03	Mup4l1	-22381	Hypo
chr3	111437194	111439609	Distal Interger	-0444	361E-04	816E-03	Sema6d	-444263	Hypo
chr3	11603321	11605166	Distal Interger	-0520	361E-04	816E-03	Olfm1	70013	Hypo
chr7	60983535	60984481	Distal Interger	-0713	361E-04	816E-03	Slc16a7	170525	Hypo
chr10	63690674	63694149	Distal Interger	-0400	362E-04	817E-03	Lyrm9	-31594	Hypo
chr7	34297019	34297455	Distal Interger	-0868	362E-04	817E-03	Dusp6	204063	Hypo
chr5	150402656	150407853	Exon (NM_001	-0350	363E-04	818E-03	Hp1bp3	-27934	Hypo
chr7	127323159	127323580	Distal Interger	-0878	363E-04	818E-03	Scaf11	300335	Hypo
chr3	111371500	111372849	Distal Interger	-0551	363E-04	820E-03	Sema6d	-511023	Hypo
chr11	34911601	34912518	Distal Interger	-0740	364E-04	821E-03	Ets2	-109018	Hypo
chr3	154759480	154760645	Intron (NM_05	-0657	364E-04	821E-03	Ncoa3	20899	Hypo
chr4	147019475	147026119	Intron (NM_0	-0357	365E-04	821E-03	Sec13	-128345	Hypo
chr9	12620036	12621427	Distal Interger	-0566	366E-04	824E-03	Treml1	20872	Hypo
chrX	118211545	118212798	Distal Interger	-0734	366E-04	824E-03	LOC691215	654554	Hypo
chr20	45893225	45894619	Intron (NM_0(	-0512	367E-04	824E-03	Afg1l	73287	Hypo
chr8	42458141	42463065	Exon (NM_05	-0375	367E-04	825E-03	Sorl1	41163	Hypo

chr10	73241414	73242554	Distal Intergen	-0636	369E-04	827E-03	Ccdc182	151186	Hypo
chr5	129671553	129673313	Distal Intergen	-0601	369E-04	827E-03	Tspan1	-19536	Hypo
chr7	29790663	29794290	Distal Intergen	-0455	369E-04	828E-03	Cradd	158617	Hypo
chr3	20540709	20541497	Distal Intergen	-0771	370E-04	830E-03	Or12k7b	11244	Hypo
chr1	8415587	8419760	Intron (NM_0:	-0367	371E-04	831E-03	Hivep2	56298	Hypo
chr18	22769860	22770836	Intron (NM_0(	-0610	371E-04	831E-03	Rit2	150812	Hypo
chr3	37454993	37460094	Distal Intergen	-0357	371E-04	831E-03	Stam2	-220183	Hypo
chr4	116013368	116015747	Promoter (<=:	-0332	372E-04	832E-03	Dguok	0	Hypo
chr18	74598995	74601719	Distal Intergen	-0456	372E-04	832E-03	Sall3	-185167	Hypo
chr18	55503872	55507464	Distal Intergen	-0447	372E-04	833E-03	Ablim3	-127492	Hypo
chr18	136756	138694	Promoter (<=:	-0345	373E-04	833E-03	Fundc2	0	Hypo
chr7	42265924	42266279	Distal Intergen	-0928	374E-04	836E-03	Acss3	183951	Hypo
chr19	30475509	30476562	Intron (NM_0(	-0538	375E-04	838E-03	Arhgap10	26875	Hypo
chr7	68259761	68261676	Distal Intergen	-0536	375E-04	838E-03	Zfp706	-85719	Hypo
chr2	42513393	42515012	Distal Intergen	-0608	375E-04	838E-03	Actbl2	-343008	Hypo
chr12	44289672	44295772	Promoter (<=:	-0314	376E-04	840E-03	Hps4	0	Hypo
chr4	113973102	113974668	Distal Intergen	-0513	376E-04	840E-03	LOC10091137	488440	Hypo
chr13	38202052	38204302	Distal Intergen	-0440	377E-04	841E-03	Mgat5	-471817	Hypo
chr19	17680038	17681110	Distal Intergen	-0672	378E-04	842E-03	Sall1	-326393	Hypo
chr2	233716009	233718471	Distal Intergen	-0534	379E-04	843E-03	Sh3glb1	66313	Hypo
chr1	152596279	152597277	Distal Intergen	-0700	379E-04	843E-03	Tsku	74291	Hypo
chr13	20765170	20767046	Distal Intergen	-0594	379E-04	844E-03	Cdh20	-233613	Hypo
chr6	8389831	8392368	Intron (NM_0	-0464	380E-04	845E-03	Prkce	58951	Hypo
chr7	18951420	18952482	Distal Intergen	-0717	380E-04	846E-03	Ric8b	-109052	Hypo
chr12	11992124	11992720	Intron (NM_0(	-0763	380E-04	846E-03	Mmd2	29367	Hypo
chr10	6367032	6368678	Distal Intergen	-0540	382E-04	848E-03	Hapstr1	406314	Hypo
chr1	177113022	177119145	Exon (NM_01:	-0338	382E-04	848E-03	Cacng3	-82845	Hypo
chr4	62423688	62425887	Intron (NM_0!	-0459	383E-04	850E-03	Lrguk	-221377	Hypo
chr5	120998891	121000633	Distal Intergen	-0482	383E-04	850E-03	Usp24	-80129	Hypo
chr1	193673683	193676007	Intron (NM_1⁴	-0546	383E-04	850E-03	Ppp2r2d	7720	Hypo
chr4	114175010	114175909	Distal Intergen	-0642	383E-04	850E-03	LOC10091137	287199	Hypo
chr12	30904196	30911282	Intron (NM_0(	-0326	383E-04	850E-03	Tmem132b	87027	Hypo
chr6	39957089	39966126	Promoter (<=:	-0387	384E-04	851E-03	Kcnf1	0	Hypo

chr5	109227405	109227791	Distal Interger	-1034	384E-04	851E-03	Caap1	186452	Hypo
chr9	70520101	70522110	Intron (NM_0:	-0525	384E-04	851E-03	Erbb4	73865	Hypo
chr4	57386513	57388165	Intron (NM_0:	-0503	387E-04	856E-03	Lipogenin	30215	Hypo
chr7	86785584	86786412	Intron (NM_0(	-0716	387E-04	856E-03	Col14a1	63490	Hypo
chr3	13099247	13110134	Promoter (<=:	-0328	387E-04	856E-03	Urm1	0	Hypo
chr10	46480715	46481673	Intron (NM_0(	-0622	391E-04	863E-03	Prpsap2	-34873	Hypo
chr10	53322697	53325090	Distal Interger	-0417	392E-04	864E-03	Ccdc42	32741	Hypo
chr3	115938591	115941045	Promoter (<=:	-0398	392E-04	864E-03	Mertk	0	Hypo
chr10	63646497	63655880	Promoter (<=:	-0328	393E-04	866E-03	Ccnq	0	Hypo
chr8	41147659	41151348	Intron (NM_1	-0356	393E-04	866E-03	Hspa8	-32049	Hypo
chr2	7041292	7042759	Distal Interger	-0541	394E-04	867E-03	Slf1	-166864	Hypo
chr15	46829878	46830323	Distal Interger	-0820	394E-04	867E-03	Gfra2	887427	Hypo
chr2	185458356	185462932	Distal Interger	-0398	395E-04	869E-03	Pde4dip	-30470	Hypo
chr2	210520188	210521261	Distal Interger	-0632	396E-04	870E-03	Bcar3	-3999	Hypo
chr14	44460250	44460990	Intron (NM_0(	-0724	400E-04	877E-03	LOC498368	17051	Hypo
chr1	88335642	88338219	Distal Interger	-0463	401E-04	879E-03	Pdcd5	-24442	Hypo
chr6	111207876	111209265	Distal Interger	-0606	401E-04	879E-03		307211	Hypo
chr7	56198866	56199423	Distal Interger	-0862	402E-04	881E-03	Hmga2	-204082	Hypo
chr8	57828734	57831409	Intron (NM_0(	-0479	402E-04	881E-03	Ppcdc	7359	Hypo
chr8	72781782	72786516	Intron (NM_0	-0385	404E-04	883E-03	Tcf12	12685	Hypo
chr8	94993204	94994517	Distal Interger	-0760	405E-04	885E-03	Dipk2a	95298	Hypo
chr1	137924626	137926148	Distal Interger	-0595	407E-04	889E-03	Mesd	50298	Hypo
chr2	104503736	104504438	Distal Interger	-0786	408E-04	890E-03	Tbl1xr1	-297283	Hypo
chr17	21959592	21963192	Intron (NM_2	-0402	408E-04	890E-03	Phactr1	76048	Hypo
chr15	38921368	38926026	Exon (NM_21:	-0375	410E-04	894E-03	Kif13b	13050	Hypo
chr2	136249612	136251585	Distal Interger	-0420	411E-04	894E-03	Foxo1	-60583	Hypo
chr5	144806591	144807208	Distal Interger	-0745	411E-04	894E-03	Ptafr	33193	Hypo
chr8	95306002	95307253	Intron (NM_0(	-0567	412E-04	896E-03	Slc9a9	73097	Hypo
chr1	211785328	211785839	Intron (NM_0	-0816	413E-04	897E-03	Tle4	12023	Hypo
chr13	6526974	6527500	Distal Interger	-0914	413E-04	898E-03	Cntnap5a	-2434006	Hypo
chr16	46734716	46735139	Distal Interger	-0946	414E-04	899E-03	Tlr3	-87250	Hypo
chr5	146121861	146122257	Distal Interger	-1044	415E-04	901E-03	Rps6ka1	-3589	Hypo
chr1	184748185	184750030	Intron (NM_0(	-0505	417E-04	904E-03	Fgfr2	100596	Hypo

chr1	185422227	185422862	Distal Interger	-0733	417E-04	905E-03	Plekha1	-5186	Hypo
chr8	95962149	95967118	Distal Interger	-0349	417E-04	905E-03	Paqr9	-147131	Hypo
chr18	47458693	47459934	Distal Interger	-0647	418E-04	905E-03	Csnk1g3	159114	Hypo
chr2	139620884	139622707	Distal Interger	-0493	418E-04	905E-03	Dclk1	-10037	Hypo
chr8	114366844	114370146	Distal Interger	-0336	418E-04	905E-03	Dync1li1	-6621	Hypo
chr7	44035178	44036409	Intron (NM_0(	-0584	419E-04	907E-03	Syt1	321394	Hypo
chr8	106141851	106143452	Distal Interger	-0536	419E-04	907E-03	Atp2c1	-10537	Hypo
chr1	138151202	138152398	Distal Interger	-0648	419E-04	907E-03	Abhd17c	-25607	Hypo
chr9	32967392	32967715	Distal Interger	-0918	420E-04	907E-03	Phf3	168279	Hypo
chr17	63961166	63961415	Distal Interger	-1026	420E-04	907E-03	Pitrm1	-133853	Hypo
chr1	132583244	132583707	Distal Interger	-0839	420E-04	908E-03	Ntrk3	-79958	Hypo
chrX	5795827	5802798	Promoter (<=1	-0338	422E-04	911E-03	Ndp	0	Hypo
chr4	28521365	28524901	Promoter (2-3	-0339	423E-04	912E-03	Cldn12	-2591	Hypo
chr17	74441660	74445044	Distal Interger	-0364	423E-04	912E-03	Fam107b	104558	Hypo
chr1	15599321	15600934	Distal Interger	-0530	423E-04	912E-03	Pde7b	-106421	Hypo
chr9	41546642	41548247	Intron (NM_0(	-0549	423E-04	912E-03	Npas2	82812	Hypo
chr15	38469278	38473564	Exon (NM_05:	-0390	423E-04	912E-03	Msra	202932	Hypo
chr14	19071032	19072684	Intron (NM_0!	-0493	424E-04	914E-03	Slc4a4	111500	Hypo
chr11	1699961	1700253	Distal Interger	-1051	425E-04	914E-03	Epha3	-226061	Hypo
chr11	69164648	69165933	Distal Interger	-0665	426E-04	917E-03	Dlg1	-61845	Hypo
chr8	42749387	42750514	Intron (NM_0(	-0638	427E-04	918E-03	Tecta	29193	Hypo
chr7	29030070	29030672	Distal Interger	-0962	427E-04	918E-03	Tmcc3	-69503	Hypo
chr1	145767431	145768840	Intron (NM_0:	-0497	427E-04	918E-03	Dlg2	640256	Hypo
chr6	110705245	110710562	Distal Interger	-0394	428E-04	918E-03	Ston2	-28869	Hypo
chr5	163029061	163031950	Intron (NM_0(	-0396	429E-04	920E-03	Nphp4	40691	Hypo
chr8	113428559	113432550	Distal Interger	-0410	429E-04	920E-03	Pdcd6ip	214223	Hypo
chr14	99598149	99600655	Distal Interger	-0435	429E-04	920E-03	Fancl	-649290	Hypo
chr6	14036808	14045442	Intron (NM_0(	-0314	431E-04	923E-03	Tmem178a	18077	Hypo
chr15	38485189	38486669	Intron (NM_0!	-0677	432E-04	925E-03	Msra	189827	Hypo
chr7	44973732	44978002	Distal Interger	-0387	432E-04	925E-03	Nav3	414257	Hypo
chr1	13345148	13348549	Distal Interger	-0445	432E-04	926E-03	Hebp2	-143371	Hypo
chr6	67628072	67629052	Distal Interger	-0680	433E-04	928E-03	Prkd1	409645	Hypo
chr8	10317771	10318452	Distal Interger	-0742	434E-04	929E-03	Ccdc82	89982	Hypo

chr13	66236822	66238668	Distal Interger	-0476	434E-04	929E-03	Glul	211181	Hypo
chr1	80776759	80779024	Distal Interger	-0450	434E-04	929E-03	Gsk3a	46667	Hypo
chr1	231229161	231232380	Intron (NM_0(	-0414	435E-04	930E-03	Rps4x-ps1	18884	Hypo
chr15	82760283	82761641	Distal Interger	-0535	435E-04	930E-03	Spry2	-64959	Hypo
chr10	16553296	16562560	Distal Interger	-0315	435E-04	930E-03	Atp6v0e1	-50564	Hypo
chr6	48374225	48375615	Distal Interger	-0571	436E-04	930E-03	Gpr22	-56739	Hypo
chr13	94126852	94132023	Distal Interger	-0369	436E-04	931E-03	Capn2	68946	Hypo
chr9	59099240	59101177	Distal Interger	-0482	437E-04	932E-03	Tyw5	-120052	Hypo
chr6	69903648	69905656	Distal Interger	-0545	438E-04	935E-03	Arhgap5	-82554	Hypo
chr1	46002329	46003852	Intron (NM_0(	-0574	438E-04	935E-03	Ldhal6b	-3061	Hypo
chr5	24423472	24423964	Distal Interger	-0831	439E-04	936E-03	Dpy19l4	-12560	Hypo
chr1	114617688	114618380	Promoter (1-2	-0590	439E-04	936E-03	Mir344b-3	-1015	Hypo
chr8	36929725	36930403	Intron (NM_0(	-0635	439E-04	936E-03	Tmem218	5120	Hypo
chr1	114500393	114502005	Distal Interger	-0494	440E-04	937E-03	Mir344b-2	114668	Hypo
chr6	39870320	39872918	Distal Interger	-0456	440E-04	937E-03	C6h2orf50	-73280	Hypo
chr17	21967241	21968137	Intron (NM_2	-0653	441E-04	937E-03	Phactr1	71103	Hypo
chr4	155990809	155992902	Distal Interger	-0377	441E-04	937E-03	Slc2a3	-16689	Hypo
chr2	153993046	153994012	Distal Interger	-0718	441E-04	937E-03	Nmd3	-18970	Hypo
chr14	94267791	94273174	Distal Interger	-0339	441E-04	937E-03	Actr2	60541	Hypo
chr8	113523080	113531095	Distal Interger	-0332	441E-04	937E-03	Pdcd6ip	115678	Hypo
chr13	90804126	90807510	Intron (NM_0(	-0387	442E-04	937E-03	Smyd3	458699	Hypo
chr17	70864439	70864798	Distal Interger	-1004	442E-04	937E-03	Celf2	-544812	Hypo
chr10	31010133	31010718	Intron (NM_0(	-0753	442E-04	937E-03	Dppa1	7038	Hypo
chr16	73387615	73392117	Exon (NM_00:	-0355	442E-04	938E-03	Csmd1	1168850	Hypo
chr9	77476566	77476906	Distal Interger	-1000	443E-04	938E-03	Slc4a3	439550	Hypo
chr1	222349536	222350039	Intron (NM_0(	-0724	443E-04	938E-03	Tmem252	32554	Hypo
chr4	54149877	54150473	Intron (NM_0!	-0743	443E-04	938E-03	Gpr37	10454	Hypo
chr17	5805082	5812899	Exon (NM_01:	-0336	443E-04	938E-03	Ntrk2	56222	Hypo
chr13	42868101	42875320	Intron (NM_0(	-0332	444E-04	938E-03	Srgap2	91738	Hypo
chr14	96572473	96572950	Distal Interger	-0951	444E-04	938E-03	Tmem17	53970	Hypo
chr4	150552060	150552384	Distal Interger	-1151	444E-04	939E-03	Cxcl12	163734	Hypo
chr9	54164480	54165421	Distal Interger	-0683	445E-04	940E-03	Slc39a10	-745581	Hypo
chr7	57867906	57868450	Distal Interger	-0910	445E-04	940E-03	Rxylt1	-85289	Hypo

chr5	24027778	24031090	Distal Interger	-0446	445E-04	940E-03	Cfap418	31060	Hypo
chr4	113929234	113929760	Distal Interger	-0721	445E-04	940E-03	LOC10091137	533348	Hypo
chr2	103337672	103339097	Distal Interger	-0550	446E-04	941E-03	Agtr1b	-417440	Hypo
chr10	20449689	20450477	Distal Interger	-0734	447E-04	941E-03	Rars1	-154497	Hypo
chr11	69172573	69176059	Distal Interger	-0384	447E-04	941E-03	Dlg1	-69770	Hypo
chr1	143956285	143960449	Distal Interger	-0373	448E-04	943E-03	Eed	-61327	Hypo
chr1	14234402	14235662	Distal Interger	-0618	450E-04	945E-03	Ifngr1	-97603	Hypo
chr14	97059913	97062327	Distal Interger	-0344	450E-04	945E-03	Fam161a	50422	Hypo
chr4	133677499	133680058	Distal Interger	-0449	450E-04	945E-03	Ppp4r2	222944	Hypo
chr1	129124363	129133358	Intron (NM_0!	-0343	450E-04	946E-03	Sv2b	19121	Hypo
chr1	95159125	95160355	Promoter (<=:	-0513	451E-04	946E-03	Myh14	-289	Hypo
chr16	32437199	32438307	Intron (NM_0(	-0634	452E-04	948E-03	Galnt7	-140383	Hypo
chr4	130867212	130868267	Distal Interger	-0602	454E-04	951E-03	Mitf	457995	Hypo
chr9	75221512	75223042	Distal Interger	-0562	454E-04	951E-03	Pincl	-221752	Hypo
chr1	159832608	159832840	Distal Interger	-1106	455E-04	954E-03	Cavin3	-4470	Hypo
chr10	56598267	56598965	Distal Interger	-0849	456E-04	955E-03	Aipl1	65957	Hypo
chr1	235036702	235039633	Intron (NM_0	-0470	456E-04	955E-03	Ide	62390	Hypo
chr13	87244051	87244667	Intron (NM_0	-0733	457E-04	955E-03	Rgs7	163956	Hypo
chr16	21039221	21041052	Distal Interger	-0471	457E-04	956E-03	Ints10	-92117	Hypo
chr7	64513001	64513806	Intron (NM_0:	-0647	457E-04	956E-03	Cpq	248821	Hypo
chr1	13003646	13005862	Distal Interger	-0522	458E-04	957E-03	Ccdc28a	-130940	Hypo
chr8	119766422	119768928	Distal Interger	-0450	459E-04	958E-03	Cx3cr1	30503	Hypo
chr20	34772630	34773177	Distal Interger	-0788	460E-04	960E-03	Msl3l2	-700014	Hypo
chr1	147868440	147868933	Distal Interger	-0821	460E-04	960E-03	Fam181b	702012	Hypo
chr18	48558320	48559823	Distal Interger	-0478	460E-04	960E-03	Zfp608	-147855	Hypo
chr11	55136158	55137636	Exon (NM_00:	-0566	461E-04	961E-03	Tmprss7	7145	Hypo
chr4	176598879	176602005	Distal Interger	-0437	461E-04	961E-03	Etnk1	472699	Hypo
chr12	37754538	37755078	Distal Interger	-0818	461E-04	962E-03	Spring1	574060	Hypo
chr1	176113722	176115659	Distal Interger	-0555	463E-04	965E-03	Usp31	136714	Hypo
chr7	46608774	46611055	Intron (NM_0(	-0480	464E-04	966E-03	Osbpl8	11791	Hypo
chr2	118182277	118183904	Distal Interger	-0528	465E-04	968E-03	Dcun1d1	583414	Hypo
chr5	123992221	123993059	Distal Interger	-0652	465E-04	968E-03	Osbpl9	-30042	Hypo
chr3	28433914	28436327	Intron (NM_0(	-0459	466E-04	969E-03	Arhgap15	444281	Hypo

chr4	10277848	10282002	Promoter (<=1	-0321	466E-04	969E-03	Rheb	0	Hypo
chr16	65939586	65941310	Distal Interger	-0519	466E-04	969E-03	Hook3	-13042	Hypo
chr17	5570237	5586114	Intron (NM_0	-0293	467E-04	971E-03	Ntrk2	283007	Hypo
chr1	176161732	176163898	Distal Interger	-0547	469E-04	974E-03	Usp31	88475	Hypo
chr17	18436769	18437849	Distal Interger	-0650	470E-04	975E-03	Rbm24	-149350	Hypo
chr7	45855147	45856634	Distal Interger	-0562	472E-04	978E-03	E2f7	-293943	Hypo
chr1	33452324	33453267	Distal Interger	-0601	472E-04	979E-03	Med10	22457	Hypo
chr4	145430950	145431473	Intron (NM_0	-0897	473E-04	979E-03	Lmcd1	37797	Hypo
chr3	29493207	29495618	Distal Interger	-0444	473E-04	979E-03	Zeb2-as1	150129	Hypo
chr2	15301256	15315663	Distal Interger	-0316	474E-04	981E-03	Ccnh	-519170	Hypo
chr14	65895635	65898918	Distal Interger	-0509	475E-04	982E-03	Qdpr	225384	Hypo
chr4	80676669	80680817	Distal Interger	-0338	476E-04	983E-03	Snx10	63973	Hypo
chr5	123333808	123335320	Exon (NM_17	-0578	476E-04	983E-03	Orc1	5472	Hypo
chr11	9203282	9204335	Distal Interger	-0633	476E-04	983E-03	Gbe1	468420	Hypo
chr16	66990682	66992412	Distal Interger	-0462	477E-04	986E-03	Htra4	-9193	Hypo
chr15	54686201	54687014	Distal Interger	-0764	478E-04	986E-03	Rgcc	-10881	Hypo
chr2	164964197	164968366	Exon (NM_00	-0336	478E-04	986E-03	Tmem144	24002	Hypo
chr2	184039883	184041144	Distal Interger	-0378	479E-04	986E-03	Hjv	-24826	Hypo
chr1	91558839	91561782	Distal Interger	-0437	479E-04	986E-03	LOC365238	-475449	Hypo
chr12	8621954	8633787	Distal Interger	-0286	479E-04	987E-03	Gpr12	98001	Hypo
chr17	18129649	18136894	Intron (NM_0	-0316	480E-04	989E-03	Cap2	66451	Hypo
chr4	42925032	42925593	Distal Interger	-0794	480E-04	989E-03	Ppp1r3a	54596	Hypo
chr16	44477073	44477900	Distal Interger	-0729	480E-04	989E-03	Cldn24	-51601	Hypo
chr4	150518939	150520243	Distal Interger	-0606	481E-04	989E-03	Cxcl12	130613	Hypo
chr17	992383	993803	Intron (NM_0	-0581	482E-04	991E-03	Slc35d2	4200	Hypo
chr1	147147786	147149369	Distal Interger	-0557	482E-04	992E-03	Fam181b	-17059	Hypo
chr4	16772414	16772821	Distal Interger	-0845	483E-04	992E-03	Gnai1	-41194	Hypo
chr16	13013255	13014083	Distal Interger	-0669	483E-04	992E-03	Ghitm	-115368	Hypo
chr10	80327797	80328725	Promoter (<=1	-0358	483E-04	992E-03	Slc35b1	0	Hypo
chr6	105082307	105084990	Distal Interger	-0415	484E-04	993E-03	Fos	-36180	Hypo
chr2	57886636	57887745	Distal Interger	-0608	484E-04	993E-03	Slc1a3	-56031	Hypo
chr19	48732093	48732679	Distal Interger	-0888	485E-04	994E-03	Cox4i1	10413	Hypo
chr4	53454587	53455363	Distal Interger	-0678	488E-04	100E-02	Tmem229a	-9260	Hypo

chr12	6059560	6065350	Distal Interger	-0344	489E-04	100E-02	Rpl35a	7372	Hypo
chr6	57651263	57652191	Distal Interger	-0587	489E-04	100E-02	Ifrd1	-362357	Hypo
chr17	70899303	70902407	Distal Interger	-0419	489E-04	100E-02	Celf2	-507203	Hypo
chr8	52909020	52910887	Distal Interger	-0529	490E-04	100E-02	LOC10012536	158125	Hypo
chr5	62358313	62367317	Promoter (<=1	-0397	491E-04	100E-02	Nr4a3	0	Hypo
chr12	10565180	10566476	Distal Interger	-0544	492E-04	101E-02	Ocm	-20480	Hypo
chr2	116126197	116130862	Distal Interger	-0360	492E-04	101E-02	Pex5l	-218215	Hypo
chr3	116727133	116727768	Intron (NM_0	-0823	493E-04	101E-02	Sirpb3	35478	Hypo
chr20	8641866	8645040	Promoter (2-3	-0330	493E-04	101E-02	Btbd9	-2022	Hypo
chr1	257051096	257051714	Distal Interger	-0820	494E-04	101E-02	Gfra1	499069	Hypo
chr7	32696191	32697307	Distal Interger	-0566	495E-04	101E-02	Ccer1	210998	Hypo
chr12	12416127	12417245	Distal Interger	-0614	495E-04	101E-02	Foxk1	-241038	Hypo
chr1	172902406	172907698	Promoter (<=1	-0317	495E-04	101E-02	Tmc7	0	Hypo
chr19	12187750	12189120	Distal Interger	-0550	495E-04	101E-02	Large1	-138820	Hypo
chr19	24884647	24885891	Intron (NM_0	-0616	495E-04	101E-02	Tbc1d9	57238	Hypo
chr1	180350119	180351270	Intron (NM_0	-0710	496E-04	101E-02	Katnip	50182	Hypo
chr3	112973409	112976267	Promoter (<=1	-0417	496E-04	101E-02	Shc4	0	Hypo
chr3	126787168	126788507	Distal Interger	-0503	496E-04	101E-02	Sptlc3	-59371	Hypo
chr6	2193462	2194123	Distal Interger	-0664	497E-04	101E-02	Nrxn1	-987167	Hypo
chr2	35614582	35615843	Intron (NM_0	-0627	498E-04	101E-02	Adamts6	193072	Hypo
chr7	9093364	9096933	Promoter (<=1	-0338	498E-04	101E-02	Csnk1g2	0	Hypo
chr13	99402421	99403469	Intron (NM_2	-0627	499E-04	101E-02	Esrrg	178050	Hypo
chr11	65007792	65009943	Intron (NM_0	-0510	500E-04	101E-02	Slc49a4	12113	Hypo
chr11	57069546	57070756	Distal Interger	-0528	500E-04	102E-02	Mir568	-15473	Hypo
chr1	14321633	14324920	Distal Interger	-0409	502E-04	102E-02	Ifngr1	-8345	Hypo
chr4	25935871	25937175	Distal Interger	-0702	502E-04	102E-02	Sri	50571	Hypo
chr6	8261537	8262322	Intron (NM_0	-0724	503E-04	102E-02	RGD1562146	84774	Hypo
chr18	67586618	67590023	Distal Interger	-0385	503E-04	102E-02	Mapk4	-25029	Hypo
chr1	50332542	50334523	Distal Interger	-0462	504E-04	102E-02	Cahm	52436	Hypo
chr11	61572712	61573327	Distal Interger	-0705	505E-04	102E-02	Igsf11	295021	Hypo
chr1	227646753	227649224	Distal Interger	-0422	506E-04	102E-02	Ranbp6	-69838	Hypo
chrX	28154974	28163380	Distal Interger	-0333	506E-04	102E-02	Gpm6b	-53386	Hypo
chr8	73608620	73610871	Intron (NM_0	-0470	507E-04	103E-02	Pygo1	-55596	Hypo

chr2	24059808	24060498	Distal Interger	-0715	508E-04	103E-02	Thbs4	-34519	Hypo
chr2	164440036	164441008	Distal Interger	-0625	508E-04	103E-02	Spmip2	-49244	Hypo
chr14	99618686	99620893	Distal Interger	-0430	508E-04	103E-02	Fancl	-629052	Hypo
chr17	61636386	61638004	Promoter (<=1	-0361	509E-04	103E-02	Wdr37	0	Hypo
chr3	112933557	112936586	Intron (NM_0(	-0435	509E-04	103E-02	Eid1	10596	Hypo
chr10	103695568	103698397	Distal Interger	-0456	510E-04	103E-02	Engase	-8772	Hypo
chr3	80232029	80237544	Distal Interger	-0299	511E-04	103E-02	Mir129-2	-29889	Hypo
chr17	46487422	46488105	Intron (NM_0(	-0717	511E-04	103E-02	Pou6f2	15241	Hypo
chr13	70760629	70763062	Intron (NM_0(	-0465	512E-04	103E-02	Mir488	77565	Hypo
chr11	31280726	31281161	Distal Interger	-0938	512E-04	103E-02	Slc5a3	-32686	Hypo
chr6	48164412	48165123	Distal Interger	-0814	512E-04	103E-02	Slc26a4	-18709	Hypo
chr9	19467454	19468300	Distal Interger	-0671	513E-04	103E-02	Cenpq	-488764	Hypo
chr1	93768008	93770419	Promoter (<=1	-0431	513E-04	103E-02	Siglec5	0	Hypo
chr7	100692762	100696204	Distal Interger	-0369	515E-04	104E-02	Khdrbs3	-141730	Hypo
chr8	112775766	112776769	Distal Interger	-0641	516E-04	104E-02	Arpp21	-622735	Hypo
chr20	22325783	22326508	Distal Interger	-0714	516E-04	104E-02	Reep3	831915	Hypo
chr8	42570517	42572727	Distal Interger	-0429	518E-04	104E-02	Sorl1	-66289	Hypo
chr6	105016474	105019927	Intron (NM_0!	-0372	518E-04	104E-02	Tmed10	6778	Hypo
chr4	169382279	169383067	Promoter (2-3	-0686	520E-04	104E-02	Atf7ip	-2805	Hypo
chr3	79729699	79731360	Intron (NM_0(	-0583	520E-04	104E-02	Ext2	66699	Hypo
chr15	35072727	35073343	Distal Interger	-0923	520E-04	104E-02	Tnfrsf19	85129	Hypo
chr20	43926886	43930398	Distal Interger	-0332	521E-04	104E-02	Cdk19	156477	Hypo
chr5	111242317	111246012	Promoter (<=1	-0383	521E-04	104E-02	Cyp2j3	0	Hypo
chr8	108649931	108654071	Exon (NM_001	-0461	522E-04	104E-02	Traip	8071	Hypo
chr2	15954720	15955053	Distal Interger	-0840	523E-04	105E-02	Rasa1	-14439	Hypo
chr17	63889320	63891042	Distal Interger	-0460	523E-04	105E-02	Pitrm1	-62007	Hypo
chr5	16643059	16644109	Intron (NM_0(	-0779	525E-04	105E-02	Lyn	3547	Hypo
chr6	121585937	121587839	Distal Interger	-0505	525E-04	105E-02	Lgmn	-15917	Hypo
chr15	46684951	46686354	Distal Interger	-0525	527E-04	105E-02	Gfra2	742500	Hypo
chr13	54820215	54821058	Distal Interger	-0631	527E-04	105E-02	B3galt2	-568348	Hypo
chr18	59076263	59080798	Distal Interger	-0330	527E-04	105E-02	Zfp532	-15895	Hypo
chr8	62352442	62353546	Distal Interger	-0580	528E-04	105E-02	Rplp1	41795	Hypo
chr3	42730041	42732322	Distal Interger	-0466	528E-04	105E-02	Cytip	-4048	Hypo

chr4	27997235	27998495	Distal Interger	-0665	528E-04	105E-02	Steap1	-278477	Hypo
chr15	68556382	68556992	Distal Interger	-0645	529E-04	106E-02	Pcdh9	1678673	Hypo
chr7	69824358	69825142	Distal Interger	-0640	530E-04	106E-02	Atp6v1c1	-9441	Hypo
chr16	28522912	28523627	Distal Interger	-0790	533E-04	106E-02	Cbr4	122064	Hypo
chr14	2252431	2260920	Intron (NM_0(	-0329	533E-04	106E-02	C14h1orf146	9862	Hypo
chr8	57961502	57962073	Distal Interger	-0835	533E-04	106E-02	Scamp2	-3572	Hypo
chr12	18581595	18582159	Distal Interger	-0890	535E-04	106E-02	Vom2r64	162186	Hypo
chr5	39398295	39399604	Intron (NM_0!	-0524	536E-04	106E-02	Fut9	165071	Hypo
chr8	110218151	110241330	Promoter (<=:	-0290	536E-04	106E-02	Cspg5	0	Hypo
chr8	71546811	71554742	Intron (NM_0	-0347	536E-04	106E-02	Lipc	80722	Hypo
chr6	4853765	4855054	Distal Interger	-0609	538E-04	107E-02	Fshr	-343771	Hypo
chr1	13029304	13030943	Distal Interger	-0563	538E-04	107E-02	Ccdc28a	-156598	Hypo
chr14	79573619	79576890	Distal Interger	-0496	542E-04	107E-02	Uqcr10	-5211	Hypo
chr1	254400616	254401535	Intron (NM_0:	-0497	543E-04	108E-02	Vti1a	44076	Hypo
chr2	170371451	170375238	Distal Interger	-0399	544E-04	108E-02	Dear	-71269	Hypo
chr6	98462897	98463864	Distal Interger	-0699	545E-04	108E-02	Zfyve26	-367417	Hypo
chr18	67414475	67416839	Distal Interger	-0467	546E-04	108E-02	Me2	-13488	Hypo
chr17	4790248	4794135	Distal Interger	-0389	547E-04	108E-02	Isca1	-111156	Hypo
chr7	109628354	109630681	Intron (NM_0!	-0468	548E-04	108E-02	Cacng2	66546	Hypo
chr7	19010378	19013032	Distal Interger	-0510	548E-04	108E-02	Polr3b	129418	Hypo
chr3	116640770	116643903	Distal Interger	-0366	548E-04	108E-02	Il1b	-57384	Hypo
chr5	116992792	116997986	Promoter (<=:	-0351	549E-04	108E-02	Pde4b	0	Hypo
chr3	118692098	118694785	Distal Interger	-0497	549E-04	108E-02	Smox	-56838	Hypo
chr17	64268544	64269339	Distal Interger	-0731	553E-04	109E-02	Klf6	279112	Hypo
chr2	115833420	115834596	Intron (NM_1:	-0610	553E-04	109E-02	Pex5l	73386	Hypo
chr12	20849149	20850995	Intron (NM_0(	-0463	554E-04	109E-02	Srrm3	27510	Hypo
chr2	41938814	41939725	Distal Interger	-0679	554E-04	109E-02	Gapt	-8977	Hypo
chr17	82170403	82171422	Distal Interger	-0675	555E-04	109E-02	Ptf1a	119122	Hypo
chr1	8185393	8186265	Intron (NM_0(	-0712	555E-04	109E-02	Aig1	82687	Hypo
chr6	14470614	14470951	Distal Interger	-1003	555E-04	109E-02	Sos1	-62919	Hypo
chr14	5423745	5425604	Intron (NM_0:	-0363	556E-04	109E-02	Mepe	6582	Hypo
chr13	98857704	98859514	Distal Interger	-0481	556E-04	109E-02	Gpatch2	72687	Hypo
chr13	79495347	79495894	Distal Interger	-0838	556E-04	109E-02	Aldh9a1	-9844	Hypo

chr2	135827986	135829008	Intron (NM_0(	-0638	557E-04	109E-02	Mgst2	134430	Hypo
chr16	32436463	32437071	Intron (NM_0(	-0697	557E-04	109E-02	Galnt7	-141619	Hypo
chr2	75134442	75134921	Distal Interger	-0811	561E-04	110E-02	Basp1	728880	Hypo
chr16	52748452	52749277	Exon (NM_00:	-0700	562E-04	110E-02	Msr1	14406	Hypo
chr7	45705152	45707165	Distal Interger	-0471	562E-04	110E-02	Nav3	-312893	Hypo
chr15	87553128	87554017	Distal Interger	-0878	563E-04	110E-02	Slitrk6	16108	Hypo
chr3	117880659	117882813	Promoter (<=:	-0331	564E-04	110E-02	Ddrgk1	0	Hypo
chr6	49740912	49741852	Distal Interger	-0649	565E-04	111E-02	Sypl1	175758	Hypo
chr17	73825583	73827584	Intron (NM_0(	-0458	566E-04	111E-02	Frmd4a	119060	Hypo
chr2	12683101	12685295	Distal Interger	-0485	567E-04	111E-02	Cetn3	593684	Hypo
chr4	47274730	47275844	Distal Interger	-0582	567E-04	111E-02	Lsm8	-30415	Hypo
chr4	59192883	59193402	Intron (NM_0(	-0786	567E-04	111E-02	Cpa4	4599	Hypo
chr5	159675790	159680070	Exon (NM_05	-0350	567E-04	111E-02	Kif1b	62708	Hypo
chr5	130249061	130251240	Intron (NM_1:	-0415	567E-04	111E-02	Toe1	22810	Hypo
chr3	166626216	166627048	Distal Interger	-0753	567E-04	111E-02	Psma7	516578	Hypo
chr13	50965314	50965890	Intron (NM_0(	-0830	568E-04	111E-02	Crb1	23371	Hypo
chr2	108833133	108834120	Intron (NM_0:	-0613	569E-04	111E-02	Nlgn1	167811	Hypo
chr2	170611413	170611884	Distal Interger	-0951	569E-04	111E-02	Dear	-311231	Hypo
chr2	4421853	4425111	Exon (NM_01	-0409	570E-04	111E-02	Pcsk1	26310	Hypo
chr10	96495769	96497884	Distal Interger	-0418	570E-04	111E-02	Kcnj2	429494	Hypo
chr7	62294442	62296331	Distal Interger	-0457	571E-04	111E-02	Atp23	433492	Hypo
chr2	100139437	100142537	Distal Interger	-0379	571E-04	111E-02	Mir124-2	4571	Hypo
chr20	24348715	24349989	Distal Interger	-0595	571E-04	111E-02	Lrrtm3	-165638	Hypo
chr2	232346540	232349764	Distal Interger	-0427	572E-04	111E-02	Pkn2	-447587	Hypo
chr11	13915572	13916027	Distal Interger	-0822	574E-04	112E-02	Lipi	312965	Hypo
chr11	75754009	75754269	Distal Interger	-1032	574E-04	112E-02	Mir28	221339	Hypo
chr19	52642484	52645487	Exon (NM_00:	-0346	576E-04	112E-02	C19h1orf198	-13048	Hypo
chr3	125974912	125975733	Distal Interger	-0701	577E-04	112E-02	Btbd3	362219	Hypo
chr14	86236446	86239982	Distal Interger	-0458	577E-04	112E-02	Ikzf1	-15083	Hypo
chr12	16318847	16320664	Promoter (2-3	-0564	578E-04	112E-02	Il3ra	2820	Hypo
chr17	36069432	36071615	Distal Interger	-0404	579E-04	112E-02	Prl5a2	387439	Hypo
chr17	85085361	85088123	Distal Interger	-0400	581E-04	113E-02	Abi1	91630	Hypo
chr7	25199809	25204775	Intron (NM_0(	-0328	581E-04	113E-02	Apaf1	374765	Hypo

chr13	39566084	39567139	Distal Interger	-0584	582E-04	113E-02	R3hdm1	-28709	Hypo
chr13	73565647	73566811	Distal Interger	-0591	584E-04	113E-02	Prdx6	-26352	Hypo
chr2	136693198	136699097	Distal Interger	-0344	584E-04	113E-02	Foxo1	381030	Hypo
chr2	216312878	216313350	Distal Interger	-0921	584E-04	113E-02	Ap1ar	-4135	Hypo
chr7	92943215	92943772	Distal Interger	-0706	584E-04	113E-02	A1bg	-445118	Hypo
chr13	65441739	65443859	Intron (NM_0	-0486	584E-04	113E-02	Lamc1	57633	Hypo
chr4	69406987	69407581	Distal Interger	-0770	585E-04	113E-02	Or9a4	5381	Hypo
chr17	16390200	16392381	Promoter (<=1	-0425	585E-04	113E-02	Id4	0	Hypo
chr2	42168416	42169810	Distal Interger	-0639	585E-04	113E-02	Plk2	199232	Hypo
chr5	76271325	76272545	Distal Interger	-0612	586E-04	113E-02	Rgs3	192833	Hypo
chrX	12302841	12306788	Promoter (<=1	-0309	586E-04	113E-02	Tspan7	0	Hypo
chr16	19359174	19361081	Exon (NM_00	-0530	587E-04	113E-02	Tm6sf2	-7743	Hypo
chr8	42566236	42568043	Distal Interger	-0459	588E-04	113E-02	Sorl1	-62008	Hypo
chr5	129530736	129532770	Promoter (<=1	-0339	588E-04	113E-02	Nsun4	0	Hypo
chr1	129114592	129120597	Intron (NM_0	-0368	588E-04	113E-02	Sv2b	31882	Hypo
chr1	176805713	176806344	Distal Interger	-0735	590E-04	114E-02	Prkcb	-26341	Hypo
chr10	20452617	20455651	Distal Interger	-0387	593E-04	114E-02	Rars1	-157425	Hypo
chr5	39889646	39890302	Distal Interger	-0761	594E-04	114E-02	Manea	321842	Hypo
chr12	14428386	14431600	Intron (NM_0	-0439	594E-04	114E-02	Mad1l1	107494	Hypo
chr14	7168575	7169528	Distal Interger	-0657	594E-04	114E-02	Arhgap24	-319080	Hypo
chr13	79673067	79676034	Distal Interger	-0408	595E-04	114E-02	Lrrc52	-52427	Hypo
chr1	142667819	142669229	Distal Interger	-0811	596E-04	114E-02	Rab38	485253	Hypo
chr19	55047514	55052103	Promoter (<=1	-0372	596E-04	114E-02	Hmgb1-ps33	-369	Hypo
chr4	10100844	10103332	Distal Interger	-0441	596E-04	114E-02	Prkag2	-102356	Hypo
chr2	110944429	110946138	Exon (NM_03	-0555	596E-04	114E-02	Pld1	50806	Hypo
chr8	28155432	28157980	Distal Interger	-0419	597E-04	114E-02	Ntm	-351204	Hypo
chr7	89540348	89543282	Intron (NM_0	-0405	597E-04	114E-02	Fam83a	17522	Hypo
chr4	46238331	46241273	Intron (NM_0	-0427	598E-04	115E-02	ST7	196013	Hypo
chr4	41733178	41734612	Distal Interger	-0641	599E-04	115E-02	Tmem168	124151	Hypo
chr3	147431712	147434848	Exon (NM_00	-0407	599E-04	115E-02	Fam83d	9771	Hypo
chr20	19354348	19357011	Intron (NM_0	-0440	601E-04	115E-02	Rhobtb1	46001	Hypo
chr7	86306101	86313826	Distal Interger	-0364	601E-04	115E-02	Enpp2	-23627	Hypo
chr1	138495385	138498193	Distal Interger	-0430	603E-04	115E-02	Fah	73312	Hypo

chr7	93196943	93197937	Distal Interger	-0581	603E-04	115E-02		368281	Hypo
chr4	153274974	153278588	Distal Interger	-0379	603E-04	115E-02	Wnk1	-21069	Hypo
chr6	57648704	57649845	Distal Interger	-0626	604E-04	115E-02	Ifrd1	-359798	Hypo
chr1	236662199	236665766	Exon (NM_00:	-0420	605E-04	115E-02	Hells	-35982	Hypo
chr1	182803432	182804063	Distal Interger	-0774	606E-04	116E-02	Cox6a2	-14158	Hypo
chr13	68291600	68292399	Intron (NM_0(	-0796	606E-04	116E-02	Fam163a	48650	Hypo
chr6	3279975	3280449	Intron (NM_0:	-0806	607E-04	116E-02	Nrxn1	98685	Hypo
chr1	34269000	34271170	Distal Interger	-0464	610E-04	116E-02	Adcy2	-104741	Hypo
chr7	129124080	129126271	Promoter (1-2	-0588	610E-04	116E-02	Col2a1	1275	Hypo
chr2	143524585	143526042	Distal Interger	-0545	611E-04	116E-02	P2ry12	-39173	Hypo
chr6	53081622	53083780	Distal Interger	-0469	613E-04	117E-02	Sostdc1	30286	Hypo
chr16	37337011	37338436	Distal Interger	-0626	618E-04	117E-02	Spcs3	31653	Hypo
chr8	121741487	121742596	Distal Interger	-0652	619E-04	117E-02	Snrk	-50706	Hypo
chr14	91382749	91383938	Promoter (1-2	-0580	619E-04	117E-02	Fbxo48	1892	Hypo
chr14	97664081	97664583	Distal Interger	-0799	619E-04	117E-02	Pus10	42690	Hypo
chr13	106295423	106297085	Exon (NM_00:	-0498	620E-04	118E-02	Plxna2	132320	Hypo
chr17	21957106	21959502	Intron (NM_2	-0434	622E-04	118E-02	Phactr1	79738	Hypo
chr6	82235007	82235549	Distal Interger	-0807	622E-04	118E-02	Fscb	295092	Hypo
chr17	30729354	30732622	Distal Interger	-0338	623E-04	118E-02	Psmg4	-11330	Hypo
chr2	173660118	173696122	Promoter (<=:	-0277	624E-04	118E-02	Mir9-1	0	Hypo
chr3	56322686	56324809	Intron (NM_0(	-0515	625E-04	118E-02	Dlx1	-31381	Hypo
chr1	236036928	236040910	Promoter (2-3	-0316	625E-04	118E-02	Lgi1	-2466	Hypo
chr4	21382890	21383280	Intron (NM_0	-0886	625E-04	118E-02	Sema3a	109994	Hypo
chr8	28356844	28367219	Distal Interger	-0296	626E-04	118E-02	Snx19	-463310	Hypo
chr4	53958414	53960481	Distal Interger	-0500	626E-04	118E-02	Gpr37	200446	Hypo
chr14	40070729	40073601	Intron (NM_0(	-0420	627E-04	118E-02	Grxcr1	52970	Hypo
chrX	10151020	10153164	Downstream (	-0525	627E-04	118E-02	Mpc1l	-3875	Hypo
chr2	192772789	192773266	Distal Interger	-0827	628E-04	119E-02	Kcnd3	-164684	Hypo
chr13	80847630	80848574	Distal Interger	-0629	629E-04	119E-02	Pbx1	-259464	Hypo
chr7	93198082	93199065	Distal Interger	-0564	629E-04	119E-02		367153	Hypo
chr6	53234588	53237008	Intron (NM_0(	-0468	630E-04	119E-02	Crppa	113150	Hypo
chr11	37004414	37005071	Distal Interger	-0701	630E-04	119E-02	Tmprss2	-30699	Hypo
chr1	129244250	129245686	Distal Interger	-0545	631E-04	119E-02	Akap13	-68716	Hypo

chr8	119947448	119950615	Distal Interger	-0434	632E-04	119E-02	Mobp	77820	Hypo
chr16	51353503	51355387	Intron (NM_0(	-0434	632E-04	119E-02	Pdgfrl	52463	Hypo
chr7	57880253	57881410	Distal Interger	-0774	634E-04	119E-02	Rxylt1	-97636	Hypo
chr10	26299365	26299833	Distal Interger	-0894	634E-04	119E-02	Gabrg2	163847	Hypo
chr10	70523921	70524994	Intron (NM_0(	-0571	635E-04	119E-02	Tbx2	-155233	Hypo
chr3	7172861	7174317	3' UTR	-0565	635E-04	119E-02	Psd4	35706	Hypo
chr10	46791968	46796172	Intron (NM_0(	-0356	635E-04	119E-02	Adora2b	-144225	Hypo
chr3	150839844	150844278	Intron (NM_0(	-0358	635E-04	119E-02	Ptprt	144385	Hypo
chr19	20846059	20848034	Distal Interger	-0421	636E-04	119E-02	Abcc12	296654	Hypo
chr4	19034565	19035993	Intron (NM_0	-0567	636E-04	119E-02	Hgf	-289262	Hypo
chr3	145173681	145177585	Promoter (<=1	-0418	636E-04	119E-02	Dlgap4	0	Hypo
chr17	74236852	74240860	Distal Interger	-0378	637E-04	119E-02	Frmd4a	-290208	Hypo
chr16	67976120	67976949	Intron (NM_0(	-0762	637E-04	119E-02	Tcim	380358	Hypo
chr11	29203148	29203555	Distal Interger	-0857	637E-04	119E-02	Tiam1	-43240	Hypo
chrX	58611927	58613370	Distal Interger	-0458	638E-04	120E-02	Pdk3	-58370	Hypo
chr6	19078951	19079465	Distal Interger	-0803	639E-04	120E-02	Fam98a	-794617	Hypo
chr2	210613445	210622066	Exon (NM_001	-0312	640E-04	120E-02	Bcar3	88185	Hypo
chr9	76965195	76969522	Promoter (<=1	-0344	641E-04	120E-02	Chpf	0	Hypo
chr2	169752429	169754609	Distal Interger	-0452	641E-04	120E-02	Fhdc1	73287	Hypo
chr6	82234421	82234790	Distal Interger	-0871	642E-04	120E-02	Fscb	295851	Hypo
chr14	43764062	43765029	Distal Interger	-0548	643E-04	120E-02	Mir328b	100994	Hypo
chr10	105007405	105010534	Intron (NM_0(	-0369	644E-04	120E-02	Rptor	128918	Hypo
chr17	72639235	72640729	Intron (NM_0(	-0541	644E-04	120E-02	Camk1d	57256	Hypo
chr18	54557153	54558656	Distal Interger	-0585	645E-04	120E-02	Csf1r	-5334	Hypo
chr2	117523162	117531551	Distal Interger	-0324	645E-04	120E-02	Sox2	-5378	Hypo
chr3	136147490	136150499	Exon (NM_001	-0392	646E-04	121E-02	Gzf1	28377	Hypo
chr7	45737623	45742352	Distal Interger	-0388	646E-04	121E-02	Nav3	-345364	Hypo
chr13	22774191	22776671	Intron (NM_0	-0360	647E-04	121E-02	Bcl2	75567	Hypo
chr2	70995230	70995902	Distal Interger	-0695	647E-04	121E-02	Cdh18	-2349103	Hypo
chr12	32267448	32276633	Promoter (<=1	-0302	648E-04	121E-02	Mtrfr	0	Hypo
chr17	70097588	70098016	Distal Interger	-0857	648E-04	121E-02	Celf2	-1311594	Hypo
chr14	33939411	33946729	Intron (NM_0(	-0300	649E-04	121E-02	Rasl11b	72417	Hypo
chr1	41929850	41930875	Distal Interger	-0641	649E-04	121E-02	Myct1	-87262	Hypo

chr8	20709666	20711547	Distal Interger	-0353	649E-04	121E-02	Pigy	20045	Hypo
chr2	91656820	91657320	Distal Interger	-0713	649E-04	121E-02	Pmp2	45486	Hypo
chr4	178274836	178276493	Distal Interger	-0420	649E-04	121E-02	Kras	-59502	Hypo
chr7	53099074	53100343	Distal Interger	-0601	650E-04	121E-02	Cpsf6	-110974	Hypo
chr7	85423817	85425219	Distal Interger	-0489	651E-04	121E-02	Tnfrsf11b	169307	Hypo
chr6	108014974	108019126	Intron (NM_0!	-0359	651E-04	121E-02	Nrxn3	372990	Hypo
chr3	96166320	96170152	Promoter (<=1	-0340	651E-04	121E-02	Bdnf	0	Hypo
chr14	74811464	74812887	Promoter (<=1	-0536	652E-04	121E-02	Afap1	-345	Hypo
chr5	20021573	20027418	Exon (NM_001	-0338	653E-04	121E-02	Tox	143854	Hypo
chr14	30662178	30664851	Distal Interger	-0375	653E-04	121E-02	Igfbp7	-72563	Hypo
chr5	61318654	61320914	Exon (NM_001	-0459	654E-04	121E-02	Anks6	29682	Hypo
chr1	218035639	218037215	Distal Interger	-0570	655E-04	121E-02	Aldh1a1	-73870	Hypo
chr8	66056965	66057976	Distal Interger	-0653	658E-04	122E-02	Pif1	-53748	Hypo
chr5	9453509	9456243	Distal Interger	-0411	658E-04	122E-02	Sgk3	-38033	Hypo
chr12	23300507	23301374	Distal Interger	-0656	659E-04	122E-02	Mir6322	478745	Hypo
chr2	101534821	101538580	Distal Interger	-0416	659E-04	122E-02	Armc1	113600	Hypo
chr2	203044671	203046649	Distal Interger	-0500	659E-04	122E-02	Olfm3	134078	Hypo
chr1	176971575	176973445	Intron (NM_0	-0506	660E-04	122E-02	Prkcb	138890	Hypo
chr1	115242358	115244364	Promoter (<=1	-0461	660E-04	122E-02	Mir344a-2	0	Hypo
chr1	9464520	9465101	Distal Interger	-0837	661E-04	122E-02	Nmbr	329631	Hypo
chr7	44965847	44969297	Distal Interger	-0409	662E-04	122E-02	Nav3	422962	Hypo
chr16	51688532	51691439	Exon (NM_001	-0367	663E-04	122E-02	Mtmr7	47249	Hypo
chr9	92274018	92275304	Distal Interger	-0691	663E-04	122E-02	Twist2	-99436	Hypo
chr7	45390386	45393586	Promoter (<=1	-0393	663E-04	122E-02	Nav3	0	Hypo
chr6	70895793	70896777	Distal Interger	-0707	664E-04	122E-02	Akap6	711618	Hypo
chr8	36928106	36929628	Intron (NM_0(	-0461	664E-04	122E-02	Tmem218	3501	Hypo
chr3	62184545	62187013	Intron (NM_0(	-0396	668E-04	123E-02	Sestd1	11997	Hypo
chr10	21285377	21286205	Intron (NM_01	-0713	669E-04	123E-02	Tenm2	330798	Hypo
chr12	22535228	22537334	Distal Interger	-0343	672E-04	124E-02	Rcc1l	-6400	Hypo
chr2	80782215	80782623	Distal Interger	-0884	673E-04	124E-02	Ctnnd2	-547031	Hypo
chr16	75413361	75416138	Intron (NM_0!	-0416	673E-04	124E-02	Dlgap2	80242	Hypo
chr19	15619383	15621232	Intron (NM_0(	-0471	673E-04	124E-02	Fto	70824	Hypo
chr5	4951377	4951982	Distal Interger	-0760	675E-04	124E-02	Msc	400041	Hypo

chr16	81002231	81002814	Distal Interger	-0799	677E-04	124E-02	Efnb2	218814	Hypo
chr7	85156638	85157759	Distal Interger	-0603	678E-04	124E-02	Samd12	-92581	Hypo
chr16	65803769	65804503	Distal Interger	-0774	679E-04	125E-02	Thap1	105439	Hypo
chr2	75697156	75699795	Distal Interger	-0376	679E-04	125E-02	Basp1	164006	Hypo
chr2	54066287	54068370	Distal Interger	-0431	679E-04	125E-02	Mroh2b	59815	Hypo
chr17	70948449	70957391	Distal Interger	-0315	681E-04	125E-02	Celf2	-452219	Hypo
chr16	46662853	46664639	Distal Interger	-0565	681E-04	125E-02	Sorbs2	-36405	Hypo
chr13	84330707	84331344	Distal Interger	-0736	682E-04	125E-02	Cd84	-15398	Hypo
chr3	81520551	81520902	Distal Interger	-1044	682E-04	125E-02	Lrrc4c	-784342	Hypo
chr19	11291429	11292290	Distal Interger	-0659	684E-04	125E-02	Gnao1	-98936	Hypo
chr1	168485450	168487126	Distal Interger	-0466	686E-04	125E-02	Psma1	-32010	Hypo
chr15	38401052	38407501	Intron (NM_0!	-0309	686E-04	125E-02	C15h8orf74	-175315	Hypo
chr2	62392108	62394340	Distal Interger	-0386	687E-04	126E-02	Cdh6	-166880	Hypo
chr8	40718275	40721941	Intron (NM_0(	-0346	688E-04	126E-02	Scn3b	87820	Hypo
chrX	11605437	11606424	Distal Interger	-0679	689E-04	126E-02	Mid1ip1	456751	Hypo
chr1	249342280	249344502	Intron (NM_0(	-0451	690E-04	126E-02	Sorcs1	249777	Hypo
chr3	146472233	146474441	Exon (NM_001	-0495	690E-04	126E-02	Ctnnbl1	84293	Hypo
chr3	62884635	62886614	Distal Interger	-0413	691E-04	126E-02	Zfp385b	-257192	Hypo
chr3	162114472	162114934	Distal Interger	-0916	691E-04	126E-02	Pmepa1	-94678	Hypo
chr10	87703716	87706261	Distal Interger	-0430	691E-04	126E-02	Adam11	-18546	Hypo
chr2	183567838	183570214	Intron (NM_1	-0407	691E-04	126E-02	Plekho1	-15466	Hypo
chr12	13784981	13785675	Distal Interger	-0706	691E-04	126E-02	Gna12	-20023	Hypo
chr3	105637791	105639801	Promoter (<=1	-0481	693E-04	126E-02	Pak6	0	Hypo
chr17	69942362	69944581	Distal Interger	-0383	693E-04	126E-02	Gata3	1297449	Hypo
chr20	15994092	15996208	Distal Interger	-0512	694E-04	126E-02	Zwint	-40128	Hypo
chr16	51370747	51372688	Intron (NM_0(	-0430	695E-04	127E-02	Pdgfrl	35162	Hypo
chr2	232152356	232153701	Distal Interger	-0528	695E-04	127E-02	Pkn2	-253403	Hypo
chr2	115883460	115893070	Intron (NM_1	-0319	699E-04	127E-02	Pex5l	14912	Hypo
chr4	59711474	59716859	Distal Interger	-0326	700E-04	127E-02	Mir29b1	-60407	Hypo
chr17	5437301	5439691	Distal Interger	-0319	703E-04	128E-02	Agtpbp1	303603	Hypo
chr8	47967873	47968497	Intron (NM_0(	-0755	706E-04	128E-02	Cadm1	120037	Hypo
chr20	18242704	18246608	Distal Interger	-0372	706E-04	128E-02	Mrln	175202	Hypo
chr14	87039010	87041438	Distal Interger	-0396	706E-04	128E-02	Cobl	-59471	Hypo

chr10	60705011	60705302	Intron (NM_1	-1007	708E-04	128E-02	Doc2b	-51744	Hypo
chr7	18841407	18843459	Promoter (<=1	-0327	709E-04	129E-02	Ric8b	0	Hypo
chr10	82615680	82616688	Distal Interger	-0603	710E-04	129E-02	Epop	9224	Hypo
chr12	21534917	21535557	Distal Interger	-0678	712E-04	129E-02	Bcl7b	-24715	Hypo
chr8	45553007	45556786	Distal Interger	-0441	713E-04	129E-02	Il10ra	21255	Hypo
chr7	74776461	74777802	Distal Interger	-0495	714E-04	129E-02	Emc2	189265	Hypo
chr8	113261151	113262493	Distal Interger	-0565	714E-04	129E-02	Pdcd6ip	384280	Hypo
chr10	66197193	66198501	Intron (NM_0	-0515	716E-04	129E-02	Asic2	-58814	Hypo
chr18	67777339	67779227	Distal Interger	-0446	716E-04	129E-02	Ska1	25810	Hypo
chr1	223040413	223044146	Intron (NM_0	-0419	717E-04	130E-02	Kank1	85036	Hypo
chr17	1566153	1568098	Intron (NM_0	-0538	718E-04	130E-02	Ptch1	15581	Hypo
chr1	172111238	172111823	Distal Interger	-0804	720E-04	130E-02	Rps15a	314214	Hypo
chr2	26332468	26338366	Exon (NM_19	-0332	720E-04	130E-02	Pde8b	42941	Hypo
chr12	33861141	33866541	Promoter (<=1	-0302	721E-04	130E-02	P2rx4	0	Hypo
chr7	126009241	126010469	Intron (NM_0	-0570	723E-04	130E-02	Tmem117	251650	Hypo
chr17	18071017	18075622	Intron (NM_0	-0337	723E-04	130E-02	Fam8a1	-35547	Hypo
chr19	4867161	4868417	Distal Interger	-0549	724E-04	130E-02	Cdh8	-625757	Hypo
chr3	146705063	146705831	Intron (NM_0	-0656	724E-04	130E-02	Rprd1b	22088	Hypo
chr17	10524964	10526156	Promoter (2-3	-0616	725E-04	131E-02	Sfxn1	-2463	Hypo
chr8	1148634	1149750	Distal Interger	-0567	725E-04	131E-02	Aasdhppt	314240	Hypo
chr13	77374470	77382247	Distal Interger	-0315	726E-04	131E-02	Tbx19	90999	Hypo
chr4	117856927	117860690	Intron (NM_1	-0450	726E-04	131E-02	Sfxn5	9092	Hypo
chr7	63743219	63746000	Distal Interger	-0390	726E-04	131E-02	Rdh7	-53844	Hypo
chr7	108996944	109006674	Intron (NM_0	-0289	727E-04	131E-02	Rbfox2	47729	Hypo
chr6	68706176	68707603	Distal Interger	-0560	727E-04	131E-02	G2e3	-56582	Hypo
chr10	52268299	52269543	Intron (NM_0	-0576	727E-04	131E-02	Gas7	39040	Hypo
chr3	116798831	116805255	Distal Interger	-0307	729E-04	131E-02	Sirpa	-15117	Hypo
chr9	3165026	3165334	Distal Interger	-1188	730E-04	131E-02	Plcl2	-128034	Hypo
chr8	95760161	95761225	Intron (NM_0	-0626	730E-04	131E-02	Paqr9	-353024	Hypo
chr3	69457662	69458599	Intron (NM_0	-0697	731E-04	131E-02	Calcrl	67051	Hypo
chr9	36971901	36972175	Distal Interger	-1039	731E-04	131E-02	Fam168b	66456	Hypo
chr2	215154722	215155082	Intron (NM_0	-0893	732E-04	131E-02	Camk2d	130718	Hypo
chr8	52961848	52962707	Distal Interger	-0650	732E-04	131E-02	LOC10012536	106305	Hypo

chr18	34118347	34121353	Intron (NM_0(	-0423	732E-04	131E-02	Sh3rf2	71468	Hypo
chr13	71391045	71391748	Distal Interger	-0809	734E-04	132E-02	Cop1	-75416	Hypo
chr1	143931576	143932660	Distal Interger	-0668	735E-04	132E-02	Eed	-36618	Hypo
chr1	184957036	184960573	Distal Interger	-0391	735E-04	132E-02	Fgfr2	-106410	Hypo
chr1	128507438	128508190	Distal Interger	-0726	735E-04	132E-02	Slco3a1	-119551	Hypo
chr11	15807645	15812991	Distal Interger	-0304	735E-04	132E-02	Usp25	203764	Hypo
chr16	80954466	80956086	Distal Interger	-0502	735E-04	132E-02	Efnb2	171049	Hypo
chr7	62526520	62527274	Distal Interger	-0685	735E-04	132E-02	Atp23	202549	Hypo
chr8	49992554	49996202	Intron (NM_0:	-0390	737E-04	132E-02	Ttc12	-146869	Hypo
chr20	8359417	8360757	Intron (NM_0(	-0656	737E-04	132E-02	Zfand3	276010	Hypo
chr11	16054228	16055457	Distal Interger	-0634	738E-04	132E-02	Mir99a	-144986	Hypo
chr3	161136882	161138332	Intron (NM_1!	-0590	739E-04	132E-02	Aurka	4004	Hypo
chr16	20530271	20532923	Distal Interger	-0449	740E-04	132E-02	Lzts1	-9886	Hypo
chr5	10001224	10003428	Distal Interger	-0417	740E-04	132E-02	Rrs1	-266105	Hypo
chr6	61448121	61450690	Intron (NM_0	-0391	740E-04	132E-02	Nrcam	45282	Hypo
chr17	67182925	67186744	Distal Interger	-0349	741E-04	132E-02	Pfkfb3	147830	Hypo
chr6	105101778	105103935	Distal Interger	-0359	742E-04	132E-02	Fos	-17235	Hypo
chr1	234525683	234528503	Distal Interger	-0459	743E-04	133E-02	Tnks2	-39687	Hypo
chr6	35088839	35096914	Intron (NM_0(	-0323	743E-04	133E-02	Cyria	43631	Hypo
chr7	69335021	69337012	Distal Interger	-0443	744E-04	133E-02	Odf1	-44891	Hypo
chr1	93962315	93963949	Distal Interger	-0480	744E-04	133E-02	Zfp819	-74299	Hypo
chr8	95757913	95759829	Intron (NM_0(	-0459	744E-04	133E-02	Paqr9	-354420	Hypo
chr6	85375184	85376975	Intron (NM_1!	-0457	746E-04	133E-02	Mdga2	231256	Hypo
chr20	32911369	32912233	Distal Interger	-0642	746E-04	133E-02	Asf1a	17407	Hypo
chr4	176646649	176647283	Distal Interger	-0714	746E-04	133E-02	Sox5	512242	Hypo
chr17	67313102	67314151	Intron (NM_0(	-0695	747E-04	133E-02	Prkcq	64553	Hypo
chr1	46580563	46584612	Exon (NM_03:	-0372	747E-04	133E-02	Synj2	61805	Hypo
chr13	38883800	38885386	Intron (NM_0:	-0482	748E-04	133E-02	Mgat5	207681	Hypo
chr6	107943461	107944718	Intron (NM_0!	-0597	751E-04	134E-02	Nrxn3	301477	Hypo
chr1	239271719	239274868	Distal Interger	-0393	754E-04	134E-02	Mir6317-1	124184	Hypo
chr1	218989095	218990714	Distal Interger	-0524	754E-04	134E-02	Gda	-6735	Hypo
chr9	88293911	88294295	Intron (NM_0	-0915	755E-04	134E-02	Inpp5d	6232	Hypo
chr7	93284878	93286398	Distal Interger	-0679	755E-04	134E-02		279820	Hypo

chr5	165115317	165118223	Distal Interger	-0390	755E-04	134E-02	Actrt2	119269	Hypo
chr17	18249874	18251546	Distal Interger	-0473	755E-04	134E-02	Rbm24	35873	Hypo
chr11	57776719	57777823	Intron (NM_0(	-0585	755E-04	134E-02	Zbtb20	13391	Hypo
chr4	120350034	120351608	Distal Interger	-0537	756E-04	134E-02	Copg1	-15137	Hypo
chr2	170281527	170288156	Distal Interger	-0332	756E-04	134E-02	Dear	12026	Hypo
chr7	114564531	114577229	Promoter (<=:	-0299	759E-04	135E-02	Pacsin2	0	Hypo
chr19	20932580	20934339	Distal Interger	-0539	760E-04	135E-02	Phkb	276266	Hypo
chr13	77794603	77796598	Intron (NM_0:	-0454	760E-04	135E-02	Adcy10	43291	Hypo
chr1	246713746	246716796	Distal Interger	-0373	761E-04	135E-02	Gsto1	-4447	Hypo
chr11	29404122	29406202	Distal Interger	-0560	762E-04	135E-02	Sod1	-50471	Hypo
chr19	15637456	15638960	Intron (NM_0(	-0494	763E-04	135E-02	Fto	53096	Hypo
chrX	49407711	49408060	Intron (NM_0(	-0951	766E-04	135E-02	Dmd	62318	Hypo
chr7	57868552	57868885	Distal Interger	-1016	767E-04	135E-02	Rxylt1	-85935	Hypo
chr5	165509582	165510787	Distal Interger	-0621	768E-04	136E-02	Hes5	-11447	Hypo
chr6	20611459	20613283	Intron (NM_0(	-0509	768E-04	136E-02	Ttc27	88832	Hypo
chr15	70052507	70057500	Intron (NM_0(	-0356	768E-04	136E-02	Pcdh9	178165	Hypo
chr4	118299788	118301393	Distal Interger	-0530	769E-04	136E-02	Nat8f5	-14750	Hypo
chr16	44458146	44458555	Distal Interger	-0940	769E-04	136E-02	Cldn24	-32674	Hypo
chr1	4899757	4900132	Distal Interger	-0908	770E-04	136E-02	Rab32	60871	Hypo
chr5	119375003	119376317	Intron (NM_1!	-0623	770E-04	136E-02	Dab1	117975	Hypo
chr9	110415523	110427042	Promoter (<=:	-0286	771E-04	136E-02	Dlgap1	0	Hypo
chr3	26067630	26069006	Distal Interger	-0506	772E-04	136E-02	Kynu	-1709749	Hypo
chr8	67335795	67336719	Distal Interger	-0744	774E-04	136E-02	Car12	60896	Hypo
chr18	14685181	14691159	Distal Interger	-0316	775E-04	136E-02	Mapre2	-380212	Hypo
chr11	15542108	15544010	Distal Interger	-0377	775E-04	136E-02	Usp25	-59871	Hypo
chr2	100729490	100730204	Distal Interger	-0695	775E-04	136E-02	Cyp7b1	-59792	Hypo
chr3	119239041	119239676	Distal Interger	-0763	776E-04	136E-02	Prnd	23485	Hypo
chr4	68679509	68681412	Intron (NM_0(	-0448	779E-04	137E-02	Tmem178b	20084	Hypo
chr9	37326257	37326788	Distal Interger	-0773	780E-04	137E-02	Plekhb2	276677	Hypo
chr19	20934584	20935435	Distal Interger	-0715	780E-04	137E-02	Phkb	275170	Hypo
chr12	10449993	10458468	Exon (NM_00:	-0351	781E-04	137E-02	Bhlha15	-25352	Hypo
chr15	82462858	82465541	Distal Interger	-0446	782E-04	137E-02	Spry2	229783	Hypo
chr10	55582854	55585357	Intron (NM_0	-0463	782E-04	137E-02	Rabep1	16522	Hypo

chr1	176265610	176266414	Distal Interger	-0762	783E-04	137E-02	Usp31	-13237	Hypo
chr3	127276934	127277937	Intron (NM_0(	-0604	784E-04	137E-02	Tasp1	121663	Hypo
chr1	32378135	32380288	Distal Interger	-0567	784E-04	137E-02	Adamts16	-50148	Hypo
chr13	95480689	95481324	Distal Interger	-0673	785E-04	137E-02	Dusp10	-132968	Hypo
chrX	9109352	9115223	Promoter (<=1	-0379	786E-04	137E-02	Gpr34	0	Hypo
chr12	8223548	8224369	Distal Interger	-0686	786E-04	137E-02	Rasl11a	38207	Hypo
chr5	100720162	100720567	Distal Interger	-0940	786E-04	137E-02	Rraga	-392774	Hypo
chr13	97040246	97041466	Distal Interger	-0799	787E-04	137E-02	Slc30a10	42103	Hypo
chr3	83825819	83826182	Distal Interger	-0819	787E-04	137E-02	Lrrc4c	1520575	Hypo
chr3	161172209	161176210	Intron (NM_0(	-0501	789E-04	138E-02	Cass4	8590	Hypo
chr15	95289611	95292707	Distal Interger	-0421	791E-04	138E-02	Gpr180	89795	Hypo
chr1	50299455	50319490	3' UTR	-0309	791E-04	138E-02	Cahm	67469	Hypo
chr18	73785713	73786472	Intron (NM_0(	-0689	793E-04	138E-02	Kcng2	22251	Hypo
chr8	84824891	84825736	Distal Interger	-0655	794E-04	138E-02	Bckdhb	-19528	Hypo
chr7	125549865	125550939	Distal Interger	-0579	794E-04	138E-02	Irak4	-131563	Hypo
chr7	102301398	102302633	Distal Interger	-0481	794E-04	138E-02	Khdrbs3	1463464	Hypo
chr7	122144151	122146387	Intron (NM_0(	-0451	795E-04	138E-02	Kif21a	32612	Hypo
chr13	46592029	46606114	Promoter (<=1	-0282	796E-04	138E-02	Gpr37l1	0	Hypo
chr9	14425586	14429736	Promoter (<=1	-0341	796E-04	138E-02	Srf	0	Hypo
chr15	70327055	70328451	Distal Interger	-0583	797E-04	138E-02	Pcdh9	-91390	Hypo
chr18	36675158	36678328	Distal Interger	-0412	798E-04	138E-02	Myot	-26986	Hypo
chr8	42543225	42543483	Distal Interger	-1003	800E-04	139E-02	Sorl1	-38997	Hypo
chr2	185080282	185083063	Distal Interger	-0364	801E-04	139E-02	Or13l2	-122316	Hypo
chr3	21858894	21861325	Intron (NM_0(	-0424	801E-04	139E-02	Dennd1a	133548	Hypo
chr13	77396468	77398330	Distal Interger	-0489	803E-04	139E-02	Tbx19	74916	Hypo
chr8	50874304	50878515	Promoter (<=1	-0336	806E-04	140E-02	Pts	0	Hypo
chr14	46822603	46824242	Exon (NM_001	-0544	807E-04	140E-02	Arap2	103843	Hypo
chr2	159803830	159805062	Distal Interger	-0585	807E-04	140E-02	Zbbx	118428	Hypo
chr11	23085189	23085592	Distal Interger	-0861	808E-04	140E-02	Mir155hg	-687876	Hypo
chr13	53100775	53102084	Distal Interger	-0536	809E-04	140E-02	Kcnt2	1436089	Hypo
chr10	73397422	73400908	Distal Interger	-0421	810E-04	140E-02	Akap1	235338	Hypo
chr3	105525608	105526044	Distal Interger	-0807	815E-04	141E-02	Bmf	-5504	Hypo
chr10	55549136	55550262	Distal Interger	-0584	817E-04	141E-02	Rabep1	-16070	Hypo

chr3	92176421	92177119	Intron (NM_0(	-0746	817E-04	141E-02	Pax6	40579	Hypo
chr13	39528706	39530032	Distal Interger	-0596	820E-04	141E-02	Mir3473	29460	Hypo
chr8	112837743	112838752	Distal Interger	-0610	824E-04	142E-02	Arpp21	-684712	Hypo
chr6	104395634	104397095	Distal Interger	-0519	825E-04	142E-02	Npc2	21066	Hypo
chr1	4093264	4094505	Promoter (2-3	-0533	825E-04	142E-02	Samd5	-2355	Hypo
chr8	36531859	36533388	Distal Interger	-0444	826E-04	142E-02	Fez1	-11147	Hypo
chr13	39538937	39539638	Distal Interger	-0585	827E-04	142E-02	Mir3473	39691	Hypo
chr1	176127337	176128360	Distal Interger	-0637	827E-04	142E-02	Usp31	124013	Hypo
chr2	235501697	235502453	Intron (NM_0!	-0691	829E-04	143E-02	Uox	14830	Hypo
chr17	8391783	8393210	Distal Interger	-0450	829E-04	143E-02	Neurog1	28905	Hypo
chr4	162146587	162147492	Distal Interger	-0711	833E-04	143E-02	LOC689770	17337	Hypo
chr15	99541378	99542480	Distal Interger	-0624	833E-04	143E-02	Zic5	24555	Hypo
chr9	107113466	107115511	Intron (NM_0(	-0426	834E-04	143E-02	Ptprm	228187	Hypo
chr5	128577940	128579131	Distal Interger	-0601	835E-04	143E-02	Tal1	-8570	Hypo
chr3	20541700	20542237	Distal Interger	-0874	835E-04	143E-02	Or12k7b	10504	Hypo
chr1	117781196	117782907	Exon (NM_00:	-0499	835E-04	143E-02	Mir211	3657	Hypo
chr7	101685227	101687282	Distal Interger	-0521	835E-04	143E-02	Khdrbs3	847293	Hypo
chr2	136232345	136234066	Distal Interger	-0540	835E-04	143E-02	Foxo1	-78102	Hypo
chr9	79055486	79057419	Distal Interger	-0458	836E-04	143E-02	Epha4	-97347	Hypo
chr13	96708118	96709077	Distal Interger	-0616	837E-04	143E-02	Rab3gap2	-48485	Hypo
chr9	104376693	104383128	Intron (NM_0	-0335	838E-04	144E-02	Man2a1	124692	Hypo
chr10	68983240	68986397	Intron (NM_0(	-0404	838E-04	144E-02	Dusp14	-26842	Hypo
chr15	54165265	54165994	Distal Interger	-0623	838E-04	144E-02	Akap11	-223660	Hypo
chr10	28873792	28875069	Distal Interger	-0514	838E-04	144E-02	Il12b	-17941	Hypo
chr5	77628417	77628778	Distal Interger	-1026	840E-04	144E-02	Tnc	-192379	Hypo
chr11	67734060	67736460	Exon (NM_00:	-0488	840E-04	144E-02	Iqcg	3813	Hypo
chrX	24342315	24345741	Distal Interger	-0406	840E-04	144E-02	Mid1	-93962	Hypo
chr10	2626408	2629734	Distal Interger	-0408	840E-04	144E-02	Rrn3	-461167	Hypo
chr2	8031921	8038781	Distal Interger	-0310	841E-04	144E-02	Nr2f1	11556	Hypo
chr1	230215272	230218616	Intron (NM_1:	-0378	841E-04	144E-02	Sgms1	40976	Hypo
chr17	82171590	82175812	Distal Interger	-0335	842E-04	144E-02	Ptf1a	120309	Hypo
chr12	18572029	18572738	Distal Interger	-0679	842E-04	144E-02	Vom2r64	152620	Hypo
chr10	25613109	25614743	Distal Interger	-0482	842E-04	144E-02	Ccng1	-430505	Hypo

chr12	31679338	31680431	Distal Interger	-0535	843E-04	144E-02	Rflna	-37021	Hypo
chr14	100637023	100642930	Distal Interger	-0383	844E-04	144E-02	Vrk2	-202496	Hypo
chr2	170924814	170931983	Distal Interger	-0292	844E-04	144E-02	Fhip1a	159499	Hypo
chr4	117228128	117229786	Exon (NM_00:	-0580	844E-04	144E-02	Cyp26b1	-169500	Hypo
chr10	57574531	57575125	Distal Interger	-0766	845E-04	144E-02	Atp2a3	-7003	Hypo
chrX	5597722	5598527	Distal Interger	-0829	847E-04	144E-02	Ndp	-197960	Hypo
chr18	51216481	51219027	Distal Interger	-0435	847E-04	144E-02	Slc12a2	-129275	Hypo
chr6	116605573	116606287	Distal Interger	-0682	850E-04	145E-02	Galc	909524	Hypo
chr7	26699883	26702393	Distal Interger	-0412	850E-04	145E-02	Mir135a	-87794	Hypo
chr13	76916523	76922154	Distal Interger	-0328	850E-04	145E-02	Atp1b1	-109427	Hypo
chr20	28301413	28309296	3' UTR	-0371	851E-04	145E-02	Vsir	19794	Hypo
chr9	33005487	33006282	Distal Interger	-0613	852E-04	145E-02	Phf3	129712	Hypo
chr5	153433896	153441593	Promoter (1-2	-0313	852E-04	145E-02	Szrd1	1650	Hypo
chr12	21170754	21171857	Intron (NM_0(	-0627	853E-04	145E-02	Hip1	37346	Hypo
chr3	92977412	92978424	Distal Interger	-0605	853E-04	145E-02	Mpped2	-204913	Hypo
chr16	23504370	23504906	Intron (NM_0(	-0754	855E-04	145E-02	Tma16	267573	Hypo
chr6	90100146	90100765	Distal Interger	-0760	856E-04	145E-02	Daam1	-346271	Hypo
chr18	14715897	14718142	Distal Interger	-0419	856E-04	145E-02	Mapre2	-353229	Hypo
chr1	155422657	155424354	Distal Interger	-0492	857E-04	145E-02	Fchsd2	-36575	Hypo
chr13	101291268	101291753	Intron (NM_0(	-0838	857E-04	145E-02	Ptpn14	22852	Hypo
chr18	28765832	28766156	Intron (NM_1!	-0883	858E-04	145E-02		20780	Hypo
chr6	105522762	105524562	Distal Interger	-0433	859E-04	146E-02	Erg28	-39684	Hypo
chr14	7442945	7443497	Distal Interger	-0789	861E-04	146E-02	Wdfy3	-163131	Hypo
chr13	42875493	42876784	Intron (NM_0(	-0572	862E-04	146E-02	Srgap2	90274	Hypo
chr9	25929931	25930439	Distal Interger	-0781	862E-04	146E-02	Ogfrl1	-87612	Hypo
chr1	94277410	94280626	Promoter (<=:	-0449	863E-04	146E-02	Klk6	0	Hypo
chr3	65157109	65157865	Distal Interger	-0777	867E-04	147E-02	Dnajc10	-74832	Hypo
chr14	12619770	12622809	Distal Interger	-0373	868E-04	147E-02	Paqr3	157140	Hypo
chr7	76249957	76251407	Distal Interger	-0525	868E-04	147E-02	Kcnv1	16433	Hypo
chr1	3064534	3067482	Distal Interger	-0432	871E-04	147E-02	Ust	-102490	Hypo
chr2	216992551	216993413	Distal Interger	-0598	872E-04	147E-02	Fam241a	-617358	Hypo
chrX	145247942	145254537	Promoter (<=:	-0326	873E-04	147E-02	Slitrk2	0	Hypo
chr14	71615325	71616608	Distal Interger	-0549	873E-04	148E-02	Hs3st1	400306	Hypo

chr3	64359560	64360263	3' UTR	-0858	873E-04	148E-02	Neurod1	3263	Hypo
chr17	70866823	70870480	Distal Interger	-0379	873E-04	148E-02	Celf2	-539130	Hypo
chr8	32826607	32827200	Distal Interger	-0762	874E-04	148E-02	Kirrel3	-38665	Hypo
chr2	166830066	166830664	Distal Interger	-0667	876E-04	148E-02	Tdo2	456847	Hypo
chr1	133132084	133133954	Distal Interger	-0437	879E-04	148E-02	Mfge8	-52015	Hypo
chr11	76194291	76196673	Intron (NM_0(	-0422	879E-04	148E-02	Lpp	103892	Hypo
chr6	105000762	105003358	Exon (NM_05:	-0354	880E-04	148E-02	Nek9	-16253	Hypo
chr14	87044448	87046130	Distal Interger	-0521	881E-04	148E-02	Cobl	-64909	Hypo
chr1	15503261	15505499	Distal Interger	-0501	882E-04	148E-02	Pde7b	-10361	Hypo
chr1	216301496	216303034	Distal Interger	-0500	885E-04	149E-02	C1h9orf40	172809	Hypo
chr17	81204994	81205910	Distal Interger	-0671	885E-04	149E-02	Commd3	-121559	Hypo
chr6	90062886	90064481	Distal Interger	-0543	886E-04	149E-02	Daam1	-382555	Hypo
chr12	25715705	25716498	Intron (NM_0(	-0569	888E-04	149E-02	Galnt17	391837	Hypo
chr18	25193101	25193383	Distal Interger	-0897	888E-04	149E-02	Nrep	-148444	Hypo
chr7	87228600	87229443	Intron (NM_0(	-0648	889E-04	149E-02	Sntb1	99872	Hypo
chr10	25209026	25215518	Distal Interger	-0309	890E-04	149E-02	Ccng1	-26422	Hypo
chr16	74025664	74026142	Distal Interger	-0773	891E-04	150E-02	Myom2	563998	Hypo
chrX	5911676	5912820	Intron (NM_0	-0642	892E-04	150E-02	Maob	4349	Hypo
chr15	82418937	82420785	Distal Interger	-0503	893E-04	150E-02	Spry2	274539	Hypo
chr3	34580469	34581369	Distal Interger	-0634	893E-04	150E-02	Ns5atp4l1	-113999	Hypo
chr13	98164003	98165694	Intron (NM_0:	-0445	895E-04	150E-02	Tgfb2	95540	Hypo
chr2	12256536	12257545	Distal Interger	-0629	896E-04	150E-02	Cetn3	167119	Hypo
chr5	150077090	150082637	Promoter (<=:	-0317	897E-04	150E-02	Ece1	0	Hypo
chr11	30441729	30445042	Distal Interger	-0373	898E-04	150E-02	Olig2	-30468	Hypo
chr6	20269352	20272629	Exon (NM_02:	-0396	899E-04	150E-02	Ltbp1	152711	Hypo
chr9	107707390	107708183	Intron (NM_0(	-0734	900E-04	151E-02	Lama1	14620	Hypo
chr10	28872654	28873638	Distal Interger	-0637	901E-04	151E-02	Il12b	-19372	Hypo
chr13	65691333	65691826	Intron (NM_0(	-0727	903E-04	151E-02	Npl	5406	Hypo
chr16	45350079	45351334	Distal Interger	-0533	903E-04	151E-02	Enpp6	-56002	Hypo
chrX	52776391	52779633	Distal Interger	-0462	903E-04	151E-02	Il1rapl1	-138741	Hypo
chr7	26880246	26881263	Distal Interger	-0571	905E-04	151E-02	Mir135a	-268157	Hypo
chr1	176128502	176129260	Distal Interger	-0700	907E-04	151E-02	Usp31	123113	Hypo
chr2	137063185	137063669	Intron (NM_0(	-0715	908E-04	151E-02	Cog6	35507	Hypo

chr2	16100700	16103539	Distal Interger	-0433	908E-04	151E-02	Rasa1	-160419	Hypo
chr5	111338429	111339209	Distal Interger	-0574	908E-04	151E-02	C5h1orf87	70740	Hypo
chr16	68928886	68929794	Intron (NM_0(	-0609	909E-04	152E-02	Ank1	39208	Hypo
chr18	48613532	48618002	Distal Interger	-0353	910E-04	152E-02	Zfp608	-203067	Hypo
chr4	46967865	46968564	Distal Interger	-0672	913E-04	152E-02	Cttnbp2	-3234	Hypo
chr2	112197686	112199738	Distal Interger	-0439	914E-04	152E-02	Cldn11	21312	Hypo
chr11	57071131	57072110	Distal Interger	-0544	918E-04	153E-02	Mir568	-17058	Hypo
chr9	54829481	54831231	Distal Interger	-0468	919E-04	153E-02	Slc39a10	-79771	Hypo
chr6	140375250	140375898	Distal Interger	-0689	922E-04	153E-02	Macc1	-35828	Hypo
chr3	139465502	139466680	Intron (NM_0(	-0511	923E-04	153E-02	Acss1	33645	Hypo
chr16	45591712	45593130	Distal Interger	-0522	925E-04	154E-02	Irf2	-41753	Hypo
chr15	45139344	45140185	Distal Interger	-0734	925E-04	154E-02	Egr3	-10698	Hypo
chr15	82689714	82699873	Promoter (<=1	-0289	926E-04	154E-02	Spry2	0	Hypo
chr2	29276246	29279079	Intron (NM_0(	-0474	926E-04	154E-02	Arhgef28	249521	Hypo
chr6	118606399	118607219	Intron (NM_0(	-0584	926E-04	154E-02	Foxn3	235182	Hypo
chr1	50279467	50281566	Intron (NM_0(	-0429	927E-04	154E-02	Cahm	105393	Hypo
chr2	82424697	82426712	Distal Interger	-0402	928E-04	154E-02	Ropn1l	26620	Hypo
chr10	75675956	75677948	Distal Interger	-0480	928E-04	154E-02	LOC10036342	167602	Hypo
chrX	152158730	152159281	Promoter (<=1	-0647	929E-04	154E-02	Slc10a3	-167	Hypo
chr6	104390789	104392464	Distal Interger	-0529	930E-04	154E-02	Npc2	25697	Hypo
chr6	24751119	24751780	Intron (NM_0(	-0768	934E-04	155E-02	Rbks	3389	Hypo
chr1	207447433	207450436	Exon (NM_175	-0406	934E-04	155E-02	Cd6	31198	Hypo
chr18	15217920	15219606	Distal Interger	-0531	935E-04	155E-02	Zfp397	-22479	Hypo
chr2	33774364	33776761	Distal Interger	-0358	936E-04	155E-02	Cd180	-79230	Hypo
chr14	87030418	87034059	Distal Interger	-0389	937E-04	155E-02	Cobl	-50879	Hypo
chr4	67394005	67394679	Distal Interger	-0719	937E-04	155E-02	Clec2l	-3587	Hypo
chr1	25676529	25678975	Distal Interger	-0461	940E-04	155E-02	Rnf217	-337693	Hypo
chr1	176947259	176952457	Intron (NM_0	-0318	941E-04	155E-02	Prkcb	114574	Hypo
chr16	56215269	56215847	Distal Interger	-0771	946E-04	156E-02	Prag1	-74116	Hypo
chr19	40636004	40638197	Intron (NM_0(	-0412	947E-04	156E-02	Cntnap4	67320	Hypo
chr20	46357212	46357880	Intron (NM_0(	-0728	949E-04	156E-02	Scml4	9701	Hypo
chr2	171020178	171027298	3' UTR	-0311	950E-04	156E-02	Fhip1a	64184	Hypo
chr11	50242136	50244445	Distal Interger	-0439	951E-04	157E-02	Bbx	-236168	Hypo

chr19	47140219	47141792	Intron (NM_1:	-0502	951E-04	157E-02	Hsbp1	-259247	Hypo
chr7	22340282	22340671	Exon (NM_00:	-1000	952E-04	157E-02	Igf1	55222	Hypo
chr4	87584018	87585861	Distal Interger	-0506	953E-04	157E-02	Rps7-ps20	16175	Hypo
chr2	218269167	218270783	Exon (NM_01:	-0355	954E-04	157E-02	Egf	31281	Hypo
chr16	46220132	46222702	Distal Interger	-0446	954E-04	157E-02	Lrp2bp	36748	Hypo
chr8	22830136	22831143	3' UTR	-0618	955E-04	157E-02	Dpy19l1	190318	Hypo
chr17	20911107	20912560	Distal Interger	-0533	955E-04	157E-02	Cd83	-4404	Hypo
chr2	28496797	28500444	Exon (NM_00:	-0376	956E-04	157E-02	Hexb	3721	Hypo
chr4	11563502	11565056	Intron (NM_0(	-0485	956E-04	157E-02	Srpk2	26117	Hypo
chr17	29367614	29369655	Distal Interger	-0483	956E-04	157E-02	Cdyl	-163301	Hypo
chr1	52571735	52574032	Distal Interger	-0460	957E-04	157E-02	Cep43	-7780	Hypo
chr6	40898056	40899143	Intron (NM_0:	-0604	958E-04	157E-02	Adam17	21557	Hypo
chr7	85427896	85429086	Distal Interger	-0459	960E-04	157E-02	Tnfrsf11b	165440	Hypo
chr7	62654750	62656095	Distal Interger	-0504	962E-04	158E-02	Atp23	73728	Hypo
chr6	48361708	48363012	Distal Interger	-0560	964E-04	158E-02	Gpr22	-44222	Hypo
chr2	167017937	167020709	Distal Interger	-0487	964E-04	158E-02	Tdo2	266802	Hypo
chr1	41890932	41895004	Distal Interger	-0369	965E-04	158E-02	Myct1	-123133	Hypo
chr10	26185812	26186817	Distal Interger	-0532	965E-04	158E-02	Gabrg2	276863	Hypo
chr18	67152388	67152842	Distal Interger	-0766	965E-04	158E-02	Mex3c	-14758	Hypo
chr15	28188973	28192249	Exon (NM_05:	-0382	969E-04	158E-02	Cebpe	-17690	Hypo
chr1	126934728	126935910	Distal Interger	-0597	969E-04	158E-02	Rgma	-193024	Hypo
chr8	23667127	23690850	Distal Interger	-0268	969E-04	158E-02	LOC500959	-18026	Hypo
chr9	80921766	80923242	Distal Interger	-0482	970E-04	158E-02	Ap1s3	86563	Hypo
chr2	183490487	183492475	Distal Interger	-0321	970E-04	158E-02	Anp32e	17827	Hypo
chr4	118048054	118050825	Promoter (<=:	-0410	971E-04	158E-02	Egr4	0	Hypo
chr11	20277505	20281268	Intron (NM_2(	-0390	971E-04	159E-02	Ncam2	172459	Hypo
chr14	66209814	66211208	Distal Interger	-0483	974E-04	159E-02	Ldb2	-65877	Hypo
chr9	108367818	108369802	Distal Interger	-0460	975E-04	159E-02	Arhgap28	-369918	Hypo
chr6	38914838	38916336	Distal Interger	-0540	976E-04	159E-02	Trib2	-346115	Hypo
chr6	41513051	41513492	Intron (NM_0(	-0808	976E-04	159E-02	Mboat2	41890	Hypo
chr6	51927079	51927897	Distal Interger	-0644	976E-04	159E-02	Snx13	167753	Hypo
chr7	24200721	24201340	Intron (NM_0(	-0749	976E-04	159E-02	Bltp3b	19077	Hypo
chr6	140184467	140186541	Intron (NM_0(	-0418	977E-04	159E-02	Itgb8	21691	Hypo

chr9	66010898	66011672	Distal Interger	-0576	977E-04	159E-02	Creb1	89448	Hypo
chr1	29417463	29417945	Distal Interger	-0799	977E-04	159E-02	Nkd2	-26035	Hypo
chr6	119646417	119651786	Intron (NM_0(	-0329	978E-04	159E-02	Ttc7b	53401	Hypo
chr8	62353746	62354976	Distal Interger	-0533	978E-04	159E-02	Rplp1	40365	Hypo
chr20	44182009	44184883	Intron (NM_0(	-0341	979E-04	159E-02	Mettl24	39613	Hypo
chr14	80874904	80879590	Exon (NM_001	-0319	980E-04	159E-02	Ykt6	42550	Hypo
chr3	146004261	146008027	Intron (NM_0:	-0371	981E-04	159E-02	Ghrh	3862	Hypo
chr1	50649568	50650063	Distal Interger	-0920	982E-04	159E-02	Qki	261870	Hypo
chr4	33930438	33930933	Intron (NM_0	-0683	983E-04	160E-02	Dync1i1	77511	Hypo
chr11	70143263	70144504	Promoter (<=1	-0368	984E-04	160E-02	Lsg1	0	Hypo
chr2	165438038	165444199	Distal Interger	-0318	985E-04	160E-02	Gask1b	390321	Hypo
chr1	155444031	155445256	Distal Interger	-0553	986E-04	160E-02	Fchsd2	-15673	Hypo
chr8	59708450	59709876	Intron (NM_0(	-0503	987E-04	160E-02	Adpgk	9006	Hypo
chr18	78464775	78468623	Distal Interger	-0341	989E-04	160E-02	Timm21	-145413	Hypo
chr5	100875455	100875986	Distal Interger	-0748	989E-04	160E-02	Rraga	-237355	Hypo
chr13	69300314	69301930	Intron (NM_0(	-0491	989E-04	160E-02	Rasal2	129705	Hypo
chr16	68775418	68778532	Exon (NM_001	-0416	992E-04	161E-02	Golga7	8043	Hypo
chr13	82113922	82115746	Distal Interger	-0472	992E-04	161E-02	Ccdc190	-31539	Hypo
chr11	57101246	57105779	Intron (NM_0(	-0330	993E-04	161E-02	Mir568	-47173	Hypo
chr1	217343442	217344469	Distal Interger	-0599	997E-04	161E-02	Anxa1	532725	Hypo
chr13	63703592	63703966	Intron (NM_0:	-0986	997E-04	161E-02	Niban1	29352	Hypo
chr3	90944613	90946135	Distal Interger	-0511	998E-04	161E-02	Cstf3	-20280	Hypo
chr13	62161346	62165549	Promoter (<=1	-0328	100E-03	162E-02	Ptgs2os	0	Hypo
chr15	82027650	82029083	Distal Interger	-0595	100E-03	162E-02	Ndfip2	-3283	Hypo
chr8	106808353	106809234	Distal Interger	-0688	100E-03	162E-02	Wdr82	-5335	Hypo
chr15	38624924	38644414	Intron (NM_0!	-0298	100E-03	162E-02	Msra	32082	Hypo
chr2	98715842	98716576	Distal Interger	-0618	100E-03	162E-02	Ythdf3	365181	Hypo
chr20	9778264	9780278	Distal Interger	-0469	100E-03	162E-02	Cryaa	-3327	Hypo
chr5	5020369	5021377	Distal Interger	-0598	101E-03	162E-02	Msc	469033	Hypo
chr2	119083684	119086665	Intron (NM_1!	-0385	101E-03	162E-02	Qrfpr	10127	Hypo
chr1	90335768	90338731	Distal Interger	-0401	101E-03	163E-02	Uri1	365925	Hypo
chr20	34773362	34774329	Distal Interger	-0616	101E-03	163E-02	Msl3l2	-698862	Hypo
chr20	4104042	4108246	Promoter (2-3	-0339	101E-03	163E-02	Fkbpl	-2727	Hypo

chr8	68020109	68021136	Distal Interger	-0520	101E-03	163E-02	Mir190a-2	-169038	Hypo
chr8	95751223	95755599	Intron (NM_0(	-0367	101E-03	163E-02	Paqr9	-358650	Hypo
chr5	119691235	119692095	Distal Interger	-0669	101E-03	163E-02	C8a	-53560	Hypo
chr10	53449190	53458905	Exon (NM_03:	-0299	101E-03	163E-02	Myh10	54801	Hypo
chr15	85079243	85079874	Distal Interger	-0741	101E-03	163E-02	Slitrk1	647416	Hypo
chr6	70505003	70508282	Intron (NM_0:	-0380	101E-03	163E-02	Akap6	320828	Hypo
chr4	83263803	83264370	Distal Interger	-0790	101E-03	163E-02	Chn2	-112541	Hypo
chr1	121288805	121289994	Intron (NM_0(	-0604	101E-03	163E-02	Ttc23	33073	Hypo
chr12	20340149	20341200	Exon (NM_05:	-0637	102E-03	163E-02	Sh2b2	-87843	Hypo
chr12	5467404	5470392	Distal Interger	-0487	102E-03	163E-02	Hsph1	76414	Hypo
chr9	92455427	92455941	Distal Interger	-0761	102E-03	163E-02	Twist2	80687	Hypo
chr18	78332257	78334152	5' UTR	-0418	102E-03	163E-02	Timm21	-12895	Hypo
chr7	45934234	45935067	Distal Interger	-0586	102E-03	163E-02	E2f7	-215510	Hypo
chr6	60903384	60907526	Distal Interger	-0331	102E-03	163E-02	Dnajb9	367537	Hypo
chr16	46673254	46673785	Distal Interger	-0773	102E-03	163E-02	Sorbs2	-46806	Hypo
chr15	95943476	95944405	Intron (NM_0(	-0684	102E-03	163E-02	Cldn10	80691	Hypo
chr20	22325135	22325637	Distal Interger	-0683	102E-03	163E-02	Reep3	831267	Hypo
chr4	65515814	65521789	Intron (NM_1!	-0379	102E-03	164E-02	Ptn	-140508	Hypo
chr11	50262626	50263493	Distal Interger	-0641	102E-03	164E-02	Bbx	-217120	Hypo
chr8	115995841	115997449	Distal Interger	-0517	102E-03	164E-02	Tgfbr2	-112603	Hypo
chr13	71656035	71656331	Distal Interger	-0939	102E-03	164E-02	Mir1843b	104487	Hypo
chr18	61733136	61734437	Intron (NM_0(	-0593	103E-03	164E-02	Ldlrad4	86425	Hypo
chr13	82267467	82268092	Distal Interger	-0717	103E-03	164E-02	Ddr2	-23927	Hypo
chr19	13992082	13993513	Distal Interger	-0596	103E-03	165E-02	Ces1a	32406	Hypo
chr5	135520912	135525267	3' UTR	-0350	103E-03	165E-02	Heyl	12752	Hypo
chr7	99674523	99675369	Distal Interger	-0671	103E-03	165E-02	Zfat	378905	Hypo
chr14	70819765	70821253	Distal Interger	-0435	103E-03	165E-02	Hs3st1	-393766	Hypo
chr15	35205773	35208001	Distal Interger	-0427	103E-03	165E-02	Tnfrsf19	-47301	Hypo
chr14	87011762	87015291	Distal Interger	-0345	103E-03	165E-02	Cobl	-32223	Hypo
chr16	6747962	6748460	Intron (NM_0	-0764	103E-03	165E-02	Colq	38647	Hypo
chr2	81197657	81199184	Distal Interger	-0569	104E-03	165E-02	Ctnnd2	-130470	Hypo
chr10	14573121	14578594	Distal Interger	-0295	104E-03	165E-02	Sox8	11224	Hypo
chr2	135118983	135121252	Distal Interger	-0407	104E-03	165E-02	Noct	-149937	Hypo

chr8	110174778	110176432	Intron (NM_0(	-0536	104E-03	165E-02	Cspg5	-44221	Hypo
chr10	89456362	89459359	Distal Interger	-0406	104E-03	165E-02	Cdc27	-6595	Hypo
chr11	27340526	27341875	Intron (NM_0(	-0591	104E-03	166E-02	Grik1	228398	Hypo
chr13	38282276	38283339	Distal Interger	-0573	104E-03	166E-02	Mgat5	-392780	Hypo
chr1	167283357	167286132	Distal Interger	-0497	105E-03	166E-02	Bmal1	-45688	Hypo
chr13	81962749	81964422	Distal Interger	-0539	105E-03	166E-02	Rgs4	-19681	Hypo
chr15	15331506	15333609	Intron (NM_0:	-0474	105E-03	166E-02	Cfap20dc	-897690	Hypo
chr3	108242157	108244096	Promoter (<=:	-0389	105E-03	166E-02	Tp53bp1	0	Hypo
chr15	99140963	99145920	Distal Interger	-0356	105E-03	166E-02	Timm8a2	13553	Hypo
chr13	90809436	90812168	Intron (NM_0(	-0447	105E-03	166E-02	Smyd3	454041	Hypo
chr17	12315278	12316116	Distal Interger	-0701	105E-03	167E-02	Auh	-19396	Hypo
chr5	113872139	113878607	Intron (NM_0(	-0357	105E-03	167E-02	Atg4c	4921	Hypo
chr6	138496122	138498445	Distal Interger	-0383	105E-03	167E-02	Rapgef5	-103329	Hypo
chr18	75034966	75035365	Distal Interger	-0813	105E-03	167E-02	Sall3	-621138	Hypo
chrX	42650007	42651819	Distal Interger	-0549	106E-03	167E-02	Prrg1	-43461	Hypo
chr4	53018492	53019493	Distal Interger	-0510	106E-03	167E-02	Ndufa5	-12812	Hypo
chr11	53041647	53043087	Distal Interger	-0570	106E-03	167E-02	Morc1	-657392	Hypo
chr1	234535680	234536347	Distal Interger	-0719	106E-03	167E-02	Tnks2	-31843	Hypo
chr16	21186555	21188031	Distal Interger	-0468	106E-03	168E-02	Csgalnact1	-47977	Hypo
chr5	38729673	38731611	Distal Interger	-0439	106E-03	168E-02	Gpr63	48261	Hypo
chr4	179127411	179129634	Intron (NM_0:	-0512	106E-03	168E-02	Sspn	230264	Hypo
chr6	76036179	76038828	Distal Interger	-0379	106E-03	168E-02	Clec14a	-151567	Hypo
chr7	115136449	115138253	Distal Interger	-0499	106E-03	168E-02	Sult4a1	101832	Hypo
chr6	57686176	57688401	Distal Interger	-0450	106E-03	168E-02	Ifrd1	-397270	Hypo
chr6	36438118	36438491	Distal Interger	-0909	107E-03	168E-02	Ddx1	-410778	Hypo
chr17	16528671	16529325	Distal Interger	-0663	107E-03	168E-02	Id4	-136715	Hypo
chr15	16607910	16611810	Distal Interger	-0323	107E-03	168E-02	Fam107a	-4523	Hypo
chr2	24314842	24318560	Distal Interger	-0338	107E-03	168E-02	Tent2	113509	Hypo
chr16	56356025	56356627	Distal Interger	-0747	107E-03	168E-02	Cldn23	-57050	Hypo
chr10	27857901	27860564	Distal Interger	-0453	107E-03	168E-02	Mir146a	-9291	Hypo
chr8	36687455	36689832	Intron (NM_0(	-0461	107E-03	168E-02	Pknox2	10532	Hypo
chr10	63729224	63733080	Intron (NM_0(	-0349	107E-03	168E-02	Lyrm9	3481	Hypo
chr3	126576293	126576930	Distal Interger	-0643	107E-03	168E-02	Sptlc3	-270948	Hypo

chr2	153081929	153085328	Distal Interger	-0409	107E-03	169E-02	Il12a	116160	Hypo
chr1	3153735	3156099	Distal Interger	-0403	107E-03	169E-02	Ust	-191691	Hypo
chr5	112242852	112246868	Distal Interger	-0344	107E-03	169E-02	Nfia	-194886	Hypo
chr4	167022491	167027414	Intron (NM_0(	-0356	107E-03	169E-02	Etv6	173460	Hypo
chr3	150699098	150701323	Intron (NM_0(	-0429	108E-03	169E-02	Ptprt	287340	Hypo
chr3	62902424	62903324	Distal Interger	-0527	108E-03	170E-02	Zfp385b	-274981	Hypo
chr9	56987027	56990908	Intron (NM_0!	-0382	108E-03	170E-02	Plcl1	85454	Hypo
chr10	73266928	73267572	Distal Interger	-0703	108E-03	170E-02	Ccdc182	176700	Hypo
chr1	205469147	205472272	Distal Interger	-0382	108E-03	170E-02	Slc22a8	-25873	Hypo
chr12	42984857	42986999	Distal Interger	-0459	108E-03	170E-02	Cmklr1	-33001	Hypo
chr6	57677371	57679759	Distal Interger	-0402	108E-03	170E-02	Ifrd1	-388465	Hypo
chr17	8438995	8440434	Distal Interger	-0524	108E-03	170E-02	Tifab	5471	Hypo
chr17	67309219	67310656	Intron (NM_0(	-0547	108E-03	170E-02	Prkcq	68048	Hypo
chr9	81096250	81096670	Distal Interger	-0786	109E-03	170E-02	Wdfy1	-3457	Hypo
chr11	68875569	68894474	3' UTR	-0289	109E-03	171E-02	LOC10254977	-8561	Hypo
chr4	179030340	179036272	3' UTR	-0356	109E-03	171E-02	Sspn	133193	Hypo
chr1	121922942	121924044	Distal Interger	-0599	109E-03	171E-02	LOC682259	309555	Hypo
chr12	43756476	43756834	Distal Interger	-0979	109E-03	171E-02	Grk3	131579	Hypo
chr7	96289192	96290074	Distal Interger	-0621	109E-03	171E-02	Asap1	-230823	Hypo
chr8	10464860	10465936	Distal Interger	-0631	109E-03	171E-02	Mtmr2	-152057	Hypo
chr18	14953756	14956012	Distal Interger	-0419	109E-03	171E-02	Mapre2	-115359	Hypo
chr9	54761617	54763058	Distal Interger	-0459	109E-03	171E-02	Slc39a10	-147944	Hypo
chr4	126685087	126686928	Intron (NM_0(	-0474	110E-03	171E-02	Magi1	123831	Hypo
chr14	95941862	95943028	Intron (NM_0(	-0587	110E-03	171E-02	Otx1	143913	Hypo
chr5	143366898	143368035	Distal Interger	-0573	110E-03	171E-02	Matn1	249394	Hypo
chr5	133556353	133565599	Distal Interger	-0288	110E-03	171E-02	RGD1563049	88221	Hypo
chr11	61686460	61687363	Distal Interger	-0673	110E-03	171E-02	Igsf11	180985	Hypo
chr9	66294601	66295051	Distal Interger	-0937	110E-03	171E-02	Cryge	137470	Hypo
chr2	118910156	118911382	Distal Interger	-0622	110E-03	171E-02	Acad9	-31788	Hypo
chr17	5010710	5013853	Distal Interger	-0352	110E-03	171E-02	Spata31d1	67956	Hypo
chr6	138579300	138580042	Distal Interger	-0648	110E-03	171E-02	Rapgef5	-21732	Hypo
chr5	47492975	47494806	Distal Interger	-0426	110E-03	172E-02	Gabrr1	-29345	Hypo
chr2	28891675	28895872	Distal Interger	-0312	110E-03	172E-02	Hexb	-387510	Hypo

chr14	53610974	53611283	Distal Interger	-0932	110E-03	172E-02	Pcdh7	-711501	Hypo
chr8	121897173	121897859	Distal Interger	-0802	110E-03	172E-02	Abhd5	-102510	Hypo
chr4	169733678	169737331	Distal Interger	-0431	111E-03	172E-02	Art4	13334	Hypo
chr12	27553045	27556412	Distal Interger	-0395	111E-03	172E-02	Ran	121836	Hypo
chr15	92822583	92823061	Intron (NM_0(	-0813	111E-03	172E-02	Gpc5	583037	Hypo
chr2	62194964	62198371	Intron (NM_0	-0352	111E-03	172E-02	Cdh6	26857	Hypo
chr14	74773451	74776047	Intron (NM_0:	-0386	111E-03	172E-02	Afap1	35072	Hypo
chr6	15082848	15086130	Distal Interger	-0365	111E-03	172E-02	Atl2	-52941	Hypo
chr11	4749407	4750617	Intron (NM_0(	-0539	111E-03	172E-02	Cadm2	29715	Hypo
chrX	71215641	71217367	Distal Interger	-0512	111E-03	172E-02	Pgk1	-54087	Hypo
chr7	25496197	25497555	3' UTR	-0497	111E-03	173E-02	Apaf1	81985	Hypo
chr18	34128899	34129611	Intron (NM_0(	-0710	111E-03	173E-02	Sh3rf2	82020	Hypo
chr17	23009049	23009533	Distal Interger	-0815	111E-03	173E-02		-25265	Hypo
chr8	48312761	48313959	Distal Interger	-0623	111E-03	173E-02	Nxpe4	-408554	Hypo
chr6	129865222	129867546	Distal Interger	-0330	111E-03	173E-02	Mok	-10598	Hypo
chr3	11626788	11633622	Distal Interger	-0328	112E-03	173E-02	Olfm1	93480	Hypo
chr2	28887615	28890301	Distal Interger	-0418	112E-03	173E-02	Hexb	-383450	Hypo
chr13	38528880	38529830	Distal Interger	-0602	112E-03	173E-02	Mgat5	-146289	Hypo
chr9	11698852	11699495	Distal Interger	-0514	112E-03	174E-02	Mocs1	-131062	Hypo
chr4	30035557	30038730	Promoter (<=1	-0329	112E-03	174E-02	Cyp51	0	Hypo
chr1	33450786	33452218	Distal Interger	-0532	113E-03	174E-02	Med10	23506	Hypo
chr6	55589144	55593296	Promoter (<=1	-0319	113E-03	174E-02	Etv1	0	Hypo
chr1	244801487	244804420	Distal Interger	-0395	113E-03	174E-02	Hps6	-48836	Hypo
chr1	225241576	225242496	Distal Interger	-0675	113E-03	174E-02	Pum3	-127581	Hypo
chr2	230683215	230685298	Distal Interger	-0452	113E-03	174E-02	Bmpr1b	-108616	Hypo
chr9	62323432	62324715	Exon (NM_03:	-0576	113E-03	174E-02	Ctla4	3996	Hypo
chr4	117841716	117843428	Intron (NM_1!	-0530	113E-03	174E-02	Sfxn5	26354	Hypo
chr2	58117004	58123602	Promoter (<=1	-0293	113E-03	174E-02	Nadk2	0	Hypo
chr11	57154237	57158847	Intron (NM_0(	-0333	113E-03	174E-02	Mir568	-100164	Hypo
chr1	15490422	15494225	Promoter (<=1	-0305	113E-03	174E-02	Pde7b	0	Hypo
chr2	41501691	41506349	Intron (NM_0(	-0330	113E-03	174E-02	Pde4d	279005	Hypo
chr17	20454333	20457044	Distal Interger	-0434	113E-03	175E-02	LOC690414	106584	Hypo
chr3	43884536	43886474	Distal Interger	-0415	113E-03	175E-02	Dapl1	-74519	Hypo

chr2	224151276	224151878	Distal Intergen	-0850	114E-03	175E-02	Nfkb1	-19445	Hypo
chr2	151489828	151490206	Intron (NM_0(	-0789	114E-03	175E-02	Mlf1	-158334	Hypo
chr18	78462255	78464648	Distal Intergen	-0360	114E-03	175E-02	Timm21	-142893	Hypo
chr5	49701430	49702104	Intron (NM_0(	-0692	114E-03	175E-02	Ifnk	-29969	Hypo
chr8	48108764	48110476	Intron (NM_0(	-0436	114E-03	175E-02	Cadm1	260928	Hypo
chr19	10874938	10876126	Distal Intergen	-0585	114E-03	175E-02	Mt4	-9029	Hypo
chr11	78647401	78650157	Exon (NM_00:	-0427	114E-03	175E-02	Etv5	38691	Hypo
chr15	95140795	95141245	Distal Intergen	-0855	114E-03	175E-02	Tgds	54332	Hypo
chr6	50748081	50748785	Distal Intergen	-0581	114E-03	175E-02	Twist1	73171	Hypo
chr1	132521657	132525156	Distal Intergen	-0443	114E-03	176E-02	Ntrk3	-18371	Hypo
chr20	33446886	33448113	Distal Intergen	-0594	115E-03	176E-02	Man1a1	-62012	Hypo
chr6	40026607	40032511	Distal Intergen	-0357	115E-03	177E-02	Pdia6	-30469	Hypo
chr6	138475241	138477710	Distal Intergen	-0411	115E-03	177E-02	Rapgef5	-124064	Hypo
chr11	32073461	32074390	Distal Intergen	-0632	115E-03	177E-02	Runx1	-138030	Hypo
chr6	56729179	56729937	Distal Intergen	-0626	115E-03	177E-02	Arl4a	354219	Hypo
chr16	74147110	74147603	Distal Intergen	-0801	115E-03	177E-02	Myom2	442537	Hypo
chr2	195321996	195328660	Promoter (<=:	-0289	115E-03	177E-02	Ahcyl1	0	Hypo
chr8	22884184	22886003	Distal Intergen	-0499	116E-03	177E-02	Dpy19l1	135458	Hypo
chr15	24057657	24058102	Intron (NM_0:	-0771	116E-03	177E-02	Parp2	23551	Hypo
chr3	61021104	61022955	Intron (NM_0(	-0382	116E-03	177E-02	Ift70a1	-115754	Hypo
chr3	149843326	149844684	Distal Intergen	-0536	116E-03	178E-02	Chd6	-85571	Hypo
chr2	231033158	231034019	Intron (NM_0:	-0677	116E-03	178E-02	Pdlim5	86204	Hypo
chr19	36541393	36543532	Distal Intergen	-0449	116E-03	178E-02	Pmfbp1	-915926	Hypo
chr12	13737381	13739451	Distal Intergen	-0550	116E-03	178E-02	Gna12	-66247	Hypo
chr8	43903361	43904557	Distal Intergen	-0529	116E-03	178E-02	Nectin1	-197219	Hypo
chr13	71629362	71631195	Distal Intergen	-0438	116E-03	178E-02	Mir1843b	77814	Hypo
chr7	125155619	125158467	Distal Intergen	-0354	116E-03	178E-02	Prickle1	-497506	Hypo
chr4	46241808	46246773	Intron (NM_0(	-0319	116E-03	178E-02	ST7	199490	Hypo
chr3	103958389	103960193	Distal Intergen	-0463	116E-03	178E-02	Spred1	-23836	Hypo
chr17	76078874	76079291	Intron (NM_0:	-0814	116E-03	178E-02	Pter	20371	Hypo
chr6	6967029	6968855	Distal Intergen	-0460	116E-03	178E-02	Epcam	-70926	Hypo
chr8	113324859	113327755	Distal Intergen	-0408	117E-03	178E-02	Pdcd6ip	319018	Hypo
chr10	8219379	8219870	Intron (NM_0(	-0690	117E-03	178E-02	Rbfox1	272903	Hypo

chr19	1628592	1629947	Distal Interger	-0496	117E-03	179E-02	Cdh11	-518500	Hypo
chr9	54814498	54817099	Distal Interger	-0355	117E-03	179E-02	Slc39a10	-93903	Hypo
chr9	3443003	3444301	Intron (NM_0(	-0614	117E-03	179E-02	Plcl2	149635	Hypo
chr7	126787062	126788939	Distal Interger	-0518	117E-03	179E-02	Nell2	-124368	Hypo
chr2	175127811	175128471	Distal Interger	-0697	117E-03	179E-02	Adar	-26802	Hypo
chr17	5279074	5283622	Distal Interger	-0348	118E-03	179E-02	Agtpbp1	145376	Hypo
chr17	19268273	19268627	Distal Interger	-0974	118E-03	179E-02	Mylip	39668	Hypo
chr15	80519150	80534449	5' UTR	-0322	118E-03	180E-02	Slain1	20503	Hypo
chr7	86198686	86200620	Distal Interger	-0532	118E-03	180E-02	Enpp2	81854	Hypo
chr12	46282427	46283959	Distal Interger	-0497	118E-03	180E-02	Lrcol1	53994	Hypo
chrX	34154388	34155583	Distal Interger	-0630	118E-03	180E-02	Ppef1	119109	Hypo
chr10	37720158	37723079	Promoter (2-3	-0349	118E-03	180E-02	Kif3a	-2891	Hypo
chr18	82066481	82067024	Distal Interger	-0803	118E-03	180E-02	Socs6	66057	Hypo
chr5	133536796	133540984	Distal Interger	-0342	119E-03	180E-02	RGD1563049	68664	Hypo
chr15	47041132	47041676	Distal Interger	-0680	119E-03	181E-02	Fndc3a	790904	Hypo
chr10	92971573	92978737	Intron (NM_0(	-0327	119E-03	181E-02	Cacng5	-134572	Hypo
chr12	13608238	13610192	Distal Interger	-0509	119E-03	181E-02	Gna12	-195506	Hypo
chr8	87716786	87719405	Promoter (1-2	-0415	119E-03	181E-02	Prss35	1805	Hypo
chr14	2931465	2932610	Intron (NM_0(	-0556	119E-03	181E-02	Hfm1	71197	Hypo
chr5	39147956	39150424	Distal Interger	-0396	119E-03	181E-02	Ufl1	-152822	Hypo
chr1	182979127	182989738	Promoter (<=1	-0301	119E-03	181E-02	Rgs10	0	Hypo
chr6	70704642	70709949	Distal Interger	-0328	120E-03	181E-02	Akap6	520467	Hypo
chr15	18816639	18819595	Distal Interger	-0347	120E-03	181E-02	Fermt2	-64861	Hypo
chr13	38573909	38576240	Distal Interger	-0405	120E-03	182E-02	Mgat5	-99879	Hypo
chr2	242405596	242407227	Intron (NM_0	-0449	120E-03	182E-02	St6galnac3	237839	Hypo
chr18	62547879	62550612	Distal Interger	-0418	120E-03	182E-02	Tcf4	-508844	Hypo
chr4	113402324	113403587	Distal Interger	-0556	121E-03	183E-02	Lrrtm4	853764	Hypo
chr3	49093448	49094595	Distal Interger	-0543	121E-03	183E-02	Fign	-332743	Hypo
chr2	32403406	32405949	Distal Interger	-0379	121E-03	183E-02	Slc30a5	-427562	Hypo
chr3	42740903	42743710	Distal Interger	-0343	121E-03	183E-02	Cytip	-14910	Hypo
chr6	121589454	121590648	Distal Interger	-0537	121E-03	183E-02	Lgmn	-19434	Hypo
chr7	46451113	46452301	Distal Interger	-0518	121E-03	183E-02	Zdhhc17	-17478	Hypo
chr6	10605210	10607968	Distal Interger	-0372	122E-03	184E-02	Zfp36l2	115178	Hypo

chr10	10691817	10698062	Promoter (<=1	-0275	122E-03	184E-02	Ubald1	0	Hypo
chr10	72705534	72707779	Promoter (1-2	-0466	122E-03	184E-02	Olr1523	1434	Hypo
chr3	90718096	90722394	Distal Interger	-0396	122E-03	184E-02	Hipk3	143918	Hypo
chr2	210584937	210597590	Intron (NM_0(	-0294	122E-03	184E-02	Bcar3	59677	Hypo
chr4	167929476	167930934	Distal Interger	-0520	123E-03	184E-02	Gprc5d	24682	Hypo
chr6	14153087	14154201	Distal Interger	-0584	123E-03	185E-02	Tmem178a	-89568	Hypo
chr13	99211480	99212412	Distal Interger	-0598	123E-03	185E-02	Esrrg	-11959	Hypo
chr1	211798733	211799684	Promoter (<=1	-0377	123E-03	185E-02	Tle4	-871	Hypo
chr2	8619677	8620745	Distal Interger	-0591	123E-03	185E-02	Nr2f1	-569340	Hypo
chr8	47211343	47212518	Distal Interger	-0414	123E-03	185E-02	LOC500990	-600869	Hypo
chr7	122559650	122561094	Intron (NM_1:	-0530	123E-03	185E-02	Slc2a13	165511	Hypo
chr17	53731807	53732728	Distal Interger	-0578	123E-03	185E-02	Lyzl1	-187941	Hypo
chr8	75866529	75868648	Intron (NM_0:	-0421	123E-03	185E-02	Myo5a	54117	Hypo
chr3	114815200	114816081	Distal Interger	-0591	124E-03	186E-02	Mrps5	-4965	Hypo
chr2	59378127	59380007	Distal Interger	-0366	124E-03	186E-02	Agxt2	41875	Hypo
chr2	117243408	117245399	Distal Interger	-0433	124E-03	186E-02	Sox2	-291530	Hypo
chr11	33105430	33119536	Exon (NM_001	-0281	124E-03	186E-02	Morc3	-32543	Hypo
chr3	114878861	114879652	Intron (NM_0	-0593	124E-03	186E-02	Mal	8484	Hypo
chr14	85790125	85791268	Intron (NM_0(	-0551	124E-03	186E-02	Vwc2	105711	Hypo
chr13	9381168	9381531	Intron (NM_0(	-0899	124E-03	186E-02	Selenok-ps2	301714	Hypo
chr10	56308636	56311846	Distal Interger	-0417	124E-03	187E-02	Wscd1	-84028	Hypo
chr12	16313630	16316581	Promoter (<=1	-0334	125E-03	187E-02	Akap17a	0	Hypo
chr12	32098344	32099901	Exon (NM_001	-0398	125E-03	187E-02	Snrnp35	19490	Hypo
chr4	53765189	53765841	Distal Interger	-0702	125E-03	187E-02	Tmem229a	-319862	Hypo
chr6	137835888	137836523	Intron (NM_0:	-0713	125E-03	187E-02	Mir153	-327072	Hypo
chr4	119758768	119760342	Intron (NM_0(	-0496	125E-03	187E-02	Antxr1	17890	Hypo
chr1	3518337	3519964	Distal Interger	-0491	125E-03	187E-02	Ust	-556293	Hypo
chr12	12868892	12869600	Distal Interger	-0817	125E-03	187E-02	Foxk1	-693803	Hypo
chr2	202991966	202993161	Distal Interger	-0581	125E-03	187E-02	Olfm3	81373	Hypo
chr2	12175161	12177448	Distal Interger	-0431	125E-03	187E-02	Cetn3	85744	Hypo
chr8	53741939	53742640	Distal Interger	-0584	125E-03	187E-02	Poglut3	-35145	Hypo
chr5	54163188	54164513	Distal Interger	-0537	125E-03	187E-02	RGD1306195	-291492	Hypo
chr4	96065363	96066925	Intron (NM_0!	-0516	125E-03	187E-02	Gng12	29117	Hypo

chr4	56679113	56679727	Intron (NM_0:	-0740	125E-03	187E-02	Grm8	42317	Hypo
chr3	162115060	162116309	Distal Intergen	-0618	125E-03	187E-02	Pmepa1	-95266	Hypo
chr10	65510302	65518511	Intron (NM_0	-0284	125E-03	187E-02	Cdk5r1	26036	Hypo
chr16	75493431	75497021	Promoter (<=:	-0372	125E-03	187E-02	Dlgap2	0	Hypo
chr8	113136561	113142158	Distal Intergen	-0311	125E-03	187E-02	Pdcd6ip	504615	Hypo
chr1	122219058	122220390	Distal Intergen	-0507	125E-03	187E-02	LOC682259	13209	Hypo
chr17	4788900	4790164	Distal Intergen	-0461	125E-03	187E-02	Isca1	-115127	Hypo
chr7	21346042	21347276	Intron (NM_0(	-0511	126E-03	187E-02	Stab2	85725	Hypo
chr9	76408505	76414419	Promoter (1-2	-0296	126E-03	187E-02	Cdk5r2	-1832	Hypo
chr15	51399993	51400935	Distal Intergen	-0694	126E-03	187E-02	Gpalpp1	28500	Hypo
chr2	31606914	31608569	Distal Intergen	-0562	126E-03	187E-02	Gtf2h2	44033	Hypo
chr9	86566056	86569708	Distal Intergen	-0357	126E-03	187E-02	Itm2c	20129	Hypo
chr14	99568175	99569349	Distal Intergen	-0611	126E-03	188E-02	Fancl	-680596	Hypo
chr18	38231912	38234257	Intron (NM_0(	-0427	126E-03	188E-02	Kcnn2	117686	Hypo
chr4	130849511	130851167	Distal Intergen	-0513	126E-03	188E-02	Mitf	440294	Hypo
chr6	19992394	19994519	Distal Intergen	-0529	127E-03	188E-02	Rasgrp3	-33906	Hypo
chr4	38842897	38843809	Distal Intergen	-0612	127E-03	188E-02	LOC500028	-924386	Hypo
chr8	100971890	100973991	Distal Intergen	-0489	127E-03	188E-02	Il20rb	35951	Hypo
chr19	20839678	20844079	Distal Intergen	-0381	127E-03	188E-02	Abcc12	290273	Hypo
chr6	72306701	72307315	Distal Intergen	-0723	127E-03	188E-02	Snx6	-32544	Hypo
chr4	162947741	162948882	Promoter (<=:	-0655	127E-03	189E-02	Olr1	0	Hypo
chr14	12701534	12702911	Distal Intergen	-0554	127E-03	189E-02	Anxa3	78723	Hypo
chr2	71263449	71264072	Distal Intergen	-0697	127E-03	189E-02	Cdh18	-2080933	Hypo
chr1	20054824	20057066	Distal Intergen	-0401	127E-03	189E-02	Akap7	-30424	Hypo
chr7	115238665	115241043	Promoter (<=:	-0355	128E-03	189E-02	Sult4a1	0	Hypo
chr16	78990464	78991647	Intron (NM_1:	-0518	128E-03	189E-02		219061	Hypo
chr4	133967910	133970839	Intron (NM_0(	-0423	128E-03	189E-02	Pdzrn3	26120	Hypo
chr2	167050977	167051539	Distal Intergen	-0676	128E-03	189E-02	Tdo2	235972	Hypo
chr14	40369455	40371568	Distal Intergen	-0409	128E-03	190E-02	Shisa3	196676	Hypo
chr15	47082377	47083305	Distal Intergen	-0609	128E-03	190E-02	Fndc3a	749275	Hypo
chr3	47127340	47128615	Distal Intergen	-0501	128E-03	190E-02	Gcg	-4411	Hypo
chr6	72211524	72211924	Distal Intergen	-0446	129E-03	190E-02	Eapp	-17800	Hypo
chr13	65443974	65444866	Intron (NM_0:	-0626	129E-03	190E-02	Lamc1	56626	Hypo

chr10	100504269	100507675	Promoter (<=1	-0430	129E-03	190E-02	Grin2c	0	Hypo
chr7	128802589	128804680	Distal Intergen	-0383	129E-03	190E-02	Rpap3	33870	Hypo
chr6	61450890	61454915	Intron (NM_0	-0321	129E-03	190E-02	Nrcam	48051	Hypo
chr2	31324241	31325326	Exon (NM_00	-0577	129E-03	191E-02	Mccc2	50631	Hypo
chr15	52142881	52143553	Distal Intergen	-0678	130E-03	191E-02	Serp2	-238916	Hypo
chr16	57739874	57741031	Distal Intergen	-0658	130E-03	191E-02	Saraf	294450	Hypo
chr15	16408664	16415637	Intron (NM_0	-0372	130E-03	191E-02	Fam3d	-123852	Hypo
chr4	57296529	57299134	Intron (NM_0	-0357	130E-03	192E-02	Lipogenin	-57164	Hypo
chr9	97437620	97438249	Distal Intergen	-0628	130E-03	192E-02	Slco6b1	-90284	Hypo
chr7	62525863	62526395	Distal Intergen	-0767	130E-03	192E-02	Atp23	203428	Hypo
chr8	23724450	23725953	Intron (NM_0	-0532	130E-03	192E-02	Septin7	4679	Hypo
chr6	68647937	68648953	Distal Intergen	-0619	130E-03	192E-02	G2e3	-115232	Hypo
chr2	24233460	24250364	Distal Intergen	-0279	130E-03	192E-02	Tent2	181705	Hypo
chr14	15963733	15965777	Distal Intergen	-0444	131E-03	192E-02	G3bp2	-21432	Hypo
chr10	21284035	21285267	Intron (NM_0	-0529	131E-03	192E-02	Tenm2	331736	Hypo
chr17	15701865	15703884	Exon (NM_00	-0424	131E-03	192E-02	Fam120a	17632	Hypo
chr5	25450377	25455166	Promoter (<=1	-0316	131E-03	193E-02	Pdp1	0	Hypo
chr4	57145410	57149751	Intron (NM_0	-0323	131E-03	193E-02	Snd1	50166	Hypo
chr3	131489441	131490693	Distal Intergen	-0566	131E-03	193E-02	Snx5	150512	Hypo
chr1	165556449	165558291	Distal Intergen	-0537	131E-03	193E-02	Galnt18	345977	Hypo
chr7	119727281	119730195	Distal Intergen	-0373	132E-03	193E-02	Zdhhc25	34602	Hypo
chr2	98714117	98714691	Distal Intergen	-0720	132E-03	193E-02	Ythdf3	363456	Hypo
chr7	33397806	33399690	Distal Intergen	-0422	132E-03	193E-02	Atp2b1	-336285	Hypo
chr15	82449039	82451713	Distal Intergen	-0392	132E-03	193E-02	Spry2	243611	Hypo
chr19	14378156	14378818	Distal Intergen	-0699	132E-03	194E-02	Irx6	-41753	Hypo
chr3	7171723	7172732	3' UTR	-0554	132E-03	194E-02	Psd4	34568	Hypo
chr2	117407304	117410069	Distal Intergen	-0390	132E-03	194E-02	Sox2	-126860	Hypo
chr8	28352580	28353165	Distal Intergen	-0715	132E-03	194E-02	Snx19	-477364	Hypo
chr8	121617787	121620110	Distal Intergen	-0396	132E-03	194E-02	Pomgnt2	27166	Hypo
chr10	77290925	77292802	Distal Intergen	-0465	132E-03	194E-02	Kif2b	-894546	Hypo
chr13	65783037	65785472	Distal Intergen	-0404	133E-03	194E-02	Rgs8	-30962	Hypo
chr9	77378748	77381833	Distal Intergen	-0356	133E-03	194E-02	Slc4a3	341732	Hypo
chr4	13519371	13520295	Intron (NM_0	-0539	133E-03	194E-02	Lrrc17	11550	Hypo

chr9	15228096	15230094	Distal Interger	-0448	133E-03	194E-02	Mrpl14	78325	Hypo
chr15	87554290	87554675	Distal Interger	-0861	133E-03	195E-02	Slitrk6	15450	Hypo
chr5	16741750	16742037	Intron (NM_0(	-0949	133E-03	195E-02	Rps20	78438	Hypo
chr18	38892843	38895904	Intron (NM_0(	-0384	133E-03	195E-02	Trim36	16955	Hypo
chr15	46082356	46082769	Distal Interger	-0895	133E-03	195E-02	Gfra2	139905	Hypo
chr2	176043328	176045741	Distal Interger	-0380	134E-03	195E-02	S100a16	26923	Hypo
chr3	46729363	46730377	Intron (NM_1	-0633	134E-03	195E-02	Slc4a10	64035	Hypo
chr3	58466005	58467214	Promoter (2-3	-0541	134E-03	196E-02	Chrna1	2618	Hypo
chr7	50518590	50522048	Distal Interger	-0367	134E-03	196E-02	Trhde	-151219	Hypo
chr9	108687613	108689094	Distal Interger	-0472	134E-03	196E-02	Tmem200c	-116969	Hypo
chr16	53911945	53912455	Intron (NM_0(	-0763	134E-03	196E-02	Mir383	-127885	Hypo
chr15	24747482	24747787	Distal Interger	-0968	134E-03	196E-02	Or5au1	-7512	Hypo
chr8	121467543	121472760	Distal Interger	-0322	134E-03	196E-02	Hhatl	-13542	Hypo
chr16	53659032	53663947	Promoter (<=1	-0305	135E-03	196E-02	Sgcz	0	Hypo
chr12	30816559	30816953	Distal Interger	-0741	135E-03	196E-02	Tmem132b	181356	Hypo
chr2	57783575	57786610	Intron (NM_0	-0484	135E-03	196E-02	Slc1a3	43974	Hypo
chr6	4762879	4765774	Distal Interger	-0421	135E-03	196E-02	Fshr	-433051	Hypo
chr2	48796681	48797114	Distal Interger	-0788	135E-03	197E-02		239293	Hypo
chr17	53168770	53170731	Distal Interger	-0429	135E-03	197E-02	Mtpap	170750	Hypo
chr3	133288159	133290712	Intron (NM_0(	-0414	136E-03	197E-02	Naa20	-31352	Hypo
chr12	19640549	19642296	Promoter (<=1	-0512	136E-03	198E-02	Vgf	-208	Hypo
chr18	79903618	79904129	Distal Interger	-0733	136E-03	198E-02	Cbln2	-37283	Hypo
chr14	36244243	36246495	Intron (NM_0	-0386	136E-03	198E-02	Commd8	195203	Hypo
chr12	11960675	11965255	Promoter (<=1	-0295	136E-03	198E-02	Mmd2	0	Hypo
chr1	90651713	90653408	Exon (NM_001	-0485	136E-03	198E-02	Uri1	51248	Hypo
chrX	27026795	27029040	Promoter (<=1	-0474	136E-03	198E-02	Tlr7	0	Hypo
chr10	20457227	20459407	Distal Interger	-0414	137E-03	198E-02	Rars1	-162035	Hypo
chr19	51269370	51274323	Promoter (<=1	-0309	137E-03	198E-02	Spata2L	0	Hypo
chr1	201923255	201924633	Distal Interger	-0500	137E-03	198E-02	Rce1	-20797	Hypo
chr18	50770725	50771945	Distal Interger	-0517	137E-03	198E-02	Prrc1	-40620	Hypo
chr16	67991105	67994434	Intron (NM_0(	-0377	137E-03	199E-02	Zmat4	366768	Hypo
chr12	807073	808926	Distal Interger	-0416	137E-03	199E-02	Stard13	-72469	Hypo
chr4	80816119	80819204	Distal Interger	-0407	138E-03	199E-02	Snx10	203423	Hypo

chr5	61127959	61136048	Intron (NM_0:	-0323	138E-03	199E-02	Gabbr2	152056	Hypo
chr11	24154452	24159499	Intron (NM_0	-0306	138E-03	199E-02	App	76930	Hypo
chr1	165551830	165552394	Distal Interger	-0699	138E-03	199E-02	Galnt18	351874	Hypo
chr8	95478858	95479384	Intron (NM_0(	-0694	138E-03	199E-02	Slc9a9	245953	Hypo
chr9	28961423	28962014	Distal Interger	-0606	138E-03	199E-02	Adgrb3	-812873	Hypo
chr19	39983154	39983477	3' UTR	-0861	138E-03	200E-02	Terf2ip	6189	Hypo
chr17	15400881	15401874	Promoter (<=:	-0650	138E-03	200E-02	Susd3	36	Hypo
chr8	36761181	36764783	Intron (NM_0(	-0351	138E-03	200E-02	Pknox2	26157	Hypo
chr1	13319320	13332851	Distal Interger	-0290	138E-03	200E-02	Hebp2	-117543	Hypo
chr13	42805721	42807469	Intron (NM_0(	-0399	138E-03	200E-02	Ikbke	-68578	Hypo
chr13	78596128	78596730	Distal Interger	-0686	138E-03	200E-02	Tada1	-36178	Hypo
chr15	37082874	37083652	Promoter (<=:	-0485	138E-03	200E-02	Wdfy2	-190	Hypo
chr1	85636150	85637009	Distal Interger	-0531	139E-03	200E-02	Lrfn3	-13810	Hypo
chr5	24096989	24098758	Intron (NM_0(	-0485	139E-03	200E-02	Plekhf2	7684	Hypo
chr19	48734783	48738089	Distal Interger	-0400	139E-03	201E-02	Cox4i1	13103	Hypo
chr14	25541280	25541988	Distal Interger	-0619	139E-03	201E-02	Tecrl	809039	Hypo
chr1	190529164	190529760	Distal Interger	-0742	139E-03	201E-02	Mki67	-6402	Hypo
chr17	81948657	81950565	Distal Interger	-0415	139E-03	201E-02	Msrb2	-23833	Hypo
chr13	33546399	33547272	Distal Interger	-0669	139E-03	201E-02	Ddx18	-803238	Hypo
chr1	185384708	185385476	Exon (NM_00:	-0530	140E-03	201E-02	Plekha1	-42572	Hypo
chr8	118259526	118263308	Exon (NM_00:	-0324	140E-03	201E-02	Golga4	51326	Hypo
chr3	133152222	133152581	Intron (NM_0(	-1002	140E-03	201E-02	Rin2	42338	Hypo
chr17	5033810	5036403	3' UTR	-0441	140E-03	202E-02	Naa35	49957	Hypo
chr10	77820613	77822389	Distal Interger	-0610	140E-03	202E-02	Utp18	1016465	Hypo
chr17	70590821	70591091	Distal Interger	-0887	141E-03	202E-02	Celf2	-818519	Hypo
chr9	110567010	110570771	Intron (NM_0:	-0347	141E-03	202E-02	Dlgap1	149187	Hypo
chr18	67576698	67583190	Distal Interger	-0308	141E-03	202E-02	Mapk4	-15109	Hypo
chr10	98844013	98858773	Exon (NM_00:	-0307	141E-03	202E-02	Cdc42ep4	-46805	Hypo
chr6	129882418	129884443	Distal Interger	-0418	141E-03	202E-02	Cinp	15022	Hypo
chr4	83273300	83275233	Distal Interger	-0494	141E-03	202E-02	Chn2	-101678	Hypo
chr7	50052408	50056321	Intron (NM_0(	-0341	141E-03	203E-02	Trhde	311050	Hypo
chr11	71132493	71134142	Exon (NM_13:	-0447	141E-03	203E-02	Opa1	50929	Hypo
chr2	41400548	41403899	Intron (NM_0(	-0300	141E-03	203E-02	Pde4d	177862	Hypo

chr6	111198981	111200279	Distal Interger	-0451	141E-03	203E-02		316197	Hypo
chr3	150754841	150758706	Intron (NM_0(	-0365	141E-03	203E-02	Ptprt	229957	Hypo
chr6	95109733	95110906	Promoter (<=:	-0519	142E-03	203E-02	Zbtb1	310	Hypo
chr14	19072926	19073690	Intron (NM_0!	-0627	142E-03	203E-02	Slc4a4	110494	Hypo
chr1	220267305	220268732	Intron (NM_0(	-0508	142E-03	203E-02	Mir204	-48199	Hypo
chr1	225795751	225798842	Distal Interger	-0332	142E-03	203E-02	Rfx3	-86909	Hypo
chr2	232795390	232797566	Distal Interger	-0502	142E-03	203E-02	Lmo4	483315	Hypo
chr15	4007815	4008167	Intron (NM_0(	-0825	142E-03	203E-02	Nudt13	14126	Hypo
chr10	15790558	15791755	Distal Interger	-0559	142E-03	204E-02	Cpeb4	-11446	Hypo
chr8	68747785	68748005	Distal Interger	-1046	142E-03	204E-02	LOC10091136	-410828	Hypo
chr1	176973727	176977771	Intron (NM_0	-0343	142E-03	204E-02	Prkcb	141042	Hypo
chr15	18218803	18219507	Promoter (1-2	-0710	142E-03	204E-02	Ptger2	1518	Hypo
chr3	56482369	56485363	Distal Interger	-0377	142E-03	204E-02	Dlx2	-109139	Hypo
chr2	119376215	119378105	Distal Interger	-0455	142E-03	204E-02	Anxa5	-31644	Hypo
chr8	71595157	71596939	Intron (NM_0	-0472	143E-03	204E-02	Lipc	38525	Hypo
chr20	6889493	6896125	Exon (NM_00:	-0309	143E-03	204E-02	Brpf3	18605	Hypo
chr1	128558173	128559893	Distal Interger	-0596	143E-03	204E-02	Slco3a1	-170286	Hypo
chr5	100220292	100221160	Distal Interger	-0640	143E-03	204E-02	Sh3gl2	567103	Hypo
chr8	93989878	93990358	Distal Interger	-0738	143E-03	205E-02	Plod2	905330	Hypo
chr13	62859645	62860781	Exon (NM_00:	-0550	143E-03	205E-02	Hmcn1	223743	Hypo
chr8	99968758	99971901	Promoter (<=:	-0394	143E-03	205E-02	Mras	0	Hypo
chr5	61426668	61428546	Distal Interger	-0409	143E-03	205E-02	Col15a1	-73417	Hypo
chr7	34296123	34296919	Distal Interger	-0662	144E-03	205E-02	Dusp6	203167	Hypo
chr2	903300	903830	Distal Interger	-0691	144E-03	205E-02	Erap1	-3028112	Hypo
chr7	110149575	110150478	Promoter (1-2	-0736	144E-03	205E-02	Cyth4	-1798	Hypo
chr20	44210760	44217418	Intron (NM_0(	-0295	144E-03	205E-02	Mettl24	68364	Hypo
chr1	93960621	93962151	Distal Interger	-0452	144E-03	205E-02	Zfp819	-76097	Hypo
chr16	12250053	12252867	Distal Interger	-0507	144E-03	205E-02	Rgr	563356	Hypo
chr14	100450343	100450860	Distal Interger	-0781	144E-03	206E-02	Vrk2	-15816	Hypo
chr14	31322997	31323702	Distal Interger	-0701	144E-03	206E-02	Paics	-107231	Hypo
chr8	22853072	22855229	Distal Interger	-0409	145E-03	206E-02	Dpy19l1	166232	Hypo
chr11	26161905	26162530	Distal Interger	-0659	145E-03	206E-02	N6amt1	-422220	Hypo
chr16	37441565	37442356	Distal Interger	-0690	145E-03	206E-02	Spcs3	136207	Hypo

chr15	14782232	14783567	Intron (NM_0:	-0509	145E-03	206E-02	Fhit	814350	Hypo
chr10	48102333	48104239	Distal Interger	-0519	146E-03	207E-02	Pmp22	306624	Hypo
chr13	71772574	71773754	Distal Interger	-0564	146E-03	207E-02	Mir1843b	221026	Hypo
chr1	97654546	97664841	Distal Interger	-0292	146E-03	207E-02	Ptpn5	-19792	Hypo
chr17	52777794	52778360	Distal Interger	-0713	146E-03	207E-02	Svil	-10534	Hypo
chr2	34248713	34254230	Distal Interger	-0342	146E-03	207E-02	Cd180	392722	Hypo
chr2	81288794	81289734	Distal Interger	-0569	146E-03	207E-02	Ctnnd2	-39920	Hypo
chr1	43306120	43311626	Intron (NM_0(	-0317	147E-03	207E-02	Ipcef1	35178	Hypo
chr5	165168554	165169491	Distal Interger	-0607	147E-03	207E-02	Actrt2	68001	Hypo
chr8	22886696	22887149	Distal Interger	-0799	147E-03	208E-02	Dpy19l1	134312	Hypo
chr10	39215762	39216240	Promoter (2-3	-0753	147E-03	208E-02	Gm2a	-2981	Hypo
chr7	98223263	98225077	Intron (NM_0(	-0456	147E-03	208E-02	Dnaaf11	20760	Hypo
chr6	10608184	10609747	Distal Interger	-0420	147E-03	208E-02	Zfp36l2	118152	Hypo
chr5	48194275	48198421	Distal Interger	-0336	147E-03	208E-02	Cnr1	-213599	Hypo
chr11	15536222	15537301	Distal Interger	-0506	148E-03	208E-02	Usp25	-66580	Hypo
chr14	91070767	91071202	Distal Interger	-0764	148E-03	208E-02	Egfr	-105865	Hypo
chr7	130911083	130916493	Intron (NM_0(	-0347	148E-03	208E-02	Cers5	-13136	Hypo
chr4	53030820	53032557	Distal Interger	-0555	148E-03	208E-02	Ndufa5	-25140	Hypo
chr17	70903063	70911793	Distal Interger	-0281	148E-03	208E-02	Celf2	-497817	Hypo
chr9	106302615	106310730	Distal Interger	-0288	148E-03	208E-02	Rab12	196069	Hypo
chr5	75449587	75449893	Intron (NM_2(	-1059	148E-03	209E-02	Mup4l1	48974	Hypo
chr7	30683665	30686267	Exon (NM_00:	-0345	149E-03	209E-02	Eea1	78672	Hypo
chr9	3466469	3467675	Intron (NM_0(	-0604	149E-03	209E-02	Plcl2	173101	Hypo
chr3	143613542	143616401	Distal Interger	-0368	149E-03	209E-02	Ahcy	-29183	Hypo
chr5	56792199	56795518	Exon (NM_00:	-0358	149E-03	209E-02	Enho	7259	Hypo
chr1	230615540	230616322	Distal Interger	-0693	149E-03	209E-02	Pten	-14981	Hypo
chr3	47551257	47552162	Intron (NM_1:	-0696	149E-03	210E-02	Gca	253874	Hypo
chr1	98898452	98901600	Distal Interger	-0404	150E-03	210E-02	Nav2	-56798	Hypo
chr1	154901478	154908519	Intron (NM_0(	-0268	150E-03	210E-02	Coa4	19262	Hypo
chr2	75760254	75760800	Distal Interger	-0787	150E-03	210E-02	Basp1	103001	Hypo
chr1	8922544	8923512	Distal Interger	-0576	150E-03	210E-02	Vta1	157088	Hypo
chr7	102291827	102292822	Distal Interger	-0488	150E-03	210E-02	Khdrbs3	1453893	Hypo
chr11	56766337	56769013	Distal Interger	-0327	150E-03	211E-02	Zdhhc23	46551	Hypo

chr9	35792407	35796859	Intron (NM_0(	-0324	151E-03	211E-02	Prim2	107635	Hypo
chr15	92595001	92596345	Intron (NM_0(	-0572	151E-03	211E-02	Gpc5	355455	Hypo
chr12	5520121	5522913	Distal Interger	-0460	151E-03	211E-02	Tex26	82236	Hypo
chr14	17875477	17876542	Intron (NM_0(	-0648	151E-03	211E-02	Cox18	9620	Hypo
chr1	173871105	173871796	Promoter (<=:	-0589	151E-03	211E-02	Acsm5	0	Hypo
chr3	47095907	47099931	Distal Interger	-0320	151E-03	211E-02	Gcg	22998	Hypo
chr4	167138791	167139335	Distal Interger	-0856	151E-03	211E-02	Bcl2l14	-78277	Hypo
chr7	32872305	32875046	Distal Interger	-0446	151E-03	211E-02	Ccer1	387112	Hypo
chr8	45359826	45361250	Promoter (<=:	-0344	152E-03	212E-02	Mpzl3	0	Hypo
chr13	54821154	54822778	Distal Interger	-0443	152E-03	212E-02	B3galt2	-566628	Hypo
chr2	25140719	25141267	Intron (NM_0:	-0685	152E-03	212E-02	Arsb	138387	Hypo
chr1	169637698	169639133	Distal Interger	-0511	152E-03	212E-02	Sox6	591889	Hypo
chr8	66087448	66090360	Distal Interger	-0357	152E-03	212E-02	Pif1	-21364	Hypo
chr15	69968218	69971157	Intron (NM_0(	-0381	152E-03	212E-02	Pcdh9	264508	Hypo
chr3	58491356	58516117	3' UTR	-0257	152E-03	212E-02	Chrna1	-21524	Hypo
chr1	234428796	234432486	Distal Interger	-0310	152E-03	212E-02	Ppp1r3c	33475	Hypo
chr2	116544619	116546175	Distal Interger	-0588	153E-03	213E-02	Ttc14	-107420	Hypo
chr5	81320861	81321863	Distal Interger	-0594	153E-03	213E-02	Brinp1	1171284	Hypo
chr16	73671476	73672562	Intron (NM_0(	-0511	153E-03	213E-02	Myom2	917578	Hypo
chr3	131570569	131572730	Distal Interger	-0422	153E-03	213E-02	Snx5	68475	Hypo
chr1	258624211	258629416	Promoter (<=:	-0338	153E-03	213E-02	Emx2	0	Hypo
chr14	6542710	6547043	Intron (NM_0(	-0335	153E-03	213E-02	Mapk10	45003	Hypo
chr8	61491115	61491619	Distal Interger	-0747	153E-03	213E-02	LOC691000	-15046	Hypo
chr1	154938275	154939926	Promoter (<=:	-0313	153E-03	213E-02	Mrpl48	0	Hypo
chr16	287716	290984	Distal Interger	-0359	153E-03	213E-02	Rps24	198079	Hypo
chr9	42111605	42112182	Distal Interger	-0836	154E-03	214E-02	Map4k4	-89438	Hypo
chr1	34125552	34125791	Distal Interger	-1034	154E-03	214E-02	Adcy2	-250120	Hypo
chr19	24951665	24952086	Distal Interger	-0787	154E-03	214E-02	Tbc1d9	-8536	Hypo
chr1	20085002	20089186	Promoter (<=:	-0298	154E-03	214E-02	Akap7	0	Hypo
chr3	116052437	116052673	Distal Interger	-0985	154E-03	214E-02	Tmem87b	-9543	Hypo
chr14	26772349	26773642	Intron (NM_1:	-0513	154E-03	214E-02	Adgrl3	50802	Hypo
chr10	30277273	30277855	Distal Interger	-0749	154E-03	214E-02	Clint1	-20849	Hypo
chr13	87268698	87270467	Intron (NM_0	-0502	155E-03	214E-02	Rgs7	138156	Hypo

chr1	230828289	230828707	Distal Interger	-0741	155E-03	215E-02	Pten	196986	Hypo
chr4	19051241	19051906	Exon (NM_01:	-0794	155E-03	215E-02	Hgf	-305938	Hypo
chr1	50276480	50277161	Intron (NM_0(	-0602	155E-03	215E-02	Cahm	109798	Hypo
chr1	231077092	231077604	Intron (NM_0(	-0784	155E-03	215E-02	Rps4x-ps1	-132673	Hypo
chr19	14878553	14881048	Distal Interger	-0431	155E-03	215E-02	Crnde	234666	Hypo
chr1	145411450	145412666	Intron (NM_0:	-0537	156E-03	215E-02	Dlg2	284275	Hypo
chr4	119523304	119523938	Intron (NM_0(	-0668	156E-03	216E-02	Gfpt1	26547	Hypo
chr16	14981186	14982920	Distal Interger	-0493	156E-03	216E-02	Eloa2l	906672	Hypo
chr5	144764716	144766269	Distal Interger	-0496	156E-03	216E-02	Ptafr	-7129	Hypo
chr6	19906373	19908579	Intron (NM_0(	-0423	156E-03	216E-02	Fam98a	32291	Hypo
chr4	107677418	107678125	Distal Interger	-0710	156E-03	216E-02	LRRTM1	-2023690	Hypo
chr5	33502126	33503222	Intron (NM_0	-0631	157E-03	216E-02	Ttpa	4589	Hypo
chr18	68951614	68953852	Distal Interger	-0385	157E-03	216E-02	Smad7	-34577	Hypo
chr4	180723204	180723794	Distal Interger	-0739	157E-03	216E-02	Ccdc91	320828	Hypo
chr1	132642369	132643640	Distal Interger	-0570	157E-03	216E-02	Mrpl46	63999	Hypo
chr1	159770414	159776346	Promoter (<=:	-0303	157E-03	216E-02	Cckbr	0	Hypo
chrX	152038260	152039954	Promoter (<=:	-0378	157E-03	216E-02	Emd	0	Hypo
chr18	72138217	72141799	Distal Interger	-0347	157E-03	216E-02	Slc14a2	-98755	Hypo
chr3	42625800	42630271	3' UTR	-0366	157E-03	216E-02	Ermn	3545	Hypo
chr12	41125222	41126996	Distal Interger	-0285	157E-03	217E-02	Sirt4	-4266	Hypo
chr3	22255411	22260758	Distal Interger	-0340	157E-03	217E-02	Nek6	-23511	Hypo
chr13	32622450	32625674	Exon (NM_00:	-0341	158E-03	217E-02	Ccdc93	14832	Hypo
chr3	115941435	115942184	Promoter (2-3	-0730	158E-03	217E-02	Mertk	2084	Hypo
chr3	17587079	17587364	Intron (NM_0(	-1027	158E-03	217E-02	Pbx3	94986	Hypo
chr13	58121403	58123165	Distal Interger	-0503	158E-03	218E-02	Brinp3	-290718	Hypo
chr14	36634981	36643251	Intron (NM_0:	-0285	158E-03	218E-02	Gabra4	44199	Hypo
chr4	169742385	169744173	Intron (NM_0(	-0497	158E-03	218E-02	Art4	6492	Hypo
chr10	87838907	87841202	Distal Interger	-0334	158E-03	218E-02	Ccdc103	-5155	Hypo
chr3	103844520	103846489	Distal Interger	-0425	159E-03	219E-02	Spred1	-137540	Hypo
chr7	84288951	84291439	Distal Interger	-0398	159E-03	219E-02	Med30	284222	Hypo
chr8	75976541	75979211	Distal Interger	-0350	159E-03	219E-02	Myo5c	-10317	Hypo
chr17	73870673	73876562	Intron (NM_0(	-0316	159E-03	219E-02	Frmd4a	70082	Hypo
chr3	116761822	116767314	Promoter (<=:	-0325	159E-03	219E-02	Sirpb3	0	Hypo

chr7	95456926	95458134	Distal Interger	-0567	159E-03	219E-02	Gsdmc	147972	Hypo
chr2	169209981	169210344	Distal Interger	-0891	159E-03	219E-02	Tlr2	-3436	Hypo
chr4	136599635	136601808	Distal Interger	-0392	160E-03	219E-02	Cntn6	-753559	Hypo
chr6	40685915	40690308	Distal Interger	-0313	160E-03	219E-02	Itgb1bp1	145439	Hypo
chr3	56362903	56364826	Distal Interger	-0439	160E-03	220E-02	Dlx1	6713	Hypo
chr7	132113900	132115670	Intron (NM_0	-0390	160E-03	220E-02	Acvrl1	-129841	Hypo
chr2	16006478	16007333	Distal Interger	-0664	160E-03	220E-02	Rasa1	-66197	Hypo
chr5	133506955	133507870	Distal Interger	-0564	160E-03	220E-02	RGD1563049	38823	Hypo
chr4	26127543	26129317	Distal Interger	-0389	161E-03	220E-02	Steap4	-27684	Hypo
chr5	99079787	99080218	Distal Interger	-0890	161E-03	220E-02	Bnc2	-63742	Hypo
chr8	121227989	121229475	Distal Interger	-0431	161E-03	220E-02	Lyzl4	23120	Hypo
chr10	30646668	30667514	Exon (NM_00	-0272	161E-03	220E-02	Nipal4	-46028	Hypo
chr4	156102797	156104883	Promoter (<=1	-0360	161E-03	220E-02	Necap1	0	Hypo
chr1	221237810	221238496	Intron (NM_0	-0662	161E-03	221E-02	Cfap95	4467	Hypo
chr4	64157937	64161263	3' UTR	-0329	162E-03	221E-02	Mtpn	25423	Hypo
chr1	204828447	204830432	Distal Interger	-0443	162E-03	221E-02	Lgals12	12327	Hypo
chr20	20638968	20642439	Distal Interger	-0345	162E-03	221E-02	Zfp365	-23801	Hypo
chr17	68236958	68238445	Distal Interger	-0446	162E-03	221E-02	Sfmbt2	-114387	Hypo
chr13	49679728	49680387	Intron (NM_0	-0600	163E-03	222E-02	Ptprc	27831	Hypo
chr2	42335743	42339005	Distal Interger	-0369	163E-03	222E-02	Plk2	366559	Hypo
chr16	74837974	74841828	Exon (NM_05	-0329	163E-03	222E-02	Cln8	-78421	Hypo
chr7	55201362	55202987	Distal Interger	-0602	163E-03	222E-02	Grip1	-109240	Hypo
chr8	59985311	59988148	Exon (NM_00	-0475	163E-03	222E-02	Celf6	9952	Hypo
chr1	24698252	24700394	Distal Interger	-0449	163E-03	222E-02	Trdn	-287839	Hypo
chr12	23262566	23263226	Distal Interger	-0746	163E-03	222E-02	Mir6322	516893	Hypo
chr7	132972017	132973957	Distal Interger	-0464	163E-03	222E-02	Krt1	7887	Hypo
chr1	234507406	234508141	Distal Interger	-0692	163E-03	222E-02	Ppp1r3c	-41445	Hypo
chr2	158046523	158047287	Distal Interger	-0553	163E-03	222E-02	Slitrk3	-349968	Hypo
chr7	83007792	83009318	Distal Interger	-0450	163E-03	222E-02	Eif3h	165118	Hypo
chr3	146474813	146475931	Intron (NM_0	-0559	163E-03	222E-02	Ctnnbl1	86873	Hypo
chr2	174875075	174877569	Promoter (<=1	-0308	163E-03	222E-02	Pmvk	0	Hypo
chr8	106801613	106803122	Promoter (<=1	-0319	164E-03	222E-02	Glyctk	0	Hypo
chr15	99029991	99031159	Intron (NM_0	-0490	164E-03	223E-02	Gpr183	19320	Hypo

chr2	14070733	14072884	Distal Interger	-0415	164E-03	223E-02	Mir9-2	-124840	Hypo
chr1	223077056	223079146	Distal Interger	-0478	164E-03	223E-02	Dmrt1	-63713	Hypo
chr10	54084820	54093331	Distal Interger	-0273	164E-03	223E-02	Rnf227	23766	Hypo
chr10	37667006	37676435	Distal Interger	-0272	164E-03	223E-02	Septin8	-8204	Hypo
chr3	119058226	119059070	Distal Interger	-0593	165E-03	223E-02	Prnp	-127098	Hypo
chr2	183036836	183039629	Exon (NM_01	-0455	165E-03	223E-02	Ctsk	-18957	Hypo
chr15	12780982	12782367	Distal Interger	-0548	165E-03	224E-02	Fezf2	7220	Hypo
chr4	24689732	24692568	Exon (NM_00	-0359	165E-03	224E-02	Mir879	31962	Hypo
chr3	68774523	68776865	Distal Interger	-0374	165E-03	224E-02	Zc3h15	29815	Hypo
chr11	13773608	13774526	Distal Interger	-0496	166E-03	224E-02	Lipi	454466	Hypo
chr9	68895404	68896375	Distal Interger	-0468	166E-03	225E-02	Cps1	281251	Hypo
chr9	98179950	98183064	Intron (NM_0	-0316	166E-03	225E-02	Pam	56918	Hypo
chr16	77731849	77737595	Intron (NM_0	-0311	166E-03	225E-02	Arhgef7	-8036	Hypo
chr6	51327949	51328808	Promoter (2-3	-0560	166E-03	225E-02	Hdac9	-2778	Hypo
chr4	126401806	126402369	Intron (NM_0	-0773	166E-03	225E-02	Magi1	408390	Hypo
chr5	47491543	47492679	Distal Interger	-0546	167E-03	225E-02	Gabrr1	-31472	Hypo
chr14	15128501	15131408	Exon (NM_00	-0310	167E-03	225E-02	Septin11	-137802	Hypo
chr20	28339074	28339817	Exon (NM_05	-0598	167E-03	225E-02	Vsir	57455	Hypo
chr15	99071084	99071555	Intron (NM_0	-0821	167E-03	225E-02	Gpr183	-20605	Hypo
chr9	70546255	70547992	Intron (NM_0	-0484	167E-03	225E-02	Erbb4	47983	Hypo
chr15	55600826	55601595	Distal Interger	-0574	167E-03	226E-02	LOC306079	80494	Hypo
chr1	227596887	227597725	Distal Interger	-0673	167E-03	226E-02	Ranbp6	-19972	Hypo
chr13	81329935	81330503	Distal Interger	-0672	168E-03	226E-02	Nuf2	392262	Hypo
chr2	165166003	165167171	Distal Interger	-0544	168E-03	226E-02	Gask1b	118286	Hypo
chr1	185367177	185368800	Intron (NM_0	-0520	168E-03	226E-02	Btbd16	31448	Hypo
chr2	119364576	119366709	Distal Interger	-0432	168E-03	226E-02	Anxa5	-20005	Hypo
chr6	9993463	9994411	Intron (NM_0	-0630	168E-03	226E-02	Abcg5	28345	Hypo
chr12	16475513	16478577	Distal Interger	-0297	168E-03	226E-02	Cyp3a62	77515	Hypo
chr8	102376657	102377645	Distal Interger	-0631	168E-03	226E-02	Ephb1	567194	Hypo
chr1	171734954	171737552	Intron (NM_0	-0393	168E-03	226E-02	Xylt1	91029	Hypo
chr17	84310213	84313175	Distal Interger	-0336	169E-03	227E-02	Gad2	-450455	Hypo
chr2	59404934	59408943	Distal Interger	-0346	169E-03	227E-02	Dnajc21	29476	Hypo
chr2	188324976	188326389	Distal Interger	-0481	169E-03	227E-02	Trim45	-24121	Hypo

chr6	6454412	6455074	Distal Interger	-0749	169E-03	227E-02	Fbxo11	-83309	Hypo
chr2	215450082	215457599	Distal Interger	-0293	169E-03	227E-02	Camk2d	426078	Hypo
chr1	171357197	171358956	Distal Interger	-0489	169E-03	227E-02	Xylt1	-284969	Hypo
chr14	103155571	103161237	Distal Interger	-0314	169E-03	227E-02	Prorsd1	99011	Hypo
chr5	71640344	71643649	Distal Interger	-0350	169E-03	227E-02	Mir32	-44192	Hypo
chr3	76499700	76502081	Intron (NM_0	-0367	169E-03	227E-02	Ptprj	59588	Hypo
chr11	16043972	16044429	Distal Interger	-0736	169E-03	227E-02	Mir99a	-156014	Hypo
chr7	56198102	56198577	Distal Interger	-0764	169E-03	227E-02	Hmga2	-203318	Hypo
chr17	6768833	6783430	Intron (NM_0(	-0254	170E-03	227E-02	Spock1	27320	Hypo
chr13	73116453	73118074	Distal Interger	-0473	170E-03	227E-02	Rabgap1l	-125087	Hypo
chr14	94597823	94598060	Distal Interger	-1009	170E-03	227E-02	Slc1a4	-37633	Hypo
chr2	214277672	214283394	Intron (NM_0	-0319	170E-03	227E-02	Ugt8	49266	Hypo
chr15	52660867	52664831	Distal Interger	-0355	170E-03	227E-02	Dnajc15	584844	Hypo
chr10	38870731	38875054	5' UTR	-0340	170E-03	228E-02	Cdc42se2	30002	Hypo
chr17	84950056	84952256	Distal Interger	-0399	171E-03	228E-02	Apbb1ip	-30685	Hypo
chrX	12272434	12275292	Intron (NM_0(	-0402	171E-03	228E-02	Tspan7	30809	Hypo
chr10	79000860	79004695	Exon (NM_00:	-0284	171E-03	228E-02	Spag9	57381	Hypo
chr10	26618974	26621233	Intron (NM_1:	-0387	171E-03	228E-02	Gabra1	29378	Hypo
chr7	31816226	31816928	Distal Interger	-0710	171E-03	228E-02	Dcn	-464324	Hypo
chr2	94979706	94981429	Distal Interger	-0452	171E-03	228E-02	Pkia	-506107	Hypo
chr5	124577680	124578886	Intron (NM_1:	-0559	171E-03	228E-02	Faf1	151616	Hypo
chr17	15565219	15567518	Exon (NM_00:	-0379	171E-03	228E-02	Wnk2	60958	Hypo
chr4	117831747	117833865	Intron (NM_1!	-0405	171E-03	228E-02	Sfxn5	35917	Hypo
chr19	51865028	51873105	Distal Interger	-0274	171E-03	229E-02	Acta1	13620	Hypo
chr7	91306816	91307461	Distal Interger	-0689	171E-03	229E-02	Trib1	100237	Hypo
chr3	146531300	146534289	Intron (NM_0(	-0410	172E-03	229E-02	Tti1	132506	Hypo
chr5	73557203	73560732	Distal Interger	-0411	172E-03	229E-02	Or2k2	22895	Hypo
chr6	57632352	57633550	Distal Interger	-0542	173E-03	230E-02	Ifrd1	-343446	Hypo
chr17	59701126	59701539	Distal Interger	-0784	173E-03	230E-02	Chrm3	-303966	Hypo
chr6	106431031	106432777	Distal Interger	-0406	173E-03	230E-02	Lrrc74a	44684	Hypo
chr2	212192992	212193433	Distal Interger	-0804	173E-03	230E-02	Tram1l1	-317697	Hypo
chr8	84649779	84651750	Distal Interger	-0453	173E-03	230E-02	Elovl4	77716	Hypo
chr3	56117592	56119582	Distal Interger	-0430	174E-03	231E-02	Dync1i2	83675	Hypo

chr1	233758538	233761344	Promoter (<=1	-0452	174E-03	231E-02	Htr7	0	Hypo
chr7	86398337	86399682	Distal Interger	-0560	174E-03	231E-02	Taf2	79934	Hypo
chr9	102723676	102724573	Distal Interger	-0614	174E-03	231E-02	Efna5	-128542	Hypo
chr18	76835096	76835545	Distal Interger	-0785	174E-03	231E-02	Zfp516	492696	Hypo
chr17	33822668	33824811	Exon (NM_001	-0453	174E-03	231E-02	Dusp22	6510	Hypo
chr3	166621186	166621942	Distal Interger	-0670	174E-03	231E-02	Psma7	521684	Hypo
chr3	49145972	49146663	Distal Interger	-0686	174E-03	231E-02	Fign	-385267	Hypo
chr1	15590857	15591517	Distal Interger	-0623	175E-03	231E-02	Pde7b	-97957	Hypo
chr7	116310656	116312568	Promoter (<=1	-0395	175E-03	232E-02	Fbln1	74	Hypo
chr17	49707597	49710551	Promoter (<=1	-0372	175E-03	232E-02	Gli3	0	Hypo
chr3	161138467	161139605	Promoter (2-3	-0524	175E-03	232E-02	Aurka	2731	Hypo
chr9	33219742	33223403	Promoter (<=1	-0281	175E-03	232E-02	Ptp4a1	0	Hypo
chr1	144145182	144146457	Distal Interger	-0491	175E-03	232E-02	Picalm	88421	Hypo
chr5	143572722	143573918	Distal Interger	-0447	175E-03	232E-02	Ptpru	450850	Hypo
chr2	158047403	158048286	Distal Interger	-0534	176E-03	232E-02	Slitrk3	-350848	Hypo
chr5	150240803	150245075	Distal Interger	-0322	176E-03	232E-02	Eif4g3	-6067	Hypo
chr2	42204387	42207843	Distal Interger	-0311	176E-03	232E-02	Plk2	235203	Hypo
chr17	73781291	73782393	Intron (NM_00	-0569	176E-03	232E-02	Prpf18	150674	Hypo
chr20	2996764	2998667	Distal Interger	-0447	176E-03	232E-02	Cb707485	25697	Hypo
chr3	125501637	125503243	Distal Interger	-0443	176E-03	232E-02	Btbd3	-109450	Hypo
chr8	78299115	78301219	Intron (NM_00	-0390	176E-03	233E-02	Lrrc1	104880	Hypo
chr4	66218951	66219377	Distal Interger	-0869	176E-03	233E-02	Akr1d1	64684	Hypo
chr3	69717267	69720875	Intron (NM_00	-0339	176E-03	233E-02	Ctnnd1	13623	Hypo
chr7	29820772	29821528	Distal Interger	-0608	177E-03	233E-02	Cradd	131379	Hypo
chr3	166039893	166040359	Distal Interger	-0798	177E-03	233E-02	Cdh26	518849	Hypo
chr17	71270572	71273546	Distal Interger	-0357	177E-03	233E-02	Celf2	-136064	Hypo
chr5	74129712	74131547	Distal Interger	-0416	177E-03	233E-02	Ugcg	96734	Hypo
chr10	73417966	73423528	Distal Interger	-0330	177E-03	233E-02	Akap1	212718	Hypo
chr18	38170982	38172154	Intron (NM_00	-0566	177E-03	234E-02	Kcnn2	56756	Hypo
chr8	53845574	53847126	Exon (NM_001	-0515	178E-03	234E-02	C8h11orf65	49208	Hypo
chr4	14615722	14618791	Intron (NM_05	-0345	178E-03	234E-02	Phtf2	400459	Hypo
chr20	6929142	6931012	Intron (NM_00	-0347	178E-03	234E-02	Pnpla1	10696	Hypo
chr9	98121288	98126032	Promoter (<=1	-0274	178E-03	234E-02	Pam	0	Hypo

chr10	66157678	66158731	Intron (NM_0(	-0535	178E-03	235E-02	Asic2	-19299	Hypo
chr15	91654500	91654974	Distal Interger	-0757	179E-03	235E-02	Mir17	-525655	Hypo
chr2	225497866	225500045	Distal Interger	-0428	179E-03	235E-02	Ppp3ca	331755	Hypo
chr2	144639577	144641220	Distal Interger	-0532	179E-03	236E-02	Mbnl1	-56613	Hypo
chr18	25654714	25655979	Distal Interger	-0612	180E-03	236E-02	Epb41l4a	-167615	Hypo
chr2	49612466	49614378	Intron (NM_0!	-0395	180E-03	236E-02	Hcn1	116695	Hypo
chr11	78641962	78643814	Intron (NM_0(	-0383	180E-03	236E-02	Etv5	33252	Hypo
chr10	46633559	46634844	Distal Interger	-0512	180E-03	236E-02	Specc1	-4164	Hypo
chr5	83592594	83593082	Distal Interger	-0783	180E-03	236E-02	Cdk5rap2	367700	Hypo
chr3	145184006	145187534	Intron (NM_1	-0389	180E-03	236E-02	Dlgap4	8527	Hypo
chr12	28336416	28337586	Intron (NM_1!	-0524	180E-03	236E-02	Tmem132d	65704	Hypo
chr5	115773963	115774915	Intron (NM_0(	-0618	181E-03	236E-02	Raver2	72637	Hypo
chr4	53584571	53585213	Distal Interger	-0666	181E-03	237E-02	Tmem229a	-139244	Hypo
chr17	20704730	20705176	Distal Interger	-0767	181E-03	237E-02	LOC690414	-141102	Hypo
chr5	59397828	59399315	Distal Interger	-0535	181E-03	237E-02	Frmpd1	-43761	Hypo
chr17	72861346	72863680	Intron (NM_0(	-0395	182E-03	238E-02	Camk1d	279367	Hypo
chr1	180353031	180356073	5' UTR	-0397	182E-03	238E-02	Katnip	53094	Hypo
chr2	203615779	203616428	Distal Interger	-0650	182E-03	238E-02	S1pr1	12682	Hypo
chr4	160509536	160512043	Distal Interger	-0375	182E-03	238E-02	Prmt8	105133	Hypo
chr15	923688	924900	Exon (NM_03:	-0579	182E-03	238E-02	Kcnma1	621611	Hypo
chr17	5801512	5804151	Exon (NM_01:	-0389	182E-03	238E-02	Ntrk2	64970	Hypo
chr12	44416942	44417437	Promoter (2-3	-0779	182E-03	238E-02	Miat	2322	Hypo
chr15	38505997	38515488	Intron (NM_0!	-0273	182E-03	238E-02	Msra	161008	Hypo
chr3	104513055	104513709	Distal Interger	-0720	183E-03	238E-02	Rasgrp1	-282999	Hypo
chr1	176094521	176095365	Distal Interger	-0732	183E-03	238E-02	Hs3st2	138360	Hypo
chr4	79777928	79780326	Distal Interger	-0435	183E-03	238E-02	Npvf	-61692	Hypo
chr12	25311438	25313777	Distal Interger	-0443	183E-03	238E-02	Galnt17	-10091	Hypo
chr2	24971437	24972017	Intron (NM_1:	-0663	183E-03	238E-02	Arsb	-30315	Hypo
chr17	20920260	20920889	Distal Interger	-0784	183E-03	239E-02	Cd83	-13557	Hypo
chr18	32953840	32954432	Distal Interger	-0626	183E-03	239E-02	Kctd16	785506	Hypo
chr9	47888472	47889231	Distal Interger	-0725	183E-03	239E-02	Wdr75	-20185	Hypo
chr3	112181308	112183357	Distal Interger	-0456	183E-03	239E-02	Slc24a5	-136602	Hypo
chr9	44916758	44918107	Exon (NR_132	-0523	184E-03	239E-02	Pantr1	25524	Hypo

chr8	79365682	79366988	Distal Interger	-0524	184E-03	239E-02	Eef1a1	-20898	Hypo
chr1	258555731	258556367	Distal Interger	-0594	184E-03	239E-02	Pdzd8	-48903	Hypo
chr1	145423602	145424760	Intron (NM_0:	-0512	184E-03	239E-02	Dlg2	296427	Hypo
chr1	168547294	168549450	Distal Interger	-0408	184E-03	239E-02	Pde3b	-57572	Hypo
chr8	65358788	65359375	Distal Interger	-0649	184E-03	239E-02	Slc24a1	106623	Hypo
chr9	23718559	23720513	Promoter (<=:	-0412	184E-03	239E-02	Gsta1	0	Hypo
chr10	13713864	13715932	Promoter (<=:	-0345	184E-03	239E-02	Syngr3	0	Hypo
chr14	46708871	46709578	Distal Interger	-0568	184E-03	239E-02	Arap2	-9182	Hypo
chr11	55128632	55130229	Promoter (<=:	-0461	184E-03	239E-02	Tmprss7	0	Hypo
chr5	135970978	135971751	Distal Interger	-0637	185E-03	240E-02	Ndufs5	7852	Hypo
chr7	27596112	27596626	Distal Interger	-0604	185E-03	240E-02	Cdk17	-87264	Hypo
chrX	97602925	97604071	Distal Interger	-0530	185E-03	240E-02	Drp2	-3648	Hypo
chr6	22717485	22718447	Distal Interger	-0569	185E-03	240E-02	Ypel5	-45794	Hypo
chr13	61761390	61763502	Distal Interger	-0432	185E-03	240E-02	Pla2g4a	258759	Hypo
chr13	49417128	49426927	Distal Interger	-0259	185E-03	240E-02	Mir181a-1	-58600	Hypo
chr11	8949838	8951420	Intron (NM_0(	-0534	185E-03	240E-02	Gbe1	214976	Hypo
chr19	24009461	24012477	Promoter (<=:	-0303	185E-03	240E-02	Cc2d1a	0	Hypo
chr16	25076715	25086410	Intron (NM_0	-0266	185E-03	240E-02	Cpe	46439	Hypo
chr10	78573188	78573896	Distal Interger	-0694	185E-03	240E-02	Utp18	264958	Hypo
chr1	146416353	146418733	Intron (NM_0:	-0413	185E-03	240E-02	Ccdc90b	-214023	Hypo
chr6	49909553	49912124	Distal Interger	-0363	185E-03	240E-02	Efcab10	-92355	Hypo
chr17	15567633	15568748	Intron (NM_0(	-0530	185E-03	240E-02	Wnk2	63372	Hypo
chr9	82392590	82394587	Distal Interger	-0485	186E-03	240E-02	Cul3	-741077	Hypo
chr6	42724953	42730491	Distal Interger	-0323	186E-03	240E-02	Rnf144a	289219	Hypo
chr1	168469850	168471950	Distal Interger	-0299	186E-03	240E-02	Psma1	-16410	Hypo
chr5	148351848	148354964	Distal Interger	-0333	186E-03	240E-02	Id3	-17820	Hypo
chr17	5505237	5508133	Distal Interger	-0389	186E-03	240E-02	Ntrk2	360988	Hypo
chr15	27787022	27788085	Distal Interger	-0580	186E-03	240E-02	Oxa1l	-25603	Hypo
chr20	18484629	18485602	Distal Interger	-0610	186E-03	240E-02	Mrln	-62819	Hypo
chr3	16775354	16777497	Distal Interger	-0402	186E-03	240E-02	Zbtb43	-11539	Hypo
chr19	45518002	45519619	Distal Interger	-0344	186E-03	241E-02	Plcg2	-27797	Hypo
chr1	203970092	203970384	Distal Interger	-1029	187E-03	241E-02	Rps6ka4	62377	Hypo
chr1	76635851	76637412	Promoter (<=:	-0295	187E-03	241E-02	Nop53	0	Hypo

chr1	184841490	184851301	Promoter (<=1	-0283	187E-03	241E-02	Fgfr2	0	Hypo
chr2	43931838	43933745	Exon (NM_001	-0462	187E-03	241E-02	Ankrd55	17328	Hypo
chr12	14492228	14493778	Intron (NM_0	-0485	188E-03	242E-02	Mad1l1	171336	Hypo
chr3	63978552	63979150	Distal Interger	-0730	188E-03	242E-02	Itga4	-183935	Hypo
chr8	95304660	95305738	Intron (NM_0	-0456	188E-03	242E-02	Slc9a9	71755	Hypo
chr3	135634756	135635173	Distal Interger	-0711	189E-03	243E-02	Foxa2	-160430	Hypo
chr4	115670067	115671904	Distal Interger	-0306	189E-03	243E-02	C4h2orf81	8362	Hypo
chr7	47146215	47148338	Distal Interger	-0421	189E-03	243E-02	Phlda1	178782	Hypo
chr12	46075539	46076444	Exon (NM_001	-0593	189E-03	243E-02	Galnt9	-7473	Hypo
chr1	152853467	152854264	Distal Interger	-0657	189E-03	243E-02	Lrrc32	66956	Hypo
chr5	46899607	46901435	Intron (NM_0	-0458	189E-03	243E-02	Gja10	95135	Hypo
chr17	6593372	6593896	Distal Interger	-0710	189E-03	243E-02	Mir874	-26753	Hypo
chr13	84279703	84281677	Exon (NM_001	-0382	189E-03	243E-02	Slamf1	9387	Hypo
chr2	195052431	195054153	Distal Interger	-0498	190E-03	243E-02	Kcnc4	45080	Hypo
chr15	47083545	47084569	Distal Interger	-0537	191E-03	245E-02	Fndc3a	748011	Hypo
chr4	25817861	25818088	Distal Interger	-0988	191E-03	245E-02	Dbf4	141204	Hypo
chr1	184581903	184583041	Distal Interger	-0474	191E-03	245E-02	Fgfr2	267585	Hypo
chr4	81661180	81661454	Intron (NM_0	-0838	191E-03	245E-02		-20770	Hypo
chr4	46838790	46841383	Intron (NM_0	-0411	191E-03	245E-02	Cttnbp2	123248	Hypo
chr17	70920795	70925037	Distal Interger	-0335	191E-03	245E-02	Celf2	-484573	Hypo
chr13	24636092	24636778	Distal Interger	-0662	192E-03	245E-02	Serpinb8	1009147	Hypo
chr1	101559962	101562343	Intron (NM_0	-0316	192E-03	245E-02	Svip	30475	Hypo
chr5	16716602	16717156	Intron (NM_0	-0867	192E-03	245E-02	Lyn	77090	Hypo
chr8	36641664	36660395	Exon (NM_001	-0265	192E-03	245E-02	Pknox2	39969	Hypo
chr8	69988447	69991365	Distal Interger	-0326	192E-03	245E-02	Ice2	-53205	Hypo
chr18	31635736	31637606	Distal Interger	-0402	192E-03	246E-02	Pabpc2	-16612	Hypo
chr2	91890752	91893245	Distal Interger	-0381	193E-03	246E-02	Fabp5	-122039	Hypo
chr6	7711069	7711723	Distal Interger	-0705	193E-03	246E-02	Tmem247	-9605	Hypo
chr8	48834302	48835214	Promoter (2-3	-0642	193E-03	246E-02	Rbm7	2280	Hypo
chr6	48166637	48168543	Distal Interger	-0414	193E-03	247E-02	Slc26a4	-20934	Hypo
chr1	157125472	157128120	Distal Interger	-0302	194E-03	247E-02	Or51e1	-20179	Hypo
chr6	110662277	110664011	Intron (NM_0	-0487	194E-03	247E-02	Ston2	12365	Hypo
chr16	44567973	44570836	Distal Interger	-0353	194E-03	247E-02	Ing2	-4761	Hypo

chr1	155413642	155415066	Distal Interger	-0449	194E-03	247E-02	Fchsd2	-45863	Hypo
chr5	49700902	49701334	Intron (NM_0(	-0779	194E-03	247E-02	Ifnk	-30739	Hypo
chr7	127694581	127702242	Distal Interger	-0333	194E-03	247E-02	Scaf11	-70666	Hypo
chr3	60951821	60954077	Exon (NM_00:	-0418	194E-03	247E-02	Ift70a1	-46471	Hypo
chr1	155881399	155887960	Promoter (2-3	-0295	194E-03	247E-02	Mir139	2571	Hypo
chr11	4243114	4243859	Distal Interger	-0560	195E-03	248E-02	Cadm2	536473	Hypo
chr13	76775840	76776310	Intron (NM_1:	-0734	195E-03	248E-02	Atp1b1	30786	Hypo
chr4	178672036	178673717	Distal Interger	-0522	195E-03	248E-02	Rassf8	-46275	Hypo
chr5	79224164	79227464	Intron (NM_0(	-0406	195E-03	248E-02	Trim32	218996	Hypo
chr2	16004745	16006369	Distal Interger	-0420	195E-03	248E-02	Rasa1	-64464	Hypo
chr1	43492201	43492727	Distal Interger	-0766	195E-03	248E-02	Cnksr3	98903	Hypo
chr18	75033264	75034574	Distal Interger	-0565	195E-03	248E-02	Sall3	-619436	Hypo
chr17	84987470	84988500	Intron (NM_0(	-0505	195E-03	248E-02	Apbb1ip	4529	Hypo
chr8	103995364	103995723	Distal Interger	-0734	195E-03	248E-02	Cdv3	-48191	Hypo
chr13	71902893	71904583	Distal Interger	-0448	195E-03	248E-02	Tnr	-187002	Hypo
chr3	69587199	69589412	Distal Interger	-0429	196E-03	248E-02	Tfpi	-10361	Hypo
chr14	14944666	14946988	Intron (NM_0(	-0370	196E-03	248E-02	Septin11	43711	Hypo
chr12	32621937	32623124	Distal Interger	-0544	196E-03	248E-02	Hip1r	-3203	Hypo
chr7	42247787	42248177	Distal Interger	-0838	196E-03	248E-02	Acss3	202053	Hypo
chr8	3715496	3716284	Intron (NM_0:	-0572	196E-03	249E-02	Ddi1	-116991	Hypo
chr4	38861022	38863487	Distal Interger	-0412	196E-03	249E-02	LOC500028	-942511	Hypo
chr8	64694361	64697552	Intron (NM_0:	-0311	196E-03	249E-02	Snapc5	17157	Hypo
chr6	111246959	111251058	Distal Interger	-0350	197E-03	249E-02		265418	Hypo
chr17	53833131	53834006	Distal Interger	-0687	197E-03	249E-02	Lyzl1	-289265	Hypo
chr17	21429500	21439272	Intron (NM_0(	-0259	197E-03	249E-02	Gfod1	29358	Hypo
chr7	108759237	108759826	Distal Interger	-0711	197E-03	249E-02	Mb	7308	Hypo
chr1	168484327	168485221	Distal Interger	-0594	197E-03	249E-02	Psma1	-30887	Hypo
chr2	115441887	115443631	Distal Interger	-0429	197E-03	249E-02	Gnb4	-41308	Hypo
chr4	24382898	24384951	Intron (NM_0(	-0368	197E-03	250E-02	Grm3	17783	Hypo
chr4	154022330	154023309	3' UTR	-0658	197E-03	250E-02	Atp6v1e1	21177	Hypo
chr4	10076390	10080688	Distal Interger	-0351	197E-03	250E-02	Prkag2	-125000	Hypo
chr2	28820367	28826521	Distal Interger	-0309	198E-03	250E-02	Hexb	-316202	Hypo
chr3	109688683	109691685	Distal Interger	-0377	198E-03	250E-02	Afg2b	-3072	Hypo

chr1	132328581	132330571	Intron (NM_0(	-0504	198E-03	250E-02	Ntrk3	172715	Hypo
chr7	115998725	116000069	Distal Interger	-0599	198E-03	250E-02	Phf21b	-14510	Hypo
chr13	67192752	67197652	Distal Interger	-0313	198E-03	250E-02	Ier5	74574	Hypo
chr20	21548382	21551445	Exon (NM_00:	-0374	198E-03	250E-02	Reep3	54514	Hypo
chr2	39662198	39665298	Exon (NM_00:	-0410	199E-03	251E-02	Ercc8	14741	Hypo
chr2	14012742	14013449	Distal Interger	-0616	199E-03	251E-02	Mir9-2	-184275	Hypo
chr12	5779114	5780194	Distal Interger	-0498	199E-03	251E-02	Alox5ap	-6128	Hypo
chr1	165034359	165035107	Intron (NM_0(	-0660	199E-03	251E-02	Irag1	25322	Hypo
chr16	46818143	46819274	Distal Interger	-0537	199E-03	251E-02	Tlr3	-3115	Hypo
chr18	54344152	54346940	Distal Interger	-0338	199E-03	251E-02	Arsi	-17476	Hypo
chr7	58406613	58408555	Intron (NM_0(	-0416	200E-03	252E-02	Ppm1h	67952	Hypo
chr9	57370995	57383761	Distal Interger	-0277	200E-03	252E-02	Plcl1	469422	Hypo
chr8	5780824	5782083	Intron (NM_0!	-0460	200E-03	252E-02	Trpc6	21437	Hypo
chr2	26268187	26269379	3' UTR	-0438	201E-03	252E-02	Wdr41	43692	Hypo
chr14	8946736	8951229	Exon (NM_00:	-0350	201E-03	252E-02	Coq2	5275	Hypo
chr2	75758771	75759549	Distal Interger	-0600	201E-03	252E-02	Basp1	104252	Hypo
chr14	95881414	95882230	Intron (NM_0(	-0671	201E-03	252E-02	Otx1	204711	Hypo
chr5	164196556	164198484	Distal Interger	-0555	201E-03	252E-02	Ajap1	-176239	Hypo
chr1	193752684	193757173	Distal Interger	-0274	201E-03	253E-02	Bnip3	-27336	Hypo
chr17	70319395	70320487	Distal Interger	-0588	201E-03	253E-02	Celf2	-1089123	Hypo
chr1	255263188	255263610	Distal Interger	-0821	202E-03	253E-02	Habp2	-52305	Hypo
chr6	27048935	27049922	Distal Interger	-0587	202E-03	253E-02	Dnajc27	-18815	Hypo
chr9	106925953	106926648	Intron (NM_0(	-0774	202E-03	253E-02	Ptprm	417050	Hypo
chr11	55568788	55571741	Exon (NM_21:	-0352	202E-03	253E-02	Btla	15241	Hypo
chr6	77302689	77304597	Distal Interger	-0435	202E-03	254E-02	Fbxo33	-370352	Hypo
chr6	138262892	138265217	Distal Interger	-0340	202E-03	254E-02	Mir153	99297	Hypo
chr1	98706385	98706894	Distal Interger	-0758	202E-03	254E-02	E2f8	-122287	Hypo
chr8	66100379	66106257	Distal Interger	-0319	203E-03	254E-02	Pif1	-5467	Hypo
chr3	166529887	166531526	Distal Interger	-0425	203E-03	254E-02	Psma7	612100	Hypo
chr4	169694631	169695264	Promoter (<=:	-0481	203E-03	254E-02	Wbp11	-200	Hypo
chr8	95960584	95962046	Distal Interger	-0454	203E-03	254E-02	Paqr9	-152203	Hypo
chr5	4958757	4960278	Distal Interger	-0493	203E-03	254E-02	Msc	407421	Hypo
chr8	81165282	81165979	Distal Interger	-0647	203E-03	254E-02	Senp6	176230	Hypo

chr5	94912105	94914224	Distal Intergen	-0410	203E-03	254E-02	Tyrp1	-366758	Hypo
chr5	16722698	16723523	Intron (NM_0	-0700	203E-03	254E-02	Lyn	83186	Hypo
chr3	50589651	50597170	Intron (NM_0	-0272	203E-03	254E-02	Csrnp3	91018	Hypo
chr2	42377446	42378139	Distal Intergen	-0653	203E-03	254E-02	Plk2	408262	Hypo
chr1	95451233	95454037	Exon (NM_00	-0373	203E-03	254E-02	Cpt1c	3244	Hypo
chr11	78493857	78500506	Exon (NM_01	-0285	203E-03	254E-02	Etv5	-108204	Hypo
chr13	40415135	40417657	Distal Intergen	-0391	203E-03	254E-02	Cxcr4	-333252	Hypo
chr17	42703863	42705992	Distal Intergen	-0346	204E-03	254E-02	Hist1h4m	5809	Hypo
chr5	77240074	77241730	Distal Intergen	-0457	204E-03	254E-02	Tnfsf8	35348	Hypo
chr20	36279070	36281114	Distal Intergen	-0427	204E-03	254E-02	Gja1	523035	Hypo
chr16	28797790	28798703	Intron (NM_1	-0607	204E-03	255E-02	Sh3rf1	100859	Hypo
chr13	87123894	87125336	Intron (NM_0	-0494	204E-03	255E-02	Grem2	-252385	Hypo
chr13	63684631	63686932	Intron (NM_0	-0401	204E-03	255E-02	Niban1	10391	Hypo
chr6	110695759	110699886	Distal Intergen	-0344	204E-03	255E-02	Ston2	-19383	Hypo
chr9	80807343	80808962	Promoter (<=	-0435	204E-03	255E-02	Scg2	0	Hypo
chr2	204564391	204569387	Exon (NM_00	-0319	204E-03	255E-02	Trmt13	-17602	Hypo
chr14	79165656	79168976	Distal Intergen	-0413	204E-03	255E-02	Lif	30995	Hypo
chr6	92821538	92824506	Distal Intergen	-0348	205E-03	255E-02	Snapc1	136753	Hypo
chr7	89622229	89623635	Distal Intergen	-0441	205E-03	255E-02	Zhx1	-11288	Hypo
chr7	39601339	39601793	Distal Intergen	-0784	205E-03	255E-02	Slc6a15	1048088	Hypo
chr5	9123845	9125949	Exon (NM_00	-0360	205E-03	255E-02	Cspp1	54817	Hypo
chr5	158752557	158753100	Distal Intergen	-0808	205E-03	255E-02	Disp3	-31751	Hypo
chr17	18125865	18129196	Intron (NM_0	-0343	205E-03	256E-02	Cap2	74149	Hypo
chr10	60195244	60197200	Promoter (1-2	-0417	205E-03	256E-02	Rpa1	-1925	Hypo
chr8	27311505	27312090	Distal Intergen	-0663	206E-03	256E-02	Ntm	492138	Hypo
chr11	33308276	33310402	Distal Intergen	-0449	206E-03	256E-02	Cldn14	-66197	Hypo
chr14	44220620	44222126	Distal Intergen	-0510	206E-03	256E-02	Pgm2	-20819	Hypo
chr1	167460011	167463550	Intron (NM_0	-0340	206E-03	256E-02	Btbd10	25513	Hypo
chr5	146121329	146121682	Distal Intergen	-0914	206E-03	256E-02	Rps6ka1	-3057	Hypo
chrX	58024165	58027816	3' UTR	-0370	206E-03	256E-02	Arx	7932	Hypo
chr8	11145914	11149477	Intron (NM_0	-0358	206E-03	256E-02	Sesn3	11997	Hypo
chr3	33987820	33990855	Distal Intergen	-0358	207E-03	257E-02	Kif5c	-41250	Hypo
chr15	29060860	29062418	Promoter (2-3	-0468	207E-03	257E-02	Fitm1	-2328	Hypo

chr6	109995232	109995993	Distal Interger	-0570	208E-03	258E-02	Dio2	-315423	Hypo
chr18	55242547	55248379	Promoter (<=1	-0279	208E-03	258E-02	Afap1l1	0	Hypo
chr4	118479645	118489936	Distal Interger	-0260	208E-03	258E-02	Add2	-7480	Hypo
chr10	72742449	72744171	Distal Interger	-0420	208E-03	258E-02	Dynll2	30889	Hypo
chr1	21160764	21161694	Distal Interger	-0585	208E-03	258E-02	Stx7	75519	Hypo
chr8	67914792	67915266	Distal Interger	-0701	208E-03	258E-02	Mir190a-2	-63721	Hypo
chr15	82403705	82408809	Distal Interger	-0273	208E-03	258E-02	Spry2	286515	Hypo
chr5	122683293	122687299	Promoter (1-2	-0346	209E-03	259E-02	Cpt2	-1247	Hypo
chr1	190395690	190399757	Distal Interger	-0317	209E-03	259E-02	Ptpre	-50017	Hypo
chr9	74333422	74333840	Distal Interger	-0803	209E-03	259E-02	Rpl37a	34561	Hypo
chr18	34657560	34658786	Intron (NR_07	-0531	209E-03	259E-02	Gpr151	-87259	Hypo
chr4	9518563	9518972	Distal Interger	-0888	209E-03	259E-02	Rpl10a	-70526	Hypo
chr8	5778780	5780674	Intron (NM_0	-0402	209E-03	259E-02	Trpc6	19393	Hypo
chrX	97515368	97515885	Promoter (<=1	-0599	210E-03	259E-02	Cenpi	-501	Hypo
chrX	28091517	28103355	Promoter (<=1	-0261	210E-03	259E-02	Gpm6b	0	Hypo
chr17	40086485	40086883	Intron (NM_0	-0734	210E-03	259E-02	Mrs2	22523	Hypo
chr6	10860861	10862853	Distal Interger	-0444	210E-03	260E-02	Haao	15076	Hypo
chr4	25817427	25817713	Distal Interger	-0869	210E-03	260E-02	Dbf4	140770	Hypo
chr2	59396271	59398960	Distal Interger	-0356	210E-03	260E-02	Dnajc21	39459	Hypo
chr14	92932466	92933173	Distal Interger	-0713	211E-03	260E-02	Meis1	360422	Hypo
chr8	46362863	46369369	Intron (NM_0	-0320	211E-03	260E-02	Sik3	50563	Hypo
chr9	5249253	5249752	Distal Interger	-0869	211E-03	260E-02	Kcnh8	-256292	Hypo
chr6	34060551	34064681	Intron (NM_0	-0343	211E-03	260E-02	Smc6	81785	Hypo
chr5	35840609	35841130	Distal Interger	-0758	211E-03	260E-02	Fbxl4	-114686	Hypo
chr19	14142044	14146645	Exon (NM_00	-0315	211E-03	261E-02	Lpcat2	5945	Hypo
chr5	153229320	153234745	Exon (NM_01	-0326	211E-03	261E-02	Padi2	20267	Hypo
chr5	141112737	141113396	3' UTR	-0669	212E-03	261E-02	A3galt2	11321	Hypo
chr5	97580840	97581805	Distal Interger	-0670	212E-03	261E-02	Ttc39b	126265	Hypo
chr1	144777573	144778654	Distal Interger	-0514	212E-03	261E-02	Tmem126b	-326134	Hypo
chr10	93799958	93803099	Intron (NM_0	-0350	212E-03	261E-02	Axin2	-96146	Hypo
chr13	98202550	98204065	Intron (NM_0	-0521	212E-03	261E-02	Tgfb2	57169	Hypo
chr1	19937871	19940416	Distal Interger	-0390	212E-03	261E-02	Smlr1	100481	Hypo
chr10	60137266	60138679	Distal Interger	-0490	212E-03	261E-02	Rpa1	54640	Hypo

chr2	28513577	28514645	Intron (NM_0(	-0529	213E-03	262E-02	Hexb	-9412	Hypo
chr16	56559221	56559935	Intron (NM_0(	-0635	213E-03	262E-02	Mfhas1	73170	Hypo
chr7	98229247	98230606	Intron (NM_0(	-0540	213E-03	262E-02	Dnaaf11	15231	Hypo
chr13	53557840	53558324	Distal Interger	-0704	213E-03	262E-02	B3galt2	-1831082	Hypo
chr7	105372511	105379567	Distal Interger	-0310	213E-03	262E-02	Ptk2	-89422	Hypo
chr2	134838352	134839315	Distal Interger	-0644	214E-03	262E-02	Slc7a11	-320994	Hypo
chr3	95763663	95764409	Promoter (<=1	-0470	214E-03	262E-02	Kif18a	0	Hypo
chr15	38535019	38540295	Intron (NM_0!	-0284	215E-03	264E-02	Msra	136201	Hypo
chr19	25049668	25050009	Intron (NM_0(	-0841	216E-03	264E-02	Tbc1d9	-106539	Hypo
chr3	104603284	104603876	Distal Interger	-0763	216E-03	264E-02	Rasgrp1	-373228	Hypo
chr17	20390818	20391275	Distal Interger	-0745	216E-03	265E-02	LOC690414	172353	Hypo
chr3	123284186	123285344	Intron (NM_0:	-0539	216E-03	265E-02	Lamp5	-87118	Hypo
chr5	92027829	92028973	Distal Interger	-0538	216E-03	265E-02	Dmac1	-2784072	Hypo
chr16	74940092	74944943	Intron (NM_0!	-0326	217E-03	265E-02	Cln8	-180539	Hypo
chr7	108985939	108992121	Intron (NM_0(	-0259	217E-03	265E-02	Rbfox2	62282	Hypo
chr1	200369143	200370972	Promoter (1-2	-0383	217E-03	265E-02	Smim38	1004	Hypo
chr3	54934949	54935783	Intron (NM_0(	-0615	217E-03	265E-02	Myo3b	61245	Hypo
chr1	148853769	148854820	Distal Interger	-0553	217E-03	265E-02	Fam181b	1687341	Hypo
chr19	38053249	38054803	Promoter (<=1	-0351	218E-03	266E-02	Zfp612	0	Hypo
chr9	56209550	56210646	Exon (NM_001	-0576	218E-03	266E-02	Pgap1	-165225	Hypo
chr6	50597358	50597723	Distal Interger	-0750	218E-03	266E-02	Ferd3l	-47656	Hypo
chr20	6684321	6686260	Promoter (2-3	-0425	218E-03	266E-02	Srpk1	-2214	Hypo
chr3	77073048	77075603	Promoter (<=1	-0408	218E-03	267E-02	Spi1	0	Hypo
chr16	2910658	2911761	Intron (NM_1	-0580	219E-03	267E-02	Erc2	61980	Hypo
chr7	30238861	30239478	Distal Interger	-0635	219E-03	267E-02	Nudt4	-34534	Hypo
chr8	76020230	76023273	Exon (NM_001	-0349	219E-03	267E-02	Myo5c	30702	Hypo
chr17	12762133	12763342	Distal Interger	-0560	219E-03	267E-02	Diras2	4568	Hypo
chr11	44961011	44961584	Distal Interger	-0793	219E-03	267E-02	Nfkbiz	178335	Hypo
chr3	104284410	104285044	Distal Interger	-0653	220E-03	268E-02	Rasgrp1	-54354	Hypo
chr1	98873184	98874750	Distal Interger	-0428	220E-03	268E-02	Nav2	-83648	Hypo
chr8	114117593	114118621	Intron (NM_0(	-0539	221E-03	269E-02	Glb1	32085	Hypo
chr11	29234386	29235690	Distal Interger	-0630	221E-03	269E-02	Tiam1	-74478	Hypo
chr2	230680499	230680825	Distal Interger	-0807	221E-03	269E-02	Bmpr1b	-105900	Hypo

chr15	7618209	7623255	Distal Interger	-0339	221E-03	269E-02	Nr1d2	93952	Hypo
chr1	25682350	25684822	Distal Interger	-0403	221E-03	269E-02	Rnf217	-331846	Hypo
chr5	138114809	138115407	Distal Interger	-0702	221E-03	269E-02	Csf3r	-183273	Hypo
chr8	63533694	63536283	Distal Interger	-0381	222E-03	269E-02	Skor1	71518	Hypo
chr12	39584662	39586901	Distal Interger	-0397	222E-03	269E-02	Taok3	-123418	Hypo
chr7	126197600	126199840	Exon (NM_00:	-0399	222E-03	269E-02	Tmem117	440009	Hypo
chr18	59025897	59033128	Distal Interger	-0289	222E-03	269E-02	Zfp532	-63565	Hypo
chr17	81687042	81688276	Distal Interger	-0476	222E-03	269E-02	Pip4k2a	-19013	Hypo
chr8	70970865	70972871	Intron (NM_1	-0416	222E-03	270E-02	Myo1e	82931	Hypo
chr1	211875693	211876337	Distal Interger	-0674	222E-03	270E-02	Tle4	-77831	Hypo
chr3	15273313	15274422	Intron (NM_0(	-0538	222E-03	270E-02	Nup214	18194	Hypo
chr3	47208288	47209235	Promoter (<=:	-0542	223E-03	270E-02	Fap	-762	Hypo
chr7	56221318	56221613	Distal Interger	-0820	223E-03	270E-02	Hmga2	-226534	Hypo
chr3	111376089	111377072	Distal Interger	-0575	223E-03	270E-02	Sema6d	-506800	Hypo
chr15	12940181	12941442	Intron (NM_1:	-0511	223E-03	270E-02	Cep15	-105133	Hypo
chr2	173699804	173704817	Distal Interger	-0340	223E-03	270E-02	Rhbg	12504	Hypo
chr11	69026067	69029869	Intron (NM_0	-0310	223E-03	270E-02	Dlg1	72934	Hypo
chr6	69578481	69579342	Intron (NM_0(	-0591	223E-03	271E-02	Nubpl	19161	Hypo
chr3	60857796	60859455	Promoter (<=:	-0345	223E-03	271E-02	Ift70b	0	Hypo
chr7	45385671	45387483	Intron (NM_0(	-0462	223E-03	271E-02	Nav3	4776	Hypo
chr13	66022437	66038513	Promoter (<=:	-0275	223E-03	271E-02	Glul	0	Hypo
chr6	68456594	68457572	Distal Interger	-0591	224E-03	271E-02	G2e3	-306613	Hypo
chr14	94117841	94118290	Distal Interger	-0787	224E-03	271E-02	Spred2	-31404	Hypo
chr17	67310937	67312258	Intron (NM_0(	-0548	224E-03	271E-02	Prkcq	66446	Hypo
chr2	10343217	10343462	Distal Interger	-0890	224E-03	271E-02	Arrdc3	-794238	Hypo
chr9	11166335	11167230	Distal Interger	-0623	225E-03	272E-02	Rftn1	361724	Hypo
chr14	12572452	12580001	Distal Interger	-0276	225E-03	272E-02	Paqr3	109822	Hypo
chr3	116630509	116635249	Distal Interger	-0310	225E-03	272E-02	Il1b	-47123	Hypo
chr19	27005266	27005912	Promoter (<=:	-0409	225E-03	272E-02	Usp38	-134	Hypo
chr1	150287651	150288569	Distal Interger	-0611	225E-03	272E-02	Mir708	-311307	Hypo
chr8	123531533	123533220	Distal Interger	-0423	225E-03	272E-02	Ccr1	28621	Hypo
chr17	73850684	73852679	Intron (NM_0(	-0420	225E-03	272E-02	Frmd4a	93965	Hypo
chr19	54380068	54381782	Distal Interger	-0573	225E-03	272E-02	Kcnk1	420411	Hypo

chr13	34945605	34946700	Intron (NM_0(	-0542	225E-03	272E-02	Dpp10	307612	Hypo
chr7	125961493	125962650	Intron (NM_0(	-0523	225E-03	272E-02	Tmem117	203902	Hypo
chr2	119236819	119239833	Distal Interger	-0329	226E-03	272E-02	Anxa5	104738	Hypo
chr2	210510042	210510530	Distal Interger	-0832	226E-03	272E-02	Bcar3	-14730	Hypo
chr1	132583796	132584768	Distal Interger	-0520	226E-03	272E-02	Ntrk3	-80510	Hypo
chr4	9729592	9731615	Distal Interger	-0420	226E-03	272E-02	Cct8l2	-122621	Hypo
chr20	8385121	8386861	Intron (NM_0(	-0473	226E-03	272E-02	Btbd9	252983	Hypo
chr16	29433322	29439077	Promoter (2-3	-0284	226E-03	272E-02	Mfap3l	-2459	Hypo
chr4	170322863	170323749	Exon (NM_01	-0566	226E-03	272E-02	Ptpro	158432	Hypo
chr17	23164398	23169989	Promoter (<=1	-0277	226E-03	272E-02	Tmem170b	0	Hypo
chr10	25191131	25192450	Distal Interger	-0456	226E-03	272E-02	Ccng1	-8527	Hypo
chr16	38405821	38407601	Exon (NM_00	-0474	226E-03	272E-02	Neil3	46516	Hypo
chr8	53855604	53857515	Exon (NM_00	-0397	227E-03	272E-02	C8h11orf65	59238	Hypo
chr6	101654943	101655549	Exon (NM_00	-0586	227E-03	273E-02	Pcnx1	89363	Hypo
chr6	69905832	69908092	Distal Interger	-0405	227E-03	273E-02	Arhgap5	-80118	Hypo
chr10	10494334	10501504	3' UTR	-0284	227E-03	273E-02	Ubn1	30506	Hypo
chr12	13614442	13615396	Distal Interger	-0635	227E-03	273E-02	Gna12	-190302	Hypo
chr3	103824607	103830881	Distal Interger	-0253	227E-03	273E-02	Spred1	-153148	Hypo
chr5	153250312	153251751	3' UTR	-0512	227E-03	273E-02	Sdhb	-13155	Hypo
chr6	3415257	3417578	Intron (NM_0	-0367	228E-03	273E-02	Nrxn1	233967	Hypo
chr8	65275416	65277824	Distal Interger	-0350	228E-03	273E-02	Rab11a	-28955	Hypo
chr2	193002493	193006799	Intron (NM_0(	-0299	228E-03	273E-02	Kcnd3	64543	Hypo
chr6	14471127	14472452	Distal Interger	-0515	229E-03	274E-02	Sos1	-61418	Hypo
chr7	33328170	33329804	Distal Interger	-0530	229E-03	274E-02	Atp2b1	-406171	Hypo
chr1	161477695	161480495	Promoter (<=1	-0344	229E-03	274E-02	Olfml1	0	Hypo
chr4	77992484	77995004	Distal Interger	-0377	229E-03	274E-02	Gpnmb	-15243	Hypo
chr8	41815899	41819146	Distal Interger	-0274	229E-03	274E-02	Lnc215	-26581	Hypo
chr20	16512690	16513568	Distal Interger	-0460	229E-03	274E-02	RGD1559903	-111786	Hypo
chr2	45077409	45077696	Distal Interger	-0799	230E-03	275E-02	Cspg4b	137329	Hypo
chr11	43322777	43324082	Intron (NM_0(	-0476	230E-03	275E-02	Tbc1d23	19422	Hypo
chr2	243777051	243777517	Intron (NM_1	-0718	230E-03	275E-02	Cryz	224940	Hypo
chr15	29059708	29060686	Distal Interger	-0603	230E-03	275E-02	Fitm1	-4060	Hypo
chr7	112209814	112212306	Promoter (<=1	-0407	230E-03	275E-02	Fam83f	0	Hypo

chr14	96401662	96404045	Distal Interger	-0392	231E-03	276E-02	Ehbp1	-56330	Hypo
chr10	90701496	90701996	Intron (NM_0(	-0678	231E-03	276E-02	Tanc2	148264	Hypo
chr16	72155482	72157077	Distal Interger	-0470	231E-03	276E-02	Csmd1	-61688	Hypo
chr7	42156202	42156888	Exon (NM_00:	-0579	231E-03	276E-02	Acss3	293342	Hypo
chr8	42113014	42115589	Distal Interger	-0287	231E-03	276E-02	Vof16	159952	Hypo
chr16	47231973	47233458	Intron (NM_0:	-0466	231E-03	276E-02	Fat1	62649	Hypo
chr12	27315319	27316697	Distal Interger	-0486	232E-03	277E-02	Sfswap	-135435	Hypo
chr16	9771806	9773608	Intron (NM_0:	-0392	232E-03	277E-02	Bmpr1a	7008	Hypo
chr16	5245738	5249097	Intron (NM_0	-0364	232E-03	277E-02	Chdh	51469	Hypo
chr20	43070495	43074261	Distal Interger	-0315	232E-03	277E-02	Rev3l	-58102	Hypo
chr16	44456800	44457883	Distal Interger	-0608	232E-03	277E-02	Cldn24	-31328	Hypo
chr10	88540122	88541044	Distal Interger	-0508	232E-03	277E-02	Lyzl6	8133	Hypo
chr16	56026125	56027065	Distal Interger	-0602	232E-03	277E-02	Prag1	114088	Hypo
chr14	74916595	74925083	Exon (NM_00:	-0300	233E-03	277E-02	Ablim2	65251	Hypo
chr6	11391617	11394277	Distal Interger	-0393	233E-03	277E-02	Pkdcc	70725	Hypo
chr2	222192098	222192415	Distal Interger	-0878	233E-03	277E-02	Ppa2	391248	Hypo
chr6	19889097	19890346	Distal Interger	-0493	233E-03	277E-02	Fam98a	15015	Hypo
chr15	37112021	37116963	Intron (NM_0(	-0332	233E-03	278E-02	Wdfy2	28179	Hypo
chr1	184056038	184056786	Distal Interger	-0549	233E-03	278E-02	Plpp4	225629	Hypo
chr1	213084241	213085479	Distal Interger	-0496	234E-03	279E-02	Psat1	133085	Hypo
chr4	119905154	119907033	Exon (NM_00:	-0413	234E-03	279E-02	Bmp10	33088	Hypo
chr15	16299194	16300940	Intron (NM_0(	-0354	234E-03	279E-02	Cfap20dc	67895	Hypo
chr16	67898959	67899286	Distal Interger	-0839	234E-03	279E-02	Tcim	303197	Hypo
chr5	20995833	20999523	Distal Interger	-0353	235E-03	279E-02	Car8	402851	Hypo
chr13	90065467	90069705	Promoter (<=:	-0264	235E-03	279E-02	Cox20	0	Hypo
chr2	209830276	209831377	Intron (NM_0	-0616	235E-03	279E-02	F3	3215	Hypo
chr2	233729850	233730194	Distal Interger	-0821	235E-03	279E-02	Sh3glb1	54590	Hypo
chr10	7572363	7572743	Distal Interger	-0815	235E-03	279E-02	Tmem114	402617	Hypo
chr19	55912692	55914061	Distal Interger	-0435	235E-03	280E-02	Nrp1	-445394	Hypo
chr7	26770800	26771035	Distal Interger	-0956	236E-03	280E-02	Mir135a	-158711	Hypo
chr14	55580771	55582414	Distal Interger	-0518	236E-03	280E-02	Stim2	1421765	Hypo
chr7	133931407	133932523	Distal Interger	-0549	236E-03	280E-02	Cistr	-59394	Hypo
chr6	67469686	67475631	Distal Interger	-0297	236E-03	280E-02	Prkd1	563066	Hypo

chr2	23449100	23450859	Intron (NM_0(	-0458	236E-03	280E-02	Rasgrf2	-88145	Hypo
chr5	56657290	56658311	Distal Interger	-0517	236E-03	280E-02	Myorg	6080	Hypo
chr1	148858008	148858670	Distal Interger	-0681	236E-03	280E-02	Fam181b	1691580	Hypo
chr2	233946488	233949427	Exon (NM_001	-0393	237E-03	281E-02	Clca1	14942	Hypo
chr2	129980537	129981450	Distal Interger	-0548	237E-03	281E-02	Pabpc4l	-170792	Hypo
chr16	54026345	54027378	Intron (NM_0(	-0536	237E-03	281E-02	Mir383	-12962	Hypo
chr12	21508545	21510773	Promoter (<=1	-0293	238E-03	282E-02	Bcl7b	0	Hypo
chr1	90387717	90392879	Distal Interger	-0343	238E-03	282E-02	Uri1	311777	Hypo
chr6	48165291	48166209	Distal Interger	-0580	238E-03	282E-02	Slc26a4	-19588	Hypo
chr14	26386977	26389508	Intron (NM_1:	-0384	238E-03	282E-02	Adgrl3	434936	Hypo
chr2	109519991	109521747	Distal Interger	-0460	238E-03	282E-02	Ect2	516164	Hypo
chr5	61626271	61627081	Distal Interger	-0580	239E-03	282E-02	Tgfbr1	-26718	Hypo
chr13	20083376	20086849	Distal Interger	-0335	239E-03	283E-02	Cdh20	-913810	Hypo
chr14	43427610	43429333	Distal Interger	-0433	239E-03	283E-02	Tlr10	16224	Hypo
chr7	119515637	119515991	Distal Interger	-0901	239E-03	283E-02	Zdhhc25	-176688	Hypo
chr2	168026447	168027007	Distal Interger	-0709	240E-03	283E-02	Npy2r	-121423	Hypo
chr3	56573984	56576461	Distal Interger	-0369	240E-03	283E-02	Itga6	-41083	Hypo
chr6	16385487	16387988	Exon (NM_01!	-0362	240E-03	283E-02	Strn	50117	Hypo
chr6	35144677	35150139	Exon (NM_001	-0319	240E-03	283E-02	Cyria	99469	Hypo
chr4	47107084	47107998	Distal Interger	-0592	240E-03	283E-02	Cttnbp2	-142453	Hypo
chr15	37661630	37663713	Promoter (1-2	-0475	240E-03	283E-02	Blk	1318	Hypo
chr6	50597965	50598465	Distal Interger	-0659	240E-03	283E-02	Ferd3l	-46914	Hypo
chr8	95746010	95746527	Intron (NM_0(	-0691	240E-03	284E-02	Paqr9	-367722	Hypo
chr8	105664627	105666882	Distal Interger	-0350	241E-03	284E-02	Mrpl3	-3302	Hypo
chr19	47687512	47691058	Promoter (<=1	-0357	241E-03	284E-02	Kcng4	0	Hypo
chr2	42485340	42486920	Distal Interger	-0420	241E-03	284E-02	Actbl2	-371100	Hypo
chr15	7582522	7583285	Distal Interger	-0615	241E-03	284E-02	Nr1d2	58265	Hypo
chr10	85329212	85329780	Promoter (2-3	-0729	241E-03	284E-02	Jup	-2155	Hypo
chr4	126383375	126384911	Intron (NM_0(	-0416	241E-03	284E-02	Magi1	425848	Hypo
chr10	63656745	63659256	Distal Interger	-0397	241E-03	284E-02	Ccnq	-9050	Hypo
chr3	56714818	56717316	Exon (NM_05:	-0396	241E-03	284E-02	Pdk1	8947	Hypo
chr5	127907377	127907977	Distal Interger	-0708	241E-03	284E-02	Foxe3	538477	Hypo
chr7	25461235	25464201	Intron (NM_0(	-0362	242E-03	284E-02	Apaf1	115339	Hypo

chr3	152546181	152547861	Intron (NM_1	-0410	242E-03	285E-02	Kcnk15	30944	Hypo
chr7	124997838	125000281	Distal Interger	-0367	242E-03	285E-02	Prickle1	-339725	Hypo
chr11	33792622	33793511	Distal Interger	-0547	242E-03	285E-02	Vps26c	47902	Hypo
chr17	71408287	71425817	Promoter (<=1	-0237	242E-03	285E-02	Celf2	0	Hypo
chr5	19961821	19962899	Intron (NM_0	-0560	243E-03	285E-02	Tox	208373	Hypo
chr2	28955901	28958056	Distal Interger	-0409	243E-03	285E-02	Hexb	-451736	Hypo
chr20	20708383	20710100	Distal Interger	-0466	243E-03	285E-02	Zfp365	42143	Hypo
chr9	62214965	62215509	Distal Interger	-0769	243E-03	286E-02	Cd28	48641	Hypo
chr11	32202922	32205189	Distal Interger	-0420	243E-03	286E-02	Runx1	-267491	Hypo
chr6	63142875	63144730	Distal Interger	-0379	244E-03	286E-02	Nova1	760813	Hypo
chr9	25046679	25050312	Intron (NM_0	-0352	244E-03	286E-02	Rims1	146056	Hypo
chr14	74407727	74409940	Intron (NM_0	-0448	244E-03	286E-02	Sorcs2	36312	Hypo
chr3	153214193	153221751	Promoter (<=1	-0257	244E-03	286E-02	Dbndd2	0	Hypo
chr1	145447836	145449007	Intron (NM_0	-0550	244E-03	286E-02	Dlg2	320661	Hypo
chrX	111372616	111374368	Distal Interger	-0509	244E-03	286E-02	Pls3	-214933	Hypo
chr14	76478483	76485654	Intron (NM_0	-0282	244E-03	286E-02	Zfyve28	10059	Hypo
chr10	68191938	68195223	Intron (NM_0	-0331	244E-03	287E-02	Rasl10b	-15230	Hypo
chr6	118581323	118584346	Intron (NM_0	-0354	244E-03	287E-02	Foxn3	258055	Hypo
chr8	51295688	51299240	Distal Interger	-0381	245E-03	287E-02	Layn	85014	Hypo
chr7	129319959	129320376	Distal Interger	-0782	245E-03	287E-02	Ccdc184	20286	Hypo
chr14	94142745	94145809	Distal Interger	-0334	245E-03	287E-02	Spred2	-3885	Hypo
chr8	62186523	62194128	Distal Interger	-0277	245E-03	287E-02	Rplp1	201213	Hypo
chr9	86872191	86874525	Intron (NM_0	-0401	245E-03	287E-02	Armc9	69308	Hypo
chr7	109424661	109425457	Promoter (<=1	-0413	246E-03	287E-02		-204	Hypo
chr9	106424892	106433785	Distal Interger	-0270	246E-03	287E-02	Rab12	73014	Hypo
chr13	66259953	66264897	Distal Interger	-0316	246E-03	288E-02	Glul	234312	Hypo
chr1	145438964	145439688	Intron (NM_0	-0596	246E-03	288E-02	Dlg2	311789	Hypo
chr15	95895906	95897540	Intron (NM_0	-0488	247E-03	288E-02	Cldn10	33121	Hypo
chr7	9560792	9570092	Promoter (<=1	-0270	247E-03	288E-02	Atp5f1d	0	Hypo
chr8	122313310	122315393	Distal Interger	-0383	247E-03	288E-02	Mir138-1	12344	Hypo
chr2	66230163	66230916	Distal Interger	-0629	247E-03	288E-02	Cdh9	-49506	Hypo
chr3	136331946	136341956	Promoter (<=1	-0294	247E-03	288E-02	Cst3	0	Hypo
chr17	24122462	24123387	Distal Interger	-0539	247E-03	289E-02	Tfap2a	90495	Hypo

chr4	28809809	28810599	Intron (NM_0	-0562	247E-03	289E-02	Cdk14	143121	Hypo
chr2	8440572	8441924	Distal Interger	-0513	247E-03	289E-02	Nr2f1	-390235	Hypo
chr1	90328378	90329520	Distal Interger	-0511	248E-03	289E-02	Uri1	375136	Hypo
chr5	77270397	77271385	Intron (NM_0	-0620	248E-03	289E-02	Tnfsf8	5693	Hypo
chr6	35069497	35073461	Intron (NM_0	-0320	248E-03	289E-02	Cyria	24289	Hypo
chr6	14000138	14000971	Distal Interger	-0652	248E-03	289E-02	Tmem178a	62548	Hypo
chr16	915457	916601	Distal Interger	-0569	248E-03	289E-02	Zmiz1	-110724	Hypo
chr3	117834169	117836452	Intron (NM_0	-0411	248E-03	290E-02	Ubox5	11259	Hypo
chr13	73534482	73539929	Promoter (<=1	-0290	249E-03	290E-02	Prdx6	0	Hypo
chr6	67181053	67182338	Distal Interger	-0499	249E-03	290E-02	Foxg1	506256	Hypo
chr1	184056952	184057979	Distal Interger	-0438	249E-03	290E-02	Plpp4	226543	Hypo
chr7	110150959	110152107	Promoter (<=1	-0535	249E-03	290E-02	Cyth4	-169	Hypo
chr2	170514613	170516055	Distal Interger	-0475	250E-03	291E-02	Dear	-214431	Hypo
chr5	151266748	151268266	Exon (NM_00	-0484	250E-03	291E-02	Htr6	43646	Hypo
chr15	56706386	56707371	Distal Interger	-0524	250E-03	291E-02	Mir1297	-141059	Hypo
chr1	146480751	146482770	Intron (NM_0	-0431	250E-03	291E-02	Ccdc90b	-149986	Hypo
chr4	53585405	53585929	Distal Interger	-0685	250E-03	291E-02	Tmem229a	-140078	Hypo
chr6	3280698	3284126	Intron (NM_0	-0320	250E-03	291E-02	Nrxn1	99408	Hypo
chr1	259630936	259631864	Distal Interger	-0576	250E-03	291E-02	Prlhr	-22545	Hypo
chr10	64473961	64482271	Promoter (<=1	-0286	251E-03	291E-02	Evi2b	0	Hypo
chr9	49548345	49548616	Intron (NM_0	-0932	251E-03	291E-02	Stat4	39924	Hypo
chr9	97436762	97437447	Distal Interger	-0562	251E-03	291E-02	Slco6b1	-89426	Hypo
chr1	193453104	193455849	Distal Interger	-0367	251E-03	292E-02	Mapk1ip1	200894	Hypo
chr5	16717382	16717915	Intron (NM_0	-0695	251E-03	292E-02	Lyn	77870	Hypo
chr4	169420141	169425175	Intron (NM_0	-0283	251E-03	292E-02	Atf7ip	34269	Hypo
chr1	12955793	12958527	Distal Interger	-0412	252E-03	292E-02	Ccdc28a	-83087	Hypo
chr10	62790328	62791658	Distal Interger	-0469	252E-03	292E-02	Pipox	-7958	Hypo
chr3	148862288	148863349	Distal Interger	-0548	252E-03	293E-02	Mafb	136682	Hypo
chr1	20112069	20114890	Intron (NM_0	-0400	252E-03	293E-02	Ccnb2	-10833	Hypo
chr14	31533741	31535520	Distal Interger	-0421	252E-03	293E-02	Exoc1	124356	Hypo
chr20	40738147	40738610	Distal Interger	-0781	252E-03	293E-02	Marcks	-47135	Hypo
chr18	25712410	25713952	Distal Interger	-0494	253E-03	293E-02	Apc	-150270	Hypo
chr15	40683937	40686354	Distal Interger	-0365	253E-03	293E-02	Stmn4	142511	Hypo

chr5	133508757	133510322	Distal Interger	-0480	253E-03	293E-02	RGD1563049	40625	Hypo
chr2	224150112	224150816	Distal Interger	-0651	253E-03	293E-02	Nfkb1	-18281	Hypo
chr2	16456119	16456862	Distal Interger	-0621	253E-03	293E-02	LOC252890	385168	Hypo
chr5	118663564	118664593	Distal Interger	-0573	253E-03	293E-02	Oma1	579836	Hypo
chr5	14793245	14793783	Distal Interger	-0635	253E-03	293E-02	Mrpl15	63250	Hypo
chr6	104566888	104569145	3' UTR	-0406	254E-03	293E-02	Arel1	24331	Hypo
chr4	123843596	123846562	Distal Interger	-0343	254E-03	293E-02	Wnt7a	62419	Hypo
chr1	167268743	167269972	Distal Interger	-0492	254E-03	293E-02	Bmal1	-61848	Hypo
chr4	59872345	59874854	Distal Interger	-0417	254E-03	293E-02	Mkln1	-127644	Hypo
chr8	110888964	110889802	Distal Interger	-0563	254E-03	293E-02	Tmie	-24161	Hypo
chr7	23609738	23610531	Intron (NM_0(	-0557	254E-03	294E-02	Ano4	41201	Hypo
chr9	60329786	60331533	Exon (NM_001	-0432	255E-03	294E-02	Flacc1	12115	Hypo
chr17	70930999	70934120	Distal Interger	-0379	255E-03	294E-02	Celf2	-475490	Hypo
chr2	191465777	191467175	3' UTR	-0480	255E-03	294E-02	Phtf1	-6073	Hypo
chr5	75520448	75521056	Distal Interger	-0549	255E-03	294E-02	Mup4l1	-21581	Hypo
chr4	87554107	87555221	Distal Interger	-0476	255E-03	294E-02	Rps7-ps20	46815	Hypo
chr12	13785958	13787382	Distal Interger	-0467	255E-03	294E-02	Gna12	-18316	Hypo
chr10	103084293	103085387	Distal Interger	-0563	255E-03	295E-02	Birc5	10816	Hypo
chr1	212706000	212706816	Distal Interger	-0578	256E-03	295E-02	Psat1	511748	Hypo
chr2	86418182	86423620	Distal Interger	-0338	256E-03	295E-02		-176908	Hypo
chr9	64162045	64162928	Intron (NM_0:	-0640	256E-03	296E-02	Nrp2	38668	Hypo
chr4	21508164	21509718	Distal Interger	-0443	257E-03	296E-02	Sema3a	-14890	Hypo
chr18	3047332	3048808	Distal Interger	-0466	257E-03	296E-02	Cables1	-28471	Hypo
chr2	99610311	99613012	Distal Interger	-0368	257E-03	296E-02	Mir124-2	-521854	Hypo
chr1	203431025	203437808	3' UTR	-0282	258E-03	297E-02	Snx15	-4852	Hypo
chr6	9528876	9530393	Intron (NM_0(	-0417	258E-03	297E-02	Camkmt	49705	Hypo
chr1	150288696	150289121	Distal Interger	-0838	258E-03	297E-02	Mir708	-310755	Hypo
chr6	93176796	93179100	Distal Interger	-0429	258E-03	297E-02	Snapc1	492011	Hypo
chr5	158926996	158932098	Exon (NM_01!	-0318	259E-03	297E-02	Mtor	42135	Hypo
chr6	14045962	14047381	Intron (NM_0(	-0524	259E-03	297E-02	Tmem178a	16138	Hypo
chr13	97790800	97793050	Distal Interger	-0407	259E-03	297E-02	Lyplal1	-132933	Hypo
chr6	5033188	5034120	Distal Interger	-0554	259E-03	298E-02	Fshr	-164705	Hypo
chr5	164514838	164516442	Promoter (<=:	-0360	259E-03	298E-02	C5h1orf174	0	Hypo

chr1	178754035	178754277	Distal Interger	-0926	259E-03	298E-02	Aqp8	760676	Hypo
chr1	254983822	254988437	Distal Interger	-0288	259E-03	298E-02	Tcf7l2	197731	Hypo
chrX	14928176	14930444	Distal Interger	-0345	260E-03	298E-02	Foxp3	-4362	Hypo
chr12	33936953	33939669	Promoter (2-3	-0342	260E-03	298E-02	P2rx7	-2852	Hypo
chr15	99542667	99543364	Distal Interger	-0688	260E-03	298E-02	Zic5	23671	Hypo
chr20	6062661	6067303	Exon (NM_00	-0334	260E-03	298E-02	Tcp11	68608	Hypo
chr10	82584355	82588276	Distal Interger	-0279	260E-03	298E-02	Srcin1	-15656	Hypo
chr8	95480230	95480649	Intron (NM_0	-0782	260E-03	298E-02	Slc9a9	247325	Hypo
chr1	202901828	202903716	Promoter (<=1	-0304	260E-03	299E-02	Kat5	0	Hypo
chr15	35881446	35883554	Distal Interger	-0480	261E-03	299E-02	Kcnrg	106954	Hypo
chr10	37678752	37680320	Distal Interger	-0525	261E-03	299E-02	Septin8	-4319	Hypo
chr2	190150492	190151565	Distal Interger	-0423	261E-03	299E-02	Tspan2	-26224	Hypo
chr14	12625095	12632009	Distal Interger	-0289	261E-03	299E-02	Anxa3	149625	Hypo
chr1	97468872	97469524	Distal Interger	-0706	261E-03	299E-02	Tsg101	-26359	Hypo
chr1	153965199	153968143	Exon (NM_08	-0351	262E-03	300E-02	Slco2b1	27549	Hypo
chr7	10072053	10074818	Exon (NM_00	-0314	262E-03	300E-02	Shc2	5348	Hypo
chr9	103386144	103386776	Distal Interger	-0631	262E-03	300E-02	Fbxl17	-180448	Hypo
chr9	15226837	15227919	Distal Interger	-0434	262E-03	300E-02	Mrpl14	80500	Hypo
chr16	10088580	10091480	Distal Interger	-0356	262E-03	300E-02	Grid1	-47355	Hypo
chr6	10846110	10847051	Promoter (<=1	-0552	263E-03	300E-02	Haao	325	Hypo
chr12	5477004	5478171	Distal Interger	-0540	263E-03	301E-02	Hsph1	86014	Hypo
chr2	165511814	165513528	Distal Interger	-0449	263E-03	301E-02	Mir2985	442970	Hypo
chr14	6547166	6549307	Intron (NM_0	-0408	263E-03	301E-02	Mapk10	49459	Hypo
chr8	119799533	119800991	Promoter (<=1	-0435	263E-03	301E-02	Cx3cr1	-102	Hypo
chr10	83294500	83296655	Distal Interger	-0327	264E-03	301E-02		27168	Hypo
chr1	194599465	194603641	Distal Interger	-0314	264E-03	301E-02	Utf1	-136698	Hypo
chr4	147380257	147382408	Intron (NM_0	-0421	264E-03	302E-02	Slc6a11	82285	Hypo
chr5	46701566	46704755	Distal Interger	-0288	264E-03	302E-02	Bach2	-12266	Hypo
chr4	156081479	156082557	Promoter (2-3	-0551	265E-03	302E-02	C3ar1	2043	Hypo
chr10	20550907	20554080	Intron (NM_0	-0348	265E-03	302E-02	Rars1	-255715	Hypo
chr7	90976320	90977133	Exon (NM_00	-0642	265E-03	302E-02	Nsmce2	40122	Hypo
chr1	145230908	145232865	Intron (NM_0	-0450	265E-03	302E-02	Dlg2	103733	Hypo
chr2	146739793	146741879	Distal Interger	-0384	266E-03	303E-02	Dhx36	152693	Hypo

chr11	56034617	56035066	Distal Interger	-0777	266E-03	303E-02	Nepro	-64185	Hypo
chr5	146832628	146848650	Intron (NM_0(	-0237	266E-03	303E-02	Man1c1	64607	Hypo
chr4	170456228	170461267	Intron (NM_0(	-0342	267E-03	304E-02	Eps8	25606	Hypo
chr2	42241285	42241972	Distal Interger	-0625	267E-03	304E-02	Plk2	272101	Hypo
chr4	58962373	58964133	Distal Interger	-0409	267E-03	304E-02	Ube2h	-32217	Hypo
chr8	54725399	54726003	Distal Interger	-0665	268E-03	304E-02	Gldn	46280	Hypo
chr1	11947369	11948681	Distal Interger	-0481	268E-03	304E-02	Cited2	-363745	Hypo
chr17	28860754	28861675	Distal Interger	-0480	268E-03	305E-02	Rpp40	-86485	Hypo
chr9	40675754	40677439	Intron (NM_0(	-0457	268E-03	305E-02	Aff3	92996	Hypo
chr10	64883416	64883673	Distal Interger	-0889	270E-03	306E-02	Crlf3	176866	Hypo
chr8	48385442	48386545	Distal Interger	-0594	270E-03	306E-02	Nxpe4	-335968	Hypo
chr11	69270868	69276049	Distal Interger	-0311	270E-03	306E-02	Bdh1	61622	Hypo
chr5	24424579	24425294	Distal Interger	-0585	270E-03	307E-02	Dpy19l4	-13667	Hypo
chr3	156357236	156360668	Intron (NM_0(	-0321	270E-03	307E-02	Peds1	8641	Hypo
chr19	43703576	43713170	Promoter (<=1	-0284	270E-03	307E-02	Maf	0	Hypo
chr6	57658179	57659331	Distal Interger	-0538	271E-03	307E-02	Ifrd1	-369273	Hypo
chrX	9929645	9930132	Distal Interger	-0764	271E-03	307E-02		50442	Hypo
chr20	24620121	24620668	Intron (NM_0(	-0703	271E-03	307E-02	Lrrtm3	104494	Hypo
chr8	108274956	108276575	Promoter (2-3	-0425	271E-03	307E-02	Sema3b	2439	Hypo
chr19	45210685	45217293	Promoter (2-3	-0286	272E-03	308E-02	Gan	2821	Hypo
chr2	57074652	57076716	Intron (NM_0(	-0373	272E-03	308E-02	Wdr70	114442	Hypo
chr3	116889443	116890580	Distal Interger	-0520	272E-03	308E-02	Pdyn	22754	Hypo
chr5	97598960	97600889	Distal Interger	-0446	272E-03	308E-02	Ttc39b	107181	Hypo
chr8	33094530	33095574	Intron (NM_0(	-0544	272E-03	308E-02	Kirrel3	228665	Hypo
chr7	27674626	27675861	Distal Interger	-0468	272E-03	308E-02	Cdk17	-8029	Hypo
chr17	64960697	64961954	Distal Interger	-0448	273E-03	308E-02	Klf6	-412246	Hypo
chr2	209697868	209699949	Distal Interger	-0418	273E-03	308E-02	Slc44a3	-69945	Hypo
chr2	183084697	183088383	Promoter (<=1	-0361	273E-03	308E-02	Ctss	0	Hypo
chr15	38830261	38847586	Promoter (<=1	-0251	273E-03	308E-02	Mir124-1	0	Hypo
chr2	38411009	38412343	Intron (NM_0!	-0416	273E-03	308E-02	Kif2a	18894	Hypo
chr4	119616969	119618416	Intron (NM_0(	-0472	273E-03	309E-02	Gfpt1	120212	Hypo
chr12	22018152	22020100	Distal Interger	-0425	273E-03	309E-02	Limk1	-6597	Hypo
chr16	1465029	1466242	Intron (NM_0(	-0552	273E-03	309E-02	Plac9	7980	Hypo

chr3	80677906	80678444	Distal Intergenic	-0633	273E-03	309E-02	Api5	-274732	Hypo
chr7	15927900	15930169	Intron (NM_0	-0291	274E-03	309E-02	LOC300308	106371	Hypo
chr8	97271212	97274561	Distal Intergenic	-0310	275E-03	310E-02	Zbtb38	13957	Hypo
chr7	84281290	84283546	Distal Intergenic	-0380	275E-03	311E-02	Med30	276561	Hypo
chr5	153242525	153247439	Exon (NM_01	-0325	275E-03	311E-02	Sdhb	-17467	Hypo
chr4	38574880	38575366	Distal Intergenic	-0805	275E-03	311E-02	LOC500028	-656369	Hypo
chr1	4715032	4715643	Distal Intergenic	-0651	276E-03	311E-02	Rab32	245360	Hypo
chr2	215718065	215718812	Distal Intergenic	-0613	276E-03	311E-02	Larp7	293997	Hypo
chr3	81835037	81837575	Distal Intergenic	-0356	276E-03	311E-02	Lrrc4c	-467669	Hypo
chr10	99923990	99926334	Distal Intergenic	-0442	276E-03	311E-02	Cd300a	-3467	Hypo
chr2	57758965	57760399	Exon (NM_01	-0485	276E-03	311E-02	Slc1a3	70185	Hypo
chr7	114080477	114083521	Distal Intergenic	-0363	276E-03	311E-02	Tcf20	-28638	Hypo
chr10	73506518	73512434	Distal Intergenic	-0266	276E-03	311E-02	Akap1	123812	Hypo
chr18	59234684	59237889	Exon (NM_00	-0368	277E-03	312E-02	Oacyl	22330	Hypo
chr5	141663673	141664924	Promoter (<=1	-0499	277E-03	312E-02		587	Hypo
chr7	29048519	29049033	Distal Intergenic	-0697	277E-03	312E-02	Tmcc3	-51142	Hypo
chr18	67726054	67726664	Distal Intergenic	-0664	278E-03	313E-02	Ska1	78373	Hypo
chr13	68290435	68291400	Intron (NM_0	-0533	278E-03	313E-02	Fam163a	49649	Hypo
chr9	13772619	13775771	Distal Intergenic	-0318	279E-03	314E-02	Trerf1	-94281	Hypo
chr8	60662954	60663371	Distal Intergenic	-0749	279E-03	314E-02	Lrrc49	497324	Hypo
chr16	66824372	66828439	Distal Intergenic	-0293	279E-03	314E-02	Tacc1	-16602	Hypo
chr6	8205038	8207977	Intron (NM_0	-0387	279E-03	314E-02	RGD1562146	28275	Hypo
chr8	65115878	65116922	Distal Intergenic	-0639	280E-03	314E-02	Rab11a	129539	Hypo
chr3	35971996	35973754	Distal Intergenic	-0419	280E-03	314E-02	Tas2r134	-103898	Hypo
chr10	88604909	88606446	Promoter (<=1	-0304	280E-03	315E-02	Gosr2	0	Hypo
chr14	56376570	56377863	Distal Intergenic	-0437	280E-03	315E-02	Stim2	626316	Hypo
chr14	37094346	37098864	Promoter (<=1	-0255	281E-03	315E-02	Gabra2	0	Hypo
chr3	78269409	78270168	Distal Intergenic	-0562	281E-03	316E-02	Large2	72016	Hypo
chr7	96154326	96155797	Distal Intergenic	-0411	282E-03	316E-02	Asap1	-95957	Hypo
chrX	27249719	27250893	Distal Intergenic	-0542	282E-03	316E-02	Tmsb4x	105042	Hypo
chr16	21138741	21141276	Distal Intergenic	-0330	283E-03	317E-02	Csgalnact1	-94732	Hypo
chr8	33453762	33462514	Promoter (1-2	-0282	283E-03	317E-02	St3gal4	1976	Hypo
chr16	73267578	73268012	Intron (NM_0	-0741	284E-03	317E-02	Csmd1	1048813	Hypo

chr18	59042399	59046893	Distal Interger	-0303	284E-03	318E-02	Zfp532	-49800	Hypo
chr6	35986525	35991987	Distal Interger	-0305	284E-03	318E-02	Ddx1	35353	Hypo
chr9	3292090	3294929	Promoter (<=1	-0293	284E-03	318E-02	Plcl2	0	Hypo
chr3	89001984	89025417	Promoter (<=1	-0232	284E-03	318E-02	Slc1a2	0	Hypo
chr5	120239841	120240456	Distal Interger	-0754	285E-03	318E-02	Plpp3	312756	Hypo
chr3	136344128	136345982	Distal Interger	-0403	285E-03	318E-02	Cst3	-3332	Hypo
chr10	2067915	2069000	Exon (NM_001	-0528	285E-03	318E-02	Pdxdc1	42555	Hypo
chr2	30472018	30476247	Distal Interger	-0292	285E-03	318E-02	Tnpo1	-202633	Hypo
chr11	36372034	36372603	Intron (NM_1	-0781	285E-03	319E-02	Dscam	134497	Hypo
chr3	133294891	133297358	Intron (NM_0	-0381	286E-03	319E-02	Naa20	-24706	Hypo
chr18	3093878	3095549	Intron (NM_0	-0438	286E-03	319E-02	Cables1	16599	Hypo
chr5	112770432	112773498	Intron (NM_0	-0329	286E-03	319E-02	Patj	-288620	Hypo
chr20	21331203	21332016	Distal Interger	-0596	286E-03	319E-02	Nrbf2	22597	Hypo
chr15	24225681	24227265	Promoter (<=1	-0520	286E-03	319E-02	Rnase10	-658	Hypo
chr18	51264892	51265450	Distal Interger	-0863	286E-03	319E-02	Slc12a2	-82852	Hypo
chr17	64959577	64960538	Distal Interger	-0461	287E-03	320E-02	Klf6	-411126	Hypo
chr1	41828334	41834433	Distal Interger	-0303	287E-03	320E-02	Myct1	-183704	Hypo
chr3	79892513	79893634	Distal Interger	-0565	287E-03	320E-02	RGD1564664	27889	Hypo
chr1	88426335	88426700	Distal Interger	-0784	287E-03	320E-02	Dpy19l3	-8996	Hypo
chr5	114656121	114657841	Distal Interger	-0411	288E-03	320E-02	Pgm1	60823	Hypo
chr11	55095051	55095631	Distal Interger	-0670	288E-03	321E-02	Tagln3	-4256	Hypo
chr4	121111878	121113134	Distal Interger	-0485	288E-03	321E-02	Mgll	-79086	Hypo
chr1	155910461	155918690	3' UTR	-0260	288E-03	321E-02	Mir139	31633	Hypo
chr1	26224291	26225230	Intron (NM_0	-0603	288E-03	321E-02	Tpd52l1	52121	Hypo
chr7	119663319	119663659	Distal Interger	-0819	289E-03	321E-02	Zdhhc25	-29020	Hypo
chr4	177144311	177160562	Promoter (<=1	-0252	289E-03	321E-02	Sox5	0	Hypo
chr2	209823837	209829523	Promoter (<=1	-0275	289E-03	321E-02	F3	0	Hypo
chr2	139460316	139463532	Distal Interger	-0298	289E-03	321E-02	Sohlh2	98070	Hypo
chr14	33809931	33810375	Intron (NM_0	-0707	290E-03	322E-02	Scfd2	126583	Hypo
chr5	128582782	128584387	Distal Interger	-0512	290E-03	322E-02	Tal1	-3314	Hypo
chr13	66119132	66122248	Distal Interger	-0334	290E-03	322E-02	Glul	93491	Hypo
chr11	20152900	20155686	Intron (NM_2	-0355	290E-03	322E-02	Ncam2	47854	Hypo
chr9	107459836	107460877	Distal Interger	-0529	291E-03	322E-02	Ptprm	-116138	Hypo

chr7	99870541	99879723	Distal Intergen	-0282	291E-03	322E-02	Zfat	174551	Hypo
chr17	55883798	55884465	Distal Intergen	-0686	291E-03	322E-02	Wac	-38658	Hypo
chr17	22709827	22715826	Promoter (<=1	-0322	291E-03	322E-02	Hivep1	0	Hypo
chr14	43281142	43281886	Distal Intergen	-0662	291E-03	323E-02	Tmem156	57723	Hypo
chr5	17188134	17189543	Promoter (<=1	-0513	291E-03	323E-02	Penk	0	Hypo
chr4	77768575	77777128	Promoter (<=1	-0310	292E-03	323E-02	Tmem176b	0	Hypo
chr16	21816571	21818307	Distal Intergen	-0415	292E-03	323E-02	Nat3	332996	Hypo
chr18	16030931	16034436	Intron (NM_0(	-0348	292E-03	323E-02	Fhod3	37607	Hypo
chr8	107914141	107915807	Distal Intergen	-0420	292E-03	323E-02	Dock3	-10627	Hypo
chr7	115689180	115690584	Distal Intergen	-0356	292E-03	324E-02	Prr5	-123370	Hypo
chr17	7991627	7992912	Distal Intergen	-0477	293E-03	324E-02	Tgfbi	-6789	Hypo
chr8	96074807	96077064	Distal Intergen	-0296	293E-03	324E-02	Paqr9	-37185	Hypo
chr5	53574869	53575554	Distal Intergen	-0728	293E-03	324E-02	RGD1306195	-880451	Hypo
chr1	50277277	50279282	Intron (NM_0(	-0399	294E-03	324E-02	Cahm	107677	Hypo
chr18	78735709	78737694	Distal Intergen	-0445	294E-03	324E-02	Timm21	-416347	Hypo
chr19	45469743	45474569	Exon (NM_001	-0277	294E-03	325E-02	Plcg2	-72847	Hypo
chr17	19883932	19884509	Distal Intergen	-0670	294E-03	325E-02	Dtnbp1	198714	Hypo
chr15	19020951	19024275	Distal Intergen	-0362	294E-03	325E-02	Ddhd1	-129999	Hypo
chr18	74419270	74427322	Distal Intergen	-0272	295E-03	325E-02	Sall3	-5442	Hypo
chr3	14727458	14727899	Distal Intergen	-0722	295E-03	325E-02	Ass1	-26169	Hypo
chr14	95364472	95365899	Distal Intergen	-0483	295E-03	325E-02	Vps54	-35833	Hypo
chr7	29110543	29113229	Intron (NM_0(	-0386	295E-03	325E-02	Tmcc3	10368	Hypo
chr11	78601861	78603389	Distal Intergen	-0481	295E-03	325E-02	Etv5	-5321	Hypo
chr8	48383825	48385130	Distal Intergen	-0507	295E-03	325E-02	Nxpe4	-337383	Hypo
chr19	21192244	21194091	Intron (NM_0(	-0394	295E-03	325E-02	Phkb	16514	Hypo
chr8	53127611	53129030	Distal Intergen	-0514	295E-03	326E-02	LOC10012536	-58599	Hypo
chr10	51722610	51723204	Promoter (2-3	-0685	296E-03	326E-02	Tmem220	2832	Hypo
chr8	5683146	5686197	Distal Intergen	-0356	296E-03	326E-02	Rpl13	12108	Hypo
chr2	136735419	136738267	Distal Intergen	-0338	297E-03	327E-02	Cog6	360909	Hypo
chr2	233836402	233837372	Exon (NM_001	-0477	297E-03	327E-02	Clca4l	8765	Hypo
chr8	116040964	116042648	Distal Intergen	-0391	297E-03	327E-02	Tgfbr2	-157726	Hypo
chr7	72377031	72379074	Distal Intergen	-0401	297E-03	327E-02	Oxr1	-149712	Hypo
chr8	123752331	123753932	Promoter (<=1	-0451	298E-03	327E-02	Ccr5	0	Hypo

chr17	74976430	74977652	Distal Interger	-0555	298E-03	328E-02	Nmt2	-15422	Hypo
chr9	26591080	26591541	Intron (NM_0(	-0824	298E-03	328E-02	Col9a1	6046	Hypo
chr17	53257062	53262053	Distal Interger	-0309	299E-03	328E-02	Mtpap	79428	Hypo
chr1	215118934	215120642	Intron (NM_0!	-0453	299E-03	329E-02	Pcsk5	146958	Hypo
chr18	68272826	68274224	Intron (NM_0	-0566	299E-03	329E-02	Acaa2	-70912	Hypo
chr1	78918821	78919255	Intron (NM_0(	-0751	299E-03	329E-02	Vasp	5685	Hypo
chr14	100702700	100704515	Distal Interger	-0402	300E-03	329E-02	Vrk2	-268173	Hypo
chr8	75974282	75976464	3' UTR	-0382	300E-03	329E-02	Myo5c	-13064	Hypo
chr6	105118636	105123078	Promoter (<=:	-0282	300E-03	329E-02	Fos	0	Hypo
chrX	151653606	151655553	Promoter (2-3	-0323	300E-03	329E-02	Arhgap4	-2490	Hypo
chr4	25377741	25379337	Exon (NM_13:	-0485	300E-03	329E-02	Abcb1b	-52655	Hypo
chr13	79414540	79418694	Intron (NM_0(	-0322	300E-03	329E-02	Uck2	19658	Hypo
chrX	35519724	35520732	3' UTR	-0581	300E-03	329E-02	Eif1ax	-6322	Hypo
chr4	178041639	178043543	Distal Interger	-0429	300E-03	330E-02	Bcat1	-28256	Hypo
chr9	7218359	7218865	Distal Interger	-0828	301E-03	330E-02	Sult1c3	48126	Hypo
chr5	83239733	83240009	Distal Interger	-0971	301E-03	330E-02	Cdk5rap2	720773	Hypo
chr15	19956074	19958817	Distal Interger	-0389	301E-03	330E-02	Cdkn3	-63792	Hypo
chr9	15474612	15476369	Promoter (1-2	-0417	301E-03	330E-02	Tcte1	-1658	Hypo
chr9	15241091	15242007	Distal Interger	-0629	301E-03	330E-02	Mrpl14	66412	Hypo
chr14	58590438	58591276	Distal Interger	-0675	302E-03	330E-02	Sod3	24569	Hypo
chr5	133504926	133506707	Distal Interger	-0377	302E-03	331E-02	RGD1563049	36794	Hypo
chr8	99645511	99647182	Exon (NM_05:	-0430	302E-03	331E-02	Pik3cb	19154	Hypo
chr6	69165320	69168590	Exon (NM_00:	-0333	302E-03	331E-02	Strn3	-31218	Hypo
chr2	169850192	169852323	Intron (NM_0:	-0425	303E-03	331E-02	Fhdc1	-22296	Hypo
chr1	166987253	166988591	Intron (NM_0(	-0454	303E-03	331E-02	Tead1	194633	Hypo
chr1	19741064	19741516	Distal Interger	-0726	303E-03	332E-02	Smlr1	-95874	Hypo
chr6	41677861	41679697	Intron (NM_0!	-0385	304E-03	332E-02	Kidins220	59567	Hypo
chr5	36623389	36624627	Distal Interger	-0507	305E-03	333E-02	Pou3f2	-539551	Hypo
chr1	65725635	65726288	Distal Interger	-0672	305E-03	333E-02	Cacng6	19983	Hypo
chr4	173199389	173199946	Distal Interger	-0602	305E-03	333E-02	Plekha5	-134109	Hypo
chr5	166184973	166186246	Promoter (<=:	-0276	305E-03	333E-02	Slc35e2b	0	Hypo
chr1	203053679	203055166	Exon (NM_00:	-0503	305E-03	333E-02	Scyl1	4317	Hypo
chr18	50860969	50863558	Distal Interger	-0373	305E-03	333E-02	Prrc1	48404	Hypo

chr14	44462061	44465377	Intron (NM_0(	-0348	305E-03	333E-02	LOC498368	12664	Hypo
chr16	25057302	25071644	Intron (NM_0	-0238	306E-03	333E-02	Cpe	27026	Hypo
chr2	218159564	218163649	Intron (NM_1:	-0343	306E-03	334E-02	Elovl6	95729	Hypo
chr1	13015774	13017803	Distal Interger	-0436	306E-03	334E-02	Ccdc28a	-143068	Hypo
chr4	147569877	147573259	Distal Interger	-0288	307E-03	334E-02	Hrh1	-72736	Hypo
chr2	231035320	231037017	Intron (NM_0!	-0446	307E-03	334E-02	Pdlim5	83206	Hypo
chr7	54493525	54494031	Distal Interger	-0641	308E-03	335E-02	Dyrk2	-131495	Hypo
chr20	2980869	2981302	Distal Interger	-0739	308E-03	335E-02	Cb707485	43062	Hypo
chr6	50747362	50747831	Distal Interger	-0753	308E-03	335E-02	Twist1	72452	Hypo
chr9	42267944	42272163	Intron (NM_0(	-0312	309E-03	336E-02	Map4k4	66324	Hypo
chr2	28925774	28927979	Distal Interger	-0311	309E-03	336E-02	Hexb	-421609	Hypo
chr3	136323206	136327993	Distal Interger	-0287	310E-03	336E-02	Cst3	12803	Hypo
chr4	147197808	147209162	Promoter (<=:	-0266	310E-03	337E-02	Atp2b2	0	Hypo
chr18	59320405	59335113	Promoter (<=:	-0272	310E-03	337E-02	Sec11c	0	Hypo
chr10	15406464	15411986	Promoter (<=:	-0378	311E-03	337E-02	Rhbdf1	0	Hypo
chr16	74164049	74164923	Distal Interger	-0558	311E-03	337E-02	Myom2	425217	Hypo
chr7	104788792	104791952	Intron (NM_0(	-0332	312E-03	338E-02	Trappc9	206400	Hypo
chr9	57415127	57416154	Distal Interger	-0522	312E-03	338E-02	Plcl1	513554	Hypo
chr4	68798664	68801793	Intron (NM_0(	-0358	312E-03	338E-02	Tmem178b	139239	Hypo
chr17	53894027	53897443	Distal Interger	-0309	312E-03	338E-02	Bambi	228373	Hypo
chr3	146705941	146706559	Intron (NM_0(	-0627	312E-03	338E-02	Rprd1b	22966	Hypo
chr18	78874082	78875171	Distal Interger	-0566	313E-03	339E-02	Timm21	-554720	Hypo
chr13	54854182	54855050	Distal Interger	-0548	313E-03	339E-02	B3galt2	-534356	Hypo
chr10	20543675	20545070	Exon (NM_02(	-0458	313E-03	339E-02	Rars1	-248483	Hypo
chr4	68366247	68368446	Promoter (<=:	-0272	313E-03	339E-02	Ndufb2	0	Hypo
chr8	24010090	24013133	Intron (NM_0(	-0353	313E-03	339E-02	Eepd1	51960	Hypo
chr2	167635711	167640714	Distal Interger	-0308	314E-03	339E-02	Map9	-67959	Hypo
chr8	21987334	21990433	Distal Interger	-0368	314E-03	339E-02	Bmper	255109	Hypo
chr6	95722857	95724268	Distal Interger	-0493	314E-03	339E-02	Max	-60891	Hypo
chr4	84538234	84544089	Distal Interger	-0287	314E-03	339E-02	Ghrhr	18542	Hypo
chr4	150518086	150518372	Distal Interger	-0800	315E-03	340E-02	Cxcl12	129760	Hypo
chr11	60171350	60172272	Distal Interger	-0602	315E-03	340E-02		-135554	Hypo
chr2	226668316	226671005	Promoter (<=:	-0271	315E-03	340E-02	Trmt10a	0	Hypo

chr5	83799335	83799986	Intron (NM_1	-0587	315E-03	340E-02	Cdk5rap2	160796	Hypo
chr7	95756739	95761686	Promoter (<=1	-0280	315E-03	340E-02	Cyrib	0	Hypo
chr1	165789993	165793680	Intron (NM_0	-0288	316E-03	341E-02	Galnt18	110588	Hypo
chr6	27107785	27109718	Distal Intergen	-0392	316E-03	341E-02	Adcy3	-15110	Hypo
chr17	8317063	8319978	Promoter (<=1	-0322	316E-03	341E-02	Cxcl14	0	Hypo
chr5	61624519	61626133	Distal Intergen	-0487	316E-03	341E-02	Tgfbr1	-27666	Hypo
chr15	76195386	76196235	Distal Intergen	-0588	316E-03	341E-02	Klf5	131128	Hypo
chr3	41833498	41836660	5' UTR	-0333	317E-03	341E-02	Gpd2	30724	Hypo
chr1	143728387	143731034	Exon (NM_001	-0349	317E-03	342E-02	Ccdc81	59892	Hypo
chr13	95665905	95666382	Distal Intergen	-0751	317E-03	342E-02	Dusp10	51613	Hypo
chr8	11567766	11568345	Intron (NM_0	-0650	317E-03	342E-02	Piwil4	11416	Hypo
chr5	61607294	61609797	3' UTR	-0364	318E-03	343E-02	Tgfbr1	-44002	Hypo
chr4	21561220	21561667	Distal Intergen	-0708	318E-03	343E-02	Sema3a	-67946	Hypo
chr14	94610828	94612219	Distal Intergen	-0411	318E-03	343E-02	Slc1a4	-50638	Hypo
chr10	69220340	69227272	Exon (NM_02	-0269	318E-03	343E-02	Acaca	137518	Hypo
chr10	30882587	30883129	Promoter (<=1	-0641	318E-03	343E-02	Havcr2	0	Hypo
chr15	53138794	53140503	Distal Intergen	-0498	319E-03	343E-02	Dnajc15	109172	Hypo
chr6	102221341	102222144	Distal Intergen	-0536	319E-03	343E-02	Rgs6	-43151	Hypo
chr13	39456770	39464179	Distal Intergen	-0279	319E-03	343E-02	Mir3473	-35067	Hypo
chr1	146378888	146381228	Intron (NM_0	-0388	319E-03	343E-02	Ccdc90b	-251528	Hypo
chr2	215710350	215712712	Distal Intergen	-0364	320E-03	344E-02	Larp7	300097	Hypo
chr10	45107991	45112665	Intron (NM_0	-0278	320E-03	344E-02	Tom1l2	45367	Hypo
chr11	79761202	79762545	Distal Intergen	-0404	320E-03	344E-02	Ephb3	96825	Hypo
chr16	44926883	44928223	Distal Intergen	-0349	321E-03	345E-02	Stox2	-46308	Hypo
chr2	196784181	196785895	Exon (NM_001	-0462	321E-03	345E-02	Slc25a24	22819	Hypo
chr13	92202915	92209215	Distal Intergen	-0312	321E-03	345E-02	Parp1	-98378	Hypo
chr2	27102371	27102994	Distal Intergen	-0648	321E-03	345E-02	Ndufs6-ps1	-98719	Hypo
chr6	116717762	116718805	Distal Intergen	-0503	322E-03	345E-02	Galc	797006	Hypo
chr1	154423291	154423772	Intron (NM_0	-0708	322E-03	345E-02	Pold3	32908	Hypo
chr19	20705593	20707982	Distal Intergen	-0350	322E-03	345E-02	Abcc12	156188	Hypo
chr4	63973337	63974665	Intron (NM_0	-0473	322E-03	345E-02	Slc13a4	13503	Hypo
chr3	153699414	153701466	Promoter (2-3	-0393	323E-03	345E-02	Slc12a5	-2436	Hypo
chr6	40641568	40643554	Distal Intergen	-0362	323E-03	345E-02	Hpcal1	163356	Hypo

chr1	214726292	214726528	Promoter (2-3	-0902	323E-03	345E-02	Gcnt1	-2944	Hypo
chr1	134148248	134153741	Promoter (<=1	-0299	323E-03	345E-02	Sema4b	0	Hypo
chr8	46310476	46315887	Promoter (<=1	-0257	323E-03	345E-02	Sik3	0	Hypo
chr1	255561028	255562776	Distal Interger	-0421	323E-03	346E-02	Dclre1a	25951	Hypo
chr2	51710415	51715911	Distal Interger	-0271	323E-03	346E-02	Zfp131	36673	Hypo
chr16	32440609	32441648	Exon (NM_00	-0578	324E-03	346E-02	Galnt7	-137042	Hypo
chr7	100418703	100420433	Distal Interger	-0409	324E-03	346E-02	Mir30d	-282231	Hypo
chr4	81941407	81941765	Distal Interger	-0814	324E-03	346E-02	Tax1bp1	119341	Hypo
chr17	30635514	30645240	Intron (NM_0	-0261	324E-03	346E-02	Psmg4	72784	Hypo
chr1	245890332	245891221	Distal Interger	-0599	324E-03	346E-02	Ina	-5554	Hypo
chr8	102021835	102024369	Distal Interger	-0316	324E-03	346E-02	Ppp2r3a	-223314	Hypo
chr8	57065157	57090820	Distal Interger	-0252	324E-03	346E-02	Lingo1	-36984	Hypo
chr17	17719123	17720540	Distal Interger	-0359	325E-03	347E-02	Nhlrc1	44748	Hypo
chr7	110152302	110154338	Promoter (<=1	-0377	325E-03	347E-02	Cyth4	26	Hypo
chr4	25940560	25941244	Distal Interger	-0632	325E-03	347E-02	Sri	46502	Hypo
chr7	43046139	43047393	Distal Interger	-0526	326E-03	347E-02	Ptprq	-29222	Hypo
chr10	53814513	53818519	Downstream (	-0308	326E-03	347E-02	Hes7	-7055	Hypo
chr15	30356183	30356897	Distal Interger	-0662	326E-03	347E-02	Gzmb	-9391	Hypo
chr4	123767922	123776746	Distal Interger	-0284	326E-03	347E-02	Hdac11	117765	Hypo
chr5	97558814	97559621	Distal Interger	-0658	326E-03	347E-02	Frem1	-117519	Hypo
chr10	53999418	54001683	Distal Interger	-0335	326E-03	347E-02	Gucy2d	-25351	Hypo
chr1	212142903	212143343	Distal Interger	-0726	326E-03	347E-02	Tle4	-345041	Hypo
chr9	65680628	65682680	Distal Interger	-0306	326E-03	347E-02	Klf7	-195908	Hypo
chr11	79329389	79330020	Distal Interger	-0673	326E-03	347E-02	Ehhadh	87462	Hypo
chr15	39501791	39502367	Intron (NM_0	-0639	326E-03	347E-02	Fbxo16	9299	Hypo
chr1	201764805	201767337	Distal Interger	-0399	327E-03	348E-02	Syt12	-5196	Hypo
chr7	29541390	29544584	Distal Interger	-0353	327E-03	348E-02	Cep83	260971	Hypo
chr8	89716217	89717270	Promoter (<=1	-0585	327E-03	348E-02	Bcl2a1	0	Hypo
chr7	33297029	33300051	Distal Interger	-0317	328E-03	348E-02	Atp2b1	-435924	Hypo
chr13	42826706	42828778	Intron (NM_0	-0411	328E-03	349E-02	Ikbke	-89563	Hypo
chr8	107358486	107359725	Intron (NM_0	-0507	328E-03	349E-02	Tex264	-35781	Hypo
chr7	46740708	46743899	Exon (NM_00	-0325	328E-03	349E-02	Bbs10	-7203	Hypo
chr13	101831814	101835588	Distal Interger	-0327	329E-03	349E-02	Prox1	-112010	Hypo

chr7	45495015	45495675	Distal Interger	-0580	329E-03	350E-02	Nav3	-102756	Hypo
chr1	193822353	193828229	Exon (NM_00	-0296	329E-03	350E-02	Jakmip3	10840	Hypo
chr8	46107185	46108658	Distal Interger	-0439	329E-03	350E-02	Bace1	-33466	Hypo
chr17	30777262	30778512	Distal Interger	-0416	330E-03	350E-02	Tubb2a	-18330	Hypo
chr2	28858978	28859808	Distal Interger	-0521	330E-03	350E-02	Hexb	-354813	Hypo
chr16	45684060	45684498	Promoter (2-3	-0718	330E-03	350E-02	Casp3	-2957	Hypo
chr4	119252370	119253059	Distal Interger	-0670	331E-03	350E-02	Anxa4	-11217	Hypo
chr4	114176123	114176759	Distal Interger	-0643	331E-03	351E-02	LOC10091137	286349	Hypo
chr9	41542274	41545307	Intron (NM_0	-0424	331E-03	351E-02	Npas2	78444	Hypo
chr4	16730265	16731936	Distal Interger	-0442	331E-03	351E-02	Gnai1	-82079	Hypo
chr6	94277064	94279881	Intron (NM_0	-0387	332E-03	351E-02	Ppp2r5e	37991	Hypo
chr6	23510326	23511492	Intron (NM_0	-0508	332E-03	351E-02	Clip4	186133	Hypo
chr7	132367555	132373771	Distal Interger	-0322	332E-03	351E-02	Nr4a1	-7620	Hypo
chr5	31812276	31815874	Distal Interger	-0327	332E-03	351E-02	Mmp16	499996	Hypo
chr20	20622950	20624638	Distal Interger	-0475	332E-03	352E-02	Rtkn2	-12999	Hypo
chr16	6820988	6821293	Distal Interger	-0866	333E-03	352E-02	Colq	-33881	Hypo
chr10	1900142	1903160	Intron (NM_0	-0337	334E-03	353E-02	Bmerb1	43415	Hypo
chr15	4159034	4161296	Exon (NM_00	-0375	334E-03	353E-02	Kcnk5	36592	Hypo
chr14	68704953	68707092	Distal Interger	-0419	334E-03	353E-02	Rab28	-538934	Hypo
chr7	122335475	122337562	Distal Interger	-0367	334E-03	353E-02	Abcd2	-23833	Hypo
chr16	4676691	4677778	Intron (NM_1	-0519	334E-03	353E-02	Cacna2d3	234573	Hypo
chr13	82604593	82606688	Intron (NM_1	-0340	334E-03	353E-02	Spata46	63410	Hypo
chr17	7353255	7353510	Distal Interger	-0949	335E-03	353E-02	Trpc7	-356073	Hypo
chr10	98299786	98300704	Intron (NM_0	-0585	335E-03	353E-02	Mir297	-83242	Hypo
chr5	59626028	59631229	Exon (NM_00	-0361	335E-03	353E-02	Dcaf10	18168	Hypo
chr4	14898123	14898948	Intron (NM_0	-0590	335E-03	353E-02	Phtf2	682860	Hypo
chr13	72691276	72692002	Intron (NM_0	-0578	335E-03	353E-02	Gpr52	112987	Hypo
chr13	69365388	69367342	Intron (NM_0	-0393	336E-03	354E-02	Rasal2	64293	Hypo
chr9	98655165	98657542	Distal Interger	-0398	336E-03	354E-02	Nudt12	97497	Hypo
chr1	222350118	222350944	Intron (NM_0	-0526	336E-03	354E-02	Tmem252	33136	Hypo
chr1	209152635	209154548	Distal Interger	-0381	336E-03	354E-02	Or5bb12	-31093	Hypo
chr7	91177711	91181864	Distal Interger	-0337	336E-03	354E-02	LOC690120	24307	Hypo
chr13	39441251	39443265	Distal Interger	-0406	337E-03	355E-02	Mir3473	-55981	Hypo

chr4	172997737	172999695	Distal Interger	-0366	337E-03	355E-02	Capza3	68464	Hypo
chr2	204879545	204881104	Distal Interger	-0437	337E-03	355E-02	Palmd	60251	Hypo
chr16	8829389	8833520	Distal Interger	-0288	337E-03	355E-02	Ptpn20	-59804	Hypo
chr15	76463649	76464091	Distal Interger	-0827	338E-03	355E-02	Klf5	399391	Hypo
chr13	38125511	38127996	Distal Interger	-0345	338E-03	355E-02	Mgat5	-548123	Hypo
chr9	11530743	11534394	Distal Interger	-0333	338E-03	355E-02	Mocs1	33396	Hypo
chr6	130816082	130818018	Distal Interger	-0398	338E-03	355E-02	Klc1	-5426	Hypo
chr1	90877317	90879025	Distal Interger	-0466	338E-03	355E-02	Plekhf1	31082	Hypo
chr4	59132156	59132566	Intron (NM_0(	-0838	339E-03	356E-02	Ssmem1	3426	Hypo
chr9	4896229	4897529	Distal Interger	-0562	339E-03	356E-02	Satb1	-142978	Hypo
chr19	9142823	9143102	Distal Interger	-0827	339E-03	356E-02	Got2	-31202	Hypo
chr3	37163073	37169914	Distal Interger	-0274	339E-03	356E-02	Stam2	64896	Hypo
chr14	19076049	19077802	Intron (NM_0!	-0446	340E-03	357E-02	Slc4a4	106382	Hypo
chr20	49145262	49146529	Intron (NM_0(	-0523	341E-03	357E-02	Hace1	109363	Hypo
chr13	72571186	72571945	Intron (NM_0(	-0587	341E-03	357E-02	Cacybp	-123376	Hypo
chr7	32631486	32632568	Distal Interger	-0544	341E-03	357E-02	Ccer1	146293	Hypo
chr1	255709238	255709911	Distal Interger	-0664	341E-03	357E-02	Adrb1	-62306	Hypo
chr5	36081076	36095961	Promoter (<=1	-0262	341E-03	357E-02	Pou3f2	0	Hypo
chr1	224672072	224674686	Distal Interger	-0344	341E-03	357E-02	Vldlr	-139770	Hypo
chr19	1523562	1525309	Distal Interger	-0553	341E-03	357E-02	Cdh11	-623138	Hypo
chr14	4284962	4291021	5' UTR	-0298	341E-03	357E-02	Lrrc8c	24184	Hypo
chr17	70391245	70393120	Distal Interger	-0394	341E-03	357E-02	Celf2	-1016490	Hypo
chr20	36498351	36499810	Distal Interger	-0418	341E-03	357E-02	Hsf2	-320054	Hypo
chr6	38561471	38571331	Promoter (<=1	-0293	341E-03	357E-02	Trib2	0	Hypo
chr14	78023634	78025040	Intron (NM_0(	-0529	341E-03	357E-02	Sfi1	23215	Hypo
chr1	153656763	153663074	Distal Interger	-0263	342E-03	357E-02	Serpinh1	-5958	Hypo
chr13	39533396	39534666	Distal Interger	-0496	342E-03	357E-02	Mir3473	34150	Hypo
chr16	26649256	26652231	Intron (NM_0(	-0380	342E-03	357E-02	Spock3	163374	Hypo
chr3	80890510	80891691	Distal Interger	-0539	342E-03	358E-02	Api5	-487336	Hypo
chr18	50041524	50043329	Promoter (<=1	-0297	342E-03	358E-02	Aldh7a1	0	Hypo
chr5	144813272	144813659	Distal Interger	-0723	343E-03	358E-02	Eya3	-39629	Hypo
chrX	121421760	121422829	Distal Interger	-0596	343E-03	359E-02	Sh2d1a	48067	Hypo
chr1	152263229	152267198	Distal Interger	-0364	344E-03	359E-02	Myo7a	146959	Hypo

chr4	120358360	120362059	Distal Interger	-0300	344E-03	359E-02	Copg1	-4686	Hypo
chr20	46763378	46765675	Intron (NM_0(	-0425	344E-03	359E-02	Pdss2	53672	Hypo
chr4	153564572	153567135	Promoter (<=:	-0257	344E-03	360E-02	Kdm5a	0	Hypo
chr11	57225830	57230946	5' UTR	-0307	345E-03	360E-02	Mir568	-171757	Hypo
chr4	65361029	65366706	Intron (NM_0	-0287	345E-03	360E-02	Ptn	8600	Hypo
chr4	79839896	79843281	Distal Interger	-0298	345E-03	360E-02	Npvf	-123660	Hypo
chr16	55769000	55772086	Intron (NM_0(	-0335	346E-03	360E-02	Trmt9b	20058	Hypo
chr19	11431815	11439845	Distal Interger	-0245	346E-03	360E-02	Ces5a	-29624	Hypo
chr4	14155179	14156502	Intron (NM_0(	-0508	346E-03	360E-02	Aptr	-15602	Hypo
chr8	30902952	30904371	Intron (NM_0(	-0491	346E-03	360E-02	Fli1	46062	Hypo
chr8	67914112	67914687	Distal Interger	-0642	347E-03	361E-02	Mir190a-2	-63041	Hypo
chr8	47747940	47750031	Distal Interger	-0418	348E-03	362E-02	Cadm1	-97805	Hypo
chr1	109589381	109590768	Intron (NM_0(	-0517	348E-03	362E-02	Atp10a	32599	Hypo
chr13	48126612	48129618	Distal Interger	-0364	348E-03	362E-02	Zfp281	30437	Hypo
chr10	15866738	15870523	Distal Interger	-0278	349E-03	363E-02	Cpeb4	-87626	Hypo
chr2	88286693	88288931	Distal Interger	-0431	349E-03	363E-02	Ralyl	-535689	Hypo
chr9	78885838	78888231	Intron (NM_0(	-0401	349E-03	363E-02	Epha4	69908	Hypo
chr17	71390761	71391489	Distal Interger	-0538	349E-03	363E-02	Celf2	-18121	Hypo
chr1	127347961	127352532	Distal Interger	-0305	349E-03	363E-02	Chd2	-47461	Hypo
chr8	37598772	37601396	Distal Interger	-0398	349E-03	363E-02	Or8b12c	10013	Hypo
chr20	35752191	35752926	Distal Interger	-0542	350E-03	363E-02	Gja1	-3109	Hypo
chr8	107313111	107313581	Intron (NM_0(	-0574	350E-03	364E-02	Tex264	9124	Hypo
chr7	131763713	131768168	Promoter (<=:	-0358	350E-03	364E-02	Bin2	40	Hypo
chr1	145437709	145438636	Intron (NM_0:	-0551	351E-03	364E-02	Dlg2	310534	Hypo
chr10	7234739	7236188	Distal Interger	-0494	351E-03	364E-02	Tmem114	64993	Hypo
chr14	34191456	34194990	Intron (NM_0(	-0320	351E-03	364E-02	Usp46	41401	Hypo
chr14	98075298	98106908	Intron (NM_0(	-0243	351E-03	365E-02	Bcl11a	44730	Hypo
chr2	23479002	23479978	Intron (NM_0(	-0558	351E-03	365E-02	Msh3	105799	Hypo
chr15	82283213	82286049	Distal Interger	-0362	351E-03	365E-02	Ndfip2	250847	Hypo
chr13	48179468	48182790	Distal Interger	-0333	352E-03	365E-02	Zfp281	83293	Hypo
chr1	148870858	148871394	Distal Interger	-0617	352E-03	365E-02	Fam181b	1704430	Hypo
chr5	97374989	97377446	Exon (NM_00:	-0335	352E-03	365E-02	Frem1	63849	Hypo
chr12	45274586	45276056	Intron (NM_0:	-0432	352E-03	365E-02	Pitpnb	22226	Hypo

chr5	156546954	156547485	Distal Interger	-0724	352E-03	365E-02	RGD1308878	190086	Hypo
chr12	19905603	19906785	Intron (NM_0(	-0507	352E-03	365E-02	Col26a1	112875	Hypo
chr9	39720910	39721306	Intron (NM_0(	-0763	352E-03	365E-02	Mgat4a	45584	Hypo
chr3	114816231	114818940	Promoter (2-3	-0321	353E-03	365E-02	Mrps5	-2106	Hypo
chr7	108025433	108027645	Promoter (<=1	-0386	353E-03	365E-02	Oplah	0	Hypo
chr15	47159277	47159570	Distal Interger	-0816	353E-03	365E-02	Fndc3a	673010	Hypo
chr9	41914312	41914893	Distal Interger	-0690	353E-03	366E-02	Cnot11	99389	Hypo
chr8	36677372	36686283	Intron (NM_0(	-0260	354E-03	366E-02	Pknox2	14081	Hypo
chr2	224901769	224902577	Distal Interger	-0597	354E-03	367E-02	Bank1	-101566	Hypo
chr6	77423344	77423763	Distal Interger	-0708	354E-03	367E-02	Fbxo33	-491007	Hypo
chr1	141388452	141389050	Intron (NM_0	-0646	354E-03	367E-02	Grm5	67323	Hypo
chr12	18910630	18915127	Promoter (<=1	-0248	354E-03	367E-02	Mepce	0	Hypo
chrX	36534452	36536560	Distal Interger	-0439	355E-03	367E-02	Cnksr2	-371575	Hypo
chr10	64290511	64291388	Distal Interger	-0577	355E-03	367E-02	Nf1	-14913	Hypo
chr10	71665050	71671225	Distal Interger	-0269	355E-03	367E-02	Dhx40	-43571	Hypo
chr14	31494823	31495251	Distal Interger	-0708	355E-03	367E-02	Exoc1	164625	Hypo
chr13	33161981	33162928	Distal Interger	-0526	355E-03	367E-02	Ddx18	-418820	Hypo
chr1	165420261	165426323	Distal Interger	-0267	356E-03	368E-02	Eif4g2	-226678	Hypo
chr16	51283109	51284377	Distal Interger	-0438	356E-03	368E-02	Mtus1	-10860	Hypo
chr16	75925653	75926774	Intron (NM_0:	-0588	356E-03	368E-02	LOC290876	-48689	Hypo
chr2	165390975	165392289	Distal Interger	-0406	356E-03	368E-02	Gask1b	343258	Hypo
chr14	33707497	33708988	Exon (NM_00:	-0478	357E-03	369E-02	Scfd2	24149	Hypo
chr3	133297549	133306937	3' UTR	-0269	357E-03	369E-02	Naa20	-15127	Hypo
chr3	153160388	153174093	Promoter (<=1	-0269	357E-03	369E-02	Sdc4	0	Hypo
chr2	117981763	117986049	Distal Interger	-0312	357E-03	369E-02	Sox2	444834	Hypo
chr8	45872065	45874146	Intron (NM_0(	-0387	358E-03	369E-02	Dscaml1	131767	Hypo
chr17	53416733	53418545	Distal Interger	-0421	358E-03	369E-02	Map3k8	33797	Hypo
chr19	13191706	13194110	Distal Interger	-0362	359E-03	370E-02	Hmgxb4	-170695	Hypo
chr11	77550312	77551339	Intron (NM_0(	-0533	359E-03	370E-02	St6gal1	-14552	Hypo
chr9	88326341	88331811	Intron (NM_0	-0257	359E-03	370E-02	Inpp5d	38662	Hypo
chr7	129841266	129841723	Distal Interger	-0621	360E-03	370E-02	Rnd1	-12881	Hypo
chr20	6057816	6060943	Intron (NM_0(	-0334	360E-03	371E-02	Tcp11	74968	Hypo
chr18	34056969	34058197	Intron (NM_0(	-0563	360E-03	371E-02	Sh3rf2	10090	Hypo

chr18	34208848	34210787	Exon (NM_00:	-0409	360E-03	371E-02	Lars1	45104	Hypo
chr1	95716828	95721681	Promoter (2-3	-0277	360E-03	371E-02	Cd37	2654	Hypo
chr4	14820918	14821627	Intron (NM_0!	-0676	360E-03	371E-02	Phtf2	605655	Hypo
chr2	42934676	42937244	Distal Interger	-0323	360E-03	371E-02	Actbl2	76656	Hypo
chr13	30008221	30011479	Distal Interger	-0379	361E-03	371E-02	Gli2	-5404	Hypo
chr2	152187660	152188911	Intron (NM_0(	-0511	361E-03	371E-02	Schip1	60489	Hypo
chr6	63131441	63131880	Distal Interger	-0722	361E-03	371E-02	Nova1	773663	Hypo
chr8	113388997	113389427	Distal Interger	-0688	361E-03	371E-02	Pdcd6ip	257346	Hypo
chr9	59091788	59092849	Distal Interger	-0580	361E-03	371E-02	Tyw5	-112600	Hypo
chr8	102834578	102835861	Intron (NM_0(	-0542	362E-03	372E-02	Ephb1	108978	Hypo
chr14	91727637	91729038	Distal Interger	-0545	362E-03	372E-02	C1d	66686	Hypo
chr9	104406265	104408146	3' UTR	-0399	362E-03	372E-02	Man2a1	154264	Hypo
chr17	33802322	33803488	Intron (NM_0(	-0510	363E-03	373E-02	Dusp22	27833	Hypo
chr1	176785684	176787029	Distal Interger	-0489	363E-03	373E-02	Chp2	16705	Hypo
chr10	40392088	40394158	Distal Interger	-0443	363E-03	373E-02	Nmur2	-210010	Hypo
chr3	65213106	65214457	Distal Interger	-0476	364E-03	373E-02	Dnajc10	-18240	Hypo
chr3	161943889	161951285	Distal Interger	-0268	364E-03	374E-02	Pck1	13633	Hypo
chr3	114820679	114821965	Promoter (<=:	-0306	365E-03	374E-02	Mrps5	0	Hypo
chr16	42942892	42943382	Distal Interger	-0653	365E-03	374E-02	Tenm3	-584612	Hypo
chr3	43585909	43586393	Intron (NM_0(	-0793	365E-03	374E-02	Ccdc148	45598	Hypo
chr19	53978699	53983588	Intron (NM_0:	-0315	365E-03	374E-02	Kcnk1	19042	Hypo
chr10	56507858	56508422	Distal Interger	-0665	365E-03	374E-02	Wscd1	111984	Hypo
chr17	70874261	70877521	Distal Interger	-0318	365E-03	374E-02	Celf2	-532089	Hypo
chr1	185422993	185423588	Distal Interger	-0593	365E-03	374E-02	Plekha1	-4460	Hypo
chr17	18122798	18124802	Intron (NM_0!	-0388	366E-03	374E-02	Cap2	78543	Hypo
chr9	60172092	60174141	Distal Interger	-0318	366E-03	374E-02	Cflar	-11235	Hypo
chr13	18379475	18380736	Intron (NM_0(	-0515	366E-03	375E-02	Cntnap5b	515638	Hypo
chr5	37624752	37625227	Distal Interger	-0673	366E-03	375E-02	Mms22l	-572552	Hypo
chr13	89487483	89490550	Distal Interger	-0360	366E-03	375E-02	Zbtb18	43170	Hypo
chr10	27130712	27136496	Intron (NM_0	-0294	366E-03	375E-02	Gabrb2	192472	Hypo
chr1	225526329	225526824	Intron (NM_0(	-0630	367E-03	376E-02	Rfx3	182018	Hypo
chr18	78490504	78491486	Distal Interger	-0554	367E-03	376E-02	Timm21	-171142	Hypo
chr15	32058310	32062243	Distal Interger	-0307	368E-03	376E-02	Zdhhc20	-19331	Hypo

chr1	177174079	177176277	Distal Interger	-0366	368E-03	376E-02	Cacng3	-25713	Hypo
chr19	24913812	24914724	Intron (NM_0(	-0601	368E-03	376E-02	Tbc1d9	28405	Hypo
chr19	48732784	48733813	Distal Interger	-0495	369E-03	377E-02	Cox4i1	11104	Hypo
chr4	61201956	61202463	Distal Interger	-0663	369E-03	377E-02	Plxna4	-39750	Hypo
chr19	51905654	51906956	Distal Interger	-0457	369E-03	377E-02	Acta1	-18929	Hypo
chr3	17492803	17493624	Exon (NM_001	-0596	369E-03	377E-02	Pbx3	188726	Hypo
chr5	61150769	61152662	Intron (NM_0:	-0483	369E-03	377E-02	Gabbr2	135442	Hypo
chr5	43031914	43032794	Intron (NM_1:	-0572	370E-03	377E-02	Epha7	56436	Hypo
chr5	25504240	25504585	Distal Interger	-0844	370E-03	377E-02	Pdp1	-49193	Hypo
chr14	7475408	7476006	Distal Interger	-0698	370E-03	378E-02	Wdfy3	-130622	Hypo
chr1	227731107	227731667	Exon (NM_001	-0619	370E-03	378E-02	Il33	9672	Hypo
chr8	33262565	33266413	Intron (NM_0(	-0319	370E-03	378E-02	St3gal4	198077	Hypo
chr7	106556047	106567558	Promoter (<=1	-0260	370E-03	378E-02	Arc	0	Hypo
chr15	38647561	38653790	Intron (NM_0!	-0317	370E-03	378E-02	Msra	22706	Hypo
chr2	98713399	98713902	Distal Interger	-0663	371E-03	378E-02	Ythdf3	362738	Hypo
chr3	21528948	21535519	Distal Interger	-0269	371E-03	378E-02	Crb2	-6702	Hypo
chr7	134537937	134539251	Distal Interger	-0542	371E-03	378E-02	Gtsf1	18173	Hypo
chr8	860403	861169	Intron (NM_0:	-0620	371E-03	378E-02	Gucy1a2	360191	Hypo
chr2	13464474	13465279	Distal Interger	-0580	371E-03	378E-02	Mir9-2	-732445	Hypo
chr3	119557026	119559951	Distal Interger	-0424	372E-03	378E-02	Cds2	42026	Hypo
chr3	117117666	117118335	Distal Interger	-0604	372E-03	378E-02	Stk35	100716	Hypo
chr13	71608178	71614267	Distal Interger	-0256	372E-03	378E-02	Mir1843b	56630	Hypo
chr11	85058146	85059443	Intron (NM_0(	-0492	372E-03	378E-02	Mzt2b	33870	Hypo
chr4	24961048	24962185	Intron (NM_0(	-0528	372E-03	378E-02	Tmem243	6457	Hypo
chr3	99360415	99361220	Promoter (1-2	-0633	373E-03	379E-02	Aven	-1355	Hypo
chr4	114345393	114345942	Distal Interger	-0679	373E-03	379E-02	LOC10091137	117166	Hypo
chr9	69528709	69529924	Distal Interger	-0464	374E-03	379E-02	Cps1	914556	Hypo
chr17	60117906	60121949	Intron (NM_0	-0295	375E-03	380E-02	Chrm3	112401	Hypo
chr15	35133240	35134140	Exon (NM_001	-0550	375E-03	380E-02	Tnfrsf19	24332	Hypo
chr1	224653239	224654231	Distal Interger	-0571	375E-03	380E-02	Vldlr	-160225	Hypo
chr1	257642190	257642814	Exon (NM_001	-0695	375E-03	381E-02	Ccdc172	12982	Hypo
chr18	42550122	42553697	Distal Interger	-0344	375E-03	381E-02	Dtwd2	348167	Hypo
chr15	95288867	95289435	Distal Interger	-0695	376E-03	381E-02	Gpr180	89051	Hypo

chr4	46985396	46986842	Distal Interger	-0491	376E-03	381E-02	Cttnbp2	-20765	Hypo
chr4	126800298	126801427	Intron (NM_0(	-0511	377E-03	382E-02	Magi1	9332	Hypo
chr9	32535795	32536715	Distal Interger	-0581	377E-03	382E-02	Phf3	599279	Hypo
chr5	79078168	79079081	Intron (NM_0(	-0561	377E-03	382E-02	Trim32	73000	Hypo
chr14	104106793	104107346	Distal Interger	-0610	378E-03	382E-02	C14h2orf73	71627	Hypo
chr15	37989252	37991388	Intron (NM_0(	-0438	378E-03	383E-02	Mir598	26560	Hypo
chr15	99028146	99029896	Intron (NM_0(	-0411	379E-03	383E-02	Gpr183	20583	Hypo
chr13	86469038	86471321	Distal Interger	-0392	380E-03	384E-02	Or6y1	75657	Hypo
chrX	119085238	119085945	Distal Interger	-0698	380E-03	384E-02	Gria3	-1153043	Hypo
chr3	90575146	90577093	Distal Interger	-0429	380E-03	384E-02	C3h11orf91	90539	Hypo
chr16	57302310	57303826	Exon (NM_00:	-0403	380E-03	384E-02	Tnks	77216	Hypo
chr3	61648338	61649017	Distal Interger	-0615	381E-03	385E-02	Plekha3	24904	Hypo
chr4	51396618	51399774	Promoter (<=:	-0326	382E-03	385E-02	Ptprz1	0	Hypo
chr20	45898429	45901407	Intron (NM_0(	-0328	382E-03	385E-02	Afg1l	66499	Hypo
chr9	86680350	86681801	Distal Interger	-0438	382E-03	385E-02	Spata3	19998	Hypo
chr10	92749542	92751246	Distal Interger	-0459	383E-03	386E-02	Cacng4	-3941	Hypo
chr10	62780907	62784012	Promoter (<=:	-0329	383E-03	386E-02	Pipox	0	Hypo
chr10	71722745	71727106	Distal Interger	-0273	383E-03	386E-02	Ypel2	76749	Hypo
chr7	102289637	102291525	Distal Interger	-0371	383E-03	386E-02	Khdrbs3	1451703	Hypo
chr6	19996568	19997289	Distal Interger	-0669	384E-03	387E-02	Rasgrp3	-38080	Hypo
chr12	11090513	11096451	Exon (NM_00:	-0280	384E-03	387E-02	Daglb	5703	Hypo
chr14	26868753	26870637	Distal Interger	-0385	384E-03	387E-02	Adgrl3	-44309	Hypo
chr1	114644168	114644953	Distal Interger	-0673	385E-03	387E-02	Mir344b-3	-11463	Hypo
chr4	28487579	28488337	Intron (NM_0(	-0529	385E-03	387E-02	Gtpbp10	10722	Hypo
chr13	81625462	81626794	Distal Interger	-0351	385E-03	387E-02	Nuf2	95971	Hypo
chr1	152187249	152188435	Intron (NM_0	-0549	386E-03	388E-02	Pak1	76039	Hypo
chr14	42806725	42808911	Intron (NM_0(	-0399	386E-03	388E-02	Smim14	23364	Hypo
chr9	44748505	44748968	Distal Interger	-0742	386E-03	388E-02	Pantr1	194663	Hypo
chr20	36120568	36122043	Distal Interger	-0383	387E-03	389E-02	Gja1	364533	Hypo
chr4	69974337	69975738	Distal Interger	-0432	387E-03	389E-02	Prss3b	-98819	Hypo
chr13	83145173	83146315	Distal Interger	-0508	387E-03	389E-02	Fcrlb	8939	Hypo
chr7	76287874	76289108	Distal Interger	-0449	388E-03	389E-02	Kcnv1	-20034	Hypo
chr5	151545539	151546306	Exon (NM_00:	-0571	388E-03	389E-02	Slc66a1	3519	Hypo

chr9	25552288	25553479	Distal Intergen	-0566	388E-03	389E-02	RGD1309049	-19490	Hypo
chr19	47011265	47015824	Intron (NM_1	-0346	388E-03	389E-02	Hsbp1	-385215	Hypo
chr11	35057122	35059631	Distal Intergen	-0373	388E-03	390E-02	Ets2	35586	Hypo
chr18	16015428	16016920	Intron (NM_0	-0427	388E-03	390E-02	Fhod3	22104	Hypo
chr8	47141879	47143852	Distal Intergen	-0454	388E-03	390E-02	LOC500990	-531405	Hypo
chr19	7410175	7411212	Distal Intergen	-0588	389E-03	390E-02	Got2	-1763092	Hypo
chr17	53166386	53168691	Distal Intergen	-0370	389E-03	390E-02	Mtpap	172790	Hypo
chr4	27035617	27036186	Distal Intergen	-0689	389E-03	390E-02	Tex47	-388224	Hypo
chr1	3298963	3301566	Distal Intergen	-0318	390E-03	391E-02	Ust	-336919	Hypo
chr1	199416286	199420821	Intron (NM_2	-0302	390E-03	391E-02	Shank2	-35453	Hypo
chr6	31278841	31281317	Distal Intergen	-0374	390E-03	391E-02	Rhob	84606	Hypo
chr17	12763468	12764958	Distal Intergen	-0457	391E-03	391E-02	Diras2	5903	Hypo
chr1	137791342	137792826	Distal Intergen	-0421	391E-03	391E-02	Cfap161	-22657	Hypo
chr8	15956927	15957469	Distal Intergen	-0743	391E-03	392E-02	Or7g25c	-66191	Hypo
chr10	30413497	30414353	Distal Intergen	-0641	391E-03	392E-02	Thg1l	-16918	Hypo
chr10	92857539	92858226	Distal Intergen	-0685	391E-03	392E-02	Cacng5	-20538	Hypo
chr14	57794061	57796424	Distal Intergen	-0326	391E-03	392E-02	Smim20	-71380	Hypo
chr7	46854253	46857707	Distal Intergen	-0319	392E-03	392E-02	Nap1l1	-75585	Hypo
chr18	73503578	73505028	Intron (NM_0	-0497	392E-03	392E-02	Pard6g	5459	Hypo
chr5	59311505	59311773	Distal Intergen	-0943	392E-03	392E-02	Polr1e	32049	Hypo
chr8	113263315	113270393	Distal Intergen	-0263	392E-03	392E-02	Pdcd6ip	376380	Hypo
chr1	145125312	145128655	Promoter (<=1	-0281	392E-03	392E-02	Dlg2	0	Hypo
chr9	42666490	42666964	Distal Intergen	-0729	392E-03	392E-02	Il1rl1	-9711	Hypo
chr2	118207254	118208016	Distal Intergen	-0686	392E-03	392E-02	Dcun1d1	559302	Hypo
chr8	5619149	5620128	Distal Intergen	-0608	393E-03	392E-02	Rpl13	78177	Hypo
chr8	68782866	68783427	Distal Intergen	-0630	393E-03	393E-02	LOC10091136	-445909	Hypo
chr1	129029369	129029904	Intron (NM_0	-0709	394E-03	393E-02	Sv2b	122575	Hypo
chr8	74789658	74790491	Distal Intergen	-0539	394E-03	394E-02	Wdr72	-69376	Hypo
chr17	26268650	26269514	Distal Intergen	-0571	394E-03	394E-02	Txndc5	-20366	Hypo
chr9	89179806	89180712	Distal Intergen	-0448	394E-03	394E-02	Arl4c	123058	Hypo
chr18	35660929	35661710	Intron (NM_0	-0616	395E-03	394E-02	Jakmip2	86065	Hypo
chr7	20616685	20620878	Intron (NM_0	-0380	395E-03	394E-02	Chst11	121983	Hypo
chr10	68071678	68072971	Distal Intergen	-0311	396E-03	395E-02	Pex12	26436	Hypo

chr10	85246650	85248223	Promoter (<=1	-0290	396E-03	395E-02	Eif1	0	Hypo
chr13	104796427	104799324	Distal Interger	-0369	397E-03	395E-02	G0s2	7939	Hypo
chr18	30661024	30662118	Distal Interger	-0563	397E-03	396E-02	Fgf1	48163	Hypo
chr8	28624401	28625702	Distal Interger	-0537	397E-03	396E-02	Snx19	-204827	Hypo
chr3	114877728	114878781	Intron (NM_0	-0526	398E-03	396E-02	Mal	9355	Hypo
chr7	98236120	98237708	Exon (NM_00	-0425	398E-03	396E-02	Dnaaf11	8129	Hypo
chr19	44664418	44668807	Promoter (1-2	-0301	398E-03	396E-02	Cdyl2	1757	Hypo
chr6	66663244	66694460	Promoter (<=1	-0224	398E-03	396E-02	Foxg1	0	Hypo
chr1	216385607	216386739	Exon (NM_00	-0507	399E-03	397E-02	Rorb	157651	Hypo
chr18	35661825	35662777	Intron (NM_0	-0494	399E-03	397E-02	Jakmip2	84998	Hypo
chr5	140584851	140586764	Distal Interger	-0371	399E-03	397E-02	Hmgb4	29958	Hypo
chr8	112420181	112420882	Distal Interger	-0624	399E-03	397E-02	Arpp21	-267150	Hypo
chr4	178808157	178810400	Distal Interger	-0351	399E-03	397E-02	Sspn	-86747	Hypo
chr15	46830878	46832910	Distal Interger	-0422	399E-03	397E-02	Gfra2	888427	Hypo
chr4	41587605	41589929	Distal Interger	-0387	400E-03	397E-02	Tas2r7l	18069	Hypo
chr8	45007016	45010973	Exon (NM_00	-0296	400E-03	398E-02	Treh	16834	Hypo
chr16	56651134	56651888	Distal Interger	-0604	400E-03	398E-02	Mfhas1	-18029	Hypo
chr1	96902477	96908472	Promoter (<=1	-0263	400E-03	398E-02	Kcnc1	0	Hypo
chr12	27563298	27565059	Distal Interger	-0432	400E-03	398E-02	Ran	113189	Hypo
chr20	24547177	24549489	Intron (NM_0	-0374	401E-03	398E-02	Lrrtm3	31550	Hypo
chr10	63042711	63044072	Intron (NM_0	-0480	401E-03	398E-02	Traf4	16212	Hypo
chr5	129617079	129620077	3' UTR	-0333	401E-03	398E-02	Lurap1	8574	Hypo
chr12	44011659	44012883	Distal Interger	-0519	402E-03	399E-02	Sez6l	-52052	Hypo
chr14	6532842	6539756	Intron (NM_0	-0259	402E-03	399E-02	Mapk10	35135	Hypo
chr5	54171114	54171781	Distal Interger	-0574	402E-03	399E-02	RGD1306195	-284224	Hypo
chr5	16734172	16734704	Intron (NM_0	-0672	402E-03	399E-02	Rps20	85771	Hypo
chr1	167746543	167747187	Distal Interger	-0581	402E-03	399E-02	Far1	89394	Hypo
chr2	175838748	175839548	Distal Interger	-0558	402E-03	399E-02	Slc27a3	18361	Hypo
chr15	45376876	45378322	3' UTR	-0407	402E-03	399E-02	Ppp3cc	-15044	Hypo
chr19	49274626	49276220	Distal Interger	-0374	403E-03	399E-02	Foxc2	88592	Hypo
chr5	115854919	115857899	Intron (NM_0	-0330	403E-03	399E-02	Jak1	23501	Hypo
chr3	139491995	139501095	Promoter (<=1	-0258	403E-03	399E-02	Acss1	0	Hypo
chr18	48267116	48267591	Distal Interger	-0696	403E-03	400E-02	Zfp608	142874	Hypo

chr15	45284078	45287434	Promoter (<=1	-0310	403E-03	400E-02	Sorbs3	0	Hypo
chr20	19450139	19452942	Distal Interger	-0359	403E-03	400E-02	Rhobtb1	-47127	Hypo
chr4	24362837	24370598	Promoter (<=1	-0247	404E-03	400E-02	Grm3	0	Hypo
chr13	79496171	79496798	Distal Interger	-0667	404E-03	400E-02	Aldh9a1	-8940	Hypo
chr16	36840605	36844165	Distal Interger	-0305	404E-03	400E-02	Gpm6a	-86445	Hypo
chr7	42181536	42185463	Exon (NM_001	-0317	404E-03	400E-02	Acss3	264767	Hypo
chr3	107397294	107397754	Intron (NM_0	-0669	404E-03	400E-02	Capn3	-10087	Hypo
chr1	252155404	252157934	Intron (NM_0	-0384	404E-03	400E-02	Add3	8018	Hypo
chr19	9658846	9664488	3' UTR	-0285	404E-03	400E-02	Mmp15	20455	Hypo
chr20	28277965	28279868	Promoter (1-2	-0457	404E-03	400E-02	Vsir	-1751	Hypo
chr2	28016787	28020359	Promoter (<=1	-0299	405E-03	400E-02	Hmgcr	0	Hypo
chr2	169847649	169849521	Intron (NM_0	-0388	405E-03	400E-02	Fhdc1	-19753	Hypo
chr3	151412441	151413085	Distal Interger	-0568	406E-03	401E-02	Srsf6	-176461	Hypo
chr14	6516497	6519807	Intron (NM_0	-0326	406E-03	401E-02	Mapk10	18790	Hypo
chr1	14316919	14320162	Distal Interger	-0338	407E-03	402E-02	Ifngr1	-13103	Hypo
chr15	95061500	95061737	Distal Interger	-1002	407E-03	402E-02	Tgds	133840	Hypo
chr4	79785482	79790254	Distal Interger	-0295	407E-03	402E-02	Npvf	-69246	Hypo
chr6	93619344	93624329	Distal Interger	-0344	407E-03	402E-02	Kcnh5	283778	Hypo
chr1	165011903	165012703	Intron (NM_0	-0586	407E-03	402E-02	Lyve1	-35594	Hypo
chr13	62823091	62825254	Exon (NM_001	-0393	407E-03	402E-02	Hmcn1	259270	Hypo
chr9	62073765	62074380	Distal Interger	-0652	407E-03	402E-02	Raph1	-83745	Hypo
chr2	20792276	20793227	Intron (NM_0	-0538	407E-03	402E-02	Vcan	67312	Hypo
chr5	16734969	16735287	Intron (NM_0	-0809	408E-03	402E-02	Rps20	85188	Hypo
chr13	97041761	97042521	Distal Interger	-0573	408E-03	402E-02	Slc30a10	43618	Hypo
chr8	113206993	113210257	Distal Interger	-0286	408E-03	402E-02	Pdcd6ip	436516	Hypo
chr4	91123682	91124081	Distal Interger	-0652	409E-03	403E-02	Ccser1	830902	Hypo
chr8	47967321	47967798	Intron (NM_0	-0660	409E-03	403E-02	Cadm1	119485	Hypo
chr6	105560825	105563288	Distal Interger	-0393	410E-03	404E-02	Erg28	-77747	Hypo
chr11	78637787	78639055	Intron (NM_0	-0440	410E-03	404E-02	Etv5	29077	Hypo
chr20	26441347	26443827	Promoter (<=1	-0273	410E-03	404E-02	Ranbp2	0	Hypo
chr3	100838686	100839277	Distal Interger	-0768	410E-03	404E-02	Actc1	-21163	Hypo
chr13	87560161	87561059	Promoter (2-3	-0670	410E-03	404E-02	Kmo	2818	Hypo
chr19	20576571	20577658	Intron (NM_1	-0523	410E-03	404E-02	Abcc12	27166	Hypo

chr8	119801188	119801646	Promoter (1-2	-0729	411E-03	404E-02	Cx3cr1	-1757	Hypo
chr14	87002933	87004830	Distal Interger	-0413	411E-03	405E-02	Cobl	-23394	Hypo
chr9	78294162	78294719	Distal Interger	-0657	412E-03	405E-02	Epha4	663420	Hypo
chr7	18851576	18852937	Distal Interger	-0535	412E-03	405E-02	Ric8b	-9208	Hypo
chr2	154509234	154510332	Distal Interger	-0471	412E-03	405E-02	Sptssb	-347107	Hypo
chr1	209105088	209107934	Distal Interger	-0395	412E-03	405E-02	Or5bb12	13608	Hypo
chr10	7572869	7573673	Distal Interger	-0491	413E-03	405E-02	Tmem114	403123	Hypo
chr2	111326639	111329394	Intron (NM_0(	-0329	413E-03	406E-02	Tnik	142252	Hypo
chr3	104198853	104205567	Intron (NM_0	-0278	413E-03	406E-02	Rasgrp1	24489	Hypo
chr13	82519058	82519683	Distal Interger	-0615	414E-03	406E-02	LOC10036108	18993	Hypo
chr18	31789277	31791228	Distal Interger	-0417	414E-03	406E-02	Pabpc2	135059	Hypo
chr13	87208259	87210237	Intron (NM_0	-0418	414E-03	406E-02	Rgs7	198386	Hypo
chr15	3491527	3492950	Distal Interger	-0408	414E-03	406E-02	Camk2g	-11143	Hypo
chr20	8412058	8414694	Intron (NM_0(	-0342	414E-03	407E-02	Btbd9	225150	Hypo
chr5	16599214	16600106	Distal Interger	-0501	414E-03	407E-02	Lyn	-39406	Hypo
chr2	98716705	98717640	Distal Interger	-0479	415E-03	407E-02	Ythdf3	366044	Hypo
chr18	31687720	31689036	Distal Interger	-0454	415E-03	407E-02	Pabpc2	33502	Hypo
chr18	27564314	27579825	Promoter (<=1	-0259	415E-03	407E-02	Psd2	0	Hypo
chr15	82328572	82329811	Distal Interger	-0531	416E-03	408E-02	Ndfip2	296206	Hypo
chr1	133304642	133305314	Distal Interger	-0550	416E-03	408E-02	Rlbp1	16982	Hypo
chr3	143841540	143842399	Intron (NM_1	-0502	416E-03	408E-02	Pigu	14831	Hypo
chr9	11699625	11699990	Distal Interger	-0722	417E-03	408E-02	Mocs1	-131835	Hypo
chr3	64918865	64919905	Intron (NM_0	-0516	417E-03	408E-02	Pde1a	104969	Hypo
chr4	136456230	136457432	Distal Interger	-0443	417E-03	408E-02	Cntn6	-897935	Hypo
chr9	106994918	106996155	Intron (NM_0(	-0485	417E-03	408E-02	Ptprm	347543	Hypo
chr14	103113	105355	Promoter (<=1	-0300	418E-03	408E-02	Csf2ra	0	Hypo
chrX	21961932	21962400	Intron (NM_0(	-0780	418E-03	409E-02	Shroom2	21099	Hypo
chr12	33153170	33155446	Distal Interger	-0326	418E-03	409E-02	Lrrc43	-60913	Hypo
chr3	82647642	82649772	Intron (NM_0(	-0392	418E-03	409E-02	Lrrc4c	342398	Hypo
chr16	17168127	17169297	Distal Interger	-0524	419E-03	409E-02	Sin3b	-8149	Hypo
chr5	152742861	152744753	Distal Interger	-0465	419E-03	409E-02	Igsf21	-230687	Hypo
chr6	137544615	137545435	Intron (NM_0	-0572	419E-03	410E-02	Ptprn2	105043	Hypo
chr1	140499377	140503765	Promoter (<=1	-0261	420E-03	410E-02	Folh1	0	Hypo

chr2	134980112	134981955	Distal Interger	-0464	421E-03	410E-02	Noct	-289234	Hypo
chr15	93238101	93238577	Intron (NM_0(	-0723	421E-03	411E-02	Gpc5	998555	Hypo
chr3	167204864	167211809	Promoter (<=:	-0246	421E-03	411E-02	Osbpl2	0	Hypo
chr3	133719527	133723049	Distal Interger	-0358	421E-03	411E-02	Crnkl1	-365198	Hypo
chr1	242397923	242400227	Distal Interger	-0416	422E-03	411E-02	Got1	-17300	Hypo
chr14	10300407	10301837	Distal Interger	-0548	422E-03	411E-02	Prkg2	-268621	Hypo
chr17	71186440	71193963	Distal Interger	-0262	422E-03	411E-02	Celf2	-215647	Hypo
chr5	47568208	47569382	3' UTR	-0514	422E-03	411E-02	Pm20d2	16830	Hypo
chr2	12088619	12090014	Promoter (<=:	-0302	423E-03	412E-02	Cetn3	0	Hypo
chr5	151030758	151031278	Distal Interger	-0736	423E-03	412E-02	Pla2g2d	14388	Hypo
chr4	169047211	169047756	Distal Interger	-0626	423E-03	412E-02	Grin2b	-4932	Hypo
chr19	9624703	9625373	Distal Interger	-0632	423E-03	412E-02	Cfap20	15724	Hypo
chr5	8447351	8447663	Distal Interger	-0813	423E-03	412E-02	Prex2	-260551	Hypo
chr3	12076882	12077656	Intron (NM_0(	-0546	424E-03	412E-02	Ak8	47928	Hypo
chr8	64274455	64277907	Distal Interger	-0333	424E-03	412E-02	Smad3	-37495	Hypo
chr18	7881451	7882282	Intron (NM_0:	-0621	424E-03	412E-02	Cdh2	113506	Hypo
chr17	5188246	5188947	Intron (NM_0(	-0660	424E-03	412E-02	Agtpbp1	54548	Hypo
chr3	65158060	65159264	Distal Interger	-0475	425E-03	413E-02	Dnajc10	-73433	Hypo
chr20	38154168	38155128	Intron (NM_0(	-0509	425E-03	413E-02	Nt5dc1	53460	Hypo
chr5	72175077	72176285	Distal Interger	-0455	425E-03	413E-02	LOC10091062	-74007	Hypo
chr1	255112954	255115041	Distal Interger	-0372	426E-03	413E-02	Habp2	-200874	Hypo
chr1	231236283	231237265	Intron (NM_0(	-0524	426E-03	413E-02	Rps4x-ps1	26006	Hypo
chr1	72481735	72483593	Distal Interger	-0413	426E-03	414E-02	Vom2r78	-554556	Hypo
chr6	100211950	100213810	Intron (NM_0(	-0424	426E-03	414E-02	Galnt16	41582	Hypo
chr16	78539066	78540147	Distal Interger	-0500	427E-03	414E-02	Irs2	50817	Hypo
chr8	52235686	52236225	Distal Interger	-0742	427E-03	414E-02	Fdx1	51119	Hypo
chr1	199403243	199410483	Intron (NM_2(	-0270	427E-03	414E-02	Shank2	-45791	Hypo
chr18	3046645	3047086	Distal Interger	-0749	427E-03	414E-02	Cables1	-30193	Hypo
chr9	65193886	65195751	Distal Interger	-0371	428E-03	415E-02	Fastkd2	24419	Hypo
chr12	14538272	14540428	Intron (NM_0(	-0396	428E-03	415E-02	Elfn1	184152	Hypo
chr13	62616657	62620740	Exon (NM_00:	-0338	428E-03	415E-02	Prg4	-112538	Hypo
chr1	176942583	176944380	Intron (NM_0	-0479	428E-03	415E-02	Prkcb	109898	Hypo
chr13	88767277	88768981	Exon (NM_17	-0435	428E-03	415E-02	Sdccag8	12628	Hypo

chr9	54763302	54764218	Distal Interger	-0503	428E-03	415E-02	Slc39a10	-146784	Hypo
chr5	67961960	67962893	Distal Interger	-0513	428E-03	415E-02	Slc44a1	-100725	Hypo
chr10	63623217	63627437	Distal Interger	-0297	428E-03	415E-02	Ccnq	20258	Hypo
chr1	50948790	50953888	Distal Interger	-0277	428E-03	415E-02	Pabpc6	-91376	Hypo
chr4	168180411	168183541	Distal Interger	-0346	429E-03	415E-02	Pbp2	-23290	Hypo
chr2	134515398	134518451	Promoter (<=1	-0348	429E-03	415E-02	Slc7a11	0	Hypo
chr2	116341907	116342385	Distal Interger	-0662	430E-03	416E-02	Ttc14	-311210	Hypo
chr18	34150878	34159643	Distal Interger	-0307	430E-03	416E-02	Lars1	96248	Hypo
chr3	21505984	21508411	Distal Interger	-0329	430E-03	416E-02	Crb2	-33810	Hypo
chr12	2644077	2644917	3' UTR	-0547	430E-03	416E-02	Timm44	-14798	Hypo
chr19	23658810	23669972	Intron (NM_0	-0260	430E-03	416E-02	Cacna1a	75674	Hypo
chr3	79811706	79812096	Intron (NM_0	-0869	431E-03	416E-02	Accs	8390	Hypo
chr7	115204970	115206364	Distal Interger	-0425	431E-03	416E-02	Sult4a1	33721	Hypo
chr1	230184864	230185360	Intron (NM_1	-0622	431E-03	416E-02	Sgms1	74232	Hypo
chrX	28114899	28118358	Distal Interger	-0321	431E-03	416E-02	Gpm6b	-13311	Hypo
chr1	9098128	9101118	Distal Interger	-0361	432E-03	417E-02	Gje1	-9990	Hypo
chr5	153476830	153477465	Intron (NM_0	-0622	432E-03	417E-02	Fbxo42	25677	Hypo
chr5	16598391	16598980	Distal Interger	-0590	433E-03	418E-02	Lyn	-40532	Hypo
chr9	110529697	110535869	Intron (NM_0	-0268	433E-03	418E-02	Dlgap1	111874	Hypo
chr2	224016492	224020860	3' UTR	-0312	433E-03	418E-02	Manba	106060	Hypo
chr16	69345084	69347256	Exon (NM_05	-0345	433E-03	418E-02	Ikbkb	25530	Hypo
chr1	172678472	172679558	Distal Interger	-0534	433E-03	418E-02	Syt17	82018	Hypo
chr14	4260692	4263455	Intron (NM_0	-0290	433E-03	418E-02	Lrrc8c	51750	Hypo
chr8	101223461	101224090	Intron (NM_0	-0606	434E-03	419E-02	Stag1	44422	Hypo
chr6	120008003	120009171	Promoter (1-2	-0552	435E-03	419E-02	Rps6ka5	-1859	Hypo
chr6	49641917	49644066	Distal Interger	-0366	435E-03	419E-02	Sypl1	76763	Hypo
chr17	44496105	44498417	Intron (NM_0	-0365	435E-03	419E-02	Elmo1	324371	Hypo
chr2	241481083	241482648	Intron (NM_0	-0402	435E-03	419E-02	Ak5	98810	Hypo
chr2	186458514	186458934	Promoter (2-3	-0718	435E-03	419E-02	Wars2	-2728	Hypo
chr7	91032258	91035045	Intron (NM_0	-0281	436E-03	419E-02	Nsmce2	96060	Hypo
chr1	161549965	161551654	Intron (NM_0	-0475	436E-03	419E-02	Ppfibp2	45075	Hypo
chr17	71161842	71165499	Distal Interger	-0348	436E-03	419E-02	Celf2	-244111	Hypo
chr17	42708993	42709347	Distal Interger	-0795	436E-03	420E-02	Hist1h2bo	8699	Hypo

chr7	125896118	125897023	Intron (NM_0(	-0523	436E-03	420E-02	Tmem117	138527	Hypo
chr19	55445104	55449008	Exon (NM_03:	-0308	437E-03	420E-02	Pard3	364822	Hypo
chr11	53095480	53095959	Distal Intergen	-0761	437E-03	420E-02	Morc1	-711225	Hypo
chr3	163127160	163127974	3' UTR	-0578	437E-03	420E-02	Gnas	8047	Hypo
chr8	63382341	63384616	Intron (NM_0(	-0340	438E-03	421E-02	Pias1	54278	Hypo
chr20	8220130	8220520	Intron (NM_0(	-0773	438E-03	421E-02	Zfand3	136723	Hypo
chr2	142958059	142960318	Distal Intergen	-0439	438E-03	421E-02	Siah2	-26307	Hypo
chr8	105914691	105916314	Intron (NM_0(	-0477	438E-03	421E-02	Aste1	-110256	Hypo
chr6	116604621	116605226	Distal Intergen	-0704	438E-03	421E-02	Galc	910585	Hypo
chr1	51754079	51754696	Intron (NM_0(	-0634	439E-03	421E-02	MGC94891	9293	Hypo
chr15	45140442	45140936	Distal Intergen	-0629	439E-03	421E-02	Egr3	-9947	Hypo
chr14	15636062	15637153	Downstream (	-0497	439E-03	421E-02	Nup54	18336	Hypo
chr17	81206124	81207100	Distal Intergen	-0531	439E-03	421E-02	Commd3	-120369	Hypo
chr20	30246444	30248903	Exon (NM_01:	-0394	439E-03	421E-02	Tacr2	37537	Hypo
chr6	21004532	21004961	Intron (NM_0(	-0732	440E-03	422E-02	Nlrc4	9266	Hypo
chrX	42317612	42318749	Distal Intergen	-0386	441E-03	422E-02	Sts	92240	Hypo
chr20	22324154	22325053	Distal Intergen	-0524	441E-03	423E-02	Reep3	830286	Hypo
chr2	83671680	83672977	Intron (NM_0(	-0460	441E-03	423E-02	Sema5a	361837	Hypo
chr11	79662441	79663836	Distal Intergen	-0399	441E-03	423E-02	Ephb3	195534	Hypo
chr5	20165674	20171670	Promoter (<=:	-0245	441E-03	423E-02	Tox	0	Hypo
chr1	211796296	211798451	Promoter (<=:	-0276	442E-03	423E-02	Tle4	0	Hypo
chr13	102494670	102496074	Distal Intergen	-0435	442E-03	423E-02	Rps6kc1	-4372	Hypo
chr6	131396927	131399697	Distal Intergen	-0385	443E-03	424E-02	Tmem179	180608	Hypo
chr8	30909334	30910483	Intron (NM_0(	-0523	443E-03	424E-02	Fli1	39950	Hypo
chr4	156089914	156092388	Distal Intergen	-0427	444E-03	424E-02	C3ar1	-5314	Hypo
chr3	68908879	68912001	Distal Intergen	-0319	444E-03	424E-02	Zswim2	103410	Hypo
chr4	9991438	9995358	Distal Intergen	-0300	444E-03	424E-02	Galntl5	-68302	Hypo
chr3	46915386	46915724	Intron (NM_1	-0723	444E-03	424E-02	Dpp4	128146	Hypo
chr15	82326970	82328435	Distal Intergen	-0471	444E-03	424E-02	Ndfip2	294604	Hypo
chr18	67763152	67767390	Distal Intergen	-0262	445E-03	425E-02	Ska1	37647	Hypo
chr1	256679298	256680908	Distal Intergen	-0437	445E-03	425E-02	Trub1	163736	Hypo
chr17	12905177	12905658	Distal Intergen	-0600	445E-03	425E-02	Diras2	147612	Hypo
chr11	79989097	79989532	Distal Intergen	-0755	445E-03	425E-02	Ephb3	-129727	Hypo

chr12	886786	887800	Distal Interger	-0547	445E-03	425E-02	Rfc3	-113215	Hypo
chr3	27566139	27567070	Distal Interger	-0514	445E-03	425E-02	Kynu	-211685	Hypo
chr6	29158633	29160889	Distal Interger	-0378	446E-03	426E-02	Klhl29	-767986	Hypo
chr1	188558736	188561155	5' UTR	-0410	446E-03	426E-02	Dhx32	16345	Hypo
chr1	174527877	174529800	Promoter (2-3	-0386	446E-03	426E-02	Zp2	-2589	Hypo
chr13	36250465	36252491	Distal Interger	-0418	449E-03	427E-02	Actr3	591346	Hypo
chr4	178861000	178862012	Distal Interger	-0552	449E-03	427E-02	Sspn	-35135	Hypo
chr4	105933631	105934275	Distal Interger	-0651	449E-03	427E-02	Jkampl	-412089	Hypo
chr8	123545432	123545938	Distal Interger	-0634	449E-03	428E-02	Ccr1	15903	Hypo
chr5	71678467	71680552	Distal Interger	-0410	449E-03	428E-02	Mir32	-82315	Hypo
chr6	10801622	10806818	Distal Interger	-0281	449E-03	428E-02	Haao	-38967	Hypo
chr15	76710171	76712961	Intron (NM_0(	-0329	449E-03	428E-02	Klf12	274779	Hypo
chr1	221637030	221640588	Distal Interger	-0312	449E-03	428E-02	Tjp2	197707	Hypo
chr9	23292087	23295980	Distal Interger	-0318	449E-03	428E-02	Paqr8	-45626	Hypo
chr6	49128210	49129183	Distal Interger	-0532	450E-03	428E-02	Nampt	-296133	Hypo
chr20	44189214	44193950	Intron (NM_0(	-0302	450E-03	428E-02	Mettl24	46818	Hypo
chr15	78484920	78486030	Promoter (<=1	-0328	451E-03	429E-02	Uchl3	0	Hypo
chr4	179245575	179248804	Intron (NM_01	-0319	451E-03	429E-02	Itpr2	155360	Hypo
chr15	52651078	52653550	Distal Interger	-0360	451E-03	429E-02	Dnajc15	596125	Hypo
chr7	8459964	8467762	Promoter (<=1	-0243	451E-03	429E-02	Matk	0	Hypo
chr11	2994565	2994929	Distal Interger	-0874	451E-03	429E-02	Pou1f1	-322095	Hypo
chr3	17493741	17494207	Exon (NM_001	-0681	452E-03	429E-02	Pbx3	188143	Hypo
chr5	139965216	139966989	Distal Interger	-0486	452E-03	429E-02	Gjb5	-283408	Hypo
chr15	6421652	6422955	Distal Interger	-0421	452E-03	429E-02	Ube2e2	-563309	Hypo
chr9	64709143	64710618	Distal Interger	-0442	452E-03	429E-02	Cmklr2	-122488	Hypo
chr6	57724287	57725803	Distal Interger	-0437	452E-03	429E-02	Ifrd1	-435381	Hypo
chr1	105627102	105627937	Distal Interger	-0539	452E-03	429E-02	Tubgcp5	-1008589	Hypo
chr15	98090443	98095507	Distal Interger	-0294	453E-03	430E-02	Farp1	-86822	Hypo
chr4	173198447	173199211	Distal Interger	-0463	453E-03	430E-02	Plekha5	-134844	Hypo
chr20	40787914	40788598	Distal Interger	-0591	454E-03	430E-02	Marcks	-96902	Hypo
chr1	7885211	7886995	Promoter (<=1	-0284	454E-03	430E-02	Fuca2	0	Hypo
chr6	68455504	68456514	Distal Interger	-0506	454E-03	430E-02	G2e3	-307671	Hypo
chr17	58487861	58491144	Intron (NM_0(	-0318	454E-03	431E-02	Ryr2	97569	Hypo

chr14	23651671	23656504	Promoter (<=1	-0243	455E-03	431E-02	Epha5	0	Hypo
chr1	155073212	155074995	Intron (NM_0(	-0377	455E-03	431E-02	Fam168a	15860	Hypo
chr6	10595680	10601878	Distal Interger	-0270	455E-03	431E-02	Zfp36l2	105648	Hypo
chr13	102996582	102999435	Distal Interger	-0325	455E-03	431E-02	Ppp2r5a	-20921	Hypo
chr4	162632029	162632825	Distal Interger	-0536	455E-03	431E-02	Clec2g	14117	Hypo
chr16	22987412	22993207	Distal Interger	-0285	456E-03	432E-02	Npy1r	53552	Hypo
chr4	69900397	69902153	Distal Interger	-0390	457E-03	432E-02	Prss3b	-24879	Hypo
chr3	126785669	126786982	Distal Interger	-0410	457E-03	432E-02	Sptlc3	-60896	Hypo
chr10	105068131	105072897	Intron (NM_0(	-0278	457E-03	432E-02	Chmp6	-119075	Hypo
chr3	146084485	146085388	Distal Interger	-0557	457E-03	433E-02	Src	-39327	Hypo
chr8	71573276	71575066	Intron (NM_0	-0394	457E-03	433E-02	Lipc	60398	Hypo
chr13	89447782	89451303	Distal Interger	-0292	457E-03	433E-02	Zbtb18	3469	Hypo
chr4	145872341	145875007	Exon (NM_001	-0352	458E-03	433E-02	Rad18	-51276	Hypo
chr13	81532222	81532703	Distal Interger	-0733	458E-03	433E-02	Nuf2	190062	Hypo
chr10	82602456	82610284	Distal Interger	-0245	458E-03	433E-02	Epop	15628	Hypo
chr9	14954760	14961702	Promoter (<=1	-0254	458E-03	433E-02	Vegfa	0	Hypo
chr4	30271171	30271756	Distal Interger	-0686	458E-03	433E-02	Mterf1	44767	Hypo
chr7	126525844	126526261	Intron (NM_01	-0764	458E-03	433E-02	Nell2	136433	Hypo
chr4	119246954	119250735	Distal Interger	-0332	459E-03	433E-02	Anxa4	-5801	Hypo
chr3	57078741	57081560	Intron (NM_0(	-0331	459E-03	433E-02	Cdca7	-241158	Hypo
chr6	69832447	69834620	Distal Interger	-0360	459E-03	433E-02	Arhgap5	-153590	Hypo
chr11	13388983	13389218	Distal Interger	-0858	459E-03	434E-02	Robo2	-349990	Hypo
chr4	150857295	150857541	Distal Interger	-0895	460E-03	434E-02	Zfp637	-147784	Hypo
chr1	112327705	112328492	Distal Interger	-0622	460E-03	434E-02	Snurf	-1204071	Hypo
chr9	79940995	79943625	Intron (NM_0(	-0379	460E-03	434E-02	Farsb	3412	Hypo
chr18	124972	126983	Distal Interger	-0336	460E-03	434E-02	Fundc2	10920	Hypo
chr2	150038851	150039625	Intron (NM_0(	-0570	461E-03	434E-02	Lekr1	147745	Hypo
chr17	18218435	18218814	Distal Interger	-0758	461E-03	434E-02	Cap2	-15090	Hypo
chr16	10136759	10140641	Promoter (<=1	-0282	461E-03	435E-02	Grid1	0	Hypo
chr6	17200053	17200738	Distal Interger	-0597	462E-03	435E-02	Crim1	-329794	Hypo
chr1	159759903	159760791	Distal Interger	-0616	462E-03	435E-02	Cnga4	7546	Hypo
chr13	91753659	91758471	Intron (NM_05	-0287	462E-03	435E-02	Cdc42bpa	69652	Hypo
chr1	98892040	98894128	Distal Interger	-0398	462E-03	435E-02	Nav2	-64270	Hypo

chr6	44006338	44007615	Promoter (2-3	-0437	462E-03	435E-02	Sox11	2739	Hypo
chr15	68801665	68803177	Distal Interger	-0381	462E-03	435E-02	Pcdh9	1432488	Hypo
chr10	98374491	98376459	Intron (NM_0(	-0406	462E-03	436E-02	Mir297	-157947	Hypo
chr11	81486414	81488043	Promoter (<=1	-0387	463E-03	436E-02	Or2m12	0	Hypo
chr4	28486853	28487328	Intron (NM_0(	-0615	463E-03	436E-02	Gtpbp10	9996	Hypo
chr3	131218202	131219324	Intron (NM_0:	-0488	463E-03	436E-02	Bfsp1	10013	Hypo
chr7	134734909	134735543	Distal Interger	-0661	463E-03	436E-02	Glycam1	-52061	Hypo
chr6	70067244	70070952	Distal Interger	-0238	464E-03	436E-02	Arhgap5	79034	Hypo
chr8	45607758	45609134	Distal Interger	-0321	464E-03	436E-02	Tmprss13	-16625	Hypo
chr20	10535120	10536925	Distal Interger	-0436	464E-03	436E-02	Gatd3a	20327	Hypo
chr12	28578040	28578940	Intron (NM_1!	-0574	464E-03	437E-02	Tmem132d	307328	Hypo
chr16	80190009	80191380	Distal Interger	-0406	465E-03	437E-02	Arglu1	-561935	Hypo
chr14	4430606	4434569	Distal Interger	-0320	465E-03	437E-02	Lrrc8b	-69526	Hypo
chr6	124843407	124845846	Distal Interger	-0369	466E-03	438E-02	Vrk1	-69055	Hypo
chr12	26838794	26840937	Promoter (<=1	-0361	466E-03	438E-02	Phkg1	0	Hypo
chr4	116057267	116059232	Intron (NM_1:	-0404	466E-03	438E-02	Actg2	-20909	Hypo
chr3	126050167	126052755	Distal Interger	-0319	466E-03	438E-02	Btbd3	437474	Hypo
chr8	49967396	49969144	Intron (NM_0:	-0418	466E-03	438E-02	Ttc12	-121711	Hypo
chr10	45099321	45107529	Intron (NM_0(	-0236	466E-03	438E-02	Tom1l2	50503	Hypo
chr3	142189708	142190783	Distal Interger	-0550	467E-03	438E-02	Mapre1	-28106	Hypo
chr3	123653483	123658381	Intron (NM_0(	-0310	467E-03	438E-02	Pak5	45549	Hypo
chr3	145018533	145021140	Exon (NM_17:	-0435	467E-03	438E-02	Epb41l1	33887	Hypo
chr8	122523383	122524699	Distal Interger	-0382	468E-03	438E-02	Zkscan7	21165	Hypo
chr4	63289818	63291549	Distal Interger	-0433	468E-03	438E-02	Cald1	-85014	Hypo
chr11	20125422	20128218	Intron (NM_2(	-0384	468E-03	439E-02	Ncam2	20376	Hypo
chr5	165066779	165067708	Distal Interger	-0522	468E-03	439E-02	Actrt2	169784	Hypo
chr14	31994152	31995163	3' UTR	-0502	468E-03	439E-02	Tmem165	23554	Hypo
chr10	74903185	74904119	Distal Interger	-0535	468E-03	439E-02	Tmem100	48406	Hypo
chr4	107678372	107678822	Distal Interger	-0810	469E-03	439E-02	LRRTM1	-2022993	Hypo
chr17	22820559	22821312	Distal Interger	-0569	469E-03	439E-02	Hivep1	-109199	Hypo
chr2	42560200	42560909	Distal Interger	-0538	469E-03	440E-02	Actbl2	-297111	Hypo
chr9	31356798	31357805	Distal Interger	-0416	470E-03	440E-02	Phf3	1778189	Hypo
chr10	53792217	53799918	Promoter (<=1	-0227	470E-03	440E-02	Vamp2	0	Hypo

chr2	112622320	112623049	Distal Interger	-0601	470E-03	440E-02	Sec62	-14744	Hypo
chr2	62390560	62391535	Distal Interger	-0548	470E-03	440E-02	Cdh6	-165332	Hypo
chr3	78763327	78770313	Distal Interger	-0288	470E-03	440E-02	Syt13	-106887	Hypo
chr2	104701395	104703627	Distal Interger	-0417	471E-03	440E-02	Tbl1xr1	-98094	Hypo
chr12	31410018	31411461	Distal Interger	-0338	471E-03	441E-02	Ncor2	-55127	Hypo
chr17	83508497	83510918	Distal Interger	-0377	472E-03	441E-02	Arhgap21	-36295	Hypo
chr10	78517869	78521880	Distal Interger	-0297	472E-03	441E-02	Utp18	316974	Hypo
chr4	12273817	12275486	Distal Interger	-0449	472E-03	441E-02	Orc5	-272571	Hypo
chr1	241096630	241097399	Intron (NM_1:	-0605	472E-03	441E-02	Mir3085	-7637	Hypo
chr8	11755429	11756468	Distal Interger	-0497	472E-03	441E-02	Gpr83	61889	Hypo
chr7	29816645	29818648	Distal Interger	-0389	472E-03	441E-02	Cradd	134259	Hypo
chr1	224714720	224715348	Distal Interger	-0628	474E-03	442E-02	Vldlr	-99108	Hypo
chr6	104387654	104388343	Distal Interger	-0633	474E-03	442E-02	Npc2	29818	Hypo
chr17	61140855	61142603	3' UTR	-0432	474E-03	442E-02	Larp4b	74753	Hypo
chr20	44178809	44181930	Exon (NM_00:	-0285	474E-03	442E-02	Mettl24	36413	Hypo
chr2	172272824	172274486	Intron (NM_0(	-0432	474E-03	442E-02	Dclk2	63764	Hypo
chr11	57388252	57390479	Intron (NM_0(	-0333	475E-03	443E-02	Mir568	-334179	Hypo
chr9	1325013	1325733	Distal Interger	-0584	477E-03	445E-02	Ptprs	-18068	Hypo
chr16	1149904	1155143	Intron (NM_0(	-0283	478E-03	445E-02	Mir3075	48841	Hypo
chr15	20977840	20978599	Distal Interger	-0543	478E-03	445E-02	Atg14	-150727	Hypo
chr3	17560843	17562084	Intron (NM_0(	-0380	478E-03	445E-02	Pbx3	120266	Hypo
chr6	75086024	75086292	Distal Interger	-0757	478E-03	445E-02	Foxa1	49896	Hypo
chr5	25499389	25499959	Distal Interger	-0589	478E-03	445E-02	Pdp1	-44342	Hypo
chr4	129945579	129950339	Distal Interger	-0293	478E-03	445E-02	Arl6ip5	130104	Hypo
chrX	48844923	48845455	Intron (NM_0(	-0743	479E-03	446E-02	Dmd	-499907	Hypo
chr2	165516091	165519318	Distal Interger	-0305	480E-03	447E-02	Mir2985	437180	Hypo
chr5	138396486	138397014	Intron (NM_1:	-0565	481E-03	447E-02	Stk40	15327	Hypo
chr2	195921842	195922283	Promoter (2-3	-0641	481E-03	447E-02	Sort1	-2079	Hypo
chr2	57751004	57751456	Distal Interger	-0700	481E-03	448E-02	Slc1a3	79128	Hypo
chr6	76930960	76933586	Promoter (<=:	-0269	481E-03	448E-02	Fbxo33	0	Hypo
chr5	24005929	24006739	Intron (NM_0(	-0568	481E-03	448E-02	Cfap418	9211	Hypo
chr6	124595897	124600729	Intron (NM_0(	-0323	482E-03	448E-02	Gskip	3359	Hypo
chr16	19666159	19666635	Distal Interger	-0653	482E-03	448E-02	Atp13a1	-34945	Hypo

chr19	11184430	11193981	Promoter (<=1	-0234	482E-03	448E-02	Gnao1	0	Hypo
chr11	30755241	30755877	Distal Interger	-0480	482E-03	448E-02	Ifngr2	-23856	Hypo
chr5	19963089	19963670	Intron (NM_0(	-0629	483E-03	449E-02	Tox	207602	Hypo
chr19	13970496	13970844	Exon (NM_001	-0699	483E-03	449E-02	Ces1a	10820	Hypo
chr7	18564320	18565040	Intron (NM_1!	-0562	483E-03	449E-02	Mterf2	-33162	Hypo
chr13	48752959	48753717	Distal Interger	-0539	484E-03	449E-02	Nr5a2	-319633	Hypo
chr10	47147673	47149174	Intron (NM_1:	-0464	484E-03	450E-02	Pigl	5513	Hypo
chr11	25654397	25655053	Distal Interger	-0585	484E-03	450E-02	Adamts5	-607595	Hypo
chr11	72260397	72261909	Distal Interger	-0430	484E-03	450E-02	Fgf12	-37038	Hypo
chr2	141071035	141075518	Distal Interger	-0273	485E-03	450E-02	Tm4sf1	390628	Hypo
chr17	54238126	54240422	Promoter (<=1	-0257	485E-03	450E-02	Crem	0	Hypo
chr13	71655511	71655827	Distal Interger	-0823	485E-03	450E-02	Mir1843b	103963	Hypo
chr9	58777330	58777639	Distal Interger	-0746	485E-03	450E-02	RGD1306941	-172206	Hypo
chr18	74135063	74136180	Intron (NM_0(	-0556	486E-03	450E-02	Nfatc1	19848	Hypo
chr17	1929406	1933287	Intron (NM_0(	-0292	486E-03	450E-02	Mir23b	-115643	Hypo
chr2	104453421	104454018	Distal Interger	-0603	486E-03	451E-02	Tbl1xr1	-347703	Hypo
chr19	17560938	17563066	Distal Interger	-0416	487E-03	451E-02	Sall1	-444437	Hypo
chr14	43364403	43365383	Promoter (2-3	-0522	487E-03	451E-02	Tlr6	2239	Hypo
chr2	41637386	41639440	Intron (NM_1:	-0423	487E-03	451E-02	Rab3c	203133	Hypo
chr16	45166559	45168450	Distal Interger	-0398	488E-03	452E-02	Enpp6	125627	Hypo
chr5	123673164	123673932	Intron (NM_0:	-0610	490E-03	453E-02	Rab3b	42762	Hypo
chr8	42468392	42470661	Exon (NM_05:	-0413	490E-03	453E-02	Sorl1	33567	Hypo
chr11	29342997	29345197	Distal Interger	-0432	490E-03	453E-02	Sod1	-111476	Hypo
chr1	87214423	87215837	Distal Interger	-0396	490E-03	453E-02	Kctd15	56142	Hypo
chr12	20862994	20865082	Intron (NM_0(	-0417	490E-03	453E-02	Srrm3	13423	Hypo
chr13	72572336	72573554	Intron (NM_0(	-0480	490E-03	453E-02	Cacybp	-124526	Hypo
chr11	31374740	31376822	Distal Interger	-0375	491E-03	453E-02	Slc5a3	60893	Hypo
chr2	129635259	129638444	Distal Interger	-0317	491E-03	454E-02	Pabpc4l	171301	Hypo
chr19	39318578	39323557	Exon (NM_001	-0295	491E-03	454E-02	Fa2h	40596	Hypo
chr17	10732830	10733819	Distal Interger	-0464	491E-03	454E-02	Drd1	191284	Hypo
chr12	20317938	20319662	Intron (NM_0!	-0434	491E-03	454E-02	Sh2b2	-109381	Hypo
chr17	70460863	70461675	Distal Interger	-0497	491E-03	454E-02	Celf2	-947935	Hypo
chr3	76848014	76848823	Exon (NM_001	-0548	492E-03	454E-02	Mtch2	17465	Hypo

chr6	52838957	52842432	Exon (NM_134	-0397	492E-03	454E-02	Bzw2	46160	Hypo
chr16	46644462	46644904	Distal Intergen	-0581	492E-03	454E-02	Sorbs2	-18014	Hypo
chr7	62659974	62660817	Distal Intergen	-0585	493E-03	454E-02	Atp23	69006	Hypo
chr8	43191451	43193583	Intron (NM_0	-0342	493E-03	455E-02	Grik4	11494	Hypo
chr10	52945804	52946661	Intron (NM_05	-0433	493E-03	455E-02	Ntn1	137658	Hypo
chr4	179869653	179875742	Promoter (<=1	-0315	493E-03	455E-02	C4h12orf71	0	Hypo
chr1	97628030	97628796	Intron (NM_0	-0657	493E-03	455E-02	Ptpn5	5958	Hypo
chr3	116471845	116474439	Distal Intergen	-0387	494E-03	455E-02	Slc20a1	43886	Hypo
chr17	28542975	28543452	Intron (NM_00	-0753	494E-03	455E-02	Fars2	202644	Hypo
chr3	90877214	90879043	Distal Intergen	-0378	494E-03	455E-02	Hipk3	-10902	Hypo
chr4	29590945	29592638	Distal Intergen	-0442	494E-03	455E-02	Fzd1	280642	Hypo
chr3	124000811	124007413	Distal Intergen	-0239	495E-03	456E-02	Snap25	-34485	Hypo
chr8	36691862	36693448	Intron (NM_00	-0307	495E-03	456E-02	Pknox2	6916	Hypo
chr10	64305094	64307512	Promoter (<=1	-0260	495E-03	456E-02	Nf1	0	Hypo
chr8	52859843	52860243	Distal Intergen	-0599	495E-03	456E-02	LOC10012536	208769	Hypo
chr8	114450378	114450995	Intron (NM_00	-0568	496E-03	456E-02	Cmtm7	23963	Hypo
chr1	177891216	177891821	Intron (NM_00	-0597	496E-03	456E-02	Arhgap17	5032	Hypo
chr11	68083656	68084872	Distal Intergen	-0576	496E-03	456E-02	Tnk2	69137	Hypo
chr4	147262376	147263980	Distal Intergen	-0398	496E-03	457E-02	Slc6a11	-33992	Hypo
chr10	100615864	100619327	Distal Intergen	-0280	497E-03	457E-02	Hid1	-5823	Hypo
chr7	91698832	91699131	Distal Intergen	-0891	497E-03	457E-02	LOC500876	-80802	Hypo
chr11	48745128	48746364	Intron (NM_11	-0471	497E-03	457E-02	Cblb	10475	Hypo
chr9	78857113	78858820	Intron (NM_00	-0367	497E-03	457E-02	Epha4	99319	Hypo
chr4	24834305	24835569	Distal Intergen	-0493	497E-03	457E-02	Elapor2	-28576	Hypo
chr9	82190453	82192672	Distal Intergen	-0365	497E-03	457E-02	Cul3	-538940	Hypo
chr8	3250042	3251025	Distal Intergen	-0552	497E-03	457E-02	Pdgfd	-237697	Hypo
chr15	706754	712111	Exon (NM_031	-0310	497E-03	457E-02	Kcnma1	404677	Hypo
chr16	68228069	68230640	Intron (NM_00	-0365	498E-03	457E-02	Zmat4	130562	Hypo
chr19	10202892	10203231	Promoter (<=1	-0832	498E-03	457E-02	Ccl17	537	Hypo
chr1	90322549	90324541	Distal Intergen	-0385	498E-03	458E-02	Uri1	380115	Hypo
chr1	134107514	134109765	Distal Intergen	-0363	499E-03	458E-02	Sema4b	-39771	Hypo
chr3	114726782	114730494	Intron (NM_01	-0264	499E-03	458E-02	Kcnip3	11626	Hypo
chr1	212143467	212145969	Distal Intergen	-0365	500E-03	459E-02	Tle4	-345605	Hypo

chr13	94165875	94167293	Exon (NM_01	-0435	500E-03	459E-02	Capn2	33676	Hypo
chr11	58832451	58833313	Intron (NM_0	-0562	501E-03	459E-02	Lsamp	362955	Hypo
chr2	30416610	30418776	Distal Interger	-0390	501E-03	459E-02	Tnpo1	-147225	Hypo
chr8	84506924	84509743	Intron (NM_0	-0303	502E-03	460E-02	Sh3bgrl2	45803	Hypo
chr2	233713217	233714538	Distal Interger	-0606	502E-03	460E-02	Sh3glb1	70246	Hypo
chr1	89420717	89425074	Intron (NM_0	-0315	502E-03	460E-02	Tshz3	31940	Hypo
chr16	81476796	81477043	Distal Interger	-0800	502E-03	460E-02	Efnb2	693379	Hypo
chr17	5631709	5634965	Intron (NM_0	-0294	503E-03	460E-02	Ntrk2	234156	Hypo
chr16	78032471	78033537	Intron (NM_0	-0508	503E-03	460E-02	Rab20	13134	Hypo
chr1	165840394	165841586	Intron (NM_0	-0483	503E-03	460E-02	Galnt18	62682	Hypo
chr2	232155716	232157814	Distal Interger	-0390	503E-03	461E-02	Pkn2	-256763	Hypo
chr14	81818571	81821539	Distal Interger	-0302	503E-03	461E-02	Adcy1	-89701	Hypo
chr7	52547790	52548677	Intron (NM_0	-0536	504E-03	461E-02	Rab3ip	12366	Hypo
chr9	23420534	23421175	Distal Interger	-0569	504E-03	461E-02	Efhc1	44694	Hypo
chr16	45598782	45600232	Distal Interger	-0270	504E-03	461E-02	Irf2	-48823	Hypo
chr14	2316320	2318983	Distal Interger	-0319	504E-03	461E-02	Lpcat2b	22989	Hypo
chr13	42658566	42661151	Intron (NM_0	-0321	504E-03	461E-02	Rassf5	41873	Hypo
chr13	94124881	94125903	Distal Interger	-0459	504E-03	461E-02	Capn2	75066	Hypo
chr1	78880072	78883183	Promoter (<=1	-0273	504E-03	461E-02	Opa3	0	Hypo
chrX	40157589	40163366	Promoter (<=1	-0252	504E-03	461E-02	Sat1	0	Hypo
chr3	11640047	11642200	Distal Interger	-0433	505E-03	461E-02	Olfm1	106739	Hypo
chr16	32439508	32440004	Intron (NM_0	-0688	505E-03	461E-02	Galnt7	-138686	Hypo
chr20	25481027	25484415	Intron (NM_0	-0322	505E-03	461E-02	Mypn	44184	Hypo
chr8	65314774	65315557	Distal Interger	-0590	505E-03	461E-02	Rab11a	-68313	Hypo
chr4	162087781	162088362	Distal Interger	-0652	506E-03	462E-02	Clec2d2	-18274	Hypo
chr6	10308998	10310932	Intron (NM_0	-0495	506E-03	462E-02	Thada	122999	Hypo
chr17	42757048	42757543	Distal Interger	-0576	507E-03	462E-02	H1f5	30859	Hypo
chr16	66985010	66989029	Distal Interger	-0257	507E-03	463E-02	Htra4	-12576	Hypo
chr9	27123976	27129499	Exon (NM_13	-0336	507E-03	463E-02	Lmbrd1	27589	Hypo
chrX	27951865	27953787	Distal Interger	-0291	507E-03	463E-02	Tceanc	-3872	Hypo
chr6	103558882	103564474	Distal Interger	-0262	508E-03	463E-02	Riox1	-15168	Hypo
chr7	115130355	115133771	Distal Interger	-0332	509E-03	464E-02	Sult4a1	106314	Hypo
chr9	99521586	99522323	Distal Interger	-0545	509E-03	464E-02	Nudt12	-766547	Hypo

chr15	35837170	35837545	Distal Interger	-0717	510E-03	465E-02	Kcnrg	62678	Hypo
chr8	70979124	70982044	Intron (NM_1	-0375	510E-03	465E-02	Myo1e	91190	Hypo
chr2	188449773	188451427	Exon (NM_00	-0405	510E-03	465E-02	Cd101	8075	Hypo
chr10	73267788	73268725	Distal Interger	-0549	510E-03	465E-02	Ccdc182	177560	Hypo
chr1	214638630	214641109	Distal Interger	-0355	511E-03	465E-02	Gcnt1	82239	Hypo
chr17	60704623	60705430	Intron (NM_0	-0539	511E-03	465E-02	Zmynd11	126261	Hypo
chr2	119242104	119242587	Distal Interger	-0559	511E-03	465E-02	Anxa5	101984	Hypo
chr15	42217009	42218773	Distal Interger	-0376	512E-03	466E-02	Dock5	-58360	Hypo
chr19	20916137	20918470	Distal Interger	-0360	512E-03	466E-02	Phkb	292135	Hypo
chr13	103510660	103511297	Distal Interger	-0612	512E-03	466E-02	Slc30a1	19623	Hypo
chr16	60987572	60990738	Promoter (<=1	-0265	512E-03	466E-02	Tti2	0	Hypo
chr7	131699179	131702311	Distal Interger	-0274	512E-03	466E-02	Pou6f1	-12336	Hypo
chr1	246090050	246091267	Intron (NM_0	-0421	513E-03	466E-02	Neurl1	22950	Hypo
chr8	69889098	69890089	Distal Interger	-0580	513E-03	466E-02	Ice2	-154481	Hypo
chr1	129771539	129772067	Distal Interger	-0665	513E-03	467E-02	Klhl25	46589	Hypo
chr4	31039458	31040815	Distal Interger	-0473	514E-03	467E-02	Cdk6	-212678	Hypo
chr16	30431770	30432019	Distal Interger	-0960	514E-03	467E-02	Galntl6	-762259	Hypo
chr19	29902548	29902907	Distal Interger	-0710	514E-03	467E-02	Ttc29	-151933	Hypo
chr3	42630353	42635176	Promoter (<=1	-0324	514E-03	467E-02	Ermn	0	Hypo
chr2	216438058	216440544	Distal Interger	-0370	514E-03	467E-02	Fam241a	-62865	Hypo
chr2	61604327	61604713	Intron (NM_0	-0668	514E-03	467E-02	Pdzd2	16433	Hypo
chr2	91893449	91894539	Distal Interger	-0432	515E-03	467E-02	Fabp5	-124736	Hypo
chr1	135426050	135426996	Intron (NM_0	-0494	515E-03	467E-02	Ap3b2	18195	Hypo
chr8	80977325	80978261	Distal Interger	-0467	515E-03	467E-02	Senp6	-10791	Hypo
chr11	85072803	85076171	Exon (NM_00	-0288	516E-03	468E-02	Mzt2b	48527	Hypo
chr14	33936270	33938185	Intron (NM_0	-0357	516E-03	468E-02	Rasl11b	80961	Hypo
chr11	55429642	55430522	Intron (NM_0	-0560	517E-03	468E-02	Cd200	19364	Hypo
chr18	42345805	42346162	Distal Interger	-0828	517E-03	469E-02	Dtwd2	555702	Hypo
chr3	134254048	134258810	Distal Interger	-0316	517E-03	469E-02	Xrn2	-178335	Hypo
chr5	137531965	137532798	Distal Interger	-0588	518E-03	469E-02	Zc3h12a	-146614	Hypo
chr1	143565733	143566162	Intron (NM_0	-0691	518E-03	469E-02	Me3	31594	Hypo
chr1	145235030	145237396	Intron (NM_0	-0384	519E-03	470E-02	Dlg2	107855	Hypo
chrX	36060512	36061486	Distal Interger	-0488	519E-03	470E-02	Rps6ka3	-437442	Hypo

chr1	214923835	214925194	Distal Intergeı	-0492	519E-03	470E-02	Rfk	123955	Hypo
chr6	137617939	137622590	Intron (NM_0:	-0328	520E-03	470E-02	Ptprn2	178367	Hypo
chr10	61648303	61650502	Exon (NM_01:	-0401	520E-03	470E-02	Cpd	36989	Hypo
chr3	58542447	58554168	Promoter (<=:	-0224	520E-03	470E-02	Chn1	0	Hypo
chr6	61489801	61493843	Intron (NM_0	-0325	520E-03	470E-02	Nrcam	86962	Hypo
chr2	27231681	27234601	3' UTR	-0312	521E-03	471E-02	Ndufs6-ps1	29968	Hypo
chr7	8705763	8706018	Distal Intergeı	-0855	521E-03	471E-02	Gng7	-8675	Hypo
chr15	39521329	39522621	Intron (NM_0(	-0514	521E-03	471E-02	Fbxo16	28837	Hypo
chr1	164932783	164940540	3' UTR	-0281	522E-03	472E-02	rnf141	16541	Hypo
chr18	17062025	17067153	Promoter (<=:	-0284	522E-03	472E-02	Celf4	0	Hypo
chr20	27212449	27212981	Distal Intergeı	-0557	523E-03	472E-02	Sowahc	17251	Hypo
chr7	62739874	62740346	Distal Intergeı	-0728	523E-03	472E-02	Atp23	-10051	Hypo
chr7	125902726	125905960	Intron (NM_0(	-0317	524E-03	472E-02	Tmem117	145135	Hypo
chr9	5249845	5250950	Distal Intergeı	-0559	524E-03	473E-02	Kcnh8	-255094	Hypo
chr7	74684198	74685335	Distal Intergeı	-0436	524E-03	473E-02	Emc2	97002	Hypo
chr17	70008141	70011112	Distal Intergeı	-0338	524E-03	473E-02	Gata3	1363228	Hypo
chr20	43686598	43687687	Distal Intergeı	-0402	524E-03	473E-02	Gtf3c6	-18216	Hypo
chr13	105135405	105136524	Distal Intergeı	-0501	525E-03	473E-02	Mir205	-99420	Hypo
chr13	44115259	44118650	Intron (NM_0(	-0333	525E-03	473E-02	Nfasc	30386	Hypo
chr10	92742731	92747123	Promoter (<=:	-0316	525E-03	473E-02	Cacng4	0	Hypo
chr5	39804194	39804962	Distal Intergeı	-0460	526E-03	473E-02	Fut9	-239519	Hypo
chr6	108076174	108076876	Intron (NM_0:	-0580	526E-03	474E-02	Nrxn3	434190	Hypo
chr14	23759370	23761669	Intron (NM_0:	-0350	526E-03	474E-02	Epha5	105977	Hypo
chr14	2407174	2407516	Distal Intergeı	-0754	526E-03	474E-02	Ephx4	-34559	Hypo
chr16	9669733	9670249	Exon (NM_01:	-0604	527E-03	474E-02	Fam25a	14225	Hypo
chr1	220349007	220351843	Intron (NM_0(	-0384	527E-03	474E-02	Mir204	32076	Hypo
chr16	54004766	54006271	Intron (NM_0(	-0395	527E-03	474E-02	Mir383	-34069	Hypo
chr2	23903669	23913446	Intron (NM_1:	-0289	527E-03	474E-02	Serinc5	56751	Hypo
chr6	138150385	138151025	Intron (NM_0:	-0586	527E-03	474E-02	Mir153	-12570	Hypo
chr5	164791849	164792220	Intron (NM_0:	-0782	528E-03	475E-02	Megf6	53497	Hypo
chr9	106250343	106253624	Distal Intergeı	-0341	528E-03	475E-02	Rab12	253175	Hypo
chr6	104387079	104387509	Distal Intergeı	-0635	528E-03	475E-02	Npc2	30652	Hypo
chr13	87149432	87151285	Intron (NM_0	-0385	528E-03	475E-02	Rgs7	257338	Hypo

chr6	118078134	118079324	Exon (NM_00:	-0473	528E-03	475E-02	Zc3h14	71661	Hypo
chr8	113560145	113568966	Distal Interger	-0250	529E-03	475E-02	Pdcd6ip	77807	Hypo
chr11	44760029	44764986	Exon (NM_00:	-0292	529E-03	475E-02	Nfkbiz	-17690	Hypo
chr6	98497476	98500070	Distal Interger	-0386	529E-03	475E-02	Zfyve26	-401996	Hypo
chr10	84099974	84103128	Exon (NM_19	-0310	529E-03	475E-02	Ccr7	5181	Hypo
chr2	19959432	19964822	Distal Interger	-0321	529E-03	475E-02	Hapln1	-666818	Hypo
chr18	51073508	51074547	Distal Interger	-0407	530E-03	476E-02	LOC10091018	-124849	Hypo
chr10	73337271	73339845	Distal Interger	-0337	530E-03	476E-02	Ccdc182	247043	Hypo
chr19	43620580	43621100	Distal Interger	-0672	530E-03	476E-02	Maf	91268	Hypo
chr7	131470919	131473822	Distal Interger	-0288	530E-03	476E-02	Mettl7a	18709	Hypo
chr17	71480819	71482167	Intron (NM_0	-0451	530E-03	476E-02	Celf2	71209	Hypo
chr13	97731404	97733310	Distal Interger	-0361	531E-03	477E-02	Lyplal1	-73537	Hypo
chr2	170557548	170571620	Distal Interger	-0246	532E-03	477E-02	Dear	-257366	Hypo
chr17	86153924	86155200	Distal Interger	-0494	532E-03	477E-02	Lyst	-124319	Hypo
chr6	39638559	39640133	Distal Interger	-0503	532E-03	477E-02	Rock2	-39238	Hypo
chr7	113384569	113386912	Promoter (<=:	-0266	533E-03	478E-02	Phf5a	0	Hypo
chr13	39426577	39433163	Distal Interger	-0263	533E-03	478E-02	Mir3473	-66083	Hypo
chr20	46597332	46598124	Intron (NM_0	-0533	533E-03	478E-02	Sobp	62760	Hypo
chr3	108166228	108168866	Distal Interger	-0377	534E-03	478E-02	Adal	53824	Hypo
chr2	39409665	39411285	Distal Interger	-0439	535E-03	479E-02	Smim15	-116971	Hypo
chr7	102300280	102301128	Distal Interger	-0544	535E-03	479E-02	Khdrbs3	1462346	Hypo
chr1	177234252	177235427	Intron (NM_0	-0518	536E-03	479E-02	Cacng3	32262	Hypo
chr15	37351361	37352104	Distal Interger	-0584	537E-03	480E-02	Ctsb	-37532	Hypo
chr5	138398415	138400384	Intron (NM_1	-0410	537E-03	480E-02	Stk40	17256	Hypo
chrX	28131056	28137077	Distal Interger	-0273	537E-03	480E-02	Gpm6b	-29468	Hypo
chr14	7174647	7175133	Distal Interger	-0713	538E-03	481E-02	Arhgap24	-325152	Hypo
chr8	41197350	41197740	Distal Interger	-0708	538E-03	481E-02	Hspa8	13953	Hypo
chr8	22677705	22680387	Intron (NM_0	-0335	538E-03	481E-02	Npsr1	70759	Hypo
chrX	52871073	52877450	Distal Interger	-0253	539E-03	481E-02	Il1rapl1	-233423	Hypo
chr2	215086493	215087853	Intron (NM_0	-0410	539E-03	481E-02	Camk2d	62489	Hypo
chrX	108990261	108990820	Distal Interger	-0744	540E-03	482E-02	Lhfpl1	-116801	Hypo
chr9	11164546	11166179	Distal Interger	-0435	540E-03	482E-02	Rftn1	359935	Hypo
chr2	216436654	216437868	Distal Interger	-0475	540E-03	482E-02	Fam241a	-61461	Hypo

chr1	223987867	223988769	Distal Interger	-0583	541E-03	482E-02	Smarca2	-202356	Hypo
chr4	82188772	82189012	Distal Interger	-0800	541E-03	482E-02	Creb5	-204718	Hypo
chr11	29257805	29258546	Distal Interger	-0604	541E-03	482E-02	Tiam1	-97897	Hypo
chr12	35428637	35431056	Distal Interger	-0340	541E-03	482E-02	Ptpn11	63201	Hypo
chr20	26742958	26743720	Distal Interger	-0503	541E-03	483E-02	Edar	-124675	Hypo
chr1	52508394	52508743	Distal Interger	-0674	541E-03	483E-02	Ccr6	-9824	Hypo
chr10	25041185	25041763	Distal Interger	-0647	542E-03	483E-02	Mat2b	78732	Hypo
chr11	78699442	78702916	Distal Interger	-0271	542E-03	483E-02	Tra2b	-86004	Hypo
chr18	61770151	61775077	Intron (NM_0(	-0245	542E-03	483E-02	Fam210a	111046	Hypo
chr8	113236062	113236639	Distal Interger	-0707	543E-03	483E-02	Pdcd6ip	410134	Hypo
chr11	71230675	71232101	Intron (NM_0(	-0439	543E-03	484E-02	Atp13a4	4214	Hypo
chr6	39617828	39619860	Distal Interger	-0345	543E-03	484E-02	E2f6	25326	Hypo
chr8	86397262	86397506	Distal Interger	-0739	543E-03	484E-02	Tent5a	-168217	Hypo
chr2	100730295	100731623	Distal Interger	-0456	544E-03	484E-02	Cyp7b1	-60597	Hypo
chr10	75073216	75081566	Promoter (<=1	-0237	544E-03	484E-02	Mmd	0	Hypo
chr17	75030556	75031331	Distal Interger	-0610	544E-03	484E-02	Nmt2	-69548	Hypo
chr14	98020069	98052866	Promoter (<=1	-0224	545E-03	485E-02	Bcl11a	0	Hypo
chr1	123953069	123953376	Distal Interger	-0714	545E-03	485E-02	Nr2f2	62826	Hypo
chr9	111351154	111352962	Distal Interger	-0553	545E-03	485E-02	Ndc80	72647	Hypo
chr4	34621970	34622637	Distal Interger	-0562	546E-03	486E-02	Sem1	69368	Hypo
chr17	67314399	67315064	Exon (NM_001	-0541	547E-03	486E-02	Prkcq	63640	Hypo
chr4	82994235	82994600	Intron (NM_0(	-0747	547E-03	486E-02	Tril	-67063	Hypo
chr2	135827009	135827724	Intron (NM_0(	-0601	547E-03	486E-02	Mgst2	133453	Hypo
chr7	128911163	128912282	Promoter (2-3	-0400	547E-03	486E-02	Slc48a1	-2941	Hypo
chr6	52911680	52912302	Intron (NM_0(	-0638	547E-03	486E-02	Ankmy2	22717	Hypo
chr5	53815787	53818590	Distal Interger	-0368	547E-03	486E-02	RGD1306195	-637415	Hypo
chr1	50521467	50522757	Distal Interger	-0432	548E-03	486E-02	Qki	133769	Hypo
chr16	9288805	9294199	Distal Interger	-0289	549E-03	487E-02	Rbp3	21267	Hypo
chr8	113122982	113125342	Distal Interger	-0443	549E-03	487E-02	Pdcd6ip	521431	Hypo
chr6	88769930	88774889	Intron (NM_1:	-0270	549E-03	487E-02	Pygl	-29670	Hypo
chr7	45776688	45779230	Distal Interger	-0340	549E-03	487E-02	E2f7	-371347	Hypo
chr18	48310062	48312430	Exon (NM_001	-0353	549E-03	487E-02	Zfp608	98035	Hypo
chr13	38568846	38569948	Distal Interger	-0506	549E-03	487E-02	Mgat5	-106171	Hypo

chr15	34838493	34839744	Intron (NM_0(	-0528	549E-03	487E-02	Spata13	59957	Hypo
chr12	10016441	10017600	Distal Interger	-0509	550E-03	487E-02	Nptx2	30745	Hypo
chr5	47421167	47423491	Promoter (<=1	-0268	550E-03	488E-02	Ube2j1	0	Hypo
chr9	82118097	82119424	Distal Interger	-0422	550E-03	488E-02	Cul3	-466584	Hypo
chr13	96180592	96181198	Distal Interger	-0688	550E-03	488E-02	Hlx	104552	Hypo
chr12	27530339	27531895	Distal Interger	-0458	550E-03	488E-02	Ran	146353	Hypo
chr7	8184792	8186876	Promoter (<=1	-0391	550E-03	488E-02	Gna15	0	Hypo
chr5	96545085	96548311	Distal Interger	-0299	550E-03	488E-02	Nfib	424671	Hypo
chr5	154093206	154097556	Exon (NM_001	-0287	550E-03	488E-02	Dnajc16	8531	Hypo
chr8	78102609	78106003	Distal Interger	-0304	551E-03	488E-02	Tinag	-217532	Hypo
chr15	19640256	19640721	Distal Interger	-0782	551E-03	488E-02	Bmp4	-17975	Hypo
chr13	67254400	67260944	Distal Interger	-0251	552E-03	489E-02	Ier5	11282	Hypo
chr4	146943111	146954661	Exon (NM_01	-0250	552E-03	489E-02	Sec13	-51981	Hypo
chr1	244438397	244440495	Exon (NM_001	-0400	553E-03	489E-02	Dpcd	29622	Hypo
chr6	8070195	8070796	Intron (NM_0	-0623	553E-03	489E-02	RGD1562146	-105967	Hypo
chr15	31710254	31712213	Promoter (2-3	-0269	553E-03	489E-02	Eef1akmt1	-2870	Hypo
chr3	143821096	143822988	Intron (NM_1	-0384	554E-03	490E-02	Pigu	34242	Hypo
chr13	76773798	76774530	Intron (NM_1	-0552	554E-03	490E-02	Atp1b1	32566	Hypo
chr17	77863168	77865187	Intron (NM_0	-0343	554E-03	490E-02	Nsun6	84788	Hypo
chr19	52308727	52310746	Exon (NM_001	-0367	554E-03	490E-02	Galnt2	33778	Hypo
chr2	203027828	203030051	Distal Interger	-0439	554E-03	490E-02	Olfm3	117235	Hypo
chr8	104278791	104281811	Distal Interger	-0321	554E-03	490E-02	Bfsp2	-215392	Hypo
chr3	108638537	108639857	Distal Interger	-0438	555E-03	491E-02	Mfap1a	-181793	Hypo
chr18	21525590	21526711	Distal Interger	-0566	556E-03	491E-02	Pik3c3	-318651	Hypo
chr3	101135116	101135616	Distal Interger	-0681	557E-03	491E-02	Aqr	-190090	Hypo
chr5	135973443	135974852	3' UTR	-0465	557E-03	491E-02	Ndufs5	4751	Hypo
chr4	19819314	19820065	Intron (NM_0	-0560	557E-03	491E-02	Pclo	229820	Hypo
chr2	99922793	99924234	Distal Interger	-0355	557E-03	491E-02	Mir124-2	-210632	Hypo
chr6	76934925	76936025	Promoter (2-3	-0463	557E-03	492E-02	Fbxo33	-2588	Hypo
chr5	160578776	160579901	Distal Interger	-0474	558E-03	492E-02	Slc2a5	-3337	Hypo
chr7	132397701	132399331	Promoter (<=1	-0279	558E-03	492E-02	Atg101	0	Hypo
chr18	25638758	25639195	Distal Interger	-0763	558E-03	492E-02	Epb41l4a	-151659	Hypo
chr8	105042027	105043129	Distal Interger	-0454	558E-03	492E-02	Acp3	-87791	Hypo

chr8	114115726	114117042	Exon (NM_001	-0474	559E-03	493E-02	Glb1	30218	Hypo
chr5	122088139	122088862	Promoter (2-3	-0590	559E-03	493E-02	Dio1	2121	Hypo
chr8	50460251	50461731	Distal Interger	-0392	559E-03	493E-02	Ncam1	-294714	Hypo
chr3	109667814	109676927	Promoter (<=1	-0271	560E-03	493E-02	Gatm	0	Hypo
chr12	32721499	32722399	Distal Interger	-0548	560E-03	493E-02	Hcar2	-3935	Hypo
chr18	34130340	34132712	Exon (NM_001	-0347	560E-03	493E-02	Sh3rf2	83461	Hypo
chr3	65086244	65087124	Distal Interger	-0580	561E-03	494E-02	Pde1a	-61370	Hypo
chr5	57430314	57431760	Intron (NM_0(	-0444	561E-03	494E-02	Unc13b	-18093	Hypo
chr15	80481773	80508911	Promoter (<=1	-0261	562E-03	495E-02	Slain1	0	Hypo
chr10	62237513	62240914	Intron (NM_0(	-0295	562E-03	495E-02	Ssh2	39887	Hypo
chr15	16773887	16775666	Exon (NM_182	-0379	562E-03	495E-02	Pdhb	21326	Hypo
chr8	120573094	120575360	Distal Interger	-0329	562E-03	495E-02	Ctnnb1	-81225	Hypo
chr16	37442560	37442947	Distal Interger	-0711	563E-03	495E-02	Spcs3	137202	Hypo
chr11	77602809	77605312	Distal Interger	-0324	564E-03	495E-02	St6gal1	-33606	Hypo
chr8	41173340	41175321	Distal Interger	-0349	564E-03	496E-02	Hspa8	-8076	Hypo
chr8	21256347	21264594	Distal Interger	-0322	564E-03	496E-02	Rp9	-251172	Hypo
chr16	83441993	83443107	Distal Interger	-0484	565E-03	496E-02	Slc10a2	-943421	Hypo
chr12	43941794	43944456	Distal Interger	-0327	565E-03	496E-02	Sez6l	-120479	Hypo
chr6	60913979	60917115	Distal Interger	-0344	565E-03	497E-02	Dnajb9	357948	Hypo
chr1	163039097	163039952	Intron (NM_0(	-0529	566E-03	497E-02	Tub	30587	Hypo
chr15	4349318	4350949	Intron (NM_03	-0377	566E-03	497E-02	Gng2	32682	Hypo
chr5	110713735	110714819	Intron (NM_0(	-0480	566E-03	497E-02	Hook1	-109682	Hypo
chr6	51211949	51212310	Intron (NM_0(	-0800	567E-03	498E-02	Hdac9	112861	Hypo
chr10	98929238	98930318	Exon (NM_001	-0630	567E-03	498E-02	Sdk2	17560	Hypo
chr3	35523712	35525183	Distal Interger	-0434	567E-03	498E-02	Rnd3	64100	Hypo
chr15	1181651	1181958	Distal Interger	-0785	568E-03	498E-02	Kcnma1	879574	Hypo
chr4	162946455	162947472	Promoter (1-2	-0474	568E-03	498E-02	Olr1	1051	Hypo
chr9	57435504	57437915	Distal Interger	-0320	568E-03	498E-02	Plcl1	533931	Hypo
chr1	200345517	200346147	Distal Interger	-0579	568E-03	498E-02	Smim38	25829	Hypo
chr11	68667642	68668362	Distal Interger	-0573	568E-03	498E-02	Cep19	18671	Hypo
chr6	120243123	120243911	Distal Interger	-0570	570E-03	499E-02	Gpr68	-77034	Hypo
chr16	6997025	7002701	Distal Interger	-0244	570E-03	499E-02	Btd	131477	Hypo
chr12	44402091	44402900	Distal Interger	-0535	570E-03	499E-02	Miat	-11720	Hypo

chr18	72185623	72186891	Distal Intergen	-0429	570E-03	499E-02	Slc14a2	-146161	Hypo
chr17	18201019	18205163	Promoter (<=1	-0280	571E-03	500E-02	Cap2	0	Hypo

Supplementary Table 5: Significantly overexpressed genes in the substantia nigra

logFC	*logCPM*	*F*	*PValue*	*FDR*	*Gene*	*DEG*
3326	9596	367537	850E-17	668E-13	Mt2A	UP
3510	6426	280713	215E-15	845E-12	Cebpd	UP
2360	5983	258928	559E-15	146E-11	Apold1	UP
3305	7362	235167	173E-14	340E-11	Mt1m	UP
5560	8849	192232	178E-13	241E-10	Hspb1	UP
2503	6670	189861	205E-13	241E-10	Ifitm3	UP
3755	5336	189111	215E-13	241E-10	Crispld2	UP
2958	7878	186630	250E-13	245E-10	Mt1	UP
5331	4461	177471	442E-13	386E-10	Bcl3	UP
5669	5339	174761	526E-13	414E-10	Socs3	UP
2961	5640	163577	111E-12	793E-10	Junb	UP
3683	6812	159324	149E-12	976E-10	Tuba1c	UP
2748	5464	155293	198E-12	120E-09	Angptl4	UP
5528	4359	151277	265E-12	149E-09	Lcn2	UP
3483	4320	144821	430E-12	225E-09	S100a11	UP
2065	5574	142399	518E-12	254E-09	Ier2	UP
7654	3906	137851	739E-12	342E-09	H19	UP
3529	5272	132996	109E-11	441E-09	Tubb6	UP
2172	6915	132700	112E-11	441E-09	Stat3	UP
6821	6394	132698	112E-11	441E-09	Timp1	UP
2862	5961	130942	130E-11	485E-09	Cdkn1a	UP
1942	7535	122781	260E-11	929E-09	C1qb	UP
1421	5917	119160	358E-11	122E-08	Vwa1	UP
2415	7177	113085	625E-11	205E-08	C1qc	UP
3011	4497	109912	844E-11	263E-08	Fcgr2b	UP
5207	4843	108947	927E-11	263E-08	Plac8	UP
1970	7022	108831	937E-11	263E-08	Clic1	UP
3408	7992	107777	104E-10	272E-08	Hmox1	UP
1381	7729	105001	136E-10	346E-08	Cnn3	UP
2644	6191	103276	162E-10	399E-08	Gpnmb	UP
1408	6628	102626	173E-10	413E-08	Itpkb	UP

1895	5754	100468	216E-10	500E-08	Mgp	UP
2567	6021	99510	239E-10	536E-08	Gadd45g	UP
6308	3728	98520	265E-10	578E-08	Inmt	UP
2470	4812	94295	415E-10	880E-08	Fgfrl1	UP
1642	4713	94077	425E-10	880E-08	Eng	UP
2465	4701	90616	623E-10	126E-07	Itgam	UP
1956	5492	87837	854E-10	165E-07	Gadd45a	UP
3383	3600	83881	136E-09	248E-07	Pla2g3	UP
6027	1948	82068	169E-09	288E-07	Ccl2	UP
2127	8029	80719	199E-09	333E-07	Gpd1	UP
0939	7289	80183	212E-09	345E-07	Irf2bpl	UP
1891	4607	80085	215E-09	345E-07	Tgm2	UP
2124	7558	79492	231E-09	364E-07	Spp1	UP
2973	4867	78721	254E-09	392E-07	Tspo	UP
1891	6477	78470	263E-09	395E-07	Igfbp2	UP
1348	6348	78360	266E-09	395E-07	LOC12009740	UP
1446	7070	76728	327E-09	465E-07	Arpc1b	UP
1298	6438	76574	334E-09	465E-07	Ece1	UP
0743	10253	75124	402E-09	515E-07	LOC12009738	UP
2072	4161	75117	402E-09	515E-07	Hrct1	UP
1290	7368	75107	403E-09	515E-07	Anxa3	UP
1066	7048	75046	406E-09	515E-07	Mrps6	UP
1387	7036	74744	422E-09	524E-07	Chi3l1	UP
0886	7814	74675	426E-09	524E-07	Picalm	UP
1196	7317	74505	436E-09	527E-07	Slc2a1	UP
3731	3471	74395	442E-09	527E-07	Maff	UP
1068	5768	74048	463E-09	543E-07	Egfl7	UP
2102	4960	73898	472E-09	546E-07	S100a6	UP
1145	6007	73749	481E-09	548E-07	Ier3	UP
4321	5373	73304	510E-09	565E-07	Lgals3	UP
1916	5357	72638	557E-09	609E-07	Rras	UP
1895	8789	71857	618E-09	666E-07	Sgk1	UP
1418	5665	71140	681E-09	720E-07	Pfkfb3	UP

1018	7726	70993	694E-09	720E-07	Epas1	UP
1108	8226	70983	695E-09	720E-07	Sat1	UP
3637	3889	69717	826E-09	826E-07	Zfp36	UP
1291	8057	69687	829E-09	826E-07	Ralgds	UP
1347	6017	69590	840E-09	826E-07	Tagln2	UP
2737	3691	69040	907E-09	880E-07	RGD1563581	UP
1903	4649	68859	929E-09	892E-07	Anxa2	UP
1257	5474	66725	125E-08	119E-06	Cdo1	UP
1556	5211	65819	142E-08	132E-06	Il4r	UP
1224	5800	65698	145E-08	133E-06	Klf6	UP
2370	4142	65417	151E-08	136E-06	Cd44	UP
1226	10698	64483	173E-08	154E-06	Cd63	UP
1852	5662	64138	182E-08	160E-06	Plin2	UP
1094	7089	63639	195E-08	169E-06	Adam15	UP
1128	7242	63627	196E-08	169E-06	Igfbp7	UP
1824	5703	63470	200E-08	170E-06	Apln	UP
1710	5958	63454	201E-08	170E-06	RGD1564664	UP
1328	5868	62943	216E-08	181E-06	Myl12a	UP
1357	6207	62682	225E-08	184E-06	Spry2	UP
1401	7460	62677	225E-08	184E-06	Rhoc	UP
1169	6102	62115	245E-08	198E-06	Id1	UP
0736	6685	60934	292E-08	234E-06	Ctps1	UP
1766	6898	60769	299E-08	238E-06	Midn	UP
0830	7260	60547	310E-08	244E-06	Hnrnpf	UP
1336	4775	59158	383E-08	295E-06	Myl9	UP
1963	4588	58947	396E-08	302E-06	Trim47	UP
4350	3847	58501	424E-08	318E-06	Fos	UP
3088	3451	57979	460E-08	341E-06	Tmem252	UP
1048	6212	57814	472E-08	347E-06	Podxl	UP
3528	3480	56821	552E-08	398E-06	Ddit4l2	UP
1307	8109	56571	574E-08	411E-06	C1qa	UP
1096	5106	56493	582E-08	412E-06	Srgn	UP
0827	10609	55829	647E-08	454E-06	Actb	UP

1622	5475	55253	710E-08	494E-06	Rbm3	UP
1475	4931	55046	734E-08	507E-06	Apcdd1	UP
1221	5762	54707	776E-08	526E-06	Ehd4	UP
0710	7302	54350	822E-08	550E-06	Wdr1	UP
1114	5526	53869	890E-08	588E-06	Pdlim4	UP
0748	8120	53600	930E-08	600E-06	Adora1	UP
1819	6235	53531	941E-08	602E-06	Vim	UP
1528	4119	53045	102E-07	642E-06	Ogfrl1	UP
1423	5405	52998	103E-07	642E-06	Spred3	UP
0831	6188	52608	110E-07	670E-06	Srxn1	UP
1610	7404	52571	110E-07	670E-06	Hspb8	UP
0852	5476	52552	111E-07	670E-06	Stk40	UP
1425	5421	52493	112E-07	672E-06	Crip1	UP
1227	5146	52360	114E-07	682E-06	Pnpla2	UP
1107	6065	51749	127E-07	739E-06	Nek6	UP
1446	6531	51278	137E-07	783E-06	Tmbim1	UP
3179	2847	51125	141E-07	798E-06	Pla1a	UP
0652	12395	50875	147E-07	827E-06	Tmsb4x	UP
0780	10407	50243	164E-07	900E-06	Glul	UP
1189	8019	50218	165E-07	900E-06	Grn	UP
1782	5066	49817	177E-07	958E-06	Tinagl1	UP
3002	4012	49257	195E-07	102E-05	Fcgr3a	UP
0906	6083	49190	197E-07	103E-05	Esam	UP
0963	6783	48248	233E-07	119E-05	Cxcl14	UP
0785	7103	47716	256E-07	129E-05	Slc39a1	UP
2207	3835	46918	295E-07	146E-05	Lrg1	UP
1415	4751	46901	296E-07	146E-05	Oaf	UP
0759	6720	46737	305E-07	149E-05	Htra1	UP
1612	4674	46609	312E-07	152E-05	Dbx2	UP
2060	3138	46562	315E-07	152E-05	Emp3	UP
0949	6211	46016	348E-07	167E-05	Azin1	UP
0679	6853	45908	355E-07	169E-05	Odc1	UP
1549	4896	45696	369E-07	174E-05	Fgl2	UP

1087	6693	45175	407E-07	187E-05	Lgmn	UP
0970	6109	44974	422E-07	193E-05	Arf4	UP
1473	6158	44917	427E-07	194E-05	Jun	UP
0646	6783	44710	443E-07	199E-05	Ccnd3	UP
1571	5626	44297	479E-07	214E-05	Dusp1	UP
1325	4730	44105	497E-07	219E-05	Coq8b	UP
0718	8347	43975	509E-07	224E-05	Cldn5	UP
1045	6858	43712	535E-07	234E-05	Fam219b	UP
1542	10848	43557	551E-07	239E-05	Gfap	UP
1216	4856	42586	663E-07	285E-05	Relt	UP
1161	5608	42477	677E-07	290E-05	Coro1a	UP
3843	3283	42078	732E-07	311E-05	LOC298795	UP
1002	4822	41897	758E-07	319E-05	Wfdc1	UP
0966	4936	41669	793E-07	332E-05	Orai1	UP
1040	6021	41369	841E-07	350E-05	Ppp1r2	UP
0948	5482	41328	847E-07	351E-05	Cavin3	UP
0832	7028	41179	872E-07	357E-05	Septin11	UP
0850	6002	41123	882E-07	360E-05	Txnrd1	UP
0940	8055	41083	889E-07	361E-05	Pim3	UP
2032	3597	40979	908E-07	366E-05	Hmgb2	UP
1020	7393	40957	912E-07	366E-05	Mcam	UP
0644	7924	40896	923E-07	367E-05	Eif4a1	UP
1448	4961	40892	923E-07	367E-05	Mcl1	UP
1127	5185	40845	932E-07	368E-05	Ripor3	UP
1095	4952	40648	969E-07	381E-05	Zdhhc18	UP
1300	5515	39472	123E-06	474E-05	Nfkbia	UP
0997	4712	39444	123E-06	474E-05	Scamp2	UP
1368	5310	39436	124E-06	474E-05	Ifi30	UP
0608	10879	38978	136E-06	515E-05	LOC10036145	UP
0984	4583	38869	139E-06	522E-05	Tm4sf1	UP
3541	3210	38445	151E-06	565E-05	Ifitm1	UP
0879	4696	38233	158E-06	586E-05	Mpi	UP
1594	3994	38126	162E-06	594E-05	Lsp1	UP

0709	7374	38022	165E-06	604E-05	Taldo1	UP
1217	4925	37928	168E-06	613E-05	Ier5	UP
1220	4392	37882	170E-06	616E-05	Hyal2	UP
0709	6483	37802	173E-06	623E-05	Ipo5	UP
2210	4038	37596	180E-06	639E-05	Kif22	UP
2000	3748	37200	196E-06	685E-05	Ifitm2	UP
0410	9055	36948	207E-06	719E-05	Cd59	UP
0754	5808	36850	211E-06	731E-05	Slc44a2	UP
1863	4076	36704	217E-06	750E-05	Upp1	UP
1298	3941	36530	226E-06	772E-05	Ptpn2	UP
0738	7012	36499	227E-06	773E-05	Ifngr1	UP
0952	5790	36484	228E-06	773E-05	Cd151	UP
1683	5895	36095	248E-06	829E-05	Pdpn	UP
1366	5096	35994	253E-06	839E-05	Zyx	UP
0637	8586	35865	260E-06	859E-05	H3f3b	UP
0969	5977	35656	272E-06	895E-05	Ctsl	UP
1366	3941	35580	276E-06	906E-05	Hebp1	UP
0968	5859	35450	284E-06	928E-05	Cdk5rap3	UP
0730	5968	35205	300E-06	967E-05	Sec61a1	UP
1989	3669	35102	307E-06	984E-05	Casp4	UP
0645	6152	34825	326E-06	102E-04	Emd	UP
0963	6727	34677	336E-06	105E-04	Ucp2	UP
1196	5824	34597	342E-06	106E-04	Acer2	UP
0549	9060	34573	344E-06	107E-04	Atf4	UP
0563	6474	34392	358E-06	110E-04	Timp3	UP
0764	5890	34192	374E-06	114E-04	LOC12009662	UP
1122	5131	34178	375E-06	114E-04	Adgrl4	UP
0874	6760	34157	377E-06	114E-04	Ccdc88c	UP
2166	3207	34156	377E-06	114E-04	Cebpb	UP
0891	5563	34028	388E-06	116E-04	Coq10b	UP
0727	6392	34012	389E-06	116E-04	Cystm1	UP
1005	5124	33965	394E-06	117E-04	Rassf1	UP
2745	5219	33805	408E-06	121E-04	Crym	UP

0950	5996	33788	409E-06	121E-04	Bag3	UP
0435	8807	33626	424E-06	124E-04	Sumo3	UP
0781	6270	33354	451E-06	131E-04	St3gal4	UP
0743	9213	33202	467E-06	134E-04	Ctsb	UP
1182	5966	32747	517E-06	147E-04	Cd38	UP
0659	6763	32725	520E-06	148E-04	Eif3d	UP
1475	4597	32695	523E-06	148E-04	Ppp1r15a	UP
1500	4941	32494	547E-06	153E-04	Cp	UP
1561	5233	32277	575E-06	159E-04	Serping1	UP
1619	5030	32232	581E-06	160E-04	Cd24	UP
0814	5364	32057	605E-06	166E-04	Nup93	UP
0665	6337	32045	607E-06	166E-04	Sqle	UP
1468	4237	31995	614E-06	167E-04	Rnf122	UP
1305	4271	31851	634E-06	172E-04	Capg	UP
0795	5438	31784	644E-06	174E-04	Snx18	UP
0988	5377	31756	648E-06	175E-04	Susd6	UP
2431	5151	31371	709E-06	188E-04	Gpx2	UP
0824	6444	31293	722E-06	191E-04	Ankrd28	UP
1527	5638	31285	723E-06	191E-04	Ctsz	UP
0613	10175	31250	729E-06	191E-04	Clu	UP
0523	7034	31240	731E-06	191E-04	G3bp1	UP
2011	3568	31234	732E-06	191E-04	Cbr3	UP
0843	7378	31228	733E-06	191E-04	LOC12010237	UP
4554	2168	31118	752E-06	195E-04	S100a8	UP
0767	6189	30978	777E-06	201E-04	Septin9	UP
0691	7237	30960	780E-06	201E-04	Ppdpf	UP
1107	4464	30951	782E-06	201E-04	Ppp1r18	UP
0842	5746	30923	787E-06	202E-04	Atp6v0e1	UP
1661	3727	30856	799E-06	203E-04	Fcgr1a	UP
1012	5006	30802	809E-06	205E-04	Fkbp5	UP
0859	4516	30626	843E-06	213E-04	Srarp	UP
1541	4318	30534	862E-06	217E-04	Slc11a1	UP
1975	3326	30187	936E-06	234E-04	Clec12a	UP

0854	5335	29996	979E-06	243E-04	Hif1a	UP
0797	6033	29703	105E-05	258E-04	Mrpl45	UP
0736	7132	29700	105E-05	258E-04	Tpm3	UP
0521	8923	29669	106E-05	258E-04	Rps2	UP
0596	6994	29660	106E-05	258E-04	Raver2	UP
1633	4180	29522	110E-05	265E-04	Btg3	UP
1145	6055	29292	116E-05	275E-04	Sox9	UP
1175	6019	29019	124E-05	293E-04	Galnt6	UP
0668	6976	28913	127E-05	299E-04	Slc3a2	UP
0936	5917	28205	151E-05	351E-04	Rps27l	UP
0867	7405	28187	152E-05	351E-04	LOC10036008	UP
0664	6220	28150	153E-05	353E-04	Cdr2l	UP
0792	6069	28063	157E-05	358E-04	Ccnd1	UP
0421	7854	27749	169E-05	385E-04	Cnbp	UP
1200	3925	27525	179E-05	405E-04	Fam110d	UP
1065	4999	27518	179E-05	405E-04	Lcp1	UP
0505	7245	27488	181E-05	407E-04	Eif2s2	UP
0383	9654	27350	187E-05	419E-04	Snx32	UP
0664	5788	27285	190E-05	425E-04	Dtymk	UP
0703	5316	27240	192E-05	428E-04	Traf7	UP
0801	7091	27179	195E-05	434E-04	Actg1	UP
1200	4950	27141	197E-05	436E-04	Mthfd2	UP
0689	7070	27025	203E-05	448E-04	Flot2	UP
2111	2996	26970	206E-05	453E-04	Plp2	UP
0699	6113	26933	208E-05	455E-04	Etf1	UP
0609	6215	26870	211E-05	461E-04	LOC10255140	UP
0393	9318	26792	215E-05	469E-04	B2m	UP
0562	7818	26765	217E-05	471E-04	Gsta1	UP
0929	5805	26691	221E-05	475E-04	Erf	UP
0485	6822	26689	221E-05	475E-04	Insig1	UP
1787	4828	26684	221E-05	475E-04	Tagln	UP
1409	4250	26648	223E-05	478E-04	Olfml2b	UP
0875	5159	26626	224E-05	480E-04	Slc25a37	UP

0772	6522	26463	234E-05	493E-04	Pnp	UP
1046	5843	26229	248E-05	517E-04	Erbin	UP
0560	6314	26198	250E-05	520E-04	Mapk14	UP
1877	4051	26154	253E-05	522E-04	Tnfrsf12a	UP
0684	7554	26149	253E-05	522E-04	Nudt4	UP
0805	6687	26149	253E-05	522E-04	Ptp4a3	UP
1239	4353	26134	254E-05	523E-04	Eva1b	UP
2434	2956	25847	274E-05	556E-04	Folr2	UP
0435	7233	25766	280E-05	566E-04	Gpx1	UP
0842	5473	25715	284E-05	572E-04	Slc41a3	UP
0367	8532	25674	287E-05	576E-04	U2af1	UP
1544	5052	25504	299E-05	599E-04	Zfp36l1	UP
1154	4549	25483	301E-05	601E-04	Ier5l	UP
0576	5772	25417	306E-05	610E-04	Kdelr2	UP
1222	4768	25396	308E-05	612E-04	Ptpn6	UP
2434	3521	25379	309E-05	613E-04	LOC10834810	UP
0583	6682	24640	376E-05	733E-04	Mapre1	UP
1008	6616	24589	381E-05	742E-04	Tyrobp	UP
1130	3953	24509	389E-05	753E-04	Qprt	UP
1680	3938	24494	391E-05	753E-04	Birc5	UP
0788	5846	24483	392E-05	753E-04	Cttnbp2nl	UP
0892	5984	24471	393E-05	753E-04	Litaf	UP
0873	5614	24470	393E-05	753E-04	Pabpc4	UP
0750	6720	24439	396E-05	757E-04	Serpinh1	UP
0812	6406	24343	407E-05	771E-04	Ifngr2	UP
0799	4712	24300	411E-05	778E-04	Ptpn12	UP
0862	5399	24202	422E-05	796E-04	Samd4b	UP
1200	4861	24163	427E-05	803E-04	Cmtm7	UP
0648	5842	24126	431E-05	807E-04	Camkk2	UP
2322	3501	24062	438E-05	818E-04	Rarres2	UP
0600	5413	24059	439E-05	818E-04	Ltbr	UP
0999	4985	24033	442E-05	822E-04	Arap1	UP
0830	5524	24006	445E-05	824E-04	Ptbp1	UP

0563	7573	23678	486E-05	893E-04	Cotl1	UP
0655	5598	23575	500E-05	917E-04	LOC10091204	UP
0527	6590	23446	518E-05	945E-04	Gpr107	UP
1657	3123	23160	560E-05	102E-03	Tnfrsf1a	UP
1508	3848	23132	564E-05	102E-03	Slc5a3	UP
0536	8563	23005	584E-05	106E-03	Fkbp1a	UP
0758	5408	22992	586E-05	106E-03	Wnt7b	UP
0330	9445	22818	615E-05	110E-03	Rpl4	UP
0431	9011	22736	629E-05	112E-03	Arpc3	UP
1645	3313	22673	640E-05	113E-03	Ly86	UP
0413	6525	22626	648E-05	114E-03	Ube2r2	UP
0774	5115	22520	668E-05	117E-03	Rab33a	UP
0662	4717	22417	687E-05	120E-03	Timm17b	UP
0532	6495	22409	689E-05	120E-03	Ppp1r14b	UP
0372	8402	22408	689E-05	120E-03	Gps2	UP
0745	6127	22356	699E-05	121E-03	Cox6a2	UP
0702	5278	22316	707E-05	122E-03	Dpm1	UP
0741	5293	22312	707E-05	122E-03	Acy1	UP
0508	6211	22300	710E-05	122E-03	Rasip1	UP
0726	5266	22257	718E-05	123E-03	Comt	UP
0512	6081	22195	731E-05	125E-03	Fam98a	UP
1116	5290	22185	733E-05	125E-03	Irf8	UP
0533	6816	21918	790E-05	133E-03	Paics	UP
1329	4850	21851	805E-05	135E-03	Scara3	UP
0552	6690	21711	837E-05	140E-03	Psmd11	UP
1053	5544	21611	861E-05	142E-03	RT1-DMb	UP
0623	5079	21606	863E-05	142E-03	Ifrd1	UP
0884	4878	21595	865E-05	142E-03	RT1-DMa	UP
2495	2066	21455	900E-05	147E-03	Lst1	UP
0597	6282	21425	908E-05	148E-03	Fbl	UP
0464	7361	21341	930E-05	151E-03	P4hb	UP
0547	6663	21339	931E-05	151E-03	Rapgef1	UP
0620	6180	21260	952E-05	153E-03	Shisa5	UP

0450	12264	21091	999E-05	160E-03	Fth1	UP
1268	4029	21041	101E-04	162E-03	Vamp5	UP
0772	6773	21012	102E-04	163E-03	Aif1	UP
1240	4870	20954	104E-04	165E-03	Tmem159	UP
1043	4203	20818	108E-04	170E-03	Alox5ap	UP
0442	6656	20801	109E-04	171E-03	Serp1	UP
0371	9423	20794	109E-04	171E-03	Rpsa	UP
0809	5524	20793	109E-04	171E-03	Slc16a1	UP
0470	6227	20719	111E-04	174E-03	Lrrc42	UP
0429	6389	20701	112E-04	174E-03	Paox	UP
0459	9718	20632	114E-04	177E-03	Ptpn11	UP
0534	5671	20556	117E-04	180E-03	Iffo1	UP
1342	3939	20543	117E-04	180E-03	Pde9a	UP
0563	5403	20430	121E-04	185E-03	Tmem184b	UP
0667	5218	20216	129E-04	195E-03	Trmt61a	UP
0726	5340	20204	129E-04	195E-03	Hilpda	UP
0564	7532	20107	133E-04	200E-03	RT1-A3	UP
0530	8783	20094	133E-04	200E-03	Agpat4	UP
0589	7364	20074	134E-04	201E-03	Jund	UP
0692	6140	19944	139E-04	208E-03	Sulf2	UP
1333	4433	19804	145E-04	215E-03	Fpgs	UP
0595	5188	19756	147E-04	217E-03	Cdc16	UP
0844	5303	19670	151E-04	222E-03	Efhd1	UP
0467	7070	19649	152E-04	223E-03	Npdc1	UP
0591	7736	19637	153E-04	223E-03	Arpc4	UP
0608	6864	19613	154E-04	224E-03	Ssbp4	UP
1474	3668	19524	158E-04	230E-03	Rac2	UP
0867	6140	19459	161E-04	233E-03	Tsc22d4	UP
0588	5494	19454	161E-04	233E-03	Xpnpep1	UP
0445	6373	19454	161E-04	233E-03	Cttn	UP
0291	8342	19418	163E-04	235E-03	Arf1	UP
0467	7389	19380	165E-04	237E-03	Arpc2	UP
0768	6582	19373	165E-04	237E-03	S100a10	UP

0621	6518	19354	166E-04	238E-03	Phykpl	UP
0514	6243	19314	168E-04	240E-03	Psme2	UP
0858	4937	19239	172E-04	245E-03	RT1-CE4	UP
0448	6277	19159	176E-04	250E-03	Ogg1	UP
0641	5687	19124	178E-04	251E-03	Hnrnpab	UP
0514	7068	19115	178E-04	251E-03	Snrpg	UP
0544	7562	19087	180E-04	253E-03	Kndc1	UP
0310	8365	19045	182E-04	255E-03	Snrpd1	UP
0535	6128	19044	182E-04	255E-03	Asns	UP
0757	4949	18999	185E-04	258E-03	Agfg1	UP
0788	4912	18911	190E-04	263E-03	Arf2	UP
0624	5973	18906	190E-04	263E-03	Stt3a	UP
0641	5505	18838	194E-04	268E-03	Rcor2	UP
1422	4163	18788	197E-04	271E-03	Acta2	UP
1847	3474	18785	197E-04	271E-03	Cd68	UP
0702	5564	18765	198E-04	272E-03	Lamb2	UP
1100	4554	18735	200E-04	274E-03	Gmfg	UP
0529	6101	18694	203E-04	277E-03	Gpsm1	UP
0310	9925	18623	207E-04	282E-03	LOC10036229	UP
0955	5173	18559	211E-04	286E-03	Slc35e4	UP
0724	5827	18538	212E-04	288E-03	Rab13	UP
0870	4459	18478	216E-04	292E-03	Arrdc1	UP
0478	10715	18438	219E-04	295E-03	Ftl1	UP
0703	5254	18353	225E-04	301E-03	Ube2d2	UP
0446	6500	18224	234E-04	312E-03	Nr2c2ap	UP
0807	5826	18175	237E-04	316E-03	Nkain1	UP
0424	7134	18039	247E-04	327E-03	H1f0	UP
1203	4226	17986	252E-04	330E-03	Sertad1	UP
0610	5265	17974	252E-04	331E-03	Acss2	UP
0731	5658	17965	253E-04	331E-03	Rnf19b	UP
0737	6912	17926	256E-04	335E-03	Rnaset2	UP
0605	5437	17777	268E-04	349E-03	Txndc12	UP
1003	4419	17698	275E-04	356E-03	Snrpa	UP

1058	4304	17632	281E-04	361E-03	Fxyd5	UP
0764	4952	17354	306E-04	389E-03	Pcna	UP
0330	9837	17299	312E-04	395E-03	Ubc	UP
0392	7167	17203	321E-04	403E-03	Anp32b	UP
0408	7202	17095	332E-04	416E-03	Rhoq	UP
0514	5646	17065	335E-04	419E-03	Ybx1	UP
0572	6048	17045	338E-04	421E-03	Selenoh	UP
0575	5539	17008	342E-04	424E-03	Tfrc	UP
4258	4469	16901	353E-04	436E-03	Pmch	UP
0487	6353	16867	357E-04	438E-03	Flot1	UP
0659	6059	16801	365E-04	445E-03	Cic	UP
0952	5659	16785	367E-04	447E-03	Atn1	UP
0480	6782	16742	372E-04	453E-03	Pfn1	UP
0539	6050	16717	375E-04	455E-03	Mpzl1	UP
0698	6314	16707	376E-04	456E-03	Nacad	UP
0751	4782	16679	379E-04	459E-03	Dusp6	UP
0516	6088	16530	398E-04	478E-03	Eif4a3	UP
0302	8769	16520	399E-04	478E-03	Hnrnpa3	UP
0510	6343	16485	403E-04	483E-03	Carhsp1	UP
0753	5158	16476	405E-04	483E-03	B3gnt2	UP
0817	5798	16458	407E-04	485E-03	Mfsd2a	UP
1899	5291	16399	415E-04	492E-03	Rgs4	UP
0658	4975	16389	416E-04	493E-03	Ube2f	UP
2255	2277	16366	419E-04	495E-03	Hcst	UP
0985	4305	16346	422E-04	497E-03	Ntmt1	UP
0976	5175	16328	424E-04	499E-03	Mob3a	UP
0767	4258	16293	429E-04	504E-03	Trip10	UP
0675	4731	16246	436E-04	509E-03	Tbc1d10a	UP
0427	5745	16221	439E-04	513E-03	Eif3el1	UP
0349	6825	16203	442E-04	514E-03	Laptm4b	UP
0542	6407	16167	447E-04	519E-03	Nop58	UP
1052	3664	16167	447E-04	519E-03	Rps4y2	UP
0533	5886	16120	454E-04	526E-03	Snrpb	UP

0324	7964	16069	461E-04	533E-03	Sod2	UP
0866	3855	16041	466E-04	536E-03	Bid	UP
0940	3946	16026	468E-04	537E-03	Hopx	UP
0363	6763	16004	471E-04	540E-03	Wdr83os	UP
0718	5201	15965	477E-04	546E-03	Tmem150a	UP
0338	9045	15961	478E-04	546E-03	Nme2	UP
1420	5362	15957	479E-04	546E-03	Ptk2b	UP
0476	6578	15900	488E-04	554E-03	Calu	UP
0663	6529	15851	495E-04	562E-03	Nacc1	UP
0420	6514	15850	496E-04	562E-03	Snrpf	UP
1338	3141	15808	502E-04	568E-03	Pmf1	UP
0491	7645	15750	512E-04	578E-03	Akap2	UP
1049	3875	15720	517E-04	582E-03	Clec10a	UP
0652	6357	15679	524E-04	587E-03	Klf9	UP
0584	5335	15671	525E-04	588E-03	Lipe	UP
0751	4769	15639	531E-04	592E-03	Gch1	UP
0634	5544	15592	539E-04	600E-03	Lta4h	UP
1001	3633	15559	545E-04	605E-03	Nip7	UP
0486	5995	15445	566E-04	625E-03	Srf	UP
0532	5948	15368	581E-04	637E-03	Adipor2	UP
0683	4458	15329	588E-04	643E-03	Ankrd13b	UP
0549	6084	15273	599E-04	654E-03	Cdkn2aipnl	UP
0711	5521	15269	600E-04	654E-03	Plat	UP
0262	8462	15238	606E-04	659E-03	Pcbp1	UP
0430	6777	15225	609E-04	660E-03	Fgfr1	UP
0319	8250	15213	611E-04	662E-03	Rab5c	UP
0605	6125	15206	613E-04	663E-03	Cdc42se1	UP
0392	8906	15192	616E-04	665E-03	Rhoa	UP
0300	9030	15182	618E-04	666E-03	Tkt	UP
0695	5654	15169	620E-04	668E-03	Hs2st1	UP
0309	8611	15142	626E-04	673E-03	Capzb	UP
0580	5788	15087	637E-04	683E-03	Dusp7	UP
0790	4290	15024	651E-04	696E-03	Mettl1	UP

0476	7879	14960	665E-04	710E-03	Slc6a8	UP
0662	6835	14945	668E-04	712E-03	Klk6	UP
0568	5923	14835	693E-04	733E-03	Srm	UP
0467	7517	14822	696E-04	735E-03	Fscn1	UP
0591	6091	14760	711E-04	748E-03	Slc9a3r2	UP
0779	5227	14646	739E-04	772E-03	Syngr2	UP
0905	4322	14617	746E-04	774E-03	Gadd45b	UP
0574	5919	14616	747E-04	774E-03	Creg1	UP
1050	4187	14600	750E-04	777E-03	LOC12010302	UP
0343	6051	14577	756E-04	782E-03	Esd	UP
0357	6984	14520	771E-04	795E-03	Arhgdia	UP
0499	5783	14473	784E-04	807E-03	Bfar	UP
0553	5541	14397	804E-04	827E-03	LOC10369419	UP
0482	5695	14388	806E-04	828E-03	Capn12	UP
0483	6115	14380	809E-04	829E-03	Tmed2	UP
0485	5957	14279	837E-04	854E-03	Impdh2	UP
0602	4931	14224	853E-04	867E-03	Elk1	UP
0624	5315	14217	855E-04	868E-03	Med19	UP
0295	9676	14183	865E-04	874E-03	Npm1	UP
0980	4346	14107	888E-04	889E-03	Mvp	UP
0488	5841	14020	914E-04	909E-03	RGD1308134	UP
1129	3628	13950	937E-04	924E-03	Gpr84	UP
0360	6764	13904	952E-04	937E-03	Cap1	UP
0450	6697	13860	966E-04	949E-03	Eprs	UP
0496	5289	13856	968E-04	949E-03	Parp1	UP
0285	8913	13819	980E-04	958E-03	Rpl15	UP
0562	5520	13806	984E-04	960E-03	Bcar1	UP
0583	5354	13805	985E-04	960E-03	Abce1	UP
0780	4660	13766	998E-04	970E-03	Kpna2	UP
0301	6712	13759	100E-03	972E-03	Bcl2l1	UP
2034	5434	13731	101E-03	978E-03	Prkcd	UP
0509	6537	13627	105E-03	101E-02	Akap12	UP
0317	7076	13605	106E-03	102E-02	Ggnbp2	UP

0799	5294	13593	106E-03	102E-02	Sox2	UP
0410	6684	13562	107E-03	103E-02	Paf1	UP
0536	5341	13540	108E-03	103E-02	Fen1	UP
0388	6565	13444	112E-03	107E-02	Selenof	UP
0621	6503	13440	112E-03	107E-02	Extl1	UP
0953	4785	13297	118E-03	111E-02	Hmgcs2	UP
0734	7035	13288	118E-03	112E-02	Vgf	UP
0840	4484	13262	119E-03	112E-02	Ext1	UP
0599	5868	13260	119E-03	112E-02	Ampd3	UP
0536	6446	13236	120E-03	113E-02	Nras	UP
0584	4713	13210	121E-03	114E-02	Gnl3	UP
0501	5652	13161	123E-03	115E-02	Xpo1	UP
0306	9739	13156	124E-03	115E-02	Myl6	UP
1319	3293	13156	124E-03	115E-02	Mgst2	UP
0995	6075	13068	127E-03	117E-02	Rbfox1	UP
0554	5316	12988	131E-03	120E-02	Rnft2	UP
0502	6407	12986	131E-03	120E-02	Psmb2	UP
0973	4197	12935	134E-03	122E-02	Arhgef39	UP
0465	6450	12820	139E-03	127E-02	Usp14	UP
1075	3813	12810	140E-03	127E-02	Eif4ebp1	UP
0581	4906	12806	140E-03	127E-02	Mkln1	UP
0785	4730	12771	142E-03	128E-02	Ubr7	UP
0398	8293	12749	143E-03	129E-02	Slc44a1	UP
0722	4507	12736	143E-03	129E-02	Aen	UP
0275	8295	12716	144E-03	130E-02	Psmb4	UP
0675	4285	12692	146E-03	131E-02	Npm3	UP
0515	7761	12661	147E-03	132E-02	Best1	UP
0870	4020	12653	148E-03	132E-02	LOC10369162	UP
0828	4090	12641	148E-03	132E-02	Mocs1	UP
0372	7948	12622	149E-03	133E-02	H2az1	UP
0481	5249	12591	151E-03	134E-02	Pgam5	UP
0337	7767	12556	153E-03	136E-02	Gnb2	UP
0515	5782	12522	155E-03	137E-02	Fermt2	UP

0552	5583	12499	156E-03	138E-02	Homer3	UP
0296	7072	12486	157E-03	138E-02	G6pd	UP
0431	6275	12482	157E-03	138E-02	Hmga1	UP
0342	9079	12461	158E-03	139E-02	Ttyh1	UP
0878	3455	12440	159E-03	140E-02	Rfc4	UP
0583	5874	12430	160E-03	140E-02	Fam131a	UP
0667	4662	12388	162E-03	142E-02	Dcakd	UP
0424	7429	12387	162E-03	142E-02	Akap17a	UP
0887	4565	12372	163E-03	142E-02	Socs2	UP
0429	5885	12344	165E-03	143E-02	RGD735065	UP
0273	7550	12208	173E-03	149E-02	Selenok	UP
0539	5415	12165	176E-03	151E-02	Kctd13	UP
0427	5632	12162	176E-03	151E-02	Magoh	UP
0255	7659	12154	177E-03	151E-02	Pcbp2	UP
0657	4667	12053	183E-03	155E-02	Tapbp	UP
0483	5586	12050	184E-03	155E-02	Slc15a4	UP
0537	5603	12013	186E-03	157E-02	Tpm1	UP
0420	6518	11959	190E-03	160E-02	Ddx24	UP
0530	5199	11941	191E-03	160E-02	Eif1a	UP
0516	5525	11881	195E-03	163E-02	Commd5	UP
0549	5127	11874	196E-03	163E-02	Ddx39a	UP
0395	7697	11800	201E-03	168E-02	Raly	UP
0339	6271	11754	205E-03	170E-02	Scamp3	UP
0249	7479	11736	206E-03	171E-02	Sar1a	UP
0717	5750	11723	207E-03	172E-02	Spsb1	UP
0425	6272	11706	208E-03	173E-02	Ssr3	UP
0408	6394	11674	211E-03	174E-02	Pip5k1c	UP
0408	6067	11654	212E-03	175E-02	Caprin1	UP
0922	4027	11638	214E-03	176E-02	Psmb8	UP
0257	8469	11637	214E-03	176E-02	Rpl6	UP
0855	3751	11635	214E-03	176E-02	Sp110	UP
0312	7012	11619	215E-03	176E-02	Rab12	UP
0627	5239	11584	218E-03	178E-02	Lrp10	UP

0610	5326	11557	220E-03	180E-02	Plpp1	UP
0796	4255	11537	222E-03	180E-02	Galr2	UP
0242	8180	11519	223E-03	181E-02	Smim14	UP
0567	4357	11508	224E-03	182E-02	Psmd5	UP
0273	9798	11502	225E-03	182E-02	Tubb5	UP
0271	7763	11474	227E-03	184E-02	Mtch2	UP
0546	4729	11470	227E-03	184E-02	Eci2	UP
0512	5524	11448	229E-03	185E-02	Rasd2	UP
0655	4503	11411	232E-03	186E-02	Slc29a1	UP
0523	4970	11388	234E-03	188E-02	Idi1	UP
0353	7680	11346	238E-03	190E-02	Hmgb1	UP
0401	8378	11316	241E-03	191E-02	Gnao1	UP
0500	5299	11300	242E-03	192E-02	Arcn1	UP
0425	5787	11255	246E-03	195E-02	Slc41a1	UP
0292	8677	11215	250E-03	197E-02	Mlc1	UP
0528	4954	11211	250E-03	197E-02	Mogs	UP
0357	7334	11139	257E-03	201E-02	LOC12009426	UP
0359	7969	11130	258E-03	202E-02	Ptprf	UP
0298	7093	11122	259E-03	202E-02	Psma3	UP
0221	8831	11110	260E-03	202E-02	Rps13	UP
0439	5628	11101	261E-03	203E-02	Nfkbib	UP
0422	6324	11079	263E-03	204E-02	Cry2	UP
0525	6493	11077	263E-03	204E-02	Cd82	UP
0461	5936	11068	264E-03	205E-02	Mapkapk2	UP
0587	5691	11059	265E-03	205E-02	Arf6	UP
0319	9182	11055	265E-03	205E-02	Arf5	UP
0887	4301	11055	265E-03	205E-02	Rasl11b	UP
0753	4425	11048	266E-03	206E-02	Nfe2l2	UP
0792	4810	11031	268E-03	206E-02	Chac1	UP
0697	4814	11031	268E-03	206E-02	Cbx4	UP
1245	3563	11028	268E-03	206E-02	Pttg1	UP
0462	6459	11024	268E-03	206E-02	Rock2	UP
0448	6269	10967	274E-03	210E-02	Rnh1	UP

2498	5565	10922	279E-03	213E-02	Slc17a7	UP
0952	2750	10897	282E-03	215E-02	Nxt1	UP
0254	9837	10893	282E-03	215E-02	LOC12009504	UP
0829	5229	10869	285E-03	217E-02	Galnt9	UP
0252	8578	10860	286E-03	217E-02	Snrpd3	UP
0370	6445	10834	288E-03	219E-02	Trafd1	UP
0454	6123	10828	289E-03	219E-02	Rnf181	UP
0722	4277	10820	290E-03	220E-02	Catsperz	UP
0446	5940	10795	293E-03	221E-02	Yipf2	UP
0367	6321	10792	293E-03	221E-02	B4galt5	UP
0845	4586	10783	294E-03	222E-02	Sult1a1	UP
0399	7397	10775	295E-03	222E-02	Sdf4	UP
0687	5713	10775	295E-03	222E-02	Ankmy1	UP
0391	6255	10767	296E-03	222E-02	U2surp	UP
0459	5520	10763	296E-03	222E-02	Api5	UP
0408	7338	10752	298E-03	223E-02	Pgls	UP
0408	7585	10740	299E-03	223E-02	Marcksl1	UP
0542	4772	10738	299E-03	223E-02	Slc17a5	UP
0744	5321	10726	301E-03	224E-02	LOC10091082	UP
0469	5813	10651	309E-03	230E-02	Rars1	UP
0255	8028	10635	311E-03	231E-02	LOC10091117	UP
0346	6213	10613	314E-03	232E-02	Nek9	UP
0516	10488	10568	319E-03	236E-02	Tf	UP
0456	5953	10545	322E-03	237E-02	Acsf2	UP
0433	6259	10479	330E-03	241E-02	Yars1	UP
0885	3944	10367	345E-03	251E-02	Exosc2	UP
0511	4931	10353	347E-03	252E-02	Frmd8	UP
0429	6783	10346	348E-03	252E-02	Wdfy1	UP
0362	6523	10341	348E-03	252E-02	Ethe1	UP
0605	5260	10331	350E-03	253E-02	Csnk2a2	UP
0456	5358	10299	354E-03	255E-02	Zfp330	UP
0373	6771	10276	357E-03	257E-02	Mars1	UP
0389	7392	10244	362E-03	260E-02	Zeb1	UP

0261	7767	10225	364E-03	262E-02	Pnpo	UP
0581	5003	10205	367E-03	263E-02	Avpi1	UP
0501	4854	10196	368E-03	264E-02	Aasdhppt	UP
0251	7769	10193	369E-03	264E-02	Csnk1a1	UP
0212	8808	10156	374E-03	267E-02	Cdc42	UP
0445	4765	10136	377E-03	269E-02	Surf6	UP
0418	8023	10129	378E-03	269E-02	Fcer1g	UP
0417	5784	10090	384E-03	272E-02	Strap	UP
0371	5812	10088	384E-03	272E-02	Polr2g	UP
0726	5537	10068	387E-03	274E-02	LOC10835053	UP
0872	4490	10066	387E-03	274E-02	Rps17l	UP
0301	7934	10049	390E-03	276E-02	Rps18	UP
1412	3111	10041	391E-03	276E-02	Clec4a3	UP
0643	5313	10016	395E-03	278E-02	Smpd5	UP
0332	5919	10004	397E-03	279E-02	LOC12009668	UP
0411	7550	9964	403E-03	282E-02	Cmip	UP
0276	7754	9963	403E-03	282E-02	Mrps18b	UP
0536	5409	9959	404E-03	282E-02	Anxa11	UP
0378	6632	9922	410E-03	285E-02	Rap1b	UP
0419	5657	9920	410E-03	285E-02	Idh1	UP
0400	5640	9917	410E-03	285E-02	Tnfaip1	UP
0739	4779	9908	412E-03	286E-02	Dap	UP
1411	1961	9894	414E-03	287E-02	Pf4	UP
0448	5647	9888	415E-03	287E-02	Mrps10	UP
0264	7029	9881	416E-03	287E-02	Ap1b1	UP
0293	7112	9843	423E-03	290E-02	C1qbp	UP
0236	7596	9838	423E-03	290E-02	Nap1l1	UP
0764	4241	9835	424E-03	290E-02	Mvd	UP
0382	6428	9807	429E-03	293E-02	Ssr4	UP
0516	5991	9779	433E-03	295E-02	Mindy1	UP
0391	5937	9771	435E-03	296E-02	Ngdn	UP
1081	3452	9757	437E-03	297E-02	Adm	UP
0493	4952	9757	437E-03	297E-02	Mknk2	UP

0897	3288	9740	440E-03	299E-02	LOC12009894	UP
0429	5883	9728	442E-03	300E-02	Aprt	UP
0427	5719	9715	444E-03	301E-02	Khdc4	UP
1068	3420	9681	450E-03	303E-02	Spc24	UP
0588	4337	9664	453E-03	305E-02	Gpr19	UP
0658	4449	9660	454E-03	305E-02	Smu1	UP
0410	6032	9653	455E-03	306E-02	Znrf1	UP
0515	5266	9646	457E-03	306E-02	Dpagt1	UP
0633	4521	9644	457E-03	306E-02	Smndc1	UP
0343	6692	9623	461E-03	309E-02	Brd2	UP
0412	6394	9581	469E-03	313E-02	Atic	UP
0335	6218	9565	471E-03	315E-02	Plekho1	UP
0342	5584	9558	473E-03	315E-02	Mrpl40	UP
0538	5820	9547	475E-03	316E-02	Tmem117	UP
0582	4687	9546	475E-03	316E-02	Nup62	UP
0294	7028	9528	478E-03	318E-02	Cct5	UP
0323	6762	9522	480E-03	318E-02	Psmd9	UP
0496	5592	9521	480E-03	318E-02	Ppp1r15b	UP
0466	7226	9511	482E-03	319E-02	Olfm1	UP
0636	5488	9501	484E-03	320E-02	Cyth4	UP
0503	6248	9491	486E-03	320E-02	Fbxl16	UP
0351	6121	9475	489E-03	322E-02	Nop16	UP
0501	4882	9472	489E-03	322E-02	Tlnrd1	UP
0451	9323	9461	491E-03	323E-02	Mag	UP
0483	6114	9461	491E-03	323E-02	Itgb1	UP
0466	6182	9453	493E-03	323E-02	Dnm2	UP
1914	2324	9411	501E-03	328E-02	Kdelr3	UP
0448	5605	9397	504E-03	329E-02	Prkar2a	UP
0348	6322	9388	506E-03	330E-02	Nf2	UP
0330	6806	9384	507E-03	330E-02	LOC10255600	UP
0268	8039	9364	511E-03	332E-02	Sec61g	UP
0332	6486	9341	515E-03	334E-02	Stx4	UP
0388	7148	9291	526E-03	340E-02	LOC10834893	UP

2284	1878	9282	528E-03	341E-02	Ube2c	UP
0391	5482	9269	530E-03	342E-02	Slc9a8	UP
0425	6118	9267	531E-03	342E-02	Lrrn2	UP
0477	5546	9265	531E-03	342E-02	Lrrc75a	UP
0408	5649	9260	532E-03	342E-02	Ube2n	UP
0428	5916	9258	533E-03	342E-02	Ube4b	UP
3490	0208	10208	536E-03	344E-02	Ccl17	UP
0556	4738	9229	539E-03	346E-02	Alyref	UP
0449	5816	9207	544E-03	349E-02	Tjp2	UP
0586	4835	9192	547E-03	350E-02	Polr1h	UP
0545	4776	9168	552E-03	352E-02	Lman2	UP
0364	7017	9166	553E-03	352E-02	Cbr1	UP
0464	5033	9162	554E-03	352E-02	Eif2s1	UP
0321	6740	9148	557E-03	354E-02	Dgcr6	UP
0462	5038	9119	563E-03	358E-02	Faf1	UP
0660	4950	9113	565E-03	358E-02	Ldb1	UP
0370	6003	9102	567E-03	359E-02	Lmbr1l	UP
0355	6749	9092	569E-03	360E-02	Acsl4	UP
0424	6244	9089	570E-03	360E-02	Cd47	UP
0458	5967	9076	573E-03	361E-02	Phc2	UP
0406	5903	9061	577E-03	363E-02	Tmed8	UP
0781	4632	9057	578E-03	363E-02	Cdkn2d	UP
0412	5680	9045	580E-03	364E-02	Tmed4	UP
0250	7654	9044	581E-03	364E-02	Mlf2	UP
0437	4575	9034	583E-03	364E-02	LOC10254824	UP
0822	4528	9003	590E-03	368E-02	Tpd52l1	UP
1040	3524	8999	591E-03	368E-02	Pstpip1	UP
0573	5777	8979	596E-03	370E-02	Twf1	UP
2630	0592	8969	599E-03	371E-02	LOC12009638	UP
0262	7600	8936	606E-03	374E-02	Rab18	UP
0479	4969	8927	609E-03	375E-02	Masp1	UP
0325	6349	8898	616E-03	379E-02	Kars	UP
0602	4800	8867	624E-03	382E-02	Dnase2	UP

3230	0148	9713	633E-03	387E-02	Ccl4	UP
0382	6644	8825	634E-03	387E-02	Pp2d1	UP
0526	7304	8809	639E-03	389E-02	Rhob	UP
0714	3831	8803	640E-03	390E-02	Nrtn	UP
0450	5266	8772	648E-03	394E-02	Rcn1	UP
0367	8318	8769	649E-03	394E-02	Fabp5	UP
0359	5815	8766	650E-03	394E-02	Nckipsd	UP
3540	0211	9607	657E-03	398E-02	LOC12009642	UP
0402	5681	8725	661E-03	399E-02	RGD1305350	UP
0383	6084	8695	669E-03	403E-02	Nutf2	UP
0438	5937	8691	670E-03	403E-02	Eftud2	UP
0263	6438	8673	675E-03	406E-02	Vps29	UP
0614	6246	8667	677E-03	407E-02	Ddit4	UP
0569	5153	8665	677E-03	407E-02	Tm7sf3	UP
0205	8681	8653	681E-03	408E-02	Psma7	UP
0372	6114	8640	684E-03	409E-02	Zfp24	UP
0642	3476	8629	687E-03	410E-02	LOC10036191	UP
0462	5060	8623	689E-03	410E-02	Tbrg4	UP
0250	6975	8613	692E-03	411E-02	Rbm17	UP
0751	4264	8605	694E-03	412E-02	Sbsn	UP
0434	4785	8564	706E-03	417E-02	LOC10255518	UP
0745	6463	8552	709E-03	418E-02	Prkcg	UP
0564	4425	8550	710E-03	418E-02	Eif2b3	UP
0164	10693	8549	710E-03	418E-02	Chchd2	UP
0216	8610	8548	710E-03	418E-02	Rack1	UP
0307	7638	8537	714E-03	419E-02	Cstb	UP
4325	0481	9353	717E-03	421E-02	LOC12010275	UP
0243	8273	8523	718E-03	421E-02	Ppp2r1a	UP
0503	6898	8520	719E-03	421E-02	LOC688754	UP
0343	7035	8517	720E-03	421E-02	Sorbs1	UP
0714	4180	8502	724E-03	422E-02	Zfpl1	UP
0731	5848	8497	726E-03	423E-02	Fam107a	UP
0263	7417	8489	728E-03	424E-02	Dnajc8	UP

0398	5710	8480	731E-03	425E-02	Srrm1	UP
0703	4201	8472	733E-03	426E-02	Amdhd2	UP
0312	6349	8450	740E-03	429E-02	B4gat1	UP
0474	5065	8450	740E-03	429E-02	NEWGENE_13	UP
3421	0203	9260	741E-03	430E-02	LOC12009430	UP
0465	5066	8441	742E-03	430E-02	Fxyd7	UP
0758	3786	8410	752E-03	434E-02	Dnajc18	UP
0557	5340	8374	763E-03	440E-02	LOC12009622	UP
0506	6339	8365	766E-03	440E-02	Cd9	UP
0309	6761	8363	767E-03	440E-02	Rbbp7	UP
0306	8385	8335	776E-03	445E-02	Tmed9	UP
0289	6634	8332	777E-03	445E-02	Trim28	UP
0207	9767	8315	782E-03	446E-02	Rpl5	UP
0433	5874	8267	798E-03	453E-02	Dpysl4	UP
0281	6595	8265	799E-03	453E-02	Atg12	UP
0340	5818	8228	811E-03	459E-02	Mtfr1l	UP
0379	6002	8228	811E-03	459E-02	Manf	UP
0329	7322	8226	812E-03	459E-02	Ap2s1	UP
0597	5191	8219	814E-03	460E-02	Sppl2b	UP
0337	7299	8197	822E-03	462E-02	Syn1	UP
0413	5981	8188	825E-03	463E-02	Zfp36l2	UP
0463	5182	8180	827E-03	463E-02	Mrpl14	UP
0251	8865	8146	839E-03	468E-02	LOC10036005	UP
0464	4895	8140	841E-03	469E-02	Ddx27	UP
0247	7487	8106	853E-03	474E-02	Kdelr1	UP
0446	5388	8053	872E-03	483E-02	Mta2	UP
0229	7382	8028	882E-03	486E-02	Gars	UP
0285	6206	8013	887E-03	488E-02	Rmdn3	UP
0398	6375	8010	888E-03	489E-02	Bag6	UP
0360	6421	7979	900E-03	494E-02	Ski	UP
0375	6606	7977	901E-03	494E-02	Lrsam1	UP
0216	8603	7974	902E-03	494E-02	Sdcbp	UP
0334	6504	7964	905E-03	496E-02	Selenos	UP

0336	6034	7938	916E-03	499E-02	Surf4	UP

Supplementary Table 6: Motifs associated with hyperacetylated peaks in substantia nigra

Motif Name	Consensus	P-value	Log P-value	q-value (Benjamini)	# of Target Sequences with Motif(of 100)	% of Target Sequences with Motif	# of Background Sequences with Motif(of 34854)	% of Background Sequences with Motif
Fra2(bZIP)/Striatum-Fra2-ChIP-Seq(GSE43429)	GGATGACTCATC	1.00E-12	-2.78E+01	0	71	71.00%	12474.2	35.79%
Fosl2(bZIP)/3T3L1-Fosl2-ChIP-Seq(GSE56872)	NATGASTCABNN	1.00E-11	-2.65E+01	0	62	62.00%	9920.2	28.46%
Stat3(Stat)/mES-Stat3-ChIP-Seq(GSE11431)	CTTCCGGGAA	1.00E-11	-2.63E+01	0	77	77.00%	14942.5	42.87%
Fra1(bZIP)/BT549-Fra1-ChIP-Seq(GSE46166)	NNATGASTCATH	1.00E-11	-2.62E+01	0	73	73.00%	13514.3	38.77%
JunB(bZIP)/DendriticCells-Junb-ChIP-Seq(GSE36099)	RATGASTCAT	1.00E-11	-2.54E+01	0	73	73.00%	13723.7	39.37%
Atf3(bZIP)/GBM-ATF3-ChIP-Seq(GSE33912)	DATGASTCATHN	1.00E-10	-2.44E+01	0	77	77.00%	15415.7	44.23%
Stat3+il21(Stat)/CD4-Stat3-ChIP-Seq(GSE19198)	SVYTTCCNGGAARB	1.00E-09	-2.24E+01	0	84	84.00%	18765.1	53.84%
BATF(bZIP)/Th17-BATF-ChIP-Seq(GSE39756)	DATGASTCAT	1.00E-09	-2.21E+01	0	75	75.00%	15282.1	43.84%
PU.1-IRF(ETS:IRF)/Bcell-PU.1-ChIP-Seq(GSE21512)	MGGAAGTGAAAC	1.00E-08	-2.01E+01	0	97	97.00%	26108.2	74.90%
Fos(bZIP)/TSC-Fos-ChIP-Seq(GSE110950)	NDATGASTCAYN	1.00E-08	-1.99E+01	0	70	70.00%	14097.5	40.45%
RUNX(Runt)/HPC7-Runx1-ChIP-Seq(GSE22178)	SAAACCACAG	1.00E-08	-1.93E+01	0	81	81.00%	18417.8	52.84%
AP-1(bZIP)/ThioMac-PU.1-ChIP-Seq(GSE21512)	VTGACTCATC	1.00E-08	-1.91E+01	0	77	77.00%	16910.1	48.51%
MafK(bZIP)/C2C12-MafK-ChIP-Seq(GSE36030)	GCTGASTCAGCA	1.00E-08	-1.85E+01	0	50	50.00%	8241.1	23.64%
Jun-AP1(bZIP)/K562-cJun-ChIP-Seq(GSE31477)	GATGASTCATCN	1.00E-08	-1.84E+01	0	48	48.00%	7693.6	22.07%
GRE(NR),IR3/RAW264.7-GRE-ChIP-Seq(Unpublished)	VAGRACAKWCTGTYC	1.00E-07	-1.82E+01	0	50	50.00%	8301.8	23.82%
MafB(bZIP)/BMM-Mafb-ChIP-Seq(GSE75722)	WNTGCTGASTCAGCANWTTY	1.00E-07	-1.81E+01	0	63	63.00%	12258.7	35.17%
STAT4(Stat)/CD4-Stat4-ChIP-Seq(GSE22104)	NYTTCCWGGAAR	1.00E-07	-1.68E+01	0	85	85.00%	20906.2	59.98%
EBF2(EBF)/BrownAdipose-EBF2-ChIP-Seq(GSE97114)	NABTCCCWDGGGAVH	1.00E-07	-1.66E+01	0	90	90.00%	23278.1	66.78%
Snail1(Zf)/LS174T-SNAIL1.HA-ChIP-Seq(GSE127183)	TRCACCTGCY	1.00E-07	-1.66E+01	0	82	82.00%	19712.2	56.55%
Fox:Ebox(Forkhead,bHLH)/Panc1-Foxa2-ChIP-Seq(GSE47459)	NNNVCTGWGYAAACASN	1.00E-07	-1.65E+01	0	87	87.00%	21920.7	62.89%
ZNF189(Zf)/HEK293-ZNF189.GFP-ChIP-Seq(GSE58341)	TGGAACAGMA	1.00E-07	-1.63E+01	0	85	85.00%	21089.2	60.50%
STAT1(Stat)/HelaS3-STAT1-ChIP-Seq(GSE12782)	NATTTCCNGGAAAT	1.00E-07	-1.62E+01	0	50	50.00%	8796.4	25.24%
RUNX2(Runt)/PCa-RUNX2-ChIP-Seq(GSE33889)	NWAACCACADNN	1.00E-06	-1.60E+01	0	83	83.00%	20317.4	58.29%
PRDM15(Zf)/ESC-Prdm15-ChIP-Seq(GSE73694)	YCCDNTCCAGGTTTT	1.00E-06	-1.58E+01	0	90	90.00%	23551.3	67.57%
STAT5(Stat)/mCD4+-Stat5-ChIP-Seq(GSE12346)	RTTTCTNAGAAA	1.00E-06	-1.57E+01	0	54	54.00%	10115.6	29.02%
Nr5a2(NR)/Pancreas-LRH1-ChIP-Seq(GSE34295)	BTCAAGGTCA	1.00E-06	-1.56E+01	0	82	82.00%	20026.3	57.46%
ELF5(ETS)/T47D-ELF5-ChIP-Seq(GSE30407)	ACVAGGAAGT	1.00E-06	-1.56E+01	0	81	81.00%	19630	56.32%
RORa(NR)/Liver-Rora-ChIP-Seq(GSE101115)	AAWCTAGGTCARDNN	1.00E-06	-1.54E+01	0	36	36.00%	5254.5	15.08%
NF1(CTF)/LNCAP-NF1-ChIP-Seq(Unpublished)	CYTGGCABNSTGCCAR	1.00E-06	-1.53E+01	0	66	66.00%	14103.7	40.46%
Bach2(bZIP)/OCILy7-Bach2-ChIP-Seq(GSE44420)	TGCTGAGTCA	1.00E-06	-1.52E+01	0	42	42.00%	6844.4	19.64%
ERG(ETS)/VCaP-ERG-ChIP-Seq(GSE14097)	ACAGGAAGTG	1.00E-06	-1.46E+01	0	99	99.00%	29217.6	83.82%
Elf4(ETS)/BMDM-Elf4-ChIP-Seq(GSE88699)	ACTTCCKGKT	1.00E-06	-1.45E+01	0	91	91.00%	24486.5	70.25%
ERE(NR),IR3/MCF7-ERa-ChIP-Seq(Unpublished)	VAGGTCACNSTGACC	1.00E-06	-1.42E+01	0	51	51.00%	9637.3	27.65%
AP-2gamma(AP2)/MCF7-TFAP2C-ChIP-Seq(GSE21234)	SCCTSAGGSCAW	1.00E-06	-1.42E+01	0	90	90.00%	24116.4	69.19%
ELF3(ETS)/PDAC-ELF3-ChIP-Seq(GSE64557)	ANCAGGAAGT	1.00E-06	-1.41E+01	0	79	79.00%	19318.2	55.42%
GRE(NR),IR3/A549-GR-ChIP-Seq(GSE32465)	NRGVACABNVTGTYCY	1.00E-06	-1.41E+01	0	35	35.00%	5273.4	15.13%
FoxL2(Forkhead)/Ovary-FoxL2-ChIP-Seq(GSE60858)	WWTRTAAACAVG	1.00E-06	-1.41E+01	0	72	72.00%	16658.3	47.79%
E2F6(E2F)/Hela-E2F6-ChIP-Seq(GSE31477)	GGCGGGAARN	1.00E-05	-1.37E+01	0	63	63.00%	13617.8	39.07%
Nr5a2(NR)/mES-Nr5a2-ChIP-Seq(GSE19019)	BTCAAGGTCA	1.00E-05	-1.37E+01	0	73	73.00%	17162.3	49.24%
EHF(ETS)/LoVo-EHF-ChIP-Seq(GSE49402)	AVCAGGAAGT	1.00E-05	-1.36E+01	0	94	94.00%	26364.2	75.64%
Ap4(bHLH)/AML-Tfap4-ChIP-Seq(GSE45738)	NAHCAGCTGD	1.00E-05	-1.34E+01	0	92	92.00%	25376.7	72.81%
Tbet(T-box)/CD8-Tbet-ChIP-Seq(GSE33802)	AGGTGTGAAM	1.00E-05	-1.33E+01	0	82	82.00%	20849	59.82%
Foxf1(Forkhead)/Lung-Foxf1-ChIP-Seq(GSE77951)	WWATRTAAACAN	1.00E-05	-1.33E+01	0	74	74.00%	17679.5	50.72%
ERRg(NR)/Kidney-ESRRG-ChIP-Seq(GSE104905)	GTGACCTTGRVN	1.00E-05	-1.33E+01	0	82	82.00%	20857.5	59.84%
Slug(Zf)/Mesoderm-Snai2-ChIP-Seq(GSE61475)	SNGCACCTGCHS	1.00E-05	-1.32E+01	0	67	67.00%	15153.2	43.47%
Rbpj1(?)/Panc1-Rbpj1-ChIP-Seq(GSE47459)	HTTTCCCASG	1.00E-05	-1.32E+01	0	96	96.00%	27643.8	79.31%
ZEB1(Zf)/PDAC-ZEB1-ChIP-Seq(GSE64557)	VCAGGTRDRY	1.00E-05	-1.31E+01	0	96	96.00%	27691.5	79.45%
Foxa2(Forkhead)/Liver-Foxa2-ChIP-Seq(GSE25694)	CYTGTTTACWYW	1.00E-05	-1.30E+01	0	73	73.00%	17411	49.95%
Tcf12(bHLH)/GM12878-Tcf12-ChIP-Seq(GSE32465)	VCAGCTGYTG	1.00E-05	-1.30E+01	0	86	86.00%	22700.2	65.13%
Fli1(ETS)/CD8-FLI-ChIP-Seq(GSE20898)	NRYTTCCGGH	1.00E-05	-1.29E+01	0	92	92.00%	25550.3	73.30%

Hoxd12(Homeobox)/ChickenMSG-Hoxd12.Flag-ChIP-Seq(GSE86088)	HDGYAATGAAAN	1.00E-05	-1.29E+01	0	91	91.00%	25059.2	71.89%
Etv2(ETS)/ES-ER71-ChIP-Seq(GSE59402)	NNAYTTCCTGHN	1.00E-05	-1.29E+01	0	87	87.00%	23189.7	66.53%
ZNF467(Zf)/HEK293-ZNF467.GFP-ChIP-Seq(GSE58341)	TGGGGAAGGGCM	1.00E-05	-1.28E+01	0	87	87.00%	23204.8	66.57%
Gfi1b(Zf)/HPC7-Gfi1b-ChIP-Seq(GSE22178)	MAATCACTGC	1.00E-05	-1.28E+01	0	68	68.00%	15676.1	44.97%
NFY(CCAAT)/Promoter	RGCCAATSRG	1.00E-05	-1.27E+01	0	73	73.00%	17524.5	50.28%
GATA3(Zf),DR8/iTreg-Gata3-ChIP-Seq(GSE20898)	AGATSTNDNNDSAGATAASN	1.00E-05	-1.27E+01	0	21	21.00%	2364.4	6.78%
GSC(Homeobox)/FrogEmbryos-GSC-ChIP-Seq(DRA000576)	RGGATTAR	1.00E-05	-1.27E+01	0	83	83.00%	21506.3	61.70%
PGR(NR)/EndoStromal-PGR-ChIP-Seq(GSE69539)	AAGAACATWHTGTTC	1.00E-05	-1.27E+01	0	42	42.00%	7488	21.48%
Zic(Zf)/Cerebellum-ZIC1.2-ChIP-Seq(GSE60731)	CCTGCTGAGH	1.00E-05	-1.26E+01	0	82	82.00%	21109.1	60.56%
MyoD(bHLH)/Myotube-MyoD-ChIP-Seq(GSE21614)	RRCAGCTGYTSY	1.00E-05	-1.25E+01	0	80	80.00%	20318.9	58.29%
ZNF341(Zf)/EBV-ZNF341-ChIP-Seq(GSE113194)	GGAACAGCCG	1.00E-05	-1.25E+01	0	78	78.00%	19537.4	56.05%
STAT6(Stat)/CD4-Stat6-ChIP-Seq(GSE22104)	ABTTCYYRRGAA	1.00E-05	-1.24E+01	0	61	61.00%	13397	38.44%
EWS:ERG-fusion(ETS)/CADO_ES1-EWS:ERG-ChIP-Seq(SRA014231)	ATTTCCTGTN	1.00E-05	-1.23E+01	0	75	75.00%	18435.2	52.89%
Tcf21(bHLH)/ArterySmoothMuscle-Tcf21-ChIP-Seq(GSE61369)	NAACAGCTGG	1.00E-05	-1.22E+01	0	85	85.00%	22535.5	64.65%
Tbx6(T-box)/ESC-Tbx6-ChIP-Seq(GSE93524)	DAGGTGTBAA	1.00E-05	-1.21E+01	0	84	84.00%	22141.7	63.52%
E2A(bHLH)/proBcell-E2A-ChIP-Seq(GSE21978)	DNRCAGCTGY	1.00E-05	-1.21E+01	0	94	94.00%	26897.3	77.17%
Foxo3(Forkhead)/U2OS-Foxo3-ChIP-Seq(E-MTAB-2701)	DGTAAACA	1.00E-05	-1.21E+01	0	68	68.00%	15925.1	45.69%
Barx1(Homeobox)/Stomach-Barx1.3xFlag-ChIP-Seq(GSE69483)	AAACMATTAN	1.00E-05	-1.21E+01	0	51	51.00%	10308	29.57%
Six2(Homeobox)/NephronProgenitor-Six2-ChIP-Seq(GSE39837)	GWAAYHTGAKMC	1.00E-05	-1.21E+01	0	79	79.00%	20098.6	57.66%
PSE(SNAPc)/K562-mStart-Seq	WAVTCACCMTAASYDAAAAG	1.00E-05	-1.20E+01	0	58	58.00%	12563.2	36.04%
PU.1(ETS)/ThioMac-PU.1-ChIP-Seq(GSE21512)	AGAGGAAGTG	1.00E-05	-1.19E+01	0	68	68.00%	15994.3	45.89%
AP-2alpha(AP2)/Hela-AP2alpha-ChIP-Seq(GSE31477)	ATGCCCTGAGGC	1.00E-05	-1.18E+01	0	81	81.00%	20993.9	60.23%
Ascl2(bHLH)/ESC-Ascl2-ChIP-Seq(GSE97712)	SSRGCAGCTGCH	1.00E-05	-1.18E+01	0	88	88.00%	24060.2	69.03%
ARE(NR)/LNCAP-AR-ChIP-Seq(GSE27824)	RGRACASNSTGTYCYB	1.00E-05	-1.16E+01	0.0001	50	50.00%	10159	29.15%
E2F1(E2F)/Hela-E2F1-ChIP-Seq(GSE22478)	CWGGCGGGAA	1.00E-05	-1.15E+01	0.0001	36	36.00%	6159.9	17.67%
BHLHA15(bHLH)/NIH3T3-BHLHB8.HA-ChIP-Seq(GSE119782)	NAMCAGCTGK	1.00E-04	-1.14E+01	0.0001	93	93.00%	26619.9	76.37%
Smad2(MAD)/ES-SMAD2-ChIP-Seq(GSE29422)	CTGTCTGG	1.00E-04	-1.13E+01	0.0001	97	97.00%	28924.8	82.98%
PPARE(NR),DR1/3T3L1-Pparg-ChIP-Seq(GSE13511)	TGACCTTTGCCCCA	1.00E-04	-1.12E+01	0.0001	85	85.00%	22946.8	65.83%
RAR:RXR(NR),DR0/ES-RAR-ChIP-Seq(GSE56893)	AGGTCAAGGTCA	1.00E-04	-1.11E+01	0.0001	38	38.00%	6818.9	19.56%
SpiB(ETS)/OCILY3-SPIB-ChIP-Seq(GSE56857)	AAAGRGGAAGTG	1.00E-04	-1.11E+01	0.0001	44	44.00%	8521.3	24.45%
HEB(bHLH)/mES-Heb-ChIP-Seq(GSE53233)	VCAGCTGBNN	1.00E-04	-1.11E+01	0.0001	98	98.00%	29631.9	85.01%
Maz(Zf)/HepG2-Maz-ChIP-Seq(GSE31477)	GGGGGGGG	1.00E-04	-1.11E+01	0.0001	93	93.00%	26754.1	76.76%
Rfx6(HTH)/Min6b1-Rfx6.HA-ChIP-Seq(GSE62844)	TGTTKCCTAGCAACM	1.00E-04	-1.10E+01	0.0001	89	89.00%	24828.8	71.23%
Atf7(bZIP)/3T3L1-Atf7-ChIP-Seq(GSE56872)	NGRTGACGTCAY	1.00E-04	-1.09E+01	0.0001	56	56.00%	12310	35.32%
MafA(bZIP)/Islet-MafA-ChIP-Seq(GSE30298)	TGCTGACTCA	1.00E-04	-1.08E+01	0.0001	78	78.00%	20182.9	57.90%
E2F4(E2F)/K562-E2F4-ChIP-Seq(GSE31477)	GGCGGGAAAH	1.00E-04	-1.08E+01	0.0001	51	51.00%	10731.5	30.79%
Zic3(Zf)/mES-Zic3-ChIP-Seq(GSE37889)	GGCCYCCTGCTGDGH	1.00E-04	-1.08E+01	0.0001	71	71.00%	17529.3	50.29%
MyoG(bHLH)/C2C12-MyoG-ChIP-Seq(GSE36024)	AACAGCTG	1.00E-04	-1.07E+01	0.0001	86	86.00%	23571.2	67.63%
Bcl6(Zf)/Liver-Bcl6-ChIP-Seq(GSE31578)	NNNCTTTCCAGGAAA	1.00E-04	-1.07E+01	0.0001	91	91.00%	25890	74.28%
Tbx21(T-box)/GM12878-TBX21-ChIP-Seq(Encode)	AGGTGTGAAA	1.00E-04	-1.07E+01	0.0001	78	78.00%	20247	58.09%
PRDM1(Zf)/Hela-PRDM1-ChIP-Seq(GSE31477)	ACTTTCACTTTC	1.00E-04	-1.07E+01	0.0001	61	61.00%	14040.8	40.28%
NF-E2(bZIP)/K562-NFE2-ChIP-Seq(GSE31477)	GATGACTCAGCA	1.00E-04	-1.06E+01	0.0001	19	19.00%	2280.1	6.54%
NeuroD1(bHLH)/Islet-NeuroD1-ChIP-Seq(GSE30298)	GCCATCTGTT	1.00E-04	-1.06E+01	0.0001	79	79.00%	20680.4	59.33%
Ascl1(bHLH)/NeuralTubes-Ascl1-ChIP-Seq(GSE55840)	NNVVCAGCTGBN	1.00E-04	-1.06E+01	0.0001	95	95.00%	27997.8	80.33%
ETV1(ETS)/GIST48-ETV1-ChIP-Seq(GSE22441)	AACCGGAAGT	1.00E-04	-1.05E+01	0.0001	95	95.00%	28013.3	80.37%
LEF1(HMG)/H1-LEF1-ChIP-Seq(GSE64758)	CCTTTGATST	1.00E-04	-1.05E+01	0.0001	66	66.00%	15830.2	45.42%
ETS1(ETS)/Jurkat-ETS1-ChIP-Seq(GSE17954)	ACAGGAAGTG	1.00E-04	-1.05E+01	0.0001	88	88.00%	24544	70.42%
CEBP:AP1(bZIP)/ThioMac-CEBPb-ChIP-Seq(GSE21512)	DRTGTTGCAA	1.00E-04	-1.05E+01	0.0001	64	64.00%	15138.5	43.43%
Hand2(bHLH)/Mesoderm-Hand2-ChIP-Seq(GSE61475)	TGACANARRCCAGRC	1.00E-04	-1.05E+01	0.0001	64	64.00%	15140.9	43.44%
CEBP(bZIP)/ThioMac-CEBPb-ChIP-Seq(GSE21512)	ATTGCGCAAC	1.00E-04	-1.05E+01	0.0001	57	57.00%	12784.6	36.68%
TCF4(bHLH)/SHSY5Y-TCF4-ChIP-Seq(GSE96915)	SMCATCTGKH	1.00E-04	-1.05E+01	0.0001	94	94.00%	27504.1	78.91%
E2F7(E2F)/Hela-E2F7-ChIP-Seq(GSE32673)	VDTTTCCCGCCA	1.00E-04	-1.04E+01	0.0001	24	24.00%	3437.2	9.86%
PBX2(Homeobox)/K562-PBX2-ChIP-Seq(Encode)	RTGATTKATRGN	1.00E-04	-1.03E+01	0.0001	65	65.00%	15551.8	44.62%
ZNF415(Zf)/HEK293-ZNF415.GFP-ChIP-Seq(GSE58341)	GRTGMTRGAGCC	1.00E-04	-1.03E+01	0.0001	65	65.00%	15580.3	44.70%

E2A(bHLH),near_PU.1/Bcell-PU.1-ChIP-Seq(GSE21512)	NVCACCTGBN	1.00E-04	-1.03E+01	0.0001	90	90.00%	25584.4	73.40%
SF1(NR)/H295R-Nr5a1-ChIP-Seq(GSE44220)	CAAGGHCANV	1.00E-04	-1.02E+01	0.0002	65	65.00%	15607.5	44.78%
FXR(NR),IR1/Liver-FXR-ChIP-Seq(Chong_et_al.)	AGGTCANTGACCTB	1.00E-04	-1.02E+01	0.0002	56	56.00%	12558.2	36.03%
ELF1(ETS)/Jurkat-ELF1-ChIP-Seq(SRA014231)	AVCCGGAAGT	1.00E-04	-1.01E+01	0.0002	68	68.00%	16738	48.02%
TEAD3(TEA)/HepG2-TEAD3-ChIP-Seq(Encode)	TRCATTCCAG	1.00E-04	-1.00E+01	0.0002	81	81.00%	21728.1	62.34%
MafF(bZIP)/HepG2-MafF-ChIP-Seq(GSE31477)	HWWGTCAGCAWWTTT	1.00E-04	-1.00E+01	0.0002	34	34.00%	6043.3	17.34%
PRDM10(Zf)/HEK293-PRDM10.eGFP-ChIP-Seq(Encode)	TGGTACATTCCA	1.00E-04	-9.86E+00	0.0002	69	69.00%	17184.4	49.30%
EBF1(EBF)/Near-E2A-ChIP-Seq(GSE21512)	GTCCCCWGGGGA	1.00E-04	-9.81E+00	0.0002	88	88.00%	24836.8	71.26%
TATA-Box(TBP)/Promoter	CCTTTTAWAGSC	1.00E-04	-9.80E+00	0.0002	85	85.00%	23511.5	67.45%
SPDEF(ETS)/VCaP-SPDEF-ChIP-Seq(SRA014231)	ASWTCCTGBT	1.00E-04	-9.80E+00	0.0002	86	86.00%	23947.3	68.70%
PPARa(NR),DR1/Liver-Ppara-ChIP-Seq(GSE47954)	VNAGGKCAAAGGTCA	1.00E-04	-9.71E+00	0.0002	86	86.00%	23987.5	68.82%
Zic2(Zf)/ESC-Zic2-ChIP-Seq(SRP197560)	CHCAGCRGGRGG	1.00E-04	-9.67E+00	0.0002	62	62.00%	14787.2	42.42%
ZNF322(Zf)/HEK293-ZNF322.GFP-ChIP-Seq(GSE58341)	GAGCCTGGTACTGWGCCTGR	1.00E-04	-9.63E+00	0.0002	51	51.00%	11172.8	32.05%
PRDM14(Zf)/H1-PRDM14-ChIP-Seq(GSE22767)	RGGTCTCTAACY	1.00E-04	-9.62E+00	0.0002	49	49.00%	10548.4	30.26%
FOXP1(Forkhead)/H9-FOXP1-ChIP-Seq(GSE31006)	NYYTGTTTACHN	1.00E-04	-9.58E+00	0.0003	51	51.00%	11194	32.12%
RARg(NR)/ES-RARg-ChIP-Seq(GSE30538)	AGGTCAAGGTCA	1.00E-04	-9.52E+00	0.0003	23	23.00%	3385.4	9.71%
CRE(bZIP)/Promoter	CSGTGACGTCAC	1.00E-04	-9.52E+00	0.0003	36	36.00%	6748	19.36%
MITF(bHLH)/MastCells-MITF-ChIP-Seq(GSE48085)	RTCATGTGAC	1.00E-04	-9.47E+00	0.0003	78	78.00%	20768.9	59.59%
RXR(NR),DR1/3T3L1-RXR-ChIP-Seq(GSE13511)	TAGGGCAAAGGTCA	1.00E-04	-9.46E+00	0.0003	88	88.00%	24981	71.67%
Tbr1(T-box)/Cortex-Tbr1-ChIP-Seq(GSE71384)	AAGGTGTKAA	1.00E-04	-9.37E+00	0.0003	86	86.00%	24131.3	69.23%
Pit1(Homeobox)/GCrat-Pit1-ChIP-Seq(GSE58009)	ATGMATATDC	1.00E-04	-9.35E+00	0.0003	65	65.00%	15971	45.82%
Atoh1(bHLH)/Cerebellum-Atoh1-ChIP-Seq(GSE22111)	VNRVCAGCTGGY	1.00E-04	-9.31E+00	0.0003	86	86.00%	24158.9	69.31%
Elk4(ETS)/Hela-Elk4-ChIP-Seq(GSE31477)	NRYTTCCGGY	1.00E-04	-9.30E+00	0.0003	69	69.00%	17430	50.01%
Max(bHLH)/K562-Max-ChIP-Seq(GSE31477)	RCCACGTGGYYN	1.00E-04	-9.27E+00	0.0003	67	67.00%	16717.9	47.96%
Zfp809(Zf)/ES-Zfp809-ChIP-Seq(GSE70799)	GGGGCTYGKCTGGGA	1.00E-04	-9.25E+00	0.0003	40	40.00%	7972.3	22.87%
ZEB2(Zf)/SNU398-ZEB2-ChIP-Seq(GSE103048)	GNMCAGGTGTGC	1.00E-04	-9.24E+00	0.0003	79	79.00%	21267.8	61.02%
ZNF416(Zf)/HEK293-ZNF416.GFP-ChIP-Seq(GSE58341)	WDNCTGGGCA	1.00E-04	-9.24E+00	0.0003	93	93.00%	27457.1	78.77%
THRb(NR)/HepG2-THRb.Flag-ChIP-Seq(Encode)	GGTCACCTGAGGTCA	1.00E-04	-9.23E+00	0.0003	68	68.00%	17094.7	49.04%
Bach1(bZIP)/K562-Bach1-ChIP-Seq(GSE31477)	AWWNTGCTGAGTCAT	1.00E-04	-9.22E+00	0.0003	17	17.00%	2103.5	6.03%
Nrf2(bZIP)/Lymphoblast-Nrf2-ChIP-Seq(GSE37589)	HTGCTGAGTCAT	1.00E-03	-9.15E+00	0.0003	16	16.00%	1906.9	5.47%
PR(NR)/T47D-PR-ChIP-Seq(GSE31130)	VAGRACAKNCTGTBC	1.00E-03	-9.06E+00	0.0004	99	99.00%	31021	89.00%
Sp5(Zf)/mES-Sp5.Flag-ChIP-Seq(GSE72989)	RGKGGGCGGAGC	1.00E-03	-9.03E+00	0.0004	85	85.00%	23847.1	68.42%
FOXK1(Forkhead)/HEK293-FOXK1-ChIP-Seq(GSE51673)	NVWTGTTTAC	1.00E-03	-9.01E+00	0.0004	75	75.00%	19803.8	56.82%
p63(p53)/Keratinocyte-p63-ChIP-Seq(GSE17611)	NNDRCATGYCYNRRCATGYH	1.00E-03	-8.95E+00	0.0004	50	50.00%	11123.3	31.91%
Hoxd13(Homeobox)/ChickenMSG-Hoxd13.Flag-ChIP-Seq(GSE86088)	NCYAATAAAA	1.00E-03	-8.83E+00	0.0005	84	84.00%	23509.9	67.45%
Hoxc9(Homeobox)/Ainv15-Hoxc9-ChIP-Seq(GSE21812)	GGCCATAAATCA	1.00E-03	-8.83E+00	0.0005	48	48.00%	10545.7	30.26%
MYB(HTH)/ERMYB-Myb-ChIPSeq(GSE22095)	GGCVGTTR	1.00E-03	-8.80E+00	0.0005	94	94.00%	28144.3	80.75%
GLIS3(Zf)/Thyroid-Glis3.GFP-ChIP-Seq(GSE103297)	CTCCCTGGGAGGCCN	1.00E-03	-8.79E+00	0.0005	93	93.00%	27638.6	79.29%
NeuroG2(bHLH)/Fibroblast-NeuroG2-ChIP-Seq(GSE75910)	ACCATCTGTT	1.00E-03	-8.78E+00	0.0005	93	93.00%	27643.3	79.31%
Ets1-distal(ETS)/CD4+-PolII-ChIP-Seq(Barski_et_al.)	MACAGGAAGT	1.00E-03	-8.78E+00	0.0005	50	50.00%	11195.2	32.12%
Myf5(bHLH)/GM-Myf5-ChIP-Seq(GSE24852)	BAACAGCTGT	1.00E-03	-8.74E+00	0.0005	72	72.00%	18789.5	53.91%
Zfp281(Zf)/ES-Zfp281-ChIP-Seq(GSE81042)	CCCCTCCCCCAC	1.00E-03	-8.69E+00	0.0005	51	51.00%	11549.2	33.13%
ZNF652/HepG2-ZNF652.Flag-ChIP-Seq(Encode)	TTAACCCTTTVNKKN	1.00E-03	-8.56E+00	0.0006	37	37.00%	7346.6	21.08%
Dlx3(Homeobox)/Kerainocytes-Dlx3-ChIP-Seq(GSE89884)	NDGTAATTAC	1.00E-03	-8.54E+00	0.0006	53	53.00%	12255.3	35.16%
ETV4(ETS)/HepG2-ETV4-ChIP-Seq(ENCODE)	ACCGGAAGTG	1.00E-03	-8.51E+00	0.0006	89	89.00%	25845.9	74.15%
WT1(Zf)/Kidney-WT1-ChIP-Seq(GSE90016)	MCTCCCMCRCAB	1.00E-03	-8.49E+00	0.0006	71	71.00%	18532.9	53.17%
KLF14(Zf)/HEK293-KLF14.GFP-ChIP-Seq(GSE58341)	RGKGGGCGKGGC	1.00E-03	-8.49E+00	0.0006	96	96.00%	29344.2	84.19%
THRa(NR)/C17.2-THRa-ChIP-Seq(GSE38347)	GGTCANYTGAGGWCA	1.00E-03	-8.48E+00	0.0006	57	57.00%	13604.5	39.03%
Mef2b(MADS)/HEK293-Mef2b.V5-ChIP-Seq(GSE67450)	GCTATTTTTGGM	1.00E-03	-8.48E+00	0.0006	65	65.00%	16361	46.94%
ZBTB18(Zf)/HEK293-ZBTB18.GFP-ChIP-Seq(GSE58341)	AACATCTGGA	1.00E-03	-8.47E+00	0.0006	61	61.00%	14965.6	42.94%
GRHL2(CP2)/HBE-GRHL2-ChIP-Seq(GSE46194)	AAACYKGTTWDACMRGTTTB	1.00E-03	-8.45E+00	0.0006	48	48.00%	10697.9	30.69%
NFkB-p65(RHD)/GM12787-p65-ChIP-Seq(GSE19485)	WGGGGATTTCCC	1.00E-03	-8.37E+00	0.0007	59	59.00%	14327.4	41.11%
IRF8(IRF)/BMDM-IRF8-ChIP-Seq(GSE77884)	GRAASTGAAAST	1.00E-03	-8.33E+00	0.0007	45	45.00%	9812	28.15%
Foxh1(Forkhead)/hESC-FOXH1-ChIP-Seq(GSE29422)	NNTGTGGATTSS	1.00E-03	-8.30E+00	0.0007	57	57.00%	13682.6	39.26%

COUP-TFII(NR)/Artia-Nr2f2-ChIP-Seq(GSE46497)	AGRGGTCA	1.00E-03	-8.28E+00	0.0007	95	95.00%	28880.7	82.86%
GABPA(ETS)/Jurkat-GABPa-ChIP-Seq(GSE17954)	RACCGGAAGT	1.00E-03	-8.24E+00	0.0007	83	83.00%	23357.1	67.01%
Erra(NR)/HepG2-Erra-ChIP-Seq(GSE31477)	CAAAGGTCAG	1.00E-03	-8.22E+00	0.0007	99	99.00%	31309.9	89.83%
HOXB13(Homeobox)/ProstateTumor-HOXB13-ChIP-Seq(GSE56288)	TTTTATKRGG	1.00E-03	-8.20E+00	0.0007	72	72.00%	19040.3	54.63%
Arnt:Ahr(bHLH)/MCF7-Arnt-ChIP-Seq(Lo_et_al.)	TBGCACGCAA	1.00E-03	-8.19E+00	0.0008	64	64.00%	16141.1	46.31%
RUNX-AML(Runt)/CD4+-PolII-ChIP-Seq(Barski_et_al.)	GCTGTGGTTW	1.00E-03	-8.16E+00	0.0008	71	71.00%	18685.2	53.61%
IRF3(IRF)/BMDM-Irf3-ChIP-Seq(GSE67343)	AGTTTCAKTTTC	1.00E-03	-8.07E+00	0.0008	43	43.00%	9305.6	26.70%
Unknown-ESC-element(?)/mES-Nanog-ChIP-Seq(GSE11724)	CACAGCAGGGGG	1.00E-03	-8.07E+00	0.0008	68	68.00%	17630	50.58%
AMYB(HTH)/Testes-AMYB-ChIP-Seq(GSE44588)	TGGCAGTTGG	1.00E-03	-8.00E+00	0.0009	91	91.00%	26995.8	77.45%
Atf1(bZIP)/K562-ATF1-ChIP-Seq(GSE31477)	GATGACGTCA	1.00E-03	-7.97E+00	0.0009	62	62.00%	15541.5	44.59%
EWS:FLI1-fusion(ETS)/SK_N_MC-EWS:FLI1-ChIP-Seq(SRA014231)	VACAGGAAAT	1.00E-03	-7.96E+00	0.0009	67	67.00%	17319.7	49.69%
NFE2L2(bZIP)/HepG2-NFE2L2-ChIP-Seq(Encode)	AWWWTGCTGAGTCAT	1.00E-03	-7.84E+00	0.001	15	15.00%	1925.6	5.52%
ZNF7(Zf)/HepG2-ZNF7.Flag-ChIP-Seq(Encode)	CTGCCWVCTTTTRTA	1.00E-03	-7.83E+00	0.001	57	57.00%	13896.2	39.87%
STAT6(Stat)/Macrophage-Stat6-ChIP-Seq(GSE38377)	TTCCKNAGAA	1.00E-03	-7.82E+00	0.001	57	57.00%	13904.1	39.89%
EKLF(Zf)/Erythrocyte-Klf1-ChIP-Seq(GSE20478)	NWGGGTGTGGCY	1.00E-03	-7.69E+00	0.0012	39	39.00%	8252.9	23.68%
ZBTB12(Zf)/HEK293-ZBTB12.GFP-ChIP-Seq(GSE58341)	NGNTCTAGAACCNGV	1.00E-03	-7.57E+00	0.0013	58	58.00%	14358.5	41.19%
ETS(ETS)/Promoter	AACCGGAAGT	1.00E-03	-7.54E+00	0.0013	51	51.00%	12049.9	34.57%
FoxD3(forkhead)/ZebrafishEmbryo-Foxd3.biotin-ChIP-seq	TGTTTAYTTAGC	1.00E-03	-7.53E+00	0.0013	69	69.00%	18255.6	52.37%
FOXK2(Forkhead)/U2OS-FOXK2-ChIP-Seq(E-MTAB-2204)	SCHTGTTTACAT	1.00E-03	-7.45E+00	0.0014	60	60.00%	15101	43.32%
Foxo1(Forkhead)/RAW-Foxo1-ChIP-Seq(Fan_et_al.)	CTGTTTAC	1.00E-03	-7.44E+00	0.0014	95	95.00%	29217.7	83.83%
Nkx3.1(Homeobox)/LNCaP-Nkx3.1-ChIP-Seq(GSE28264)	AAGCACTTAA	1.00E-03	-7.38E+00	0.0015	98	98.00%	30942.9	88.77%
n-Myc(bHLH)/mES-nMyc-ChIP-Seq(GSE11431)	VRCCACGTGG	1.00E-03	-7.36E+00	0.0016	66	66.00%	17255.9	49.51%
Hoxa11(Homeobox)/ChickenMSG-Hoxa11.Flag-ChIP-Seq(GSE86088)	TTTTATGGCM	1.00E-03	-7.33E+00	0.0016	93	93.00%	28252.3	81.06%
Pdx1(Homeobox)/Islet-Pdx1-ChIP-Seq(SRA008281)	YCATYAATCA	1.00E-03	-7.32E+00	0.0016	65	65.00%	16918.2	48.54%
Sox21(HMG)/ESC-SOX21-ChIP-Seq(GSE110505)	BCCWTTGTBYKV	1.00E-03	-7.31E+00	0.0016	93	93.00%	28259.9	81.08%
TEAD2(TEA)/Py2T-Tead2-ChIP-Seq(GSE55709)	CCWGGAATGY	1.00E-03	-7.31E+00	0.0016	55	55.00%	13474.9	38.66%
Rfx5(HTH)/GM12878-Rfx5-ChIP-Seq(GSE31477)	SCCTAGCAACAG	1.00E-03	-7.29E+00	0.0016	48	48.00%	11195	32.12%
Isl1(Homeobox)/Neuron-Isl1-ChIP-Seq(GSE31456)	CTAATKGV	1.00E-03	-7.24E+00	0.0017	92	92.00%	27805.7	79.77%
ZNF519(Zf)/HEK293-ZNF519.GFP-ChIP-Seq(GSE58341)	GAGSCCGAGC	1.00E-03	-7.20E+00	0.0018	36	36.00%	7559	21.69%
Sox2(HMG)/mES-Sox2-ChIP-Seq(GSE11431)	BCCATTGTTC	1.00E-03	-7.19E+00	0.0018	73	73.00%	19915.3	57.14%
CLOCK(bHLH)/Liver-Clock-ChIP-Seq(GSE39860)	GHCACGTG	1.00E-03	-7.18E+00	0.0018	57	57.00%	14210.7	40.77%
COUP-TFII(NR)/K562-NR2F1-ChIP-Seq(Encode)	GKBCARAGGTCA	1.00E-03	-7.16E+00	0.0018	91	91.00%	27370.6	78.53%
RORg(NR)/Liver-Rorc-ChIP-Seq(GSE101115)	WAABTAGGTCAV	1.00E-03	-7.12E+00	0.0019	21	21.00%	3486.7	10.00%
Smad4(MAD)/ESC-SMAD4-ChIP-Seq(GSE29422)	VBSYGTCTGG	1.00E-03	-7.10E+00	0.0019	95	95.00%	29361.6	84.24%
PU.1:IRF8(ETS:IRF)/pDC-Irf8-ChIP-Seq(GSE66899)	GGAAGTGAAAST	1.00E-03	-7.04E+00	0.002	33	33.00%	6750.5	19.37%
Otx2(Homeobox)/EpiLC-Otx2-ChIP-Seq(GSE56098)	NYTAATCCYB	1.00E-03	-7.03E+00	0.002	66	66.00%	17420.5	49.98%
ETS:RUNX(ETS,Runt)/Jurkat-RUNX1-ChIP-Seq(GSE17954)	RCAGGATGTGGT	1.00E-03	-7.02E+00	0.002	23	23.00%	4021.6	11.54%
Twist2(bHLH)/Myoblast-Twist2.Ty1-ChIP-Seq(GSE127998)	MCAGCTGBYH	1.00E-03	-7.01E+00	0.002	94	94.00%	28887.3	82.88%
DLX5(Homeobox)/BasalGanglia-Dlx5-ChIP-seq(GSE124936)	SSTAATTA	1.00E-03	-7.00E+00	0.002	56	56.00%	13959.5	40.05%
HNF4a(NR),DR1/HepG2-HNF4a-ChIP-Seq(GSE25021)	CARRGKBCAAAGTYCA	1.00E-03	-6.97E+00	0.0021	58	58.00%	14653.4	42.04%
HIF2a(bHLH)/785_O-HIF2a-ChIP-Seq(GSE34871)	GCACGTACCC	1.00E-03	-6.96E+00	0.0021	43	43.00%	9769.5	28.03%
Six1(Homeobox)/Myoblast-Six1-ChIP-Chip(GSE20150)	GKVTCADRTTWC	1.00E-03	-6.94E+00	0.0021	33	33.00%	6785.8	19.47%
IRF4(IRF)/GM12878-IRF4-ChIP-Seq(GSE32465)	ACTGAAACCA	1.00E-02	-6.81E+00	0.0024	47	47.00%	11096	31.83%
Sp2(Zf)/HEK293-Sp2.eGFP-ChIP-Seq(Encode)	YGGCCCCGCCCC	1.00E-02	-6.79E+00	0.0025	92	92.00%	28010.6	80.36%
CArG(MADS)/PUER-Srf-ChIP-Seq(Sullivan_et_al.)	CCATATATGGNM	1.00E-02	-6.78E+00	0.0025	39	39.00%	8625.5	24.75%
Olig2(bHLH)/Neuron-Olig2-ChIP-Seq(GSE30882)	RCCATMTGTT	1.00E-02	-6.76E+00	0.0025	95	95.00%	29504.5	84.65%
TEAD4(TEA)/Tropoblast-Tead4-ChIP-Seq(GSE37350)	CCWGGAATGY	1.00E-02	-6.74E+00	0.0026	70	70.00%	19033	54.61%
ZNF692(Zf)/HEK293-ZNF692.GFP-ChIP-Seq(GSE58341)	GTGGGCCCCA	1.00E-02	-6.72E+00	0.0026	28	28.00%	5456.8	15.66%
CHR(?)/Hela-CellCycle-Expression	SRGTTTCAAA	1.00E-02	-6.69E+00	0.0027	53	53.00%	13112.9	37.62%
Tcf7(HMG)/GM12878-TCF7-ChIP-Seq(Encode)	CTTTGATGTGSB	1.00E-02	-6.66E+00	0.0027	41	41.00%	9286.8	26.64%
Sox6(HMG)/Myotubes-Sox6-ChIP-Seq(GSE32627)	CCATTGTTNY	1.00E-02	-6.66E+00	0.0027	87	87.00%	25816	74.07%
DMRT1(DM)/Testis-DMRT1-ChIP-Seq(GSE64892)	TWGHWACAWTGTWDC	1.00E-02	-6.64E+00	0.0027	31	31.00%	6328.8	18.16%
BMAL1(bHLH)/Liver-Bmal1-ChIP-Seq(GSE39860)	GNCACGTG	1.00E-02	-6.64E+00	0.0027	91	91.00%	27609	79.21%
NFkB-p50,p52(RHD)/Monocyte-p50-ChIP-Chip(Schreiber_et_al.)	GGGGGAATCCCC	1.00E-02	-6.61E+00	0.0028	23	23.00%	4145.2	11.89%

Lhx3(Homeobox)/Neuron-Lhx3-ChIP-Seq(GSE31456)	ADBTAATTAR	1.00E-02	-6.60E+00	0.0028	81	81.00%	23342.8	66.97%
CRX(Homeobox)/Retina-Crx-ChIP-Seq(GSE20012)	GCTAATCC	1.00E-02	-6.53E+00	0.003	96	96.00%	30127.3	86.43%
EAR2(NR)/K562-NR2F6-ChIP-Seq(Encode)	NRBCARRGGTCA	1.00E-02	-6.48E+00	0.0031	88	88.00%	26338.5	75.56%
DLX1(Homeobox)/BasalGanglia-Dlx1-ChIP-seq(GSE124936)	NSNNTAATTA	1.00E-02	-6.39E+00	0.0034	74	74.00%	20719	59.44%
Zac1(Zf)/Neuro2A-Plagl1-ChIP-Seq(GSE75942)	HAWGRGGCCM	1.00E-02	-6.38E+00	0.0034	100	100.00%	32700.2	93.82%
Tcf3(HMG)/mES-Tcf3-ChIP-Seq(GSE11724)	ASWTCAAAGG	1.00E-02	-6.35E+00	0.0035	35	35.00%	7611	21.84%
Tgif1(Homeobox)/mES-Tgif1-ChIP-Seq(GSE55404)	YTGWCADY	1.00E-02	-6.28E+00	0.0037	100	100.00%	32732.7	93.91%
Gata4(Zf)/Heart-Gata4-ChIP-Seq(GSE35151)	NBWGATAAGR	1.00E-02	-6.25E+00	0.0038	69	69.00%	18925.4	54.30%
KLF6(Zf)/PDAC-KLF6-ChIP-Seq(GSE64557)	MKGGGYGTGGCC	1.00E-02	-6.23E+00	0.0039	82	82.00%	23939	68.68%
KLF10(Zf)/HEK293-KLF10.GFP-ChIP-Seq(GSE58341)	GGGGGTGTGTCC	1.00E-02	-6.23E+00	0.0039	69	69.00%	18939.6	54.34%
LRF(Zf)/Erythroblasts-ZBTB7A-ChIP-Seq(GSE74977)	AAGACCCYYN	1.00E-02	-6.21E+00	0.004	89	89.00%	26913.5	77.21%
GATA3(Zf)/iTreg-Gata3-ChIP-Seq(GSE20898)	AGATAASR	1.00E-02	-6.20E+00	0.004	81	81.00%	23552.7	67.57%
DMRT6(DM)/Testis-DMRT6-ChIP-Seq(GSE60440)	YDGHTACAWTGTADC	1.00E-02	-6.15E+00	0.0042	28	28.00%	5666.4	16.26%
ZNF264(Zf)/HEK293-ZNF264.GFP-ChIP-Seq(GSE58341)	RGGGCACTAACY	1.00E-02	-6.10E+00	0.0044	64	64.00%	17204	49.36%
Lhx1(Homeobox)/EmbryoCarcinoma-Lhx1-ChIP-Seq(GSE70957)	NNYTAATTAR	1.00E-02	-6.06E+00	0.0045	67	67.00%	18306.8	52.52%
Pax8(Paired,Homeobox)/Thyroid-Pax8-ChIP-Seq(GSE26938)	GTCATGCHTGRCTGS	1.00E-02	-6.04E+00	0.0046	47	47.00%	11477.6	32.93%
MNT(bHLH)/HepG2-MNT-ChIP-Seq(Encode)	DGCACACGTG	1.00E-02	-5.99E+00	0.0048	75	75.00%	21318.7	61.16%
NFAT(RHD)/Jurkat-NFATC1-ChIP-Seq(Jolma_et_al.)	ATTTTCCATT	1.00E-02	-5.96E+00	0.0049	66	66.00%	18000.2	51.64%
Pbx3(Homeobox)/GM12878-PBX3-ChIP-Seq(GSE32465)	SCTGTCAMTCAN	1.00E-02	-5.96E+00	0.0049	38	38.00%	8686.4	24.92%
Atf2(bZIP)/3T3L1-Atf2-ChIP-Seq(GSE56872)	NRRTGACGTCAT	1.00E-02	-5.92E+00	0.0051	41	41.00%	9630.1	27.63%
Foxa3(Forkhead)/Liver-Foxa3-ChIP-Seq(GSE77670)	BSNTGTTTACWYWGN	1.00E-02	-5.87E+00	0.0054	38	38.00%	8729.4	25.04%
NPAS(bHLH)/Liver-NPAS-ChIP-Seq(GSE39860)	NVCACGTG	1.00E-02	-5.86E+00	0.0054	87	87.00%	26218.7	75.22%
Bcl11a(Zf)/HSPC-BCL11A-ChIP-Seq(GSE104676)	TYTGACCASWRG	1.00E-02	-5.86E+00	0.0054	68	68.00%	18781.2	53.88%
Esrrb(NR)/mES-Esrrb-ChIP-Seq(GSE11431)	KTGACCTTGA	1.00E-02	-5.85E+00	0.0054	66	66.00%	18063.5	51.82%
bZIP:IRF(bZIP,IRF)/Th17-BatF-ChIP-Seq(GSE39756)	NAGTTTCABTHTGACTNW	1.00E-02	-5.77E+00	0.0058	45	45.00%	10969.5	31.47%
Cdx2(Homeobox)/mES-Cdx2-ChIP-Seq(GSE14586)	GYMATAAAAH	1.00E-02	-5.76E+00	0.0058	50	50.00%	12599.8	36.15%
Nkx2.5(Homeobox)/HL1-Nkx2.5.biotin-ChIP-Seq(GSE21529)	RRSCACTYAA	1.00E-02	-5.76E+00	0.0058	97	97.00%	30987.5	88.90%
ZFX(Zf)/mES-Zfx-ChIP-Seq(GSE11431)	AGGCCTRG	1.00E-02	-5.76E+00	0.0058	89	89.00%	27138.2	77.86%
KLF5(Zf)/LoVo-KLF5-ChIP-Seq(GSE49402)	DGGGYGKGGC	1.00E-02	-5.76E+00	0.0058	87	87.00%	26272.7	75.38%
c-Myc(bHLH)/mES-cMyc-ChIP-Seq(GSE11431)	VVCCACGTGG	1.00E-02	-5.75E+00	0.0058	54	54.00%	13941.9	40.00%
FOXM1(Forkhead)/MCF7-FOXM1-ChIP-Seq(GSE72977)	TRTTTACTTW	1.00E-02	-5.75E+00	0.0058	71	71.00%	19951	57.24%
TEAD1(TEAD)/HepG2-TEAD1-ChIP-Seq(Encode)	CYRCATTCCA	1.00E-02	-5.74E+00	0.0058	71	71.00%	19954.8	57.25%
CTCF(Zf)/CD4+-CTCF-ChIP-Seq(Barski_et_al.)	AYAGTGCCMYCTRGTGGCCA	1.00E-02	-5.72E+00	0.0059	27	27.00%	5546.9	15.91%
Gli2(Zf)/GM2-Gli2-ChIP-Chip(GSE112702)	YSTGGGTGGTCT	1.00E-02	-5.72E+00	0.0059	41	41.00%	9729.1	27.91%
ZNF675(Zf)/HEK293-ZNF675.GFP-ChIP-Seq(GSE58341)	ARGAGGMCAAAATGW	1.00E-02	-5.70E+00	0.006	24	24.00%	4719.1	13.54%
RBPJ:Ebox(?,bHLH)/Panc1-Rbpj1-ChIP-Seq(GSE47459)	GGGRAARRGRMCAGMTG	1.00E-02	-5.69E+00	0.006	46	46.00%	11335.1	32.52%
Hoxa13(Homeobox)/ChickenMSG-Hoxa13.Flag-ChIP-Seq(GSE86088)	CYHATAAAAN	1.00E-02	-5.67E+00	0.0061	93	93.00%	29000.3	83.20%
Hoxd10(Homeobox)/ChickenMSG-Hoxd10.Flag-ChIP-Seq(GSE86088)	GGCMATGAAA	1.00E-02	-5.59E+00	0.0066	71	71.00%	20043.4	57.50%
DLX2(Homeobox)/BasalGanglia-Dlx2-ChIP-seq(GSE124936)	NNNTAATTAS	1.00E-02	-5.57E+00	0.0067	77	77.00%	22325.5	64.05%
Atf4(bZIP)/MEF-Atf4-ChIP-Seq(GSE35681)	MTGATGCAAT	1.00E-02	-5.57E+00	0.0067	28	28.00%	5892.1	16.90%
CTCF-SatelliteElement(Zf?)/CD4+-CTCF-ChIP-Seq(Barski_et_al.)	TGCAGTTCCMVNWRTGGCCA	1.00E-02	-5.56E+00	0.0067	7	7.00%	690.3	1.98%
Hoxa10(Homeobox)/ChickenMSG-Hoxa10.Flag-ChIP-Seq(GSE86088)	GGYAATGAAA	1.00E-02	-5.52E+00	0.0069	48	48.00%	12072.1	34.63%
Nkx6.1(Homeobox)/Islet-Nkx6.1-ChIP-Seq(GSE40975)	GKTAATGR	1.00E-02	-5.51E+00	0.007	92	92.00%	28614.6	82.09%
Hoxd11(Homeobox)/ChickenMSG-Hoxd11.Flag-ChIP-Seq(GSE86088)	VGCCATAAAA	1.00E-02	-5.50E+00	0.007	93	93.00%	29081	83.43%
HLF(bZIP)/HSC-HLF.Flag-ChIP-Seq(GSE69817)	RTTATGYAAB	1.00E-02	-5.48E+00	0.0071	57	57.00%	15114.2	43.36%
BMYB(HTH)/Hela-BMYB-ChIP-Seq(GSE27030)	NHAACBGYYV	1.00E-02	-5.43E+00	0.0075	87	87.00%	26448.8	75.88%
KLF1(Zf)/HUDEP2-KLF1-CutnRun(GSE136251)	VDGGGYGGGGCY	1.00E-02	-5.36E+00	0.008	77	77.00%	22452.2	64.41%
ZNF711(Zf)/SHSY5Y-ZNF711-ChIP-Seq(GSE20673)	AGGCCTAG	1.00E-02	-5.33E+00	0.0082	94	94.00%	29643.6	85.05%
ZNF165(Zf)/WHIM12-ZNF165-ChIP-Seq(GSE65937)	AAGGKGRCGCAGGCA	1.00E-02	-5.30E+00	0.0084	26	26.00%	5432.4	15.59%
Tbx20(T-box)/Heart-Tbx20-ChIP-Seq(GSE29636)	GGTGYTGACAGS	1.00E-02	-5.29E+00	0.0085	33	33.00%	7484.9	21.47%
RUNX1(Runt)/Jurkat-RUNX1-ChIP-Seq(GSE29180)	AAACCACARM	1.00E-02	-5.27E+00	0.0086	77	77.00%	22505.5	64.57%
FOXA1(Forkhead)/LNCAP-FOXA1-ChIP-Seq(GSE27824)	WAAGTAAACA	1.00E-02	-5.21E+00	0.0091	74	74.00%	21392.1	61.37%
Pitx1:Ebox(Homeobox,bHLH)/Hindlimb-Pitx1-ChIP-Seq(GSE41591)	YTAATTRAWWCCAGATGT	1.00E-02	-5.18E+00	0.0093	22	22.00%	4361.5	12.51%
Elk1(ETS)/Hela-Elk1-ChIP-Seq(GSE31477)	HACTTCCGGY	1.00E-02	-5.15E+00	0.0096	63	63.00%	17407.8	49.94%

SCRT1(Zf)/HEK293-SCRT1.eGFP-ChIP-Seq(Encode)	GCAACAGGTG	1.00E-02	-5.11E+00	0.0099	37	37.00%	8790.4	25.22%
Tgif2(Homeobox)/mES-Tgif2-ChIP-Seq(GSE55404)	TGTCANYT	1.00E-02	-5.10E+00	0.01	100	100.00%	33123.4	95.03%
KLF3(Zf)/MEF-Klf3-ChIP-Seq(GSE44748)	NRGCCCCRCCCHBNN	1.00E-02	-5.09E+00	0.0101	60	60.00%	16387.8	47.02%
ZNF143|STAF(Zf)/CUTLL-ZNF143-ChIP-Seq(GSE29600)	ATTTCCCAGVAKSCY	1.00E-02	-5.08E+00	0.0101	51	51.00%	13314.5	38.20%
Reverb(NR),DR2/RAW-Reverba.biotin-ChIP-Seq(GSE45914)	GTRGGTCASTGGGTCA	1.00E-02	-5.06E+00	0.0103	26	26.00%	5529.7	15.86%
Sox3(HMG)/NPC-Sox3-ChIP-Seq(GSE33059)	CCWTTGTY	1.00E-02	-5.04E+00	0.0105	89	89.00%	27514.9	78.94%
ZSCAN22(Zf)/HEK293-ZSCAN22.GFP-ChIP-Seq(GSE58341)	SMCAGTCWGAKGGAGGAGGC	1.00E-02	-5.04E+00	0.0105	19	19.00%	3602.6	10.34%
USF1(bHLH)/GM12878-Usf1-ChIP-Seq(GSE32465)	SGTCACGTGR	1.00E-02	-4.92E+00	0.0117	48	48.00%	12414.9	35.62%
Meis1(Homeobox)/MastCells-Meis1-ChIP-Seq(GSE48085)	VGCTGWCAVB	1.00E-02	-4.88E+00	0.0122	92	92.00%	28929.4	83.00%
Srebp2(bHLH)/HepG2-Srebp2-ChIP-Seq(GSE31477)	CGGTCACSCCAC	1.00E-02	-4.87E+00	0.0122	24	24.00%	5040.1	14.46%
OCT:OCT(POU,Homeobox)/NPC-OCT6-ChIP-Seq(GSE43916)	YATGCATATRCATRT	1.00E-02	-4.87E+00	0.0122	17	17.00%	3131.9	8.99%
Tbx5(T-box)/HL1-Tbx5.biotin-ChIP-Seq(GSE21529)	AGGTGTCA	1.00E-02	-4.81E+00	0.0129	100	100.00%	33219.8	95.31%
PAX3:FKHR-fusion(Paired,Homeobox)/Rh4-PAX3:FKHR-ChIP-Seq	ACCRTGACTAATTNN	1.00E-02	-4.81E+00	0.0129	24	24.00%	5067.3	14.54%
PRDM9(Zf)/Testis-DMC1-ChIP-Seq(GSE35498)	ADGGYAGYAGCATCT	1.00E-02	-4.80E+00	0.013	48	48.00%	12487.6	35.83%
Tlx?(NR)/NPC-H3K4me1-ChIP-Seq(GSE16256)	CTGGCAGSCTGCCA	1.00E-02	-4.77E+00	0.0133	55	55.00%	14857.7	42.63%
Hoxa9(Homeobox)/ChickenMSG-Hoxa9.Flag-ChIP-Seq(GSE86088)	RGCAATNAAA	1.00E-02	-4.77E+00	0.0133	95	95.00%	30391.9	87.19%
Klf4(Zf)/mES-Klf4-ChIP-Seq(GSE11431)	GCCACACCCA	1.00E-02	-4.75E+00	0.0134	50	50.00%	13179.3	37.81%
HIF-1b(HLH)/T47D-HIF1b-ChIP-Seq(GSE59937)	RTACGTGC	1.00E-02	-4.72E+00	0.0138	74	74.00%	21698.5	62.25%
Gata1(Zf)/K562-GATA1-ChIP-Seq(GSE18829)	SAGATAAGRV	1.00E-02	-4.72E+00	0.0138	50	50.00%	13202.8	37.88%
Prop1(Homeobox)/GHFT1-PROP1.biotin-ChIP-Seq(GSE77302)	NTAATBNAATTA	1.00E-02	-4.71E+00	0.0138	40	40.00%	9945.2	28.53%
BORIS(Zf)/K562-CTCFL-ChIP-Seq(GSE32465)	CNNBRGCGCCCCCTGSTGGC	1.00E-02	-4.67E+00	0.0143	33	33.00%	7786.6	22.34%
Oct4(POU,Homeobox)/mES-Oct4-ChIP-Seq(GSE11431)	ATTTGCATAW	1.00E-02	-4.65E+00	0.0146	37	37.00%	9034.4	25.92%
E2F3(E2F)/MEF-E2F3-ChIP-Seq(GSE71376)	BTKGGCGGGAAA	1.00E-02	-4.65E+00	0.0146	55	55.00%	14934.3	42.85%
Smad3(MAD)/NPC-Smad3-ChIP-Seq(GSE36673)	TWGTCTGV	1.00E-02	-4.63E+00	0.0148	100	100.00%	33279.3	95.48%
Lhx2(Homeobox)/HFSC-Lhx2-ChIP-Seq(GSE48068)	TAATTAGN	1.00E-02	-4.61E+00	0.015	64	64.00%	18103.8	51.94%
Srebp1a(bHLH)/HepG2-Srebp1a-ChIP-Seq(GSE31477)	RTCACSCCAY	1.00E-01	-4.57E+00	0.0156	30	30.00%	6929.7	19.88%
CDX4(Homeobox)/ZebrafishEmbryos-Cdx4.Myc-ChIP-Seq(GSE48254)	NGYCATAAAWCH	1.00E-01	-4.53E+00	0.0161	58	58.00%	16041.5	46.02%
Gata6(Zf)/HUG1N-GATA6-ChIP-Seq(GSE51936)	YCTTATCTBN	1.00E-01	-4.53E+00	0.0161	62	62.00%	17443.3	50.04%
Sp1(Zf)/Promoter	GGCCCCGCCCCC	1.00E-01	-4.51E+00	0.0164	38	38.00%	9427.7	27.05%
Mef2c(MADS)/GM12878-Mef2c-ChIP-Seq(GSE32465)	DCYAAAAATAGM	1.00E-01	-4.50E+00	0.0165	40	40.00%	10067.1	28.88%
FOXA1(Forkhead)/MCF7-FOXA1-ChIP-Seq(GSE26831)	WAAGTAAACA	1.00E-01	-4.46E+00	0.0171	66	66.00%	18918.1	54.28%
CUX1(Homeobox)/K562-CUX1-ChIP-Seq(GSE92882)	TATCGATNAN	1.00E-01	-4.46E+00	0.0171	44	44.00%	11380.8	32.65%
RARa(NR)/K562-RARa-ChIP-Seq(Encode)	TTGAMCTTTG	1.00E-01	-4.42E+00	0.0178	99	99.00%	32677.1	93.75%
Duxbl(Homeobox)/NIH3T3-Duxbl.HA-ChIP-Seq(GSE119782)	TAAYCYAATCAA	1.00E-01	-4.40E+00	0.018	10	10.00%	1523.8	4.37%
IRF:BATF(IRF:bZIP)/pDC-Irf8-ChIP-Seq(GSE66899)	CTTTCANTATGACTV	1.00E-01	-4.40E+00	0.018	17	17.00%	3290.4	9.44%
Egr1(Zf)/K562-Egr1-ChIP-Seq(GSE32465)	TGCGTGGGYG	1.00E-01	-4.39E+00	0.0181	66	66.00%	18970.2	54.43%
IRF1(IRF)/PBMC-IRF1-ChIP-Seq(GSE43036)	GAAAGTGAAAGT	1.00E-01	-4.32E+00	0.0194	21	21.00%	4423.2	12.69%
GATA3(Zf),DR4/iTreg-Gata3-ChIP-Seq(GSE20898)	AGATGKDGAGATAAG	1.00E-01	-4.22E+00	0.0212	12	12.00%	2052.7	5.89%
ZNF669(Zf)/HEK293-ZNF669.GFP-ChIP-Seq(GSE58341)	GARTGGTCATCGCCC	1.00E-01	-4.22E+00	0.0212	14	14.00%	2560	7.34%
Bapx1(Homeobox)/VertebralCol-Bapx1-ChIP-Seq(GSE36672)	TTRAGTGSYK	1.00E-01	-4.17E+00	0.0221	94	94.00%	30209	86.67%
TCFL2(HMG)/K562-TCF7L2-ChIP-Seq(GSE29196)	ACWTCAAAGG	1.00E-01	-4.17E+00	0.0221	15	15.00%	2838	8.14%
Pit1+1bp(Homeobox)/GCrat-Pit1-ChIP-Seq(GSE58009)	ATGCATAATTCA	1.00E-01	-4.16E+00	0.0222	27	27.00%	6236.1	17.89%
HIC1(Zf)/Treg-ZBTB29-ChIP-Seq(GSE99889)	TGCCAGCB	1.00E-01	-4.15E+00	0.0225	95	95.00%	30690	88.05%
Gata2(Zf)/K562-GATA2-ChIP-Seq(GSE18829)	BBCTTATCTS	1.00E-01	-4.12E+00	0.023	52	52.00%	14259.8	40.91%
NPAS2(bHLH)/Liver-NPAS2-ChIP-Seq(GSE39860)	KCCACGTGAC	1.00E-01	-4.11E+00	0.0233	74	74.00%	22107	63.42%
VDR(NR),DR3/GM10855-VDR+vitD-ChIP-Seq(GSE22484)	ARAGGTCANWGAGTTCANNN	1.00E-01	-4.09E+00	0.0236	32	32.00%	7792.1	22.36%
Nkx2.1(Homeobox)/LungAC-Nkx2.1-ChIP-Seq(GSE43252)	RSCACTYRAG	1.00E-01	-4.09E+00	0.0236	99	99.00%	32802.1	94.11%
Ptf1a(bHLH)/Panc1-Ptf1a-ChIP-Seq(GSE47459)	ACAGCTGTTN	1.00E-01	-4.07E+00	0.0239	98	98.00%	32239.3	92.49%
Znf263(Zf)/K562-Znf263-ChIP-Seq(GSE31477)	CVGTSCTCCC	1.00E-01	-4.07E+00	0.024	94	94.00%	30264.9	86.83%
HOXA2(Homeobox)/mES-Hoxa2-ChIP-Seq(Donaldson_et_al.)	GYCATCMATCAT	1.00E-01	-4.05E+00	0.0243	14	14.00%	2618.7	7.51%
Eomes(T-box)/H9-Eomes-ChIP-Seq(GSE26097)	ATTAACACCT	1.00E-01	-4.04E+00	0.0244	91	91.00%	28936.5	83.02%
IRF2(IRF)/Erythroblas-IRF2-ChIP-Seq(GSE36985)	GAAASYGAAASY	1.00E-01	-4.03E+00	0.0245	17	17.00%	3426.3	9.83%
En1(Homeobox)/SUM149-EN1-ChIP-Seq(GSE120957)	NDCTAATTAS	1.00E-01	-3.99E+00	0.0254	82	82.00%	25258.2	72.47%
Unknown(Homeobox)/Limb-p300-ChIP-Seq	SSCMATWAAA	1.00E-01	-3.97E+00	0.0259	48	48.00%	13013.7	37.34%

Sox17(HMG)/Endoderm-Sox17-ChIP-Seq(GSE61475)	CCATTGTTYB	1.00E-01	-3.97E+00	0.0259	59	59.00%	16776.5	48.13%
Npas4(bHLH)/Neuron-Npas4-ChIP-Seq(GSE127793)	NHRTCACGACDN	1.00E-01	-3.89E+00	0.028	62	62.00%	17889.5	51.32%
Tcfcp2l1(CP2)/mES-Tcfcp2l1-ChIP-Seq(GSE11431)	NRAACCRGTTYRAACCRGYT	1.00E-01	-3.89E+00	0.028	27	27.00%	6379.7	18.30%
Sox15(HMG)/CPA-Sox15-ChIP-Seq(GSE62909)	RAACAATGGN	1.00E-01	-3.88E+00	0.0281	73	73.00%	21890.2	62.80%
Chop(bZIP)/MEF-Chop-ChIP-Seq(GSE35681)	ATTGCATCAT	1.00E-01	-3.84E+00	0.0291	21	21.00%	4634	13.29%
Pknox1(Homeobox)/ES-Prep1-ChIP-Seq(GSE63282)	SCTGTCAVTCAV	1.00E-01	-3.84E+00	0.0292	33	33.00%	8249.5	23.67%
PAX5(Paired,Homeobox)/GM12878-PAX5-ChIP-Seq(GSE32465)	GCAGCCAAGCRTGACH	1.00E-01	-3.82E+00	0.0295	44	44.00%	11790.3	33.83%
GATA(Zf),IR3/iTreg-Gata3-ChIP-Seq(GSE20898)	NNNNNBAGATAWYATCTVHN	1.00E-01	-3.78E+00	0.0306	17	17.00%	3526.4	10.12%
Sox9(HMG)/Limb-SOX9-ChIP-Seq(GSE73225)	AGGVNCCTTTGT	1.00E-01	-3.77E+00	0.031	71	71.00%	21231.5	60.91%
HRE(HSF)/Striatum-HSF1-ChIP-Seq(GSE38000)	TTCTAGAABNTTCTA	1.00E-01	-3.71E+00	0.0328	32	32.00%	8015	22.99%
Mef2a(MADS)/HL1-Mef2a.biotin-ChIP-Seq(GSE21529)	CYAAAAATAG	1.00E-01	-3.69E+00	0.0334	38	38.00%	9928.5	28.48%
bHLHE41(bHLH)/proB-Bhlhe41-ChIP-Seq(GSE93764)	KCACGTGMCN	1.00E-01	-3.68E+00	0.0334	74	74.00%	22406.2	64.28%
Hoxb4(Homeobox)/ES-Hoxb4-ChIP-Seq(GSE34014)	TGATTRATGGCY	1.00E-01	-3.60E+00	0.036	22	22.00%	5037	14.45%
ISRE(IRF)/ThioMac-LPS-Expression(GSE23622)	AGTTTCASTTTC	1.00E-01	-3.60E+00	0.036	11	11.00%	1987.1	5.70%
Usf2(bHLH)/C2C12-Usf2-ChIP-Seq(GSE36030)	GTCACGTGGT	1.00E-01	-3.54E+00	0.0381	36	36.00%	9382.1	26.92%
Egr2(Zf)/Thymocytes-Egr2-ChIP-Seq(GSE34254)	NGCGTGGGCGGR	1.00E-01	-3.54E+00	0.0381	26	26.00%	6265	17.97%
c-Jun-CRE(bZIP)/K562-cJun-ChIP-Seq(GSE31477)	ATGACGTCATCY	1.00E-01	-3.51E+00	0.0391	34	34.00%	8767	25.15%
NF1-halfsite(CTF)/LNCaP-NF1-ChIP-Seq(Unpublished)	YTGCCAAG	1.00E-01	-3.46E+00	0.0408	91	91.00%	29274.3	83.99%
TEAD(TEA)/Fibroblast-PU.1-ChIP-Seq(Unpublished)	YCWGGAATGY	1.00E-01	-3.46E+00	0.0408	53	53.00%	15074.3	43.25%
LXRE(NR),DR4/RAW-LXRb.biotin-ChIP-Seq(GSE21512)	RGGTTACTANAGGTCA	1.00E-01	-3.40E+00	0.0431	11	11.00%	2050.6	5.88%
GFY(?)/Promoter	ACTACAATTCCC	1.00E-01	-3.40E+00	0.0432	13	13.00%	2582.8	7.41%
HNF6(Homeobox)/Liver-Hnf6-ChIP-Seq(ERP000394)	NTATYGATCH	1.00E-01	-3.38E+00	0.044	39	39.00%	10461.1	30.01%
c-Myc(bHLH)/LNCAP-cMyc-ChIP-Seq(Unpublished)	VCCACGTG	1.00E-01	-3.36E+00	0.0444	43	43.00%	11781.6	33.80%
Six4(Homeobox)/MCF7-SIX4-ChIP-Seq(Encode)	TGWAAYCTGABACCB	1.00E-01	-3.33E+00	0.0459	9	9.00%	1563.7	4.49%
Pax7(Paired,Homeobox),long/Myoblast-Pax7-ChIP-Seq(GSE25064)	TAATCHGATTAC	1.00E-01	-3.32E+00	0.0459	4	4.00%	431.9	1.24%
GATA:SCL(Zf,bHLH)/Ter119-SCL-ChIP-Seq(GSE18720)	CRGCTGBNGNSNNSAGATAA	1.00E-01	-3.27E+00	0.0484	19	19.00%	4326.3	12.41%
Brn2(POU,Homeobox)/NPC-Brn2-ChIP-Seq(GSE35496)	ATGAATATTC	1.00E-01	-3.27E+00	0.0484	10	10.00%	1836.6	5.27%
Tbox:Smad(T-box,MAD)/ESCd5-Smad2_3-ChIP-Seq(GSE29422)	AGGTGHCAGACA	1.00E-01	-3.26E+00	0.0485	29	29.00%	7353.5	21.10%
LHX9(Homeobox)/Hct116-LHX9.V5-ChIP-Seq(GSE116822)	NGCTAATTAG	1.00E-01	-3.26E+00	0.0486	73	73.00%	22357	64.14%
FOXA1:AR(Forkhead,NR)/LNCAP-AR-ChIP-Seq(GSE27824)	AGTAAACAAAAAAGAACAND	1.00E-01	-3.22E+00	0.0502	12	12.00%	2379.7	6.83%
ETS:E-box(ETS,bHLH)/HPC7-Scl-ChIP-Seq(GSE22178)	AGGAARCAGCTG	1.00E-01	-3.20E+00	0.0509	17	17.00%	3779.2	10.84%
Twist(bHLH)/HMLE-TWIST1-ChIP-Seq(Chang_et_al)	VCAKCTGGNNNCCAGMTGBN	1.00E-01	-3.10E+00	0.0565	21	21.00%	5006.8	14.36%
PBX1(Homeobox)/MCF7-PBX1-ChIP-Seq(GSE28007)	GSCTGTCACTCA	1.00E-01	-3.09E+00	0.0569	16	16.00%	3547.7	10.18%
TR4(NR),DR1/Hela-TR4-ChIP-Seq(GSE24685)	GAGGTCAAAGGTCA	1.00E-01	-3.07E+00	0.0575	21	21.00%	5019.7	14.40%
Hnf6b(Homeobox)/LNCaP-Hnf6b-ChIP-Seq(GSE106305)	TATTGAYY	1.00E-01	-3.06E+00	0.0584	52	52.00%	15051.4	43.18%
Phox2a(Homeobox)/Neuron-Phox2a-ChIP-Seq(GSE31456)	YTAATYNRATTA	1.00E-01	-3.06E+00	0.0584	26	26.00%	6551.1	18.80%
RORgt(NR)/EL4-RORgt.Flag-ChIP-Seq(GSE56019)	AAYTAGGTCA	1.00E-01	-3.04E+00	0.0587	20	20.00%	4738.2	13.59%
RORgt(NR)/EL4-RORgt.Flag-ChIP-Seq(GSE56019)	AAYTAGGTCA	1.00E-01	-3.04E+00	0.0587	20	20.00%	4738.2	13.59%
X-box(HTH)/NPC-H3K4me1-ChIP-Seq(GSE16256)	GGTTGCCATGGCAA	1.00E-01	-3.02E+00	0.0598	19	19.00%	4453.7	12.78%
Oct6(POU,Homeobox)/NPC-Pou3f1-ChIP-Seq(GSE35496)	WATGCAAATGAG	1.00E-01	-3.01E+00	0.06	32	32.00%	8462.4	24.28%
TRPS1(Zf)/MCF7-TRPS1-ChIP-Seq(GSE107013)	AGATAAGANN	1.00E-01	-3.00E+00	0.0607	85	85.00%	27131.6	77.84%
EBF(EBF)/proBcell-EBF-ChIP-Seq(GSE21978)	DGTCCCYRGGGA	1.00E-01	-2.99E+00	0.0611	33	33.00%	8798.4	25.24%
HRE(HSF)/HepG2-HSF1-ChIP-Seq(GSE31477)	BSTTCTRGAABVTTCYAGAA	1.00E-01	-2.95E+00	0.0634	25	25.00%	6309	18.10%
Rfx1(HTH)/NPC-H3K4me1-ChIP-Seq(GSE16256)	KGTTGCCATGGCAA	1.00E-01	-2.87E+00	0.0685	29	29.00%	7610.7	21.83%
NFIL3(bZIP)/HepG2-NFIL3-ChIP-Seq(Encode)	VTTACGTAAYNNNNN	1.00E-01	-2.86E+00	0.0689	44	44.00%	12502	35.87%
Nkx2.2(Homeobox)/NPC-Nkx2.2-ChIP-Seq(GSE61673)	BTBRAGTGSN	1.00E-01	-2.84E+00	0.07	92	92.00%	30081.4	86.30%
ZNF41(Zf)/HEK293-ZNF41.GFP-ChIP-Seq(GSE58341)	CCTCATGGTGYCYTWYTCCCTTGTG	1.00E-01	-2.74E+00	0.0772	6	6.00%	984.6	2.82%
HOXA1(Homeobox)/mES-Hoxa1-ChIP-Seq(SRP084292)	TGATKGATGR	1.00E-01	-2.74E+00	0.0774	24	24.00%	6135.4	17.60%
E2F(E2F)/Hela-CellCycle-Expression	TTSGCGCGAAAA	1.00E-01	-2.73E+00	0.0779	8	8.00%	1492.7	4.28%
Pitx1(Homeobox)/Chicken-Pitx1-ChIP-Seq(GSE38910)	TAATCCCN	1.00E-01	-2.69E+00	0.081	100	100.00%	33931.5	97.35%
Cux2(Homeobox)/Liver-Cux2-ChIP-Seq(GSE35985)	HNRAATCAAT	1.00E-01	-2.68E+00	0.0812	31	31.00%	8383.2	24.05%
CREB5(bZIP)/LNCaP-CREB5.V5-ChIP-Seq(GSE137775)	VVATGACGTCAT	1.00E-01	-2.64E+00	0.0848	35	35.00%	9712.9	27.87%
OCT:OCT-short(POU,Homeobox)/NPC-OCT6-ChIP-Seq(GSE43916)	ATGCATAATGCATRW	1.00E-01	-2.49E+00	0.0977	41	41.00%	11814.5	33.90%
HIF-1a(bHLH)/MCF7-HIF1a-ChIP-Seq(GSE28352)	TACGTGCV	1.00E-01	-2.49E+00	0.0977	27	27.00%	7249.3	20.80%

ZKSCAN1(Zf)/HepG2-ZKSCAN1-ChIP-Seq(Encode)	GCACAYAGTAGGKCY	1.00E-01	-2.47E+00	0.0997	8	8.00%	1582.8	4.54%
Klf9(Zf)/GBM-Klf9-ChIP-Seq(GSE62211)	GCCACRCCCACY	1.00E-01	-2.45E+00	0.1014	47	47.00%	13876.6	39.81%
CEBP:CEBP(bZIP)/MEF-Chop-ChIP-Seq(GSE35681)	NTNATGCAAYMNNHTGMAAY	1.00E-01	-2.34E+00	0.1121	14	14.00%	3341	9.59%
Oct2(POU,Homeobox)/Bcell-Oct2-ChIP-Seq(GSE21512)	ATATGCAAAT	1.00E+00	-2.29E+00	0.1179	22	22.00%	5817.1	16.69%
Brachyury(T-box)/Mesoendoderm-Brachyury-ChIP-exo(GSE54963)	ANTTMRCASBNNNGTGYKAAN	1.00E+00	-2.25E+00	0.1224	29	29.00%	8078.6	23.18%
p53(p53)/Saos-p53-ChIP-Seq(GSE15780)	RRCATGYCYRGRCATGYYYN	1.00E+00	-2.21E+00	0.1264	14	14.00%	3413.7	9.79%
p53(p53)/Saos-p53-ChIP-Seq	RRCATGYCYRGRCATGYYYN	1.00E+00	-2.21E+00	0.1264	14	14.00%	3413.7	9.79%
NRF(NRF)/Promoter	STGCGCATGCGC	1.00E+00	-2.13E+00	0.137	21	21.00%	5620.2	16.12%
GATA(Zf),IR4/iTreg-Gata3-ChIP-Seq(GSE20898)	NAGATWNBNATCTNN	1.00E+00	-2.11E+00	0.1395	9	9.00%	2003.1	5.75%
bHLHE40(bHLH)/HepG2-BHLHE40-ChIP-Seq(GSE31477)	KCACGTGMCN	1.00E+00	-2.10E+00	0.1404	31	31.00%	8858.3	25.41%
Nur77(NR)/K562-NR4A1-ChIP-Seq(GSE31363)	TGACCTTTNCNT	1.00E+00	-2.05E+00	0.1471	21	21.00%	5679.4	16.29%
PAX6(Paired,Homeobox)/Forebrain-Pax6-ChIP-Seq(GSE66961)	NGTGTTCAVTSAAGCGKAAA	1.00E+00	-2.00E+00	0.154	12	12.00%	2935.4	8.42%
NFAT:AP1(RHD,bZIP)/Jurkat-NFATC1-ChIP-Seq(Jolma_et_al.)	SARTGGAAAAWRTGAGTCAB	1.00E+00	-1.97E+00	0.1579	19	19.00%	5106.6	14.65%
Oct11(POU,Homeobox)/NCIH1048-POU2F3-ChIP-seq(GSE115123)	GATTTGCATA	1.00E+00	-1.97E+00	0.1579	24	24.00%	6697.6	19.22%
Sox10(HMG)/SciaticNerve-Sox3-ChIP-Seq(GSE35132)	CCWTTGTYYB	1.00E+00	-1.97E+00	0.1579	83	83.00%	27198.9	78.03%
MYNN(Zf)/HEK293-MYNN.eGFP-ChIP-Seq(Encode)	TTCAAAWTAAAAGTC	1.00E+00	-1.90E+00	0.1686	23	23.00%	6436.6	18.47%
E-box(bHLH)/Promoter	SSGGTCACGTGA	1.00E+00	-1.88E+00	0.1726	11	11.00%	2708.7	7.77%
AR-halfsite(NR)/LNCaP-AR-ChIP-Seq(GSE27824)	CCAGGAACAG	1.00E+00	-1.79E+00	0.1871	100	100.00%	34236.3	98.22%
p73(p53)/Trachea-p73-ChIP-Seq(PRJNA310161)	NRRRCAWGTCCDGRCATGYY	1.00E+00	-1.78E+00	0.1885	8	8.00%	1869.7	5.36%
GFY-Staf(?,Zf)/Promoter	RACTACAATTCCCAGAAKGC	1.00E+00	-1.78E+00	0.1892	10	10.00%	2465.2	7.07%
Zfp57(Zf)/H1-ZFP57.HA-ChIP-Seq(GSE115387)	NANTGCSGCA	1.00E+00	-1.75E+00	0.1936	30	30.00%	8861.5	25.42%
ZNF136(Zf)/HEK293-ZNF136.GFP-ChIP-Seq(GSE58341)	YTKGATAHAGTATTCTWGGTNGGCA	1.00E+00	-1.72E+00	0.1987	10	10.00%	2496.4	7.16%
TFE3(bHLH)/MEF-TFE3-ChIP-Seq(GSE75757)	GTCACGTGACYV	1.00E+00	-1.72E+00	0.1987	11	11.00%	2801.4	8.04%
THRb(NR)/Liver-NR1A2-ChIP-Seq(GSE52613)	TRAGGTCA	1.00E+00	-1.71E+00	0.2005	100	100.00%	34265.3	98.31%
ZNF528(Zf)/HEK293-ZNF528.GFP-ChIP-Seq(GSE58341)	AGAAATGACTTCCCT	1.00E+00	-1.71E+00	0.2005	2	2.00%	270.6	0.78%
Mouse_Recombination_Hotspot(Zf)/Testis-DMC1-ChIP-Seq	ACTYKNATTCGTGNTACTTC	1.00E+00	-1.61E+00	0.2209	8	8.00%	1960.2	5.62%
DUX4(Homeobox)/Myoblasts-DUX4.V5-ChIP-Seq(GSE75791)	NWTAAYCYAATCAWN	1.00E+00	-1.59E+00	0.2237	4	4.00%	810.4	2.33%
Nanog(Homeobox)/mES-Nanog-ChIP-Seq(GSE11724)	RGCCATTAAC	1.00E+00	-1.50E+00	0.2439	99	99.00%	33871.7	97.18%
Oct4:Sox17(POU,Homeobox,HMG)/F9-Sox17-ChIP-Seq(GSE44553)	CCATTGTATGCAAAT	1.00E+00	-1.47E+00	0.2504	13	13.00%	3594.3	10.31%
ZFP3(Zf)/HEK293-ZFP3.GFP-ChIP-Seq(GSE58341)	GGGTTTTGAAGGATGARTAGGAGTT	1.00E+00	-1.41E+00	0.2673	1	1.00%	98.3	0.28%
GLI3(Zf)/Limb-GLI3-ChIP-Chip(GSE11077)	CGTGGGTGGTCC	1.00E+00	-1.34E+00	0.2852	17	17.00%	5000	14.34%
ZNF768(Zf)/Rajj-ZNF768-ChIP-Seq(GSE111879)	RHHCAGAGAGGB	1.00E+00	-1.33E+00	0.2867	7	7.00%	1813.2	5.20%
OCT4-SOX2-TCF-NANOG(POU,Homeobox,HMG)/mES-Oct4-ChIP-Seq	ATTTGCATAACAATG	1.00E+00	-1.30E+00	0.2961	13	13.00%	3739.6	10.73%
ZNF16(Zf)/HEK293-ZNF16.GFP-ChIP-Seq(GSE58341)	MACCTTCYATGGCTCCCTAKTGCCY	1.00E+00	-1.29E+00	0.2969	2	2.00%	359.8	1.03%
Brn1(POU,Homeobox)/NPC-Brn1-ChIP-Seq(GSE35496)	TATGCWAATBAV	1.00E+00	-1.27E+00	0.3031	21	21.00%	6391.5	18.34%
p53(p53)/mES-cMyc-ChIP-Seq(GSE11431)	ACATGCCCGGGCAT	1.00E+00	-1.21E+00	0.3208	3	3.00%	666.3	1.91%
HINFP(Zf)/K562-HINFP.eGFP-ChIP-Seq(Encode)	TWVGGTCCGC	1.00E+00	-1.20E+00	0.3222	25	25.00%	7805.9	22.40%
SCL(bHLH)/HPC7-Scl-ChIP-Seq(GSE13511)	AVCAGCTG	1.00E+00	-1.16E+00	0.3348	100	100.00%	34453.7	98.85%
RFX(HTH)/K562-RFX3-ChIP-Seq(SRA012198)	CGGTTGCCATGGCAAC	1.00E+00	-1.16E+00	0.3351	13	13.00%	3864.8	11.09%
Sox4(HMG)/proB-Sox4-ChIP-Seq(GSE50066)	YCTTTGTTCC	1.00E+00	-1.13E+00	0.3447	60	60.00%	19946.6	57.23%
PAX5(Paired,Homeobox),condensed/GM12878-PAX5-ChIP-Seq	GTCACGCTCSCTGM	1.00E+00	-1.09E+00	0.3558	13	13.00%	3927.3	11.27%
NF1:FOXA1(CTF,Forkhead)/LNCAP-FOXA1-ChIP-Seq(GSE27824)	WNTGTTTRYTTTGGCA	1.00E+00	-1.07E+00	0.3622	6	6.00%	1662.5	4.77%
Rfx2(HTH)/LoVo-RFX2-ChIP-Seq(GSE49402)	GTTGCCATGGCAACM	1.00E+00	-1.07E+00	0.3632	14	14.00%	4284.7	12.29%
NFkB-p65-Rel(RHD)/ThioMac-LPS-Expression(GSE23622)	GGAAATTCCC	1.00E+00	-1.02E+00	0.3808	7	7.00%	2024.6	5.81%
Mef2d(MADS)/Retina-Mef2d-ChIP-Seq(GSE61391)	GCTATTTTTAGC	1.00E+00	-9.51E-01	0.4056	16	16.00%	5085.6	14.59%
RAR:RXR(NR),DR5/ES-RAR-ChIP-Seq(GSE56893)	RGGTCADNNAGAGGTCAV	1.00E+00	-9.00E-01	0.4258	5	5.00%	1456.9	4.18%
Phox2b(Homeobox)/CLBGA-PHOX2B-ChIP-Seq(GSE90683)	TTAATTNAATTA	1.00E+00	-8.31E-01	0.4552	11	11.00%	3535.2	10.14%
HNF1b(Homeobox)/PDAC-HNF1B-ChIP-Seq(GSE64557)	GTTAATNATTAA	1.00E+00	-7.41E-01	0.4971	9	9.00%	2955.7	8.48%
Hnf1(Homeobox)/Liver-Foxa2-Chip-Seq(GSE25694)	GGTTAAWCATTAA	1.00E+00	-6.34E-01	0.5517	8	8.00%	2735.6	7.85%
ZNF382(Zf)/HEK293-ZNF382.GFP-ChIP-Seq(GSE58341)	GNCTGTASTRNTGBCTCHTT	1.00E+00	-6.34E-01	0.5517	3	3.00%	972.7	2.79%
REST-NRSF(Zf)/Jurkat-NRSF-ChIP-Seq	GGMGCTGTCCATGGTGCTGA	1.00E+00	-5.58E-01	0.5928	1	1.00%	295.1	0.85%
NRF1(NRF)/MCF7-NRF1-ChIP-Seq(Unpublished)	CTGCGCATGCGC	1.00E+00	-5.30E-01	0.6079	11	11.00%	3950.2	11.33%
GFX(?)/Promoter	ATTCTCGCGAGA	1.00E+00	-5.03E-01	0.6231	1	1.00%	323	0.93%
YY1(Zf)/Promoter	CAAGATGGCGGC	1.00E+00	-4.62E-01	0.6479	7	7.00%	2615.8	7.50%

T1ISRE(IRF)/ThioMac-Ifnb-Expression	ACTTTCGTTTCT	1.00E+00	-4.60E-01	0.6479	1	1.00%	346.7	0.99%
Sox7(HMG)/ESC-Sox7-ChIP-Seq(GSE133899)	VVRRAACAATGG	1.00E+00	-4.20E-01	0.6724	25	25.00%	9184.7	26.35%
JunD(bZIP)/K562-JunD-ChIP-Seq	ATGACGTCATCN	1.00E+00	-4.00E-01	0.6842	8	8.00%	3089.8	8.86%
Pax7(Paired,Homeobox),longest/Myoblast-Pax7-ChIP-Seq(GSE25064)	NTAATTDGCYAATTANNWWD	1.00E+00	-2.69E-01	0.7784	1	1.00%	500.5	1.44%
ZBTB33(Zf)/GM12878-ZBTB33-ChIP-Seq(GSE32465)	GGVTCTCGCGAGAAC	1.00E+00	-2.60E-01	0.7838	2	2.00%	973	2.79%
Ronin(THAP)/ES-Thap11-ChIP-Seq(GSE51522)	RACTACAACTCCCAGVAKGC	1.00E+00	-1.57E-01	0.8667	2	2.00%	1176.7	3.38%
ZNF317(Zf)/HEK293-ZNF317.GFP-ChIP-Seq(GSE58341)	GTCWGCTGTYYCTCT	1.00E+00	-1.43E-01	0.8771	7	7.00%	3402.8	9.76%
Pax7(Paired,Homeobox)/Myoblast-Pax7-ChIP-Seq(GSE25064)	TAATCAATTA	1.00E+00	-1.21E-01	0.8944	3	3.00%	1762.6	5.06%
DUX(Homeobox)/C2C12-Dux-ChIP-Seq(GSE87279)	BCWGATTCAATCAAN	1.00E+00	0.00E+00	1	0	0.00%	65.1	0.19%
EBNA1(EBV-virus)/Raji-EBNA1-ChIP-Seq(GSE30709)	GGYAGCAYDTGCTDCCCNNN	1.00E+00	0.00E+00	1	0	0.00%	757.3	2.17%
OCT:OCT(POU,Homeobox,IR1)/NPC-Brn2-ChIP-Seq(GSE35496)	ATGAATWATTCATGA	1.00E+00	0.00E+00	1	0	0.00%	331.9	0.95%
OCT:OCT(POU,Homeobox)/NPC-Brn1-ChIP-Seq(GSE35496)	ATGAATATTCATGAG	1.00E+00	0.00E+00	1	0	0.00%	218	0.63%

Supplementary Table 7: Motifs associated with hypoacetylated peaks in substantia nigra

Motif Name	Consensus	P-value	Log P-value	q-value (Benjamini)	# of Target Sequences with Motif(of 99)	% of Target Sequences with Motif	# of Background Sequences with Motif(of 32607)	% of Background Sequences with Motif
HRE(HSF)/Striatum-HSF1-ChIP-Seq(GSE38000)	TTCTAGAABNTTCTA	1.00E-07	-1.72E+01	0	45	45.45%	6785.6	20.81%
HOXA1(Homeobox)/mES-Hoxa1-ChIP-Seq(SRP084292)	TGATKGATGR	1.00E-06	-1.49E+01	0.0001	45	45.45%	7322.4	22.46%
HOXA2(Homeobox)/mES-Hoxa2-ChIP-Seq(Donaldson_et_al.)	GYCATCMATCAT	1.00E-05	-1.32E+01	0.0003	26	26.26%	3164.3	9.70%
Cux2(Homeobox)/Liver-Cux2-ChIP-Seq(GSE35985)	HNRAATCAAT	1.00E-05	-1.21E+01	0.0006	58	58.59%	11845.7	36.33%
ZNF675(Zf)/HEK293-ZNF675.GFP-ChIP-Seq(GSE58341)	ARGAGGMCAAAATGW	1.00E-04	-1.11E+01	0.0013	30	30.30%	4457.9	13.67%
HNF6(Homeobox)/Liver-Hnf6-ChIP-Seq(ERP000394)	NTATYGATCH	1.00E-04	-1.02E+01	0.0026	62	62.63%	13785.1	42.28%
Foxh1(Forkhead)/hESC-FOXH1-ChIP-Seq(GSE29422)	NNTGTGGATTSS	1.00E-04	-1.00E+01	0.0028	67	67.68%	15533.9	47.64%
PAX3:FKHR-fusion(Paired,Homeobox)/Rh4-PAX3:FKHR-ChIP-Seq(GSE19063)	ACCRTGACTAATTNN	1.00E-04	-9.35E+00	0.0048	38	38.38%	6966.6	21.37%
GRHL2(CP2)/HBE-GRHL2-ChIP-Seq(GSE46194)	AAACYKGTTWDACMRGTTTB	1.00E-03	-9.12E+00	0.0053	48	48.48%	9868.3	30.27%
Sox7(HMG)/ESC-Sox7-ChIP-Seq(GSE133899)	VVRRAACAATGG	1.00E-03	-8.89E+00	0.006	50	50.51%	10549.3	32.35%
Sox17(HMG)/Endoderm-Sox17-ChIP-Seq(GSE61475)	CCATTGTTYB	1.00E-03	-8.65E+00	0.007	76	76.77%	19286.6	59.15%
ZNF382(Zf)/HEK293-ZNF382.GFP-ChIP-Seq(GSE58341)	GNCTGTASTRNTGBCTCHTT	1.00E-03	-8.57E+00	0.007	10	10.10%	812.5	2.49%
OCT:OCT-short(POU,Homeobox)/NPC-OCT6-ChIP-Seq(GSE43916)	ATGCATWATGCATRW	1.00E-03	-8.19E+00	0.0094	72	72.73%	18042.2	55.33%
SCRT1(Zf)/HEK293-SCRT1.eGFP-ChIP-Seq(Encode)	GCAACAGGTG	1.00E-03	-7.91E+00	0.0115	35	35.35%	6623.1	20.31%
GATA:SCL(Zf,bHLH)/Ter119-SCL-ChIP-Seq(GSE18720)	CRGCTGBNGNSNNSAGATAA	1.00E-03	-7.05E+00	0.0255	21	21.21%	3314.7	10.17%
Pdx1(Homeobox)/Islet-Pdx1-ChIP-Seq(SRA008281)	YCATYAATCA	1.00E-03	-7.05E+00	0.0255	78	78.79%	20763.1	63.68%
SPDEF(ETS)/VCaP-SPDEF-ChIP-Seq(SRA014231)	ASWTCCTGBT	1.00E-02	-6.80E+00	0.0289	75	75.76%	19777.9	60.66%
PRDM10(Zf)/HEK293-PRDM10.eGFP-ChIP-Seq(Encode)	TGGTACATTCCA	1.00E-02	-6.72E+00	0.0295	54	54.55%	12706.7	38.97%
ZNF189(Zf)/HEK293-ZNF189.GFP-ChIP-Seq(GSE58341)	TGGAACAGMA	1.00E-02	-6.58E+00	0.0322	68	68.69%	17405.1	53.38%
GSC(Homeobox)/FrogEmbryos-GSC-ChIP-Seq(DRA000576)	RGGATTAR	1.00E-02	-6.56E+00	0.0322	84	84.85%	23309.1	71.49%
TEAD(TEA)/Fibroblast-PU.1-ChIP-Seq(Unpublished)	YCWGGAATGY	1.00E-02	-6.03E+00	0.0502	61	61.62%	15316.2	46.97%
n-Myc(bHLH)/mES-nMyc-ChIP-Seq(GSE11431)	VRCCACGTGG	1.00E-02	-6.02E+00	0.0502	52	52.53%	12414.4	38.07%
Cdx2(Homeobox)/mES-Cdx2-ChIP-Seq(GSE14586)	GYMATAAAAH	1.00E-02	-5.99E+00	0.0502	69	69.70%	18059	55.39%
E2F6(E2F)/Hela-E2F6-ChIP-Seq(GSE31477)	GGCGGGAARN	1.00E-02	-5.98E+00	0.0502	33	33.33%	6783.5	20.80%
CREB5(bZIP)/LNCaP-CREB5.V5-ChIP-Seq(GSE137775)	VVATGACGTCAT	1.00E-02	-5.89E+00	0.0502	46	46.46%	10622.7	32.58%
ZEB1(Zf)/PDAC-ZEB1-ChIP-Seq(GSE64557)	VCAGGTRDRY	1.00E-02	-5.80E+00	0.051	78	78.79%	21390	65.60%
PBX2(Homeobox)/K562-PBX2-ChIP-Seq(Encode)	RTGATTKATRGN	1.00E-02	-5.79E+00	0.051	73	73.74%	19577.1	60.04%
Snail1(Zf)/LS174T-SNAIL1.HA-ChIP-Seq(GSE127183)	TRCACCTGCY	1.00E-02	-5.78E+00	0.051	54	54.55%	13174.1	40.40%
PRDM15(Zf)/ESC-Prdm15-ChIP-Seq(GSE73694)	YCCDNTCCAGGTTTT	1.00E-02	-5.77E+00	0.051	73	73.74%	19586.5	60.07%
Foxa2(Forkhead)/Liver-Foxa2-ChIP-Seq(GSE25694)	CYTGTTTACWYW	1.00E-02	-5.76E+00	0.051	75	75.76%	20314.2	62.30%
Sox2(HMG)/mES-Sox2-ChIP-Seq(GSE11431)	BCCATTGTTC	1.00E-02	-5.67E+00	0.051	78	78.79%	21459.7	65.82%
NFkB-p65(RHD)/GM12787-p65-ChIP-Seq(GSE19485)	WGGGGATTTCCC	1.00E-02	-5.67E+00	0.051	45	45.45%	10426.7	31.98%
Elf4(ETS)/BMDM-Elf4-ChIP-Seq(GSE88699)	ACTTCCKGKT	1.00E-02	-5.67E+00	0.051	73	73.74%	19643.2	60.24%
Srebp2(bHLH)/HepG2-Srebp2-ChIP-Seq(GSE31477)	CGGTCACSCCAC	1.00E-02	-5.54E+00	0.051	17	17.17%	2765.1	8.48%
ZNF467(Zf)/HEK293-ZNF467.GFP-ChIP-Seq(GSE58341)	TGGGGAAGGGCM	1.00E-02	-5.53E+00	0.051	58	58.59%	14596.5	44.77%
DUX4(Homeobox)/Myoblasts-DUX4.V5-ChIP-Seq(GSE75791)	NWTAAYCYAATCAWN	1.00E-02	-5.52E+00	0.051	13	13.13%	1850.4	5.68%
FOXP1(Forkhead)/H9-FOXP1-ChIP-Seq(GSE31006)	NYYTGTTTACHN	1.00E-02	-5.51E+00	0.051	56	56.57%	13959.8	42.81%
bHLHE41(bHLH)/proB-Bhlhe41-ChIP-Seq(GSE93764)	KCACGTGMCN	1.00E-02	-5.51E+00	0.051	63	63.64%	16267.1	49.89%
Oct6(POU,Homeobox)/NPC-Pou3f1-ChIP-Seq(GSE35496)	WATGCAAATGAG	1.00E-02	-5.48E+00	0.051	52	52.53%	12697.9	38.94%
Atf7(bZIP)/3T3L1-Atf7-ChIP-Seq(GSE56872)	NGRTGACGTCAY	1.00E-02	-5.37E+00	0.0511	47	47.47%	11189.4	34.32%
Hnf6b(Homeobox)/LNCaP-Hnf6b-ChIP-Seq(GSE106305)	TATTGAYY	1.00E-02	-5.34E+00	0.0514	72	72.73%	19466.2	59.70%
Tcf21(bHLH)/ArterySmoothMuscle-Tcf21-ChIP-Seq(GSE61369)	NAACAGCTGG	1.00E-02	-5.28E+00	0.0533	64	64.65%	16732.2	51.32%
RUNX(Runt)/HPC7-Runx1-ChIP-Seq(GSE22178)	SAAACCACAG	1.00E-02	-5.25E+00	0.0535	61	61.62%	15742.3	48.28%
RUNX2(Runt)/PCa-RUNX2-ChIP-Seq(GSE33889)	NWAACCACADNN	1.00E-02	-5.23E+00	0.0535	68	68.69%	18127.7	55.60%
RUNX-AML(Runt)/CD4+-PolII-ChIP-Seq(Barski_et_al.)	GCTGTGGTTW	1.00E-02	-5.23E+00	0.0535	62	62.63%	16089.6	49.35%

Bcl11a(Zf)/HSPC-BCL11A-ChIP-Seq(GSE104676)	TYTGACCASWRG	1.00E-02	-5.20E+00	0.0535	56	56.57%	14128.4	43.33%
EAR2(NR)/K562-NR2F6-ChIP-Seq(Encode)	NRBCARRGGTCA	1.00E-02	-5.18E+00	0.0535	76	76.77%	20997.1	64.40%
CArG(MADS)/PUER-Srf-ChIP-Seq(Sullivan_et_al.)	CCATATATGGNM	1.00E-02	-5.14E+00	0.0538	38	38.38%	8593.8	26.36%
LEF1(HMG)/H1-LEF1-ChIP-Seq(GSE64758)	CCTTTGATST	1.00E-02	-5.10E+00	0.055	66	66.67%	17520.2	53.73%
HIC1(Zf)/Treg-ZBTB29-ChIP-Seq(GSE99889)	TGCCAGCB	1.00E-02	-5.00E+00	0.0593	85	85.86%	24492.1	75.12%
Duxbl(Homeobox)/NIH3T3-Duxbl.HA-ChIP-Seq(GSE119782)	TAAYCYAATCAA	1.00E-02	-4.98E+00	0.0593	15	15.15%	2441.1	7.49%
c-Myc(bHLH)/mES-cMyc-ChIP-Seq(GSE11431)	VVCCACGTGG	1.00E-02	-4.89E+00	0.0637	42	42.42%	9915.3	30.41%
Unknown-ESC-element(?)/mES-Nanog-ChIP-Seq(GSE11724)	CACAGCAGGGGG	1.00E-02	-4.89E+00	0.0637	45	45.45%	10831	33.22%
NFkB-p50,p52(RHD)/Monocyte-p50-ChIP-Chip(Schreiber_et_al.)	GGGGGAATCCCC	1.00E-02	-4.88E+00	0.0637	12	12.12%	1774.9	5.44%
ISRE(IRF)/ThioMac-LPS-Expression(GSE23622)	AGTTTCASTTTC	1.00E-02	-4.87E+00	0.0637	14	14.14%	2236.4	6.86%
Ap4(bHLH)/AML-Tfap4-ChIP-Seq(GSE45738)	NAHCAGCTGD	1.00E-02	-4.87E+00	0.0637	72	72.73%	19741.1	60.55%
OCT:OCT(POU,Homeobox)/NPC-Brn1-ChIP-Seq(GSE35496)	ATGAATATTCATGAG	1.00E-02	-4.83E+00	0.0637	5	5.05%	404.2	1.24%
Zic2(Zf)/ESC-Zic2-ChIP-Seq(SRP197560)	CHCAGCRGGRGG	1.00E-02	-4.80E+00	0.0637	35	35.35%	7880.8	24.17%
ELF5(ETS)/T47D-ELF5-ChIP-Seq(GSE30407)	ACVAGGAAGT	1.00E-02	-4.75E+00	0.0645	64	64.65%	17044.7	52.28%
Pitx1:Ebox(Homeobox,bHLH)/Hindlimb-Pitx1-ChIP-Seq(GSE41591)	YTAATTRAWWCCAGATGT	1.00E-02	-4.69E+00	0.0672	31	31.31%	6779.7	20.79%
Tbx20(T-box)/Heart-Tbx20-ChIP-Seq(GSE29636)	GGTGYTGACAGS	1.00E-02	-4.69E+00	0.0672	27	27.27%	5656.5	17.35%
Tbx21(T-box)/GM12878-TBX21-ChIP-Seq(Encode)	AGGTGTGAAA	1.00E-02	-4.66E+00	0.0672	70	70.71%	19161.9	58.77%
c-Jun-CRE(bZIP)/K562-cJun-ChIP-Seq(GSE31477)	ATGACGTCATCY	1.00E-02	-4.62E+00	0.0691	35	35.35%	7972.5	24.45%
Oct4:Sox17(POU,Homeobox,HMG)/F9-Sox17-ChIP-Seq(GSE44553)	CCATTGTATGCAAAT	1.00E-01	-4.56E+00	0.0718	27	27.27%	5713.7	17.52%
Foxa3(Forkhead)/Liver-Foxa3-ChIP-Seq(GSE77670)	BSNTGTTTACWYWGN	1.00E-01	-4.55E+00	0.0718	47	47.47%	11641.7	35.70%
Pit1+1bp(Homeobox)/GCrat-Pit1-ChIP-Seq(GSE58009)	ATGCATAATTCA	1.00E-01	-4.55E+00	0.0718	46	46.46%	11330.3	34.75%
Smad2(MAD)/ES-SMAD2-ChIP-Seq(GSE29422)	CTGTCTGG	1.00E-01	-4.51E+00	0.0721	81	81.82%	23229.8	71.25%
IRF3(IRF)/BMDM-Irf3-ChIP-Seq(GSE67343)	AGTTTCAKTTTC	1.00E-01	-4.47E+00	0.0743	44	44.44%	10758.5	33.00%
Oct11(POU,Homeobox)/NCIH1048-POU2F3-ChIP-seq(GSE115123)	GATTTGCATA	1.00E-01	-4.46E+00	0.0743	43	43.43%	10454.1	32.06%
ELF3(ETS)/PDAC-ELF3-ChIP-Seq(GSE64557)	ANCAGGAAGT	1.00E-01	-4.44E+00	0.0745	63	63.64%	16902.4	51.84%
Pax7(Paired,Homeobox)/Myoblast-Pax7-ChIP-Seq(GSE25064)	TAATCAATTA	1.00E-01	-4.43E+00	0.0745	19	19.19%	3614	11.08%
EBF(EBF)/proBcell-EBF-ChIP-Seq(GSE21978)	DGTCCCYRGGGA	1.00E-01	-4.40E+00	0.0753	22	22.22%	4417.9	13.55%
Nkx2.2(Homeobox)/NPC-Nkx2.2-ChIP-Seq(GSE61673)	BTBRAGTGSN	1.00E-01	-4.39E+00	0.0753	89	89.90%	26418.7	81.02%
Tbet(T-box)/CD8-Tbet-ChIP-Seq(GSE33802)	AGGTGTGAAM	1.00E-01	-4.37E+00	0.0754	72	72.73%	20046.1	61.48%
WT1(Zf)/Kidney-WT1-ChIP-Seq(GSE90016)	MCTCCCMCRCAB	1.00E-01	-4.37E+00	0.0754	43	43.43%	10509.4	32.23%
ZNF136(Zf)/HEK293-ZNF136.GFP-ChIP-Seq(GSE58341)	YTKGATAHAGTATTCTWGGTNGGCA	1.00E-01	-4.36E+00	0.0754	19	19.19%	3638.9	11.16%
Atf2(bZIP)/3T3L1-Atf2-ChIP-Seq(GSE56872)	NRRTGACGTCAT	1.00E-01	-4.36E+00	0.0754	36	36.36%	8400.7	25.76%
OCT4-SOX2-TCF-NANOG(POU,Homeobox,HMG)/mES-Oct4-ChIP-Seq(GSE11431)	ATTTGCATAACAATG	1.00E-01	-4.31E+00	0.0756	32	32.32%	7252.4	22.24%
Otx2(Homeobox)/EpiLC-Otx2-ChIP-Seq(GSE56098)	NYTAATCCYB	1.00E-01	-4.30E+00	0.0757	68	68.69%	18694.5	57.34%
JunD(bZIP)/K562-JunD-ChIP-Seq	ATGACGTCATCN	1.00E-01	-4.28E+00	0.0763	15	15.15%	2649.9	8.13%
MyoG(bHLH)/C2C12-MyoG-ChIP-Seq(GSE36024)	AACAGCTG	1.00E-01	-4.28E+00	0.0763	63	63.64%	17003.3	52.15%
ZNF415(Zf)/HEK293-ZNF415.GFP-ChIP-Seq(GSE58341)	GRTGMTRGAGCC	1.00E-01	-4.26E+00	0.0763	40	40.40%	9655.2	29.61%
RORgt(NR)/EL4-RORgt.Flag-ChIP-Seq(GSE56019)	AAYTAGGTCA	1.00E-01	-4.16E+00	0.082	22	22.22%	4515.7	13.85%
RORgt(NR)/EL4-RORgt.Flag-ChIP-Seq(GSE56019)	AAYTAGGTCA	1.00E-01	-4.16E+00	0.082	22	22.22%	4515.7	13.85%
HRE(HSF)/HepG2-HSF1-ChIP-Seq(GSE31477)	BSTTCTRGAABVTTCYAGAA	1.00E-01	-4.13E+00	0.0832	21	21.21%	4256.1	13.05%
BHLHA15(bHLH)/NIH3T3-BHLHB8.HA-ChIP-Seq(GSE119782)	NAMCAGCTGK	1.00E-01	-4.12E+00	0.0832	77	77.78%	21991.6	67.45%
ZEB2(Zf)/SNU398-ZEB2-ChIP-Seq(GSE103048)	GNMCAGGTGTGC	1.00E-01	-4.11E+00	0.0832	54	54.55%	14138.2	43.36%
Brn2(POU,Homeobox)/NPC-Brn2-ChIP-Seq(GSE35496)	ATGAATATTC	1.00E-01	-4.09E+00	0.0839	20	20.20%	4006.8	12.29%
Brn1(POU,Homeobox)/NPC-Brn1-ChIP-Seq(GSE35496)	TATGCWAATBAV	1.00E-01	-4.08E+00	0.0839	41	41.41%	10063.5	30.86%
Znf263(Zf)/K562-Znf263-ChIP-Seq(GSE31477)	CVGTSCTCCC	1.00E-01	-4.04E+00	0.0857	78	78.79%	22404.5	68.71%
p73(p53)/Trachea-p73-ChIP-Seq(PRJNA310161)	NRRRCAWGTCCDGRCATGYY	1.00E-01	-4.02E+00	0.0865	9	9.09%	1304.8	4.00%
RUNX1(Runt)/Jurkat-RUNX1-ChIP-Seq(GSE29180)	AAACCACARM	1.00E-01	-3.99E+00	0.0882	72	72.73%	20288.6	62.22%
Tbx6(T-box)/ESC-Tbx6-ChIP-Seq(GSE93524)	DAGGTGTBAA	1.00E-01	-3.97E+00	0.0896	68	68.69%	18912.6	58.00%
Max(bHLH)/K562-Max-ChIP-Seq(GSE31477)	RCCACGTGGYYN	1.00E-01	-3.96E+00	0.0896	51	51.52%	13269.2	40.70%
MafB(bZIP)/BMM-Mafb-ChIP-Seq(GSE75722)	WNTGCTGASTCAGCANWTTY	1.00E-01	-3.96E+00	0.0896	45	45.45%	11375.7	34.89%
MNT(bHLH)/HepG2-MNT-ChIP-Seq(Encode)	DGCACACGTG	1.00E-01	-3.91E+00	0.092	69	69.70%	19297.7	59.19%

ZSCAN22(Zf)/HEK293-ZSCAN22.GFP-ChIP-Seq(GSE58341)	SMCAGTCWGAKGGAGGAGGC	1.00E-01	-3.87E+00	0.095	10	10.10%	1568	4.81%
p63(p53)/Keratinocyte-p63-ChIP-Seq(GSE17611)	NNDRCATGYCYNRRCATGYH	1.00E-01	-3.84E+00	0.0961	35	35.35%	8388.6	25.73%
Zac1(Zf)/Neuro2A-Plagl1-ChIP-Seq(GSE75942)	HAWGRGGCCM	1.00E-01	-3.82E+00	0.0975	88	88.89%	26331.4	80.76%
Nur77(NR)/K562-NR4A1-ChIP-Seq(GSE31363)	TGACCTTTNCNT	1.00E-01	-3.80E+00	0.0987	27	27.27%	6075.5	18.63%
TCF4(bHLH)/SHSY5Y-TCF4-ChIP-Seq(GSE96915)	SMCATCTGKH	1.00E-01	-3.76E+00	0.1017	80	80.81%	23321.9	71.53%
EWS:FLI1-fusion(ETS)/SK_N_MC-EWS:FLI1-ChIP-Seq(SRA014231)	VACAGGAAAT	1.00E-01	-3.73E+00	0.1032	52	52.53%	13742.2	42.15%
Foxf1(Forkhead)/Lung-Foxf1-ChIP-Seq(GSE77951)	WWATRTAAACAN	1.00E-01	-3.72E+00	0.1037	78	78.79%	22615.3	69.36%
FOXM1(Forkhead)/MCF7-FOXM1-ChIP-Seq(GSE72977)	TRTTTACTTW	1.00E-01	-3.68E+00	0.1064	81	81.82%	23739.8	72.81%
CUX1(Homeobox)/K562-CUX1-ChIP-Seq(GSE92882)	TATCGATNAN	1.00E-01	-3.66E+00	0.1077	60	60.61%	16414	50.34%
COUP-TFII(NR)/K562-NR2F1-ChIP-Seq(Encode)	GKBCARAGGTCA	1.00E-01	-3.66E+00	0.1077	77	77.78%	22293.1	68.37%
PAX6(Paired,Homeobox)/Forebrain-Pax6-ChIP-Seq(GSE66961)	NGTGTTCAVTSAAGCGKAAA	1.00E-01	-3.62E+00	0.1099	14	14.14%	2616.4	8.02%
NeuroD1(bHLH)/Islet-NeuroD1-ChIP-Seq(GSE30298)	GCCATCTGTT	1.00E-01	-3.62E+00	0.1099	56	56.57%	15122.3	46.38%
HEB(bHLH)/mES-Heb-ChIP-Seq(GSE53233)	VCAGCTGBNN	1.00E-01	-3.61E+00	0.1099	78	78.79%	22691.1	69.59%
Six1(Homeobox)/Myoblast-Six1-ChIP-Chip(GSE20150)	GKVTCADRTTWC	1.00E-01	-3.54E+00	0.1161	30	30.30%	7083.7	21.73%
NF1-halfsite(CTF)/LNCaP-NF1-ChIP-Seq(Unpublished)	YTGCCAAG	1.00E-01	-3.53E+00	0.1164	82	82.83%	24211.7	74.26%
Ascl1(bHLH)/NeuralTubes-Ascl1-ChIP-Seq(GSE55840)	NNVVCAGCTGBN	1.00E-01	-3.52E+00	0.1165	73	73.74%	20964.6	64.30%
Zic(Zf)/Cerebellum-ZIC1.2-ChIP-Seq(GSE60731)	CCTGCTGAGH	1.00E-01	-3.52E+00	0.1165	55	55.56%	14867.3	45.60%
HNF4a(NR),DR1/HepG2-HNF4a-ChIP-Seq(GSE25021)	CARRGKBCAAAGTYCA	1.00E-01	-3.49E+00	0.1172	45	45.45%	11677.3	35.81%
CEBP(bZIP)/ThioMac-CEBPb-ChIP-Seq(GSE21512)	ATTGCGCAAC	1.00E-01	-3.47E+00	0.1197	58	58.59%	15890.5	48.74%
DMRT6(DM)/Testis-DMRT6-ChIP-Seq(GSE60440)	YDGHTACAWTGTADC	1.00E-01	-3.44E+00	0.1213	32	32.32%	7730.4	23.71%
Zic3(Zf)/mES-Zic3-ChIP-Seq(GSE37889)	GGCCYCCTGCTGDGH	1.00E-01	-3.42E+00	0.1235	37	37.37%	9252.9	28.38%
FOXK2(Forkhead)/U2OS-FOXK2-ChIP-Seq(E-MTAB-2204)	SCHTGTTTACAT	1.00E-01	-3.38E+00	0.127	62	62.63%	17285.8	53.01%
Sox6(HMG)/Myotubes-Sox6-ChIP-Seq(GSE32627)	CCATTGTTNY	1.00E-01	-3.34E+00	0.131	90	90.91%	27404.1	84.05%
ZNF341(Zf)/EBV-ZNF341-ChIP-Seq(GSE113194)	GGAACAGCCG	1.00E-01	-3.32E+00	0.1321	52	52.53%	14031.9	43.04%
USF1(bHLH)/GM12878-Usf1-ChIP-Seq(GSE32465)	SGTCACGTGR	1.00E-01	-3.30E+00	0.1348	36	36.36%	9026.1	27.68%
FOXK1(Forkhead)/HEK293-FOXK1-ChIP-Seq(GSE51673)	NVWTGTTTAC	1.00E-01	-3.29E+00	0.1348	79	79.80%	23271.3	71.37%
Ascl2(bHLH)/ESC-Ascl2-ChIP-Seq(GSE97712)	SSRGCAGCTGCH	1.00E-01	-3.28E+00	0.1348	60	60.61%	16687.8	51.18%
Gata1(Zf)/K562-GATA1-ChIP-Seq(GSE18829)	SAGATAAGRV	1.00E-01	-3.28E+00	0.1348	52	52.53%	14062.9	43.13%
GLIS3(Zf)/Thyroid-Glis3.GFP-ChIP-Seq(GSE103297)	CTCCCTGGGAGGCCN	1.00E-01	-3.28E+00	0.1348	61	61.62%	17025.4	52.22%
ERRg(NR)/Kidney-ESRRG-ChIP-Seq(GSE104905)	GTGACCTTGRVN	1.00E-01	-3.28E+00	0.1348	59	59.60%	16360.1	50.18%
NFAT(RHD)/Jurkat-NFATC1-ChIP-Seq(Jolma_et_al.)	ATTTTCCATT	1.00E-01	-3.27E+00	0.1348	67	67.68%	19064	58.47%
Oct2(POU,Homeobox)/Bcell-Oct2-ChIP-Seq(GSE21512)	ATATGCAAAT	1.00E-01	-3.26E+00	0.1348	38	38.38%	9662.2	29.63%
Phox2b(Homeobox)/CLBGA-PHOX2B-ChIP-Seq(GSE90683)	TTAATTNAATTA	1.00E-01	-3.26E+00	0.1348	32	32.32%	7845	24.06%
FOXA1:AR(Forkhead,NR)/LNCAP-AR-ChIP-Seq(GSE27824)	AGTAAACAAAAAAGAACAND	1.00E-01	-3.25E+00	0.1348	16	16.16%	3282.1	10.07%
Unknown(Homeobox)/Limb-p300-ChIP-Seq	SSCMATWAAA	1.00E-01	-3.25E+00	0.1348	61	61.62%	17048.9	52.29%
E2A(bHLH),near_PU.1/Bcell-PU.1-ChIP-Seq(GSE21512)	NVCACCTGBN	1.00E-01	-3.23E+00	0.1348	64	64.65%	18069.6	55.42%
Six2(Homeobox)/NephronProgenitor-Six2-ChIP-Seq(GSE39837)	GWAAYHTGAKMC	1.00E-01	-3.23E+00	0.1348	72	72.73%	20823.5	63.86%
NFIL3(bZIP)/HepG2-NFIL3-ChIP-Seq(Encode)	VTTACGTAAYNNNNN	1.00E-01	-3.21E+00	0.1348	60	60.61%	16745.2	51.36%
Gata2(Zf)/K562-GATA2-ChIP-Seq(GSE18829)	BBCTTATCTS	1.00E-01	-3.19E+00	0.1348	56	56.57%	15438.7	47.35%
CEBP:AP1(bZIP)/ThioMac-CEBPb-ChIP-Seq(GSE21512)	DRTGTTGCAA	1.00E-01	-3.18E+00	0.1348	61	61.62%	17099.3	52.44%
VDR(NR),DR3/GM10855-VDR+vitD-ChIP-Seq(GSE22484)	ARAGGTCANWGAGTTCANNN	1.00E-01	-3.17E+00	0.1348	22	22.22%	4975.1	15.26%
Esrrb(NR)/mES-Esrrb-ChIP-Seq(GSE11431)	KTGACCTTGA	1.00E-01	-3.17E+00	0.1348	53	53.54%	14471.8	44.38%
FXR(NR),IR1/Liver-FXR-ChIP-Seq(Chong_et_al.)	AGGTCANTGACCTB	1.00E-01	-3.14E+00	0.1369	33	33.33%	8218.7	25.21%
FoxL2(Forkhead)/Ovary-FoxL2-ChIP-Seq(GSE60858)	WWTRTAAACAVG	1.00E-01	-3.13E+00	0.1378	74	74.75%	21600	66.25%
ETS:E-box(ETS,bHLH)/HPC7-Scl-ChIP-Seq(GSE22178)	AGGAARCAGCTG	1.00E-01	-3.11E+00	0.1398	14	14.14%	2810.7	8.62%
Twist(bHLH)/HMLE-TWIST1-ChIP-Seq(Chang_et_al)	VCAKCTGGNNNCCAGMTGBN	1.00E-01	-3.11E+00	0.1398	15	15.15%	3076.7	9.44%
EBNA1(EBV-virus)/Raji-EBNA1-ChIP-Seq(GSE30709)	GGYAGCAYDTGCTDCCCNNN	1.00E-01	-3.09E+00	0.1403	4	4.04%	440.7	1.35%
ZNF416(Zf)/HEK293-ZNF416.GFP-ChIP-Seq(GSE58341)	WDNCTGGGCA	1.00E-01	-3.08E+00	0.1403	71	71.72%	20584.2	63.13%
ZNF7(Zf)/HepG2-ZNF7.Flag-ChIP-Seq(Encode)	CTGCCWVCTTTTRTA	1.00E-01	-3.07E+00	0.1415	53	53.54%	14550.1	44.62%
Tcf7(HMG)/GM12878-TCF7-ChIP-Seq(Encode)	CTTTGATGTGSB	1.00E-01	-3.03E+00	0.1463	39	39.39%	10132.2	31.08%
EBF2(EBF)/BrownAdipose-EBF2-ChIP-Seq(GSE97114)	NABTCCCWDGGGAVH	1.00E-01	-3.01E+00	0.148	58	58.59%	16237.3	49.80%

TEAD4(TEA)/Tropoblast-Tead4-ChIP-Seq(GSE37350)	CCWGGAATGY	1.00E-01	-3.01E+00	0.148	61	61.62%	17237	52.87%
Nr5a2(NR)/Pancreas-LRH1-ChIP-Seq(GSE34295)	BTCAAGGTCA	1.00E-01	-2.98E+00	0.1506	55	55.56%	15273.6	46.84%
Srebp1a(bHLH)/HepG2-Srebp1a-ChIP-Seq(GSE31477)	RTCACSCCAY	1.00E-01	-2.95E+00	0.1532	20	20.20%	4525	13.88%
FOXA1(Forkhead)/MCF7-FOXA1-ChIP-Seq(GSE26831)	WAAGTAAACA	1.00E-01	-2.94E+00	0.154	79	79.80%	23521.7	72.14%
Gfi1b(Zf)/HPC7-Gfi1b-ChIP-Seq(GSE22178)	MAATCACTGC	1.00E-01	-2.92E+00	0.1556	54	54.55%	14988.1	45.97%
STAT1(Stat)/HelaS3-STAT1-ChIP-Seq(GSE12782)	NATTTCCNGGAAAT	1.00E-01	-2.90E+00	0.1591	36	36.36%	9299.3	28.52%
E2A(bHLH)/proBcell-E2A-ChIP-Seq(GSE21978)	DNRCAGCTGY	1.00E-01	-2.89E+00	0.1591	67	67.68%	19358.1	59.37%
IRF8(IRF)/BMDM-IRF8-ChIP-Seq(GSE77884)	GRAASTGAAAST	1.00E-01	-2.88E+00	0.1596	38	38.38%	9928.9	30.45%
MyoD(bHLH)/Myotube-MyoD-ChIP-Seq(GSE21614)	RRCAGCTGYTSY	1.00E-01	-2.84E+00	0.1646	51	51.52%	14078.1	43.18%
Elk4(ETS)/Hela-Elk4-ChIP-Seq(GSE31477)	NRYTTCCGGY	1.00E-01	-2.83E+00	0.1651	43	43.43%	11527.2	35.35%
Slug(Zf)/Mesoderm-Snai2-ChIP-Seq(GSE61475)	SNGCACCTGCHS	1.00E-01	-2.83E+00	0.1651	35	35.35%	9038.9	27.72%
Tlx?(NR)/NPC-H3K4me1-ChIP-Seq(GSE16256)	CTGGCAGSCTGCCA	1.00E-01	-2.82E+00	0.1653	35	35.35%	9047.8	27.75%
CDX4(Homeobox)/ZebrafishEmbryos-Cdx4.Myc-ChIP-Seq(GSE48254)	NGYCATAAAWCH	1.00E-01	-2.81E+00	0.1653	71	71.72%	20794.7	63.78%
NFY(CCAAT)/Promoter	RGCCAATSRG	1.00E-01	-2.79E+00	0.1674	56	56.57%	15750.5	48.31%
PSE(SNAPc)/K562-mStart-Seq	WAVTCACCMTAASYDAAAAG	1.00E-01	-2.78E+00	0.1686	53	53.54%	14778.1	45.32%
NPAS(bHLH)/Liver-NPAS-ChIP-Seq(GSE39860)	NVCACGTG	1.00E-01	-2.75E+00	0.1731	81	81.82%	24388.3	74.80%
ZNF16(Zf)/HEK293-ZNF16.GFP-ChIP-Seq(GSE58341)	MACCTTCYATGGCTCCCTAKTGCCY	1.00E-01	-2.74E+00	0.1736	2	2.02%	136.2	0.42%
TEAD1(TEAD)/HepG2-TEAD1-ChIP-Seq(Encode)	CYRCATTCCA	1.00E-01	-2.73E+00	0.1736	66	66.67%	19144.1	58.71%
NFAT:AP1(RHD,bZIP)/Jurkat-NFATC1-ChIP-Seq(Jolma_et_al.)	SARTGGAAAAWRTGAGTCAB	1.00E-01	-2.72E+00	0.174	23	23.23%	5515.4	16.92%
Foxo3(Forkhead)/U2OS-Foxo3-ChIP-Seq(E-MTAB-2701)	DGTAAACA	1.00E-01	-2.71E+00	0.1758	67	67.68%	19507.8	59.83%
KLF10(Zf)/HEK293-KLF10.GFP-ChIP-Seq(GSE58341)	GGGGGTGTGTCC	1.00E-01	-2.68E+00	0.1805	46	46.46%	12605.6	38.66%
Sox9(HMG)/Limb-SOX9-ChIP-Seq(GSE73225)	AGGVNCCTTTGT	1.00E-01	-2.63E+00	0.1885	70	70.71%	20604.6	63.19%
DLX5(Homeobox)/BasalGanglia-Dlx5-ChIP-seq(GSE124936)	SSTAATTA	1.00E-01	-2.59E+00	0.1935	66	66.67%	19263.2	59.08%
Sox4(HMG)/proB-Sox4-ChIP-Seq(GSE50066)	YCTTTGTTCC	1.00E-01	-2.59E+00	0.1935	71	71.72%	20977.2	64.34%
GATA3(Zf),DR4/iTreg-Gata3-ChIP-Seq(GSE20898)	AGATGKDGAGATAAG	1.00E-01	-2.58E+00	0.1937	13	13.13%	2766.9	8.49%
PPARE(NR),DR1/3T3L1-Pparg-ChIP-Seq(GSE13511)	TGACCTTTGCCCCA	1.00E-01	-2.57E+00	0.1946	61	61.62%	17598.5	53.97%
ERE(NR),IR3/MCF7-ERa-ChIP-Seq(Unpublished)	VAGGTCACNSTGACC	1.00E-01	-2.54E+00	0.1989	26	26.26%	6516.2	19.98%
OCT:OCT(POU,Homeobox)/NPC-OCT6-ChIP-Seq(GSE43916)	YATGCATATRCATRT	1.00E-01	-2.54E+00	0.1989	23	23.23%	5631.8	17.27%
Smad4(MAD)/ESC-SMAD4-ChIP-Seq(GSE29422)	VBSYGTCTGG	1.00E-01	-2.54E+00	0.1989	79	79.80%	23831.1	73.09%
HLF(bZIP)/HSC-HLF.Flag-ChIP-Seq(GSE69817)	RTTATGYAAB	1.00E-01	-2.50E+00	0.204	67	67.68%	19684.9	60.37%
PU.1-IRF(ETS:IRF)/Bcell-PU.1-ChIP-Seq(GSE21512)	MGGAAGTGAAAC	1.00E-01	-2.50E+00	0.204	80	80.81%	24221.4	74.29%
NF1(CTF)/LNCAP-NF1-ChIP-Seq(Unpublished)	CYTGGCABNSTGCCAR	1.00E-01	-2.47E+00	0.2071	32	32.32%	8381.5	25.71%
Sox15(HMG)/CPA-Sox15-ChIP-Seq(GSE62909)	RAACAATGGN	1.00E-01	-2.47E+00	0.2071	79	79.80%	23883.3	73.25%
JunB(bZIP)/DendriticCells-Junb-ChIP-Seq(GSE36099)	RATGASTCAT	1.00E-01	-2.44E+00	0.2112	48	48.48%	13443.5	41.23%
Tbr1(T-box)/Cortex-Tbr1-ChIP-Seq(GSE71384)	AAGGTGTKAA	1.00E-01	-2.42E+00	0.2141	79	79.80%	23922.1	73.37%
AP-2alpha(AP2)/Hela-AP2alpha-ChIP-Seq(GSE31477)	ATGCCCTGAGGC	1.00E-01	-2.42E+00	0.2141	40	40.40%	10911	33.46%
COUP-TFII(NR)/Artia-Nr2f2-ChIP-Seq(GSE46497)	AGRGGTCA	1.00E-01	-2.41E+00	0.2151	78	78.79%	23580.4	72.32%
PRDM9(Zf)/Testis-DMC1-ChIP-Seq(GSE35498)	ADGGYAGYAGCATCT	1.00E-01	-2.39E+00	0.2174	34	34.34%	9061.7	27.79%
PPARa(NR),DR1/Liver-Ppara-ChIP-Seq(GSE47954)	VNAGGKCAAAGGTCA	1.00E-01	-2.39E+00	0.2174	66	66.67%	19442.2	59.63%
Gata4(Zf)/Heart-Gata4-ChIP-Seq(GSE35151)	NBWGATAAGR	1.00E-01	-2.38E+00	0.2182	70	70.71%	20818.3	63.85%
YY1(Zf)/Promoter	CAAGATGGCGGC	1.00E-01	-2.33E+00	0.2275	8	8.08%	1542.9	4.73%
TEAD2(TEA)/Py2T-Tead2-ChIP-Seq(GSE55709)	CCWGGAATGY	1.00E-01	-2.32E+00	0.2297	44	44.44%	12272.2	37.64%
ZNF652/HepG2-ZNF652.Flag-ChIP-Seq(Encode)	TTAACCCTTTVNKKN	1.00E+00	-2.30E+00	0.233	31	31.31%	8212.7	25.19%
STAT5(Stat)/mCD4+-Stat5-ChIP-Seq(GSE12346)	RTTTCTNAGAAA	1.00E+00	-2.28E+00	0.2356	38	38.38%	10402.8	31.90%
PU.1:IRF8(ETS:IRF)/pDC-Irf8-ChIP-Seq(GSE66899)	GGAAGTGAAAST	1.00E+00	-2.26E+00	0.2403	25	25.25%	6418.4	19.68%
Twist2(bHLH)/Myoblast-Twist2.Ty1-ChIP-Seq(GSE127998)	MCAGCTGBYH	1.00E+00	-2.25E+00	0.2403	81	81.82%	24776.8	75.99%
TEAD3(TEA)/HepG2-TEAD3-ChIP-Seq(Encode)	TRCATTCCAG	1.00E+00	-2.25E+00	0.2403	71	71.72%	21279.1	65.26%
NeuroG2(bHLH)/Fibroblast-NeuroG2-ChIP-Seq(GSE75910)	ACCATCTGTT	1.00E+00	-2.24E+00	0.2415	78	78.79%	23727	72.77%
KLF14(Zf)/HEK293-KLF14.GFP-ChIP-Seq(GSE58341)	RGKGGGCGKGGC	1.00E+00	-2.23E+00	0.2415	64	64.65%	18911.2	58.00%
BMAL1(bHLH)/Liver-Bmal1-ChIP-Seq(GSE39860)	GNCACGTG	1.00E+00	-2.22E+00	0.2426	83	83.84%	25522.4	78.28%
Atf4(bZIP)/MEF-Atf4-ChIP-Seq(GSE35681)	MTGATGCAAT	1.00E+00	-2.22E+00	0.2426	29	29.29%	7661.7	23.50%

Npas4(bHLH)/Neuron-Npas4-ChIP-Seq(GSE127793)	NHRTCACGACDN	1.00E+00	-2.19E+00	0.2478	48	48.48%	13675.9	41.94%
ZNF711(Zf)/SHSY5Y-ZNF711-ChIP-Seq(GSE20673)	AGGCCTAG	1.00E+00	-2.18E+00	0.2493	69	69.70%	20659.8	63.36%
Nkx2.5(Homeobox)/HL1-Nkx2.5.biotin-ChIP-Seq(GSE21529)	RRSCACTYAA	1.00E+00	-2.16E+00	0.2523	90	90.91%	28147.8	86.33%
HOXB13(Homeobox)/ProstateTumor-HOXB13-ChIP-Seq(GSE56288)	TTTTATKRGG	1.00E+00	-2.16E+00	0.2523	75	75.76%	22746.2	69.76%
Atf3(bZIP)/GBM-ATF3-ChIP-Seq(GSE33912)	DATGASTCATHN	1.00E+00	-2.15E+00	0.253	53	53.54%	15341.6	47.05%
Atf1(bZIP)/K562-ATF1-ChIP-Seq(GSE31477)	GATGACGTCA	1.00E+00	-2.14E+00	0.253	51	51.52%	14691.3	45.06%
BORIS(Zf)/K562-CTCFL-ChIP-Seq(GSE32465)	CNNBRGCGCCCCCTGSTGGC	1.00E+00	-2.14E+00	0.253	15	15.15%	3549.8	10.89%
BATF(bZIP)/Th17-BATF-ChIP-Seq(GSE39756)	DATGASTCAT	1.00E+00	-2.13E+00	0.254	53	53.54%	15359.4	47.11%
Nkx3.1(Homeobox)/LNCaP-Nkx3.1-ChIP-Seq(GSE28264)	AAGCACTTAA	1.00E+00	-2.13E+00	0.254	92	92.93%	28935.3	88.74%
RBPJ:Ebox(?,bHLH)/Panc1-Rbpj1-ChIP-Seq(GSE47459)	GGGRAARRGRMCAGMTG	1.00E+00	-2.11E+00	0.2576	28	28.28%	7444.8	22.83%
Tcfcp2l1(CP2)/mES-Tcfcp2l1-ChIP-Seq(GSE11431)	NRAACCRGTTYRAACCRGYT	1.00E+00	-2.09E+00	0.2594	16	16.16%	3866.1	11.86%
Oct4(POU,Homeobox)/mES-Oct4-ChIP-Seq(GSE11431)	ATTTGCATAW	1.00E+00	-2.09E+00	0.2594	48	48.48%	13767.4	42.22%
Chop(bZIP)/MEF-Chop-ChIP-Seq(GSE35681)	ATTGCATCAT	1.00E+00	-2.08E+00	0.2596	24	24.24%	6244.2	19.15%
FOXA1(Forkhead)/LNCAP-FOXA1-ChIP-Seq(GSE27824)	WAAGTAAACA	1.00E+00	-2.08E+00	0.2596	82	82.83%	25280.3	77.53%
DMRT1(DM)/Testis-DMRT1-ChIP-Seq(GSE64892)	TWGHWACAWTGTWDC	1.00E+00	-2.07E+00	0.2599	33	33.33%	9017.3	27.66%
PBX1(Homeobox)/MCF7-PBX1-ChIP-Seq(GSE28007)	GSCTGTCACTCA	1.00E+00	-2.07E+00	0.2599	10	10.10%	2181.1	6.69%
Gli2(Zf)/GM2-Gli2-ChIP-Chip(GSE112702)	YSTGGGTGGTCT	1.00E+00	-2.06E+00	0.2618	20	20.20%	5065.8	15.54%
GATA3(Zf),DR8/iTreg-Gata3-ChIP-Seq(GSE20898)	AGATSTNDNNDSAGATAASN	1.00E+00	-2.04E+00	0.2647	12	12.12%	2753.9	8.45%
MITF(bHLH)/MastCells-MITF-ChIP-Seq(GSE48085)	RTCATGTGAC	1.00E+00	-2.03E+00	0.2661	60	60.61%	17769.8	54.50%
Myf5(bHLH)/GM-Myf5-ChIP-Seq(GSE24852)	BAACAGCTGT	1.00E+00	-2.03E+00	0.2661	47	47.47%	13504.4	41.42%
GRE(NR),IR3/RAW264.7-GRE-ChIP-Seq(Unpublished)	VAGRACAKWCTGTYC	1.00E+00	-2.03E+00	0.2661	28	28.28%	7508.9	23.03%
NFkB-p65-Rel(RHD)/ThioMac-LPS-Expression(GSE23622)	GGAAATTCCC	1.00E+00	-2.02E+00	0.2661	7	7.07%	1395.3	4.28%
Hoxc9(Homeobox)/Ainv15-Hoxc9-ChIP-Seq(GSE21812)	GGCCATAAATCA	1.00E+00	-2.02E+00	0.2661	50	50.51%	14486	44.43%
STAT6(Stat)/Macrophage-Stat6-ChIP-Seq(GSE38377)	TTCCKNAGAA	1.00E+00	-2.01E+00	0.2661	51	51.52%	14822.2	45.46%
Lhx2(Homeobox)/HFSC-Lhx2-ChIP-Seq(GSE48068)	TAATTAGN	1.00E+00	-2.01E+00	0.2661	75	75.76%	22880.8	70.17%
HINFP(Zf)/K562-HINFP.eGFP-ChIP-Seq(Encode)	TWVGGTCCGC	1.00E+00	-2.00E+00	0.2669	13	13.13%	3061.1	9.39%
LRF(Zf)/Erythroblasts-ZBTB7A-ChIP-Seq(GSE74977)	AAGACCCYYN	1.00E+00	-1.98E+00	0.2713	58	58.59%	17161.3	52.63%
CTCF-SatelliteElement(Zf?)/CD4+-CTCF-ChIP-Seq(Barski_et_al.)	TGCAGTTCCMVNWRTGGCCA	1.00E+00	-1.97E+00	0.2713	3	3.03%	424.5	1.30%
ETS:RUNX(ETS,Runt)/Jurkat-RUNX1-ChIP-Seq(GSE17954)	RCAGGATGTGGT	1.00E+00	-1.97E+00	0.2713	10	10.10%	2229.5	6.84%
Stat3(Stat)/mES-Stat3-ChIP-Seq(GSE11431)	CTTCCGGGAA	1.00E+00	-1.97E+00	0.2713	42	42.42%	11960.4	36.68%
Stat3+il21(Stat)/CD4-Stat3-ChIP-Seq(GSE19198)	SVYTTCCNGGAARB	1.00E+00	-1.96E+00	0.2713	55	55.56%	16182.9	49.63%
Hand2(bHLH)/Mesoderm-Hand2-ChIP-Seq(GSE61475)	TGACANARRCCAGRC	1.00E+00	-1.95E+00	0.273	40	40.40%	11340.8	34.78%
ZNF143|STAF(Zf)/CUTLL-ZNF143-ChIP-Seq(GSE29600)	ATTTCCCAGVAKSCY	1.00E+00	-1.94E+00	0.2737	32	32.32%	8823.4	27.06%
En1(Homeobox)/SUM149-EN1-ChIP-Seq(GSE120957)	NDCTAATTAS	1.00E+00	-1.93E+00	0.2748	89	89.90%	27939.7	85.69%
Brachyury(T-box)/Mesoendoderm-Brachyury-ChIP-exo(GSE54963)	ANTTMRCASBNNNGTGYKAAN	1.00E+00	-1.91E+00	0.2787	29	29.29%	7914.7	24.27%
Tbox:Smad(T-box,MAD)/ESCd5-Smad2_3-ChIP-Seq(GSE29422)	AGGTGHCAGACA	1.00E+00	-1.90E+00	0.2817	20	20.20%	5179.7	15.89%
SpiB(ETS)/OCILY3-SPIB-ChIP-Seq(GSE56857)	AAAGRGGAAGTG	1.00E+00	-1.88E+00	0.2871	27	27.27%	7329.5	22.48%
Pit1(Homeobox)/GCrat-Pit1-ChIP-Seq(GSE58009)	ATGMATATDC	1.00E+00	-1.85E+00	0.2936	73	73.74%	22349.4	68.54%
Elk1(ETS)/Hela-Elk1-ChIP-Seq(GSE31477)	HACTTCCGGY	1.00E+00	-1.84E+00	0.2953	39	39.39%	11129.7	34.13%
Atoh1(bHLH)/Cerebellum-Atoh1-ChIP-Seq(GSE22111)	VNRVCAGCTGGY	1.00E+00	-1.83E+00	0.2958	62	62.63%	18642.7	57.18%
Hoxd12(Homeobox)/ChickenMSG-Hoxd12.Flag-ChIP-Seq(GSE86088)	HDGYAATGAAAN	1.00E+00	-1.81E+00	0.3008	89	89.90%	28031.6	85.97%
p53(p53)/Saos-p53-ChIP-Seq(GSE15780)	RRCATGYCYRGRCATGYYYN	1.00E+00	-1.81E+00	0.3008	12	12.12%	2884.6	8.85%
p53(p53)/Saos-p53-ChIP-Seq	RRCATGYCYRGRCATGYYYN	1.00E+00	-1.81E+00	0.3008	12	12.12%	2884.6	8.85%
Meis1(Homeobox)/MastCells-Meis1-ChIP-Seq(GSE48085)	VGCTGWCAVB	1.00E+00	-1.74E+00	0.3181	80	80.81%	24877.3	76.30%
NRF(NRF)/Promoter	STGCGCATGCGC	1.00E+00	-1.73E+00	0.3203	12	12.12%	2928.7	8.98%
CLOCK(bHLH)/Liver-Clock-ChIP-Seq(GSE39860)	GHCACGTG	1.00E+00	-1.73E+00	0.3204	37	37.37%	10605.7	32.53%
Eomes(T-box)/H9-Eomes-ChIP-Seq(GSE26097)	ATTAACACCT	1.00E+00	-1.71E+00	0.3255	89	89.90%	28113.2	86.22%
EHF(ETS)/LoVo-EHF-ChIP-Seq(GSE49402)	AVCAGGAAGT	1.00E+00	-1.69E+00	0.3306	75	75.76%	23200.8	71.16%
Usf2(bHLH)/C2C12-Usf2-ChIP-Seq(GSE36030)	GTCACGTGGT	1.00E+00	-1.68E+00	0.3317	27	27.27%	7504.8	23.02%
Pax7(Paired,Homeobox),long/Myoblast-Pax7-ChIP-Seq(GSE25064)	TAATCHGATTAC	1.00E+00	-1.66E+00	0.3368	5	5.05%	1004	3.08%
AP-2gamma(AP2)/MCF7-TFAP2C-ChIP-Seq(GSE21234)	SCCTSAGGSCAW	1.00E+00	-1.66E+00	0.3369	46	46.46%	13573.9	41.63%

CRE(bZIP)/Promoter	CSGTGACGTCAC	1.00E+00	-1.66E+00	0.3369	17	17.17%	4462	13.68%
GATA(Zf),IR3/iTreg-Gata3-ChIP-Seq(GSE20898)	NNNNNBAGATAWYATCTVHN	1.00E+00	-1.64E+00	0.3415	18	18.18%	4778.9	14.66%
Bcl6(Zf)/Liver-Bcl6-ChIP-Seq(GSE31578)	NNNCTTTCCAGGAAA	1.00E+00	-1.63E+00	0.3428	77	77.78%	23951.1	73.46%
ZNF264(Zf)/HEK293-ZNF264.GFP-ChIP-Seq(GSE58341)	RGGGCACTAACY	1.00E+00	-1.63E+00	0.3428	46	46.46%	13606.6	41.73%
CTCF(Zf)/CD4+-CTCF-ChIP-Seq(Barski_et_al.)	AYAGTGCCMYCTRGTGGCCA	1.00E+00	-1.62E+00	0.3428	12	12.12%	2997.9	9.19%
Pknox1(Homeobox)/ES-Prep1-ChIP-Seq(GSE63282)	SCTGTCAVTCAV	1.00E+00	-1.62E+00	0.3428	23	23.23%	6321.1	19.39%
ZFX(Zf)/mES-Zfx-ChIP-Seq(GSE11431)	AGGCCTRG	1.00E+00	-1.61E+00	0.3429	61	61.62%	18554	56.90%
IRF2(IRF)/Erythroblas-IRF2-ChIP-Seq(GSE36985)	GAAASYGAAASY	1.00E+00	-1.61E+00	0.3429	14	14.14%	3596.8	11.03%
PRDM1(Zf)/Hela-PRDM1-ChIP-Seq(GSE31477)	ACTTTCACTTTC	1.00E+00	-1.59E+00	0.3476	49	49.49%	14625.3	44.86%
Olig2(bHLH)/Neuron-Olig2-ChIP-Seq(GSE30882)	RCCATMTGTT	1.00E+00	-1.59E+00	0.3476	85	85.86%	26781.3	82.14%
Pax8(Paired,Homeobox)/Thyroid-Pax8-ChIP-Seq(GSE26938)	GTCATGCHTGRCTGS	1.00E+00	-1.58E+00	0.3484	24	24.24%	6666.7	20.45%
GATA(Zf),IR4/iTreg-Gata3-ChIP-Seq(GSE20898)	NAGATWNBNATCTNN	1.00E+00	-1.55E+00	0.3568	11	11.11%	2748.8	8.43%
Prop1(Homeobox)/GHFT1-PROP1.biotin-ChIP-Seq(GSE77302)	NTAATBNAATTA	1.00E+00	-1.55E+00	0.3568	57	57.58%	17298.1	53.05%
PGR(NR)/EndoStromal-PGR-ChIP-Seq(GSE69539)	AAGAACATWHTGTTC	1.00E+00	-1.55E+00	0.3568	29	29.29%	8262.6	25.34%
Sox21(HMG)/ESC-SOX21-ChIP-Seq(GSE110505)	BCCWTTGTBYKV	1.00E+00	-1.55E+00	0.3568	89	89.90%	28246.4	86.63%
Egr1(Zf)/K562-Egr1-ChIP-Seq(GSE32465)	TGCGTGGGYG	1.00E+00	-1.54E+00	0.3568	37	37.37%	10809.4	33.15%
Gata6(Zf)/HUG1N-GATA6-ChIP-Seq(GSE51936)	YCTTATCTBN	1.00E+00	-1.54E+00	0.3568	64	64.65%	19643.2	60.24%
THRb(NR)/HepG2-THRb.Flag-ChIP-Seq(Encode)	GGTCACCTGAGGTCA	1.00E+00	-1.53E+00	0.3568	35	35.35%	10178.2	31.22%
Sox10(HMG)/SciaticNerve-Sox3-ChIP-Seq(GSE35132)	CCWTTGTYYB	1.00E+00	-1.53E+00	0.3568	87	87.88%	27544.9	84.48%
EBF1(EBF)/Near-E2A-ChIP-Seq(GSE21512)	GTCCCCWGGGGA	1.00E+00	-1.53E+00	0.3568	52	52.53%	15679.7	48.09%
p53(p53)/mES-cMyc-ChIP-Seq(GSE11431)	ACATGCCCGGGCAT	1.00E+00	-1.53E+00	0.3568	2	2.02%	287.4	0.88%
RXR(NR),DR1/3T3L1-RXR-ChIP-Seq(GSE13511)	TAGGGCAAAGGTCA	1.00E+00	-1.51E+00	0.359	62	62.63%	19009.1	58.30%
ETV4(ETS)/HepG2-ETV4-ChIP-Seq(ENCODE)	ACCGGAAGTG	1.00E+00	-1.50E+00	0.3598	62	62.63%	19016.1	58.32%
ETS(ETS)/Promoter	AACCGGAAGT	1.00E+00	-1.49E+00	0.3635	27	27.27%	7692.4	23.59%
Tcf12(bHLH)/GM12878-Tcf12-ChIP-Seq(GSE32465)	VCAGCTGYTG	1.00E+00	-1.45E+00	0.3772	52	52.53%	15779	48.39%
Nkx2.1(Homeobox)/LungAC-Nkx2.1-ChIP-Seq(GSE43252)	RSCACTYRAG	1.00E+00	-1.45E+00	0.3772	94	94.95%	30148.4	92.46%
STAT6(Stat)/CD4-Stat6-ChIP-Seq(GSE22104)	ABTTCYYRRGAA	1.00E+00	-1.44E+00	0.3797	49	49.49%	14813.7	45.43%
Fli1(ETS)/CD8-FLI-ChIP-Seq(GSE20898)	NRYTTCCGGH	1.00E+00	-1.43E+00	0.3797	65	65.66%	20102.1	61.65%
Phox2a(Homeobox)/Neuron-Phox2a-ChIP-Seq(GSE31456)	YTAATYNRATTA	1.00E+00	-1.43E+00	0.3797	42	42.42%	12544.8	38.47%
Lhx3(Homeobox)/Neuron-Lhx3-ChIP-Seq(GSE31456)	ADBTAATTAR	1.00E+00	-1.42E+00	0.3797	86	86.87%	27288.6	83.69%
Tcf3(HMG)/mES-Tcf3-ChIP-Seq(GSE11724)	ASWTCAAAGG	1.00E+00	-1.42E+00	0.3797	28	28.28%	8078.6	24.78%
CRX(Homeobox)/Retina-Crx-ChIP-Seq(GSE20012)	GCTAATCC	1.00E+00	-1.42E+00	0.3797	92	92.93%	29432.8	90.27%
MafK(bZIP)/C2C12-MafK-ChIP-Seq(GSE36030)	GCTGASTCAGCA	1.00E+00	-1.41E+00	0.3819	22	22.22%	6210.7	19.05%
Ets1-distal(ETS)/CD4+-PolII-ChIP-Seq(Barski_et_al.)	MACAGGAAGT	1.00E+00	-1.41E+00	0.3819	31	31.31%	9045.7	27.74%
Dlx3(Homeobox)/Kerainocytes-Dlx3-ChIP-Seq(GSE89884)	NDGTAATTAC	1.00E+00	-1.40E+00	0.3826	58	58.59%	17818.7	54.65%
Fox:Ebox(Forkhead,bHLH)/Panc1-Foxa2-ChIP-Seq(GSE47459)	NNNVCTGWGYAAACASN	1.00E+00	-1.38E+00	0.3887	72	72.73%	22523.1	69.08%
EWS:ERG-fusion(ETS)/CADO_ES1-EWS:ERG-ChIP-Seq(SRA014231)	ATTTCCTGTN	1.00E+00	-1.38E+00	0.3887	57	57.58%	17516	53.72%
MafF(bZIP)/HepG2-MafF-ChIP-Seq(GSE31477)	HWWGTCAGCAWWTTT	1.00E+00	-1.36E+00	0.394	29	29.29%	8464.9	25.96%
HNF1b(Homeobox)/PDAC-HNF1B-ChIP-Seq(GSE64557)	GTTAATNATTAA	1.00E+00	-1.35E+00	0.3949	21	21.21%	5952.6	18.26%
CHR(?)/Hela-CellCycle-Expression	SRGTTTCAAA	1.00E+00	-1.35E+00	0.3949	56	56.57%	17218.8	52.81%
TFE3(bHLH)/MEF-TFE3-ChIP-Seq(GSE75757)	GTCACGTGACYV	1.00E+00	-1.35E+00	0.3949	9	9.09%	2291.1	7.03%
Reverb(NR),DR2/RAW-Reverba.biotin-ChIP-Seq(GSE45914)	GTRGGTCASTGGGTCA	1.00E+00	-1.32E+00	0.4021	12	12.12%	3206.2	9.83%
IRF1(IRF)/PBMC-IRF1-ChIP-Seq(GSE43036)	GAAAGTGAAAGT	1.00E+00	-1.32E+00	0.4044	18	18.18%	5055.7	15.51%
Pbx3(Homeobox)/GM12878-PBX3-ChIP-Seq(GSE32465)	SCTGTCAMTCAN	1.00E+00	-1.31E+00	0.4044	23	23.23%	6619	20.30%
ZNF768(Zf)/Rajj-ZNF768-ChIP-Seq(GSE111879)	RHHCAGAGAGGB	1.00E+00	-1.28E+00	0.4182	4	4.04%	881.5	2.70%
Fra1(bZIP)/BT549-Fra1-ChIP-Seq(GSE46166)	NNATGASTCATH	1.00E+00	-1.27E+00	0.4182	45	45.45%	13717.1	42.07%
Lhx1(Homeobox)/EmbryoCarcinoma-Lhx1-ChIP-Seq(GSE70957)	NNYTAATTAR	1.00E+00	-1.27E+00	0.4182	74	74.75%	23333.1	71.56%
E2F1(E2F)/Hela-E2F1-ChIP-Seq(GSE22478)	CWGGCGGGAA	1.00E+00	-1.25E+00	0.4255	9	9.09%	2357.3	7.23%
KLF6(Zf)/PDAC-KLF6-ChIP-Seq(GSE64557)	MKGGGYGTGGCC	1.00E+00	-1.24E+00	0.4275	44	44.44%	13438.2	41.21%
MYNN(Zf)/HEK293-MYNN.eGFP-ChIP-Seq(Encode)	TTCAAAWTAAAAGTC	1.00E+00	-1.23E+00	0.4301	32	32.32%	9574.4	29.36%
ZKSCAN1(Zf)/HepG2-ZKSCAN1-ChIP-Seq(Encode)	GCACAYAGTAGGKCY	1.00E+00	-1.23E+00	0.4301	4	4.04%	901.7	2.77%

REST-NRSF(Zf)/Jurkat-NRSF-ChIP-Seq	GGMGCTGTCCATGGTGCTGA	1.00E+00	-1.22E+00	0.4315	1	1.01%	115.9	0.36%
PAX5(Paired,Homeobox)/GM12878-PAX5-ChIP-Seq(GSE32465)	GCAGCCAAGCRTGACH	1.00E+00	-1.22E+00	0.4315	25	25.25%	7354.4	22.56%
Rbpj1(?)/Panc1-Rbpj1-ChIP-Seq(GSE47459)	HTTTCCCASG	1.00E+00	-1.20E+00	0.4393	75	75.76%	23765	72.89%
Rfx5(HTH)/GM12878-Rfx5-ChIP-Seq(GSE31477)	SCCTAGCAACAG	1.00E+00	-1.19E+00	0.4418	30	30.30%	8988.3	27.57%
Mouse_Recombination_Hotspot(Zf)/Testis-DMC1-ChIP-Seq(GSE24438)	ACTYKNATTCGTGNTACTTC	1.00E+00	-1.17E+00	0.4477	9	9.09%	2412.5	7.40%
CEBP:CEBP(bZIP)/MEF-Chop-ChIP-Seq(GSE35681)	NTNATGCAAYMNNHTGMAAY	1.00E+00	-1.16E+00	0.4511	17	17.17%	4896.3	15.02%
GLI3(Zf)/Limb-GLI3-ChIP-Chip(GSE11077)	CGTGGGTGGTCC	1.00E+00	-1.14E+00	0.46	9	9.09%	2437.7	7.48%
Etv2(ETS)/ES-ER71-ChIP-Seq(GSE59402)	NNAYTTCCTGHN	1.00E+00	-1.12E+00	0.4645	61	61.62%	19186.3	58.84%
bZIP:IRF(bZIP,IRF)/Th17-BatF-ChIP-Seq(GSE39756)	NAGTTTCABTHTGACTNW	1.00E+00	-1.11E+00	0.4672	45	45.45%	13940.8	42.76%
Barx1(Homeobox)/Stomach-Barx1.3xFlag-ChIP-Seq(GSE69483)	AAACMATTAN	1.00E+00	-1.11E+00	0.4672	48	48.48%	14923.4	45.77%
E2F3(E2F)/MEF-E2F3-ChIP-Seq(GSE71376)	BTKGGCGGGAAA	1.00E+00	-1.11E+00	0.4675	26	26.26%	7808.6	23.95%
DLX2(Homeobox)/BasalGanglia-Dlx2-ChIP-seq(GSE124936)	NNNTAATTAS	1.00E+00	-1.11E+00	0.4675	82	82.83%	26247.2	80.50%
PR(NR)/T47D-PR-ChIP-Seq(GSE31130)	VAGRACAKNCTGTBC	1.00E+00	-1.08E+00	0.4771	90	90.91%	29016.5	88.99%
Hoxb4(Homeobox)/ES-Hoxb4-ChIP-Seq(GSE34014)	TGATTRATGGCY	1.00E+00	-1.07E+00	0.483	20	20.20%	5946.4	18.24%
ELF1(ETS)/Jurkat-ELF1-ChIP-Seq(SRA014231)	AVCCGGAAGT	1.00E+00	-1.06E+00	0.483	34	34.34%	10441.4	32.02%
Mef2c(MADS)/GM12878-Mef2c-ChIP-Seq(GSE32465)	DCYAAAAATAGM	1.00E+00	-1.05E+00	0.4853	46	46.46%	14357.9	44.04%
Fra2(bZIP)/Striatum-Fra2-ChIP-Seq(GSE43429)	GGATGACTCATC	1.00E+00	-1.05E+00	0.4875	38	38.38%	11762.4	36.07%
Hnf1(Homeobox)/Liver-Foxa2-Chip-Seq(GSE25694)	GGTTAAWCATTAA	1.00E+00	-1.02E+00	0.4977	19	19.19%	5679	17.42%
Rfx6(HTH)/Min6b1-Rfx6.HA-ChIP-Seq(GSE62844)	TGTTKCCTAGCAACM	1.00E+00	-1.02E+00	0.498	62	62.63%	19674.2	60.34%
MafA(bZIP)/Islet-MafA-ChIP-Seq(GSE30298)	TGCTGACTCA	1.00E+00	-9.98E-01	0.5071	53	53.54%	16740.7	51.34%
ARE(NR)/LNCAP-AR-ChIP-Seq(GSE27824)	RGRACASNSTGTYCYB	1.00E+00	-9.96E-01	0.5071	27	27.27%	8276	25.38%
TR4(NR),DR1/Hela-TR4-ChIP-Seq(GSE24685)	GAGGTCAAAGGTCA	1.00E+00	-9.93E-01	0.5071	11	11.11%	3178	9.75%
Sox3(HMG)/NPC-Sox3-ChIP-Seq(GSE33059)	CCWTTGTY	1.00E+00	-9.82E-01	0.5105	87	87.88%	28091.7	86.16%
RAR:RXR(NR),DR5/ES-RAR-ChIP-Seq(GSE56893)	RGGTCADNNAGAGGTCAV	1.00E+00	-9.77E-01	0.5114	3	3.03%	723.2	2.22%
ZNF317(Zf)/HEK293-ZNF317.GFP-ChIP-Seq(GSE58341)	GTCWGCTGTYYCTCT	1.00E+00	-9.67E-01	0.5146	9	9.09%	2573.3	7.89%
AP-1(bZIP)/ThioMac-PU.1-ChIP-Seq(GSE21512)	VTGACTCATC	1.00E+00	-9.60E-01	0.517	52	52.53%	16473.2	50.52%
Fosl2(bZIP)/3T3L1-Fosl2-ChIP-Seq(GSE56872)	NATGASTCABNN	1.00E+00	-9.58E-01	0.517	28	28.28%	8653.7	26.54%
ZNF669(Zf)/HEK293-ZNF669.GFP-ChIP-Seq(GSE58341)	GARTGGTCATCGCCC	1.00E+00	-9.41E-01	0.5236	5	5.05%	1349.7	4.14%
SF1(NR)/H295R-Nr5a1-ChIP-Seq(GSE44220)	CAAGGHCANV	1.00E+00	-9.31E-01	0.5271	37	37.37%	11614.3	35.62%
Nr5a2(NR)/mES-Nr5a2-ChIP-Seq(GSE19019)	BTCAAGGTCA	1.00E+00	-9.30E-01	0.5271	40	40.40%	12593.5	38.62%
Hoxa9(Homeobox)/ChickenMSG-Hoxa9.Flag-ChIP-Seq(GSE86088)	RGCAATNAAA	1.00E+00	-9.30E-01	0.5271	94	94.95%	30535.1	93.65%
Fos(bZIP)/TSC-Fos-ChIP-Seq(GSE110950)	NDATGASTCAYN	1.00E+00	-9.19E-01	0.5288	45	45.45%	14246.3	43.69%
DLX1(Homeobox)/BasalGanglia-Dlx1-ChIP-seq(GSE124936)	NSNNTAATTA	1.00E+00	-9.12E-01	0.531	78	78.79%	25154.7	77.15%
ZNF322(Zf)/HEK293-ZNF322.GFP-ChIP-Seq(GSE58341)	GAGCCTGGTACTGWGCCTGR	1.00E+00	-9.03E-01	0.5341	19	19.19%	5826.3	17.87%
TATA-Box(TBP)/Promoter	CCTTTTAWAGSC	1.00E+00	-8.92E-01	0.5383	77	77.78%	24849.4	76.21%
Hoxa13(Homeobox)/ChickenMSG-Hoxa13.Flag-ChIP-Seq(GSE86088)	CYHATAAAAN	1.00E+00	-8.86E-01	0.5401	94	94.95%	30572.7	93.77%
HIF-1b(HLH)/T47D-HIF1b-ChIP-Seq(GSE59937)	RTACGTGC	1.00E+00	-8.78E-01	0.5427	55	55.56%	17597.8	53.97%
GABPA(ETS)/Jurkat-GABPa-ChIP-Seq(GSE17954)	RACCGGAAGT	1.00E+00	-8.73E-01	0.5436	55	55.56%	17605.4	54.00%
Sp2(Zf)/HEK293-Sp2.eGFP-ChIP-Seq(Encode)	YGGCCCCGCCCC	1.00E+00	-8.63E-01	0.5476	52	52.53%	16637.5	51.03%
ZNF41(Zf)/HEK293-ZNF41.GFP-ChIP-Seq(GSE58341)	CCTCATGGTGYCYTWYTCCCTTGTG	1.00E+00	-8.62E-01	0.5476	3	3.03%	779.3	2.39%
IRF:BATF(IRF:bZIP)/pDC-Irf8-ChIP-Seq(GSE66899)	CTTTCANTATGACTV	1.00E+00	-8.59E-01	0.5476	16	16.16%	4916.3	15.08%
PU.1(ETS)/ThioMac-PU.1-ChIP-Seq(GSE21512)	AGAGGAAGTG	1.00E+00	-8.39E-01	0.5562	39	39.39%	12421.8	38.10%
Maz(Zf)/HepG2-Maz-ChIP-Seq(GSE31477)	GGGGGGGG	1.00E+00	-8.21E-01	0.5645	50	50.51%	16056.5	49.24%
IRF4(IRF)/GM12878-IRF4-ChIP-Seq(GSE32465)	ACTGAAACCA	1.00E+00	-8.05E-01	0.5721	38	38.38%	12154.3	37.28%
NFE2L2(bZIP)/HepG2-NFE2L2-ChIP-Seq(Encode)	AWWWTGCTGAGTCAT	1.00E+00	-7.75E-01	0.5878	7	7.07%	2110.5	6.47%
ZNF692(Zf)/HEK293-ZNF692.GFP-ChIP-Seq(GSE58341)	GTGGGCCCCA	1.00E+00	-7.69E-01	0.5892	7	7.07%	2115.3	6.49%
Sp5(Zf)/mES-Sp5.Flag-ChIP-Seq(GSE72989)	RGKGGGCGGAGC	1.00E+00	-7.62E-01	0.5919	40	40.40%	12887.2	39.52%
RORa(NR)/Liver-Rora-ChIP-Seq(GSE101115)	AAWCTAGGTCARDNN	1.00E+00	-7.52E-01	0.5961	16	16.16%	5058.7	15.51%
Arnt:Ahr(bHLH)/MCF7-Arnt-ChIP-Seq(Lo_et_al.)	TBGCACGCAA	1.00E+00	-7.28E-01	0.6085	37	37.37%	11967.2	36.70%
Six4(Homeobox)/MCF7-SIX4-ChIP-Seq(Encode)	TGWAAYCTGABACCB	1.00E+00	-7.25E-01	0.6087	5	5.05%	1506.9	4.62%
Bach2(bZIP)/OCILy7-Bach2-ChIP-Seq(GSE44420)	TGCTGAGTCA	1.00E+00	-7.14E-01	0.6141	17	17.17%	5440.5	16.69%

Ptf1a(bHLH)/Panc1-Ptf1a-ChIP-Seq(GSE47459)	ACAGCTGTTN	1.00E+00	-7.12E-01	0.6141	84	84.85%	27434.5	84.14%
Bapx1(Homeobox)/VertebralCol-Bapx1-ChIP-Seq(GSE36672)	TTRAGTGSYK	1.00E+00	-7.06E-01	0.6154	86	86.87%	28101.8	86.19%
Smad3(MAD)/NPC-Smad3-ChIP-Seq(GSE36673)	TWGTCTGV	1.00E+00	-6.88E-01	0.6244	91	91.92%	29765.2	91.29%
PRDM14(Zf)/H1-PRDM14-ChIP-Seq(GSE22767)	RGGTCTCTAACY	1.00E+00	-6.78E-01	0.6291	26	26.26%	8452.5	25.92%
RAR:RXR(NR),DR0/ES-RAR-ChIP-Seq(GSE56893)	AGGTCAAGGTCA	1.00E+00	-6.77E-01	0.6291	14	14.14%	4509.3	13.83%
EKLF(Zf)/Erythrocyte-Klf1-ChIP-Seq(GSE20478)	NWGGGTGTGGCY	1.00E+00	-6.50E-01	0.6436	13	13.13%	4219.6	12.94%
ZBTB12(Zf)/HEK293-ZBTB12.GFP-ChIP-Seq(GSE58341)	NGNTCTAGAACCNGV	1.00E+00	-6.46E-01	0.6443	33	33.33%	10816.7	33.17%
TCFL2(HMG)/K562-TCF7L2-ChIP-Seq(GSE29196)	ACWTCAAAGG	1.00E+00	-6.33E-01	0.6505	9	9.09%	2917.1	8.95%
Tbx5(T-box)/HL1-Tbx5.biotin-ChIP-Seq(GSE21529)	AGGTGTCA	1.00E+00	-6.33E-01	0.6505	91	91.92%	29831.2	91.49%
ETV1(ETS)/GIST48-ETV1-ChIP-Seq(GSE22441)	AACCGGAAGT	1.00E+00	-6.30E-01	0.6505	69	69.70%	22662.4	69.50%
Zfp809(Zf)/ES-Zfp809-ChIP-Seq(GSE70799)	GGGGCTYGKCTGGGA	1.00E+00	-6.28E-01	0.6505	10	10.10%	3256.4	9.99%
THRa(NR)/C17.2-THRa-ChIP-Seq(GSE38347)	GGTCANYTGAGGWCA	1.00E+00	-6.11E-01	0.6582	26	26.26%	8580.2	26.32%
E2F7(E2F)/Hela-E2F7-ChIP-Seq(GSE32673)	VDTTTCCCGCCA	1.00E+00	-5.83E-01	0.6747	4	4.04%	1297.7	3.98%
KLF1(Zf)/HUDEP2-KLF1-CutnRun(GSE136251)	VDGGGYGGGGCY	1.00E+00	-5.81E-01	0.6747	35	35.35%	11613.5	35.62%
GATA3(Zf)/iTreg-Gata3-ChIP-Seq(GSE20898)	AGATAASR	1.00E+00	-5.65E-01	0.6836	77	77.78%	25400.1	77.90%
Hoxa10(Homeobox)/ChickenMSG-Hoxa10.Flag-ChIP-Seq(GSE86088)	GGYAATGAAA	1.00E+00	-5.63E-01	0.6836	47	47.47%	15604.1	47.86%
Rfx1(HTH)/NPC-H3K4me1-ChIP-Seq(GSE16256)	KGTTGCCATGGCAA	1.00E+00	-5.54E-01	0.6874	15	15.15%	5036.6	15.45%
KLF5(Zf)/LoVo-KLF5-ChIP-Seq(GSE49402)	DGGGYGKGGC	1.00E+00	-5.38E-01	0.6965	46	46.46%	15336.8	47.04%
GRE(NR),IR3/A549-GR-ChIP-Seq(GSE32465)	NRGVACABNVTGTYCY	1.00E+00	-5.37E-01	0.6965	13	13.13%	4395	13.48%
Mef2b(MADS)/HEK293-Mef2b.V5-ChIP-Seq(GSE67450)	GCTATTTTTGGM	1.00E+00	-5.24E-01	0.7024	60	60.61%	19957.5	61.21%
HIF2a(bHLH)/785_O-HIF2a-ChIP-Seq(GSE34871)	GCACGTACCC	1.00E+00	-5.15E-01	0.7067	21	21.21%	7115.3	21.82%
Pax7(Paired,Homeobox),longest/Myoblast-Pax7-ChIP-Seq(GSE25064)	NTAATTDGCYAATTANNWWD	1.00E+00	-5.09E-01	0.7092	4	4.04%	1369.8	4.20%
LHX9(Homeobox)/Hct116-LHX9.V5-ChIP-Seq(GSE116822)	NGCTAATTAG	1.00E+00	-5.02E-01	0.7119	77	77.78%	25526.1	78.29%
AR-halfsite(NR)/LNCaP-AR-ChIP-Seq(GSE27824)	CCAGGAACAG	1.00E+00	-4.90E-01	0.719	96	96.97%	31567.4	96.82%
Isl1(Homeobox)/Neuron-Isl1-ChIP-Seq(GSE31456)	CTAATKGV	1.00E+00	-4.88E-01	0.719	85	85.86%	28120.9	86.25%
E2F4(E2F)/K562-E2F4-ChIP-Seq(GSE31477)	GGCGGGAAAH	1.00E+00	-4.78E-01	0.7235	14	14.14%	4837.7	14.84%
RORg(NR)/Liver-Rorc-ChIP-Seq(GSE101115)	WAABTAGGTCAV	1.00E+00	-4.60E-01	0.735	10	10.10%	3508.2	10.76%
Hoxd10(Homeobox)/ChickenMSG-Hoxd10.Flag-ChIP-Seq(GSE86088)	GGCMATGAAA	1.00E+00	-4.55E-01	0.7364	69	69.70%	23052.3	70.70%
Hoxa11(Homeobox)/ChickenMSG-Hoxa11.Flag-ChIP-Seq(GSE86088)	TTTTATGGCM	1.00E+00	-4.52E-01	0.7371	91	91.92%	30070	92.22%
GFY(?)/Promoter	ACTACAATTCCC	1.00E+00	-4.41E-01	0.7427	5	5.05%	1799	5.52%
Hoxd13(Homeobox)/ChickenMSG-Hoxd13.Flag-ChIP-Seq(GSE86088)	NCYAATAAAA	1.00E+00	-4.21E-01	0.756	82	82.83%	27297.3	83.72%
Klf4(Zf)/mES-Klf4-ChIP-Seq(GSE11431)	GCCACACCCA	1.00E+00	-3.92E-01	0.776	18	18.18%	6382.4	19.57%
LXRE(NR),DR4/RAW-LXRb.biotin-ChIP-Seq(GSE21512)	RGGTTACTANAGGTCA	1.00E+00	-3.92E-01	0.776	4	4.04%	1506.9	4.62%
FoxD3(forkhead)/ZebrafishEmbryo-Foxd3.biotin-ChIP-seq(GSE106676)	TGTTTAYTTAGC	1.00E+00	-3.75E-01	0.7854	64	64.65%	21655.5	66.42%
HIF-1a(bHLH)/MCF7-HIF1a-ChIP-Seq(GSE28352)	TACGTGCV	1.00E+00	-3.65E-01	0.7914	14	14.14%	5078.9	15.58%
BMYB(HTH)/Hela-BMYB-ChIP-Seq(GSE27030)	NHAACBGYYV	1.00E+00	-3.56E-01	0.7967	75	75.76%	25236.9	77.40%
ETS1(ETS)/Jurkat-ETS1-ChIP-Seq(GSE17954)	ACAGGAAGTG	1.00E+00	-3.54E-01	0.7967	58	58.59%	19778.1	60.66%
c-Myc(bHLH)/LNCAP-cMyc-ChIP-Seq(Unpublished)	VCCACGTG	1.00E+00	-3.32E-01	0.8113	20	20.20%	7225.4	22.16%
Egr2(Zf)/Thymocytes-Egr2-ChIP-Seq(GSE34254)	NGCGTGGGCGGR	1.00E+00	-3.18E-01	0.8206	7	7.07%	2717.7	8.34%
Foxo1(Forkhead)/RAW-Foxo1-ChIP-Seq(Fan_et_al.)	CTGTTTAC	1.00E+00	-3.16E-01	0.8206	84	84.85%	28159.4	86.36%
Mef2d(MADS)/Retina-Mef2d-ChIP-Seq(GSE61391)	GCTATTTTTAGC	1.00E+00	-3.00E-01	0.8314	19	19.19%	6979.8	21.41%
Hoxd11(Homeobox)/ChickenMSG-Hoxd11.Flag-ChIP-Seq(GSE86088)	VGCCATAAAA	1.00E+00	-2.83E-01	0.8437	90	90.91%	30053.1	92.17%
T1ISRE(IRF)/ThioMac-Ifnb-Expression	ACTTTCGTTTCT	1.00E+00	-2.64E-01	0.8579	1	1.01%	478.2	1.47%
X-box(HTH)/NPC-H3K4me1-ChIP-Seq(GSE16256)	GGTTGCCATGGCAA	1.00E+00	-2.40E-01	0.8759	8	8.08%	3266.2	10.02%
NPAS2(bHLH)/Liver-NPAS2-ChIP-Seq(GSE39860)	KCCACGTGAC	1.00E+00	-2.28E-01	0.8849	52	52.53%	18307.5	56.15%
Nkx6.1(Homeobox)/Islet-Nkx6.1-ChIP-Seq(GSE40975)	GKTAATGR	1.00E+00	-2.26E-01	0.8849	91	91.92%	30464.1	93.43%
ZBTB18(Zf)/HEK293-ZBTB18.GFP-ChIP-Seq(GSE58341)	AACATCTGGA	1.00E+00	-2.25E-01	0.8849	32	32.32%	11695.8	35.87%
Klf9(Zf)/GBM-Klf9-ChIP-Seq(GSE62211)	GCCACRCCCACY	1.00E+00	-2.05E-01	0.8982	16	16.16%	6266.8	19.22%
Ronin(THAP)/ES-Thap11-ChIP-Seq(GSE51522)	RACTACAACTCCCAGVAKGC	1.00E+00	-1.99E-01	0.9015	1	1.01%	559.7	1.72%
bHLHE40(bHLH)/HepG2-BHLHE40-ChIP-Seq(GSE31477)	KCACGTGMCN	1.00E+00	-1.97E-01	0.9015	15	15.15%	5945.1	18.23%
STAT4(Stat)/CD4-Stat4-ChIP-Seq(GSE22104)	NYTTCCWGGAAR	1.00E+00	-1.95E-01	0.9015	59	59.60%	20724.6	63.56%

MYB(HTH)/ERMYB-Myb-ChIPSeq(GSE22095)	GGCVGTTR	1.00E+00	-1.86E-01	0.9067	77	77.78%	26419.5	81.03%
Mef2a(MADS)/HL1-Mef2a.biotin-ChIP-Seq(GSE21529)	CYAAAAATAG	1.00E+00	-1.82E-01	0.9082	35	35.35%	12917.4	39.62%
RFX(HTH)/K562-RFX3-ChIP-Seq(SRA012198)	CGGTTGCCATGGCAAC	1.00E+00	-1.77E-01	0.9107	5	5.05%	2310.3	7.09%
AMYB(HTH)/Testes-AMYB-ChIP-Seq(GSE44588)	TGGCAGTTGG	1.00E+00	-1.69E-01	0.9153	72	72.73%	24955.1	76.54%
Zfp57(Zf)/H1-ZFP57.HA-ChIP-Seq(GSE115387)	NANTGCSGCA	1.00E+00	-1.53E-01	0.9275	9	9.09%	3934.6	12.07%
NRF1(NRF)/MCF7-NRF1-ChIP-Seq(Unpublished)	CTGCGCATGCGC	1.00E+00	-1.49E-01	0.9296	3	3.03%	1574.2	4.83%
TRPS1(Zf)/MCF7-TRPS1-ChIP-Seq(GSE107013)	AGATAAGANN	1.00E+00	-1.45E-01	0.9302	83	83.84%	28388.6	87.07%
GFY-Staf(?,Zf)/Promoter	RACTACAATTCCCAGAAKGC	1.00E+00	-1.37E-01	0.9362	3	3.03%	1611.7	4.94%
NF1:FOXA1(CTF,Forkhead)/LNCAP-FOXA1-ChIP-Seq(GSE27824)	WNTGTTTRYTTTGGCA	1.00E+00	-1.34E-01	0.9362	4	4.04%	2044.3	6.27%
Erra(NR)/HepG2-Erra-ChIP-Seq(GSE31477)	CAAAGGTCAG	1.00E+00	-1.22E-01	0.9449	76	76.77%	26419.7	81.03%
Tgif1(Homeobox)/mES-Tgif1-ChIP-Seq(GSE55404)	YTGWCADY	1.00E+00	-1.09E-01	0.9556	91	91.92%	30780.5	94.40%
ZNF165(Zf)/WHIM12-ZNF165-ChIP-Seq(GSE65937)	AAGGKGRCGCAGGCA	1.00E+00	-9.90E-02	0.9626	4	4.04%	2189.3	6.71%
OCT:OCT(POU,Homeobox,IR1)/NPC-Brn2-ChIP-Seq(GSE35496)	ATGAATWATTCATGA	1.00E+00	-8.16E-02	0.9772	1	1.01%	828.3	2.54%
Zfp281(Zf)/ES-Zfp281-ChIP-Seq(GSE81042)	CCCCTCCCCCAC	1.00E+00	-7.38E-02	0.9824	12	12.12%	5547.7	17.01%
KLF3(Zf)/MEF-Klf3-ChIP-Seq(GSE44748)	NRGCCCCRCCCHBNN	1.00E+00	-7.30E-02	0.9824	17	17.17%	7418.9	22.75%
PAX5(Paired,Homeobox),condensed/GM12878-PAX5-ChIP-Seq(GSE32465)	GTCACGCTCSCTGM	1.00E+00	-7.11E-02	0.9824	4	4.04%	2344.5	7.19%
ZNF519(Zf)/HEK293-ZNF519.GFP-ChIP-Seq(GSE58341)	GAGSCCGAGC	1.00E+00	-6.51E-02	0.9839	6	6.06%	3244.2	9.95%
NF-E2(bZIP)/K562-NFE2-ChIP-Seq(GSE31477)	GATGACTCAGCA	1.00E+00	-6.36E-02	0.9839	3	3.03%	1939	5.95%
RARa(NR)/K562-RARa-ChIP-Seq(Encode)	TTGAMCTTTG	1.00E+00	-5.23E-02	0.9919	87	87.88%	30007.7	92.03%
E-box(bHLH)/Promoter	SSGGTCACGTGA	1.00E+00	-4.76E-02	0.9942	2	2.02%	1561.2	4.79%
Tgif2(Homeobox)/mES-Tgif2-ChIP-Seq(GSE55404)	TGTCANYT	1.00E+00	-4.18E-02	0.9976	91	91.92%	31091.8	95.36%
ERG(ETS)/VCaP-ERG-ChIP-Seq(GSE14097)	ACAGGAAGTG	1.00E+00	-4.17E-02	0.9976	68	68.69%	24724.4	75.83%
RARg(NR)/ES-RARg-ChIP-Seq(GSE30538)	AGGTCAAGGTCA	1.00E+00	-3.45E-02	1	3	3.03%	2192.6	6.72%
Jun-AP1(bZIP)/K562-cJun-ChIP-Seq(GSE31477)	GATGASTCATCN	1.00E+00	-2.83E-02	1	13	13.13%	6527.6	20.02%
Bach1(bZIP)/K562-Bach1-ChIP-Seq(GSE31477)	AWWNTGCTGAGTCAT	1.00E+00	-1.89E-02	1	2	2.02%	1901	5.83%
SCL(bHLH)/HPC7-Scl-ChIP-Seq(GSE13511)	AVCAGCTG	1.00E+00	-1.45E-02	1	92	92.93%	31561.7	96.80%
Rfx2(HTH)/LoVo-RFX2-ChIP-Seq(GSE49402)	GTTGCCATGGCAACM	1.00E+00	-1.23E-02	1	3	3.03%	2602.6	7.98%
THRb(NR)/Liver-NR1A2-ChIP-Seq(GSE52613)	TRAGGTCA	1.00E+00	-1.22E-02	1	91	91.92%	31385	96.26%
Nrf2(bZIP)/Lymphoblast-Nrf2-ChIP-Seq(GSE37589)	HTGCTGAGTCAT	1.00E+00	-7.58E-03	1	1	1.01%	1571	4.82%
Sp1(Zf)/Promoter	GGCCCCGCCCCC	1.00E+00	-4.00E-03	1	3	3.03%	3028.1	9.29%
Nanog(Homeobox)/mES-Nanog-ChIP-Seq(GSE11724)	RGCCATTAAC	1.00E+00	-1.38E-03	1	93	93.94%	32063.8	98.34%
Pitx1(Homeobox)/Chicken-Pitx1-ChIP-Seq(GSE38910)	TAATCCCN	1.00E+00	-5.01E-04	1	93	93.94%	32150.5	98.60%
DUX(Homeobox)/C2C12-Dux-ChIP-Seq(GSE87279)	BCWGATTCAATCAAN	1.00E+00	0.00E+00	1	0	0.00%	150.5	0.46%
E2F(E2F)/Hela-CellCycle-Expression	TTSGCGCGAAAA	1.00E+00	0.00E+00	1	0	0.00%	607.7	1.86%
GFX(?)/Promoter	ATTCTCGCGAGA	1.00E+00	0.00E+00	1	0	0.00%	132.8	0.41%
ZBTB33(Zf)/GM12878-ZBTB33-ChIP-Seq(GSE32465)	GGVTCTCGCGAGAAC	1.00E+00	0.00E+00	1	0	0.00%	386.8	1.19%
ZFP3(Zf)/HEK293-ZFP3.GFP-ChIP-Seq(GSE58341)	GGGTTTTGAAGGATGARTAGGAGTT	1.00E+00	0.00E+00	1	0	0.00%	28.7	0.09%
ZNF528(Zf)/HEK293-ZNF528.GFP-ChIP-Seq(GSE58341)	AGAAATGACTTCCCT	1.00E+00	0.00E+00	1	0	0.00%	253.6	0.78%

Supplementary Table 8: Acetylation changes in complement-related genes in the substantia nigra

chr	*start*	*end*	*annotation*	*logFC*	*PValue*	*FDR*	*GENE*	*GENENAME*	*distanceToTSS*	*DAR*
chr5	149122084	149124344	Promoter (<=1	1428	552E-08	240E-05	C1qb	complement (	48	Hyper
chr5	149127558	149128451	Promoter (2-3	2136	351E-06	000050	C1qc	complement (	2281	Hyper
chr5	149133535	149136488	Promoter (<=1	0755	912E-05	000540	C1qa	complement (	0	Hyper
chr1	182658508	182660142	Promoter (<=1	1085	246E-04	001044	Itgam	integrin subur	0	Hyper
chr5	149124589	149125529	Promoter (<=1	1223	921E-04	002507	C1qb	complement (	-197	Hyper
chr5	149128659	149131064	Promoter (<=1	0644	217E-03	004334	C1qc	complement (	0	Hyper
chr15	40109523	40111537	Distal Interger	0578	631E-03	008454	Clu	clusterin	-49531	non-DAR
chr1	182679395	182680892	Intron (NM_0	0614	406E-02	024223	Itgam	integrin subur	20219	non-DAR
chr5	149131205	149132445	Promoter (<=1	0577	501E-02	027106	C1qc	complement (	-473	non-DAR
chr5	119659491	119660608	Distal Interger	0381	183E-01	050871	C8a	complement (	-21816	non-DAR
chr13	31531306	31532431	Distal Interger	-0361	200E-01	053185	C1ql2	complement (	60156	non-DAR
chr3	18427157	18429010	Distal Interger	-0194	207E-01	054135	C5	complement (	-65163	non-DAR
chr1	76943723	76946132	Promoter (1-2	0166	438E-01	074697	C5ar2	complement (	-1342	non-DAR
chr4	157444916	157445832	Promoter (2-3	0161	565E-01	082565	C1s	complement (	-2661	non-DAR
chr9	2116263	2118925	Promoter (1-2	-0123	565E-01	082577	C3	complement (	-1897	non-DAR
chr3	18436715	18438164	Distal Interger	-0136	591E-01	083888	C5	complement (	-74721	non-DAR
chr1	76941457	76943368	Promoter (<=1	0072	761E-01	091618	C5ar2	complement (	0	non-DAR
chr10	55703654	55705321	Promoter (<=1	0022	907E-01	097071	C1qbp	complement (	0	non-DAR
